# Estimating global, regional, and national daily and cumulative infections with SARS-CoV-2 through Nov 14, 2021: a statistical analysis

**DOI:** 10.1016/S0140-6736(22)00484-6

**Published:** 2022-06-25

**Authors:** COVID-19 Cumulative Infection Collaborators, Ryan M Barber, Reed J D Sorensen, David M Pigott, Catherine Bisignano, Austin Carter, Joanne O Amlag, James K Collins, Cristiana Abbafati, Christopher Adolph, Adrien Allorant, Aleksandr Y Aravkin, Bree L Bang-Jensen, Emma Castro, Suman Chakrabarti, Rebecca M Cogen, Emily Combs, Haley Comfort, Kimberly Cooperrider, Xiaochen Dai, Farah Daoud, Amanda Deen, Lucas Earl, Megan Erickson, Samuel B Ewald, Alize J Ferrari, Abraham D Flaxman, Joseph Jon Frostad, Nancy Fullman, John R Giles, Gaorui Guo, Jiawei He, Monika Helak, Erin N Hulland, Bethany M Huntley, Alice Lazzar-Atwood, Kate E LeGrand, Stephen S Lim, Akiaja Lindstrom, Emily Linebarger, Rafael Lozano, Beatrice Magistro, Deborah Carvalho Malta, Johan Månsson, Ana M Mantilla Herrera, Ali H Mokdad, Lorenzo Monasta, Mohsen Naghavi, Shuhei Nomura, Christopher M Odell, Latera Tesfaye Olana, Samuel M Ostroff, Maja Pasovic, Spencer A Pease, Robert C Reiner Jr, Grace Reinke, Antonio Luiz P Ribeiro, Damian F Santomauro, Aleksei Sholokhov, Emma E Spurlock, Ruri Syailendrawati, Roman Topor-Madry, Anh Truc Vo, Theo Vos, Rebecca Walcott, Ally Walker, Kirsten E Wiens, Charles Shey Wiysonge, Nahom Alemseged Worku, Peng Zheng, Simon I Hay, Emmanuela Gakidou, Christopher J L Murray

## Abstract

**Background:**

Timely, accurate, and comprehensive estimates of SARS-CoV-2 daily infection rates, cumulative infections, the proportion of the population that has been infected at least once, and the effective reproductive number (R_effective_) are essential for understanding the determinants of past infection, current transmission patterns, and a population's susceptibility to future infection with the same variant. Although several studies have estimated cumulative SARS-CoV-2 infections in select locations at specific points in time, all of these analyses have relied on biased data inputs that were not adequately corrected for. In this study, we aimed to provide a novel approach to estimating past SARS-CoV-2 daily infections, cumulative infections, and the proportion of the population infected, for 190 countries and territories from the start of the pandemic to Nov 14, 2021. This approach combines data from reported cases, reported deaths, excess deaths attributable to COVID-19, hospitalisations, and seroprevalence surveys to produce more robust estimates that minimise constituent biases.

**Methods:**

We produced a comprehensive set of global and location-specific estimates of daily and cumulative SARS-CoV-2 infections through Nov 14, 2021, using data largely from Johns Hopkins University (Baltimore, MD, USA) and national databases for reported cases, hospital admissions, and reported deaths, as well as seroprevalence surveys identified through previous reviews, SeroTracker, and governmental organisations. We corrected these data for known biases such as lags in reporting, accounted for under-reporting of deaths by use of a statistical model of the proportion of excess mortality attributable to SARS-CoV-2, and adjusted seroprevalence surveys for waning antibody sensitivity, vaccinations, and reinfection from SARS-CoV-2 escape variants. We then created an empirical database of infection–detection ratios (IDRs), infection–hospitalisation ratios (IHRs), and infection–fatality ratios (IFRs). To estimate a complete time series for each location, we developed statistical models to predict the IDR, IHR, and IFR by location and day, testing a set of predictors justified through published systematic reviews. Next, we combined three series of estimates of daily infections (cases divided by IDR, hospitalisations divided by IHR, and deaths divided by IFR), into a more robust estimate of daily infections. We then used daily infections to estimate cumulative infections and the cumulative proportion of the population with one or more infections, and we then calculated posterior estimates of cumulative IDR, IHR, and IFR using cumulative infections and the corrected data on reported cases, hospitalisations, and deaths. Finally, we converted daily infections into a historical time series of R_effective_ by location and day based on assumptions of duration from infection to infectiousness and time an individual spent being infectious. For each of these quantities, we estimated a distribution based on an ensemble framework that captured uncertainty in data sources, model design, and parameter assumptions.

**Findings:**

Global daily SARS-CoV-2 infections fluctuated between 3 million and 17 million new infections per day between April, 2020, and October, 2021, peaking in mid-April, 2021, primarily as a result of surges in India. Between the start of the pandemic and Nov 14, 2021, there were an estimated 3·80 billion (95% uncertainty interval 3·44–4·08) total SARS-CoV-2 infections and reinfections combined, and an estimated 3·39 billion (3·08–3·63) individuals, or 43·9% (39·9–46·9) of the global population, had been infected one or more times. 1·34 billion (1·20–1·49) of these infections occurred in south Asia, the highest among the seven super-regions, although the sub-Saharan Africa super-region had the highest infection rate (79·3 per 100 population [69·0–86·4]). The high-income super-region had the fewest infections (239 million [226–252]), and southeast Asia, east Asia, and Oceania had the lowest infection rate (13·0 per 100 population [8·4–17·7]). The cumulative proportion of the population ever infected varied greatly between countries and territories, with rates higher than 70% in 40 countries and lower than 20% in 39 countries. There was no discernible relationship between R_effective_ and total immunity, and even at total immunity levels of 80%, we observed no indication of an abrupt drop in R_effective_, indicating that there is not a clear herd immunity threshold observed in the data.

**Interpretation:**

COVID-19 has already had a staggering impact on the world up to the beginning of the omicron (B.1.1.529) wave, with over 40% of the global population infected at least once by Nov 14, 2021. The vast differences in cumulative proportion of the population infected across locations could help policy makers identify the transmission-prevention strategies that have been most effective, as well as the populations at greatest risk for future infection. This information might also be useful for targeted transmission-prevention interventions, including vaccine prioritisation. Our statistical approach to estimating SARS-CoV-2 infection allows estimates to be updated and disseminated rapidly on the basis of newly available data, which has and will be crucially important for timely COVID-19 research, science, and policy responses.

**Funding:**

Bill & Melinda Gates Foundation, J Stanton, T Gillespie, and J and E Nordstrom.

## Introduction

Measuring SARS-CoV-2's daily infection rate, cumulative infections, and the proportion of the population with one or more infections is essential for understanding the determinants of past transmission, identifying ongoing inequities, predicting future trajectories of the COVID-19 pandemic, and, in theory, prioritising vaccination allocations. Daily infections are also the crucial input into measuring the changing effective reproductive number (R_effective_, the number of subsequent infections caused by a new infection).^1^, ^2^, ^3^ A robust assessment of R_effective_ by day in each location is useful to help evaluate the effect of the wide range of non-pharmaceutical interventions that have been deployed during the pandemic. The R_effective_ over time is also a crucial input into future forecasts of COVID-19.^4^ Cumulative infections can help us identify which nations and communities have been able to keep transmission at lower levels, potentially creating the opportunity to learn from these success stories. Finally, a sound measurement of the proportion of the population ever infected could help to identify which communities are at greater risk of future transmission and might be a factor that should be considered in vaccine prioritisation.^5^

**Research in context**

**Evidence before this study**

This study was conceptualised and developed from the start of the pandemic to fill a void in the provision of timely estimates of SARS-CoV-2 infections for tracking the pandemic and to provide inputs to epidemiological models of transmission. Several research groups have estimated SARS-CoV-2 daily or cumulative infections in select locations at specific points in time. For example, the US Centers for Disease Control and Prevention estimates cumulative infections by approximating the infection–detection ratio (IDR) using assumptions about the portion of the population who will seek care. The Serotracker project reports on the universe of seroprevalence surveys and some attributes of these surveys, but it does not make estimates of cumulative infections based on these data. Noh and Danuser (2021) used reported deaths and published estimates of the infection–fatality ratio (IFR) to estimate cumulative infections for US states and select countries. To our knowledge, however, no source has provided estimates, either periodic or regularly updated, of global daily and cumulative SARS-CoV-2 infections at this resolution (399 administrative units).

**Added value of this study**

This study is the first comprehensive analysis of global daily and cumulative SARS-CoV-2 infections to date and improves upon previous infection estimation strategies in several important ways. First, we combined three approaches that have been used to estimate daily infections: cases divided by the IDR, hospitalisations divided by the infection–hospitalisation ratio (IHR), and deaths divided by the IFR. Combining these estimates gave us a more robust estimate of daily infections that was less susceptible to biases within and between each type of measure. Second, estimates of total COVID-19 deaths derived from a comprehensive assessment of excess mortality and a statistical estimate of the portion of excess mortality directly due to COVID-19 allowed for more meaningful interpretation of spatial heterogeneity in total COVID-19 mortality rates. Third, we used a systematic analysis of available seroprevalence data matched in space and time to cases, hospitalisations, and deaths to empirically estimate the IDR, IHR, and IFR. Because the IHR and IFR are profoundly age related, we also estimated age-standardised ratios for these quantities. Fourth, for locations without seroprevalence surveys, we used statistical models based on the available empirical data and the testing of a wide range of covariates to predict the IDR, IHR, and IFR. Fifth, we used daily infections to estimate cumulative infections and, with assumptions on cross-variant immunity, the cumulative number of individuals with one or more infections, as well as posterior estimates of cumulative IDR, IHR, and IFR. Sixth, we incorporated corrections to the primary data into the analysis to deal with known biases such as waning antibody test sensitivity. Seventh, our ensemble model reflects the uncertainty of the data sources, model design, and parameter assumptions included in the analysis. Finally, the methods developed to triangulate on daily infections, cumulative infections, and the proportion of the population infected once or more than once have been developed into easily applied statistical code, so estimates can be shared and updated rapidly and iteratively on the basis of the frequency of newly reported data.

**Implications of all the available evidence**

SARS-CoV-2 has been extremely widespread, causing 3·80 billion (95% uncertainty interval 3·44–4·08) infections and reinfections as of Nov 14, 2021, infecting 43·9% (39·9–46·9) of the world's population. The proportion of the population infected has varied greatly across countries, suggesting that host immunity characteristics and national and local policies play a crucial role in determining patterns of transmission. Our comprehensive modelling approach provides a database of daily infections and effective reproductive number by location from the beginning of the pandemic to Nov 14, 2021, which can be used to develop insights into the determinants of transmission, identify ongoing inequities, establish standards for vaccine prioritisation, and more.

Several studies have estimated cumulative infections in select countries at specific points in time.^6^, ^7^, ^8^, ^9^ Some of these studies have used seroprevalence surveys, while others have made estimates of infections by assuming a particular infection–detection ratio (IDR).^7^, ^10^, ^11^, ^12^ One study estimated infections in the USA and other select countries,^13^ and other studies have done multinational systematic reviews and meta-analyses of seroprevalence surveys.^14^, ^15^ The fundamental problem in all of these analyses is that each of the data series observed has potential biases: reported cases capture only a portion of infections, and this portion will be a function of the availability of testing; reported deaths capture only a subset of total COVID-19 deaths, and the infection–fatality ratio (IFR) can vary widely over time and across locations;^16^, ^17^, ^18^, ^19^ the proportion of patients with an infection who are admitted to hospital can also vary over time and location; and seroprevalence surveys can be influenced by sampling design, waning of sensitivity of antibody tests, and vaccination rates. Few studies have combined data from reported cases, reported deaths, hospitalisations, and seroprevalence surveys to triangulate daily infections, and WHO only routinely reports confirmed cases, not estimated infections.^20^ The use of such sources of incomplete, biased, and heterogeneous case data uncritically in research, science, and policy will result in inferences confounded to unknown levels by these known problems.

In this study, we present an approach to estimating past SARS-CoV-2 daily infections, cumulative infections through Nov 14, 2021, and the proportion of the population with one or more infections on the basis of reported cases, total deaths attributable to COVID-19, hospitalisations, and seroprevalence surveys. This approach attempts to deal with the biases in each of these measures and use them all to triangulate daily infections. With this statistical approach to the fusion of these data streams, we aimed to provide a method that can be applied on a rapid and ongoing basis, so that these estimates remain maximally relevant for research, science, and policy and can be immediately and freely available. Importantly, we incorporated various sources of uncertainty in daily infections into the analysis to help informed assessment of the variation in space and time of the fidelity of the estimates.

## Methods

### Overview

We derived comprehensive global estimates of daily and cumulative SARS-CoV-2 infections for the duration of the COVID-19 pandemic, using the heterogeneous universe of reported epidemiological data (iteratively curated, corrected, and calibrated into an internally complete and consistent time series at national and subnational levels) to further timely research, discovery, and policy inference. Our approach can be divided into seven steps, which are applied by use of an ensemble model framework. First, we developed a dataset of reported COVID-19 cases, total COVID-19 deaths, and hospitalisations (where available), corrected for known biases such as lags in reporting. Second, we identified representative SARS-CoV-2 seroprevalence surveys that could be used to create a database of cumulative infections and adjusted them for waning antibody sensitivity, vaccinations, and reinfection from escape variants. Third, using adjusted seroprevalence survey data matched to cases, hospitalisations, and deaths, we created an empirical database of IDRs, infection–hospitalisation ratios (IHRs), and IFRs. Fourth, for locations without seroprevalence surveys and to estimate a complete time series for each location, we developed statistical models to predict the IDR, IHR, and IFR by location and day, as a function of a wide range of covariates. Fifth, three series of estimates of daily infections (cases divided by IDR, hospitalisations divided by IHR, and deaths divided by IFR) were combined into a more robust estimate of daily infections. Sixth, we used the combined time series of daily infections to estimate cumulative infections and the cumulative proportion of the population with one or more infections, and calculate posterior estimates of cumulative IDR, IHR, and IFR. Seventh, we converted daily infections into a historical time series of R_effective_ by location and day, on the basis of assumptions of duration of the period from infection to infectiousness and time an individual spent being infectious. Estimates are given for all ages and both sexes combined for 190 countries and territories, and for subnational locations in ten of those countries, aggregated into 21 regions, seven super-regions,^21^ and globally, from the start of the COVID-19 pandemic through Nov 14, 2021.

This study complies with the Guidelines for Accurate and Transparent Health Estimates Reporting recommendations (appendix 1, section 2).^22^ All code used in the analysis can be found online.

### Ensemble framework

Our model system includes many component parts that are inherently uncertain, ranging from input data sources and parameter assumptions to model specification. To account for this, we developed an ensemble framework wherein we varied the data and model settings across 100 iterations of the analysis, which were then run independently to yield 100 estimates of infections. These sources of uncertainty include seroprevalence survey error; bootstrapped samples of our seroprevalence database; estimates of seroreversion rates; estimates of total COVID-19 mortality; parameterisation of cross-variant immunity, increased risk of hospitalisation and death from non-ancestral SARS-CoV-2 variants, and durations associated with COVID-19 natural history; covariate selection and specification of statistical models of the IDR, IHR, and IFR; and triangulation of infections on the basis of cases, hospitalisations, and deaths (more details regarding the ensemble framework in appendix 1, section 9).

### Data inputs and corrections

Data of reported cases were obtained largely from Johns Hopkins University (Baltimore, MD, USA),^23^ with exceptions and additions noted in appendix 1 (section 4.1) and appendix 2 (section 4). Hospital admissions were largely sourced from national databases such as that of the Department of Health and Human Services (HHS) in the USA and the *Secretaria de Vigilância em Saúde* in Brazil (for an exhaustive list see appendix 2, section 1). Deaths were based on reported deaths data from Johns Hopkins University^23^ and various national sources from locations where data inconsistencies were evident in the Johns Hopkins University datasets (more details in appendix 1, section 4.3, and appendix 2, section 2). To account for the prevalent issue of under-reporting in COVID-19 deaths, we applied a scalar of reported to total COVID-19 deaths in our analysis. Total COVID-19 deaths, as defined by WHO, are all deaths where the deceased individuals were actively infected with SARS-CoV-2 at the time of the death. Estimates of total COVID-19 mortality were constructed with use of the statistical model developed by the COVID-19 Excess Mortality Collaborators to predict the excess mortality rate for all locations between Jan 1, 2020, and Nov 14, 2021.^16^ To estimate total COVID-19 mortality, we predicted a counterfactual excess mortality rate due to COVID-19 in which the IDR was set to the maximum observed values among all locations. The predicted excess mortality rate from this counterfactual analysis, corrected for under-reporting, resulted from insufficient testing and changes in mortality driven by behaviours such as deferred health care during periods of lockdown. We used the ratio of this counterfactual excess mortality rate and the prediction for the same period as a proxy for the proportion of excess mortality that is total COVID-19 mortality. Subsequently, a scalar of reported COVID-19 deaths to total COVID-19 deaths can be derived (more details in appendix 1, section 9.4). We identified seroprevalence surveys through a search protocol that leveraged previous reviews,^24^, ^25^ SeroTracker,^26^ and routine inclusion of national and subnational surveys undertaken by governmental organisations. Studies that focused on specific subsets of the population—either a specific subpopulation such as health-care workers or specific locations such as specific cities—were typically excluded as a result of not being representative. In total, we identified 2817 seroprevalence survey datapoints (of 6420 reviewed) for inclusion in this analysis.

Although most data streams for daily cases, deaths, and hospitalisations are indexed by date of report, some are indexed by date of event; in these instances, lags in reporting create misleading trends in the most recent days of data. These trends are gradually corrected over time as reporting systems catch up but, to prevent this occurrence from influencing our models, we needed to evaluate each individual data source and determine an appropriate number of days to exclude in any iteration of the analyses.

Some hospital admissions data series only became available starting from weeks or months after the beginning of the COVID-19 pandemic—for example, the HHS database began in July, 2020. However, total cumulative hospitalisations are required to create our empirical estimate of IHR. In these instances, we leveraged information from the metrics that did have complete time coverage (cases and deaths) to impute the earlier portion of the admissions time series (appendix 1, section 4.2).

### Seroprevalence survey adjustments

Seroprevalence surveys were corrected for vaccination, because vaccination generates a positive anti-spike antibody test in most individuals who receive the vaccine.^27^ In locations where vaccination rates have increased over time, population levels of anti-spike antibodies will be elevated. To correct for this, we adjusted seroprevalence estimates downward on the basis of vaccination rates in adults in every location, accounting for vaccination of previously infected individuals (appendix 1, section 5.1).

Seroprevalence surveys provide an estimate of the number of individuals who have been infected with SARS-CoV-2 one or more times; these surveys do not detect repeat infections in a single individual. Because reinfection can be common in settings where escape variants such as beta (B.1.351), gamma (P.1), and delta (B.1.617.2) are present,^28^, ^29^, ^30^ we had to adjust seroprevalence data to estimate the cumulative number of infections—that is, to include both first and any subsequent infections. We used a level of cross-variant immunity of 30% to 70% between escape variants and ancestral variants and alpha (B.1.1.7), on the basis of an empirical analysis conducted by the COVID-19 Forecasting Team (unpublished). This estimate did not take into account that some individuals could have been infected more than once with ancestral variants.^31^ A detailed explanation of how we adjusted for escape variant prevalence is given in appendix 1 (section 5.2).

Lastly, seroprevalence surveys were corrected for waning sensitivity of antibody tests. We identified eight categories of antibody tests; for each of these, we used a reported curve of sensitivity over time.^32^, ^33^, ^34^ To implement the correction based on waning, we used initial estimates of the timing of infection based on reported deaths. We did not adjust for specificity, as reported specificity for all available commercial assays included in the analysis is over 95% and mostly over 98% (more details in appendix 1, sections 5.3 and 9.3).^35^

### Empirical estimates of the IDR, IHR, and IFR

**Figure 1:**
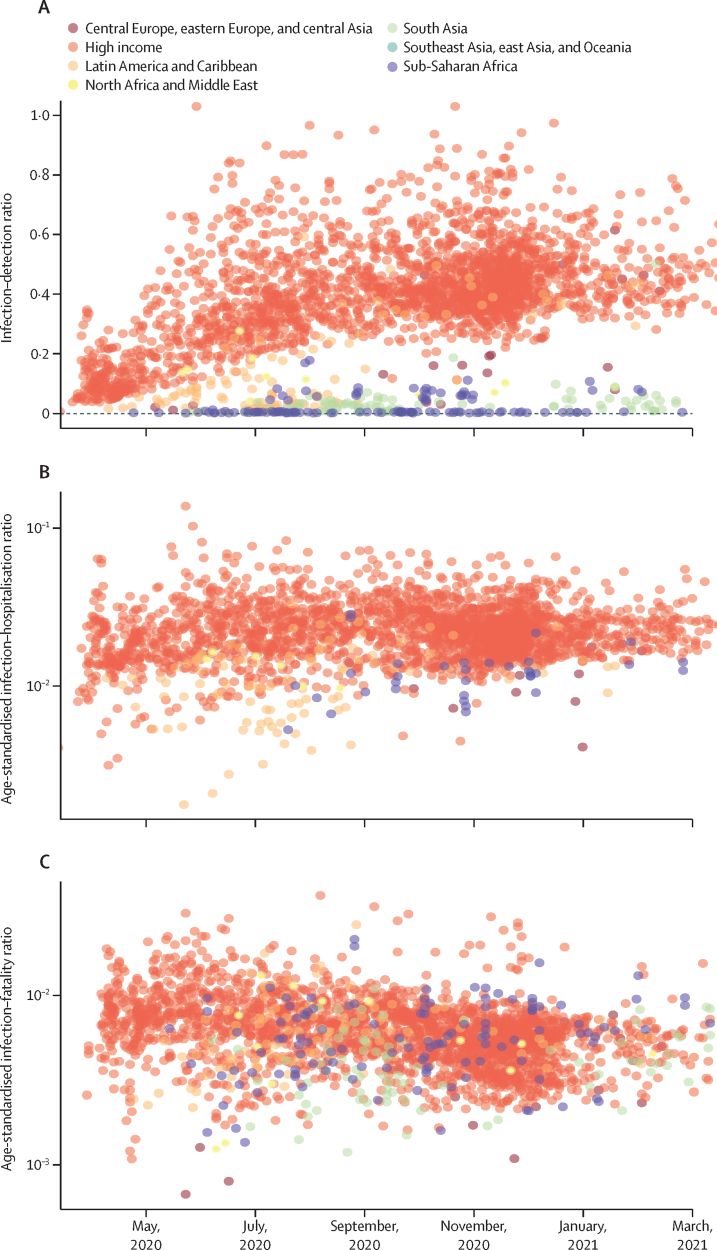
Empirical measurements over time of infection–detection ratios (A), age-standardised infection–hospitalisation ratios (B), and age-standardised infection–fatality ratios (C) The y-axis for infection–hospitalisation ratios and infection–fatality ratios is shown in log base 10.

Using the adjusted seroprevalence data we have described, we created a dataset of 2817 empirical measurements of the IDR in which the numerator was the cumulative number of confirmed cases and the denominator was the number of cumulative infections and reinfections combined. We aligned cases and seroprevalence on the basis of individual record data suggesting that exposure to a laboratory-confirmed case was typically 10–13 days^36^ and exposure to seroconversion was 14–17 days.^37^, ^38^, ^39^ Figure 1A shows these empirical estimates of location-specific IDR over the course of the pandemic. For the purposes of visualising the data, the IDR data are time-localised to the average date of infection based on the model estimate and daily cases.

Using adjusted seroprevalence surveys matched to cumulative hospitalisations, we developed a dataset of 2580 empirical estimates of the IHR. Based on the same data and analysis used to determine the lag for cases,^36^ we used a 10–13-day lag for hospitalisations. Far fewer locations reported hospitalisations, so less information was available for this metric than for the IDR. We used 703 surveys that included age-specific seroprevalence data to estimate the IHR age pattern, and we then used indirect age standardisation to estimate the age-standardised IHR across locations and used those age-standardised estimates in the modelling of the IHR (more details on indirect standardisation methods in appendix 1, section 6.1). Figure 1B shows the universe of available age-standardised IHR over time. For the purposes of visualising the data, IHR data are time-localised to the average date of admission.

Using the 718 seroprevalence surveys with age-specific detail, the COVID-19 Forecasting Team^40^ estimated the age pattern of the IFR. We used this age pattern to create a dataset of age-standardised IFR data using 2817 pairs of adjusted seroprevalence surveys and death data, assuming 22–28 days from exposure to death on the basis of analyses of patient-level data in the USA.^41^ Time indexing of IFR data was based on the average date of death for each observation. Figure 1C shows the relationship between age-standardised IFR and time.

### Statistical models of the IDR, IHR, and IFR

To generate estimates of daily infections from cases, hospitalisations, and deaths, we needed estimates of the IDR, IHR, and IFR by location for each day during the pandemic. We used a cascading implementation of a Bayesian regression framework^42^ to estimate each of these measures (more details in appendix 1, section 6.2). The cascading regression model allows for a flexible fit to the key covariates, including the option to specify them as splines, and borrows strength across locations. After parameterising the relationship of seroprevalence to cases, hospitalisations, and deaths through predictive models of IDR, IHR, and IFR, we used local covariates and age structure to generate predictions of these ratios in both in-sample and out-of-sample locations based on our hierarchical cascade model. For the IDR model, the most spatially and temporally consistent predictive relationship was between testing per person and the IDR. To capture the rise in health system capacity to deliver testing, we used the observed maximum testing rate up to a given date as the covariate. Additionally, we included universal health-care coverage, the Healthcare Access and Quality (HAQ) Index, and the proportion of the population older than 65 years as covariates that each submodel selected from in our ensemble. These covariates were estimated for all locations as part of the Global Burden of Diseases, Injuries, and Risk Factors Study (GBD; appendix 1, sections 6.3 and 9.6).^43^

Predictive covariates for IHR and IFR were primarily based on a list of underlying medical conditions identified by the US Centers for Disease Control and Prevention (CDC) as increasing the risk of severe illness from SARS-CoV-2 infection.^44^ We cross-referenced this list with a study of individuals admitted to hospital in the USA^41^ that evaluated the increased risk of in-hospital death to identify seven possible covariates, all of which were included in our models as age-standardised prevalence in the population (estimated as part of GBD): obesity, smoking, diabetes, cancer, chronic obstructive pulmonary disease, cardiovascular disease, and chronic kidney disease.^43^ Several of these covariates, most prominently obesity, were further supported by relationships in US claims^45^ and Brazil hospitalisations data.^46^ To this list, we also added universal health-care coverage and the HAQ Index. We then tested all possible combinations of these covariates and selected the top 100 most predictive combinations to use across submodels in our ensemble models of IHR and IFR (more details in appendix 1, section 9.6). We estimated age-standardised IHR and IFR using these covariates and then converted estimates back to all-age IHR and IFR to reflect population structure. We accounted for reductions in the IFR due to improved treatment over the course of the pandemic by including a spline on time in the regressions in addition to the ensemble covariates (more details on these models in appendix 1, sections 6.4, 6.5, and 9.6).

Vaccines and variants also affect the likelihood of severe disease and death, and thus influence both the IHR and the IFR. First, vaccination strategies that prioritise older age groups before younger ones can temporarily increase the relative proportion of infections that occur in younger individuals, thus lowering the population-level IFR and IHR for at least a period of time. Additionally, COVID-19 vaccines have been shown to confer higher levels of protection from severe disease and death than from mild infection, also serving to lower the overall IFR and IHR. The prevalence of variants with higher likelihood of severe disease and death can conversely increase these ratios,^47^ and the introduction of escape variants can increase them further by reducing vaccine efficacy. More information on how we accounted for these features can be found in appendix 1 (section 6.6).

### Robust estimates of daily infections

We then paired the estimates of our ratio models with data that were reported by local jurisdictions—accounting for reporting biases in cases through the testing covariate and in deaths through the total COVID-19 death scalars—to estimate infections in a manner that was sensitive to local context, even in the absence of seroprevalence data. By dividing cases by the modelled IDR, hospitalisations by the modelled IHR, and deaths by the modelled IFR, we produced three daily infections time series (or two if only cases and deaths were reported for a given location). Estimates based on each input data type were shifted back in time by their respective lags, such that they were all indexed on date of infection. We then fit a time series spline model using all three data sources as inputs to triangulate a best estimate of daily infections. After deriving this mean estimate of daily infections, we sampled the residuals of the intermediate case-based, hospitalisation-based, and deaths-based infection estimates independently in each submodel and refit the infections curve to these data; this enabled us to more accurately reflect the volatility in reporting practices, such as for deaths, in our ensemble distribution of daily infections (more details in appendix 1, section 7).

### Cumulative infections and cumulative proportion of the population infected at least once

Daily infections, including reinfections, were summed to derive an estimate of cumulative infections. With this estimate of cumulative infections, we then returned to reported cases, reported hospitalisations, and total COVID-19 deaths to produce posterior estimates of cumulative IDR, IHR, and IFR. Where the reported data were not available, the posterior ratio estimate would be equal to the prediction from the ratio model. To estimate the proportion of individuals who were infected with SARS-CoV-2 at least once by Nov 14, 2021, we used the same assumptions already described. The crucial assumptions required were cross-variant immunity, the prevalence of escape variants, and the assumption that exposure to escape variants is independent of the probability of previous infection with ancestral variants.

Figures found in appendix 3 show cases, hospitalisations (where available), deaths, IDR, IHR, IFR, daily infections, cumulative infections, and cumulative proportion of the population infected at least once for 399 locations.

### R_effective_ estimation in the past

Using daily infections, we directly estimated R_effective_ in the past by location and day, where R_effective_ at time *t* is:

$$R_{effective}(t)=\frac{\inf ections(t+\theta )}{\inf ections(t)}$$

The assumptions required for this estimation are the duration from infection to being infectious and the period of infectiousness, collectively represented as θ. We used ranges of 3–5 days for both assumptions to generate estimates of R_effective_ in the past. These estimates are useful for identifying the effect of different non-pharmaceutical interventions on transmission in different settings. An R_effective_ lower than 1·0 indicates that the epidemic is shrinking, whereas an R_effective_ higher than 1·0 indicates that the epidemic is growing.

We compared R_effective_ to an estimate of total immunity in the population of location *l* at time *t* (presented as weekly averages), where this value is calculated as:

$$totalimmunity_{l,t}=1-(1-prop.\inf ected_{l,t})X(1-prop.effectivelyvaccinated_{l,t})$$

The proportion of the population effectively vaccinated is a function of doses administered and brand-specific efficacy and is discounted for existing natural immunity at the time of delivery.

### Role of the funding source

The funders of the study had no role in the study design, data collection, data analysis, data interpretation, or the writing of the report.

## Results

**Figure 2:**
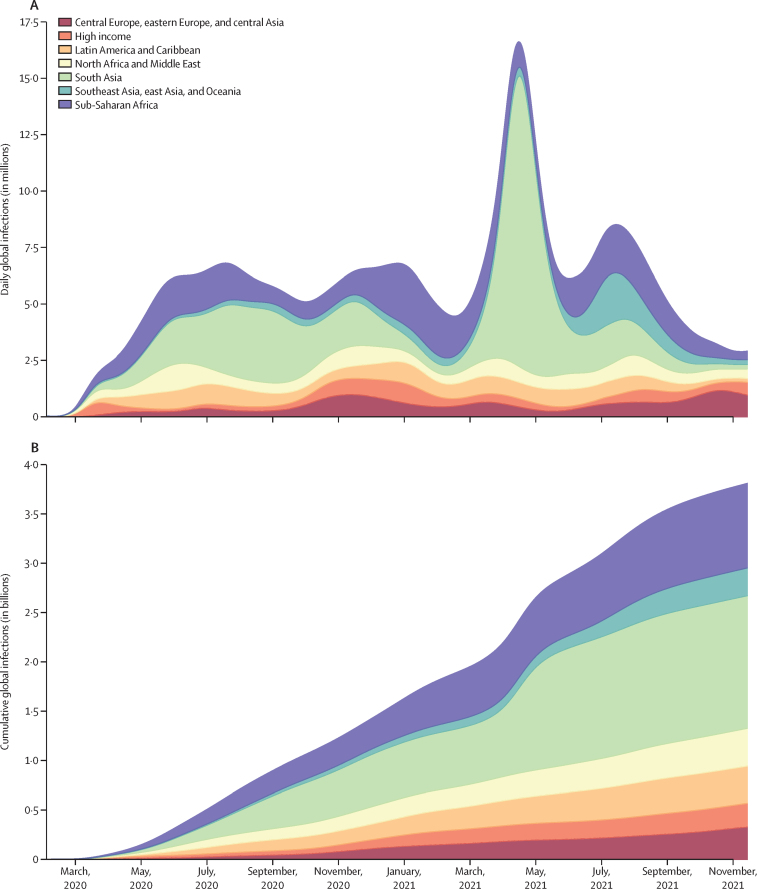
Daily (A) and cumulative (B) infections by super-region from Feb 4, 2020, to Nov 14, 2021

**Figure 3:**
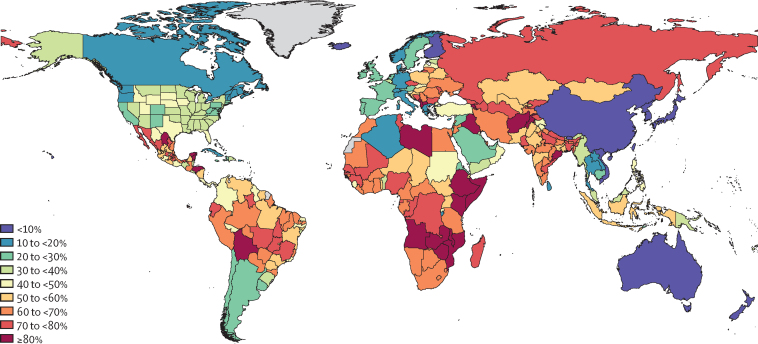
Cumulative proportion of the population infected with SARS-CoV-2 at least once by Nov 14, 2021, by country and territory The first administrative level is mapped for countries that are modelled at that level and have a population greater than 100 million.

Globally, daily SARS-CoV-2 infections steadily increased over the first several months of the pandemic, surpassing 3 million daily infections for the first time in mid-April, 2020, and then doubling to 6 million per day 6 weeks later (figure 2A). Global daily infections remained higher than 5 million per day until dipping slightly below that threshold after a period of decline in January and February, 2021. Driven primarily by the delta variant surge in India, global daily infections soared to a pandemic high of nearly 17 million in April, 2021, then dropped as low as 6 million by June, 2021, before delta variant waves in other parts of the world led to another global surge peaking at over 8 million infections per day in July, 2021. This peak was followed by the longest sustained decline of the pandemic at the global level, wherein global infections dropped below 3 million per day by the end of October, 2021, for the first time in 18 months. Between the start of the pandemic and Nov 14, 2021, there were an estimated 3·80 billion (95% uncertainty interval [UI] 3·44–4·08) SARS-CoV-2 infections and reinfections globally (table, figure 2B). Nearly 1·5 billion of these infections occurred in south Asia (1·34 billion [1·20–1·49]), the most infections of all seven super-regions, whereas the highest infection rate was estimated in sub-Saharan Africa (79·3 per 100 population [95% UI 69·0–86·4]). Four other super-regions each had infection rates greater than 60 per 100 population (table): central Europe, eastern Europe, and central Asia (78·4 [49·3–93·2]); south Asia (74·3 [66·5–82·6]); Latin America and the Caribbean (64·1 [57·2–71·3]); and north Africa and the Middle East (62·7 [46·2–79·0]). Southeast Asia, east Asia, and Oceania had the lowest infection rate (13·0 per 100 population [8·4–17·7]) of all seven super-regions, whereas the high-income super-region had nearly double that infection rate but the fewest infections (239 million [95% UI 226–252]; table). At the global level, the cumulative proportion of the population infected with SARS-CoV-2 one or more times reached 13·7% (95% UI 12·2–15·1) by the end of the first wave of global infections on Oct 1, 2020, increasing to 24·1% (21·9–25·8) by the end of the second wave on Feb 15, 2021. More than a third of the global population had been exposed to COVID-19 after the delta variant surge in India (35·0% [32·2–37·3]). And by Nov 14, 2021, 43·9% (39·9–46·9) of the global population (3·39 billion individuals [3·08–3·63]) had been infected with SARS-CoV-2 at least once. The cumulative proportion infected at least once varied greatly across countries and territories (table, figure 3). Over 70% of the population had been infected in 40 countries, including over 80% in 17 countries and across states in Mexico, India, and Pakistan. More than half the population had been infected in an additional 55 countries and territories across every super-region, except high income. Notable cross-border variations were observed in some parts of the world, such as at the interface of western and central Europe, where the percentage of the population infected was substantially lower in Germany, Austria, and Italy than in the bordering nations Poland, Czechia, Slovakia, Hungary, and Slovenia. In South America, a clear demarcation can be seen splitting the tropical and Andean nations from the southern nations and the Brazilian state Rio Grande do Sul. Countries in mainland southeast Asia such as Laos, Thailand, and Vietnam, maintained a much lower percentage of population infected than neighbouring south Asian countries or island nations within the region, such as Indonesia and the Philippines. The cumulative percentage of population infected varied widely within most countries for which subnational units were modelled in this analysis, varying by a factor of two across administrative units in Brazil, India, Italy, and Mexico; a factor of three in Germany and Spain; and over a factor of four in the USA (table).

**Table:** Cumulative total COVID-19 deaths, infections, proportion of the population infected, infection–detection ratio, infection–hospitalisation ratio, and infection–fatality ratio up to Nov 14, 2021, by location

			**Cumulative total COVID-19 deaths**	**Cumulative total COVID-19 death rate (per 100 000 population)**	**Cumulative infections**	**Cumulative infection rate (per 100 population)**	**Cumulative percentage infected**	**Cumulative infection–detection ratio**	**Cumulative infection–hospitalisation ratio**	**Cumulative infection–fatality ratio**
**Global**			**15 100 000 (11 200 000–20 200 000)**	**194·5 (144·5–261·6)**	**3 800 000 000 (3 440 000 000–4 080 000 000)**	**49·1 (44·4–52·7)**	**43·9% (39·9–46·9)**	**6·9% (6·4–7·6)**	**1·2% (1·0–1·6)**	**0·4% (0·3–0·5)**
**Central Europe, eastern Europe, and central Asia**			**1 510 000 (1 280 000–1 880 000)**	**361·2 (305·4–449·7)**	**328 000 000 (206 000 000–390 000 000)**	**78·4 (49·3–93·2)**	**67·7% (45·7–77·8)**	**10·1% (8·3–15·6)**	**2·0% (1·5–2·5)**	**0·5% (0·4–0·8)**
Central Asia			217 000 (157 000–317 000)	231·8 (168·1–338·5)	68 900 000 (40 900 000–95 600 000)	73·7 (43·7–102·2)	64·5% (41·1–84·7)	6·2% (4·2–10·2)	1·0% (0·8–1·7)	0·4% (0·2–0·6)
	Armenia		15 900 (11 500–22 700)	525·0 (379·6–753·1)	2 940 000 (1 790 000–3 610 000)	97·5 (59·4–119·7)	81·4% (55·7–90·3)	11·8% (9·3–18·8)	1·7% (1·2–2·6)	0·6% (0·4–1·0)
	Azerbaijan		41 500 (30 000–59 600)	403·6 (291·7–579·4)	9 560 000 (4 640 000–12 600 000)	93·0 (45·2–122·7)	77·4% (42·3–90·9)	6·5% (4·6–12·5)	1·2% (0·8–2·0)	0·5% (0·3–1·0)
	Georgia		11 900 (11 000–16 400)	325·9 (299·4–448·3)	3 460 000 (1 580 000–4 400 000)	94·5 (43·0–120·0)	79·4% (40·4–94·2)	26·6% (18·8–52·9)	1·8% (1·3–3·0)	0·4% (0·3–0·9)
	Kazakhstan		49 800 (36 000–69 500)	270·8 (195·7–377·9)	12 200 000 (6 320 000–20 100 000)	66·2 (34·3–109·2)	58·6% (33·0–86·1)	9·8% (5·2–16·7)	1·2% (0·9–2·0)	0·5% (0·2–0·9)
	Kyrgyzstan		18 700 (13 400–27 600)	286·3 (205·3–421·8)	5 570 000 (4 150 000–7 640 000)	85·3 (63·6–116·9)	76·1% (61·2–92·1)	3·4% (2·4–4·4)	0·9% (0·6–1·6)	0·4% (0·2–0·6)
	Mongolia		4540 (3590–5970)	134·1 (106·0–176·2)	2 190 000 (1 510 000–2 490 000)	64·6 (44·5–73·6)	58·2% (41·6–64·2)	30·7% (26·5–43·7)	0·8% (0·6–1·5)	0·2% (0·2–0·3)
	Tajikistan		10 400 (5710–17 900)	110·0 (60·2–188·5)	7 650 000 (5 120 000–9 960 000)	80·6 (53·9–104·9)	69·9% (50·1–84·7)	1·2% (0·4–2·2)	0·7% (0·5–1·3)	0·2% (0·1–0·3)
	Turkmenistan		9750 (5190–16 500)	191·9 (102·2–324·3)	4 100 000 (2 740 000–5 330 000)	80·6 (53·9–104·9)	70·0% (50·3–84·7)	4·3% (1·7–8·1)	1·1% (0·8–1·9)	0·3% (0·1–0·5)
	Uzbekistan		54 300 (38 400–80 000)	161·1 (114·0–237·6)	21 300 000 (7 470 000–33 200 000)	63·2 (22·2–98·5)	56·8% (21·7–82·8)	1·0% (0·6–2·6)	0·8% (0·6–1·5)	0·3% (0·1–0·8)
Central Europe			506 000 (393 000–681 000)	442·6 (344·0–596·5)	74 600 000 (54 100 000–92 200 000)	65·3 (47·4–80·7)	59·5% (44·3–72·7)	18·1% (14·4–24·4)	1·7% (1·2–2·4)	0·8% (0·5–1·1)
	Albania		14 000 (10 000–19 900)	513·7 (367·4–731·2)	2 780 000 (1 890 000–3 120 000)	102·1 (69·5–114·8)	86·9% (64·0–92·7)	7·3% (6·3–10·5)	1·4% (1·0–2·0)	0·5% (0·4–0·8)
	Bosnia and Herzegovina		16 300 (12 000–22 700)	493·5 (364·4–688·8)	2 870 000 (2 070 000–3 540 000)	87·0 (62·8–107·1)	76·7% (57·8–90·4)	9·7% (7·7–13·2)	1·7% (1·2–2·5)	0·6% (0·4–0·9)
	Bulgaria		61 700 (48 000–83 000)	889·1 (692·8–1197·5)	6 020 000 (3 290 000–7 880 000)	86·8 (47·4–113·7)	74·3% (44·8–89·6)	12·1% (8·7–20·9)	2·0% (1·5–2·9)	1·2% (0·7–2·1)
	Croatia		15 900 (12 200–20 900)	374·5 (287·7–492·7)	2 570 000 (2 160 000–3 430 000)	60·5 (50·8–80·8)	55·5% (47·5–71·1)	23·3% (17·2–27·4)	2·2% (1·6–2·6)	0·7% (0·5–1·0)
	Czechia		38 400 (31 500–49 300)	360·7 (295·7–462·8)	7 980 000 (5 730 000–9 530 000)	75·0 (53·8–89·5)	70·1% (51·6–81·5)	26·4% (21·8–36·1)	1·9% (1·6–2·7)	0·5% (0·4–0·7)
	Hungary		39 100 (32 100–51 600)	404·5 (331·6–533·4)	5 620 000 (3 980 000–7 420 000)	58·1 (41·2–76·7)	53·6% (38·9–69·4)	19·4% (14·1–26·8)	2·0% (1·5–2·9)	0·8% (0·5–1·2)
	Montenegro		3300 (2470–4670)	532·1 (397·5–753·3)	686 000 (644 000–731 000)	110·5 (103·8–117·9)	89·4% (87·9–90·5)	22·8% (21·3–24·2)	1·4% (1·0–1·9)	0·5% (0·4–0·7)
	North Macedonia		16 200 (11 800–22 200)	753·1 (550·2–1033·6)	2 110 000 (1 500 000–2 440 000)	97·9 (69·9–113·6)	84·2% (64·0–91·6)	10·3% (8·7–14·2)	1·4% (1·0–2·0)	0·8% (0·5–1·2)
	Poland		153 000 (116 000–205 000)	397·3 (303·0–534·6)	20 500 000 (10 300 000–30 000 000)	53·3 (26·7–77·9)	50·6% (26·1–71·9)	18·1% (11·5–33·8)	1·4% (0·9–2·7)	0·9% (0·5–1·6)
	Romania		94 900 (71 600–133 000)	493·2 (372·1–691·9)	13 400 000 (9 130 000–17 900 000)	69·6 (47·5–93·0)	62·1% (44·0–80·9)	13·7% (9·9–19·4)	1·8% (1·3–2·6)	0·8% (0·5–1·3)
	Serbia		28 100 (20 900–40 300)	321·8 (239·1–460·2)	6 400 000 (4 450 000–8 410 000)	73·2 (50·9–96·2)	64·5% (47·3–81·1)	20·0% (14·8–28·0)	1·7% (1·2–2·4)	0·5% (0·3–0·7)
	Slovakia		19 300 (16 000–23 100)	354·9 (293·7–424·2)	2 760 000 (2 060 000–4 250 000)	50·8 (37·9–78·2)	47·9% (36·5–71·4)	41·9% (26·3–54·4)	1·8% (1·3–2·6)	0·8% (0·5–1·1)
	Slovenia		5640 (4930–6670)	272·1 (237·5–321·4)	936 000 (634 000–1 550 000)	45·1 (30·6–74·8)	42·2% (29·1–66·4)	48·8% (26·7–65·9)	1·9% (1·4–2·8)	0·7% (0·4–1·1)
Eastern Europe			786 000 (725 000–881 000)	374·5 (345·1–419·5)	184 000 000 (66 200 000–227 000 000)	87·7 (31·5–108·1)	73·6% (30·1–87·3)	9·2% (6·5–22·4)	2·5% (1·8–3·2)	0·5% (0·4–1·4)
	Belarus		66 100 (47 800–95 400)	695·8 (503·1–1004·2)	6 430 000 (2 520 000–10 700 000)	67·7 (26·6–112·9)	59·4% (25·7–90·6)	11·9% (6·0–25·7)	2·8% (2·0–3·6)	1·3% (0·6–2·7)
	Estonia		4520 (3750–5150)	344·4 (285·7–392·4)	425 000 (326 000–760 000)	32·4 (24·8–57·9)	30·9% (24·1–53·2)	53·9% (31·8–67·7)	3·1% (1·8–3·9)	1·2% (0·7–1·5)
	Latvia		9340 (7250–11 800)	487·8 (378·7–616·4)	1 050 000 (530 000–1 900 000)	55·1 (27·7–99·2)	49·8% (26·7–83·1)	26·6% (13·2–47·3)	3·0% (2·1–3·9)	1·2% (0·6–2·0)
	Lithuania		15 400 (12 200–19 000)	551·9 (435·2–680·8)	1 930 000 (1 110 000–2 870 000)	69·2 (39·6–102·7)	61·1% (37·2–82·6)	25·8% (16·1–41·9)	2·8% (2·0–3·6)	0·9% (0·5–1·5)
	Moldova		12 700 (9210–18 500)	344·0 (249·7–502·6)	3 040 000 (1 700 000–4 070 000)	82·5 (46·0–110·4)	73·1% (43·2–91·7)	12·6% (8·9–21·3)	2·4% (1·7–3·1)	0·5% (0·3–0·8)
	Russia		552 000 (552 000–552 000)	376·4 (376·4–376·4)	139 000 000 (36 800 000–166 000 000)	94·5 (25·1–113·4)	78·2% (24·3–87·2)	8·1% (5·6–25·4)	2·5% (1·7–3·2)	0·5% (0·4–1·7)
	Ukraine		126 000 (92 000–180 000)	286·1 (208·8–408·2)	32 600 000 (16 800 000–50 200 000)	73·9 (38·1–114·0)	64·4% (36·5–90·6)	12·1% (7·1–21·2)	2·8% (2·0–3·6)	0·5% (0·3–0·9)
**High income**			**2 330 000 (1 990 000–2 770 000)**	**214·9 (183·5–256·0)**	**239 000 000 (226 000 000–252 000 000)**	**22·1 (20·9–23·3)**	**21·3% (20·1–22·5)**	**44·6% (42·3–47·2)**	**3·3% (3·1–3·5)**	**1·0% (0·9–1·2)**
Australasia			1920 (1920–1920)	6·6 (6·6–6·6)	356 000 (328 000–392 000)	1·2 (1·1–1·3)	1·2% (1·1–1·3)	57·4% (51·8–62·1)	2·5% (2·2–2·8)	0·6% (0·5–0·6)
	Australia		1890 (1890–1890)	7·7 (7·7–7·7)	340 000 (313 000–375 000)	1·4 (1·3–1·5)	1·4% (1·3–1·5)	57·6% (52·0–62·5)	2·6% (2·3–2·9)	0·6% (0·5–0·7)
	New Zealand		36 (36–36)	0·8 (0·8–0·8)	16 000 (13 900–18 200)	0·4 (0·3–0·4)	0·3% (0·3–0·4)	53·7% (47·3–60·8)	1·5% (1·4–1·6)	0·3% (0·3–0·4)
High-income Asia Pacific			102 000 (79 400–138 000)	54·3 (42·4–73·9)	7 890 000 (6 630 000–9 480 000)	4·2 (3·5–5·1)	4·2% (3·5–5·0)	31·1% (25·6–36·6)	4·1% (3·7–4·7)	1·3% (1·0–1·7)
	Brunei		125 (96–194)	28·5 (21·9–44·3)	35 400 (27 400–50 800)	8·1 (6·3–11·6)	7·8% (5·9–11·3)	43·3% (29·4–54·4)	1·7% (1·4–1·9)	0·4% (0·3–0·5)
	Japan		97 700 (75 600–135 000)	76·5 (59·2–105·3)	6 450 000 (5 080 000–8 020 000)	5·0 (4·0–6·3)	5·0% (4·0–6·2)	27·1% (21·5–33·9)	4·5% (3·9–5·0)	1·5% (1·1–2·1)
	Singapore		585 (585–585)	10·3 (10·3–10·3)	408 000 (355 000–501 000)	7·2 (6·3–8·8)	7·1% (6·2–8·7)	64·0% (51·6–72·8)	2·4% (2·1–2·7)	0·2% (0·2–0·2)
	South Korea		3260 (3110–4420)	6·1 (5·8–8·3)	993 000 (744 000–1 260 000)	1·9 (1·4–2·4)	1·8% (1·4–2·3)	44·6% (34·7–58·0)	2·6% (2·3–2·9)	0·4% (0·3–0·6)
High-income North America			1 020 000 (857 000–1 220 000)	278·7 (235·0–333·5)	118 000 000 (109 000 000–125 000 000)	32·3 (29·9–34·2)	30·9% (28·8–32·8)	42·3% (39·8–45·6)	3·4% (3·2–3·7)	0·9% (0·7–1·1)
	Canada		38 700 (31 400–46 600)	106·0 (86·0–127·7)	4 620 000 (3 700 000–5 710 000)	12·7 (10·1–15·6)	12·4% (9·9–15·2)	38·9% (31·2–48·1)	1·8% (1·4–2·2)	0·9% (0·7–1·2)
		Alberta	4880 (3520–6280)	114·8 (82·7–147·6)	760 000 (570 000–1 160 000)	17·9 (13·4–27·3)	17·4% (13·2–26·5)	45·4% (29·0–58·6)	2·2% (1·4–2·8)	0·7% (0·4–0·9)
		British Columbia	4280 (3190–5470)	87·0 (64·8–111·0)	707 000 (494 000–952 000)	14·4 (10·0–19·3)	14·1% (9·9–18·8)	31·6% (22·8–43·8)	1·8% (1·3–2·5)	0·7% (0·4–1·0)
		Manitoba	1930 (1330–3120)	145·3 (100·1–235·1)	505 000 (178 000–728 000)	38·0 (13·4–54·9)	36·1% (13·1–50·7)	14·9% (9·2–37·6)	1·1% (0·6–2·7)	0·5% (0·2–1·1)
		New Brunswick	262 (183–353)	34·9 (24·4–47·0)	30 700 (20 400–54 000)	4·1 (2·7–7·2)	3·6% (2·3–6·7)	27·7% (14·8–39·3)	1·4% (1·0–1·9)	1·0% (0·5–1·4)
		Newfoundland and Labrador	85 (48–121)	17·0 (9·5–24·2)	9400 (6320–15 500)	1·9 (1·3–3·1)	1·6% (1·0–2·6)	22·9% (13·2–32·2)	1·5% (1·1–1·9)	1·2% (0·7–1·8)
		Northwest Territories	28 (21–37)	65·4 (49·6–84·8)	5010 (3650–7060)	11·6 (8·4–16·3)	8·1% (4·7–13·3)	42·0% (28·9–55·7)	1·5% (1·1–2·0)	0·6% (0·4–0·9)
		Nova Scotia	276 (179–371)	29·3 (19·0–39·4)	23 800 (17 500–34 300)	2·5 (1·9–3·6)	2·5% (1·8–3·6)	35·5% (23·6–46·5)	1·6% (1·1–2·1)	1·3% (0·8–1·9)
		Nunavut	14 (10–17)	36·4 (27·7–45·2)	2130 (1630–2840)	5·6 (4·3–7·5)	4·7% (2·7–6·0)	32·2% (23·5–41·1)	1·5% (1·1–2·0)	0·7% (0·4–0·9)
		Ontario	13 600 (10 100–16 900)	95·7 (71·0–118·9)	1 520 000 (1 060 000–2 070 000)	10·7 (7·5–14·6)	10·6% (7·4–14·4)	41·7% (29·7–57·9)	2·1% (1·5–3·0)	0·9% (0·6–1·3)
		Quebec	12 000 (11 500–14 300)	145·6 (140·0–174·4)	849 000 (630 000–1 150 000)	10·3 (7·7–14·0)	10·2% (7·6–13·9)	54·0% (38·6–70·3)	1·5% (1·2–2·0)	1·5% (1·0–2·1)
		Saskatchewan	1370 (1030–1790)	120·6 (90·2–157·5)	208 000 (144 000–304 000)	18·3 (12·7–26·7)	17·5% (12·1–25·1)	40·2% (26·6–56·0)	1·6% (1·1–2·1)	0·7% (0·4–1·0)
		Yukon	22 (17–27)	56·1 (42·7–68·0)	5570 (4120–7780)	14·1 (10·4–19·7)	6·2% (1·7–16·1)	27·0% (18·8–35·6)	1·2% (0·9–1·7)	0·6% (0·4–0·9)
	USA		977 000 (826 000–1 170 000)	298·0 (251·9–356·8)	113 000 000 (105 000 000–120 000 000)	34·5 (32·0–36·6)	33·0% (30·8–35·1)	42·4% (40·0–45·7)	3·5% (3·3–3·8)	0·9% (0·7–1·1)
		Alabama	24 100 (19 500–29 600)	485·1 (390·9–594·0)	2 070 000 (1 710 000–2 430 000)	41·5 (34·3–48·9)	39·3% (32·7–45·3)	41·2% (34·7–49·5)	4·1% (3·4–4·9)	1·2% (0·9–1·5)
		Alaska	1350 (1060–1650)	171·1 (134·0–209·6)	281 000 (228 000–364 000)	35·6 (29·0–46·2)	33·6% (27·6–42·6)	53·9% (41·1–65·6)	1·9% (1·4–2·3)	0·5% (0·4–0·7)
		Arizona	26 200 (22 300–31 000)	361·7 (307·5–427·6)	2 560 000 (2 020 000–3 080 000)	35·3 (27·9–42·6)	33·8% (27·0–40·8)	49·7% (40·8–62·2)	4·0% (3·3–5·0)	1·1% (0·9–1·4)
		Arkansas	12 400 (10 300–14 700)	406·2 (335·4–480·7)	1 090 000 (831 000–1 310 000)	35·7 (27·2–42·7)	34·1% (26·2–40·5)	48·8% (40·2–63·3)	4·2% (3·4–5·4)	1·2% (0·9–1·5)
		California	98 600 (81 400–118 000)	247·3 (204·2–296·0)	11 000 000 (8 960 000–13 200 000)	27·5 (22·5–33·2)	26·7% (22·0–32·3)	46·6% (38·2–56·5)	3·3% (2·7–4·0)	0·9% (0·7–1·2)
		Colorado	9780 (8640–11 500)	181·1 (160·0–213·6)	1 670 000 (1 300 000–2 140 000)	30·9 (24·1–39·6)	29·6% (23·4–37·4)	50·1% (38·3–63·3)	3·6% (2·7–4·5)	0·7% (0·5–0·8)
		Connecticut	9240 (8790–10 500)	250·2 (238·0–285·4)	814 000 (659 000–999 000)	22·0 (17·8–27·1)	21·6% (17·6–26·5)	52·0% (41·8–63·3)	4·1% (3·3–5·0)	1·2% (0·9–1·5)
		Delaware	3130 (2690–3660)	320·8 (276·2–375·5)	268 000 (224 000–327 000)	27·5 (22·9–33·5)	26·6% (22·2–32·3)	57·4% (46·5–68·2)	4·2% (3·4–5·0)	1·2% (0·9–1·5)
		Washington, DC	1190 (1190–1190)	182·8 (182·8–182·8)	154 000 (120 000–191 000)	23·7 (18·4–29·4)	23·2% (18·0–28·5)	43·7% (34·9–55·5)	6·0% (4·8–7·6)	0·8% (0·6–1·0)
		Florida	77 000 (64 100–93 900)	363·5 (302·7–443·5)	9 200 000 (7 830 000–10 700 000)	43·5 (37·0–50·5)	40·8% (35·1–47·4)	40·8% (34·8–47·6)	3·9% (3·3–4·5)	0·9% (0·7–1·1)
		Georgia	38 200 (31 200–47 400)	358·5 (292·6–443·9)	4 250 000 (3 540 000–5 070 000)	39·8 (33·2–47·6)	37·8% (32·2–44·4)	39·4% (32·7–46·8)	4·3% (3·6–5·1)	0·9% (0·7–1·2)
		Hawaii	1050 (1040–1200)	70·8 (69·9–80·4)	149 000 (119 000–207 000)	10·0 (8·0–13·9)	9·8% (7·8–13·6)	59·8% (42·0–73·1)	4·9% (3·5–6·0)	0·7% (0·5–0·9)
		Idaho	5120 (4130–6160)	294·8 (237·9–355·0)	773 000 (579 000–1 010 000)	44·5 (33·4–58·4)	41·7% (32·0–54·0)	40·4% (30·2–52·8)	2·2% (1·7–2·9)	0·7% (0·5–1·0)
		Illinois	36 800 (28 900–46 100)	282·1 (221·5–353·5)	4 960 000 (4 220 000–5 760 000)	38·0 (32·3–44·2)	36·5% (31·2–42·4)	36·3% (31·1–42·5)	2·6% (2·2–3·1)	0·8% (0·6–1·0)
		Indiana	18 200 (17 100–20 900)	270·3 (254·2–311·2)	2 140 000 (1 610 000–2 760 000)	31·9 (24·0–41·1)	30·6% (23·5–39·1)	52·0% (39·3–67·6)	4·0% (3·1–5·3)	0·9% (0·7–1·2)
		Iowa	7270 (7210–7940)	232·3 (230·3–253·7)	1 340 000 (1 050 000–1 660 000)	42·7 (33·5–52·9)	40·6% (32·3–49·4)	39·5% (31·5–49·7)	2·5% (2·0–3·2)	0·6% (0·5–0·7)
		Kansas	8220 (6690–9860)	276·9 (225·3–332·0)	1 030 000 (797 000–1 300 000)	34·7 (26·8–43·8)	33·3% (26·0–41·9)	45·5% (35·5–57·8)	3·4% (2·7–4·4)	0·8% (0·6–1·1)
		Kentucky	16 000 (13 000–19 400)	356·0 (289·5–430·6)	1 710 000 (1 350 000–2 170 000)	37·9 (30·1–48·2)	35·9% (29·0–44·9)	46·3% (36·0–57·5)	6·5% (5·1–8·1)	1·0% (0·7–1·3)
		Louisiana	20 000 (16 400–24 100)	436·3 (356·4–525·4)	1 720 000 (1 380 000–2 040 000)	37·5 (30·1–44·4)	35·9% (29·1–41·4)	45·1% (37·7–55·7)	4·6% (3·8–5·7)	1·2% (0·9–1·6)
		Maine	1970 (1370–2580)	145·7 (101·5–190·6)	209 000 (158 000–308 000)	15·4 (11·7–22·7)	15·0% (11·5–21·9)	58·7% (38·4–74·9)	2·9% (1·9–3·7)	1·1% (0·6–1·6)
		Maryland	16 500 (13 100–20 600)	268·2 (212·9–335·1)	1 590 000 (1 210 000–2 040 000)	25·9 (19·8–33·3)	25·2% (19·4–32·1)	37·2% (28·4–48·0)	4·0% (3·1–5·2)	1·1% (0·8–1·4)
		Massachusetts	19 200 (19 200–19 200)	287·5 (287·5–287·5)	1 420 000 (1 240 000–1 760 000)	21·3 (18·6–26·4)	20·8% (18·2–25·8)	64·5% (51·6–73·2)	3·6% (2·9–4·1)	1·4% (1·1–1·6)
		Michigan	26 300 (24 400–31 500)	270·6 (250·9–324·3)	3 330 000 (2 840 000–3 890 000)	34·2 (29·3–40·0)	32·8% (28·4–37·7)	44·2% (37·6–51·6)	3·4% (2·9–4·0)	0·9% (0·7–1·1)
		Minnesota	9150 (9130–9370)	164·4 (164·1–168·3)	1 820 000 (1 450 000–2 280 000)	32·7 (26·0–41·0)	31·3% (25·2–39·2)	49·9% (39·3–61·5)	2·4% (1·9–3·0)	0·5% (0·4–0·7)
		Mississippi	16 000 (12 700–19 900)	531·6 (424·3–662·8)	1 250 000 (1 030 000–1 460 000)	41·5 (34·2–48·7)	39·4% (32·8–45·5)	41·5% (35·0–49·9)	3·3% (2·8–4·0)	1·3% (1·0–1·6)
		Missouri	18 600 (15 100–22 500)	298·2 (242·6–360·0)	2 280 000 (1 870 000–2 860 000)	36·6 (29·9–45·8)	35·0% (28·9–43·2)	40·1% (31·6–48·4)	3·3% (2·6–4·0)	0·8% (0·6–1·1)
		Montana	2960 (2590–3550)	284·6 (248·7–341·4)	408 000 (300 000–545 000)	39·2 (28·9–52·4)	36·9% (27·3–48·4)	47·8% (34·8–63·3)	5·0% (3·6–6·6)	0·8% (0·6–1·1)
		Nebraska	3870 (3020–4740)	202·4 (157·8–247·4)	819 000 (630 000–994 000)	42·8 (32·9–51·9)	40·6% (31·5–48·2)	37·9% (30·7–48·5)	2·3% (1·9–3·0)	0·5% (0·4–0·7)
		Nevada	8750 (7820–10 300)	272·1 (243·1–320·1)	1 320 000 (1 090 000–1 530 000)	41·0 (34·0–47·6)	38·8% (32·8–45·0)	34·9% (29·8–41·7)	3·3% (2·8–4·0)	0·7% (0·6–0·9)
		New Hampshire	2700 (2200–3190)	199·3 (162·4–236·1)	260 000 (211 000–338 000)	19·2 (15·6–25·0)	18·7% (15·3–24·0)	61·2% (46·7–74·0)	3·0% (2·3–3·6)	1·1% (0·8–1·5)
		New Jersey	31 000 (28 100–38 100)	343·1 (311·0–422·0)	3 130 000 (2 610 000–3 660 000)	34·6 (28·8–40·5)	33·7% (28·2–39·1)	40·0% (33·9–47·6)	3·8% (3·2–4·5)	1·0% (0·8–1·3)
		New Mexico	7640 (6170–9100)	349·8 (282·3–416·3)	666 000 (535 000–824 000)	30·5 (24·5–37·7)	29·3% (23·8–35·9)	47·2% (37·6–57·8)	3·7% (3·0–4·5)	1·2% (0·9–1·6)
		New York	68 800 (56 800–84 200)	347·7 (286·8–425·4)	6 550 000 (5 760 000–7 660 000)	33·1 (29·1–38·7)	32·3% (28·3–37·7)	41·4% (35·2–47·0)	4·0% (3·4–4·6)	1·1% (0·9–1·4)
		North Carolina	29 300 (24 000–35 300)	276·6 (227·1–333·7)	3 450 000 (2 730 000–4 160 000)	32·6 (25·7–39·3)	31·2% (24·8–37·1)	44·8% (36·6–55·9)	2·8% (2·3–3·4)	0·9% (0·7–1·2)
		North Dakota	1850 (1850–1850)	260·8 (260·8–260·8)	280 000 (219 000–377 000)	39·5 (30·9–53·3)	37·4% (29·5–49·5)	58·6% (42·4–73·1)	3·3% (2·4–4·1)	0·7% (0·5–0·9)
		Ohio	35 800 (28 500–45 800)	309·6 (246·5–395·3)	4 890 000 (3 800 000–6 040 000)	42·3 (32·8–52·1)	39·9% (31·3–48·5)	34·6% (27·7–44·0)	3·0% (2·4–3·8)	0·8% (0·6–1·1)
		Oklahoma	15 900 (12 900–19 000)	403·1 (327·4–481·9)	1 550 000 (1 130 000–2 030 000)	39·3 (28·7–51·4)	37·3% (27·9–48·5)	43·8% (32·7–58·5)	5·2% (3·9–7·0)	1·1% (0·8–1·5)
		Oregon	8640 (6780–10 200)	214·3 (168·3–253·5)	814 000 (538 000–1 230 000)	20·2 (13·3–30·6)	19·6% (13·1–29·1)	50·6% (31·6–72·2)	2·8% (1·7–4·0)	1·2% (0·7–1·9)
		Pennsylvania	34 500 (32 300–41 400)	265·7 (248·6–318·7)	3 990 000 (3 270 000–4 900 000)	30·7 (25·2–37·7)	29·7% (24·6–36·3)	43·2% (34·9–52·1)	3·9% (3·1–4·6)	0·9% (0·7–1·1)
		Rhode Island	2880 (2880–2880)	276·0 (276·0–276·0)	273 000 (249 000–322 000)	26·2 (23·8–30·8)	25·5% (23·3–29·9)	69·5% (58·9–76·2)	2·5% (2·1–2·7)	1·1% (0·9–1·2)
		South Carolina	20 900 (17 900–25 000)	414·9 (354·4–496·5)	1 890 000 (1 540 000–2 290 000)	37·5 (30·6–45·4)	35·6% (29·5–42·6)	48·8% (40·0–59·3)	3·2% (2·6–3·9)	1·1% (0·9–1·5)
		South Dakota	2340 (2270–2670)	270·4 (262·8–308·7)	366 000 (267 000–505 000)	42·3 (30·8–58·4)	40·3% (29·7–54·8)	46·2% (32·6–61·5)	3·2% (2·2–4·2)	0·7% (0·5–0·9)
		Tennessee	22 000 (18 500–26 100)	327·3 (275·7–388·4)	2 730 000 (2 230 000–3 340 000)	40·5 (33·1–49·7)	38·5% (31·9–46·6)	48·6% (39·2–58·9)	3·2% (2·6–3·8)	0·9% (0·7–1·1)
		Texas	98 400 (78 600–124 000)	346·2 (276·6–436·6)	13 000 000 (10 700 000–15 200 000)	45·7 (37·6–53·6)	43·0% (35·4–50·3)	33·7% (28·5–40·6)	3·3% (2·8–3·9)	0·8% (0·6–1·1)
		Utah	3940 (3360–4680)	125·3 (106·8–148·5)	1 370 000 (1 120 000–1 740 000)	43·7 (35·5–55·4)	41·0% (34·0–50·4)	43·7% (33·8–53·0)	2·0% (1·5–2·4)	0·3% (0·2–0·4)
		Vermont	1650 (1210–2030)	263·3 (193·5–323·2)	73 700 (65 100–105 000)	11·7 (10·4–16·7)	11·4% (9·7–16·2)	66·9% (46·8–74·4)	3·0% (2·1–3·3)	2·4% (1·7–3·1)
		Virginia	21 100 (18 100–24 400)	242·4 (207·6–280·3)	1 850 000 (1 380 000–2 520 000)	21·3 (15·8–29·0)	20·8% (15·5–27·9)	53·1% (38·3–69·8)	3·7% (2·6–4·8)	1·2% (0·8–1·7)
		Washington	11 200 (9010–13 800)	152·8 (123·4–188·6)	1 320 000 (1 040 000–1 810 000)	18·1 (14·3–24·8)	17·6% (14·0–23·8)	59·7% (42·7–74·0)	3·2% (2·3–4·0)	0·9% (0·6–1·2)
		West Virginia	7570 (6260–8590)	407·0 (336·7–461·6)	580 000 (405 000–818 000)	31·2 (21·8–44·0)	29·8% (21·1–41·4)	52·2% (35·6–72·0)	3·9% (2·6–5·3)	1·5% (0·9–2·1)
		Wisconsin	10 300 (9670–12 000)	174·4 (164·4–204·2)	2 300 000 (1 820 000–2 800 000)	39·0 (31·0–47·7)	37·2% (29·9–45·3)	42·7% (34·4–53·2)	3·5% (2·8–4·3)	0·5% (0·4–0·6)
		Wyoming	1680 (1330–2030)	275·8 (217·4–333·2)	291 000 (205 000–402 000)	47·7 (33·5–65·8)	44·0% (31·8–61·1)	39·1% (27·5–54·0)	2·9% (2·0–4·0)	0·7% (0·4–0·9)
Southern Latin America			176 000 (161 000–235 000)	263·3 (240·8–351·5)	17 300 000 (13 000 000–24 000 000)	25·9 (19·5–36·0)	25·1% (19·2–34·0)	44·1% (31·1–57·4)	2·5% (1·8–3·3)	1·0% (0·8–1·4)
	Argentina		129 000 (116 000–184 000)	285·8 (257·3–407·9)	12 600 000 (8 920 000–18 500 000)	27·9 (19·8–41·0)	27·0% (19·6–38·4)	43·5% (28·7–59·6)	1·9% (1·3–2·6)	1·1% (0·7–1·5)
	Chile		38 000 (37 900–37 900)	208·7 (208·5–208·5)	3 940 000 (3 090 000–4 870 000)	21·7 (17·0–26·8)	21·1% (16·6–25·8)	45·1% (35·9–56·8)	4·1% (3·3–5·2)	1·0% (0·8–1·3)
	Uruguay		8840 (6720–12 800)	257·4 (195·7–372·0)	788 000 (626 000–961 000)	22·9 (18·2–28·0)	22·3% (17·9–27·1)	51·5% (41·5–63·7)	4·1% (3·2–5·3)	1·1% (0·9–1·5)
Western Europe			1 030 000 (888 000–1 210 000)	237·0 (203·5–277·0)	96 000 000 (88 100 000–105 000 000)	22·0 (20·2–24·2)	21·4% (19·6–23·5)	48·9% (44·3–53·1)	3·3% (3·0–3·5)	1·1% (1·0–1·4)
	Andorra		276 (223–356)	331·8 (268·9–428·5)	40 400 (28 200–52 000)	48·6 (33·9–62·6)	46·4% (32·7–59·1)	42·0% (32·0–59·2)	3·2% (2·9–3·5)	0·7% (0·5–1·1)
	Austria		14 700 (12 300–16 600)	164·6 (137·5–185·7)	2 040 000 (1 730 000–2 660 000)	22·9 (19·4–29·9)	22·2% (18·9–28·7)	55·2% (41·7–64·1)	3·6% (3·1–4·1)	0·8% (0·6–1·0)
	Belgium		28 300 (26 400–34 000)	248·1 (231·6–297·6)	3 820 000 (3 080 000–4 680 000)	33·4 (27·0–41·0)	32·0% (26·1–38·9)	44·6% (35·6–54·6)	2·4% (1·9–2·9)	0·8% (0·6–1·0)
	Cyprus		732 (592–1010)	55·7 (45·1–77·0)	186 000 (179 000–198 000)	14·2 (13·6–15·1)	13·9% (13·3–14·8)	70·9% (66·4–73·8)	3·2% (2·6–3·7)	0·4% (0·3–0·6)
	Denmark		8600 (7250–9790)	148·2 (125·0–168·7)	799 000 (701 000–936 000)	13·8 (12·1–16·1)	13·5% (11·9–15·9)	59·0% (49·8–66·3)	2·5% (2·1–2·9)	1·2% (0·9–1·4)
	Finland		6170 (4680–7630)	111·5 (84·5–137·9)	484 000 (413 000–589 000)	8·7 (7·5–10·6)	8·6% (7·3–10·5)	38·1% (31·2–44·0)	3·5% (3·0–4·1)	1·5% (1·2–1·8)
	France		136 000 (115 000–165 000)	205·0 (173·7–249·5)	15 800 000 (12 300 000–23 300 000)	23·9 (18·6–35·2)	23·3% (18·1–33·8)	49·0% (32·5–61·5)	3·7% (2·5–4·7)	0·9% (0·6–1·3)
	Germany		160 000 (129 000–199 000)	188·6 (151·6–234·8)	12 400 000 (11 200 000–13 800 000)	14·6 (13·2–16·3)	14·4% (12·9–16·0)	45·0% (40·2–49·7)	3·0% (2·7–3·3)	1·4% (1·1–1·7)
		Baden-Württemberg	19 600 (14 900–25 400)	174·5 (132·1–225·6)	1 640 000 (1 370 000–1 970 000)	14·5 (12·2–17·6)	14·2% (11·9–17·2)	48·6% (39·9–57·9)	2·8% (2·3–3·3)	1·3% (1·0–1·9)
		Bavaria	26 000 (19 800–33 300)	196·0 (148·9–251·1)	2 240 000 (1 810 000–2 730 000)	16·9 (13·6–20·6)	16·5% (13·4–19·9)	48·5% (39·1–58·9)	2·5% (2·0–3·0)	1·3% (1·0–1·7)
		Berlin	7010 (5370–9110)	192·7 (147·7–250·5)	591 000 (514 000–667 000)	16·3 (14·1–18·3)	15·9% (13·9–17·9)	45·0% (39·6–51·8)	3·0% (2·6–3·4)	1·2% (0·9–1·6)
		Brandenburg	5490 (4460–6720)	213·7 (173·8–261·7)	387 000 (334 000–442 000)	15·1 (13·0–17·2)	14·8% (12·8–16·8)	42·9% (37·3–49·2)	2·8% (2·4–3·2)	1·5% (1·3–1·9)
		Bremen	1120 (848–1410)	161·4 (122·2–203·8)	91 000 (79 700–107 000)	13·1 (11·5–15·4)	12·9% (11·3–15·2)	42·7% (36·0–48·6)	3·3% (2·8–3·7)	1·3% (1·0–1·7)
		Hamburg	3190 (2490–4170)	173·0 (134·7–225·6)	240 000 (203 000–286 000)	13·0 (11·0–15·5)	12·8% (10·9–15·2)	46·7% (38·6–54·9)	2·6% (2·1–3·0)	1·4% (1·1–1·8)
		Hesse	12 700 (10 000–16 100)	198·5 (157·3–252·5)	926 000 (825 000–1 070 000)	14·5 (12·9–16·8)	14·3% (12·8–16·4)	43·5% (37·4–48·7)	3·3% (2·8–3·6)	1·4% (1·2–1·8)
		Lower Saxony	11 500 (8900–13 800)	140·1 (108·6–168·4)	769 000 (610 000–872 000)	9·4 (7·4–10·7)	9·3% (7·4–10·6)	47·7% (41·6–59·7)	2·5% (2·2–3·1)	1·6% (1·2–2·1)
		Mecklenburg-Vorpommern	2630 (1900–3330)	158·2 (113·9–199·8)	173 000 (150 000–204 000)	10·4 (9·0–12·2)	10·2% (8·9–12·1)	38·6% (32·5–44·1)	3·5% (3·0–4·0)	1·7% (1·2–2·1)
		North Rhine-Westphalia	34 400 (26 800–44 000)	186·4 (145·1–238·2)	2 790 000 (2 510 000–3 150 000)	15·1 (13·6–17·1)	14·9% (13·4–16·8)	40·9% (36·2–45·4)	3·9% (3·5–4·4)	1·3% (1·0–1·7)
		Rhineland-Palatinate	6860 (5230–8620)	163·8 (125·0–205·8)	468 000 (382 000–581 000)	11·2 (9·1–13·9)	11·0% (9·0–13·6)	48·2% (38·8–58·9)	2·6% (2·1–3·2)	1·5% (1·2–2·0)
		Saarland	1770 (1340–2240)	172·1 (130·2–218·0)	127 000 (108 000–151 000)	12·4 (10·5–14·6)	12·1% (10·3–14·4)	46·3% (39·1–54·3)	2·3% (1·9–2·7)	1·5% (1·1–2·0)
		Saxony	13 200 (11 100–16 800)	312·3 (263·3–399·0)	983 000 (794 000–1 150 000)	23·3 (18·8–27·4)	22·6% (18·4–26·5)	46·6% (39·4–56·9)	2·7% (2·2–3·2)	1·5% (1·1–1·9)
		Saxony-Anhalt	4960 (4080–6060)	213·8 (175·9–261·1)	341 000 (302 000–388 000)	14·7 (13·0–16·7)	14·4% (12·8–16·4)	42·7% (37·3–48·0)	3·2% (2·8–3·6)	1·6% (1·3–2·0)
		Schleswig-Holstein	3730 (2870–4660)	126·2 (97·1–157·9)	222 000 (189 000–259 000)	7·5 (6·4–8·8)	7·4% (6·3–8·7)	42·5% (36·3–49·4)	3·2% (2·7–3·7)	1·8% (1·4–2·3)
		Thuringia	6010 (5100–7600)	267·9 (227·2–338·7)	442 000 (386 000–524 000)	19·7 (17·2–23·4)	19·2% (16·8–22·5)	45·0% (38·2–51·3)	3·3% (2·8–3·8)	1·5% (1·2–2·0)
	Greece		19 100 (16 800–22 600)	184·5 (162·1–218·9)	1 600 000 (1 430 000–1 790 000)	15·5 (13·9–17·3)	15·2% (13·6–16·9)	56·9% (50·8–63·9)	4·1% (3·5–4·8)	1·4% (1·1–1·6)
	Iceland		35 (35–35)	10·1 (10·1–10·1)	28 100 (25 000–33 300)	8·2 (7·3–9·6)	7·9% (7·0–9·4)	62·0% (52·3–69·0)	2·8% (2·3–3·2)	0·1% (0·1–0·1)
	Ireland		5570 (5570–5570)	113·5 (113·5–113·5)	1 130 000 (863 000–1 590 000)	23·0 (17·6–32·4)	22·3% (17·1–31·1)	49·6% (34·4–63·9)	1·3% (0·9–1·6)	0·5% (0·4–0·7)
	Israel		8670 (8130–9870)	93·2 (87·3–106·1)	2 330 000 (2 090 000–2 680 000)	25·0 (22·4–28·8)	24·3% (21·8–27·7)	57·7% (49·9–64·1)	2·5% (2·2–2·8)	0·4% (0·3–0·4)
	Italy		227 000 (182 000–278 000)	375·9 (301·6–460·3)	12 000 000 (9 360 000–17 700 000)	19·8 (15·5–29·4)	19·6% (15·4–28·6)	43·0% (28·2–53·2)	3·9% (3·4–4·4)	2·0% (1·3–2·7)
		Abruzzo	4020 (3160–4960)	306·3 (240·5–377·9)	198 000 (151 000–359 000)	15·1 (11·5–27·4)	14·9% (11·4–26·8)	46·1% (24·2–57·6)	4·2% (3·6–4·7)	2·2% (1·2–3·0)
		Basilicata	1500 (1130–1860)	266·3 (201·3–331·0)	79 300 (57 500–121 000)	14·1 (10·2–21·6)	13·9% (10·1–21·2)	41·1% (25·9–54·5)	3·9% (3·3–4·4)	2·0% (1·2–2·9)
		Calabria	5580 (4060–7240)	288·7 (210·1–374·2)	337 000 (241 000–537 000)	17·4 (12·5–27·8)	17·2% (12·4–27·1)	28·5% (17·1–38·1)	3·5% (2·9–4·1)	1·8% (1·1–2·6)
		Campania	16 100 (12 500–19 600)	278·3 (217·0–340·0)	1 010 000 (734 000–1 630 000)	17·5 (12·7–28·3)	17·3% (12·6–27·6)	50·3% (29·8–66·2)	3·5% (2·9–4·0)	1·7% (1·0–2·4)
		Emilia-Romagna	17 400 (14 200–21 100)	383·9 (314·5–465·5)	955 000 (751 000–1 420 000)	21·1 (16·6–31·4)	20·8% (16·4–30·6)	48·8% (31·8–59·9)	4·2% (3·7–4·7)	1·9% (1·3–2·5)
		Friuli-Venezia Giulia	4360 (3890–5160)	359·0 (320·6–425·1)	240 000 (182 000–466 000)	19·8 (15·0–38·4)	19·4% (14·8–37·3)	56·5% (27·6–70·8)	4·4% (3·9–4·9)	2·0% (1·0–2·7)
		Lazio	16 100 (13 300–19 300)	281·4 (232·8–338·1)	843 000 (587 000–1 630 000)	14·8 (10·3–28·4)	14·6% (10·2–27·7)	52·6% (25·8–71·5)	4·0% (3·6–4·5)	2·1% (1·0–3·0)
		Liguria	8660 (7190–10 800)	559·3 (463·9–700·3)	287 000 (204 000–428 000)	18·6 (13·2–27·6)	18·3% (13·1–27·0)	43·9% (28·3–59·0)	4·7% (4·1–5·2)	3·2% (1·9–4·6)
		Lombardia	52 200 (41 200–66 400)	522·1 (412·1–664·4)	2 650 000 (2 020 000–3 810 000)	26·5 (20·2–38·1)	26·3% (20·1–37·6)	36·0% (24·3–45·7)	3·9% (3·4–4·4)	2·0% (1·3–2·9)
		Marche	6390 (5000–8000)	409·5 (320·2–512·7)	331 000 (255 000–501 000)	21·2 (16·3–32·1)	21·0% (16·2–31·4)	38·1% (24·5–47·8)	4·2% (3·6–4·8)	2·0% (1·3–2·9)
		Molise	1680 (1360–2110)	552·1 (445·5–694·3)	52 500 (33 100–83 300)	17·2 (10·9–27·4)	16·9% (10·7–27·1)	30·7% (18·1–45·5)	4·1% (3·5–4·6)	3·4% (2·1–5·5)
		Piemonte	24 300 (20 000–29 200)	555·1 (456·4–666·4)	991 000 (677 000–1 720 000)	22·6 (15·4–39·2)	22·4% (15·3–38·6)	42·1% (23·0–58·7)	4·1% (3·6–4·5)	2·6% (1·5–3·6)
		Prov autonoma di Bolzano	1430 (1210–1840)	271·1 (229·9–348·1)	161 000 (123 000–281 000)	30·6 (23·4–53·3)	29·7% (22·9–50·7)	56·3% (30·6–69·8)	3·5% (3·0–3·9)	1·0% (0·5–1·3)
		Prov autonoma di Trento	1600 (1380–1930)	292·4 (252·2–353·4)	113 000 (84 600–182 000)	20·6 (15·4–33·2)	20·4% (15·3–32·7)	47·2% (28·3–60·7)	3·8% (3·4–4·3)	1·5% (0·9–2·1)
		Puglia	15 600 (11 900–19 300)	384·5 (293·7–477·6)	811 000 (560 000–1 260 000)	20·0 (13·8–31·2)	19·9% (13·8–30·6)	36·0% (22·1–49·7)	3·8% (3·2–4·4)	2·0% (1·2–3·0)
		Sardegna	4060 (3110–5190)	249·5 (191·3–319·0)	211 000 (155 000–334 000)	13·0 (9·5–20·5)	12·9% (9·5–20·1)	39·9% (24·2–52·2)	3·9% (3·3–4·5)	2·0% (1·2–2·9)
		Sicilia	16 500 (12 800–20 600)	329·1 (254·1–410·3)	849 000 (589 000–1 320 000)	16·9 (11·7–26·3)	16·6% (11·6–25·6)	39·8% (24·3–54·5)	3·6% (3·0–4·1)	2·1% (1·2–3·0)
		Toscana	11 200 (9330–13 700)	300·2 (249·3–365·6)	656 000 (478 000–1 190 000)	17·5 (12·8–31·8)	17·3% (12·7–30·9)	48·6% (25·2–62·5)	3·9% (3·4–4·3)	1·9% (0·9–2·7)
		Umbria	2860 (2290–3550)	314·8 (251·9–391·0)	142 000 (105 000–232 000)	15·6 (11·6–25·6)	15·5% (11·5–25·1)	49·4% (29·0–63·7)	4·2% (3·6–4·7)	2·1% (1·2–3·0)
		Valle d'Aosta	631 (499–767)	494·4 (391·5–601·6)	33 500 (25 000–53 700)	26·2 (19·6–42·1)	25·7% (19·3–41·5)	40·4% (24·0–51·6)	4·0% (3·5–4·5)	2·0% (1·1–2·8)
		Veneto	14 600 (12 000–17 600)	295·3 (243·2–355·8)	1 010 000 (755 000–1 930 000)	20·4 (15·3–39·1)	20·0% (15·1–37·6)	54·3% (27·0–69·1)	3·9% (3·4–4·3)	1·5% (0·7–2·2)
	Luxembourg		974 (854–1190)	157·5 (138·0–192·9)	153 000 (134 000–185 000)	24·7 (21·7–29·8)	24·1% (21·2–29·1)	57·9% (47·7–65·5)	3·6% (3·0–4·1)	0·7% (0·5–0·8)
	Malta		672 (475–887)	153·1 (108·0–202·0)	68 500 (59 900–81 100)	15·6 (13·6–18·5)	14·9% (13·0–17·5)	57·3% (48·0–65·1)	3·0% (2·7–3·4)	1·1% (0·9–1·4)
	Monaco		47 (37–55)	125·0 (97·9–146·2)	6570 (5810–7330)	17·5 (15·5–19·5)	12·8% (11·2–14·4)	55·9% (50·3–62·5)	4·2% (3·9–4·6)	0·7% (0·6–0·9)
	Netherlands		38 000 (32 400–43 100)	221·6 (188·8–251·0)	4 840 000 (4 120 000–6 600 000)	28·2 (24·0–38·4)	27·1% (23·2–36·9)	57·5% (41·7–66·4)	1·7% (1·2–1·9)	0·9% (0·6–1·1)
	Norway		973 (968–984)	18·2 (18·1–18·4)	614 000 (356 000–1 630 000)	11·5 (6·6–30·5)	11·2% (6·6–28·9)	50·4% (15·8–71·4)	1·2% (0·4–1·7)	0·2% (0·1–0·3)
	Portugal		35 800 (29 600–42 200)	336·5 (278·3–396·6)	2 370 000 (1 890 000–3 080 000)	22·3 (17·8–28·9)	21·9% (17·5–28·2)	48·6% (36·8–59·9)	3·6% (3·1–4·0)	1·6% (1·1–2·2)
	San Marino		98 (91–127)	295·7 (274·8–382·6)	14 300 (11 200–16 800)	43·1 (33·9–50·6)	40·7% (32·2–47·7)	41·8% (35·0–52·5)	3·2% (2·9–3·5)	0·7% (0·6–0·9)
	Spain		145 000 (120 000–174 000)	314·3 (260·6–379·1)	11 800 000 (10 400 000–14 200 000)	25·5 (22·6–30·8)	24·9% (22·0–30·0)	45·3% (37·3–50·9)	4·1% (3·4–4·6)	1·2% (1·0–1·5)
		Andalusia	20 900 (16 900–27 100)	254·2 (204·7–328·8)	2 150 000 (1 550 000–4 270 000)	26·1 (18·9–51·8)	25·6% (18·6–49·1)	41·4% (19·4–53·3)	2·9% (1·4–3·7)	1·1% (0·4–1·5)
		Aragon	5320 (4370–6640)	412·0 (338·6–514·2)	381 000 (305 000–486 000)	29·5 (23·6–37·6)	28·8% (23·3–36·4)	44·9% (34·6–55·0)	4·9% (3·7–6·0)	1·4% (0·9–1·9)
		Asturias	3010 (2390–3670)	300·9 (238·5–367·1)	176 000 (127 000–302 000)	17·6 (12·7–30·2)	17·3% (12·6–29·4)	43·8% (24·6–58·2)	6·9% (3·7–9·0)	1·8% (0·9–2·5)
		Balearic Islands	2530 (1870–3220)	224·7 (166·6–286·0)	217 000 (149 000–388 000)	19·3 (13·3–34·5)	18·8% (13·0–32·6)	51·6% (27·0–70·0)	3·7% (1·9–5·0)	1·3% (0·7–2·0)
		Basque Country	6830 (5700–8310)	316·3 (264·0–384·8)	477 000 (431 000–542 000)	22·1 (19·9–25·1)	21·6% (19·5–24·6)	60·7% (53·2–66·9)	4·8% (4·2–5·2)	1·5% (1·1–1·9)
		Canary Islands	2930 (2220–3880)	138·9 (105·4–184·2)	390 000 (239 000–812 000)	18·5 (11·3–38·5)	18·1% (11·2–36·4)	29·3% (12·7–42·8)	2·5% (1·1–3·7)	0·9% (0·4–1·5)
		Cantabria	1260 (967–1480)	222·4 (170·1–261·1)	104 000 (79 900–155 000)	18·4 (14·1–27·3)	18·1% (13·9–26·8)	48·0% (31·5–61·3)	4·5% (2·9–5·7)	1·3% (0·7–1·7)
		Castile and León	8850 (7140–11 200)	376·8 (304·2–475·2)	644 000 (576 000–801 000)	27·4 (24·5–34·1)	27·0% (24·3–33·6)	49·2% (39·2–54·4)	5·1% (4·1–5·7)	1·4% (1·0–1·8)
		Castilla–La Mancha	8180 (7170–9610)	411·4 (360·4–482·9)	552 000 (463 000–730 000)	27·7 (23·3–36·7)	27·3% (23·0–35·9)	46·4% (34·6–54·4)	3·6% (2·6–5·6)	1·5% (1·1–1·9)
		Catalonia	32 000 (27 100–39 000)	426·0 (360·8–519·7)	2 250 000 (1 770 000–3 080 000)	30·0 (23·6–41·0)	29·0% (22·9–39·1)	47·0% (33·5–58·2)	3·0% (2·2–3·7)	1·5% (1·0–1·9)
		Ceuta	224 (181–271)	269·8 (218·0–326·0)	18 100 (14 500–23 000)	21·8 (17·4–27·8)	18·3% (14·4–24·0)	43·4% (33·4–53·2)	2·7% (2·1–3·3)	1·3% (0·9–1·7)
		Community of Madrid	24 500 (20 700–31 300)	375·1 (318·1–479·2)	2 270 000 (1 910 000–2 750 000)	34·8 (29·2–42·2)	34·0% (28·7–41·4)	41·4% (33·8–48·9)	6·3% (5·1–7·6)	1·1% (0·9–1·4)
		Extremadura	3120 (2490–3680)	298·4 (238·8–352·4)	213 000 (169 000–325 000)	20·4 (16·2–31·1)	20·1% (16·0–30·6)	50·8% (32·1–61·7)	3·4% (2·1–4·1)	1·5% (1·0–2·0)
		Galicia	5120 (4090–6120)	193·8 (154·7–231·7)	334 000 (283 000–408 000)	12·6 (10·7–15·4)	12·5% (10·6–15·2)	56·7% (45·9–66·2)	5·4% (4·3–6·3)	1·6% (1·1–1·9)
		La Rioja	1120 (916–1440)	360·1 (295·6–464·1)	66 200 (58 500–86 400)	21·3 (18·9–27·9)	21·0% (18·6–27·5)	61·9% (47·0–69·4)	6·6% (5·0–7·4)	1·7% (1·3–2·3)
		Melilla	254 (188–334)	300·1 (221·5–394·5)	37 100 (21 700–68 000)	43·9 (25·6–80·3)	39·8% (21·8–72·7)	33·4% (16·5–51·7)	2·7% (1·3–4·1)	0·8% (0·4–1·4)
		Murcia	3190 (2550–3830)	218·4 (174·7–261·9)	275 000 (209 000–490 000)	18·8 (14·3–33·5)	18·6% (14·2–32·8)	55·1% (29·8–69·6)	4·4% (3·8–5·1)	1·2% (0·7–1·6)
		Navarre	2020 (1650–2520)	315·1 (257·3–394·1)	199 000 (179 000–238 000)	31·1 (27·9–37·2)	30·2% (27·3–36·1)	57·8% (48·1–64·1)	2·9% (2·4–3·2)	1·0% (0·7–1·3)
		Valencian Community	13 300 (10 500–16 100)	271·4 (215·4–329·2)	997 000 (829 000–1 220 000)	20·4 (16·9–25·0)	20·0% (16·6–24·5)	54·8% (44·2–65·0)	4·3% (3·5–5·2)	1·4% (1·0–1·8)
	Sweden		16 600 (15 100–19 000)	162·8 (147·6–186·3)	2 320 000 (1 980 000–2 800 000)	22·7 (19·3–27·4)	22·4% (19·2–27·0)	52·2% (42·7–60·6)	2·6% (2·1–3·0)	0·7% (0·6–0·9)
	Switzerland		13 000 (11 000–15 700)	147·7 (125·3–179·1)	1 770 000 (1 490 000–2 200 000)	20·2 (17·0–25·0)	19·7% (16·7–24·4)	56·7% (45·4–66·7)	2·1% (1·6–2·4)	0·8% (0·6–1·0)
	UK		168 000 (167 000–171 000)	250·5 (248·6–254·1)	19 400 000 (18 200 000–20 600 000)	28·8 (27·0–30·7)	27·7% (26·1–29·5)	54·2% (51·0–57·5)	3·1% (2·9–3·3)	0·9% (0·8–0·9)
		England	143 000 (143 000–143 000)	252·1 (252·1–252·1)	16 700 000 (15 500 000–17 900 000)	29·4 (27·4–31·6)	28·3% (26·4–30·4)	53·5% (49·9–57·2)	3·1% (2·9–3·3)	0·9% (0·8–0·9)
		Northern Ireland	4420 (3770–5790)	228·4 (194·8–299·0)	474 000 (421 000–582 000)	24·5 (21·8–30·1)	23·6% (21·0–28·5)	66·8% (54·0–74·2)	3·1% (2·5–3·5)	1·0% (0·7–1·4)
		Scotland	12 500 (11 900–14 300)	227·2 (216·3–258·3)	1 430 000 (1 300 000–1 580 000)	26·0 (23·6–28·7)	25·0% (22·8–27·5)	54·1% (48·8–59·5)	2·9% (2·6–3·1)	0·9% (0·8–1·1)
		Wales	8800 (8800–8800)	276·0 (276·0–276·0)	817 000 (724 000–1 010 000)	25·6 (22·7–31·6)	24·7% (22·0–30·1)	61·4% (49·6–68·8)	4·6% (3·7–5·1)	1·1% (0·9–1·2)
**Latin America and Caribbean**			**2 470 000 (1 870 000–3 370 000)**	**423·2 (320·3–576·3)**	**375 000 000 (334 000 000–417 000 000)**	**64·1 (57·2–71·3)**	**57·4% (51·7–63·1)**	**10·8% (9·7–12·1)**	**0·9% (0·8–1·1)**	**0·7% (0·5–0·9)**
Andean Latin America			530 000 (375 000–755 000)	833·9 (590·3–1186·7)	50 400 000 (36 500 000–60 500 000)	79·3 (57·4–95·1)	69·0% (52·9–83·1)	6·6% (5·4–9·0)	0·9% (0·7–1·3)	1·1% (0·7–1·7)
	Bolivia		135 000 (87 000–205 000)	1125·0 (723·9–1708·8)	12 600 000 (9 320 000–14 500 000)	104·9 (77·6–121·0)	85·8% (69·1–91·0)	4·3% (3·7–5·7)	0·7% (0·5–1·1)	1·1% (0·7–1·8)
	Ecuador		94 200 (66 900–134 000)	535·3 (380·6–764·5)	14 100 000 (10 100 000–17 000 000)	80·2 (57·2–96·7)	70·2% (53·5–83·8)	3·8% (3·1–5·2)	0·8% (0·7–1·2)	0·7% (0·5–1·1)
	Peru		301 000 (217 000–420 000)	885·6 (639·2–1234·9)	23 700 000 (15 900 000–30 900 000)	69·8 (46·8–90·8)	62·3% (44·5–80·9)	9·6% (7·2–14·0)	1·0% (0·9–1·4)	1·3% (0·9–2·0)
Caribbean			87 200 (54 600–147 000)	184·8 (115·7–312·5)	12 100 000 (6 460 000–17 300 000)	25·7 (13·7–36·7)	25·0% (13·5–35·2)	17·4% (11·3–30·2)	1·1% (0·8–1·8)	0·8% (0·5–1·4)
	Antigua and Barbuda		114 (114–114)	128·7 (128·7–128·7)	17 400 (10 100–26 000)	19·7 (11·4–29·4)	16·9% (9·1–26·3)	25·1% (15·7–40·7)	1·1% (0·9–1·5)	0·7% (0·4–1·2)
	The Bahamas		897 (660–1440)	238·0 (175·2–383·2)	116 000 (59 400–178 000)	30·8 (15·8–47·1)	29·5% (15·0–44·8)	21·3% (12·7–38·1)	1·3% (1·0–1·8)	0·9% (0·5–1·6)
	Barbados		394 (211–635)	132·4 (70·9–213·2)	48 400 (35 200–64 200)	16·3 (11·8–21·6)	15·4% (11·1–20·5)	52·5% (38·5–69·6)	1·7% (1·3–2·4)	1·1% (0·7–1·6)
	Belize		805 (535–1360)	196·2 (130·5–331·4)	170 000 (74 200–287 000)	41·5 (18·1–70·0)	38·6% (17·6–62·5)	20·1% (10·5–40·4)	0·8% (0·6–1·2)	0·6% (0·3–1·3)
	Bermuda		140 (106–206)	219·1 (165·8–321·7)	10 000 (8430–13 200)	15·6 (13·2–20·6)	12·1% (10·2–16·3)	58·0% (43·4–67·9)	2·7% (1·9–3·8)	1·4% (1·0–2·1)
	Cuba		25 100 (13 400–52 900)	220·6 (118·0–465·5)	2 080 000 (1 510 000–3 000 000)	18·3 (13·3–26·4)	17·9% (13·2–25·4)	47·9% (32·1–63·5)	2·2% (1·6–2·9)	1·2% (0·6–2·1)
	Dominica		105 (55–178)	152·8 (80·2–258·7)	13 900 (9080–21 100)	20·2 (13·2–30·7)	18·1% (11·6–28·6)	44·4% (27·8–63·9)	1·1% (0·8–1·6)	0·9% (0·5–1·5)
	Dominican Republic		17 900 (8510–31 900)	164·7 (78·2–293·3)	3 680 000 (1 530 000–5 630 000)	33·8 (14·1–51·7)	33·3% (14·0–50·6)	12·4% (7·2–26·5)	0·8% (0·6–1·3)	0·5% (0·3–1·1)
	Grenada		247 (200–412)	239·3 (193·6–399·0)	25 100 (11 600–38 000)	24·3 (11·3–36·8)	23·0% (10·4–35·2)	25·9% (15·5–50·6)	1·3% (1·0–1·8)	1·1% (0·6–2·3)
	Guyana		2200 (968–4290)	285·1 (125·6–556·6)	351 000 (142 000–600 000)	45·6 (18·5–77·9)	42·7% (18·0–69·9)	12·2% (6·3–26·3)	0·9% (0·6–1·4)	0·7% (0·4–1·6)
	Haiti		22 200 (9690–40 600)	178·7 (78·1–327·3)	3 790 000 (1 120 000–6 410 000)	30·5 (9·0–51·7)	29·6% (9·0–49·0)	0·8% (0·4–2·2)	0·6% (0·4–1·2)	0·8% (0·3–2·3)
	Jamaica		5400 (2710–9530)	192·1 (96·4–339·1)	582 000 (296 000–894 000)	20·7 (10·5–31·8)	20·6% (10·5–31·6)	17·1% (10·2–30·7)	1·4% (1·1–1·9)	1·0% (0·5–1·9)
	Puerto Rico		5360 (3890–6480)	152·2 (110·5–184·0)	450 000 (348 000–576 000)	12·8 (9·9–16·4)	12·6% (9·8–16·1)	42·5% (32·5–53·9)	2·2% (1·7–2·8)	1·2% (0·8–1·7)
	Saint Kitts and Nevis		44 (28–68)	73·8 (46·9–113·4)	7120 (4610–11 700)	12·0 (7·8–19·6)	8·8% (4·4–17·7)	41·6% (23·9–60·3)	1·4% (1·0–1·9)	0·7% (0·4–1·2)
	Saint Lucia		417 (266–711)	238·9 (152·3–407·1)	46 100 (24 500–75 200)	26·4 (14·0–43·0)	24·3% (12·9–39·2)	30·0% (17·0–52·1)	1·5% (1·1–2·0)	1·0% (0·6–1·9)
	Saint Vincent and the Grenadines		195 (95–344)	172·0 (83·9–303·8)	20 400 (11 400–31 900)	18·1 (10·1–28·2)	16·5% (9·0–26·6)	34·2% (20·5–57·0)	1·3% (1·0–1·9)	1·0% (0·5–2·2)
	Suriname		2230 (1190–4700)	387·4 (206·4–815·3)	321 000 (159 000–491 000)	55·8 (27·6–85·2)	52·1% (26·5–78·4)	17·4% (10·3–31·8)	1·1% (0·9–1·8)	0·8% (0·4–1·8)
	Trinidad and Tobago		3030 (1850–5440)	218·6 (133·6–392·4)	362 000 (167 000–595 000)	26·1 (12·1–42·9)	25·1% (11·9–40·0)	20·9% (11·6–40·5)	1·5% (1·2–2·1)	1·2% (0·7–2·2)
	Virgin Islands		459 (248–861)	441·6 (238·2–827·9)	26 800 (14 200–44 800)	25·7 (13·7–43·1)	23·4% (12·0–39·1)	30·0% (16·7–52·4)	0·8% (0·5–1·4)	1·9% (1·0–3·6)
Central Latin America			1 120 000 (794 000–1 560 000)	446·5 (317·5–625·5)	164 000 000 (143 000 000–185 000 000)	65·6 (57·2–74·0)	59·1% (52·2–65·9)	7·7% (6·7–8·7)	0·7% (0·6–0·8)	0·7% (0·5–1·0)
	Colombia		156 000 (128 000–209 000)	327·3 (267·4–438·0)	23 200 000 (17 900 000–28 600 000)	48·5 (37·4–59·8)	45·2% (35·3–54·1)	22·2% (17·7–28·3)	0·9% (0·7–1·0)	0·7% (0·5–1·0)
	Costa Rica		7210 (7210–7210)	153·0 (152·8–152·8)	2 260 000 (1 430 000–3 130 000)	48·0 (30·4–66·4)	44·6% (28·9–60·9)	26·2% (18·1–39·6)	1·4% (1·0–2·2)	0·3% (0·2–0·5)
	El Salvador		22 600 (15 500–32 100)	361·7 (248·3–512·7)	2 340 000 (1 510 000–3 270 000)	37·4 (24·1–52·3)	36·0% (23·6–49·4)	5·4% (3·7–7·9)	0·7% (0·6–0·9)	1·0% (0·6–1·8)
	Guatemala		43 900 (27 400–64 600)	246·9 (154·3–363·6)	12 500 000 (8 460 000–17 100 000)	70·4 (47·6–95·9)	62·8% (44·8–81·9)	5·1% (3·6–7·3)	0·4% (0·3–0·6)	0·4% (0·2–0·6)
	Honduras		47 500 (30 900–76 500)	483·8 (314·5–779·1)	9 360 000 (7 040 000–11 500 000)	95·3 (71·7–117·4)	81·4% (66·4–91·7)	4·1% (3·3–5·4)	0·5% (0·4–0·7)	0·5% (0·3–0·8)
	Mexico		678 000 (480 000–951 000)	542·6 (383·9–761·5)	92 200 000 (84 200 000–101 000 000)	73·8 (67·4–80·9)	65·7% (61·1–70·8)	4·2% (3·9–4·6)	0·7% (0·6–0·7)	0·7% (0·5–1·1)
		Aguascalientes	5880 (4280–8080)	424·5 (309·1–583·3)	760 000 (380 000–1 050 000)	54·9 (27·4–76·0)	51·2% (26·7–70·0)	5·4% (3·5–9·8)	1·0% (0·6–1·8)	0·9% (0·5–1·7)
		Baja California	19 200 (13 100–26 900)	496·7 (338·4–696·6)	3 540 000 (2 530 000–4 240 000)	91·6 (65·5–110·0)	79·2% (59·4–89·4)	2·4% (1·9–3·2)	0·6% (0·5–0·8)	0·6% (0·4–1·0)
		Baja California Sur	3340 (3000–4560)	406·3 (364·9–554·2)	670 000 (430 000–931 000)	81·4 (52·3–113·1)	70·5% (48·6–88·6)	8·9% (6·1–13·3)	0·7% (0·5–1·1)	0·5% (0·3–0·8)
		Campeche	4240 (3210–6010)	456·1 (345·7–646·6)	578 000 (398 000–848 000)	62·2 (42·8–91·2)	56·2% (40·1–78·6)	4·3% (2·9–6·1)	0·7% (0·5–1·0)	0·8% (0·5–1·3)
		Chiapas	19 600 (13 300–27 500)	334·0 (225·7–468·1)	2 300 000 (1 320 000–3 550 000)	39·0 (22·4–60·3)	37·1% (21·9–55·6)	1·1% (0·7–1·8)	0·2% (0·1–0·4)	0·9% (0·5–1·5)
		Chihuahua	21 200 (14 600–29 000)	601·2 (413·9–820·8)	1 530 000 (560 000–2 860 000)	43·4 (15·9–81·1)	41·5% (15·7–76·0)	6·2% (2·6–13·4)	1·3% (0·6–2·9)	1·9% (0·7–4·0)
		Coahuila	17 700 (12 300–24 800)	565·5 (392·1–792·6)	2 880 000 (2 040 000–3 470 000)	91·9 (65·3–110·8)	80·4% (60·4–92·1)	3·5% (2·8–4·8)	0·5% (0·4–0·7)	0·7% (0·4–1·0)
		Colima	2500 (2460–2800)	333·1 (328·5–374·2)	390 000 (224 000–590 000)	52·0 (29·9–78·7)	47·8% (28·8–69·7)	9·2% (5·6–14·8)	1·2% (0·7–1·9)	0·7% (0·4–1·1)
		Durango	7120 (4770–9750)	396·9 (265·9–544·0)	1 510 000 (889 000–2 010 000)	84·1 (49·6–112·3)	73·2% (46·6–90·6)	3·6% (2·5–5·6)	0·4% (0·3–0·7)	0·5% (0·3–1·0)
		Guanajuato	27 300 (19 300–38 600)	442·9 (312·8–627·0)	5 510 000 (3 820 000–6 920 000)	89·3 (62·0–112·3)	78·2% (57·9–92·1)	3·7% (2·8–5·2)	0·5% (0·4–0·7)	0·5% (0·3–0·9)
		Guerrero	13 600 (9510–18 900)	366·2 (256·6–510·9)	2 000 000 (1 600 000–2 340 000)	54·0 (43·3–63·1)	50·6% (41·9–59·0)	3·9% (3·3–4·8)	0·6% (0·5–0·8)	0·7% (0·5–1·0)
		Hidalgo	13 800 (9910–19 500)	456·7 (327·6–643·2)	2 630 000 (1 840 000–3 340 000)	87·0 (60·8–110·6)	75·4% (55·7–89·4)	2·5% (1·9–3·4)	0·7% (0·5–0·9)	0·5% (0·3–0·9)
		Jalisco	29 400 (22 900–41 300)	359·5 (280·3–505·2)	4 980 000 (3 670 000–6 590 000)	61·0 (44·9–80·7)	56·0% (42·5–71·6)	3·4% (2·5–4·5)	0·7% (0·5–1·0)	0·6% (0·4–0·9)
		México	124 000 (87 400–176 000)	717·5 (504·4–1017·3)	13 300 000 (10 300 000–16 500 000)	76·9 (59·2–95·3)	69·9% (55·0–82·7)	3·0% (2·3–3·8)	0·7% (0·6–0·9)	1·0% (0·6–1·4)
		Mexico City	90 700 (64 900–127 000)	1029·0 (735·8–1436·0)	8 450 000 (7 030 000–9 510 000)	95·9 (79·7–107·9)	83·5% (71·3–89·1)	11·8% (10·4–14·1)	1·0% (0·9–1·2)	1·1% (0·7–1·6)
		Michoacán de Ocampo	17 200 (11 800–24 600)	368·5 (253·4–526·1)	2 300 000 (1 740 000–2 970 000)	49·3 (37·3–63·7)	46·5% (36·0–59·4)	3·3% (2·5–4·2)	0·7% (0·5–0·9)	0·8% (0·5–1·2)
		Morelos	9600 (7010–13 700)	487·3 (356·0–698·2)	1 500 000 (1 010 000–2 170 000)	76·4 (51·4–110·2)	69·0% (48·9–90·8)	3·5% (2·3–4·9)	0·7% (0·5–1·0)	0·7% (0·4–1·1)
		Nayarit	3640 (3120–4950)	296·5 (253·7–403·0)	726 000 (496 000–1 040 000)	59·1 (40·4–84·3)	54·0% (38·1–75·3)	4·9% (3·3–6·9)	0·9% (0·6–1·3)	0·5% (0·3–0·8)
		Nuevo León	27 900 (19 700–39 500)	530·1 (373·7–748·9)	4 660 000 (3 620 000–5 310 000)	88·3 (68·7–100·7)	76·0% (62·3–84·9)	4·5% (3·9–5·7)	0·7% (0·6–0·9)	0·6% (0·4–0·9)
		Oaxaca	15 000 (10 100–21 200)	361·8 (244·9–510·4)	2 090 000 (1 490 000–2 700 000)	50·5 (35·9–65·2)	47·2% (34·2–58·7)	4·1% (3·1–5·6)	0·6% (0·4–0·8)	0·7% (0·5–1·3)
		Puebla	43 600 (30 600–63 100)	672·6 (471·4–973·8)	5 820 000 (4 420 000–6 880 000)	89·7 (68·2–106·1)	78·0% (62·4–88·2)	2·2% (1·8–2·8)	0·5% (0·5–0·7)	0·8% (0·5–1·4)
		Querétaro	8970 (6900–12 300)	410·4 (315·9–564·0)	1 130 000 (394 000–2 040 000)	51·9 (18·0–93·2)	48·0% (17·8–80·8)	11·2% (4·9–25·2)	1·3% (0·6–3·0)	1·0% (0·4–2·3)
		Quintana Roo	7570 (5200–10 700)	444·0 (304·6–625·0)	1 710 000 (1 300 000–2 050 000)	100·0 (76·2–119·9)	82·6% (66·5–91·0)	3·6% (2·9–4·6)	0·5% (0·4–0·7)	0·5% (0·3–0·7)
		San Luis Potosí	14 200 (10 000–20 000)	505·6 (355·1–709·6)	1 690 000 (1 220 000–2 160 000)	60·0 (43·4–76·8)	55·6% (41·8–69·4)	6·4% (4·9–8·6)	0·7% (0·5–0·9)	0·9% (0·6–1·4)
		Sinaloa	15 200 (10 300–21 700)	516·3 (348·2–736·2)	2 470 000 (1 740 000–3 190 000)	83·7 (58·9–108·1)	72·8% (54·5–86·4)	3·1% (2·3–4·3)	0·8% (0·6–1·0)	0·6% (0·4–1·0)
		Sonora	15 700 (10 700–21 900)	526·9 (358·6–736·7)	2 530 000 (1 990 000–3 110 000)	84·9 (66·9–104·6)	74·4% (60·8–85·3)	4·7% (3·8–6·0)	0·7% (0·6–0·9)	0·6% (0·4–1·0)
		Tabasco	13 700 (9290–19 400)	551·5 (373·8–780·4)	2 330 000 (1 710 000–2 820 000)	93·9 (68·9–113·7)	79·3% (61·5–88·9)	6·3% (5·1–8·4)	0·5% (0·4–0·7)	0·6% (0·4–1·0)
		Tamaulipas	18 500 (12 600–26 500)	527·8 (359·0–757·7)	2 500 000 (1 730 000–3 310 000)	71·4 (49·3–94·4)	63·6% (46·6–80·7)	4·3% (3·1–6·0)	0·5% (0·4–0·7)	0·8% (0·4–1·2)
		Tlaxcala	11 500 (7940–16 400)	860·8 (594·6–1230·1)	1 100 000 (810 000–1 420 000)	82·4 (60·6–106·3)	73·8% (57·7–89·9)	2·7% (2·1–3·6)	0·6% (0·4–0·8)	1·1% (0·7–1·7)
		Veracruz de Ignacio de la Llave	37 900 (26 300–54 200)	464·7 (323·1–665·0)	5 540 000 (3 820 000–7 410 000)	67·9 (46·8–90·9)	60·8% (43·8–76·6)	2·3% (1·7–3·3)	0·6% (0·4–0·8)	0·7% (0·4–1·1)
		Yucatán	9640 (6480–14 000)	441·3 (296·6–639·3)	2 190 000 (1 390 000–2 630 000)	100·2 (63·4–120·2)	82·2% (57·4–91·7)	3·6% (2·8–5·4)	0·6% (0·4–0·8)	0·5% (0·3–0·8)
		Zacatecas	8240 (5780–11 500)	514·1 (360·6–714·4)	915 000 (574 000–1 300 000)	57·1 (35·8–80·8)	53·6% (34·7–73·0)	4·8% (3·2–7·3)	0·8% (0·5–1·1)	0·9% (0·6–1·5)
	Nicaragua		17 600 (11 400–25 400)	270·2 (175·4–390·1)	3 860 000 (3 120 000–4 500 000)	59·3 (48·0–69·1)	55·4% (46·1–63·5)	3·9% (2·7–4·9)	0·6% (0·5–0·8)	0·5% (0·3–0·7)
	Panama		8940 (7330–12 900)	214·9 (176·2–309·6)	1 740 000 (1 330 000–2 230 000)	41·8 (32·0–53·5)	40·5% (31·4–51·1)	28·0% (21·4–35·8)	0·7% (0·6–0·9)	0·5% (0·3–0·8)
	Venezuela		134 000 (86 800–193 000)	478·1 (309·1–687·4)	16 600 000 (13 500 000–19 400 000)	59·3 (48·0–69·1)	54·3% (45·1–62·7)	7·6% (6·2–9·8)	0·9% (0·7–1·1)	0·8% (0·5–1·2)
Tropical Latin America			739 000 (651 000–933 000)	330·5 (290·9–417·2)	148 000 000 (122 000 000–174 000 000)	66·1 (54·6–78·0)	59·2% (50·1–68·1)	15·4% (12·9–18·4)	1·2% (1·0–1·5)	0·5% (0·4–0·6)
	Brazil		720 000 (634 000–907 000)	332·1 (292·7–418·5)	143 000 000 (118 000 000–169 000 000)	66·0 (54·4–78·0)	59·0% (50·0–68·1)	15·5% (13·0–18·7)	1·2% (1·0–1·5)	0·5% (0·4–0·6)
		Acre	2200 (1840–2750)	237·4 (198·2–297·0)	501 000 (307 000–735 000)	54·1 (33·1–79·4)	49·8% (31·9–69·2)	18·4% (12·0–28·7)	0·9% (0·6–1·4)	0·5% (0·3–0·7)
		Alagoas	6660 (6320–8280)	181·9 (172·6–226·3)	2 130 000 (1 080 000–3 320 000)	58·1 (29·4–90·8)	52·8% (28·1–77·5)	12·3% (7·3–22·4)	1·0% (0·6–1·9)	0·3% (0·2–0·6)
		Amapá	2890 (1990–3820)	342·5 (235·2–452·4)	653 000 (470 000–821 000)	77·3 (55·6–97·2)	68·1% (51·3–82·2)	19·4% (15·1–26·3)	0·9% (0·7–1·2)	0·5% (0·3–0·6)
		Amazonas	17 900 (13 800–24 000)	424·0 (325·9–568·4)	3 260 000 (2 180 000–4 370 000)	77·3 (51·8–103·4)	67·7% (48·7–86·2)	13·6% (9·8–19·7)	1·1% (0·8–1·6)	0·6% (0·4–0·8)
		Bahia	39 800 (29 800–52 600)	249·5 (186·8–330·0)	10 600 000 (6 230 000–14 400 000)	66·5 (39·0–90·1)	59·7% (37·4–77·1)	12·4% (8·7–20·2)	0·7% (0·5–1·1)	0·4% (0·3–0·7)
		Ceará	45 400 (34 800–59 100)	452·2 (346·6–588·6)	7 860 000 (5 700 000–10 300 000)	78·4 (56·8–102·7)	68·5% (52·4–85·7)	12·4% (9·2–16·6)	0·8% (0·6–1·1)	0·6% (0·4–0·8)
		Distrito Federal	11 000 (10 900–11 500)	362·7 (361·5–381·3)	1 750 000 (805 000–3 600 000)	57·9 (26·6–118·7)	51·5% (25·8–91·3)	35·5% (14·4–64·2)	3·0% (1·2–5·4)	0·8% (0·3–1·4)
		Espírito Santo	15 200 (13 000–19 800)	382·7 (327·9–497·3)	2 890 000 (1 980 000–4 050 000)	72·7 (49·8–101·9)	64·2% (45·8–81·8)	22·2% (15·2–31·2)	0·5% (0·3–0·7)	0·6% (0·4–0·9)
		Goiás	26 900 (24 300–33 800)	391·4 (353·5–491·3)	6 190 000 (4 990 000–7 130 000)	90·1 (72·5–103·7)	78·0% (63·4–86·1)	15·2% (13·0–18·6)	1·1% (1·0–1·4)	0·4% (0·4–0·6)
		Maranhão	28 400 (19 600–44 100)	339·2 (234·3–527·7)	6 460 000 (2 250 000–9 300 000)	77·3 (26·9–111·2)	67·1% (26·1–90·6)	6·5% (3·9–16·2)	0·3% (0·2–0·8)	0·5% (0·3–1·2)
		Mato Grosso	14 700 (13 700–18 800)	408·7 (380·8–523·0)	2 900 000 (2 190 000–3 590 000)	80·4 (61·0–99·7)	70·2% (55·5–84·7)	19·3% (15·3–25·0)	1·0% (0·8–1·3)	0·5% (0·4–0·7)
		Mato Grosso do Sul	9690 (9650–10 400)	341·1 (339·5–367·1)	1 920 000 (1 110 000–2 840 000)	67·7 (38·9–100·1)	60·5% (37·0–80·8)	20·7% (13·3–34·2)	1·7% (1·1–2·8)	0·5% (0·3–0·9)
		Minas Gerais	65 600 (55 800–85 800)	302·6 (257·4–395·5)	18 900 000 (12 600 000–24 800 000)	87·1 (58·3–114·5)	75·4% (53·8–93·2)	12·1% (8·9–17·4)	1·0% (0·7–1·4)	0·4% (0·2–0·6)
		Pará	21 700 (16 700–29 600)	234·7 (181·1–320·3)	5 950 000 (3 370 000–8 410 000)	64·4 (36·5–90·9)	57·9% (34·7–76·4)	10·8% (7·2–18·0)	0·8% (0·5–1·3)	0·4% (0·3–0·6)
		Paraíba	9470 (9460–9690)	216·2 (215·8–221·1)	3 220 000 (1 980 000–4 460 000)	73·5 (45·1–101·8)	65·0% (42·2–85·7)	14·8% (10·3–23·3)	0·9% (0·6–1·4)	0·3% (0·2–0·5)
		Paraná	41 600 (40 500–49 000)	365·4 (355·7–430·5)	7 290 000 (5 560 000–9 120 000)	64·0 (48·8–80·0)	57·9% (45·5–70·2)	22·0% (17·3–28·3)	1·7% (1·4–2·2)	0·6% (0·5–0·8)
		Pernambuco	28 100 (21 100–36 400)	277·1 (208·8–359·2)	4 960 000 (3 240 000–6 380 000)	48·9 (32·0–63·0)	45·6% (30·6–57·8)	13·4% (10·0–19·7)	0·8% (0·6–1·2)	0·6% (0·4–0·9)
		Piauí	8940 (7130–11 000)	242·1 (192·9–297·6)	2 610 000 (1 490 000–3 580 000)	70·6 (40·3–97·0)	62·6% (38·1–80·4)	13·0% (9·0–21·6)	0·9% (0·6–1·5)	0·4% (0·2–0·6)
		Rio de Janeiro	73 400 (68 600–92 400)	415·5 (388·0–522·6)	8 760 000 (6 300 000–11 800 000)	49·6 (35·6–66·8)	46·0% (34·3–58·8)	15·7% (11·3–21·2)	2·0% (1·4–2·7)	0·9% (0·6–1·2)
		Rio Grande do Norte	8080 (7400–9770)	216·0 (198·0–261·3)	1 930 000 (1 450 000–2 490 000)	51·6 (38·8–66·6)	47·8% (37·1–59·1)	20·0% (15·2–26·1)	1·0% (0·7–1·3)	0·4% (0·3–0·6)
		Rio Grande do Sul	35 900 (35 800–37 700)	317·8 (316·5–333·8)	4 010 000 (2 930 000–7 750 000)	35·5 (25·9–68·6)	33·6% (25·1–62·1)	39·5% (19·2–50·8)	3·0% (1·4–3·8)	1·0% (0·5–1·2)
		Rondônia	8380 (6600–10 200)	471·6 (371·5–572·4)	1 320 000 (1 020 000–1 690 000)	74·6 (57·7–95·1)	66·0% (52·1–80·2)	21·2% (16·3–26·9)	1·1% (0·8–1·4)	0·6% (0·5–0·9)
		Roraima	2040 (2020–2210)	340·8 (337·4–369·5)	489 000 (373 000–635 000)	81·7 (62·3–106·0)	71·1% (55·3–89·8)	26·8% (20·1–34·3)	0·9% (0·7–1·2)	0·4% (0·3–0·6)
		Santa Catarina	19 900 (19 800–21 000)	277·8 (276·8–293·2)	5 470 000 (4 180 000–7 460 000)	76·4 (58·4–104·3)	67·5% (52·8–88·1)	23·0% (16·5–29·4)	1·4% (1·0–1·7)	0·4% (0·3–0·5)
		São Paulo	165 000 (153 000–212 000)	361·9 (335·7–465·0)	28 800 000 (22 200 000–35 600 000)	63·2 (48·9–78·1)	57·4% (45·2–70·2)	15·6% (12·4–19·9)	1·8% (1·5–2·3)	0·6% (0·5–0·9)
		Sergipe	6050 (6030–6360)	251·2 (250·4–264·2)	852 000 (666 000–1 690 000)	35·4 (27·7–70·0)	33·5% (26·9–63·3)	34·1% (16·5–41·7)	1·9% (0·9–2·4)	0·7% (0·4–0·9)
		Tocantins	4900 (3890–6010)	298·5 (237·0–365·9)	1 400 000 (896 000–1 810 000)	85·2 (54·6–110·3)	73·5% (50·0–90·0)	17·1% (12·8–25·9)	0·7% (0·5–1·0)	0·4% (0·3–0·5)
	Paraguay		19 600 (16 400–26 700)	282·3 (236·9–384·6)	4 830 000 (3 370 000–6 210 000)	69·7 (48·6–89·7)	62·8% (45·5–76·7)	9·8% (7·4–13·7)	1·1% (0·9–1·4)	0·4% (0·3–0·6)
**North Africa and Middle East**			**1 430 000 (986 000–2 160 000)**	**235·6 (162·0–354·6)**	**382 000 000 (281 000 000–481 000 000)**	**62·7 (46·2–79·0)**	**55·3% (40·8–67·1)**	**6·8% (5·2–8·9)**	**1·0% (0·8–1·5)**	**0·4% (0·2–0·7)**
	Afghanistan		164 000 (92 400–274 000)	429·1 (241·3–715·9)	39 600 000 (19 600 000–47 100 000)	103·4 (51·2–123·1)	84·8% (47·4–93·4)	0·4% (0·3–0·8)	0·5% (0·3–0·9)	0·5% (0·2–1·1)
	Algeria		44 300 (31 900–62 000)	105·7 (76·2–148·1)	7 800 000 (4 760 000–13 100 000)	18·6 (11·4–31·3)	18·4% (11·3–30·5)	2·9% (1·6–4·4)	1·2% (0·9–1·7)	0·6% (0·3–1·1)
	Bahrain		3570 (2510–5160)	247·4 (174·1–357·7)	875 000 (552 000–1 180 000)	60·6 (38·3–82·1)	55·2% (36·0–71·7)	32·9% (23·4–50·2)	1·3% (1·0–1·6)	0·4% (0·2–0·7)
	Egypt		204 000 (143 000–292 000)	206·2 (144·7–294·9)	75 300 000 (15 200 000–114 000 000)	76·0 (15·3–114·9)	65·4% (15·1–89·4)	0·7% (0·3–2·3)	1·0% (0·8–1·4)	0·4% (0·2–1·8)
	Iran		234 000 (154 000–389 000)	277·5 (183·1–461·9)	56 900 000 (40 500 000–86 000 000)	67·5 (48·0–102·0)	60·5% (45·1–85·3)	11·1% (7·1–15·1)	1·3% (1·0–1·8)	0·4% (0·2–0·8)
	Iraq		156 000 (97 500–265 000)	370·8 (231·5–628·3)	41 900 000 (26 200 000–50 200 000)	99·4 (62·1–119·2)	82·4% (57·1–91·7)	5·1% (4·1–8·0)	0·9% (0·7–1·3)	0·4% (0·2–0·8)
	Jordan		21 100 (13 200–34 300)	181·3 (113·5–295·0)	8 330 000 (5 440 000–10 300 000)	71·6 (46·8–88·1)	64·9% (44·9–77·4)	11·5% (9·1–17·1)	0·8% (0·6–1·3)	0·3% (0·2–0·5)
	Kuwait		3140 (2460–4460)	70·8 (55·5–100·8)	1 970 000 (1 110 000–3 320 000)	44·4 (25·1–75·1)	41·9% (24·6–66·3)	23·0% (12·4–37·2)	1·2% (1·0–1·5)	0·2% (0·1–0·3)
	Lebanon		30 300 (20 800–50 400)	585·2 (401·6–974·0)	3 750 000 (2 430 000–4 880 000)	72·5 (46·9–94·3)	67·3% (45·6–83·7)	18·3% (13·6–27·4)	1·8% (1·4–2·2)	0·9% (0·5–1·5)
	Libya		29 600 (18 800–47 400)	439·8 (279·7–703·3)	6 880 000 (4 660 000–8 110 000)	102·1 (69·1–120·5)	83·8% (63·8–91·7)	5·5% (4·6–8·0)	1·3% (1·0–1·8)	0·5% (0·3–0·8)
	Morocco		136 000 (86 600–233 000)	377·6 (240·7–649·2)	27 800 000 (13 600 000–39 100 000)	77·3 (37·8–108·9)	67·2% (36·0–86·3)	3·7% (2·4–7·0)	0·9% (0·7–1·4)	0·5% (0·3–1·1)
	Oman		10 800 (7990–14 200)	234·9 (174·2–310·2)	2 060 000 (1 340 000–2 980 000)	44·9 (29·3–64·9)	42·0% (28·1–61·0)	15·4% (10·3–22·7)	1·1% (0·7–1·6)	0·5% (0·3–0·9)
	Palestine		10 600 (7010–16 000)	214·8 (141·5–322·4)	4 730 000 (3 000 000–5 830 000)	95·4 (60·6–117·7)	79·9% (56·3–90·5)	9·4% (7·3–14·3)	0·8% (0·6–1·2)	0·2% (0·1–0·4)
	Qatar		1340 (898–1890)	46·9 (31·4–66·1)	1 950 000 (1 230 000–2 380 000)	68·0 (43·0–83·2)	61·0% (41·4–72·8)	12·9% (10·2–19·7)	1·8% (1·4–2·8)	0·1% (0·0–0·1)
	Saudi Arabia		27 900 (19 600–38 200)	78·1 (55·0–106·9)	8 150 000 (4 870 000–13 900 000)	22·8 (13·6–38·9)	22·4% (13·6–37·3)	7·2% (4·0–11·3)	1·1% (0·8–1·4)	0·4% (0·2–0·6)
	Sudan		66 200 (42 900–101 000)	162·2 (105·1–248·1)	21 100 000 (7 720 000–34 900 000)	51·7 (18·9–85·6)	49·7% (18·8–77·6)	0·2% (0·1–0·5)	0·7% (0·5–1·0)	0·4% (0·2–1·0)
	Syria		21 200 (14 000–30 300)	146·4 (96·6–209·4)	3 010 000 (1 350 000–5 160 000)	20·7 (9·3–35·6)	20·2% (9·2–33·9)	1·8% (0·9–3·5)	1·3% (1·0–1·8)	0·8% (0·4–1·5)
	Tunisia		62 400 (43 900–102 000)	539·5 (379·2–880·4)	10 600 000 (6 280 000–13 600 000)	91·6 (54·3–117·8)	77·9% (51·1–91·2)	7·0% (5·3–11·5)	1·8% (1·3–2·3)	0·6% (0·4–1·2)
	Turkey		145 000 (104 000–217 000)	178·2 (127·7–267·3)	44 100 000 (26 200 000–70 200 000)	54·2 (32·2–86·3)	49·6% (31·1–76·6)	23·0% (13·6–36·4)	1·4% (1·1–1·8)	0·4% (0·2–0·7)
	United Arab Emirates		8600 (5540–12 800)	93·0 (59·9–138·0)	2 460 000 (1 350 000–3 690 000)	26·6 (14·6–40·0)	26·0% (14·5–38·4)	32·4% (20·1–54·9)	1·3% (1·0–1·8)	0·4% (0·2–0·7)
	Yemen		53 900 (28 800–91 300)	171·2 (91·5–289·8)	12 500 000 (4 210 000–26 200 000)	39·6 (13·4–83·2)	37·5% (13·1–71·1)	0·1% (0·0–0·2)	0·6% (0·4–1·0)	0·6% (0·2–1·6)
**South Asia**			**4 500 000 (3 190 000–6 340 000)**	**249·1 (176·8–351·0)**	**1 340 000 000 (1 200 000 000–1 490 000 000)**	**74·3 (66·5–82·6)**	**65·8% (59·5–71·5)**	**2·8% (2·5–3·2)**	**1·2% (0·9–1·8)**	**0·3% (0·2–0·5)**
	Bangladesh		353 000 (236 000–508 000)	221·7 (148·4–319·2)	137 000 000 (98 900 000–185 000 000)	86·0 (62·1–116·0)	74·3% (57·2–92·4)	1·2% (0·9–1·6)	1·3% (1·0–2·0)	0·3% (0·2–0·4)
	Bhutan		108 (55–158)	14·3 (7·3–20·9)	20 200 (14 200–27 500)	2·7 (1·9–3·6)	2·2% (1·3–3·3)	13·4% (9·5–18·4)	1·1% (0·8–1·5)	0·5% (0·3–0·8)
	India		3 480 000 (2 520 000–4 910 000)	250·1 (181·6–353·4)	1 000 000 000 (881 000 000–1 120 000 000)	72·1 (63·4–80·3)	64·3% (57·7–70·4)	3·4% (3·1–3·9)	1·2% (0·9–1·9)	0·3% (0·3–0·5)
		Andhra Pradesh	166 000 (119 000–252 000)	307·2 (219·0–465·6)	43 600 000 (28 400 000–61 000 000)	80·5 (52·4–112·5)	70·2% (48·4–93·4)	5·0% (3·4–7·3)	1·5% (1·1–2·1)	0·4% (0·2–0·8)
		Arunachal Pradesh	1520 (767–2310)	88·5 (44·6–133·9)	675 000 (434 000–1 230 000)	39·2 (25·2–71·4)	37·1% (24·3–64·9)	8·8% (4·6–12·7)	1·0% (0·7–1·5)	0·2% (0·1–0·4)
		Assam	101 000 (66 000–145 000)	279·7 (183·1–403·5)	28 700 000 (18 400 000–39 700 000)	79·6 (50·9–110·1)	71·7% (47·2–95·8)	2·3% (1·6–3·4)	1·1% (0·8–1·8)	0·4% (0·2–0·7)
		Bihar	276 000 (175 000–396 000)	226·3 (143·1–324·1)	94 900 000 (39 700 000–117 000 000)	77·7 (32·5–95·8)	69·1% (31·2–83·3)	0·8% (0·6–1·8)	0·9% (0·7–1·5)	0·3% (0·2–0·7)
		Chhattisgarh	130 000 (88 700–188 000)	409·0 (279·8–593·9)	23 100 000 (17 300 000–34 000 000)	73·0 (54·5–107·1)	65·3% (50·5–89·4)	4·5% (3·0–5·8)	1·1% (0·8–1·8)	0·6% (0·3–1·0)
		Delhi	53 000 (33 500–81 300)	272·4 (172·0–417·9)	19 400 000 (13 700 000–23 200 000)	99·9 (70·6–119·3)	82·7% (63·5–91·7)	7·6% (6·2–10·5)	1·4% (1·1–1·9)	0·3% (0·2–0·4)
		Goa	3670 (3490–5210)	239·7 (228·4–341·0)	917 000 (614 000–1 300 000)	60·0 (40·2–85·3)	55·1% (38·4–74·1)	20·2% (13·7–29·1)	1·9% (1·6–2·5)	0·4% (0·3–0·6)
		Gujarat	111 000 (78 500–147 000)	160·4 (113·3–212·0)	53 600 000 (29 000 000–74 000 000)	77·4 (41·8–106·9)	67·9% (39·4–85·6)	1·6% (1·1–2·9)	1·2% (0·9–1·8)	0·2% (0·1–0·4)
		Haryana	113 000 (78 900–164 000)	389·4 (270·8–562·3)	21 900 000 (15 500 000–28 800 000)	75·3 (53·1–98·7)	67·1% (48·2–83·2)	3·6% (2·7–5·0)	1·3% (1·0–2·0)	0·5% (0·3–0·9)
		Himachal Pradesh	28 400 (17 900–41 600)	373·1 (235·4–545·9)	4 030 000 (2 950 000–5 740 000)	52·9 (38·7–75·5)	49·1% (37·4–66·3)	5·8% (4·0–7·7)	1·5% (1·2–2·2)	0·7% (0·4–1·2)
		Jammu and Kashmir	35 100 (23 100–49 100)	250·7 (165·1–350·4)	7 230 000 (3 960 000–9 500 000)	51·6 (28·2–67·8)	47·8% (27·5–62·3)	5·1% (3·8–9·0)	1·2% (0·9–1·7)	0·5% (0·3–0·8)
		Jharkhand	70 500 (48 500–93 800)	186·1 (127·9–247·6)	24 500 000 (8 010 000–40 700 000)	64·6 (21·2–107·4)	58·3% (20·5–89·9)	1·7% (0·9–4·4)	1·1% (0·8–1·8)	0·3% (0·1–0·9)
		Karnataka	244 000 (173 000–363 000)	358·3 (254·1–533·6)	47 200 000 (26 400 000–60 100 000)	69·4 (38·8–88·4)	62·3% (37·2–76·7)	6·5% (5·0–11·3)	1·3% (1·0–2·0)	0·5% (0·3–0·9)
		Kerala	96 300 (68 000–141 000)	275·5 (194·5–402·6)	16 100 000 (9 300 000–31 600 000)	46·0 (26·6–90·3)	43·9% (26·0–85·2)	35·4% (16·2–55·1)	2·0% (1·6–2·5)	0·7% (0·3–1·1)
		Madhya Pradesh	191 000 (136 000–262 000)	215·1 (153·1–295·8)	61 600 000 (27 800 000–85 800 000)	69·4 (31·3–96·7)	62·1% (30·2–80·8)	1·4% (0·9–2·9)	1·1% (0·8–1·7)	0·3% (0·2–0·7)
		Maharashtra	530 000 (372 000–775 000)	425·0 (298·2–621·4)	73 200 000 (52 700 000–116 000 000)	58·7 (42·3–92·8)	53·6% (39·8–78·3)	9·4% (5·7–12·6)	1·4% (1·1–2·0)	0·8% (0·3–1·3)
		Manipur	14 900 (9280–22 700)	424·9 (264·4–647·7)	2 950 000 (1 860 000–3 660 000)	84·1 (52·9–104·4)	76·0% (49·7–90·4)	4·4% (3·4–6·7)	1·3% (0·9–1·9)	0·5% (0·3–0·9)
		Meghalaya	7530 (4600–10 800)	220·5 (134·7–314·9)	2 440 000 (1 650 000–3 370 000)	71·5 (48·4–98·7)	66·4% (45·6–90·6)	3·6% (2·5–5·1)	1·0% (0·7–1·6)	0·3% (0·2–0·5)
		Mizoram	2170 (1340–3280)	170·4 (105·2–257·2)	810 000 (628 000–906 000)	63·5 (49·3–71·0)	62·1% (48·4–68·9)	16·7% (14·7–21·2)	1·2% (0·9–1·7)	0·3% (0·2–0·4)
		Nagaland	4490 (2600–7110)	229·5 (133·1–363·5)	1 300 000 (823 000–2 000 000)	66·3 (42·1–102·4)	60·3% (40·1–90·1)	2·6% (1·6–3·9)	1·1% (0·8–1·6)	0·4% (0·2–0·6)
		Odisha	110 000 (78 600–152 000)	235·6 (168·5–325·9)	44 900 000 (29 700 000–50 900 000)	96·3 (63·6–109·1)	84·0% (59·2–89·9)	2·4% (2·1–3·6)	1·3% (0·9–2·0)	0·3% (0·2–0·4)
		Punjab	115 000 (76 100–175 000)	371·5 (244·9–563·8)	19 000 000 (10 200 000–23 700 000)	61·0 (32·8–76·3)	55·4% (31·7–66·9)	3·4% (2·5–5·9)	1·6% (1·3–2·3)	0·6% (0·4–1·2)
		Rajasthan	115 000 (78 300–160 000)	143·1 (97·4–199·1)	51 900 000 (30 600 000–66 900 000)	64·5 (38·1–83·3)	58·2% (36·6–73·3)	2·0% (1·4–3·1)	1·1% (0·8–1·6)	0·2% (0·1–0·4)
		Sikkim	831 (489–1240)	125·7 (74·1–187·3)	330 000 (229 000–486 000)	49·9 (34·6–73·6)	46·8% (33·4–67·4)	10·1% (6·6–14·1)	1·3% (1·0–1·8)	0·3% (0·1–0·4)
		Tamil Nadu	220 000 (142 000–312 000)	276·0 (178·5–391·4)	59 700 000 (50 700 000–69 800 000)	74·8 (63·5–87·4)	66·8% (57·4–76·4)	4·6% (3·9–5·4)	1·7% (1·3–2·4)	0·4% (0·2–0·5)
		Telangana	46 600 (32 900–63 300)	119·8 (84·6–162·5)	28 300 000 (13 600 000–39 900 000)	72·7 (35·0–102·6)	64·3% (33·5–85·8)	2·5% (1·7–5·0)	1·3% (1·0–1·8)	0·2% (0·1–0·4)
		Tripura	7820 (4980–11 500)	194·3 (123·6–284·7)	2 170 000 (1 590 000–3 200 000)	54·0 (39·4–79·6)	49·6% (36·7–68·7)	4·0% (2·6–5·3)	1·2% (0·9–1·8)	0·4% (0·2–0·6)
		Uttar Pradesh	443 000 (297 000–628 000)	182·3 (122·3–258·6)	200 000 000 (119 000 000–267 000 000)	82·2 (49·1–110·0)	72·1% (46·1–93·8)	0·9% (0·6–1·4)	1·0% (0·7–1·6)	0·2% (0·1–0·4)
		Uttarakhand	55 900 (35 900–82 500)	472·7 (303·6–697·8)	7 960 000 (4 880 000–11 300 000)	67·3 (41·3–95·7)	61·3% (38·4–82·2)	4·5% (3·0–7·0)	1·3% (1·0–1·9)	0·7% (0·4–1·2)
		West Bengal	184 000 (119 000–271 000)	184·5 (119·3–272·0)	61 400 000 (16 300 000–89 000 000)	61·6 (16·3–89·4)	55·7% (16·2–77·0)	3·1% (1·8–9·9)	1·4% (1·0–1·9)	0·4% (0·2–1·2)
	Nepal		105 000 (71 800–153 000)	344·4 (236·0–501·6)	25 400 000 (21 600 000–36 400 000)	83·5 (71·0–119·6)	73·4% (63·0–95·3)	3·3% (2·3–3·8)	1·1% (0·8–1·7)	0·4% (0·2–0·6)
	Pakistan		561 000 (323 000–823 000)	250·5 (144·2–367·5)	176 000 000 (128 000 000–219 000 000)	78·4 (57·1–97·5)	68·3% (51·6–81·5)	0·7% (0·6–1·0)	0·8% (0·6–1·3)	0·3% (0·2–0·5)
		Azad Jammu & Kashmir	11 000 (6820–15 800)	253·0 (157·3–365·1)	3 510 000 (2 390 000–4 730 000)	80·9 (55·1–109·1)	71·4% (51·9–90·9)	1·0% (0·7–1·4)	1·1% (0·8–1·6)	0·3% (0·2–0·5)
		Balochistan	15 000 (7900–23 700)	111·7 (59·0–176·5)	8 310 000 (3 620 000–12 300 000)	62·0 (27·1–91·8)	57·3% (26·7–80·5)	0·4% (0·3–0·9)	0·6% (0·4–1·0)	0·2% (0·1–0·5)
		Gilgit-Baltistan	4350 (2340–6720)	195·5 (105·1–301·7)	1 740 000 (1 030 000–2 070 000)	78·1 (46·4–92·9)	78·1% (46·4–92·9)	0·6% (0·5–1·0)	0·6% (0·4–1·0)	0·3% (0·1–0·5)
		Islamabad Capital Territory	3190 (2190–4260)	148·2 (101·7–197·5)	1 260 000 (652 000–1 800 000)	58·7 (30·3–83·4)	53·9% (29·2–72·4)	9·1% (6·0–16·5)	1·1% (0·7–2·1)	0·3% (0·2–0·5)
		Khyber Pakhtunkhwa	157 000 (75 100–255 000)	412·1 (197·1–669·6)	39 500 000 (26 400 000–46 800 000)	103·6 (69·4–122·9)	84·9% (63·0–91·9)	0·5% (0·4–0·7)	0·7% (0·4–1·2)	0·4% (0·2–0·7)
		Punjab	265 000 (163 000–384 000)	233·0 (143·1–336·6)	75 500 000 (43 100 000–104 000 000)	66·3 (37·8–91·1)	59·8% (35·9–79·3)	0·6% (0·4–1·0)	0·9% (0·7–1·5)	0·4% (0·2–0·6)
		Sindh	105 000 (69 800–147 000)	211·0 (139·8–294·2)	45 900 000 (29 100 000–55 000 000)	92·0 (58·3–110·3)	77·9% (53·4–89·1)	1·1% (0·9–1·6)	0·8% (0·6–1·2)	0·2% (0·1–0·4)
**Southeast Asia, east Asia, and Oceania**			**1 060 000 (723 000–1 660 000)**	**48·9 (33·5–77·0)**	**281 000 000 (181 000 000–382 000 000)**	**13·0 (8·4–17·7)**	**12·1% (8·0–15·9)**	**5·4% (3·8–8·1)**	**1·4% (1·1–2·0)**	**0·4% (0·3–0·6)**
East Asia			16 200 (8820–23 300)	1·1 (0·6–1·6)	2 630 000 (1 470 000–4 790 000)	0·2 (0·1–0·3)	0·2% (0·1–0·3)	5·6% (2·8–9·2)	0·2% (0·1–0·4)	0·7% (0·3–1·0)
	China		14 700 (7490–21 900)	1·0 (0·5–1·5)	2 460 000 (1 340 000–4 550 000)	0·2 (0·1–0·3)	0·2% (0·1–0·3)	5·3% (2·5–8·8)	0·2% (0·1–0·4)	0·6% (0·3–1·0)
	North Korea		593 (417–957)	2·3 (1·6–3·6)	76 100 (49 800–128 000)	0·3 (0·2–0·5)	0·3% (0·2–0·5)	1·5% (1·1–1·9)	2·1% (1·6–2·8)	0·8% (0·6–1·0)
	Taiwan (province of China)		845 (845–845)	3·6 (3·6–3·6)	99 700 (65 100–166 000)	0·4 (0·3–0·7)	0·4% (0·3–0·7)	17·7% (10·0–25·4)	4·5% (3·7–5·2)	0·9% (0·5–1·3)
Oceania			11 600 (6280–19 300)	87·1 (47·3–145·3)	4 540 000 (2 310 000–6 970 000)	34·2 (17·4–52·5)	32·1% (16·8–47·4)	2·6% (1·5–4·6)	0·9% (0·6–1·5)	0·3% (0·2–0·6)
	Fiji		1300 (832–2140)	143·1 (91·3–234·7)	307 000 (149 000–457 000)	33·7 (16·4–50·2)	32·8% (16·3–48·0)	18·6% (11·5–35·1)	1·4% (1·1–2·1)	0·5% (0·3–1·0)
	Guam		295 (259–439)	172·6 (151·8–257·1)	83 900 (56 500–120 000)	49·2 (33·1–70·4)	45·3% (30·5–62·9)	23·9% (15·9–33·9)	1·8% (1·5–2·2)	0·4% (0·2–0·5)
	Northern Mariana Islands		7 (4–10)	16·3 (10·6–22·7)	3010 (1960–4490)	7·1 (4·6–10·6)	1·2% (0·3–2·7)	21·1% (13·6–30·7)	0·0% (0·0–0·0)	0·3% (0·1–0·4)
	Papua New Guinea		9920 (5200–17 100)	100·6 (52·7–173·1)	4 150 000 (1 960 000–6 510 000)	42·0 (19·9–66·0)	39·3% (19·4–59·0)	0·9% (0·5–1·8)	0·9% (0·6–1·4)	0·3% (0·1–0·6)
	Vanuatu		36 (13–69)	12·1 (4·4–23·6)	2950 (1080–6660)	1·0 (0·4–2·3)	0·8% (0·3–2·1)	0·3% (0·1–0·5)	0·7% (0·5–1·2)	1·5% (0·5–4·2)
Southeast Asia			1 030 000 (702 000–1 630 000)	152·5 (104·1–242·4)	274 000 000 (175 000 000–372 000 000)	40·7 (25·9–55·3)	37·7% (24·8–49·7)	5·5% (3·9–8·3)	1·4% (1·1–2·0)	0·4% (0·3–0·6)
	Cambodia		14 300 (9720–21 700)	86·1 (58·5–130·4)	3 700 000 (1 630 000–5 720 000)	22·3 (9·8–34·4)	21·7% (9·7–32·8)	3·6% (2·1–7·4)	1·0% (0·8–1·5)	0·4% (0·3–1·0)
	Indonesia		639 000 (410 000–1 090 000)	246·2 (158·2–419·2)	161 000 000 (97 100 000–229 000 000)	62·1 (37·4–88·4)	56·6% (35·4–76·2)	2·8% (1·9–4·4)	1·4% (1·1–2·0)	0·4% (0·3–0·7)
	Laos		1090 (669–1680)	15·2 (9·3–23·4)	1 250 000 (583 000–2 070 000)	17·5 (8·1–28·9)	17·1% (8·0–28·1)	6·3% (3·2–11·4)	1·0% (0·7–1·5)	0·2% (0·1–0·5)
	Malaysia		40 700 (30 600–59 300)	130·0 (97·7–189·3)	10 200 000 (6 780 000–14 300 000)	32·4 (21·7–45·8)	31·7% (21·4–44·2)	26·6% (18·2–38·5)	1·7% (1·4–2·0)	0·4% (0·3–0·6)
	Maldives		270 (247–362)	54·2 (49·5–72·6)	187 000 (140 000–324 000)	37·5 (28·1–65·0)	35·4% (26·8–58·3)	51·3% (28·1–65·1)	1·3% (1·1–1·5)	0·2% (0·1–0·2)
	Mauritius		269 (266–292)	21·1 (20·8–22·9)	181 000 (117 000–265 000)	14·2 (9·2–20·7)	14·1% (9·1–20·5)	31·4% (19·9–45·8)	2·0% (1·6–2·4)	0·4% (0·2–0·6)
	Myanmar		85 900 (53 500–140 000)	157·1 (97·8–256·3)	17 800 000 (9 630 000–25 300 000)	32·6 (17·6–46·3)	31·4% (17·2–43·8)	3·1% (2·1–5·4)	1·4% (1·0–2·1)	0·5% (0·3–0·9)
	Philippines		158 000 (111 000–236 000)	140·5 (99·3–210·2)	59 200 000 (36 900 000–84 800 000)	52·8 (32·9–75·6)	48·6% (31·3–66·6)	5·1% (3·3–7·7)	1·2% (0·9–1·7)	0·3% (0·2–0·5)
	Seychelles		121 (120–122)	118·3 (117·6–119·7)	52 600 (41 700–60 500)	51·5 (40·9–59·3)	45·8% (37·6–51·1)	44·3% (38·3–55·4)	1·5% (1·3–1·9)	0·2% (0·2–0·3)
	Sri Lanka		14 000 (14 000–14 100)	64·1 (64·0–64·6)	3 400 000 (2 520 000–4 460 000)	15·6 (11·5–20·4)	15·3% (11·4–19·9)	17·0% (12·6–22·3)	2·0% (1·7–2·4)	0·4% (0·3–0·6)
	Thailand		28 300 (21 600–36 900)	40·3 (30·8–52·7)	8 100 000 (4 370 000–12 800 000)	11·6 (6·2–18·3)	11·4% (6·2–18·0)	28·3% (16·3–47·9)	2·7% (2·2–3·2)	0·4% (0·2–0·7)
	Timor-Leste		1120 (741–1730)	84·1 (55·5–129·7)	410 000 (209 000–619 000)	30·7 (15·7–46·4)	29·6% (15·3–43·8)	5·3% (3·2–9·5)	0·9% (0·6–1·3)	0·3% (0·2–0·5)
	Vietnam		45 200 (28 100–69 600)	46·9 (29·1–72·2)	8 460 000 (4 760 000–14 900 000)	8·8 (4·9–15·4)	8·7% (4·9–15·3)	15·1% (8·1–25·1)	1·9% (1·5–2·4)	0·7% (0·4–1·0)
**Sub-Saharan Africa**			**1 750 000 (1 100 000–2 560 000)**	**162·6 (102·0–237·8)**	**855 000 000 (744 000 000–932 000 000)**	**79·3 (69·0–86·4)**	**70·5% (61·6–75·9)**	**0·7% (0·7–0·8)**	**0·6% (0·4–0·9)**	**0·2% (0·1–0·3)**
Central sub-Saharan Africa			161 000 (95 900–244 000)	122·2 (72·9–185·3)	117 000 000 (76 100 000–138 000 000)	89·2 (57·9–104·6)	76·3% (52·8–86·1)	0·2% (0·1–0·3)	0·5% (0·4–0·9)	0·1% (0·1–0·2)
	Angola		53 200 (32 100–80 800)	176·6 (106·5–267·9)	30 000 000 (15 300 000–37 000 000)	99·7 (50·8–122·9)	84·0% (47·5–95·8)	0·2% (0·2–0·4)	0·5% (0·4–0·8)	0·2% (0·1–0·4)
	Central African Republic		12 000 (6660–21 300)	226·6 (125·8–401·2)	3 640 000 (2 620 000–5 330 000)	68·7 (49·4–100·6)	61·9% (46·2–84·0)	0·3% (0·2–0·4)	0·4% (0·3–0·9)	0·3% (0·2–0·6)
	Congo (Brazzaville)		8880 (5780–13 400)	168·7 (109·7–254·2)	4 330 000 (3 090 000–5 270 000)	82·2 (58·7–100·0)	71·7% (53·3–85·9)	0·4% (0·4–0·6)	0·5% (0·4–0·9)	0·2% (0·1–0·4)
	DR Congo		81 100 (48 400–133 000)	92·5 (55·2–151·9)	76 900 000 (46 300 000–91 000 000)	87·7 (52·8–103·8)	75·1% (48·5–86·0)	0·1% (0·1–0·1)	0·5% (0·3–0·9)	0·1% (0·1–0·2)
	Equatorial Guinea		2280 (1510–3600)	160·6 (106·3–253·3)	1 100 000 (797 000–1 340 000)	77·4 (56·1–94·2)	70·2% (53·6–83·6)	1·2% (1·0–1·7)	0·5% (0·4–0·8)	0·2% (0·1–0·4)
	Gabon		3170 (2120–4420)	181·4 (121·3–252·8)	1 300 000 (652 000–1 870 000)	74·5 (37·3–107·1)	65·5% (35·9–86·5)	3·1% (2·0–5·7)	0·7% (0·6–1·1)	0·3% (0·2–0·6)
Eastern sub-Saharan Africa			827 000 (509 000–1 300 000)	200·8 (123·5–316·2)	344 000 000 (304 000 000–378 000 000)	83·5 (73·7–91·9)	72·9% (64·3–79·2)	0·5% (0·4–0·5)	0·5% (0·4–0·9)	0·2% (0·1–0·4)
	Burundi		4080 (2580–6320)	34·2 (21·6–52·9)	1 850 000 (1 360 000–2 400 000)	15·5 (11·4–20·1)	15·3% (11·3–19·8)	1·1% (0·8–1·5)	0·5% (0·3–0·9)	0·2% (0·1–0·4)
	Comoros		1200 (743–1760)	168·0 (104·1–246·6)	479 000 (362 000–606 000)	67·0 (50·7–84·9)	67·0% (50·7–84·8)	0·9% (0·7–1·2)	0·6% (0·5–1·1)	0·3% (0·2–0·4)
	Djibouti		3250 (2070–4690)	270·2 (172·3–389·6)	770 000 (450 000–1 180 000)	64·0 (37·4–98·4)	58·2% (35·5–84·3)	1·8% (1·1–3·0)	0·7% (0·5–1·1)	0·4% (0·2–0·7)
	Eritrea		4110 (2490–6280)	61·3 (37·1–93·6)	1 910 000 (1 430 000–2 490 000)	28·4 (21·3–37·1)	27·7% (20·8–36·1)	0·4% (0·3–0·5)	0·6% (0·4–1·0)	0·3% (0·2–0·5)
	Ethiopia		170 000 (99 100–273 000)	158·2 (92·1–253·5)	105 000 000 (81 900 000–124 000 000)	97·8 (76·1–115·0)	83·2% (67·9–90·9)	0·4% (0·3–0·5)	0·5% (0·3–0·8)	0·2% (0·1–0·3)
	Kenya		145 000 (88 500–244 000)	288·1 (176·3–486·4)	50 700 000 (40 700 000–57 600 000)	101·0 (81·1–114·6)	84·1% (71·6–92·3)	0·5% (0·4–0·6)	0·6% (0·4–1·0)	0·3% (0·2–0·5)
	Madagascar		52 400 (32 400–87 200)	196·4 (121·3–326·6)	23 400 000 (18 500 000–29 000 000)	87·7 (69·3–108·7)	75·5% (63·0–90·2)	0·2% (0·2–0·2)	0·5% (0·4–0·9)	0·2% (0·1–0·4)
	Malawi		45 700 (29 500–70 800)	247·9 (160·0–383·8)	17 000 000 (11 100 000–19 100 000)	92·4 (60·0–103·5)	86·7% (57·3–94·4)	0·4% (0·3–0·6)	0·6% (0·4–1·0)	0·3% (0·2–0·5)
	Mozambique		63 900 (41 000–88 500)	216·4 (138·9–299·8)	30 300 000 (23 000 000–33 500 000)	102·6 (77·8–113·4)	89·3% (68·9–98·1)	0·5% (0·5–0·7)	0·5% (0·4–0·9)	0·2% (0·1–0·3)
	Rwanda		18 600 (12 500–26 800)	146·4 (98·5–211·5)	5 980 000 (3 350 000–9 270 000)	47·1 (26·4–73·1)	44·2% (25·4–66·0)	1·8% (1·1–3·0)	0·6% (0·5–1·0)	0·3% (0·2–0·6)
	Somalia		75 400 (36 900–143 000)	370·6 (181·3–702·7)	19 500 000 (15 700 000–23 700 000)	95·8 (77·4–116·7)	80·5% (68·4–90·0)	0·1% (0·1–0·1)	0·4% (0·3–0·8)	0·4% (0·2–0·9)
	South Sudan		12 400 (6520–20 300)	133·4 (70·3–218·4)	5 620 000 (3 370 000–9 350 000)	60·6 (36·3–100·7)	56·5% (35·5–88·4)	0·2% (0·1–0·4)	0·4% (0·3–0·8)	0·2% (0·1–0·5)
	Tanzania		101 000 (62 300–165 000)	178·5 (109·9–290·8)	40 700 000 (33 800 000–45 500 000)	71·7 (59·5–80·3)	64·7% (55·1–71·5)	0·4% (0·3–0·5)	0·6% (0·5–1·0)	0·3% (0·2–0·4)
	Uganda		61 200 (41 600–92 500)	148·8 (101·2–224·9)	22 700 000 (16 500 000–30 900 000)	55·1 (40·2–75·1)	51·4% (37·6–69·5)	0·6% (0·4–0·8)	0·5% (0·4–0·9)	0·3% (0·2–0·4)
	Zambia		68 400 (44 600–113 000)	375·1 (244·8–618·7)	17 700 000 (12 100 000–20 300 000)	97·0 (66·2–111·1)	87·7% (60·8–98·7)	1·2% (1·0–1·7)	0·6% (0·5–1·0)	0·4% (0·2–0·7)
Southern sub-Saharan Africa			378 000 (273 000–533 000)	481·5 (347·5–678·1)	58 200 000 (47 800 000–67 900 000)	74·1 (60·8–86·4)	67·8% (56·3–77·3)	6·1% (5·1–7·3)	1·0% (0·8–1·2)	0·7% (0·5–1·0)
	Botswana		14 800 (10 000–20 900)	633·5 (428·8–893·6)	1 600 000 (749 000–2 300 000)	68·5 (32·0–98·5)	65·8% (31·4–94·8)	13·6% (8·4–25·9)	0·9% (0·7–1·2)	1·0% (0·5–2·3)
	Eswatini		11 300 (7440–17 900)	985·6 (651·5–1568·4)	866 000 (451 000–1 280 000)	75·8 (39·5–112·3)	67·4% (38·0–94·3)	5·8% (3·6–10·3)	0·7% (0·5–0·9)	1·4% (0·8–2·7)
	Lesotho		15 200 (9540–23 900)	725·6 (456·3–1140·9)	1 430 000 (713 000–2 190 000)	68·3 (34·1–104·8)	61·9% (32·1–90·9)	1·7% (1·0–3·0)	0·8% (0·6–1·0)	1·2% (0·6–2·9)
	Namibia		15 200 (10 700–23 200)	634·5 (444·4–963·9)	1 720 000 (1 010 000–2 450 000)	71·6 (41·8–102·1)	66·4% (39·9–93·7)	7·9% (5·3–12·8)	0·9% (0·7–1·2)	0·9% (0·6–1·7)
	South Africa		257 000 (190 000–370 000)	461·9 (341·4–664·9)	38 600 000 (30 500 000–46 100 000)	69·4 (54·9–82·9)	64·0% (50·8–75·1)	7·8% (6·4–9·7)	1·1% (0·9–1·4)	0·7% (0·5–1·1)
	Zimbabwe		65 000 (41 400–93 400)	433·3 (276·1–622·0)	14 000 000 (6 470 000–16 100 000)	93·3 (43·1–107·4)	83·1% (40·9–95·6)	1·0% (0·8–2·1)	0·7% (0·5–0·9)	0·5% (0·3–1·0)
Western sub-Saharan Africa			387 000 (235 000–548 000)	84·9 (51·5–120·1)	336 000 000 (270 000 000–387 000 000)	73·7 (59·2–84·8)	67·0% (55·5–77·2)	0·2% (0·2–0·3)	0·5% (0·4–0·9)	0·1% (0·1–0·2)
	Benin		7000 (4520–10 500)	55·2 (35·7–82·7)	4 260 000 (2 520 000–6 580 000)	33·6 (19·9–52·0)	32·2% (19·5–49·0)	0·6% (0·4–1·0)	0·5% (0·4–0·9)	0·2% (0·1–0·3)
	Burkina Faso		14 400 (8450–23 800)	63·7 (37·2–104·7)	17 100 000 (11 700 000–22 800 000)	75·4 (51·7–100·3)	68·5% (49·1–86·7)	0·1% (0·1–0·1)	0·5% (0·4–0·8)	0·1% (0·1–0·2)
	Cape Verde		560 (421–801)	99·3 (74·7–142·2)	368 000 (196 000–508 000)	65·3 (34·8–90·2)	65·2% (34·6–90·1)	11·1% (7·5–19·5)	1·0% (0·8–1·4)	0·2% (0·1–0·3)
	Cameroon		33 400 (21 300–49 400)	114·9 (73·1–169·7)	20 200 000 (2 570 000–30 400 000)	69·4 (8·8–104·5)	61·8% (8·8–86·8)	0·9% (0·3–4·2)	0·5% (0·4–0·8)	0·3% (0·1–1·5)
	Chad		12 600 (6640–19 700)	76·7 (40·5–120·2)	9 070 000 (4 990 000–14 600 000)	55·3 (30·4–88·8)	54·0% (30·0–85·6)	0·1% (0·0–0·1)	0·4% (0·3–0·7)	0·2% (0·1–0·3)
	Côte d'Ivoire		28 200 (16 900–41 700)	107·8 (64·4–159·2)	20 000 000 (13 900 000–25 200 000)	76·4 (53·0–96·1)	68·2% (49·4–80·7)	0·3% (0·2–0·4)	0·5% (0·4–0·9)	0·1% (0·1–0·2)
	The Gambia		5370 (3490–7590)	239·2 (155·5–338·0)	1 980 000 (1 300 000–2 580 000)	88·4 (58·0–114·7)	75·2% (53·2–89·8)	0·5% (0·4–0·8)	0·5% (0·4–0·9)	0·3% (0·2–0·4)
	Ghana		28 300 (19 600–38 900)	89·7 (62·2–123·3)	21 800 000 (14 800 000–26 700 000)	69·0 (46·8–84·5)	62·3% (44·1–75·0)	0·6% (0·5–0·9)	0·7% (0·5–1·1)	0·1% (0·1–0·2)
	Guinea		22 500 (12 800–37 300)	177·9 (101·6–294·9)	11 500 000 (7 760 000–15 100 000)	91·0 (61·4–119·3)	77·5% (57·5–91·6)	0·3% (0·2–0·4)	0·5% (0·4–0·9)	0·2% (0·1–0·4)
	Guinea-Bissau		3600 (2180–5130)	189·2 (114·6–270·0)	1 340 000 (799 000–2 080 000)	70·5 (42·0–109·7)	65·3% (41·4–87·7)	0·5% (0·3–0·8)	0·5% (0·3–0·8)	0·3% (0·2–0·6)
	Liberia		6790 (4300–10 100)	141·8 (89·7–210·9)	3 230 000 (2 020 000–4 510 000)	67·5 (42·1–94·2)	60·8% (39·9–80·5)	0·2% (0·1–0·3)	0·6% (0·5–1·0)	0·2% (0·1–0·4)
	Mali		25 300 (15 500–36 800)	115·3 (70·6–168·0)	17 500 000 (10 900 000–22 200 000)	79·7 (49·6–101·2)	75·0% (48·2–93·3)	0·1% (0·1–0·2)	0·5% (0·4–0·8)	0·2% (0·1–0·3)
	Mauritania		6340 (3660–9250)	158·0 (91·3–230·5)	2 970 000 (1 730 000–4 510 000)	74·0 (43·2–112·3)	67·6% (41·8–90·7)	1·4% (0·9–2·2)	0·7% (0·5–1·0)	0·2% (0·1–0·4)
	Niger		12 900 (6910–19 600)	55·2 (29·7–83·9)	12 600 000 (6 640 000–18 900 000)	54·2 (28·5–81·0)	51·0% (28·0–72·3)	0·1% (0·0–0·1)	0·4% (0·3–0·7)	0·1% (0·1–0·3)
	Nigeria		133 000 (68 300–197 000)	62·0 (31·8–91·9)	170 000 000 (126 000 000–217 000 000)	79·2 (58·5–101·2)	72·3% (55·1–88·5)	0·1% (0·1–0·2)	0·5% (0·4–0·8)	0·1% (0·0–0·1)
	São Tomé and Príncipe		201 (132–284)	98·1 (64·0–138·4)	109 000 (82 700–137 000)	52·9 (40·3–66·9)	52·9% (40·2–66·8)	3·4% (2·6–4·4)	0·5% (0·4–0·9)	0·2% (0·1–0·3)
	Senegal		32 900 (21 100–50 700)	217·4 (139·7–334·9)	13 800 000 (10 700 000–16 500 000)	91·2 (70·5–108·8)	78·3% (65·1–87·4)	0·5% (0·4–0·7)	0·5% (0·4–0·9)	0·2% (0·2–0·4)
	Sierra Leone		6330 (4070–9140)	76·4 (49·2–110·3)	3 520 000 (2 160 000–4 930 000)	42·5 (26·1–59·5)	40·5% (25·5–55·3)	0·2% (0·1–0·3)	0·5% (0·4–0·9)	0·2% (0·1–0·3)
	Togo		7490 (4910–10 900)	94·5 (62·0–138·1)	4 690 000 (3 220 000–6 130 000)	59·3 (40·7–77·4)	53·9% (38·5–68·7)	0·6% (0·4–0·8)	0·6% (0·4–1·0)	0·2% (0·1–0·3)

Data are estimates (95% uncertainty interval).

Cumulative total COVID-19 deaths and death rates on Nov 14, 2021, can be found in the table and appendix 1 (section 9.4). Although roughly 5·6 million deaths due to COVID-19 had been reported by this date, estimated total deaths attributable to COVID-19 were nearly three times as high at 15·1 million (95% UI 11·2–20·2)—a rate of 195 deaths per 100 000 people (145–262). Across all countries and territories, the estimated death rate ranged from no more than 1 per 100 000 people in New Zealand and China to 1125 (724–1709) in Bolivia. Death rates over 450 per 100 000 were estimated in 23 countries, as well as many states in Mexico; multiple states in Brazil, Italy, and the USA; and one in India. At least one country in every super-region except southeast Asia, east Asia, and Oceania surpassed 300 estimated deaths per 100 000, 51 in total. Estimated death rates remained very low throughout much of east and southeast Asia, high-income Asia Pacific, Australasia, and select countries such as Norway, Iceland, and Qatar.

Posterior estimates of the IDR showed that 44·6% (95% UI 42·3–47·2) of COVID-19 infections were detected in the high-income super-region, with 18 countries and parts of Canada, Italy, Spain, and the USA detecting over half of the COVID-19 infections that occurred in those locations by Nov 14, 2021. Countries in Latin America and the Caribbean and central Europe, eastern Europe, and central Asia detected about 10% of infections on average, and fewer than 10% of infections were identified in each of the remaining four super-regions (table). The IHR varied by a factor of four across super-regions, and the IFR by a factor of five. The highest IHR and IFR were estimated primarily in countries with older population structures, such as Japan. The lowest IDR, IHR, and IFR were all detected in sub-Saharan Africa, where only the southern region exceeded 0·5% for any ratio (table).

**Figure 4:**
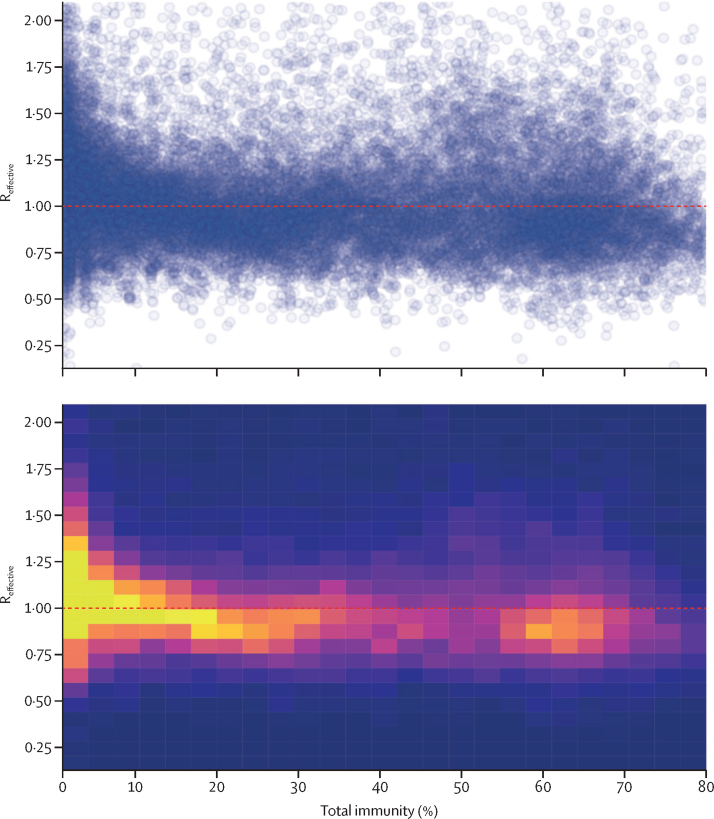
*R*_effective_ by total immunity Proportion of total immunity shown starting at 1%. R_effective_=effective reproductive number.

During the first 20 months of the pandemic, R_effective_ varied widely across locations and time, from lower than 0·1 to higher than 2·0. Only 39% of location-weeks for which total immunity was under 10% had R_effective_ lower than 1. Between 10% and 20% total immunity, this proportion increased to 56%, and between 20% and 30% total immunity, we observed an additional increase to 65% of location-weeks with an R_effective_ lower than 1 (figure 4). However, over the range of 30–60% total immunity, the percentage of observations with R_effective_ lower than 1 decreased back to 55%. This absence of a clear relationship highlights the many other factors such as seasonality, physical distancing mandates, mask use, and new variant spread that have influenced R_effective_ over time. From 60% to 70% total immunity, we observed 60% of observations with R_effective_ lower than 1, and above 70% total immunity, 72% of location-weeks had an R_effective_ lower than 1. Although these data suggest transmission to be somewhat lower at the highest levels of total immunity observed thus far, even with total immunity at 80%, we saw no indication of an abrupt drop in R_effective_.

## Discussion

In this study, we estimated that global daily SARS-CoV-2 infections fluctuated between 3 million and 17 million new cases per day from April, 2020, to October, 2021. In total, we estimated that between the start of the pandemic and Nov 14, 2021, there were 3·80 billion (95% UI 3·44–4·08) total SARS-CoV-2 infections and reinfections combined and that 3·39 billion (3·08–3·63) individuals had been infected with SARS-CoV-2 one or more times. The proportion of the population that had been infected at least once ranged from under 1% to over 80% across countries and territories. The highest cumulative infection rates were estimated in sub-Saharan Africa; central Europe, eastern Europe, and central Asia; and south Asia. Translating daily infections into R_effective_ showed no clear herd immunity threshold.

Cumulative infection rates through Nov 14, 2021, varied greatly across countries and territories and between subnational units within countries. This variation can be explained by a combination of factors including policies enacted by governments to encourage mask use and reduce social interaction,^48^, ^49^, ^50^ presence of escape variants, testing and contact tracing capacity,^51^, ^52^ previous exposure to other coronaviruses,^53^ baseline patterns of social interaction, and more. For instance, greatly different levels of cumulative infection were found in some neighbouring countries with similar patterns of non-COVID-19 disease burden, such as Norway and Sweden.^43^ In these two countries, testing and contact tracing strategies, government restrictions, and mobility patterns varied substantially,^54^ contributing to substantially different SARS-CoV-2 infection outcomes. Other countries, such as Australia and New Zealand, have shown how early and effective lockdowns, combined with geographical isolation and travel restrictions, have kept transmission low throughout the study period.^18^, ^55^, ^56^ Excess mortality and seroprevalence data available suggest that some of the most severe COVID-19 epidemics occurred in eastern Europe and central Asia. This might be related to comparatively less public intervention, such as mask mandates or stay-at-home orders.^57^, ^58^ But, although it might be tempting to ascribe all variations in cumulative infections to effective public health action in different countries, the April–September, 2021, surges in many southeast Asian countries where, up to the end of March, public health responses to the pandemic had been swift and believed to be effective,^59^, ^60^, ^61^ suggest that other factors might also be contributing to these patterns.^57^, ^58^

The empirical measurements of the IDR suggest that it was low early in the pandemic, when testing was scarce, and increased as testing capacity expanded. On average, the IDR increased steadily, especially over the course of the first year of the pandemic, but with marked variation across countries. This variation highlights how analyses based on the assumption that SARS-CoV-2 IDR is constant across location and time^7^ could be very misleading. Although we expect that, in general, IDR increased as testing capacity increased, national guidance on who should be tested, and changes in that guidance over the course of the pandemic, might also affect the IDR. For example, on May 1, 2021, the CDC issued guidance not to test vaccinated individuals who had been exposed to COVID-19 but did not have symptoms.^62^ Likewise, the advent of workplace and school testing programmes in the later months of 2021 might also shift the IDR up in some countries. Great care needs to be taken when interpreting trends based only on reported cases in the later phases of the pandemic. In many settings, hospitalisations—which tend to be a robust measure of more severe disease—are likely to be more informative than confirmed infections.

Our analysis suggests that the cumulative IFR across countries and territories ranged from 0·1% to 2·0% as of Nov 14, 2021. Age standardisation has been shown to explain a considerable portion of this variation,^40^ but substantial differences remain in the available data. Some of this variation appears to be due to the prevalence of certain comorbidites,^63^ and some could be residual errors in the estimation of excess mortality or seroprevalence in the available data. Nevertheless, it might turn out that other factors, such as previous exposure to other coronaviruses, help explain the considerable variation in the age-standardised IFR that is observed in the data. Such variation in the IFR should caution against studies that assume the IFR (either all-age or age-standardised) is constant across locations and over time. The temporal analysis of the IFR supports the clinical observation that the IFR was initially much higher in March and April, 2020, and subsequently declined as clinical practice improved, particularly in approaches to oxygenation and the use of corticosteroids.^64^, ^65^, ^66^, ^67^ Trials of some oral antivirals have shown substantial effectiveness in preventing severe disease and death, suggesting that the IFR might decline further in the coming months if these and other antivirals become widely available and if diagnostic capacity is able to support early treatment.^68^, ^69^

We did not find a clear relationship between R_effective_ and total immunity up to 60%. Over 60%, R_effective_ was more often under 1·0 than over 1·0. Despite this finding, we observed no obvious herd immunity threshold in the data. The generally weak relationship between R_effective_ and the total immunity highlights the powerful role of other factors driving infection, including physical distancing mandates, seasonality, mask use, and the emergence of new variants over the study period (especially the delta variant) in mediating this relationship. Although figure 4 does not show us the prospects for reaching herd immunity in each location for any given season or variant, the overall relationship points to the very high degree of combined natural and vaccine-derived immunity that might be needed to block community transmission (especially in the winter months).

This empirical analysis has several important limitations. First, some seroprevalence surveys (such as the CDC monitoring of laboratory data) might be biased, but the direction of the bias is difficult to ascertain. Additionally, in reporting serosurveys, various corrections can be applied to produce estimates, including the use of sampling weights, correcting for manufacturing sensitivity and specificity, or, in some instances, full correction for waning detectability. Where possible, we attempted to standardise for this by extracting data that were adjusted for sampling frame and manufacturing sensitivity, but not more complex corrections. If this was not possible, we used the raw numerator and denominator as reported. In some instances, no metadata were provided to describe whether any correction had been applied. In all instances, these values were treated as equivalent. Second, we have assumed that one of the key covariates for the IDR is demonstrated testing capacity. By construction, this variable cannot decline as it is the maximum value of previously observed daily testing rates. In some countries, changes in guidance on who gets tested could lead to declines in effective testing and the IDR, and we may have missed these changes. The CDC guidance in spring, 2021, not to test vaccinated individuals who were asymptomatic or mildly symptomatic is an example of such a policy. Third, vaccination increases the proportion of the population who test positive on anti-spike antibody tests. We note in some locations, particularly in the UK, attempts to account for vaccination rates resulted in decreasing estimates of seroprevalence over time, suggesting that assumptions about the probability of vaccinated individuals being identified in serological surveys in those locations are incompatible with the data collected; in these instances, we excluded the seroprevalence data from the analysis. Fourth, matched seroprevalence surveys with reported cumulative cases, hospitalisations, and deaths provide an interval measure of the IDR, IHR, and IFR from the beginning of the pandemic to the period of the survey. We used these interval measures to derive relationships for the daily IDR, IHR, and IFR. This approach decreases our ability to identify drivers of shorter-term fluctuations in these key rates. Fifth, the availability of hospital admissions data in low-income and middle-income settings was generally low, minimising its effect on the estimation process in many countries. Sixth, we used estimates of total COVID-19 mortality based on the measurement or estimation of excess mortality multiplied by a statistical estimate of the proportion of excess mortality directly attributable to infection with SARS-CoV-2. This statistical estimation was based on removing the effect of a low IDR and reduced mobility that might be a proxy for deferred care and other health effects of isolation. This estimate of the proportion of excess mortality that is total COVID-19 has wide UIs. Eventually, better data will emerge on causes of death during the pandemic that will hopefully refine the estimate of total COVID-19 deaths. The wide uncertainty in the ratio of total COVID-19 to reported COVID-19 is reflected in the uncertainty analysis in this study. Seventh, our model permitted a maximum of two infections per individual—in the case where a person gets an ancestral or alpha variant infection, they might also be infected with a beta, gamma, or delta variant. There is evidence of waning naturally derived immunity, suggesting that an individual might become more broadly susceptible to reinfection sometime after exposure.^70^

This empirical analysis of past COVID-19 infections ends at the point where the omicron (B.1.1.529) wave was first detected in Gauteng province in South Africa. Omicron is much more transmissible than previous variants and has shown immune escape.^71^ Since Nov 14, 2021, the omicron wave has taken off in all countries and territories. Because of much lower severity of disease, the IDR is likely to have dropped considerably during the omicron wave. Models suggest that more than 50% of the world might have been infected with omicron already—however, a detailed analysis will have to await new seroprevalence data emerging in the coming months. Cumulative infections for COVID-19 through to March, 2022, might be nearly double what occurred through Nov 14, 2021.

### Conclusion

COVID-19 has had a staggering impact on the world, with 3·39 billion (95% UI 3·08–3·63) people infected with SARS-CoV-2 at least once as of Nov 14, 2021. These findings highlight the potential for COVID-19 to have a continued and profound impact on the world's population. The vast differences in cumulative proportion of the population infected across countries and territories can help policy makers identify locations whose transmission-prevention strategies should be emulated, as well as those populations at greatest risk of future infection—a factor that should be considered in global vaccine prioritisation. Our statistical approach to estimating SARS-CoV-2 infection, which can be applied routinely and will allow for rapid availability of estimates, will be crucially important for research, science, and policy efforts towards pandemic preparedness, response, and control in the coming months and years. It has and continues to be made freely available to all on a routine basis.

For the **analysis code** see https://github.com/ihmeuw/covid-historical-model and https://github.com/ihmeuw/covid-model-infections

For the **latest estimates of daily infections** see https://covid19.healthdata.org

## Data sharing

To download the data used in these analyses, please visit the Global Health Data Exchange website (http://ghdx.healthdata.org/record/ihme-data/covid_19_cumulative_infections). Data sources are also listed by location and institution in appendix 2.

## Declaration of interests

C Adolph reports support for the present manuscript from the Benificus Foundation for collection of data on state level social distancing policies in the USA. X Dai reports support for the present manuscript from paid salary through their employment at the Institute for Health Metrics and Evaluation and the University of Washington. A Flaxman reports stock or stock options from Agathos for technical advising on health metrics; and other support from Janssen, SwssRe, Merck for Mothers, and Sanofi for technical advising on simulation modelling, all outside the submitted work. N Fullman reports funding support for work unrelated to this Article from WHO as a consultant from June to September, 2019, and Gates Ventures since June, 2020, all outside the submitted work. S Nomura reports support for the present manuscript from a Ministry of Education, Culture, Sports, Science and Technology of Japan grant. D M Pigott reports support for the present manuscript from the Bill & Melinda Gates Foundation. All other authors declare no competing interests.
