# Supplementary material for: Estimating global, regional, and national daily and cumulative infections with SARS-CoV-2 through Nov 14, 2021: a statistical analysis
Source: Lancet. 2022 Jun 25;399(10344):2351–80. doi: 10.1016/S0140-6736(22)00484-6 (PMC8993157; doi:10.1016/S0140-6736(22)00484-6)
Supplement: Supplementary appendix 1 [file mmc1.pdf]

THE LANCET

Supplementary appendix 1

This appendix formed part of the original submission and has been peer reviewed. We post it as supplied by the authors.

Supplement to: COVID-19 Cumulative Infection Collaborators. Estimating global, regional, and national daily and cumulative infections with SARS-CoV-2 through Nov 14, 2021: a statistical analysis. *Lancet* 2022; published online April 8. https://doi.org/10.1016/S0140-6736(22)00484-6.

Appendix 1: supplementary methods and results to "Estimating global, regional, and national daily and cumulative infections with SARS-CoV-2 through Nov 14, 2021: a statistical analysis"

This appendix provides further methodological details and supplementary results for "Estimating global, regional, and national daily and cumulative infections with SARS-CoV-2 through Nov 14, 2021: a statistical analysis".

Table of Contents

Section 1: List of abbreviations 4
Section 2: GATHER compliance 4
Table S1. GATHER checklist 4
Section 3: Locations, populations, and time period of analysis 5
Section 4: Data inputs 5
Section 4.1: Reported cases data 5
Section 4.2: Hospital admissions data 5
Section 4.3: Reported deaths data 6
Section 4.4: Seroprevalence data 6
Section 5: Seroprevalence survey adjustments 6
Section 5.1: Adjusting for vaccinations 6
Section 5.2: Adjusting for reinfection from escape variants 7
Section 5.3: Adjusting for seroreversion 7
Section 6: Modelling IDR, IHR, and IFR 8
Section 6.1: Indirect age-standardisation 8
Section 6.2: Bayesian cascading regression framework 8
Section 6.3: Modelling IDR 8
Table S2: IDR model lambdas 9
Section 6.4: Modelling IHR 9
Table S3: IHR model lambdas 9
Section 6.5: Modelling IFR 9
Table S3: IFR model lambdas 10
Section 6.6: Effect of vaccinations and variants on IHR and IFR 10
Section 7: Robust estimate of daily infections 11
Section 8: Cumulative infections and the cumulative proportion of the population infected at least once 12
Section 9: Ensemble model framework 12
Section 9.1: Overview 12
Section 9.2: Seroprevalence data 12
Section 9.3: Sensitivity decay functions 12
Figure S1: Sensitivity decay functions for eight commercial serology assays 13
Section 9.4: Total COVID-19 scalars 13
Figure S2: Cumulative total COVID-19 death rate (per 100,000 population) by November 14, 2021 15
Section 9.5: Ratio model parameters 15
Section 9.6: IDR, IHR, and IFR model 15
Table S4: Covid co-morbidity covariates and priors 16
Section 9.7: Estimating daily infections 16

Section 10: Sensitivity analyses....16
Figure S3: Sensitivity analyses....17
Section 11: References....18
Section 12: Author contributions....19
Managing the estimation or publications process....19
Writing the first draft of the manuscript....19
Primary responsibility for applying analytical methods to produce estimates....19
Primary responsibility for seeking, cataloguing, extracting, or cleaning data; designing or coding figures and tables....19
Providing data or critical feedback on data sources....19
Developing methods or computational machinery....19
Providing critical feedback on methods or results....19
Drafting the work or revising it critically for important intellectual content....19
Managing the overall research enterprise....19

Section 1: List of abbreviations

Abbreviation	Full phrase
B.1.1.7	SARS-CoV-2 alpha variant
B.1.351	SARS-CoV-2 beta variant
B.1.617	SARS-CoV-2 delta variant
B.1.1.529	SARS-CoV-2 omicron variant
COVID-19	coronavirus disease 2019
CDC	Centers for Disease Control and Prevention
GATHER	Guidelines for Accurate and Transparent Health Estimates Reporting
GBD	Global Burden of Diseases, Injuries, and Risk Factors Study
IDR	infection–detection ratio
IFR	infection–fatality ratio
IHR	infection–hospitalisation ratio
MRTool	Meta-Regression Tool
P.1	SARS-CoV-2 Gamma variant
RT-PCR	reverse transcription-polymerase chain reaction
SARS-CoV-2	severe acute respiratory syndrome coronavirus 2
UI	uncertainty interval
WHO	World Health Organization

Section 2: GATHER compliance

This study complies with GATHER recommendations.[1] We have documented the steps in our analytical procedures and detailed the data sources used. See table S1 for the GATHER checklist. The GATHER recommendations can be found on the GATHER website.

Table S1. GATHER checklist

Item #	Checklist item	Reported on page #
**Objectives and funding**		
**1**	Define the indicator(s), populations (including age, sex, and geographic entities), and time period(s) for which estimates were made.	Main text: "overview" section in the methods<br>Appendix 1: section 3
**2**	List the funding sources for the work.	Main text: "funding" section of the summary, "role of the funding source" section of the methods, and "acknowledgments" section (to be added after resubmission)
**Data Inputs**		
For all data inputs from multiple sources that are synthesized as part of the study:		
**3**	Describe how the data were identified and how the data were accessed.	Main text: "data inputs and corrections" section in the methods<br>Appendix 1: section 4
**4**	Specify the inclusion and exclusion criteria. Identify all ad-hoc exclusions.	Main text: "data inputs and corrections" section in the methods<br>Appendix 1: section 4
**5**	Provide information on all included data sources and their main characteristics. For each data source used, report reference information or contact name/institution, population represented, data collection method, year(s) of data collection, sex and age range, diagnostic criteria or measurement method, and sample size, as relevant.	Appendix 2;<br>http://ghdx.healthdata.org/record/ihme-data/covid_19_cumulative_infections
**6**	Identify and describe any categories of input data that have potentially important biases (e.g., based on characteristics listed in item 5).	Main text: limitations section in the discussion
For data inputs that contribute to the analysis but were not synthesized as part of the study:		
**7**	Describe and give sources for any other data inputs.	N/A
For all data inputs:		
**8**	Provide all data inputs in a file format from which data can be efficiently extracted (e.g., a spreadsheet rather than a PDF), including all relevant meta-data listed in item 5. For any data inputs that cannot be shared because of ethical or legal reasons, such as third-party ownership, provide a contact name or the name of the institution that retains the right to the data.	Data inputs available at:<br>http://ghdx.healthdata.org/record/ihme-data/covid_19_cumulative_infections;<br>Institution names for all data inputs by location given in appendix 2.
**Data analysis**		

**9**	Provide a conceptual overview of the data analysis method. A diagram may be helpful.	Main text: "overview" section of the methods
**10**	Provide a detailed description of all steps of the analysis, including mathematical formulae. This description should cover, as relevant, data cleaning, data pre-processing, data adjustments and weighting of data sources, and mathematical or statistical model(s).	Main text: short description of all steps of the analysis given in the methods section<br>Appendix 1: detailed description of all steps of the analysis + mathematical formulae given in sections 5–9
**11**	Describe how candidate models were evaluated and how the final model(s) were selected.	Main text: consideration of different covariates in the methods section
**12**	Provide the results of an evaluation of model performance, if done, as well as the results of any relevant sensitivity analysis.	Appendix 1: sections 9, 10
**13**	Describe methods for calculating uncertainty of the estimates. State which sources of uncertainty were, and were not, accounted for in the uncertainty analysis.	Main text: "ensemble framework" and "robust estimate of daily infections" sections of the methods<br>Appendix 1: sections 7, 9
**14**	State how analytic or statistical source code used to generate estimates can be accessed.	All code used in the analysis can be found online (https://github.com/ihmeuw/covid-historical-model; https://github.com/ihmeuw/covid-model-infections).
**Results and Discussion**		
**15**	Provide published estimates in a file format from which data can be efficiently extracted.	Published estimates in excel format available at http://ghdx.healthdata.org/record/ihme-data/covid_19_cumulative_infections
**16**	Report a quantitative measure of the uncertainty of the estimates (e.g. uncertainty intervals).	Uncertainty intervals are reported alongside all estimates in the abstract and main results sections in the main text and all results in appendix 1
**17**	Interpret results in light of existing evidence. If updating a previous set of estimates, describe the reasons for changes in estimates.	Main text: "interpretation" section of the summary and discussion section
**18**	Discuss limitations of the estimates. Include a discussion of any modelling assumptions or data limitations that affect interpretation of the estimates.	Main text: limitations section in the discussion

Section 3: Locations, populations, and time period of analysis

Daily infections, cumulative infections, proportion of the population infected one or more times, and $R_{effective}$ were modelled for 190 countries and territories, including subnational analyses for 10 countries and territories, aggregated into 21 regions, seven super-regions,[2] and globally. All estimates are for all ages and both males and females combined. The time period of analysis was from the start of the coronavirus disease 2019 (COVID-19) pandemic through November 14, 2021.

Section 4: Data inputs

Section 4.1: Reported cases data

Data on reported cases primarily came from Johns Hopkins University,[3] supplemented by location-specific datasets extracted either directly from ministries of health, departments of public health, or other third parties. Sources are outlined in appendix 2 (section 4, table S4). Cases were defined, depending on the local context, as either an individual who has received a positive test result, whether RT-PCR, antigen, or antibody (regardless of symptoms status) or an individual who has symptoms consistent with a clinical definition of COVID-19. Adjustments to the time series were periodically required, either to account for interruptions in daily reporting due to, for instance, major public holidays, or more systematic issues, such as reporting backlogs of cases accumulated in laboratory processing, or adjustments due to changes in case definitions. A catalogue of these corrections is available through the associated GHDx record (http://ghdx.healthdata.org/record/ihme-data/covid_19_cumulative_infections).

Section 4.2: Hospital admissions data

Data on reported daily admissions, or cumulative hospitalisations, was typically sourced from ministries of health, or multi-jurisdiction agencies such as the US Department of Health and Human Services (HHS), or the European Centres for Disease Control. Sources are outlined in appendix 2 (section 1, table S1). Adjustments to the time series

were periodically required, either to account for interruptions in daily reporting due to, for instance, major public holidays, or more systematic issues, such as changes in COVID case definitions. A catalogue of these corrections is available through the associated GHDx record.

For time series that were incomplete for the entirety of the pandemic, such as the US HHS admissions dataset, we imputed the missing time-series portions by first running separate linear regressions for each location with missingness. The dependent variable was admissions, and the independent variable was infections derived from both daily deaths divided by the infection–fatality ratio (IFR)[4] and from cases divided by the infection–detection ratio (IDR) for the period in time that overlapped with the admissions data. We then used the coefficient from this model, a naïve infection–hospitalisation ratio (IHR) estimate, to predict out of sample for the period that was missing admissions, using the average of the two separately derived infections estimates. To avoid any disjoints at the day of transition from imputed data to the observed, we gradually transitioned from the former to the latter over the tail period of the imputation. We did this by determining the ratio of the average observed admissions over first week of data to the predicted admissions for that week, linearly interpolated from a ratio of 1 at 60 days before observed to the calculated residual ratio at the first day of observed, and multiplied the imputation model predictions by that ratio during that period. We then included the imputed admissions along with the observed in our hospitalisations database.

Section 4.3: Reported deaths data

Data on reported daily deaths primarily came from Johns Hopkins University,[3] supplemented by location-specific datasets extracted either directly from ministries of health, departments of public health, or other third parties. Sources are outlined in appendix 2 (section 2, table S2). Adjustments to the time series were periodically required, either to account for interruptions in daily reporting due to, for instance, major public holidays, or more systematic issues, such as reporting backlogs of deaths accumulated in vital registration system processing, or adjustments due to changes in case definitions and reconciliation of death certificates. A catalog of these corrections is available through the associated GHDx record.

Section 4.4: Seroprevalence data

Data on serosurveys reporting antibody positivity were collated on an ongoing basis. Two key data types were tracked—ongoing serological surveys conducted by governmental organisations and released periodically, and publications of antibody surveys published in preprint servers and traditional journals. For the latter, we leveraged existing published reviews[5,6] and cross-referenced the Serotracker database.[7] Sources used in this study and used as location-representative studies are outlined in appendix 2 (section 3, table S3). Data that were deemed not to be representative of the general population in the most-detailed geographical location in our modelling hierarchy covering the study site were excluded. Additionally, we excluded studies that reported less than 0.03 seroprevalence, as we found that measurements taken with little signal in the population resulted in empirical estimates of IFR not generalisable across locations, or even within location after broader exposure of the population.

Section 5: Seroprevalence survey adjustments

Section 5.1: Adjusting for vaccinations

Methods for estimating vaccination rates are described by the COVID-19 Forecasting Team.[8] Seroprevalence studies that use anti-spike tests have been shown to identify the vast majority of individuals tested who have received a vaccine.[9] In order to prevent this from influencing our estimates of cumulative infections, we had to determine the proportion of the sample that is likely to have been vaccinated but not infected. The formula for this adjustment is:

$$p_{true} = 1 - \frac{1 - p_{obs}}{1 - v \times 0.9}$$

where true seroprevalence, p_{true} is based on observed seroprevalence, p_{obs} assuming 90% of vaccinated individuals, v would test positive.[9] This is applied only to data based on anti-spike assays, unless those data indicate they have excluded vaccinated people from their survey population.

Section 5.2: Adjusting for reinfection from escape variants

Methods for estimating variant prevalence are described by the COVID-19 Forecasting Team.[8] In settings with escape variants present, seroprevalence surveys provide an estimate of the cumulative number of individuals with one or more infections. To compute the IFR, IHR, and IDR, we needed an estimate of cumulative infections, including reinfections. We estimated the number of cumulative infections from seroprevalence surveys, based on the prevalence of escape variants (Beta, Gamma, and Delta) and a level of cross-variant immunity between the escape variants and ancestral variants or other variants that do not show immune escape, such as Alpha (more details can be found in section 9.5). For this stage of our analysis, we approximated the time pattern of past infection using deaths divided by a preliminary estimate of the IFR described subsequently—we later used the seroprevalence with all corrections applied to re-estimate the IFR. The formula for the correction for escape variant prevalence is:

$$I_t^a = \frac{\sum_{d=1}^{t} i_d^o (1 - p_d^e)}{population}$$

$$U_t = I_t^a (1 - c)$$

$$I_t^{a,e} = \frac{\sum_{d=1}^{t} U_d i_d^o p_d^e}{population}$$

$$S_t = \frac{I_t^o}{I_t^o - I_t^{a,e}}$$

where cumulative ancestral-type infections at time t, I_t^a, is a function of daily observed infections, i_d^o, and daily escape variant prevalence, p_d^e; unprotected population fraction at time t, U_t, is the percentage of individuals exposed to ancestral-strain COVID not protected by cross-variant immunity, c; and ancestral-type infections re-infected with escape-variant COVID at time t, $I_t^{a,e}$, is then the product of unprotected exposed, observed infections, and escape variant prevalence. The adjustment scalar at time t, S_t, was then applied to seroprevalence data to account for repeat infections.

Section 5.3: Adjusting for seroreversion

Published studies[10–13] following cohorts of patients with positive viral tests show declining antibody test sensitivity as a function of time since infection. They have shown that different commercial tests have different rates of declining sensitivity, which may be related to the isotype or antigen target. To correct each reported seroprevalence survey for underreporting due to sensitivity, we used information on the specific test used in each survey, the pattern of declining sensitivity over time, and information on the time pattern of infections. For studies that use assays for which we do not have data on sensitivity decay, we used the average sensitivity curve among the assays we did have after matching on antigen target and isotype.

As with the correction for multiple infections, we used an initial approximation of infections in the form of deaths divided by a preliminary IFR estimated based on seroprevalence without accounting for sensitivity decay. Independently for each seroprevalence observation, we determined how many past infections would have tested positive based on the number of days between exposure and the midpoint of the serology study dates, determined directly by the sensitivity curve matched to the data based on antibody test (more detail in section 9.3). We then determine a seroreversion factor and adjust the seroprevalence observation as detailed below:

$$seroreversion\ factor = \frac{population - \sum_{d=0}^{t} infections_d}{population - \sum_{d=0}^{t} infections_d * sensitivity}$$

$$seroprevalence_{adj} = 1 - (1 - \ seroprevalence_{reported}) * \ seroreversion\ factor$$

Seroprevalence observations before (square) and after (circle) adjustment for vaccination and seroreversion are represented in the cumulative infected plot for each location with serological data in appendix 3.

Section 6: Modelling IDR, IHR, and IFR

Section 6.1: Indirect age-standardisation

We used estimates of the global age patterns of the IFR, and seroprevalence described by COVID-19 Forecasting Team.[4] We directly adapted those methods directly to estimate the age pattern of IHR, using hospitalisations in the place of deaths, based on 703 surveys and 2812 age-specific observations. These estimates allow us to control for the effect of age structure in the IHR and IFR models. The formula for the age-standardising scaling factor for either ratio can be represented as:

$$r^g = \sum_{a=0}^{125} \frac{r_a^g sp_a^g pop_a^g}{sp_a^g pop_a^g}$$

$$r^l = \sum_{a=0}^{125} \frac{r_a^g sp_a^g pop_a^l}{sp_a^g pop_a^l}$$

$$s^l = \frac{r^g}{r^l}$$

where the global all-age ratio, r^g, given global age-specific ratio, r_a^g, and global age-specific seroprevalence, sp_a^g, is a function of the global population age structure, pop_a^g; and the location-specific all-age ratio, r^l, is based on local population age structure, pop_a^l, given the same ratio and seroprevalence age pattern. The scaling factor, s^l, is the ratio of the global to location-specific all-age ratio s. The empirical estimates of IHR and IFR were multiplied by this value prior to modelling to implement the indirect age-standardisation, and the predictions from the models were then divided by this value to re-incorporate local age effects.

Section 6.2: Bayesian cascading regression framework

Models for IFR, IHR, and IDR were fit using MRTool, an open-source Bayesian meta-regression library developed at IHME.[14,15] We implemented a "cascading" framework wherein after a global model is fit using all available data, subsequent models are fit using only data pertaining to subsets of a geographical hierarchy, with levels for super-region, region, country, and subnational (where possible). We used an adapted version of the Global Burden of Diseases, Injuries, and Risk Factors Study (GBD) location hierarchy in this algorithm. In each of these models, the mean and standard deviation of the coefficients estimated in the "parent" location model were passed on to "child" location models as Gaussian priors. So, the first step would be to fit a model with all available data. Then, for example, a model for the high-income super-region is fit using data from all locations within that super-region and is also informed by the data from other super-regions through the priors that are derived from the global model coefficients. Similarly, a model for western Europe uses data directly from locations within that region and is also informed by the high-income model through the priors. Taking this a step further down the "cascade," the model for the United Kingdom uses only data associated with the four nations and is also informed by the western European parent model through the priors. Lastly, a model for England would be run using only local data and would be informed by priors from the UK model. Locations without seroprevalence data use the parameters estimated from the model of the nearest parent location paired with local covariate values for prediction.

For the pair of each level and covariate, we specified a hyperparameter, λ, which is a multiplier on the standard deviation of the prior. This enabled us to quantify a degree of confidence for each covariate at each level of the cascade. Certain parameters can more plausibly deviate from a parent model fit than others, which may be difficult to accurately capture in data-sparse sub-models. It also acts as an important safeguard against overinterpreting super-region or region-level variation that is used to predict out-of-sample, while allowing location-specific model fits more freedom.

Section 6.3: Modelling IDR

We estimated IDR using the formula:

$$logit(IDR) = \beta_o + \beta_1 \log(testing\ capacity\ rate) + \sum_{i=2}^{N} \beta_i ensemble\ covariate$$

where the testing capacity rate is defined as the maximum observed testing rate at a given point in time. Using uniform prior functionality of MRTool, we constrain β_1 to be positive. The IDR is a cumulative measure, while testing capacity is specific to a point in time—to reconcile this, we transformed the testing capacity variable to be the infection-weighted average of daily testing capacity rate up to the date of the survey data. The estimate of infections used for this transformation was deaths divided by our final estimate of IFR. To make predictions of daily IDR, this specification allowed us to simply apply the estimated parameters to the log daily testing capacity rate. Details on the estimation of testing rates by location over time are described elsewhere by the COVID-19 Forecasting Team.[8]. The process for determining ensemble covariates is described in Section 9.6. Table S2 shows the λ values used for each hierarchy level and covariate in the IDR model.

Table S2: IDR model lambdas

Hierarchy level	Intercept	Testing capacity per capita	Ensemble covariate
Super-region	3	3	3
Region	3	3	3
Country	100	100	100
Admin1	100	100	100
Admin2	100	100	100

Early on in the pandemic when testing rates were low, severely ill patients would have gone to hospital and many would have been diagnosed. This more targeted testing can result in an underestimate of the IDR (and thus overestimate of infections) during this period—to combat this, we set a location-specific minimum value of the IDR. For each location, we tested values of 0.01%, 0.1%, and each percent from 1% to 10%, and selected the floor that minimised root mean square error in resultant cumulative infections (ie, the cumulative sum of cases divided by the bounded IDR estimate) with respect to seroprevalence data. Locations without seroprevalence data inherited the floor of the nearest parent location with other child locations that had data.

Section 6.4: Modelling IHR

We estimated IHR using the formula:

$$logit(age\ standardised\ IHR) = \beta_0 + \sum_{i=1}^{N} \beta_i ensemble\ covariate$$

using the process for applying and backing out of age-standardisation described above. The process for determining ensemble covariates is described in Section 9.6. Table S3 shows the λ values used for each hierarchy level and covariate in the IHR model.

Table S3: IHR model lambdas

Hierarchy level	Intercept	Ensemble covariates
Super-region	3	3
Region	3	3
Country	100	100
Admin1	100	100
Admin2	100	100

Section 6.5: Modelling IFR

We estimated IFR using the formula:

$$logit(age\ standardised\ IFR) = \beta_o + \sum_{i=1}^{2} \beta_i B_{i-1}(time) + \sum_{i=2}^{N} \beta_i ensemble\ covariate$$

where the spline on time is linear, with one knot. We constrained the slope in the period before the knot to be negative, and the slope in the period following the knot to be 0. Additionally, we included a Gaussian prior of N(–0.002, 0.001^2) on slope of the declining portion of the time period in the global stage of the cascade, based on analysis of patient-level hospital data in the USA,[16] and is constrained to be no less than five times the mean of that slope for any location. The process for determining knot placement and ensemble covariates is described in Section 9.6.

Table S3 shows the λ values used for each hierarchy level and covariate in the IFR model.

Table S3: IFR model lambdas

Hierarchy level	Intercept	Time	Ensemble covariates
Super-region	3	2	3
Region	3	2	3
Country	100	10	100
Admin1	100	10	100
Admin2	100	10	100

Section 6.6: Effect of vaccinations and variants on IHR and IFR

Due to the marked age patterns of IHR and IFR, differential vaccination rates in older age groups have a significant effect on all-age ratios. Methods described by the COVID-19 Forecasting Team[8] produced estimates of vaccination rates across locations over time, stratified by adults under 65 and 65+. We split IHR and IFR into those age groups by first calculating relative ratios under 65 and 65+ using their respective global age pattern models paired with location- and age-specific populations. To do this we divided the population-weighted average of the age-specific ratios under 65 by the population-weighted average of all ages, and then performed the same procedure using ages 65 and above. We multiplied the location- and time-specific IHR and IFR estimated by the all-age models described previously by these age group relative ratios to get location-, time-, and age-group-specific ratios. Note that by using population-weighting, we assumed a uniform age pattern of infections in this stage – this was done to maintain consistency with assumptions around transmission intensity described by the COVID-19 Forecasting Team.[8]

Next, we accounted for vaccination rates by age group across locations and over time. We used estimates of the proportion of the population effectively vaccinated, ie, with complete immunity, as well as the proportion who remain susceptible to infection but are protected from severe disease. Those effectively vaccinated and protected from escape variants (Beta, Gamma, Delta) were separately counted from ancestral and Alpha strains. Details on the estimation of these quantities, as well as the prevalence of variants, are described by the COVID-19 Forecasting Team.[8] We used the formula:

$$r_{a,v} = r_a \frac{1 - (e_{a,v} + p_{a,v})}{1 - e_{a,v}}$$

where the ratio for a given age group and variant grouping (ancestral and Alpha or Beta, Gamma, and Delta), $r_{a,v}$, is equal to the original age group ratio, r_a, accounting for age- and variant-group-specific proportions of the population effectively vaccinated, $e_{a,v}$, or protected, $p_{a,v}$. We then recombined the age groups based on the populations and effective vaccination rates:

$$r_v = r_{<65,v} pop_{<65}\left(1 - e_{<65,v}\right) + r_{65+,v} pop_{65+}(1 - e_{65+,v})$$

where the all-age ratio for either variant group, r_v, is equal to the sum of the under 65 and 65+ ratios, $r_{<65,v}$ and $r_{65+,v}$, respectively, weighed by the proportion of the age group populations that are not effectively vaccinated for the given variant group. The final IHR and IFR are defined as:

$$r = r_{non-escape}p_{ancestral} + r_{non-escape}p_A \times srr + r_{escape}p_{B,G,D} \times srr$$

where proportion of infections with ancestral variant, $p_{ancestral}$, and Alpha variant, p_A, would have an IHR or IFR determined by the preceding steps for non-escape variant group, $r_{non-escape}$, while Beta, Gamma, and Delta variant infections, $p_{B,G,D}$, would have the escape variant IHR or IFR, r_{escape}. In order to account for increased likelihood of hospitalisation and death for non-ancestral variants (Alpha, Beta, Gamma, and Delta), we then scaled the IHR and IFR in the presence of non-ancestral variants by the severity risk ratio srr. Details regarding the severity risk ratio can be found in section 9.5.

Because IHR and IFR observations are cumulative, any data associated with a seroprevalence study done after the introduction of vaccines or the invasion of non-ancestral variants in a given location would be impacted by these variables. Our aim was to estimate curves devoid of these effects, and then to deterministically implement them by location-day using the methods described above. To do this, we must first remove the marginal effect of vaccines and variants from our model observations. For this we again used the results of the naïve IFR model, but then performed the operations described previously in this section to produce estimates that account for vaccines and variants. For each seroprevalence point at time T in a given location, we apply the adjustment:

$$s_T = \frac{\sum_{t=0}^{T} \frac{ifr_t^u}{ifr_t^a} infections_t}{\sum_{t=0}^{T} infections_t}$$

$$r_T^a = r_T^o s_T$$

where ratio model observation (including relevant seroprevalence survey adjustments described in Section 5) at time T, r_T^o, is revised scalar s_T, which is the infection-weighted average of the ratio of unadjusted naïve IFR, ifr_t^u, to adjusted naïve IFR, ifr_t^a. The adjusted ratio data, r_T^a, is then used in a subsequent final model.

Section 7: Robust estimate of daily infections

Three estimates of daily infections were generated by dividing reported cases by the daily estimate of the IDR, dividing hospitalisations (where available) by the daily estimate of the IHR, and dividing deaths by the daily estimate of the IFR. Where hospitalisations are not available, the robust estimate of daily infections would be based solely on cases/IDR and deaths/IFR. Before this transformation, we smooth the case, hospitalisation, and death time series using a cubic spline with knots every 7 days. We then check that each of these infections estimates results in plausible relationships with respect to the originally reported units; that is, we enforce that the ratio of cases to each source of infections is less than 0.8, that the ratio of hospitalisations to each source of infections is less than 0.65, and that the ratio of deaths to each source of infections is less than 0.65. The approach we used to combine the series into a single composite estimate of daily infections was designed to deal with the compositional bias problem caused by varying temporal coverage in reporting among cases, hospitalisations, and deaths, and due to different lags in the time between infection and those events. The unit of the analysis in the initial stage of synthesising these measures was the first difference in log daily values. We incorporated these data into a random knots spline regression using MRTool wherein we provided a number of knots and a number of unique knot combinations to an algorithm that ran a model with each combination and made a weighted composite estimate from the sub-models based on in-sample performance. We specified one knot per 28 days of data and tested ten random knot combinations of a quadratic spline. We then converted the estimate into log daily values by taking the cumulative sum and found the initial value of the composite time series by fitting a model to the average log daily residual of the three original curves with respect to the composite.

We then conducted a fit-refit procedure that aimed to capture variability of the time trend suggested by noise in the reported data. We first converted the observed (unsmoothed) daily cases, hospitalisations, and deaths into unsmoothed infections by dividing them by the estimated time series of IDR, IHR, and IFR, respectively. We then used the log of these values to compute the residuals with respect to the log mean infections curve we estimated in

the previous step and calculated the robust standard deviation using a rolling 120-day window in order to be sensitive to changes in reporting practices. We then used the mean estimate of infections and the robust standard deviation to resample daily infections and refit the infections curve to these data using the same specifications used to estimate the original mean curve.

For six countries—North Korea, Tajikistan, Turkmenistan, Nicaragua, Venezuela, and Tanzania—case, hospitalisation, and death data are either completely missing or deemed to be so unreliable that we exclude them from the analysis and instead report the average daily infection rate among other countries in their respective regions.

Section 8: Cumulative infections and the cumulative proportion of the population infected at least once

We used the adjustment scalar described in section 5.2 to determine the proportion of the population infected at least once at a given point in time in a given location by dividing the cumulative sum of estimated infections by that scalar.

Section 9: Ensemble model framework

Section 9.1: Overview

To incorporate uncertainty in both data and assumptions that feed into our model, we developed an ensemble model framework wherein the methods described in previous sections were performed independently using 100 different variations of relevant data, model composition, and parameterisation. The general structure of that system is provided here.

Section 9.2: Seroprevalence data

For all but two seroprevalence observation, a lower and upper bound was reported in addition to the mean – from these we derive the standard error, and for each point sample 100 times from a logit-normal distribution with that mean and standard error. For the two studies that did not report uncertainty intervals, we used the mean and sample size to calculate the binomial standard error. To preserve correlation across multiple measurements within a given location while keeping inter-location samples independent, we sorted the samples across different observations within location to match the sort order of one arbitrarily chosen observation in that location. Next, we produced one bootstrapped sample of each of these 100 representations of our seroprevalence database. The resultant 100 datasets were each used as inputs to separate model pipelines.

Section 9.3: Sensitivity decay functions

Studies from which we extracted commercial assay sensitivity reported mean sensitivity by assay with uncertainty, either as continuous functions or at various points over time, indexed in time from either exposure or seroconversion – those that reported from exposure have been re-indexed to start at seroconversion. From these data we then produced 100 correlated samples of sensitivity and interpolated/extrapolated each of those 100 samples over time by fitting a smoothing spline over the logit transformed data with a number of evenly spaced knots that was equal to two fewer than the number of reported time points (minimum two knots). For those that reported sensitivity as continuous, ten knots were used. Where multiple studies reported sensitivity for a given assay, we separately fit these smooth curves by assay and study, then took the average of the curves. For studies that already applied a manufacturer correction for baseline sensitivity, we shift the curves up to equal 100% sensitivity at seroconversion. Figure S1 shows these curves we were able to extract from these studies, shown here extrapolated out to two years. The lighter blue lines are the 100 samples, while the dark line is the mean.

Figure S1: Sensitivity decay functions for eight commercial serology assays

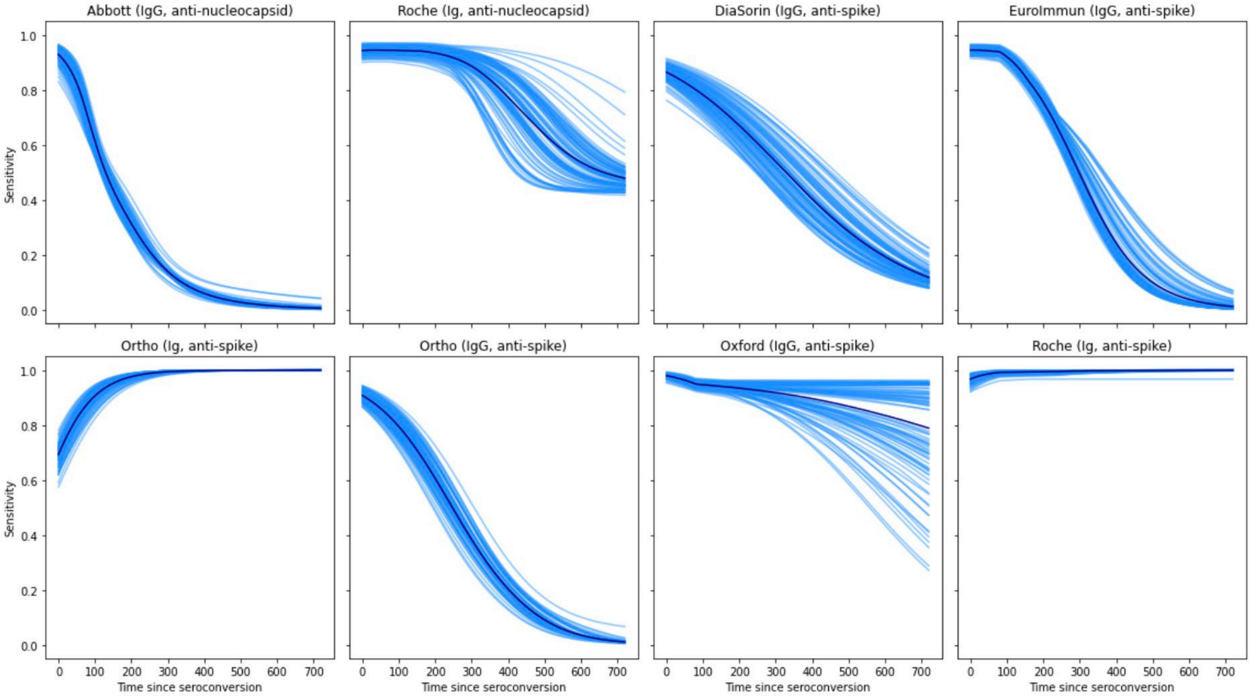

Section 9.4: Total COVID-19 scalars

While timely reporting of deaths due to COVID-19 is available in most countries, under-reporting is prevalent even for countries with functional and complete vital registration system, particularly severe during the initial stage of the epidemic when testing is low. In our analysis, we define total COVID-19 deaths as all deaths where the deceased were actively infected by SARS-CoV-2 at the time of the death. To accurately capture the progression of the epidemic, we have applied a scalar of reported to total COVID-19 death counts for all locations included in our analysis. The total COVID-19 scalar is generated based on a separate model developed to estimate excess mortality due to COVID-19 by following the three steps described below.

1. COVID-19 Excess Mortality Collaborators have developed an ensemble modelling framework to estimate the excess mortality for national and subnational locations where all-cause mortality data are reported by week or month during the COVID-19 epidemic. In all, they find such data for 74 countries and an additional 266 subnational locations from a subset of the 74 countries. To capture both the seasonality and secular trend in mortality over time, six different models were included in the ensemble model they used. Details of these models are described here:
   http://www.healthdata.org/sites/default/files/files/Projects/COVID/2021/Estimation-of-excess-mortality-due-to-COVID.pdf
   The ensemble model provides excess mortality estimates for each location with reported all-cause mortality data after accounting for late registration, which varies by location as suggested by the analysis done by the COVID-19 Excess Mortality Collaborators. In addition to the excess mortality directly estimated using reported all-cause mortality data, they have also included excess mortality rate estimates provided by the Medical Research Council of South Africa at both the national and provincial levels. While detailed temporal data on all-cause mortality data from India are not available, they were able to use reported deaths from the Civil Registration System for nine states for selected months in both 2020 and 2021 to estimate excess mortality for those periods by comparing to the average numbers of deaths from the same states for year 2018 and 2019, after accounting for under-registration of deaths by state.

2. Using the empirical excess mortality estimates described in the above step, they developed a new statistical model to predict excess mortality rate for all locations, national and subnational, included in our analysis

for the period of January 1, 2020, to November 14, 2021. Based on the meta-analysis conducted by the US Centers for Disease Control and Prevention, they evaluated all relevant and available covariates both pertaining to the COVID-19 epidemic and general population health metrics. To build a parsimonious model that includes covariates with sensible direction of effects on excess mortality rate, they have run Least Absolute Shrinkage and Selection Operator (LASSO) regression to identify the following covariates included in their final model:

a. Cumulative seroprevalence with lag
b. Mobility with lag
c. Infection–detection ratio with lag
d. Reported COVID-19 crude death rate
e. Crude death rate for year 2019
f. Universal Healthcare Coverage
g. Prevalence of smoking for year 2019
h. Crude death rate due to HIV/AIDS for year 2019
i. Inpatient admission rate for year 2019
j. Quality of death registration system
k. Average latitude
l. Proportion of population over age 75 in 2019
m. Healthcare Access and Quality Index for year 2019
n. Diabetes death rate in 2019
o. Cardiovascular disease death rate in 2019

After the model selection using mean level input data, they have run the estimation process 100 times based on draw-level excess mortality and covariates to account for uncertainty in all variables used in their analysis. Residuals from the in-sample fitting step are used in predicting excess mortality rate by location such that prediction matches the input excess mortality rates when they are available for the locations. For the purpose of making predictions for locations where we do not have empirical estimates of excess mortality, average residuals were generated for each GBD region and super-region. Given the sparse data, both in terms of time period and regions covered, from India, a country-level residual was used in prediction by averaging the state-level residuals from the nine states where we have data on excess mortality.

Using the estimated draw-level fixed effects and the residuals together with draw-level covariates, we predict 100 draws of excess mortality rate for all locations included in our analysis for the period of January 1, 2020, to December 31st, 2021.

3. The statistical model for the estimation of excess mortality rate described above enables us to approximate the fraction of excess mortality that can be directly attributable to COVID-19. Using this model, we can compute counterfactual excess mortality estimates where mobility is set to pre-pandemic level, and the IDR is set to the maximum observed level among all locations. By doing so, we correct excess mortality estimates for changes due to under-reporting from lack of testing and behavioural changes in care-seeking and social distancing. The ratio between these counterfactual excess mortality estimates and our estimates of excess mortality described in step 2 approximates the proportion of excess mortality for each location that can be attributed to total COVID during our estimation time period. However, in location and draw combinations where the ratio of reported COVID-19 deaths over estimated excess mortality is higher than the ratio based on our counterfactual analysis, reported COVID-19 deaths is used as the estimate of total COVID-19 deaths. Then, finally, for each location, the total COVID-19 estimates are used to generate the scalar for reported COVID-19 deaths.

Figure S2: Cumulative total COVID-19 death rate (per 100,000 population) by November 14, 2021

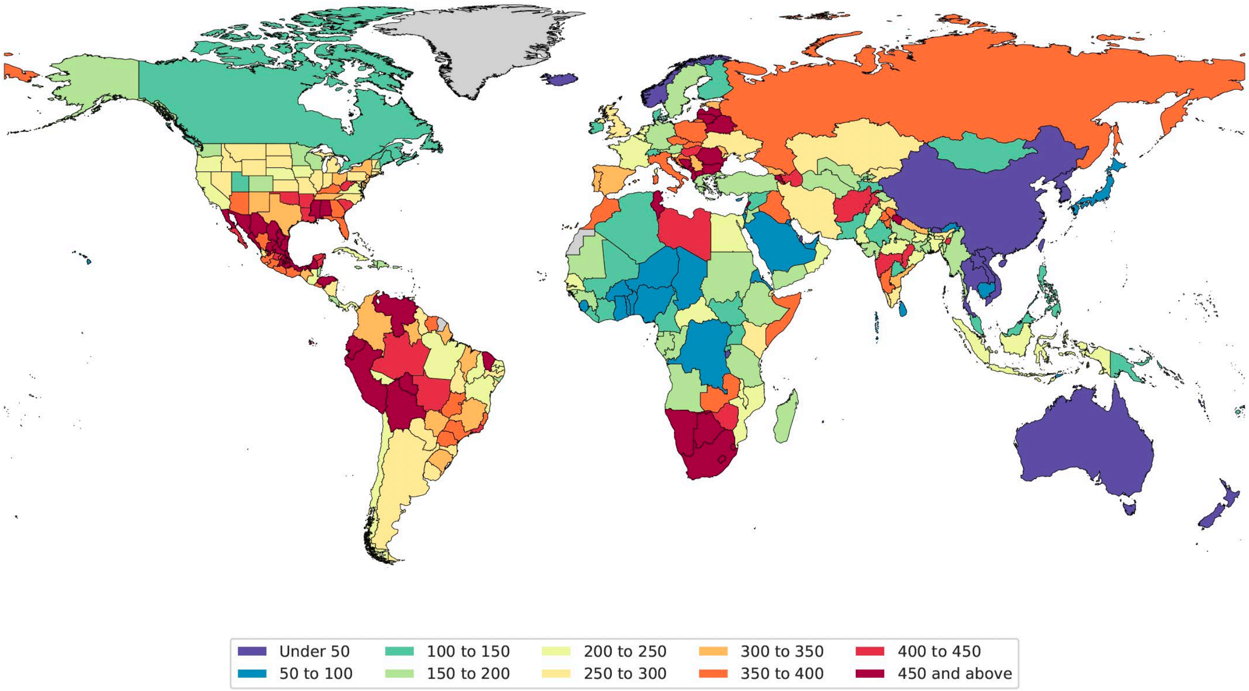

Section 9.5: Ratio model parameters

There are several parameters featured in our model pipeline that we vary by sub-model. First, we generate 100 samples of cross-variant immunity from the distribution $U[0.3, 0.7]$ – bounds set based on an empirical analysis using the SEIR model described by the COVID-19 Forecasting Team.[8] Second, we generate 100 samples of severity risk ratio for all non-ancestral variants relative to ancestral based on Challen et al.,[17] who reported an increased risk of mortality of 1.64 (95% CI: 1.32–2.04) among 54,906 matched pairs of patients infected with ancestral or Alpha; we applied this multiplier to both IHR and IFR. Third, we varied the duration from exposure to case detection and hospitalisation ($U[10, 13]$)), exposure to seroconversion ($U[14, 17]$), and hospitalisation to death ($U[12, 15]$)) for each sub-model.

Section 9.6: IDR, IHR, and IFR model

The 100 sets of seroprevalence data, sensitivity decay curves, total COVID-19 deaths, and model parameters were then combined and used in 100 independent model pipelines using methods described in Sections 5 through 8. For the ratio models described in Section 6, we further diversify our model pool by varying the covariates used in the IDR, IHR, and IFR.

We identified three suitable covariates with which to estimate the IDR (in addition to testing capacity rate, as described in Section 6.3): universal health care coverage (UHC), Healthcare Access and Quality (HAQ) Index, and proportion of the population age 65 years and older. Each of these was estimated as part of the GBD – details regarding their estimation have been published elsewhere. Briefly, UHC is a (0, 1) bound index of universal health coverage tracer interventions for prevention and treatment services; HAQ is (0, 1) bound index measuring health service delivery using causes of death amenable to personal health care. Each sub-model in the ensemble is randomly assigned either one of these three covariates or no additional covariates. Testing capacity rate was used in every sub-model.

For estimating IHR and IFR, our potential covariates include conditions identified by the CDC as having an increased risk of severe illness that are supported by meta-analysis or systematic review (https://www.cdc.gov/coronavirus/2019-ncov/science/science-briefs/underlying-evidence-table.html). We cross-referenced this list with a study that evaluated the risk of in-hospital death to select potential covariates,[16] and use

the odds ratios reported in that study to more broadly represent increased risk of worse outcomes by including them as Gaussian priors in our IFR and IHR models – these covariates and priors can be found in table S4. In addition, we also include HAQ Index and UHC in the covariate pool for these models (without priors, but with constraints forcing coefficients to be negative). As with HAQ Index and UHC, these values are all estimates of the GBD.

Table S4: Covid co-morbidity covariates and priors

Underlying medical condition (age-standardised prevalence in the population)	Prior	Number of sub-models
**Obesity**	$N(0.36, 0.0122)$	100
**Smoking**	$N(0.1, 0.0083)$	72
**Diabetes**	$N(0.1, 0.0024)$	52
**Cancer**	$N(0.22, 0.004)$	40
**Chronic obstructive pulmonary disease**	$N(0.07, 0.0032)$	50
**Cardiovascular disease**	$N(0.19, 0.0049)$	40
**Chronic kidney disease**	$N(0, 0.0048)$	50

We tested 383 possible combinations of these covariates (restricting UHC and HAQ Index from being present in the same model) in the global tier of our age-standardised IFR model (see Sections 6.1, 6.2, and 6.5 for more details) and selected the 100 combinations that were most predictive to use in our ensemble. We excluded data from the US and UK from this step in order to prevent the serial measurements across many subnational units in those countries from being overly influential in the selection of covariates. The number of sub-models that a given covariate is featured in can be found in table S2. UHC was included in 36 models, and HAQ Index was in 56.

Patient data from hospitals in the United States, Brazil, and Mexico suggest improvements in diagnosing and treating COVID-19 resulted in lower mortality risk relative to the earliest months of the pandemic. However, these variables are exogenous to our model, and cumulative population death and seroprevalence data can be insufficient to precisely identify the duration of that period of decline – especially when the data are not serially measured, something that occurs in only very few countries. To estimate a marginal time effect in the IFR in a manner that reflects uncertainty in the shape of said effect, we randomly assigned the placement of the spline knot described in Section 6.5 for each sub-model. The selection pool consisted of 10 possible inflection points – the first day of each month from June 2020 to March 2021.

Section 9.7: Estimating daily infections

When triangulating infections based on cases, hospitalisations, and deaths as described in Section 7, we observed that, for example, infections derived from cases and the IDR might suggest significantly different levels of past infection than deaths divided by the IFR – specifically in locations without any seroprevalence observations and where predictive covariates lacked concordance across the different ratio models after being applied to local data. By simply "splitting the difference" in these instances, we greatly under-estimate the variation among our component parts, even in this ensemble approach. To combat this, we have used an algorithm that randomly selects one of the input measures (cases, hospitalisations, and deaths) to preferentially weight in each sub-model when triangulating infections using MRTool by assigning a lower variance to the infections derived from those data. We also vary the degree of that weighting for each sample by drawing a variance from the distribution $U[0.1, 0.9]$, while data from the measures not selected in that sub-model retain a variance equal to 1. This yields a distribution of posterior infections estimates that more adequately reflects the heterogeneity of the observed data, assumptions, and model estimates by which it is informed.

Section 10: Sensitivity analyses

To explicitly demonstrate the effects of some of our model assumptions, we have run our analytic pipeline five additional times, removing the effect of one model assumption from each run. We included comparisons of our estimates of cumulative infection rate to these in figure S2, which graphs locations by super-regions showing the reference estimates on the x-axis and the results of the sensitivity analyses conducted for each column on the y-axis.

Figure S3: Sensitivity analyses

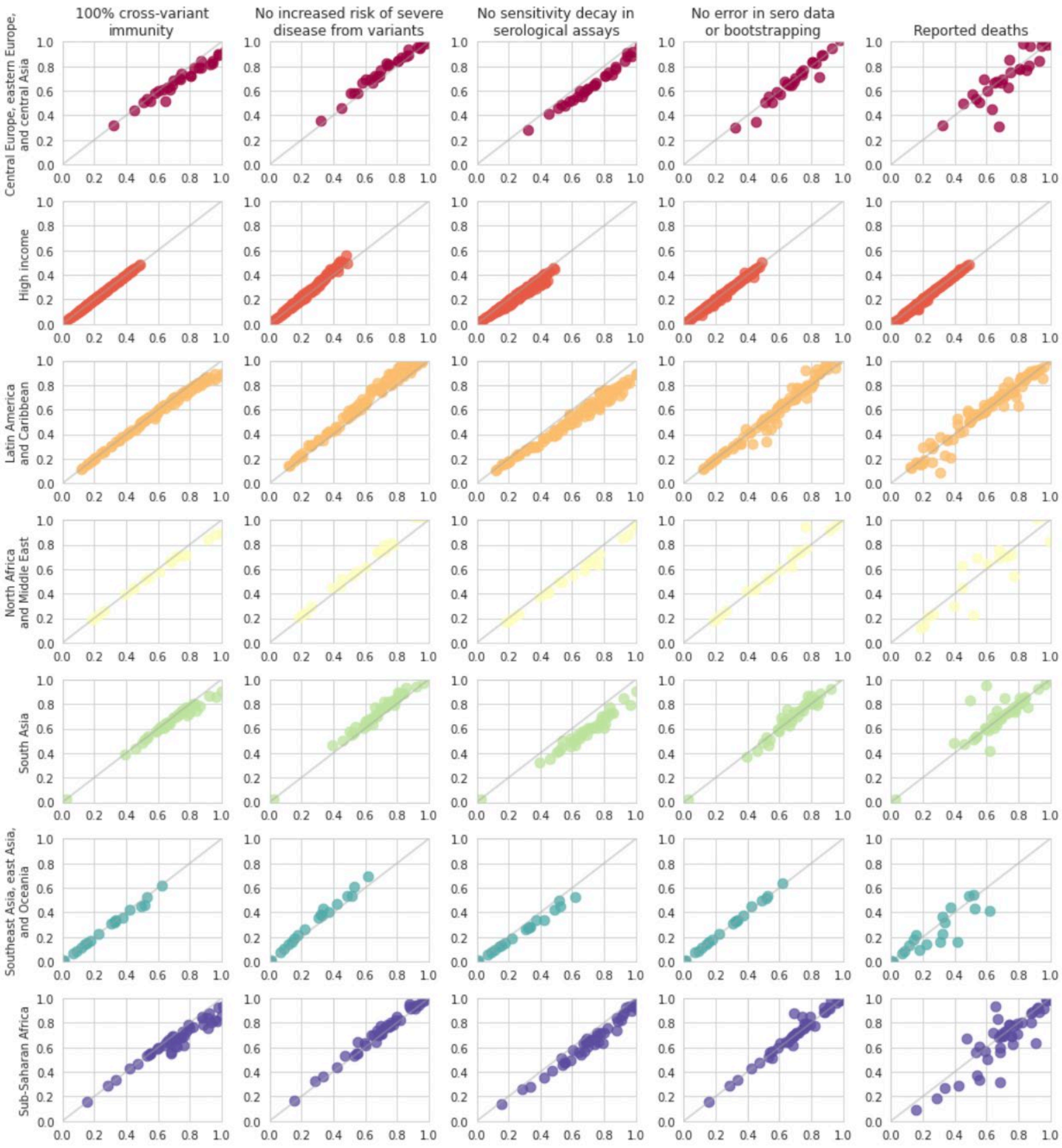

Section 11: References

1 Stevens GA, Alkema L, Black RE, *et al.* Guidelines for Accurate and Transparent Health Estimates Reporting: the GATHER statement. *The Lancet* 2016; **388**: e19–23.

2 Murray CJ, Ezzati M, Flaxman AD, *et al.* GBD 2010: design, definitions, and metrics. *Lancet* 2012; **380**: 2063–6.

3 Dong E, Du H, Gardner L. An interactive web-based dashboard to track COVID-19 in real time. *Lancet Infect Dis* 2020; **20**: 533–4.

4 COVID-19 Forecasting Team. Variation in the COVID-19 infection–fatality ratio by age, time, and geography during the pre-vaccine era: a systematic analysis. *Lancet* 2022; published online Feb 24.

5 Ioannidis, John P A. Infection fatality rate of COVID-19 inferred from seroprevalence data. *Bull World Health Organ* 2021; **99**: 19-33F.

6 Levin AT, Hanage WP, Owusu-Boaitey N, Cochran KB, Walsh SP, Meyerowitz-Katz G. Assessing the age specificity of infection fatality rates for COVID-19: systematic review, meta-analysis, and public policy implications. *Eur J Epidemiol* 2020; **35**: 1123–38.

7 Arora RK, Joseph A, Wyk JV, *et al.* SeroTracker: a global SARS-CoV-2 seroprevalence dashboard. *Lancet Infect Dis* 2021; **21**: e75–6.

8 COVID-19 Forecasting Team. Modelling global COVID-19 scenarios through March 2022. *Lancet* in review.

9 Wei J, Stoesser N, Matthews PC, *et al.* The impact of SARS-CoV-2 vaccines on antibody responses in the general population in the United Kingdom. *medRxiv* 2021; : 2021.04.22.21255911.

10 Muecksch F, Wise H, Batchelor B, *et al.* Longitudinal serological analysis and neutralizing antibody levels in coronavirus disease 2019 convalescent patients. *J Infect Dis* 2021; **223**: 389–98.

11 Peluso MJ, Takahashi S, Hakim J, *et al.* SARS-CoV-2 antibody magnitude and detectability are driven by disease severity, timing, and assay. *medRxiv* 2021; : 2021.03.03.21251639.

12 Perez-Saez J, Zaballa M-E, Yerly S, *et al.* Persistence and detection of anti-SARS-CoV-2 antibodies: immunoassay heterogeneity and implications for serosurveillance. *medRxiv* 2021; : 2021.03.16.21253710.

13 Lumley SF, Wei J, O'Donnell D, *et al.* The duration, dynamics, and determinants of severe acute respiratory syndrome coronavirus 2 (SARS-CoV-2) antibody responses in individual healthcare workers. *Clin Infect Dis Off Publ Infect Dis Soc Am* 2021; **73**: e699–709.

14 Rajeev Mittal. MRTool. IHME Math Sciences, 2021 https://github.com/ihmeuw-msca/mrtool (accessed June 30, 2021).

15 Zheng P, Barber R, Sorensen RJD, Murray CJL, Aravkin AY. Trimmed constrained mixed effects models: formulations and algorithms. *J Comput Graph Stat* 2021; **0**: 1–13.

16 Roth GA, Emmons-Bell S, Alger HM, *et al.* Trends in patient characteristics and COVID-19 in-hospital mortality in the United States during the COVID-19 pandemic. *JAMA Netw Open* 2021; **4**: e218828.

17 Challen R, Brooks-Pollock E, Read JM, Dyson L, Tsaneva-Atanasova K, Danon L. Risk of mortality in patients infected with SARS-CoV-2 variant of concern 202012/1: matched cohort study. *BMJ* 2021; **372**: n579.

Section 12: Author contributions

Managing the estimation or publications process

Ryan M Barber, Catherine Bisignano, Emmanuela Gakidou, Stephen S Lim, Simon I Hay, David M Pigott, Joanne O Amlag, Bethany M Huntley, and Christopher J L Murray.

Writing the first draft of the manuscript

Ryan M Barber, Catherine Bisignano, and Christopher J L Murray.

Primary responsibility for applying analytical methods to produce estimates

Ryan M Barber, Reed J D Sorensen, Austin Carter, and James K Collins.

Primary responsibility for seeking, cataloguing, extracting, or cleaning data; designing or coding figures and tables

Ryan M Barber, Reed J D Sorensen, David M Pigott, Bree L Bang-Jensen, Rebecca M Cogen, Emily Combs, Lucas Earl, Samuel B Ewald, Alize J Ferrari, Gaorui Guo, Monika Helak, Erin N Hulland, Alice Lazzar-Atwood, Kate E LeGrand, Akiaja Lindstrom, Ana M Mantilla Herrera, Ali H Mokdad, Mohsen Naghavi, Maja Pasovic, Damian Francesco Santomauro, Emma Elizabeth Spurlock, and Ruri Syailendrawati.

Providing data or critical feedback on data sources

Ryan M Barber, Reed J D Sorensen, David M Pigott, Cristiana Abbafati, Christopher Adolph, Bree L Bang-Jensen, Suman Chakrabarti, Haley Comfort, Xiaochen Dai, Megan Erickson, Abraham D Flaxman, Nancy Fullman, John R Giles, Gaorui Guo, Jiawei He, Monika Helak, Erin N Hulland, Kate E LeGrand, Rafael Lozano, Johan Månsson, Beatrice Magistro, Deborah Carvalho Malta, Ali H Mokdad, Lorenzo Monasta, Mohsen Naghavi, Shuhei Nomura, Latera Tesfaye Olana, Maja Pasovic, Spencer A Pease, Grace Reinke, Antonio Luiz P Ribeiro, Damian Francesco Santomauro, Ruri Syailendrawati, Anh Truc Vo, Theo Vos, Rebecca Walcott, Charles Shey Wiysonge, Nahom Alemseged Worku, Emmanuela Gakidou, and Christopher J L Murray.

Developing methods or computational machinery

Ryan M Barber, Reed J D Sorensen, Adrien Allorant, Aleksandr Y Aravkin, Austin Carter, Emma Castro, Suman Chakrabarti, James K Collins, Haley Comfort, Kimberly Cooperrider, Xiaochen Dai, Farah Daoud, Abraham D Flaxman, Joseph Jon Frostad, John R Giles, Jiawei He, Emily Linebarger, Ali H Mokdad, Mohsen Naghavi, Spencer A Pease, Robert C Reiner Jr, Aleksei Sholokhov, Kirsten E Wiens, and Peng Zheng, and Christopher J L Murray.

Providing critical feedback on methods or results

Ryan M Barber, Reed J D Sorensen, David M Pigott, Austin Carter, James K Collins, Cristiana Abbafati, Xiaochen Dai, Abraham D Flaxman, John R Giles, Deborah Carvalho Malta, Ali H Mokdad, Mohsen Naghavi, Christopher M Odell, Latera Tesfaye Olana, Samuel M Ostroff, Maja Pasovic, Robert C Reiner Jr, Antonio Luiz P Ribeiro, Emma Elizabeth Spurlock, Roman Topor-Madry, Theo Vos, Ally Walker, Charles Shey Wiysonge, and Nahom Alemseged Worku, Simon I Hay, Emmanuela Gakidou, Christopher J L Murray,.

Drafting the work or revising it critically for important intellectual content

Ryan M Barber, Reed J D Sorensen, David M Pigott, Catherine Bisignano, Cristiana Abbafati, Stephen S Lim, Deborah Carvalho Malta, Ali H Mokdad, Lorenzo Monasta, Mohsen Naghavi, Samuel M Ostroff, Maja Pasovic, Antonio Luiz P Ribeiro, Roman Topor-Madry, Theo Vos, Charles Shey Wiysonge, Simon I Hay, and Christopher J L Murray.

Managing the overall research enterprise

Emmanuela Gakidou, Stephen S Lim, Simon I Hay, David M Pigott, Joanne O Amlag, Bethany M Huntley, Rafael Lozano, Ali H Mokdad, Robert C Reiner Jr, and Christopher J L Murray.
