# Supplementary material for: Estimating global, regional, and national daily and cumulative infections with SARS-CoV-2 through Nov 14, 2021: a statistical analysis
Source: Lancet. 2022 Jun 25;399(10344):2351–80. doi: 10.1016/S0140-6736(22)00484-6 (PMC8993157; doi:10.1016/S0140-6736(22)00484-6)
Supplement: Supplementary appendix 2 [file mmc2.pdf]

THE LANCET

Supplementary appendix 2

This appendix formed part of the original submission and has been peer reviewed. We post it as supplied by the authors.

Supplement to: COVID-19 Cumulative Infection Collaborators. Estimating global, regional, and national daily and cumulative infections with SARS-CoV-2 through Nov 14, 2021: a statistical analysis. *Lancet* 2022; published online April 8. https://doi.org/10.1016/S0140-6736(22)00484-6.

Appendix 2: data sources for "Estimating global, regional, and national daily and cumulative infections with SARS-CoV-2 through Nov 14, 2021: a statistical analysis"

This appendix provides information on data sources for "Estimating global, regional, and national daily and cumulative infections with SARS-CoV-2 through Nov 14, 2021: a statistical analysis".

Table of Contents

Section 1: Admissions and cumulative hospitalisations sources 3
- Figure S1. Hospitalisation data coverage by location 3
- Table S1. Hospitalisation data sources by location 3

Section 2: Deaths sources 13
- Figure S2. Death data coverage by location 13

Section 3: Seroprevalence sources 29
- Figure S3. Seroprevalence data coverage by location 29

Section 4: Cases sources 44
- Figure S4. Case data coverage by location 44

Section 5: Testing sources 60
- Figure S5. Testing data coverage by location 60
- Table S5. Testing data sources by location 60

Section 6: Age-stratified hospitalisations sources 74
- Figure S6. Age-stratified hospitalisation data coverage by location 74
- Table S6. Age-stratified hospitalisation data sources by location 74

Section 1: Admissions and cumulative hospitalisations sources

Figure S1. Hospitalisation data coverage by location

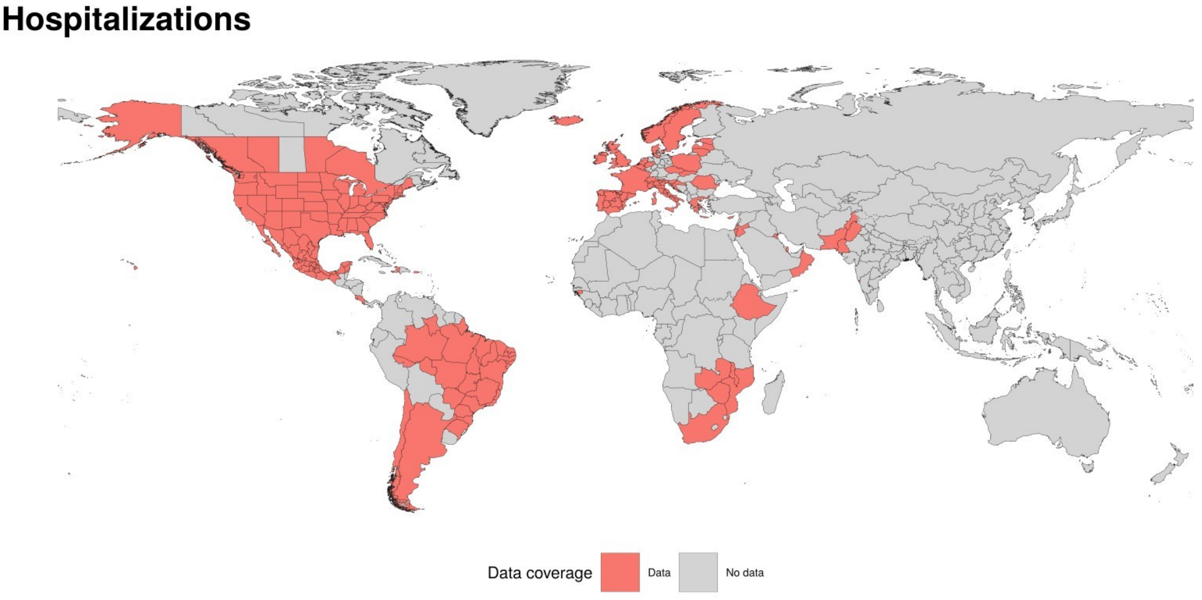

Table S1. Hospitalisation data sources by location

Location	Source
Andorra	Government of Andorra. Andorra COVID-19 Data. Andorra: Government of Andorra.
Argentina	Ministry of Health (Argentina). Argentina Ministry of Health COVID-19 Cases. Buenos Aires, Argentina: Ministry of Health (Argentina).
Belgium	Sciensano (Belgium). Belgium COVID-19 Confirmed Cases by Date, Age, Sex and Province.
Belgium	Sciensano (Belgium). Belgium COVID-19 Mortality by Date, Age, Sex, and Region 2020.
Brazil, Acre	Ministry of Health (Brazil). Brazil - Severe Acute Respiratory Syndrome Database - including data from COVID-19. Rio de Janeiro, Brazil: Ministry of Health (Brazil).
Brazil, Alagoas	Ministry of Health (Brazil). Brazil - Severe Acute Respiratory Syndrome Database - including data from COVID-19. Rio de Janeiro, Brazil: Ministry of Health (Brazil).
Brazil, Amapá	Ministry of Health (Brazil). Brazil - Severe Acute Respiratory Syndrome Database - including data from COVID-19. Rio de Janeiro, Brazil: Ministry of Health (Brazil).
Brazil, Amazonas	Ministry of Health (Brazil). Brazil - Severe Acute Respiratory Syndrome Database - including data from COVID-19. Rio de Janeiro, Brazil: Ministry of Health (Brazil).
Brazil, Bahia	Ministry of Health (Brazil). Brazil - Severe Acute Respiratory Syndrome Database - including data from COVID-19. Rio de Janeiro, Brazil: Ministry of Health (Brazil).
Brazil, Ceará	Ministry of Health (Brazil). Brazil - Severe Acute Respiratory Syndrome Database - including data from COVID-19. Rio de Janeiro, Brazil: Ministry of Health (Brazil).
Brazil, Distrito Federal	Ministry of Health (Brazil). Brazil - Severe Acute Respiratory Syndrome Database - including data from COVID-19. Rio de Janeiro, Brazil: Ministry of Health (Brazil).
Brazil, Espírito Santo	Ministry of Health (Brazil). Brazil - Severe Acute Respiratory Syndrome Database - including data from COVID-19. Rio de Janeiro, Brazil: Ministry of Health (Brazil).
Brazil, Goiás	Ministry of Health (Brazil). Brazil - Severe Acute Respiratory Syndrome Database - including data from COVID-19. Rio de Janeiro, Brazil: Ministry of Health (Brazil).
Brazil, Maranhão	Ministry of Health (Brazil). Brazil - Severe Acute Respiratory Syndrome Database - including data from COVID-19. Rio de Janeiro, Brazil: Ministry of Health (Brazil).
Brazil, Mato Grosso	Ministry of Health (Brazil). Brazil - Severe Acute Respiratory Syndrome Database - including data from COVID-19. Rio de Janeiro, Brazil: Ministry of Health (Brazil).
Brazil, Mato Grosso do Sul	Ministry of Health (Brazil). Brazil - Severe Acute Respiratory Syndrome Database - including data from COVID-19. Rio de Janeiro, Brazil: Ministry of Health (Brazil).

Brazil, Minas Gerais	Ministry of Health (Brazil). Brazil - Severe Acute Respiratory Syndrome Database - including data from COVID-19. Rio de Janeiro, Brazil: Ministry of Health (Brazil).
Brazil, Pará	Ministry of Health (Brazil). Brazil - Severe Acute Respiratory Syndrome Database - including data from COVID-19. Rio de Janeiro, Brazil: Ministry of Health (Brazil).
Brazil, Paraíba	Ministry of Health (Brazil). Brazil - Severe Acute Respiratory Syndrome Database - including data from COVID-19. Rio de Janeiro, Brazil: Ministry of Health (Brazil).
Brazil, Paraná	Ministry of Health (Brazil). Brazil - Severe Acute Respiratory Syndrome Database - including data from COVID-19. Rio de Janeiro, Brazil: Ministry of Health (Brazil).
Brazil, Pernambuco	Ministry of Health (Brazil). Brazil - Severe Acute Respiratory Syndrome Database - including data from COVID-19. Rio de Janeiro, Brazil: Ministry of Health (Brazil).
Brazil, Piauí	Ministry of Health (Brazil). Brazil - Severe Acute Respiratory Syndrome Database - including data from COVID-19. Rio de Janeiro, Brazil: Ministry of Health (Brazil).
Brazil, Rio de Janeiro	Ministry of Health (Brazil). Brazil - Severe Acute Respiratory Syndrome Database - including data from COVID-19. Rio de Janeiro, Brazil: Ministry of Health (Brazil).
Brazil, Rio Grande do Norte	Ministry of Health (Brazil). Brazil - Severe Acute Respiratory Syndrome Database - including data from COVID-19. Rio de Janeiro, Brazil: Ministry of Health (Brazil).
Brazil, Rio Grande do Sul	Ministry of Health (Brazil). Brazil - Severe Acute Respiratory Syndrome Database - including data from COVID-19. Rio de Janeiro, Brazil: Ministry of Health (Brazil).
Brazil, Rondônia	Ministry of Health (Brazil). Brazil - Severe Acute Respiratory Syndrome Database - including data from COVID-19. Rio de Janeiro, Brazil: Ministry of Health (Brazil).
Brazil, Roraima	Ministry of Health (Brazil). Brazil - Severe Acute Respiratory Syndrome Database - including data from COVID-19. Rio de Janeiro, Brazil: Ministry of Health (Brazil).
Brazil, Santa Catarina	Ministry of Health (Brazil). Brazil - Severe Acute Respiratory Syndrome Database - including data from COVID-19. Rio de Janeiro, Brazil: Ministry of Health (Brazil).
Brazil, São Paulo	Ministry of Health (Brazil). Brazil - Severe Acute Respiratory Syndrome Database - including data from COVID-19. Rio de Janeiro, Brazil: Ministry of Health (Brazil).
Brazil, Sergipe	Ministry of Health (Brazil). Brazil - Severe Acute Respiratory Syndrome Database - including data from COVID-19. Rio de Janeiro, Brazil: Ministry of Health (Brazil).
Brazil, Tocantins	Ministry of Health (Brazil). Brazil - Severe Acute Respiratory Syndrome Database - including data from COVID-19. Rio de Janeiro, Brazil: Ministry of Health (Brazil).
Canada, Alberta	Government of Alberta (Canada). Canada - COVID-19 Alberta Statistics: Severe Outcomes. Canada: Government of Alberta (Canada).
Canada, British Columbia	British Columbia Centre for Disease Control (BCCDC). Canada - British Columbia COVID-19 Situation Report Weekly Bulletin. British Columbia Centre for Disease Control (BCCDC).
Canada, Manitoba	Government of Manitoba (Canada). Canada - Manitoba Provincial Respiratory Surveillance Report COVID-19 and Seasonal Influenza Week 53 2020. Canada: Government of Manitoba (Canada).
Canada, Ontario	Public Health Ontario (Canada). Canada - Daily Epidemiologic Summary of COVID-19 in Ontario 2020. Canada: Public Health Ontario (Canada), 2020.
Canada, Ontario	Public Health Ontario (Canada). Canada - Ontario Daily Epidemiological Summary of COVID-19 2021. Canada: Public Health Ontario (Canada).
Chile	Ministry of Health (Chile), Ministry of Science, Technology, Knowledge and Innovation (Chile). Chile COVID-19 Total Cases by Commune. Chile: Ministry of Science, Technology, Knowledge and Innovation (Chile).
Chile	Department of Epidemiology, Ministry of Health (Chile). Chile Epidemiological Report on COVID-19 Disease. Santiago, Chile: Ministry of Health (Chile).
Costa Rica	Distance State University (UNED) (Costa Rica), Ministry of Health (Costa Rica). Costa Rica COVID-19 National Situation - Distance State University. San Jose, Costa Rica: Distance State University (UNED) (Costa Rica).
Croatia	Croatian National Institute of Public Health. Croatia National Institute of Public Health COVID-19 Report. Zagreb, Croatia: Croatian National Institute of Public Health.
Cyprus	European Centre for Disease Prevention and Control. Hospital and ICU Admission Rates and Current Occupancy for COVID-19 - European Centre for Disease Prevention and Control.
Czechia	Ministry of Health (Czech Republic). Czechia COVID-19 Open Data Sets. Prague, Czechia: Ministry of Health (Czech Republic).
Denmark	State Serum Institute (Denmark). Denmark State Serum Institute Monitoring of COVID-19. Denmark: State Serum Institute (Denmark).
Estonia	Health Board (Estonia). Estonia COVID-19 Open Data. Estonia: Health Board (Estonia).
Ethiopia	Ethiopian Public Health Institute (EPHI), Ministry of Health (Ethiopia). Ethiopia Public Health Institute COVID-19 Weekly Bulletin. Addis Ababa, Ethiopia: Ethiopian Public Health Institute (EPHI).
France	Government of France, Public Health France. France Covid-19 Information - Average number of new daily hospitalisations.
Germany	Robert Koch Institute (Germany). COVID-19 Hospitalisations in Germany. Robert Koch Institute (Germany).
Germany	Robert Koch Institute. Coronavirus Disease 2019 (COVID-19) Daily Situation Report - Robert Koch Institute. Berlin, Germany: Robert Koch Institute.
Germany	Wikipedia. COVID-19 pandemic in Germany. San Francisco, United States of America: Wikipedia.
Germany, Baden-Württemberg	Robert Koch Institute (Germany). COVID-19 Hospitalisations in Germany. Robert Koch Institute (Germany).

Germany, Bavaria	Robert Koch Institute (Germany). COVID-19 Hospitalisations in Germany. Robert Koch Institute (Germany).
Germany, Berlin	Robert Koch Institute (Germany). COVID-19 Hospitalisations in Germany. Robert Koch Institute (Germany).
Germany, Brandenburg	Robert Koch Institute (Germany). COVID-19 Hospitalisations in Germany. Robert Koch Institute (Germany).
Germany, Bremen	Robert Koch Institute (Germany). COVID-19 Hospitalisations in Germany. Robert Koch Institute (Germany).
Germany, Hamburg	Robert Koch Institute (Germany). COVID-19 Hospitalisations in Germany. Robert Koch Institute (Germany).
Germany, Hesse	Robert Koch Institute (Germany). COVID-19 Hospitalisations in Germany. Robert Koch Institute (Germany).
Germany, Lower Saxony	Robert Koch Institute (Germany). COVID-19 Hospitalisations in Germany. Robert Koch Institute (Germany).
Germany, Mecklenburg-Vorpommern	Robert Koch Institute (Germany). COVID-19 Hospitalisations in Germany. Robert Koch Institute (Germany).
Germany, North Rhine-Westphalia	State Government of North Rhine-Westphalia. Germany - North Rhine-Westphalia COVID-19 Dashboard.
Germany, Rhineland-Palatinate	Robert Koch Institute (Germany). COVID-19 Hospitalisations in Germany. Robert Koch Institute (Germany).
Germany, Saarland	Robert Koch Institute (Germany). COVID-19 Hospitalisations in Germany. Robert Koch Institute (Germany).
Germany, Saxony	Robert Koch Institute (Germany). COVID-19 Hospitalisations in Germany. Robert Koch Institute (Germany).
Germany, Saxony-Anhalt	Robert Koch Institute (Germany). COVID-19 Hospitalisations in Germany. Robert Koch Institute (Germany).
Germany, Schleswig-Holstein	State Government of Schleswig-Holstein (Germany). Germany - Schleswig-Holstein COVID-19 Cases, Hospitalisations, and Deaths. Germany: State Government of Schleswig-Holstein (Germany).
Germany, Thuringia	Robert Koch Institute (Germany). COVID-19 Hospitalisations in Germany. Robert Koch Institute (Germany).
Greece	European Centre for Disease Prevention and Control. Hospital and ICU Admission Rates and Current Occupancy for COVID-19 - European Centre for Disease Prevention and Control.
Guinea-Bissau	COVID-19 High Commission (Guinea Bissau), Ministry of Public Health (Guinea-Bissau). Guinea-Bissau COVID-19 High Commission Facebook Updates. Menlo Park, United States of America: Facebook. Retrieved on March 22, 2021 from https://www.facebook.com/accovid19/
Haiti	Ministry of Public Health and Population (Haiti). Haiti COVID-19 Surveillance. Port-au-Prince, Haiti: Ministry of Public Health and Population (Haiti).
Iceland	European Centre for Disease Prevention and Control. Hospital and ICU Admission Rates and Current Occupancy for COVID-19 - European Centre for Disease Prevention and Control.
India, Goa	Integrated Disease Surveillance Programme (IDSP) (India). India - Goa COVID-19 Media Bulletin. Goa, India: Government of Goa (India).
Ireland	Health Service Executive (HSE) (Ireland). Ireland COVID-19 Acute Hospitalisation. GeoHive.
Israel	European Centre for Disease Prevention and Control, Ministry of Health (Israel). Israel Weekly Number of New Hospitalisations Due to COVID-19. United Kingdom: Our World in Data.
Italy	European Centre for Disease Prevention and Control. Hospital and ICU Admission Rates and Current Occupancy for COVID-19 - European Centre for Disease Prevention and Control.
Jordan	Ministry of Health (Jordan). Jordan Ministry of Health COVID-19 Updates. Amman, Jordan: Ministry of Health (Jordan).
Latvia	European Centre for Disease Prevention and Control. Hospital and ICU Admission Rates and Current Occupancy for COVID-19 - European Centre for Disease Prevention and Control.
Luxembourg	Ministry of Health (Luxembourg). Luxembourg COVID-19 Hospital Admissions and Discharges - Ministry of Health.
Malawi	Public Health Institute of Malawi (PHIM). Malawi Covid-19 Weekly Epidemiologcial Report. Malawi: Public Health Institute of Malawi (PHIM).
Malta	European Centre for Disease Prevention and Control. Hospital and ICU Admission Rates and Current Occupancy for COVID-19 - European Centre for Disease Prevention and Control.
Mexico	Government of Mexico. Mexico Health Secretary COVID-19 Daily Technical Release.
Mexico	Directorate General of Epidemiology, Secretariat of Health (Mexico). Mexico General Directorate of Epidemiology COVID-19 Daily Cases, Nationally and by State.
Mexico, Aguascalientes	Government of Mexico. Mexico Health Secretary COVID-19 Daily Technical Release.
Mexico, Aguascalientes	Directorate General of Epidemiology, Secretariat of Health (Mexico). Mexico General Directorate of Epidemiology COVID-19 Daily Cases, Nationally and by State.
Mexico, Baja California	Government of Mexico. Mexico Health Secretary COVID-19 Daily Technical Release.
Mexico, Baja California	Directorate General of Epidemiology, Secretariat of Health (Mexico). Mexico General Directorate of Epidemiology COVID-19 Daily Cases, Nationally and by State.
Mexico, Baja California Sur	Government of Mexico. Mexico Health Secretary COVID-19 Daily Technical Release.

Mexico, Baja California Sur	Directorate General of Epidemiology, Secretariat of Health (Mexico). Mexico General Directorate of Epidemiology COVID-19 Daily Cases, Nationally and by State.
Mexico, Campeche	Government of Mexico. Mexico Health Secretary COVID-19 Daily Technical Release.
Mexico, Campeche	Directorate General of Epidemiology, Secretariat of Health (Mexico). Mexico General Directorate of Epidemiology COVID-19 Daily Cases, Nationally and by State.
Mexico, Chiapas	Government of Mexico. Mexico Health Secretary COVID-19 Daily Technical Release.
Mexico, Chiapas	Directorate General of Epidemiology, Secretariat of Health (Mexico). Mexico General Directorate of Epidemiology COVID-19 Daily Cases, Nationally and by State.
Mexico, Chihuahua	Government of Mexico. Mexico Health Secretary COVID-19 Daily Technical Release.
Mexico, Chihuahua	Directorate General of Epidemiology, Secretariat of Health (Mexico). Mexico General Directorate of Epidemiology COVID-19 Daily Cases, Nationally and by State.
Mexico, Coahuila	Government of Mexico. Mexico Health Secretary COVID-19 Daily Technical Release.
Mexico, Coahuila	Directorate General of Epidemiology, Secretariat of Health (Mexico). Mexico General Directorate of Epidemiology COVID-19 Daily Cases, Nationally and by State.
Mexico, Colima	Government of Mexico. Mexico Health Secretary COVID-19 Daily Technical Release.
Mexico, Colima	Directorate General of Epidemiology, Secretariat of Health (Mexico). Mexico General Directorate of Epidemiology COVID-19 Daily Cases, Nationally and by State.
Mexico, Durango	Government of Mexico. Mexico Health Secretary COVID-19 Daily Technical Release.
Mexico, Durango	Directorate General of Epidemiology, Secretariat of Health (Mexico). Mexico General Directorate of Epidemiology COVID-19 Daily Cases, Nationally and by State.
Mexico, Guanajuato	Government of Mexico. Mexico Health Secretary COVID-19 Daily Technical Release.
Mexico, Guanajuato	Directorate General of Epidemiology, Secretariat of Health (Mexico). Mexico General Directorate of Epidemiology COVID-19 Daily Cases, Nationally and by State.
Mexico, Guerrero	Government of Mexico. Mexico Health Secretary COVID-19 Daily Technical Release.
Mexico, Guerrero	Directorate General of Epidemiology, Secretariat of Health (Mexico). Mexico General Directorate of Epidemiology COVID-19 Daily Cases, Nationally and by State.
Mexico, Hidalgo	Government of Mexico. Mexico Health Secretary COVID-19 Daily Technical Release.
Mexico, Hidalgo	Directorate General of Epidemiology, Secretariat of Health (Mexico). Mexico General Directorate of Epidemiology COVID-19 Daily Cases, Nationally and by State.
Mexico, Jalisco	Government of Mexico. Mexico Health Secretary COVID-19 Daily Technical Release.
Mexico, Jalisco	Directorate General of Epidemiology, Secretariat of Health (Mexico). Mexico General Directorate of Epidemiology COVID-19 Daily Cases, Nationally and by State.
Mexico, México	Government of Mexico. Mexico Health Secretary COVID-19 Daily Technical Release.
Mexico, México	Directorate General of Epidemiology, Secretariat of Health (Mexico). Mexico General Directorate of Epidemiology COVID-19 Daily Cases, Nationally and by State.
Mexico, Mexico City	Government of Mexico. Mexico Health Secretary COVID-19 Daily Technical Release.
Mexico, Mexico City	Directorate General of Epidemiology, Secretariat of Health (Mexico). Mexico General Directorate of Epidemiology COVID-19 Daily Cases, Nationally and by State.
Mexico, Michoacán de Ocampo	Government of Mexico. Mexico Health Secretary COVID-19 Daily Technical Release.
Mexico, Michoacán de Ocampo	Directorate General of Epidemiology, Secretariat of Health (Mexico). Mexico General Directorate of Epidemiology COVID-19 Daily Cases, Nationally and by State.
Mexico, Morelos	Government of Mexico. Mexico Health Secretary COVID-19 Daily Technical Release.
Mexico, Morelos	Directorate General of Epidemiology, Secretariat of Health (Mexico). Mexico General Directorate of Epidemiology COVID-19 Daily Cases, Nationally and by State.
Mexico, Nayarit	Government of Mexico. Mexico Health Secretary COVID-19 Daily Technical Release.
Mexico, Nayarit	Directorate General of Epidemiology, Secretariat of Health (Mexico). Mexico General Directorate of Epidemiology COVID-19 Daily Cases, Nationally and by State.
Mexico, Nuevo León	Government of Mexico. Mexico Health Secretary COVID-19 Daily Technical Release.
Mexico, Nuevo León	Directorate General of Epidemiology, Secretariat of Health (Mexico). Mexico General Directorate of Epidemiology COVID-19 Daily Cases, Nationally and by State.
Mexico, Oaxaca	Government of Mexico. Mexico Health Secretary COVID-19 Daily Technical Release.
Mexico, Oaxaca	Directorate General of Epidemiology, Secretariat of Health (Mexico). Mexico General Directorate of Epidemiology COVID-19 Daily Cases, Nationally and by State.
Mexico, Puebla	Government of Mexico. Mexico Health Secretary COVID-19 Daily Technical Release.
Mexico, Puebla	Directorate General of Epidemiology, Secretariat of Health (Mexico). Mexico General Directorate of Epidemiology COVID-19 Daily Cases, Nationally and by State.

Mexico, Querétaro	Government of Mexico. Mexico Health Secretary COVID-19 Daily Technical Release.
Mexico, Querétaro	Directorate General of Epidemiology, Secretariat of Health (Mexico). Mexico General Directorate of Epidemiology COVID-19 Daily Cases, Nationally and by State.
Mexico, Quintana Roo	Government of Mexico. Mexico Health Secretary COVID-19 Daily Technical Release.
Mexico, Quintana Roo	Directorate General of Epidemiology, Secretariat of Health (Mexico). Mexico General Directorate of Epidemiology COVID-19 Daily Cases, Nationally and by State.
Mexico, San Luis Potosí	Government of Mexico. Mexico Health Secretary COVID-19 Daily Technical Release.
Mexico, San Luis Potosí	Directorate General of Epidemiology, Secretariat of Health (Mexico). Mexico General Directorate of Epidemiology COVID-19 Daily Cases, Nationally and by State.
Mexico, Sinaloa	Government of Mexico. Mexico Health Secretary COVID-19 Daily Technical Release.
Mexico, Sinaloa	Directorate General of Epidemiology, Secretariat of Health (Mexico). Mexico General Directorate of Epidemiology COVID-19 Daily Cases, Nationally and by State.
Mexico, Sonora	Government of Mexico. Mexico Health Secretary COVID-19 Daily Technical Release.
Mexico, Sonora	Directorate General of Epidemiology, Secretariat of Health (Mexico). Mexico General Directorate of Epidemiology COVID-19 Daily Cases, Nationally and by State.
Mexico, Tabasco	Government of Mexico. Mexico Health Secretary COVID-19 Daily Technical Release.
Mexico, Tabasco	Directorate General of Epidemiology, Secretariat of Health (Mexico). Mexico General Directorate of Epidemiology COVID-19 Daily Cases, Nationally and by State.
Mexico, Tamaulipas	Government of Mexico. Mexico Health Secretary COVID-19 Daily Technical Release.
Mexico, Tamaulipas	Directorate General of Epidemiology, Secretariat of Health (Mexico). Mexico General Directorate of Epidemiology COVID-19 Daily Cases, Nationally and by State.
Mexico, Tlaxcala	Government of Mexico. Mexico Health Secretary COVID-19 Daily Technical Release.
Mexico, Tlaxcala	Directorate General of Epidemiology, Secretariat of Health (Mexico). Mexico General Directorate of Epidemiology COVID-19 Daily Cases, Nationally and by State.
Mexico, Veracruz de Ignacio de la Llave	Government of Mexico. Mexico Health Secretary COVID-19 Daily Technical Release.
Mexico, Veracruz de Ignacio de la Llave	Directorate General of Epidemiology, Secretariat of Health (Mexico). Mexico General Directorate of Epidemiology COVID-19 Daily Cases, Nationally and by State.
Mexico, Yucatán	Government of Mexico. Mexico Health Secretary COVID-19 Daily Technical Release.
Mexico, Yucatán	Directorate General of Epidemiology, Secretariat of Health (Mexico). Mexico General Directorate of Epidemiology COVID-19 Daily Cases, Nationally and by State.
Mexico, Zacatecas	Government of Mexico. Mexico Health Secretary COVID-19 Daily Technical Release.
Mexico, Zacatecas	Directorate General of Epidemiology, Secretariat of Health (Mexico). Mexico General Directorate of Epidemiology COVID-19 Daily Cases, Nationally and by State.
Mozambique	Ministry of Health (Mozambique). Mozambique COVID-19 Daily Bulletin - Ministry of Health.
Mozambique	National Institute of Health (Mozambique). Mozambique National Institute of Health COVID-19 Daily Surveillance Bulletin. Maputo, Mozambique: National Institute of Health (Mozambique).
Netherlands	National Coordination Center for Patient Distribution (LCPS) (Netherlands). Netherlands Covid-19 Hospital Admissions.
Northern Mariana Islands	The COVID Tracking Project, The Atlantic. United States COVID Tracking Project 2021. United States of America: The Atlantic, 2021.
Norway	Norwegian Institute of Public Health. Norway COVID-19 Daily Status Report- Norwegian Institute of Public Health. Oslo, Norway: Norwegian Institute of Public Health.
Oman	Ministry of Health (Oman). Oman Ministry of Health COVID-19 Statement. Muscat, Oman: Ministry of Health (Oman).
Pakistan, Azad Jammu & Kashmir	Government of Sindh (Pakistan). Pakistan - Sindh COVID 19 Daily Situation Report. Karachi, Pakistan: Government of Sindh (Pakistan).
Pakistan, Balochistan	Government of Sindh (Pakistan). Pakistan - Sindh COVID 19 Daily Situation Report. Karachi, Pakistan: Government of Sindh (Pakistan).
Pakistan, Gilgit-Baltistan	Government of Sindh (Pakistan). Pakistan - Sindh COVID 19 Daily Situation Report. Karachi, Pakistan: Government of Sindh (Pakistan).
Pakistan, Islamabad Capital Territory	Government of Sindh (Pakistan). Pakistan - Sindh COVID 19 Daily Situation Report. Karachi, Pakistan: Government of Sindh (Pakistan).
Pakistan, Khyber Pakhtunkhwa	Government of Sindh (Pakistan). Pakistan - Sindh COVID 19 Daily Situation Report. Karachi, Pakistan: Government of Sindh (Pakistan).
Pakistan, Punjab	Government of Sindh (Pakistan). Pakistan - Sindh COVID 19 Daily Situation Report. Karachi, Pakistan: Government of Sindh (Pakistan).
Pakistan, Sindh	Government of Sindh (Pakistan). Pakistan - Sindh COVID 19 Daily Situation Report. Karachi, Pakistan: Government of Sindh (Pakistan).
Poland	Ministry of Health (Poland). Poland COVID-19 Hospital Admission Patient Counts, by Province - Ministry of Health.

Puerto Rico	U.S. Department of Health and Human Services. United States - COVID-19 Reported Patient Impact and Hospital Capacity by State Timeseries. Washington DC, United States of America: U.S. Department of Health and Human Services.
Qatar	Ministry of Public Health (Qatar). Qatar Ministry of Public Health Twitter Communications and COVID-19 Updates. Doha, Qatar: Ministry of Public Health (Qatar).
Slovenia	European Centre for Disease Prevention and Control. Hospital and ICU Admission Rates and Current Occupancy for COVID-19 - European Centre for Disease Prevention and Control.
South Africa	Department of Health (South Africa). South Africa National Department of Health Update on Coronavirus. Pretoria, South Africa: Department of Health (South Africa), 2020.
South Africa	National Institute for Communicable Diseases (South Africa). South Africa COVID-19 Hospital Surveillance (DatCov) Report - National Institute for Communicable Diseases.
Spain	European Centre for Disease Prevention and Control. Hospital and ICU Admission Rates and Current Occupancy for COVID-19 - European Centre for Disease Prevention and Control.
Spain, Andalusia	Andalusia Ministry of Health and Families (Spain). Spain - Andalusia Ministry of Health and Families Coronavirus Report.
Spain, Andalusia	Ministry of Health, Consumption and Social Welfare (Spain). Spain Ministry of Health, Consumption, and Social Welfare COVID-19 Situation Update. Spain: Ministry of Health, Consumption and Social Welfare (Spain).
Spain, Aragon	Ministry of Health, Consumption and Social Welfare (Spain). Spain Ministry of Health, Consumption, and Social Welfare COVID-19 Situation Update. Spain: Ministry of Health, Consumption and Social Welfare (Spain).
Spain, Aragon	Institute of Health Carlos III (Spain), Ministry of Health, Consumption and Social Welfare (Spain). Spain Carlos III Health Institute Situation of COVID-19. Madrid, Spain: Institute of Health Carlos III (Spain).
Spain, Asturias	Ministry of Health, Consumption and Social Welfare (Spain). Spain Ministry of Health, Consumption, and Social Welfare COVID-19 Situation Update. Spain: Ministry of Health, Consumption and Social Welfare (Spain).
Spain, Asturias	Institute of Health Carlos III (Spain), Ministry of Health, Consumption and Social Welfare (Spain). Spain Carlos III Health Institute Situation of COVID-19. Madrid, Spain: Institute of Health Carlos III (Spain).
Spain, Balearic Islands	Ministry of Health, Consumption and Social Welfare (Spain). Spain Ministry of Health, Consumption, and Social Welfare COVID-19 Situation Update. Spain: Ministry of Health, Consumption and Social Welfare (Spain).
Spain, Balearic Islands	Institute of Health Carlos III (Spain), Ministry of Health, Consumption and Social Welfare (Spain). Spain Carlos III Health Institute Situation of COVID-19. Madrid, Spain: Institute of Health Carlos III (Spain).
Spain, Basque Country	Ministry of Health, Consumption and Social Welfare (Spain). Spain Ministry of Health, Consumption, and Social Welfare COVID-19 Situation Update. Spain: Ministry of Health, Consumption and Social Welfare (Spain).
Spain, Basque Country	Institute of Health Carlos III (Spain), Ministry of Health, Consumption and Social Welfare (Spain). Spain Carlos III Health Institute Situation of COVID-19. Madrid, Spain: Institute of Health Carlos III (Spain).
Spain, Canary Islands	Ministry of Health, Consumption and Social Welfare (Spain). Spain Ministry of Health, Consumption, and Social Welfare COVID-19 Situation Update. Spain: Ministry of Health, Consumption and Social Welfare (Spain).
Spain, Canary Islands	Institute of Health Carlos III (Spain), Ministry of Health, Consumption and Social Welfare (Spain). Spain Carlos III Health Institute Situation of COVID-19. Madrid, Spain: Institute of Health Carlos III (Spain).
Spain, Cantabria	Ministry of Health, Consumption and Social Welfare (Spain). Spain Ministry of Health, Consumption, and Social Welfare COVID-19 Situation Update. Spain: Ministry of Health, Consumption and Social Welfare (Spain).
Spain, Cantabria	Institute of Health Carlos III (Spain), Ministry of Health, Consumption and Social Welfare (Spain). Spain Carlos III Health Institute Situation of COVID-19. Madrid, Spain: Institute of Health Carlos III (Spain).
Spain, Castile and León	Ministry of Health, Consumption and Social Welfare (Spain). Spain Ministry of Health, Consumption, and Social Welfare COVID-19 Situation Update. Spain: Ministry of Health, Consumption and Social Welfare (Spain).
Spain, Castile and León	Institute of Health Carlos III (Spain), Ministry of Health, Consumption and Social Welfare (Spain). Spain Carlos III Health Institute Situation of COVID-19. Madrid, Spain: Institute of Health Carlos III (Spain).
Spain, Castilla–La Mancha	Ministry of Health, Consumption and Social Welfare (Spain). Spain Ministry of Health, Consumption, and Social Welfare COVID-19 Situation Update. Spain: Ministry of Health, Consumption and Social Welfare (Spain).
Spain, Castilla–La Mancha	Institute of Health Carlos III (Spain), Ministry of Health, Consumption and Social Welfare (Spain). Spain Carlos III Health Institute Situation of COVID-19. Madrid, Spain: Institute of Health Carlos III (Spain).
Spain, Catalonia	Ministry of Health, Consumption and Social Welfare (Spain). Spain Ministry of Health, Consumption, and Social Welfare COVID-19 Situation Update. Spain: Ministry of Health, Consumption and Social Welfare (Spain).
Spain, Catalonia	Institute of Health Carlos III (Spain), Ministry of Health, Consumption and Social Welfare (Spain). Spain Carlos III Health Institute Situation of COVID-19. Madrid, Spain: Institute of Health Carlos III (Spain).
Spain, Ceuta	Ministry of Health, Consumption and Social Welfare (Spain). Spain Ministry of Health, Consumption, and Social Welfare COVID-19 Situation Update. Spain: Ministry of Health, Consumption and Social Welfare (Spain).
Spain, Ceuta	Institute of Health Carlos III (Spain), Ministry of Health, Consumption and Social Welfare (Spain). Spain Carlos III Health Institute Situation of COVID-19. Madrid, Spain: Institute of Health Carlos III (Spain).
Spain, Community of Madrid	City of Madrid (Spain), Madrid Health Service (Spain). Spain - Madrid Health Service COVID-19 Current Situation Daily Status Report. Madrid, Spain: City of Madrid (Spain).
Spain, Community of Madrid	Ministry of Health, Consumption and Social Welfare (Spain). Spain Ministry of Health, Consumption, and Social Welfare COVID-19 Situation Update. Spain: Ministry of Health, Consumption and Social Welfare (Spain).
Spain, Extremadura	Ministry of Health, Consumption and Social Welfare (Spain). Spain Ministry of Health, Consumption, and Social Welfare COVID-19 Situation Update. Spain: Ministry of Health, Consumption and Social Welfare (Spain).
Spain, Extremadura	Institute of Health Carlos III (Spain), Ministry of Health, Consumption and Social Welfare (Spain). Spain Carlos III Health Institute Situation of COVID-19. Madrid, Spain: Institute of Health Carlos III (Spain).
Spain, Galicia	Ministry of Health, Consumption and Social Welfare (Spain). Spain Ministry of Health, Consumption, and Social Welfare COVID-19 Situation Update. Spain: Ministry of Health, Consumption and Social Welfare (Spain).

Spain, Galicia	Institute of Health Carlos III (Spain), Ministry of Health, Consumption and Social Welfare (Spain). Spain Carlos III Health Institute Situation of COVID-19. Madrid, Spain: Institute of Health Carlos III (Spain).
Spain, La Rioja	Ministry of Health, Consumption and Social Welfare (Spain). Spain Ministry of Health, Consumption, and Social Welfare COVID-19 Situation Update. Spain: Ministry of Health, Consumption and Social Welfare (Spain).
Spain, La Rioja	Institute of Health Carlos III (Spain), Ministry of Health, Consumption and Social Welfare (Spain). Spain Carlos III Health Institute Situation of COVID-19. Madrid, Spain: Institute of Health Carlos III (Spain).
Spain, Melilla	Ministry of Health, Consumption and Social Welfare (Spain). Spain Ministry of Health, Consumption, and Social Welfare COVID-19 Situation Update. Spain: Ministry of Health, Consumption and Social Welfare (Spain).
Spain, Melilla	Institute of Health Carlos III (Spain), Ministry of Health, Consumption and Social Welfare (Spain). Spain Carlos III Health Institute Situation of COVID-19. Madrid, Spain: Institute of Health Carlos III (Spain).
Spain, Murcia	Ministry of Health, Consumption and Social Welfare (Spain). Spain Ministry of Health, Consumption, and Social Welfare COVID-19 Situation Update. Spain: Ministry of Health, Consumption and Social Welfare (Spain).
Spain, Murcia	Institute of Health Carlos III (Spain), Ministry of Health, Consumption and Social Welfare (Spain). Spain Carlos III Health Institute Situation of COVID-19. Madrid, Spain: Institute of Health Carlos III (Spain).
Spain, Navarre	Government of Navarra (Spain). Spain - Navarra COVID-19 Testing, Deaths, Hospitalisation Data May-June 2020. Spain: Government of Navarra (Spain), 2020.
Spain, Navarre	Ministry of Health, Consumption and Social Welfare (Spain). Spain Ministry of Health, Consumption, and Social Welfare COVID-19 Situation Update. Spain: Ministry of Health, Consumption and Social Welfare (Spain).
Spain, Valencian Community	Ministry of Health, Consumption and Social Welfare (Spain). Spain Ministry of Health, Consumption, and Social Welfare COVID-19 Situation Update. Spain: Ministry of Health, Consumption and Social Welfare (Spain).
Spain, Valencian Community	Institute of Health Carlos III (Spain), Ministry of Health, Consumption and Social Welfare (Spain). Spain Carlos III Health Institute Situation of COVID-19. Madrid, Spain: Institute of Health Carlos III (Spain).
Sweden	National Board of Health and Welfare (Sweden). Sweden National Board of Health and Welfare COVID-19 Newly Enrolled Patients. Stockholm, Sweden: National Board of Health and Welfare (Sweden).
Switzerland	Federal Office of Public Health (Switzerland). Switzerland Federal Office of Public Health New Coronavirus Current Situation. Berne, Switzerland: Federal Office of Public Health (Switzerland).
UK	Department of Health (United Kingdom), NHS England, Public Health England. United Kingdom COVID-19 Cases. London, England: Public Health England, 2020.
UK	Office for National Statistics (ONS) (United Kingdom). United Kingdom - England and Wales Deaths Registered Weekly, Provisional.
UK	National Health Service (United Kingdom), Public Health England. United Kingdom Coronavirus (COVID-19) Data Dashboard.
UK, England	Department of Health (United Kingdom), NHS England, Public Health England. United Kingdom COVID-19 Cases. London, England: Public Health England, 2020.
UK, England	Office for National Statistics (ONS) (United Kingdom). United Kingdom - England and Wales Deaths Registered Weekly, Provisional.
UK, England	National Health Service (United Kingdom), Public Health England. United Kingdom Coronavirus (COVID-19) Data Dashboard.
UK, Northern Ireland	Department of Health (United Kingdom), NHS England, Public Health England. United Kingdom COVID-19 Cases. London, England: Public Health England, 2020.
UK, Northern Ireland	Office for National Statistics (ONS) (United Kingdom). United Kingdom - England and Wales Deaths Registered Weekly, Provisional.
UK, Northern Ireland	National Health Service (United Kingdom), Public Health England. United Kingdom Coronavirus (COVID-19) Data Dashboard.
UK, Scotland	Department of Health (United Kingdom), NHS England, Public Health England. United Kingdom COVID-19 Cases. London, England: Public Health England, 2020.
UK, Scotland	Office for National Statistics (ONS) (United Kingdom). United Kingdom - England and Wales Deaths Registered Weekly, Provisional.
UK, Scotland	National Health Service (United Kingdom), Public Health England. United Kingdom Coronavirus (COVID-19) Data Dashboard.
UK, Wales	Department of Health (United Kingdom), NHS England, Public Health England. United Kingdom COVID-19 Cases. London, England: Public Health England, 2020.
UK, Wales	Office for National Statistics (ONS) (United Kingdom). United Kingdom - England and Wales Deaths Registered Weekly, Provisional.
UK, Wales	National Health Service (United Kingdom), Public Health England. United Kingdom Coronavirus (COVID-19) Data Dashboard.
USA, Alabama	U.S. Department of Health and Human Services. United States - COVID-19 Reported Patient Impact and Hospital Capacity by State Timeseries. Washington DC, United States of America: U.S. Department of Health and Human Services.
USA, Alaska	U.S. Department of Health and Human Services. United States - COVID-19 Reported Patient Impact and Hospital Capacity by State Timeseries. Washington DC, United States of America: U.S. Department of Health and Human Services.
USA, Arizona	U.S. Department of Health and Human Services. United States - COVID-19 Reported Patient Impact and Hospital Capacity by State Timeseries. Washington DC, United States of America: U.S. Department of Health and Human Services.

USA, Arkansas	U.S. Department of Health and Human Services. United States - COVID-19 Reported Patient Impact and Hospital Capacity by State Timeseries. Washington DC, United States of America: U.S. Department of Health and Human Services.
USA, California	U.S. Department of Health and Human Services. United States - COVID-19 Reported Patient Impact and Hospital Capacity by State Timeseries. Washington DC, United States of America: U.S. Department of Health and Human Services.
USA, Colorado	U.S. Department of Health and Human Services. United States - COVID-19 Reported Patient Impact and Hospital Capacity by State Timeseries. Washington DC, United States of America: U.S. Department of Health and Human Services.
USA, Connecticut	U.S. Department of Health and Human Services. United States - COVID-19 Reported Patient Impact and Hospital Capacity by State Timeseries. Washington DC, United States of America: U.S. Department of Health and Human Services.
USA, Delaware	U.S. Department of Health and Human Services. United States - COVID-19 Reported Patient Impact and Hospital Capacity by State Timeseries. Washington DC, United States of America: U.S. Department of Health and Human Services.
USA, Florida	U.S. Department of Health and Human Services. United States - COVID-19 Reported Patient Impact and Hospital Capacity by State Timeseries. Washington DC, United States of America: U.S. Department of Health and Human Services.
USA, Georgia	U.S. Department of Health and Human Services. United States - COVID-19 Reported Patient Impact and Hospital Capacity by State Timeseries. Washington DC, United States of America: U.S. Department of Health and Human Services.
USA, Hawaii	U.S. Department of Health and Human Services. United States - COVID-19 Reported Patient Impact and Hospital Capacity by State Timeseries. Washington DC, United States of America: U.S. Department of Health and Human Services.
USA, Idaho	U.S. Department of Health and Human Services. United States - COVID-19 Reported Patient Impact and Hospital Capacity by State Timeseries. Washington DC, United States of America: U.S. Department of Health and Human Services.
USA, Illinois	U.S. Department of Health and Human Services. United States - COVID-19 Reported Patient Impact and Hospital Capacity by State Timeseries. Washington DC, United States of America: U.S. Department of Health and Human Services.
USA, Indiana	U.S. Department of Health and Human Services. United States - COVID-19 Reported Patient Impact and Hospital Capacity by State Timeseries. Washington DC, United States of America: U.S. Department of Health and Human Services.
USA, Iowa	U.S. Department of Health and Human Services. United States - COVID-19 Reported Patient Impact and Hospital Capacity by State Timeseries. Washington DC, United States of America: U.S. Department of Health and Human Services.
USA, Kansas	U.S. Department of Health and Human Services. United States - COVID-19 Reported Patient Impact and Hospital Capacity by State Timeseries. Washington DC, United States of America: U.S. Department of Health and Human Services.
USA, Kentucky	U.S. Department of Health and Human Services. United States - COVID-19 Reported Patient Impact and Hospital Capacity by State Timeseries. Washington DC, United States of America: U.S. Department of Health and Human Services.
USA, Louisiana	U.S. Department of Health and Human Services. United States - COVID-19 Reported Patient Impact and Hospital Capacity by State Timeseries. Washington DC, United States of America: U.S. Department of Health and Human Services.
USA, Maine	U.S. Department of Health and Human Services. United States - COVID-19 Reported Patient Impact and Hospital Capacity by State Timeseries. Washington DC, United States of America: U.S. Department of Health and Human Services.
USA, Maryland	U.S. Department of Health and Human Services. United States - COVID-19 Reported Patient Impact and Hospital Capacity by State Timeseries. Washington DC, United States of America: U.S. Department of Health and Human Services.
USA, Massachusetts	U.S. Department of Health and Human Services. United States - COVID-19 Reported Patient Impact and Hospital Capacity by State Timeseries. Washington DC, United States of America: U.S. Department of Health and Human Services.
USA, Michigan	U.S. Department of Health and Human Services. United States - COVID-19 Reported Patient Impact and Hospital Capacity by State Timeseries. Washington DC, United States of America: U.S. Department of Health and Human Services.
USA, Minnesota	U.S. Department of Health and Human Services. United States - COVID-19 Reported Patient Impact and Hospital Capacity by State Timeseries. Washington DC, United States of America: U.S. Department of Health and Human Services.
USA, Mississippi	U.S. Department of Health and Human Services. United States - COVID-19 Reported Patient Impact and Hospital Capacity by State Timeseries. Washington DC, United States of America: U.S. Department of Health and Human Services.
USA, Missouri	U.S. Department of Health and Human Services. United States - COVID-19 Reported Patient Impact and Hospital Capacity by State Timeseries. Washington DC, United States of America: U.S. Department of Health and Human Services.

USA, Montana	U.S. Department of Health and Human Services. United States - COVID-19 Reported Patient Impact and Hospital Capacity by State Timeseries. Washington DC, United States of America: U.S. Department of Health and Human Services.
USA, Nebraska	U.S. Department of Health and Human Services. United States - COVID-19 Reported Patient Impact and Hospital Capacity by State Timeseries. Washington DC, United States of America: U.S. Department of Health and Human Services.
USA, Nevada	U.S. Department of Health and Human Services. United States - COVID-19 Reported Patient Impact and Hospital Capacity by State Timeseries. Washington DC, United States of America: U.S. Department of Health and Human Services.
USA, New Hampshire	U.S. Department of Health and Human Services. United States - COVID-19 Reported Patient Impact and Hospital Capacity by State Timeseries. Washington DC, United States of America: U.S. Department of Health and Human Services.
USA, New Jersey	U.S. Department of Health and Human Services. United States - COVID-19 Reported Patient Impact and Hospital Capacity by State Timeseries. Washington DC, United States of America: U.S. Department of Health and Human Services.
USA, New Mexico	U.S. Department of Health and Human Services. United States - COVID-19 Reported Patient Impact and Hospital Capacity by State Timeseries. Washington DC, United States of America: U.S. Department of Health and Human Services.
USA, New York	U.S. Department of Health and Human Services. United States - COVID-19 Reported Patient Impact and Hospital Capacity by State Timeseries. Washington DC, United States of America: U.S. Department of Health and Human Services.
USA, North Carolina	U.S. Department of Health and Human Services. United States - COVID-19 Reported Patient Impact and Hospital Capacity by State Timeseries. Washington DC, United States of America: U.S. Department of Health and Human Services.
USA, North Dakota	U.S. Department of Health and Human Services. United States - COVID-19 Reported Patient Impact and Hospital Capacity by State Timeseries. Washington DC, United States of America: U.S. Department of Health and Human Services.
USA, Ohio	U.S. Department of Health and Human Services. United States - COVID-19 Reported Patient Impact and Hospital Capacity by State Timeseries. Washington DC, United States of America: U.S. Department of Health and Human Services.
USA, Oklahoma	U.S. Department of Health and Human Services. United States - COVID-19 Reported Patient Impact and Hospital Capacity by State Timeseries. Washington DC, United States of America: U.S. Department of Health and Human Services.
USA, Oregon	U.S. Department of Health and Human Services. United States - COVID-19 Reported Patient Impact and Hospital Capacity by State Timeseries. Washington DC, United States of America: U.S. Department of Health and Human Services.
USA, Pennsylvania	U.S. Department of Health and Human Services. United States - COVID-19 Reported Patient Impact and Hospital Capacity by State Timeseries. Washington DC, United States of America: U.S. Department of Health and Human Services.
USA, Rhode Island	U.S. Department of Health and Human Services. United States - COVID-19 Reported Patient Impact and Hospital Capacity by State Timeseries. Washington DC, United States of America: U.S. Department of Health and Human Services.
USA, South Carolina	U.S. Department of Health and Human Services. United States - COVID-19 Reported Patient Impact and Hospital Capacity by State Timeseries. Washington DC, United States of America: U.S. Department of Health and Human Services.
USA, South Dakota	U.S. Department of Health and Human Services. United States - COVID-19 Reported Patient Impact and Hospital Capacity by State Timeseries. Washington DC, United States of America: U.S. Department of Health and Human Services.
USA, Tennessee	U.S. Department of Health and Human Services. United States - COVID-19 Reported Patient Impact and Hospital Capacity by State Timeseries. Washington DC, United States of America: U.S. Department of Health and Human Services.
USA, Texas	U.S. Department of Health and Human Services. United States - COVID-19 Reported Patient Impact and Hospital Capacity by State Timeseries. Washington DC, United States of America: U.S. Department of Health and Human Services.
USA, Utah	U.S. Department of Health and Human Services. United States - COVID-19 Reported Patient Impact and Hospital Capacity by State Timeseries. Washington DC, United States of America: U.S. Department of Health and Human Services.
USA, Vermont	U.S. Department of Health and Human Services. United States - COVID-19 Reported Patient Impact and Hospital Capacity by State Timeseries. Washington DC, United States of America: U.S. Department of Health and Human Services.
USA, Virginia	U.S. Department of Health and Human Services. United States - COVID-19 Reported Patient Impact and Hospital Capacity by State Timeseries. Washington DC, United States of America: U.S. Department of Health and Human Services.
USA, Washington	U.S. Department of Health and Human Services. United States - COVID-19 Reported Patient Impact and Hospital Capacity by State Timeseries. Washington DC, United States of America: U.S. Department of Health and Human Services.
USA, Washington	Washington State Department of Health. United States - Washington State Department of Health COVID-19 Deaths by County, Date, Age Group and Sex. Olympia, United States of America: Washington State Department of Health.

USA, Washington	Washington State Department of Health. United States - Washington State Department of Health COVID-19 Cases by County and CDC Event Date.
USA, Washington	U.S. Department of Health and Human Services. United States - COVID-19 Reported Patient Impact and Hospital Capacity by Facility. Washington DC, United States of America: U.S. Department of Health and Human Services.
USA, Washington, DC	U.S. Department of Health and Human Services. United States - COVID-19 Reported Patient Impact and Hospital Capacity by State Timeseries. Washington DC, United States of America: U.S. Department of Health and Human Services.
USA, West Virginia	U.S. Department of Health and Human Services. United States - COVID-19 Reported Patient Impact and Hospital Capacity by State Timeseries. Washington DC, United States of America: U.S. Department of Health and Human Services.
USA, Wisconsin	U.S. Department of Health and Human Services. United States - COVID-19 Reported Patient Impact and Hospital Capacity by State Timeseries. Washington DC, United States of America: U.S. Department of Health and Human Services.
USA, Wyoming	U.S. Department of Health and Human Services. United States - COVID-19 Reported Patient Impact and Hospital Capacity by State Timeseries. Washington DC, United States of America: U.S. Department of Health and Human Services.
Virgin Islands	U.S. Department of Health and Human Services. United States - COVID-19 Reported Patient Impact and Hospital Capacity by State Timeseries. Washington DC, United States of America: U.S. Department of Health and Human Services.
Zambia	Ministry of Health (Zambia), National Public Health Institute (NPHI) (Zambia). Zambia National Public Health Institute Twitter Communications and COVID-19 Updates. Lusaka, Zambia: National Public Health Institute (NPHI) (Zambia).
Zimbabwe	Ministry of Health and Child Welfare (Zimbabwe). Zimbabwe COVID-19 Daily Update - Ministry of Health and Child Care Twitter.

Section 2: Deaths sources

Figure S2. Death data coverage by location

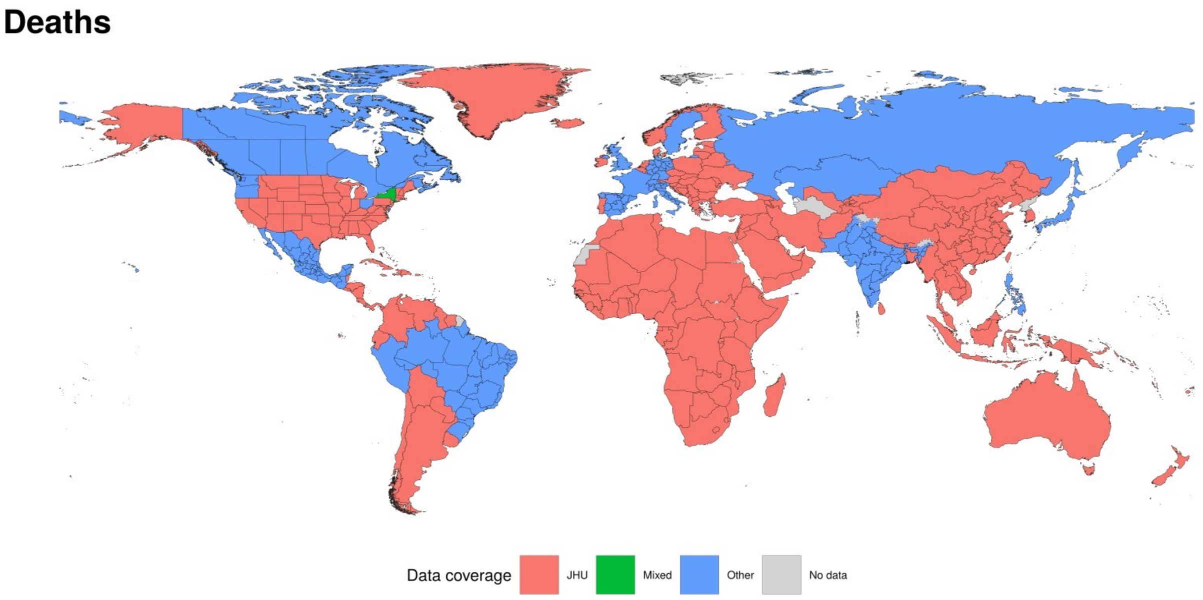

Table S2. Death data sources by location

Location	Source
Afghanistan	Johns Hopkins University. 2019 Novel Coronavirus COVID-19 (2019-nCoV) Data Repository by Johns Hopkins CSSE. Baltimore, Maryland: Johns Hopkins University.
Albania	Johns Hopkins University. 2019 Novel Coronavirus COVID-19 (2019-nCoV) Data Repository by Johns Hopkins CSSE. Baltimore, Maryland: Johns Hopkins University.
Algeria	Johns Hopkins University. 2019 Novel Coronavirus COVID-19 (2019-nCoV) Data Repository by Johns Hopkins CSSE. Baltimore, Maryland: Johns Hopkins University.
American Samoa	Johns Hopkins University. 2019 Novel Coronavirus COVID-19 (2019-nCoV) Data Repository by Johns Hopkins CSSE. Baltimore, Maryland: Johns Hopkins University.
Andorra	Johns Hopkins University. 2019 Novel Coronavirus COVID-19 (2019-nCoV) Data Repository by Johns Hopkins CSSE. Baltimore, Maryland: Johns Hopkins University.
Angola	Johns Hopkins University. 2019 Novel Coronavirus COVID-19 (2019-nCoV) Data Repository by Johns Hopkins CSSE. Baltimore, Maryland: Johns Hopkins University.
Antigua and Barbuda	Johns Hopkins University. 2019 Novel Coronavirus COVID-19 (2019-nCoV) Data Repository by Johns Hopkins CSSE. Baltimore, Maryland: Johns Hopkins University.
Argentina	Johns Hopkins University. 2019 Novel Coronavirus COVID-19 (2019-nCoV) Data Repository by Johns Hopkins CSSE. Baltimore, Maryland: Johns Hopkins University.
Armenia	Johns Hopkins University. 2019 Novel Coronavirus COVID-19 (2019-nCoV) Data Repository by Johns Hopkins CSSE. Baltimore, Maryland: Johns Hopkins University.
Australia	Johns Hopkins University. 2019 Novel Coronavirus COVID-19 (2019-nCoV) Data Repository by Johns Hopkins CSSE. Baltimore, Maryland: Johns Hopkins University.
Austria	Johns Hopkins University. 2019 Novel Coronavirus COVID-19 (2019-nCoV) Data Repository by Johns Hopkins CSSE. Baltimore, Maryland: Johns Hopkins University.
Azerbaijan	Johns Hopkins University. 2019 Novel Coronavirus COVID-19 (2019-nCoV) Data Repository by Johns Hopkins CSSE. Baltimore, Maryland: Johns Hopkins University.
Bahrain	Johns Hopkins University. 2019 Novel Coronavirus COVID-19 (2019-nCoV) Data Repository by Johns Hopkins CSSE. Baltimore, Maryland: Johns Hopkins University.
Bangladesh	Johns Hopkins University. 2019 Novel Coronavirus COVID-19 (2019-nCoV) Data Repository by Johns Hopkins CSSE. Baltimore, Maryland: Johns Hopkins University.
Barbados	Johns Hopkins University. 2019 Novel Coronavirus COVID-19 (2019-nCoV) Data Repository by Johns Hopkins CSSE. Baltimore, Maryland: Johns Hopkins University.
Belarus	Johns Hopkins University. 2019 Novel Coronavirus COVID-19 (2019-nCoV) Data Repository by Johns Hopkins CSSE. Baltimore, Maryland: Johns Hopkins University.
Belgium	Johns Hopkins University. 2019 Novel Coronavirus COVID-19 (2019-nCoV) Data Repository by Johns Hopkins CSSE. Baltimore, Maryland: Johns Hopkins University.

Belize	Johns Hopkins University. 2019 Novel Coronavirus COVID-19 (2019-nCoV) Data Repository by Johns Hopkins CSSE. Baltimore, Maryland: Johns Hopkins University.
Benin	Johns Hopkins University. 2019 Novel Coronavirus COVID-19 (2019-nCoV) Data Repository by Johns Hopkins CSSE. Baltimore, Maryland: Johns Hopkins University.
Bermuda	Johns Hopkins University. 2019 Novel Coronavirus COVID-19 (2019-nCoV) Data Repository by Johns Hopkins CSSE. Baltimore, Maryland: Johns Hopkins University.
Bhutan	Johns Hopkins University. 2019 Novel Coronavirus COVID-19 (2019-nCoV) Data Repository by Johns Hopkins CSSE. Baltimore, Maryland: Johns Hopkins University.
Bolivia	Johns Hopkins University. 2019 Novel Coronavirus COVID-19 (2019-nCoV) Data Repository by Johns Hopkins CSSE. Baltimore, Maryland: Johns Hopkins University.
Bosnia and Herzegovina	Johns Hopkins University. 2019 Novel Coronavirus COVID-19 (2019-nCoV) Data Repository by Johns Hopkins CSSE. Baltimore, Maryland: Johns Hopkins University.
Botswana	Johns Hopkins University. 2019 Novel Coronavirus COVID-19 (2019-nCoV) Data Repository by Johns Hopkins CSSE. Baltimore, Maryland: Johns Hopkins University.
Brazil, Acre	Ministry of Health (Brazil). Brazil Ministry of Health COVID-19 Coronavirus Panel. Rio de Janeiro, Brazil: Ministry of Health (Brazil).
Brazil, Alagoas	Ministry of Health (Brazil). Brazil Ministry of Health COVID-19 Coronavirus Panel. Rio de Janeiro, Brazil: Ministry of Health (Brazil).
Brazil, Amapá	Ministry of Health (Brazil). Brazil Ministry of Health COVID-19 Coronavirus Panel. Rio de Janeiro, Brazil: Ministry of Health (Brazil).
Brazil, Amazonas	Ministry of Health (Brazil). Brazil Ministry of Health COVID-19 Coronavirus Panel. Rio de Janeiro, Brazil: Ministry of Health (Brazil).
Brazil, Bahia	Ministry of Health (Brazil). Brazil Ministry of Health COVID-19 Coronavirus Panel. Rio de Janeiro, Brazil: Ministry of Health (Brazil).
Brazil, Ceará	Ministry of Health (Brazil). Brazil Ministry of Health COVID-19 Coronavirus Panel. Rio de Janeiro, Brazil: Ministry of Health (Brazil).
Brazil, Distrito Federal	Ministry of Health (Brazil). Brazil Ministry of Health COVID-19 Coronavirus Panel. Rio de Janeiro, Brazil: Ministry of Health (Brazil).
Brazil, Espírito Santo	Ministry of Health (Brazil). Brazil Ministry of Health COVID-19 Coronavirus Panel. Rio de Janeiro, Brazil: Ministry of Health (Brazil).
Brazil, Goiás	Ministry of Health (Brazil). Brazil Ministry of Health COVID-19 Coronavirus Panel. Rio de Janeiro, Brazil: Ministry of Health (Brazil).
Brazil, Maranhão	Ministry of Health (Brazil). Brazil Ministry of Health COVID-19 Coronavirus Panel. Rio de Janeiro, Brazil: Ministry of Health (Brazil).
Brazil, Mato Grosso	Ministry of Health (Brazil). Brazil Ministry of Health COVID-19 Coronavirus Panel. Rio de Janeiro, Brazil: Ministry of Health (Brazil).
Brazil, Mato Grosso do Sul	Ministry of Health (Brazil). Brazil Ministry of Health COVID-19 Coronavirus Panel. Rio de Janeiro, Brazil: Ministry of Health (Brazil).
Brazil, Minas Gerais	Ministry of Health (Brazil). Brazil Ministry of Health COVID-19 Coronavirus Panel. Rio de Janeiro, Brazil: Ministry of Health (Brazil).
Brazil, Pará	Ministry of Health (Brazil). Brazil Ministry of Health COVID-19 Coronavirus Panel. Rio de Janeiro, Brazil: Ministry of Health (Brazil).
Brazil, Paraíba	Ministry of Health (Brazil). Brazil Ministry of Health COVID-19 Coronavirus Panel. Rio de Janeiro, Brazil: Ministry of Health (Brazil).
Brazil, Paraná	Ministry of Health (Brazil). Brazil Ministry of Health COVID-19 Coronavirus Panel. Rio de Janeiro, Brazil: Ministry of Health (Brazil).
Brazil, Pernambuco	Ministry of Health (Brazil). Brazil Ministry of Health COVID-19 Coronavirus Panel. Rio de Janeiro, Brazil: Ministry of Health (Brazil).
Brazil, Piauí	Ministry of Health (Brazil). Brazil Ministry of Health COVID-19 Coronavirus Panel. Rio de Janeiro, Brazil: Ministry of Health (Brazil).
Brazil, Rio de Janeiro	Ministry of Health (Brazil). Brazil Ministry of Health COVID-19 Coronavirus Panel. Rio de Janeiro, Brazil: Ministry of Health (Brazil).
Brazil, Rio Grande do Norte	Ministry of Health (Brazil). Brazil Ministry of Health COVID-19 Coronavirus Panel. Rio de Janeiro, Brazil: Ministry of Health (Brazil).
Brazil, Rio Grande do Sul	Ministry of Health (Brazil). Brazil Ministry of Health COVID-19 Coronavirus Panel. Rio de Janeiro, Brazil: Ministry of Health (Brazil).
Brazil, Rondônia	Ministry of Health (Brazil). Brazil Ministry of Health COVID-19 Coronavirus Panel. Rio de Janeiro, Brazil: Ministry of Health (Brazil).
Brazil, Roraima	Ministry of Health (Brazil). Brazil Ministry of Health COVID-19 Coronavirus Panel. Rio de Janeiro, Brazil: Ministry of Health (Brazil).
Brazil, Santa Catarina	Ministry of Health (Brazil). Brazil Ministry of Health COVID-19 Coronavirus Panel. Rio de Janeiro, Brazil: Ministry of Health (Brazil).
Brazil, São Paulo	Ministry of Health (Brazil). Brazil Ministry of Health COVID-19 Coronavirus Panel. Rio de Janeiro, Brazil: Ministry of Health (Brazil).
Brazil, Sergipe	Ministry of Health (Brazil). Brazil Ministry of Health COVID-19 Coronavirus Panel. Rio de Janeiro, Brazil: Ministry of Health (Brazil).

Brazil, Tocantins	Ministry of Health (Brazil). Brazil Ministry of Health COVID-19 Coronavirus Panel. Rio de Janeiro, Brazil: Ministry of Health (Brazil).
Brunei	Johns Hopkins University. 2019 Novel Coronavirus COVID-19 (2019-nCoV) Data Repository by Johns Hopkins CSSE. Baltimore, Maryland: Johns Hopkins University.
Bulgaria	Johns Hopkins University. 2019 Novel Coronavirus COVID-19 (2019-nCoV) Data Repository by Johns Hopkins CSSE. Baltimore, Maryland: Johns Hopkins University.
Burkina Faso	Johns Hopkins University. 2019 Novel Coronavirus COVID-19 (2019-nCoV) Data Repository by Johns Hopkins CSSE. Baltimore, Maryland: Johns Hopkins University.
Burundi	Johns Hopkins University. 2019 Novel Coronavirus COVID-19 (2019-nCoV) Data Repository by Johns Hopkins CSSE. Baltimore, Maryland: Johns Hopkins University.
Cambodia	Johns Hopkins University. 2019 Novel Coronavirus COVID-19 (2019-nCoV) Data Repository by Johns Hopkins CSSE. Baltimore, Maryland: Johns Hopkins University.
Cameroon	Johns Hopkins University. 2019 Novel Coronavirus COVID-19 (2019-nCoV) Data Repository by Johns Hopkins CSSE. Baltimore, Maryland: Johns Hopkins University.
Canada, Alberta	Government of Canada. Canada Public Health Infobase Number of Total Cases of COVID-19. Ottawa, Canada: Government of Canada.
Canada, Alberta	Government of Canada. Canada Coronavirus Disease 2019 (COVID-19) Daily Epidemiology Update. Ottawa, Canada: Government of Canada.
Canada, British Columbia	Government of Canada. Canada Public Health Infobase Number of Total Cases of COVID-19. Ottawa, Canada: Government of Canada.
Canada, British Columbia	Government of Canada. Canada Coronavirus Disease 2019 (COVID-19) Daily Epidemiology Update. Ottawa, Canada: Government of Canada.
Canada, Manitoba	Government of Canada. Canada Public Health Infobase Number of Total Cases of COVID-19. Ottawa, Canada: Government of Canada.
Canada, Manitoba	Government of Canada. Canada Coronavirus Disease 2019 (COVID-19) Daily Epidemiology Update. Ottawa, Canada: Government of Canada.
Canada, New Brunswick	Government of Canada. Canada Public Health Infobase Number of Total Cases of COVID-19. Ottawa, Canada: Government of Canada.
Canada, New Brunswick	Government of Canada. Canada Coronavirus Disease 2019 (COVID-19) Daily Epidemiology Update. Ottawa, Canada: Government of Canada.
Canada, Newfoundland and Labrador	Government of Canada. Canada Public Health Infobase Number of Total Cases of COVID-19. Ottawa, Canada: Government of Canada.
Canada, Newfoundland and Labrador	Government of Canada. Canada Coronavirus Disease 2019 (COVID-19) Daily Epidemiology Update. Ottawa, Canada: Government of Canada.
Canada, Northwest Territories	Government of Canada. Canada Public Health Infobase Number of Total Cases of COVID-19. Ottawa, Canada: Government of Canada.
Canada, Northwest Territories	Government of Canada. Canada Coronavirus Disease 2019 (COVID-19) Daily Epidemiology Update. Ottawa, Canada: Government of Canada.
Canada, Nova Scotia	Government of Canada. Canada Public Health Infobase Number of Total Cases of COVID-19. Ottawa, Canada: Government of Canada.
Canada, Nova Scotia	Government of Canada. Canada Coronavirus Disease 2019 (COVID-19) Daily Epidemiology Update. Ottawa, Canada: Government of Canada.
Canada, Nunavut	Government of Canada. Canada Public Health Infobase Number of Total Cases of COVID-19. Ottawa, Canada: Government of Canada.
Canada, Nunavut	Government of Canada. Canada Coronavirus Disease 2019 (COVID-19) Daily Epidemiology Update. Ottawa, Canada: Government of Canada.
Canada, Ontario	Government of Canada. Canada Public Health Infobase Number of Total Cases of COVID-19. Ottawa, Canada: Government of Canada.
Canada, Ontario	Government of Canada. Canada Coronavirus Disease 2019 (COVID-19) Daily Epidemiology Update. Ottawa, Canada: Government of Canada.
Canada, Prince Edward Island	Government of Canada. Canada Public Health Infobase Number of Total Cases of COVID-19. Ottawa, Canada: Government of Canada.
Canada, Prince Edward Island	Government of Canada. Canada Coronavirus Disease 2019 (COVID-19) Daily Epidemiology Update. Ottawa, Canada: Government of Canada.
Canada, Quebec	Government of Canada. Canada Public Health Infobase Number of Total Cases of COVID-19. Ottawa, Canada: Government of Canada.
Canada, Quebec	Government of Canada. Canada Coronavirus Disease 2019 (COVID-19) Daily Epidemiology Update. Ottawa, Canada: Government of Canada.
Canada, Saskatchewan	Government of Canada. Canada Public Health Infobase Number of Total Cases of COVID-19. Ottawa, Canada: Government of Canada.
Canada, Saskatchewan	Government of Canada. Canada Coronavirus Disease 2019 (COVID-19) Daily Epidemiology Update. Ottawa, Canada: Government of Canada.
Canada, Yukon	Government of Canada. Canada Public Health Infobase Number of Total Cases of COVID-19. Ottawa, Canada: Government of Canada.
Canada, Yukon	Government of Canada. Canada Coronavirus Disease 2019 (COVID-19) Daily Epidemiology Update. Ottawa, Canada: Government of Canada.

Cape Verde	Johns Hopkins University. 2019 Novel Coronavirus COVID-19 (2019-nCoV) Data Repository by Johns Hopkins CSSE. Baltimore, Maryland: Johns Hopkins University.
Central African Republic	Johns Hopkins University. 2019 Novel Coronavirus COVID-19 (2019-nCoV) Data Repository by Johns Hopkins CSSE. Baltimore, Maryland: Johns Hopkins University.
Chad	Johns Hopkins University. 2019 Novel Coronavirus COVID-19 (2019-nCoV) Data Repository by Johns Hopkins CSSE. Baltimore, Maryland: Johns Hopkins University.
Chile	Johns Hopkins University. 2019 Novel Coronavirus COVID-19 (2019-nCoV) Data Repository by Johns Hopkins CSSE. Baltimore, Maryland: Johns Hopkins University.
China	Johns Hopkins University. 2019 Novel Coronavirus COVID-19 (2019-nCoV) Data Repository by Johns Hopkins CSSE. Baltimore, Maryland: Johns Hopkins University.
Colombia	Johns Hopkins University. 2019 Novel Coronavirus COVID-19 (2019-nCoV) Data Repository by Johns Hopkins CSSE. Baltimore, Maryland: Johns Hopkins University.
Comoros	Johns Hopkins University. 2019 Novel Coronavirus COVID-19 (2019-nCoV) Data Repository by Johns Hopkins CSSE. Baltimore, Maryland: Johns Hopkins University.
Congo (Brazzaville)	Johns Hopkins University. 2019 Novel Coronavirus COVID-19 (2019-nCoV) Data Repository by Johns Hopkins CSSE. Baltimore, Maryland: Johns Hopkins University.
Costa Rica	Johns Hopkins University. 2019 Novel Coronavirus COVID-19 (2019-nCoV) Data Repository by Johns Hopkins CSSE. Baltimore, Maryland: Johns Hopkins University.
Côte d'Ivoire	Johns Hopkins University. 2019 Novel Coronavirus COVID-19 (2019-nCoV) Data Repository by Johns Hopkins CSSE. Baltimore, Maryland: Johns Hopkins University.
Croatia	Johns Hopkins University. 2019 Novel Coronavirus COVID-19 (2019-nCoV) Data Repository by Johns Hopkins CSSE. Baltimore, Maryland: Johns Hopkins University.
Cuba	Johns Hopkins University. 2019 Novel Coronavirus COVID-19 (2019-nCoV) Data Repository by Johns Hopkins CSSE. Baltimore, Maryland: Johns Hopkins University.
Cyprus	Johns Hopkins University. 2019 Novel Coronavirus COVID-19 (2019-nCoV) Data Repository by Johns Hopkins CSSE. Baltimore, Maryland: Johns Hopkins University.
Czechia	Johns Hopkins University. 2019 Novel Coronavirus COVID-19 (2019-nCoV) Data Repository by Johns Hopkins CSSE. Baltimore, Maryland: Johns Hopkins University.
Denmark	Johns Hopkins University. 2019 Novel Coronavirus COVID-19 (2019-nCoV) Data Repository by Johns Hopkins CSSE. Baltimore, Maryland: Johns Hopkins University.
Djibouti	Johns Hopkins University. 2019 Novel Coronavirus COVID-19 (2019-nCoV) Data Repository by Johns Hopkins CSSE. Baltimore, Maryland: Johns Hopkins University.
Dominica	Johns Hopkins University. 2019 Novel Coronavirus COVID-19 (2019-nCoV) Data Repository by Johns Hopkins CSSE. Baltimore, Maryland: Johns Hopkins University.
Dominican Republic	Johns Hopkins University. 2019 Novel Coronavirus COVID-19 (2019-nCoV) Data Repository by Johns Hopkins CSSE. Baltimore, Maryland: Johns Hopkins University.
DR Congo	Johns Hopkins University. 2019 Novel Coronavirus COVID-19 (2019-nCoV) Data Repository by Johns Hopkins CSSE. Baltimore, Maryland: Johns Hopkins University.
Ecuador	Johns Hopkins University. 2019 Novel Coronavirus COVID-19 (2019-nCoV) Data Repository by Johns Hopkins CSSE. Baltimore, Maryland: Johns Hopkins University.
Egypt	Johns Hopkins University. 2019 Novel Coronavirus COVID-19 (2019-nCoV) Data Repository by Johns Hopkins CSSE. Baltimore, Maryland: Johns Hopkins University.
El Salvador	Johns Hopkins University. 2019 Novel Coronavirus COVID-19 (2019-nCoV) Data Repository by Johns Hopkins CSSE. Baltimore, Maryland: Johns Hopkins University.
Equatorial Guinea	Johns Hopkins University. 2019 Novel Coronavirus COVID-19 (2019-nCoV) Data Repository by Johns Hopkins CSSE. Baltimore, Maryland: Johns Hopkins University.
Eritrea	Johns Hopkins University. 2019 Novel Coronavirus COVID-19 (2019-nCoV) Data Repository by Johns Hopkins CSSE. Baltimore, Maryland: Johns Hopkins University.
Estonia	Johns Hopkins University. 2019 Novel Coronavirus COVID-19 (2019-nCoV) Data Repository by Johns Hopkins CSSE. Baltimore, Maryland: Johns Hopkins University.
Eswatini	Johns Hopkins University. 2019 Novel Coronavirus COVID-19 (2019-nCoV) Data Repository by Johns Hopkins CSSE. Baltimore, Maryland: Johns Hopkins University.
Ethiopia	Johns Hopkins University. 2019 Novel Coronavirus COVID-19 (2019-nCoV) Data Repository by Johns Hopkins CSSE. Baltimore, Maryland: Johns Hopkins University.
Federated States of Micronesia	Johns Hopkins University. 2019 Novel Coronavirus COVID-19 (2019-nCoV) Data Repository by Johns Hopkins CSSE. Baltimore, Maryland: Johns Hopkins University.
Fiji	Johns Hopkins University. 2019 Novel Coronavirus COVID-19 (2019-nCoV) Data Repository by Johns Hopkins CSSE. Baltimore, Maryland: Johns Hopkins University.
Finland	National Institute for Health and Welfare (THL) (Finland). Finland COVID-19 Cases in the Infectious Disease Registry. Helsinki, Finland: National Institute for Health and Welfare (THL) (Finland).
France	Etalab (France), Ministry of Health (France), Ministry of Health and Solidarity (DRESS) (France). France COVID-19 Epidemic Monitoring Dashboard.
Gabon	Johns Hopkins University. 2019 Novel Coronavirus COVID-19 (2019-nCoV) Data Repository by Johns Hopkins CSSE. Baltimore, Maryland: Johns Hopkins University.
Georgia	Johns Hopkins University. 2019 Novel Coronavirus COVID-19 (2019-nCoV) Data Repository by Johns Hopkins CSSE. Baltimore, Maryland: Johns Hopkins University.

Germany, Baden-Württemberg	Robert Koch Institute. Coronavirus Disease 2019 (COVID-19) Daily Situation Report - Robert Koch Institute. Berlin, Germany: Robert Koch Institute.
Germany, Baden-Württemberg	Wikipedia. COVID-19 pandemic in Germany. San Francisco, United States of America: Wikipedia.
Germany, Bavaria	Robert Koch Institute. Coronavirus Disease 2019 (COVID-19) Daily Situation Report - Robert Koch Institute. Berlin, Germany: Robert Koch Institute.
Germany, Bavaria	Wikipedia. COVID-19 pandemic in Germany. San Francisco, United States of America: Wikipedia.
Germany, Berlin	Robert Koch Institute. Coronavirus Disease 2019 (COVID-19) Daily Situation Report - Robert Koch Institute. Berlin, Germany: Robert Koch Institute.
Germany, Berlin	Wikipedia. COVID-19 pandemic in Germany. San Francisco, United States of America: Wikipedia.
Germany, Brandenburg	Robert Koch Institute. Coronavirus Disease 2019 (COVID-19) Daily Situation Report - Robert Koch Institute. Berlin, Germany: Robert Koch Institute.
Germany, Brandenburg	Wikipedia. COVID-19 pandemic in Germany. San Francisco, United States of America: Wikipedia.
Germany, Bremen	Robert Koch Institute. Coronavirus Disease 2019 (COVID-19) Daily Situation Report - Robert Koch Institute. Berlin, Germany: Robert Koch Institute.
Germany, Bremen	Wikipedia. COVID-19 pandemic in Germany. San Francisco, United States of America: Wikipedia.
Germany, Hamburg	Robert Koch Institute. Coronavirus Disease 2019 (COVID-19) Daily Situation Report - Robert Koch Institute. Berlin, Germany: Robert Koch Institute.
Germany, Hamburg	Wikipedia. COVID-19 pandemic in Germany. San Francisco, United States of America: Wikipedia.
Germany, Hesse	Robert Koch Institute. Coronavirus Disease 2019 (COVID-19) Daily Situation Report - Robert Koch Institute. Berlin, Germany: Robert Koch Institute.
Germany, Hesse	Wikipedia. COVID-19 pandemic in Germany. San Francisco, United States of America: Wikipedia.
Germany, Lower Saxony	Robert Koch Institute. Coronavirus Disease 2019 (COVID-19) Daily Situation Report - Robert Koch Institute. Berlin, Germany: Robert Koch Institute.
Germany, Lower Saxony	Wikipedia. COVID-19 pandemic in Germany. San Francisco, United States of America: Wikipedia.
Germany, Mecklenburg-Vorpommern	Robert Koch Institute. Coronavirus Disease 2019 (COVID-19) Daily Situation Report - Robert Koch Institute. Berlin, Germany: Robert Koch Institute.
Germany, Mecklenburg-Vorpommern	Wikipedia. COVID-19 pandemic in Germany. San Francisco, United States of America: Wikipedia.
Germany, North Rhine-Westphalia	Robert Koch Institute. Coronavirus Disease 2019 (COVID-19) Daily Situation Report - Robert Koch Institute. Berlin, Germany: Robert Koch Institute.
Germany, North Rhine-Westphalia	Wikipedia. COVID-19 pandemic in Germany. San Francisco, United States of America: Wikipedia.
Germany, Rhineland-Palatinate	Robert Koch Institute. Coronavirus Disease 2019 (COVID-19) Daily Situation Report - Robert Koch Institute. Berlin, Germany: Robert Koch Institute.
Germany, Rhineland-Palatinate	Wikipedia. COVID-19 pandemic in Germany. San Francisco, United States of America: Wikipedia.
Germany, Saarland	Robert Koch Institute. Coronavirus Disease 2019 (COVID-19) Daily Situation Report - Robert Koch Institute. Berlin, Germany: Robert Koch Institute.
Germany, Saarland	Wikipedia. COVID-19 pandemic in Germany. San Francisco, United States of America: Wikipedia.
Germany, Saxony	Robert Koch Institute. Coronavirus Disease 2019 (COVID-19) Daily Situation Report - Robert Koch Institute. Berlin, Germany: Robert Koch Institute.
Germany, Saxony	Wikipedia. COVID-19 pandemic in Germany. San Francisco, United States of America: Wikipedia.
Germany, Saxony-Anhalt	Robert Koch Institute. Coronavirus Disease 2019 (COVID-19) Daily Situation Report - Robert Koch Institute. Berlin, Germany: Robert Koch Institute.
Germany, Saxony-Anhalt	Wikipedia. COVID-19 pandemic in Germany. San Francisco, United States of America: Wikipedia.
Germany, Schleswig-Holstein	Robert Koch Institute. Coronavirus Disease 2019 (COVID-19) Daily Situation Report - Robert Koch Institute. Berlin, Germany: Robert Koch Institute.
Germany, Schleswig-Holstein	Wikipedia. COVID-19 pandemic in Germany. San Francisco, United States of America: Wikipedia.
Germany, Thuringia	Robert Koch Institute. Coronavirus Disease 2019 (COVID-19) Daily Situation Report - Robert Koch Institute. Berlin, Germany: Robert Koch Institute.
Germany, Thuringia	Wikipedia. COVID-19 pandemic in Germany. San Francisco, United States of America: Wikipedia.
Ghana	Johns Hopkins University. 2019 Novel Coronavirus COVID-19 (2019-nCoV) Data Repository by Johns Hopkins CSSE. Baltimore, Maryland: Johns Hopkins University.
Greece	Johns Hopkins University. 2019 Novel Coronavirus COVID-19 (2019-nCoV) Data Repository by Johns Hopkins CSSE. Baltimore, Maryland: Johns Hopkins University.

Greenland	Johns Hopkins University. 2019 Novel Coronavirus COVID-19 (2019-nCoV) Data Repository by Johns Hopkins CSSE. Baltimore, Maryland: Johns Hopkins University.
Grenada	Johns Hopkins University. 2019 Novel Coronavirus COVID-19 (2019-nCoV) Data Repository by Johns Hopkins CSSE. Baltimore, Maryland: Johns Hopkins University.
Guam	Johns Hopkins University. 2019 Novel Coronavirus COVID-19 (2019-nCoV) Data Repository by Johns Hopkins CSSE. Baltimore, Maryland: Johns Hopkins University.
Guatemala	Ministry of Public Health and Social Assistance (Guatemala). Guatemala COVID-19 Situation Deaths. Guatemala City, Guatemala: Ministry of Public Health and Social Assistance (Guatemala).
Guinea	Johns Hopkins University. 2019 Novel Coronavirus COVID-19 (2019-nCoV) Data Repository by Johns Hopkins CSSE. Baltimore, Maryland: Johns Hopkins University.
Guinea-Bissau	Johns Hopkins University. 2019 Novel Coronavirus COVID-19 (2019-nCoV) Data Repository by Johns Hopkins CSSE. Baltimore, Maryland: Johns Hopkins University.
Guyana	Johns Hopkins University. 2019 Novel Coronavirus COVID-19 (2019-nCoV) Data Repository by Johns Hopkins CSSE. Baltimore, Maryland: Johns Hopkins University.
Haiti	Johns Hopkins University. 2019 Novel Coronavirus COVID-19 (2019-nCoV) Data Repository by Johns Hopkins CSSE. Baltimore, Maryland: Johns Hopkins University.
Honduras	Johns Hopkins University. 2019 Novel Coronavirus COVID-19 (2019-nCoV) Data Repository by Johns Hopkins CSSE. Baltimore, Maryland: Johns Hopkins University.
Hungary	Johns Hopkins University. 2019 Novel Coronavirus COVID-19 (2019-nCoV) Data Repository by Johns Hopkins CSSE. Baltimore, Maryland: Johns Hopkins University.
Iceland	Johns Hopkins University. 2019 Novel Coronavirus COVID-19 (2019-nCoV) Data Repository by Johns Hopkins CSSE. Baltimore, Maryland: Johns Hopkins University.
India, Andhra Pradesh	COVID-19 India. India COVID-19 Crowdsourced Patient Database: National Level Time Series, State-Wise Stats and Test Counts. India: COVID-19 India.
India, Andhra Pradesh	COVID-19 India. India COVID-19 Crowdsourced Patient Database: State Level Daily Changes. India: COVID-19 India.
India, Arunachal Pradesh	COVID-19 India. India COVID-19 Crowdsourced Patient Database: National Level Time Series, State-Wise Stats and Test Counts. India: COVID-19 India.
India, Arunachal Pradesh	COVID-19 India. India COVID-19 Crowdsourced Patient Database: State Level Daily Changes. India: COVID-19 India.
India, Assam	COVID-19 India. India COVID-19 Crowdsourced Patient Database: National Level Time Series, State-Wise Stats and Test Counts. India: COVID-19 India.
India, Assam	COVID-19 India. India COVID-19 Crowdsourced Patient Database: State Level Daily Changes. India: COVID-19 India.
India, Bihar	COVID-19 India. India COVID-19 Crowdsourced Patient Database: National Level Time Series, State-Wise Stats and Test Counts. India: COVID-19 India.
India, Bihar	COVID-19 India. India COVID-19 Crowdsourced Patient Database: State Level Daily Changes. India: COVID-19 India.
India, Chhattisgarh	COVID-19 India. India COVID-19 Crowdsourced Patient Database: National Level Time Series, State-Wise Stats and Test Counts. India: COVID-19 India.
India, Chhattisgarh	COVID-19 India. India COVID-19 Crowdsourced Patient Database: State Level Daily Changes. India: COVID-19 India.
India, Dadra and Nagar Haveli and Daman and Diu	COVID-19 India. India COVID-19 Crowdsourced Patient Database: National Level Time Series, State-Wise Stats and Test Counts. India: COVID-19 India.
India, Dadra and Nagar Haveli and Daman and Diu	COVID-19 India. India COVID-19 Crowdsourced Patient Database: State Level Daily Changes. India: COVID-19 India.
India, Delhi	COVID-19 India. India COVID-19 Crowdsourced Patient Database: National Level Time Series, State-Wise Stats and Test Counts. India: COVID-19 India.
India, Delhi	COVID-19 India. India COVID-19 Crowdsourced Patient Database: State Level Daily Changes. India: COVID-19 India.
India, Goa	COVID-19 India. India COVID-19 Crowdsourced Patient Database: National Level Time Series, State-Wise Stats and Test Counts. India: COVID-19 India.
India, Goa	COVID-19 India. India COVID-19 Crowdsourced Patient Database: State Level Daily Changes. India: COVID-19 India.
India, Gujarat	COVID-19 India. India COVID-19 Crowdsourced Patient Database: National Level Time Series, State-Wise Stats and Test Counts. India: COVID-19 India.
India, Gujarat	COVID-19 India. India COVID-19 Crowdsourced Patient Database: State Level Daily Changes. India: COVID-19 India.
India, Haryana	COVID-19 India. India COVID-19 Crowdsourced Patient Database: National Level Time Series, State-Wise Stats and Test Counts. India: COVID-19 India.
India, Haryana	COVID-19 India. India COVID-19 Crowdsourced Patient Database: State Level Daily Changes. India: COVID-19 India.
India, Himachal Pradesh	COVID-19 India. India COVID-19 Crowdsourced Patient Database: National Level Time Series, State-Wise Stats and Test Counts. India: COVID-19 India.

India, Himachal Pradesh	COVID-19 India. India COVID-19 Crowdsourced Patient Database: State Level Daily Changes. India: COVID-19 India.
India, Jammu & Kashmir and Ladakh	COVID-19 India. India COVID-19 Crowdsourced Patient Database: National Level Time Series, State-Wise Stats and Test Counts. India: COVID-19 India.
India, Jammu & Kashmir and Ladakh	COVID-19 India. India COVID-19 Crowdsourced Patient Database: State Level Daily Changes. India: COVID-19 India.
India, Jharkhand	COVID-19 India. India COVID-19 Crowdsourced Patient Database: National Level Time Series, State-Wise Stats and Test Counts. India: COVID-19 India.
India, Jharkhand	COVID-19 India. India COVID-19 Crowdsourced Patient Database: State Level Daily Changes. India: COVID-19 India.
India, Karnataka	COVID-19 India. India COVID-19 Crowdsourced Patient Database: National Level Time Series, State-Wise Stats and Test Counts. India: COVID-19 India.
India, Karnataka	COVID-19 India. India COVID-19 Crowdsourced Patient Database: State Level Daily Changes. India: COVID-19 India.
India, Kerala	COVID-19 India. India COVID-19 Crowdsourced Patient Database: National Level Time Series, State-Wise Stats and Test Counts. India: COVID-19 India.
India, Kerala	COVID-19 India. India COVID-19 Crowdsourced Patient Database: State Level Daily Changes. India: COVID-19 India.
India, Madhya Pradesh	COVID-19 India. India COVID-19 Crowdsourced Patient Database: National Level Time Series, State-Wise Stats and Test Counts. India: COVID-19 India.
India, Madhya Pradesh	COVID-19 India. India COVID-19 Crowdsourced Patient Database: State Level Daily Changes. India: COVID-19 India.
India, Maharashtra	COVID-19 India. India COVID-19 Crowdsourced Patient Database: National Level Time Series, State-Wise Stats and Test Counts. India: COVID-19 India.
India, Maharashtra	COVID-19 India. India COVID-19 Crowdsourced Patient Database: State Level Daily Changes. India: COVID-19 India.
India, Manipur	COVID-19 India. India COVID-19 Crowdsourced Patient Database: National Level Time Series, State-Wise Stats and Test Counts. India: COVID-19 India.
India, Manipur	COVID-19 India. India COVID-19 Crowdsourced Patient Database: State Level Daily Changes. India: COVID-19 India.
India, Meghalaya	COVID-19 India. India COVID-19 Crowdsourced Patient Database: National Level Time Series, State-Wise Stats and Test Counts. India: COVID-19 India.
India, Meghalaya	COVID-19 India. India COVID-19 Crowdsourced Patient Database: State Level Daily Changes. India: COVID-19 India.
India, Mizoram	COVID-19 India. India COVID-19 Crowdsourced Patient Database: National Level Time Series, State-Wise Stats and Test Counts. India: COVID-19 India.
India, Mizoram	COVID-19 India. India COVID-19 Crowdsourced Patient Database: State Level Daily Changes. India: COVID-19 India.
India, Nagaland	COVID-19 India. India COVID-19 Crowdsourced Patient Database: National Level Time Series, State-Wise Stats and Test Counts. India: COVID-19 India.
India, Nagaland	COVID-19 India. India COVID-19 Crowdsourced Patient Database: State Level Daily Changes. India: COVID-19 India.
India, Odisha	COVID-19 India. India COVID-19 Crowdsourced Patient Database: National Level Time Series, State-Wise Stats and Test Counts. India: COVID-19 India.
India, Odisha	COVID-19 India. India COVID-19 Crowdsourced Patient Database: State Level Daily Changes. India: COVID-19 India.
India, Punjab	COVID-19 India. India COVID-19 Crowdsourced Patient Database: National Level Time Series, State-Wise Stats and Test Counts. India: COVID-19 India.
India, Punjab	COVID-19 India. India COVID-19 Crowdsourced Patient Database: State Level Daily Changes. India: COVID-19 India.
India, Rajasthan	COVID-19 India. India COVID-19 Crowdsourced Patient Database: National Level Time Series, State-Wise Stats and Test Counts. India: COVID-19 India.
India, Rajasthan	COVID-19 India. India COVID-19 Crowdsourced Patient Database: State Level Daily Changes. India: COVID-19 India.
India, Sikkim	COVID-19 India. India COVID-19 Crowdsourced Patient Database: National Level Time Series, State-Wise Stats and Test Counts. India: COVID-19 India.
India, Sikkim	COVID-19 India. India COVID-19 Crowdsourced Patient Database: State Level Daily Changes. India: COVID-19 India.
India, Tamil Nadu	COVID-19 India. India COVID-19 Crowdsourced Patient Database: National Level Time Series, State-Wise Stats and Test Counts. India: COVID-19 India.
India, Tamil Nadu	COVID-19 India. India COVID-19 Crowdsourced Patient Database: State Level Daily Changes. India: COVID-19 India.
India, Telangana	COVID-19 India. India COVID-19 Crowdsourced Patient Database: National Level Time Series, State-Wise Stats and Test Counts. India: COVID-19 India.
India, Telangana	COVID-19 India. India COVID-19 Crowdsourced Patient Database: State Level Daily Changes. India: COVID-19 India.

India, Tripura	COVID-19 India. India COVID-19 Crowdsourced Patient Database: National Level Time Series, State-Wise Stats and Test Counts. India: COVID-19 India.
India, Tripura	COVID-19 India. India COVID-19 Crowdsourced Patient Database: State Level Daily Changes. India: COVID-19 India.
India, Uttar Pradesh	COVID-19 India. India COVID-19 Crowdsourced Patient Database: National Level Time Series, State-Wise Stats and Test Counts. India: COVID-19 India.
India, Uttar Pradesh	COVID-19 India. India COVID-19 Crowdsourced Patient Database: State Level Daily Changes. India: COVID-19 India.
India, Uttarakhand	COVID-19 India. India COVID-19 Crowdsourced Patient Database: National Level Time Series, State-Wise Stats and Test Counts. India: COVID-19 India.
India, Uttarakhand	COVID-19 India. India COVID-19 Crowdsourced Patient Database: State Level Daily Changes. India: COVID-19 India.
India, West Bengal	COVID-19 India. India COVID-19 Crowdsourced Patient Database: National Level Time Series, State-Wise Stats and Test Counts. India: COVID-19 India.
India, West Bengal	COVID-19 India. India COVID-19 Crowdsourced Patient Database: State Level Daily Changes. India: COVID-19 India.
Indonesia	Johns Hopkins University. 2019 Novel Coronavirus COVID-19 (2019-nCoV) Data Repository by Johns Hopkins CSSE. Baltimore, Maryland: Johns Hopkins University.
Iran	Johns Hopkins University. 2019 Novel Coronavirus COVID-19 (2019-nCoV) Data Repository by Johns Hopkins CSSE. Baltimore, Maryland: Johns Hopkins University.
Iraq	Johns Hopkins University. 2019 Novel Coronavirus COVID-19 (2019-nCoV) Data Repository by Johns Hopkins CSSE. Baltimore, Maryland: Johns Hopkins University.
Ireland	Johns Hopkins University. 2019 Novel Coronavirus COVID-19 (2019-nCoV) Data Repository by Johns Hopkins CSSE. Baltimore, Maryland: Johns Hopkins University.
Israel	Johns Hopkins University. 2019 Novel Coronavirus COVID-19 (2019-nCoV) Data Repository by Johns Hopkins CSSE. Baltimore, Maryland: Johns Hopkins University.
Italy, Abruzzo	Department of Civil Protection (Italy). Italy COVID-19 Situation Monitoring - Department of Civil Protection. Rome, Italy: Department of Civil Protection (Italy).
Italy, Basilicata	Department of Civil Protection (Italy). Italy COVID-19 Situation Monitoring - Department of Civil Protection. Rome, Italy: Department of Civil Protection (Italy).
Italy, Calabria	Department of Civil Protection (Italy). Italy COVID-19 Situation Monitoring - Department of Civil Protection. Rome, Italy: Department of Civil Protection (Italy).
Italy, Campania	Department of Civil Protection (Italy). Italy COVID-19 Situation Monitoring - Department of Civil Protection. Rome, Italy: Department of Civil Protection (Italy).
Italy, Emilia-Romagna	Department of Civil Protection (Italy). Italy COVID-19 Situation Monitoring - Department of Civil Protection. Rome, Italy: Department of Civil Protection (Italy).
Italy, Friuli-Venezia Giulia	Department of Civil Protection (Italy). Italy COVID-19 Situation Monitoring - Department of Civil Protection. Rome, Italy: Department of Civil Protection (Italy).
Italy, Lazio	Department of Civil Protection (Italy). Italy COVID-19 Situation Monitoring - Department of Civil Protection. Rome, Italy: Department of Civil Protection (Italy).
Italy, Liguria	Department of Civil Protection (Italy). Italy COVID-19 Situation Monitoring - Department of Civil Protection. Rome, Italy: Department of Civil Protection (Italy).
Italy, Lombardia	Department of Civil Protection (Italy). Italy COVID-19 Situation Monitoring - Department of Civil Protection. Rome, Italy: Department of Civil Protection (Italy).
Italy, Marche	Department of Civil Protection (Italy). Italy COVID-19 Situation Monitoring - Department of Civil Protection. Rome, Italy: Department of Civil Protection (Italy).
Italy, Molise	Department of Civil Protection (Italy). Italy COVID-19 Situation Monitoring - Department of Civil Protection. Rome, Italy: Department of Civil Protection (Italy).
Italy, Piemonte	Department of Civil Protection (Italy). Italy COVID-19 Situation Monitoring - Department of Civil Protection. Rome, Italy: Department of Civil Protection (Italy).
Italy, Prov. autonoma di Bolzano	Department of Civil Protection (Italy). Italy COVID-19 Situation Monitoring - Department of Civil Protection. Rome, Italy: Department of Civil Protection (Italy).
Italy, Prov. autonoma di Trento	Department of Civil Protection (Italy). Italy COVID-19 Situation Monitoring - Department of Civil Protection. Rome, Italy: Department of Civil Protection (Italy).
Italy, Puglia	Department of Civil Protection (Italy). Italy COVID-19 Situation Monitoring - Department of Civil Protection. Rome, Italy: Department of Civil Protection (Italy).
Italy, Sardegna	Department of Civil Protection (Italy). Italy COVID-19 Situation Monitoring - Department of Civil Protection. Rome, Italy: Department of Civil Protection (Italy).
Italy, Sicilia	Department of Civil Protection (Italy). Italy COVID-19 Situation Monitoring - Department of Civil Protection. Rome, Italy: Department of Civil Protection (Italy).
Italy, Toscana	Department of Civil Protection (Italy). Italy COVID-19 Situation Monitoring - Department of Civil Protection. Rome, Italy: Department of Civil Protection (Italy).
Italy, Umbria	Department of Civil Protection (Italy). Italy COVID-19 Situation Monitoring - Department of Civil Protection. Rome, Italy: Department of Civil Protection (Italy).
Italy, Valle d'Aosta	Department of Civil Protection (Italy). Italy COVID-19 Situation Monitoring - Department of Civil Protection. Rome, Italy: Department of Civil Protection (Italy).

Italy, Veneto	Department of Civil Protection (Italy). Italy COVID-19 Situation Monitoring - Department of Civil Protection. Rome, Italy: Department of Civil Protection (Italy).
Jamaica	Johns Hopkins University. 2019 Novel Coronavirus COVID-19 (2019-nCoV) Data Repository by Johns Hopkins CSSE. Baltimore, Maryland: Johns Hopkins University.
Japan	Ministry of Health, Labour and Welfare (Japan). Japan Coronavirus Disease (COVID-19) Situation Report.
Jordan	Johns Hopkins University. 2019 Novel Coronavirus COVID-19 (2019-nCoV) Data Repository by Johns Hopkins CSSE. Baltimore, Maryland: Johns Hopkins University.
Kazakhstan	*
Kenya	Johns Hopkins University. 2019 Novel Coronavirus COVID-19 (2019-nCoV) Data Repository by Johns Hopkins CSSE. Baltimore, Maryland: Johns Hopkins University.
Kiribati	Johns Hopkins University. 2019 Novel Coronavirus COVID-19 (2019-nCoV) Data Repository by Johns Hopkins CSSE. Baltimore, Maryland: Johns Hopkins University.
Kuwait	Johns Hopkins University. 2019 Novel Coronavirus COVID-19 (2019-nCoV) Data Repository by Johns Hopkins CSSE. Baltimore, Maryland: Johns Hopkins University.
Kyrgyzstan	Johns Hopkins University. 2019 Novel Coronavirus COVID-19 (2019-nCoV) Data Repository by Johns Hopkins CSSE. Baltimore, Maryland: Johns Hopkins University.
Laos	Johns Hopkins University. 2019 Novel Coronavirus COVID-19 (2019-nCoV) Data Repository by Johns Hopkins CSSE. Baltimore, Maryland: Johns Hopkins University.
Latvia	Johns Hopkins University. 2019 Novel Coronavirus COVID-19 (2019-nCoV) Data Repository by Johns Hopkins CSSE. Baltimore, Maryland: Johns Hopkins University.
Lebanon	Johns Hopkins University. 2019 Novel Coronavirus COVID-19 (2019-nCoV) Data Repository by Johns Hopkins CSSE. Baltimore, Maryland: Johns Hopkins University.
Lesotho	Johns Hopkins University. 2019 Novel Coronavirus COVID-19 (2019-nCoV) Data Repository by Johns Hopkins CSSE. Baltimore, Maryland: Johns Hopkins University.
Liberia	Johns Hopkins University. 2019 Novel Coronavirus COVID-19 (2019-nCoV) Data Repository by Johns Hopkins CSSE. Baltimore, Maryland: Johns Hopkins University.
Libya	Johns Hopkins University. 2019 Novel Coronavirus COVID-19 (2019-nCoV) Data Repository by Johns Hopkins CSSE. Baltimore, Maryland: Johns Hopkins University.
Lithuania	Johns Hopkins University. 2019 Novel Coronavirus COVID-19 (2019-nCoV) Data Repository by Johns Hopkins CSSE. Baltimore, Maryland: Johns Hopkins University.
Luxembourg	Johns Hopkins University. 2019 Novel Coronavirus COVID-19 (2019-nCoV) Data Repository by Johns Hopkins CSSE. Baltimore, Maryland: Johns Hopkins University.
Madagascar	Johns Hopkins University. 2019 Novel Coronavirus COVID-19 (2019-nCoV) Data Repository by Johns Hopkins CSSE. Baltimore, Maryland: Johns Hopkins University.
Malawi	Johns Hopkins University. 2019 Novel Coronavirus COVID-19 (2019-nCoV) Data Repository by Johns Hopkins CSSE. Baltimore, Maryland: Johns Hopkins University.
Malaysia	Johns Hopkins University. 2019 Novel Coronavirus COVID-19 (2019-nCoV) Data Repository by Johns Hopkins CSSE. Baltimore, Maryland: Johns Hopkins University.
Maldives	Johns Hopkins University. 2019 Novel Coronavirus COVID-19 (2019-nCoV) Data Repository by Johns Hopkins CSSE. Baltimore, Maryland: Johns Hopkins University.
Mali	Johns Hopkins University. 2019 Novel Coronavirus COVID-19 (2019-nCoV) Data Repository by Johns Hopkins CSSE. Baltimore, Maryland: Johns Hopkins University.
Malta	Johns Hopkins University. 2019 Novel Coronavirus COVID-19 (2019-nCoV) Data Repository by Johns Hopkins CSSE. Baltimore, Maryland: Johns Hopkins University.
Marshall Islands	Johns Hopkins University. 2019 Novel Coronavirus COVID-19 (2019-nCoV) Data Repository by Johns Hopkins CSSE. Baltimore, Maryland: Johns Hopkins University.
Mauritania	Johns Hopkins University. 2019 Novel Coronavirus COVID-19 (2019-nCoV) Data Repository by Johns Hopkins CSSE. Baltimore, Maryland: Johns Hopkins University.
Mauritius	Johns Hopkins University. 2019 Novel Coronavirus COVID-19 (2019-nCoV) Data Repository by Johns Hopkins CSSE. Baltimore, Maryland: Johns Hopkins University.
Mexico, Aguascalientes	Directorate General of Epidemiology, Secretariat of Health (Mexico), National Institute of Statistics and Geography (INEGI) (Mexico). Mexico COVID-19 Deaths 2020.
Mexico, Baja California	Directorate General of Epidemiology, Secretariat of Health (Mexico), National Institute of Statistics and Geography (INEGI) (Mexico). Mexico COVID-19 Deaths 2020.
Mexico, Baja California Sur	Directorate General of Epidemiology, Secretariat of Health (Mexico), National Institute of Statistics and Geography (INEGI) (Mexico). Mexico COVID-19 Deaths 2020.
Mexico, Campeche	Directorate General of Epidemiology, Secretariat of Health (Mexico), National Institute of Statistics and Geography (INEGI) (Mexico). Mexico COVID-19 Deaths 2020.
Mexico, Chiapas	Directorate General of Epidemiology, Secretariat of Health (Mexico), National Institute of Statistics and Geography (INEGI) (Mexico). Mexico COVID-19 Deaths 2020.
Mexico, Chihuahua	Directorate General of Epidemiology, Secretariat of Health (Mexico), National Institute of Statistics and Geography (INEGI) (Mexico). Mexico COVID-19 Deaths 2020.
Mexico, Coahuila	Directorate General of Epidemiology, Secretariat of Health (Mexico), National Institute of Statistics and Geography (INEGI) (Mexico). Mexico COVID-19 Deaths 2020.
Mexico, Colima	Directorate General of Epidemiology, Secretariat of Health (Mexico), National Institute of Statistics and Geography (INEGI) (Mexico). Mexico COVID-19 Deaths 2020.

Mexico, Durango	Directorate General of Epidemiology, Secretariat of Health (Mexico), National Institute of Statistics and Geography (INEGI) (Mexico). Mexico COVID-19 Deaths 2020.
Mexico, Guanajuato	Directorate General of Epidemiology, Secretariat of Health (Mexico), National Institute of Statistics and Geography (INEGI) (Mexico). Mexico COVID-19 Deaths 2020.
Mexico, Guerrero	Directorate General of Epidemiology, Secretariat of Health (Mexico), National Institute of Statistics and Geography (INEGI) (Mexico). Mexico COVID-19 Deaths 2020.
Mexico, Hidalgo	Directorate General of Epidemiology, Secretariat of Health (Mexico), National Institute of Statistics and Geography (INEGI) (Mexico). Mexico COVID-19 Deaths 2020.
Mexico, Jalisco	Directorate General of Epidemiology, Secretariat of Health (Mexico), National Institute of Statistics and Geography (INEGI) (Mexico). Mexico COVID-19 Deaths 2020.
Mexico, México	Directorate General of Epidemiology, Secretariat of Health (Mexico), National Institute of Statistics and Geography (INEGI) (Mexico). Mexico COVID-19 Deaths 2020.
Mexico, Mexico City	Directorate General of Epidemiology, Secretariat of Health (Mexico), National Institute of Statistics and Geography (INEGI) (Mexico). Mexico COVID-19 Deaths 2020.
Mexico, Michoacán de Ocampo	Directorate General of Epidemiology, Secretariat of Health (Mexico), National Institute of Statistics and Geography (INEGI) (Mexico). Mexico COVID-19 Deaths 2020.
Mexico, Morelos	Directorate General of Epidemiology, Secretariat of Health (Mexico), National Institute of Statistics and Geography (INEGI) (Mexico). Mexico COVID-19 Deaths 2020.
Mexico, Nayarit	Directorate General of Epidemiology, Secretariat of Health (Mexico), National Institute of Statistics and Geography (INEGI) (Mexico). Mexico COVID-19 Deaths 2020.
Mexico, Nuevo León	Directorate General of Epidemiology, Secretariat of Health (Mexico), National Institute of Statistics and Geography (INEGI) (Mexico). Mexico COVID-19 Deaths 2020.
Mexico, Oaxaca	Directorate General of Epidemiology, Secretariat of Health (Mexico), National Institute of Statistics and Geography (INEGI) (Mexico). Mexico COVID-19 Deaths 2020.
Mexico, Puebla	Directorate General of Epidemiology, Secretariat of Health (Mexico), National Institute of Statistics and Geography (INEGI) (Mexico). Mexico COVID-19 Deaths 2020.
Mexico, Querétaro	Directorate General of Epidemiology, Secretariat of Health (Mexico), National Institute of Statistics and Geography (INEGI) (Mexico). Mexico COVID-19 Deaths 2020.
Mexico, Quintana Roo	Directorate General of Epidemiology, Secretariat of Health (Mexico), National Institute of Statistics and Geography (INEGI) (Mexico). Mexico COVID-19 Deaths 2020.
Mexico, San Luis Potosí	Directorate General of Epidemiology, Secretariat of Health (Mexico), National Institute of Statistics and Geography (INEGI) (Mexico). Mexico COVID-19 Deaths 2020.
Mexico, Sinaloa	Directorate General of Epidemiology, Secretariat of Health (Mexico), National Institute of Statistics and Geography (INEGI) (Mexico). Mexico COVID-19 Deaths 2020.
Mexico, Sonora	Directorate General of Epidemiology, Secretariat of Health (Mexico), National Institute of Statistics and Geography (INEGI) (Mexico). Mexico COVID-19 Deaths 2020.
Mexico, Tabasco	Directorate General of Epidemiology, Secretariat of Health (Mexico), National Institute of Statistics and Geography (INEGI) (Mexico). Mexico COVID-19 Deaths 2020.
Mexico, Tamaulipas	Directorate General of Epidemiology, Secretariat of Health (Mexico), National Institute of Statistics and Geography (INEGI) (Mexico). Mexico COVID-19 Deaths 2020.
Mexico, Tlaxcala	Directorate General of Epidemiology, Secretariat of Health (Mexico), National Institute of Statistics and Geography (INEGI) (Mexico). Mexico COVID-19 Deaths 2020.
Mexico, Veracruz de Ignacio de la Llave	Directorate General of Epidemiology, Secretariat of Health (Mexico), National Institute of Statistics and Geography (INEGI) (Mexico). Mexico COVID-19 Deaths 2020.
Mexico, Yucatán	Directorate General of Epidemiology, Secretariat of Health (Mexico), National Institute of Statistics and Geography (INEGI) (Mexico). Mexico COVID-19 Deaths 2020.
Mexico, Zacatecas	Directorate General of Epidemiology, Secretariat of Health (Mexico), National Institute of Statistics and Geography (INEGI) (Mexico). Mexico COVID-19 Deaths 2020.
Moldova	Johns Hopkins University. 2019 Novel Coronavirus COVID-19 (2019-nCoV) Data Repository by Johns Hopkins CSSE. Baltimore, Maryland: Johns Hopkins University.
Monaco	Johns Hopkins University. 2019 Novel Coronavirus COVID-19 (2019-nCoV) Data Repository by Johns Hopkins CSSE. Baltimore, Maryland: Johns Hopkins University.
Mongolia	Johns Hopkins University. 2019 Novel Coronavirus COVID-19 (2019-nCoV) Data Repository by Johns Hopkins CSSE. Baltimore, Maryland: Johns Hopkins University.
Montenegro	Johns Hopkins University. 2019 Novel Coronavirus COVID-19 (2019-nCoV) Data Repository by Johns Hopkins CSSE. Baltimore, Maryland: Johns Hopkins University.
Morocco	Johns Hopkins University. 2019 Novel Coronavirus COVID-19 (2019-nCoV) Data Repository by Johns Hopkins CSSE. Baltimore, Maryland: Johns Hopkins University.
Mozambique	Johns Hopkins University. 2019 Novel Coronavirus COVID-19 (2019-nCoV) Data Repository by Johns Hopkins CSSE. Baltimore, Maryland: Johns Hopkins University.
Myanmar	Johns Hopkins University. 2019 Novel Coronavirus COVID-19 (2019-nCoV) Data Repository by Johns Hopkins CSSE. Baltimore, Maryland: Johns Hopkins University.
Namibia	Johns Hopkins University. 2019 Novel Coronavirus COVID-19 (2019-nCoV) Data Repository by Johns Hopkins CSSE. Baltimore, Maryland: Johns Hopkins University.
Nepal	Johns Hopkins University. 2019 Novel Coronavirus COVID-19 (2019-nCoV) Data Repository by Johns Hopkins CSSE. Baltimore, Maryland: Johns Hopkins University.

Netherlands	Johns Hopkins University. 2019 Novel Coronavirus COVID-19 (2019-nCoV) Data Repository by Johns Hopkins CSSE. Baltimore, Maryland: Johns Hopkins University.
New Zealand	Johns Hopkins University. 2019 Novel Coronavirus COVID-19 (2019-nCoV) Data Repository by Johns Hopkins CSSE. Baltimore, Maryland: Johns Hopkins University.
Nicaragua	Johns Hopkins University. 2019 Novel Coronavirus COVID-19 (2019-nCoV) Data Repository by Johns Hopkins CSSE. Baltimore, Maryland: Johns Hopkins University.
Niger	Johns Hopkins University. 2019 Novel Coronavirus COVID-19 (2019-nCoV) Data Repository by Johns Hopkins CSSE. Baltimore, Maryland: Johns Hopkins University.
Nigeria	Johns Hopkins University. 2019 Novel Coronavirus COVID-19 (2019-nCoV) Data Repository by Johns Hopkins CSSE. Baltimore, Maryland: Johns Hopkins University.
North Macedonia	Johns Hopkins University. 2019 Novel Coronavirus COVID-19 (2019-nCoV) Data Repository by Johns Hopkins CSSE. Baltimore, Maryland: Johns Hopkins University.
Northern Mariana Islands	Johns Hopkins University. 2019 Novel Coronavirus COVID-19 (2019-nCoV) Data Repository by Johns Hopkins CSSE. Baltimore, Maryland: Johns Hopkins University.
Norway	Johns Hopkins University. 2019 Novel Coronavirus COVID-19 (2019-nCoV) Data Repository by Johns Hopkins CSSE. Baltimore, Maryland: Johns Hopkins University.
Oman	Johns Hopkins University. 2019 Novel Coronavirus COVID-19 (2019-nCoV) Data Repository by Johns Hopkins CSSE. Baltimore, Maryland: Johns Hopkins University.
Pakistan, Azad Jammu & Kashmir	Ministry of National Health Services, Regulations & Coordination (Pakistan). Pakistan - Azad Jammu and Kashmir COVID-19 Statistics. Islamabad, Pakistan: Government of Pakistan.
Pakistan, Azad Jammu & Kashmir	Ministry of National Health Services, Regulations & Coordination (Pakistan). Pakistan COVID-19 Dashboard.
Pakistan, Balochistan	Ministry of National Health Services, Regulations & Coordination (Pakistan). Pakistan - Balochistan COVID-19 Statistics. Islamabad, Pakistan: Government of Pakistan.
Pakistan, Balochistan	Ministry of National Health Services, Regulations & Coordination (Pakistan). Pakistan COVID-19 Dashboard.
Pakistan, Gilgit-Baltistan	Ministry of National Health Services, Regulations & Coordination (Pakistan). Pakistan - Gilgit-Baltistan COVID-19 Statistics. Islamabad, Pakistan: Government of Pakistan.
Pakistan, Gilgit-Baltistan	Ministry of National Health Services, Regulations & Coordination (Pakistan). Pakistan COVID-19 Dashboard.
Pakistan, Islamabad Capital Territory	Ministry of National Health Services, Regulations & Coordination (Pakistan). Pakistan - Islamabad COVID-19 Statistics. Islamabad, Pakistan: Government of Pakistan.
Pakistan, Islamabad Capital Territory	Ministry of National Health Services, Regulations & Coordination (Pakistan). Pakistan COVID-19 Dashboard.
Pakistan, Khyber Pakhtunkhwa	Ministry of National Health Services, Regulations & Coordination (Pakistan). Pakistan - Khyber Pakhtunkhwa COVID-19 Statistics 2020.
Pakistan, Khyber Pakhtunkhwa	Ministry of National Health Services, Regulations & Coordination (Pakistan). Pakistan COVID-19 Dashboard.
Pakistan, Punjab	Ministry of National Health Services, Regulations & Coordination (Pakistan). Pakistan - Punjab COVID-19 Statistics. Islamabad, Pakistan: Government of Pakistan.
Pakistan, Punjab	Ministry of National Health Services, Regulations & Coordination (Pakistan). Pakistan COVID-19 Dashboard.
Pakistan, Sindh	Wikipedia. COVID-19 pandemic in Pakistan. San Francisco, United States of America: Wikipedia. Retrieved on April 29, 2021 from https://en.wikipedia.org/wiki/COVID-19_pandemic_in_Pakistan
Pakistan, Sindh	Ministry of National Health Services, Regulations & Coordination (Pakistan). Pakistan - Sindh COVID-19 Statistics. Islamabad, Pakistan: Government of Pakistan.
Pakistan, Sindh	Ministry of National Health Services, Regulations & Coordination (Pakistan). Pakistan COVID-19 Dashboard.
Palau	Johns Hopkins University. 2019 Novel Coronavirus COVID-19 (2019-nCoV) Data Repository by Johns Hopkins CSSE. Baltimore, Maryland: Johns Hopkins University.
Palestine	Johns Hopkins University. 2019 Novel Coronavirus COVID-19 (2019-nCoV) Data Repository by Johns Hopkins CSSE. Baltimore, Maryland: Johns Hopkins University.
Panama	Johns Hopkins University. 2019 Novel Coronavirus COVID-19 (2019-nCoV) Data Repository by Johns Hopkins CSSE. Baltimore, Maryland: Johns Hopkins University.
Papua New Guinea	Johns Hopkins University. 2019 Novel Coronavirus COVID-19 (2019-nCoV) Data Repository by Johns Hopkins CSSE. Baltimore, Maryland: Johns Hopkins University.
Paraguay	Johns Hopkins University. 2019 Novel Coronavirus COVID-19 (2019-nCoV) Data Repository by Johns Hopkins CSSE. Baltimore, Maryland: Johns Hopkins University.
Peru	Ministry of Health (Peru), National Center for Epidemiology, Prevention and Disease Control (Peru). Peru Deaths by COVID-19.
Philippines	Department of Health (Philippines). Philippines Department of Health COVID-19 Tracker. Manila, Philippines: Department of Health (Philippines).
Poland	Johns Hopkins University. 2019 Novel Coronavirus COVID-19 (2019-nCoV) Data Repository by Johns Hopkins CSSE. Baltimore, Maryland: Johns Hopkins University.
Portugal	Johns Hopkins University. 2019 Novel Coronavirus COVID-19 (2019-nCoV) Data Repository by Johns Hopkins CSSE. Baltimore, Maryland: Johns Hopkins University.
Puerto Rico	Johns Hopkins University. 2019 Novel Coronavirus COVID-19 (2019-nCoV) Data Repository by Johns Hopkins CSSE. Baltimore, Maryland: Johns Hopkins University.

Qatar	Johns Hopkins University. 2019 Novel Coronavirus COVID-19 (2019-nCoV) Data Repository by Johns Hopkins CSSE. Baltimore, Maryland: Johns Hopkins University.
Romania	Johns Hopkins University. 2019 Novel Coronavirus COVID-19 (2019-nCoV) Data Repository by Johns Hopkins CSSE. Baltimore, Maryland: Johns Hopkins University.
Russia	Federal State Statistics Service (Russia). Russia Natural Movement of the Population 2021. Moscow, Russian Federation: Federal State Statistics Service (Russia).
Rwanda	Johns Hopkins University. 2019 Novel Coronavirus COVID-19 (2019-nCoV) Data Repository by Johns Hopkins CSSE. Baltimore, Maryland: Johns Hopkins University.
Saint Kitts and Nevis	Johns Hopkins University. 2019 Novel Coronavirus COVID-19 (2019-nCoV) Data Repository by Johns Hopkins CSSE. Baltimore, Maryland: Johns Hopkins University.
Saint Lucia	Johns Hopkins University. 2019 Novel Coronavirus COVID-19 (2019-nCoV) Data Repository by Johns Hopkins CSSE. Baltimore, Maryland: Johns Hopkins University.
Saint Vincent and the Grenadines	Johns Hopkins University. 2019 Novel Coronavirus COVID-19 (2019-nCoV) Data Repository by Johns Hopkins CSSE. Baltimore, Maryland: Johns Hopkins University.
Samoa	Johns Hopkins University. 2019 Novel Coronavirus COVID-19 (2019-nCoV) Data Repository by Johns Hopkins CSSE. Baltimore, Maryland: Johns Hopkins University.
San Marino	Johns Hopkins University. 2019 Novel Coronavirus COVID-19 (2019-nCoV) Data Repository by Johns Hopkins CSSE. Baltimore, Maryland: Johns Hopkins University.
São Tomé and Príncipe	Johns Hopkins University. 2019 Novel Coronavirus COVID-19 (2019-nCoV) Data Repository by Johns Hopkins CSSE. Baltimore, Maryland: Johns Hopkins University.
Saudi Arabia	Johns Hopkins University. 2019 Novel Coronavirus COVID-19 (2019-nCoV) Data Repository by Johns Hopkins CSSE. Baltimore, Maryland: Johns Hopkins University.
Senegal	Johns Hopkins University. 2019 Novel Coronavirus COVID-19 (2019-nCoV) Data Repository by Johns Hopkins CSSE. Baltimore, Maryland: Johns Hopkins University.
Serbia	Johns Hopkins University. 2019 Novel Coronavirus COVID-19 (2019-nCoV) Data Repository by Johns Hopkins CSSE. Baltimore, Maryland: Johns Hopkins University.
Seychelles	Johns Hopkins University. 2019 Novel Coronavirus COVID-19 (2019-nCoV) Data Repository by Johns Hopkins CSSE. Baltimore, Maryland: Johns Hopkins University.
Sierra Leone	Johns Hopkins University. 2019 Novel Coronavirus COVID-19 (2019-nCoV) Data Repository by Johns Hopkins CSSE. Baltimore, Maryland: Johns Hopkins University.
Singapore	Johns Hopkins University. 2019 Novel Coronavirus COVID-19 (2019-nCoV) Data Repository by Johns Hopkins CSSE. Baltimore, Maryland: Johns Hopkins University.
Slovakia	Johns Hopkins University. 2019 Novel Coronavirus COVID-19 (2019-nCoV) Data Repository by Johns Hopkins CSSE. Baltimore, Maryland: Johns Hopkins University.
Slovenia	Johns Hopkins University. 2019 Novel Coronavirus COVID-19 (2019-nCoV) Data Repository by Johns Hopkins CSSE. Baltimore, Maryland: Johns Hopkins University.
Solomon Islands	Johns Hopkins University. 2019 Novel Coronavirus COVID-19 (2019-nCoV) Data Repository by Johns Hopkins CSSE. Baltimore, Maryland: Johns Hopkins University.
Somalia	Johns Hopkins University. 2019 Novel Coronavirus COVID-19 (2019-nCoV) Data Repository by Johns Hopkins CSSE. Baltimore, Maryland: Johns Hopkins University.
South Africa	Johns Hopkins University. 2019 Novel Coronavirus COVID-19 (2019-nCoV) Data Repository by Johns Hopkins CSSE. Baltimore, Maryland: Johns Hopkins University.
South Korea	Johns Hopkins University. 2019 Novel Coronavirus COVID-19 (2019-nCoV) Data Repository by Johns Hopkins CSSE. Baltimore, Maryland: Johns Hopkins University.
South Sudan	Johns Hopkins University. 2019 Novel Coronavirus COVID-19 (2019-nCoV) Data Repository by Johns Hopkins CSSE. Baltimore, Maryland: Johns Hopkins University.
Spain, Andalusia	Andalusia Ministry of Health and Families (Spain). Spain - Andalusia Ministry of Health and Families Coronavirus Report.
Spain, Aragon	Ministry of Health, Consumption and Social Welfare (Spain). Spain Ministry of Health, Consumption, and Social Welfare COVID-19 Situation Update. Spain: Ministry of Health, Consumption and Social Welfare (Spain).
Spain, Aragon	Institute of Health Carlos III (Spain), Ministry of Health, Consumption and Social Welfare (Spain). Spain Carlos III Health Institute Situation of COVID-19. Madrid, Spain: Institute of Health Carlos III (Spain).
Spain, Asturias	Ministry of Health, Consumption and Social Welfare (Spain). Spain Ministry of Health, Consumption, and Social Welfare COVID-19 Situation Update. Spain: Ministry of Health, Consumption and Social Welfare (Spain).
Spain, Asturias	Institute of Health Carlos III (Spain), Ministry of Health, Consumption and Social Welfare (Spain). Spain Carlos III Health Institute Situation of COVID-19. Madrid, Spain: Institute of Health Carlos III (Spain).
Spain, Balearic Islands	Government of the Balearic Islands. Spain - Balearic Islands Ministry of Health and Consumption News About the Coronavirus COVID-19.
Spain, Basque Country	Basque Government Department of Health. Spain - Basque Country Information on the Evolution of the Coronavirus Epidemiological Bulletin.
Spain, Canary Islands	Government of the Canary Islands (Spain). Spain - Canary Islands Government COVID-19 Dashboard.
Spain, Cantabria	Cantabrian Health Service (Spain), Government of Cantabria (Spain). Spain - Cantabria Epidemiological Situation of COVID-19. Spain: Cantabrian Health Service (Spain).
Spain, Castile and León	Castile and León Board, Health Commission (Spain). Spain - Castile and León Open Data: Coronavirus (COVID-19) Epidemiological Situation.
Spain, Castile and León	Castile and León Board, Health Commission (Spain). Spain - Castile and León Open Data: Situation of Hospitalized by Coronavirus.

Spain, Castilla–La Mancha	Ministry of Health, Consumption and Social Welfare (Spain). Spain Ministry of Health, Consumption, and Social Welfare COVID-19 Situation Update. Spain: Ministry of Health, Consumption and Social Welfare (Spain).
Spain, Castilla–La Mancha	Institute of Health Carlos III (Spain), Ministry of Health, Consumption and Social Welfare (Spain). Spain Carlos III Health Institute Situation of COVID-19. Madrid, Spain: Institute of Health Carlos III (Spain).
Spain, Catalonia	Statistical Institute of Catalonia (IDESCAT) (Spain). Spain - Catalonia COVID-19 Weekly Confirmed Cases and Deaths. Barcelona, Spain: Statistical Institute of Catalonia (IDESCAT) (Spain).
Spain, Catalonia	Statistical Institute of Catalonia (IDESCAT) (Spain). Spain - Catalonia COVID-19 Daily Confirmed Cases and Deaths 2020. Barcelona, Spain: Statistical Institute of Catalonia (IDESCAT) (Spain), 2020.
Spain, Ceuta	Ministry of Health, Consumption and Social Welfare (Spain). Spain Ministry of Health, Consumption, and Social Welfare COVID-19 Situation Update. Spain: Ministry of Health, Consumption and Social Welfare (Spain).
Spain, Ceuta	Institute of Health Carlos III (Spain), Ministry of Health, Consumption and Social Welfare (Spain). Spain Carlos III Health Institute Situation of COVID-19. Madrid, Spain: Institute of Health Carlos III (Spain).
Spain, Community of Madrid	Ministry of Health, Consumption and Social Welfare (Spain). Spain Ministry of Health, Consumption, and Social Welfare COVID-19 Situation Update. Spain: Ministry of Health, Consumption and Social Welfare (Spain).
Spain, Community of Madrid	City of Madrid (Spain), Madrid Health Service (Spain). Spain - Madrid Health Service COVID-19 Current Situation Daily Status Report. Madrid, Spain: City of Madrid (Spain).
Spain, Extremadura	Ministry of Health, Consumption and Social Welfare (Spain). Spain Ministry of Health, Consumption, and Social Welfare COVID-19 Situation Update. Spain: Ministry of Health, Consumption and Social Welfare (Spain).
Spain, Extremadura	Institute of Health Carlos III (Spain), Ministry of Health, Consumption and Social Welfare (Spain). Spain Carlos III Health Institute Situation of COVID-19. Madrid, Spain: Institute of Health Carlos III (Spain).
Spain, Galicia	Galician Healthcare Service (Spain), Regional Government of Galicia (Spain). Spain - Galicia Regional Government COVID-19 Press Releases 2020. Spain: Regional Government of Galicia (Spain).
Spain, La Rioja	Government of La Rioja (Spain). Spain - La Rioja Covid-19 Tests Performed per Days. Brazil: Government of La Rioja (Spain).
Spain, Melilla	Ministry of Health, Consumption and Social Welfare (Spain). Spain Ministry of Health, Consumption, and Social Welfare COVID-19 Situation Update. Spain: Ministry of Health, Consumption and Social Welfare (Spain).
Spain, Melilla	Institute of Health Carlos III (Spain), Ministry of Health, Consumption and Social Welfare (Spain). Spain Carlos III Health Institute Situation of COVID-19. Madrid, Spain: Institute of Health Carlos III (Spain).
Spain, Murcia	Institute of Health Carlos III (Spain), Ministry of Health, Consumption and Social Welfare (Spain). Spain Carlos III Health Institute Situation of COVID-19. Madrid, Spain: Institute of Health Carlos III (Spain).
Spain, Murcia	Ministry of Health of the Region of Murcia (Spain). COVID-19 Region of Murcia - Spain. Spain: Ministry of Health of the Region of Murcia (Spain).
Spain, Navarre	Government of Navarra (Spain). Spain - Navarra COVID-19 Testing, Deaths, Hospitalisation Data May-June 2020. Spain: Government of Navarra (Spain), 2020.
Spain, Valencian Community	Valencia Ministry of Universal Health and Public Health (Spain). Spain - Valencia COVID-19: Monitoring of the Situation. Spain: Valencia Ministry of Universal Health and Public Health (Spain).
Sri Lanka	Johns Hopkins University. 2019 Novel Coronavirus COVID-19 (2019-nCoV) Data Repository by Johns Hopkins CSSE. Baltimore, Maryland: Johns Hopkins University.
Sudan	Johns Hopkins University. 2019 Novel Coronavirus COVID-19 (2019-nCoV) Data Repository by Johns Hopkins CSSE. Baltimore, Maryland: Johns Hopkins University.
Suriname	Johns Hopkins University. 2019 Novel Coronavirus COVID-19 (2019-nCoV) Data Repository by Johns Hopkins CSSE. Baltimore, Maryland: Johns Hopkins University.
Sweden	Public Health Agency of Sweden. Sweden Public Health Agency COVID-19 Confirmed Cases Daily Update. Östersund, Sweden: Public Health Agency of Sweden.
Switzerland	Federal Office of Public Health (Switzerland). Switzerland Federal Office of Public Health New Coronavirus Current Situation. Berne, Switzerland: Federal Office of Public Health (Switzerland).
Syria	Johns Hopkins University. 2019 Novel Coronavirus COVID-19 (2019-nCoV) Data Repository by Johns Hopkins CSSE. Baltimore, Maryland: Johns Hopkins University.
Taiwan (Province of China)	Johns Hopkins University. 2019 Novel Coronavirus COVID-19 (2019-nCoV) Data Repository by Johns Hopkins CSSE. Baltimore, Maryland: Johns Hopkins University.
Tajikistan	Johns Hopkins University. 2019 Novel Coronavirus COVID-19 (2019-nCoV) Data Repository by Johns Hopkins CSSE. Baltimore, Maryland: Johns Hopkins University.
Tanzania	Johns Hopkins University. 2019 Novel Coronavirus COVID-19 (2019-nCoV) Data Repository by Johns Hopkins CSSE. Baltimore, Maryland: Johns Hopkins University.
Thailand	Johns Hopkins University. 2019 Novel Coronavirus COVID-19 (2019-nCoV) Data Repository by Johns Hopkins CSSE. Baltimore, Maryland: Johns Hopkins University.
The Bahamas	Johns Hopkins University. 2019 Novel Coronavirus COVID-19 (2019-nCoV) Data Repository by Johns Hopkins CSSE. Baltimore, Maryland: Johns Hopkins University.
The Gambia	Johns Hopkins University. 2019 Novel Coronavirus COVID-19 (2019-nCoV) Data Repository by Johns Hopkins CSSE. Baltimore, Maryland: Johns Hopkins University.
Timor-Leste	Johns Hopkins University. 2019 Novel Coronavirus COVID-19 (2019-nCoV) Data Repository by Johns Hopkins CSSE. Baltimore, Maryland: Johns Hopkins University.
Togo	Johns Hopkins University. 2019 Novel Coronavirus COVID-19 (2019-nCoV) Data Repository by Johns Hopkins CSSE. Baltimore, Maryland: Johns Hopkins University.
Tonga	Johns Hopkins University. 2019 Novel Coronavirus COVID-19 (2019-nCoV) Data Repository by Johns Hopkins CSSE. Baltimore, Maryland: Johns Hopkins University.

Trinidad and Tobago	Johns Hopkins University. 2019 Novel Coronavirus COVID-19 (2019-nCoV) Data Repository by Johns Hopkins CSSE. Baltimore, Maryland: Johns Hopkins University.
Tunisia	Johns Hopkins University. 2019 Novel Coronavirus COVID-19 (2019-nCoV) Data Repository by Johns Hopkins CSSE. Baltimore, Maryland: Johns Hopkins University.
Turkey	Johns Hopkins University. 2019 Novel Coronavirus COVID-19 (2019-nCoV) Data Repository by Johns Hopkins CSSE. Baltimore, Maryland: Johns Hopkins University.
Uganda	Johns Hopkins University. 2019 Novel Coronavirus COVID-19 (2019-nCoV) Data Repository by Johns Hopkins CSSE. Baltimore, Maryland: Johns Hopkins University.
UK, England	Office for National Statistics (ONS) (United Kingdom). United Kingdom - England and Wales Deaths Registered Weekly, Provisional.
UK, Northern Ireland	Office for National Statistics (ONS) (United Kingdom). United Kingdom - England and Wales Deaths Registered Weekly, Provisional.
UK, Scotland	Office for National Statistics (ONS) (United Kingdom). United Kingdom - England and Wales Deaths Registered Weekly, Provisional.
UK, Wales	Office for National Statistics (ONS) (United Kingdom). United Kingdom - England and Wales Deaths Registered Weekly, Provisional.
Ukraine	Johns Hopkins University. 2019 Novel Coronavirus COVID-19 (2019-nCoV) Data Repository by Johns Hopkins CSSE. Baltimore, Maryland: Johns Hopkins University.
United Arab Emirates	Johns Hopkins University. 2019 Novel Coronavirus COVID-19 (2019-nCoV) Data Repository by Johns Hopkins CSSE. Baltimore, Maryland: Johns Hopkins University.
Uruguay	Johns Hopkins University. 2019 Novel Coronavirus COVID-19 (2019-nCoV) Data Repository by Johns Hopkins CSSE. Baltimore, Maryland: Johns Hopkins University.
USA, Alabama	Johns Hopkins University. 2019 Novel Coronavirus COVID-19 (2019-nCoV) Data Repository by Johns Hopkins CSSE. Baltimore, Maryland: Johns Hopkins University.
USA, Alaska	Johns Hopkins University. 2019 Novel Coronavirus COVID-19 (2019-nCoV) Data Repository by Johns Hopkins CSSE. Baltimore, Maryland: Johns Hopkins University.
USA, Arizona	Johns Hopkins University. 2019 Novel Coronavirus COVID-19 (2019-nCoV) Data Repository by Johns Hopkins CSSE. Baltimore, Maryland: Johns Hopkins University.
USA, Arkansas	Johns Hopkins University. 2019 Novel Coronavirus COVID-19 (2019-nCoV) Data Repository by Johns Hopkins CSSE. Baltimore, Maryland: Johns Hopkins University.
USA, California	Johns Hopkins University. 2019 Novel Coronavirus COVID-19 (2019-nCoV) Data Repository by Johns Hopkins CSSE. Baltimore, Maryland: Johns Hopkins University.
USA, Colorado	Johns Hopkins University. 2019 Novel Coronavirus COVID-19 (2019-nCoV) Data Repository by Johns Hopkins CSSE. Baltimore, Maryland: Johns Hopkins University.
USA, Connecticut	Johns Hopkins University. 2019 Novel Coronavirus COVID-19 (2019-nCoV) Data Repository by Johns Hopkins CSSE. Baltimore, Maryland: Johns Hopkins University.
USA, Delaware	Delaware Division of Public Health (United States). United States - Delaware Division of Public Health Coronavirus Disease (COVID-19) Data Dashboard. United States of America: Delaware Division of Public Health (United States).
USA, Florida	Johns Hopkins University. 2019 Novel Coronavirus COVID-19 (2019-nCoV) Data Repository by Johns Hopkins CSSE. Baltimore, Maryland: Johns Hopkins University.
USA, Georgia	Johns Hopkins University. 2019 Novel Coronavirus COVID-19 (2019-nCoV) Data Repository by Johns Hopkins CSSE. Baltimore, Maryland: Johns Hopkins University.
USA, Hawaii	Hawaii State Department of Health. United States - Hawaii Department of Health COVID-19 Current Situation. Honolulu, HI, United States of America: Hawaii State Department of Health.
USA, Idaho	Johns Hopkins University. 2019 Novel Coronavirus COVID-19 (2019-nCoV) Data Repository by Johns Hopkins CSSE. Baltimore, Maryland: Johns Hopkins University.
USA, Illinois	Johns Hopkins University. 2019 Novel Coronavirus COVID-19 (2019-nCoV) Data Repository by Johns Hopkins CSSE. Baltimore, Maryland: Johns Hopkins University.
USA, Indiana	Johns Hopkins University. 2019 Novel Coronavirus COVID-19 (2019-nCoV) Data Repository by Johns Hopkins CSSE. Baltimore, Maryland: Johns Hopkins University.
USA, Iowa	Johns Hopkins University. 2019 Novel Coronavirus COVID-19 (2019-nCoV) Data Repository by Johns Hopkins CSSE. Baltimore, Maryland: Johns Hopkins University.
USA, Kansas	Johns Hopkins University. 2019 Novel Coronavirus COVID-19 (2019-nCoV) Data Repository by Johns Hopkins CSSE. Baltimore, Maryland: Johns Hopkins University.
USA, Kentucky	Johns Hopkins University. 2019 Novel Coronavirus COVID-19 (2019-nCoV) Data Repository by Johns Hopkins CSSE. Baltimore, Maryland: Johns Hopkins University.
USA, Louisiana	Johns Hopkins University. 2019 Novel Coronavirus COVID-19 (2019-nCoV) Data Repository by Johns Hopkins CSSE. Baltimore, Maryland: Johns Hopkins University.
USA, Maine	Johns Hopkins University. 2019 Novel Coronavirus COVID-19 (2019-nCoV) Data Repository by Johns Hopkins CSSE. Baltimore, Maryland: Johns Hopkins University.
USA, Maryland	Johns Hopkins University. 2019 Novel Coronavirus COVID-19 (2019-nCoV) Data Repository by Johns Hopkins CSSE. Baltimore, Maryland: Johns Hopkins University.
USA, Massachusetts	Johns Hopkins University. 2019 Novel Coronavirus COVID-19 (2019-nCoV) Data Repository by Johns Hopkins CSSE. Baltimore, Maryland: Johns Hopkins University.
USA, Michigan	Johns Hopkins University. 2019 Novel Coronavirus COVID-19 (2019-nCoV) Data Repository by Johns Hopkins CSSE. Baltimore, Maryland: Johns Hopkins University.

USA, Minnesota	Johns Hopkins University. 2019 Novel Coronavirus COVID-19 (2019-nCoV) Data Repository by Johns Hopkins CSSE. Baltimore, Maryland: Johns Hopkins University.
USA, Mississippi	Johns Hopkins University. 2019 Novel Coronavirus COVID-19 (2019-nCoV) Data Repository by Johns Hopkins CSSE. Baltimore, Maryland: Johns Hopkins University.
USA, Missouri	Johns Hopkins University. 2019 Novel Coronavirus COVID-19 (2019-nCoV) Data Repository by Johns Hopkins CSSE. Baltimore, Maryland: Johns Hopkins University.
USA, Montana	Johns Hopkins University. 2019 Novel Coronavirus COVID-19 (2019-nCoV) Data Repository by Johns Hopkins CSSE. Baltimore, Maryland: Johns Hopkins University.
USA, Nebraska	Johns Hopkins University. 2019 Novel Coronavirus COVID-19 (2019-nCoV) Data Repository by Johns Hopkins CSSE. Baltimore, Maryland: Johns Hopkins University.
USA, Nevada	Johns Hopkins University. 2019 Novel Coronavirus COVID-19 (2019-nCoV) Data Repository by Johns Hopkins CSSE. Baltimore, Maryland: Johns Hopkins University.
USA, New Hampshire	Johns Hopkins University. 2019 Novel Coronavirus COVID-19 (2019-nCoV) Data Repository by Johns Hopkins CSSE. Baltimore, Maryland: Johns Hopkins University.
USA, New Jersey	Johns Hopkins University. 2019 Novel Coronavirus COVID-19 (2019-nCoV) Data Repository by Johns Hopkins CSSE. Baltimore, Maryland: Johns Hopkins University.
USA, New Mexico	Johns Hopkins University. 2019 Novel Coronavirus COVID-19 (2019-nCoV) Data Repository by Johns Hopkins CSSE. Baltimore, Maryland: Johns Hopkins University.
USA, New York	New York Times. COVID-19 Cumulative Deaths and Cases in the United States by County - New York Times. New York, United States of America: New York Times.
USA, New York	New York City Department of Health and Mental Hygiene. United States - New York City Department of Health and Mental Hygiene COVID-19 Data. New York, NY, United States of America: New York City Department of Health and Mental Hygiene.
USA, New York	Johns Hopkins University. 2019 Novel Coronavirus COVID-19 (2019-nCoV) Data Repository by Johns Hopkins CSSE. Baltimore, Maryland: Johns Hopkins University.
USA, North Carolina	Johns Hopkins University. 2019 Novel Coronavirus COVID-19 (2019-nCoV) Data Repository by Johns Hopkins CSSE. Baltimore, Maryland: Johns Hopkins University.
USA, North Dakota	Johns Hopkins University. 2019 Novel Coronavirus COVID-19 (2019-nCoV) Data Repository by Johns Hopkins CSSE. Baltimore, Maryland: Johns Hopkins University.
USA, Ohio	Ohio Department of Health. United States - Ohio Department of Health Coronavirus (COVID-19) Updates. Columbus, OH, United States of America: Ohio Department of Health.
USA, Oklahoma	Johns Hopkins University. 2019 Novel Coronavirus COVID-19 (2019-nCoV) Data Repository by Johns Hopkins CSSE. Baltimore, Maryland: Johns Hopkins University.
USA, Oregon	Oregon Health Authority (United States). United States - Oregon Health Authority COVID-19 Updates. Salem, United States of America: Oregon Health Authority (United States).
USA, Pennsylvania	Johns Hopkins University. 2019 Novel Coronavirus COVID-19 (2019-nCoV) Data Repository by Johns Hopkins CSSE. Baltimore, Maryland: Johns Hopkins University.
USA, Rhode Island	Johns Hopkins University. 2019 Novel Coronavirus COVID-19 (2019-nCoV) Data Repository by Johns Hopkins CSSE. Baltimore, Maryland: Johns Hopkins University.
USA, South Carolina	Johns Hopkins University. 2019 Novel Coronavirus COVID-19 (2019-nCoV) Data Repository by Johns Hopkins CSSE. Baltimore, Maryland: Johns Hopkins University.
USA, South Dakota	Johns Hopkins University. 2019 Novel Coronavirus COVID-19 (2019-nCoV) Data Repository by Johns Hopkins CSSE. Baltimore, Maryland: Johns Hopkins University.
USA, Tennessee	Johns Hopkins University. 2019 Novel Coronavirus COVID-19 (2019-nCoV) Data Repository by Johns Hopkins CSSE. Baltimore, Maryland: Johns Hopkins University.
USA, Texas	Johns Hopkins University. 2019 Novel Coronavirus COVID-19 (2019-nCoV) Data Repository by Johns Hopkins CSSE. Baltimore, Maryland: Johns Hopkins University.
USA, Utah	Johns Hopkins University. 2019 Novel Coronavirus COVID-19 (2019-nCoV) Data Repository by Johns Hopkins CSSE. Baltimore, Maryland: Johns Hopkins University.
USA, Vermont	Johns Hopkins University. 2019 Novel Coronavirus COVID-19 (2019-nCoV) Data Repository by Johns Hopkins CSSE. Baltimore, Maryland: Johns Hopkins University.
USA, Virginia	Johns Hopkins University. 2019 Novel Coronavirus COVID-19 (2019-nCoV) Data Repository by Johns Hopkins CSSE. Baltimore, Maryland: Johns Hopkins University.
USA, Washington	New York Times. COVID-19 Cumulative Deaths and Cases in the United States by County - New York Times. New York, United States of America: New York Times.
USA, Washington, DC	Johns Hopkins University. 2019 Novel Coronavirus COVID-19 (2019-nCoV) Data Repository by Johns Hopkins CSSE. Baltimore, Maryland: Johns Hopkins University.
USA, West Virginia	Johns Hopkins University. 2019 Novel Coronavirus COVID-19 (2019-nCoV) Data Repository by Johns Hopkins CSSE. Baltimore, Maryland: Johns Hopkins University.
USA, Wisconsin	Johns Hopkins University. 2019 Novel Coronavirus COVID-19 (2019-nCoV) Data Repository by Johns Hopkins CSSE. Baltimore, Maryland: Johns Hopkins University.
USA, Wyoming	Johns Hopkins University. 2019 Novel Coronavirus COVID-19 (2019-nCoV) Data Repository by Johns Hopkins CSSE. Baltimore, Maryland: Johns Hopkins University.
Uzbekistan	Johns Hopkins University. 2019 Novel Coronavirus COVID-19 (2019-nCoV) Data Repository by Johns Hopkins CSSE. Baltimore, Maryland: Johns Hopkins University.
Vanuatu	Johns Hopkins University. 2019 Novel Coronavirus COVID-19 (2019-nCoV) Data Repository by Johns Hopkins CSSE. Baltimore, Maryland: Johns Hopkins University.

Venezuela	Johns Hopkins University. 2019 Novel Coronavirus COVID-19 (2019-nCoV) Data Repository by Johns Hopkins CSSE. Baltimore, Maryland: Johns Hopkins University.
Vietnam	Johns Hopkins University. 2019 Novel Coronavirus COVID-19 (2019-nCoV) Data Repository by Johns Hopkins CSSE. Baltimore, Maryland: Johns Hopkins University.
Virgin Islands	Johns Hopkins University. 2019 Novel Coronavirus COVID-19 (2019-nCoV) Data Repository by Johns Hopkins CSSE. Baltimore, Maryland: Johns Hopkins University.
Yemen	Johns Hopkins University. 2019 Novel Coronavirus COVID-19 (2019-nCoV) Data Repository by Johns Hopkins CSSE. Baltimore, Maryland: Johns Hopkins University.
Zambia	Johns Hopkins University. 2019 Novel Coronavirus COVID-19 (2019-nCoV) Data Repository by Johns Hopkins CSSE. Baltimore, Maryland: Johns Hopkins University.
Zimbabwe	Johns Hopkins University. 2019 Novel Coronavirus COVID-19 (2019-nCoV) Data Repository by Johns Hopkins CSSE. Baltimore, Maryland: Johns Hopkins University.

Section 3: Seroprevalence sources

Figure S3. Seroprevalence data coverage by location

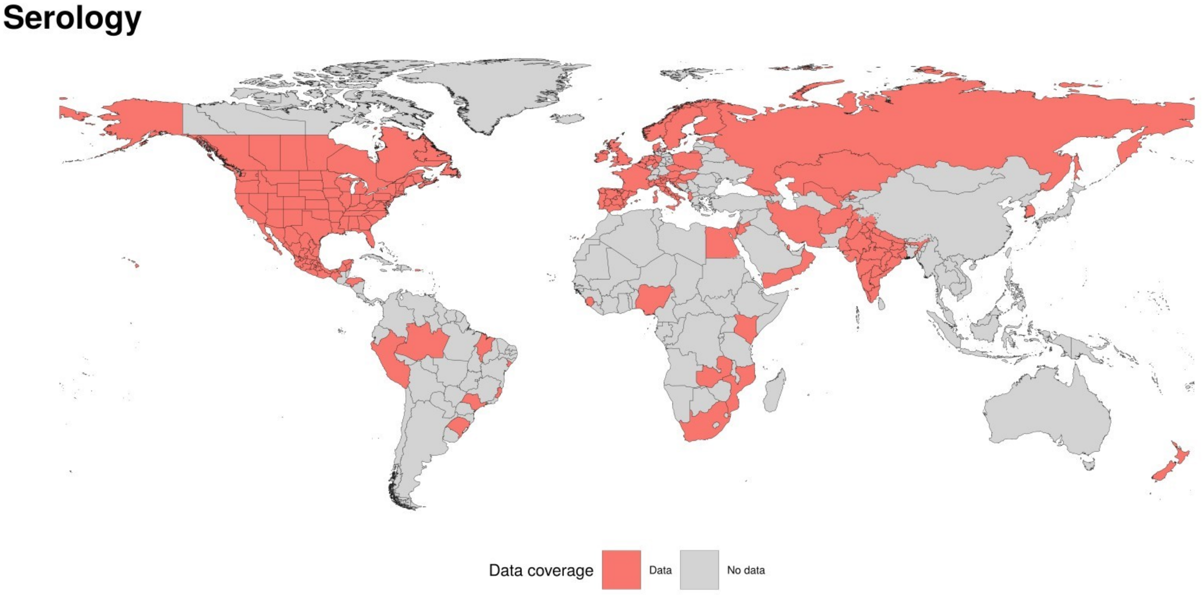

Table S3. Seroprevalence data sources by location

Location	Source
Afghanistan	World Health Organization (WHO). Prevalence of COVID-19 and its Related Deaths in Afghanistan: A Nationwide, Population-Based Seroepidemiological Study July 2020. Kabul, Afghanistan: Ministry of Public Health (Afghanistan), 2021.
Albania	Sulcebe G, Ylli A, Cenko F, Kurti-Prifti M. Rapid increase of SARS-CoV-2 seroprevalence during the 2020 pandemic year in the population of the city of Tirana, Albania. Preprint. medRxiv. 2021.
Andorra	Government of Andorra. [Martinez Benazet thanks institutions, volunteers and the population who have participated in the national antibody study]. Government of Andorra [Internet]. 2020 May 28; Press Releases.
Andorra	Royo-Cebrecos C, Vilanova D, López J, Arroyo V, Francisco G, Pons M, Carrasco MG, Piqué JM, Sanz S, Dobaño C, García-Basteiro A. Mass SARS-CoV-2 serological screening for the Principality of Andorra. Preprint. Res Sq. 2020.
Angola	Sebastião CS, Galangue M, Gaston C, Van-Dunen R, Quivinja J, Lunbungululo E, Alfredo D, Sozinho A, Teixeira A, Manico E, Machado D, Mateus A, David Z, Paixão J, Neto Z, de Vasconcelos JN, Morais J. Seroprevalence of anti-SARS-CoV-2 antibodies and risk factors among healthy blood donors in Luanda, Angola. BMC Infect Dis. 2021; 21(1): 1131.
Austria	Statistics Austria. 4.7% of the Austrian population had antibodies against SARS-CoV -2 in mid / late October. Vienna, Austria: Statistics Austria, 2020.
Belgium	Sciensano (Belgium). Belgium Prevalence of anti-SARS-CoV-2 antibodies (IgG) among blood donors. Belgium: Sciensano (Belgium).
Belgium	Herzog S, De Bie J, Abrams S, Wouters I, Ekinci E, Patteet L, Coppens A, De Spiegeleer S, Beutels P, Van Damme P, Hens N, Theeten H. Seroprevalence of IgG antibodies against SARS coronavirus 2 in Belgium - a serial prospective cross-sectional nationwide study of residual samples. Preprint. medRxiv. 2020.
Brazil, Amazonas	Buss LF, Prete CA Jr, Abrahim CMM, Mendrone A Jr, Salomon T, de Almeida-Neto C, FranÃ§a RFO, Belotti MC, Carvalho MPSS, Costa AG, Crispim MAE, Ferreira SC, Fraiji NA, Gurzenda S, Whittaker C, Kamaura LT, Takecian PL, da Silva Peixoto P, Oikawa MK, Nishiya AS, Rocha V, Salles NA, de Souza Santos AA, da Silva MA, Custer B, Parag KV, Barral-Netto M, Kraemer MUG, Pereira RHM, Pybus OG, Busch MP, Castro MC, Dye C, Nascimento VH, Faria NR, Sabino EC. Three-quarters attack rate of SARS-CoV-2 in the Brazilian Amazon during a largely unmitigated epidemic. Science. 2021; 371(6526): 288-292.
Brazil, Espírito Santo	Gomes CC, Cerutti Jr C, Zandonade E, Maciel ELN, Carvalho de Alencar FE, Almada GL, Cardoso OA, Medeiros Jabor P, Zanotti RL, Queiroz Reuter T, Gomes de Andrade VL, Bastos WM, Fernandes de Medeiros Jr N. A population-based study of the prevalence of COVID-19 infection in Espírito Santo, Brazil: methodology and results of the first stage. Preprint. medRxiv. 2020.
Brazil, Espírito Santo	Government of Espírito Santo (Brazil). Brazil - Government of Espírito Santo COVID-19 Serological Survey.
Brazil, Maranhão	Silva AAM da, Lima Neto LG, Azevedo C de MP e S de, Costa LMM da, Bragança MLBM, Barros Filho AKD, Wittlin

	BB, Souza BF de, Oliveira BLCA de, Carvalho CA de, Thomaz ÉBAF, Simões Neto EA, Leite Júnior JF, Cosme LMSS, Campos MAG, Queiroz RC de S, Costa SS, Carvalho VA de, Simóes VMF, Alves MTSS de B e, Santos AM dos. Population-based seroprevalence of SARS-CoV-2 is more than halfway through the herd immunity threshold in the State of Maranhao, Brazil. Preprint. medRxiv. 2020.
Brazil, Maranhão	Secretary of State for Health (Brazil - Maranhão). Prevalence of SARS-CoV-2 virus infection in Maranhão, Brazil, Final Research Report, Phase II, October 2020.
Brazil, Rio Grande do Sul	Government of Rio Grande do Sol. Brazil - Rio Grande Do Sol Epicovid19 Relevance Study 2020.
Brazil, São Paulo	Albuquerque JOM, Kamioka GA, Madalosso G, Costa SA, Ferreira PB, Pino FA, Sato APS, Carvalho ACA, Amorim ABP, Aires CC, Kataoka APAG, Savani ESMM, Bessa TAF, Aguiar BS, Failla MA, Santos EA, Brito EMT, Santos MCH, Silva SMS, Caldeira LAV, Zamarco LC, Fonseca SMS, Lima MMC, Marques IA, Silva FEV, Glasser PR, Burihan PCPR, Cavazzana CL, Lara RC, Mello DS, Pellini ACG, Nishio FY, Kian FM, Braga ES, Bertelli NMP, Fracini W, GonÃ§alves MDA, Zular PS, Piva RS, Masi E de. Prevalence evolution of SARS-CoV-2 infection in the Municipality of São Paulo, 2020 - 2021. medRxiv. 2021.
Brazil, São Paulo	Buss LF, Prete CA Jr, Abrahim CMM, Mendrone A Jr, Salomon T, de Almeida-Neto C, FranÃ§a RFO, Belotti MC, Carvalho MPSS, Costa AG, Crispim MAE, Ferreira SC, Fraiji NA, Gurzenda S, Whittaker C, Kamaura LT, Takecian PL, da Silva Peixoto P, Oikawa MK, Nishiya AS, Rocha V, Salles NA, de Souza Santos AA, da Silva MA, Custer B, Parag KV, Barral-Netto M, Kraemer MUG, Pereira RHM, Pybus OG, Busch MP, Castro MC, Dye C, Nascimento VH, Faria NR, Sabino EC. Three-quarters attack rate of SARS-CoV-2 in the Brazilian Amazon during a largely unmitigated epidemic. Science. 2021; 371(6526): 288-292.
Brazil, São Paulo	Brazilian Institute of Public Opinion and Statistics (IBOPE), Fleury Group, SEMEIA Institute (Brazil). Brazil - São Paulo Household Survey to Estimate the Seroprevalence of SARS-CoV-2 Infection, Pilot Study (SoroEpi MSP).
Brazil, São Paulo	Brazilian Institute of Public Opinion and Statistics (IBOPE), Fleury Group, SEMEIA Institute (Brazil). Brazil - São Paulo Household Survey to Estimate the Seroprevalence of SARS-CoV-2 Infection, Phase 2 (SoroEpi MSP).
Brazil, Sergipe	Federal University of Sergipe (Brazil). Brazil - Evolution of the Prevalence of Infection by SARS-CoV-2 in Sergipe. Third Phase Results: February 18 to March 30, 2021..
Burkina Faso	Lewis HC, Ware H, Whelan M, Subissi L, Li Z, Ma X, Nardone A, Valenciano M, Cheng B, Noel K, Cao C, Yanes-Lane M, Herring B, Talisuna A, Nsenga N, Balde T, Clifton DA, Van Kerkhove M, Buckeridge DL, Bobrovitz N, Okeibunor J, Arora RK, Bergeri I, the UNITY Studies Collaborator Group. SARS-CoV-2 infection in Africa: A systematic review and meta-analysis of standardised seroprevalence studies, from January 2020 to December 2021. Preprint. medRxiv. 2022.
Cameroon	Nwosu K, Fokam J, Wanda F, Mama L, Orel E, Ray N, Meke J, Tassegning A, Takou D, Mimbe E, Stoll B, Guillebert J, Comte E, Keiser O, Ciaffi L. SARS-CoV-2 antibody seroprevalence and associated risk factors in an urban district of Cameroon. Preprint. Res Sq. 2021.
Cameroon	Fai KN, Corine TM, Bebell LM, Mboringong AB, Nguimbis EBPT, Nsaibirni R, Mbarga NF, Eteki L, Nikolay B, Essomba RG, Ndifon M, Ntone R, Hamadou A, Matchim L, Tchiasso D, Abah Abah AS, Essaka R, Peppa S, Crescence F, Ouamba JP, Koku MT, Mandeng N, Fanne M, Eyangoh S, Mballa GAE, Esso L, EpÃ©e E, Njouom R, Okomo Assoumou MC, Boum Y. Serologic response to SARS-CoV-2 in an African population. Sci Afr. 2021; 12: e00802.
Canada, Alberta	Charlton CL, Nguyen LT, Bailey A, Fenton J, Plitt SS, Marohn C, Lau C, Hinshaw D, Lutsiak C, Simmonds K, Kanji JN, Zelyas N, Lee N, Mengel M, Tipples G. Pre-Vaccine Positivity of SARS-CoV-2 Antibodies in Alberta, Canada during the First Two Waves of the COVID-19 Pandemic. Microbiol Spectr. 2021; 9(1): e0029121.
Canada, Alberta	Canadian Blood Services. Canada COVID-19 Seroprevalence Report - January 12, 2021. Ottawa, Canada: Canadian Blood Services, 2021.
Canada, British Columbia	Tang X, Sharma A, Pasic M, et al.. SARS-CoV-2 Seroprevalence During the First and Second Pandemic Waves in Canada. Preprint.. Prepr Lancet. 2021.
Canada, British Columbia	Canadian Blood Services. Canada COVID-19 Seroprevalence Report - January 12, 2021. Ottawa, Canada: Canadian Blood Services, 2021.
Canada, Manitoba	Canadian Blood Services. Canada COVID-19 Seroprevalence Report - December 18, 2020. Ottawa, Canada: Canadian Blood Services, 2020.
Canada, Manitoba	Canadian Blood Services. Canada COVID-19 Seroprevalence Report - January 12, 2021. Ottawa, Canada: Canadian Blood Services, 2021.
Canada, Ontario	Bolotin S, Tran V, Osman S, Brown KA, Buchan SA, Joh E, Deeks SL, Allen VG. SARS-CoV-2 Seroprevalence Survey Estimates Are Affected by Anti-Nucleocapsid Antibody Decline. J Infect Dis. 2021; 223(8): 1334-1338.
Canada, Ontario	Investigators A to beat coronavirus/Action pour battre le coronavirus (Ab-CS, Jha P. COVID Seroprevalence, Symptoms and Mortality During the First Wave of SARS-CoV-2 in Canada. medRxiv. 2021.
Canada, Ontario	Tang X, Sharma A, Pasic M, et al.. SARS-CoV-2 Seroprevalence During the First and Second Pandemic Waves in Canada. Preprint.. Prepr Lancet. 2021.
Canada, Ontario	Government of Ontario (Canada), Public Health Ontario (Canada). Canada COVID-19 Seroprevalence in Ontario: March 27, 2020 to June 30, 2020. Canada: Public Health Ontario (Canada), 2020.
Canada, Ontario	Public Health Ontario (Canada). Canada COVID-19 Seroprevalence in Ontario: July 4 to July 31, 2020. Canada: Public Health Ontario (Canada), 2020.
Canada, Ontario	Public Health Ontario (Canada). Canada COVID-19 Seroprevalence in Ontario: August 1 to August 31, 2020. Canada: Public Health Ontario (Canada), 2020.
Canada, Ontario	Government of Ontario (Canada), Public Health Ontario (Canada). Canada COVID-19 Seroprevalence in Ontario: September 3 to October 30, 2020. Canada: Public Health Ontario (Canada), 2020.
Canada, Quebec	Investigators A to beat coronavirus/Action pour battre le coronavirus (Ab-CS, Jha P. COVID Seroprevalence, Symptoms and Mortality During the First Wave of SARS-CoV-2 in Canada. medRxiv. 2021.
Canada, Quebec	Tang X, Sharma A, Pasic M, et al.. SARS-CoV-2 Seroprevalence During the First and Second Pandemic Waves in Canada. Preprint.. Prepr Lancet. 2021.

Canada, Quebec	Hema-Quebec (Canada), Quebec National Institute of Public Health (Canada). Canada - Quebec COVID-19 First Wave Blood Donor Seroprevalence Study 2020. Canada: Hema-Quebec (Canada).
Central African Republic	Manirakiza A, Malaka C, Yambiyo BM, Diemer S-CH, Longo J de D, Namseneï J, Coti-Reckoundji CSG, Bouhouda M, Darnycka BMR, Roungou JB, Komas NP, Grésenguet G, Vernet G, Vernet M-A, Nakoune E. Very high relative seroprevalence of anti-SARS-CoV-2 antibodies among communities in Bangui, Central African Republic. Preprint. medRxiv. 2021.
Chile	Sauré D, O'Ryan M, Torres JP, Zuniga M, Santelices E, Basso LJ. Dynamic IgG seropositivity after rollout of CoronaVac and BNT162b2 COVID-19 vaccines in Chile: a sentinel surveillance study. Lancet Infect Dis. 2021.
Congo (Brazzaville)	Batchi-Bouyou AL, Lobaloba Ingoba L, Ndounga M, Vouvoungui JC, Mfoutou Mapanguy CC, Boumpoutou KR, Ntoumi F. High SARS-CoV-2 IgG/IGM seroprevalence in asymptomatic Congolese in Brazzaville, the Republic of Congo. Int J Infect Dis. 2021; 106: 3-7.
Croatia	Vilibic-Cavlek T, Stevanovic V, Ilic M, Barbic L, Capak K, Tabain I, Krleza JL, Ferenc T, Hruskar Z, Topic RZ, Kaliterna V, Antolovic-Pozgain A, Kucinar J, Koscak I, Mayer D, Sviben M, Antolasic L, Milasincic L, Bucic L, Ferencak I, Kaic B. SARS-CoV-2 Seroprevalence and Neutralizing Antibody Response after the First and Second COVID-19 Pandemic Wave in Croatia. Pathogens. 2021; 10(6): 774.
Czechia	Piler P, Thon V, Andrýsková L, Doležel K, Kostka D, Pavlík T, Dušek L, Pikhart H, Bobák M, Matic S, Klánová J. Dynamics of seroconversion of anti-SARS-CoV-2 IgG antibodies in the Czech unvaccinated population: nationwide prospective seroconversion (PROSECO) study. Preprint. medRxiv. 2021.
Denmark	Blood Donor (Denmark). Denmark Blood Donors are Now Being Re-examined for Coronavirus Infection. Denmark: Blood Donor (Denmark), 2020.
Denmark	Erikstrup C, Hother CE, Pedersen OBV, Mølbak K, Skov RL, Holm DK, Sækmose SG, Nilsson AC, Brooks PT, Boldsen JK, Mikkelsen C, Gybel-Brask M, Sørensen E, Dinh KM, Mikkelsen S, Møller BK, Haunstrup T, Harritshøj L, Jensen BA, Hjalgrim H, Lillevang ST, Ullum H. Estimation of SARS-CoV-2 infection fatality rate by real-time antibody screening of blood donors. Clin Infect Dis. 2020.
Denmark	Espenhain L, Tribler S, Jørgensen CS, Hansen CH, Sönksen UW, Ethelberg S. Prevalence of SARS-CoV-2 antibodies in Denmark 2020: results from nationwide, population-based sero-epidemiological surveys. Preprint. medRxiv. 2021; 2021040721254703.
DR Congo	Lewis HC, Ware H, Whelan M, Subissi L, Li Z, Ma X, Nardone A, Valenciano M, Cheng B, Noel K, Cao C, Yanes-Lane M, Herring B, Talisuna A, Nsenga N, Balde T, Clifton DA, Van Kerkhove M, Buckeridge DL, Bobrovitz N, Okeibunor J, Arora RK, Bergeri I, the UNITY Studies Collaborator Group. SARS-CoV-2 infection in Africa: A systematic review and meta-analysis of standardised seroprevalence studies, from January 2020 to December 2021. Preprint. medRxiv. 2022.
Egypt	Gomaa MR, El Rifay AS, Shehata M, Kandeil A, Nabil Kamel M, Marouf MA, GabAllah M, El Taweel A, Kayed AE, Kutkat O, Moatasim Y, Mahmoud SH, Abo Shama NM, El Sayes M, Mostafa A, El-Shesheny R, McKenzie PP, Webby RJ, Kayali G, Ali MA. Incidence, household transmission, and neutralizing antibody seroprevalence of Coronavirus Disease 2019 in Egypt: Results of a community-based cohort. PLOS Pathog. 2021; 17(3): e1009413.
Estonia	Soeorg H, Jõgi P, Naaber P, Ottas A, Toompere K, Lutsar I. Anti-spike protein receptor-binding domain IgG levels after COVID-19 infection or vaccination against SARS-CoV-2 in a seroprevalence study. Preprint. medRxiv. 2021.
Estonia	University of Tartu. Estonia COVID-19 Coronavirus prevalence study.
Ethiopia	Gudina EK, Ali S, Girma E, Gize A, Tegene B, Hundie GB, Sime WT, Ambachew R, Gebreyohanns A, Bekele M, Bakuli A, Elsbernd K, Merkt S, Contento L, Hoelscher M, Hasenauer J, Wieser A, Kroidl A. Seroepidemiology and model-based prediction of SARS-CoV-2 in Ethiopia: longitudinal cohort study among front-line hospital workers and communities. Lancet Glob Health. 2021; 9(11): e1517-e1527.
Ethiopia	Tadesse EB, Endris AA, Solomon H, Alayu M, Kebede A, Eshetu K, Teka G, Seid BE, Ahmed J, Abayneh SA, Moges B, Gerawork H, Sugerman D, Assefa Z, Abayneh A, Abate E, Taddese L. Seroprevalence and risk factors for SARS-CoV-2 Infection in selected urban areas in Ethiopia: a cross-sectional evaluation during July 2020. Int J Infect Dis. 2021; 111: 179-185.
Ethiopia	Shaweno T, Abdulhamid I, Bezabih L, Teshome D, Derese B, Tafesse H, Shaweno D. Sero-prevalence of SARS-CoV-2 Antibody Among Adults in the General Population in Diredawa, Ethiopia. Preprint. Res Sq. 2021.
France	Warszawski J, Meyer L, Franck J-E, Rahib D, Lydié N, Gosselin A, Counil E, Kreling R, Novelli S, Slama R, Raynaud P, Bagein G, Costemalle V, Sillard P, Fourie T, de Lamballerie X, Bajos N, Epicov Team. Trends in social exposure to SARS-Cov-2 in France. Evidence from the national socio-epidemiological cohort - EPICOV. Preprint. medRxiv. 2021.
France	Vu SL, Jones G, Anna F, Rose T, Richard J-B, Bernard-Stoecklin S, Goyard S, Demeret C, Helynck O, Robin C, Monnet V, Facci LP de, Ungeheuer M-N, Léon L, Guillois Y, Filleul L, Charneau P, Lévy-Bruhl D, van der Werf S, Noel H. Prevalence of SARS-CoV-2 antibodies in France: results from nationwide serological surveillance. Preprint. medRxiv. 2020.
Gabon	Mveang Nzoghe A, Leboueny M, Kuissi Kamgaing E, Maloupazoa Siawaya AC, Bongho EC, Mvoundza Ndjindji O, Padzys GS, Ndeboko B, Ategbo S, Djoba Siawaya JF. Circulating anti-SARS-CoV-2 nucleocapsid (N)-protein antibodies and anti-SARS-CoV-2 spike (S)-protein antibodies in an African setting: herd immunity, not there yet!. BMC Res Notes. 2021; 14(1): 152.
Georgia	*
Ghana	Lewis HC, Ware H, Whelan M, Subissi L, Li Z, Ma X, Nardone A, Valenciano M, Cheng B, Noel K, Cao C, Yanes-Lane M, Herring B, Talisuna A, Nsenga N, Balde T, Clifton DA, Van Kerkhove M, Buckeridge DL, Bobrovitz N, Okeibunor J, Arora RK, Bergeri I, the UNITY Studies Collaborator Group. SARS-CoV-2 infection in Africa: A systematic review and meta-analysis of standardised seroprevalence studies, from January 2020 to December 2021. Preprint. medRxiv. 2022.
Honduras	Núñez MRM, Lara JÁ, Pleitez SJA, Ramírez GA. [Seroepidemiological surveillance of SARS-CoV-2 circulation in 41 municipalities in Honduras with no report of active cases, COVID-19, June 16-23, 2020]. Poblac Salud Mesoam. 2021; 18(2).

India	Murhekar MV, Bhatnagar T, Thangaraj J, Saravanakumar V, Kumar MS, Selvaraju S, Rade K, Kumar C, Sabarinathan R, Turuk A, Asthana S, Balachandar R, Bangar SD, Bansal AK, Chopra V, Das D, Deb AK, Devi KR, Dhikav V, Dwivedi GR, ICMR Serosurveillance Group (2021). I SARS-CoV-2 seroprevalence among the general population and healthcare workers in India, December 2020-January 2021. 2021; 108: 145-155.
India	Government of India. India COVID-19 Official Government of India updates - Twitter.
India, Andhra Pradesh	Pandey A. Andhra Pradesh Sero Survey Show Nearly 20% of Stateâ€™s Population Exposed to Covid-19. India Today [Internet]. 2020 Sept 11.
India, Andhra Pradesh	Sadam R. 20% of Andhra Exposed to Covid, Sero-survey Finds, Exposure Highest Where Case Tally Lowest. ThePrint [Internet]. 2020 Sept 11.
India, Andhra Pradesh	Rao U. Andhra Pradesh: '59% found to have Covid antibodies in sero-survey'. The Times of India [Internet]. 2021 May 27; Visakhapatnam.
India, Andhra Pradesh	*
India, Andhra Pradesh	Mordani S. Seropositivity highest in Madhya Pradesh, lowest in Kerala, finds ICMR's national sero-survey. India Today [Internet]. 2021 Jul 28.
India, Assam	Pathak J, Das M, Siddique K. Assessment of Anti-SARS CoV-2 seroprevalence in habitants of Assam (AASSHA): report of the first serosurvey in Assam. Int J Community Med Public Health. 2021; 8(5): 2490-6.
India, Assam	*
India, Assam	Mordani S. Seropositivity highest in Madhya Pradesh, lowest in Kerala, finds ICMR's national sero-survey. India Today [Internet]. 2021 Jul 28.
India, Bihar	*
India, Bihar	Mordani S. Seropositivity highest in Madhya Pradesh, lowest in Kerala, finds ICMR's national sero-survey. India Today [Internet]. 2021 Jul 28.
India, Chhattisgarh	*
India, Chhattisgarh	Mordani S. Seropositivity highest in Madhya Pradesh, lowest in Kerala, finds ICMR's national sero-survey. India Today [Internet]. 2021 Jul 28.
India, Delhi	Misra P, Kant S, Guleria R, Rai SK, WHO Unity Seroprevalence study team of AIIMS. Serological prevalence of SARS-CoV-2 antibody among children and young age (between age 2-17 years) group in India: An interim result from a large multi-centric population-based seroepidemiological study. Preprint. medRxiv. 2021.
India, Delhi	Jain P. Over 56% of Delhi has Covid-19 Antibodies: Health Minister Satyendar Jain on 5th Serosurvey. India Today [Internet]. 2021 Feb 2.
India, Delhi	AlokKNMishra. Covid antibodies found in 97% of Delhi residents. Times of India [Internet]. 2021 Oct 29; Delhi News.
India, Delhi	Sharma N, Sharma P, Basu S, Saxena S, Chawla R, Dushyant K, Mundeja N, Marak ZS, Singh S, Singh GK, Rustagi R. The seroprevalence and trends of SARS-CoV-2 in Delhi, India: A repeated population-based seroepidemiological study. Preprint. medRxiv. 2020
India, Gujarat	Singh PP, Tamang R, Shukla M, Pathak A, Srivastava A, Gupta P, Bhatt A, Shrivastava AK, Upadhyay SK, Singh A, Maurya S, Saxena P, Singh V, Chaubey AK, Mishra DK, Patel Y, Pandey RK, Srivastava A, Khanam N, Das D, Bandopadhyay A, Chorol U, Pasupuleti N, Kumar S, Prakash S, Mishra A, Dubey PK, Parihar A, Basu P, Sequeira JJ, Kc L, Vijayalaxmi V, BhatK VS, Ijinu TP, Aggarwal DD, Prakash A, Yadav K, Yadav A, Upadhyay V, Mukim G, Bhandari A, Ghosh A, Kumar A, Yadav VK, Nigam K, Harshey A, Das T, Devadas D, Mishra SP, Kumar A, Yadav AK, Singh NK, Kaur M, Kumar S, Srivastava N, Sharma C, Chowdhury R, Jain D, Kumar A, Shukla R, Mishra RK, Singh R, Tripathi YB, Mishra VN, Mustak MS, Rai N, Rawat SK, Survajhala P, Singh KK, Mallick CB, Shrivastava P, Chaubey G. Estimation of real-infection and immunity against SARS-CoV-2 in Indian populations. Preprint. medRxiv. 2021.
India, Gujarat	Mordani S. Seropositivity highest in Madhya Pradesh, lowest in Kerala, finds ICMR's national sero-survey. India Today [Internet]. 2021 Jul 28.
India, Haryana	Health Department, Haryana (India). India - Haryana Covid -19 Sero Survey Round 1, August 2020. India: Health Department, Haryana (India).
India, Haryana	Health Department, Haryana (India). India - Haryana Covid-19 Sero Survey Round 2, October 2020.
India, Haryana	Health Department, Haryana (India). India - Haryana Covid-19 Sero Survey Round 3, September 2021.
India, Haryana	Mordani S. Seropositivity highest in Madhya Pradesh, lowest in Kerala, finds ICMR's national sero-survey. India Today [Internet]. 2021 Jul 28.
India, Himachal Pradesh	Mordani S. Seropositivity highest in Madhya Pradesh, lowest in Kerala, finds ICMR's national sero-survey. India Today [Internet]. 2021 Jul 28.
India, Jammu & Kashmir and Ladakh	Mordani S. Seropositivity highest in Madhya Pradesh, lowest in Kerala, finds ICMR's national sero-survey. India Today [Internet]. 2021 Jul 28.
India, Jharkhand	*
India, Jharkhand	Mordani S. Seropositivity highest in Madhya Pradesh, lowest in Kerala, finds ICMR's national sero-survey. India Today [Internet]. 2021 Jul 28.
India, Jharkhand	The Times of India. Jharkhand: 45% of population in 10 districts positive before 2nd wave: Sero survey. Mumbai, India: The Times of India, 2021.
India, Karnataka	Singh PP, Tamang R, Shukla M, Pathak A, Srivastava A, Gupta P, Bhatt A, Shrivastava AK, Upadhyay SK, Singh A, Maurya S, Saxena P, Singh V, Chaubey AK, Mishra DK, Patel Y, Pandey RK, Srivastava A, Khanam N, Das D, Bandopadhyay A, Chorol U, Pasupuleti N, Kumar S, Prakash S, Mishra A, Dubey PK, Parihar A, Basu P, Sequeira JJ, Kc

	L, Vijayalaxmi V, BhatK VS, Ijinu TP, Aggarwal DD, Prakash A, Yadav K, Yadav A, Upadhyay V, Mukim G, Bhandari A, Ghosh A, Kumar A, Yadav VK, Nigam K, Harshey A, Das T, Devadas D, Mishra SP, Kumar A, Yadav AK, Singh NK, Kaur M, Kumar S, Srivastava N, Sharma C, Chowdhury R, Jain D, Kumar A, Shukla R, Mishra RK, Singh R, Tripathi YB, Mishra VN, Mustak MS, Rai N, Rawat SK, Survajhala P, Singh KK, Mallick CB, Shrivastava P, Chaubey G. Estimation of real-infection and immunity against SARS-CoV-2 in Indian populations. Preprint. medRxiv. 2021.
India, Karnataka	*
India, Karnataka	Mordani S. Seropositivity highest in Madhya Pradesh, lowest in Kerala, finds ICMR's national sero-survey. India Today [Internet]. 2021 Jul 28.
India, Karnataka	Babu GR, Sundaresan R, Athreya S, Akhtar J, Pandey PK, Maroor PS, Padma MR, Lalitha R, Shariff M, Krishnappa L, Manjunath CN, Sudarshan MK, Gururaj G, Ranganath TS, Vasanth KDE, Banandur P, Ravi D, Shiju S, Lobo E, Satapathy A, Alahari L, Dinesh P, Thakar V, Desai A, Rangaiah A, Munivenkatappa A, S K, Basawarajappa SG, Sreedhara HG, Kc S, B AK, Umar N, Ba M, Vasanthapuram R. The burden of active infection and anti-SARS-CoV-2 IgG antibodies in the general population: Results from a statewide sentinel-based population survey in Karnataka, India. Int J Infect Dis. 2021; 108: 27-36.
India, Kerala	Department of Health and Family Welfare, Government of Kerala (India). India - Kerala COVID 19 Sero Surveillance Report August - September 2021. India: Department of Health and Family Welfare, Government of Kerala (India), 2021.
India, Kerala	*
India, Kerala	Mordani S. Seropositivity highest in Madhya Pradesh, lowest in Kerala, finds ICMR's national sero-survey. India Today [Internet]. 2021 Jul 28.
India, Madhya Pradesh	Singh PP, Tamang R, Shukla M, Pathak A, Srivastava A, Gupta P, Bhatt A, Shrivastava AK, Upadhyay SK, Singh A, Maurya S, Saxena P, Singh V, Chaubey AK, Mishra DK, Patel Y, Pandey RK, Srivastava A, Khanam N, Das D, Bandopadhyay A, Chorol U, Pasupuleti N, Kumar S, Prakash S, Mishra A, Dubey PK, Parihar A, Basu P, Sequeira JJ, Kc L, Vijayalaxmi V, BhatK VS, Ijinu TP, Aggarwal DD, Prakash A, Yadav K, Yadav A, Upadhyay V, Mukim G, Bhandari A, Ghosh A, Kumar A, Yadav VK, Nigam K, Harshey A, Das T, Devadas D, Mishra SP, Kumar A, Yadav AK, Singh NK, Kaur M, Kumar S, Srivastava N, Sharma C, Chowdhury R, Jain D, Kumar A, Shukla R, Mishra RK, Singh R, Tripathi YB, Mishra VN, Mustak MS, Rai N, Rawat SK, Survajhala P, Singh KK, Mallick CB, Shrivastava P, Chaubey G. Estimation of real-infection and immunity against SARS-CoV-2 in Indian populations. Preprint. medRxiv. 2021.
India, Madhya Pradesh	Mordani S. Seropositivity highest in Madhya Pradesh, lowest in Kerala, finds ICMR's national sero-survey. India Today [Internet]. 2021 Jul 28.
India, Maharashtra	*
India, Maharashtra	Mordani S. Seropositivity highest in Madhya Pradesh, lowest in Kerala, finds ICMR's national sero-survey. India Today [Internet]. 2021 Jul 28.
India, Odisha	Misra P, Kant S, Guleria R, Rai SK, WHO Unity Seroprevalence study team of AIIMS. Serological prevalence of SARS-CoV-2 antibody among children and young age (between age 2-17 years) group in India: An interim result from a large multi-centric population-based seroepidemiological study. Preprint. medRxiv. 2021.
India, Odisha	*
India, Odisha	Mordani S. Seropositivity highest in Madhya Pradesh, lowest in Kerala, finds ICMR's national sero-survey. India Today [Internet]. 2021 Jul 28.
India, Punjab	Mordani S. Seropositivity highest in Madhya Pradesh, lowest in Kerala, finds ICMR's national sero-survey. India Today [Internet]. 2021 Jul 28.
India, Rajasthan	Mordani S. Seropositivity highest in Madhya Pradesh, lowest in Kerala, finds ICMR's national sero-survey. India Today [Internet]. 2021 Jul 28.
India, Tamil Nadu	Selvavinayagam TS, Somasundaram A, Selvam JM, Ramachandran S, Sampath P, Vijayalakshmi V, Kumar CAB, Subramaniam S, Raju S, Avudaiselvi R, Prakash V, Yogananth N, Subramanian G, Roshini A, Dhiliban DN, Imad S, Tandel V, Parasa R, Sachdeva S, Malani A. Seroprevalence in Tamil Nadu through Indiaâ€™s two COVID waves: Evidence on antibody decline following infection and vaccination. Preprint. medRxiv. 2021.
India, Tamil Nadu	*
India, Tamil Nadu	Mordani S. Seropositivity highest in Madhya Pradesh, lowest in Kerala, finds ICMR's national sero-survey. India Today [Internet]. 2021 Jul 28.
India, Telangana	Mordani S. Seropositivity highest in Madhya Pradesh, lowest in Kerala, finds ICMR's national sero-survey. India Today [Internet]. 2021 Jul 28.
India, Telangana	Naushin S, Sardana V, Ujjainiya R, et al. Insights from a Pan India Sero-Epidemiological survey (Phenome-India Cohort) for SARS-CoV2. Elife. 2021; 10: e66537.
India, Tripura	Deb D. 33.98% of Tripura has COVID-19 antibody in them, shows sero survey. The Indian Express [Internet]. 2020 Nov 12; North East India.
India, Tripura	Misra P, Kant S, Guleria R, Rai SK, WHO Unity Seroprevalence study team of AIIMS. Serological prevalence of SARS-CoV-2 antibody among children and young age (between age 2-17 years) group in India: An interim result from a large multi-centric population-based seroepidemiological study. Preprint. medRxiv. 2021.
India, Uttar Pradesh	Singh PP, Tamang R, Shukla M, Pathak A, Srivastava A, Gupta P, Bhatt A, Shrivastava AK, Upadhyay SK, Singh A, Maurya S, Saxena P, Singh V, Chaubey AK, Mishra DK, Patel Y, Pandey RK, Srivastava A, Khanam N, Das D, Bandopadhyay A, Chorol U, Pasupuleti N, Kumar S, Prakash S, Mishra A, Dubey PK, Parihar A, Basu P, Sequeira JJ, Kc L, Vijayalaxmi V, BhatK VS, Ijinu TP, Aggarwal DD, Prakash A, Yadav K, Yadav A, Upadhyay V, Mukim G, Bhandari A, Ghosh A, Kumar A, Yadav VK, Nigam K, Harshey A, Das T, Devadas D, Mishra SP, Kumar A, Yadav AK, Singh NK, Kaur M, Kumar S, Srivastava N, Sharma C, Chowdhury R, Jain D, Kumar A, Shukla R, Mishra RK, Singh R,

	Tripathi YB, Mishra VN, Mustak MS, Rai N, Rawat SK, Survajhala P, Singh KK, Mallick CB, Shrivastava P, Chaubey G. Estimation of real-infection and immunity against SARS-CoV-2 in Indian populations. Preprint. medRxiv. 2021.
India, Uttar Pradesh	Misra P, Kant S, Guleria R, Rai SK, WHO Unity Seroprevalence study team of AIIMS. Serological prevalence of SARS-CoV-2 antibody among children and young age (between age 2-17 years) group in India: An interim result from a large multi-centric population-based seroepidemiological study. Preprint. medRxiv. 2021.
India, Uttar Pradesh	*
India, Uttar Pradesh	Mordani S. Seropositivity highest in Madhya Pradesh, lowest in Kerala, finds ICMR's national sero-survey. India Today [Internet]. 2021 Jul 28.
India, Uttarakhand	Mordani S. Seropositivity highest in Madhya Pradesh, lowest in Kerala, finds ICMR's national sero-survey. India Today [Internet]. 2021 Jul 28.
India, West Bengal	Kolkata: One in Four People Have Covid Antibodies. The Statesman [Internet]. 2020 Sept 10.
India, West Bengal	Gautham, K. One in Three Exposed to Covid Virus, Reveals Second Chennai Sero Survey . The Times of India [Internet]. 2020 Oct 22.
India, West Bengal	*
India, West Bengal	Mordani S. Seropositivity highest in Madhya Pradesh, lowest in Kerala, finds ICMR's national sero-survey. India Today [Internet]. 2021 Jul 28.
Iran	Khalagi K, Gharibzadeh S, Khalili D, Mansournia MA, Mirab Samiee S, Aghamohamadi S, Mir-Mohammad-Ali Roodaki M, Hashemi SM, Tayeri K, Namdari Tabar H, Azadmanesh K, Tabrizi JS, Mohammad K, Hajipour F, Namaki S, Raeisi A, Ostovar A. Prevalence of COVID-19 in Iran: results of the first survey of the Iranian COVID-19 Serological Surveillance programme. Clin Microbiol Infect. 2021.
Ireland	Butler D, Coyne D, Pomeroy L, Williams P, Holder P, Carterson A, Field S, Waters A, O'Flaherty N. Confirmed circulation of SARS-CoV-2 in Irish blood donors prior to first national notification of infection. J Clin Virol. 2021; 146: 105045.
Ireland	Health Service Executive (HSE) (Ireland), University College Dublin. Ireland Study to Investigate COVID-19 Infection in People Living in Ireland (SCOPI) 2020. Dublin, Ireland: Health Protection Surveillance Centre (HPSC) (Ireland), 2020.
Israel	Ministry of Health (Israel). Israel - Press Releases Results of the National Serological Survey for Novel Coronavirus. Jerusalem, Israel: Ministry of Health (Israel), 2020.
Israel	Reicher S, Ratzon R, Ben-Sahar S, Hermoni-Alon S, Mossinson D, Shenhar Y, Friger M, Lustig Y, Alroy-Preis S, Anis E, Sadetzki S, Kaliner E. Nationwide seroprevalence of antibodies against SARS-CoV-2 in Israel. Eur J Epidemiol. 2021.
Italy, Liguria	Ministry of Health (Italy), National Institute of Statistics (Italy). Italy SARS-CoV-2 Seroprevalence Survey, First Results 2020.
Italy, Lombardia	Ministry of Health (Italy), National Institute of Statistics (Italy). Italy SARS-CoV-2 Seroprevalence Survey, First Results 2020.
Italy, Prov. autonoma di Bolzano	Ministry of Health (Italy), National Institute of Statistics (Italy). Italy SARS-CoV-2 Seroprevalence Survey, First Results 2020.
Italy, Prov. autonoma di Trento	Ministry of Health (Italy), National Institute of Statistics (Italy). Italy SARS-CoV-2 Seroprevalence Survey, First Results 2020.
Italy, Valle d'Aosta	Ministry of Health (Italy), National Institute of Statistics (Italy). Italy SARS-CoV-2 Seroprevalence Survey, First Results 2020.
Jordan	Bellizzi S, Alsawalha L, Sheikh Ali S, Sharkas G, Muthu N, Ghazo M, Hayajneh W, Profili MC, Obeidat NM. A three-phase population based sero-epidemiological study: Assessing the trend in prevalence of SARS-CoV-2 during COVID-19 pandemic in Jordan. One Health. 2021; 13: 100292.
Jordan	Sughayer MA, Mansour A, Nuirat AA, Souan L, Ghanem M, Siag M, Alhassoon S. Dramatic Rise of Seroprevalence Rates of SARS-CoV-2 Antibodies among Healthy Blood Donors: The evolution of a Pandemic. Preprint. medRxiv. 2021.
Jordan	Sughayer MA, Mansour A, Nuirat AA, Souan L, Abdel-Razeq R, Siag M. A second dramatic rise in seroprevalence rates of SARS-CoV-2 antibodies among adult healthy blood donors in Jordan; have we achieved herd immunity? Preprint. medRxiv. 2021; p 2021081521261584.
Kenya	Munywoki P, Nasimiyu C, Alando M, Otieno N, Ombok C, Njoroge R, Kikwai G, Odhiambo D, Osita M, Ouma A, Odour C, Juma B, Ochieng C, Mutisya I, Ngere I, Dawa J, Osoro E, Njenga M, Bigogo G, Munyua P, Lo T, Hunsperger E, Herman-Roloff A. Seroprevalence and risk factors of SARS-CoV-2 infection in an urban informal settlement in Nairobi, Kenya, December 2020 [version 1; peer review: 1 approved with reservations]. F1000Res. 2021; 10(853).
Kenya	Nyagwange J, Kutima B, Mwai K, Karanja HK, Gitonga JN, Mugo D, Uyoga S, Tuju J, Ochola-Oyier LI, Ndungu F, Bejon P, Agweyu A, Adetifa IMO, Scott JAG, Warimwe GM. Comparative performance of WANTAI ELISA for total immunoglobulin to receptor binding protein and an ELISA for IgG to spike protein in detecting SARS-CoV-2 antibodies in Kenyan populations. J Clin Virol. 2022; 146: 105061.
Kenya	Uyoga S, Adetifa IMO, Karanja HK, Nyagwange J, Tuju J, Wanjiku P, Aman R, Mwangangi M, Amoth P, Kasera K, Ng'ang'a W, Rombo C, Yegon CK, Kithi K, Odhiambo E, Rotich T, Orgut I, Kihara S, Otiende M, Bottomley C, Mupe ZN, Kagucia EW, Gallagher K, Etyang A, Voller S, Gitonga J, Mugo D, Agoti CN, Otieno E, Ndwiga L, Lambe T, Wright D, Barasa E, Tsofa B, Bejon P, Ochola-Oyier LI, Agweyu A, Scott AGJ, Warimwe GM. Seroprevalence of anti-SARS-CoV-2 IgG antibodies in Kenyan blood donors. Preprint. medRxiv. 2020.
Kenya	Brand SPC, Ojal J, Aziza R, Were V, Okiro EA, Kombe IK, Mburu C, Ogero M, Agweyu A, Warimwe GM, Nyagwange J, Karanja H, Gitonga JN, Mugo D, Uyoga S, Adetifa IMO, Scott JAG, Otieno E, Murunga N, Otiende M, Ochola-Oyier

	LI, Agoti CN, Githinji G, Kasera K, Amoth P, Mwangangi M, Aman R, Ng'ang'a W, Tsofa B, Bejon P, Keeling MJ, Nokes DJ, Barasa E. COVID-19 transmission dynamics underlying epidemic waves in Kenya. Science. 2021; eabk0414.
Kenya	Ngere IA, Dawa J, Hunsperger E, Otieno N, Masika M, Amoth P, Makayotto L, Nasimiyu C, Gunn BM, Nyawanda B, Oluga O, Ngunu C, Mirieri H, Gachohi J, Marwanga D, Munywoki P, Odhiambo D, Alando MD, Breiman R, Anzala O, Njenga MK, Bulterys M, Herman-Roloff A, Osoro E. High Seroprevalence of SARS-CoV-2 Eight Months After Introduction in Nairobi, Kenya. Preprint. Prepr Lancet. 2021.
Kyrgyzstan	Popova AY, Kasymov OT, Smolenski VY, , Egorova SA, Nurmatov ZS, Milichkina AM, Suranbaeva GS, Khamitova IV, Zueva EV, Ivanov VA, Nuridinova ZN, Derkenbaeva A, Drobyshevskaya VG, Sattarova GZ, Gubanova AV, Zhimbaeva OB, Razumovskaya A, Verbov VN, Likhachev IV, Totolian AA. SARS-CoV-2 Herd Immunity of the Kyrgyz Population in 2021. Preprint. Res Sq. 2021.
Madagascar	Razafimahatratra SL, Ndiaye MDB, Rasoloharimanana LT, Dussart P, Sahondranirina PH, Randriamanantany ZA, Schoenhals M. Seroprevalence of ancestral and Beta SARS-CoV-2 antibodies in Malagasy blood donors. Lancet Glob Health. 2021; 9(10): e1363-e1364.
Malawi	Lewis HC, Ware H, Whelan M, Subissi L, Li Z, Ma X, Nardone A, Valenciano M, Cheng B, Noel K, Cao C, Yanes-Lane M, Herring B, Talisuna A, Nsenga N, Balde T, Clifton DA, Van Kerkhove M, Buckeridge DL, Bobrovitz N, Okeibunor J, Arora RK, Bergeri I, the UNITY Studies Collaborator Group. SARS-CoV-2 infection in Africa: A systematic review and meta-analysis of standardised seroprevalence studies, from January 2020 to December 2021. Preprint. medRxiv. 2022.
Malawi	Mandolo J, Msefula J, Henrion MYR, Brown C, Moyo B, Samon A, Moyo-Gwete T, Makhado Z, Ayres F, Motlou T, Mzindle N, Kalata N, Muula AS, Kwatra G, Nsamala N, Likaka A, Mfune T, Moore PL, Mbaya B, French N, Heyderman RS, Swarthout T, Jambo KC. SARS-CoV-2 exposure in Malawian blood donors: an analysis of seroprevalence and variant dynamics between January 2020 and July 2021. BMC Med. 2021; 19(1): 303.
Mali	Sagara I, Woodford J, Kone M, Assadou MH, Katile A, Attaher O, Zeguime A, Doucoure M, Higbee E, Lane J, Doritchamou J, Zaidi I, Esposito D, Kwan J, Sadtler K, Dicko A, Duffy P. Rapidly increasing SARS-CoV-2 seroprevalence and limited clinical disease in three Malian communities: a prospective cohort study. Preprint. medRxiv. 2021.
Mexico	Health Secretary, National Institute of Public Health (Mexico). Preliminary results of the National Health and Nutrition Survey COVID-19. Communiqué 255, 2020 Dec 16.
Mexico	Muñoz-Medina JE, Grajales-Muñiz C, Salas-Lais AG, Fernandes-Matano L, López-Macías C, Monroy-Muñoz IE, Santos Coy-Arechavaleta A, Palomec-Nava ID, Duque-Molina C, Madera-Sandoval RL, Rivero-Arredondo V, González-Ibarra J, Alvarado-Yaah JE, Rojas-Mendoza T, Santacruz-Tinoco CE, González-Bonilla CR, Borja-Aburto VH. SARS-CoV-2 IgG Antibodies Seroprevalence and Sera Neutralizing Activity in MEXICO: A National Cross-Sectional Study during 2020. Microorganisms. 2021; 9(4).
Mexico, Aguascalientes	Government of Mexico, National Institute of Public Health (Mexico). Mexico National Health and Nutrition Survey (ENSANUT) about COVID-19 2020. Cuernavaca, Mexico: National Institute of Public Health (Mexico), 2020.
Mexico, Baja California	Government of Mexico, National Institute of Public Health (Mexico). Mexico National Health and Nutrition Survey (ENSANUT) about COVID-19 2020. Cuernavaca, Mexico: National Institute of Public Health (Mexico), 2020.
Mexico, Baja California Sur	Government of Mexico, National Institute of Public Health (Mexico). Mexico National Health and Nutrition Survey (ENSANUT) about COVID-19 2020. Cuernavaca, Mexico: National Institute of Public Health (Mexico), 2020.
Mexico, Campeche	Government of Mexico, National Institute of Public Health (Mexico). Mexico National Health and Nutrition Survey (ENSANUT) about COVID-19 2020. Cuernavaca, Mexico: National Institute of Public Health (Mexico), 2020.
Mexico, Chiapas	Government of Mexico, National Institute of Public Health (Mexico). Mexico National Health and Nutrition Survey (ENSANUT) about COVID-19 2020. Cuernavaca, Mexico: National Institute of Public Health (Mexico), 2020.
Mexico, Chihuahua	Government of Mexico, National Institute of Public Health (Mexico). Mexico National Health and Nutrition Survey (ENSANUT) about COVID-19 2020. Cuernavaca, Mexico: National Institute of Public Health (Mexico), 2020.
Mexico, Coahuila	Government of Mexico, National Institute of Public Health (Mexico). Mexico National Health and Nutrition Survey (ENSANUT) about COVID-19 2020. Cuernavaca, Mexico: National Institute of Public Health (Mexico), 2020.
Mexico, Colima	Government of Mexico, National Institute of Public Health (Mexico). Mexico National Health and Nutrition Survey (ENSANUT) about COVID-19 2020. Cuernavaca, Mexico: National Institute of Public Health (Mexico), 2020.
Mexico, Durango	Government of Mexico, National Institute of Public Health (Mexico). Mexico National Health and Nutrition Survey (ENSANUT) about COVID-19 2020. Cuernavaca, Mexico: National Institute of Public Health (Mexico), 2020.
Mexico, Guanajuato	Government of Mexico, National Institute of Public Health (Mexico). Mexico National Health and Nutrition Survey (ENSANUT) about COVID-19 2020. Cuernavaca, Mexico: National Institute of Public Health (Mexico), 2020.
Mexico, Guerrero	Government of Mexico, National Institute of Public Health (Mexico). Mexico National Health and Nutrition Survey (ENSANUT) about COVID-19 2020. Cuernavaca, Mexico: National Institute of Public Health (Mexico), 2020.
Mexico, Hidalgo	Government of Mexico, National Institute of Public Health (Mexico). Mexico National Health and Nutrition Survey (ENSANUT) about COVID-19 2020. Cuernavaca, Mexico: National Institute of Public Health (Mexico), 2020.
Mexico, Jalisco	Government of Mexico, National Institute of Public Health (Mexico). Mexico National Health and Nutrition Survey (ENSANUT) about COVID-19 2020. Cuernavaca, Mexico: National Institute of Public Health (Mexico), 2020.
Mexico, México	Government of Mexico, National Institute of Public Health (Mexico). Mexico National Health and Nutrition Survey (ENSANUT) about COVID-19 2020. Cuernavaca, Mexico: National Institute of Public Health (Mexico), 2020.
Mexico, Mexico City	Government of Mexico, National Institute of Public Health (Mexico). Mexico National Health and Nutrition Survey (ENSANUT) about COVID-19 2020. Cuernavaca, Mexico: National Institute of Public Health (Mexico), 2020.
Mexico, Michoacán de Ocampo	Government of Mexico, National Institute of Public Health (Mexico). Mexico National Health and Nutrition Survey (ENSANUT) about COVID-19 2020. Cuernavaca, Mexico: National Institute of Public Health (Mexico), 2020.
Mexico, Morelos	Government of Mexico, National Institute of Public Health (Mexico). Mexico National Health and Nutrition Survey (ENSANUT) about COVID-19 2020. Cuernavaca, Mexico: National Institute of Public Health (Mexico), 2020.
Mexico, Nayarit	Government of Mexico, National Institute of Public Health (Mexico). Mexico National Health and Nutrition Survey (ENSANUT) about COVID-19 2020. Cuernavaca, Mexico: National Institute of Public Health (Mexico), 2020.

Mexico, Nuevo León	Government of Mexico, National Institute of Public Health (Mexico). Mexico National Health and Nutrition Survey (ENSANUT) about COVID-19 2020. Cuernavaca, Mexico: National Institute of Public Health (Mexico), 2020.
Mexico, Oaxaca	Government of Mexico, National Institute of Public Health (Mexico). Mexico National Health and Nutrition Survey (ENSANUT) about COVID-19 2020. Cuernavaca, Mexico: National Institute of Public Health (Mexico), 2020.
Mexico, Puebla	Government of Mexico, National Institute of Public Health (Mexico). Mexico National Health and Nutrition Survey (ENSANUT) about COVID-19 2020. Cuernavaca, Mexico: National Institute of Public Health (Mexico), 2020.
Mexico, Querétaro	Government of Mexico, National Institute of Public Health (Mexico). Mexico National Health and Nutrition Survey (ENSANUT) about COVID-19 2020. Cuernavaca, Mexico: National Institute of Public Health (Mexico), 2020.
Mexico, Quintana Roo	Government of Mexico, National Institute of Public Health (Mexico). Mexico National Health and Nutrition Survey (ENSANUT) about COVID-19 2020. Cuernavaca, Mexico: National Institute of Public Health (Mexico), 2020.
Mexico, San Luis Potosí	Government of Mexico, National Institute of Public Health (Mexico). Mexico National Health and Nutrition Survey (ENSANUT) about COVID-19 2020. Cuernavaca, Mexico: National Institute of Public Health (Mexico), 2020.
Mexico, Sinaloa	Government of Mexico, National Institute of Public Health (Mexico). Mexico National Health and Nutrition Survey (ENSANUT) about COVID-19 2020. Cuernavaca, Mexico: National Institute of Public Health (Mexico), 2020.
Mexico, Sonora	Government of Mexico, National Institute of Public Health (Mexico). Mexico National Health and Nutrition Survey (ENSANUT) about COVID-19 2020. Cuernavaca, Mexico: National Institute of Public Health (Mexico), 2020.
Mexico, Tabasco	Government of Mexico, National Institute of Public Health (Mexico). Mexico National Health and Nutrition Survey (ENSANUT) about COVID-19 2020. Cuernavaca, Mexico: National Institute of Public Health (Mexico), 2020.
Mexico, Tamaulipas	Government of Mexico, National Institute of Public Health (Mexico). Mexico National Health and Nutrition Survey (ENSANUT) about COVID-19 2020. Cuernavaca, Mexico: National Institute of Public Health (Mexico), 2020.
Mexico, Tlaxcala	Government of Mexico, National Institute of Public Health (Mexico). Mexico National Health and Nutrition Survey (ENSANUT) about COVID-19 2020. Cuernavaca, Mexico: National Institute of Public Health (Mexico), 2020.
Mexico, Veracruz de Ignacio de la Llave	Government of Mexico, National Institute of Public Health (Mexico). Mexico National Health and Nutrition Survey (ENSANUT) about COVID-19 2020. Cuernavaca, Mexico: National Institute of Public Health (Mexico), 2020.
Mexico, Yucatán	Government of Mexico, National Institute of Public Health (Mexico). Mexico National Health and Nutrition Survey (ENSANUT) about COVID-19 2020. Cuernavaca, Mexico: National Institute of Public Health (Mexico), 2020.
Mexico, Zacatecas	Government of Mexico, National Institute of Public Health (Mexico). Mexico National Health and Nutrition Survey (ENSANUT) about COVID-19 2020. Cuernavaca, Mexico: National Institute of Public Health (Mexico), 2020.
Nepal	Ministry of Health and Population (Nepal), National Public Health Laboratory (Nepal), World Health Organization (WHO), World Health Organization Regional Office for South-East Asia (SEARO). Nepal National Seroprevalence Survey for COVID-19 2021 Round 2.
Nepal	Ministry of Health and Population (Nepal), National Public Health Laboratory (Nepal), World Health Organization (WHO), World Health Organization Regional Office for South-East Asia (SEARO). Nepal National Sero-Prevalence Survey for COVID-19 2020 Round 1.
Netherlands	Vos ERA, den Hartog G, Schepp RM, Kaaijk P, van Vliet J, Helm K, Smits G, Wijmenga-Monsuur A, Verberk JDM, van Boven M, van Binnendijk RS, de Melker HE, Mollema L, van der Klis FRM. Nationwide seroprevalence of SARS-CoV-2 and identification of risk factors in the general population of the Netherlands during the first epidemic wave. J Epidemiol Community Health. 2020.
Netherlands	Vos ERA, van Boven M, den Hartog G, Backer JA, Klinkenberg D, van Hagen CCE, Boshuizen H, van Binnendijk RS, Mollema L, van der Klis FRM, de Melker HE. Associations between measures of social distancing and SARS-CoV-2 seropositivity: a nationwide population-based study in the Netherlands. Clin Infect Dis. 2021.
Netherlands	Slot E, Hogema BM, Reusken CBEM, Reimerink JH, Molier M, Karregat JHM, IJlst J, Novotný VMJ, van Lier RAW, Zaaijer HL. Low SARS-CoV-2 seroprevalence in blood donors in the early COVID-19 epidemic in the Netherlands. Nat Commun. 2020; 11(1): 5744.
Netherlands	Sanquin (Netherlands). Netherlands - Coronavirus antibodies in an average of 18.6% of the donors. Sanquin (Netherlands) [Internet]. 17 Mar 2021; News.
Nigeria	Ifeorah I, Nna E, Okeke U et al. Sero-pravelence of SARS CoV-2 IgM and IgG Antibodies Amongst Blood Donors in Nigeria. Preprint. Res Sq. 2021.
Nigeria	Lewis HC, Ware H, Whelan M, Subissi L, Li Z, Ma X, Nardone A, Valenciano M, Cheng B, Noel K, Cao C, Yanes-Lane M, Herring B, Talisuna A, Nsenga N, Balde T, Clifton DA, Van Kerkhove M, Buckeridge DL, Bobrovitz N, Okeibunor J, Arora RK, Bergeri I, the UNITY Studies Collaborator Group. SARS-CoV-2 infection in Africa: A systematic review and meta-analysis of standardised seroprevalence studies, from January 2020 to December 2021. Preprint. medRxiv. 2022.
Nigeria	Chechet GD, Kwaga JKP, Yahaya J, MacLeod A, Adamson WE. SARS-CoV-2 seroprevalence in Kaduna State, Nigeria during October/November 2021, following three waves of infection and immediately prior to detection of the Omicron variant. medRxiv. 2021; p 2021122121268166.
Nigeria	Nigeria Centre for Disease Control. Federal Ministry of Health Implements Massive Distribution of Personal Protective Equipment to Primary Healthcare Centres in Nigeria. Nigeria Centre for Disease Control [Internet]. 28 May 2021; News.
Nigeria	Majiya H, Aliyu-Paiko M, Balogu VT, Musa DA, Salihu IM, Kawu AA, Bashir IY, Sani AR, Baba J, Muhammad AT, Jibril FL, Bala E, Obaje NG, Aliyu YB, Muhammad RG, Mohammed H, Gimba UN, Uthman A, Liman HM, Alhaji SA, James JK, Makusidi MM, Isah MD, Abdullahi I, Ndagi U, Waziri B, Bisallah CI, Dadi-Mamud NJ, Ibrahim K, Adamu AK. Seroprevalence of COVID-19 in Niger State. Preprint. medRxiv. 2020.
Norway	Tunheim G., Kran, AB., Rø, G., Hungnes O., Lund-Johansen, F., Tran, T., Andersen JT., Vaage, JT. "Seroprevalence of SARS-CoV-2 in the Norwegian population measured in residual sera collected in late summer 2020". [Seroprevalens av SARS-CoV-2 i den norske befolkningen, målt i restsera samlet inn på sensommeren 2020] Report 2020. Oslo: Norwegian Institute of Public Health, 2020.
Norway	Tunheim G, Rø GØI, Tran T, Kran AB, Andersen JT, Vaage EB, Kolderup A, Vaage JT, Lund-Johansen F, Hungnes O. Trends in seroprevalence of SARS-CoV-2 and infection fatality rate in the Norwegian population through the first year of the COVID-19 pandemic. Influenza Other Respir Viruses. 2021.

Oman	Al-Abri SS, Al-Wahaibi A, Al-Kindi H, Kurup PJ, Al-Maqbali A, Al-Mayahi Z, Al-Tobi MH, Al-Katheri SH, Albusaidi S, Al-Sukaiti MH, Al Balushi AYM, Abdelgadir IO, Al-Shehi N, Morkos E, Al-Maani A, Al-Rawahi B, Alyaquobi F, Alqayoudhi A, Al-Harthy K, Al-Khalili S, Al-Rashdi A, Al-Shukri I, Al Ghafri TS, Al-Hashmi F, Al Jassasi SM, Alshaqsi N, Mitra N, Al Aamry HS, Shah P, Al Marbouai HH, Al Araimi AH, Kair IM, Al Manji AM, Almallak AS, Al Alawi FK, Vaidya V, Muqeetullah M, Alrashdi H, Al Jamoudi SSN, Alshaqsi A, Al Sharji A, Al Shukeiri H, Al-Abri B, Al-Rawahi S, Al-Lamki SH, Al-Manji A, Al-Jardani A. SARS-COV-2 antibody seroprevalence in the general population of Oman: results from four successive nationwide seroepidemiological surveys. Int J Infect Dis. 2021.
Pakistan, Khyber Pakhtunkhwa	Haq M, Rehman A, Ahmad J, Zafar U, Ahmed S, Khan MA, Naveed A, Rajab H, Muhammad F, Naushad W, Aman M, Rehman HU, Ahmad S, Anwar S, Haq NU. SARS-CoV-2: big seroprevalence data from Pakistan-is herd immunity at hand?. Infection. 2021.
Pakistan, Sindh	Haq M, Rehman A, Ahmad J, Zafar U, Ahmed S, Khan MA, Naveed A, Rajab H, Muhammad F, Naushad W, Aman M, Rehman HU, Ahmad S, Anwar S, Haq NU. SARS-CoV-2: big seroprevalence data from Pakistan-is herd immunity at hand?. Infection. 2021.
Peru	Huamaní C, Velásquez L, Montes S, Mayanga-Herrera A, Bernabé-Ortiz A. Population-based seroprevalence of SARS-CoV-2 antibodies in a high-altitude setting in Peru. Preprint. medRxiv. 2021.
Poland	*
Poland	Zejda JE, Brożek GM, Kowalska M, Barański K, Kaleta-Pilarska A, Nowakowski A, Xia Y, Buszman P. Seroprevalence of Anti-SARS-CoV-2 Antibodies in a Random Sample of Inhabitants of the Katowice Region, Poland. Int J Environ Res Public Health. 2021; 18(6).
Portugal	Canto E Castro L, Pereira AHG, Ribeiro R, Alves C, Veloso L, Vicente V, Alves D, Domingues I, Silva C, Gomes A, Serrano M, Afonso Â, Veldhoen M, de Sousa MJR, de Sousa JGR, de Sousa G, Mota MM, Silva-Santos B, Ribeiro RM. Prevalence of SARS-CoV-2 Antibodies after First 6 Months of COVID-19 Pandemic, Portugal. Emerg Infect Dis. 2021; 27(11).
Portugal	Ministry of Health (Portugal). Portugal National Serological Survey COVID-19 Preliminary results 2020. Lisbon, Portugal: Ministry of Health (Portugal), 2020.
Portugal	Ministry of Health (Portugal). Portugal National Serological Survey COVID-19 (2nd phase) 2021. Lisbon, Portugal: Ministry of Health (Portugal), 2020.
Portugal	Castro L, Gomes A, et al. Longitudinal SARS-CoV-2 seroprevalence in Portugal and antibody maintenance 12 months after the start of the COVID-19 pandemic. Preprint. Res Sq. 2021.
Puerto Rico	Puerto Rico Public Health Trust (PRPHT). Puerto Rico Community Assessment for Response to Public Health Emergencies (CASPER) 2020.
Puerto Rico	Centers for Disease Control and Prevention (CDC). CDC COVID Data Tracker Nationwide Blood Donor Seroprevalence Survey. Atlanta, United States of America: Centers for Disease Control and Prevention (CDC).
Puerto Rico	Centers for Disease Control and Prevention (CDC). CDC COVID Data Tracker Commercial Laboratory Seroprevalence Survey Data. Atlanta, United States of America: Centers for Disease Control and Prevention (CDC).
Qatar	Abu-Raddad LJ, Chemaitelly H, Ayoub HH, Al Kanaani Z, Al Khal A, Al Kuwari E, Butt AA, Coyle P, Jeremijenko A, Kaleeckal AH, Latif AN, Owen RC, Rahim HFA, Al Abdulla SA, Al Kuwari MG, Kandy MC, Saeb H, Ahmed SNN, Al Romaihi HE, Bansal D, Dalton L, Al-Thani MH, Bertollini R. Characterizing the Qatar advanced-phase SARS-CoV-2 epidemic. Sci Rep. 2021; 11(1): 6233.
Russia	Popova AY, Andreeva EE, Babura EA, Balakhonov SV, Bashketova NS, Bulanov MV, Valeullina NN, Goryaev DV, Detkovskaya NN, Ezhlova EB, Zaitseva NN, Istorik OA, Kovalchuk IV, Kozlovskikh DN, Kombarova SV, Kurganova OP, Kutyrev VV, Lomovtsev AE, Lukicheva LA, Lyalina LV, Melnikova AA, Mikailova OM, Noskov AK, Noskova LN, Oglezneva EE, Osmolovskay TP, Patyashina MA, Penkovskaya NA, Samoilova LV, Smirnov VS, Stepanova TF, Trotsenko OE, Totolyan AA. [Features of developing SARS-CoV-2 nucleocapsid protein population-based seroprevalence during the first wave of the COVID-19 epidemic in the Russian Federation]. Infektsiia Immun. 2021; 11(2): 297-323.
Russia	Interfax. Popova declared immunity to coronavirus in 14% of those tested. Interfax [Internet]. 2020 Jun 10.
Senegal	Seck SM, Mbow M, Kane Y, Cisse MM, Faye G, Kama A, Sarr M, Nitcheu P, Dahaba M, Diallo IM, Diawara MS, Latou LNM, Dia Y, Mboup S. Prevalence of SARS-CoV-2 Antibodies in Hemodialysis Patients in Senegal: A Multicenter Cross-Sectional Study. Preprint. Res Sq. 2021.
Senegal	Talla C, Loucoubar C, Roka JL, Barry A, Ndiaye S, Diarra M, Faye O, Dia M, Tall A, Ndiaye O, et al. Seroprevalence of Anti-SARS-CoV-2 Antibodies in Senegal: A National Population-Based Cross-Sectional Survey, between October and November 2020. Preprint. Prepr Lancet. 2021.
Slovenia	Poljak M, Oštrbenk Valenčak A, Štrumbelj E, Maver Vodičar P, Vehovar V, Resman Rus K, Korva M, Knap N, Seme K, Petrovec M, Zupan B, Demšar J, Kurdija S, Avšič Županc T. Seroprevalence of severe acute respiratory syndrome coronavirus 2 in Slovenia: results of two rounds of a nationwide population study on a probability-based sample, challenges and lessons learned. Clin Microbiol Infect. 2021.
South Africa	George JA, Khoza S, Mayne E, Dlamini S, Kone N, Jassat W, Chetty K, Centner C, Pillay T, Maphayi MR, Mabuza DV, Maposa I, Cassim N. Sentinel seroprevalence of SARS-CoV-2 in the Gauteng province, South Africa August to October 2020. Preprint. medRxiv. 2021.
South Africa	Mutevedzi PC, Kawonga M, Kwatra G, Moultrie A, Baillie V, Mabena N, Mathibe MN, Rafuma MM, Maposa I, Abbott G, Hugo J, Ikalafeng B, Adelekan T, Lukhele M, Madhi SA. Estimated SARS-CoV-2 infection rate and fatality risk in Gauteng Province, South Africa: a population-based seroepidemiological survey. Int J Epidemiol. 2021.
South Africa	Centers for Disease Control and Prevention (CDC). South Africa COVID-19 Special Public Surveillance Report, 12 March 2021 .
South Africa	Lewis HC, Ware H, Whelan M, Subissi L, Li Z, Ma X, Nardone A, Valenciano M, Cheng B, Noel K, Cao C, Yanes-Lane M, Herring B, Talisuna A, Nsenga N, Balde T, Clifton DA, Van Kerkhove M, Buckeridge DL, Bobrovitz N, Okeibunor J,

	Arora RK, Bergeri I, the UNITY Studies Collaborator Group. SARS-CoV-2 infection in Africa: A systematic review and meta-analysis of standardised seroprevalence studies, from January 2020 to December 2021. Preprint. medRxiv. 2022.
South Africa	Kleynhans J, Tempia S, Wolter N, Gottberg A von, Bhiman JN, Buys A, Moyes J, McMorrow ML, Kahn K, Gómez-Olivé FX, Tollman S, Martinson NA, Wafawanaka F, Lebina L, Toit J du, Jassat W, Neti M, Brauer M, Cohen C, Group for the P-C. Longitudinal SARS-CoV-2 seroprevalence in a rural and urban community household cohort in South Africa, during the first and second waves July 2020-March 2021. Preprint. medRxiv. 2021.
South Africa	Shaw JA, Meiring M, Cummins T, Chegou NN, Claassen C, Du Plessis N, Flinn M, Hiemstra A, Kleynhans L, Leukes V, Loxton AG, MacDonald C, Mtala N, Reuter H, Simon D, Stanley K, Tromp G, Preiser W, Malherbe ST, Walzl G. Higher SARS-CoV-2 seroprevalence in workers with lower socioeconomic status in Cape Town, South Africa. PLoS One. 2021; 16(2): e0247852.
South Africa	Sykes W, Mhlanga L, Swanevelder R, Glatt TN, Grebe E, Coleman C, Pieterson N, Cable R, Welte A, van den Berg K, Vermeulen M. Prevalence of anti-SARS-CoV-2 antibodies among blood donors in Northern Cape, KwaZulu-Natal, Eastern Cape, and Free State provinces of South Africa in January 2021. Preprint. Res Sq. 2021.
South Sudan	Wiens KE, Mawien PN, Rumunu J, Slater D, Jones FK, Moheed S, Caflisch A, Bior BK, Jacob IA, Lako RL, Guyo AG, Olu OO, Maleghemi S, Baguma A, Hassen JJ, Baya SK, Deng L, Lessler J, Demby MN, Sanchez V, Mills R, Fraser C, Charles RC, Harris JB, Azman AS, Wamala JF. Seroprevalence of Severe Acute Respiratory Syndrome Coronavirus 2 IgG in Juba, South Sudan, 20201. Emerg Infect Dis. 2021; 27(6): 1598-1606.
Spain	Government of Spain, Institute of Health Carlos III (Spain). Spain - National Study of Sero-epidemiology of Infection by Sars-Cov-2.
Spain, Andalusia	Government of Spain, Institute of Health Carlos III (Spain). Spain - National Study of Sero-epidemiology of Infection by Sars-Cov-2.
Spain, Aragon	Government of Spain, Institute of Health Carlos III (Spain). Spain - National Study of Sero-epidemiology of Infection by Sars-Cov-2.
Spain, Asturias	Government of Spain, Institute of Health Carlos III (Spain). Spain - National Study of Sero-epidemiology of Infection by Sars-Cov-2.
Spain, Balearic Islands	Government of Spain, Institute of Health Carlos III (Spain). Spain - National Study of Sero-epidemiology of Infection by Sars-Cov-2.
Spain, Basque Country	Government of Spain, Institute of Health Carlos III (Spain). Spain - National Study of Sero-epidemiology of Infection by Sars-Cov-2.
Spain, Canary Islands	Government of Spain, Institute of Health Carlos III (Spain). Spain - National Study of Sero-epidemiology of Infection by Sars-Cov-2.
Spain, Cantabria	Iruzubieta P, Fernández-Lanas T, Rasines L, Cayon L, Álvarez-Cancelo A, Santos-Laso A, García-Blanco A, Curiel-Olmo S, Cabezas J, Wallmann R, Fábrega E, Martínez-Taboada VM, Hernández JL, López-Hoyos M, Lazarus JV, Crespo J. Feasibility of large-scale population testing for SARS-CoV-2 detection by self-testing at home. Sci Rep. 2021; 11(1): 9819.
Spain, Cantabria	Government of Spain, Institute of Health Carlos III (Spain). Spain - National Study of Sero-epidemiology of Infection by Sars-Cov-2.
Spain, Castile and León	Martín MC, González MI, Holgado N, Jimenez AI, Ortega N, Page I, Parrado A, Pérez M, Blanco-Peris L. SARS-CoV-2 seroprevalence and gender-related haematological features in asymptomatic blood donors. Preprint. medRxiv. 2021.
Spain, Castile and León	Government of Spain, Institute of Health Carlos III (Spain). Spain - National Study of Sero-epidemiology of Infection by Sars-Cov-2.
Spain, Castilla–La Mancha	Government of Spain, Institute of Health Carlos III (Spain). Spain - National Study of Sero-epidemiology of Infection by Sars-Cov-2.
Spain, Catalonia	Karachaliou M, Moncunill G, Espinosa A, Vinyals G, Jiménez A, Vidal M, Santano R, Barrios D, Puyol L, Carreras A, Mayer L, Rubio R, Cortés B, Pleguezuelos V, Gordo C, Fossati S, Rivas I, Casabonne D, Vrijheid M, Izquierdo L, Aguilar R, Basagaña X, Aymerich J, Cid R de, Dobaño C, Kogevinas M. SARS-CoV-2 seroprevalence and characteristics of post-infection immunity in a general population cohort study in Catalonia, Spain. Preprint. Res Sq. 2021.
Spain, Catalonia	Government of Spain, Institute of Health Carlos III (Spain). Spain - National Study of Sero-epidemiology of Infection by Sars-Cov-2.
Spain, Ceuta	Government of Spain, Institute of Health Carlos III (Spain). Spain - National Study of Sero-epidemiology of Infection by Sars-Cov-2.
Spain, Community of Madrid	Government of Spain, Institute of Health Carlos III (Spain). Spain - National Study of Sero-epidemiology of Infection by Sars-Cov-2.
Spain, Extremadura	Government of Spain, Institute of Health Carlos III (Spain). Spain - National Study of Sero-epidemiology of Infection by Sars-Cov-2.
Spain, Galicia	Government of Spain, Institute of Health Carlos III (Spain). Spain - National Study of Sero-epidemiology of Infection by Sars-Cov-2.
Spain, La Rioja	Government of Spain, Institute of Health Carlos III (Spain). Spain - National Study of Sero-epidemiology of Infection by Sars-Cov-2.
Spain, Melilla	Government of Spain, Institute of Health Carlos III (Spain). Spain - National Study of Sero-epidemiology of Infection by Sars-Cov-2.
Spain, Murcia	Government of Spain, Institute of Health Carlos III (Spain). Spain - National Study of Sero-epidemiology of Infection by Sars-Cov-2.
Spain, Navarre	Government of Spain, Institute of Health Carlos III (Spain). Spain - National Study of Sero-epidemiology of Infection by Sars-Cov-2.
Spain, Valencian Community	Government of Spain, Institute of Health Carlos III (Spain). Spain - National Study of Sero-epidemiology of Infection by Sars-Cov-2.

Sweden	Public Health Agency of Sweden. Presence of antibodies to SARS-CoV-2 in Sweden, 26 April - 9 May 2021. Östersund, Sweden: Public Health Agency of Sweden.
Sweden	Public Health Agency of Sweden. Detection of Antibodies After Review of Covid-19 in Blood Donors (Sub-report 2). Östersund, Sweden: Public Health Agency of Sweden.
Uganda	Lewis HC, Ware H, Whelan M, Subissi L, Li Z, Ma X, Nardone A, Valenciano M, Cheng B, Noel K, Cao C, Yanes-Lane M, Herring B, Talisuna A, Nsenga N, Balde T, Clifton DA, Van Kerkhove M, Buckeridge DL, Bobrovitz N, Okeibunor J, Arora RK, Bergeri I, the UNITY Studies Collaborator Group. SARS-CoV-2 infection in Africa: A systematic review and meta-analysis of standardised seroprevalence studies, from January 2020 to December 2021. Preprint. medRxiv. 2022.
UK, England	Office for National Statistics (ONS) (United Kingdom). United Kingdom Coronavirus (COVID-19) Infection Survey Pilot 2020. Newport, United Kingdom: Office for National Statistics (ONS) (United Kingdom), 2020.
UK, England	Office for National Statistics (ONS) (United Kingdom). United Kingdom Coronavirus (COVID-19) Infection Survey. Newport, United Kingdom: Office for National Statistics (ONS) (United Kingdom), 2020.
UK, England	Office for National Statistics (ONS) (United Kingdom). England - Coronavirus (COVID-19) Infection Survey: Antibody Data, January 2021. Newport, United Kingdom: Office for National Statistics (ONS) (United Kingdom), 2021.
UK, England	National Health Service (United Kingdom), Public Health England. United Kingdom - England Weekly National COVID-19 Surveillance Report. London, United Kingdom: Government of the United Kingdom.
UK, England	Ward H, Atchison C, Whitaker M, Ainslie KEC, Elliott J, Okell L, Redd R, Ashby D, Donnelly CA, Barclay W, Darzi A, Cooke G, Riley S, Elliott P. SARS-CoV-2 antibody prevalence in England following the first peak of the pandemic. Nat Commun. 2021; 12(905).
UK, England	Ward H, Cooke G, Atchison C, Whitaker M, Elliott J, Moshe M, Brown JC, Flower B, Daunt A, Ainslie K, Ashby D, Donnelly C, Riley S, Darzi A, Barclay W, Elliott P, for the React study team. Declining prevalence of antibody positivity to SARS-CoV-2: a community study of 365,000 adults. Preprint. medRxiv. 2020.
UK, England	Ward H, Whitaker M, Tang SN, Atchison C, Darzi A, Donnelly CA, Diggle PJ, Ashby D, Riley S, Barclay WS, Elliott P, Cooke G. Vaccine uptake and SARS-CoV-2 antibody prevalence among 207,337 adults during May 2021 in England: REACT-2 study. Preprint. medRxiv. 2021.
UK, Northern Ireland	Office for National Statistics (ONS) (United Kingdom). England - Coronavirus (COVID-19) Infection Survey: Antibody Data, January 2021. Newport, United Kingdom: Office for National Statistics (ONS) (United Kingdom), 2021.
UK, Scotland	Office for National Statistics (ONS) (United Kingdom). England - Coronavirus (COVID-19) Infection Survey: Antibody Data, January 2021. Newport, United Kingdom: Office for National Statistics (ONS) (United Kingdom), 2021.
UK, Scotland	Thompson CP, Grayson N, Paton R, Bolton JS, Lourenço J, Penman B, Lee LN, Odon V, Mongkolsapaya J, Chinnakannan S, Dejnirattisai W, Edmans M, Fyfe A, Imlach C, Kooblall K, Lim N, Liu C, Lopez-Camacho C, McInally CA, Ramamurthy N, Ratcliff J, Supasa P, Wang B, Mentzer AJ, Turner M, Sampson O, Semple C, Baillie JK, ISARIC4C Investigators, Harvala H, Screaton G, Temperton N, Klenerman P, Jarvis L, Gupta S, Simmonds P. Detection of neutralising antibodies to SARS coronavirus 2 to determine population exposure in Scottish blood donors between March and May 2020. Preprint. medRxiv. 2020.
UK, Scotland	Public Health Scotland, Scottish National Blood Transfusion Service (SNBTS), The Centre for Virus Research (CVR) at the University of Glasgow. Scotland Enhanced Surveillance of Covid-19 Dashboard. Edinburgh, Scotland: Public Health Scotland.
UK, Wales	Office for National Statistics (ONS) (United Kingdom). England - Coronavirus (COVID-19) Infection Survey: Antibody Data, January 2021. Newport, United Kingdom: Office for National Statistics (ONS) (United Kingdom), 2021.
USA, Alabama	Centers for Disease Control and Prevention (CDC). CDC COVID Data Tracker Nationwide Blood Donor Seroprevalence Survey. Atlanta, United States of America: Centers for Disease Control and Prevention (CDC).
USA, Alabama	Centers for Disease Control and Prevention (CDC). CDC COVID Data Tracker Commercial Laboratory Seroprevalence Survey Data. Atlanta, United States of America: Centers for Disease Control and Prevention (CDC).
USA, Alabama	Anand S, Montez-Rath M, Han J, Bozeman J, Kerschmann R, Beyer P, Parsonnet J, Chertow GM. Prevalence of SARS-CoV-2 antibodies in a large nationwide sample of patients on dialysis in the USA: a cross-sectional study. Lancet. 2020.
USA, Alaska	Centers for Disease Control and Prevention (CDC). CDC COVID Data Tracker Nationwide Blood Donor Seroprevalence Survey. Atlanta, United States of America: Centers for Disease Control and Prevention (CDC).
USA, Alaska	Centers for Disease Control and Prevention (CDC). CDC COVID Data Tracker Commercial Laboratory Seroprevalence Survey Data. Atlanta, United States of America: Centers for Disease Control and Prevention (CDC).
USA, Arizona	Centers for Disease Control and Prevention (CDC). CDC COVID Data Tracker Nationwide Blood Donor Seroprevalence Survey. Atlanta, United States of America: Centers for Disease Control and Prevention (CDC).
USA, Arizona	Centers for Disease Control and Prevention (CDC). CDC COVID Data Tracker Commercial Laboratory Seroprevalence Survey Data. Atlanta, United States of America: Centers for Disease Control and Prevention (CDC).
USA, Arizona	Anand S, Montez-Rath M, Han J, Bozeman J, Kerschmann R, Beyer P, Parsonnet J, Chertow GM. Prevalence of SARS-CoV-2 antibodies in a large nationwide sample of patients on dialysis in the USA: a cross-sectional study. Lancet. 2020.
USA, Arkansas	Centers for Disease Control and Prevention (CDC). CDC COVID Data Tracker Nationwide Blood Donor Seroprevalence Survey. Atlanta, United States of America: Centers for Disease Control and Prevention (CDC).
USA, Arkansas	Centers for Disease Control and Prevention (CDC). CDC COVID Data Tracker Commercial Laboratory Seroprevalence Survey Data. Atlanta, United States of America: Centers for Disease Control and Prevention (CDC).
USA, Arkansas	Anand S, Montez-Rath M, Han J, Bozeman J, Kerschmann R, Beyer P, Parsonnet J, Chertow GM. Prevalence of SARS-CoV-2 antibodies in a large nationwide sample of patients on dialysis in the USA: a cross-sectional study. Lancet. 2020.
USA, California	Centers for Disease Control and Prevention (CDC). CDC COVID Data Tracker Nationwide Blood Donor Seroprevalence Survey. Atlanta, United States of America: Centers for Disease Control and Prevention (CDC).
USA, California	Centers for Disease Control and Prevention (CDC). CDC COVID Data Tracker Commercial Laboratory Seroprevalence Survey Data. Atlanta, United States of America: Centers for Disease Control and Prevention (CDC).

USA, California	Anand S, Montez-Rath M, Han J, Bozeman J, Kerschmann R, Beyer P, Parsonnet J, Chertow GM. Prevalence of SARS-CoV-2 antibodies in a large nationwide sample of patients on dialysis in the USA: a cross-sectional study. Lancet. 2020.
USA, Colorado	Centers for Disease Control and Prevention (CDC). CDC COVID Data Tracker Nationwide Blood Donor Seroprevalence Survey. Atlanta, United States of America: Centers for Disease Control and Prevention (CDC).
USA, Colorado	Centers for Disease Control and Prevention (CDC). CDC COVID Data Tracker Commercial Laboratory Seroprevalence Survey Data. Atlanta, United States of America: Centers for Disease Control and Prevention (CDC).
USA, Connecticut	Centers for Disease Control and Prevention (CDC). CDC COVID Data Tracker Nationwide Blood Donor Seroprevalence Survey. Atlanta, United States of America: Centers for Disease Control and Prevention (CDC).
USA, Connecticut	Havers FP, Reed C, Lim T, Montgomery JM, Klena JD, Hall AJ, Fry AM, Cannon DL, Chiang CF, Gibbons A, Krapiunaya I, Morales-Betoulle M, Roguski K, Rasheed MAU, Freeman B, Lester S, Mills L, Carroll DS, Owen SM, Johnson JA, Semenova V, Blackmore C, Blog D, Chai SJ, Dunn A, Hand J, Jain S, Lindquist S, Lynfield R, Pritchard S, Sokol T, Sosa L, Turabelidze G, Watkins SM, Wiesman J, Williams RW, Yendell S, Schiffer J, Thornburg NJ. Seroprevalence of Antibodies to SARS-CoV-2 in 10 Sites in the United States, March 23-May 12, 2020. JAMA Intern Med. 2020.
USA, Connecticut	Centers for Disease Control and Prevention (CDC). CDC COVID Data Tracker Commercial Laboratory Seroprevalence Survey Data. Atlanta, United States of America: Centers for Disease Control and Prevention (CDC).
USA, Connecticut	Anand S, Montez-Rath M, Han J, Bozeman J, Kerschmann R, Beyer P, Parsonnet J, Chertow GM. Prevalence of SARS-CoV-2 antibodies in a large nationwide sample of patients on dialysis in the USA: a cross-sectional study. Lancet. 2020.
USA, Delaware	Centers for Disease Control and Prevention (CDC). CDC COVID Data Tracker Nationwide Blood Donor Seroprevalence Survey. Atlanta, United States of America: Centers for Disease Control and Prevention (CDC).
USA, Delaware	Centers for Disease Control and Prevention (CDC). CDC COVID Data Tracker Commercial Laboratory Seroprevalence Survey Data. Atlanta, United States of America: Centers for Disease Control and Prevention (CDC).
USA, Delaware	Anand S, Montez-Rath M, Han J, Bozeman J, Kerschmann R, Beyer P, Parsonnet J, Chertow GM. Prevalence of SARS-CoV-2 antibodies in a large nationwide sample of patients on dialysis in the USA: a cross-sectional study. Lancet. 2020.
USA, Florida	Centers for Disease Control and Prevention (CDC). CDC COVID Data Tracker Nationwide Blood Donor Seroprevalence Survey. Atlanta, United States of America: Centers for Disease Control and Prevention (CDC).
USA, Florida	Centers for Disease Control and Prevention (CDC). CDC COVID Data Tracker Commercial Laboratory Seroprevalence Survey Data. Atlanta, United States of America: Centers for Disease Control and Prevention (CDC).
USA, Florida	Anand S, Montez-Rath M, Han J, Bozeman J, Kerschmann R, Beyer P, Parsonnet J, Chertow GM. Prevalence of SARS-CoV-2 antibodies in a large nationwide sample of patients on dialysis in the USA: a cross-sectional study. Lancet. 2020.
USA, Georgia	Centers for Disease Control and Prevention (CDC). CDC COVID Data Tracker Nationwide Blood Donor Seroprevalence Survey. Atlanta, United States of America: Centers for Disease Control and Prevention (CDC).
USA, Georgia	Centers for Disease Control and Prevention (CDC). CDC COVID Data Tracker Commercial Laboratory Seroprevalence Survey Data. Atlanta, United States of America: Centers for Disease Control and Prevention (CDC).
USA, Georgia	Anand S, Montez-Rath M, Han J, Bozeman J, Kerschmann R, Beyer P, Parsonnet J, Chertow GM. Prevalence of SARS-CoV-2 antibodies in a large nationwide sample of patients on dialysis in the USA: a cross-sectional study. Lancet. 2020.
USA, Hawaii	Centers for Disease Control and Prevention (CDC). CDC COVID Data Tracker Nationwide Blood Donor Seroprevalence Survey. Atlanta, United States of America: Centers for Disease Control and Prevention (CDC).
USA, Hawaii	Centers for Disease Control and Prevention (CDC). CDC COVID Data Tracker Commercial Laboratory Seroprevalence Survey Data. Atlanta, United States of America: Centers for Disease Control and Prevention (CDC).
USA, Idaho	Centers for Disease Control and Prevention (CDC). CDC COVID Data Tracker Nationwide Blood Donor Seroprevalence Survey. Atlanta, United States of America: Centers for Disease Control and Prevention (CDC).
USA, Idaho	Centers for Disease Control and Prevention (CDC). CDC COVID Data Tracker Commercial Laboratory Seroprevalence Survey Data. Atlanta, United States of America: Centers for Disease Control and Prevention (CDC).
USA, Illinois	Centers for Disease Control and Prevention (CDC). CDC COVID Data Tracker Nationwide Blood Donor Seroprevalence Survey. Atlanta, United States of America: Centers for Disease Control and Prevention (CDC).
USA, Illinois	Centers for Disease Control and Prevention (CDC). CDC COVID Data Tracker Commercial Laboratory Seroprevalence Survey Data. Atlanta, United States of America: Centers for Disease Control and Prevention (CDC).
USA, Indiana	Centers for Disease Control and Prevention (CDC). CDC COVID Data Tracker Nationwide Blood Donor Seroprevalence Survey. Atlanta, United States of America: Centers for Disease Control and Prevention (CDC).
USA, Indiana	Centers for Disease Control and Prevention (CDC). CDC COVID Data Tracker Commercial Laboratory Seroprevalence Survey Data. Atlanta, United States of America: Centers for Disease Control and Prevention (CDC).
USA, Indiana	Menachemi N, Yiannoutsos CT, Dixon BE, Duszynski TJ, Fadel WF, Wools-Kaloustian KK, Unruh Needleman N, Box K, Caine V, Norwood C, Weaver L, Halverson PK. Population Point Prevalence of SARS-CoV-2 Infection Based on a Statewide Random Sample - Indiana, April 25-29, 2020. MMWR Morb Mortal Wkly Rep. 2020; 69(29): 960-964.
USA, Indiana	Anand S, Montez-Rath M, Han J, Bozeman J, Kerschmann R, Beyer P, Parsonnet J, Chertow GM. Prevalence of SARS-CoV-2 antibodies in a large nationwide sample of patients on dialysis in the USA: a cross-sectional study. Lancet. 2020.
USA, Iowa	Centers for Disease Control and Prevention (CDC). CDC COVID Data Tracker Nationwide Blood Donor Seroprevalence Survey. Atlanta, United States of America: Centers for Disease Control and Prevention (CDC).
USA, Iowa	Centers for Disease Control and Prevention (CDC). CDC COVID Data Tracker Commercial Laboratory Seroprevalence Survey Data. Atlanta, United States of America: Centers for Disease Control and Prevention (CDC).
USA, Kansas	Centers for Disease Control and Prevention (CDC). CDC COVID Data Tracker Nationwide Blood Donor Seroprevalence Survey. Atlanta, United States of America: Centers for Disease Control and Prevention (CDC).
USA, Kansas	Centers for Disease Control and Prevention (CDC). CDC COVID Data Tracker Commercial Laboratory Seroprevalence Survey Data. Atlanta, United States of America: Centers for Disease Control and Prevention (CDC).
USA, Kentucky	Centers for Disease Control and Prevention (CDC). CDC COVID Data Tracker Nationwide Blood Donor Seroprevalence Survey. Atlanta, United States of America: Centers for Disease Control and Prevention (CDC).

USA, Kentucky	Centers for Disease Control and Prevention (CDC). CDC COVID Data Tracker Commercial Laboratory Seroprevalence Survey Data. Atlanta, United States of America: Centers for Disease Control and Prevention (CDC).
USA, Louisiana	Centers for Disease Control and Prevention (CDC). CDC COVID Data Tracker Nationwide Blood Donor Seroprevalence Survey. Atlanta, United States of America: Centers for Disease Control and Prevention (CDC).
USA, Louisiana	Havers FP, Reed C, Lim T, Montgomery JM, Klena JD, Hall AJ, Fry AM, Cannon DL, Chiang CF, Gibbons A, Krapiunaya I, Morales-Betoulle M, Roguski K, Rasheed MAU, Freeman B, Lester S, Mills L, Carroll DS, Owen SM, Johnson JA, Semenova V, Blackmore C, Blog D, Chai SJ, Dunn A, Hand J, Jain S, Lindquist S, Lynfield R, Pritchard S, Sokol T, Sosa L, Turabelidze G, Watkins SM, Wiesman J, Williams RW, Yendell S, Schiffer J, Thornburg NJ. Seroprevalence of Antibodies to SARS-CoV-2 in 10 Sites in the United States, March 23-May 12, 2020. JAMA Intern Med. 2020.
USA, Louisiana	Centers for Disease Control and Prevention (CDC). CDC COVID Data Tracker Commercial Laboratory Seroprevalence Survey Data. Atlanta, United States of America: Centers for Disease Control and Prevention (CDC).
USA, Maine	Centers for Disease Control and Prevention (CDC). CDC COVID Data Tracker Nationwide Blood Donor Seroprevalence Survey. Atlanta, United States of America: Centers for Disease Control and Prevention (CDC).
USA, Maine	Centers for Disease Control and Prevention (CDC). CDC COVID Data Tracker Commercial Laboratory Seroprevalence Survey Data. Atlanta, United States of America: Centers for Disease Control and Prevention (CDC).
USA, Maryland	Centers for Disease Control and Prevention (CDC). CDC COVID Data Tracker Nationwide Blood Donor Seroprevalence Survey. Atlanta, United States of America: Centers for Disease Control and Prevention (CDC).
USA, Maryland	Centers for Disease Control and Prevention (CDC). CDC COVID Data Tracker Commercial Laboratory Seroprevalence Survey Data. Atlanta, United States of America: Centers for Disease Control and Prevention (CDC).
USA, Massachusetts	Centers for Disease Control and Prevention (CDC). CDC COVID Data Tracker Nationwide Blood Donor Seroprevalence Survey. Atlanta, United States of America: Centers for Disease Control and Prevention (CDC).
USA, Massachusetts	Centers for Disease Control and Prevention (CDC). CDC COVID Data Tracker Commercial Laboratory Seroprevalence Survey Data. Atlanta, United States of America: Centers for Disease Control and Prevention (CDC).
USA, Michigan	Centers for Disease Control and Prevention (CDC). CDC COVID Data Tracker Nationwide Blood Donor Seroprevalence Survey. Atlanta, United States of America: Centers for Disease Control and Prevention (CDC).
USA, Michigan	Centers for Disease Control and Prevention (CDC). CDC COVID Data Tracker Commercial Laboratory Seroprevalence Survey Data. Atlanta, United States of America: Centers for Disease Control and Prevention (CDC).
USA, Minnesota	Centers for Disease Control and Prevention (CDC). CDC COVID Data Tracker Nationwide Blood Donor Seroprevalence Survey. Atlanta, United States of America: Centers for Disease Control and Prevention (CDC).
USA, Minnesota	Centers for Disease Control and Prevention (CDC). CDC COVID Data Tracker Commercial Laboratory Seroprevalence Survey Data. Atlanta, United States of America: Centers for Disease Control and Prevention (CDC).
USA, Minnesota	Anand S, Montez-Rath M, Han J, Bozeman J, Kerschmann R, Beyer P, Parsonnet J, Chertow GM. Prevalence of SARS-CoV-2 antibodies in a large nationwide sample of patients on dialysis in the USA: a cross-sectional study. Lancet. 2020.
USA, Mississippi	Centers for Disease Control and Prevention (CDC). CDC COVID Data Tracker Nationwide Blood Donor Seroprevalence Survey. Atlanta, United States of America: Centers for Disease Control and Prevention (CDC).
USA, Mississippi	Centers for Disease Control and Prevention (CDC). CDC COVID Data Tracker Commercial Laboratory Seroprevalence Survey Data. Atlanta, United States of America: Centers for Disease Control and Prevention (CDC).
USA, Mississippi	Anand S, Montez-Rath M, Han J, Bozeman J, Kerschmann R, Beyer P, Parsonnet J, Chertow GM. Prevalence of SARS-CoV-2 antibodies in a large nationwide sample of patients on dialysis in the USA: a cross-sectional study. Lancet. 2020.
USA, Missouri	Centers for Disease Control and Prevention (CDC). CDC COVID Data Tracker Nationwide Blood Donor Seroprevalence Survey. Atlanta, United States of America: Centers for Disease Control and Prevention (CDC).
USA, Missouri	Havers FP, Reed C, Lim T, Montgomery JM, Klena JD, Hall AJ, Fry AM, Cannon DL, Chiang CF, Gibbons A, Krapiunaya I, Morales-Betoulle M, Roguski K, Rasheed MAU, Freeman B, Lester S, Mills L, Carroll DS, Owen SM, Johnson JA, Semenova V, Blackmore C, Blog D, Chai SJ, Dunn A, Hand J, Jain S, Lindquist S, Lynfield R, Pritchard S, Sokol T, Sosa L, Turabelidze G, Watkins SM, Wiesman J, Williams RW, Yendell S, Schiffer J, Thornburg NJ. Seroprevalence of Antibodies to SARS-CoV-2 in 10 Sites in the United States, March 23-May 12, 2020. JAMA Intern Med. 2020.
USA, Missouri	Centers for Disease Control and Prevention (CDC). CDC COVID Data Tracker Commercial Laboratory Seroprevalence Survey Data. Atlanta, United States of America: Centers for Disease Control and Prevention (CDC).
USA, Missouri	Anand S, Montez-Rath M, Han J, Bozeman J, Kerschmann R, Beyer P, Parsonnet J, Chertow GM. Prevalence of SARS-CoV-2 antibodies in a large nationwide sample of patients on dialysis in the USA: a cross-sectional study. Lancet. 2020.
USA, Montana	Centers for Disease Control and Prevention (CDC). CDC COVID Data Tracker Nationwide Blood Donor Seroprevalence Survey. Atlanta, United States of America: Centers for Disease Control and Prevention (CDC).
USA, Montana	Centers for Disease Control and Prevention (CDC). CDC COVID Data Tracker Commercial Laboratory Seroprevalence Survey Data. Atlanta, United States of America: Centers for Disease Control and Prevention (CDC).
USA, Nebraska	Centers for Disease Control and Prevention (CDC). CDC COVID Data Tracker Nationwide Blood Donor Seroprevalence Survey. Atlanta, United States of America: Centers for Disease Control and Prevention (CDC).
USA, Nebraska	Centers for Disease Control and Prevention (CDC). CDC COVID Data Tracker Commercial Laboratory Seroprevalence Survey Data. Atlanta, United States of America: Centers for Disease Control and Prevention (CDC).
USA, Nebraska	Anand S, Montez-Rath M, Han J, Bozeman J, Kerschmann R, Beyer P, Parsonnet J, Chertow GM. Prevalence of SARS-CoV-2 antibodies in a large nationwide sample of patients on dialysis in the USA: a cross-sectional study. Lancet. 2020.
USA, Nevada	Centers for Disease Control and Prevention (CDC). CDC COVID Data Tracker Nationwide Blood Donor Seroprevalence Survey. Atlanta, United States of America: Centers for Disease Control and Prevention (CDC).
USA, Nevada	Centers for Disease Control and Prevention (CDC). CDC COVID Data Tracker Commercial Laboratory Seroprevalence Survey Data. Atlanta, United States of America: Centers for Disease Control and Prevention (CDC).

USA, New Hampshire	Centers for Disease Control and Prevention (CDC). CDC COVID Data Tracker Nationwide Blood Donor Seroprevalence Survey. Atlanta, United States of America: Centers for Disease Control and Prevention (CDC).
USA, New Hampshire	Centers for Disease Control and Prevention (CDC). CDC COVID Data Tracker Commercial Laboratory Seroprevalence Survey Data. Atlanta, United States of America: Centers for Disease Control and Prevention (CDC).
USA, New Jersey	Centers for Disease Control and Prevention (CDC). CDC COVID Data Tracker Nationwide Blood Donor Seroprevalence Survey. Atlanta, United States of America: Centers for Disease Control and Prevention (CDC).
USA, New Jersey	Centers for Disease Control and Prevention (CDC). CDC COVID Data Tracker Commercial Laboratory Seroprevalence Survey Data. Atlanta, United States of America: Centers for Disease Control and Prevention (CDC).
USA, New Jersey	Anand S, Montez-Rath M, Han J, Bozeman J, Kerschmann R, Beyer P, Parsonnet J, Chertow GM. Prevalence of SARS-CoV-2 antibodies in a large nationwide sample of patients on dialysis in the USA: a cross-sectional study. Lancet. 2020.
USA, New Mexico	Centers for Disease Control and Prevention (CDC). CDC COVID Data Tracker Nationwide Blood Donor Seroprevalence Survey. Atlanta, United States of America: Centers for Disease Control and Prevention (CDC).
USA, New Mexico	Centers for Disease Control and Prevention (CDC). CDC COVID Data Tracker Commercial Laboratory Seroprevalence Survey Data. Atlanta, United States of America: Centers for Disease Control and Prevention (CDC).
USA, New York	Centers for Disease Control and Prevention (CDC). CDC COVID Data Tracker Nationwide Blood Donor Seroprevalence Survey. Atlanta, United States of America: Centers for Disease Control and Prevention (CDC).
USA, New York	Centers for Disease Control and Prevention (CDC). CDC COVID Data Tracker Commercial Laboratory Seroprevalence Survey Data. Atlanta, United States of America: Centers for Disease Control and Prevention (CDC).
USA, New York	Rosenberg ES, Tesoriero JM, Rosenthal EM, Chung R, Barranco MA, Styer LM, Parker MM, John Leung SY, Morne JE, Greene D, Holtgrave DR, Hoefer D, Kumar J, Udo T, Hutton B, Zucker HA. Cumulative incidence and diagnosis of SARS-CoV-2 infection in New York. Ann Epidemiol. 2020; 48: 23-29e4.
USA, North Carolina	Centers for Disease Control and Prevention (CDC). CDC COVID Data Tracker Nationwide Blood Donor Seroprevalence Survey. Atlanta, United States of America: Centers for Disease Control and Prevention (CDC).
USA, North Carolina	Centers for Disease Control and Prevention (CDC). CDC COVID Data Tracker Commercial Laboratory Seroprevalence Survey Data. Atlanta, United States of America: Centers for Disease Control and Prevention (CDC).
USA, North Carolina	Anand S, Montez-Rath M, Han J, Bozeman J, Kerschmann R, Beyer P, Parsonnet J, Chertow GM. Prevalence of SARS-CoV-2 antibodies in a large nationwide sample of patients on dialysis in the USA: a cross-sectional study. Lancet. 2020.
USA, North Dakota	Centers for Disease Control and Prevention (CDC). CDC COVID Data Tracker Nationwide Blood Donor Seroprevalence Survey. Atlanta, United States of America: Centers for Disease Control and Prevention (CDC).
USA, North Dakota	Centers for Disease Control and Prevention (CDC). CDC COVID Data Tracker Commercial Laboratory Seroprevalence Survey Data. Atlanta, United States of America: Centers for Disease Control and Prevention (CDC).
USA, Ohio	Centers for Disease Control and Prevention (CDC). CDC COVID Data Tracker Nationwide Blood Donor Seroprevalence Survey. Atlanta, United States of America: Centers for Disease Control and Prevention (CDC).
USA, Ohio	Centers for Disease Control and Prevention (CDC). CDC COVID Data Tracker Commercial Laboratory Seroprevalence Survey Data. Atlanta, United States of America: Centers for Disease Control and Prevention (CDC).
USA, Ohio	Ohio Department of Health, Ohio State University. United States - Ohio Prevalence of Current and Past COVID-19 in Adults July 2020.
USA, Ohio	Anand S, Montez-Rath M, Han J, Bozeman J, Kerschmann R, Beyer P, Parsonnet J, Chertow GM. Prevalence of SARS-CoV-2 antibodies in a large nationwide sample of patients on dialysis in the USA: a cross-sectional study. Lancet. 2020.
USA, Oklahoma	Centers for Disease Control and Prevention (CDC). CDC COVID Data Tracker Nationwide Blood Donor Seroprevalence Survey. Atlanta, United States of America: Centers for Disease Control and Prevention (CDC).
USA, Oklahoma	Centers for Disease Control and Prevention (CDC). CDC COVID Data Tracker Commercial Laboratory Seroprevalence Survey Data. Atlanta, United States of America: Centers for Disease Control and Prevention (CDC).
USA, Oregon	Centers for Disease Control and Prevention (CDC). CDC COVID Data Tracker Nationwide Blood Donor Seroprevalence Survey. Atlanta, United States of America: Centers for Disease Control and Prevention (CDC).
USA, Oregon	Centers for Disease Control and Prevention (CDC). CDC COVID Data Tracker Commercial Laboratory Seroprevalence Survey Data. Atlanta, United States of America: Centers for Disease Control and Prevention (CDC).
USA, Pennsylvania	Centers for Disease Control and Prevention (CDC). CDC COVID Data Tracker Nationwide Blood Donor Seroprevalence Survey. Atlanta, United States of America: Centers for Disease Control and Prevention (CDC).
USA, Pennsylvania	Centers for Disease Control and Prevention (CDC). CDC COVID Data Tracker Commercial Laboratory Seroprevalence Survey Data. Atlanta, United States of America: Centers for Disease Control and Prevention (CDC).
USA, Pennsylvania	Anand S, Montez-Rath M, Han J, Bozeman J, Kerschmann R, Beyer P, Parsonnet J, Chertow GM. Prevalence of SARS-CoV-2 antibodies in a large nationwide sample of patients on dialysis in the USA: a cross-sectional study. Lancet. 2020.
USA, Rhode Island	Centers for Disease Control and Prevention (CDC). CDC COVID Data Tracker Nationwide Blood Donor Seroprevalence Survey. Atlanta, United States of America: Centers for Disease Control and Prevention (CDC).
USA, Rhode Island	Centers for Disease Control and Prevention (CDC). CDC COVID Data Tracker Commercial Laboratory Seroprevalence Survey Data. Atlanta, United States of America: Centers for Disease Control and Prevention (CDC).
USA, South Carolina	Centers for Disease Control and Prevention (CDC). CDC COVID Data Tracker Nationwide Blood Donor Seroprevalence Survey. Atlanta, United States of America: Centers for Disease Control and Prevention (CDC).
USA, South Carolina	Centers for Disease Control and Prevention (CDC). CDC COVID Data Tracker Commercial Laboratory Seroprevalence Survey Data. Atlanta, United States of America: Centers for Disease Control and Prevention (CDC).
USA, South Carolina	Anand S, Montez-Rath M, Han J, Bozeman J, Kerschmann R, Beyer P, Parsonnet J, Chertow GM. Prevalence of SARS-CoV-2 antibodies in a large nationwide sample of patients on dialysis in the USA: a cross-sectional study. Lancet. 2020.
USA, South Dakota	Centers for Disease Control and Prevention (CDC). CDC COVID Data Tracker Nationwide Blood Donor Seroprevalence Survey. Atlanta, United States of America: Centers for Disease Control and Prevention (CDC).
USA, South Dakota	Centers for Disease Control and Prevention (CDC). CDC COVID Data Tracker Commercial Laboratory Seroprevalence Survey Data. Atlanta, United States of America: Centers for Disease Control and Prevention (CDC).

USA, Tennessee	Centers for Disease Control and Prevention (CDC). CDC COVID Data Tracker Nationwide Blood Donor Seroprevalence Survey. Atlanta, United States of America: Centers for Disease Control and Prevention (CDC).
USA, Tennessee	Centers for Disease Control and Prevention (CDC). CDC COVID Data Tracker Commercial Laboratory Seroprevalence Survey Data. Atlanta, United States of America: Centers for Disease Control and Prevention (CDC).
USA, Tennessee	Anand S, Montez-Rath M, Han J, Bozeman J, Kerschmann R, Beyer P, Parsonnet J, Chertow GM. Prevalence of SARS-CoV-2 antibodies in a large nationwide sample of patients on dialysis in the USA: a cross-sectional study. Lancet. 2020.
USA, Texas	Centers for Disease Control and Prevention (CDC). CDC COVID Data Tracker Nationwide Blood Donor Seroprevalence Survey. Atlanta, United States of America: Centers for Disease Control and Prevention (CDC).
USA, Texas	Centers for Disease Control and Prevention (CDC). CDC COVID Data Tracker Commercial Laboratory Seroprevalence Survey Data. Atlanta, United States of America: Centers for Disease Control and Prevention (CDC).
USA, Texas	Anand S, Montez-Rath M, Han J, Bozeman J, Kerschmann R, Beyer P, Parsonnet J, Chertow GM. Prevalence of SARS-CoV-2 antibodies in a large nationwide sample of patients on dialysis in the USA: a cross-sectional study. Lancet. 2020.
USA, Utah	Centers for Disease Control and Prevention (CDC). CDC COVID Data Tracker Nationwide Blood Donor Seroprevalence Survey. Atlanta, United States of America: Centers for Disease Control and Prevention (CDC).
USA, Utah	Havers FP, Reed C, Lim T, Montgomery JM, Klena JD, Hall AJ, Fry AM, Cannon DL, Chiang CF, Gibbons A, Krapiunaya I, Morales-Betoulle M, Roguski K, Rasheed MAU, Freeman B, Lester S, Mills L, Carroll DS, Owen SM, Johnson JA, Semenova V, Blackmore C, Blog D, Chai SJ, Dunn A, Hand J, Jain S, Lindquist S, Lynfield R, Pritchard S, Sokol T, Sosa L, Turabelidze G, Watkins SM, Wiesman J, Williams RW, Yendell S, Schiffer J, Thornburg NJ. Seroprevalence of Antibodies to SARS-CoV-2 in 10 Sites in the United States, March 23-May 12, 2020. JAMA Intern Med. 2020.
USA, Utah	Centers for Disease Control and Prevention (CDC). CDC COVID Data Tracker Commercial Laboratory Seroprevalence Survey Data. Atlanta, United States of America: Centers for Disease Control and Prevention (CDC).
USA, Utah	Anand S, Montez-Rath M, Han J, Bozeman J, Kerschmann R, Beyer P, Parsonnet J, Chertow GM. Prevalence of SARS-CoV-2 antibodies in a large nationwide sample of patients on dialysis in the USA: a cross-sectional study. Lancet. 2020.
USA, Vermont	Centers for Disease Control and Prevention (CDC). CDC COVID Data Tracker Nationwide Blood Donor Seroprevalence Survey. Atlanta, United States of America: Centers for Disease Control and Prevention (CDC).
USA, Vermont	Centers for Disease Control and Prevention (CDC). CDC COVID Data Tracker Commercial Laboratory Seroprevalence Survey Data. Atlanta, United States of America: Centers for Disease Control and Prevention (CDC).
USA, Virginia	Centers for Disease Control and Prevention (CDC). CDC COVID Data Tracker Nationwide Blood Donor Seroprevalence Survey. Atlanta, United States of America: Centers for Disease Control and Prevention (CDC).
USA, Virginia	Centers for Disease Control and Prevention (CDC). CDC COVID Data Tracker Commercial Laboratory Seroprevalence Survey Data. Atlanta, United States of America: Centers for Disease Control and Prevention (CDC).
USA, Washington	Centers for Disease Control and Prevention (CDC). CDC COVID Data Tracker Nationwide Blood Donor Seroprevalence Survey. Atlanta, United States of America: Centers for Disease Control and Prevention (CDC).
USA, Washington	Centers for Disease Control and Prevention (CDC). CDC COVID Data Tracker Commercial Laboratory Seroprevalence Survey Data. Atlanta, United States of America: Centers for Disease Control and Prevention (CDC).
USA, Washington, DC	Centers for Disease Control and Prevention (CDC). CDC COVID Data Tracker Nationwide Blood Donor Seroprevalence Survey. Atlanta, United States of America: Centers for Disease Control and Prevention (CDC).
USA, Washington, DC	Centers for Disease Control and Prevention (CDC). CDC COVID Data Tracker Commercial Laboratory Seroprevalence Survey Data. Atlanta, United States of America: Centers for Disease Control and Prevention (CDC).
USA, West Virginia	Centers for Disease Control and Prevention (CDC). CDC COVID Data Tracker Nationwide Blood Donor Seroprevalence Survey. Atlanta, United States of America: Centers for Disease Control and Prevention (CDC).
USA, West Virginia	Centers for Disease Control and Prevention (CDC). CDC COVID Data Tracker Commercial Laboratory Seroprevalence Survey Data. Atlanta, United States of America: Centers for Disease Control and Prevention (CDC).
USA, Wisconsin	Centers for Disease Control and Prevention (CDC). CDC COVID Data Tracker Nationwide Blood Donor Seroprevalence Survey. Atlanta, United States of America: Centers for Disease Control and Prevention (CDC).
USA, Wisconsin	Centers for Disease Control and Prevention (CDC). CDC COVID Data Tracker Commercial Laboratory Seroprevalence Survey Data. Atlanta, United States of America: Centers for Disease Control and Prevention (CDC).
USA, Wyoming	Centers for Disease Control and Prevention (CDC). CDC COVID Data Tracker Nationwide Blood Donor Seroprevalence Survey. Atlanta, United States of America: Centers for Disease Control and Prevention (CDC).
USA, Wyoming	Centers for Disease Control and Prevention (CDC). CDC COVID Data Tracker Commercial Laboratory Seroprevalence Survey Data. Atlanta, United States of America: Centers for Disease Control and Prevention (CDC).
Uzbekistan	Rakhimov RA, Ibadullaeva NS, Khikmatullaeva AS, Abdukadirova MA, Sadirova SS, Lokteva LM, Rakhimov RR, Bayjanov AK, Samatova IR. Formation of Herd Immunity to SARS-CoV-2 in the Regions of Uzbekistan. Eur J Mol Clinl Med. 2021; 8(3): 574-81.
Yemen	Bin Ghouth aS, Al-Shoteri S, Mahmoud N, Musani A, Baoom N, Al-Waleedi AA, Buliva E, Kareem EA, Naiene JD, Crestani R, Mikiko S, Barakat A, Al-Ariqi L, Al-sakkaf KAZ, Shaief A, Thabet N, Murshed A, Samouel O. SARS-CoV-2 Seroprevalence in Aden, Yemen a Population-Based Study. Preprint. Prepr Lancet. 2021.
Zimbabwe	Fryatt A, Simms V, Bandason T, Redzo N, Olaru ID, Ndhlovu CE, Mujuru H, Rusakaniko S, Hoelscher M, Rubio-Acero R, Paunovic I, Wieser A, Chonzi P, Masunda K, Ferrand RA, Kranzer K. Community SARS-CoV-2 seroprevalence before and after the second wave of SARS-CoV-2 infection in Harare, Zimbabwe. EClinicalMedicine. 2021; 41: 101172.

Section 4: Cases sources

Figure S4. Case data coverage by location

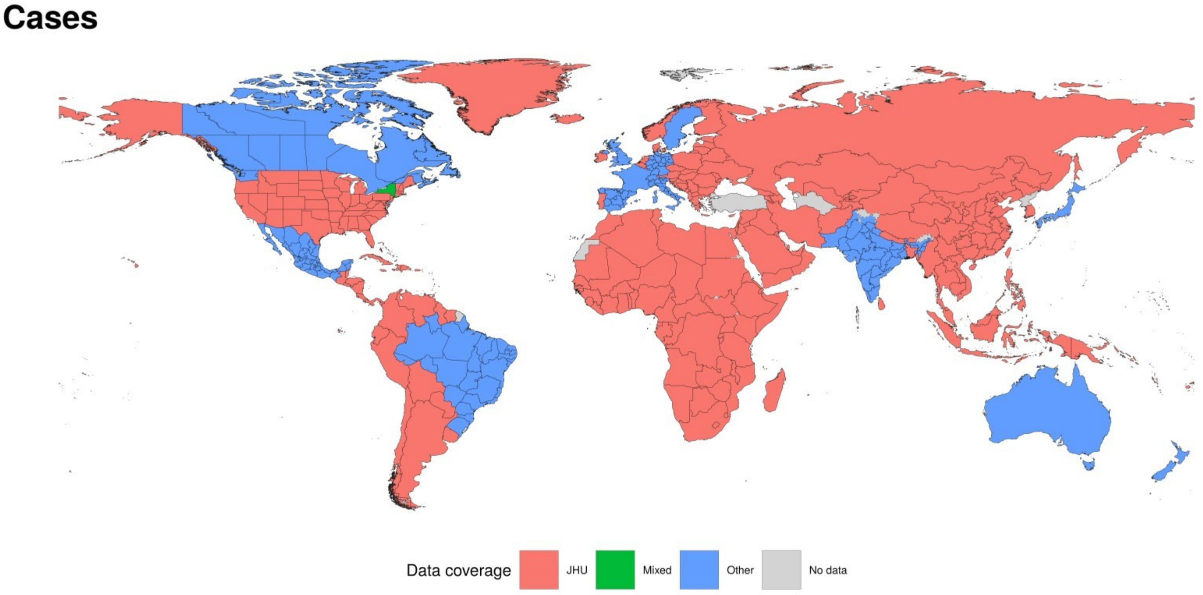

Table S4. Case data sources by location

Location	Source
Afghanistan	Johns Hopkins University. 2019 Novel Coronavirus COVID-19 (2019-nCoV) Data Repository by Johns Hopkins CSSE. Baltimore, Maryland: Johns Hopkins University.
Albania	Johns Hopkins University. 2019 Novel Coronavirus COVID-19 (2019-nCoV) Data Repository by Johns Hopkins CSSE. Baltimore, Maryland: Johns Hopkins University.
Algeria	Johns Hopkins University. 2019 Novel Coronavirus COVID-19 (2019-nCoV) Data Repository by Johns Hopkins CSSE. Baltimore, Maryland: Johns Hopkins University.
American Samoa	Johns Hopkins University. 2019 Novel Coronavirus COVID-19 (2019-nCoV) Data Repository by Johns Hopkins CSSE. Baltimore, Maryland: Johns Hopkins University.
Andorra	Johns Hopkins University. 2019 Novel Coronavirus COVID-19 (2019-nCoV) Data Repository by Johns Hopkins CSSE. Baltimore, Maryland: Johns Hopkins University.
Angola	Johns Hopkins University. 2019 Novel Coronavirus COVID-19 (2019-nCoV) Data Repository by Johns Hopkins CSSE. Baltimore, Maryland: Johns Hopkins University.
Antigua and Barbuda	Johns Hopkins University. 2019 Novel Coronavirus COVID-19 (2019-nCoV) Data Repository by Johns Hopkins CSSE. Baltimore, Maryland: Johns Hopkins University.
Argentina	Johns Hopkins University. 2019 Novel Coronavirus COVID-19 (2019-nCoV) Data Repository by Johns Hopkins CSSE. Baltimore, Maryland: Johns Hopkins University.
Armenia	Johns Hopkins University. 2019 Novel Coronavirus COVID-19 (2019-nCoV) Data Repository by Johns Hopkins CSSE. Baltimore, Maryland: Johns Hopkins University.
Australia	Australian Capital Territory Government, Centre for Disease Control, Department of Health (Northern Territory, Australia), Covid Live, Department of Health (Australia), Department of Health and Human Services (Tasmania), Department of Human Services (Victoria, Australia), Government of Western Australia, New South Wales Department of Health, New South Wales Ministry of Health, Queensland Department of Health, South Australian Department of Health. Australia COVID-19 Data Dashboard - COVID Live. Australia: Covid Live.
Austria	Johns Hopkins University. 2019 Novel Coronavirus COVID-19 (2019-nCoV) Data Repository by Johns Hopkins CSSE. Baltimore, Maryland: Johns Hopkins University.
Azerbaijan	Johns Hopkins University. 2019 Novel Coronavirus COVID-19 (2019-nCoV) Data Repository by Johns Hopkins CSSE. Baltimore, Maryland: Johns Hopkins University.
Bahrain	Johns Hopkins University. 2019 Novel Coronavirus COVID-19 (2019-nCoV) Data Repository by Johns Hopkins CSSE. Baltimore, Maryland: Johns Hopkins University.
Bangladesh	Johns Hopkins University. 2019 Novel Coronavirus COVID-19 (2019-nCoV) Data Repository by Johns Hopkins CSSE. Baltimore, Maryland: Johns Hopkins University.
Barbados	Johns Hopkins University. 2019 Novel Coronavirus COVID-19 (2019-nCoV) Data Repository by Johns Hopkins CSSE. Baltimore, Maryland: Johns Hopkins University.

Belarus	Johns Hopkins University. 2019 Novel Coronavirus COVID-19 (2019-nCoV) Data Repository by Johns Hopkins CSSE. Baltimore, Maryland: Johns Hopkins University.
Belgium	Johns Hopkins University. 2019 Novel Coronavirus COVID-19 (2019-nCoV) Data Repository by Johns Hopkins CSSE. Baltimore, Maryland: Johns Hopkins University.
Belize	Johns Hopkins University. 2019 Novel Coronavirus COVID-19 (2019-nCoV) Data Repository by Johns Hopkins CSSE. Baltimore, Maryland: Johns Hopkins University.
Benin	Johns Hopkins University. 2019 Novel Coronavirus COVID-19 (2019-nCoV) Data Repository by Johns Hopkins CSSE. Baltimore, Maryland: Johns Hopkins University.
Bermuda	Johns Hopkins University. 2019 Novel Coronavirus COVID-19 (2019-nCoV) Data Repository by Johns Hopkins CSSE. Baltimore, Maryland: Johns Hopkins University.
Bhutan	Johns Hopkins University. 2019 Novel Coronavirus COVID-19 (2019-nCoV) Data Repository by Johns Hopkins CSSE. Baltimore, Maryland: Johns Hopkins University.
Bolivia	Johns Hopkins University. 2019 Novel Coronavirus COVID-19 (2019-nCoV) Data Repository by Johns Hopkins CSSE. Baltimore, Maryland: Johns Hopkins University.
Bosnia and Herzegovina	Johns Hopkins University. 2019 Novel Coronavirus COVID-19 (2019-nCoV) Data Repository by Johns Hopkins CSSE. Baltimore, Maryland: Johns Hopkins University.
Botswana	Johns Hopkins University. 2019 Novel Coronavirus COVID-19 (2019-nCoV) Data Repository by Johns Hopkins CSSE. Baltimore, Maryland: Johns Hopkins University.
Brazil, Acre	Ministry of Health (Brazil). Brazil Ministry of Health COVID-19 Coronavirus Panel. Rio de Janeiro, Brazil: Ministry of Health (Brazil).
Brazil, Alagoas	Ministry of Health (Brazil). Brazil Ministry of Health COVID-19 Coronavirus Panel. Rio de Janeiro, Brazil: Ministry of Health (Brazil).
Brazil, Amapá	Ministry of Health (Brazil). Brazil Ministry of Health COVID-19 Coronavirus Panel. Rio de Janeiro, Brazil: Ministry of Health (Brazil).
Brazil, Amazonas	Ministry of Health (Brazil). Brazil Ministry of Health COVID-19 Coronavirus Panel. Rio de Janeiro, Brazil: Ministry of Health (Brazil).
Brazil, Bahia	Ministry of Health (Brazil). Brazil Ministry of Health COVID-19 Coronavirus Panel. Rio de Janeiro, Brazil: Ministry of Health (Brazil).
Brazil, Ceará	Ministry of Health (Brazil). Brazil Ministry of Health COVID-19 Coronavirus Panel. Rio de Janeiro, Brazil: Ministry of Health (Brazil).
Brazil, Distrito Federal	Ministry of Health (Brazil). Brazil Ministry of Health COVID-19 Coronavirus Panel. Rio de Janeiro, Brazil: Ministry of Health (Brazil).
Brazil, Espírito Santo	Ministry of Health (Brazil). Brazil Ministry of Health COVID-19 Coronavirus Panel. Rio de Janeiro, Brazil: Ministry of Health (Brazil).
Brazil, Goiás	Ministry of Health (Brazil). Brazil Ministry of Health COVID-19 Coronavirus Panel. Rio de Janeiro, Brazil: Ministry of Health (Brazil).
Brazil, Maranhão	Ministry of Health (Brazil). Brazil Ministry of Health COVID-19 Coronavirus Panel. Rio de Janeiro, Brazil: Ministry of Health (Brazil).
Brazil, Mato Grosso	Ministry of Health (Brazil). Brazil Ministry of Health COVID-19 Coronavirus Panel. Rio de Janeiro, Brazil: Ministry of Health (Brazil).
Brazil, Mato Grosso do Sul	Ministry of Health (Brazil). Brazil Ministry of Health COVID-19 Coronavirus Panel. Rio de Janeiro, Brazil: Ministry of Health (Brazil).
Brazil, Minas Gerais	Ministry of Health (Brazil). Brazil Ministry of Health COVID-19 Coronavirus Panel. Rio de Janeiro, Brazil: Ministry of Health (Brazil).
Brazil, Pará	Ministry of Health (Brazil). Brazil Ministry of Health COVID-19 Coronavirus Panel. Rio de Janeiro, Brazil: Ministry of Health (Brazil).
Brazil, Paraíba	Ministry of Health (Brazil). Brazil Ministry of Health COVID-19 Coronavirus Panel. Rio de Janeiro, Brazil: Ministry of Health (Brazil).
Brazil, Paraná	Ministry of Health (Brazil). Brazil Ministry of Health COVID-19 Coronavirus Panel. Rio de Janeiro, Brazil: Ministry of Health (Brazil).
Brazil, Pernambuco	Ministry of Health (Brazil). Brazil Ministry of Health COVID-19 Coronavirus Panel. Rio de Janeiro, Brazil: Ministry of Health (Brazil).
Brazil, Piauí	Ministry of Health (Brazil). Brazil Ministry of Health COVID-19 Coronavirus Panel. Rio de Janeiro, Brazil: Ministry of Health (Brazil).
Brazil, Rio de Janeiro	Ministry of Health (Brazil). Brazil Ministry of Health COVID-19 Coronavirus Panel. Rio de Janeiro, Brazil: Ministry of Health (Brazil).
Brazil, Rio Grande do Norte	Ministry of Health (Brazil). Brazil Ministry of Health COVID-19 Coronavirus Panel. Rio de Janeiro, Brazil: Ministry of Health (Brazil).
Brazil, Rio Grande do Sul	Ministry of Health (Brazil). Brazil Ministry of Health COVID-19 Coronavirus Panel. Rio de Janeiro, Brazil: Ministry of Health (Brazil).
Brazil, Rondônia	Ministry of Health (Brazil). Brazil Ministry of Health COVID-19 Coronavirus Panel. Rio de Janeiro, Brazil: Ministry of Health (Brazil).
Brazil, Roraima	Ministry of Health (Brazil). Brazil Ministry of Health COVID-19 Coronavirus Panel. Rio de Janeiro, Brazil: Ministry of Health (Brazil).
Brazil, Santa Catarina	Ministry of Health (Brazil). Brazil Ministry of Health COVID-19 Coronavirus Panel. Rio de Janeiro, Brazil: Ministry of Health (Brazil).

Brazil, São Paulo	Ministry of Health (Brazil). Brazil Ministry of Health COVID-19 Coronavirus Panel. Rio de Janeiro, Brazil: Ministry of Health (Brazil).
Brazil, Sergipe	Ministry of Health (Brazil). Brazil Ministry of Health COVID-19 Coronavirus Panel. Rio de Janeiro, Brazil: Ministry of Health (Brazil).
Brazil, Tocantins	Ministry of Health (Brazil). Brazil Ministry of Health COVID-19 Coronavirus Panel. Rio de Janeiro, Brazil: Ministry of Health (Brazil).
Brunei	Johns Hopkins University. 2019 Novel Coronavirus COVID-19 (2019-nCoV) Data Repository by Johns Hopkins CSSE. Baltimore, Maryland: Johns Hopkins University.
Bulgaria	Johns Hopkins University. 2019 Novel Coronavirus COVID-19 (2019-nCoV) Data Repository by Johns Hopkins CSSE. Baltimore, Maryland: Johns Hopkins University.
Burkina Faso	Johns Hopkins University. 2019 Novel Coronavirus COVID-19 (2019-nCoV) Data Repository by Johns Hopkins CSSE. Baltimore, Maryland: Johns Hopkins University.
Burundi	Johns Hopkins University. 2019 Novel Coronavirus COVID-19 (2019-nCoV) Data Repository by Johns Hopkins CSSE. Baltimore, Maryland: Johns Hopkins University.
Cambodia	Johns Hopkins University. 2019 Novel Coronavirus COVID-19 (2019-nCoV) Data Repository by Johns Hopkins CSSE. Baltimore, Maryland: Johns Hopkins University.
Cameroon	Johns Hopkins University. 2019 Novel Coronavirus COVID-19 (2019-nCoV) Data Repository by Johns Hopkins CSSE. Baltimore, Maryland: Johns Hopkins University.
Canada, Alberta	Government of Canada. Canada Public Health Infobase Number of Total Cases of COVID-19. Ottawa, Canada: Government of Canada.
Canada, Alberta	Government of Canada. Canada Coronavirus Disease 2019 (COVID-19) Daily Epidemiology Update. Ottawa, Canada: Government of Canada.
Canada, British Columbia	Government of Canada. Canada Public Health Infobase Number of Total Cases of COVID-19. Ottawa, Canada: Government of Canada.
Canada, British Columbia	Government of Canada. Canada Coronavirus Disease 2019 (COVID-19) Daily Epidemiology Update. Ottawa, Canada: Government of Canada.
Canada, Manitoba	Government of Canada. Canada Public Health Infobase Number of Total Cases of COVID-19. Ottawa, Canada: Government of Canada.
Canada, Manitoba	Government of Canada. Canada Coronavirus Disease 2019 (COVID-19) Daily Epidemiology Update. Ottawa, Canada: Government of Canada.
Canada, New Brunswick	Government of Canada. Canada Public Health Infobase Number of Total Cases of COVID-19. Ottawa, Canada: Government of Canada.
Canada, New Brunswick	Government of Canada. Canada Coronavirus Disease 2019 (COVID-19) Daily Epidemiology Update. Ottawa, Canada: Government of Canada.
Canada, Newfoundland and Labrador	Government of Canada. Canada Public Health Infobase Number of Total Cases of COVID-19. Ottawa, Canada: Government of Canada.
Canada, Newfoundland and Labrador	Government of Canada. Canada Coronavirus Disease 2019 (COVID-19) Daily Epidemiology Update. Ottawa, Canada: Government of Canada.
Canada, Northwest Territories	Government of Canada. Canada Public Health Infobase Number of Total Cases of COVID-19. Ottawa, Canada: Government of Canada.
Canada, Northwest Territories	Government of Canada. Canada Coronavirus Disease 2019 (COVID-19) Daily Epidemiology Update. Ottawa, Canada: Government of Canada.
Canada, Nova Scotia	Government of Canada. Canada Public Health Infobase Number of Total Cases of COVID-19. Ottawa, Canada: Government of Canada.
Canada, Nova Scotia	Government of Canada. Canada Coronavirus Disease 2019 (COVID-19) Daily Epidemiology Update. Ottawa, Canada: Government of Canada.
Canada, Nunavut	Government of Canada. Canada Public Health Infobase Number of Total Cases of COVID-19. Ottawa, Canada: Government of Canada.
Canada, Nunavut	Government of Canada. Canada Coronavirus Disease 2019 (COVID-19) Daily Epidemiology Update. Ottawa, Canada: Government of Canada.
Canada, Ontario	Government of Canada. Canada Public Health Infobase Number of Total Cases of COVID-19. Ottawa, Canada: Government of Canada.
Canada, Ontario	Government of Canada. Canada Coronavirus Disease 2019 (COVID-19) Daily Epidemiology Update. Ottawa, Canada: Government of Canada.
Canada, Prince Edward Island	Government of Canada. Canada Public Health Infobase Number of Total Cases of COVID-19. Ottawa, Canada: Government of Canada.
Canada, Prince Edward Island	Government of Canada. Canada Coronavirus Disease 2019 (COVID-19) Daily Epidemiology Update. Ottawa, Canada: Government of Canada.
Canada, Quebec	Government of Canada. Canada Public Health Infobase Number of Total Cases of COVID-19. Ottawa, Canada: Government of Canada.
Canada, Quebec	Government of Canada. Canada Coronavirus Disease 2019 (COVID-19) Daily Epidemiology Update. Ottawa, Canada: Government of Canada.
Canada, Saskatchewan	Government of Canada. Canada Public Health Infobase Number of Total Cases of COVID-19. Ottawa, Canada: Government of Canada.

Canada, Saskatchewan	Government of Canada. Canada Coronavirus Disease 2019 (COVID-19) Daily Epidemiology Update. Ottawa, Canada: Government of Canada.
Canada, Yukon	Government of Canada. Canada Public Health Infobase Number of Total Cases of COVID-19. Ottawa, Canada: Government of Canada.
Canada, Yukon	Government of Canada. Canada Coronavirus Disease 2019 (COVID-19) Daily Epidemiology Update. Ottawa, Canada: Government of Canada.
Cape Verde	Johns Hopkins University. 2019 Novel Coronavirus COVID-19 (2019-nCoV) Data Repository by Johns Hopkins CSSE. Baltimore, Maryland: Johns Hopkins University.
Central African Republic	Johns Hopkins University. 2019 Novel Coronavirus COVID-19 (2019-nCoV) Data Repository by Johns Hopkins CSSE. Baltimore, Maryland: Johns Hopkins University.
Chad	Johns Hopkins University. 2019 Novel Coronavirus COVID-19 (2019-nCoV) Data Repository by Johns Hopkins CSSE. Baltimore, Maryland: Johns Hopkins University.
Chile	Johns Hopkins University. 2019 Novel Coronavirus COVID-19 (2019-nCoV) Data Repository by Johns Hopkins CSSE. Baltimore, Maryland: Johns Hopkins University.
China	Johns Hopkins University. 2019 Novel Coronavirus COVID-19 (2019-nCoV) Data Repository by Johns Hopkins CSSE. Baltimore, Maryland: Johns Hopkins University.
Colombia	Johns Hopkins University. 2019 Novel Coronavirus COVID-19 (2019-nCoV) Data Repository by Johns Hopkins CSSE. Baltimore, Maryland: Johns Hopkins University.
Comoros	Johns Hopkins University. 2019 Novel Coronavirus COVID-19 (2019-nCoV) Data Repository by Johns Hopkins CSSE. Baltimore, Maryland: Johns Hopkins University.
Congo (Brazzaville)	Johns Hopkins University. 2019 Novel Coronavirus COVID-19 (2019-nCoV) Data Repository by Johns Hopkins CSSE. Baltimore, Maryland: Johns Hopkins University.
Costa Rica	Johns Hopkins University. 2019 Novel Coronavirus COVID-19 (2019-nCoV) Data Repository by Johns Hopkins CSSE. Baltimore, Maryland: Johns Hopkins University.
Côte d'Ivoire	Johns Hopkins University. 2019 Novel Coronavirus COVID-19 (2019-nCoV) Data Repository by Johns Hopkins CSSE. Baltimore, Maryland: Johns Hopkins University.
Croatia	Johns Hopkins University. 2019 Novel Coronavirus COVID-19 (2019-nCoV) Data Repository by Johns Hopkins CSSE. Baltimore, Maryland: Johns Hopkins University.
Cuba	Johns Hopkins University. 2019 Novel Coronavirus COVID-19 (2019-nCoV) Data Repository by Johns Hopkins CSSE. Baltimore, Maryland: Johns Hopkins University.
Cyprus	Johns Hopkins University. 2019 Novel Coronavirus COVID-19 (2019-nCoV) Data Repository by Johns Hopkins CSSE. Baltimore, Maryland: Johns Hopkins University.
Czechia	Johns Hopkins University. 2019 Novel Coronavirus COVID-19 (2019-nCoV) Data Repository by Johns Hopkins CSSE. Baltimore, Maryland: Johns Hopkins University.
Denmark	Johns Hopkins University. 2019 Novel Coronavirus COVID-19 (2019-nCoV) Data Repository by Johns Hopkins CSSE. Baltimore, Maryland: Johns Hopkins University.
Djibouti	Johns Hopkins University. 2019 Novel Coronavirus COVID-19 (2019-nCoV) Data Repository by Johns Hopkins CSSE. Baltimore, Maryland: Johns Hopkins University.
Dominica	Johns Hopkins University. 2019 Novel Coronavirus COVID-19 (2019-nCoV) Data Repository by Johns Hopkins CSSE. Baltimore, Maryland: Johns Hopkins University.
Dominican Republic	Johns Hopkins University. 2019 Novel Coronavirus COVID-19 (2019-nCoV) Data Repository by Johns Hopkins CSSE. Baltimore, Maryland: Johns Hopkins University.
DR Congo	Johns Hopkins University. 2019 Novel Coronavirus COVID-19 (2019-nCoV) Data Repository by Johns Hopkins CSSE. Baltimore, Maryland: Johns Hopkins University.
Ecuador	Johns Hopkins University. 2019 Novel Coronavirus COVID-19 (2019-nCoV) Data Repository by Johns Hopkins CSSE. Baltimore, Maryland: Johns Hopkins University.
Egypt	Johns Hopkins University. 2019 Novel Coronavirus COVID-19 (2019-nCoV) Data Repository by Johns Hopkins CSSE. Baltimore, Maryland: Johns Hopkins University.
El Salvador	Johns Hopkins University. 2019 Novel Coronavirus COVID-19 (2019-nCoV) Data Repository by Johns Hopkins CSSE. Baltimore, Maryland: Johns Hopkins University.
Equatorial Guinea	Johns Hopkins University. 2019 Novel Coronavirus COVID-19 (2019-nCoV) Data Repository by Johns Hopkins CSSE. Baltimore, Maryland: Johns Hopkins University.
Eritrea	Johns Hopkins University. 2019 Novel Coronavirus COVID-19 (2019-nCoV) Data Repository by Johns Hopkins CSSE. Baltimore, Maryland: Johns Hopkins University.
Estonia	Johns Hopkins University. 2019 Novel Coronavirus COVID-19 (2019-nCoV) Data Repository by Johns Hopkins CSSE. Baltimore, Maryland: Johns Hopkins University.
Eswatini	Johns Hopkins University. 2019 Novel Coronavirus COVID-19 (2019-nCoV) Data Repository by Johns Hopkins CSSE. Baltimore, Maryland: Johns Hopkins University.
Ethiopia	Johns Hopkins University. 2019 Novel Coronavirus COVID-19 (2019-nCoV) Data Repository by Johns Hopkins CSSE. Baltimore, Maryland: Johns Hopkins University.
Federated States of Micronesia	Johns Hopkins University. 2019 Novel Coronavirus COVID-19 (2019-nCoV) Data Repository by Johns Hopkins CSSE. Baltimore, Maryland: Johns Hopkins University.
Fiji	Johns Hopkins University. 2019 Novel Coronavirus COVID-19 (2019-nCoV) Data Repository by Johns Hopkins CSSE. Baltimore, Maryland: Johns Hopkins University.
Finland	Johns Hopkins University. 2019 Novel Coronavirus COVID-19 (2019-nCoV) Data Repository by Johns Hopkins CSSE. Baltimore, Maryland: Johns Hopkins University.

France	Etalab (France), Ministry of Health (France), Ministry of Health and Solidarity (DRESS) (France). France COVID-19 Epidemic Monitoring Dashboard.
Gabon	Johns Hopkins University. 2019 Novel Coronavirus COVID-19 (2019-nCoV) Data Repository by Johns Hopkins CSSE. Baltimore, Maryland: Johns Hopkins University.
Georgia	Johns Hopkins University. 2019 Novel Coronavirus COVID-19 (2019-nCoV) Data Repository by Johns Hopkins CSSE. Baltimore, Maryland: Johns Hopkins University.
Germany, Baden-Württemberg	Robert Koch Institute. Coronavirus Disease 2019 (COVID-19) Daily Situation Report - Robert Koch Institute. Berlin, Germany: Robert Koch Institute.
Germany, Baden-Württemberg	Wikipedia. COVID-19 pandemic in Germany. San Francisco, United States of America: Wikipedia.
Germany, Bavaria	Robert Koch Institute. Coronavirus Disease 2019 (COVID-19) Daily Situation Report - Robert Koch Institute. Berlin, Germany: Robert Koch Institute.
Germany, Bavaria	Wikipedia. COVID-19 pandemic in Germany. San Francisco, United States of America: Wikipedia.
Germany, Berlin	Robert Koch Institute. Coronavirus Disease 2019 (COVID-19) Daily Situation Report - Robert Koch Institute. Berlin, Germany: Robert Koch Institute.
Germany, Berlin	Wikipedia. COVID-19 pandemic in Germany. San Francisco, United States of America: Wikipedia.
Germany, Brandenburg	Robert Koch Institute. Coronavirus Disease 2019 (COVID-19) Daily Situation Report - Robert Koch Institute. Berlin, Germany: Robert Koch Institute.
Germany, Brandenburg	Wikipedia. COVID-19 pandemic in Germany. San Francisco, United States of America: Wikipedia.
Germany, Bremen	Robert Koch Institute. Coronavirus Disease 2019 (COVID-19) Daily Situation Report - Robert Koch Institute. Berlin, Germany: Robert Koch Institute.
Germany, Bremen	Wikipedia. COVID-19 pandemic in Germany. San Francisco, United States of America: Wikipedia.
Germany, Hamburg	Robert Koch Institute. Coronavirus Disease 2019 (COVID-19) Daily Situation Report - Robert Koch Institute. Berlin, Germany: Robert Koch Institute.
Germany, Hamburg	Wikipedia. COVID-19 pandemic in Germany. San Francisco, United States of America: Wikipedia.
Germany, Hesse	Robert Koch Institute. Coronavirus Disease 2019 (COVID-19) Daily Situation Report - Robert Koch Institute. Berlin, Germany: Robert Koch Institute.
Germany, Hesse	Wikipedia. COVID-19 pandemic in Germany. San Francisco, United States of America: Wikipedia.
Germany, Lower Saxony	Robert Koch Institute. Coronavirus Disease 2019 (COVID-19) Daily Situation Report - Robert Koch Institute. Berlin, Germany: Robert Koch Institute.
Germany, Lower Saxony	Wikipedia. COVID-19 pandemic in Germany. San Francisco, United States of America: Wikipedia.
Germany, Mecklenburg-Vorpommern	Robert Koch Institute. Coronavirus Disease 2019 (COVID-19) Daily Situation Report - Robert Koch Institute. Berlin, Germany: Robert Koch Institute.
Germany, Mecklenburg-Vorpommern	Wikipedia. COVID-19 pandemic in Germany. San Francisco, United States of America: Wikipedia.
Germany, North Rhine-Westphalia	Robert Koch Institute. Coronavirus Disease 2019 (COVID-19) Daily Situation Report - Robert Koch Institute. Berlin, Germany: Robert Koch Institute.
Germany, North Rhine-Westphalia	Wikipedia. COVID-19 pandemic in Germany. San Francisco, United States of America: Wikipedia.
Germany, Rhineland-Palatinate	Robert Koch Institute. Coronavirus Disease 2019 (COVID-19) Daily Situation Report - Robert Koch Institute. Berlin, Germany: Robert Koch Institute.
Germany, Rhineland-Palatinate	Wikipedia. COVID-19 pandemic in Germany. San Francisco, United States of America: Wikipedia.
Germany, Saarland	Robert Koch Institute. Coronavirus Disease 2019 (COVID-19) Daily Situation Report - Robert Koch Institute. Berlin, Germany: Robert Koch Institute.
Germany, Saarland	Wikipedia. COVID-19 pandemic in Germany. San Francisco, United States of America: Wikipedia.
Germany, Saxony	Robert Koch Institute. Coronavirus Disease 2019 (COVID-19) Daily Situation Report - Robert Koch Institute. Berlin, Germany: Robert Koch Institute.
Germany, Saxony	Wikipedia. COVID-19 pandemic in Germany. San Francisco, United States of America: Wikipedia.
Germany, Saxony-Anhalt	Robert Koch Institute. Coronavirus Disease 2019 (COVID-19) Daily Situation Report - Robert Koch Institute. Berlin, Germany: Robert Koch Institute.
Germany, Saxony-Anhalt	Wikipedia. COVID-19 pandemic in Germany. San Francisco, United States of America: Wikipedia.
Germany, Schleswig-Holstein	Robert Koch Institute. Coronavirus Disease 2019 (COVID-19) Daily Situation Report - Robert Koch Institute. Berlin, Germany: Robert Koch Institute.
Germany, Schleswig-Holstein	Wikipedia. COVID-19 pandemic in Germany. San Francisco, United States of America: Wikipedia.
Germany, Thuringia	Robert Koch Institute. Coronavirus Disease 2019 (COVID-19) Daily Situation Report - Robert Koch Institute. Berlin, Germany: Robert Koch Institute.

Germany, Thuringia	Wikipedia. COVID-19 pandemic in Germany. San Francisco, United States of America: Wikipedia.
Ghana	Johns Hopkins University. 2019 Novel Coronavirus COVID-19 (2019-nCoV) Data Repository by Johns Hopkins CSSE. Baltimore, Maryland: Johns Hopkins University.
Greece	Johns Hopkins University. 2019 Novel Coronavirus COVID-19 (2019-nCoV) Data Repository by Johns Hopkins CSSE. Baltimore, Maryland: Johns Hopkins University.
Greenland	Johns Hopkins University. 2019 Novel Coronavirus COVID-19 (2019-nCoV) Data Repository by Johns Hopkins CSSE. Baltimore, Maryland: Johns Hopkins University.
Grenada	Johns Hopkins University. 2019 Novel Coronavirus COVID-19 (2019-nCoV) Data Repository by Johns Hopkins CSSE. Baltimore, Maryland: Johns Hopkins University.
Guam	Johns Hopkins University. 2019 Novel Coronavirus COVID-19 (2019-nCoV) Data Repository by Johns Hopkins CSSE. Baltimore, Maryland: Johns Hopkins University.
Guatemala	Johns Hopkins University. 2019 Novel Coronavirus COVID-19 (2019-nCoV) Data Repository by Johns Hopkins CSSE. Baltimore, Maryland: Johns Hopkins University.
Guinea	Johns Hopkins University. 2019 Novel Coronavirus COVID-19 (2019-nCoV) Data Repository by Johns Hopkins CSSE. Baltimore, Maryland: Johns Hopkins University.
Guinea-Bissau	Johns Hopkins University. 2019 Novel Coronavirus COVID-19 (2019-nCoV) Data Repository by Johns Hopkins CSSE. Baltimore, Maryland: Johns Hopkins University.
Guyana	Johns Hopkins University. 2019 Novel Coronavirus COVID-19 (2019-nCoV) Data Repository by Johns Hopkins CSSE. Baltimore, Maryland: Johns Hopkins University.
Haiti	Johns Hopkins University. 2019 Novel Coronavirus COVID-19 (2019-nCoV) Data Repository by Johns Hopkins CSSE. Baltimore, Maryland: Johns Hopkins University.
Honduras	Johns Hopkins University. 2019 Novel Coronavirus COVID-19 (2019-nCoV) Data Repository by Johns Hopkins CSSE. Baltimore, Maryland: Johns Hopkins University.
Hungary	Johns Hopkins University. 2019 Novel Coronavirus COVID-19 (2019-nCoV) Data Repository by Johns Hopkins CSSE. Baltimore, Maryland: Johns Hopkins University.
Iceland	Johns Hopkins University. 2019 Novel Coronavirus COVID-19 (2019-nCoV) Data Repository by Johns Hopkins CSSE. Baltimore, Maryland: Johns Hopkins University.
India, Andhra Pradesh	COVID-19 India. India COVID-19 Crowdsourced Patient Database: National Level Time Series, State-Wise Stats and Test Counts. India: COVID-19 India.
India, Andhra Pradesh	COVID-19 India. India COVID-19 Crowdsourced Patient Database: State Level Daily Changes. India: COVID-19 India.
India, Arunachal Pradesh	COVID-19 India. India COVID-19 Crowdsourced Patient Database: National Level Time Series, State-Wise Stats and Test Counts. India: COVID-19 India.
India, Arunachal Pradesh	COVID-19 India. India COVID-19 Crowdsourced Patient Database: State Level Daily Changes. India: COVID-19 India.
India, Assam	COVID-19 India. India COVID-19 Crowdsourced Patient Database: National Level Time Series, State-Wise Stats and Test Counts. India: COVID-19 India.
India, Assam	COVID-19 India. India COVID-19 Crowdsourced Patient Database: State Level Daily Changes. India: COVID-19 India.
India, Bihar	COVID-19 India. India COVID-19 Crowdsourced Patient Database: National Level Time Series, State-Wise Stats and Test Counts. India: COVID-19 India.
India, Bihar	COVID-19 India. India COVID-19 Crowdsourced Patient Database: State Level Daily Changes. India: COVID-19 India.
India, Chhattisgarh	COVID-19 India. India COVID-19 Crowdsourced Patient Database: National Level Time Series, State-Wise Stats and Test Counts. India: COVID-19 India.
India, Chhattisgarh	COVID-19 India. India COVID-19 Crowdsourced Patient Database: State Level Daily Changes. India: COVID-19 India.
India, Dadra and Nagar Haveli and Daman and Diu	COVID-19 India. India COVID-19 Crowdsourced Patient Database: National Level Time Series, State-Wise Stats and Test Counts. India: COVID-19 India.
India, Dadra and Nagar Haveli and Daman and Diu	COVID-19 India. India COVID-19 Crowdsourced Patient Database: State Level Daily Changes. India: COVID-19 India.
India, Delhi	COVID-19 India. India COVID-19 Crowdsourced Patient Database: National Level Time Series, State-Wise Stats and Test Counts. India: COVID-19 India.
India, Delhi	COVID-19 India. India COVID-19 Crowdsourced Patient Database: State Level Daily Changes. India: COVID-19 India.
India, Goa	COVID-19 India. India COVID-19 Crowdsourced Patient Database: National Level Time Series, State-Wise Stats and Test Counts. India: COVID-19 India.
India, Goa	COVID-19 India. India COVID-19 Crowdsourced Patient Database: State Level Daily Changes. India: COVID-19 India.
India, Gujarat	COVID-19 India. India COVID-19 Crowdsourced Patient Database: National Level Time Series, State-Wise Stats and Test Counts. India: COVID-19 India.
India, Gujarat	COVID-19 India. India COVID-19 Crowdsourced Patient Database: State Level Daily Changes. India: COVID-19 India.
India, Haryana	COVID-19 India. India COVID-19 Crowdsourced Patient Database: National Level Time Series, State-Wise Stats and Test Counts. India: COVID-19 India.
India, Haryana	COVID-19 India. India COVID-19 Crowdsourced Patient Database: State Level Daily Changes. India: COVID-19 India.

India, Himachal Pradesh	COVID-19 India. India COVID-19 Crowdsourced Patient Database: National Level Time Series, State-Wise Stats and Test Counts. India: COVID-19 India.
India, Himachal Pradesh	COVID-19 India. India COVID-19 Crowdsourced Patient Database: State Level Daily Changes. India: COVID-19 India.
India, Jammu & Kashmir and Ladakh	COVID-19 India. India COVID-19 Crowdsourced Patient Database: National Level Time Series, State-Wise Stats and Test Counts. India: COVID-19 India.
India, Jammu & Kashmir and Ladakh	COVID-19 India. India COVID-19 Crowdsourced Patient Database: State Level Daily Changes. India: COVID-19 India.
India, Jharkhand	COVID-19 India. India COVID-19 Crowdsourced Patient Database: National Level Time Series, State-Wise Stats and Test Counts. India: COVID-19 India.
India, Jharkhand	COVID-19 India. India COVID-19 Crowdsourced Patient Database: State Level Daily Changes. India: COVID-19 India.
India, Karnataka	COVID-19 India. India COVID-19 Crowdsourced Patient Database: National Level Time Series, State-Wise Stats and Test Counts. India: COVID-19 India.
India, Karnataka	COVID-19 India. India COVID-19 Crowdsourced Patient Database: State Level Daily Changes. India: COVID-19 India.
India, Kerala	COVID-19 India. India COVID-19 Crowdsourced Patient Database: National Level Time Series, State-Wise Stats and Test Counts. India: COVID-19 India.
India, Kerala	COVID-19 India. India COVID-19 Crowdsourced Patient Database: State Level Daily Changes. India: COVID-19 India.
India, Madhya Pradesh	COVID-19 India. India COVID-19 Crowdsourced Patient Database: National Level Time Series, State-Wise Stats and Test Counts. India: COVID-19 India.
India, Madhya Pradesh	COVID-19 India. India COVID-19 Crowdsourced Patient Database: State Level Daily Changes. India: COVID-19 India.
India, Maharashtra	COVID-19 India. India COVID-19 Crowdsourced Patient Database: National Level Time Series, State-Wise Stats and Test Counts. India: COVID-19 India.
India, Maharashtra	COVID-19 India. India COVID-19 Crowdsourced Patient Database: State Level Daily Changes. India: COVID-19 India.
India, Manipur	COVID-19 India. India COVID-19 Crowdsourced Patient Database: National Level Time Series, State-Wise Stats and Test Counts. India: COVID-19 India.
India, Manipur	COVID-19 India. India COVID-19 Crowdsourced Patient Database: State Level Daily Changes. India: COVID-19 India.
India, Meghalaya	COVID-19 India. India COVID-19 Crowdsourced Patient Database: National Level Time Series, State-Wise Stats and Test Counts. India: COVID-19 India.
India, Meghalaya	COVID-19 India. India COVID-19 Crowdsourced Patient Database: State Level Daily Changes. India: COVID-19 India.
India, Mizoram	COVID-19 India. India COVID-19 Crowdsourced Patient Database: National Level Time Series, State-Wise Stats and Test Counts. India: COVID-19 India.
India, Mizoram	COVID-19 India. India COVID-19 Crowdsourced Patient Database: State Level Daily Changes. India: COVID-19 India.
India, Nagaland	COVID-19 India. India COVID-19 Crowdsourced Patient Database: National Level Time Series, State-Wise Stats and Test Counts. India: COVID-19 India.
India, Nagaland	COVID-19 India. India COVID-19 Crowdsourced Patient Database: State Level Daily Changes. India: COVID-19 India.
India, Odisha	COVID-19 India. India COVID-19 Crowdsourced Patient Database: National Level Time Series, State-Wise Stats and Test Counts. India: COVID-19 India.
India, Odisha	COVID-19 India. India COVID-19 Crowdsourced Patient Database: State Level Daily Changes. India: COVID-19 India.
India, Punjab	COVID-19 India. India COVID-19 Crowdsourced Patient Database: National Level Time Series, State-Wise Stats and Test Counts. India: COVID-19 India.
India, Punjab	COVID-19 India. India COVID-19 Crowdsourced Patient Database: State Level Daily Changes. India: COVID-19 India.
India, Rajasthan	COVID-19 India. India COVID-19 Crowdsourced Patient Database: National Level Time Series, State-Wise Stats and Test Counts. India: COVID-19 India.
India, Rajasthan	COVID-19 India. India COVID-19 Crowdsourced Patient Database: State Level Daily Changes. India: COVID-19 India.
India, Sikkim	COVID-19 India. India COVID-19 Crowdsourced Patient Database: National Level Time Series, State-Wise Stats and Test Counts. India: COVID-19 India.
India, Sikkim	COVID-19 India. India COVID-19 Crowdsourced Patient Database: State Level Daily Changes. India: COVID-19 India.
India, Tamil Nadu	COVID-19 India. India COVID-19 Crowdsourced Patient Database: National Level Time Series, State-Wise Stats and Test Counts. India: COVID-19 India.
India, Tamil Nadu	COVID-19 India. India COVID-19 Crowdsourced Patient Database: State Level Daily Changes. India: COVID-19 India.
India, Telangana	COVID-19 India. India COVID-19 Crowdsourced Patient Database: National Level Time Series, State-Wise Stats and Test Counts. India: COVID-19 India.
India, Telangana	COVID-19 India. India COVID-19 Crowdsourced Patient Database: State Level Daily Changes. India: COVID-19 India.
India, Tripura	COVID-19 India. India COVID-19 Crowdsourced Patient Database: National Level Time Series, State-Wise Stats and Test Counts. India: COVID-19 India.
India, Tripura	COVID-19 India. India COVID-19 Crowdsourced Patient Database: State Level Daily Changes. India: COVID-19 India.
India, Uttar Pradesh	COVID-19 India. India COVID-19 Crowdsourced Patient Database: National Level Time Series, State-Wise Stats and Test Counts. India: COVID-19 India.

India, Uttar Pradesh	COVID-19 India. India COVID-19 Crowdsourced Patient Database: State Level Daily Changes. India: COVID-19 India.
India, Uttarakhand	COVID-19 India. India COVID-19 Crowdsourced Patient Database: National Level Time Series, State-Wise Stats and Test Counts. India: COVID-19 India.
India, Uttarakhand	COVID-19 India. India COVID-19 Crowdsourced Patient Database: State Level Daily Changes. India: COVID-19 India.
India, West Bengal	COVID-19 India. India COVID-19 Crowdsourced Patient Database: National Level Time Series, State-Wise Stats and Test Counts. India: COVID-19 India.
India, West Bengal	COVID-19 India. India COVID-19 Crowdsourced Patient Database: State Level Daily Changes. India: COVID-19 India.
Indonesia	Johns Hopkins University. 2019 Novel Coronavirus COVID-19 (2019-nCoV) Data Repository by Johns Hopkins CSSE. Baltimore, Maryland: Johns Hopkins University.
Iran	Johns Hopkins University. 2019 Novel Coronavirus COVID-19 (2019-nCoV) Data Repository by Johns Hopkins CSSE. Baltimore, Maryland: Johns Hopkins University.
Iraq	Johns Hopkins University. 2019 Novel Coronavirus COVID-19 (2019-nCoV) Data Repository by Johns Hopkins CSSE. Baltimore, Maryland: Johns Hopkins University.
Ireland	Johns Hopkins University. 2019 Novel Coronavirus COVID-19 (2019-nCoV) Data Repository by Johns Hopkins CSSE. Baltimore, Maryland: Johns Hopkins University.
Israel	Johns Hopkins University. 2019 Novel Coronavirus COVID-19 (2019-nCoV) Data Repository by Johns Hopkins CSSE. Baltimore, Maryland: Johns Hopkins University.
Italy, Abruzzo	Department of Civil Protection (Italy). Italy COVID-19 Situation Monitoring - Department of Civil Protection. Rome, Italy: Department of Civil Protection (Italy).
Italy, Basilicata	Department of Civil Protection (Italy). Italy COVID-19 Situation Monitoring - Department of Civil Protection. Rome, Italy: Department of Civil Protection (Italy).
Italy, Calabria	Department of Civil Protection (Italy). Italy COVID-19 Situation Monitoring - Department of Civil Protection. Rome, Italy: Department of Civil Protection (Italy).
Italy, Campania	Department of Civil Protection (Italy). Italy COVID-19 Situation Monitoring - Department of Civil Protection. Rome, Italy: Department of Civil Protection (Italy).
Italy, Emilia-Romagna	Department of Civil Protection (Italy). Italy COVID-19 Situation Monitoring - Department of Civil Protection. Rome, Italy: Department of Civil Protection (Italy).
Italy, Friuli-Venezia Giulia	Department of Civil Protection (Italy). Italy COVID-19 Situation Monitoring - Department of Civil Protection. Rome, Italy: Department of Civil Protection (Italy).
Italy, Lazio	Department of Civil Protection (Italy). Italy COVID-19 Situation Monitoring - Department of Civil Protection. Rome, Italy: Department of Civil Protection (Italy).
Italy, Liguria	Department of Civil Protection (Italy). Italy COVID-19 Situation Monitoring - Department of Civil Protection. Rome, Italy: Department of Civil Protection (Italy).
Italy, Lombardia	Department of Civil Protection (Italy). Italy COVID-19 Situation Monitoring - Department of Civil Protection. Rome, Italy: Department of Civil Protection (Italy).
Italy, Marche	Department of Civil Protection (Italy). Italy COVID-19 Situation Monitoring - Department of Civil Protection. Rome, Italy: Department of Civil Protection (Italy).
Italy, Molise	Department of Civil Protection (Italy). Italy COVID-19 Situation Monitoring - Department of Civil Protection. Rome, Italy: Department of Civil Protection (Italy).
Italy, Piemonte	Department of Civil Protection (Italy). Italy COVID-19 Situation Monitoring - Department of Civil Protection. Rome, Italy: Department of Civil Protection (Italy).
Italy, Prov. autonoma di Bolzano	Department of Civil Protection (Italy). Italy COVID-19 Situation Monitoring - Department of Civil Protection. Rome, Italy: Department of Civil Protection (Italy).
Italy, Prov. autonoma di Trento	Department of Civil Protection (Italy). Italy COVID-19 Situation Monitoring - Department of Civil Protection. Rome, Italy: Department of Civil Protection (Italy).
Italy, Puglia	Department of Civil Protection (Italy). Italy COVID-19 Situation Monitoring - Department of Civil Protection. Rome, Italy: Department of Civil Protection (Italy).
Italy, Sardegna	Department of Civil Protection (Italy). Italy COVID-19 Situation Monitoring - Department of Civil Protection. Rome, Italy: Department of Civil Protection (Italy).
Italy, Sicilia	Department of Civil Protection (Italy). Italy COVID-19 Situation Monitoring - Department of Civil Protection. Rome, Italy: Department of Civil Protection (Italy).
Italy, Toscana	Department of Civil Protection (Italy). Italy COVID-19 Situation Monitoring - Department of Civil Protection. Rome, Italy: Department of Civil Protection (Italy).
Italy, Umbria	Department of Civil Protection (Italy). Italy COVID-19 Situation Monitoring - Department of Civil Protection. Rome, Italy: Department of Civil Protection (Italy).
Italy, Valle d'Aosta	Department of Civil Protection (Italy). Italy COVID-19 Situation Monitoring - Department of Civil Protection. Rome, Italy: Department of Civil Protection (Italy).
Italy, Veneto	Department of Civil Protection (Italy). Italy COVID-19 Situation Monitoring - Department of Civil Protection. Rome, Italy: Department of Civil Protection (Italy).
Jamaica	Johns Hopkins University. 2019 Novel Coronavirus COVID-19 (2019-nCoV) Data Repository by Johns Hopkins CSSE. Baltimore, Maryland: Johns Hopkins University.
Japan	Ministry of Health, Labour and Welfare (Japan). Japan Coronavirus Disease (COVID-19) Situation Report.
Jordan	Johns Hopkins University. 2019 Novel Coronavirus COVID-19 (2019-nCoV) Data Repository by Johns Hopkins CSSE. Baltimore, Maryland: Johns Hopkins University.

Kazakhstan	Johns Hopkins University. 2019 Novel Coronavirus COVID-19 (2019-nCoV) Data Repository by Johns Hopkins CSSE. Baltimore, Maryland: Johns Hopkins University.
Kenya	Johns Hopkins University. 2019 Novel Coronavirus COVID-19 (2019-nCoV) Data Repository by Johns Hopkins CSSE. Baltimore, Maryland: Johns Hopkins University.
Kiribati	Johns Hopkins University. 2019 Novel Coronavirus COVID-19 (2019-nCoV) Data Repository by Johns Hopkins CSSE. Baltimore, Maryland: Johns Hopkins University.
Kuwait	Johns Hopkins University. 2019 Novel Coronavirus COVID-19 (2019-nCoV) Data Repository by Johns Hopkins CSSE. Baltimore, Maryland: Johns Hopkins University.
Kyrgyzstan	Johns Hopkins University. 2019 Novel Coronavirus COVID-19 (2019-nCoV) Data Repository by Johns Hopkins CSSE. Baltimore, Maryland: Johns Hopkins University.
Laos	Johns Hopkins University. 2019 Novel Coronavirus COVID-19 (2019-nCoV) Data Repository by Johns Hopkins CSSE. Baltimore, Maryland: Johns Hopkins University.
Latvia	Johns Hopkins University. 2019 Novel Coronavirus COVID-19 (2019-nCoV) Data Repository by Johns Hopkins CSSE. Baltimore, Maryland: Johns Hopkins University.
Lebanon	Johns Hopkins University. 2019 Novel Coronavirus COVID-19 (2019-nCoV) Data Repository by Johns Hopkins CSSE. Baltimore, Maryland: Johns Hopkins University.
Lesotho	Johns Hopkins University. 2019 Novel Coronavirus COVID-19 (2019-nCoV) Data Repository by Johns Hopkins CSSE. Baltimore, Maryland: Johns Hopkins University.
Liberia	Johns Hopkins University. 2019 Novel Coronavirus COVID-19 (2019-nCoV) Data Repository by Johns Hopkins CSSE. Baltimore, Maryland: Johns Hopkins University.
Libya	Johns Hopkins University. 2019 Novel Coronavirus COVID-19 (2019-nCoV) Data Repository by Johns Hopkins CSSE. Baltimore, Maryland: Johns Hopkins University.
Lithuania	Johns Hopkins University. 2019 Novel Coronavirus COVID-19 (2019-nCoV) Data Repository by Johns Hopkins CSSE. Baltimore, Maryland: Johns Hopkins University.
Luxembourg	Johns Hopkins University. 2019 Novel Coronavirus COVID-19 (2019-nCoV) Data Repository by Johns Hopkins CSSE. Baltimore, Maryland: Johns Hopkins University.
Madagascar	Johns Hopkins University. 2019 Novel Coronavirus COVID-19 (2019-nCoV) Data Repository by Johns Hopkins CSSE. Baltimore, Maryland: Johns Hopkins University.
Malawi	Johns Hopkins University. 2019 Novel Coronavirus COVID-19 (2019-nCoV) Data Repository by Johns Hopkins CSSE. Baltimore, Maryland: Johns Hopkins University.
Malaysia	Johns Hopkins University. 2019 Novel Coronavirus COVID-19 (2019-nCoV) Data Repository by Johns Hopkins CSSE. Baltimore, Maryland: Johns Hopkins University.
Maldives	Johns Hopkins University. 2019 Novel Coronavirus COVID-19 (2019-nCoV) Data Repository by Johns Hopkins CSSE. Baltimore, Maryland: Johns Hopkins University.
Mali	Johns Hopkins University. 2019 Novel Coronavirus COVID-19 (2019-nCoV) Data Repository by Johns Hopkins CSSE. Baltimore, Maryland: Johns Hopkins University.
Malta	Johns Hopkins University. 2019 Novel Coronavirus COVID-19 (2019-nCoV) Data Repository by Johns Hopkins CSSE. Baltimore, Maryland: Johns Hopkins University.
Marshall Islands	Johns Hopkins University. 2019 Novel Coronavirus COVID-19 (2019-nCoV) Data Repository by Johns Hopkins CSSE. Baltimore, Maryland: Johns Hopkins University.
Mauritania	Johns Hopkins University. 2019 Novel Coronavirus COVID-19 (2019-nCoV) Data Repository by Johns Hopkins CSSE. Baltimore, Maryland: Johns Hopkins University.
Mauritius	Johns Hopkins University. 2019 Novel Coronavirus COVID-19 (2019-nCoV) Data Repository by Johns Hopkins CSSE. Baltimore, Maryland: Johns Hopkins University.
Mexico, Aguascalientes	Directorate General of Epidemiology, Secretariat of Health (Mexico). Mexico General Directorate of Epidemiology COVID-19 Daily Cases, Nationally and by State.
Mexico, Baja California	Directorate General of Epidemiology, Secretariat of Health (Mexico). Mexico General Directorate of Epidemiology COVID-19 Daily Cases, Nationally and by State.
Mexico, Baja California Sur	Directorate General of Epidemiology, Secretariat of Health (Mexico). Mexico General Directorate of Epidemiology COVID-19 Daily Cases, Nationally and by State.
Mexico, Campeche	Directorate General of Epidemiology, Secretariat of Health (Mexico). Mexico General Directorate of Epidemiology COVID-19 Daily Cases, Nationally and by State.
Mexico, Chiapas	Directorate General of Epidemiology, Secretariat of Health (Mexico). Mexico General Directorate of Epidemiology COVID-19 Daily Cases, Nationally and by State.
Mexico, Chihuahua	Directorate General of Epidemiology, Secretariat of Health (Mexico). Mexico General Directorate of Epidemiology COVID-19 Daily Cases, Nationally and by State.
Mexico, Coahuila	Directorate General of Epidemiology, Secretariat of Health (Mexico). Mexico General Directorate of Epidemiology COVID-19 Daily Cases, Nationally and by State.
Mexico, Colima	Directorate General of Epidemiology, Secretariat of Health (Mexico). Mexico General Directorate of Epidemiology COVID-19 Daily Cases, Nationally and by State.
Mexico, Durango	Directorate General of Epidemiology, Secretariat of Health (Mexico). Mexico General Directorate of Epidemiology COVID-19 Daily Cases, Nationally and by State.
Mexico, Guanajuato	Directorate General of Epidemiology, Secretariat of Health (Mexico). Mexico General Directorate of Epidemiology COVID-19 Daily Cases, Nationally and by State.
Mexico, Guerrero	Directorate General of Epidemiology, Secretariat of Health (Mexico). Mexico General Directorate of Epidemiology COVID-19 Daily Cases, Nationally and by State.

Mexico, Hidalgo	Directorate General of Epidemiology, Secretariat of Health (Mexico). Mexico General Directorate of Epidemiology COVID-19 Daily Cases, Nationally and by State.
Mexico, Jalisco	Directorate General of Epidemiology, Secretariat of Health (Mexico). Mexico General Directorate of Epidemiology COVID-19 Daily Cases, Nationally and by State.
Mexico, México	Directorate General of Epidemiology, Secretariat of Health (Mexico). Mexico General Directorate of Epidemiology COVID-19 Daily Cases, Nationally and by State.
Mexico, Mexico City	Directorate General of Epidemiology, Secretariat of Health (Mexico). Mexico General Directorate of Epidemiology COVID-19 Daily Cases, Nationally and by State.
Mexico, Michoacán de Ocampo	Directorate General of Epidemiology, Secretariat of Health (Mexico). Mexico General Directorate of Epidemiology COVID-19 Daily Cases, Nationally and by State.
Mexico, Morelos	Directorate General of Epidemiology, Secretariat of Health (Mexico). Mexico General Directorate of Epidemiology COVID-19 Daily Cases, Nationally and by State.
Mexico, Nayarit	Directorate General of Epidemiology, Secretariat of Health (Mexico). Mexico General Directorate of Epidemiology COVID-19 Daily Cases, Nationally and by State.
Mexico, Nuevo León	Directorate General of Epidemiology, Secretariat of Health (Mexico). Mexico General Directorate of Epidemiology COVID-19 Daily Cases, Nationally and by State.
Mexico, Oaxaca	Directorate General of Epidemiology, Secretariat of Health (Mexico). Mexico General Directorate of Epidemiology COVID-19 Daily Cases, Nationally and by State.
Mexico, Puebla	Directorate General of Epidemiology, Secretariat of Health (Mexico). Mexico General Directorate of Epidemiology COVID-19 Daily Cases, Nationally and by State.
Mexico, Querétaro	Directorate General of Epidemiology, Secretariat of Health (Mexico). Mexico General Directorate of Epidemiology COVID-19 Daily Cases, Nationally and by State.
Mexico, Quintana Roo	Directorate General of Epidemiology, Secretariat of Health (Mexico). Mexico General Directorate of Epidemiology COVID-19 Daily Cases, Nationally and by State.
Mexico, San Luis Potosí	Directorate General of Epidemiology, Secretariat of Health (Mexico). Mexico General Directorate of Epidemiology COVID-19 Daily Cases, Nationally and by State.
Mexico, Sinaloa	Directorate General of Epidemiology, Secretariat of Health (Mexico). Mexico General Directorate of Epidemiology COVID-19 Daily Cases, Nationally and by State.
Mexico, Sonora	Directorate General of Epidemiology, Secretariat of Health (Mexico). Mexico General Directorate of Epidemiology COVID-19 Daily Cases, Nationally and by State.
Mexico, Tabasco	Directorate General of Epidemiology, Secretariat of Health (Mexico). Mexico General Directorate of Epidemiology COVID-19 Daily Cases, Nationally and by State.
Mexico, Tamaulipas	Directorate General of Epidemiology, Secretariat of Health (Mexico). Mexico General Directorate of Epidemiology COVID-19 Daily Cases, Nationally and by State.
Mexico, Tlaxcala	Directorate General of Epidemiology, Secretariat of Health (Mexico). Mexico General Directorate of Epidemiology COVID-19 Daily Cases, Nationally and by State.
Mexico, Veracruz de Ignacio de la Llave	Directorate General of Epidemiology, Secretariat of Health (Mexico). Mexico General Directorate of Epidemiology COVID-19 Daily Cases, Nationally and by State.
Mexico, Yucatán	Directorate General of Epidemiology, Secretariat of Health (Mexico). Mexico General Directorate of Epidemiology COVID-19 Daily Cases, Nationally and by State.
Mexico, Zacatecas	Directorate General of Epidemiology, Secretariat of Health (Mexico). Mexico General Directorate of Epidemiology COVID-19 Daily Cases, Nationally and by State.
Moldova	Johns Hopkins University. 2019 Novel Coronavirus COVID-19 (2019-nCoV) Data Repository by Johns Hopkins CSSE. Baltimore, Maryland: Johns Hopkins University.
Monaco	Johns Hopkins University. 2019 Novel Coronavirus COVID-19 (2019-nCoV) Data Repository by Johns Hopkins CSSE. Baltimore, Maryland: Johns Hopkins University.
Mongolia	Johns Hopkins University. 2019 Novel Coronavirus COVID-19 (2019-nCoV) Data Repository by Johns Hopkins CSSE. Baltimore, Maryland: Johns Hopkins University.
Montenegro	Johns Hopkins University. 2019 Novel Coronavirus COVID-19 (2019-nCoV) Data Repository by Johns Hopkins CSSE. Baltimore, Maryland: Johns Hopkins University.
Morocco	Johns Hopkins University. 2019 Novel Coronavirus COVID-19 (2019-nCoV) Data Repository by Johns Hopkins CSSE. Baltimore, Maryland: Johns Hopkins University.
Mozambique	Johns Hopkins University. 2019 Novel Coronavirus COVID-19 (2019-nCoV) Data Repository by Johns Hopkins CSSE. Baltimore, Maryland: Johns Hopkins University.
Myanmar	Johns Hopkins University. 2019 Novel Coronavirus COVID-19 (2019-nCoV) Data Repository by Johns Hopkins CSSE. Baltimore, Maryland: Johns Hopkins University.
Namibia	Johns Hopkins University. 2019 Novel Coronavirus COVID-19 (2019-nCoV) Data Repository by Johns Hopkins CSSE. Baltimore, Maryland: Johns Hopkins University.
Nepal	Johns Hopkins University. 2019 Novel Coronavirus COVID-19 (2019-nCoV) Data Repository by Johns Hopkins CSSE. Baltimore, Maryland: Johns Hopkins University.
Netherlands	Johns Hopkins University. 2019 Novel Coronavirus COVID-19 (2019-nCoV) Data Repository by Johns Hopkins CSSE. Baltimore, Maryland: Johns Hopkins University.
New Zealand	Ministry of Health (New Zealand). New Zealand COVID-19 Dashboard - Ministry of Health.
Nicaragua	Johns Hopkins University. 2019 Novel Coronavirus COVID-19 (2019-nCoV) Data Repository by Johns Hopkins CSSE. Baltimore, Maryland: Johns Hopkins University.
Niger	Johns Hopkins University. 2019 Novel Coronavirus COVID-19 (2019-nCoV) Data Repository by Johns Hopkins CSSE. Baltimore, Maryland: Johns Hopkins University.

Nigeria	Johns Hopkins University. 2019 Novel Coronavirus COVID-19 (2019-nCoV) Data Repository by Johns Hopkins CSSE. Baltimore, Maryland: Johns Hopkins University.
North Macedonia	Johns Hopkins University. 2019 Novel Coronavirus COVID-19 (2019-nCoV) Data Repository by Johns Hopkins CSSE. Baltimore, Maryland: Johns Hopkins University.
Northern Mariana Islands	Johns Hopkins University. 2019 Novel Coronavirus COVID-19 (2019-nCoV) Data Repository by Johns Hopkins CSSE. Baltimore, Maryland: Johns Hopkins University.
Norway	Johns Hopkins University. 2019 Novel Coronavirus COVID-19 (2019-nCoV) Data Repository by Johns Hopkins CSSE. Baltimore, Maryland: Johns Hopkins University.
Oman	Johns Hopkins University. 2019 Novel Coronavirus COVID-19 (2019-nCoV) Data Repository by Johns Hopkins CSSE. Baltimore, Maryland: Johns Hopkins University.
Pakistan, Azad Jammu & Kashmir	Ministry of National Health Services, Regulations & Coordination (Pakistan). Pakistan - Azad Jammu and Kashmir COVID-19 Statistics. Islamabad, Pakistan: Government of Pakistan.
Pakistan, Azad Jammu & Kashmir	Ministry of National Health Services, Regulations & Coordination (Pakistan). Pakistan COVID-19 Dashboard.
Pakistan, Balochistan	Ministry of National Health Services, Regulations & Coordination (Pakistan). Pakistan - Balochistan COVID-19 Statistics. Islamabad, Pakistan: Government of Pakistan.
Pakistan, Balochistan	Ministry of National Health Services, Regulations & Coordination (Pakistan). Pakistan COVID-19 Dashboard.
Pakistan, Gilgit-Baltistan	Ministry of National Health Services, Regulations & Coordination (Pakistan). Pakistan - Gilgit-Baltistan COVID-19 Statistics. Islamabad, Pakistan: Government of Pakistan.
Pakistan, Gilgit-Baltistan	Ministry of National Health Services, Regulations & Coordination (Pakistan). Pakistan COVID-19 Dashboard.
Pakistan, Islamabad Capital Territory	Ministry of National Health Services, Regulations & Coordination (Pakistan). Pakistan - Islamabad COVID-19 Statistics. Islamabad, Pakistan: Government of Pakistan.
Pakistan, Islamabad Capital Territory	Ministry of National Health Services, Regulations & Coordination (Pakistan). Pakistan COVID-19 Dashboard.
Pakistan, Khyber Pakhtunkhwa	Ministry of National Health Services, Regulations & Coordination (Pakistan). Pakistan - Khyber Pakhtunkhwa COVID-19 Statistics 2020.
Pakistan, Khyber Pakhtunkhwa	Ministry of National Health Services, Regulations & Coordination (Pakistan). Pakistan COVID-19 Dashboard.
Pakistan, Punjab	Ministry of National Health Services, Regulations & Coordination (Pakistan). Pakistan - Punjab COVID-19 Statistics. Islamabad, Pakistan: Government of Pakistan.
Pakistan, Punjab	Ministry of National Health Services, Regulations & Coordination (Pakistan). Pakistan COVID-19 Dashboard.
Pakistan, Sindh	Wikipedia. COVID-19 pandemic in Pakistan. San Francisco, United States of America: Wikipedia. Retrieved on April 29, 2021 from https://en.wikipedia.org/wiki/COVID-19_pandemic_in_Pakistan
Pakistan, Sindh	Ministry of National Health Services, Regulations & Coordination (Pakistan). Pakistan - Sindh COVID-19 Statistics. Islamabad, Pakistan: Government of Pakistan.
Pakistan, Sindh	Ministry of National Health Services, Regulations & Coordination (Pakistan). Pakistan COVID-19 Dashboard.
Palau	Johns Hopkins University. 2019 Novel Coronavirus COVID-19 (2019-nCoV) Data Repository by Johns Hopkins CSSE. Baltimore, Maryland: Johns Hopkins University.
Palestine	Johns Hopkins University. 2019 Novel Coronavirus COVID-19 (2019-nCoV) Data Repository by Johns Hopkins CSSE. Baltimore, Maryland: Johns Hopkins University.
Panama	Johns Hopkins University. 2019 Novel Coronavirus COVID-19 (2019-nCoV) Data Repository by Johns Hopkins CSSE. Baltimore, Maryland: Johns Hopkins University.
Papua New Guinea	Johns Hopkins University. 2019 Novel Coronavirus COVID-19 (2019-nCoV) Data Repository by Johns Hopkins CSSE. Baltimore, Maryland: Johns Hopkins University.
Paraguay	Johns Hopkins University. 2019 Novel Coronavirus COVID-19 (2019-nCoV) Data Repository by Johns Hopkins CSSE. Baltimore, Maryland: Johns Hopkins University.
Peru	Johns Hopkins University. 2019 Novel Coronavirus COVID-19 (2019-nCoV) Data Repository by Johns Hopkins CSSE. Baltimore, Maryland: Johns Hopkins University.
Philippines	Johns Hopkins University. 2019 Novel Coronavirus COVID-19 (2019-nCoV) Data Repository by Johns Hopkins CSSE. Baltimore, Maryland: Johns Hopkins University.
Poland	Johns Hopkins University. 2019 Novel Coronavirus COVID-19 (2019-nCoV) Data Repository by Johns Hopkins CSSE. Baltimore, Maryland: Johns Hopkins University.
Portugal	Johns Hopkins University. 2019 Novel Coronavirus COVID-19 (2019-nCoV) Data Repository by Johns Hopkins CSSE. Baltimore, Maryland: Johns Hopkins University.
Puerto Rico	Johns Hopkins University. 2019 Novel Coronavirus COVID-19 (2019-nCoV) Data Repository by Johns Hopkins CSSE. Baltimore, Maryland: Johns Hopkins University.
Qatar	Johns Hopkins University. 2019 Novel Coronavirus COVID-19 (2019-nCoV) Data Repository by Johns Hopkins CSSE. Baltimore, Maryland: Johns Hopkins University.
Romania	Johns Hopkins University. 2019 Novel Coronavirus COVID-19 (2019-nCoV) Data Repository by Johns Hopkins CSSE. Baltimore, Maryland: Johns Hopkins University.
Russia	Johns Hopkins University. 2019 Novel Coronavirus COVID-19 (2019-nCoV) Data Repository by Johns Hopkins CSSE. Baltimore, Maryland: Johns Hopkins University.
Rwanda	Johns Hopkins University. 2019 Novel Coronavirus COVID-19 (2019-nCoV) Data Repository by Johns Hopkins CSSE. Baltimore, Maryland: Johns Hopkins University.

Saint Kitts and Nevis	Johns Hopkins University. 2019 Novel Coronavirus COVID-19 (2019-nCoV) Data Repository by Johns Hopkins CSSE. Baltimore, Maryland: Johns Hopkins University.
Saint Lucia	Johns Hopkins University. 2019 Novel Coronavirus COVID-19 (2019-nCoV) Data Repository by Johns Hopkins CSSE. Baltimore, Maryland: Johns Hopkins University.
Saint Vincent and the Grenadines	Johns Hopkins University. 2019 Novel Coronavirus COVID-19 (2019-nCoV) Data Repository by Johns Hopkins CSSE. Baltimore, Maryland: Johns Hopkins University.
Samoa	Johns Hopkins University. 2019 Novel Coronavirus COVID-19 (2019-nCoV) Data Repository by Johns Hopkins CSSE. Baltimore, Maryland: Johns Hopkins University.
San Marino	Johns Hopkins University. 2019 Novel Coronavirus COVID-19 (2019-nCoV) Data Repository by Johns Hopkins CSSE. Baltimore, Maryland: Johns Hopkins University.
São Tomé and Príncipe	Johns Hopkins University. 2019 Novel Coronavirus COVID-19 (2019-nCoV) Data Repository by Johns Hopkins CSSE. Baltimore, Maryland: Johns Hopkins University.
Saudi Arabia	Johns Hopkins University. 2019 Novel Coronavirus COVID-19 (2019-nCoV) Data Repository by Johns Hopkins CSSE. Baltimore, Maryland: Johns Hopkins University.
Senegal	Johns Hopkins University. 2019 Novel Coronavirus COVID-19 (2019-nCoV) Data Repository by Johns Hopkins CSSE. Baltimore, Maryland: Johns Hopkins University.
Serbia	Johns Hopkins University. 2019 Novel Coronavirus COVID-19 (2019-nCoV) Data Repository by Johns Hopkins CSSE. Baltimore, Maryland: Johns Hopkins University.
Seychelles	Johns Hopkins University. 2019 Novel Coronavirus COVID-19 (2019-nCoV) Data Repository by Johns Hopkins CSSE. Baltimore, Maryland: Johns Hopkins University.
Sierra Leone	Johns Hopkins University. 2019 Novel Coronavirus COVID-19 (2019-nCoV) Data Repository by Johns Hopkins CSSE. Baltimore, Maryland: Johns Hopkins University.
Singapore	Johns Hopkins University. 2019 Novel Coronavirus COVID-19 (2019-nCoV) Data Repository by Johns Hopkins CSSE. Baltimore, Maryland: Johns Hopkins University.
Slovakia	Johns Hopkins University. 2019 Novel Coronavirus COVID-19 (2019-nCoV) Data Repository by Johns Hopkins CSSE. Baltimore, Maryland: Johns Hopkins University.
Slovenia	Johns Hopkins University. 2019 Novel Coronavirus COVID-19 (2019-nCoV) Data Repository by Johns Hopkins CSSE. Baltimore, Maryland: Johns Hopkins University.
Solomon Islands	Johns Hopkins University. 2019 Novel Coronavirus COVID-19 (2019-nCoV) Data Repository by Johns Hopkins CSSE. Baltimore, Maryland: Johns Hopkins University.
Somalia	Johns Hopkins University. 2019 Novel Coronavirus COVID-19 (2019-nCoV) Data Repository by Johns Hopkins CSSE. Baltimore, Maryland: Johns Hopkins University.
South Africa	Johns Hopkins University. 2019 Novel Coronavirus COVID-19 (2019-nCoV) Data Repository by Johns Hopkins CSSE. Baltimore, Maryland: Johns Hopkins University.
South Korea	Johns Hopkins University. 2019 Novel Coronavirus COVID-19 (2019-nCoV) Data Repository by Johns Hopkins CSSE. Baltimore, Maryland: Johns Hopkins University.
South Sudan	Johns Hopkins University. 2019 Novel Coronavirus COVID-19 (2019-nCoV) Data Repository by Johns Hopkins CSSE. Baltimore, Maryland: Johns Hopkins University.
Spain, Andalusia	Andalusia Ministry of Health and Families (Spain). Spain - Andalusia Ministry of Health and Families Coronavirus Report.
Spain, Aragon	Ministry of Health, Consumption and Social Welfare (Spain). Spain Ministry of Health, Consumption, and Social Welfare COVID-19 Situation Update. Spain: Ministry of Health, Consumption and Social Welfare (Spain).
Spain, Aragon	Institute of Health Carlos III (Spain), Ministry of Health, Consumption and Social Welfare (Spain). Spain Carlos III Health Institute Situation of COVID-19. Madrid, Spain: Institute of Health Carlos III (Spain).
Spain, Asturias	Ministry of Health, Consumption and Social Welfare (Spain). Spain Ministry of Health, Consumption, and Social Welfare COVID-19 Situation Update. Spain: Ministry of Health, Consumption and Social Welfare (Spain).
Spain, Asturias	Institute of Health Carlos III (Spain), Ministry of Health, Consumption and Social Welfare (Spain). Spain Carlos III Health Institute Situation of COVID-19. Madrid, Spain: Institute of Health Carlos III (Spain).
Spain, Balearic Islands	Government of the Balearic Islands. Spain - Balearic Islands Ministry of Health and Consumption News About the Coronavirus COVID-19.
Spain, Basque Country	Basque Government Department of Health. Spain - Basque Country Information on the Evolution of the Coronavirus Epidemiological Bulletin.
Spain, Canary Islands	Government of the Canary Islands (Spain). Spain - Canary Islands Government COVID-19 Dashboard.
Spain, Cantabria	Cantabrian Health Service (Spain), Government of Cantabria (Spain). Spain - Cantabria Epidemiological Situation of COVID-19. Spain: Cantabrian Health Service (Spain).
Spain, Castile and León	Castile and León Board, Health Commission (Spain). Spain - Castile and León Open Data: Coronavirus (COVID-19) Epidemiological Situation.
Spain, Castile and León	Castile and León Board, Health Commission (Spain). Spain - Castile and León Open Data: Situation of Hospitalized by Coronavirus.
Spain, Castilla–La Mancha	Ministry of Health, Consumption and Social Welfare (Spain). Spain Ministry of Health, Consumption, and Social Welfare COVID-19 Situation Update. Spain: Ministry of Health, Consumption and Social Welfare (Spain).
Spain, Castilla–La Mancha	Institute of Health Carlos III (Spain), Ministry of Health, Consumption and Social Welfare (Spain). Spain Carlos III Health Institute Situation of COVID-19. Madrid, Spain: Institute of Health Carlos III (Spain).
Spain, Catalonia	Statistical Institute of Catalonia (IDESCAT) (Spain). Spain - Catalonia COVID-19 Weekly Confirmed Cases and Deaths. Barcelona, Spain: Statistical Institute of Catalonia (IDESCAT) (Spain).
Spain, Catalonia	Statistical Institute of Catalonia (IDESCAT) (Spain). Spain - Catalonia COVID-19 Daily Confirmed Cases and Deaths 2020. Barcelona, Spain: Statistical Institute of Catalonia (IDESCAT) (Spain), 2020.

Spain, Ceuta	Ministry of Health, Consumption and Social Welfare (Spain). Spain Ministry of Health, Consumption, and Social Welfare COVID-19 Situation Update. Spain: Ministry of Health, Consumption and Social Welfare (Spain).
Spain, Ceuta	Institute of Health Carlos III (Spain), Ministry of Health, Consumption and Social Welfare (Spain). Spain Carlos III Health Institute Situation of COVID-19. Madrid, Spain: Institute of Health Carlos III (Spain).
Spain, Community of Madrid	Ministry of Health, Consumption and Social Welfare (Spain). Spain Ministry of Health, Consumption, and Social Welfare COVID-19 Situation Update. Spain: Ministry of Health, Consumption and Social Welfare (Spain).
Spain, Community of Madrid	City of Madrid (Spain), Madrid Health Service (Spain). Spain - Madrid Health Service COVID-19 Current Situation Daily Status Report. Madrid, Spain: City of Madrid (Spain).
Spain, Extremadura	Ministry of Health, Consumption and Social Welfare (Spain). Spain Ministry of Health, Consumption, and Social Welfare COVID-19 Situation Update. Spain: Ministry of Health, Consumption and Social Welfare (Spain).
Spain, Extremadura	Institute of Health Carlos III (Spain), Ministry of Health, Consumption and Social Welfare (Spain). Spain Carlos III Health Institute Situation of COVID-19. Madrid, Spain: Institute of Health Carlos III (Spain).
Spain, Galicia	Galician Healthcare Service (Spain), Regional Government of Galicia (Spain). Spain - Galicia Regional Government COVID-19 Press Releases 2020. Spain: Regional Government of Galicia (Spain).
Spain, La Rioja	Government of La Rioja (Spain). Spain - La Rioja Covid-19 Tests Performed per Days. Brazil: Government of La Rioja (Spain).
Spain, Melilla	Ministry of Health, Consumption and Social Welfare (Spain). Spain Ministry of Health, Consumption, and Social Welfare COVID-19 Situation Update. Spain: Ministry of Health, Consumption and Social Welfare (Spain).
Spain, Melilla	Institute of Health Carlos III (Spain), Ministry of Health, Consumption and Social Welfare (Spain). Spain Carlos III Health Institute Situation of COVID-19. Madrid, Spain: Institute of Health Carlos III (Spain).
Spain, Murcia	Institute of Health Carlos III (Spain), Ministry of Health, Consumption and Social Welfare (Spain). Spain Carlos III Health Institute Situation of COVID-19. Madrid, Spain: Institute of Health Carlos III (Spain).
Spain, Murcia	Ministry of Health of the Region of Murcia (Spain). COVID-19 Region of Murcia - Spain. Spain: Ministry of Health of the Region of Murcia (Spain).
Spain, Navarre	Government of Navarra (Spain). Spain - Navarra COVID-19 Testing, Deaths, Hospitalisation Data May-June 2020. Spain: Government of Navarra (Spain), 2020.
Spain, Valencian Community	Valencia Ministry of Universal Health and Public Health (Spain). Spain - Valencia COVID-19: Monitoring of the Situation. Spain: Valencia Ministry of Universal Health and Public Health (Spain).
Sri Lanka	Johns Hopkins University. 2019 Novel Coronavirus COVID-19 (2019-nCoV) Data Repository by Johns Hopkins CSSE. Baltimore, Maryland: Johns Hopkins University.
Sudan	Johns Hopkins University. 2019 Novel Coronavirus COVID-19 (2019-nCoV) Data Repository by Johns Hopkins CSSE. Baltimore, Maryland: Johns Hopkins University.
Suriname	Johns Hopkins University. 2019 Novel Coronavirus COVID-19 (2019-nCoV) Data Repository by Johns Hopkins CSSE. Baltimore, Maryland: Johns Hopkins University.
Sweden	Public Health Agency of Sweden. Sweden Public Health Agency COVID-19 Confirmed Cases Daily Update. Östersund, Sweden: Public Health Agency of Sweden.
Switzerland	Federal Office of Public Health (Switzerland). Switzerland Federal Office of Public Health New Coronavirus Current Situation. Berne, Switzerland: Federal Office of Public Health (Switzerland).
Syria	Johns Hopkins University. 2019 Novel Coronavirus COVID-19 (2019-nCoV) Data Repository by Johns Hopkins CSSE. Baltimore, Maryland: Johns Hopkins University.
Taiwan (Province of China)	Johns Hopkins University. 2019 Novel Coronavirus COVID-19 (2019-nCoV) Data Repository by Johns Hopkins CSSE. Baltimore, Maryland: Johns Hopkins University.
Tajikistan	Johns Hopkins University. 2019 Novel Coronavirus COVID-19 (2019-nCoV) Data Repository by Johns Hopkins CSSE. Baltimore, Maryland: Johns Hopkins University.
Tanzania	Johns Hopkins University. 2019 Novel Coronavirus COVID-19 (2019-nCoV) Data Repository by Johns Hopkins CSSE. Baltimore, Maryland: Johns Hopkins University.
Thailand	Johns Hopkins University. 2019 Novel Coronavirus COVID-19 (2019-nCoV) Data Repository by Johns Hopkins CSSE. Baltimore, Maryland: Johns Hopkins University.
The Bahamas	Johns Hopkins University. 2019 Novel Coronavirus COVID-19 (2019-nCoV) Data Repository by Johns Hopkins CSSE. Baltimore, Maryland: Johns Hopkins University.
The Gambia	Johns Hopkins University. 2019 Novel Coronavirus COVID-19 (2019-nCoV) Data Repository by Johns Hopkins CSSE. Baltimore, Maryland: Johns Hopkins University.
Timor-Leste	Johns Hopkins University. 2019 Novel Coronavirus COVID-19 (2019-nCoV) Data Repository by Johns Hopkins CSSE. Baltimore, Maryland: Johns Hopkins University.
Togo	Johns Hopkins University. 2019 Novel Coronavirus COVID-19 (2019-nCoV) Data Repository by Johns Hopkins CSSE. Baltimore, Maryland: Johns Hopkins University.
Tonga	Johns Hopkins University. 2019 Novel Coronavirus COVID-19 (2019-nCoV) Data Repository by Johns Hopkins CSSE. Baltimore, Maryland: Johns Hopkins University.
Trinidad and Tobago	Johns Hopkins University. 2019 Novel Coronavirus COVID-19 (2019-nCoV) Data Repository by Johns Hopkins CSSE. Baltimore, Maryland: Johns Hopkins University.
Tunisia	Johns Hopkins University. 2019 Novel Coronavirus COVID-19 (2019-nCoV) Data Repository by Johns Hopkins CSSE. Baltimore, Maryland: Johns Hopkins University.
Uganda	Johns Hopkins University. 2019 Novel Coronavirus COVID-19 (2019-nCoV) Data Repository by Johns Hopkins CSSE. Baltimore, Maryland: Johns Hopkins University.
UK, England	Department of Health (United Kingdom), NHS England, Public Health England. United Kingdom COVID-19 Cases. London, England: Public Health England, 2020.

UK, England	National Health Service (United Kingdom), Public Health England. United Kingdom Coronavirus (COVID-19) Data Dashboard.
UK, Northern Ireland	Department of Health (United Kingdom), NHS England, Public Health England. United Kingdom COVID-19 Cases. London, England: Public Health England, 2020.
UK, Northern Ireland	National Health Service (United Kingdom), Public Health England. United Kingdom Coronavirus (COVID-19) Data Dashboard.
UK, Scotland	Department of Health (United Kingdom), NHS England, Public Health England. United Kingdom COVID-19 Cases. London, England: Public Health England, 2020.
UK, Scotland	National Health Service (United Kingdom), Public Health England. United Kingdom Coronavirus (COVID-19) Data Dashboard.
UK, Wales	Department of Health (United Kingdom), NHS England, Public Health England. United Kingdom COVID-19 Cases. London, England: Public Health England, 2020.
UK, Wales	National Health Service (United Kingdom), Public Health England. United Kingdom Coronavirus (COVID-19) Data Dashboard.
Ukraine	Johns Hopkins University. 2019 Novel Coronavirus COVID-19 (2019-nCoV) Data Repository by Johns Hopkins CSSE. Baltimore, Maryland: Johns Hopkins University.
United Arab Emirates	Johns Hopkins University. 2019 Novel Coronavirus COVID-19 (2019-nCoV) Data Repository by Johns Hopkins CSSE. Baltimore, Maryland: Johns Hopkins University.
Uruguay	Johns Hopkins University. 2019 Novel Coronavirus COVID-19 (2019-nCoV) Data Repository by Johns Hopkins CSSE. Baltimore, Maryland: Johns Hopkins University.
USA, Alabama	Johns Hopkins University. 2019 Novel Coronavirus COVID-19 (2019-nCoV) Data Repository by Johns Hopkins CSSE. Baltimore, Maryland: Johns Hopkins University.
USA, Alaska	Johns Hopkins University. 2019 Novel Coronavirus COVID-19 (2019-nCoV) Data Repository by Johns Hopkins CSSE. Baltimore, Maryland: Johns Hopkins University.
USA, Arizona	Johns Hopkins University. 2019 Novel Coronavirus COVID-19 (2019-nCoV) Data Repository by Johns Hopkins CSSE. Baltimore, Maryland: Johns Hopkins University.
USA, Arkansas	Johns Hopkins University. 2019 Novel Coronavirus COVID-19 (2019-nCoV) Data Repository by Johns Hopkins CSSE. Baltimore, Maryland: Johns Hopkins University.
USA, California	Johns Hopkins University. 2019 Novel Coronavirus COVID-19 (2019-nCoV) Data Repository by Johns Hopkins CSSE. Baltimore, Maryland: Johns Hopkins University.
USA, Colorado	Johns Hopkins University. 2019 Novel Coronavirus COVID-19 (2019-nCoV) Data Repository by Johns Hopkins CSSE. Baltimore, Maryland: Johns Hopkins University.
USA, Connecticut	Johns Hopkins University. 2019 Novel Coronavirus COVID-19 (2019-nCoV) Data Repository by Johns Hopkins CSSE. Baltimore, Maryland: Johns Hopkins University.
USA, Delaware	Delaware Division of Public Health (United States). United States - Delaware Division of Public Health Coronavirus Disease (COVID-19) Data Dashboard. United States of America: Delaware Division of Public Health (United States).
USA, Florida	Johns Hopkins University. 2019 Novel Coronavirus COVID-19 (2019-nCoV) Data Repository by Johns Hopkins CSSE. Baltimore, Maryland: Johns Hopkins University.
USA, Georgia	Johns Hopkins University. 2019 Novel Coronavirus COVID-19 (2019-nCoV) Data Repository by Johns Hopkins CSSE. Baltimore, Maryland: Johns Hopkins University.
USA, Hawaii	Johns Hopkins University. 2019 Novel Coronavirus COVID-19 (2019-nCoV) Data Repository by Johns Hopkins CSSE. Baltimore, Maryland: Johns Hopkins University.
USA, Idaho	Johns Hopkins University. 2019 Novel Coronavirus COVID-19 (2019-nCoV) Data Repository by Johns Hopkins CSSE. Baltimore, Maryland: Johns Hopkins University.
USA, Illinois	Johns Hopkins University. 2019 Novel Coronavirus COVID-19 (2019-nCoV) Data Repository by Johns Hopkins CSSE. Baltimore, Maryland: Johns Hopkins University.
USA, Indiana	Johns Hopkins University. 2019 Novel Coronavirus COVID-19 (2019-nCoV) Data Repository by Johns Hopkins CSSE. Baltimore, Maryland: Johns Hopkins University.
USA, Iowa	Johns Hopkins University. 2019 Novel Coronavirus COVID-19 (2019-nCoV) Data Repository by Johns Hopkins CSSE. Baltimore, Maryland: Johns Hopkins University.
USA, Kansas	Johns Hopkins University. 2019 Novel Coronavirus COVID-19 (2019-nCoV) Data Repository by Johns Hopkins CSSE. Baltimore, Maryland: Johns Hopkins University.
USA, Kentucky	Johns Hopkins University. 2019 Novel Coronavirus COVID-19 (2019-nCoV) Data Repository by Johns Hopkins CSSE. Baltimore, Maryland: Johns Hopkins University.
USA, Louisiana	Johns Hopkins University. 2019 Novel Coronavirus COVID-19 (2019-nCoV) Data Repository by Johns Hopkins CSSE. Baltimore, Maryland: Johns Hopkins University.
USA, Maine	Johns Hopkins University. 2019 Novel Coronavirus COVID-19 (2019-nCoV) Data Repository by Johns Hopkins CSSE. Baltimore, Maryland: Johns Hopkins University.
USA, Maryland	Johns Hopkins University. 2019 Novel Coronavirus COVID-19 (2019-nCoV) Data Repository by Johns Hopkins CSSE. Baltimore, Maryland: Johns Hopkins University.
USA, Massachusetts	Johns Hopkins University. 2019 Novel Coronavirus COVID-19 (2019-nCoV) Data Repository by Johns Hopkins CSSE. Baltimore, Maryland: Johns Hopkins University.
USA, Michigan	Johns Hopkins University. 2019 Novel Coronavirus COVID-19 (2019-nCoV) Data Repository by Johns Hopkins CSSE. Baltimore, Maryland: Johns Hopkins University.
USA, Minnesota	Johns Hopkins University. 2019 Novel Coronavirus COVID-19 (2019-nCoV) Data Repository by Johns Hopkins CSSE. Baltimore, Maryland: Johns Hopkins University.

USA, Mississippi	Johns Hopkins University. 2019 Novel Coronavirus COVID-19 (2019-nCoV) Data Repository by Johns Hopkins CSSE. Baltimore, Maryland: Johns Hopkins University.
USA, Missouri	Johns Hopkins University. 2019 Novel Coronavirus COVID-19 (2019-nCoV) Data Repository by Johns Hopkins CSSE. Baltimore, Maryland: Johns Hopkins University.
USA, Montana	Johns Hopkins University. 2019 Novel Coronavirus COVID-19 (2019-nCoV) Data Repository by Johns Hopkins CSSE. Baltimore, Maryland: Johns Hopkins University.
USA, Nebraska	Johns Hopkins University. 2019 Novel Coronavirus COVID-19 (2019-nCoV) Data Repository by Johns Hopkins CSSE. Baltimore, Maryland: Johns Hopkins University.
USA, Nevada	Johns Hopkins University. 2019 Novel Coronavirus COVID-19 (2019-nCoV) Data Repository by Johns Hopkins CSSE. Baltimore, Maryland: Johns Hopkins University.
USA, New Hampshire	Johns Hopkins University. 2019 Novel Coronavirus COVID-19 (2019-nCoV) Data Repository by Johns Hopkins CSSE. Baltimore, Maryland: Johns Hopkins University.
USA, New Jersey	Johns Hopkins University. 2019 Novel Coronavirus COVID-19 (2019-nCoV) Data Repository by Johns Hopkins CSSE. Baltimore, Maryland: Johns Hopkins University.
USA, New Mexico	Johns Hopkins University. 2019 Novel Coronavirus COVID-19 (2019-nCoV) Data Repository by Johns Hopkins CSSE. Baltimore, Maryland: Johns Hopkins University.
USA, New York	New York Times. COVID-19 Cumulative Deaths and Cases in the United States by County - New York Times. New York, United States of America: New York Times.
USA, New York	Johns Hopkins University. 2019 Novel Coronavirus COVID-19 (2019-nCoV) Data Repository by Johns Hopkins CSSE. Baltimore, Maryland: Johns Hopkins University.
USA, North Carolina	Johns Hopkins University. 2019 Novel Coronavirus COVID-19 (2019-nCoV) Data Repository by Johns Hopkins CSSE. Baltimore, Maryland: Johns Hopkins University.
USA, North Dakota	Johns Hopkins University. 2019 Novel Coronavirus COVID-19 (2019-nCoV) Data Repository by Johns Hopkins CSSE. Baltimore, Maryland: Johns Hopkins University.
USA, Ohio	Johns Hopkins University. 2019 Novel Coronavirus COVID-19 (2019-nCoV) Data Repository by Johns Hopkins CSSE. Baltimore, Maryland: Johns Hopkins University.
USA, Oklahoma	Johns Hopkins University. 2019 Novel Coronavirus COVID-19 (2019-nCoV) Data Repository by Johns Hopkins CSSE. Baltimore, Maryland: Johns Hopkins University.
USA, Oregon	Johns Hopkins University. 2019 Novel Coronavirus COVID-19 (2019-nCoV) Data Repository by Johns Hopkins CSSE. Baltimore, Maryland: Johns Hopkins University.
USA, Pennsylvania	Johns Hopkins University. 2019 Novel Coronavirus COVID-19 (2019-nCoV) Data Repository by Johns Hopkins CSSE. Baltimore, Maryland: Johns Hopkins University.
USA, Rhode Island	Johns Hopkins University. 2019 Novel Coronavirus COVID-19 (2019-nCoV) Data Repository by Johns Hopkins CSSE. Baltimore, Maryland: Johns Hopkins University.
USA, South Carolina	Johns Hopkins University. 2019 Novel Coronavirus COVID-19 (2019-nCoV) Data Repository by Johns Hopkins CSSE. Baltimore, Maryland: Johns Hopkins University.
USA, South Dakota	Johns Hopkins University. 2019 Novel Coronavirus COVID-19 (2019-nCoV) Data Repository by Johns Hopkins CSSE. Baltimore, Maryland: Johns Hopkins University.
USA, Tennessee	Johns Hopkins University. 2019 Novel Coronavirus COVID-19 (2019-nCoV) Data Repository by Johns Hopkins CSSE. Baltimore, Maryland: Johns Hopkins University.
USA, Texas	Johns Hopkins University. 2019 Novel Coronavirus COVID-19 (2019-nCoV) Data Repository by Johns Hopkins CSSE. Baltimore, Maryland: Johns Hopkins University.
USA, Utah	Johns Hopkins University. 2019 Novel Coronavirus COVID-19 (2019-nCoV) Data Repository by Johns Hopkins CSSE. Baltimore, Maryland: Johns Hopkins University.
USA, Vermont	Johns Hopkins University. 2019 Novel Coronavirus COVID-19 (2019-nCoV) Data Repository by Johns Hopkins CSSE. Baltimore, Maryland: Johns Hopkins University.
USA, Virginia	Johns Hopkins University. 2019 Novel Coronavirus COVID-19 (2019-nCoV) Data Repository by Johns Hopkins CSSE. Baltimore, Maryland: Johns Hopkins University.
USA, Washington	New York Times. COVID-19 Cumulative Deaths and Cases in the United States by County - New York Times. New York, United States of America: New York Times.
USA, Washington, DC	Johns Hopkins University. 2019 Novel Coronavirus COVID-19 (2019-nCoV) Data Repository by Johns Hopkins CSSE. Baltimore, Maryland: Johns Hopkins University.
USA, West Virginia	Johns Hopkins University. 2019 Novel Coronavirus COVID-19 (2019-nCoV) Data Repository by Johns Hopkins CSSE. Baltimore, Maryland: Johns Hopkins University.
USA, Wisconsin	Johns Hopkins University. 2019 Novel Coronavirus COVID-19 (2019-nCoV) Data Repository by Johns Hopkins CSSE. Baltimore, Maryland: Johns Hopkins University.
USA, Wyoming	Johns Hopkins University. 2019 Novel Coronavirus COVID-19 (2019-nCoV) Data Repository by Johns Hopkins CSSE. Baltimore, Maryland: Johns Hopkins University.
Uzbekistan	Johns Hopkins University. 2019 Novel Coronavirus COVID-19 (2019-nCoV) Data Repository by Johns Hopkins CSSE. Baltimore, Maryland: Johns Hopkins University.
Vanuatu	Johns Hopkins University. 2019 Novel Coronavirus COVID-19 (2019-nCoV) Data Repository by Johns Hopkins CSSE. Baltimore, Maryland: Johns Hopkins University.
Venezuela	Johns Hopkins University. 2019 Novel Coronavirus COVID-19 (2019-nCoV) Data Repository by Johns Hopkins CSSE. Baltimore, Maryland: Johns Hopkins University.
Vietnam	Johns Hopkins University. 2019 Novel Coronavirus COVID-19 (2019-nCoV) Data Repository by Johns Hopkins CSSE. Baltimore, Maryland: Johns Hopkins University.

Virgin Islands	Johns Hopkins University. 2019 Novel Coronavirus COVID-19 (2019-nCoV) Data Repository by Johns Hopkins CSSE. Baltimore, Maryland: Johns Hopkins University.
Yemen	Johns Hopkins University. 2019 Novel Coronavirus COVID-19 (2019-nCoV) Data Repository by Johns Hopkins CSSE. Baltimore, Maryland: Johns Hopkins University.
Zambia	Johns Hopkins University. 2019 Novel Coronavirus COVID-19 (2019-nCoV) Data Repository by Johns Hopkins CSSE. Baltimore, Maryland: Johns Hopkins University.
Zimbabwe	Johns Hopkins University. 2019 Novel Coronavirus COVID-19 (2019-nCoV) Data Repository by Johns Hopkins CSSE. Baltimore, Maryland: Johns Hopkins University.

Section 5: Testing sources

Figure S5. Testing data coverage by location

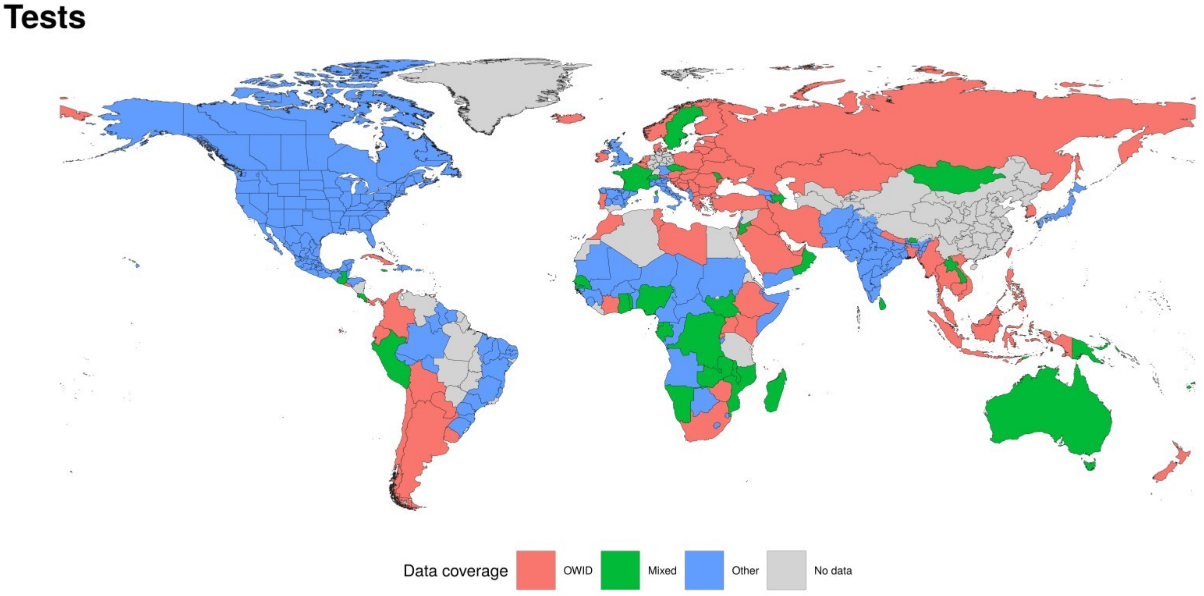

Table S5. Testing data sources by location

Location	Source
Afghanistan	Ministry of Public Health (Afghanistan). Afghanistan COVID-19 Epidemic Monitoring Dashboard. Kabul, Afghanistan: Ministry of Public Health (Afghanistan).
Albania	Ministry of Health and Social Protection (Albania). Albania Ministry of Health and Social Protection Updated Information on COVID-19.
Andorra	European Centre for Disease Prevention and Control, Global Burden of Disease Collaborative Network, Our World in Data, United Nations (UN), World Bank. Our World in Data Complete COVID-19 Dataset.
Angola	Government of Angola. Angola Interministerial Commission: State of COVID-19. Luanda, Angola: Government of Angola.
Angola	European Centre for Disease Prevention and Control, Global Burden of Disease Collaborative Network, Our World in Data, United Nations (UN), World Bank. Our World in Data Complete COVID-19 Dataset.
Antigua and Barbuda	Government of Antigua and Barbuda, Ministry of Health, Wellness & the Environment (Antigua and Barbuda). Antigua and Barbuda - COVID-19 Dashboard. Antigua and Barbuda: Ministry of Health, Wellness & the Environment (Antigua and Barbuda).
Argentina	European Centre for Disease Prevention and Control, Global Burden of Disease Collaborative Network, Our World in Data, United Nations (UN), World Bank. Our World in Data Complete COVID-19 Dataset.
Armenia	European Centre for Disease Prevention and Control, Global Burden of Disease Collaborative Network, Our World in Data, United Nations (UN), World Bank. Our World in Data Complete COVID-19 Dataset.
Australia	European Centre for Disease Prevention and Control, Global Burden of Disease Collaborative Network, Our World in Data, United Nations (UN), World Bank. Our World in Data Complete COVID-19 Dataset.
Australia	Coronavirus (COVID-19) in Australia. Coronavirus (COVID-19) in Australia - Fairfacts Data. Australia: Coronavirus (COVID-19) in Australia.
Austria	European Centre for Disease Prevention and Control, Global Burden of Disease Collaborative Network, Our World in Data, United Nations (UN), World Bank. Our World in Data Complete COVID-19 Dataset.
Azerbaijan	Cabinet of Ministers (Azerbaijan), Koronavirusinfo (Azerbaijan). Azerbaijan Operational Headquarters under the Cabinet of Ministers Latest Situation with COVID-19. Azerbaijan: Koronavirusinfo (Azerbaijan).
Azerbaijan	European Centre for Disease Prevention and Control, Global Burden of Disease Collaborative Network, Our World in Data, United Nations (UN), World Bank. Our World in Data Complete COVID-19 Dataset.
Bahrain	European Centre for Disease Prevention and Control, Global Burden of Disease Collaborative Network, Our World in Data, United Nations (UN), World Bank. Our World in Data Complete COVID-19 Dataset.
Bangladesh	European Centre for Disease Prevention and Control, Global Burden of Disease Collaborative Network, Our World in Data, United Nations (UN), World Bank. Our World in Data Complete COVID-19 Dataset.
Barbados	Government of Barbados. Barbados Government Information Service COVID-19 Update. Bridgetown, Barbados: Government of Barbados.

Belarus	European Centre for Disease Prevention and Control, Global Burden of Disease Collaborative Network, Our World in Data, United Nations (UN), World Bank. Our World in Data Complete COVID-19 Dataset.
Belgium	European Centre for Disease Prevention and Control, Global Burden of Disease Collaborative Network, Our World in Data, United Nations (UN), World Bank. Our World in Data Complete COVID-19 Dataset.
Belize	Ministry of Health (Belize). Belize Office of the Director of Health Services COVID-19 Update. Belmopan, Belize: Ministry of Health (Belize).
Benin	Government of Benin. Benin Update on the Coronavirus Situation.
Benin	Government of Benin. Benin Coronavirus Information (COVID-19).
Bermuda	Government of Bermuda. Bermuda COVID-19 Update . Hamilton, Bermuda: Government of Bermuda.
Bhutan	European Centre for Disease Prevention and Control, Global Burden of Disease Collaborative Network, Our World in Data, United Nations (UN), World Bank. Our World in Data Complete COVID-19 Dataset.
Bhutan	Ministry of Health (Bhutan). Bhutan National Situational Update on COVID-19. Thimpu, Bhutan: Ministry of Health (Bhutan).
Bolivia	European Centre for Disease Prevention and Control, Global Burden of Disease Collaborative Network, Our World in Data, United Nations (UN), World Bank. Our World in Data Complete COVID-19 Dataset.
Bosnia and Herzegovina	European Centre for Disease Prevention and Control, Global Burden of Disease Collaborative Network, Our World in Data, United Nations (UN), World Bank. Our World in Data Complete COVID-19 Dataset.
Botswana	European Centre for Disease Prevention and Control, Global Burden of Disease Collaborative Network, Our World in Data, United Nations (UN), World Bank. Our World in Data Complete COVID-19 Dataset.
Botswana	Government of Botswana. Botswana COVID-19 Data Dashboard. Gaborone, Botswana: Government of Botswana.
Brazil	European Centre for Disease Prevention and Control, Global Burden of Disease Collaborative Network, Our World in Data, United Nations (UN), World Bank. Our World in Data Complete COVID-19 Dataset.
Brazil, Acre	Government of Acre (Brazil). Brazil - Acre COVID-19 Transparency Portal. Brazil: Government of Acre (Brazil).
Brazil, Amazonas	Amazonas Health Surveillance Foundation (Brazil). Brazil - Amazonas COVID-19 Daily Bulletin. Brazil: Amazonas Health Surveillance Foundation (Brazil).
Brazil, Bahia	Government of the State of Bahia (Brazil). Brazil - Bahia COVID-19 Epidemiological Bulletin. Government of the State of Bahia (Brazil).
Brazil, Ceará	Ceará School of Public Health (Brazil), Secretary of Health of the State of Ceara (Brazil). Brazil - Ceará COVID-19 Epidemiological Bulletins. Brazil: Secretary of Health of the State of Ceara (Brazil).
Brazil, Espírito Santo	Government of Espírito Santo (Brazil). Brazil - Espírito Santo COVID-19 Panel.
Brazil, Goiás	Goiás State Secretariat of Health. Brazil - Goiás State Secretariat of Health Covid Tests. Goiás, Brazil: Goiás State Secretariat of Health.
Brazil, Maranhão	Secretary of State for Health (Brazil - Maranhão). Brazil - Maranhão COVID-19 Testing Data.
Brazil, Minas Gerais	Minas Gerais Ministry of Health (Brazil). Brazil - Minas Gerais Coronavirus Epidemiological Bulletin.
Brazil, Paraíba	Government of the State of Paraíba. Brazil - Paraíba COVID-19 Epidemiological Data.
Brazil, Paraná	Government of Paraná (Brazil), Secretary of Health (Parana). Brazil - Paraná COVID-19 Epidemiological Data. Curitiba, Brazil: Secretary of Health (Parana), 2020.
Brazil, Pernambuco	State Department of Health Pernambuco (Brazil). COVID-19 in the World, in Brazil and in Pernambuco.
Brazil, Piauí	Piaui State Government. Brazil - Piaui COVID-19 Epidemiological Dashboard. Teresina, Brazil: Piaui State Government.
Brazil, Rio Grande do Norte	Rio Grande do Norte State Health Department (Brazil). Brazil - Rio Grande do Norte COVID-19 Epidemiological Report.
Brazil, Rio Grande do Sul	Rio Grande do Sul State Health Department (Brazil). Brazil - Rio Grande do Sul COVID-19 Testing Results. Porto Alegre, Brazil: Rio Grande do Sul State Health Department (Brazil).
Brazil, Rondônia	Government of the State of Rondonia. Brazil - Rondonia Daily Newsletter on Coronavirus.
Brazil, Roraima	State Secretariat of Health of Roraima (Brazil). Brazil - Roraima COVID-19 Epidemiological Bulletin.
Brazil, Santa Catarina	Government of Santa Catarina (Brazil). Brazil - Santa Catarina Coronavirus Epidemiological Bulletin.
Brazil, São Paulo	Sao Paulo State Government (Brazil). Brazil - Sao Paulo COVID-19 Total Tests Performed. Brazil: Sao Paulo State Government (Brazil).
Brazil, Sergipe	Sergipe State Government. Brazil - Sergipe Epidemiological Bulletin for Update on Coronavirus Disease 2019 (Covid-19).
Brazil, Tocantins	Ministry of Health of Tocantins (Brazil). Brazil - Tocantins COVID-19 Epidemiological Bulletin 2020. Palmas, Brazil: Ministry of Health of Tocantins (Brazil), 2020.
Brazil, Tocantins	Ministry of Health of Tocantins (Brazil). Brazil - Tocantins COVID-19 Epidemiological Bulletin 2021. Palmas, Brazil: Ministry of Health of Tocantins (Brazil), 2020.
Brunei	Ministry of Health (Brunei Darussalam), The Bruneian. The Bruneian COVID-19 Updates. Menlo Park, United States of America: Facebook.
Brunei	Ministry of Health (Brunei Darussalam). Brunei Ministry of Health Press Release on the Current Situation of the COVID-19 Infection. Bandar Seri Begawa, Brunei Darussalam: Ministry of Health (Brunei Darussalam).

Bulgaria	European Centre for Disease Prevention and Control, Global Burden of Disease Collaborative Network, Our World in Data, United Nations (UN), World Bank. Our World in Data Complete COVID-19 Dataset.
Burkina Faso	Ministry of Health (Burkina Faso). Burkina Faso Ministry of Health COVID-19 Dashboard.
Burkina Faso	European Centre for Disease Prevention and Control, Global Burden of Disease Collaborative Network, Our World in Data, United Nations (UN), World Bank. Our World in Data Complete COVID-19 Dataset.
Burundi	Ministry of Public Health (Burundi). Burundi Ministry of Public Health Twitter Communications and COVID-19 Updates. Bujumbura, Burundi: Ministry of Public Health (Burundi).
Burundi	European Centre for Disease Prevention and Control, Global Burden of Disease Collaborative Network, Our World in Data, United Nations (UN), World Bank. Our World in Data Complete COVID-19 Dataset.
Cambodia	European Centre for Disease Prevention and Control, Global Burden of Disease Collaborative Network, Our World in Data, United Nations (UN), World Bank. Our World in Data Complete COVID-19 Dataset.
Cameroon	Ministry of Public Health (Cameroon). Cameroon COVID-19 Press Briefing. Yaoundé, Cameroon: Ministry of Public Health (Cameroon).
Canada	Government of Canada. Canada Public Health Infobase Number of Total Cases of COVID-19. Ottawa, Canada: Government of Canada.
Canada	Government of Canada. Canada Coronavirus Disease 2019 (COVID-19) Daily Epidemiology Update. Ottawa, Canada: Government of Canada.
Canada, Alberta	Government of Canada. Canada Public Health Infobase Number of Total Cases of COVID-19. Ottawa, Canada: Government of Canada.
Canada, Alberta	Government of Canada. Canada Coronavirus Disease 2019 (COVID-19) Daily Epidemiology Update. Ottawa, Canada: Government of Canada.
Canada, British Columbia	Government of Canada. Canada Public Health Infobase Number of Total Cases of COVID-19. Ottawa, Canada: Government of Canada.
Canada, British Columbia	Government of Canada. Canada Coronavirus Disease 2019 (COVID-19) Daily Epidemiology Update. Ottawa, Canada: Government of Canada.
Canada, Manitoba	Government of Canada. Canada Public Health Infobase Number of Total Cases of COVID-19. Ottawa, Canada: Government of Canada.
Canada, Manitoba	Government of Canada. Canada Coronavirus Disease 2019 (COVID-19) Daily Epidemiology Update. Ottawa, Canada: Government of Canada.
Canada, New Brunswick	Government of Canada. Canada Public Health Infobase Number of Total Cases of COVID-19. Ottawa, Canada: Government of Canada.
Canada, New Brunswick	Government of Canada. Canada Coronavirus Disease 2019 (COVID-19) Daily Epidemiology Update. Ottawa, Canada: Government of Canada.
Canada, Newfoundland and Labrador	Government of Canada. Canada Public Health Infobase Number of Total Cases of COVID-19. Ottawa, Canada: Government of Canada.
Canada, Newfoundland and Labrador	Government of Canada. Canada Coronavirus Disease 2019 (COVID-19) Daily Epidemiology Update. Ottawa, Canada: Government of Canada.
Canada, Northwest Territories	Government of Canada. Canada Public Health Infobase Number of Total Cases of COVID-19. Ottawa, Canada: Government of Canada.
Canada, Northwest Territories	Government of Canada. Canada Coronavirus Disease 2019 (COVID-19) Daily Epidemiology Update. Ottawa, Canada: Government of Canada.
Canada, Nova Scotia	Government of Canada. Canada Public Health Infobase Number of Total Cases of COVID-19. Ottawa, Canada: Government of Canada.
Canada, Nova Scotia	Government of Canada. Canada Coronavirus Disease 2019 (COVID-19) Daily Epidemiology Update. Ottawa, Canada: Government of Canada.
Canada, Nunavut	Government of Canada. Canada Public Health Infobase Number of Total Cases of COVID-19. Ottawa, Canada: Government of Canada.
Canada, Nunavut	Government of Canada. Canada Coronavirus Disease 2019 (COVID-19) Daily Epidemiology Update. Ottawa, Canada: Government of Canada.
Canada, Ontario	Government of Canada. Canada Public Health Infobase Number of Total Cases of COVID-19. Ottawa, Canada: Government of Canada.
Canada, Ontario	Government of Canada. Canada Coronavirus Disease 2019 (COVID-19) Daily Epidemiology Update. Ottawa, Canada: Government of Canada.
Canada, Prince Edward Island	Government of Canada. Canada Public Health Infobase Number of Total Cases of COVID-19. Ottawa, Canada: Government of Canada.
Canada, Prince Edward Island	Government of Canada. Canada Coronavirus Disease 2019 (COVID-19) Daily Epidemiology Update. Ottawa, Canada: Government of Canada.
Canada, Quebec	Government of Canada. Canada Public Health Infobase Number of Total Cases of COVID-19. Ottawa, Canada: Government of Canada.
Canada, Quebec	Government of Canada. Canada Coronavirus Disease 2019 (COVID-19) Daily Epidemiology Update. Ottawa, Canada: Government of Canada.
Canada, Saskatchewan	Government of Canada. Canada Public Health Infobase Number of Total Cases of COVID-19. Ottawa, Canada: Government of Canada.
Canada, Saskatchewan	Government of Canada. Canada Coronavirus Disease 2019 (COVID-19) Daily Epidemiology Update. Ottawa, Canada: Government of Canada.
Canada, Yukon	Government of Canada. Canada Public Health Infobase Number of Total Cases of COVID-19. Ottawa, Canada: Government of Canada.

Canada, Yukon	Government of Canada. Canada Coronavirus Disease 2019 (COVID-19) Daily Epidemiology Update. Ottawa, Canada: Government of Canada.
Cape Verde	Ministry of Health and Social Security (Cape Verde). Cape Verde COVID-19 Current Situation. Cabo Verde: Ministry of Health and Social Security (Cape Verde).
Cape Verde	Ministry of Health and Social Security (Cape Verde). Cape Verde COVID-19 Epidemiological Bulletin. Praia, Cabo Verde: Government of Cape Verde, 2020.
Cape Verde	European Centre for Disease Prevention and Control, Global Burden of Disease Collaborative Network, Our World in Data, United Nations (UN), World Bank. Our World in Data Complete COVID-19 Dataset.
Central African Republic	Ministry of Health and Population (MSP) (Central African Republic). Central African Republic COVID-19 Daily Situation Report. Central African Republic: Ministry of Health and Population (MSP) (Central African Republic).
Central African Republic	European Centre for Disease Prevention and Control, Global Burden of Disease Collaborative Network, Our World in Data, United Nations (UN), World Bank. Our World in Data Complete COVID-19 Dataset.
Chad	Ministry of Public Health (Chad). Chad Ministry of Public Health COVID-19 Press Releases. N'Djamena, Chad: Ministry of Public Health (Chad).
Chad	European Centre for Disease Prevention and Control, Global Burden of Disease Collaborative Network, Our World in Data, United Nations (UN), World Bank. Our World in Data Complete COVID-19 Dataset.
Chile	European Centre for Disease Prevention and Control, Global Burden of Disease Collaborative Network, Our World in Data, United Nations (UN), World Bank. Our World in Data Complete COVID-19 Dataset.
China	European Centre for Disease Prevention and Control, Global Burden of Disease Collaborative Network, Our World in Data, United Nations (UN), World Bank. Our World in Data Complete COVID-19 Dataset.
China, Hong Kong Special Administrative Region of China	European Centre for Disease Prevention and Control, Global Burden of Disease Collaborative Network, Our World in Data, United Nations (UN), World Bank. Our World in Data Complete COVID-19 Dataset.
Colombia	European Centre for Disease Prevention and Control, Global Burden of Disease Collaborative Network, Our World in Data, United Nations (UN), World Bank. Our World in Data Complete COVID-19 Dataset.
Comoros	Ministry of Health, Social Cohesion, Solidarity, and Gender Promotion (Comoros), Stop Coronavirus (Comoros). Comoros Ministry of Health, Solidarity, Social Protection and Gender Promotion COVID-19 Press Releases. Comoros: Stop Coronavirus (Comoros).
Congo (Brazzaville)	Ministry of Health, Population, Promotion (Congo). Congo COVID-19 Epidemiological Situation. Congo: Ministry of Health and Population (Congo).
Costa Rica	Distance State University (UNED) (Costa Rica), Ministry of Health (Costa Rica). Costa Rica COVID-19 National Situation - Distance State University. San Jose, Costa Rica: Distance State University (UNED) (Costa Rica).
Côte d'Ivoire	European Centre for Disease Prevention and Control, Global Burden of Disease Collaborative Network, Our World in Data, United Nations (UN), World Bank. Our World in Data Complete COVID-19 Dataset.
Croatia	European Centre for Disease Prevention and Control, Global Burden of Disease Collaborative Network, Our World in Data, United Nations (UN), World Bank. Our World in Data Complete COVID-19 Dataset.
Cuba	European Centre for Disease Prevention and Control, Global Burden of Disease Collaborative Network, Our World in Data, United Nations (UN), World Bank. Our World in Data Complete COVID-19 Dataset.
Cyprus	European Centre for Disease Prevention and Control, Global Burden of Disease Collaborative Network, Our World in Data, United Nations (UN), World Bank. Our World in Data Complete COVID-19 Dataset.
Czechia	Ministry of Health (Czech Republic). Czechia COVID-19 Open Data Sets. Prague, Czechia: Ministry of Health (Czech Republic).
Czechia	European Centre for Disease Prevention and Control, Global Burden of Disease Collaborative Network, Our World in Data, United Nations (UN), World Bank. Our World in Data Complete COVID-19 Dataset.
Denmark	European Centre for Disease Prevention and Control, Global Burden of Disease Collaborative Network, Our World in Data, United Nations (UN), World Bank. Our World in Data Complete COVID-19 Dataset.
Djibouti	Ministry of Health (Djibouti). Djibouti COVID-19 Statistics. Djibouti: Government of Djibouti.
Dominica	Dominica Ministry of Health, Wellness and New Health Investment Coronavirus (COVID-19) Report.
Dominican Republic	General Directorate of Epidemiology, Ministry of Public Health and Social Assistance (Dominican Republic). Dominican Republic General Directorate of Epidemiology Coronavirus Disease 2019 (COVID-19) Special Bulletin.
DR Congo	Democratic Republic of the Congo Multisectoral Committee on the Response to COVID-19. Democratic Republic of the Congo Multisectoral Committee on the Response to COVID-19 Bulletin.
DR Congo	European Centre for Disease Prevention and Control, Global Burden of Disease Collaborative Network, Our World in Data, United Nations (UN), World Bank. Our World in Data Complete COVID-19 Dataset.
Ecuador	European Centre for Disease Prevention and Control, Global Burden of Disease Collaborative Network, Our World in Data, United Nations (UN), World Bank. Our World in Data Complete COVID-19 Dataset.
El Salvador	European Centre for Disease Prevention and Control, Global Burden of Disease Collaborative Network, Our World in Data, United Nations (UN), World Bank. Our World in Data Complete COVID-19 Dataset.
Equatorial Guinea	European Centre for Disease Prevention and Control, Global Burden of Disease Collaborative Network, Our World in Data, United Nations (UN), World Bank. Our World in Data Complete COVID-19 Dataset.
Estonia	European Centre for Disease Prevention and Control, Global Burden of Disease Collaborative Network, Our World in Data, United Nations (UN), World Bank. Our World in Data Complete COVID-19 Dataset.
Eswatini	Botswana International University Of Science And Technology, DataConvergence, Ministry of Health (Eswatini), National Research Foundation (South Africa), University of Eswatini, University of Witwatersrand. Eswatini COVID-19 Dashboard. Eswatini: Ministry of Health (Eswatini).

Eswatini	Government of Eswatini, Ministry of Health (Eswatini). Eswatini COVID-19 Daily Situation Report. Eswatini: Government of Eswatini.
Ethiopia	European Centre for Disease Prevention and Control, Global Burden of Disease Collaborative Network, Our World in Data, United Nations (UN), World Bank. Our World in Data Complete COVID-19 Dataset.
Fiji	European Centre for Disease Prevention and Control, Global Burden of Disease Collaborative Network, Our World in Data, United Nations (UN), World Bank. Our World in Data Complete COVID-19 Dataset.
Finland	European Centre for Disease Prevention and Control, Global Burden of Disease Collaborative Network, Our World in Data, United Nations (UN), World Bank. Our World in Data Complete COVID-19 Dataset.
France	European Centre for Disease Prevention and Control, Global Burden of Disease Collaborative Network, Our World in Data, United Nations (UN), World Bank. Our World in Data Complete COVID-19 Dataset.
Gabon	Government of Gabon. Gabon COVID-19 Epidemiological Situation. Gabon: Steering Committee for the Monitoring and Response Plan Against the Coronavirus Epidemic (Gabon).
Georgia	*
Germany	European Centre for Disease Prevention and Control, Global Burden of Disease Collaborative Network, Our World in Data, United Nations (UN), World Bank. Our World in Data Complete COVID-19 Dataset.
Germany, Bavaria	Bavarian State Office for Health and Food Safety (Germany). Overview of the Number of Cases of Coronavirus Infections in Bavaria.
Germany, Bremen	Bremen Senate Press Office (Germany). Germany - Bremen COVID-19 Press Releases.
Ghana	Ghana Health Service. Ghana Health Service Coronavirus Disease (COVID-19) Updates.
Ghana	European Centre for Disease Prevention and Control, Global Burden of Disease Collaborative Network, Our World in Data, United Nations (UN), World Bank. Our World in Data Complete COVID-19 Dataset.
Greece	European Centre for Disease Prevention and Control, Global Burden of Disease Collaborative Network, Our World in Data, United Nations (UN), World Bank. Our World in Data Complete COVID-19 Dataset.
Grenada	Ministry of Health (Grenada). Grenada Ministry of Health COVID-19 Report 2020. Ministry of Health (Grenada).
Guatemala	Guatemala Ministry of Health and Social Assistance. Guatemala Daily Report Of COVID-19 Cases. Menlo Park, United States of America: Facebook.
Guatemala	European Centre for Disease Prevention and Control, Global Burden of Disease Collaborative Network, Our World in Data, United Nations (UN), World Bank. Our World in Data Complete COVID-19 Dataset.
Guinea	National Health Security Agency (ANSS) (Guinea). Guinea Ministry of Health COVID-19 Epidemiological Situation. Guinea: National Health Security Agency (ANSS) (Guinea).
Guinea-Bissau	INFOCOVID-19 (Guinea-Bissau). Guinea-Bissau INFO COVID-19 Epidemiological Situation. Guinea-Bissau: INFOCOVID-19 (Guinea-Bissau).
Guyana	Ministry of Health (Guyana). Guyana Ministry of Public Health COVID-19 Dashboard.
Guyana	European Centre for Disease Prevention and Control, Global Burden of Disease Collaborative Network, Our World in Data, United Nations (UN), World Bank. Our World in Data Complete COVID-19 Dataset.
Haiti	Ministry of Public Health and Population (Haiti). Haiti COVID-19 Surveillance. Port-au-Prince, Haiti: Ministry of Public Health and Population (Haiti).
Haiti	European Centre for Disease Prevention and Control, Global Burden of Disease Collaborative Network, Our World in Data, United Nations (UN), World Bank. Our World in Data Complete COVID-19 Dataset.
Honduras	National Risk Management System (SINAGER) (Honduras). Honduras National Risk Management System COVID-19 Statement. Honduras: Government of Honduras.
Hungary	European Centre for Disease Prevention and Control, Global Burden of Disease Collaborative Network, Our World in Data, United Nations (UN), World Bank. Our World in Data Complete COVID-19 Dataset.
Iceland	European Centre for Disease Prevention and Control, Global Burden of Disease Collaborative Network, Our World in Data, United Nations (UN), World Bank. Our World in Data Complete COVID-19 Dataset.
India	European Centre for Disease Prevention and Control, Global Burden of Disease Collaborative Network, Our World in Data, United Nations (UN), World Bank. Our World in Data Complete COVID-19 Dataset.
India, Andhra Pradesh	COVID-19 India. India COVID-19 Crowdsourced Patient Database: State Level Testing Data. India: COVID-19 India.
India, Arunachal Pradesh	COVID-19 India. India COVID-19 Crowdsourced Patient Database: State Level Testing Data. India: COVID-19 India.
India, Assam	COVID-19 India. India COVID-19 Crowdsourced Patient Database: State Level Testing Data. India: COVID-19 India.
India, Bihar	COVID-19 India. India COVID-19 Crowdsourced Patient Database: State Level Testing Data. India: COVID-19 India.
India, Chhattisgarh	COVID-19 India. India COVID-19 Crowdsourced Patient Database: State Level Testing Data. India: COVID-19 India.
India, Dadra and Nagar Haveli and Daman and Diu	COVID-19 India. India COVID-19 Crowdsourced Patient Database: State Level Testing Data. India: COVID-19 India.
India, Delhi	COVID-19 India. India COVID-19 Crowdsourced Patient Database: State Level Testing Data. India: COVID-19 India.
India, Goa	COVID-19 India. India COVID-19 Crowdsourced Patient Database: State Level Testing Data. India: COVID-19 India.
India, Gujarat	COVID-19 India. India COVID-19 Crowdsourced Patient Database: State Level Testing Data. India: COVID-19 India.
India, Haryana	COVID-19 India. India COVID-19 Crowdsourced Patient Database: State Level Testing Data. India: COVID-19 India.

India, Himachal Pradesh	COVID-19 India. India COVID-19 Crowdsourced Patient Database: State Level Testing Data. India: COVID-19 India.
India, Jammu & Kashmir and Ladakh	COVID-19 India. India COVID-19 Crowdsourced Patient Database: State Level Testing Data. India: COVID-19 India.
India, Jharkhand	COVID-19 India. India COVID-19 Crowdsourced Patient Database: State Level Testing Data. India: COVID-19 India.
India, Karnataka	COVID-19 India. India COVID-19 Crowdsourced Patient Database: State Level Testing Data. India: COVID-19 India.
India, Kerala	COVID-19 India. India COVID-19 Crowdsourced Patient Database: State Level Testing Data. India: COVID-19 India.
India, Madhya Pradesh	COVID-19 India. India COVID-19 Crowdsourced Patient Database: State Level Testing Data. India: COVID-19 India.
India, Maharashtra	COVID-19 India. India COVID-19 Crowdsourced Patient Database: State Level Testing Data. India: COVID-19 India.
India, Manipur	COVID-19 India. India COVID-19 Crowdsourced Patient Database: State Level Testing Data. India: COVID-19 India.
India, Meghalaya	COVID-19 India. India COVID-19 Crowdsourced Patient Database: State Level Testing Data. India: COVID-19 India.
India, Mizoram	COVID-19 India. India COVID-19 Crowdsourced Patient Database: State Level Testing Data. India: COVID-19 India.
India, Nagaland	COVID-19 India. India COVID-19 Crowdsourced Patient Database: State Level Testing Data. India: COVID-19 India.
India, Odisha	COVID-19 India. India COVID-19 Crowdsourced Patient Database: State Level Testing Data. India: COVID-19 India.
India, Punjab	COVID-19 India. India COVID-19 Crowdsourced Patient Database: State Level Testing Data. India: COVID-19 India.
India, Rajasthan	COVID-19 India. India COVID-19 Crowdsourced Patient Database: State Level Testing Data. India: COVID-19 India.
India, Sikkim	COVID-19 India. India COVID-19 Crowdsourced Patient Database: State Level Testing Data. India: COVID-19 India.
India, Tamil Nadu	COVID-19 India. India COVID-19 Crowdsourced Patient Database: State Level Testing Data. India: COVID-19 India.
India, Telangana	COVID-19 India. India COVID-19 Crowdsourced Patient Database: State Level Testing Data. India: COVID-19 India.
India, Tripura	COVID-19 India. India COVID-19 Crowdsourced Patient Database: State Level Testing Data. India: COVID-19 India.
India, Uttar Pradesh	COVID-19 India. India COVID-19 Crowdsourced Patient Database: State Level Testing Data. India: COVID-19 India.
India, Uttarakhand	COVID-19 India. India COVID-19 Crowdsourced Patient Database: State Level Testing Data. India: COVID-19 India.
India, West Bengal	COVID-19 India. India COVID-19 Crowdsourced Patient Database: State Level Testing Data. India: COVID-19 India.
Indonesia	European Centre for Disease Prevention and Control, Global Burden of Disease Collaborative Network, Our World in Data, United Nations (UN), World Bank. Our World in Data Complete COVID-19 Dataset.
Iran	European Centre for Disease Prevention and Control, Global Burden of Disease Collaborative Network, Our World in Data, United Nations (UN), World Bank. Our World in Data Complete COVID-19 Dataset.
Iraq	European Centre for Disease Prevention and Control, Global Burden of Disease Collaborative Network, Our World in Data, United Nations (UN), World Bank. Our World in Data Complete COVID-19 Dataset.
Ireland	European Centre for Disease Prevention and Control, Global Burden of Disease Collaborative Network, Our World in Data, United Nations (UN), World Bank. Our World in Data Complete COVID-19 Dataset.
Israel	European Centre for Disease Prevention and Control, Global Burden of Disease Collaborative Network, Our World in Data, United Nations (UN), World Bank. Our World in Data Complete COVID-19 Dataset.
Italy	European Centre for Disease Prevention and Control, Global Burden of Disease Collaborative Network, Our World in Data, United Nations (UN), World Bank. Our World in Data Complete COVID-19 Dataset.
Italy, Abruzzo	Department of Civil Protection (Italy). Italy COVID-19 Situation Monitoring - Department of Civil Protection. Rome, Italy: Department of Civil Protection (Italy).
Italy, Basilicata	Department of Civil Protection (Italy). Italy COVID-19 Situation Monitoring - Department of Civil Protection. Rome, Italy: Department of Civil Protection (Italy).
Italy, Calabria	Department of Civil Protection (Italy). Italy COVID-19 Situation Monitoring - Department of Civil Protection. Rome, Italy: Department of Civil Protection (Italy).
Italy, Campania	Department of Civil Protection (Italy). Italy COVID-19 Situation Monitoring - Department of Civil Protection. Rome, Italy: Department of Civil Protection (Italy).
Italy, Emilia-Romagna	Department of Civil Protection (Italy). Italy COVID-19 Situation Monitoring - Department of Civil Protection. Rome, Italy: Department of Civil Protection (Italy).
Italy, Friuli-Venezia Giulia	Department of Civil Protection (Italy). Italy COVID-19 Situation Monitoring - Department of Civil Protection. Rome, Italy: Department of Civil Protection (Italy).
Italy, Lazio	Department of Civil Protection (Italy). Italy COVID-19 Situation Monitoring - Department of Civil Protection. Rome, Italy: Department of Civil Protection (Italy).
Italy, Liguria	Department of Civil Protection (Italy). Italy COVID-19 Situation Monitoring - Department of Civil Protection. Rome, Italy: Department of Civil Protection (Italy).
Italy, Lombardia	Department of Civil Protection (Italy). Italy COVID-19 Situation Monitoring - Department of Civil Protection. Rome, Italy: Department of Civil Protection (Italy).
Italy, Marche	Department of Civil Protection (Italy). Italy COVID-19 Situation Monitoring - Department of Civil Protection. Rome, Italy: Department of Civil Protection (Italy).

Italy, Molise	Department of Civil Protection (Italy). Italy COVID-19 Situation Monitoring - Department of Civil Protection. Rome, Italy: Department of Civil Protection (Italy).
Italy, Piemonte	Department of Civil Protection (Italy). Italy COVID-19 Situation Monitoring - Department of Civil Protection. Rome, Italy: Department of Civil Protection (Italy).
Italy, Prov. autonoma di Bolzano	Department of Civil Protection (Italy). Italy COVID-19 Situation Monitoring - Department of Civil Protection. Rome, Italy: Department of Civil Protection (Italy).
Italy, Prov. autonoma di Trento	Department of Civil Protection (Italy). Italy COVID-19 Situation Monitoring - Department of Civil Protection. Rome, Italy: Department of Civil Protection (Italy).
Italy, Puglia	Department of Civil Protection (Italy). Italy COVID-19 Situation Monitoring - Department of Civil Protection. Rome, Italy: Department of Civil Protection (Italy).
Italy, Sardegna	Department of Civil Protection (Italy). Italy COVID-19 Situation Monitoring - Department of Civil Protection. Rome, Italy: Department of Civil Protection (Italy).
Italy, Sicilia	Department of Civil Protection (Italy). Italy COVID-19 Situation Monitoring - Department of Civil Protection. Rome, Italy: Department of Civil Protection (Italy).
Italy, Toscana	Department of Civil Protection (Italy). Italy COVID-19 Situation Monitoring - Department of Civil Protection. Rome, Italy: Department of Civil Protection (Italy).
Italy, Umbria	Department of Civil Protection (Italy). Italy COVID-19 Situation Monitoring - Department of Civil Protection. Rome, Italy: Department of Civil Protection (Italy).
Italy, Valle d'Aosta	Department of Civil Protection (Italy). Italy COVID-19 Situation Monitoring - Department of Civil Protection. Rome, Italy: Department of Civil Protection (Italy).
Italy, Veneto	Department of Civil Protection (Italy). Italy COVID-19 Situation Monitoring - Department of Civil Protection. Rome, Italy: Department of Civil Protection (Italy).
Jamaica	Ministry of Health and Wellness (Jamaica). Jamaica Ministry of Health and Wellness Coronavirus Updates. Jamaica: Ministry of Health and Wellness (Jamaica).
Jamaica	European Centre for Disease Prevention and Control, Global Burden of Disease Collaborative Network, Our World in Data, United Nations (UN), World Bank. Our World in Data Complete COVID-19 Dataset.
Japan	Ministry of Health, Labour and Welfare (Japan). Japan Coronavirus Disease (COVID-19) Situation Report.
Jordan	European Centre for Disease Prevention and Control, Global Burden of Disease Collaborative Network, Our World in Data, United Nations (UN), World Bank. Our World in Data Complete COVID-19 Dataset.
Kazakhstan	European Centre for Disease Prevention and Control, Global Burden of Disease Collaborative Network, Our World in Data, United Nations (UN), World Bank. Our World in Data Complete COVID-19 Dataset.
Kenya	European Centre for Disease Prevention and Control, Global Burden of Disease Collaborative Network, Our World in Data, United Nations (UN), World Bank. Our World in Data Complete COVID-19 Dataset.
Kuwait	European Centre for Disease Prevention and Control, Global Burden of Disease Collaborative Network, Our World in Data, United Nations (UN), World Bank. Our World in Data Complete COVID-19 Dataset.
Laos	European Centre for Disease Prevention and Control, Global Burden of Disease Collaborative Network, Our World in Data, United Nations (UN), World Bank. Our World in Data Complete COVID-19 Dataset.
Latvia	European Centre for Disease Prevention and Control, Global Burden of Disease Collaborative Network, Our World in Data, United Nations (UN), World Bank. Our World in Data Complete COVID-19 Dataset.
Lebanon	Ministry of Public Health (Lebanon). Lebanon Ministry of Public Health Monitoring of COVID-19 Infection. Beirut, Lebanon: Ministry of Public Health (Lebanon).
Lesotho	National COVID-19 Secretariat (NACOSEC) (Lesotho). Lesotho National COVID-19 Secretariat (NACOSEC) Twitter Communications and COVID-19 Updates. Lesotho: National COVID-19 Secretariat (NACOSEC) (Lesotho).
Libya	European Centre for Disease Prevention and Control, Global Burden of Disease Collaborative Network, Our World in Data, United Nations (UN), World Bank. Our World in Data Complete COVID-19 Dataset.
Lithuania	European Centre for Disease Prevention and Control, Global Burden of Disease Collaborative Network, Our World in Data, United Nations (UN), World Bank. Our World in Data Complete COVID-19 Dataset.
Luxembourg	European Centre for Disease Prevention and Control, Global Burden of Disease Collaborative Network, Our World in Data, United Nations (UN), World Bank. Our World in Data Complete COVID-19 Dataset.
Madagascar	European Centre for Disease Prevention and Control, Global Burden of Disease Collaborative Network, Our World in Data, United Nations (UN), World Bank. Our World in Data Complete COVID-19 Dataset.
Malawi	European Centre for Disease Prevention and Control, Global Burden of Disease Collaborative Network, Our World in Data, United Nations (UN), World Bank. Our World in Data Complete COVID-19 Dataset.
Malaysia	European Centre for Disease Prevention and Control, Global Burden of Disease Collaborative Network, Our World in Data, United Nations (UN), World Bank. Our World in Data Complete COVID-19 Dataset.
Maldives	European Centre for Disease Prevention and Control, Global Burden of Disease Collaborative Network, Our World in Data, United Nations (UN), World Bank. Our World in Data Complete COVID-19 Dataset.
Mali	Ministry of Health (Mali). Mali Ministry of Health and Social Affairs Communique on the Monitoring of Prevention and Response Actions to Coronavirus Disease.
Malta	European Centre for Disease Prevention and Control, Global Burden of Disease Collaborative Network, Our World in Data, United Nations (UN), World Bank. Our World in Data Complete COVID-19 Dataset.
Mauritania	Ministry of Health (Mauritania). Mauritania COVID-19 Situation Report. Nouakchott, Mauritania: Ministry of Health (Mauritania).
Mauritius	Ministry of Health and Wellness (Mauritius). Mauritius Ministry of Health and Wellness COVID-19 Statistics. Mauritius: Ministry of Health and Wellness (Mauritius).
Mexico	Government of Mexico. Mexico General Directorate of Epidemiology COVID-19 Database.

Mexico, Aguascalientes	Government of Mexico. Mexico General Directorate of Epidemiology COVID-19 Database.
Mexico, Baja California	Government of Mexico. Mexico General Directorate of Epidemiology COVID-19 Database.
Mexico, Baja California Sur	Government of Mexico. Mexico General Directorate of Epidemiology COVID-19 Database.
Mexico, Campeche	Government of Mexico. Mexico General Directorate of Epidemiology COVID-19 Database.
Mexico, Chiapas	Government of Mexico. Mexico General Directorate of Epidemiology COVID-19 Database.
Mexico, Chihuahua	Government of Mexico. Mexico General Directorate of Epidemiology COVID-19 Database.
Mexico, Coahuila	Government of Mexico. Mexico General Directorate of Epidemiology COVID-19 Database.
Mexico, Colima	Government of Mexico. Mexico General Directorate of Epidemiology COVID-19 Database.
Mexico, Durango	Government of Mexico. Mexico General Directorate of Epidemiology COVID-19 Database.
Mexico, Guanajuato	Government of Mexico. Mexico General Directorate of Epidemiology COVID-19 Database.
Mexico, Guerrero	Government of Mexico. Mexico General Directorate of Epidemiology COVID-19 Database.
Mexico, Hidalgo	Government of Mexico. Mexico General Directorate of Epidemiology COVID-19 Database.
Mexico, Jalisco	Government of Mexico. Mexico General Directorate of Epidemiology COVID-19 Database.
Mexico, México	Government of Mexico. Mexico General Directorate of Epidemiology COVID-19 Database.
Mexico, Mexico City	Government of Mexico. Mexico General Directorate of Epidemiology COVID-19 Database.
Mexico, Michoacán de Ocampo	Government of Mexico. Mexico General Directorate of Epidemiology COVID-19 Database.
Mexico, Morelos	Government of Mexico. Mexico General Directorate of Epidemiology COVID-19 Database.
Mexico, Nayarit	Government of Mexico. Mexico General Directorate of Epidemiology COVID-19 Database.
Mexico, Nuevo León	Government of Mexico. Mexico General Directorate of Epidemiology COVID-19 Database.
Mexico, Oaxaca	Government of Mexico. Mexico General Directorate of Epidemiology COVID-19 Database.
Mexico, Puebla	Government of Mexico. Mexico General Directorate of Epidemiology COVID-19 Database.
Mexico, Querétaro	Government of Mexico. Mexico General Directorate of Epidemiology COVID-19 Database.
Mexico, Quintana Roo	Government of Mexico. Mexico General Directorate of Epidemiology COVID-19 Database.
Mexico, San Luis Potosí	Government of Mexico. Mexico General Directorate of Epidemiology COVID-19 Database.
Mexico, Sinaloa	Government of Mexico. Mexico General Directorate of Epidemiology COVID-19 Database.
Mexico, Sonora	Government of Mexico. Mexico General Directorate of Epidemiology COVID-19 Database.
Mexico, Tabasco	Government of Mexico. Mexico General Directorate of Epidemiology COVID-19 Database.
Mexico, Tamaulipas	Government of Mexico. Mexico General Directorate of Epidemiology COVID-19 Database.
Mexico, Tlaxcala	Government of Mexico. Mexico General Directorate of Epidemiology COVID-19 Database.
Mexico, Veracruz de Ignacio de la Llave	Government of Mexico. Mexico General Directorate of Epidemiology COVID-19 Database.
Mexico, Yucatán	Government of Mexico. Mexico General Directorate of Epidemiology COVID-19 Database.
Mexico, Zacatecas	Government of Mexico. Mexico General Directorate of Epidemiology COVID-19 Database.
Moldova	National Agency For Public Health (ANSP) (Moldova). Moldova Epidemiological Situation Due to Infection with the New Type of Coronavirus (COVID-19).
Moldova	European Centre for Disease Prevention and Control, Global Burden of Disease Collaborative Network, Our World in Data, United Nations (UN), World Bank. Our World in Data Complete COVID-19 Dataset.
Mongolia	Ministry of Health (Mongolia). Mongolia Ministry of Health and Social Protection COVID-19 Situation Report. Ulaanbaatar, Mongolia: Ministry of Health (Mongolia).
Mongolia	European Centre for Disease Prevention and Control, Global Burden of Disease Collaborative Network, Our World in Data, United Nations (UN), World Bank. Our World in Data Complete COVID-19 Dataset.
Montenegro	Institute for Public Health (Montenegro). Montenegro Institute of Public Health COVID-19 Dashboard June 2020. Podgorica, Montenegro: Institute for Public Health (Montenegro), 2020.
Morocco	European Centre for Disease Prevention and Control, Global Burden of Disease Collaborative Network, Our World in Data, United Nations (UN), World Bank. Our World in Data Complete COVID-19 Dataset.
Mozambique	European Centre for Disease Prevention and Control, Global Burden of Disease Collaborative Network, Our World in Data, United Nations (UN), World Bank. Our World in Data Complete COVID-19 Dataset.

Mozambique	National Institute of Health (Mozambique). Mozambique National Institute of Health COVID-19 Daily Surveillance Bulletin. Maputo, Mozambique: National Institute of Health (Mozambique).
Myanmar	European Centre for Disease Prevention and Control, Global Burden of Disease Collaborative Network, Our World in Data, United Nations (UN), World Bank. Our World in Data Complete COVID-19 Dataset.
Namibia	European Centre for Disease Prevention and Control, Global Burden of Disease Collaborative Network, Our World in Data, United Nations (UN), World Bank. Our World in Data Complete COVID-19 Dataset.
Nepal	European Centre for Disease Prevention and Control, Global Burden of Disease Collaborative Network, Our World in Data, United Nations (UN), World Bank. Our World in Data Complete COVID-19 Dataset.
Netherlands	European Centre for Disease Prevention and Control, Global Burden of Disease Collaborative Network, Our World in Data, United Nations (UN), World Bank. Our World in Data Complete COVID-19 Dataset.
New Zealand	European Centre for Disease Prevention and Control, Global Burden of Disease Collaborative Network, Our World in Data, United Nations (UN), World Bank. Our World in Data Complete COVID-19 Dataset.
Niger	Ministry of Public Health (Niger). Niger Ministry of Public Health COVID-19 Twitter Communications. Niamey, Niger: Ministry of Public Health (Niger).
Niger	Ministry of Public Health (Niger). Niger Ministry of Public Health Coronavirus Situation.
Nigeria	European Centre for Disease Prevention and Control, Global Burden of Disease Collaborative Network, Our World in Data, United Nations (UN), World Bank. Our World in Data Complete COVID-19 Dataset.
North Macedonia	European Centre for Disease Prevention and Control, Global Burden of Disease Collaborative Network, Our World in Data, United Nations (UN), World Bank. Our World in Data Complete COVID-19 Dataset.
Norway	European Centre for Disease Prevention and Control, Global Burden of Disease Collaborative Network, Our World in Data, United Nations (UN), World Bank. Our World in Data Complete COVID-19 Dataset.
Oman	European Centre for Disease Prevention and Control, Global Burden of Disease Collaborative Network, Our World in Data, United Nations (UN), World Bank. Our World in Data Complete COVID-19 Dataset.
Pakistan	European Centre for Disease Prevention and Control, Global Burden of Disease Collaborative Network, Our World in Data, United Nations (UN), World Bank. Our World in Data Complete COVID-19 Dataset.
Pakistan, Azad Jammu & Kashmir	Ministry of National Health Services, Regulations & Coordination (Pakistan). Pakistan COVID-19 Dashboard.
Pakistan, Balochistan	Ministry of National Health Services, Regulations & Coordination (Pakistan). Pakistan COVID-19 Dashboard.
Pakistan, Gilgit-Baltistan	Ministry of National Health Services, Regulations & Coordination (Pakistan). Pakistan COVID-19 Dashboard.
Pakistan, Islamabad Capital Territory	Ministry of National Health Services, Regulations & Coordination (Pakistan). Pakistan COVID-19 Dashboard.
Pakistan, Khyber Pakhtunkhwa	Ministry of National Health Services, Regulations & Coordination (Pakistan). Pakistan COVID-19 Dashboard.
Pakistan, Punjab	Ministry of National Health Services, Regulations & Coordination (Pakistan). Pakistan COVID-19 Dashboard.
Pakistan, Sindh	Ministry of National Health Services, Regulations & Coordination (Pakistan). Pakistan COVID-19 Dashboard.
Palau	Ministry of Health and Social Assistance (Guatemala). Palau Ministry of Health Coronavirus Disease 2019 (COVID-19) Situation Report.
Palestine	Ministry of Health (Palestine). Palestine Ministry of Health COVID-19 Surveillance System 2020. Nablus, Palestine: Ministry of Health (Palestine).
Panama	European Centre for Disease Prevention and Control, Global Burden of Disease Collaborative Network, Our World in Data, United Nations (UN), World Bank. Our World in Data Complete COVID-19 Dataset.
Papua New Guinea	Department of Health (Papua New Guinea). Papua New Guinea Coronavirus Disease 2019 (COVID-19) Situation Reports. Papua New Guinea: Department of Health (Papua New Guinea).
Paraguay	European Centre for Disease Prevention and Control, Global Burden of Disease Collaborative Network, Our World in Data, United Nations (UN), World Bank. Our World in Data Complete COVID-19 Dataset.
Peru	General Directorate of Epidemiology, Ministry of Health (Peru). Peru National Center for Epidemiology, Prevention and Control of Diseases COVID-19 Surveillance, Prevention and Control.
Peru	Ministry of Health (Peru). Peru Ministry of Health COVID-19 Situation. Lima, Peru: Ministry of Health (Peru).
Peru	Ministry of Health (Peru). Peru Ministry of Health Twitter Communications and COVID-19 Updates. Lima, Peru: Ministry of Health (Peru).
Philippines	European Centre for Disease Prevention and Control, Global Burden of Disease Collaborative Network, Our World in Data, United Nations (UN), World Bank. Our World in Data Complete COVID-19 Dataset.
Poland	European Centre for Disease Prevention and Control, Global Burden of Disease Collaborative Network, Our World in Data, United Nations (UN), World Bank. Our World in Data Complete COVID-19 Dataset.
Portugal	European Centre for Disease Prevention and Control, Global Burden of Disease Collaborative Network, Our World in Data, United Nations (UN), World Bank. Our World in Data Complete COVID-19 Dataset.
Puerto Rico	U.S. Department of Health and Human Services. United States - COVID-19 Diagnostic Laboratory Testing (PCR Testing) Time Series. Washington DC, United States of America: U.S. Department of Health and Human Services.
Qatar	European Centre for Disease Prevention and Control, Global Burden of Disease Collaborative Network, Our World in Data, United Nations (UN), World Bank. Our World in Data Complete COVID-19 Dataset.
Romania	European Centre for Disease Prevention and Control, Global Burden of Disease Collaborative Network, Our World in Data, United Nations (UN), World Bank. Our World in Data Complete COVID-19 Dataset.

Russia	European Centre for Disease Prevention and Control, Global Burden of Disease Collaborative Network, Our World in Data, United Nations (UN), World Bank. Our World in Data Complete COVID-19 Dataset.
Rwanda	European Centre for Disease Prevention and Control, Global Burden of Disease Collaborative Network, Our World in Data, United Nations (UN), World Bank. Our World in Data Complete COVID-19 Dataset.
Saint Kitts and Nevis	European Centre for Disease Prevention and Control, Global Burden of Disease Collaborative Network, Our World in Data, United Nations (UN), World Bank. Our World in Data Complete COVID-19 Dataset.
Saint Lucia	Ministry of Health (Saint Lucia). Saint Lucia COVID-19 Data Dashboard. Saint Lucia: Ministry of Health (Saint Lucia).
Saint Vincent and the Grenadines	Ministry of Health, Wellness and the Environment (Saint Vincent and the Grenadines). Saint Vincent and the Grenadines COVID-19 Report - Ministry of Health, Wellness and the Environment. Kingstown, Saint Vincent and the Grenadines: Ministry of Health, Wellness and the Environment (Saint Vincent and the Grenadines).
Saint Vincent and the Grenadines	European Centre for Disease Prevention and Control, Global Burden of Disease Collaborative Network, Our World in Data, United Nations (UN), World Bank. Our World in Data Complete COVID-19 Dataset.
São Tomé and Príncipe	Ministry of Health (Sao Tome and Principe). Sao Tome and Principe Corona Virus (COVID-19) Daily Bulletin . Ministry of Health (Sao Tome and Principe).
Saudi Arabia	European Centre for Disease Prevention and Control, Global Burden of Disease Collaborative Network, Our World in Data, United Nations (UN), World Bank. Our World in Data Complete COVID-19 Dataset.
Senegal	European Centre for Disease Prevention and Control, Global Burden of Disease Collaborative Network, Our World in Data, United Nations (UN), World Bank. Our World in Data Complete COVID-19 Dataset.
Serbia	European Centre for Disease Prevention and Control, Global Burden of Disease Collaborative Network, Our World in Data, United Nations (UN), World Bank. Our World in Data Complete COVID-19 Dataset.
Sierra Leone	Ministry of Health and Sanitation (Sierra Leone). Sierra Leone Coronavirus Disease (COVID-19) Situational Reports. 2020.
Singapore	European Centre for Disease Prevention and Control, Global Burden of Disease Collaborative Network, Our World in Data, United Nations (UN), World Bank. Our World in Data Complete COVID-19 Dataset.
Slovakia	European Centre for Disease Prevention and Control, Global Burden of Disease Collaborative Network, Our World in Data, United Nations (UN), World Bank. Our World in Data Complete COVID-19 Dataset.
Slovenia	European Centre for Disease Prevention and Control, Global Burden of Disease Collaborative Network, Our World in Data, United Nations (UN), World Bank. Our World in Data Complete COVID-19 Dataset.
Somalia	Humanitarian Response, United Nations Office for the Coordination of Humanitarian Affairs (OCHA), World Health Organization (WHO). Somalia COVID-19 Dashboard - WHO. Geneva, Switzerland: World Health Organization (WHO).
South Africa	European Centre for Disease Prevention and Control, Global Burden of Disease Collaborative Network, Our World in Data, United Nations (UN), World Bank. Our World in Data Complete COVID-19 Dataset.
South Korea	European Centre for Disease Prevention and Control, Global Burden of Disease Collaborative Network, Our World in Data, United Nations (UN), World Bank. Our World in Data Complete COVID-19 Dataset.
South Sudan	European Centre for Disease Prevention and Control, Global Burden of Disease Collaborative Network, Our World in Data, United Nations (UN), World Bank. Our World in Data Complete COVID-19 Dataset.
Spain	European Centre for Disease Prevention and Control, Global Burden of Disease Collaborative Network, Our World in Data, United Nations (UN), World Bank. Our World in Data Complete COVID-19 Dataset.
Spain	Ministry of Health, Consumption and Social Welfare (Spain). Spain Ministry of Health, Consumption, and Social Welfare COVID-19 Situation Update. Spain: Ministry of Health, Consumption and Social Welfare (Spain).
Spain, Andalusia	Ministry of Health, Consumption and Social Welfare (Spain). Spain Ministry of Health, Consumption, and Social Welfare COVID-19 Situation Update. Spain: Ministry of Health, Consumption and Social Welfare (Spain).
Spain, Aragon	Government of Aragon (Spain). Spain - Aragon Open Data: Daily Facts and Figures About the Coronavirus.
Spain, Asturias	Government of Asturias (Spain). Spain - Asturias Open Data: COVID-19 Evolution 2020.
Spain, Balearic Islands	Government of the Balearic Islands. Spain - Balearic Islands Ministry of Health and Consumption News About the Coronavirus COVID-19.
Spain, Basque Country	Basque Government Department of Health. Spain - Basque Country Information on the Evolution of the Coronavirus Epidemiological Bulletin.
Spain, Canary Islands	Government of the Canary Islands (Spain). Spain - Canary Islands Government COVID-19 Dashboard.
Spain, Cantabria	Cantabrian Health Service (Spain), Government of Cantabria (Spain). Spain - Cantabria Epidemiological Situation of COVID-19. Spain: Cantabrian Health Service (Spain).
Spain, Castile and León	Castile and León Board, Health Commission (Spain). Spain - Castile and León Open Data: Coronavirus (COVID-19) Epidemiological Situation.
Spain, Castile and León	Castile and León Board, Health Commission (Spain). Spain - Castile and León Open Data: Coronavirus Tests.
Spain, Castilla–La Mancha	Ministry of Health, Consumption and Social Welfare (Spain). Spain Ministry of Health, Consumption, and Social Welfare COVID-19 Situation Update. Spain: Ministry of Health, Consumption and Social Welfare (Spain).
Spain, Catalonia	Ministry of Health, Consumption and Social Welfare (Spain). Spain Ministry of Health, Consumption, and Social Welfare COVID-19 Situation Update. Spain: Ministry of Health, Consumption and Social Welfare (Spain).
Spain, Ceuta	Ministry of Health, Consumption and Social Welfare (Spain). Spain Ministry of Health, Consumption, and Social Welfare COVID-19 Situation Update. Spain: Ministry of Health, Consumption and Social Welfare (Spain).
Spain, Community of Madrid	Ministry of Health, Consumption and Social Welfare (Spain). Spain Ministry of Health, Consumption, and Social Welfare COVID-19 Situation Update. Spain: Ministry of Health, Consumption and Social Welfare (Spain).

Spain, Extremadura	Ministry of Health, Consumption and Social Welfare (Spain). Spain Ministry of Health, Consumption, and Social Welfare COVID-19 Situation Update. Spain: Ministry of Health, Consumption and Social Welfare (Spain).
Spain, Galicia	Galician Healthcare Service (Spain), Regional Government of Galicia (Spain). Spain - Galicia Regional Government COVID-19 Press Releases 2020. Spain: Regional Government of Galicia (Spain).
Spain, La Rioja	Government of La Rioja (Spain). Spain - La Rioja Covid-19 Tests Performed per Days. Brazil: Government of La Rioja (Spain).
Spain, Melilla	Ministry of Health, Consumption and Social Welfare (Spain). Spain Ministry of Health, Consumption, and Social Welfare COVID-19 Situation Update. Spain: Ministry of Health, Consumption and Social Welfare (Spain).
Spain, Murcia	Institute of Health Carlos III (Spain), Ministry of Health, Consumption and Social Welfare (Spain). Spain Carlos III Health Institute Situation of COVID-19. Madrid, Spain: Institute of Health Carlos III (Spain).
Spain, Murcia	Ministry of Health of the Region of Murcia (Spain). COVID-19 Region of Murcia - Spain. Spain: Ministry of Health of the Region of Murcia (Spain).
Spain, Navarre	Government of Navarra (Spain). Spain - Navarra COVID-19 Testing, Deaths, Hospitalisation Data May-June 2020. Spain: Government of Navarra (Spain), 2020.
Spain, Valencian Community	Ministry of Health, Consumption and Social Welfare (Spain). Spain Ministry of Health, Consumption, and Social Welfare COVID-19 Situation Update. Spain: Ministry of Health, Consumption and Social Welfare (Spain).
Sri Lanka	European Centre for Disease Prevention and Control, Global Burden of Disease Collaborative Network, Our World in Data, United Nations (UN), World Bank. Our World in Data Complete COVID-19 Dataset.
Sri Lanka	Health Promotion Bureau (Sri Lanka). Sri Lanka Health Promotion Bureau COVID-2019 Situation Report. Sri Lanka: Health Promotion Bureau (Sri Lanka).
Sri Lanka	Ministry of Health and Indigenous Medical Services (Sri Lanka). Sri Lanka Epidemiology Unit Coronavirus Disease 2019 (COVID-19) Situation Report.
Sudan	Sudan Health Observatory. Sudan Health Observatory COVID-19 Situation and Updates. Sudan: Sudan Health Observatory.
Sudan	Federal Ministry of Health (Sudan). Sudan Federal Ministry of Health COVID-19 Daily Situation Report. Khartoum, Sudan: Federal Ministry of Health (Sudan).
Suriname	Public Health Office (Suriname). Suriname COVID-19 Situation Report 2020. Suriname: Directorate of National Security (Suriname).
Suriname	European Centre for Disease Prevention and Control, Global Burden of Disease Collaborative Network, Our World in Data, United Nations (UN), World Bank. Our World in Data Complete COVID-19 Dataset.
Sweden	Public Health Agency of Sweden. Sweden Public Health Agency COVID-19 Weekly Report. Östersund, Sweden: Public Health Agency of Sweden.
Sweden	European Centre for Disease Prevention and Control, Global Burden of Disease Collaborative Network, Our World in Data, United Nations (UN), World Bank. Our World in Data Complete COVID-19 Dataset.
Switzerland	Federal Office of Public Health (Switzerland). Switzerland COVID-19 PCR Antigen Tests by Region.
Switzerland	European Centre for Disease Prevention and Control, Global Burden of Disease Collaborative Network, Our World in Data, United Nations (UN), World Bank. Our World in Data Complete COVID-19 Dataset.
Taiwan (Province of China)	European Centre for Disease Prevention and Control, Global Burden of Disease Collaborative Network, Our World in Data, United Nations (UN), World Bank. Our World in Data Complete COVID-19 Dataset.
Thailand	European Centre for Disease Prevention and Control, Global Burden of Disease Collaborative Network, Our World in Data, United Nations (UN), World Bank. Our World in Data Complete COVID-19 Dataset.
The Bahamas	Ministry of Health (Bahamas). Bahamas - COVID 19 Dashboard. Bahamas: Ministry of Health (Bahamas).
The Bahamas	European Centre for Disease Prevention and Control, Global Burden of Disease Collaborative Network, Our World in Data, United Nations (UN), World Bank. Our World in Data Complete COVID-19 Dataset.
The Gambia	Ministry of Health (Gambia), World Health Organization (WHO). Gambia COVID-19 Situational Outbreak Report. Gambia: Ministry of Health (Gambia).
Timor-Leste	European Centre for Disease Prevention and Control, Global Burden of Disease Collaborative Network, Our World in Data, United Nations (UN), World Bank. Our World in Data Complete COVID-19 Dataset.
Togo	European Centre for Disease Prevention and Control, Global Burden of Disease Collaborative Network, Our World in Data, United Nations (UN), World Bank. Our World in Data Complete COVID-19 Dataset.
Trinidad and Tobago	European Centre for Disease Prevention and Control, Global Burden of Disease Collaborative Network, Our World in Data, United Nations (UN), World Bank. Our World in Data Complete COVID-19 Dataset.
Tunisia	European Centre for Disease Prevention and Control, Global Burden of Disease Collaborative Network, Our World in Data, United Nations (UN), World Bank. Our World in Data Complete COVID-19 Dataset.
Turkey	European Centre for Disease Prevention and Control, Global Burden of Disease Collaborative Network, Our World in Data, United Nations (UN), World Bank. Our World in Data Complete COVID-19 Dataset.
Uganda	European Centre for Disease Prevention and Control, Global Burden of Disease Collaborative Network, Our World in Data, United Nations (UN), World Bank. Our World in Data Complete COVID-19 Dataset.
UK	European Centre for Disease Prevention and Control, Global Burden of Disease Collaborative Network, Our World in Data, United Nations (UN), World Bank. Our World in Data Complete COVID-19 Dataset.
UK	Department of Health (United Kingdom), NHS England, Public Health England. United Kingdom COVID-19 Cases. London, England: Public Health England, 2020.
UK	Department of Health (United Kingdom), NHS England, Public Health England. United Kingdom COVID-19 Testing Data. London, England: Public Health England.
UK, England	Department of Health (United Kingdom), NHS England, Public Health England. United Kingdom COVID-19 Cases. London, England: Public Health England, 2020.

UK, England	Department of Health (United Kingdom), NHS England, Public Health England. United Kingdom COVID-19 Testing Data. London, England: Public Health England.
UK, Northern Ireland	Department of Health (United Kingdom), NHS England, Public Health England. United Kingdom COVID-19 Cases. London, England: Public Health England, 2020.
UK, Northern Ireland	Department of Health (United Kingdom), NHS England, Public Health England. United Kingdom COVID-19 Testing Data. London, England: Public Health England.
UK, Scotland	Department of Health (United Kingdom), NHS England, Public Health England. United Kingdom COVID-19 Cases. London, England: Public Health England, 2020.
UK, Scotland	Department of Health (United Kingdom), NHS England, Public Health England. United Kingdom COVID-19 Testing Data. London, England: Public Health England.
UK, Wales	Department of Health (United Kingdom), NHS England, Public Health England. United Kingdom COVID-19 Cases. London, England: Public Health England, 2020.
UK, Wales	Department of Health (United Kingdom), NHS England, Public Health England. United Kingdom COVID-19 Testing Data. London, England: Public Health England.
Ukraine	European Centre for Disease Prevention and Control, Global Burden of Disease Collaborative Network, Our World in Data, United Nations (UN), World Bank. Our World in Data Complete COVID-19 Dataset.
United Arab Emirates	European Centre for Disease Prevention and Control, Global Burden of Disease Collaborative Network, Our World in Data, United Nations (UN), World Bank. Our World in Data Complete COVID-19 Dataset.
Uruguay	European Centre for Disease Prevention and Control, Global Burden of Disease Collaborative Network, Our World in Data, United Nations (UN), World Bank. Our World in Data Complete COVID-19 Dataset.
USA	European Centre for Disease Prevention and Control, Global Burden of Disease Collaborative Network, Our World in Data, United Nations (UN), World Bank. Our World in Data Complete COVID-19 Dataset.
USA, Alabama	U.S. Department of Health and Human Services. United States - COVID-19 Diagnostic Laboratory Testing (PCR Testing) Time Series. Washington DC, United States of America: U.S. Department of Health and Human Services.
USA, Alaska	U.S. Department of Health and Human Services. United States - COVID-19 Diagnostic Laboratory Testing (PCR Testing) Time Series. Washington DC, United States of America: U.S. Department of Health and Human Services.
USA, Arizona	U.S. Department of Health and Human Services. United States - COVID-19 Diagnostic Laboratory Testing (PCR Testing) Time Series. Washington DC, United States of America: U.S. Department of Health and Human Services.
USA, Arkansas	U.S. Department of Health and Human Services. United States - COVID-19 Diagnostic Laboratory Testing (PCR Testing) Time Series. Washington DC, United States of America: U.S. Department of Health and Human Services.
USA, California	U.S. Department of Health and Human Services. United States - COVID-19 Diagnostic Laboratory Testing (PCR Testing) Time Series. Washington DC, United States of America: U.S. Department of Health and Human Services.
USA, Colorado	U.S. Department of Health and Human Services. United States - COVID-19 Diagnostic Laboratory Testing (PCR Testing) Time Series. Washington DC, United States of America: U.S. Department of Health and Human Services.
USA, Connecticut	U.S. Department of Health and Human Services. United States - COVID-19 Diagnostic Laboratory Testing (PCR Testing) Time Series. Washington DC, United States of America: U.S. Department of Health and Human Services.
USA, Delaware	U.S. Department of Health and Human Services. United States - COVID-19 Diagnostic Laboratory Testing (PCR Testing) Time Series. Washington DC, United States of America: U.S. Department of Health and Human Services.
USA, Florida	U.S. Department of Health and Human Services. United States - COVID-19 Diagnostic Laboratory Testing (PCR Testing) Time Series. Washington DC, United States of America: U.S. Department of Health and Human Services.
USA, Georgia	U.S. Department of Health and Human Services. United States - COVID-19 Diagnostic Laboratory Testing (PCR Testing) Time Series. Washington DC, United States of America: U.S. Department of Health and Human Services.
USA, Hawaii	U.S. Department of Health and Human Services. United States - COVID-19 Diagnostic Laboratory Testing (PCR Testing) Time Series. Washington DC, United States of America: U.S. Department of Health and Human Services.
USA, Idaho	U.S. Department of Health and Human Services. United States - COVID-19 Diagnostic Laboratory Testing (PCR Testing) Time Series. Washington DC, United States of America: U.S. Department of Health and Human Services.
USA, Illinois	U.S. Department of Health and Human Services. United States - COVID-19 Diagnostic Laboratory Testing (PCR Testing) Time Series. Washington DC, United States of America: U.S. Department of Health and Human Services.
USA, Indiana	U.S. Department of Health and Human Services. United States - COVID-19 Diagnostic Laboratory Testing (PCR Testing) Time Series. Washington DC, United States of America: U.S. Department of Health and Human Services.
USA, Iowa	U.S. Department of Health and Human Services. United States - COVID-19 Diagnostic Laboratory Testing (PCR Testing) Time Series. Washington DC, United States of America: U.S. Department of Health and Human Services.
USA, Kansas	U.S. Department of Health and Human Services. United States - COVID-19 Diagnostic Laboratory Testing (PCR Testing) Time Series. Washington DC, United States of America: U.S. Department of Health and Human Services.
USA, Kentucky	U.S. Department of Health and Human Services. United States - COVID-19 Diagnostic Laboratory Testing (PCR Testing) Time Series. Washington DC, United States of America: U.S. Department of Health and Human Services.
USA, Louisiana	U.S. Department of Health and Human Services. United States - COVID-19 Diagnostic Laboratory Testing (PCR Testing) Time Series. Washington DC, United States of America: U.S. Department of Health and Human Services.
USA, Maine	U.S. Department of Health and Human Services. United States - COVID-19 Diagnostic Laboratory Testing (PCR Testing) Time Series. Washington DC, United States of America: U.S. Department of Health and Human Services.
USA, Maryland	U.S. Department of Health and Human Services. United States - COVID-19 Diagnostic Laboratory Testing (PCR Testing) Time Series. Washington DC, United States of America: U.S. Department of Health and Human Services.
USA, Massachusetts	U.S. Department of Health and Human Services. United States - COVID-19 Diagnostic Laboratory Testing (PCR Testing) Time Series. Washington DC, United States of America: U.S. Department of Health and Human Services.
USA, Michigan	U.S. Department of Health and Human Services. United States - COVID-19 Diagnostic Laboratory Testing (PCR Testing) Time Series. Washington DC, United States of America: U.S. Department of Health and Human Services.

USA, Minnesota	U.S. Department of Health and Human Services. United States - COVID-19 Diagnostic Laboratory Testing (PCR Testing) Time Series. Washington DC, United States of America: U.S. Department of Health and Human Services.
USA, Mississippi	U.S. Department of Health and Human Services. United States - COVID-19 Diagnostic Laboratory Testing (PCR Testing) Time Series. Washington DC, United States of America: U.S. Department of Health and Human Services.
USA, Missouri	U.S. Department of Health and Human Services. United States - COVID-19 Diagnostic Laboratory Testing (PCR Testing) Time Series. Washington DC, United States of America: U.S. Department of Health and Human Services.
USA, Montana	U.S. Department of Health and Human Services. United States - COVID-19 Diagnostic Laboratory Testing (PCR Testing) Time Series. Washington DC, United States of America: U.S. Department of Health and Human Services.
USA, Nebraska	U.S. Department of Health and Human Services. United States - COVID-19 Diagnostic Laboratory Testing (PCR Testing) Time Series. Washington DC, United States of America: U.S. Department of Health and Human Services.
USA, Nevada	U.S. Department of Health and Human Services. United States - COVID-19 Diagnostic Laboratory Testing (PCR Testing) Time Series. Washington DC, United States of America: U.S. Department of Health and Human Services.
USA, New Hampshire	U.S. Department of Health and Human Services. United States - COVID-19 Diagnostic Laboratory Testing (PCR Testing) Time Series. Washington DC, United States of America: U.S. Department of Health and Human Services.
USA, New Jersey	U.S. Department of Health and Human Services. United States - COVID-19 Diagnostic Laboratory Testing (PCR Testing) Time Series. Washington DC, United States of America: U.S. Department of Health and Human Services.
USA, New Mexico	U.S. Department of Health and Human Services. United States - COVID-19 Diagnostic Laboratory Testing (PCR Testing) Time Series. Washington DC, United States of America: U.S. Department of Health and Human Services.
USA, New York	U.S. Department of Health and Human Services. United States - COVID-19 Diagnostic Laboratory Testing (PCR Testing) Time Series. Washington DC, United States of America: U.S. Department of Health and Human Services.
USA, North Carolina	U.S. Department of Health and Human Services. United States - COVID-19 Diagnostic Laboratory Testing (PCR Testing) Time Series. Washington DC, United States of America: U.S. Department of Health and Human Services.
USA, North Dakota	U.S. Department of Health and Human Services. United States - COVID-19 Diagnostic Laboratory Testing (PCR Testing) Time Series. Washington DC, United States of America: U.S. Department of Health and Human Services.
USA, Ohio	U.S. Department of Health and Human Services. United States - COVID-19 Diagnostic Laboratory Testing (PCR Testing) Time Series. Washington DC, United States of America: U.S. Department of Health and Human Services.
USA, Oklahoma	U.S. Department of Health and Human Services. United States - COVID-19 Diagnostic Laboratory Testing (PCR Testing) Time Series. Washington DC, United States of America: U.S. Department of Health and Human Services.
USA, Oregon	U.S. Department of Health and Human Services. United States - COVID-19 Diagnostic Laboratory Testing (PCR Testing) Time Series. Washington DC, United States of America: U.S. Department of Health and Human Services.
USA, Pennsylvania	U.S. Department of Health and Human Services. United States - COVID-19 Diagnostic Laboratory Testing (PCR Testing) Time Series. Washington DC, United States of America: U.S. Department of Health and Human Services.
USA, Rhode Island	U.S. Department of Health and Human Services. United States - COVID-19 Diagnostic Laboratory Testing (PCR Testing) Time Series. Washington DC, United States of America: U.S. Department of Health and Human Services.
USA, South Carolina	U.S. Department of Health and Human Services. United States - COVID-19 Diagnostic Laboratory Testing (PCR Testing) Time Series. Washington DC, United States of America: U.S. Department of Health and Human Services.
USA, South Dakota	U.S. Department of Health and Human Services. United States - COVID-19 Diagnostic Laboratory Testing (PCR Testing) Time Series. Washington DC, United States of America: U.S. Department of Health and Human Services.
USA, Tennessee	U.S. Department of Health and Human Services. United States - COVID-19 Diagnostic Laboratory Testing (PCR Testing) Time Series. Washington DC, United States of America: U.S. Department of Health and Human Services.
USA, Texas	U.S. Department of Health and Human Services. United States - COVID-19 Diagnostic Laboratory Testing (PCR Testing) Time Series. Washington DC, United States of America: U.S. Department of Health and Human Services.
USA, Utah	U.S. Department of Health and Human Services. United States - COVID-19 Diagnostic Laboratory Testing (PCR Testing) Time Series. Washington DC, United States of America: U.S. Department of Health and Human Services.
USA, Vermont	U.S. Department of Health and Human Services. United States - COVID-19 Diagnostic Laboratory Testing (PCR Testing) Time Series. Washington DC, United States of America: U.S. Department of Health and Human Services.
USA, Virginia	U.S. Department of Health and Human Services. United States - COVID-19 Diagnostic Laboratory Testing (PCR Testing) Time Series. Washington DC, United States of America: U.S. Department of Health and Human Services.
USA, Washington	Washington State Department of Health. United States - Washington State Department of Health COVID-19 Cases by County and CDC Event Date.
USA, Washington	Washington State Department of Health. United States - Washington State Department of Health COVID-19 Deaths by County, Date, Age Group and Sex. Olympia, United States of America: Washington State Department of Health.
USA, Washington	Washington State Department of Health. United States - Washington State Department of Health COVID-19 Tests by County and Specimen Collection Date. Olympia, United States of America: Washington State Department of Health.
USA, Washington, DC	U.S. Department of Health and Human Services. United States - COVID-19 Diagnostic Laboratory Testing (PCR Testing) Time Series. Washington DC, United States of America: U.S. Department of Health and Human Services.
USA, West Virginia	U.S. Department of Health and Human Services. United States - COVID-19 Diagnostic Laboratory Testing (PCR Testing) Time Series. Washington DC, United States of America: U.S. Department of Health and Human Services.
USA, Wisconsin	U.S. Department of Health and Human Services. United States - COVID-19 Diagnostic Laboratory Testing (PCR Testing) Time Series. Washington DC, United States of America: U.S. Department of Health and Human Services.
USA, Wyoming	U.S. Department of Health and Human Services. United States - COVID-19 Diagnostic Laboratory Testing (PCR Testing) Time Series. Washington DC, United States of America: U.S. Department of Health and Human Services.
Vietnam	European Centre for Disease Prevention and Control, Global Burden of Disease Collaborative Network, Our World in Data, United Nations (UN), World Bank. Our World in Data Complete COVID-19 Dataset.
Virgin Islands	U.S. Department of Health and Human Services. United States - COVID-19 Diagnostic Laboratory Testing (PCR Testing) Time Series. Washington DC, United States of America: U.S. Department of Health and Human Services.

Yemen	Humanitarian Response, United Nations Office for the Coordination of Humanitarian Affairs (OCHA), ReliefWeb. Yemen COVID-19 Preparedness and Response Snapshot 2020.
Yemen	Yemen Supreme National Emergency Committee for Covid19. Yemen Supreme National Emergency Committee for COVID-19 - Twitter.
Zambia	European Centre for Disease Prevention and Control, Global Burden of Disease Collaborative Network, Our World in Data, United Nations (UN), World Bank. Our World in Data Complete COVID-19 Dataset.
Zimbabwe	European Centre for Disease Prevention and Control, Global Burden of Disease Collaborative Network, Our World in Data, United Nations (UN), World Bank. Our World in Data Complete COVID-19 Dataset.

Section 6: Age-stratified hospitalisations sources

Figure S6. Age-stratified hospitalisation data coverage by location

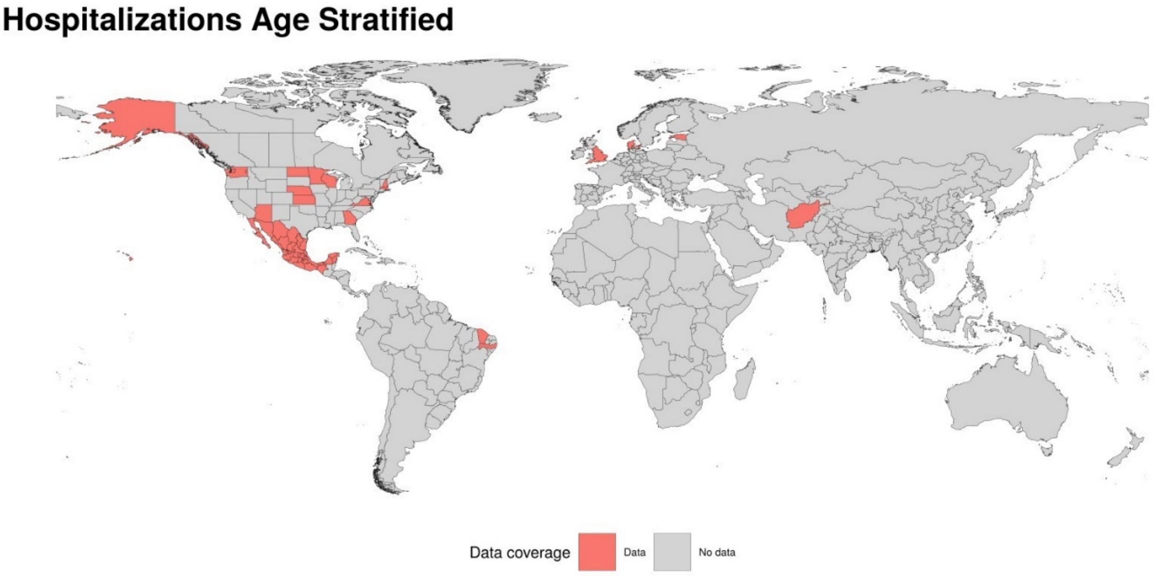

Table S6. Age-stratified hospitalisation data sources by location

Location	Source
Afghanistan	Ministry of Public Health (Afghanistan). Afghanistan COVID-19 Epidemic Monitoring Dashboard. Kabul, Afghanistan: Ministry of Public Health (Afghanistan).
Brazil, Ceará	Secretary of Health of the State of Ceara (Brazil).. Brazil - CearÃ¡ COVID-19 Epidemiological Bulletins. Brazil: Secretary of Health of the State of Ceara (Brazil)., 2020.
Brazil, Pernambuco	State Department of Health Pernambuco (Brazil). COVID-19 in the World, in Brazil and in Pernambuco.
Denmark	Danish Health Authority. Denmark Inpatients: Gender and Age - Accumulated.
Denmark	Statens Serum Institute. Denmark Covid-19 Deaths.
Estonia	Health Board (Estonia). Estonia COVID-19 Open Data. Estonia: Health Board (Estonia).
Mexico	Directorate General of Epidemiology, Secretariat of Health (Mexico). Mexico Epidemiological Report on the Situation of COVID-19. 2020.
Mexico, Aguascalientes	Directorate General of Epidemiology, Secretariat of Health (Mexico). Mexico Epidemiological Report on the Situation of COVID-19. 2020.
Mexico, Baja California	Directorate General of Epidemiology, Secretariat of Health (Mexico). Mexico Epidemiological Report on the Situation of COVID-19. 2020.
Mexico, Baja California Sur	Directorate General of Epidemiology, Secretariat of Health (Mexico). Mexico Epidemiological Report on the Situation of COVID-19. 2020.
Mexico, Campeche	Directorate General of Epidemiology, Secretariat of Health (Mexico). Mexico Epidemiological Report on the Situation of COVID-19. 2020.
Mexico, Chiapas	Directorate General of Epidemiology, Secretariat of Health (Mexico). Mexico Epidemiological Report on the Situation of COVID-19. 2020.
Mexico, Chihuahua	Directorate General of Epidemiology, Secretariat of Health (Mexico). Mexico Epidemiological Report on the Situation of COVID-19. 2020.
Mexico, Coahuila	Directorate General of Epidemiology, Secretariat of Health (Mexico). Mexico Epidemiological Report on the Situation of COVID-19. 2020.
Mexico, Colima	Directorate General of Epidemiology, Secretariat of Health (Mexico). Mexico Epidemiological Report on the Situation of COVID-19. 2020.
Mexico, Durango	Directorate General of Epidemiology, Secretariat of Health (Mexico). Mexico Epidemiological Report on the Situation of COVID-19. 2020.
Mexico, Guanajuato	Directorate General of Epidemiology, Secretariat of Health (Mexico). Mexico Epidemiological Report on the Situation of COVID-19. 2020.

Mexico, Guerrero	Directorate General of Epidemiology, Secretariat of Health (Mexico). Mexico Epidemiological Report on the Situation of COVID-19. 2020.
Mexico, Hidalgo	Directorate General of Epidemiology, Secretariat of Health (Mexico). Mexico Epidemiological Report on the Situation of COVID-19. 2020.
Mexico, Jalisco	Directorate General of Epidemiology, Secretariat of Health (Mexico). Mexico Epidemiological Report on the Situation of COVID-19. 2020.
Mexico, México	Directorate General of Epidemiology, Secretariat of Health (Mexico). Mexico Epidemiological Report on the Situation of COVID-19. 2020.
Mexico, Mexico City	Directorate General of Epidemiology, Secretariat of Health (Mexico). Mexico Epidemiological Report on the Situation of COVID-19. 2020.
Mexico, Michoacán de Ocampo	Directorate General of Epidemiology, Secretariat of Health (Mexico). Mexico Epidemiological Report on the Situation of COVID-19. 2020.
Mexico, Morelos	Directorate General of Epidemiology, Secretariat of Health (Mexico). Mexico Epidemiological Report on the Situation of COVID-19. 2020.
Mexico, Nayarit	Directorate General of Epidemiology, Secretariat of Health (Mexico). Mexico Epidemiological Report on the Situation of COVID-19. 2020.
Mexico, Nuevo León	Directorate General of Epidemiology, Secretariat of Health (Mexico). Mexico Epidemiological Report on the Situation of COVID-19. 2020.
Mexico, Oaxaca	Directorate General of Epidemiology, Secretariat of Health (Mexico). Mexico Epidemiological Report on the Situation of COVID-19. 2020.
Mexico, Puebla	Directorate General of Epidemiology, Secretariat of Health (Mexico). Mexico Epidemiological Report on the Situation of COVID-19. 2020.
Mexico, Querétaro	Directorate General of Epidemiology, Secretariat of Health (Mexico). Mexico Epidemiological Report on the Situation of COVID-19. 2020.
Mexico, Quintana Roo	Directorate General of Epidemiology, Secretariat of Health (Mexico). Mexico Epidemiological Report on the Situation of COVID-19. 2020.
Mexico, San Luis Potosí	Directorate General of Epidemiology, Secretariat of Health (Mexico). Mexico Epidemiological Report on the Situation of COVID-19. 2020.
Mexico, Sinaloa	Directorate General of Epidemiology, Secretariat of Health (Mexico). Mexico Epidemiological Report on the Situation of COVID-19. 2020.
Mexico, Sonora	Directorate General of Epidemiology, Secretariat of Health (Mexico). Mexico Epidemiological Report on the Situation of COVID-19. 2020.
Mexico, Tabasco	Directorate General of Epidemiology, Secretariat of Health (Mexico). Mexico Epidemiological Report on the Situation of COVID-19. 2020.
Mexico, Tamaulipas	Directorate General of Epidemiology, Secretariat of Health (Mexico). Mexico Epidemiological Report on the Situation of COVID-19. 2020.
Mexico, Tlaxcala	Directorate General of Epidemiology, Secretariat of Health (Mexico). Mexico Epidemiological Report on the Situation of COVID-19. 2020.
Mexico, Veracruz de Ignacio de la Llave	Directorate General of Epidemiology, Secretariat of Health (Mexico). Mexico Epidemiological Report on the Situation of COVID-19. 2020.
Mexico, Yucatán	Directorate General of Epidemiology, Secretariat of Health (Mexico). Mexico Epidemiological Report on the Situation of COVID-19. 2020.
Mexico, Zacatecas	Directorate General of Epidemiology, Secretariat of Health (Mexico). Mexico Epidemiological Report on the Situation of COVID-19. 2020.
UK, England	National Health Service (United Kingdom), Public Health England. United Kingdom Coronavirus (COVID-19) Data Dashboard.
USA, Alaska	Alaska Department of Health and Social Services. United States - Alaska Department of Public Health and Social Services COVID-19 Demographic Distribution of Confirmed Case. Anchorage, AK, United States of America: Alaska Department of Health and Social Services, 2020.
USA, Arizona	Arizona Department of Health Services (ADHS). United States - Arizona Department of Health Services COVID-19 Data 2020. Phoenix, AZ, United States of America: Arizona Department of Health Services (ADHS), 2020.
USA, Georgia	Georgia Department of Public Health (United States). United States - Georgia Department of Public Health COVID-19 Daily Status Report 2020. Atlanta, GA, United States of America: Georgia Department of Public Health (United States), 2020.
USA, Hawaii	Hawaii State Department of Health. United States - Hawaii Department of Health COVID-19 Current Situation 2020. Honolulu, HI, United States of America: Hawaii State Department of Health, 2020.
USA, Kansas	Kansas Department of Health and Environment. United States - Kansas Department of Health and Environment Coronavirus Disease 2019 (COVID-19) Case Summary 2020. Topeka, KS, United States of America: Kansas Department of Health and Environment, 2020.

USA, Massachusetts	Massachuttes Department of Public Health (MDPH). United States - Massachusetts Department of Public Health COVID-19 Cases, Quarantine and Monitoring 2020. Massachuttes Department of Public Health (MDPH), 2020.
USA, Minnesota	Minnesota Department of Health. United States - Minnesota Department of Health Situation Update for COVID-19. St. Paul, United States of America: Minnesota Department of Health, 2020.
USA, Nebraska	Nebraska Department of Health and Human Services. United States - Nebraska Department of Health and Human Services Coronavirus COVID-19 Cases Dashboard. 2020.
USA, New Hampshire	New Hampshire Department of Health and Human Services. United States - New Hampshire Department of Health and Human Services 2019 Novel Coronavirus (COVID-19) Summary Report 2020. Concord, New Hampshire: New Hampshire Department of Health and Human Services, 2020.
USA, North Dakota	North Dakota Department of Health (United States). United States - North Dakota Department of Health Coronavirus Cases 2020. United States of America: North Dakota Department of Health (United States), 2020.
USA, Rhode Island	Rhode Island Department of Health (United States). United States - Rhode Island Department of Health COVID-19 Data Tracker 2020. Providence, RI, United States of America: Rhode Island Department of Health (United States), 2020.
USA, Virginia	Virginia Department of Health (United States). United States - Virginia Department of Health COVID-19 Cases 2020. United States of America: Virginia Department of Health (United States), 2020.
USA, Virginia	Virginia Hospital and Healthcare Association (United States). United States - Virginia Hospital and Healthcare Association COVID-19 Dashboard. 2020.
USA, Washington	Washington State Department of Health. United States - Washington State Department of Health COVID-19 Data Dashboard. Olympia, United States of America: Washington State Department of Health, 2020.
USA, Wisconsin	Wisconsin Department of Health Services. United States - Wisconsin Department of Health Services COVID-19 Data 2020. Madison, WI, United States of America: Wisconsin Department of Health Services, 2020.
