# Supplementary material for: Estimating global, regional, and national daily and cumulative infections with SARS-CoV-2 through Nov 14, 2021: a statistical analysis
Source: Lancet. 2022 Jun 25;399(10344):2351–80. doi: 10.1016/S0140-6736(22)00484-6 (PMC8993157; doi:10.1016/S0140-6736(22)00484-6)
Supplement: Supplementary appendix 3 [file mmc3.pdf]

THE LANCET

Supplementary appendix 3

This appendix formed part of the original submission and has been peer reviewed. We post it as supplied by the authors.

Supplement to: COVID-19 Cumulative Infection Collaborators. Estimating global, regional, and national daily and cumulative infections with SARS-CoV-2 through Nov 14, 2021: a statistical analysis. *Lancet* 2022; published online April 8. https://doi.org/10.1016/S0140-6736(22)00484-6.

Appendix 3: location-specific plots for "Estimating global, regional, and national daily and cumulative infections with SARS-CoV-2 through Nov 14, 2021: a statistical analysis"

This appendix provides further location-specific plots for "Estimating global, regional, and national daily and cumulative infections with SARS-CoV-2 through Nov 14, 2021: a statistical analysis".

Armenia

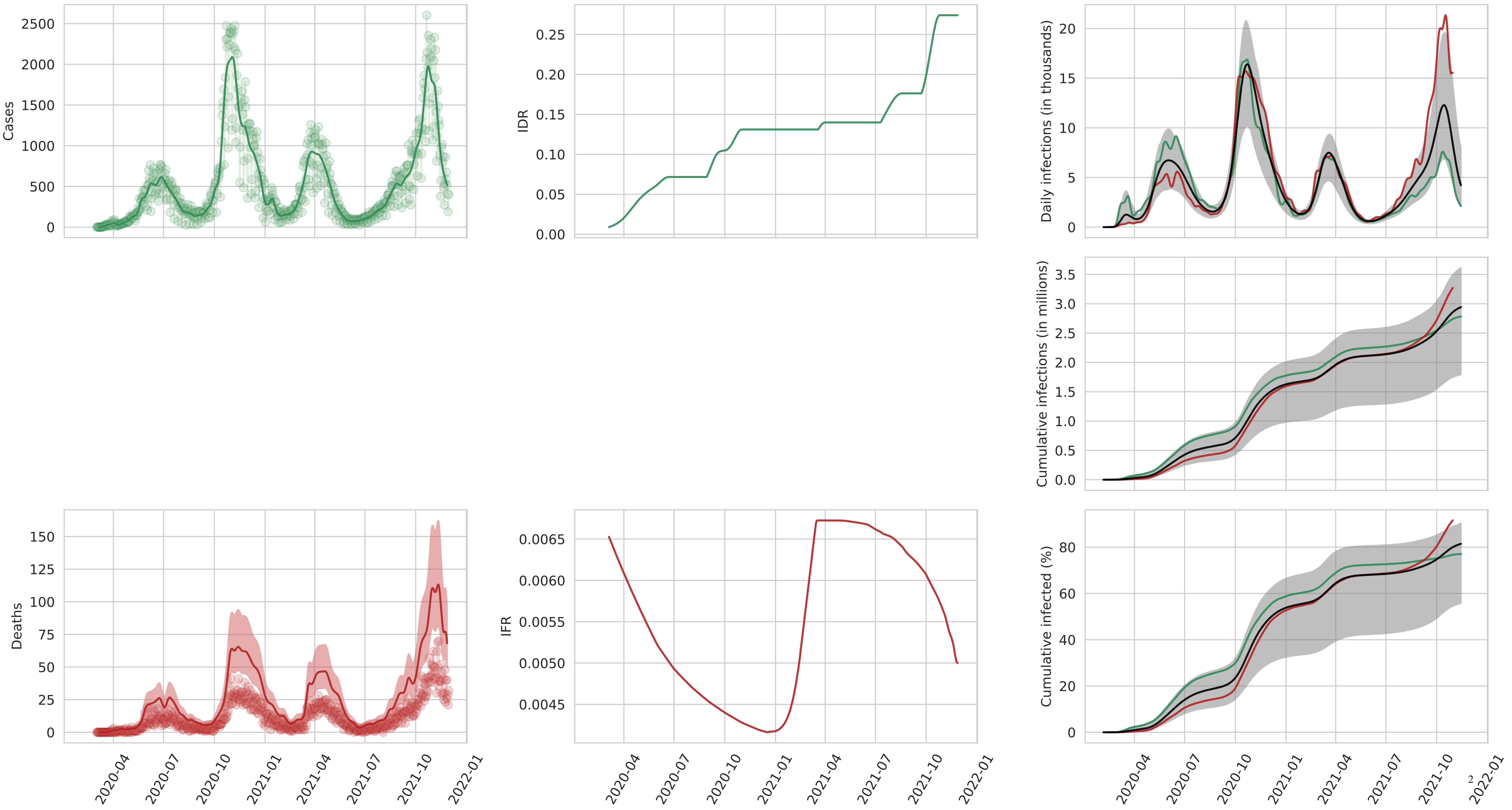

Cases
IDR
Daily infections (in thousands)
Cumulative infections (in millions)
Deaths
IFR
Cumulative infected (%)
2020-04
2020-07
2020-10
2021-01
2021-04
2021-07
2021-10
2022-01

Azerbaijan

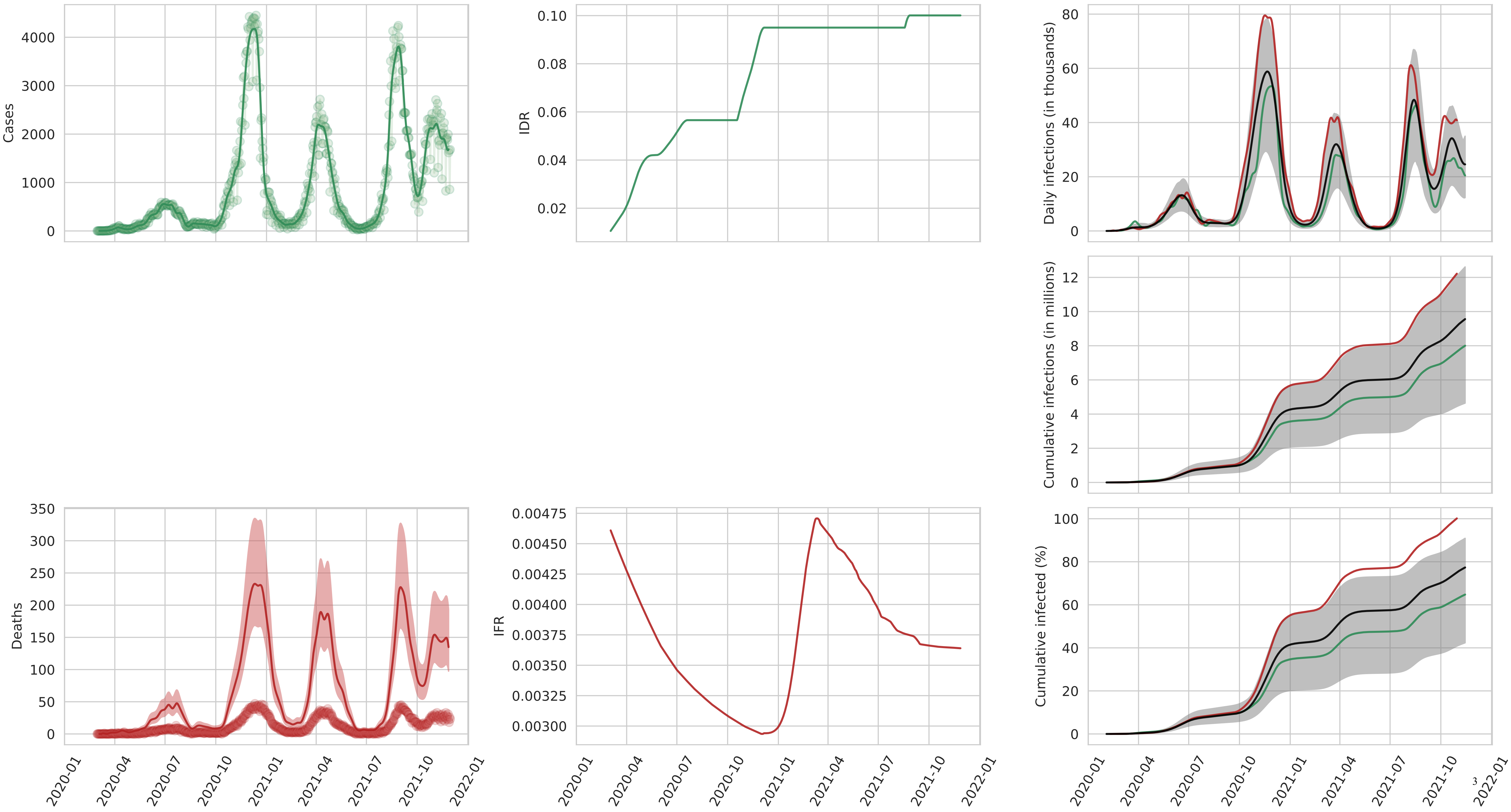

Cases
IDR
Daily infections (in thousands)
Cumulative infections (in millions)
Deaths
IFR
Cumulative infected (%)
2020-01
2020-04
2020-07
2020-10
2021-01
2021-04
2021-07
2021-10
2022-01

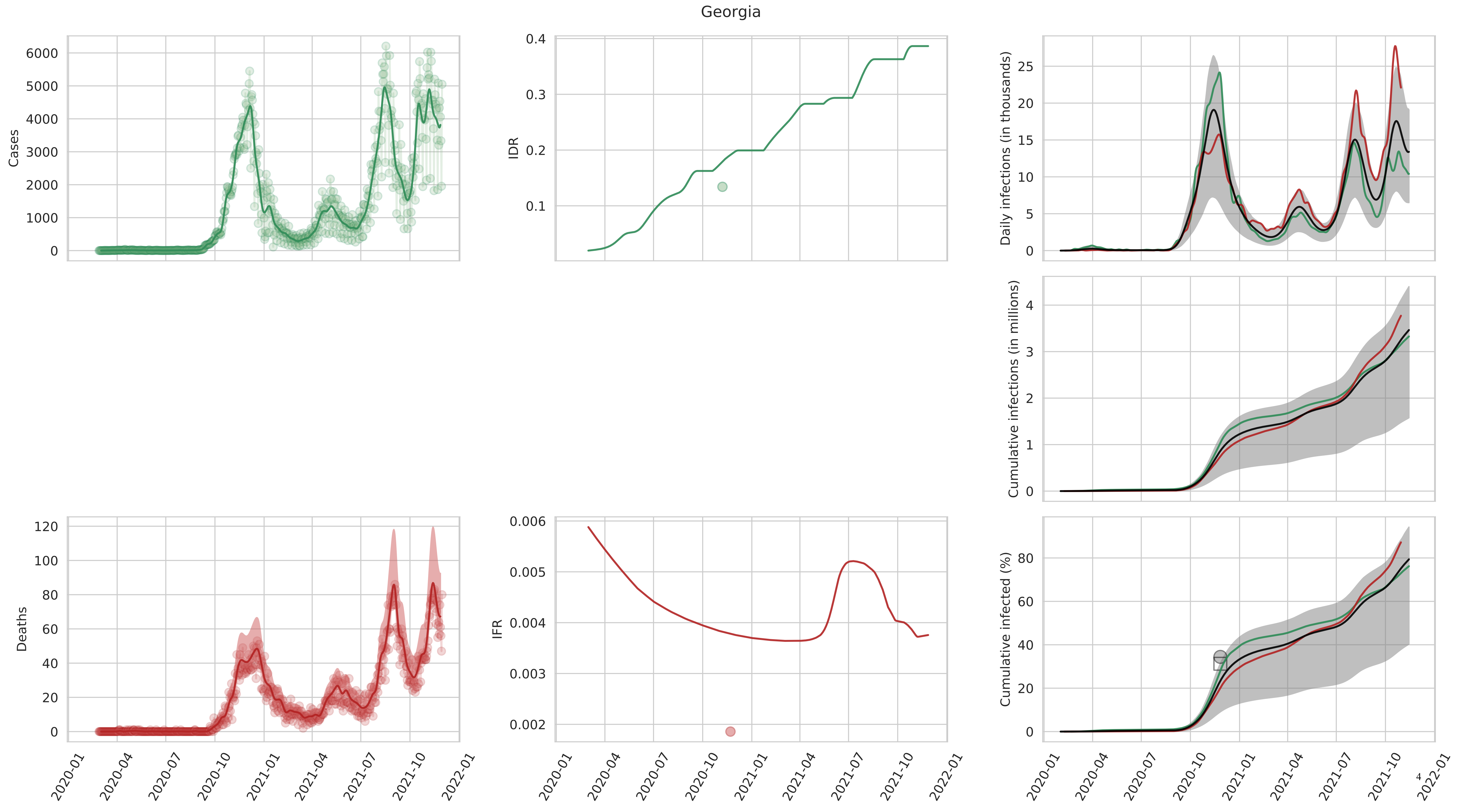

Georgia
Cases
IDR
Daily infections (in thousands)
Cumulative infections (in millions)
Deaths
IFR
Cumulative infected (%)

Kazakhstan

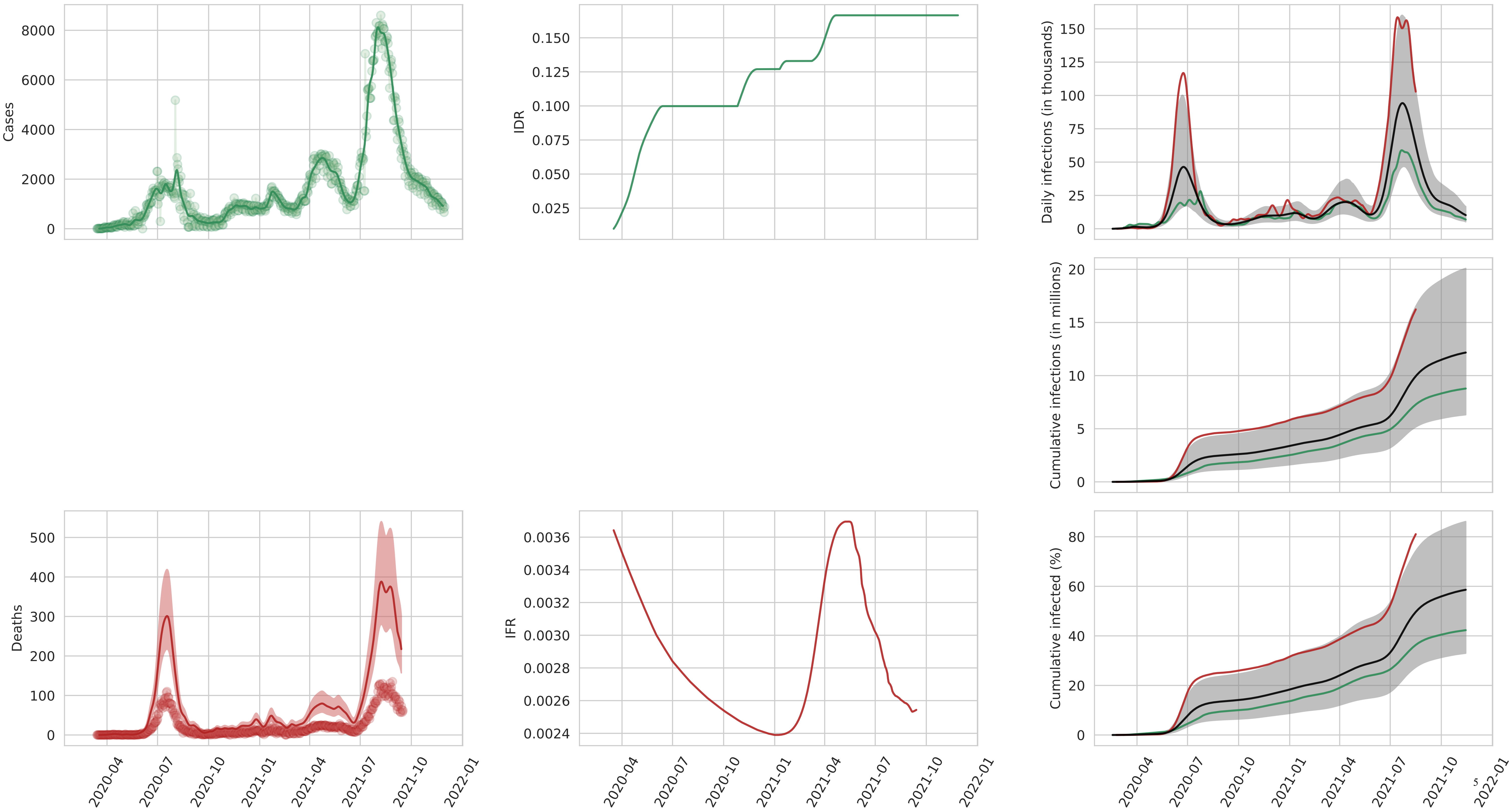

Cases
8000
6000
4000
2000
0
IDR
0.150
0.125
0.100
0.075
0.050
0.025
Daily infections (in thousands)
150
125
100
75
50
25
0
Cumulative infections (in millions)
20
15
10
5
0
Deaths
500
400
300
200
100
0
IFR
0.0036
0.0034
0.0032
0.0030
0.0028
0.0026
0.0024
Cumulative infected (%)
80
60
40
20
0
2020-04
2020-07
2020-10
2021-01
2021-04
2021-07
2021-10
2022-01

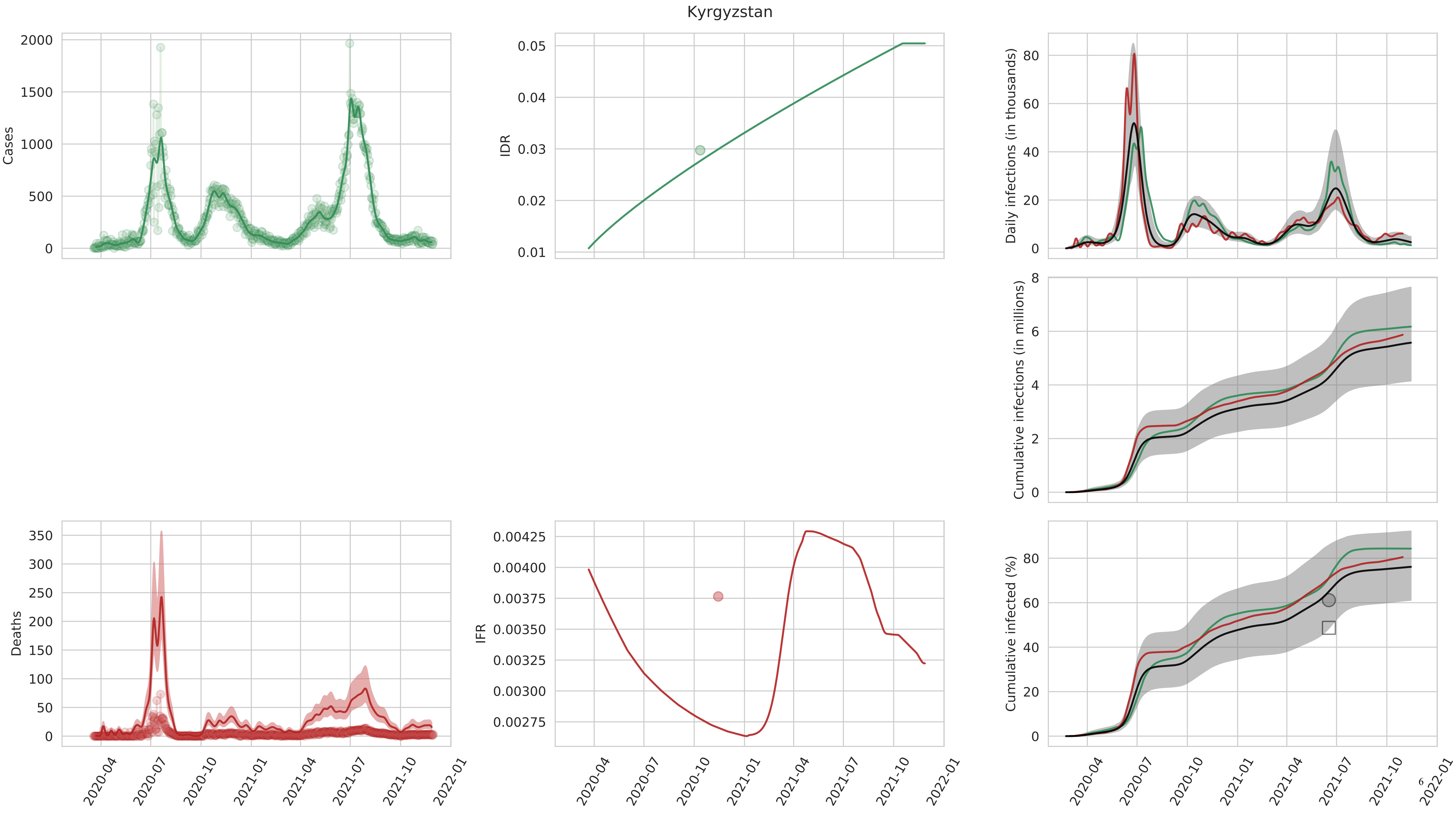
Kyrgyzstan
Cases
IDR
Daily infections (in thousands)
Cumulative infections (in millions)
Deaths
IFR
Cumulative infected (%)
2020-04
2020-07
2020-10
2021-01
2021-04
2021-07
2021-10
2022-01
6

Mongolia

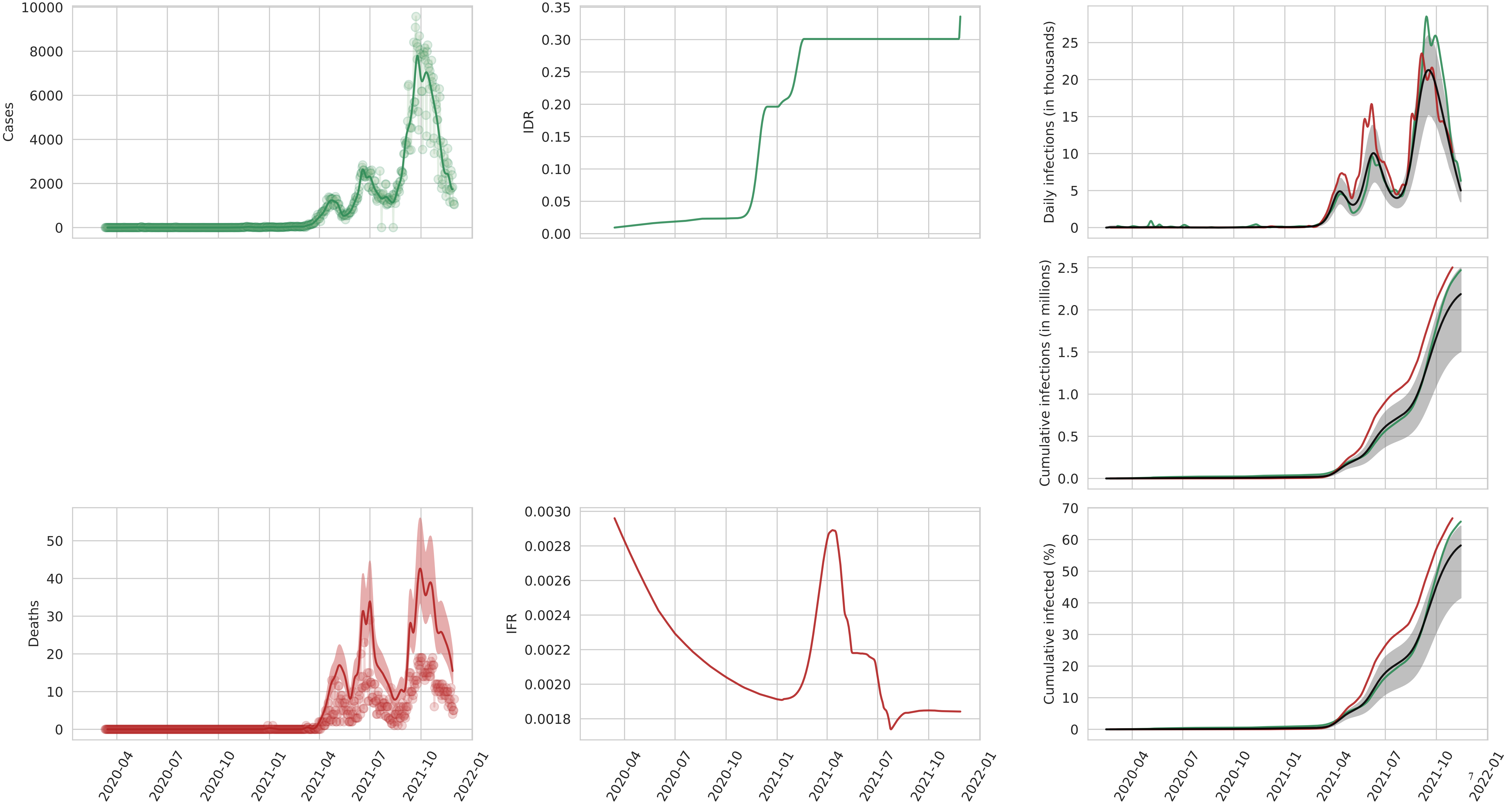
Cases
IDR
Daily infections (in thousands)
Cumulative infections (in millions)
Deaths
IFR
Cumulative infected (%)
2020-04
2020-07
2020-10
2021-01
2021-04
2021-07
2021-10
2022-01

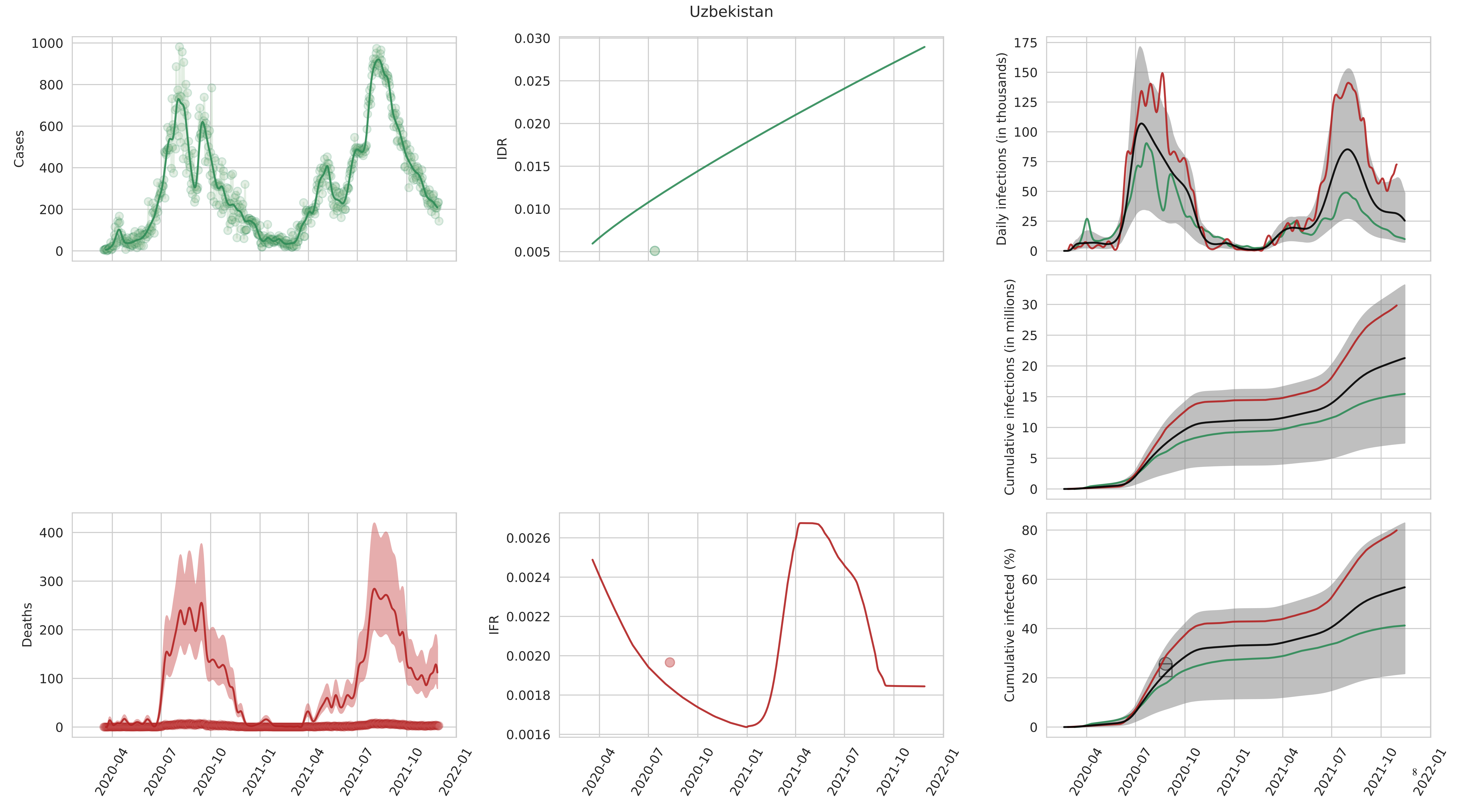

Uzbekistan
Cases
IDR
Daily infections (in thousands)
Cumulative infections (in millions)
Deaths
IFR
Cumulative infected (%)
2020-04
2020-07
2020-10
2021-01
2021-04
2021-07
2021-10
2022-01

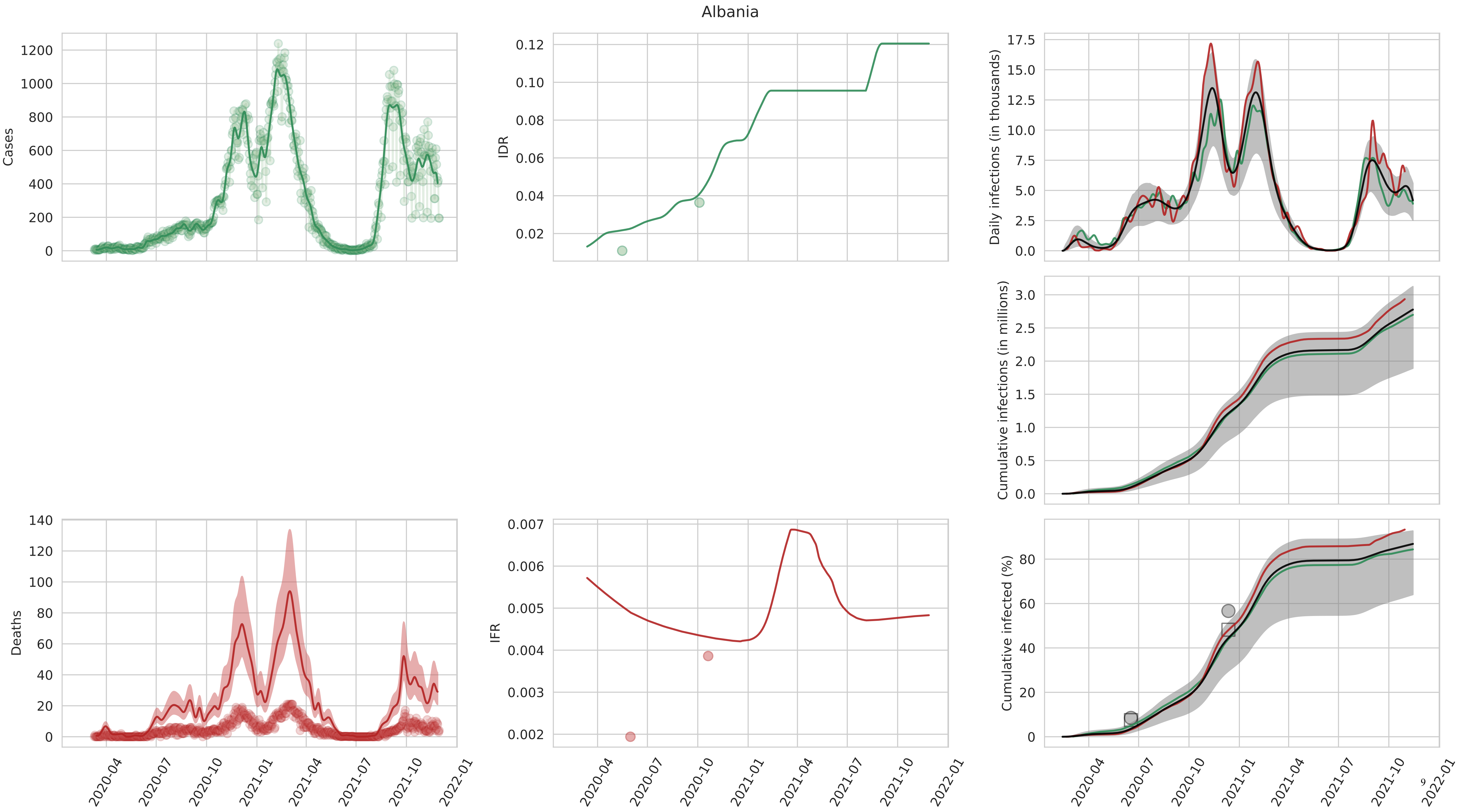
Albania
Cases
IDR
Daily infections (in thousands)
Cumulative infections (in millions)
Deaths
IFR
Cumulative infected (%)
2020-04
2020-07
2020-10
2021-01
2021-04
2021-07
2021-10
2022-01

Bosnia and Herzegovina

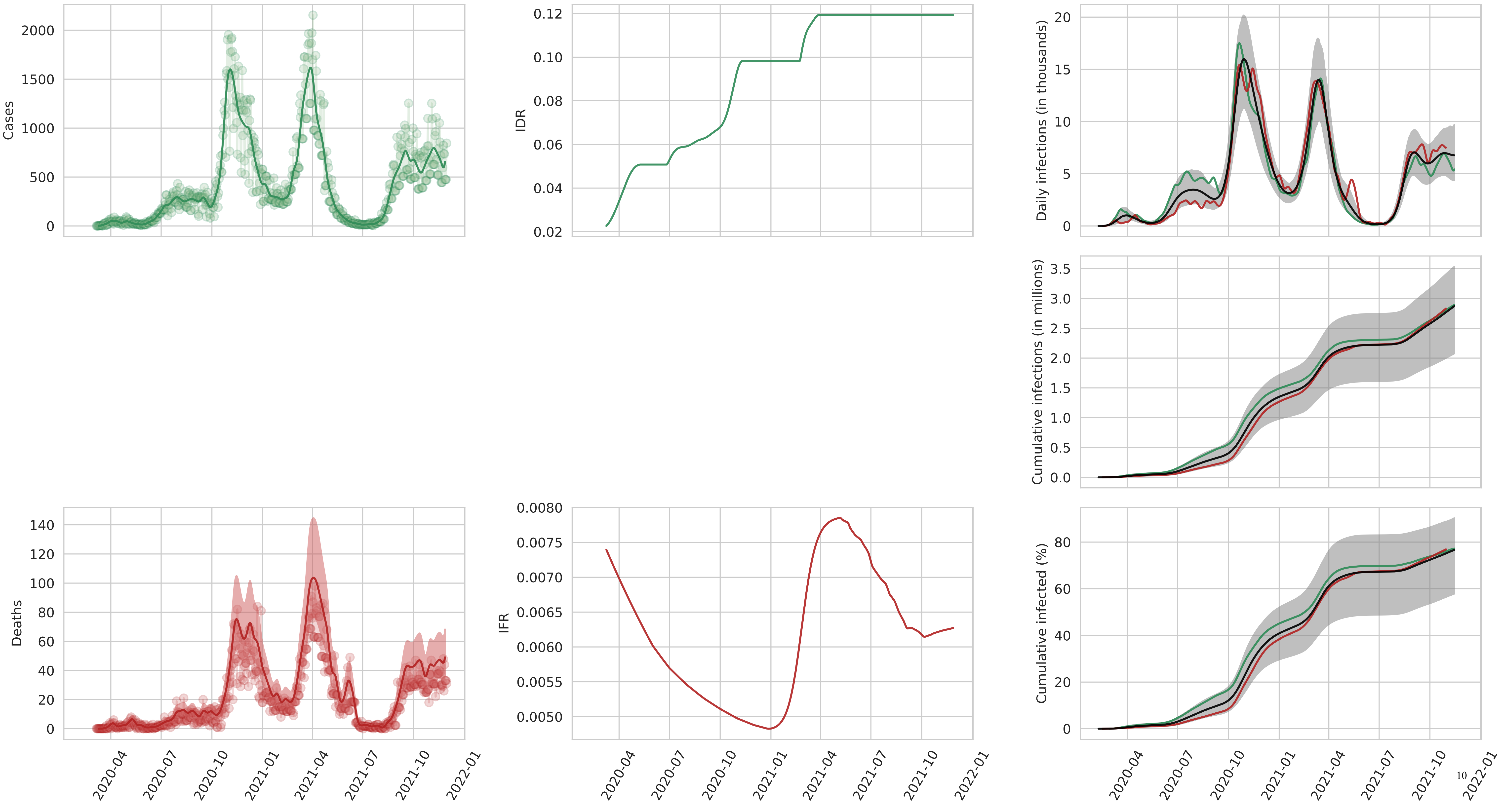

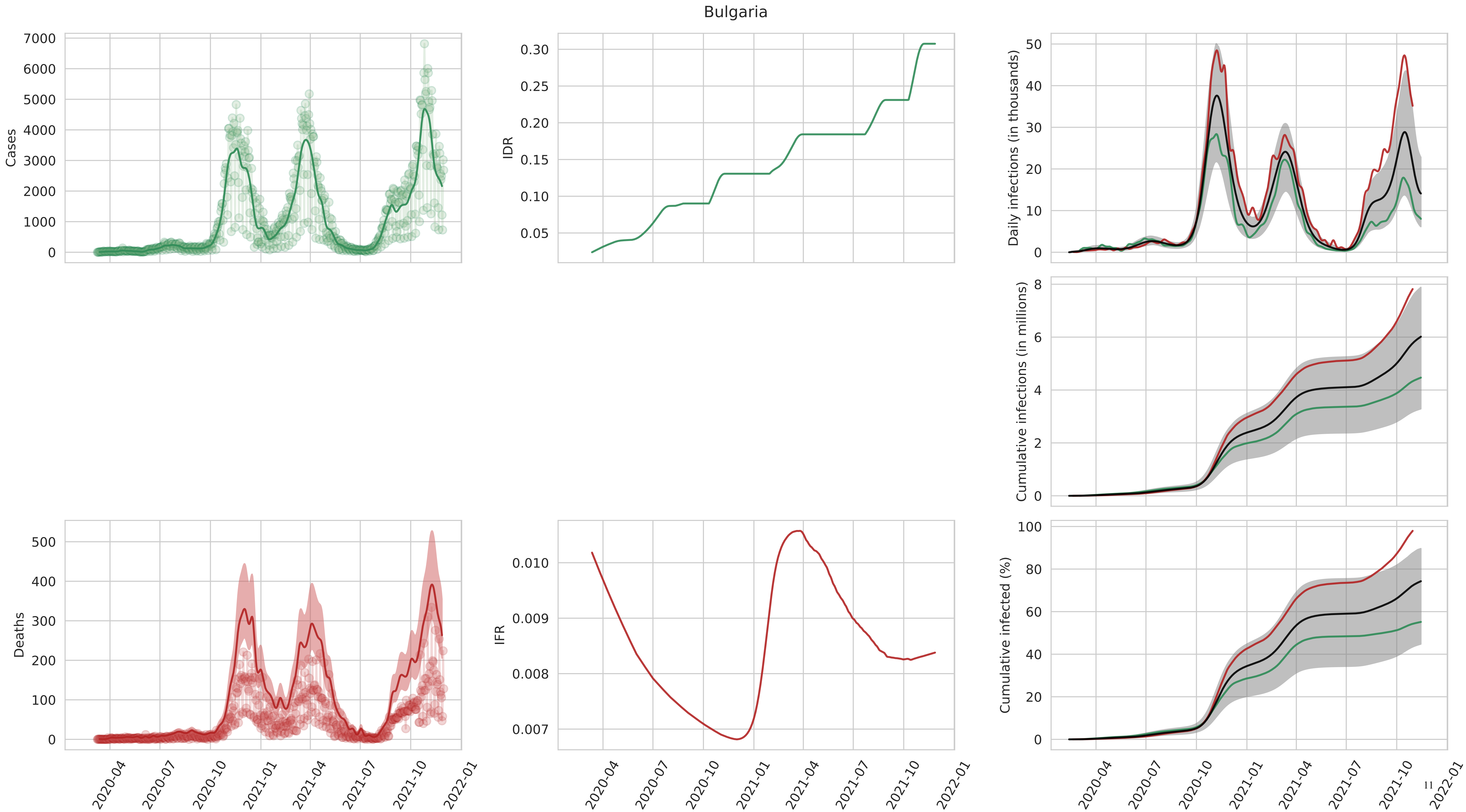
Bulgaria
Cases
IDR
Daily infections (in thousands)
Cumulative infections (in millions)
Deaths
IFR
Cumulative infected (%)
2020-04
2020-07
2020-10
2021-01
2021-04
2021-07
2021-10
2022-01

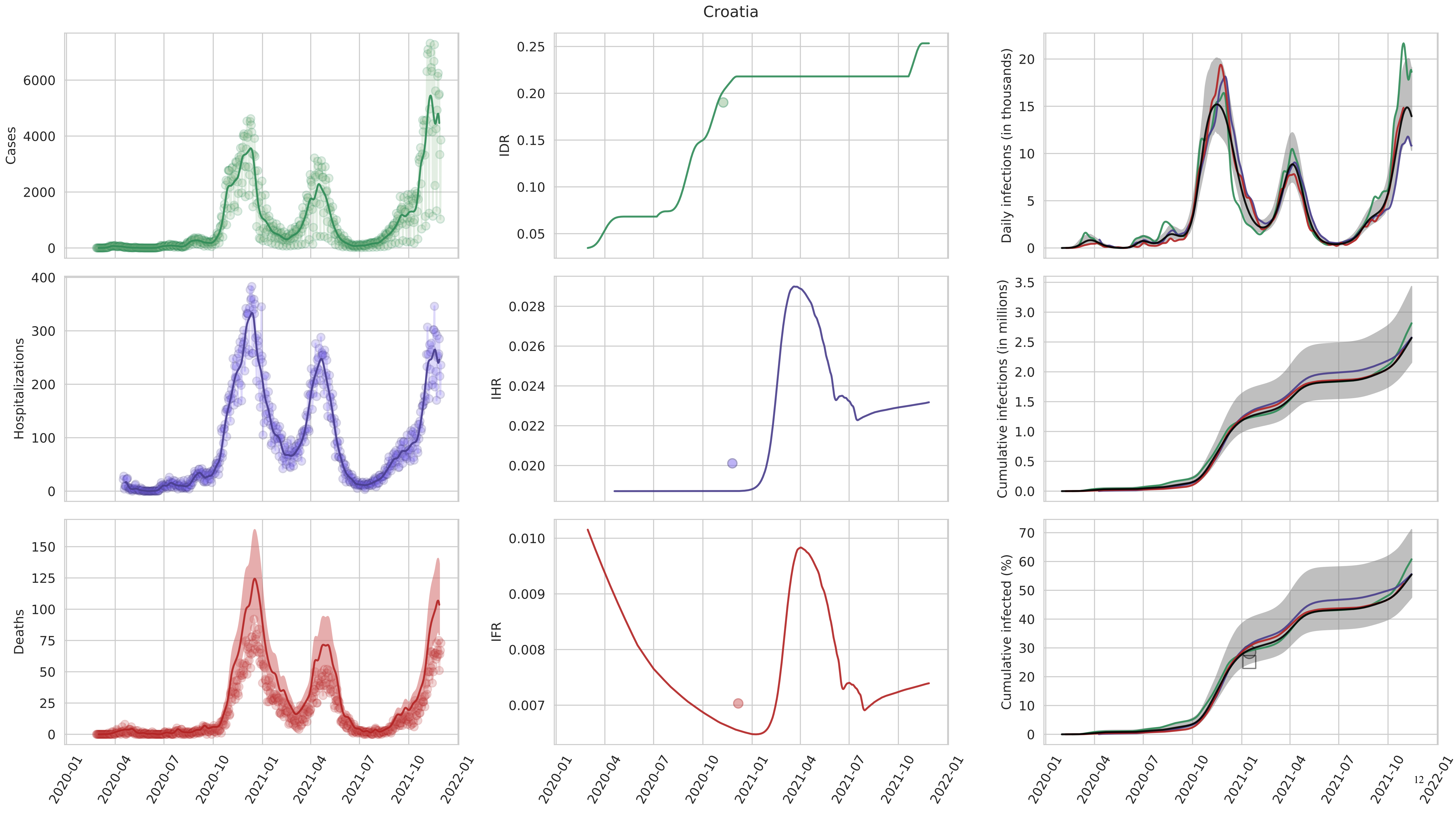
Croatia
Cases
0
2000
4000
6000
Hospitalizations
0
100
200
300
400
Deaths
0
25
50
75
100
125
150
IDR
0.05
0.10
0.15
0.20
0.25
IHR
0.020
0.022
0.024
0.026
0.028
IFR
0.007
0.008
0.009
0.010
Daily infections (in thousands)
0
5
10
15
20
Cumulative infections (in millions)
0.0
0.5
1.0
1.5
2.0
2.5
3.0
3.5
Cumulative infected (%)
0
10
20
30
40
50
60
70
2020-01
2020-04
2020-07
2020-10
2021-01
2021-04
2021-07
2021-10
2022-01

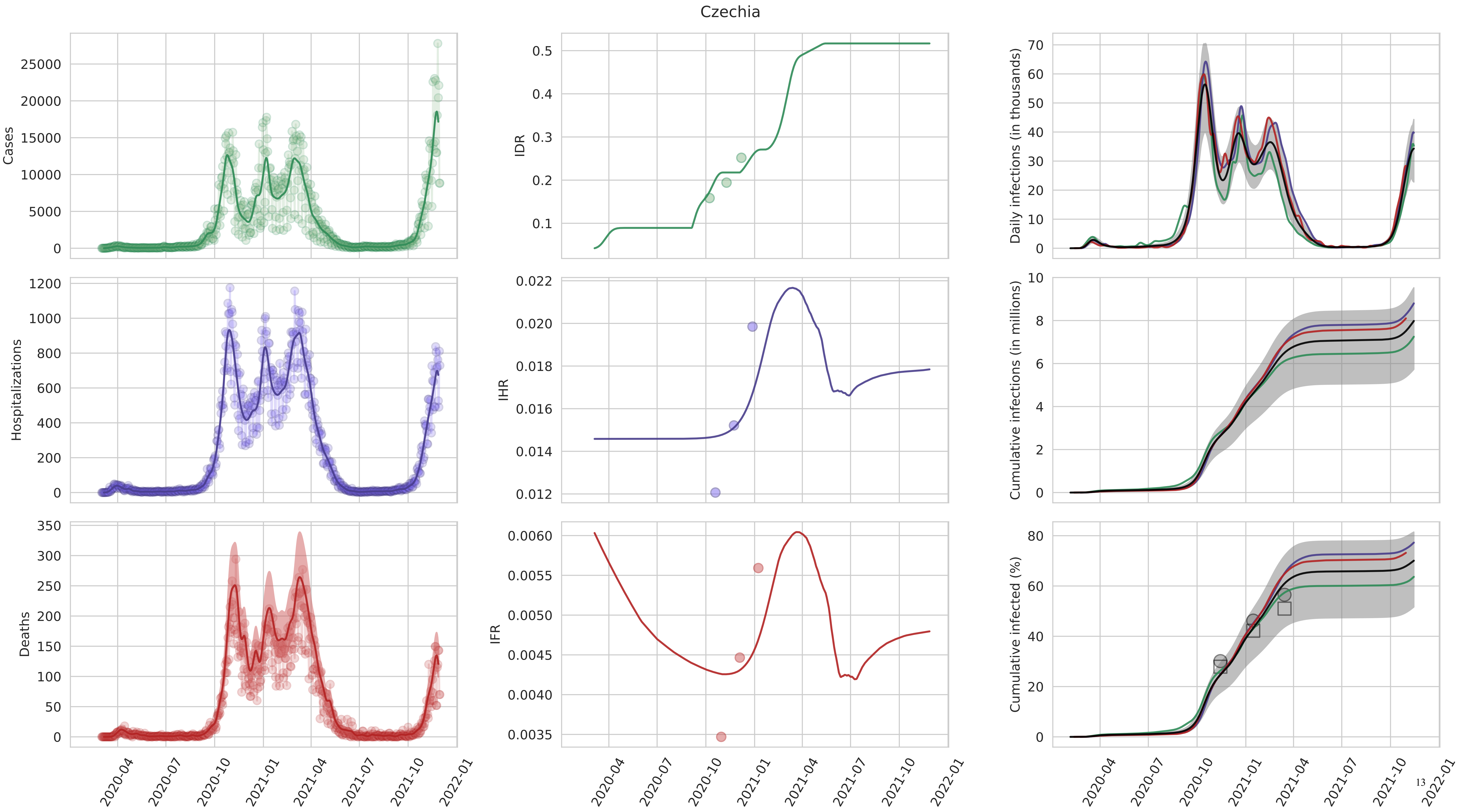
Czechia
Cases
IDR
Daily infections (in thousands)
Hospitalizations
IHR
Cumulative infections (in millions)
Deaths
IFR
Cumulative infected (%)
2020-04
2020-07
2020-10
2021-01
2021-04
2021-07
2021-10
2022-01

Hungary

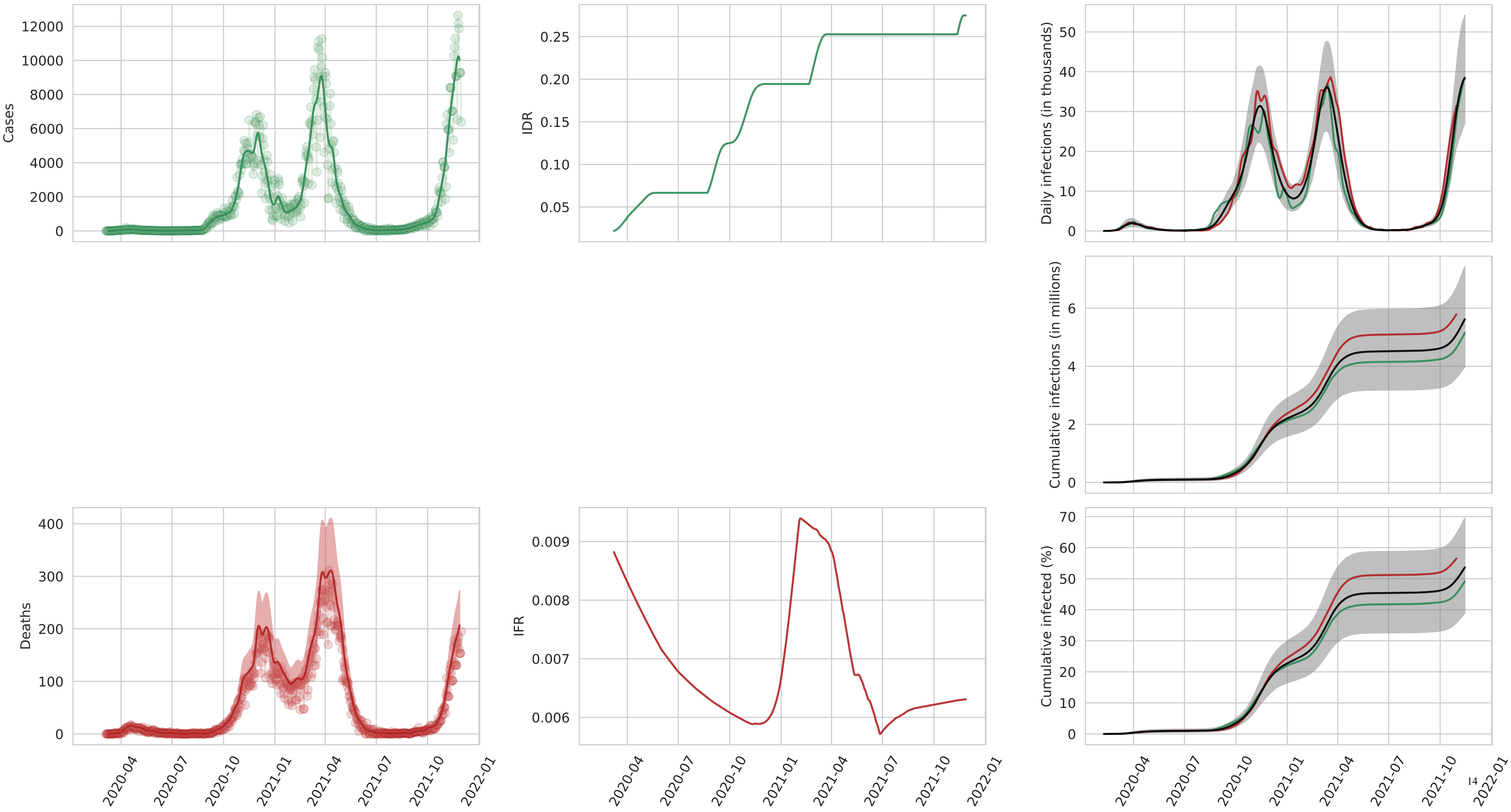

Cases
IDR
Daily infections (in thousands)
Cumulative infections (in millions)
Deaths
IFR
Cumulative infected (%)
2020-04
2020-07
2020-10
2021-01
2021-04
2021-07
2021-10
2022-01

Montenegro

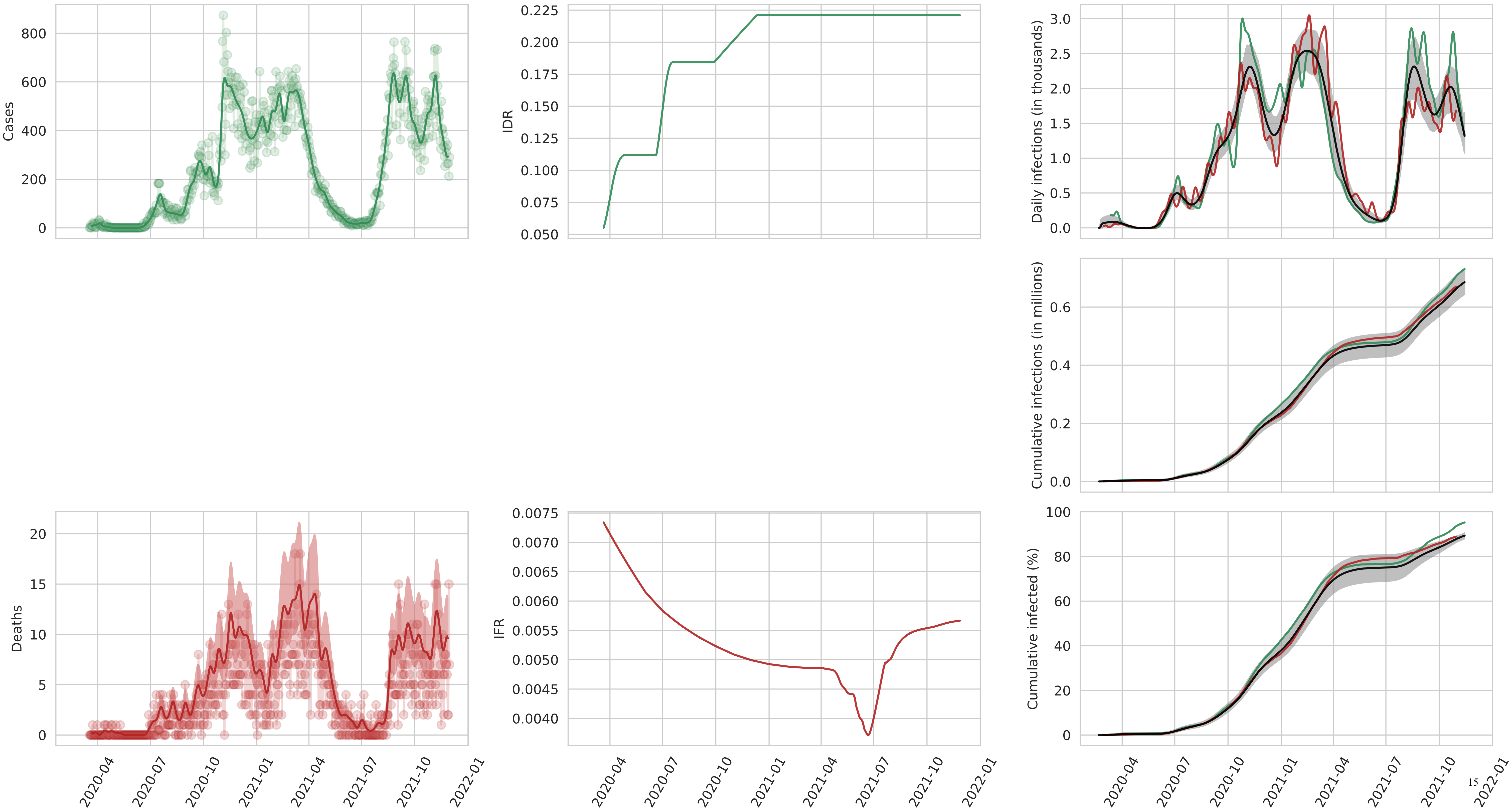

Cases
IDR
Daily infections (in thousands)
Cumulative infections (in millions)
Deaths
IFR
Cumulative infected (%)
2020-04
2020-07
2020-10
2021-01
2021-04
2021-07
2021-10
2022-01

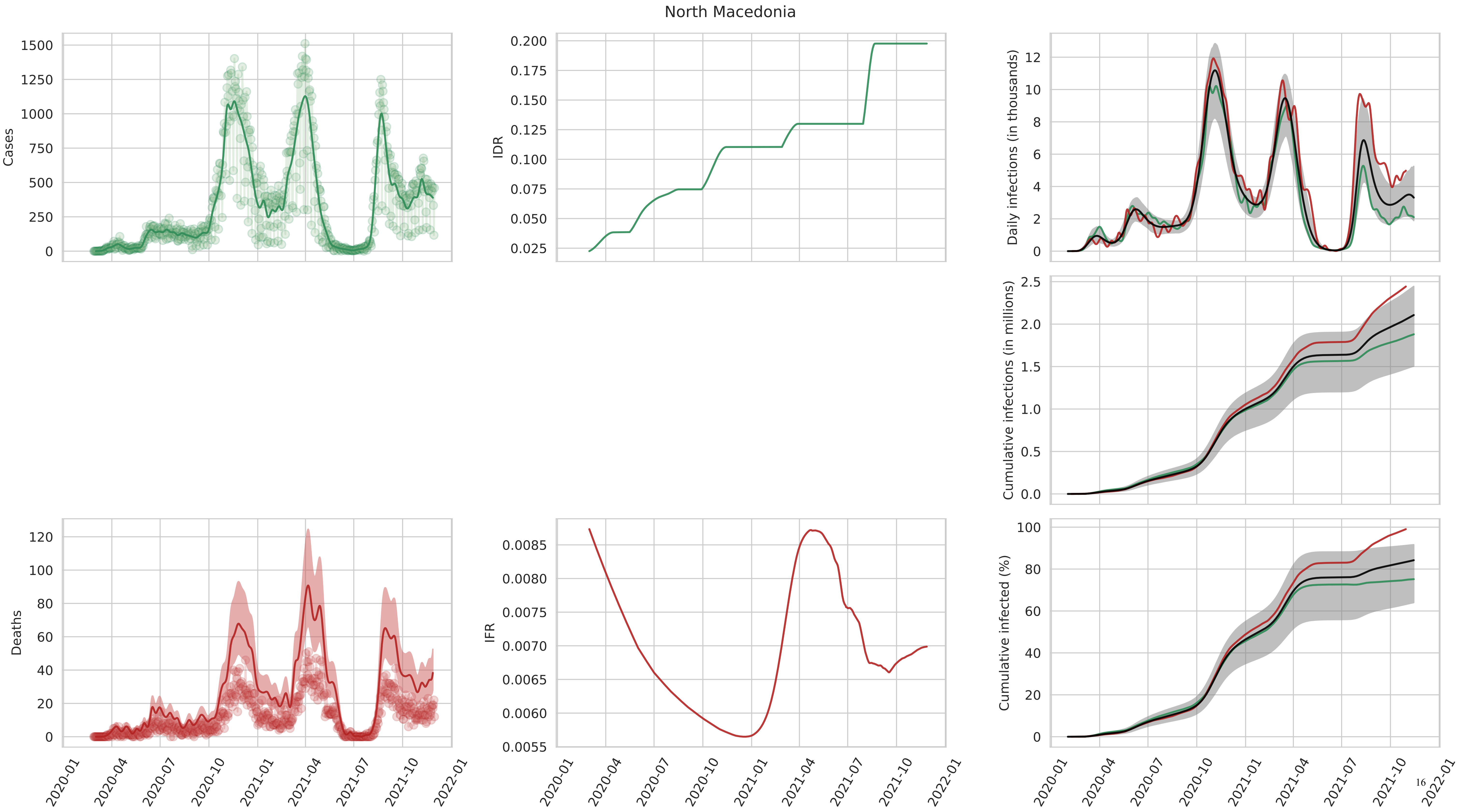
North Macedonia
Cases
IDR
Daily infections (in thousands)
Cumulative infections (in millions)
Deaths
IFR
Cumulative infected (%)
2020-01
2020-04
2020-07
2020-10
2021-01
2021-04
2021-07
2021-10
2022-01

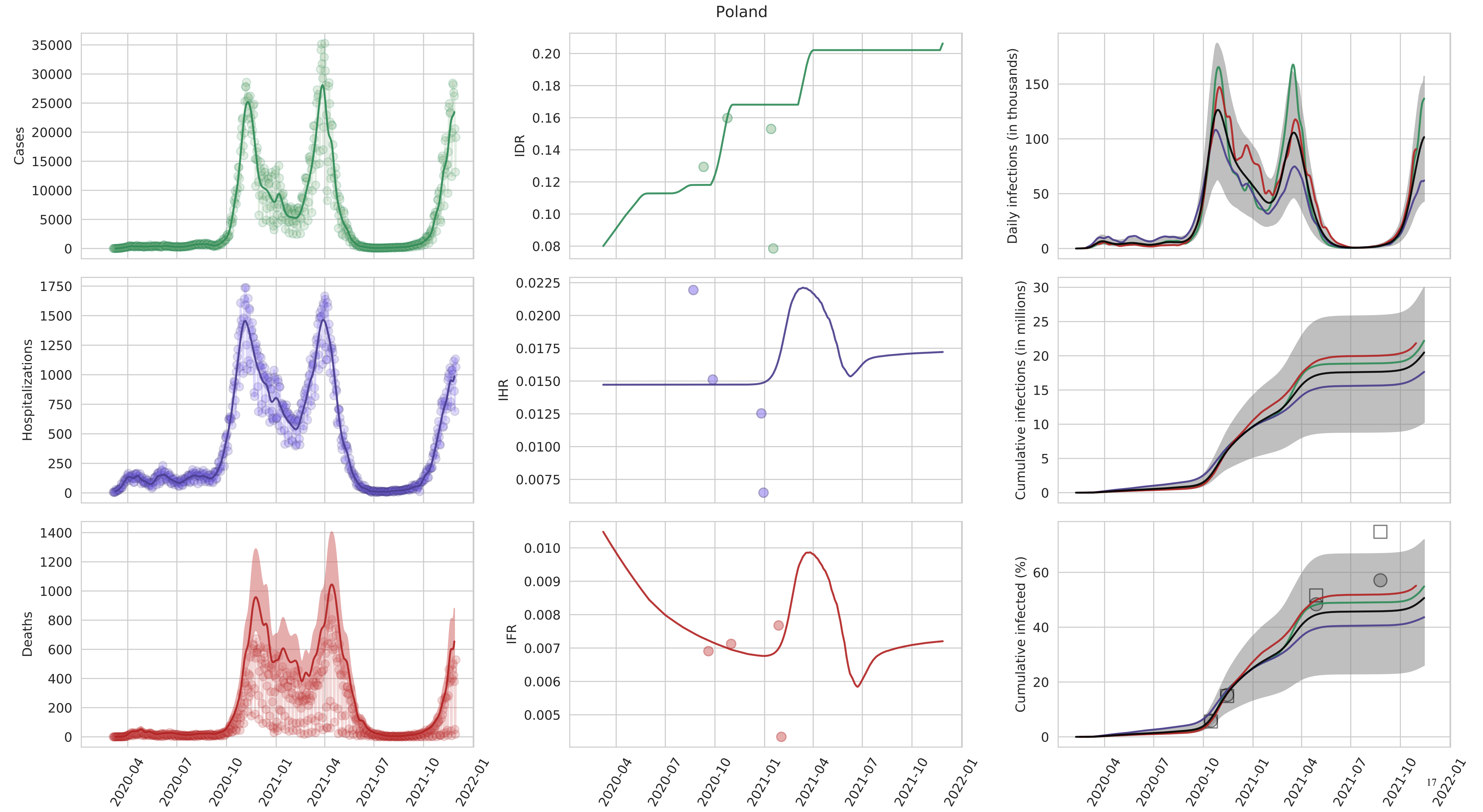

Poland
Cases
IDR
Daily infections (in thousands)
Hospitalizations
IHR
Cumulative infections (in millions)
Deaths
IFR
Cumulative infected (%)
2020-04
2020-07
2020-10
2021-01
2021-04
2021-07
2021-10
2022-01

Romania

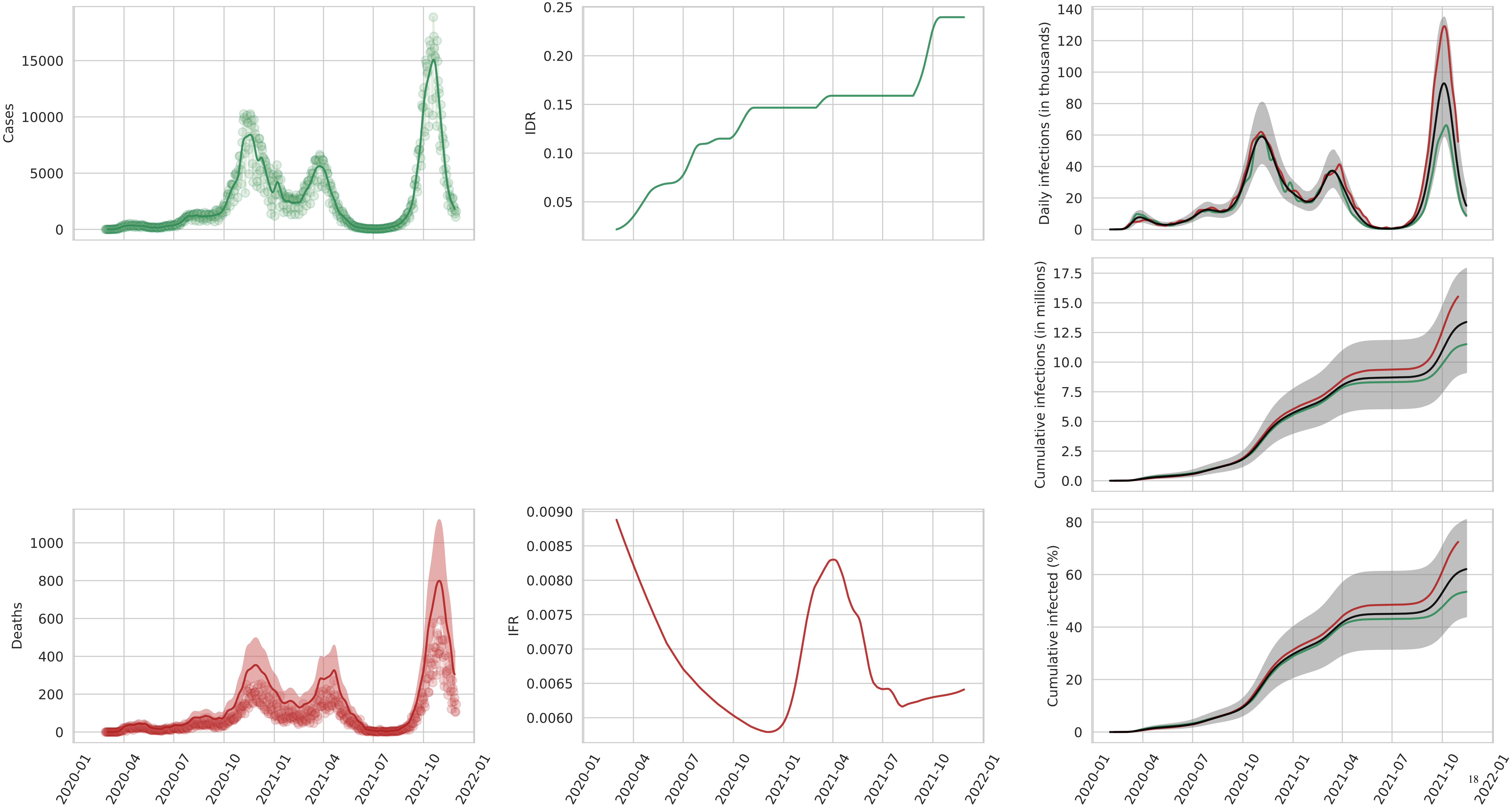

Cases
IDR
Daily infections (in thousands)
Cumulative infections (in millions)
Deaths
IFR
Cumulative infected (%)
2020-01
2020-04
2020-07
2020-10
2021-01
2021-04
2021-07
2021-10
2022-01

Serbia

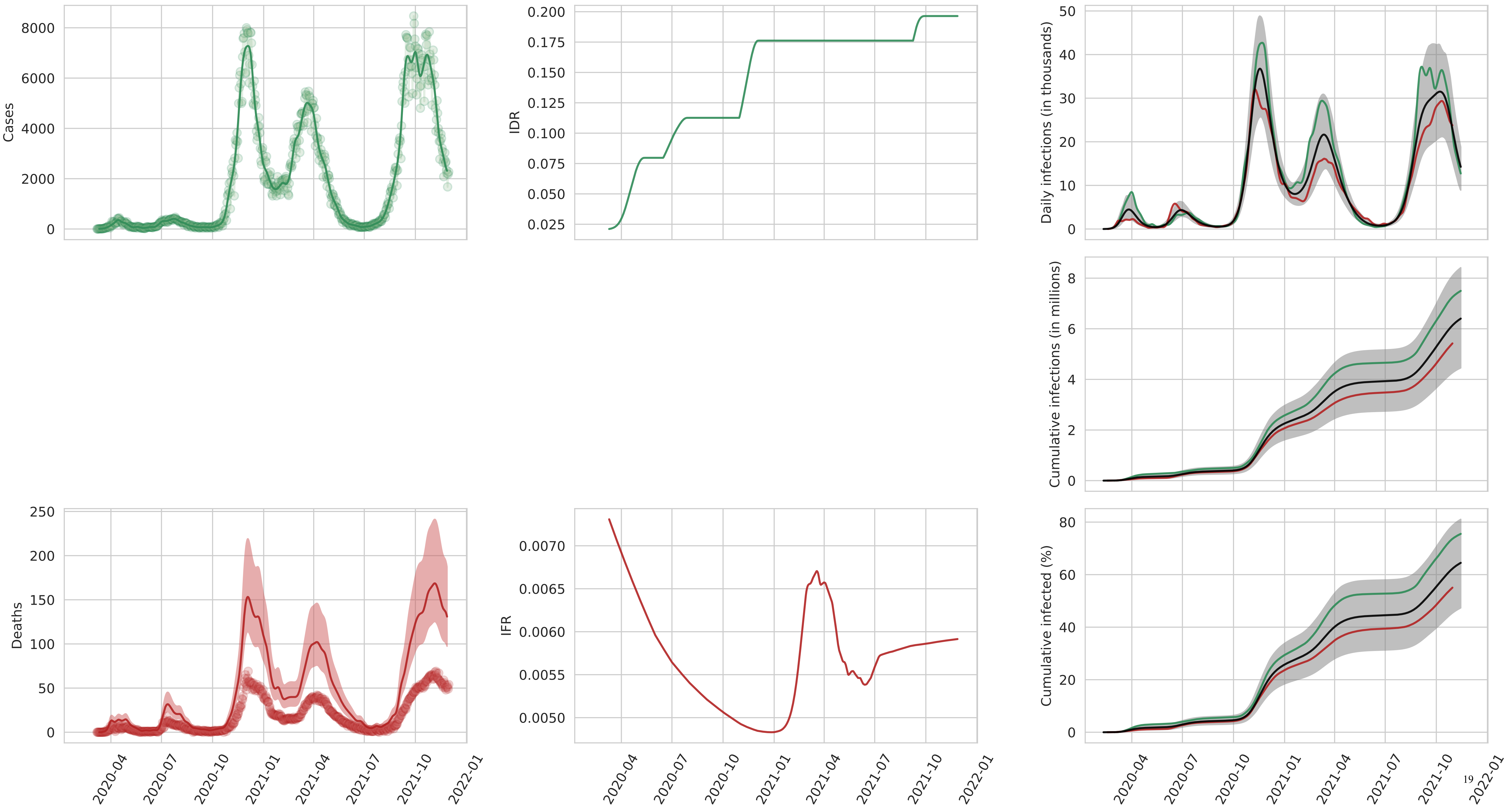

Cases
IDR
Daily infections (in thousands)
Cumulative infections (in millions)
Deaths
IFR
Cumulative infected (%)

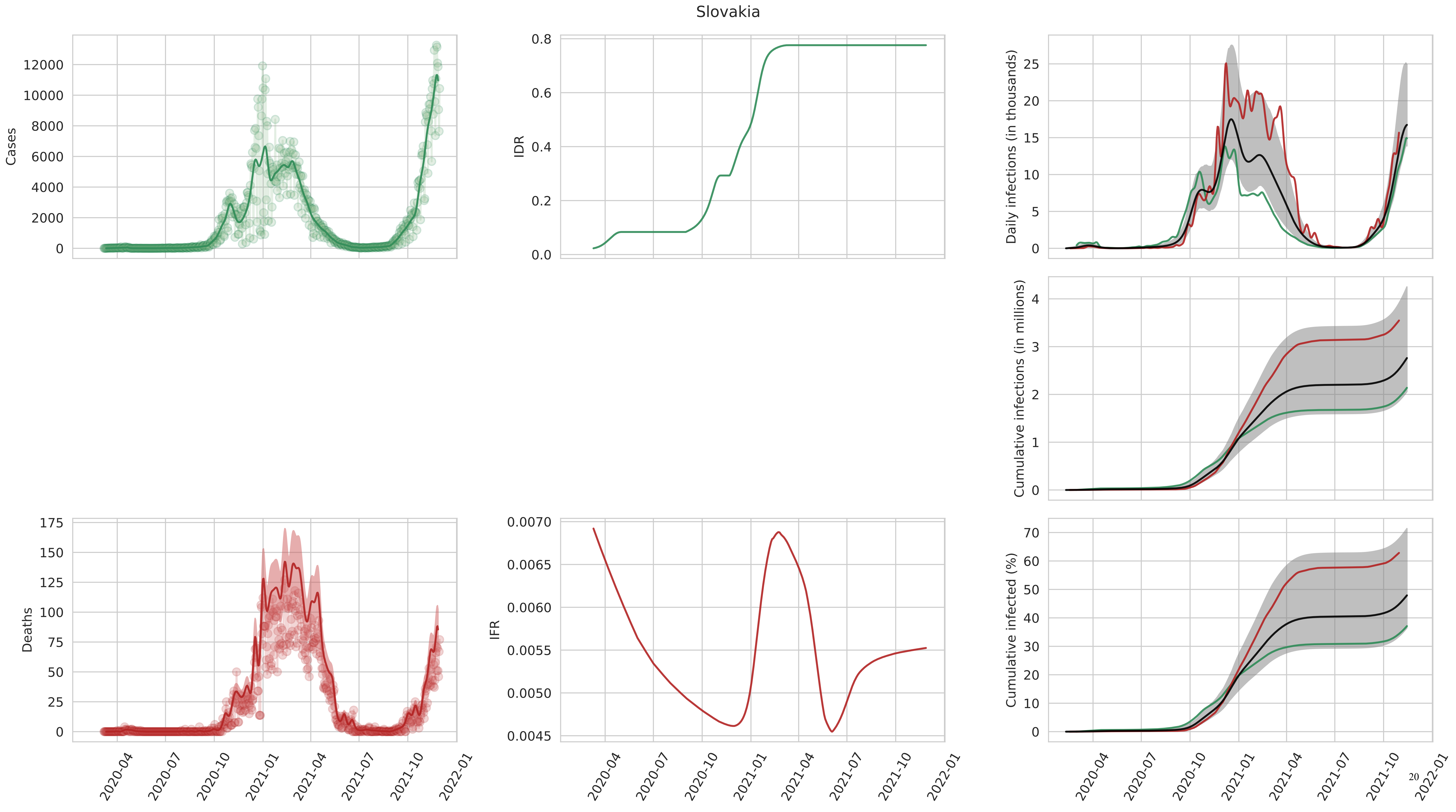

Slovakia
Cases
IDR
Daily infections (in thousands)
Cumulative infections (in millions)
Deaths
IFR
Cumulative infected (%)
2020-04
2020-07
2020-10
2021-01
2021-04
2021-07
2021-10
2022-01

Slovenia

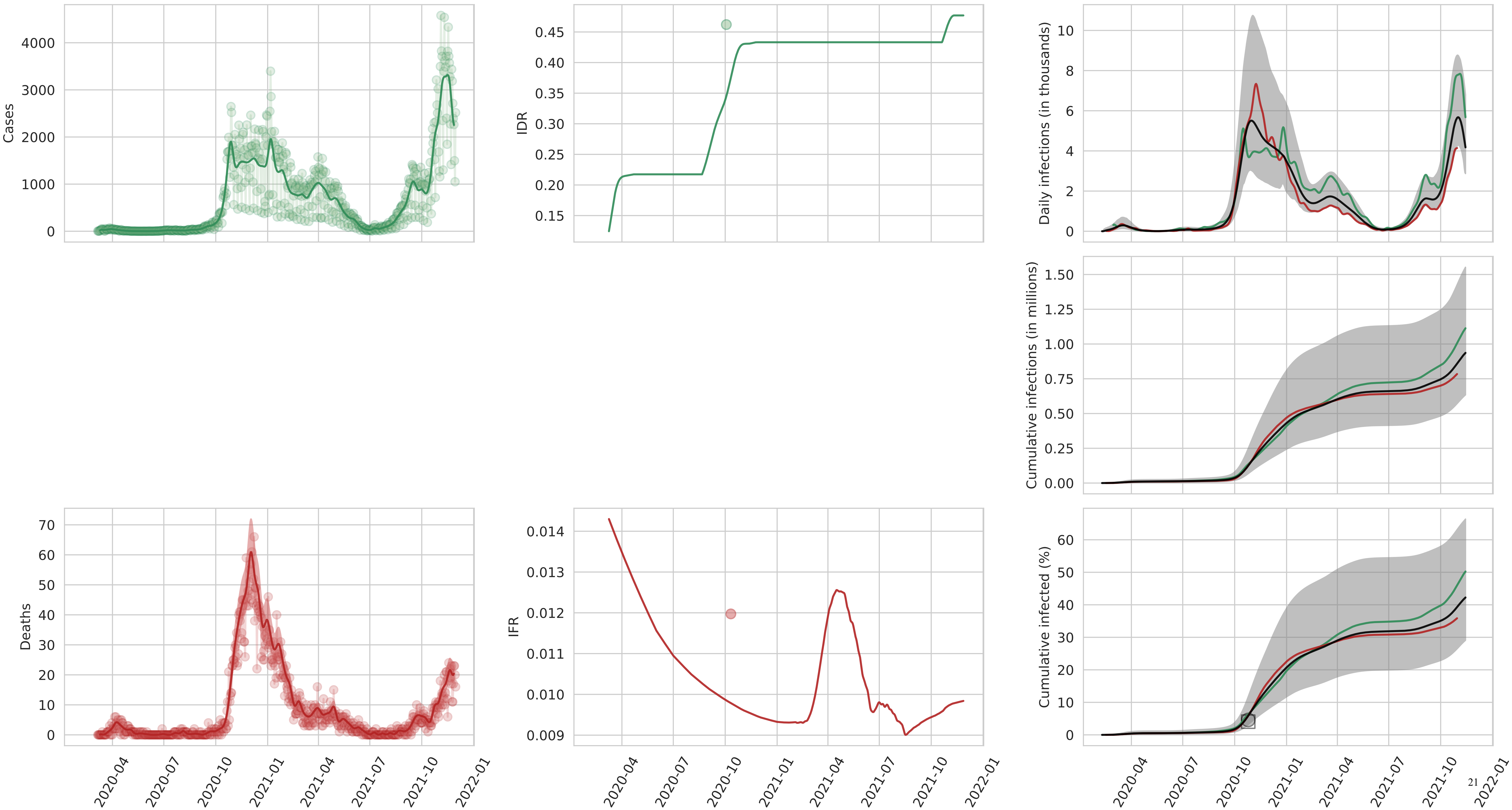

Cases
IDR
Daily infections (in thousands)
Cumulative infections (in millions)
Deaths
IFR
Cumulative infected (%)
2020-04
2020-07
2020-10
2021-01
2021-04
2021-07
2021-10
2022-01

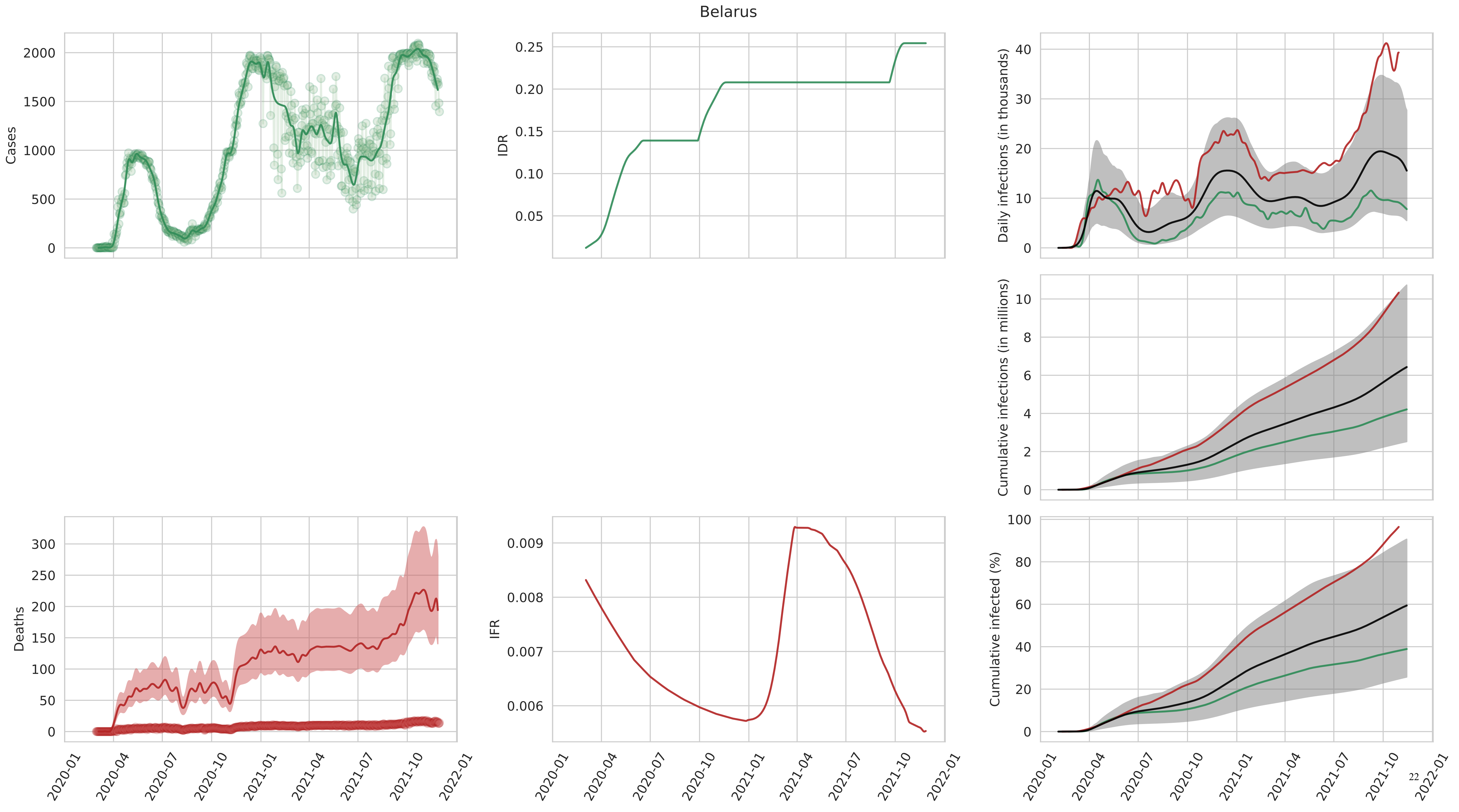
Belarus
Cases
IDR
Daily infections (in thousands)
Cumulative infections (in millions)
Deaths
IFR
Cumulative infected (%)
2020-01
2020-04
2020-07
2020-10
2021-01
2021-04
2021-07
2021-10
2022-01

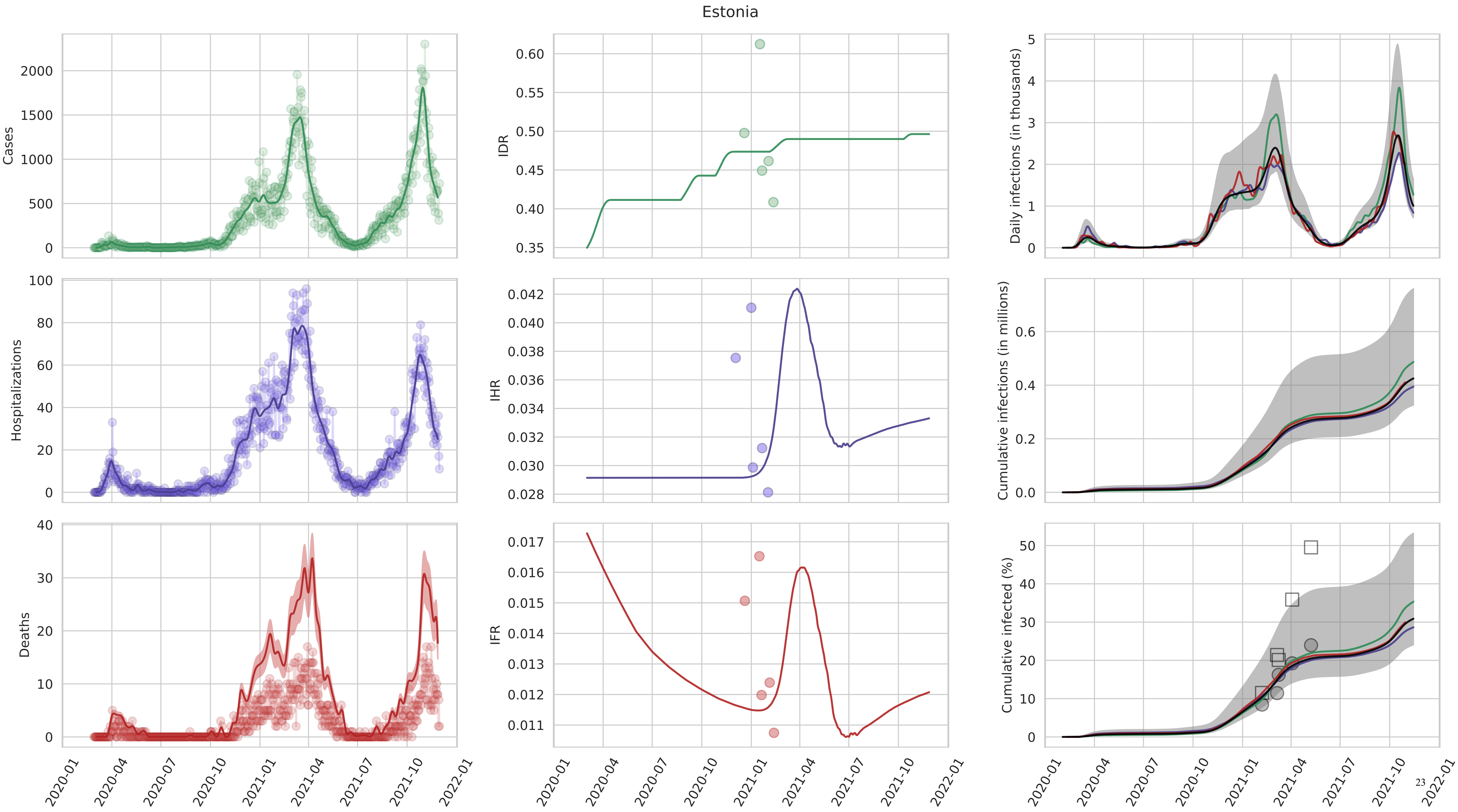

Estonia
Cases
IDR
Daily infections (in thousands)
Hospitalizations
IHR
Cumulative infections (in millions)
Deaths
IFR
Cumulative infected (%)
2020-01
2020-04
2020-07
2020-10
2021-01
2021-04
2021-07
2021-10
2022-01

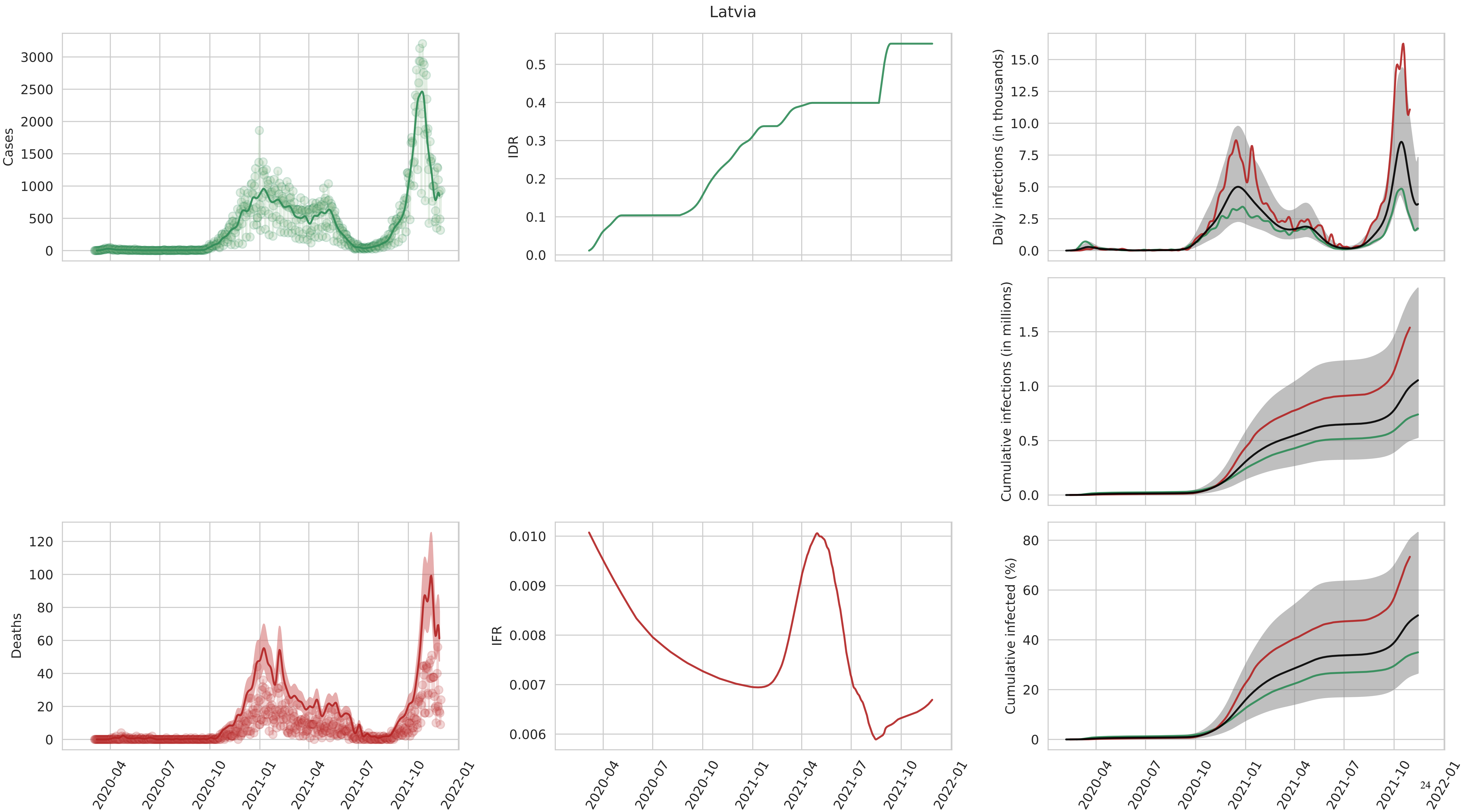
Latvia
Cases
IDR
Daily infections (in thousands)
Cumulative infections (in millions)
Deaths
IFR
Cumulative infected (%)

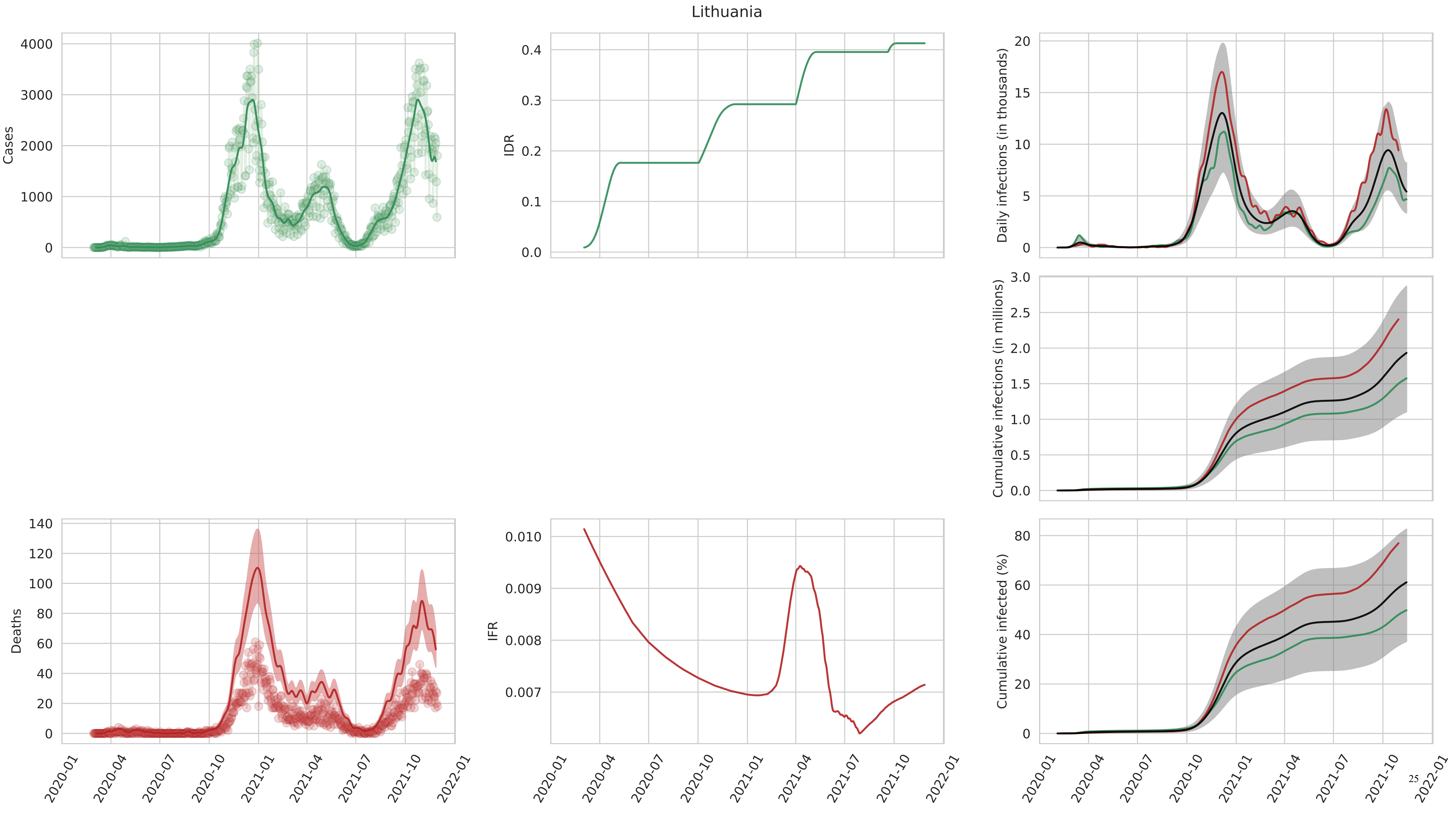
Lithuania
Cases
IDR
Daily infections (in thousands)
Cumulative infections (in millions)
Deaths
IFR
Cumulative infected (%)
2020-01
2020-04
2020-07
2020-10
2021-01
2021-04
2021-07
2021-10
2022-01

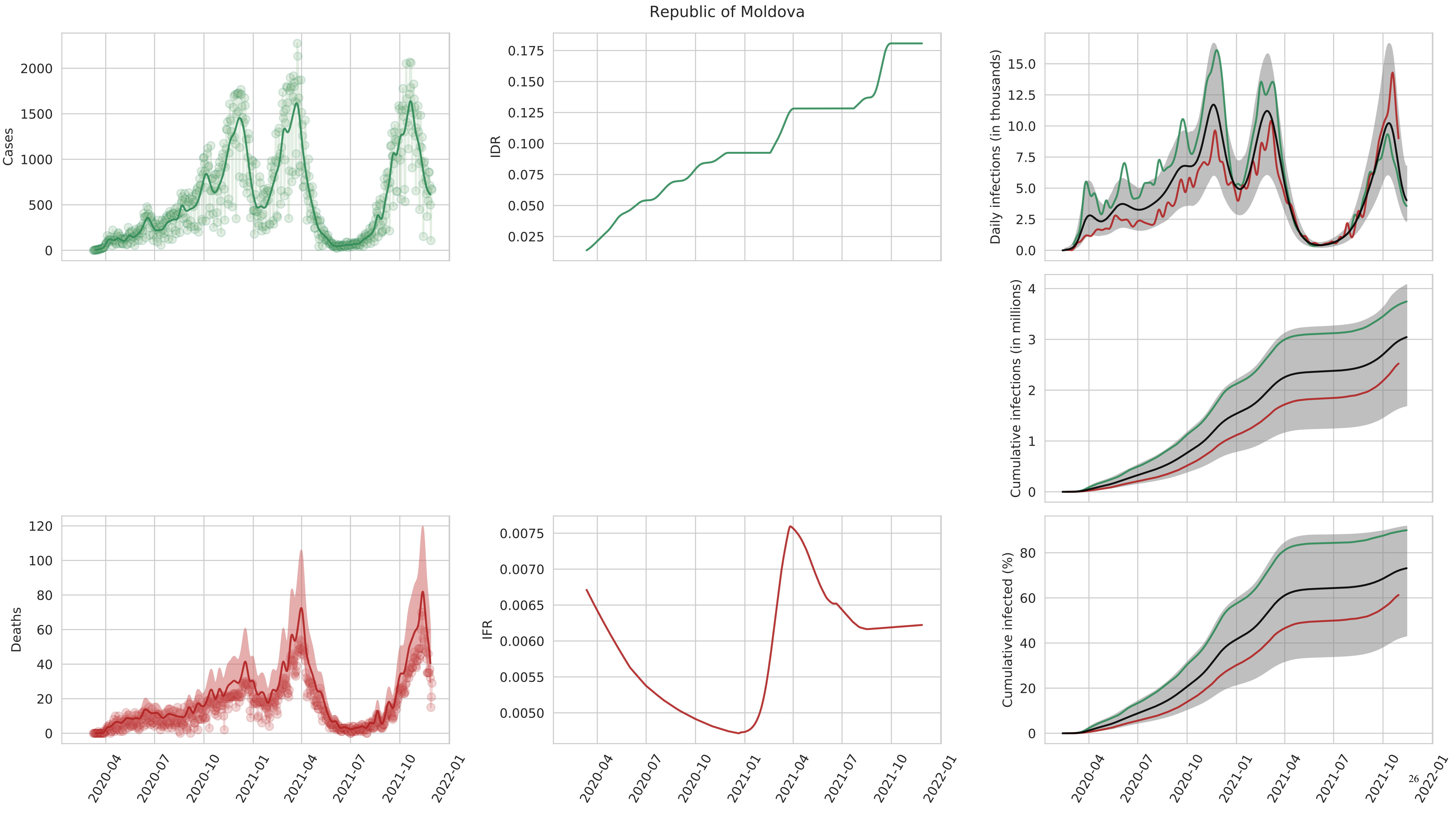
Republic of Moldova
Cases
IDR
Daily infections (in thousands)
Cumulative infections (in millions)
Deaths
IFR
Cumulative infected (%)
2020-04
2020-07
2020-10
2021-01
2021-04
2021-07
2021-10
2022-01

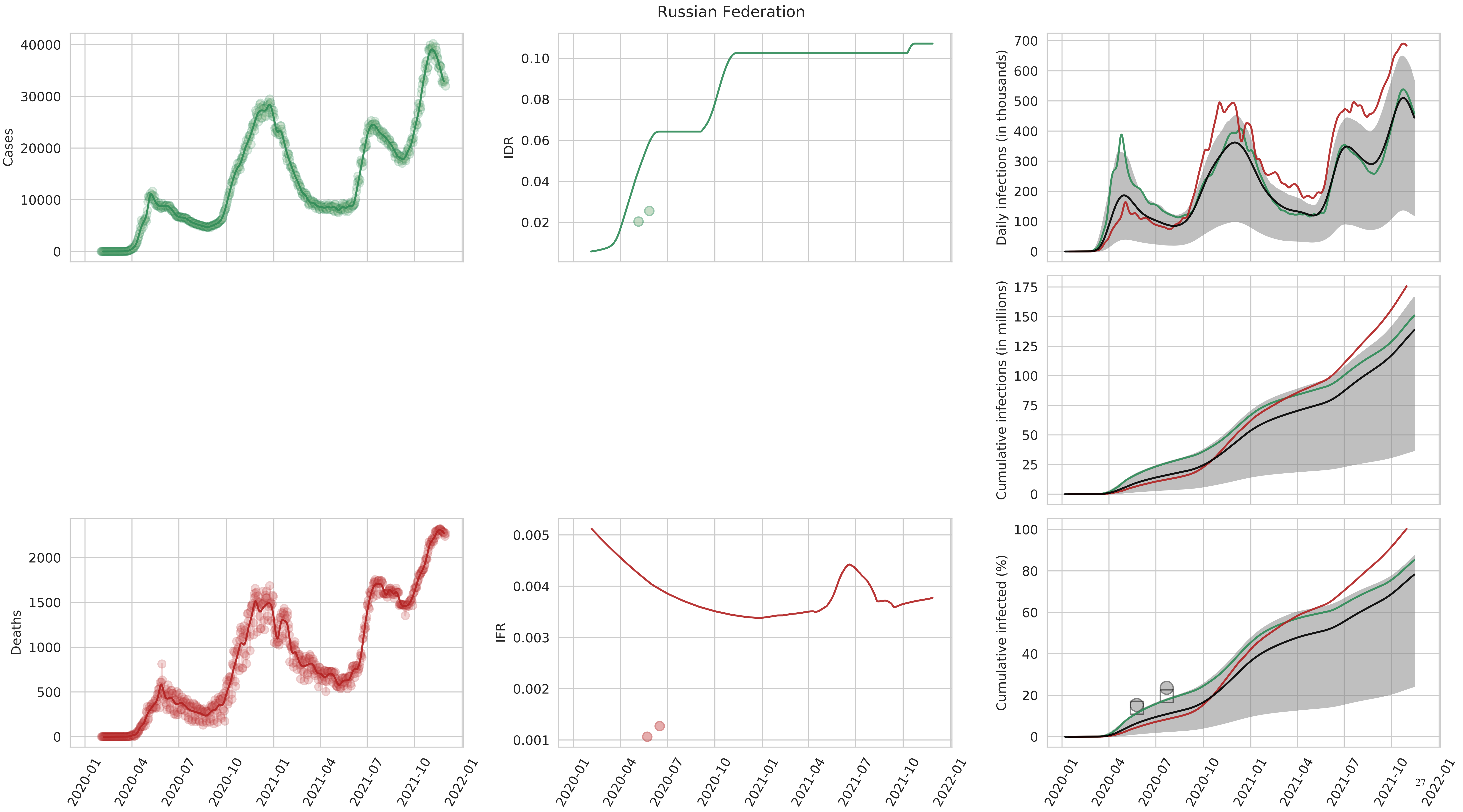
Russian Federation
Cases
Deaths
IDR
IFR
Daily infections (in thousands)
Cumulative infections (in millions)
Cumulative infected (%)
2020-01
2020-04
2020-07
2020-10
2021-01
2021-04
2021-07
2021-10
2022-01

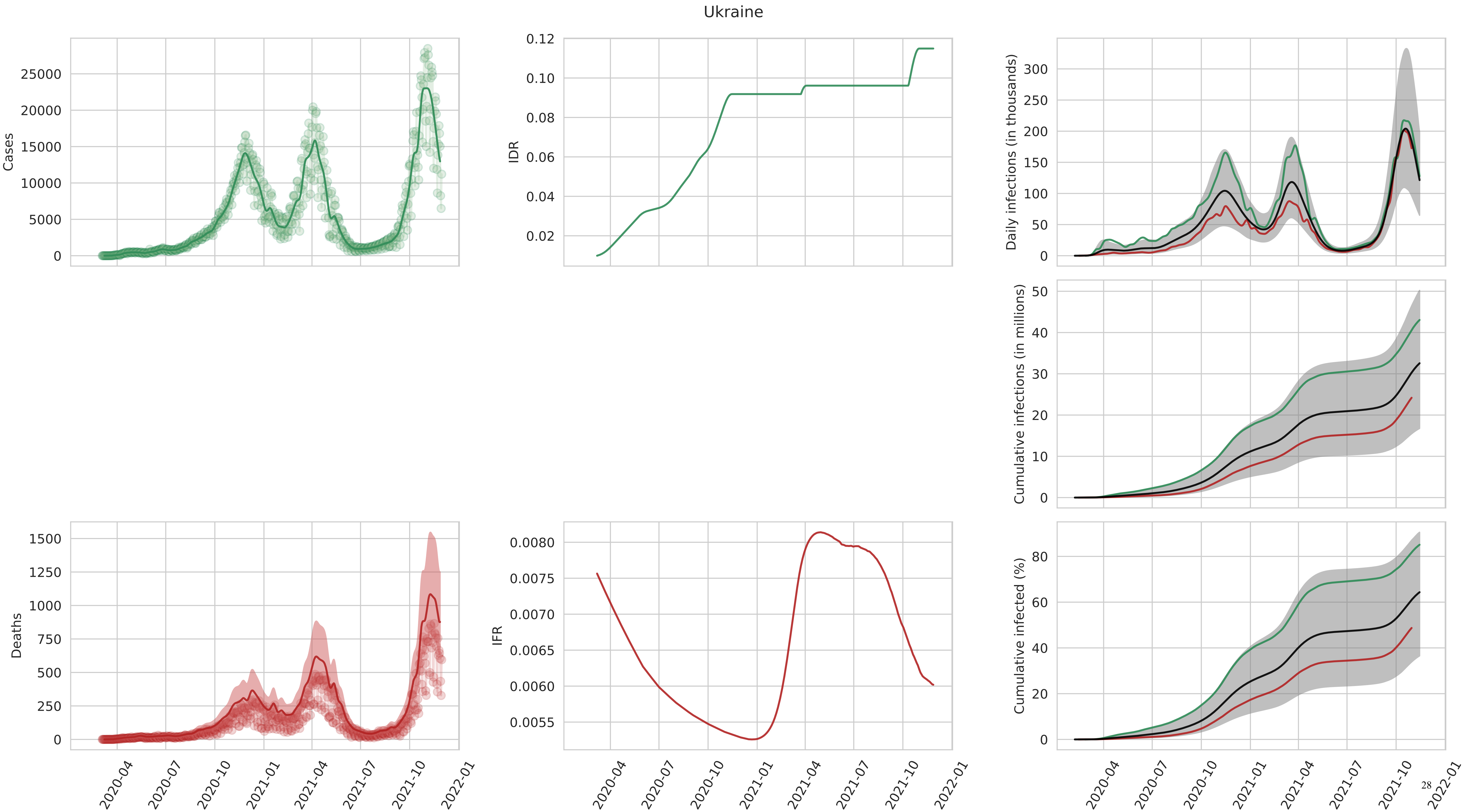
Ukraine
Cases
IDR
Daily infections (in thousands)
Cumulative infections (in millions)
Deaths
IFR
Cumulative infected (%)
2020-04
2020-07
2020-10
2021-01
2021-04
2021-07
2021-10
2022-01

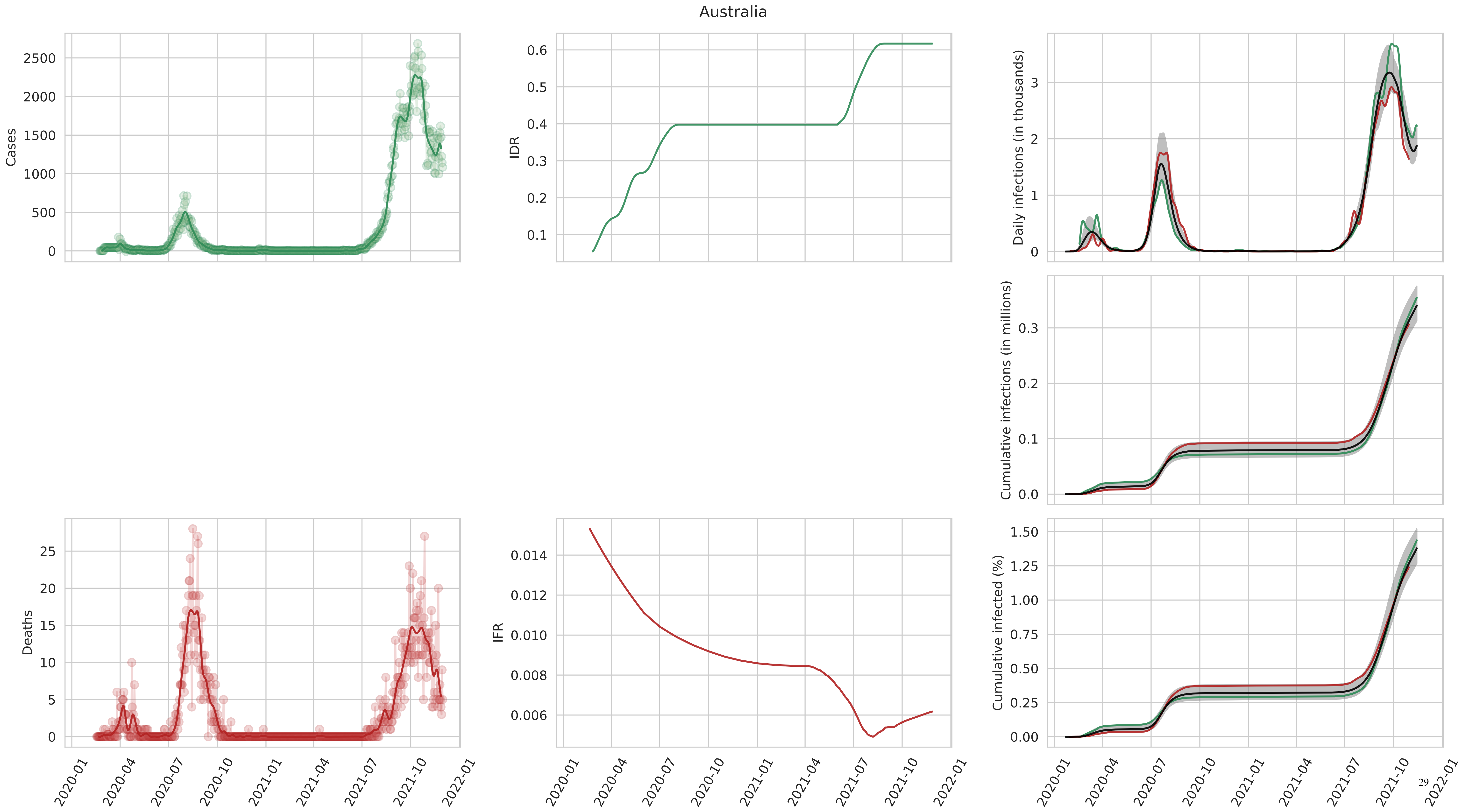
Australia
Cases
IDR
Daily infections (in thousands)
Cumulative infections (in millions)
Deaths
IFR
Cumulative infected (%)
2020-01
2020-04
2020-07
2020-10
2021-01
2021-04
2021-07
2021-10
2022-01

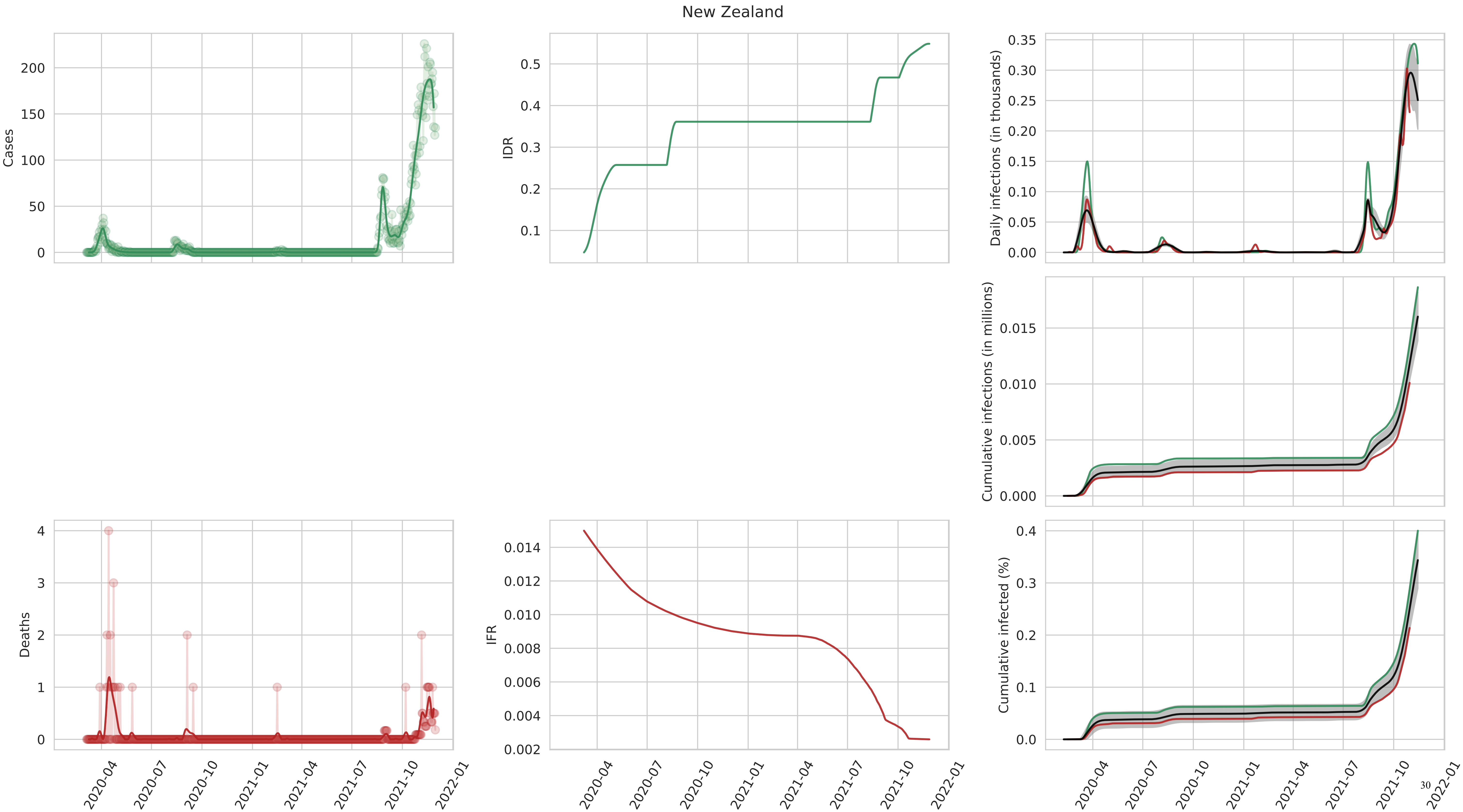

New Zealand
Cases
IDR
Daily infections (in thousands)
Cumulative infections (in millions)
Deaths
IFR
Cumulative infected (%)
2020-04
2020-07
2020-10
2021-01
2021-04
2021-07
2021-10
2022-01

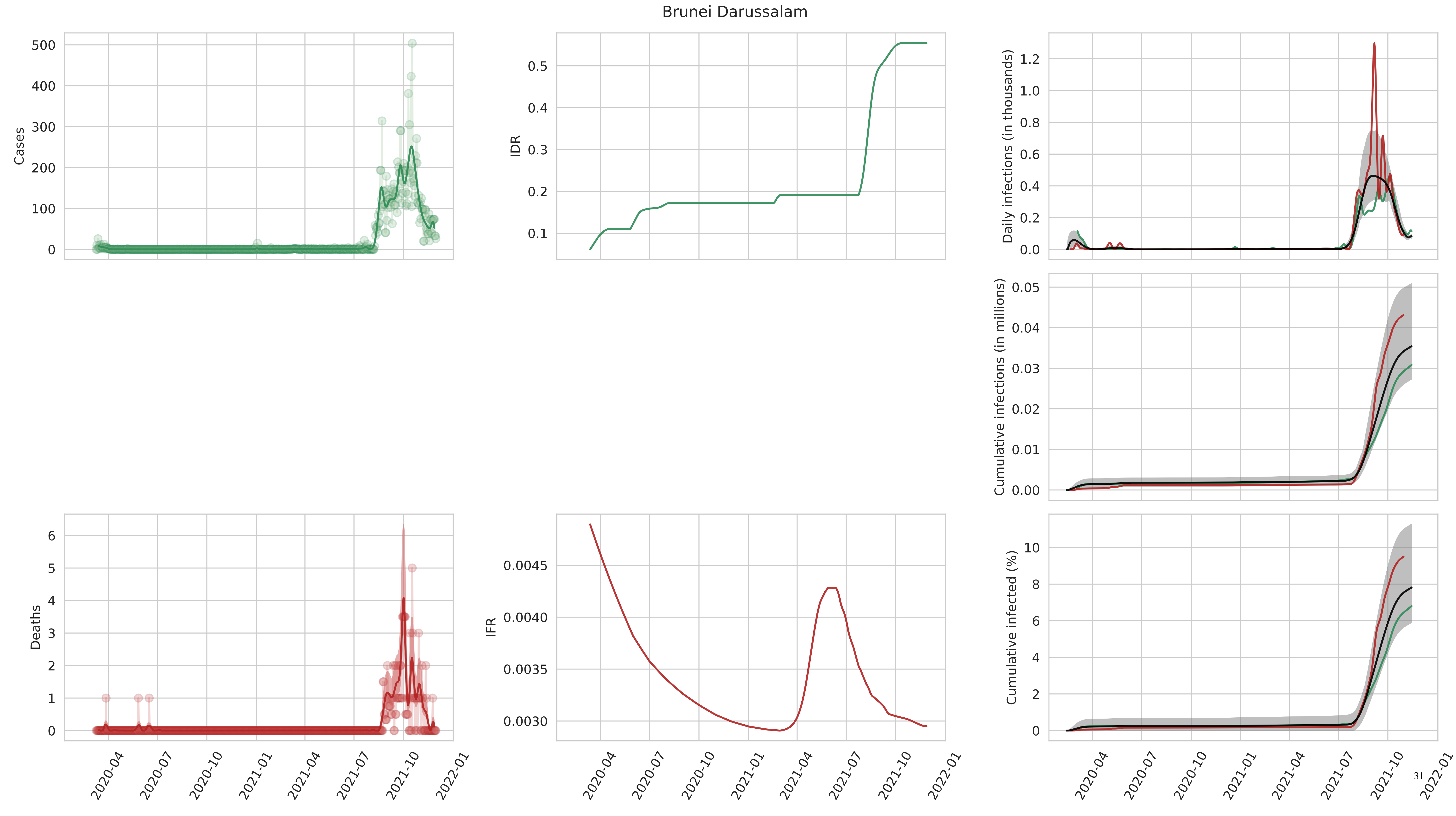
Brunei Darussalam
Cases
IDR
Daily infections (in thousands)
Cumulative infections (in millions)
Deaths
IFR
Cumulative infected (%)
2020-04
2020-07
2020-10
2021-01
2021-04
2021-07
2021-10
2022-01

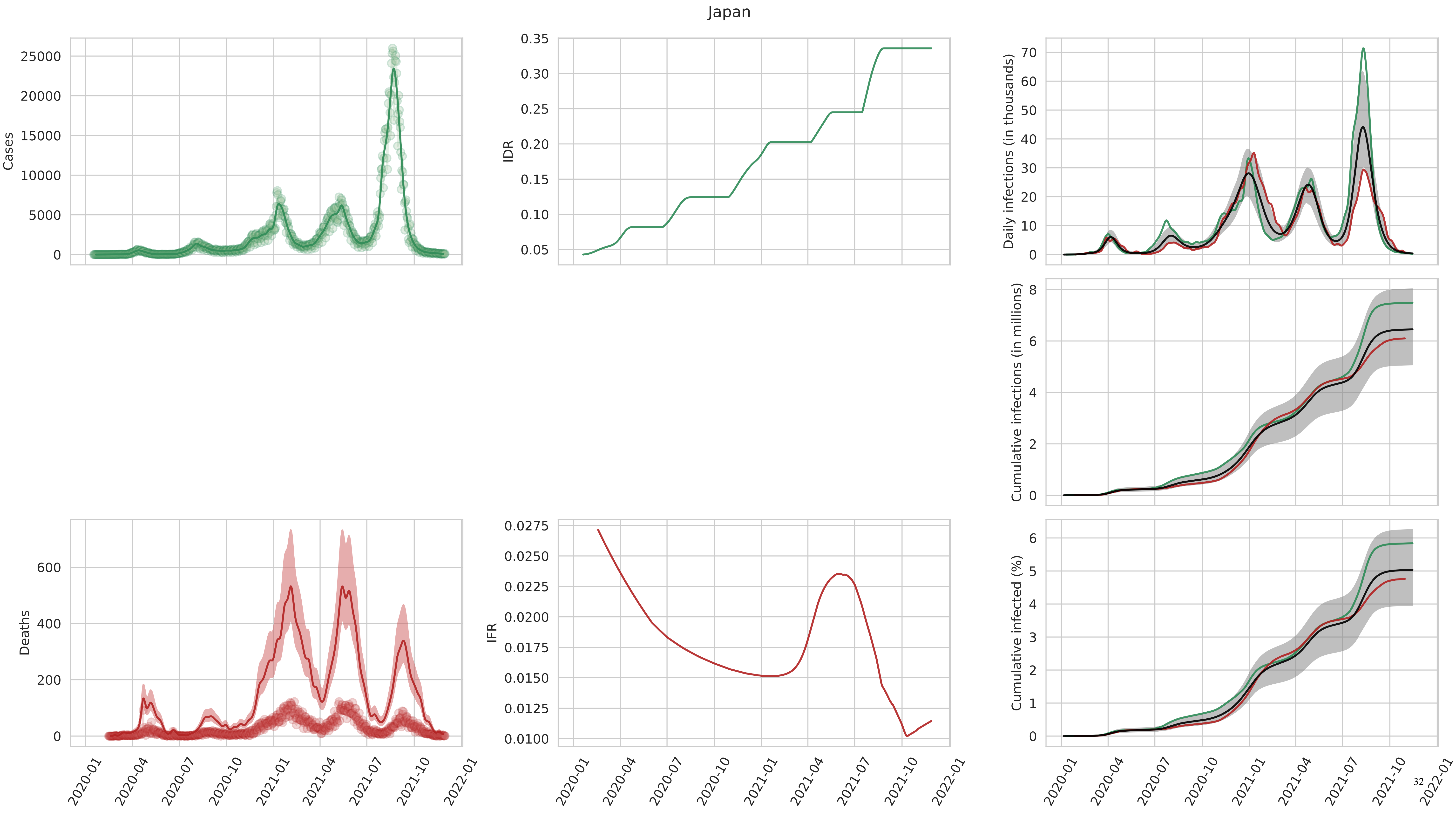
Japan
Cases
IDR
Daily infections (in thousands)
Cumulative infections (in millions)
Deaths
IFR
Cumulative infected (%)
2020-01
2020-04
2020-07
2020-10
2021-01
2021-04
2021-07
2021-10
2022-01

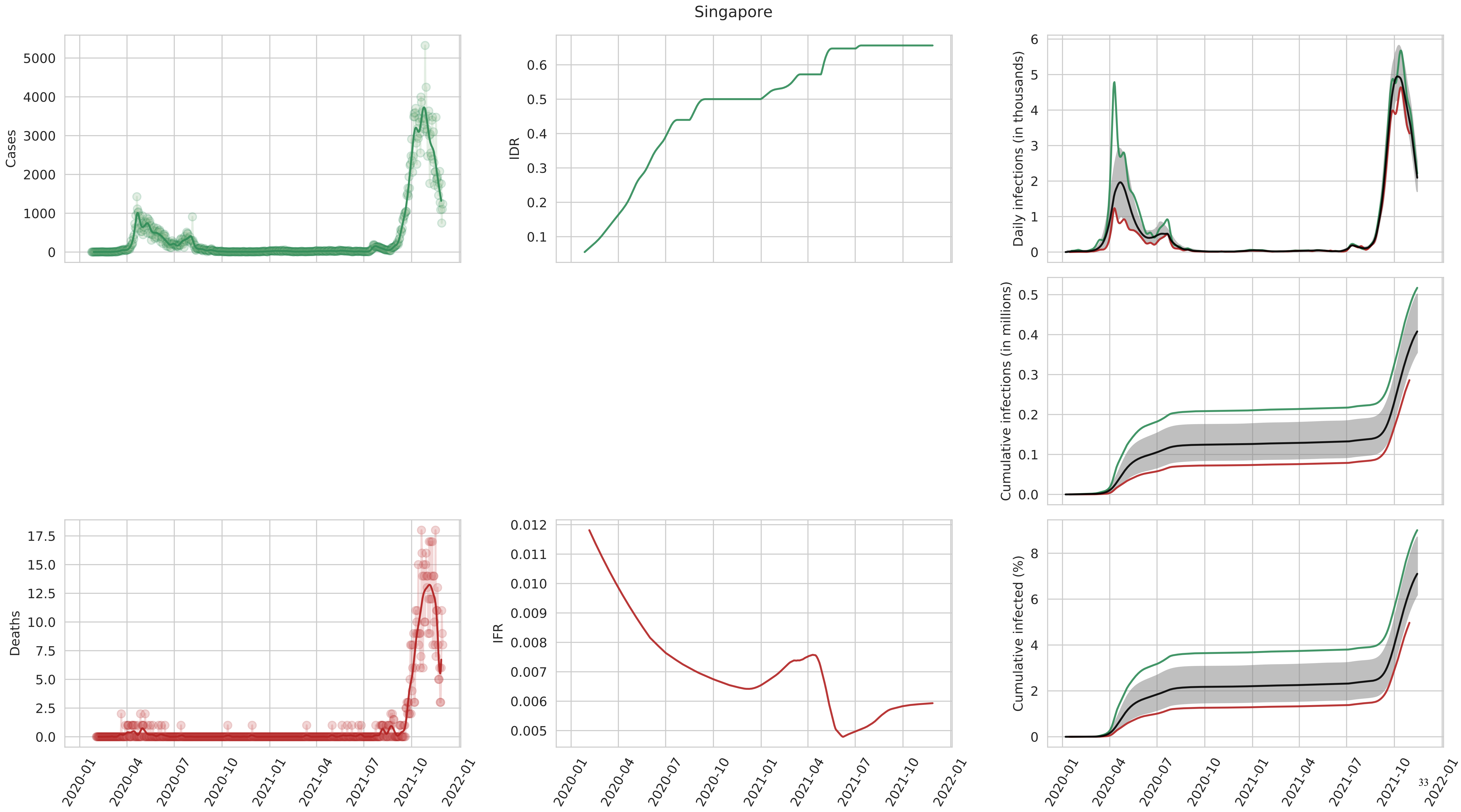
Singapore
Cases
IDR
Daily infections (in thousands)
Cumulative infections (in millions)
Deaths
IFR
Cumulative infected (%)
2020-01
2020-04
2020-07
2020-10
2021-01
2021-04
2021-07
2021-10
2022-01

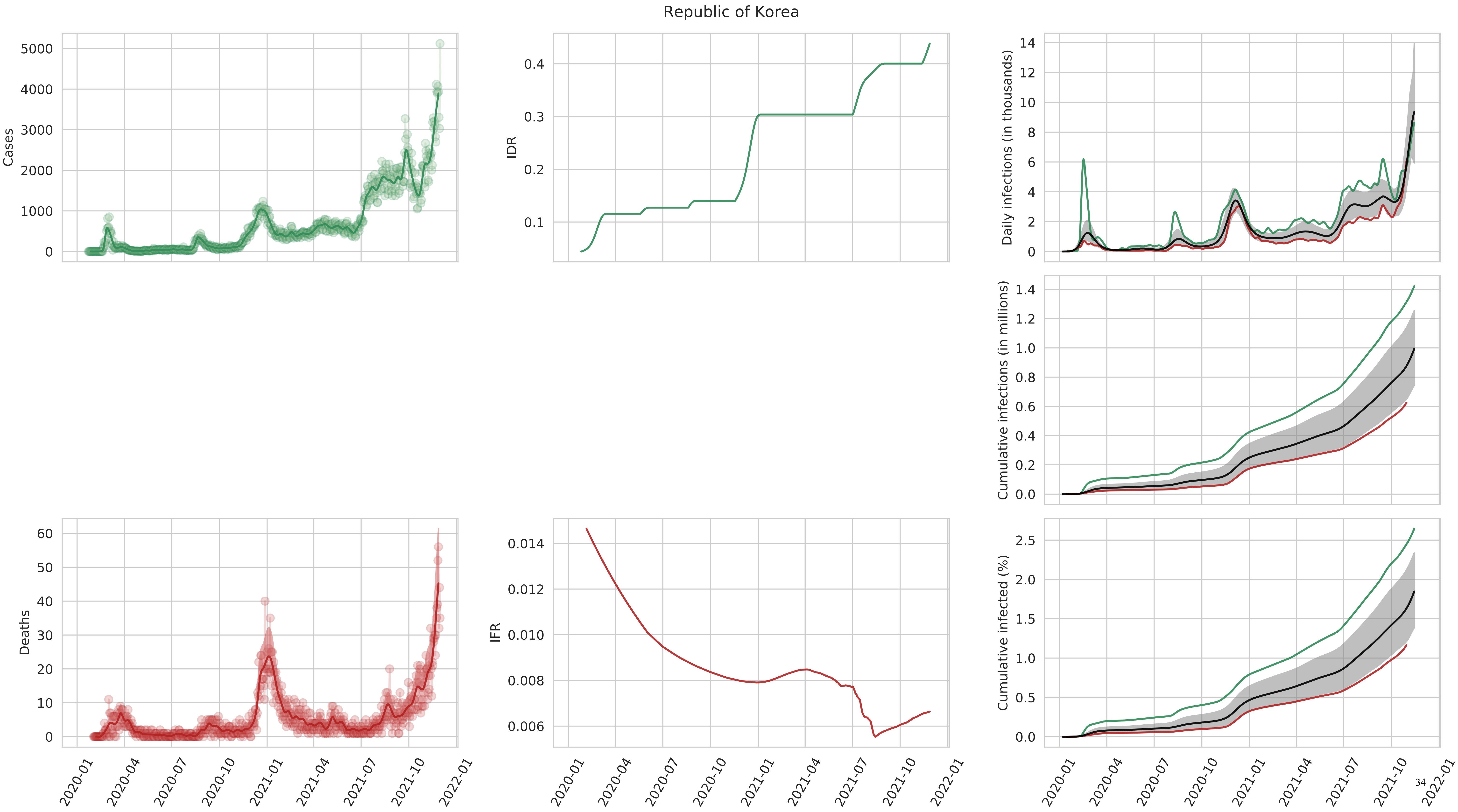
Republic of Korea
Cases
IDR
Daily infections (in thousands)
Cumulative infections (in millions)
Deaths
IFR
Cumulative infected (%)
2020-01
2020-04
2020-07
2020-10
2021-01
2021-04
2021-07
2021-10
2022-01

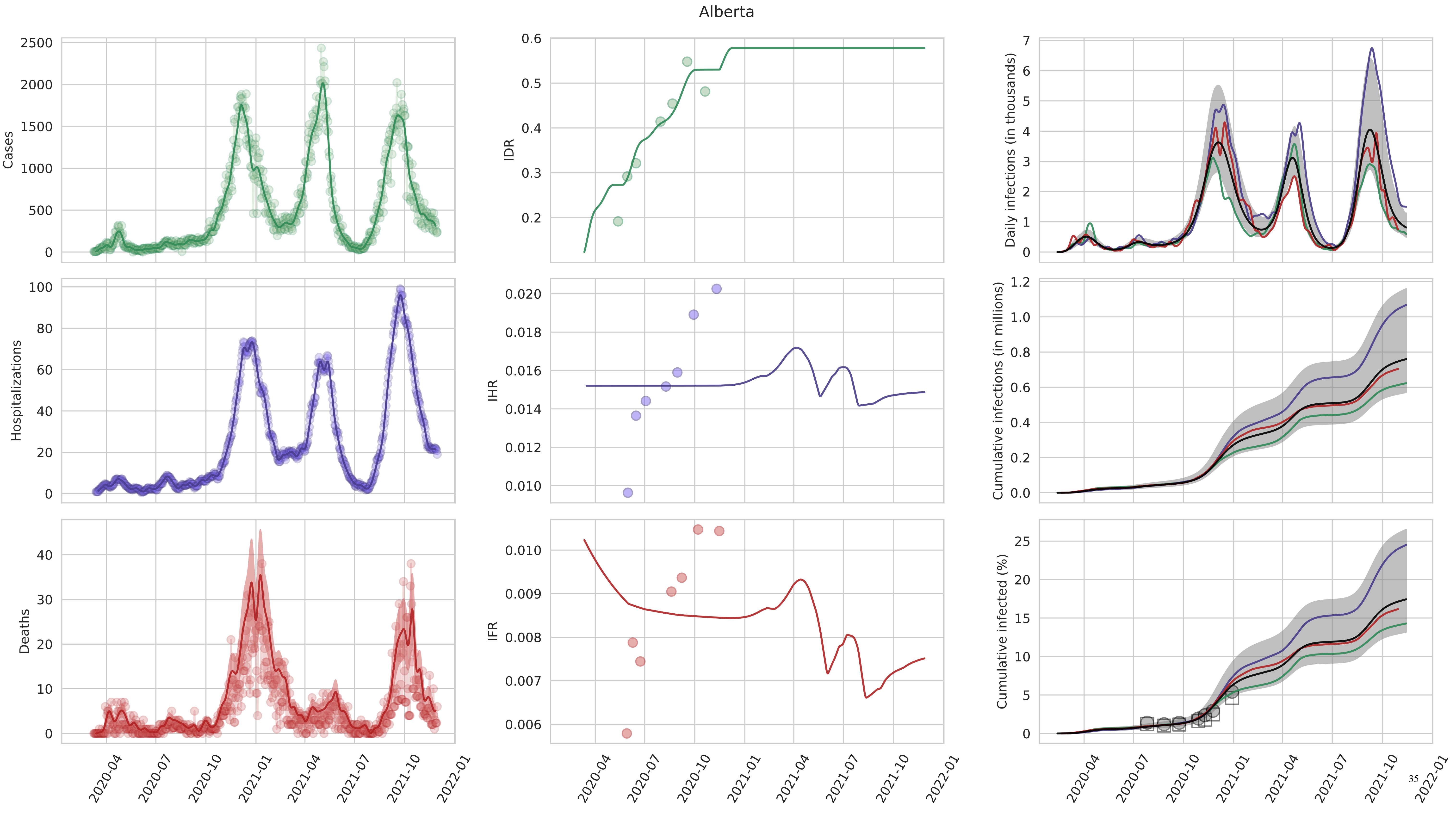
Alberta
Cases
IDR
Daily infections (in thousands)
Hospitalizations
IHR
Cumulative infections (in millions)
Deaths
IFR
Cumulative infected (%)

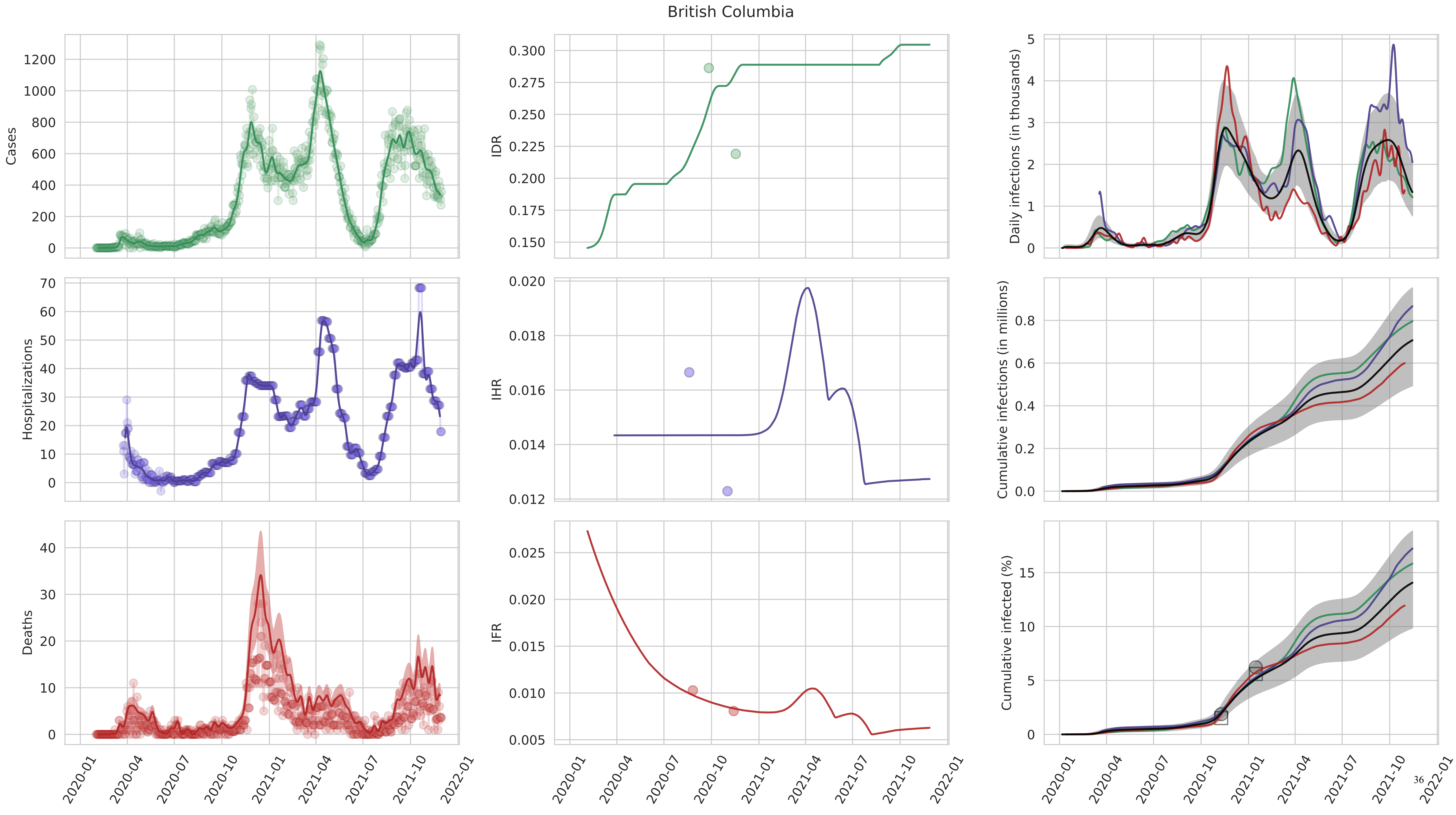
British Columbia
Cases
Hospitalizations
Deaths
IDR
IHR
IFR
Daily infections (in thousands)
Cumulative infections (in millions)
Cumulative infected (%)
2020-01
2020-04
2020-07
2020-10
2021-01
2021-04
2021-07
2021-10
2022-01

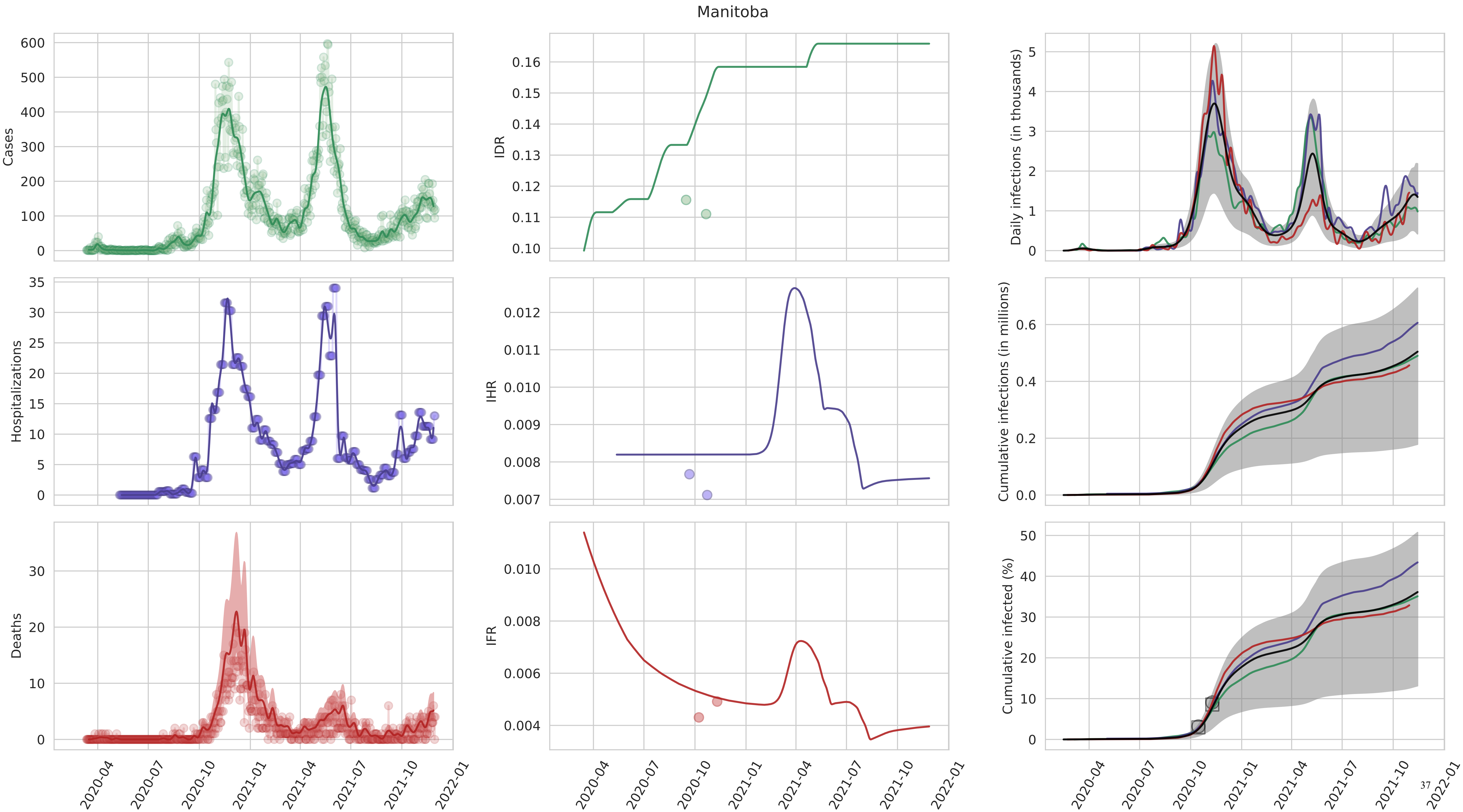
Manitoba
Cases
IDR
Daily infections (in thousands)
Hospitalizations
IHR
Cumulative infections (in millions)
Deaths
IFR
Cumulative infected (%)
2020-04
2020-07
2020-10
2021-01
2021-04
2021-07
2021-10
2022-01

New Brunswick

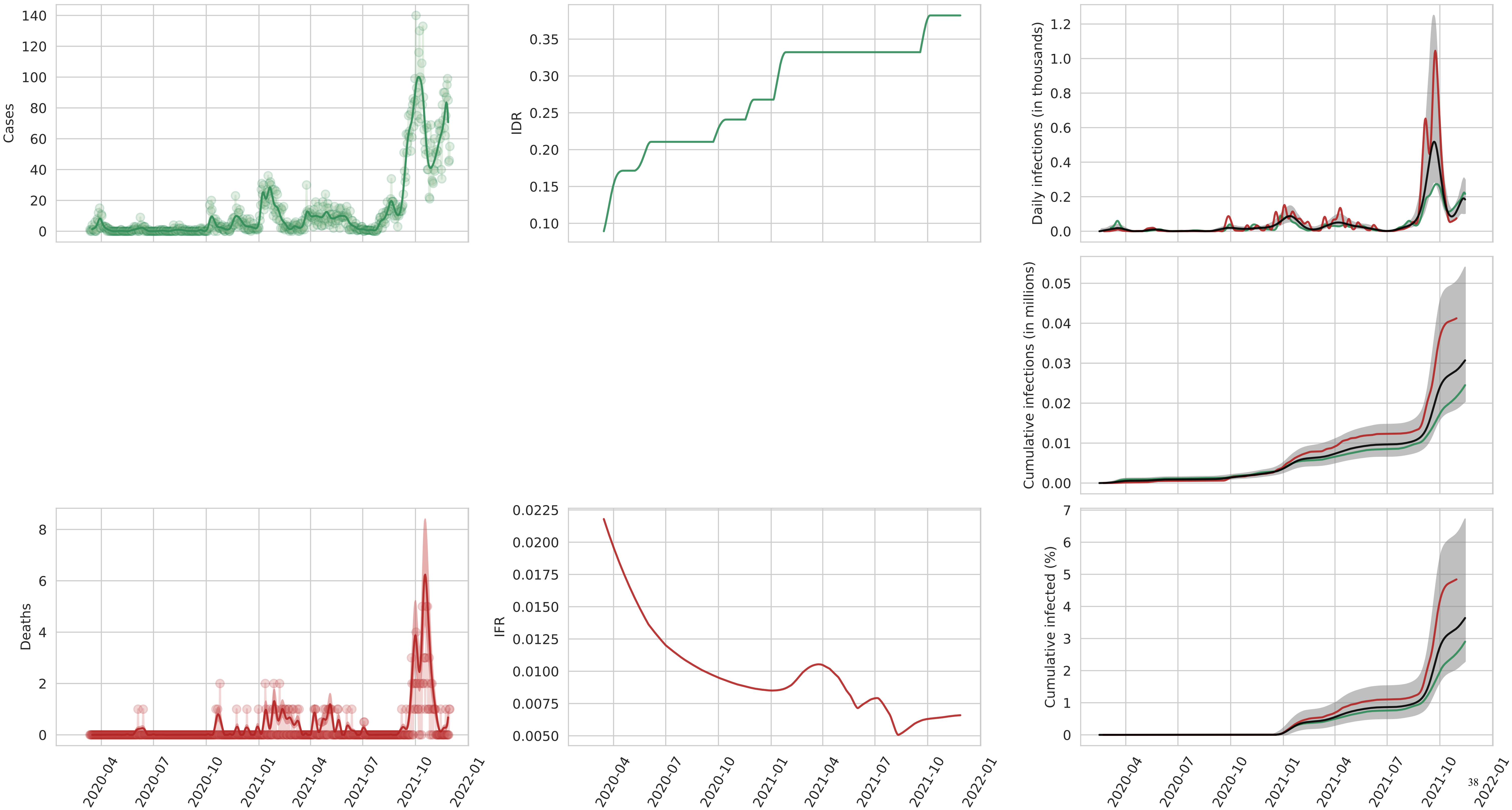

Newfoundland and Labrador

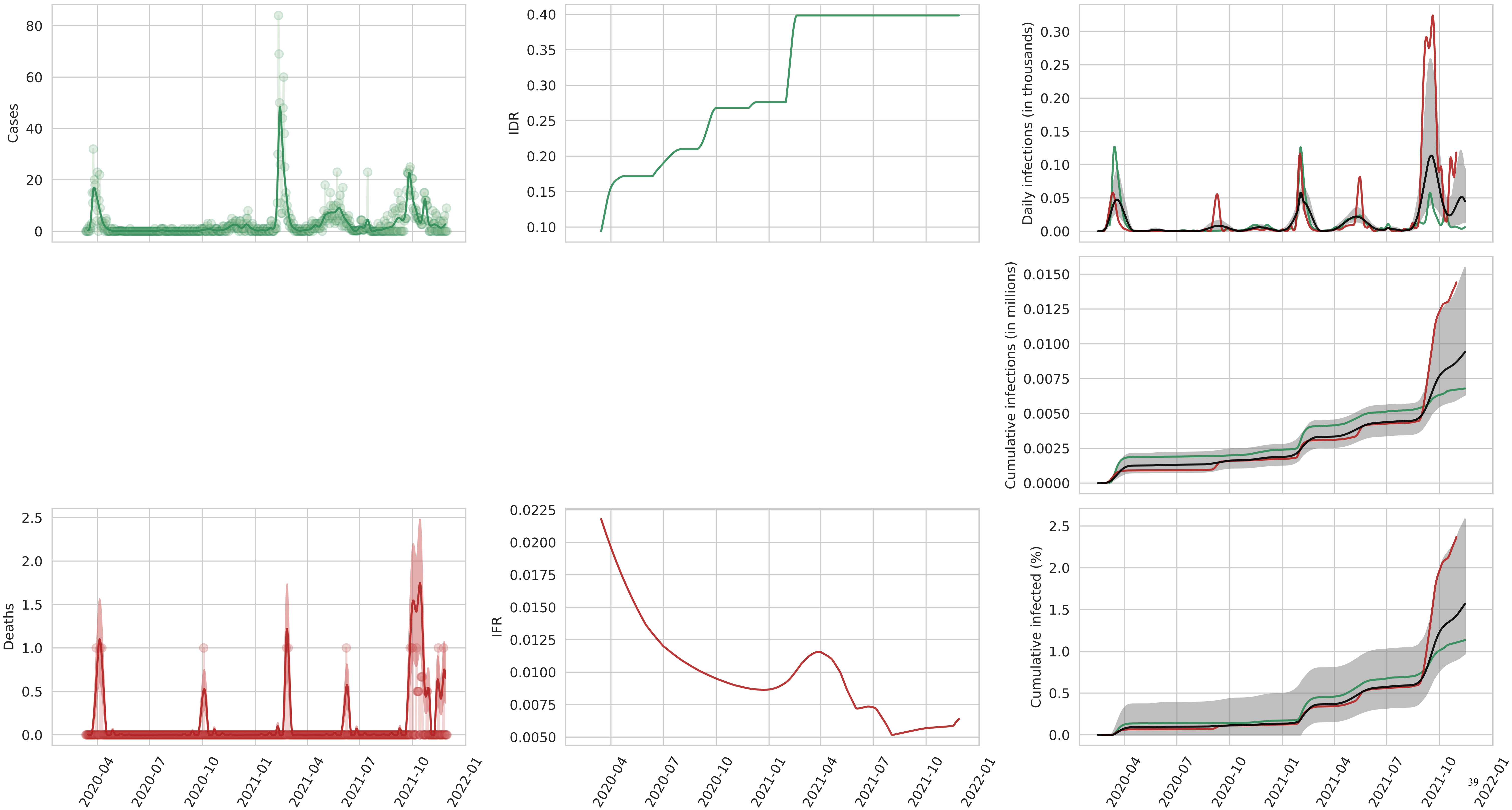

Northwest Territories

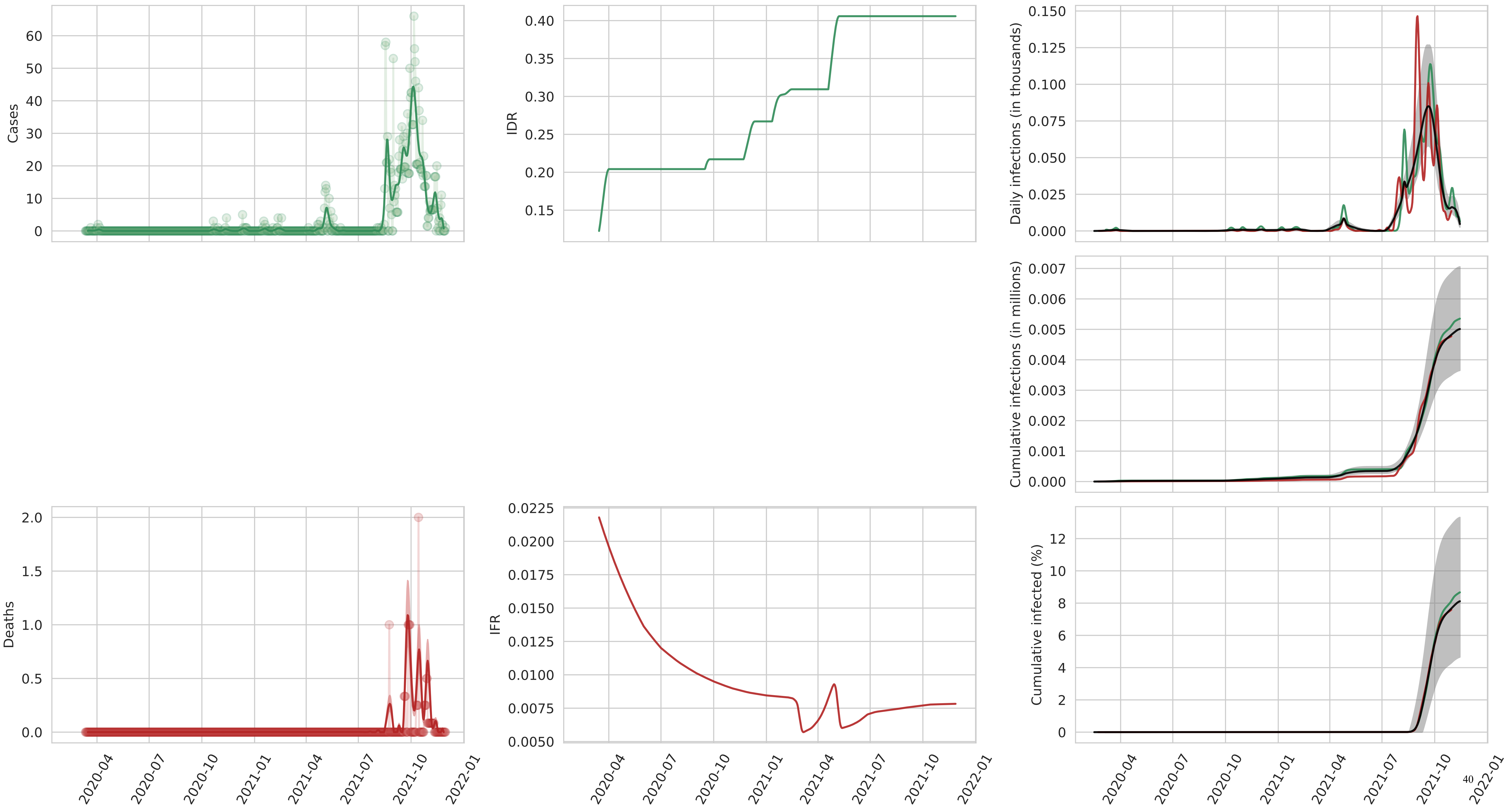

Nova Scotia

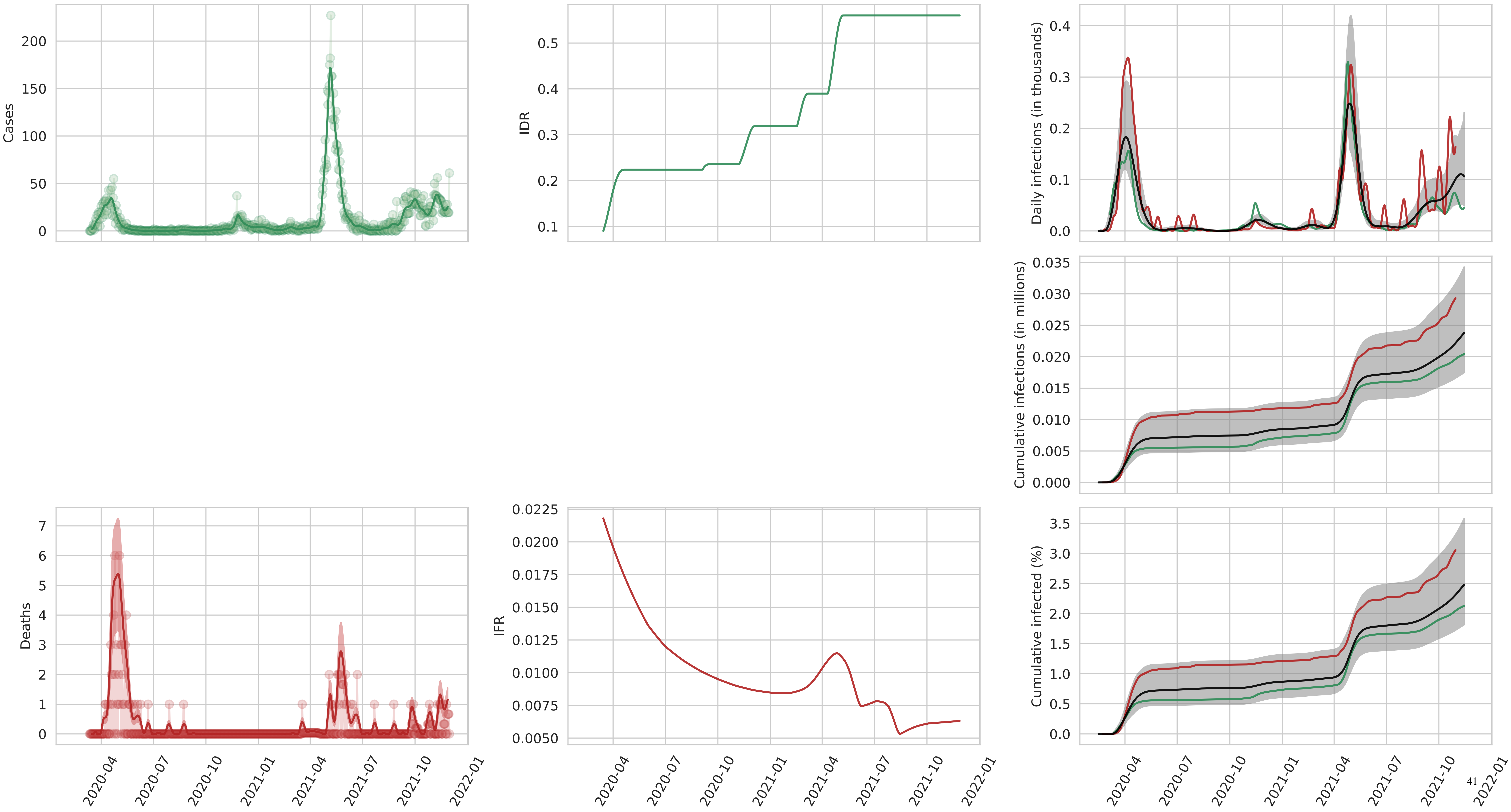
Cases
IDR
Daily infections (in thousands)
Cumulative infections (in millions)
Deaths
IFR
Cumulative infected (%)
2020-04
2020-07
2020-10
2021-01
2021-04
2021-07
2021-10
2022-01

Nunavut

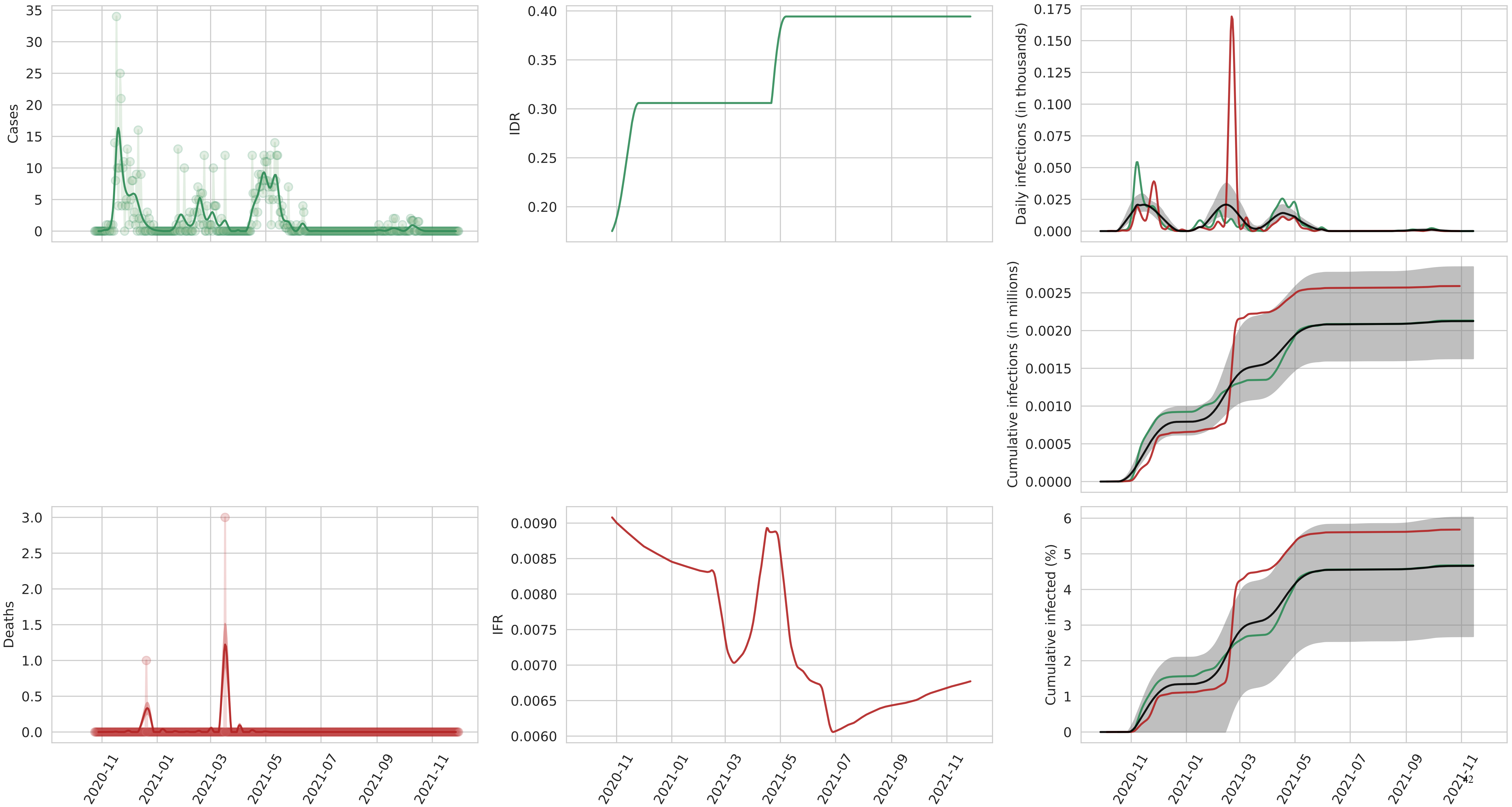
Cases
IDR
Daily infections (in thousands)
Cumulative infections (in millions)
Deaths
IFR
Cumulative infected (%)
2020-11
2021-01
2021-03
2021-05
2021-07
2021-09
2021-11

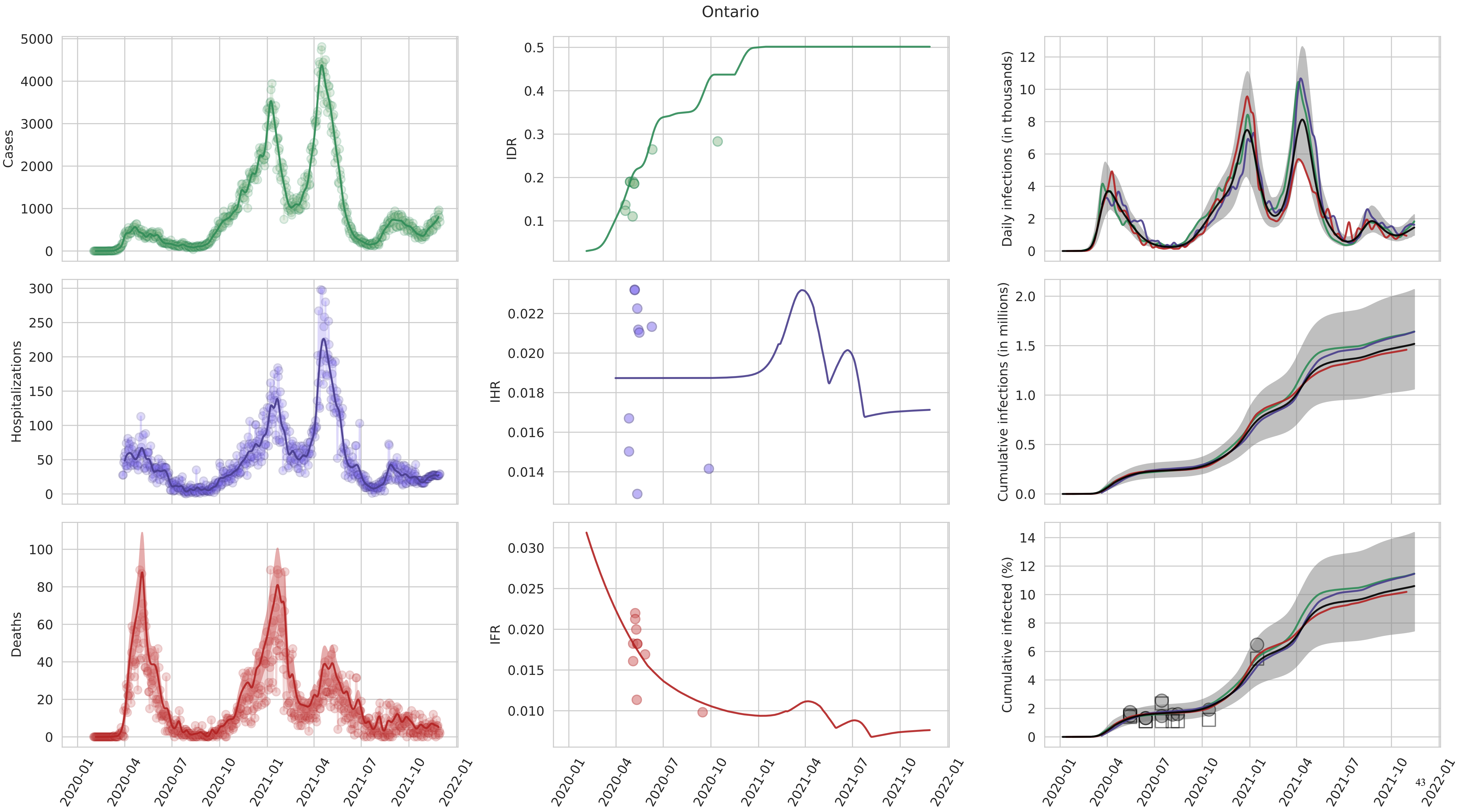

Ontario
Cases
Hospitalizations
Deaths
IDR
IHR
IFR
Daily infections (in thousands)
Cumulative infections (in millions)
Cumulative infected (%)

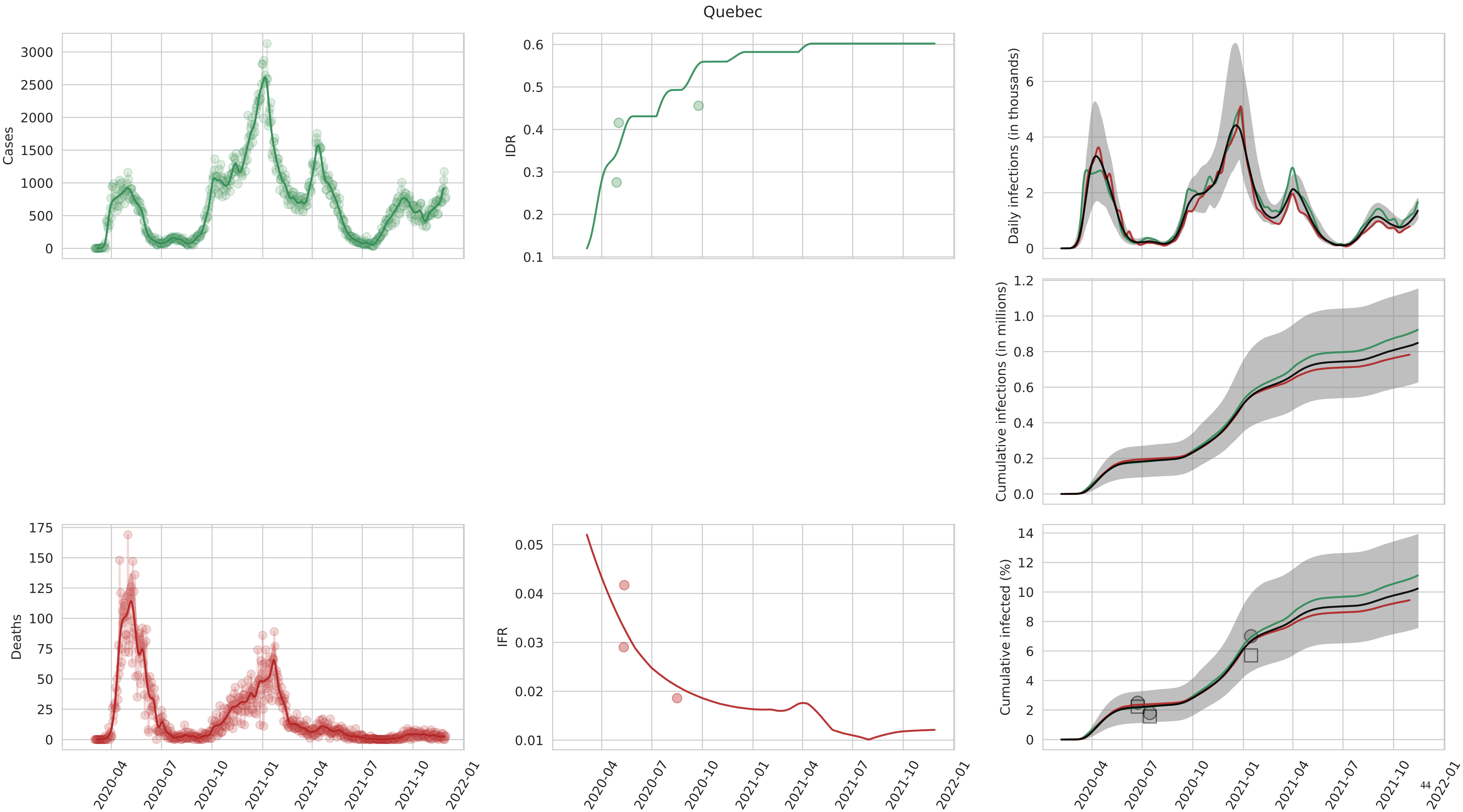
Quebec
Cases
IDR
Daily infections (in thousands)
Cumulative infections (in millions)
Deaths
IFR
Cumulative infected (%)
2020-04
2020-07
2020-10
2021-01
2021-04
2021-07
2021-10
2022-01

Saskatchewan

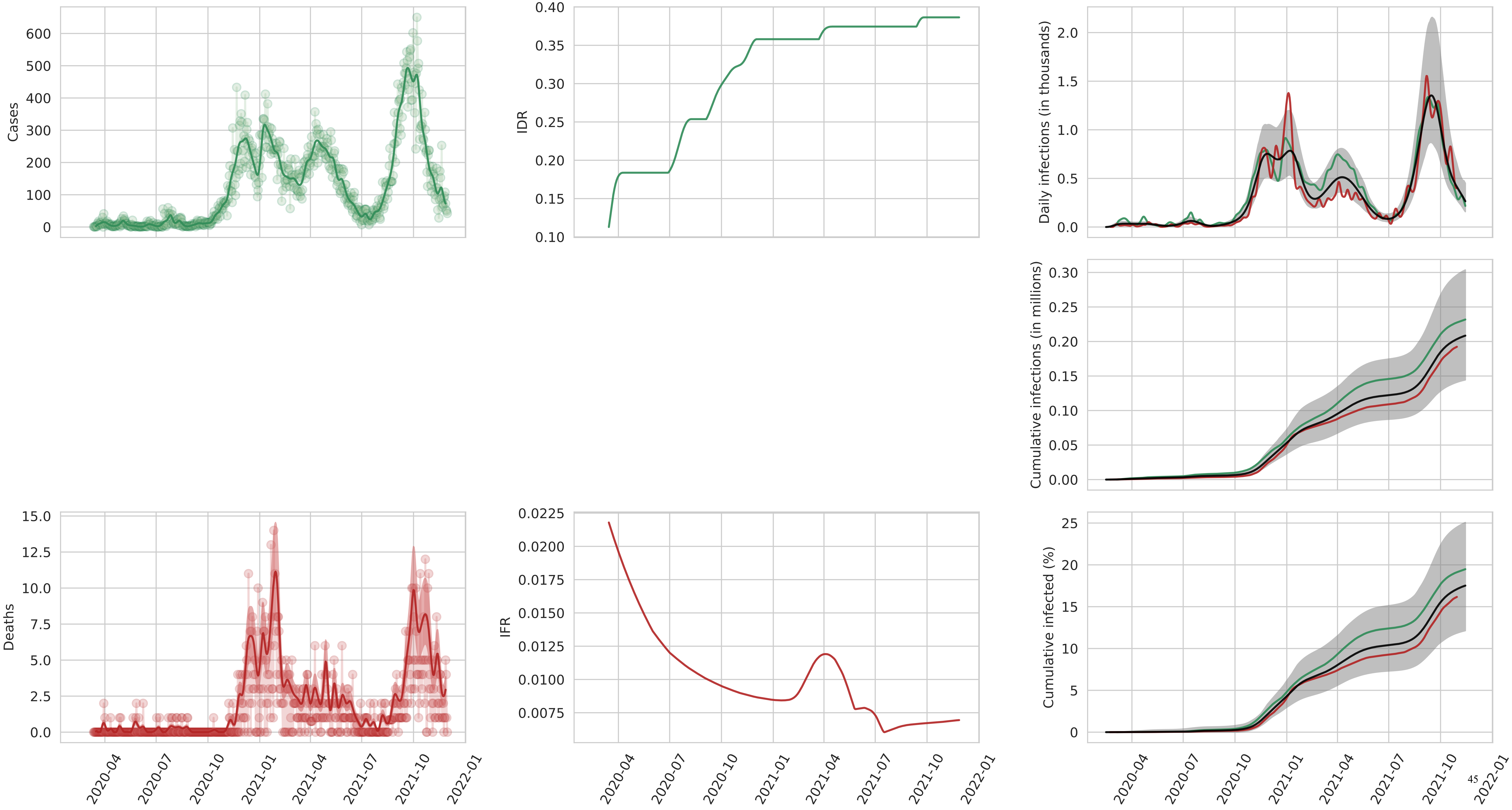

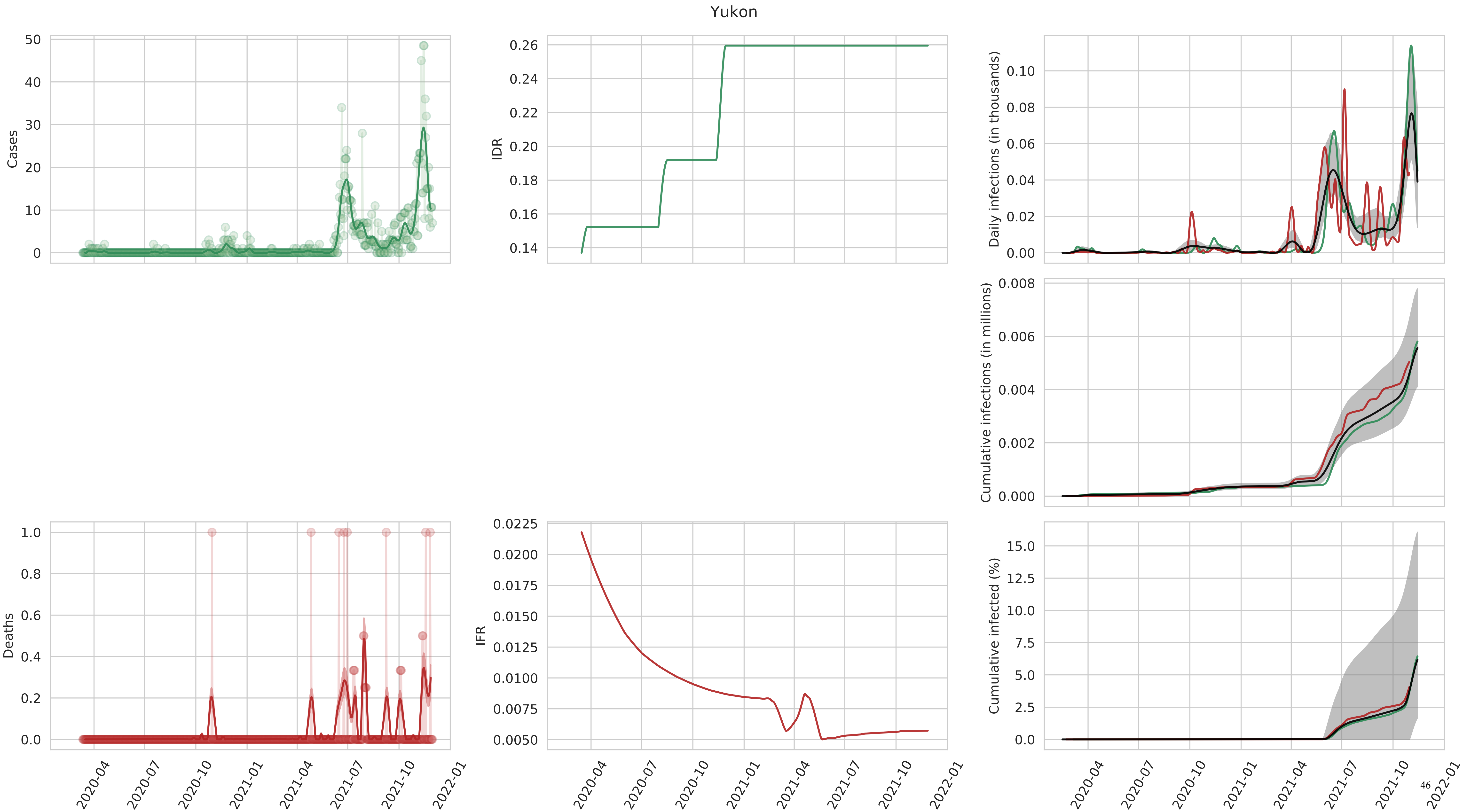
Yukon
Cases
IDR
Daily infections (in thousands)
Cumulative infections (in millions)
Deaths
IFR
Cumulative infected (%)

Alabama

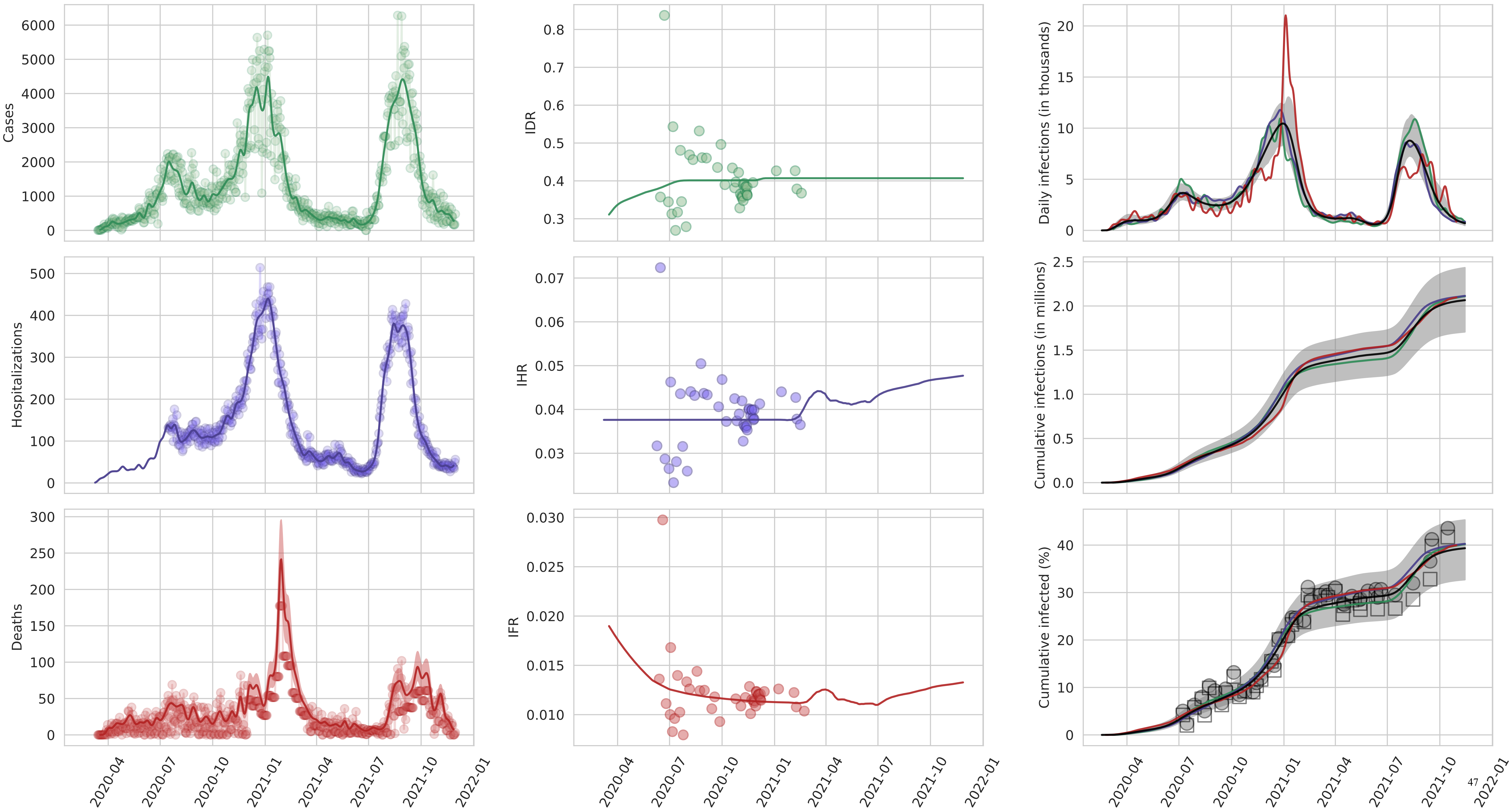
Cases
Hospitalizations
Deaths
IDR
IHR
IFR
Daily infections (in thousands)
Cumulative infections (in millions)
Cumulative infected (%)
2020-04
2020-07
2020-10
2021-01
2021-04
2021-07
2021-10
2022-01

Alaska

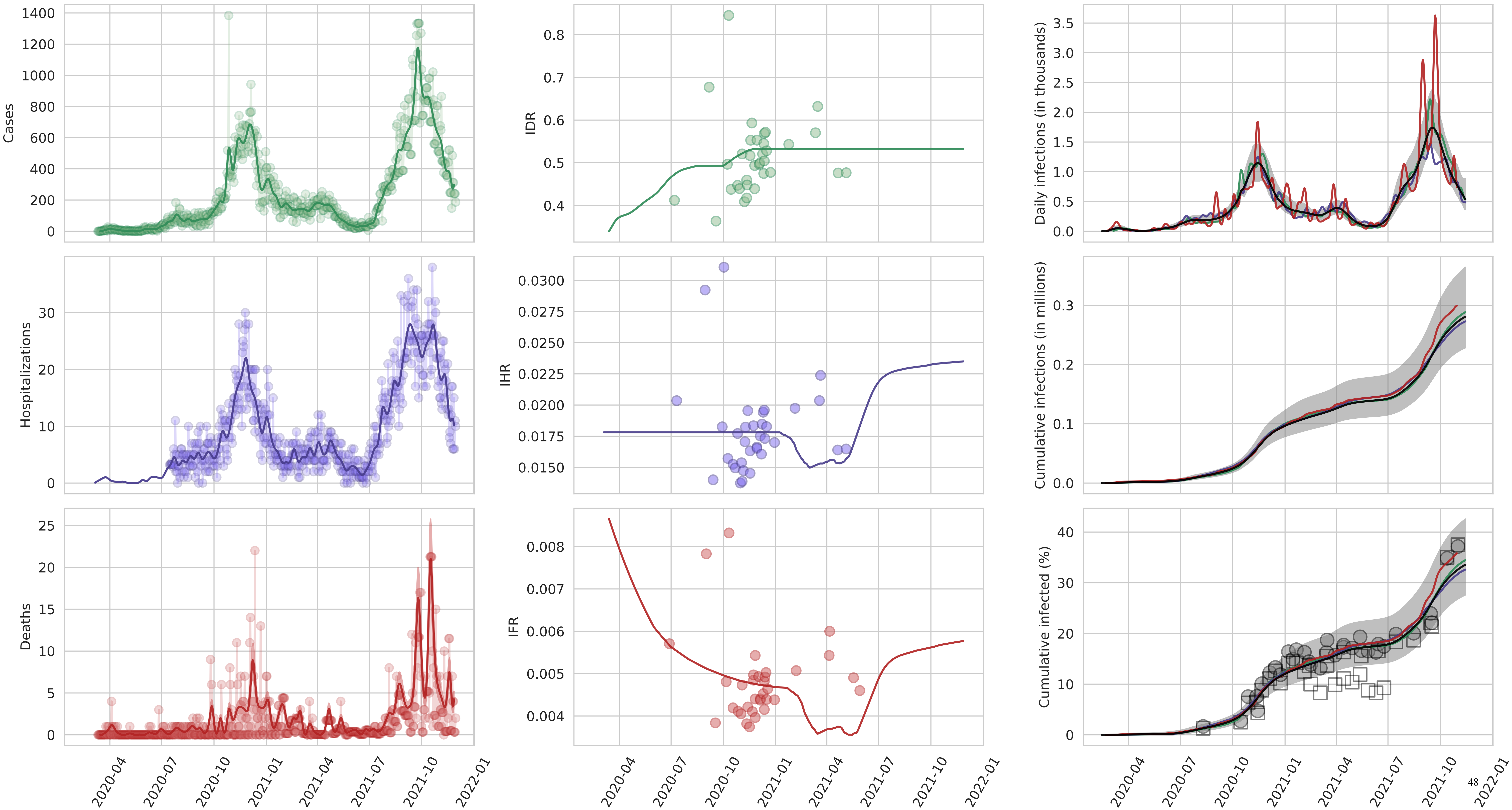

Cases
Hospitalizations
Deaths
IDR
IHR
IFR
Daily infections (in thousands)
Cumulative infections (in millions)
Cumulative infected (%)
2020-04
2020-07
2020-10
2021-01
2021-04
2021-07
2021-10
2022-01

Arizona

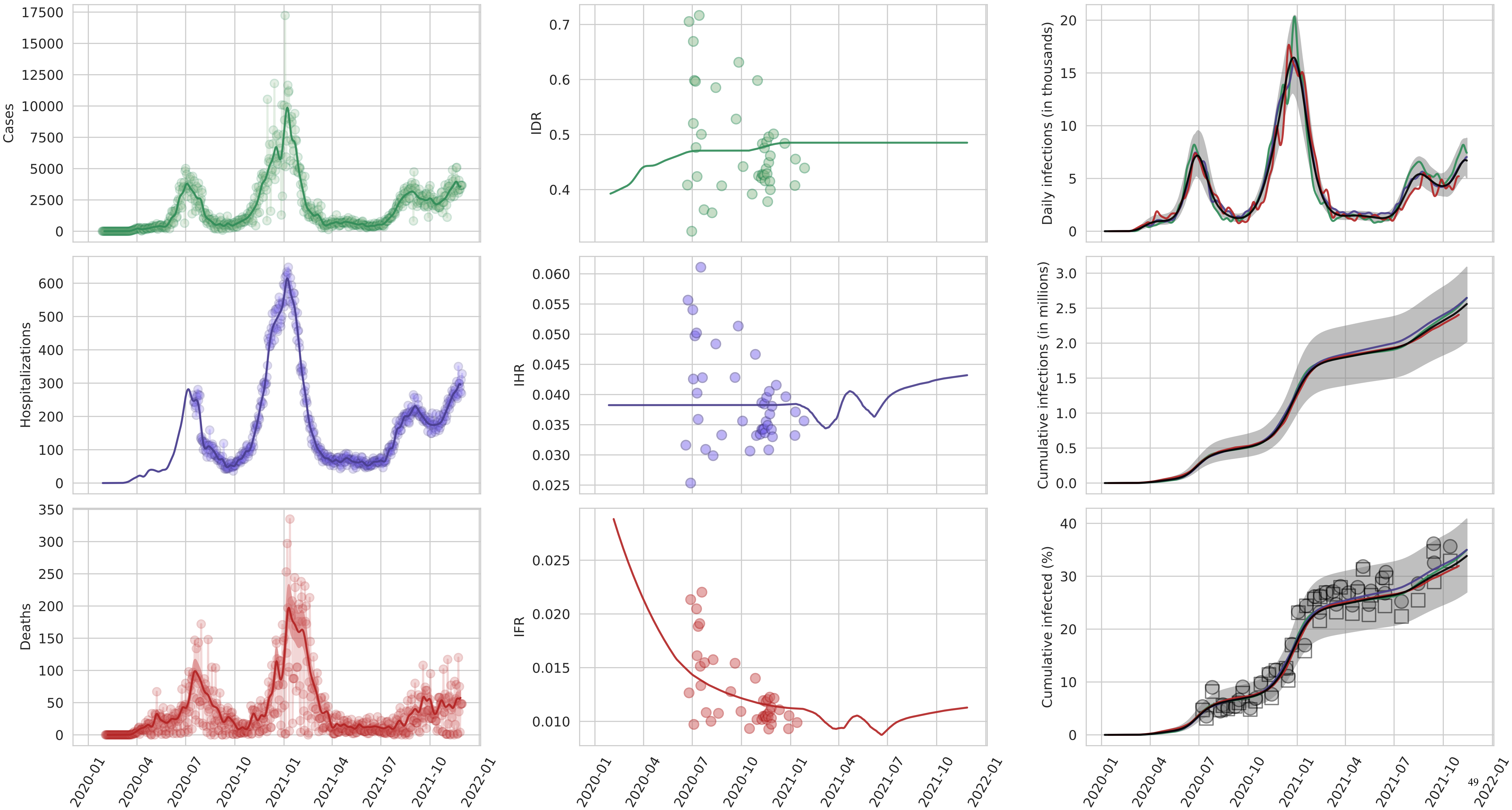

Cases
Hospitalizations
Deaths
IDR
IHR
IFR
Daily infections (in thousands)
Cumulative infections (in millions)
Cumulative infected (%)

Arkansas

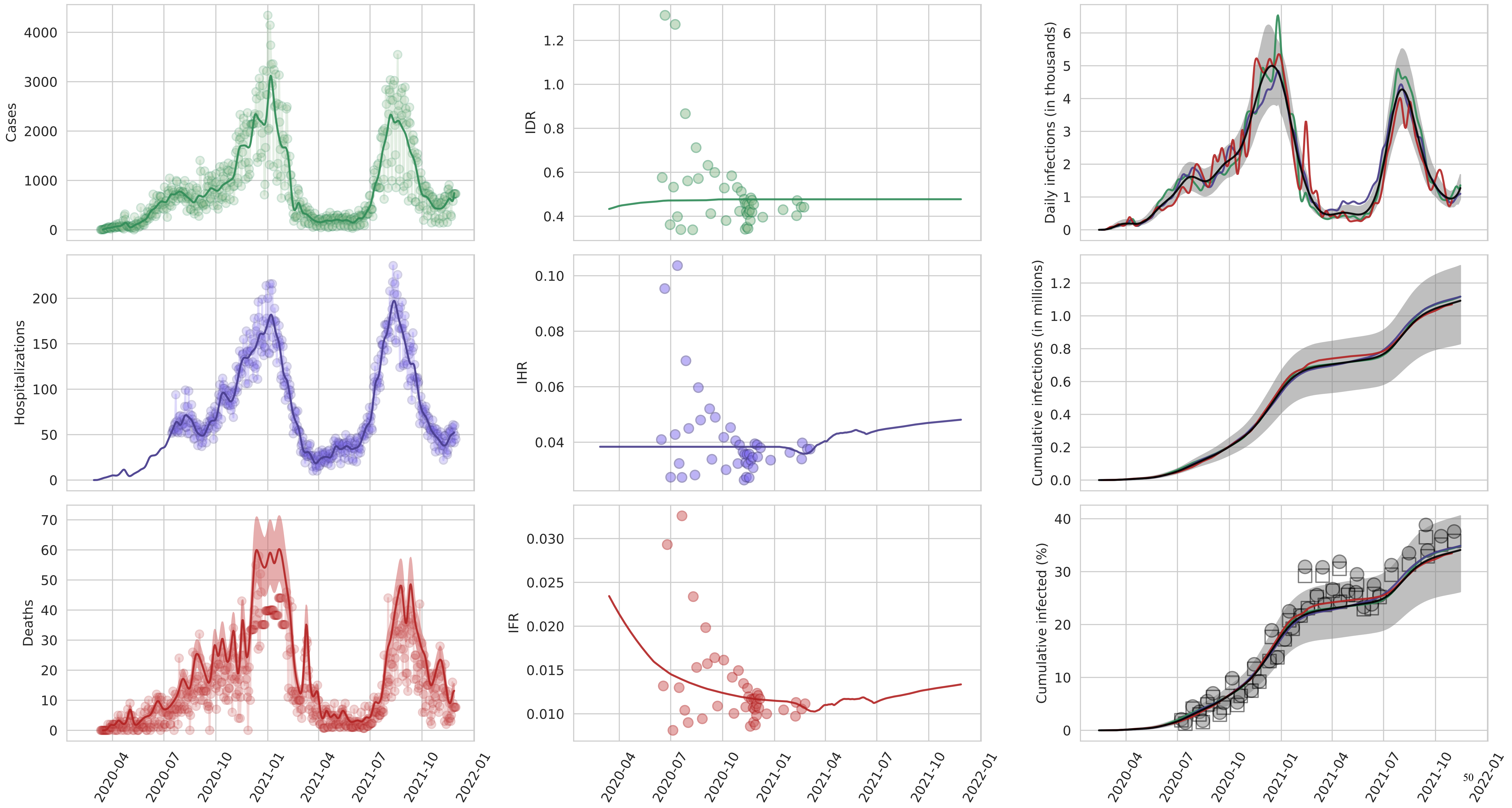

Cases
IDR
Daily infections (in thousands)
Hospitalizations
IHR
Cumulative infections (in millions)
Deaths
IFR
Cumulative infected (%)
2020-04
2020-07
2020-10
2021-01
2021-04
2021-07
2021-10
2022-01

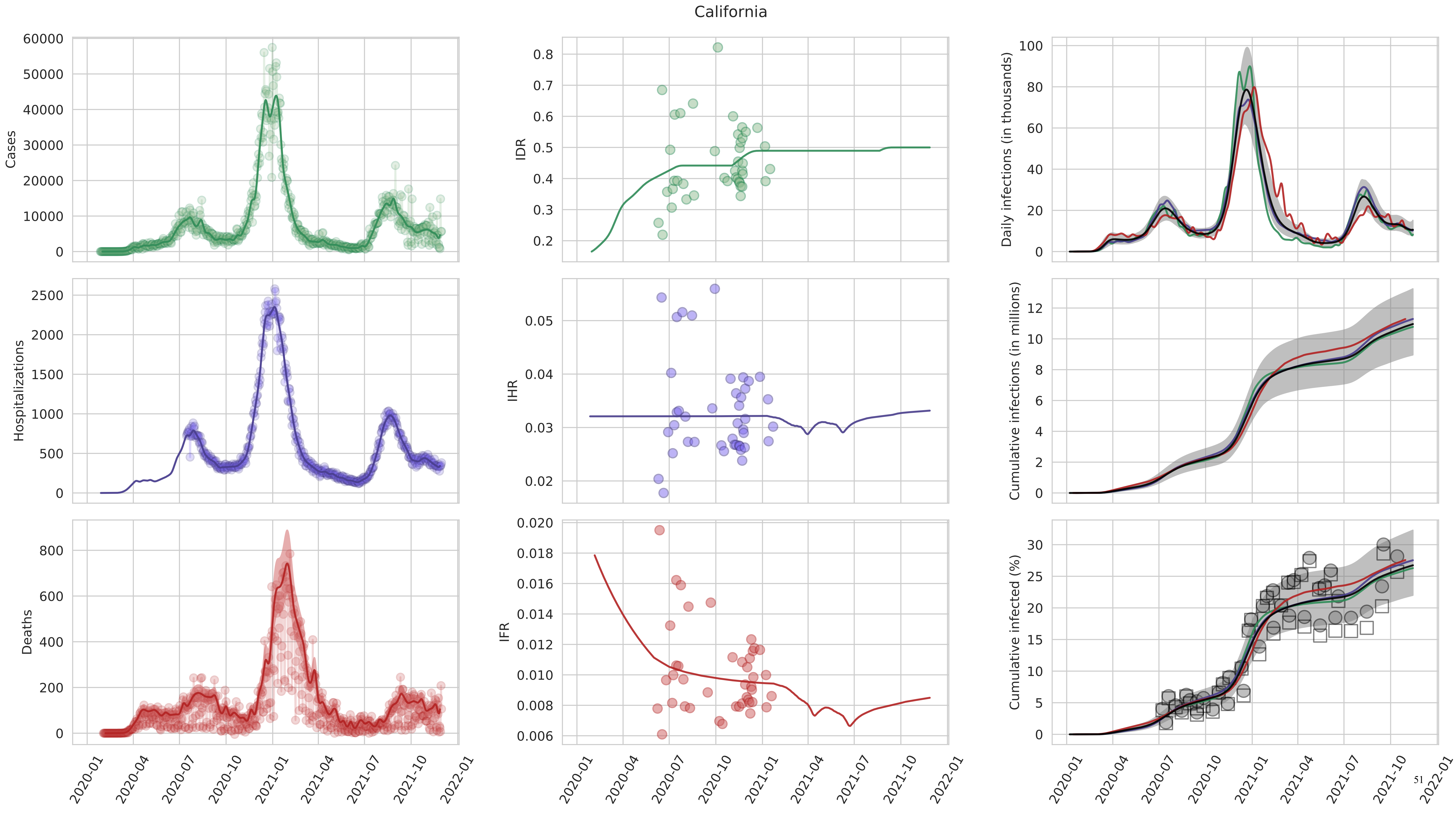
California
Cases
Hospitalizations
Deaths
IDR
IHR
IFR
Daily infections (in thousands)
Cumulative infections (in millions)
Cumulative infected (%)
2020-01
2020-04
2020-07
2020-10
2021-01
2021-04
2021-07
2021-10
2022-01

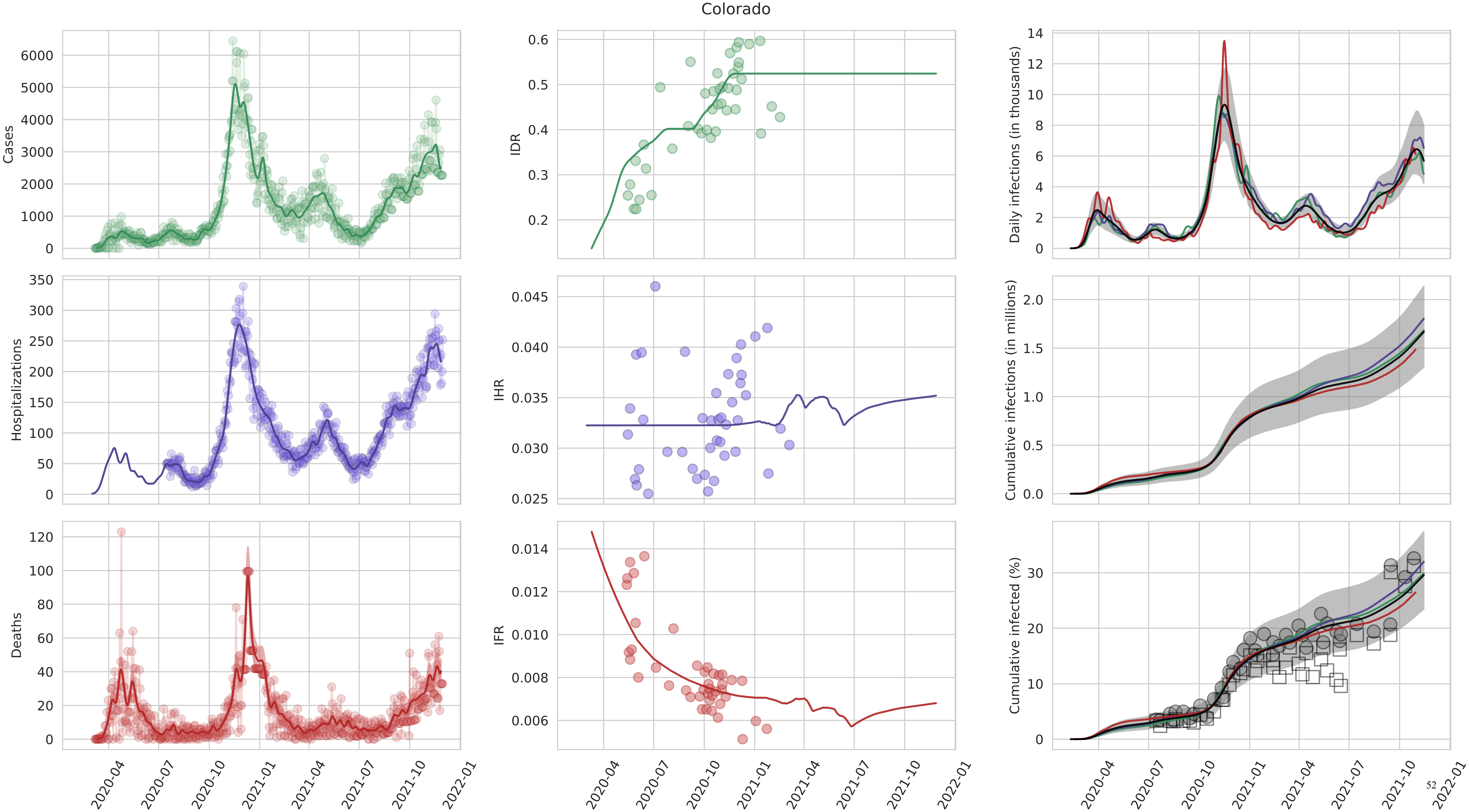

Colorado
Cases
IDR
Daily infections (in thousands)
Hospitalizations
IHR
Cumulative infections (in millions)
Deaths
IFR
Cumulative infected (%)
2020-04
2020-07
2020-10
2021-01
2021-04
2021-07
2021-10
2022-01

Connecticut

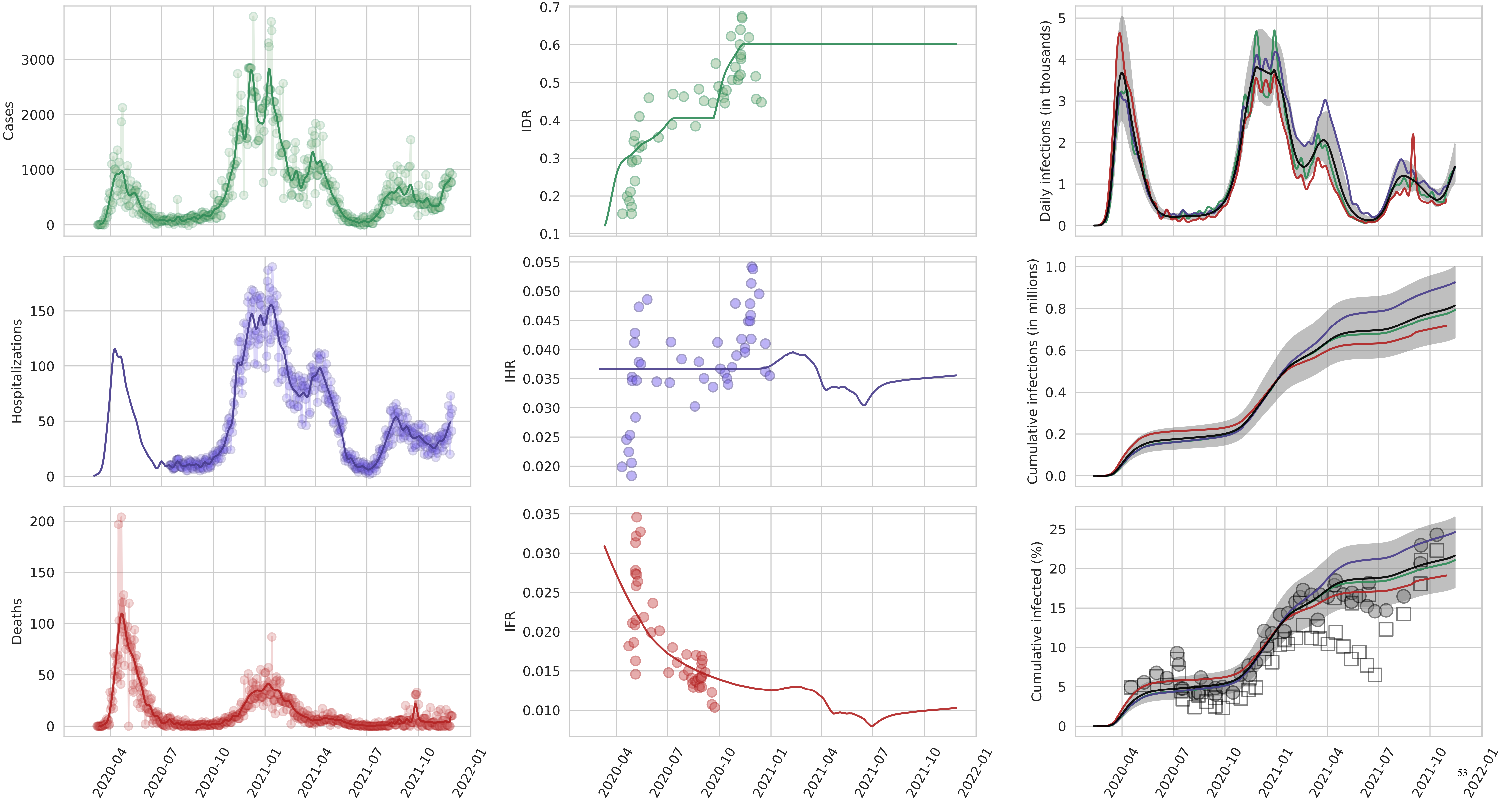

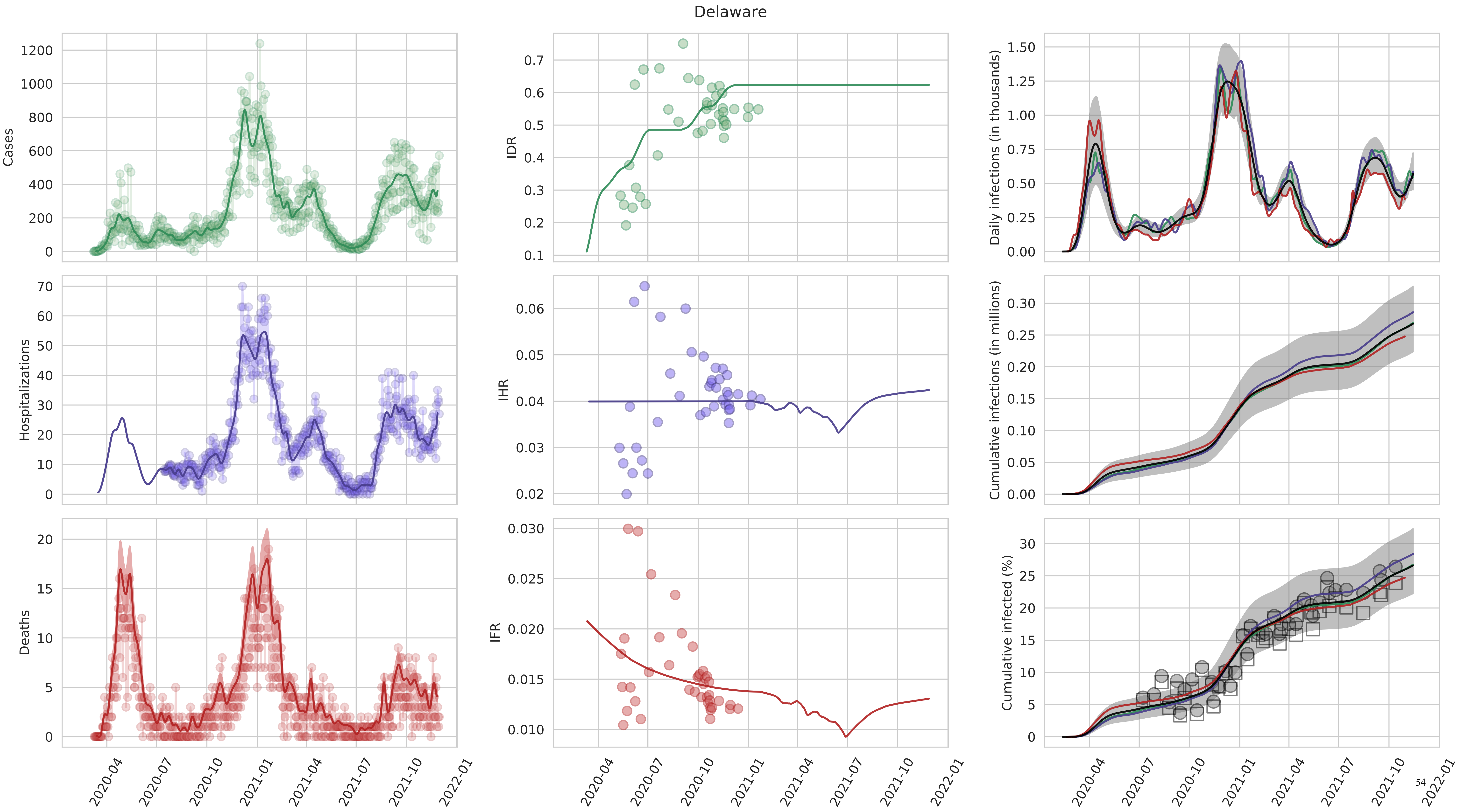

Delaware
Cases
Hospitalizations
Deaths
IDR
IHR
IFR
Daily infections (in thousands)
Cumulative infections (in millions)
Cumulative infected (%)
2020-04
2020-07
2020-10
2021-01
2021-04
2021-07
2021-10
2022-01

District of Columbia

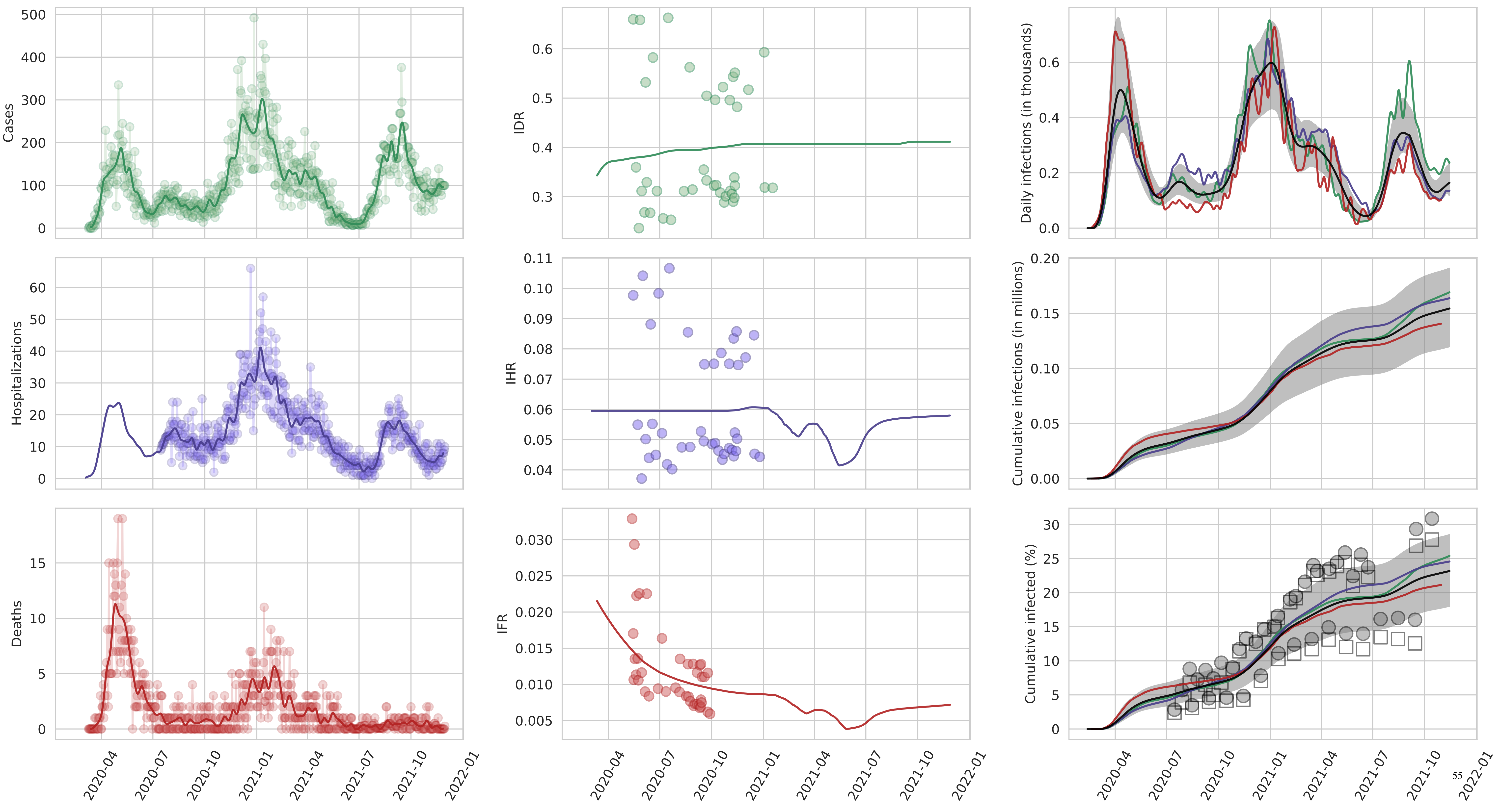

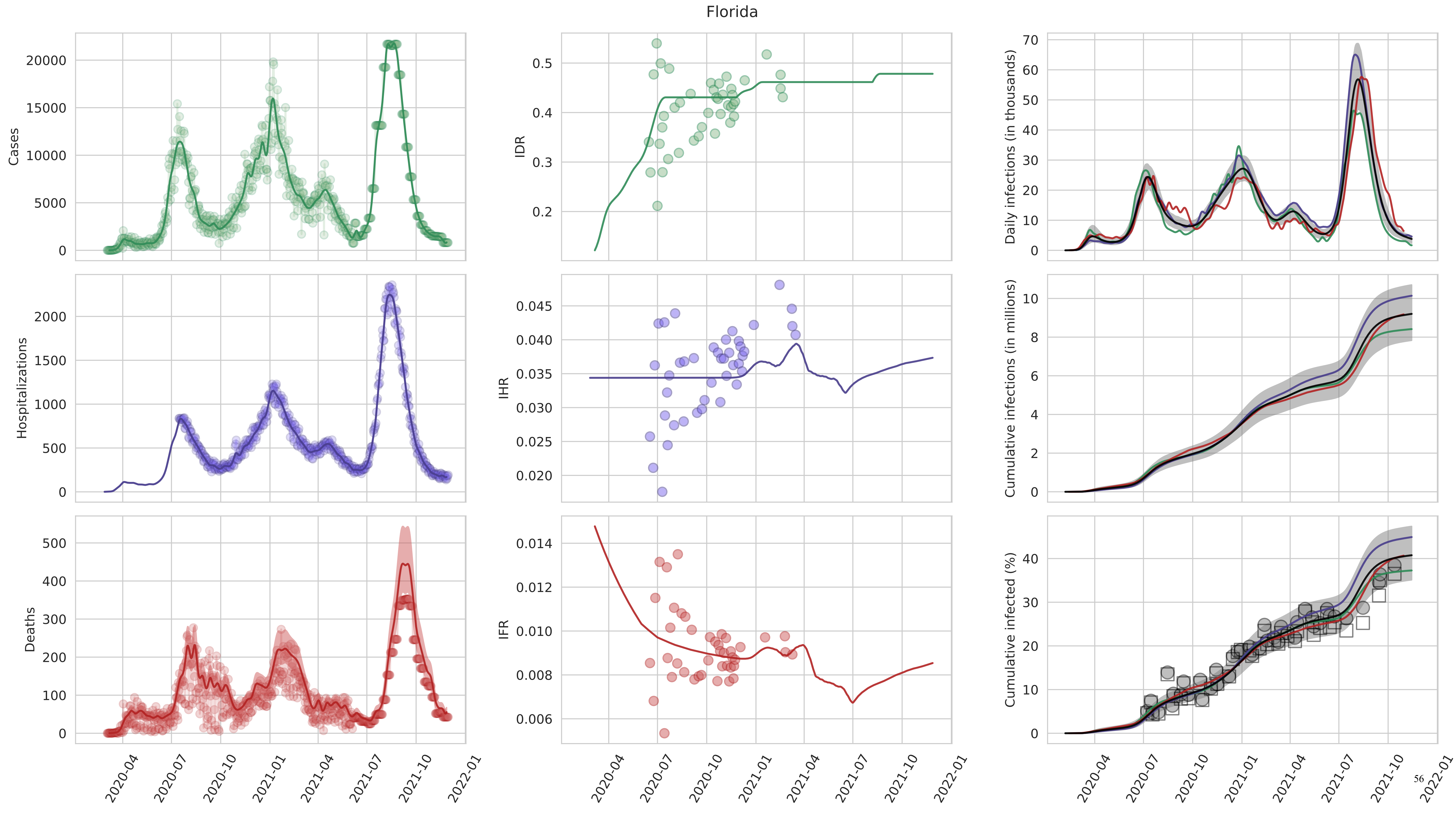
Florida
Cases
IDR
Daily infections (in thousands)
Hospitalizations
IHR
Cumulative infections (in millions)
Deaths
IFR
Cumulative infected (%)
2020-04
2020-07
2020-10
2021-01
2021-04
2021-07
2021-10
2022-01

Georgia

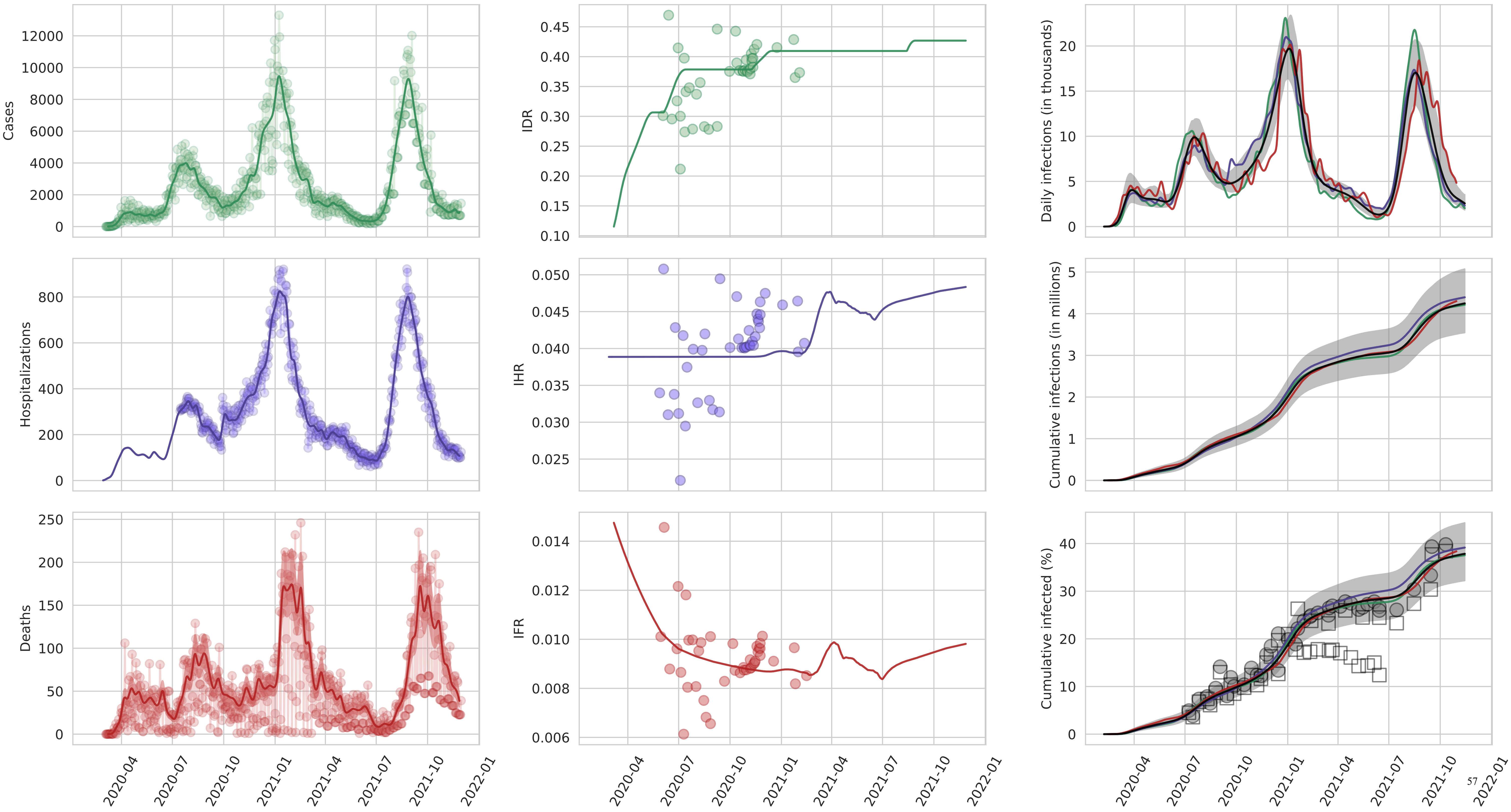

Cases
Hospitalizations
Deaths
IDR
IHR
IFR
Daily infections (in thousands)
Cumulative infections (in millions)
Cumulative infected (%)
2020-04
2020-07
2020-10
2021-01
2021-04
2021-07
2021-10
2022-01

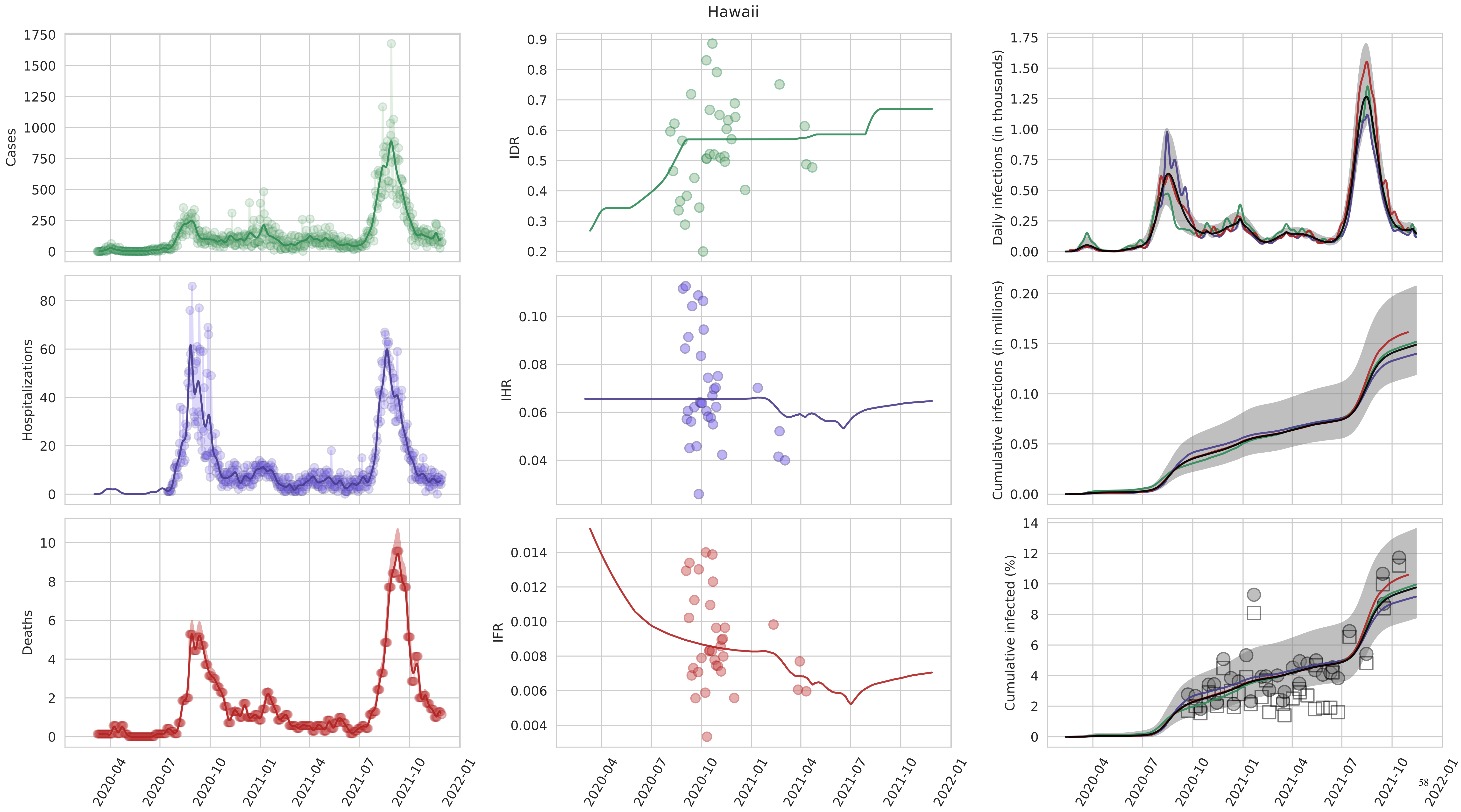
Hawaii
Cases
Hospitalizations
Deaths
IDR
IHR
IFR
Daily infections (in thousands)
Cumulative infections (in millions)
Cumulative infected (%)
2020-04
2020-07
2020-10
2021-01
2021-04
2021-07
2021-10
2022-01

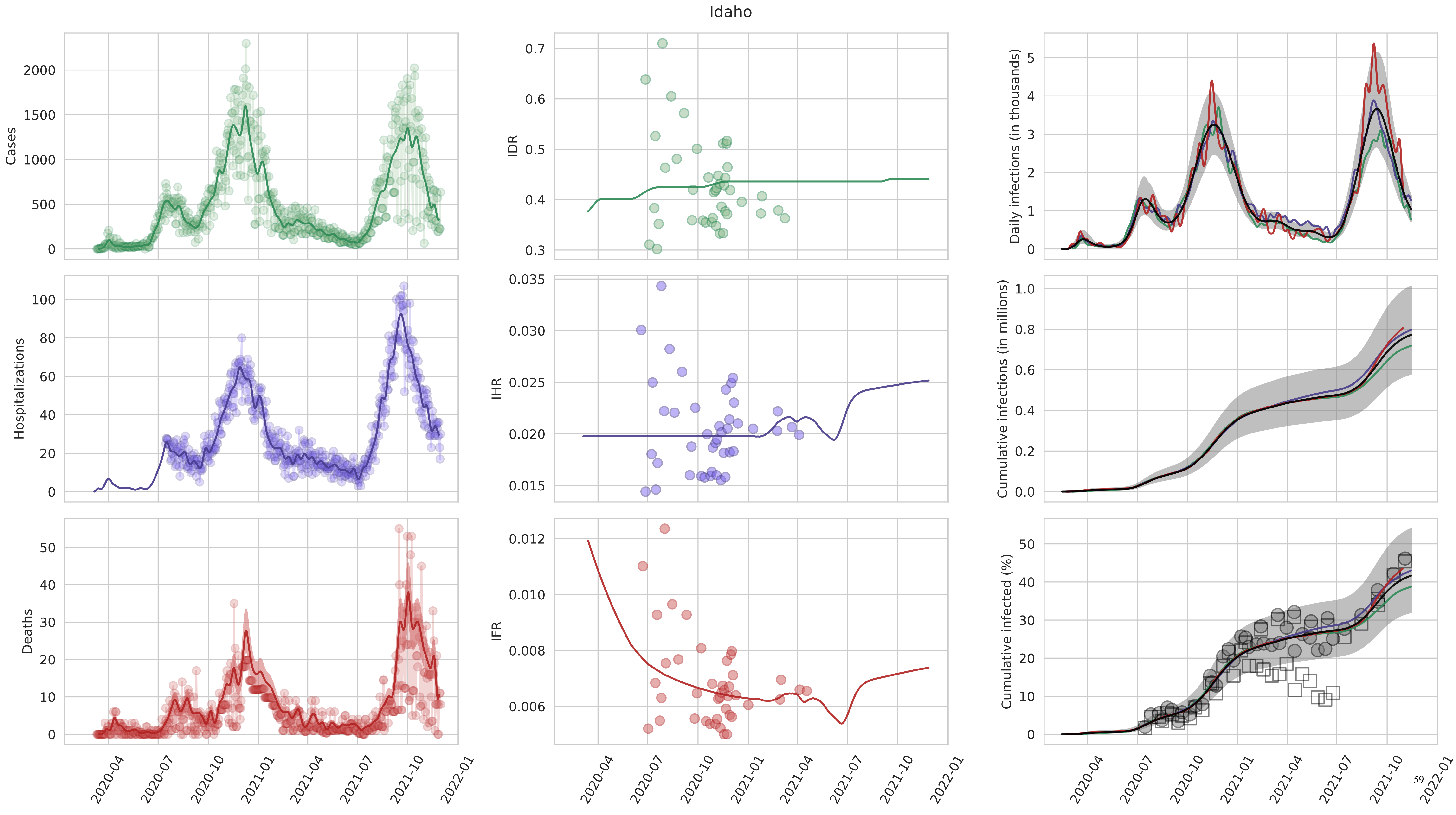
Idaho
Cases
Hospitalizations
Deaths
IDR
IHR
IFR
Daily infections (in thousands)
Cumulative infections (in millions)
Cumulative infected (%)
2020-04
2020-07
2020-10
2021-01
2021-04
2021-07
2021-10
2022-01

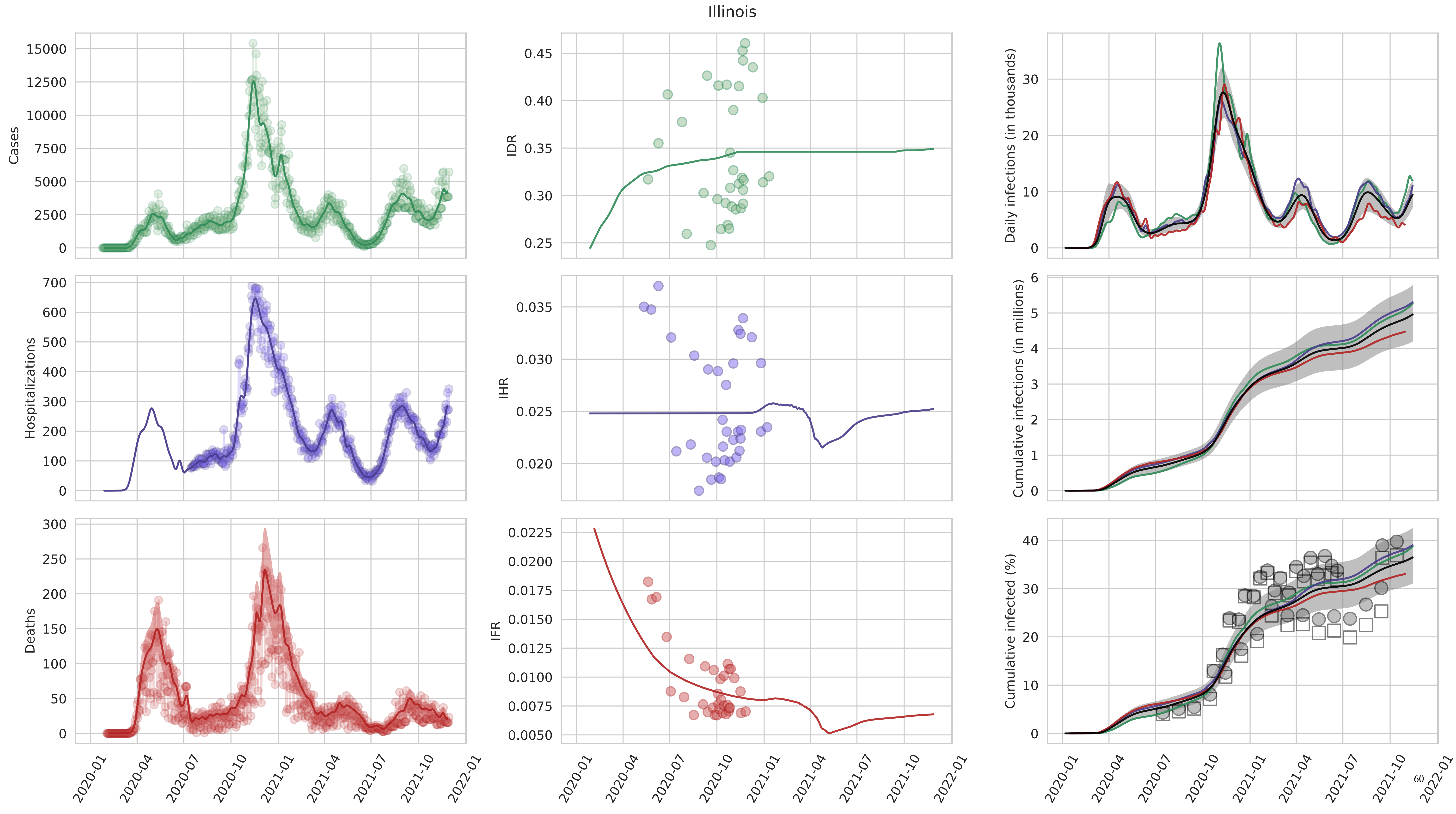
Illinois
Cases
IDR
Daily infections (in thousands)
Hospitalizations
IHR
Cumulative infections (in millions)
Deaths
IFR
Cumulative infected (%)
2020-01
2020-04
2020-07
2020-10
2021-01
2021-04
2021-07
2021-10
2022-01

Indiana

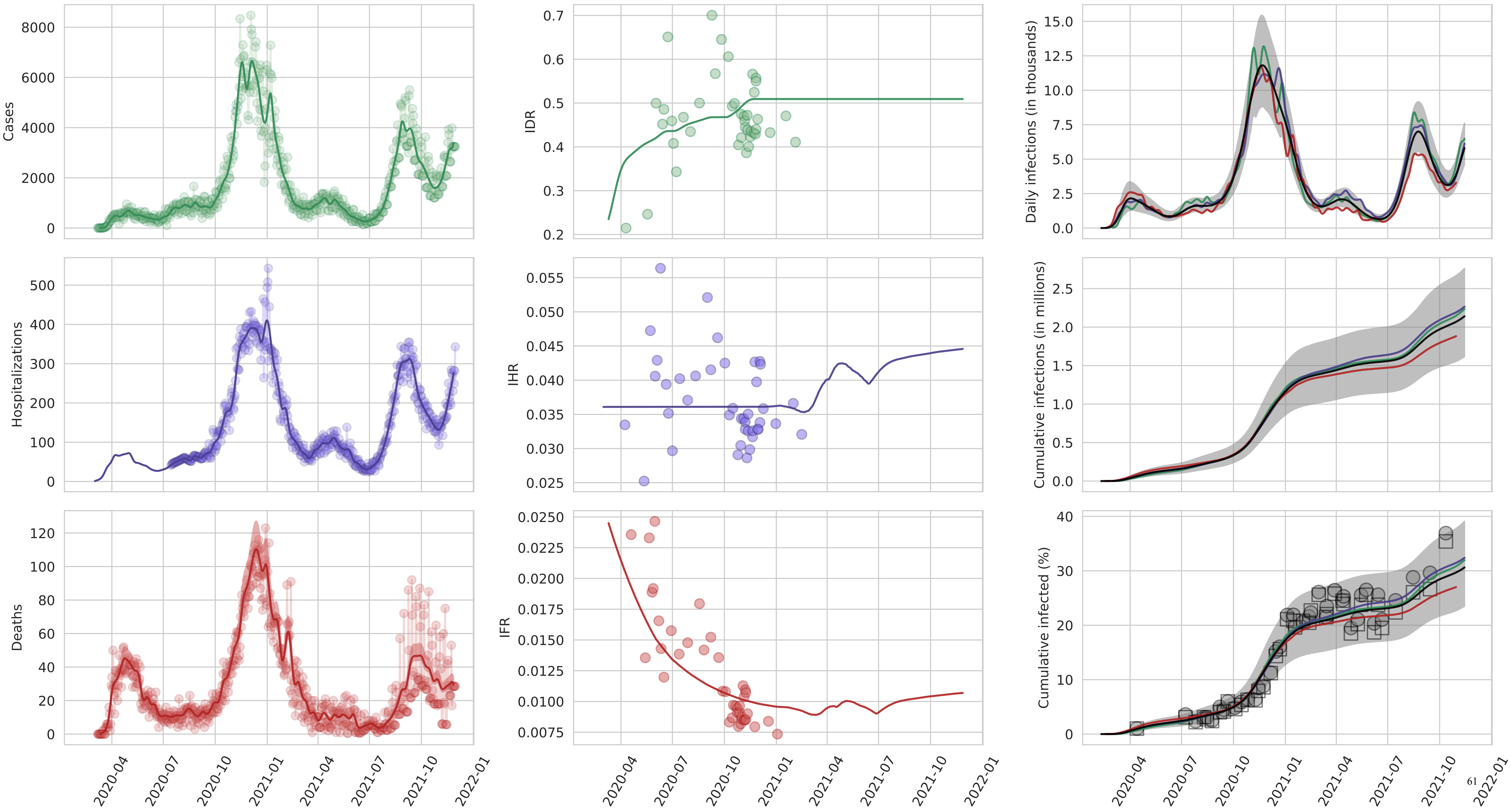
Cases
IDR
Daily infections (in thousands)
Hospitalizations
IHR
Cumulative infections (in millions)
Deaths
IFR
Cumulative infected (%)
2020-04
2020-07
2020-10
2021-01
2021-04
2021-07
2021-10
2022-01

Iowa

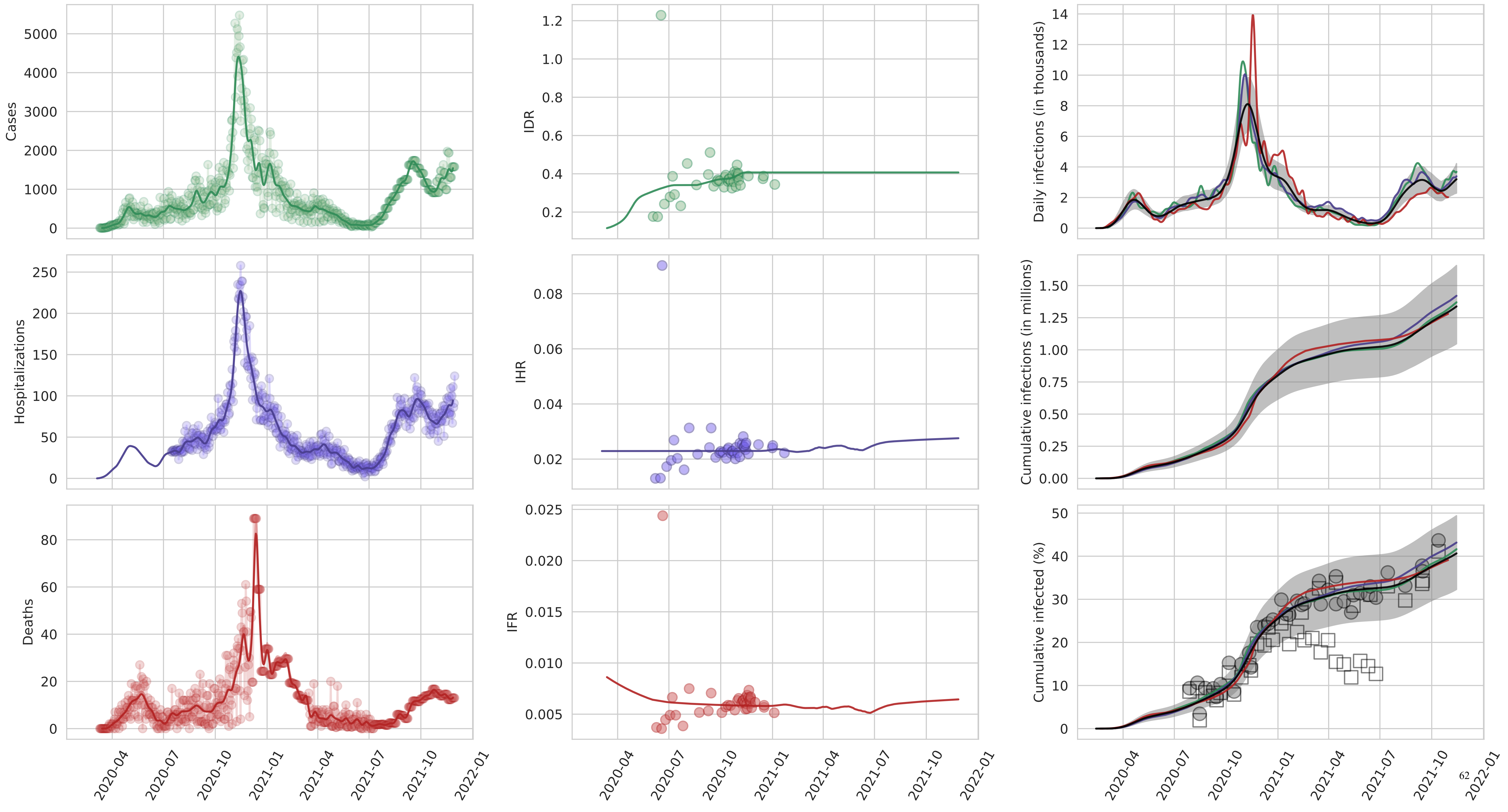
Cases
IDR
Daily infections (in thousands)
Hospitalizations
IHR
Cumulative infections (in millions)
Deaths
IFR
Cumulative infected (%)
2020-04
2020-07
2020-10
2021-01
2021-04
2021-07
2021-10
2022-01

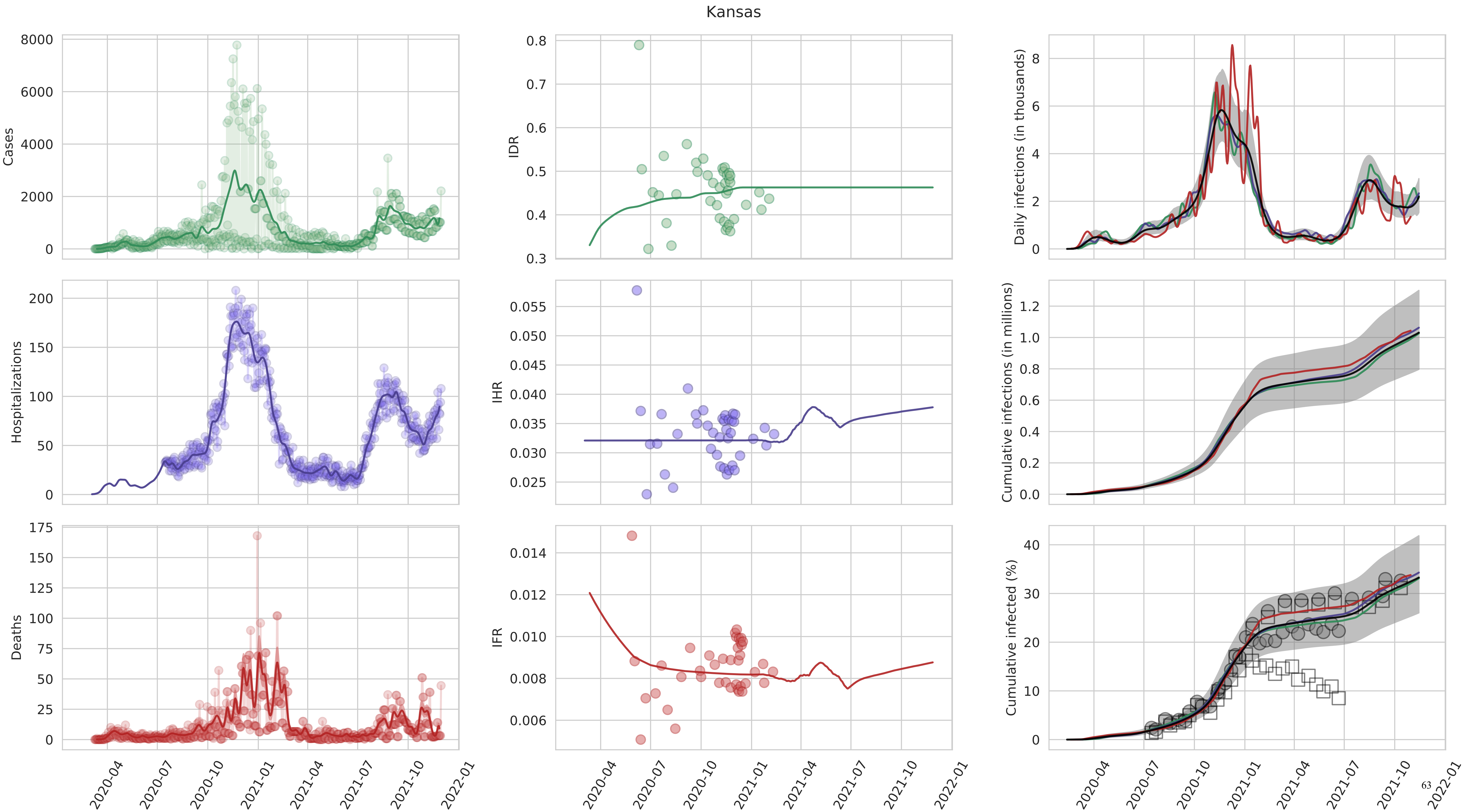

Kansas
Cases
IDR
Daily infections (in thousands)
Hospitalizations
IHR
Cumulative infections (in millions)
Deaths
IFR
Cumulative infected (%)
2020-04
2020-07
2020-10
2021-01
2021-04
2021-07
2021-10
2022-01

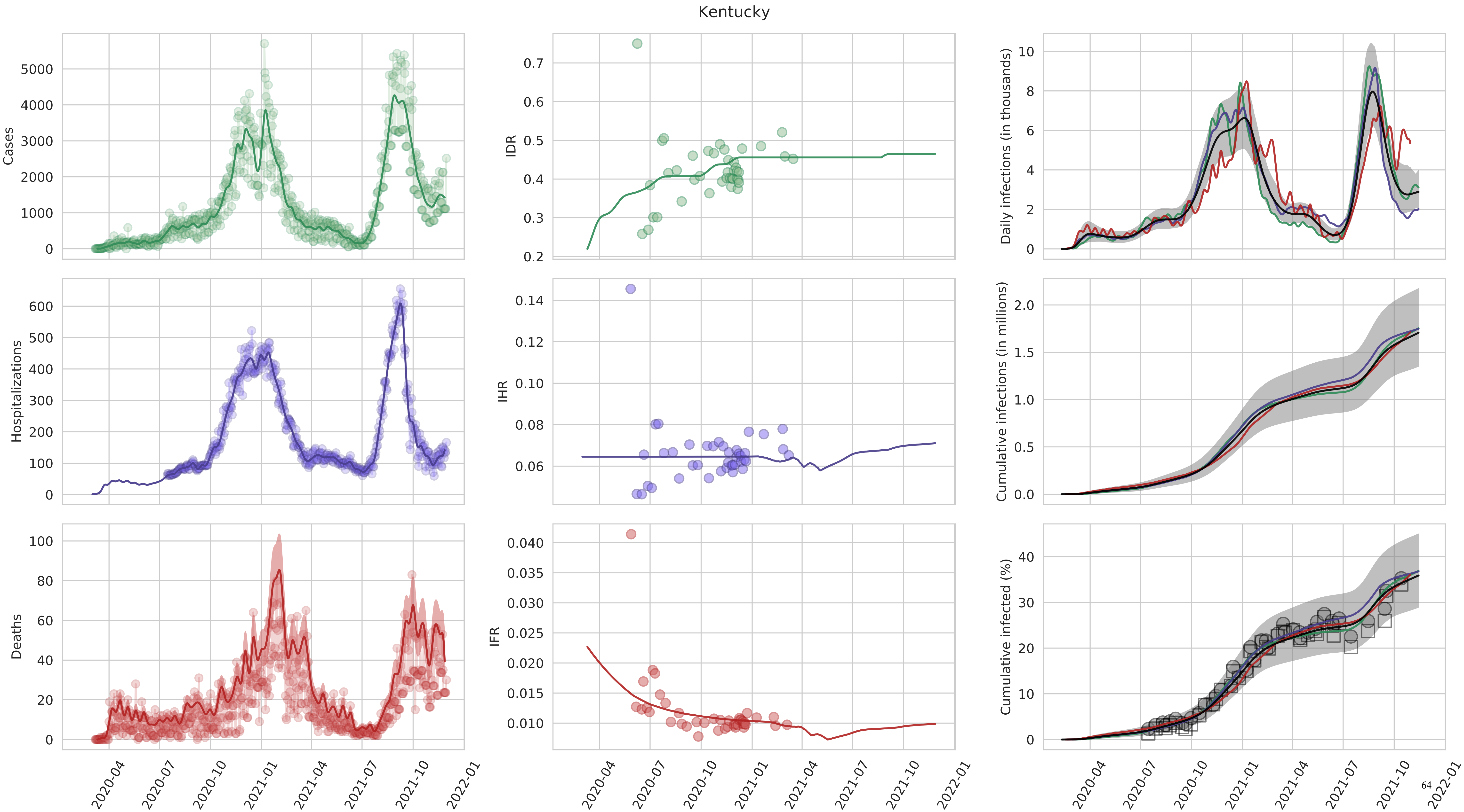

Kentucky
Cases
IDR
Daily infections (in thousands)
Hospitalizations
IHR
Cumulative infections (in millions)
Deaths
IFR
Cumulative infected (%)

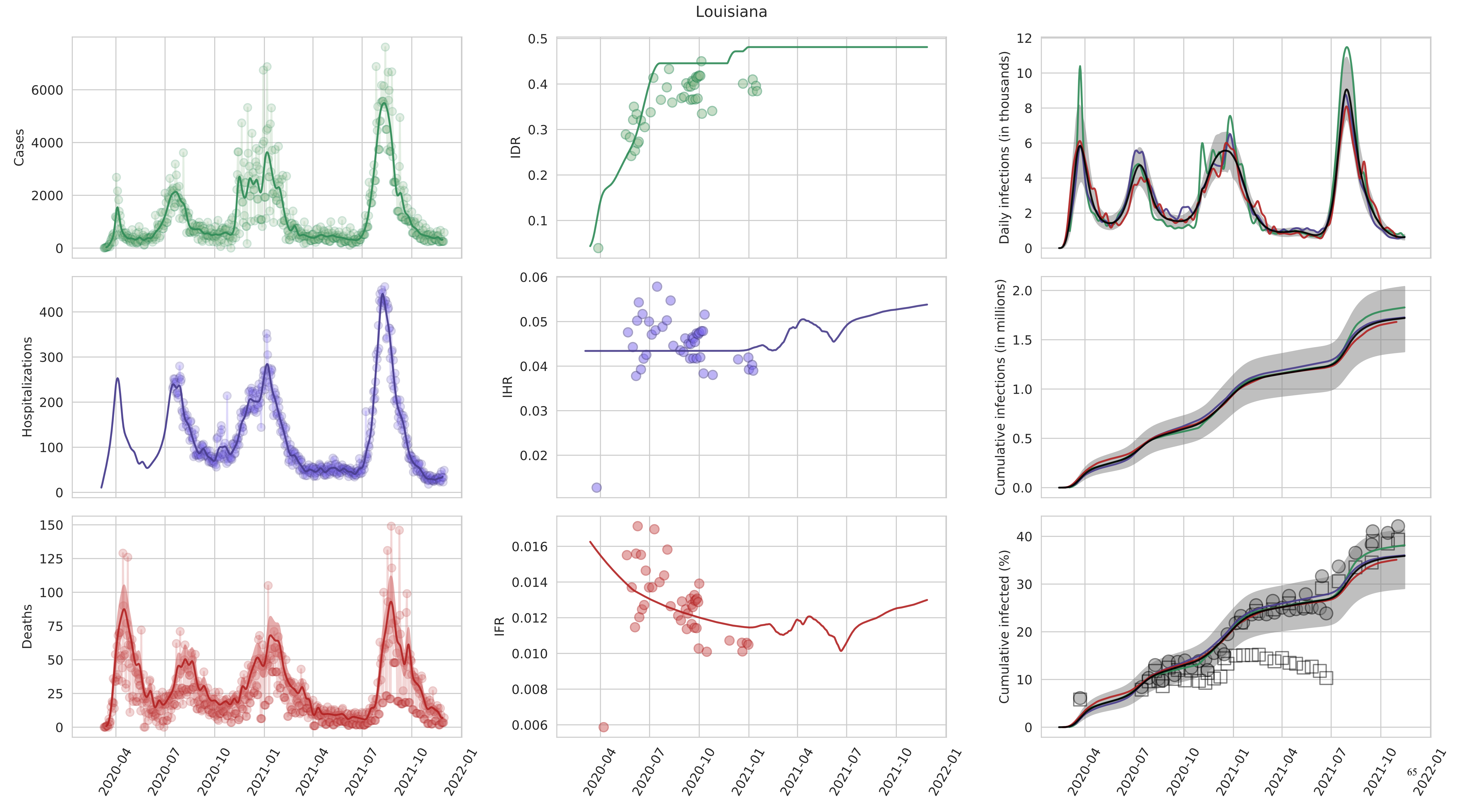
Louisiana
Cases
IDR
Daily infections (in thousands)
Hospitalizations
IHR
Cumulative infections (in millions)
Deaths
IFR
Cumulative infected (%)
2020-04
2020-07
2020-10
2021-01
2021-04
2021-07
2021-10
2022-01

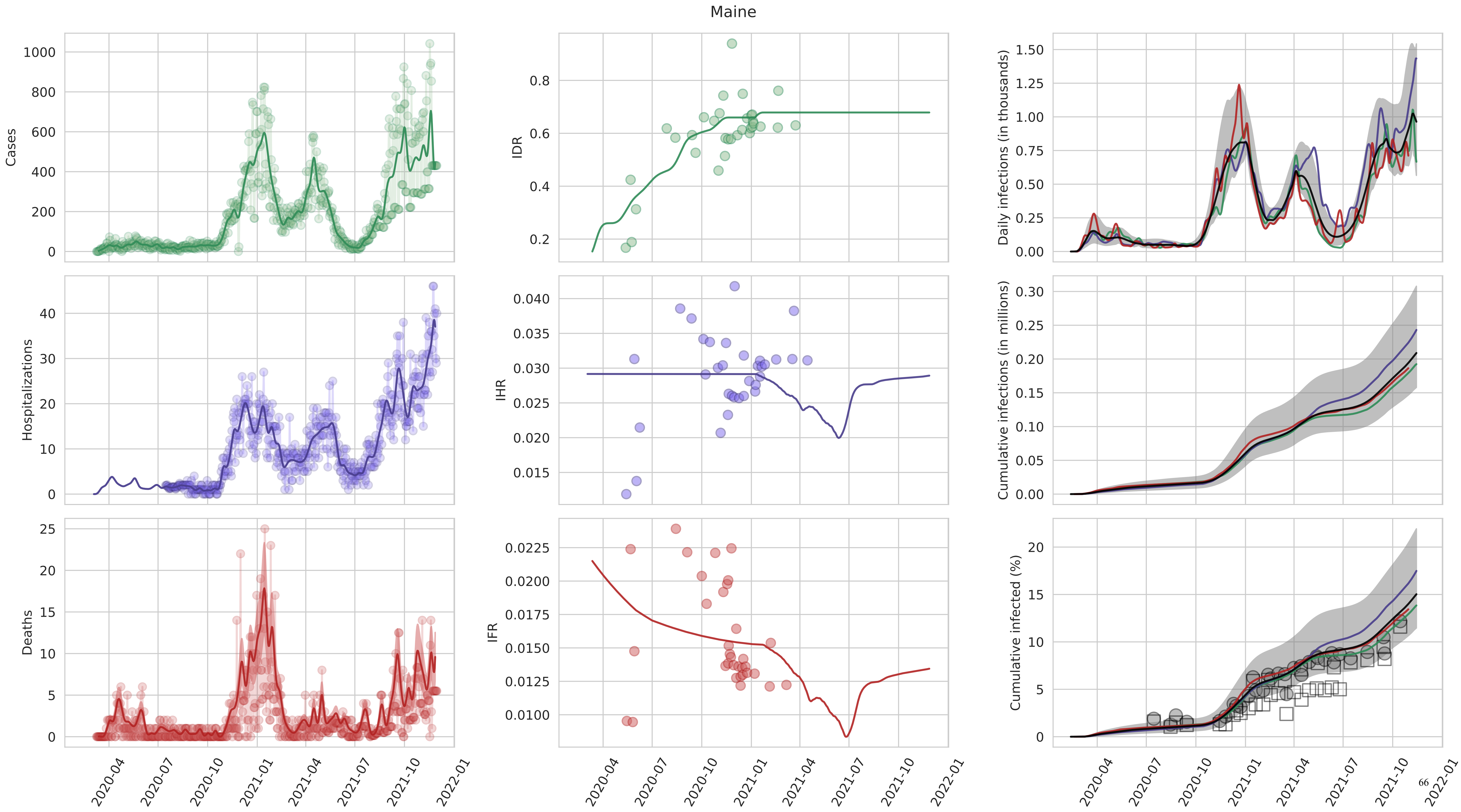
Maine
Cases
Hospitalizations
Deaths
IDR
IHR
IFR
Daily infections (in thousands)
Cumulative infections (in millions)
Cumulative infected (%)
2020-04
2020-07
2020-10
2021-01
2021-04
2021-07
2021-10
2022-01

Maryland

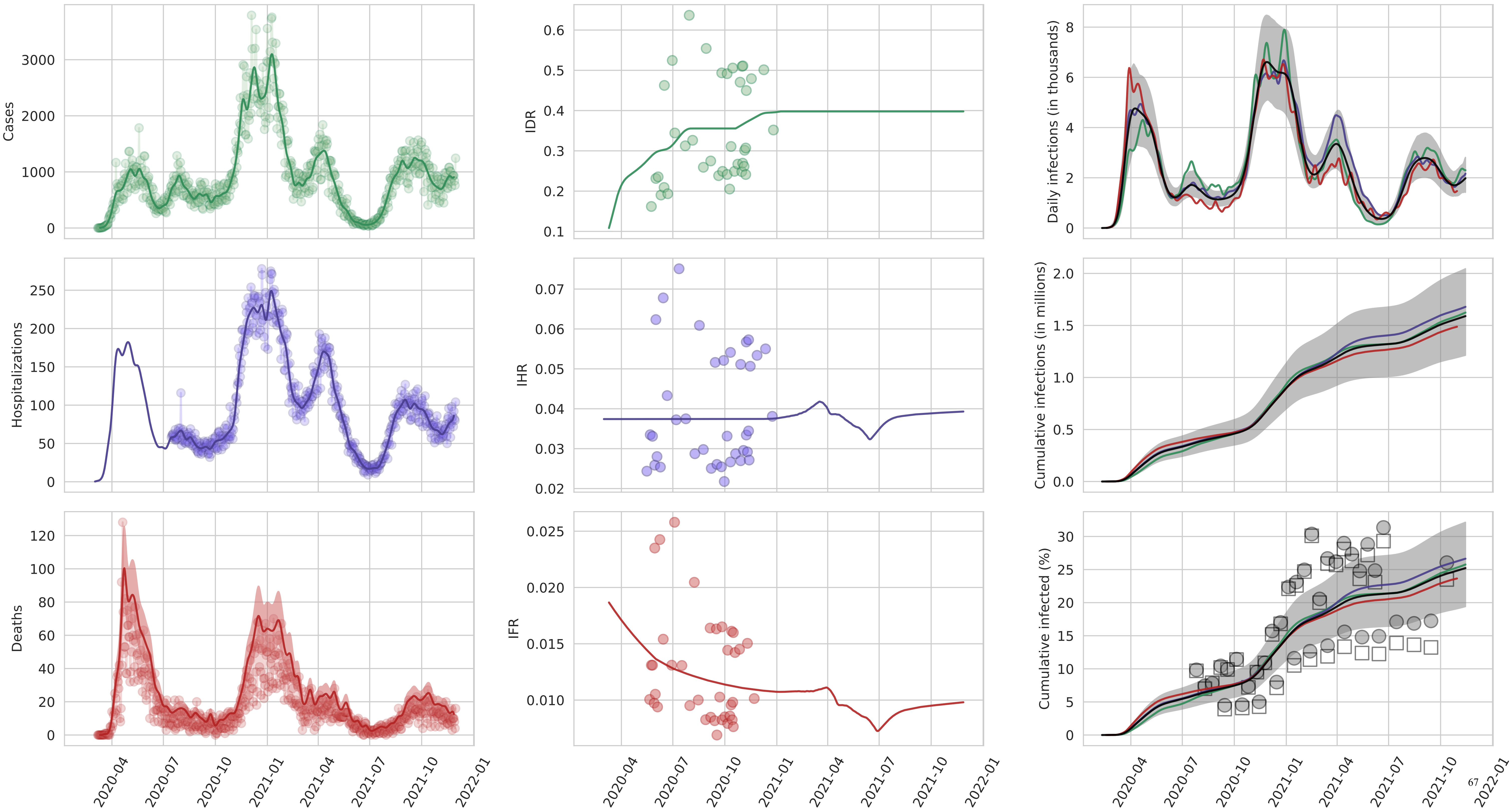

Cases
Hospitalizations
Deaths
IDR
IHR
IFR
Daily infections (in thousands)
Cumulative infections (in millions)
Cumulative infected (%)
2020-04
2020-07
2020-10
2021-01
2021-04
2021-07
2021-10
2022-01

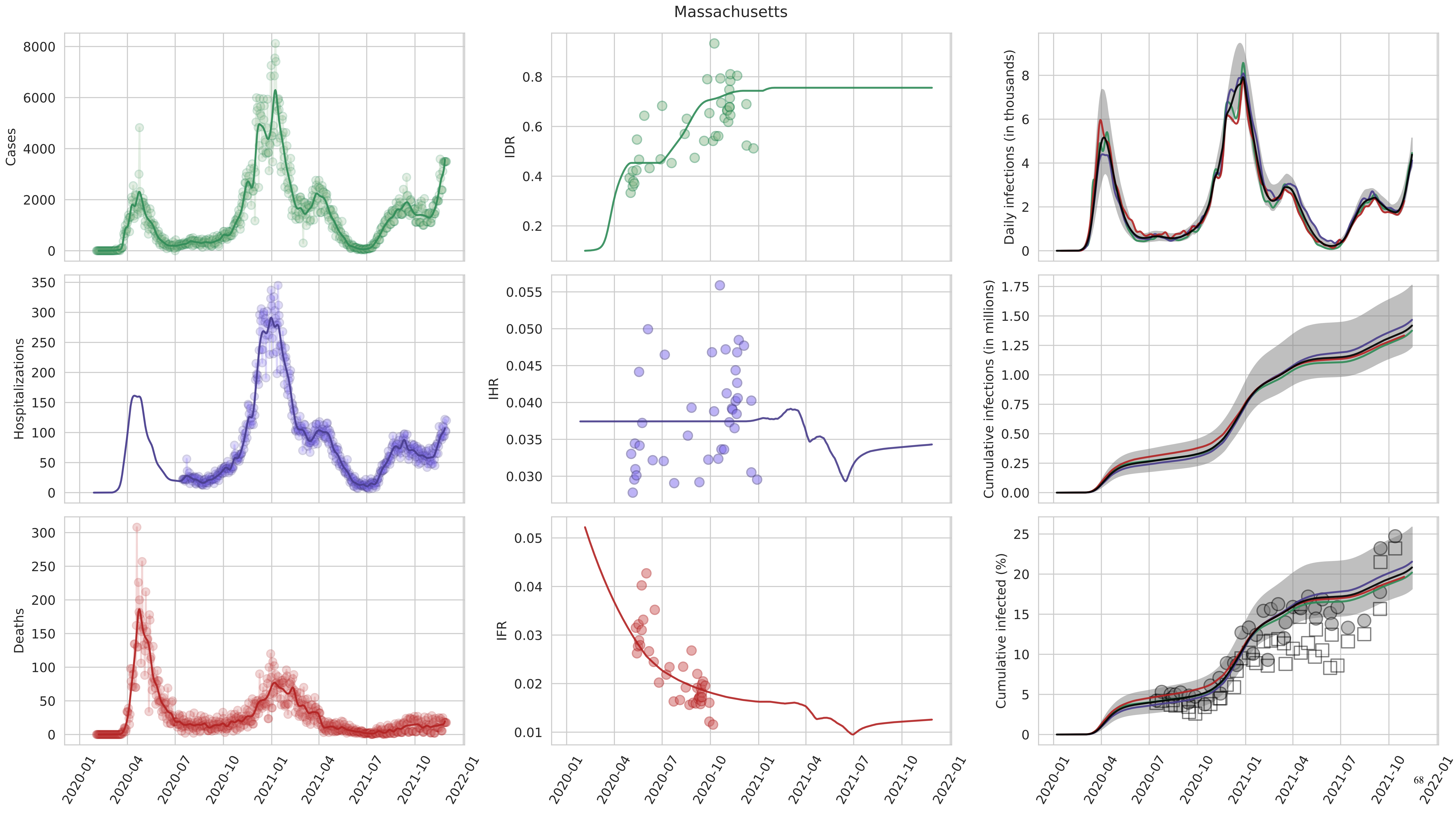
Massachusetts
Cases
IDR
Daily infections (in thousands)
Hospitalizations
IHR
Cumulative infections (in millions)
Deaths
IFR
Cumulative infected (%)
2020-01
2020-04
2020-07
2020-10
2021-01
2021-04
2021-07
2021-10
2022-01

Michigan

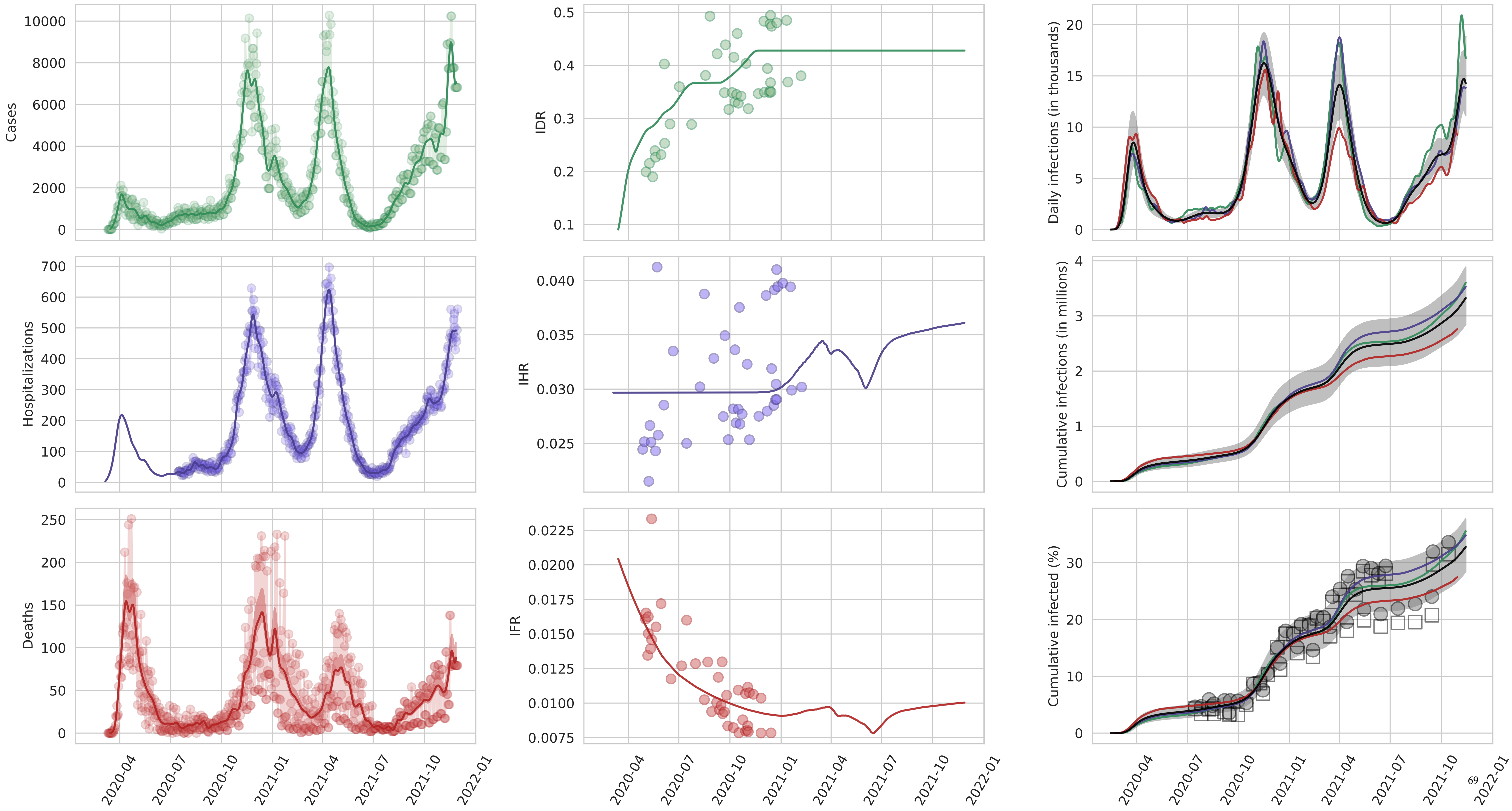
Cases
IDR
Daily infections (in thousands)
Hospitalizations
IHR
Cumulative infections (in millions)
Deaths
IFR
Cumulative infected (%)
2020-04
2020-07
2020-10
2021-01
2021-04
2021-07
2021-10
2022-01

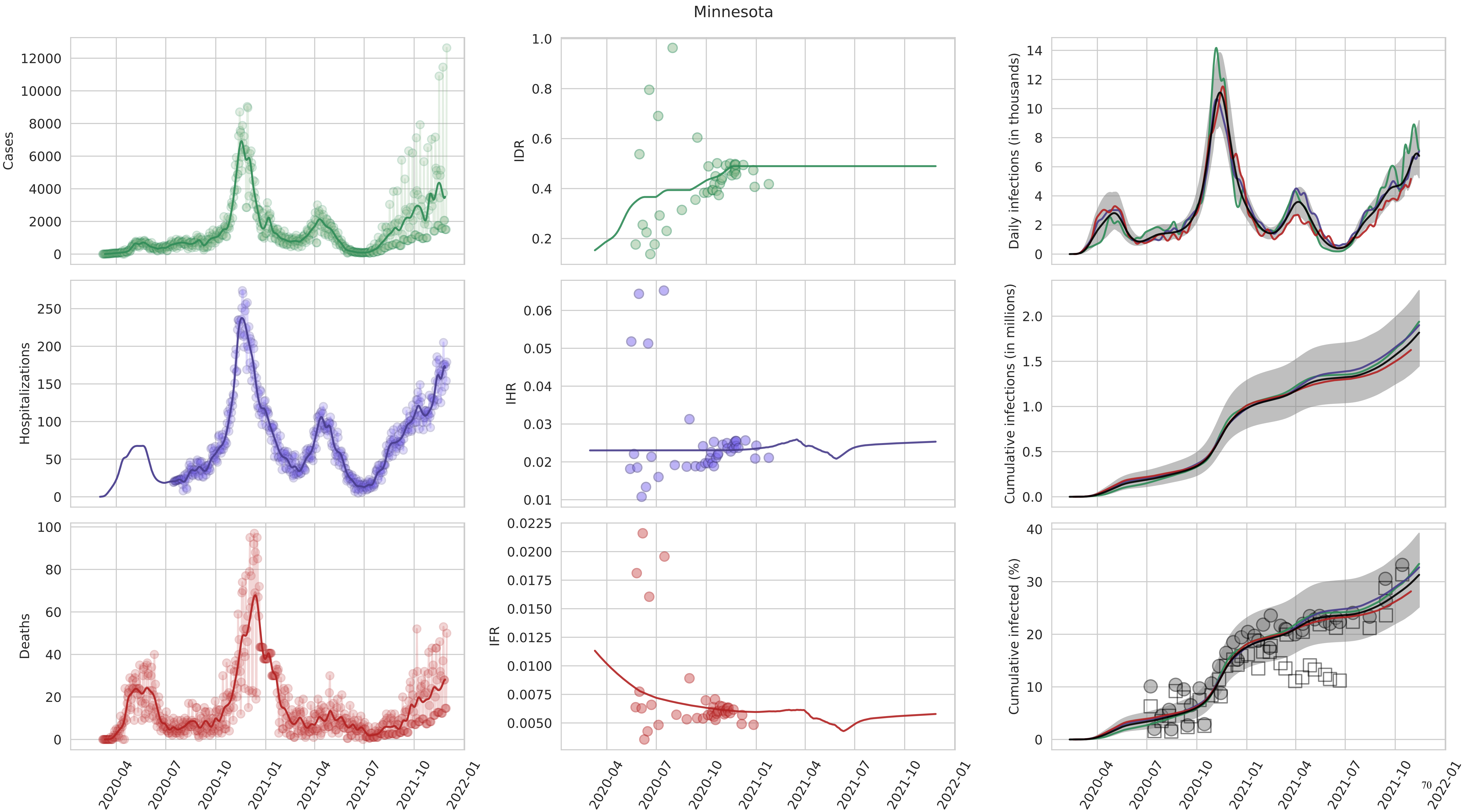
Minnesota
Cases
Hospitalizations
Deaths
IDR
IHR
IFR
Daily infections (in thousands)
Cumulative infections (in millions)
Cumulative infected (%)
2020-04
2020-07
2020-10
2021-01
2021-04
2021-07
2021-10
2022-01

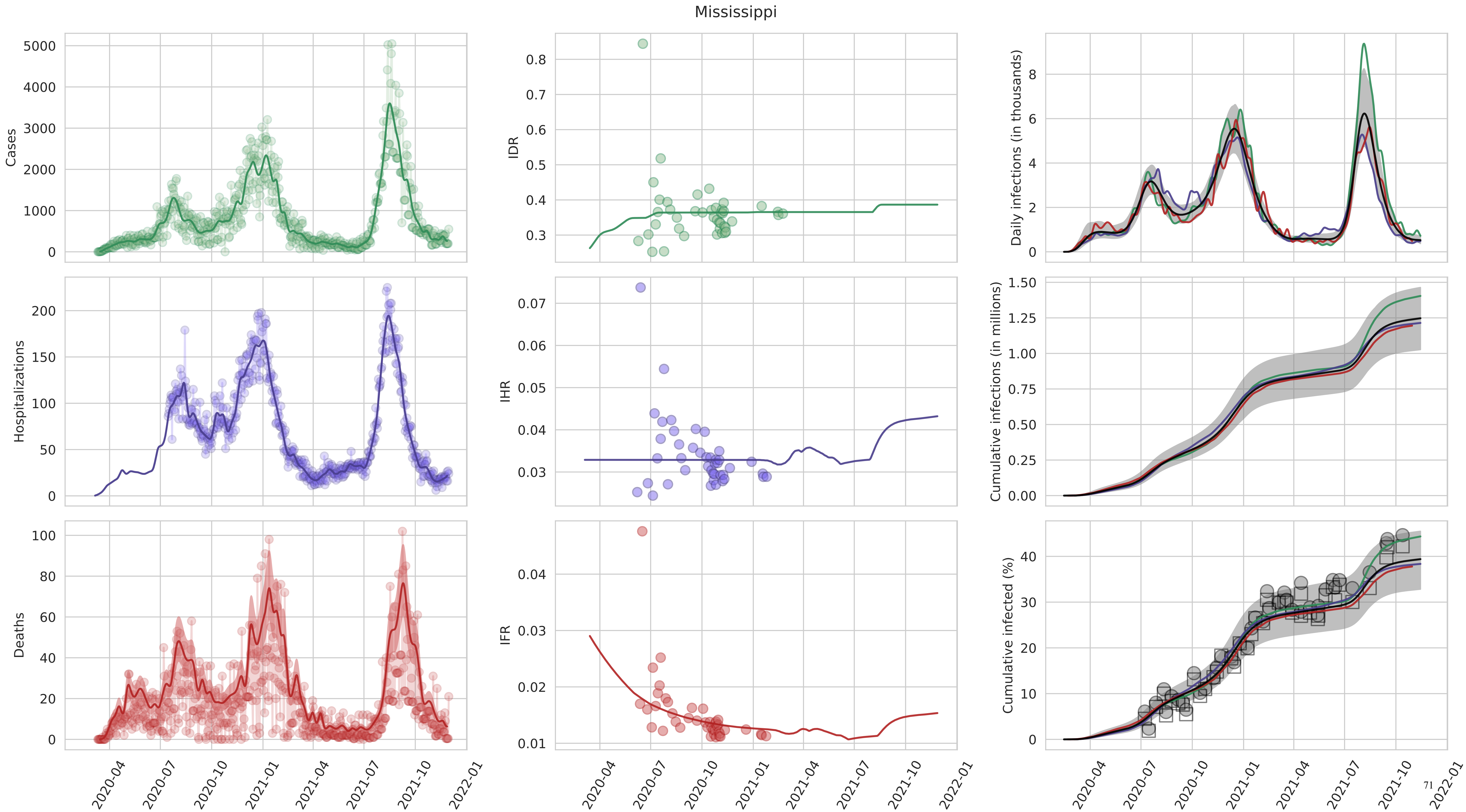

Mississippi
Cases
IDR
Daily infections (in thousands)
Hospitalizations
IHR
Cumulative infections (in millions)
Deaths
IFR
Cumulative infected (%)
2020-04
2020-07
2020-10
2021-01
2021-04
2021-07
2021-10
2022-01

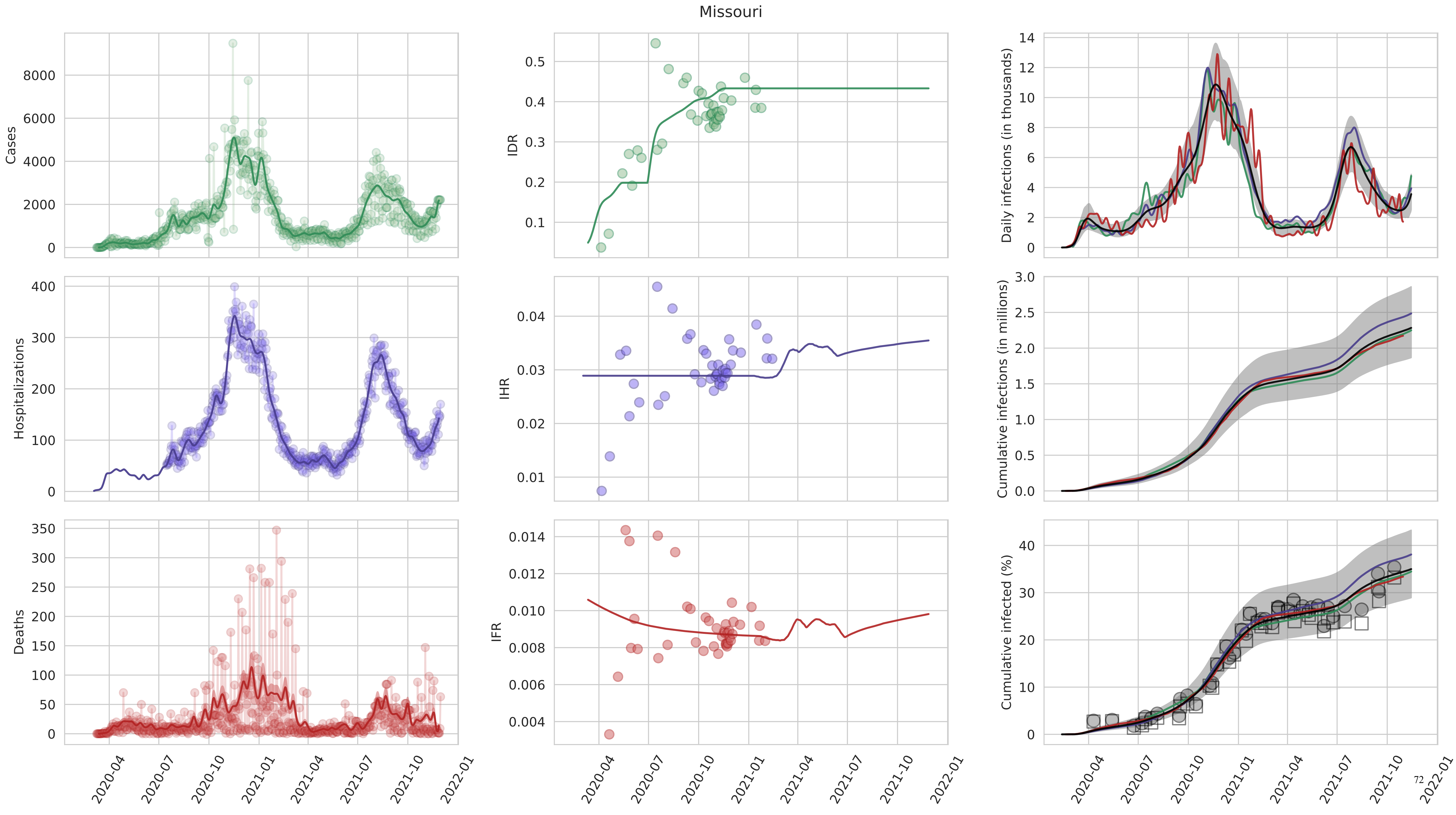

Missouri
Cases
Hospitalizations
Deaths
IDR
IHR
IFR
Daily infections (in thousands)
Cumulative infections (in millions)
Cumulative infected (%)
2020-04
2020-07
2020-10
2021-01
2021-04
2021-07
2021-10
2022-01

Montana

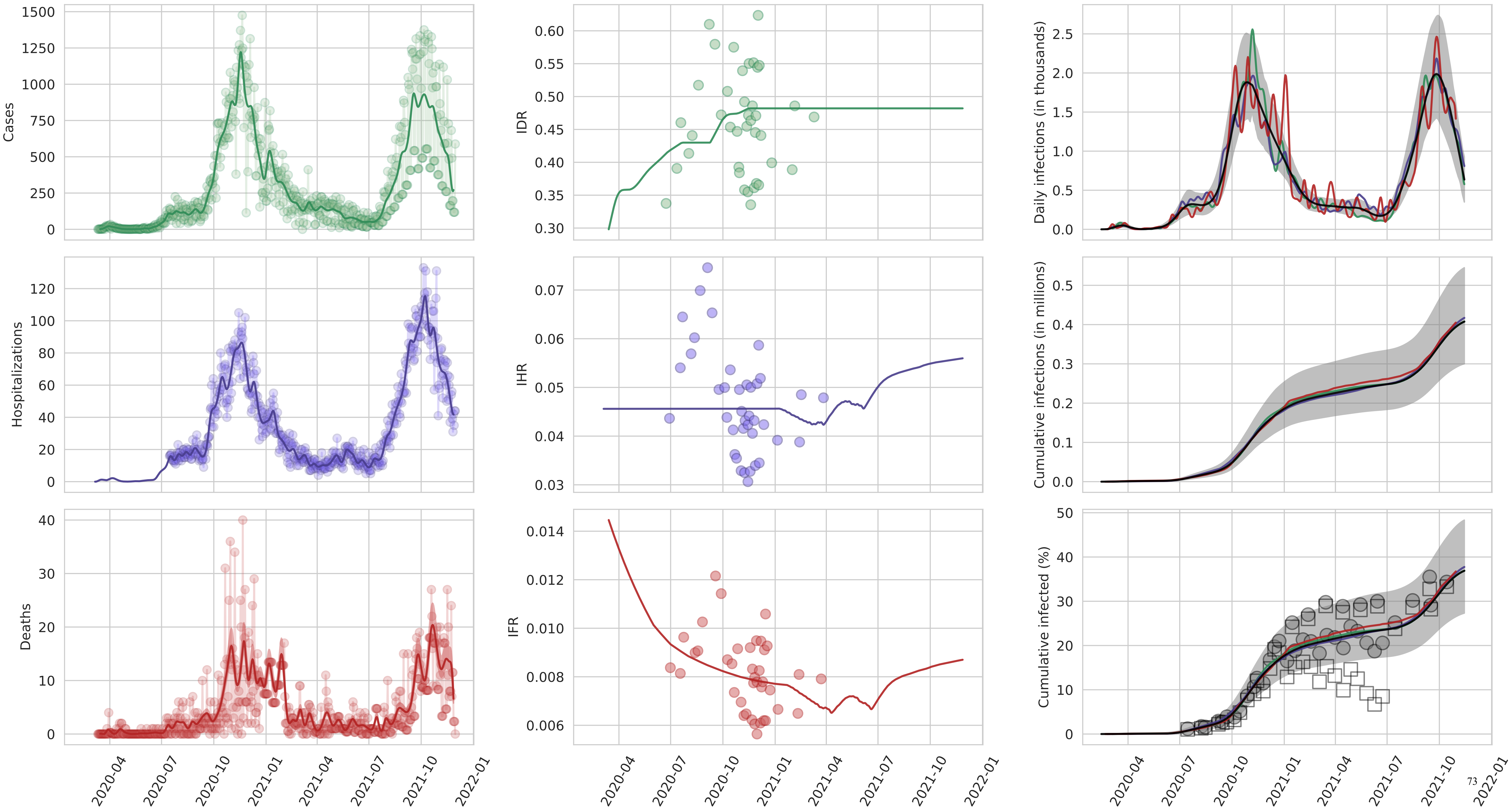

Cases
Hospitalizations
Deaths
IDR
IHR
IFR
Daily infections (in thousands)
Cumulative infections (in millions)
Cumulative infected (%)
2020-04
2020-07
2020-10
2021-01
2021-04
2021-07
2021-10
2022-01

Nebraska

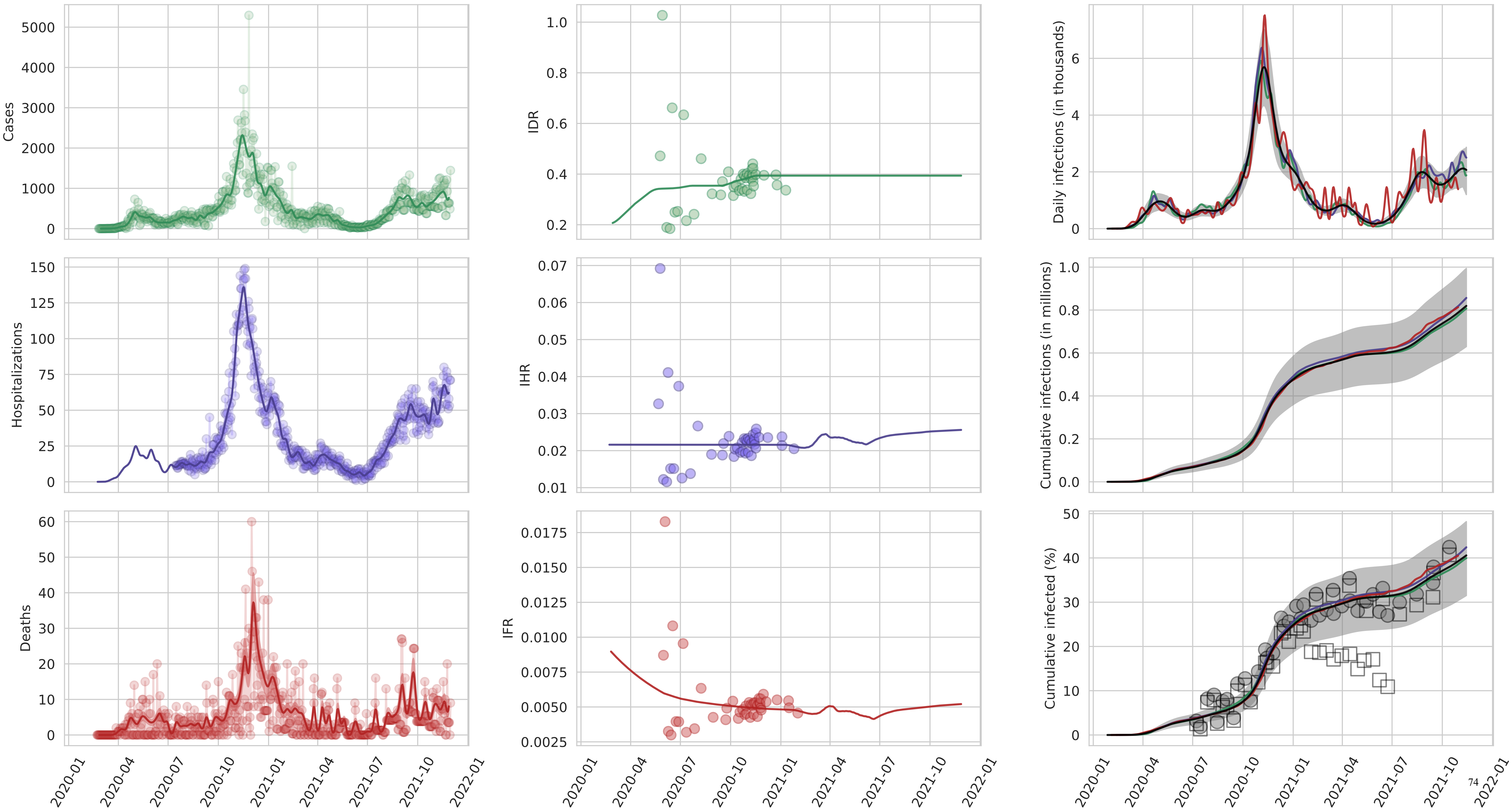

Cases
IDR
Daily infections (in thousands)
Hospitalizations
IHR
Cumulative infections (in millions)
Deaths
IFR
Cumulative infected (%)
2020-01
2020-04
2020-07
2020-10
2021-01
2021-04
2021-07
2021-10
2022-01

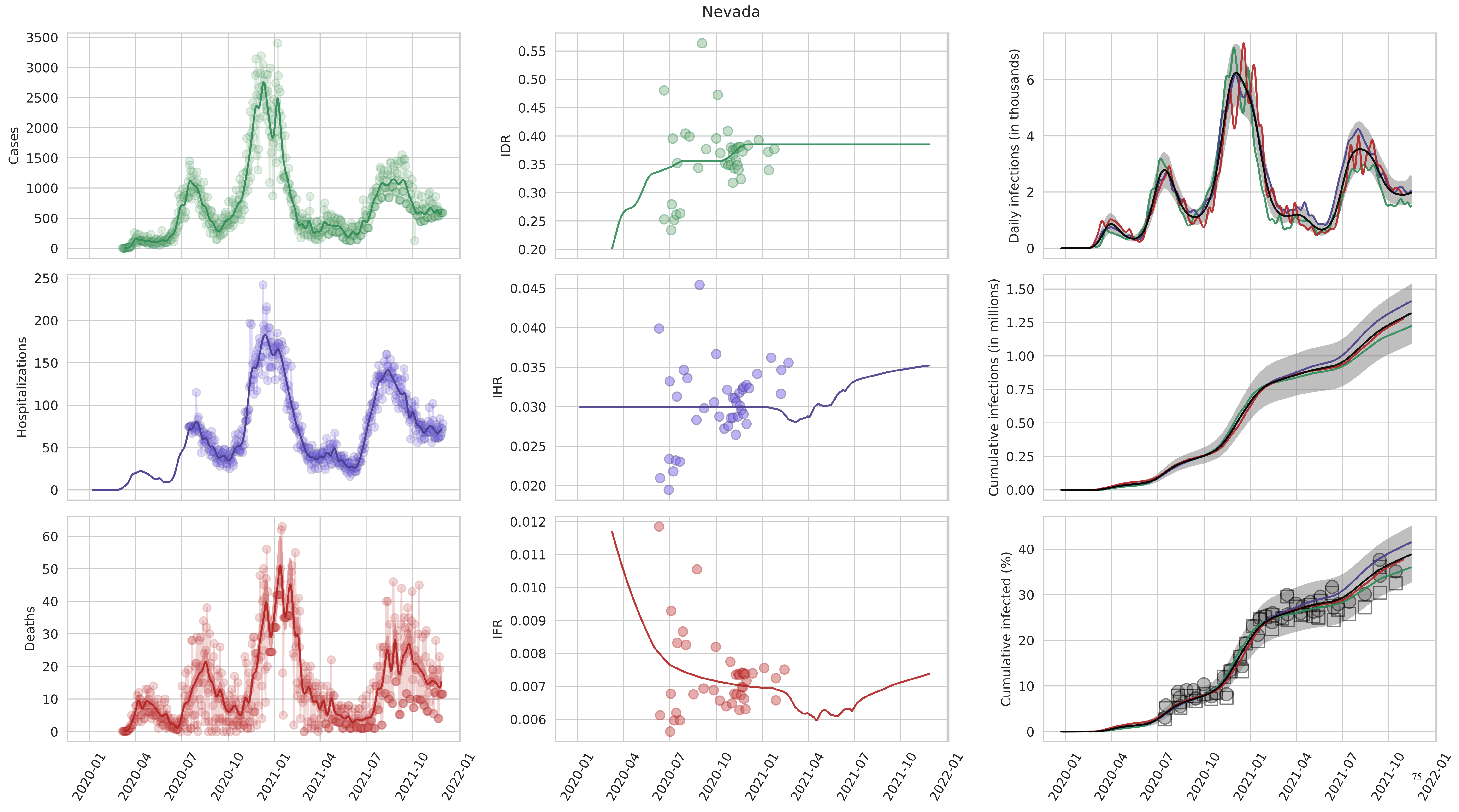
Nevada
Cases
Hospitalizations
Deaths
IDR
IHR
IFR
Daily infections (in thousands)
Cumulative infections (in millions)
Cumulative infected (%)
2020-01
2020-04
2020-07
2020-10
2021-01
2021-04
2021-07
2021-10
2022-01

New Hampshire

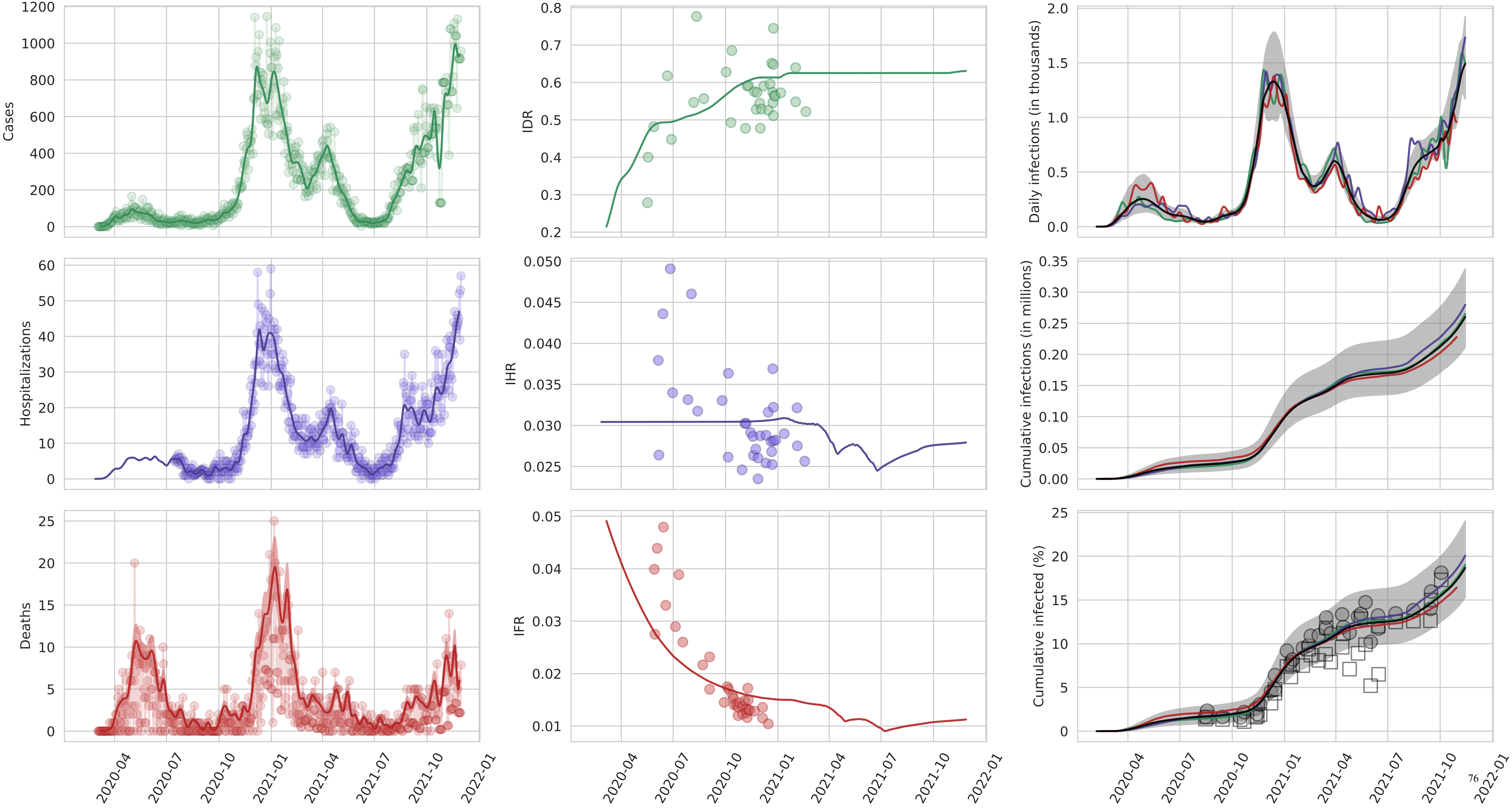

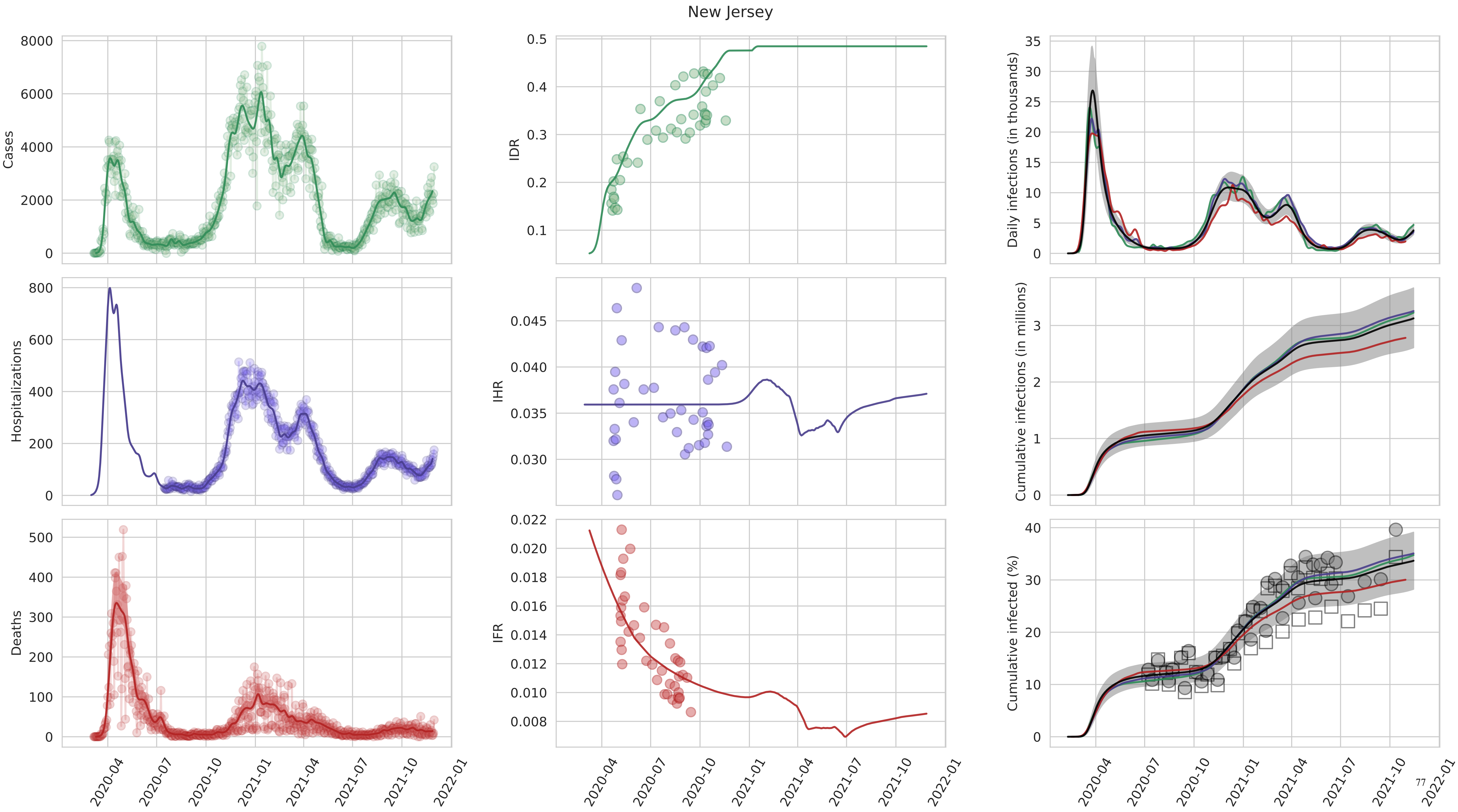
New Jersey
Cases
Hospitalizations
Deaths
IDR
IHR
IFR
Daily infections (in thousands)
Cumulative infections (in millions)
Cumulative infected (%)
2020-04
2020-07
2020-10
2021-01
2021-04
2021-07
2021-10
2022-01

New Mexico

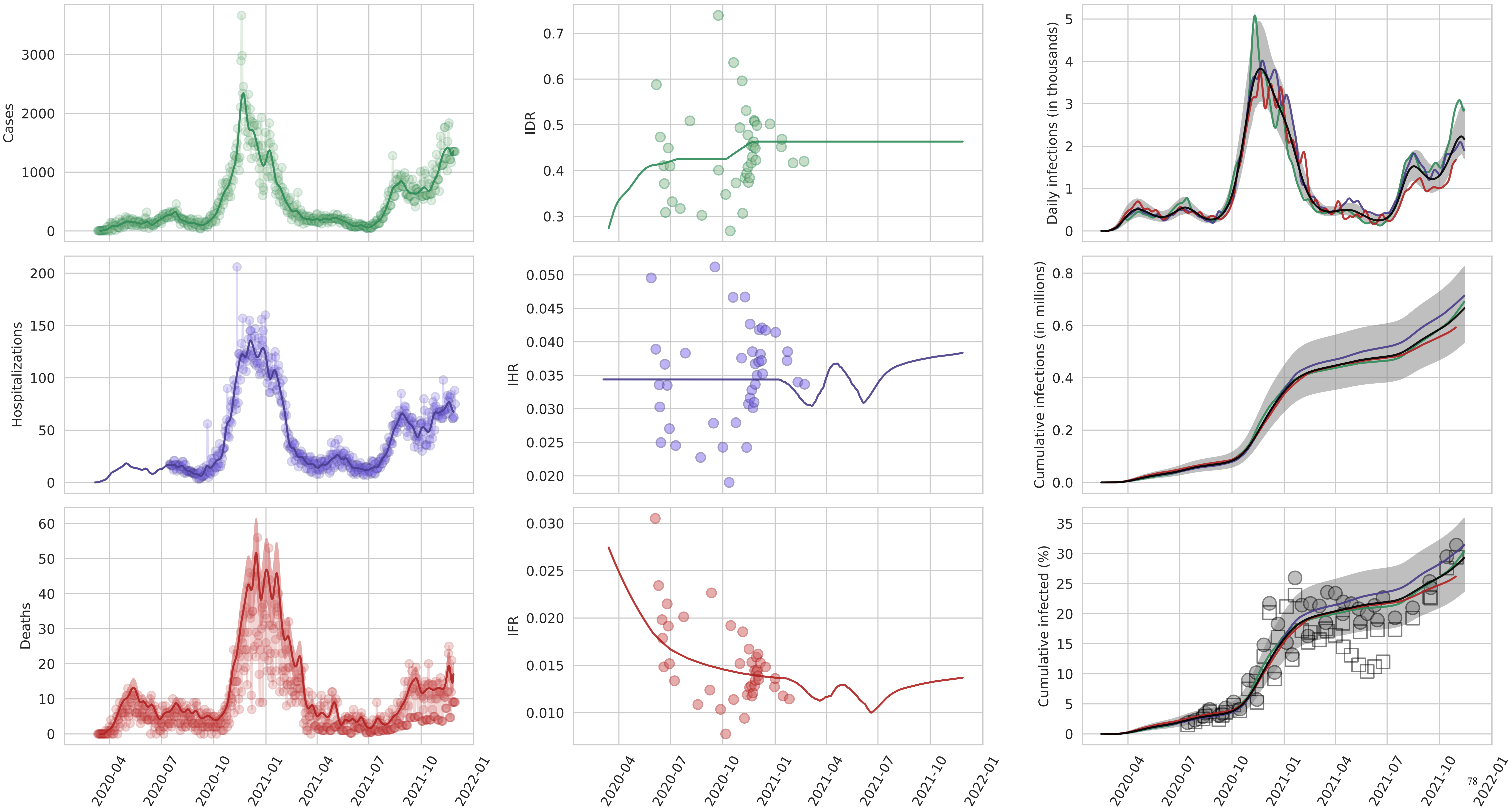

Cases
IDR
Daily infections (in thousands)
Hospitalizations
IHR
Cumulative infections (in millions)
Deaths
IFR
Cumulative infected (%)
2020-04
2020-07
2020-10
2021-01
2021-04
2021-07
2021-10
2022-01

New York

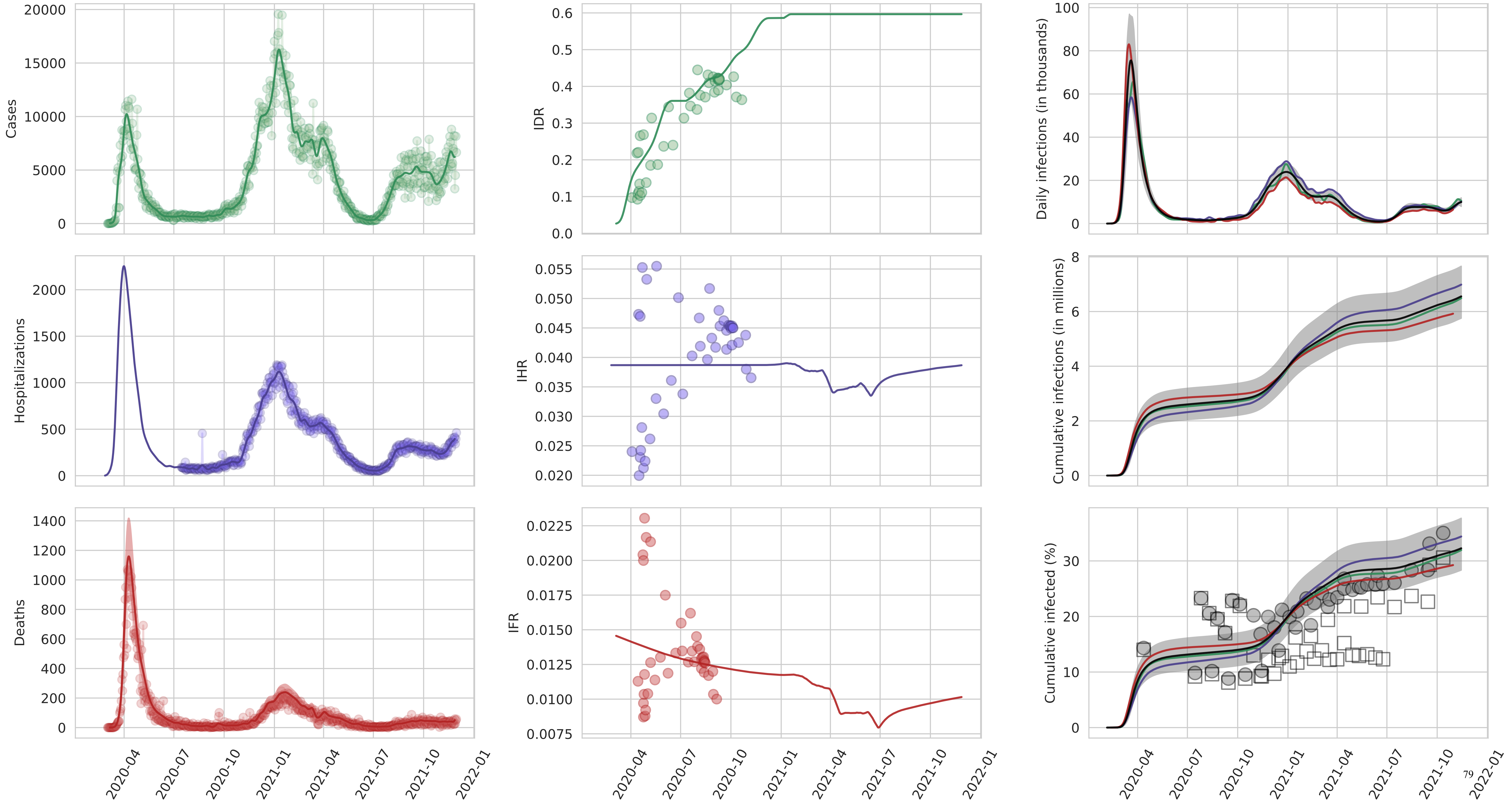
Cases
Hospitalizations
Deaths
IDR
IHR
IFR
Daily infections (in thousands)
Cumulative infections (in millions)
Cumulative infected (%)
2020-04
2020-07
2020-10
2021-01
2021-04
2021-07
2021-10
2022-01

North Carolina

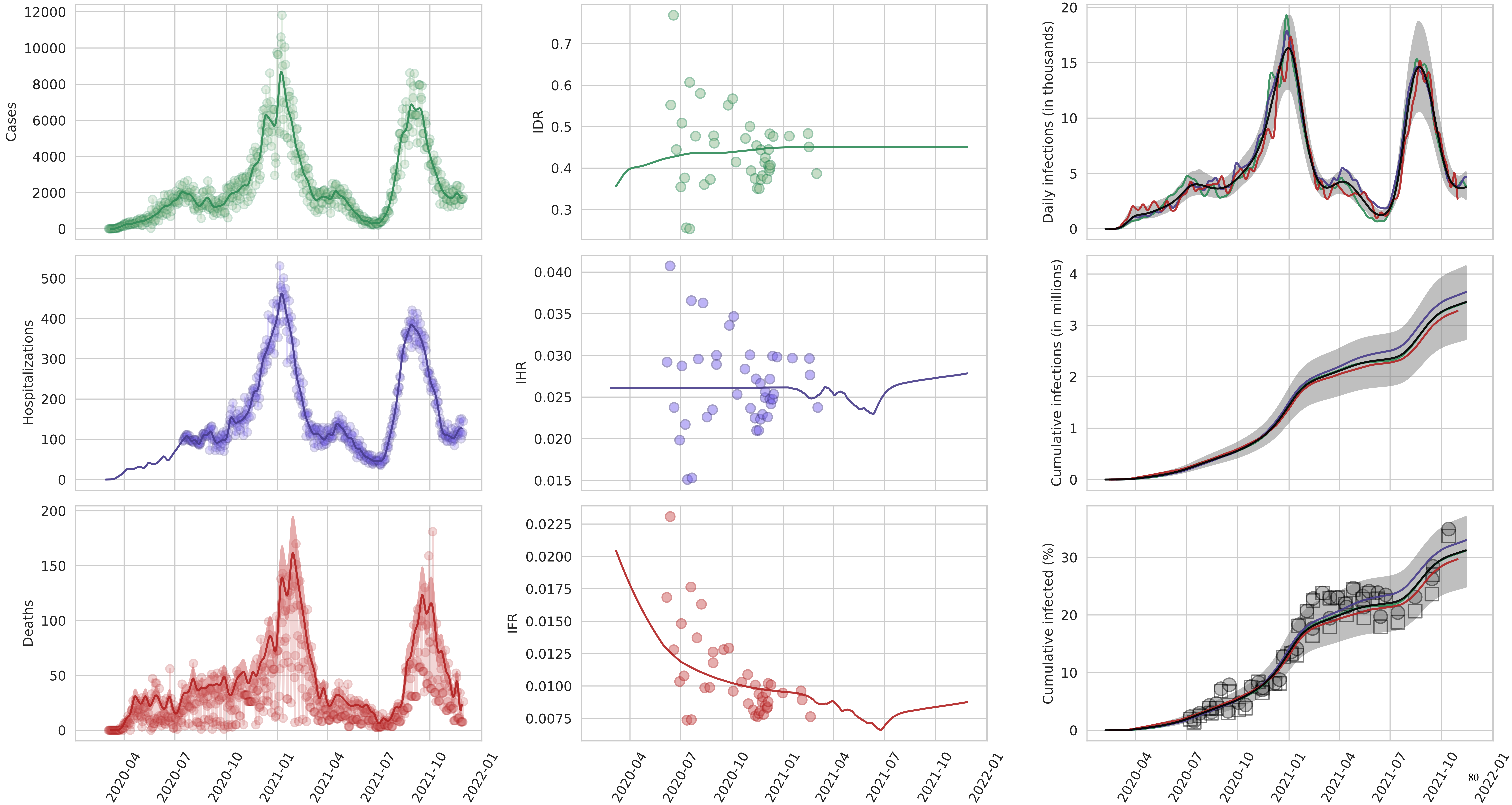

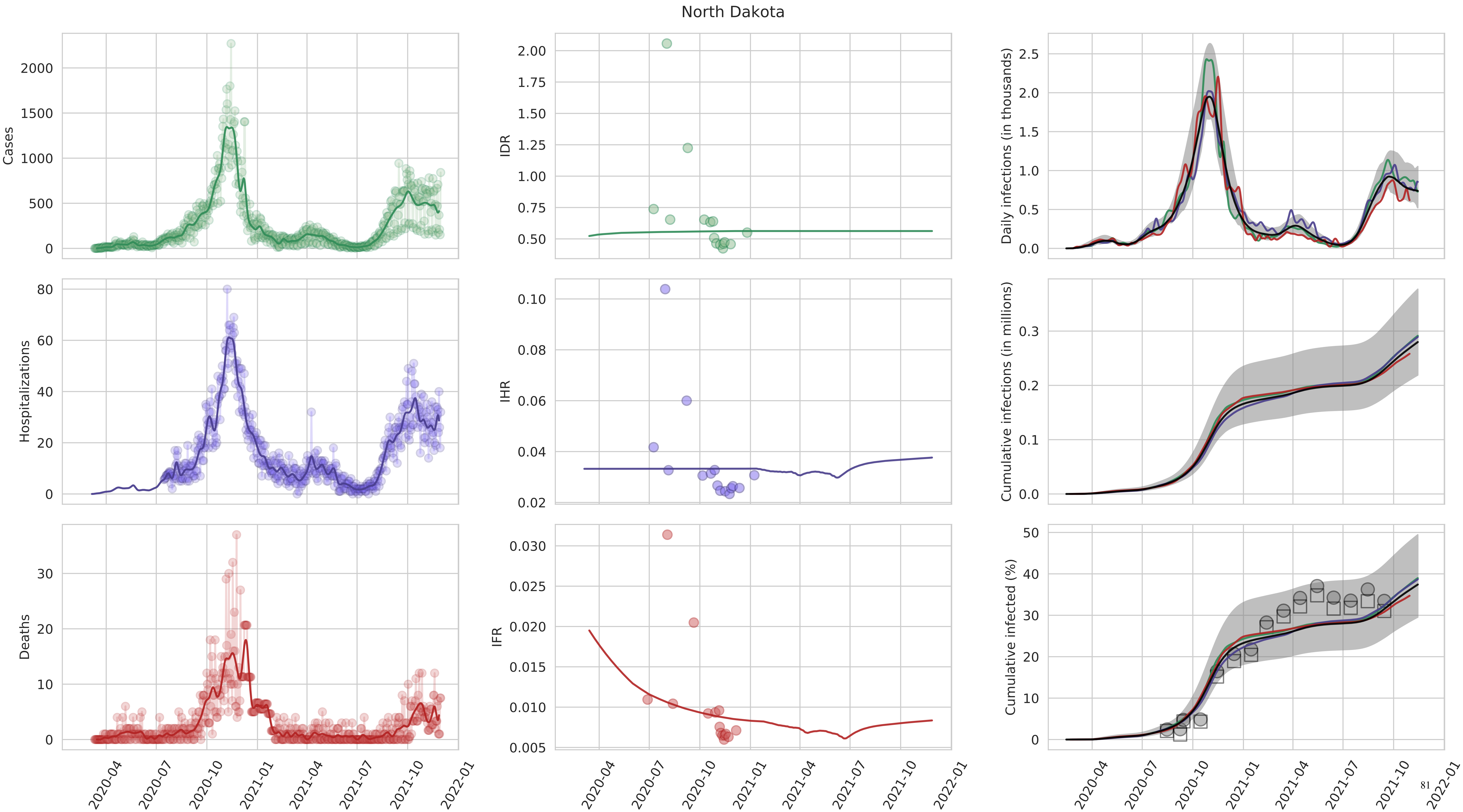
North Dakota
Cases
Hospitalizations
Deaths
IDR
IHR
IFR
Daily infections (in thousands)
Cumulative infections (in millions)
Cumulative infected (%)

Ohio

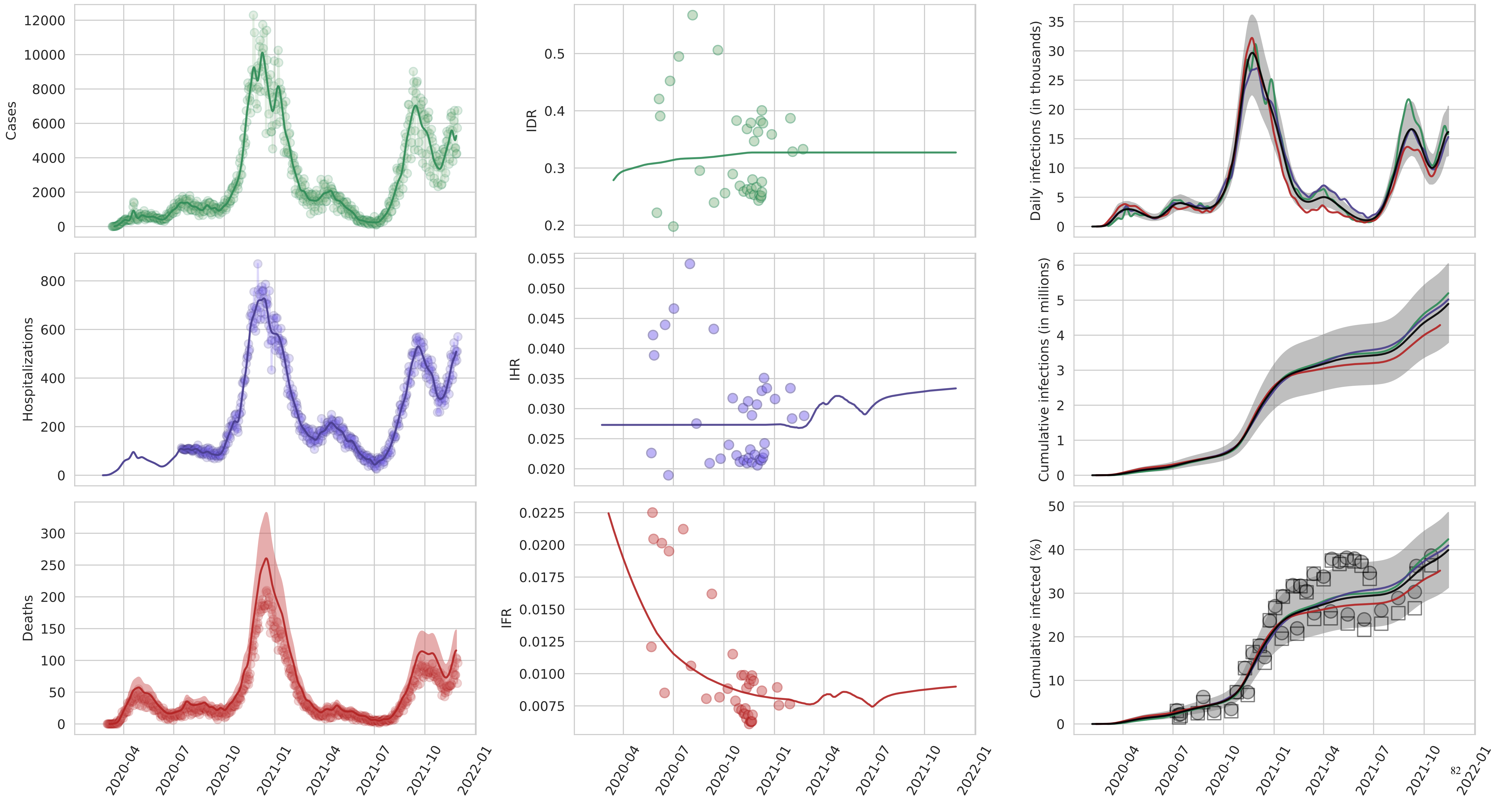

Cases
Hospitalizations
Deaths
IDR
IHR
IFR
Daily infections (in thousands)
Cumulative infections (in millions)
Cumulative infected (%)
2020-04
2020-07
2020-10
2021-01
2021-04
2021-07
2021-10
2022-01

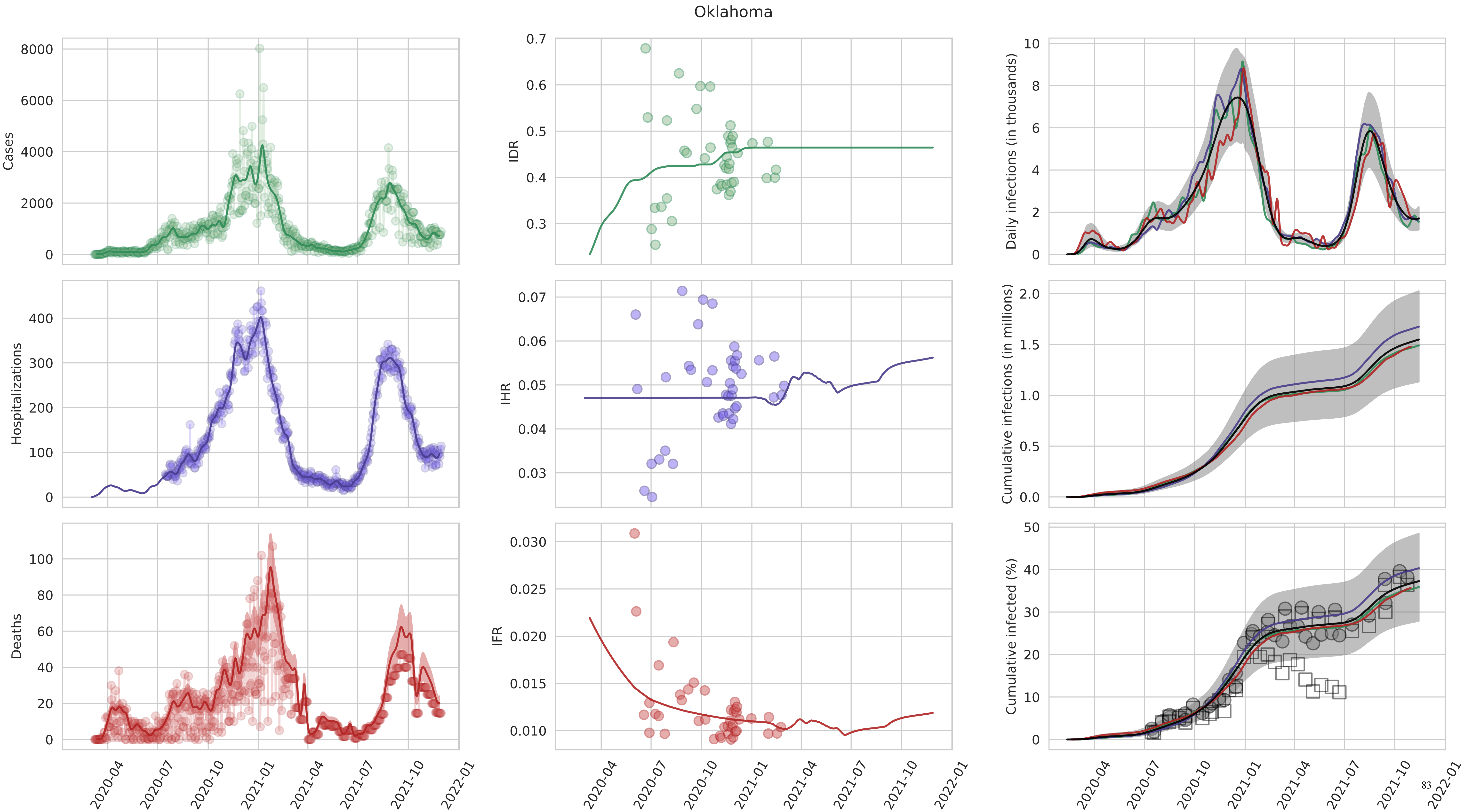

Oklahoma
Cases
Hospitalizations
Deaths
IDR
IHR
IFR
Daily infections (in thousands)
Cumulative infections (in millions)
Cumulative infected (%)
2020-04
2020-07
2020-10
2021-01
2021-04
2021-07
2021-10
2022-01

Oregon

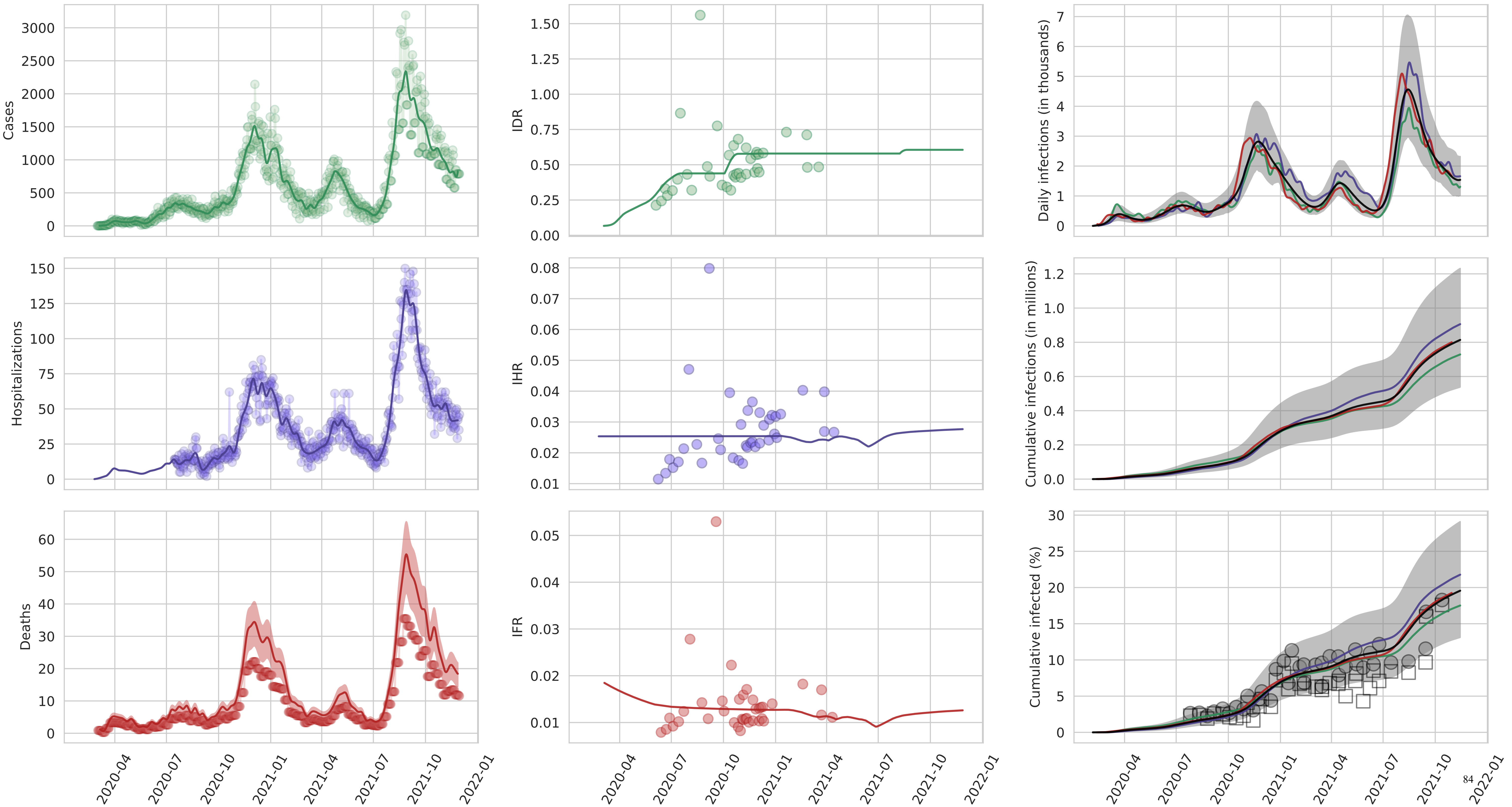

Cases
IDR
Daily infections (in thousands)
Hospitalizations
IHR
Cumulative infections (in millions)
Deaths
IFR
Cumulative infected (%)
2020-04
2020-07
2020-10
2021-01
2021-04
2021-07
2021-10
2022-01

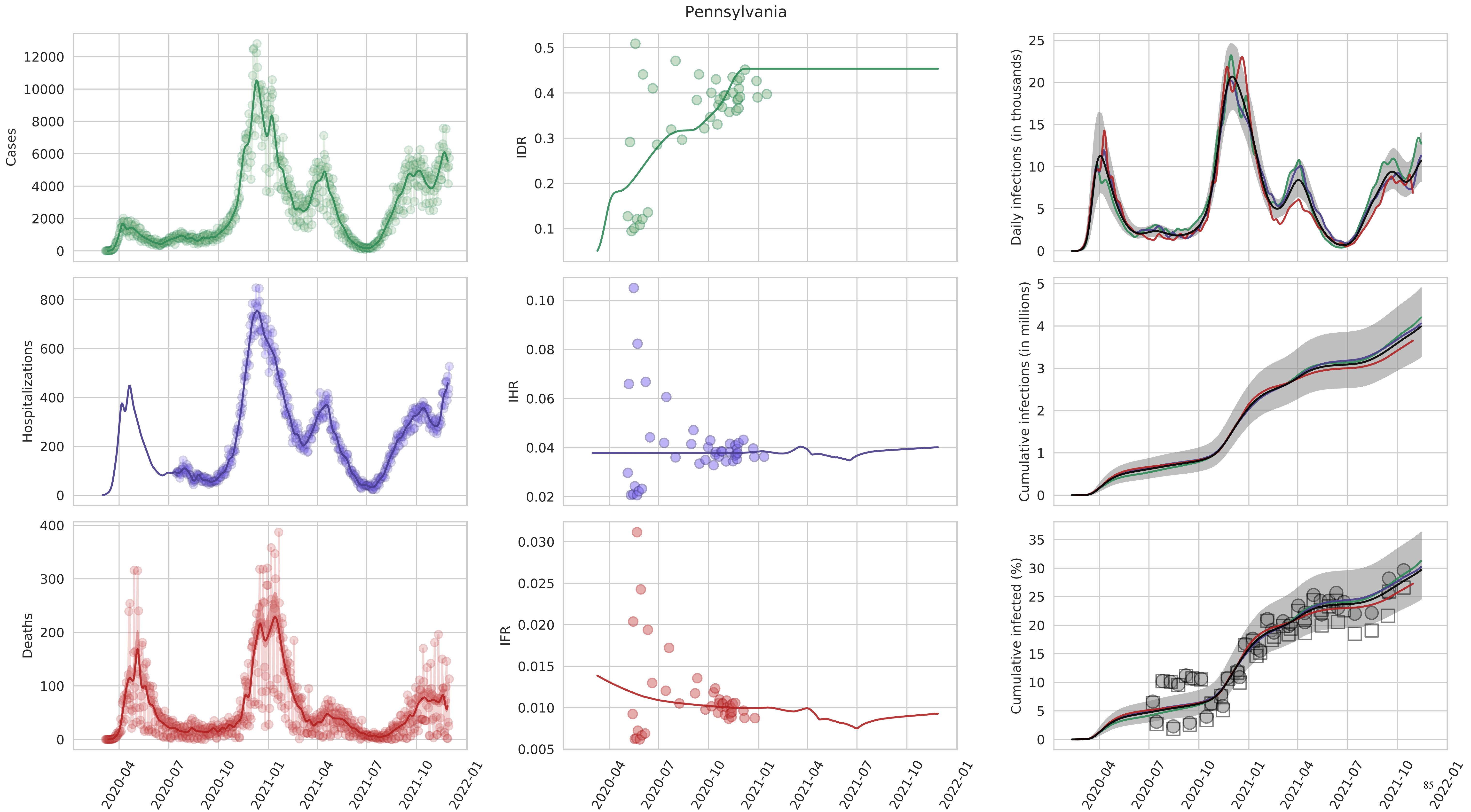
Pennsylvania
Cases
Hospitalizations
Deaths
IDR
IHR
IFR
Daily infections (in thousands)
Cumulative infections (in millions)
Cumulative infected (%)
2020-04
2020-07
2020-10
2021-01
2021-04
2021-07
2021-10
2022-01

Rhode Island

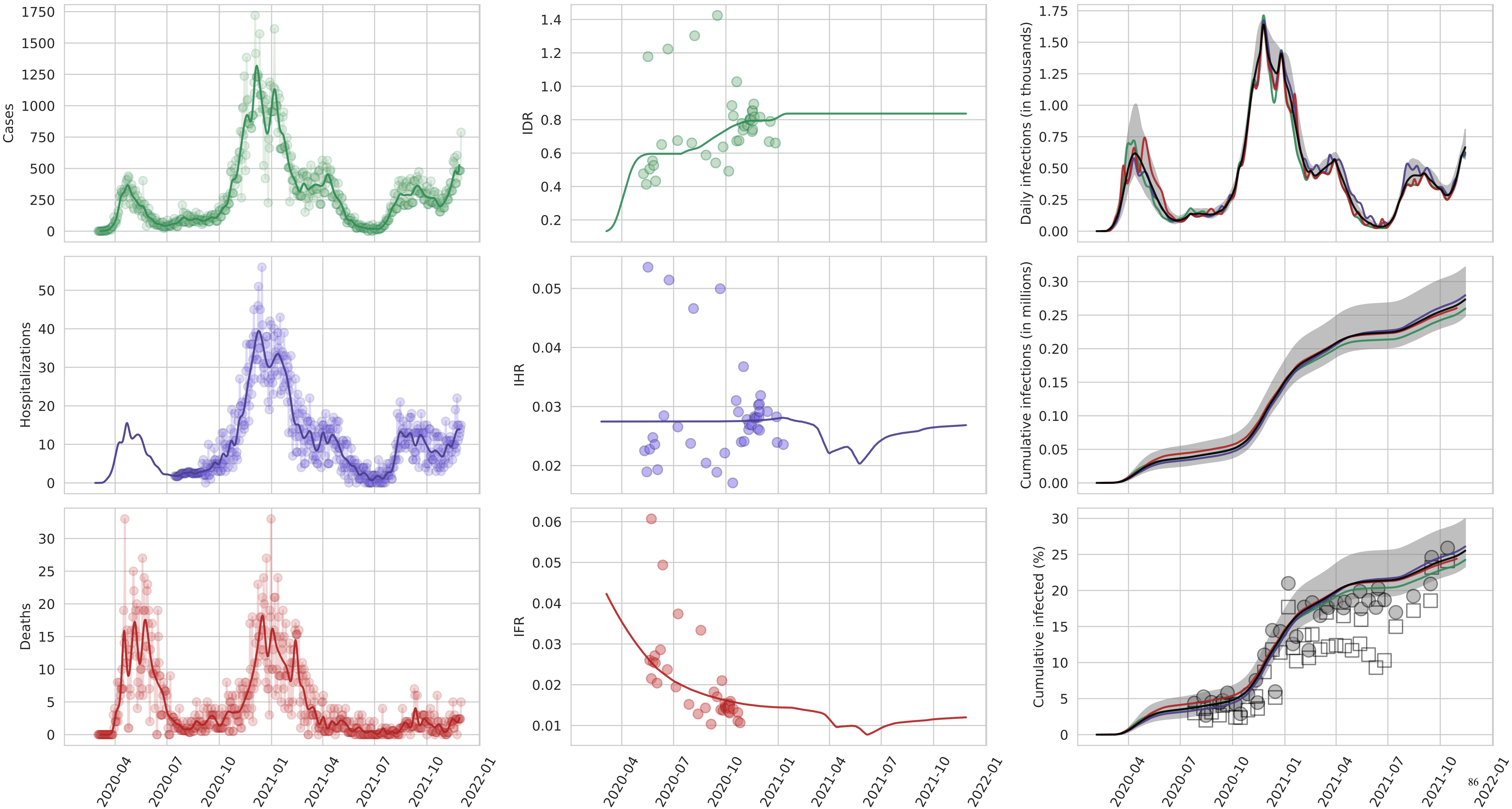

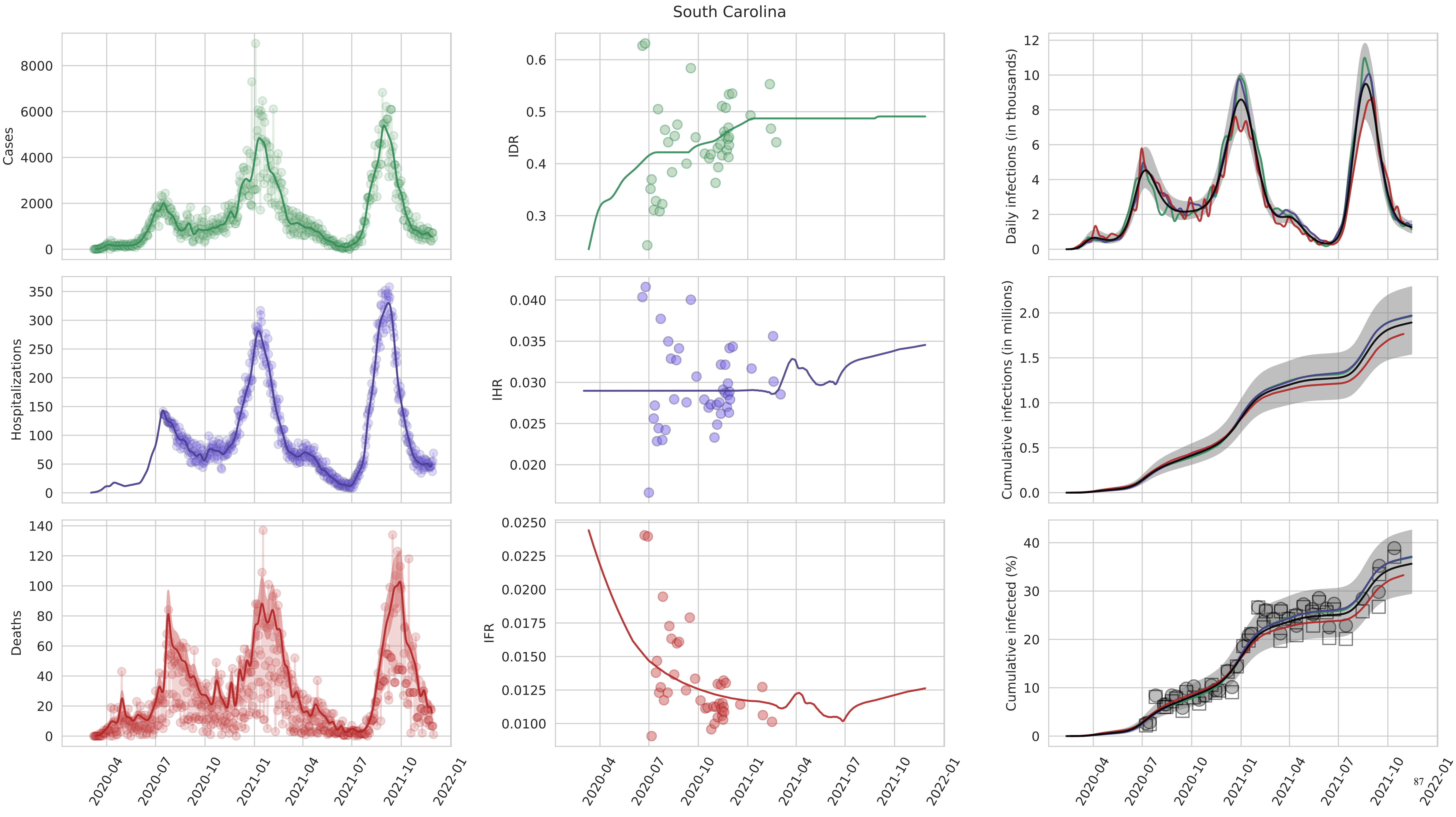
South Carolina
Cases
IDR
Daily infections (in thousands)
Hospitalizations
IHR
Cumulative infections (in millions)
Deaths
IFR
Cumulative infected (%)
2020-04
2020-07
2020-10
2021-01
2021-04
2021-07
2021-10
2022-01

South Dakota

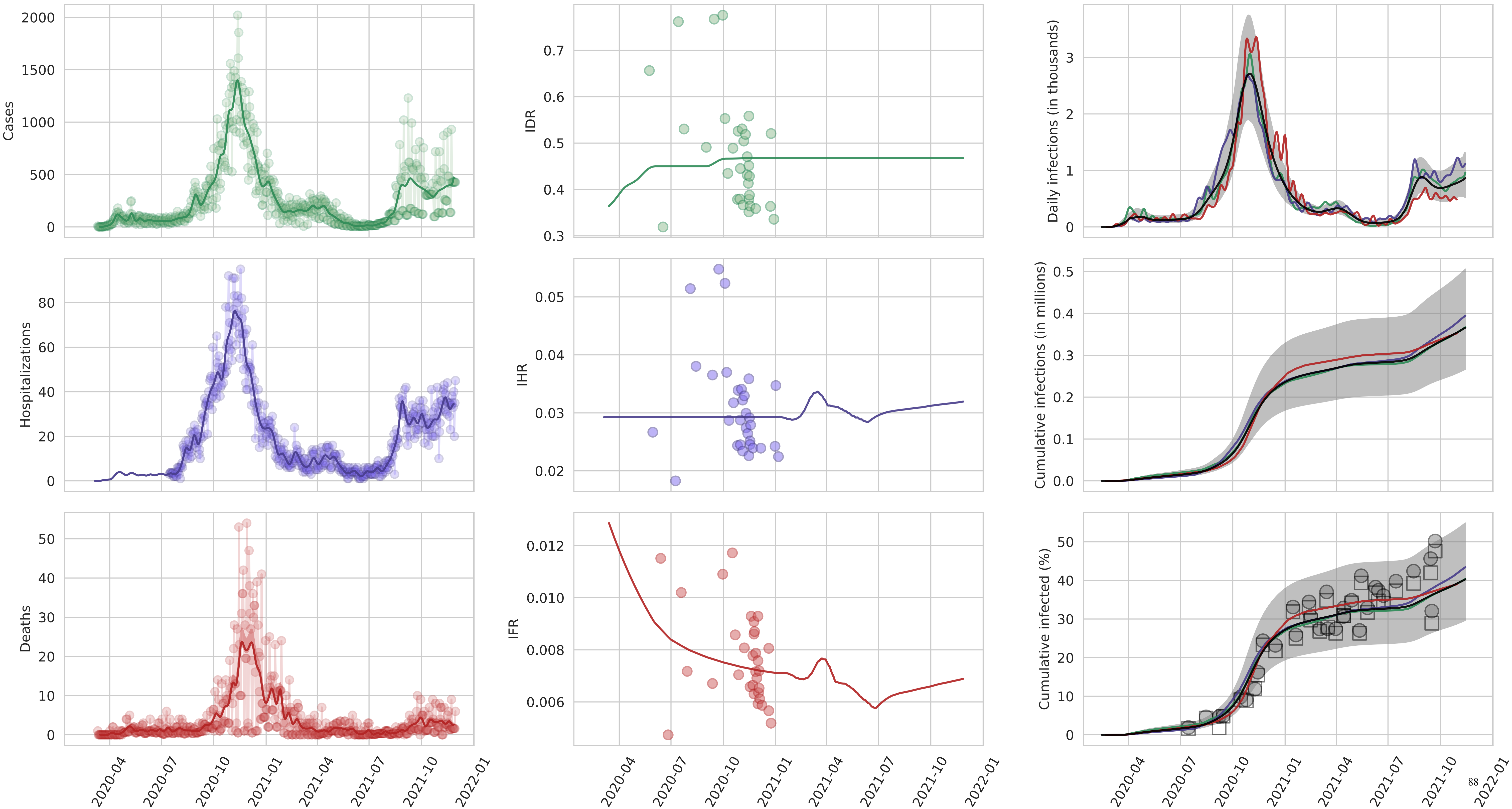

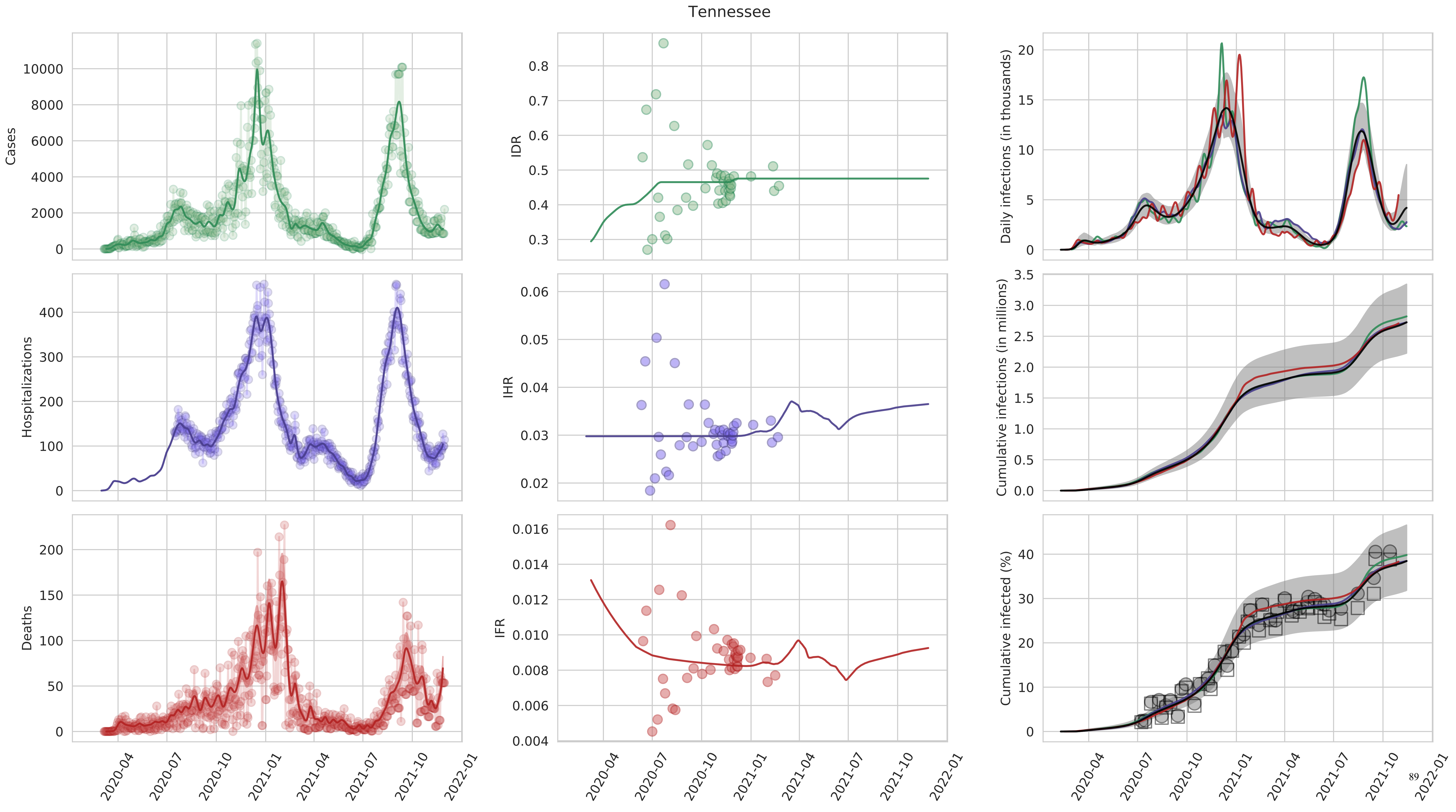
Tennessee
Cases
Hospitalizations
Deaths
IDR
IHR
IFR
Daily infections (in thousands)
Cumulative infections (in millions)
Cumulative infected (%)
2020-04
2020-07
2020-10
2021-01
2021-04
2021-07
2021-10
2022-01

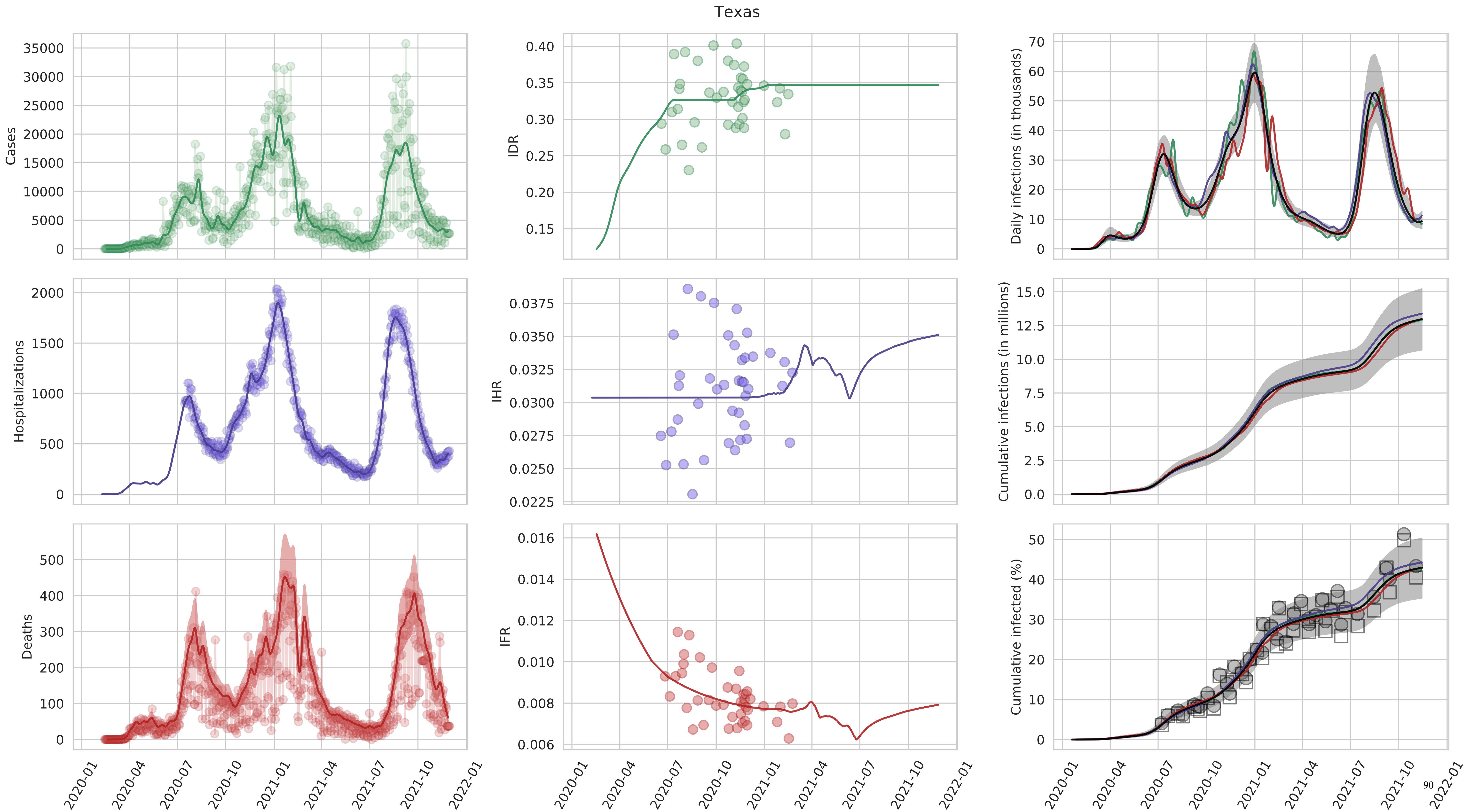

Texas
Cases
IDR
Daily infections (in thousands)
Hospitalizations
IHR
Cumulative infections (in millions)
Deaths
IFR
Cumulative infected (%)
2020-01
2020-04
2020-07
2020-10
2021-01
2021-04
2021-07
2021-10
2022-01

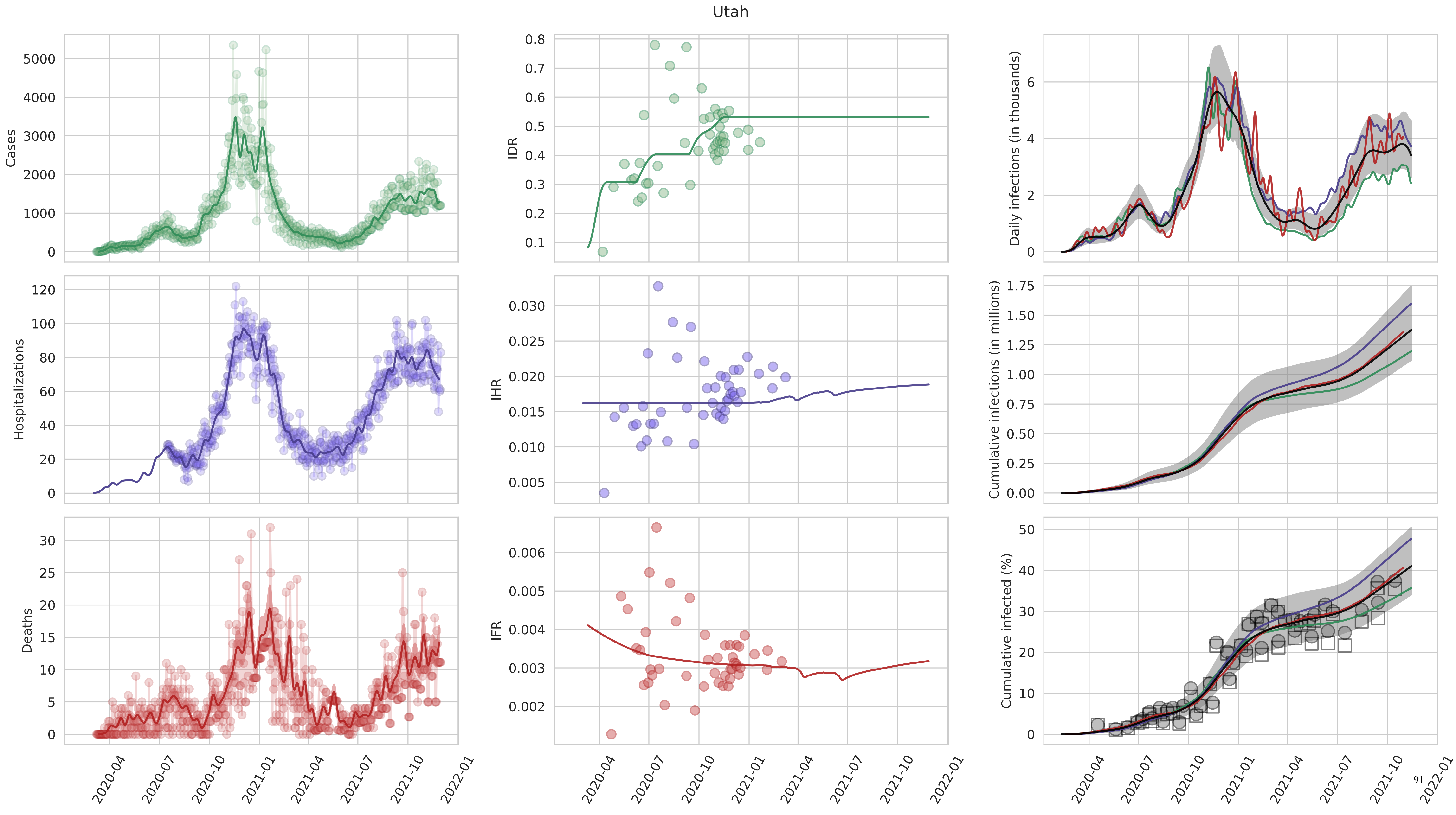
Utah
Cases
IDR
Daily infections (in thousands)
Hospitalizations
IHR
Cumulative infections (in millions)
Deaths
IFR
Cumulative infected (%)
2020-04
2020-07
2020-10
2021-01
2021-04
2021-07
2021-10
2022-01

Vermont

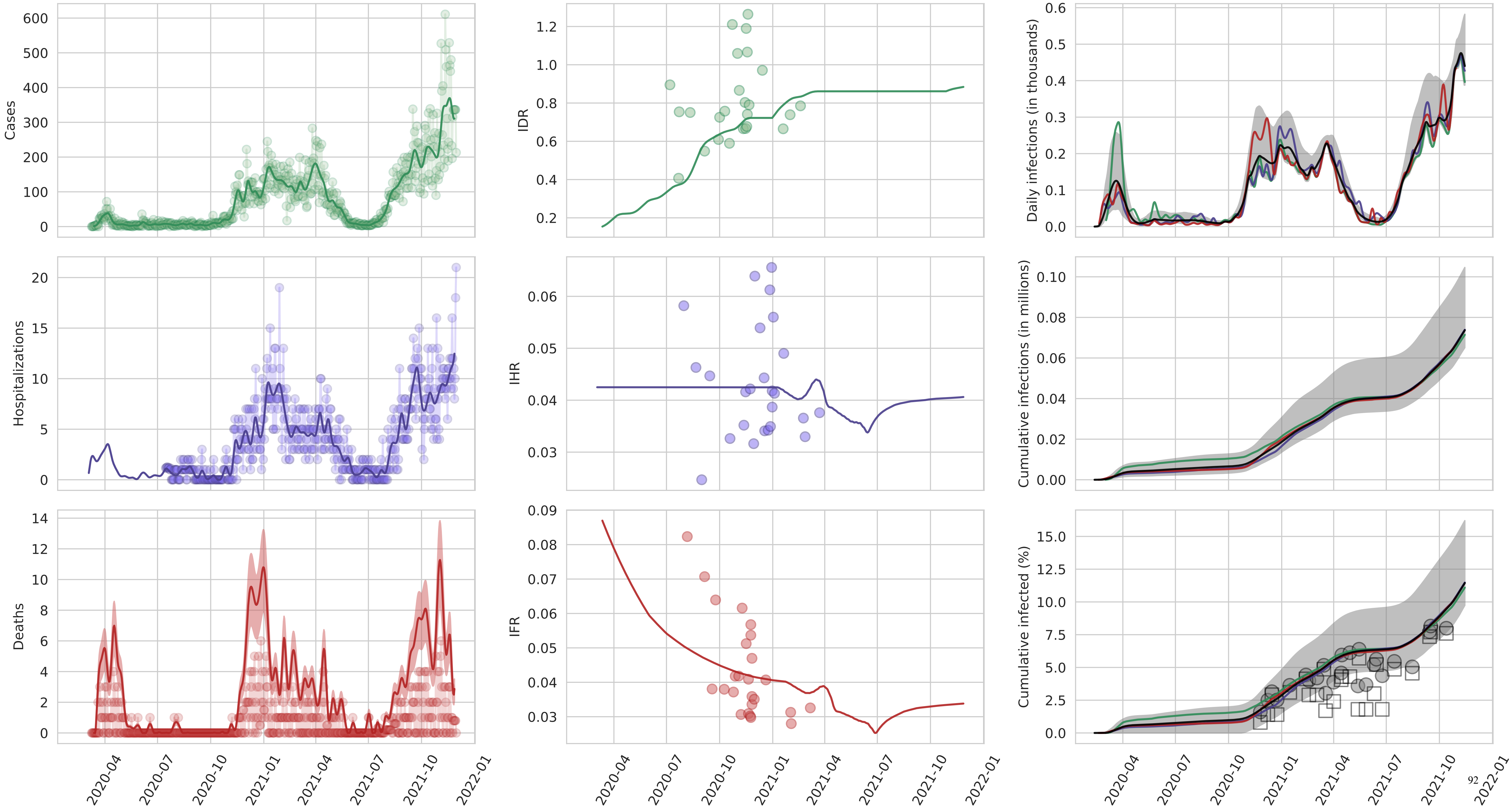
Cases
Hospitalizations
Deaths
IDR
IHR
IFR
Daily infections (in thousands)
Cumulative infections (in millions)
Cumulative infected (%)
2020-04
2020-07
2020-10
2021-01
2021-04
2021-07
2021-10
2022-01

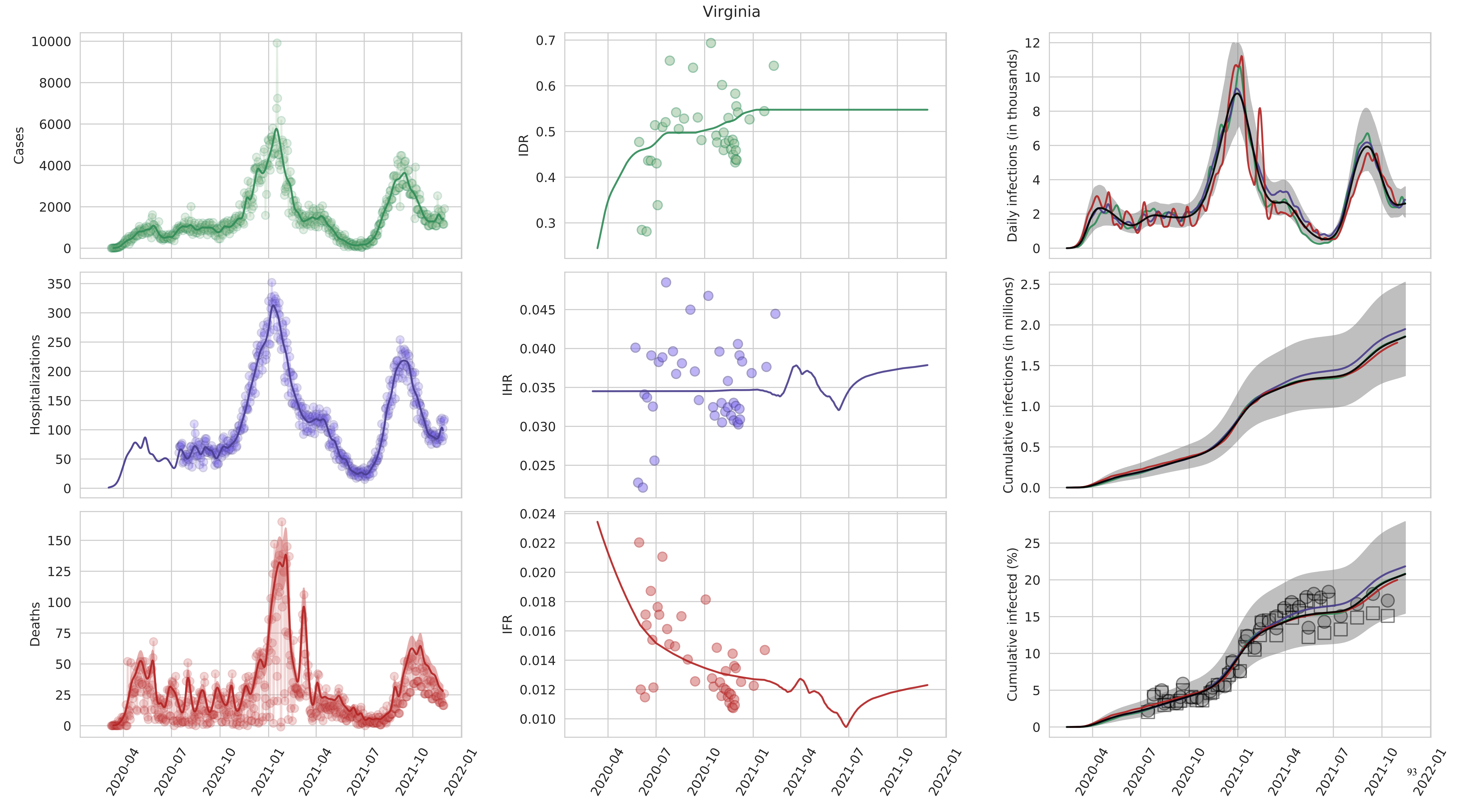
Virginia
Cases
Hospitalizations
Deaths
IDR
IHR
IFR
Daily infections (in thousands)
Cumulative infections (in millions)
Cumulative infected (%)
2020-04
2020-07
2020-10
2021-01
2021-04
2021-07
2021-10
2022-01

West Virginia

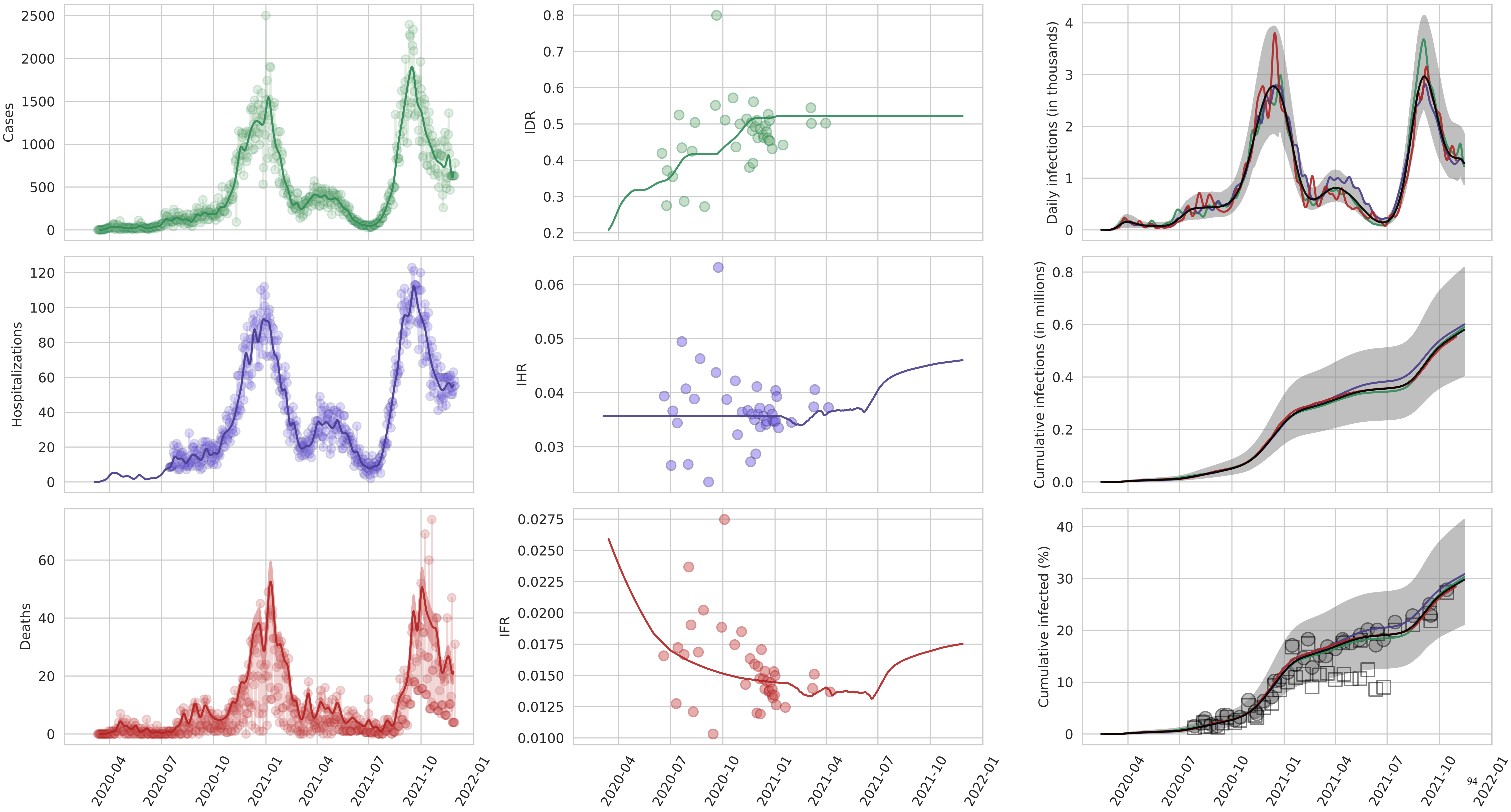

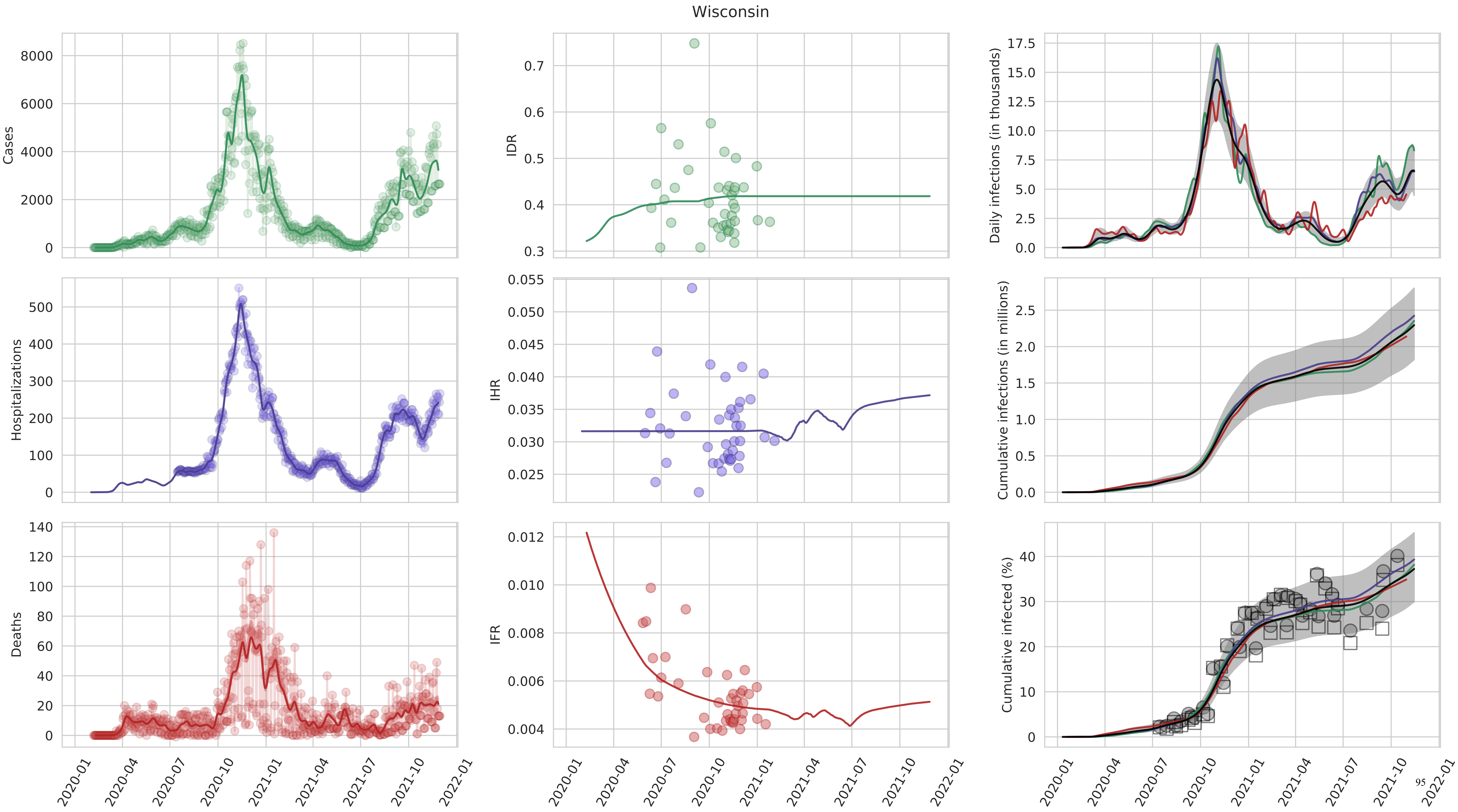

Wisconsin
Cases
IDR
Daily infections (in thousands)
Hospitalizations
IHR
Cumulative infections (in millions)
Deaths
IFR
Cumulative infected (%)
2020-01
2020-04
2020-07
2020-10
2021-01
2021-04
2021-07
2021-10
2022-01

Wyoming

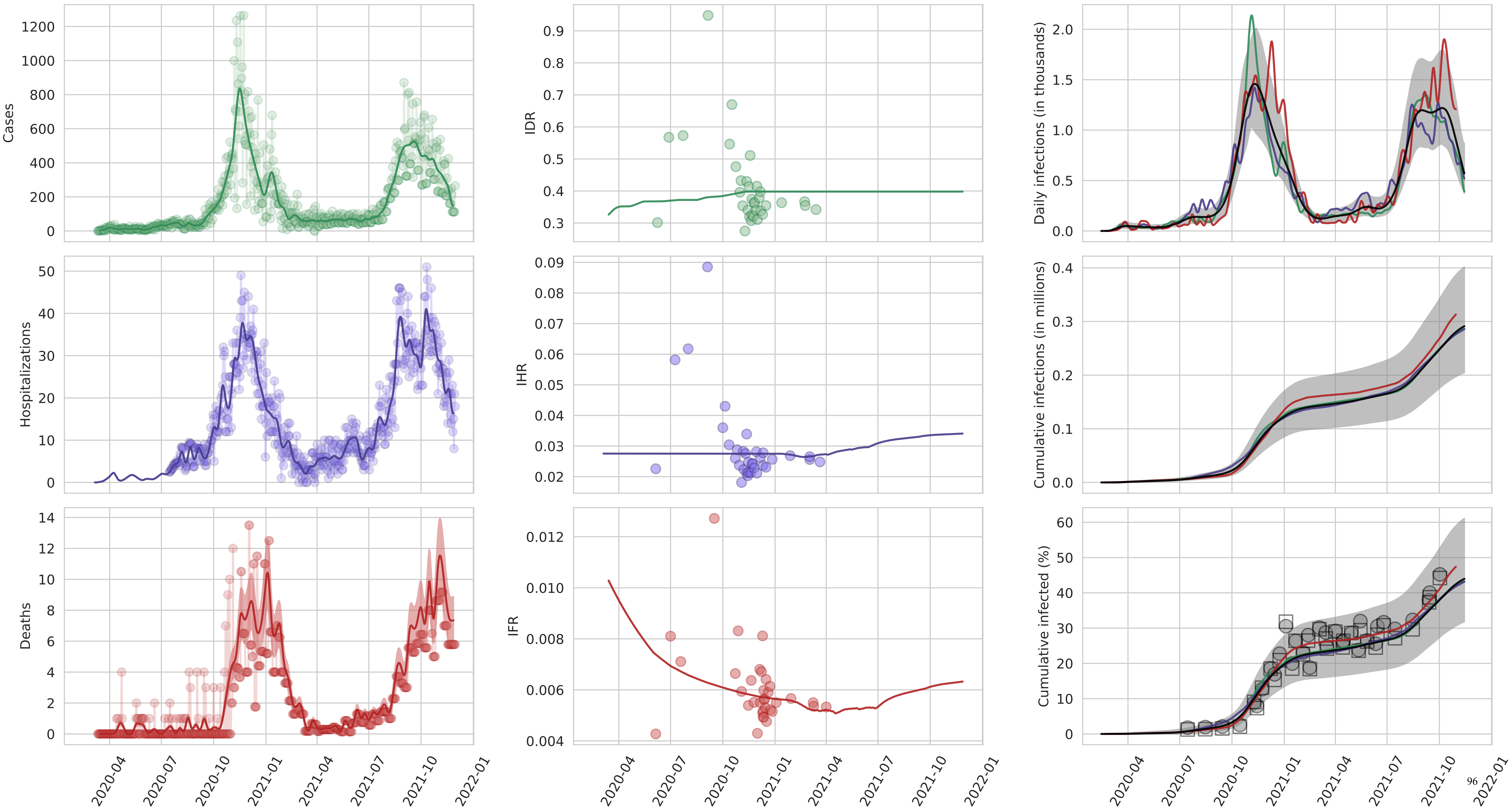

Cases
Hospitalizations
Deaths
IDR
IHR
IFR
Daily infections (in thousands)
Cumulative infections (in millions)
Cumulative infected (%)
2020-04
2020-07
2020-10
2021-01
2021-04
2021-07
2021-10
2022-01

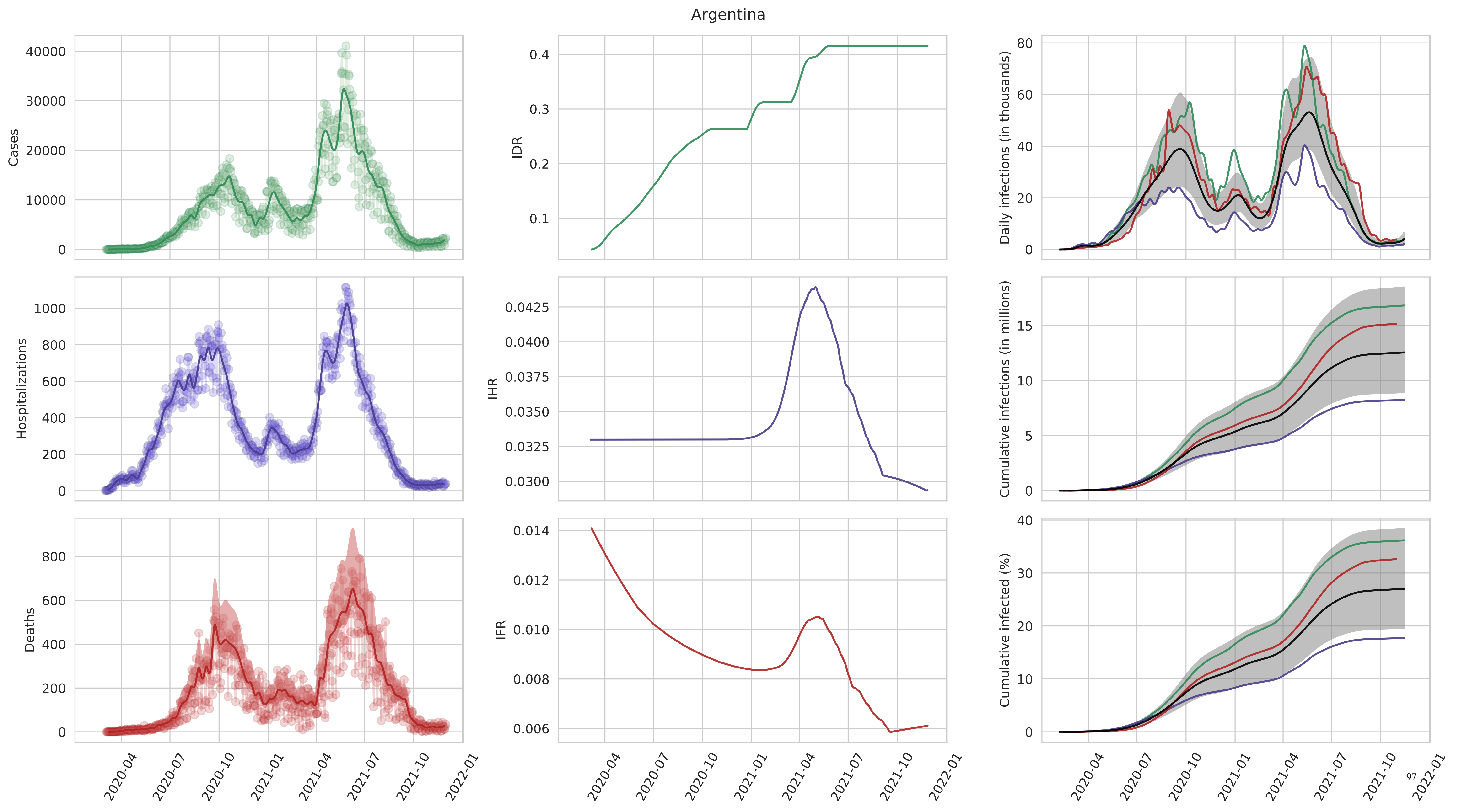
Argentina
Cases
Hospitalizations
Deaths
IDR
IHR
IFR
Daily infections (in thousands)
Cumulative infections (in millions)
Cumulative infected (%)
2020-04
2020-07
2020-10
2021-01
2021-04
2021-07
2021-10
2022-01

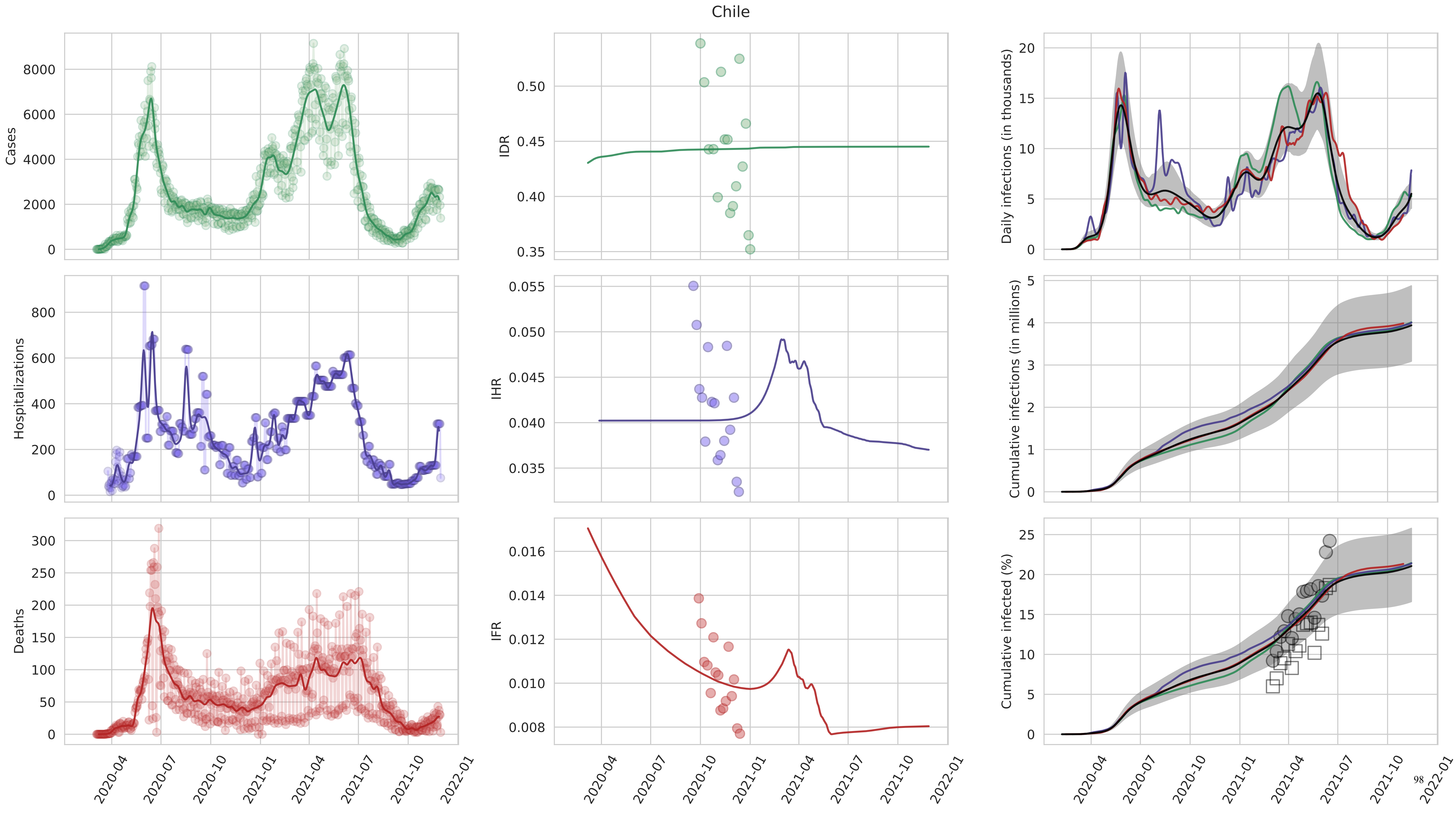
Chile
Cases
Hospitalizations
Deaths
IDR
IHR
IFR
Daily infections (in thousands)
Cumulative infections (in millions)
Cumulative infected (%)
2020-04
2020-07
2020-10
2021-01
2021-04
2021-07
2021-10
2022-01

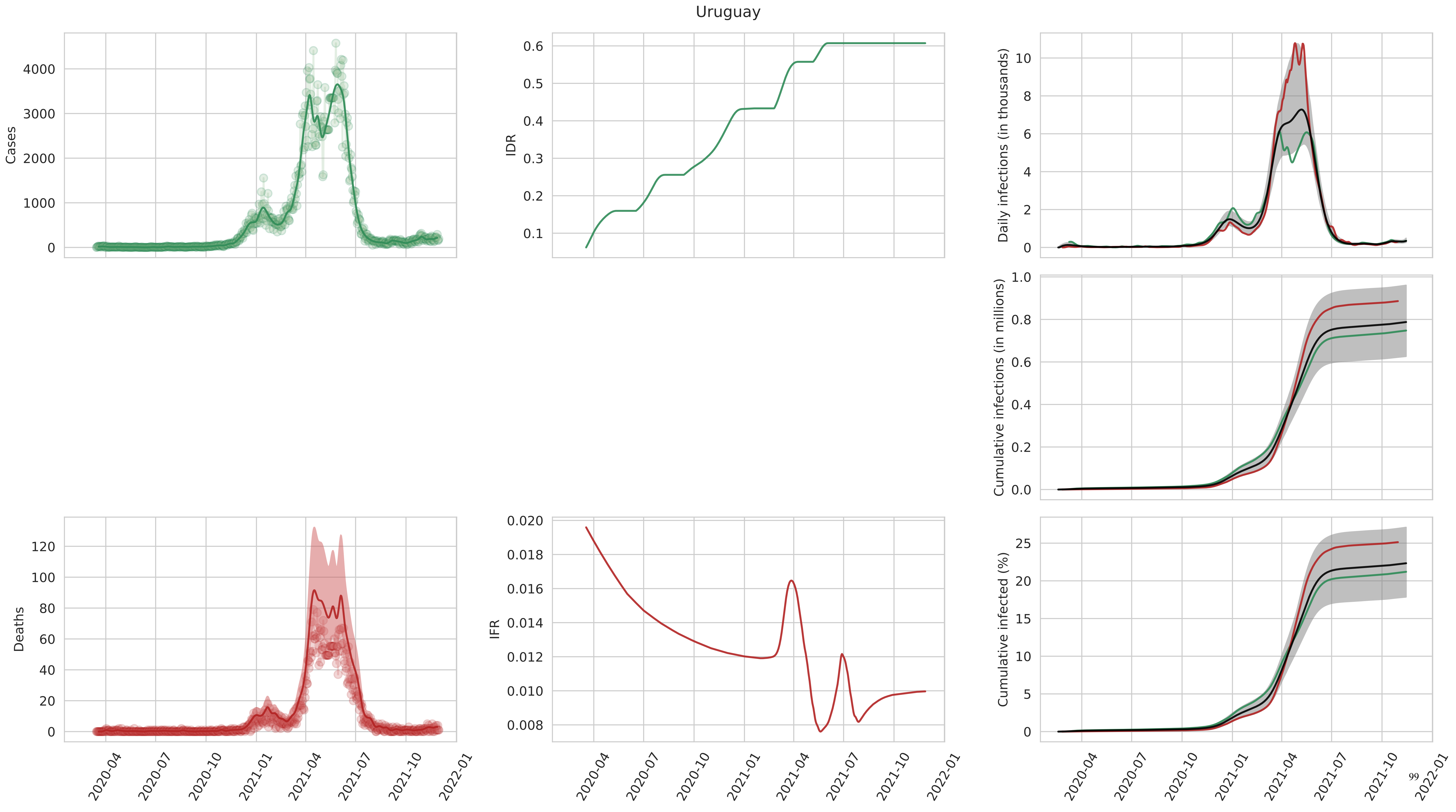
Uruguay
Cases
IDR
Daily infections (in thousands)
Cumulative infections (in millions)
Deaths
IFR
Cumulative infected (%)
2020-04
2020-07
2020-10
2021-01
2021-04
2021-07
2021-10
2022-01

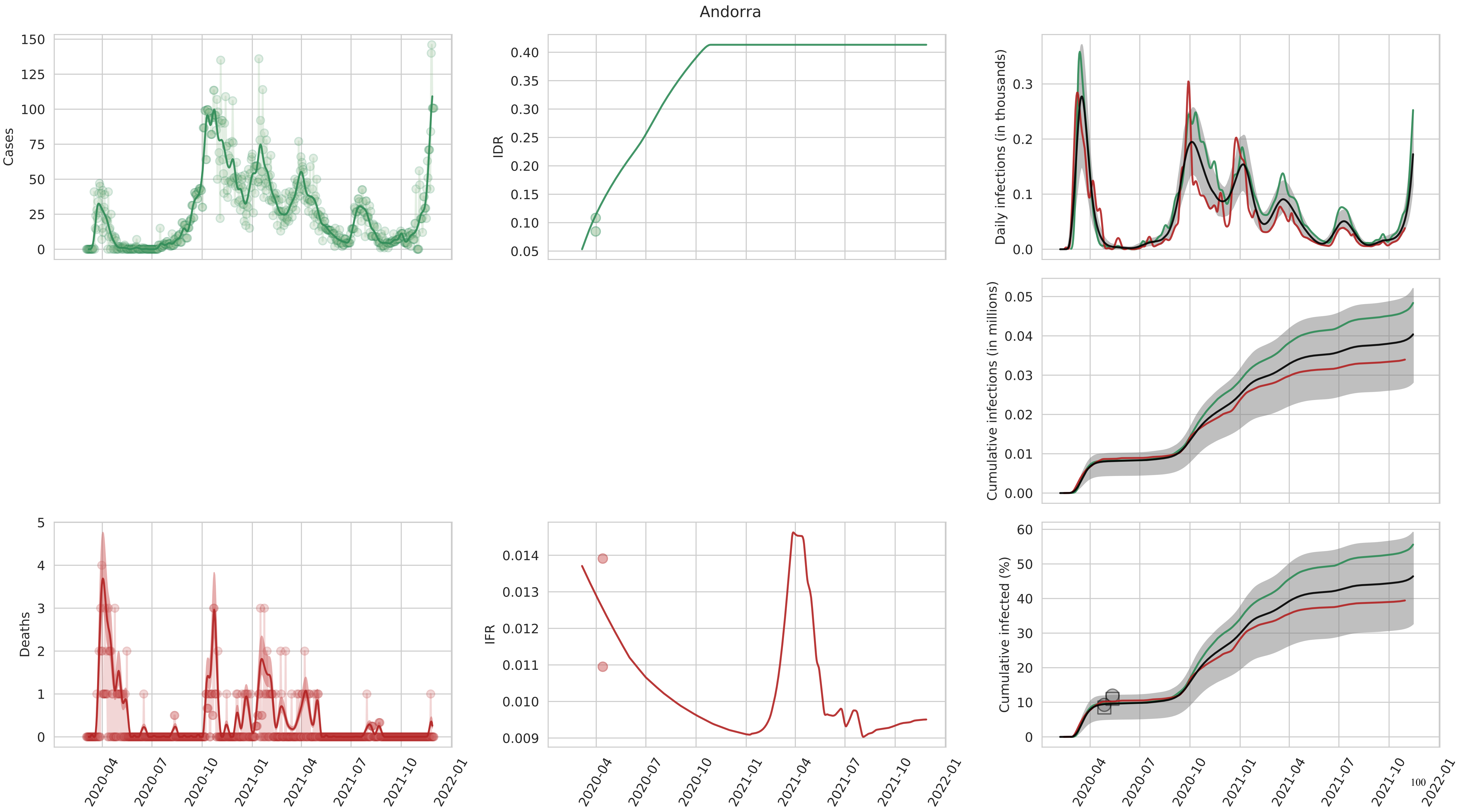
Andorra
Cases
IDR
Daily infections (in thousands)
Cumulative infections (in millions)
Deaths
IFR
Cumulative infected (%)
2020-04
2020-07
2020-10
2021-01
2021-04
2021-07
2021-10
2022-01

Austria

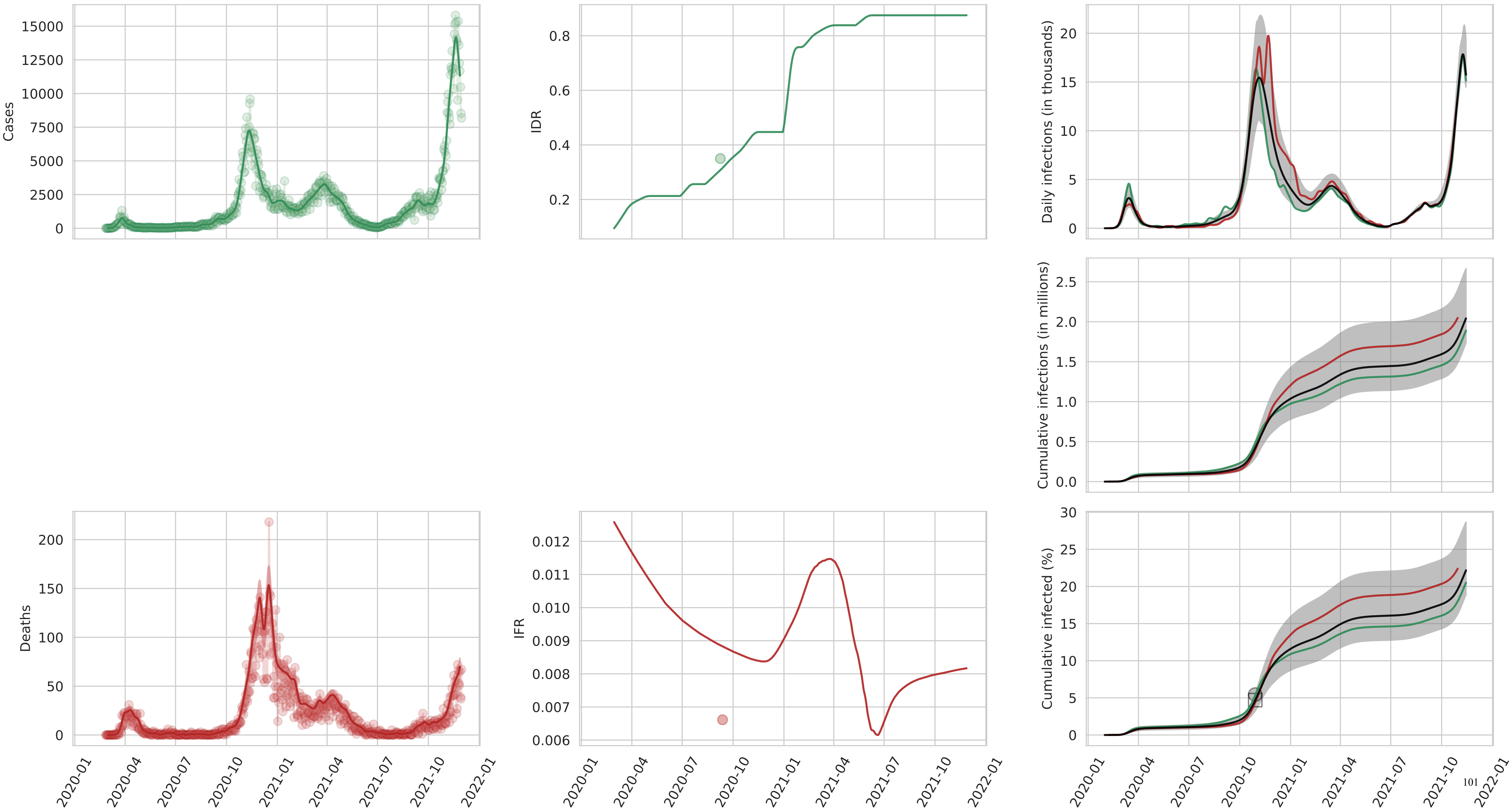

Cases
IDR
Daily infections (in thousands)
Cumulative infections (in millions)
Deaths
IFR
Cumulative infected (%)
2020-01
2020-04
2020-07
2020-10
2021-01
2021-04
2021-07
2021-10
2022-01

Belgium

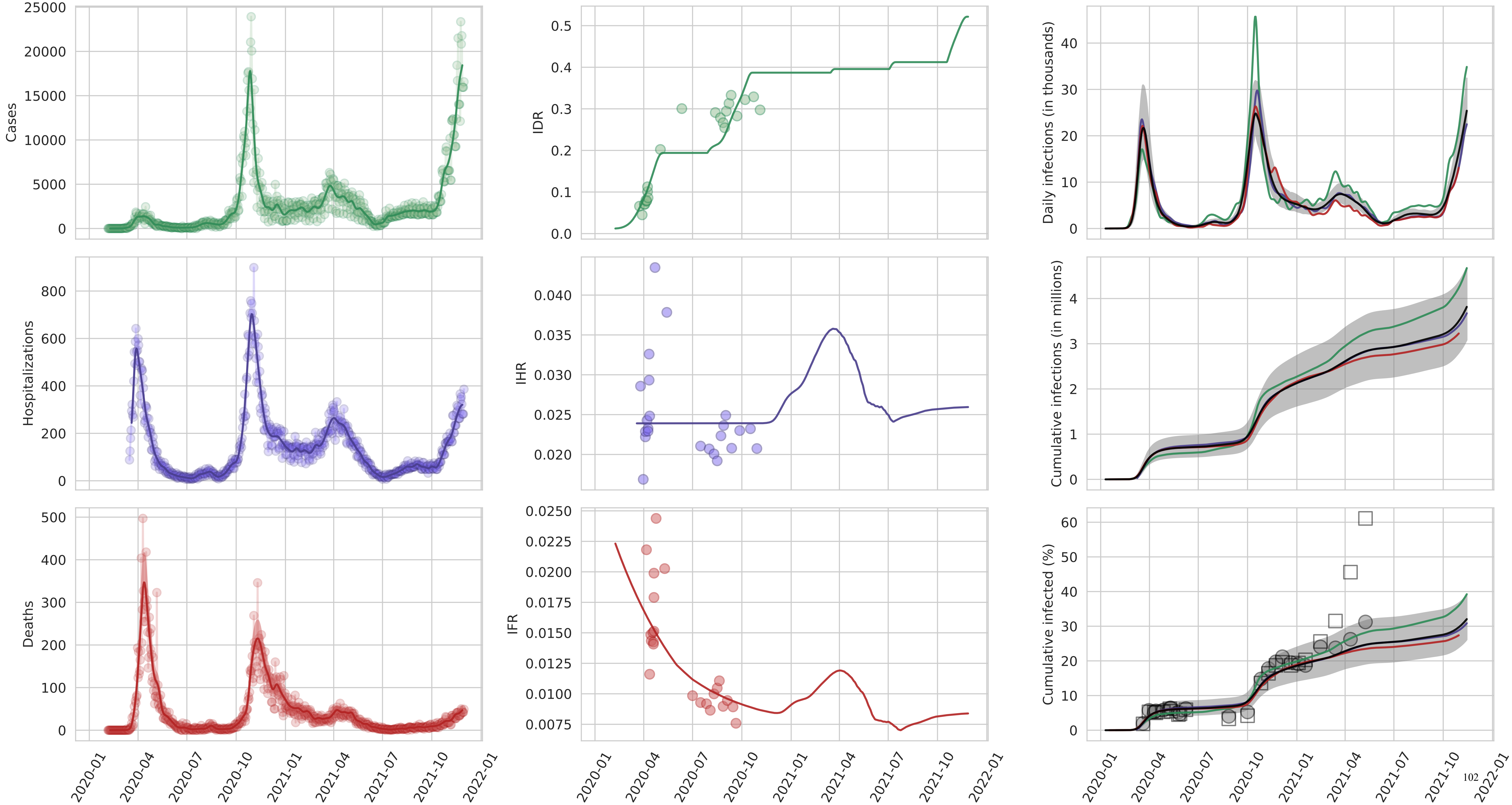

Cases
Hospitalizations
Deaths
IDR
IHR
IFR
Daily infections (in thousands)
Cumulative infections (in millions)
Cumulative infected (%)
2020-01
2020-04
2020-07
2020-10
2021-01
2021-04
2021-07
2021-10
2022-01

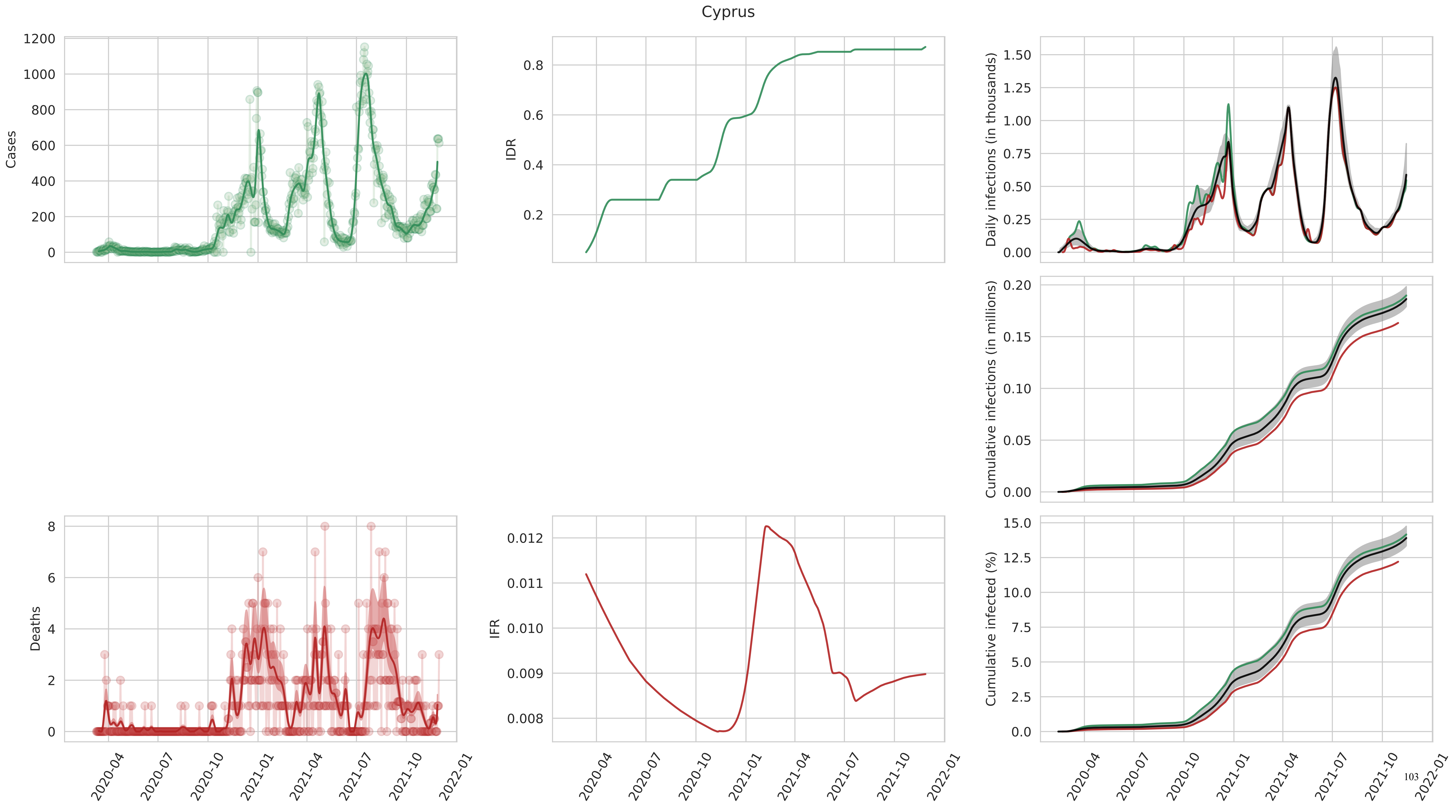

Cyprus
Cases
IDR
Daily infections (in thousands)
Cumulative infections (in millions)
Deaths
IFR
Cumulative infected (%)
2020-04
2020-07
2020-10
2021-01
2021-04
2021-07
2021-10
2022-01

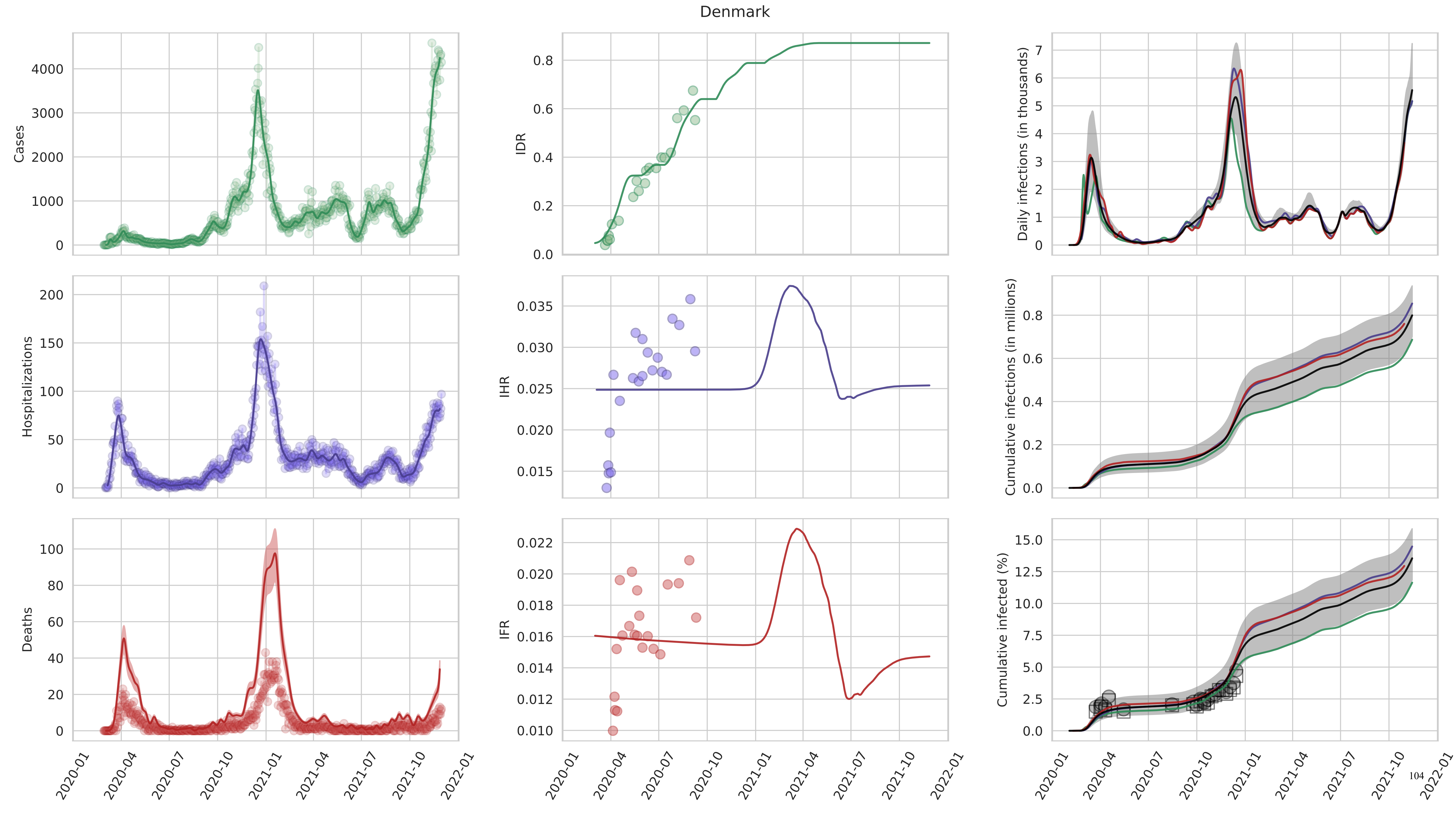
Denmark
Cases
IDR
Daily infections (in thousands)
Hospitalizations
IHR
Cumulative infections (in millions)
Deaths
IFR
Cumulative infected (%)
2020-01
2020-04
2020-07
2020-10
2021-01
2021-04
2021-07
2021-10
2022-01

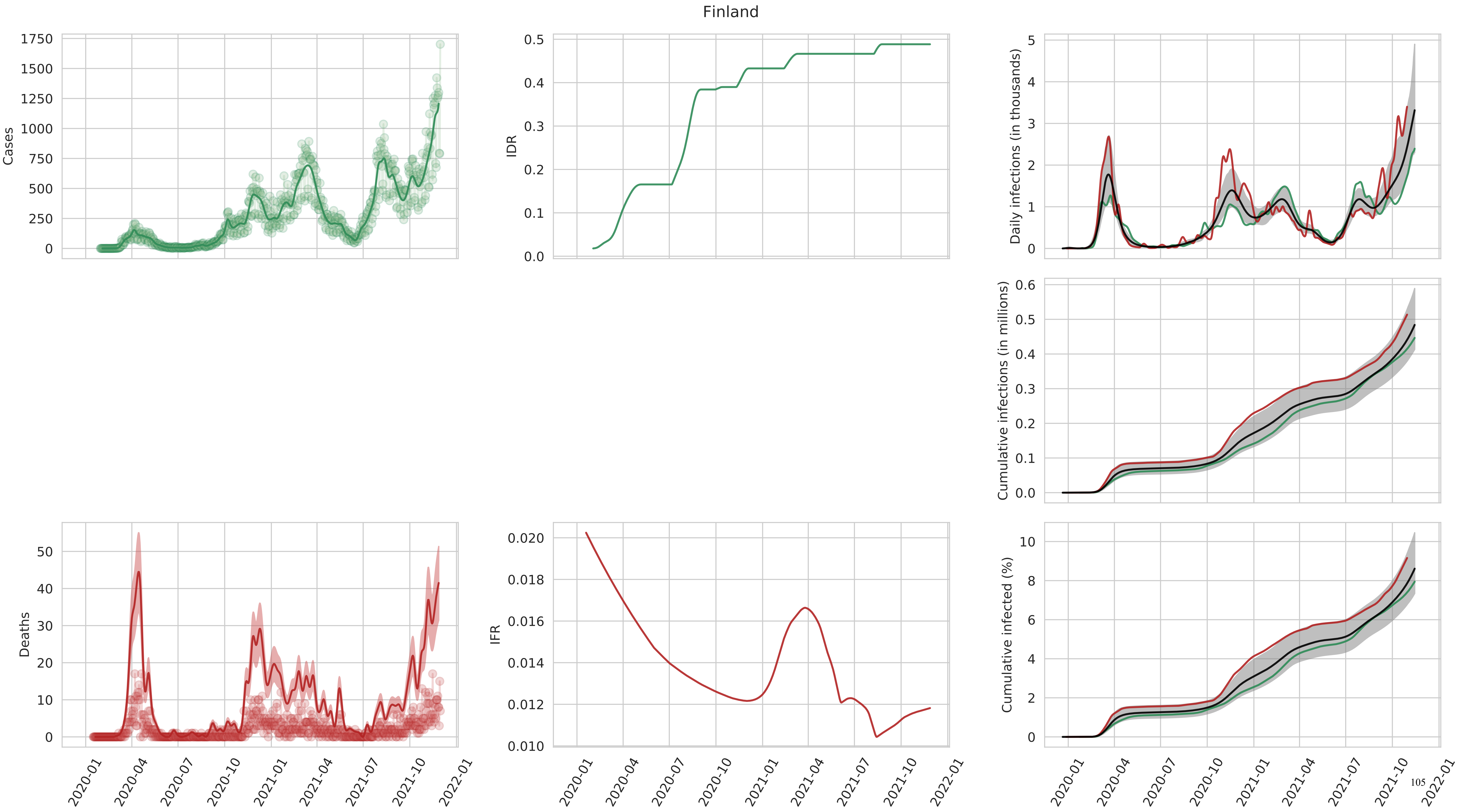
Finland
Cases
IDR
Daily infections (in thousands)
Cumulative infections (in millions)
Deaths
IFR
Cumulative infected (%)
2020-01
2020-04
2020-07
2020-10
2021-01
2021-04
2021-07
2021-10
2022-01

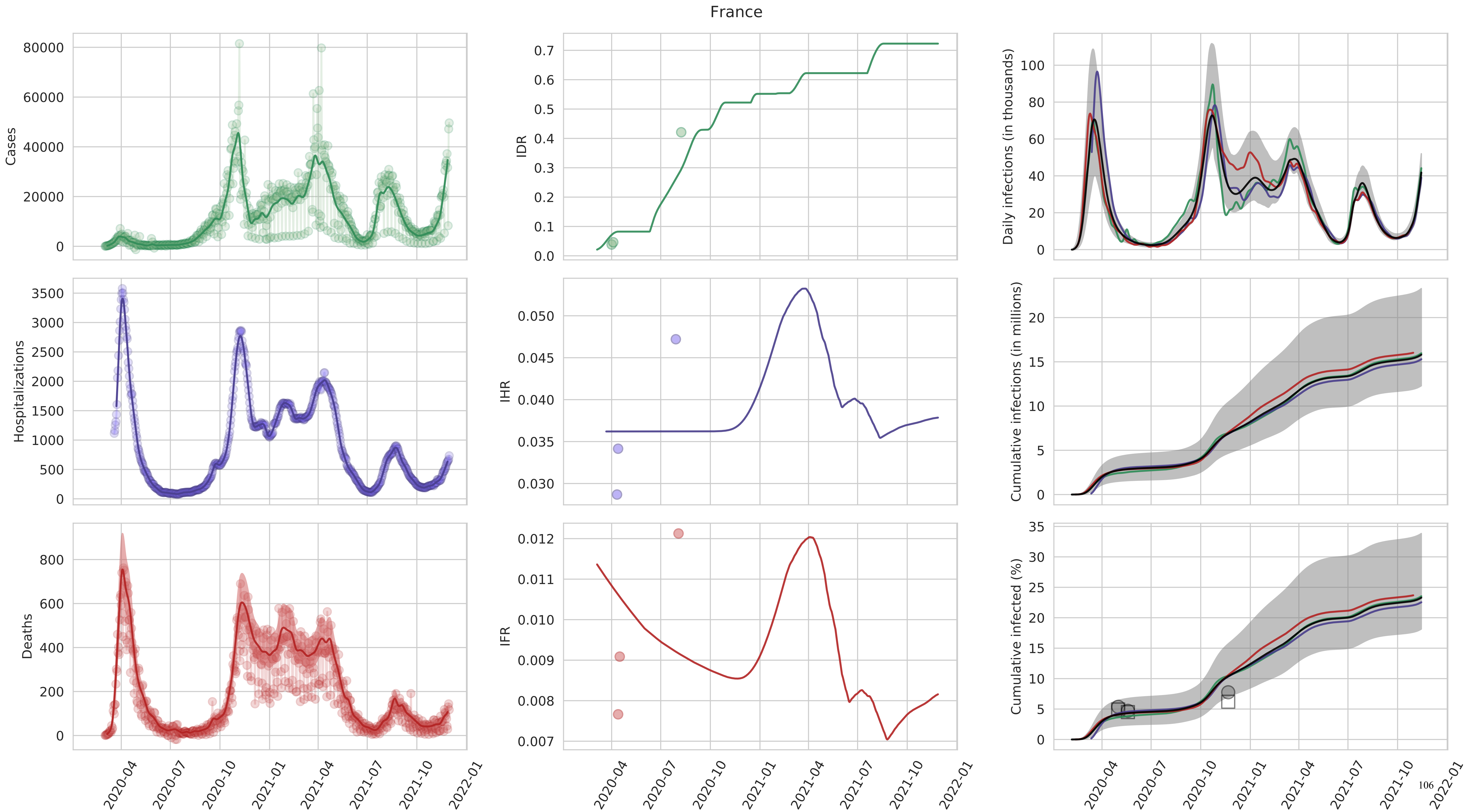
France
Cases
Hospitalizations
Deaths
IDR
IHR
IFR
Daily infections (in thousands)
Cumulative infections (in millions)
Cumulative infected (%)
2020-04
2020-07
2020-10
2021-01
2021-04
2021-07
2021-10
2022-01

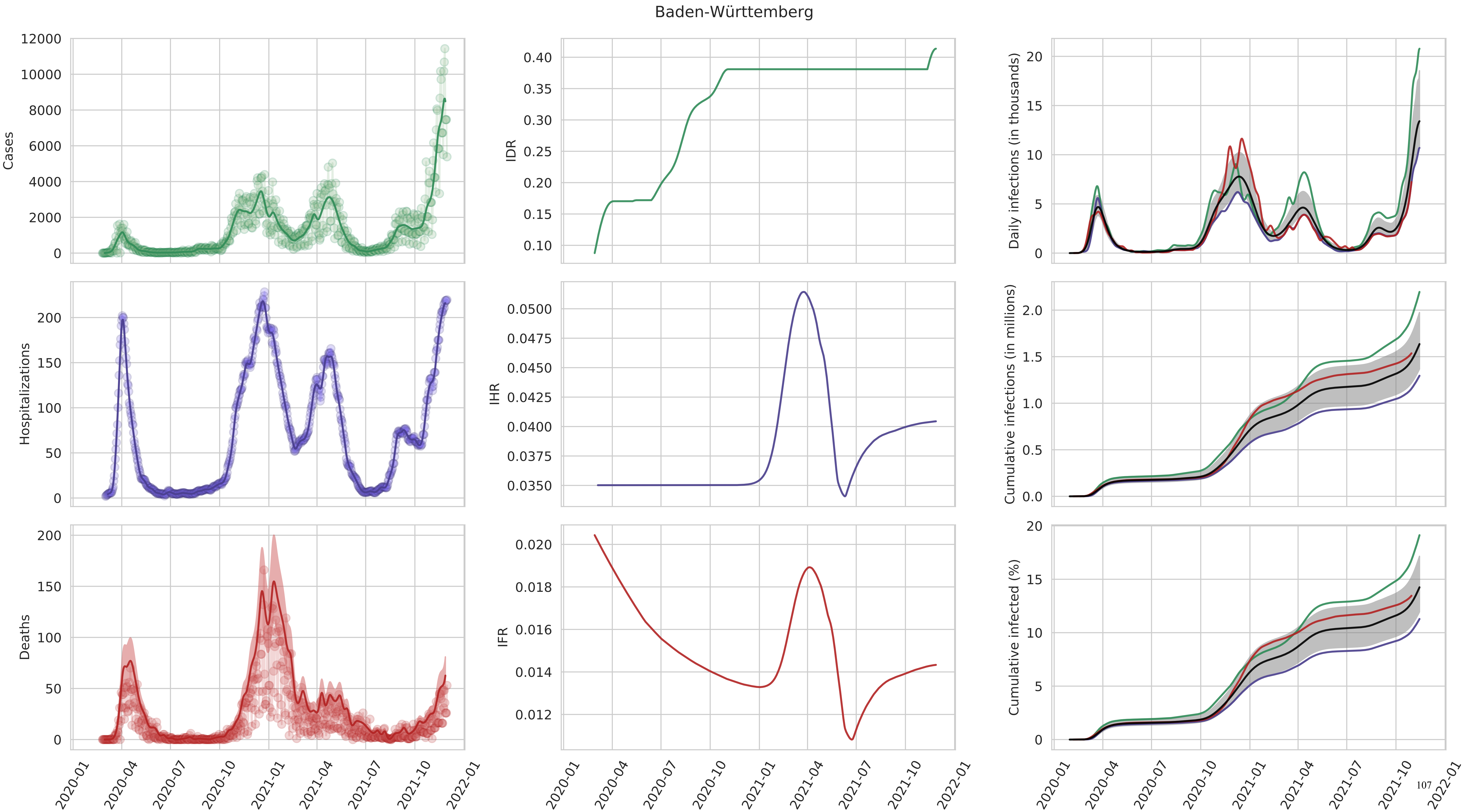

Baden-Württemberg
Cases
IDR
Daily infections (in thousands)
Hospitalizations
IHR
Cumulative infections (in millions)
Deaths
IFR
Cumulative infected (%)

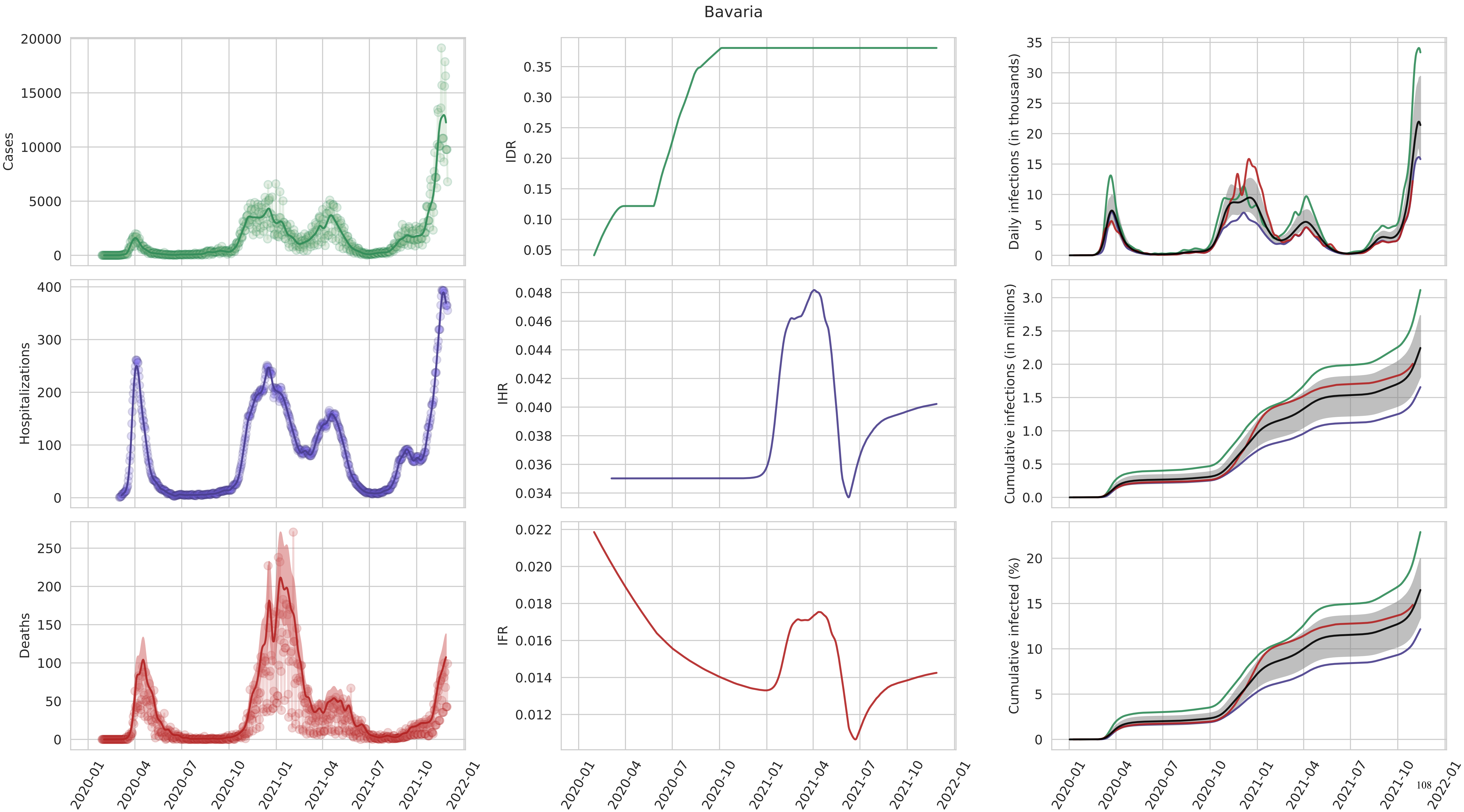
Bavaria
Cases
IDR
Daily infections (in thousands)
Hospitalizations
IHR
Cumulative infections (in millions)
Deaths
IFR
Cumulative infected (%)
2020-01
2020-04
2020-07
2020-10
2021-01
2021-04
2021-07
2021-10
2022-01

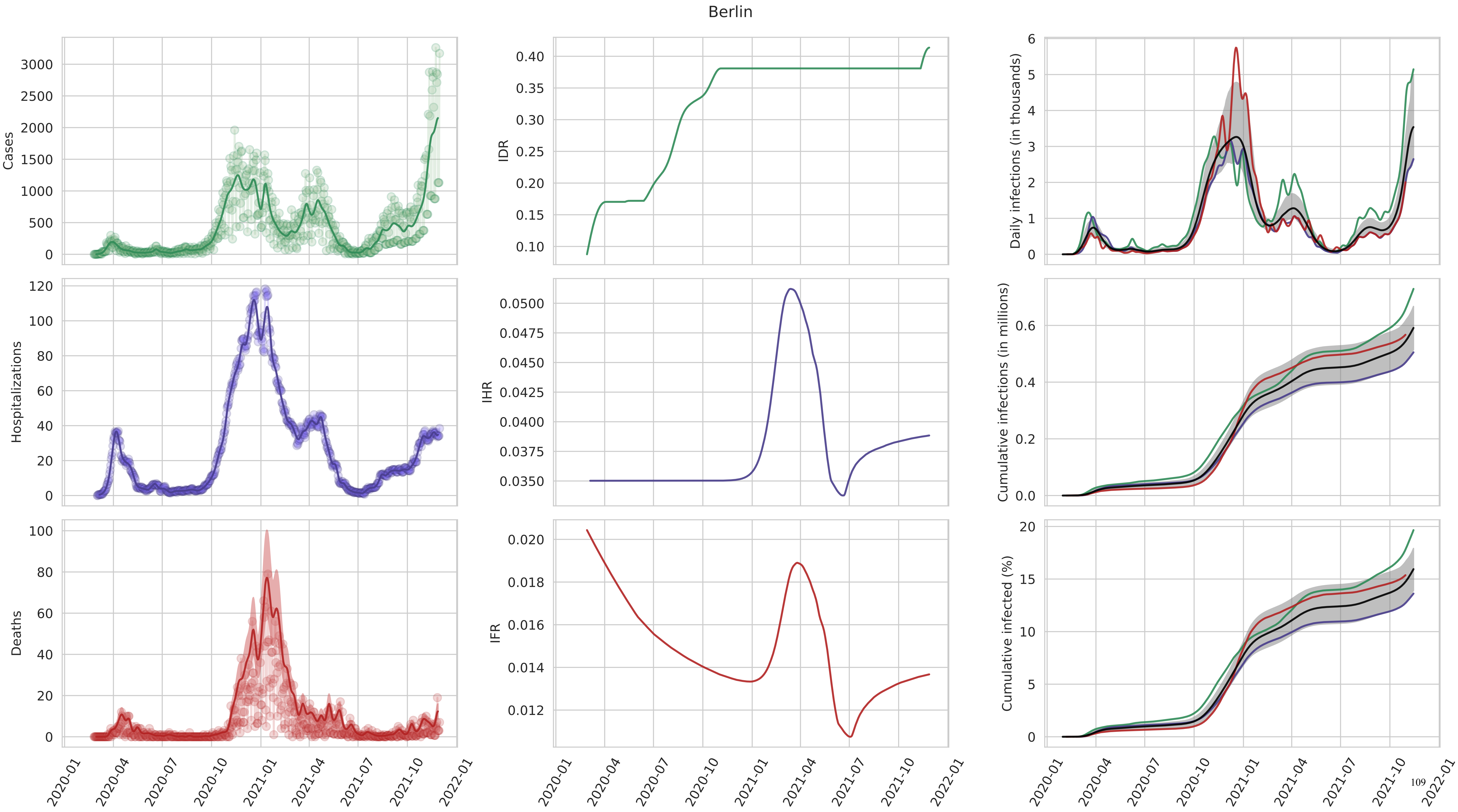
Berlin
Cases
Hospitalizations
Deaths
IDR
IHR
IFR
Daily infections (in thousands)
Cumulative infections (in millions)
Cumulative infected (%)
2020-01
2020-04
2020-07
2020-10
2021-01
2021-04
2021-07
2021-10
2022-01

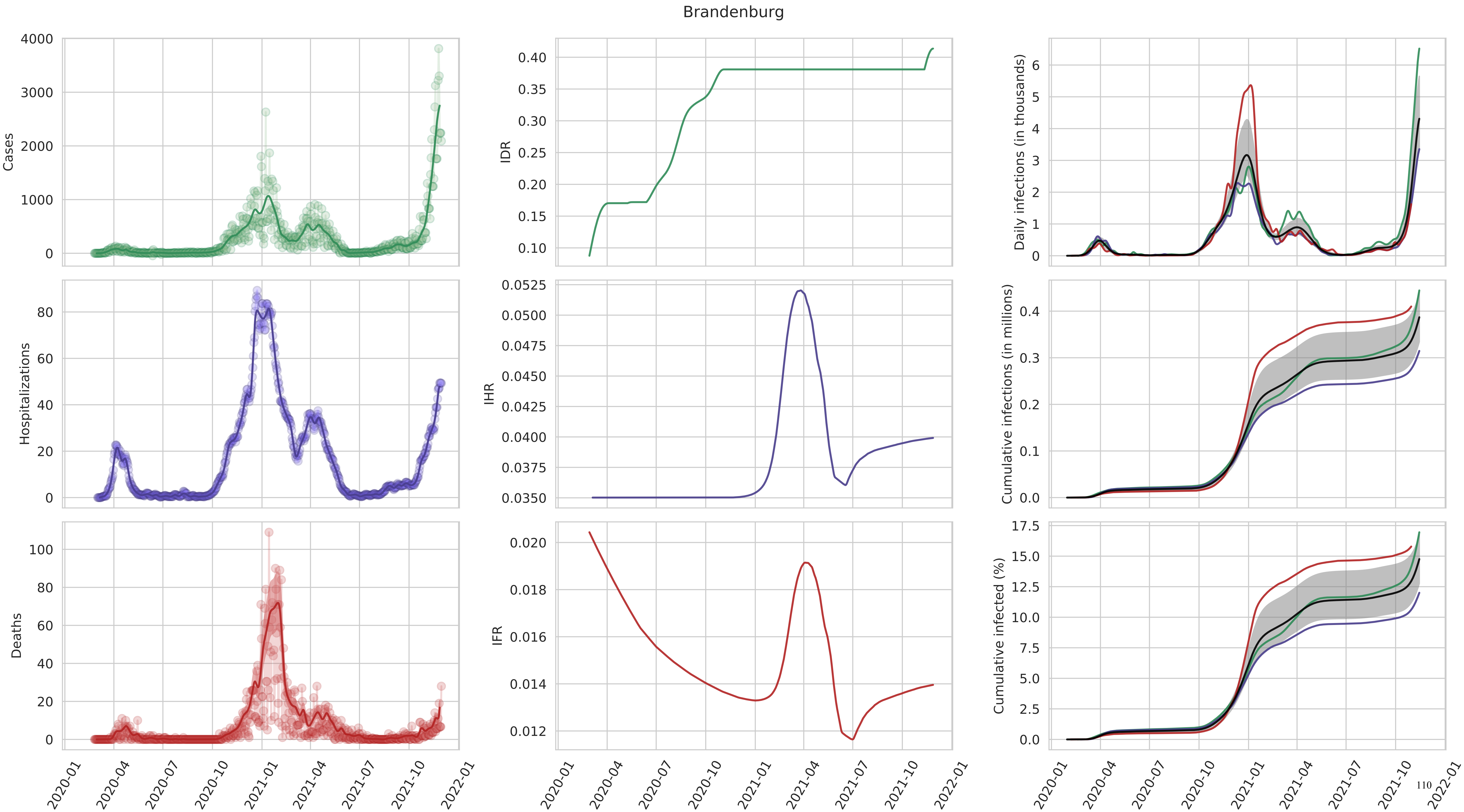
Brandenburg
Cases
Hospitalizations
Deaths
IDR
IHR
IFR
Daily infections (in thousands)
Cumulative infections (in millions)
Cumulative infected (%)
2020-01
2020-04
2020-07
2020-10
2021-01
2021-04
2021-07
2021-10
2022-01

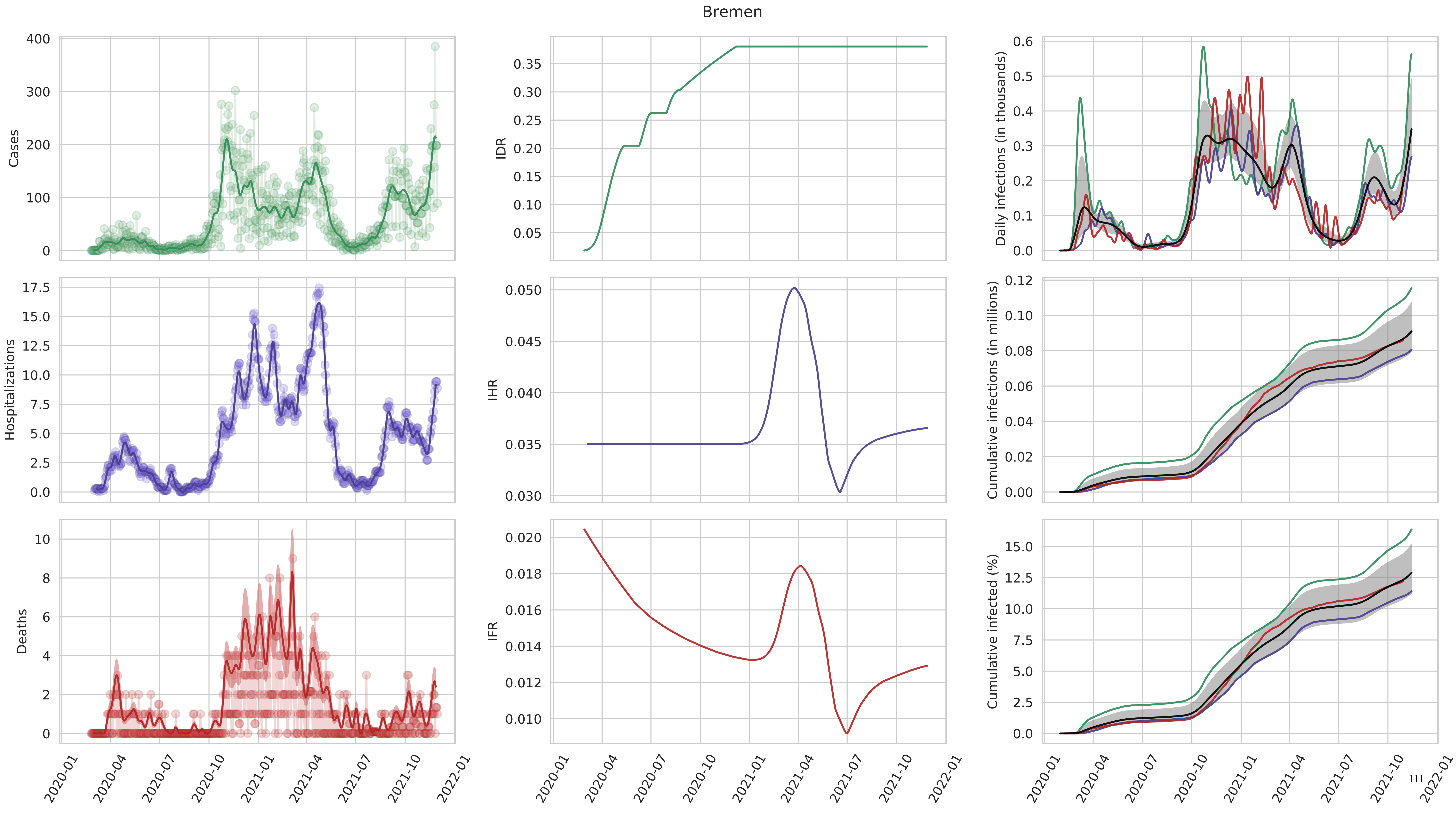
Bremen
Cases
Hospitalizations
Deaths
IDR
IHR
IFR
Daily infections (in thousands)
Cumulative infections (in millions)
Cumulative infected (%)
2020-01
2020-04
2020-07
2020-10
2021-01
2021-04
2021-07
2021-10
2022-01

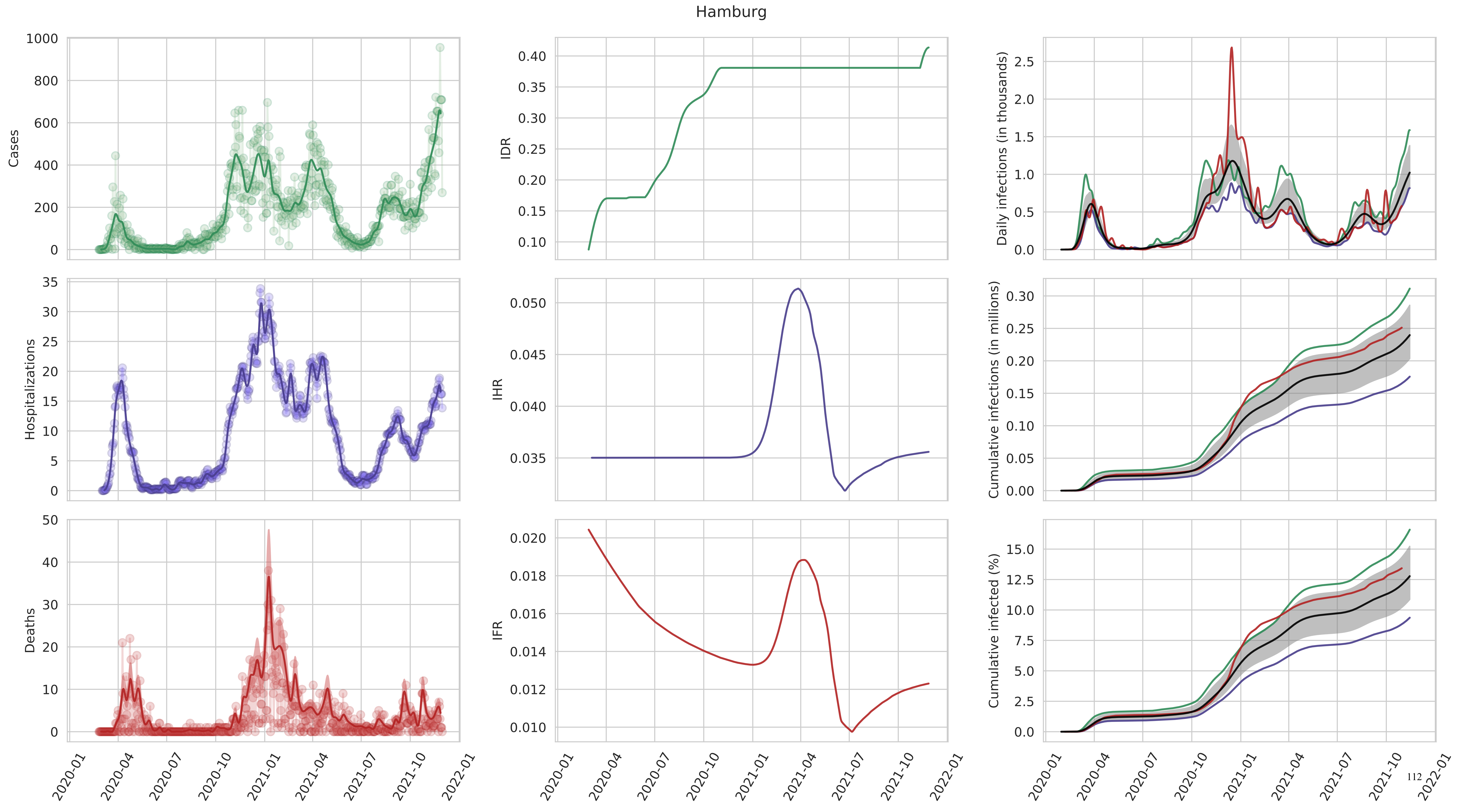
Hamburg
Cases
IDR
Daily infections (in thousands)
Hospitalizations
IHR
Cumulative infections (in millions)
Deaths
IFR
Cumulative infected (%)

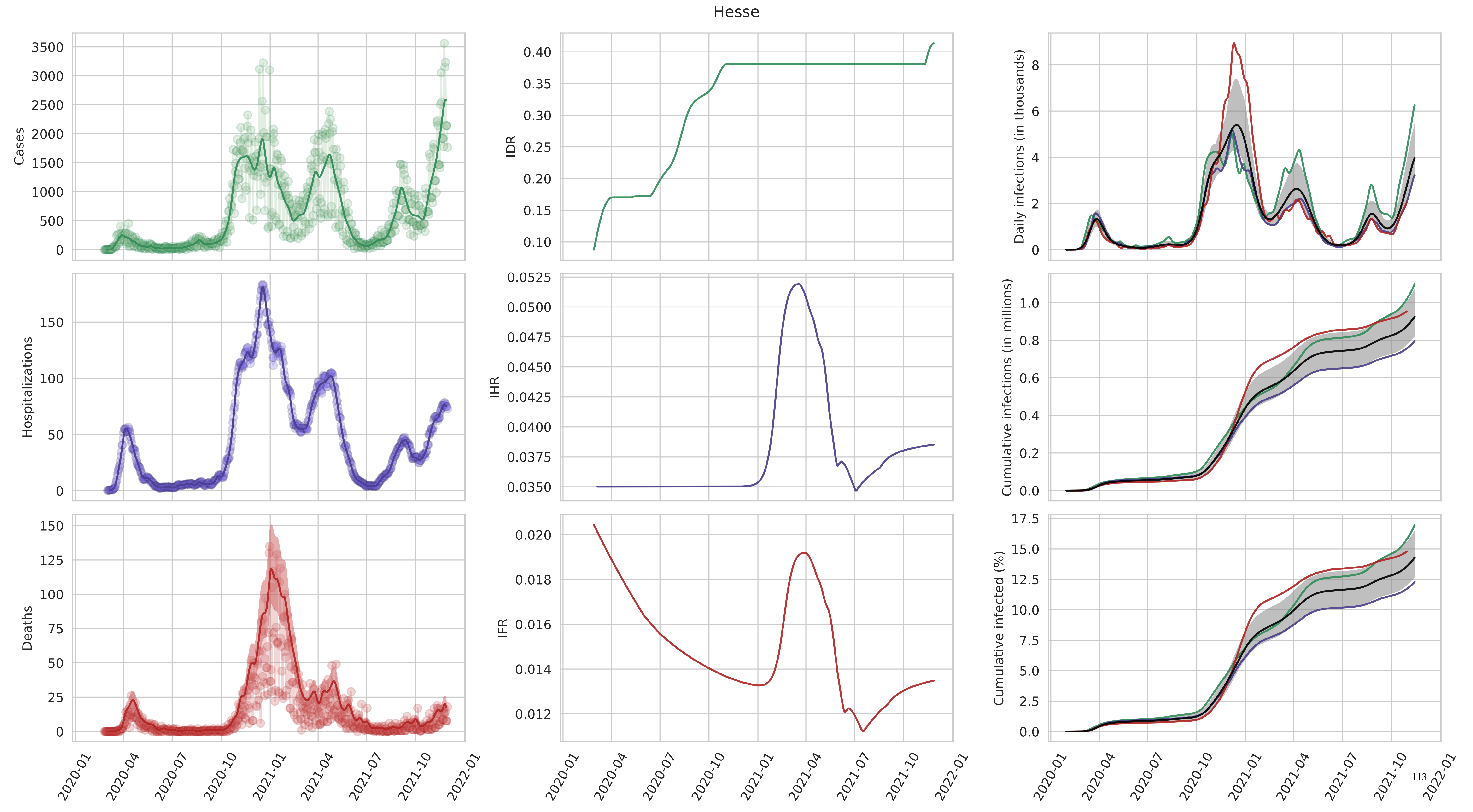
Hesse
Cases
Hospitalizations
Deaths
IDR
IHR
IFR
Daily infections (in thousands)
Cumulative infections (in millions)
Cumulative infected (%)
2020-01
2020-04
2020-07
2020-10
2021-01
2021-04
2021-07
2021-10
2022-01

Lower Saxony

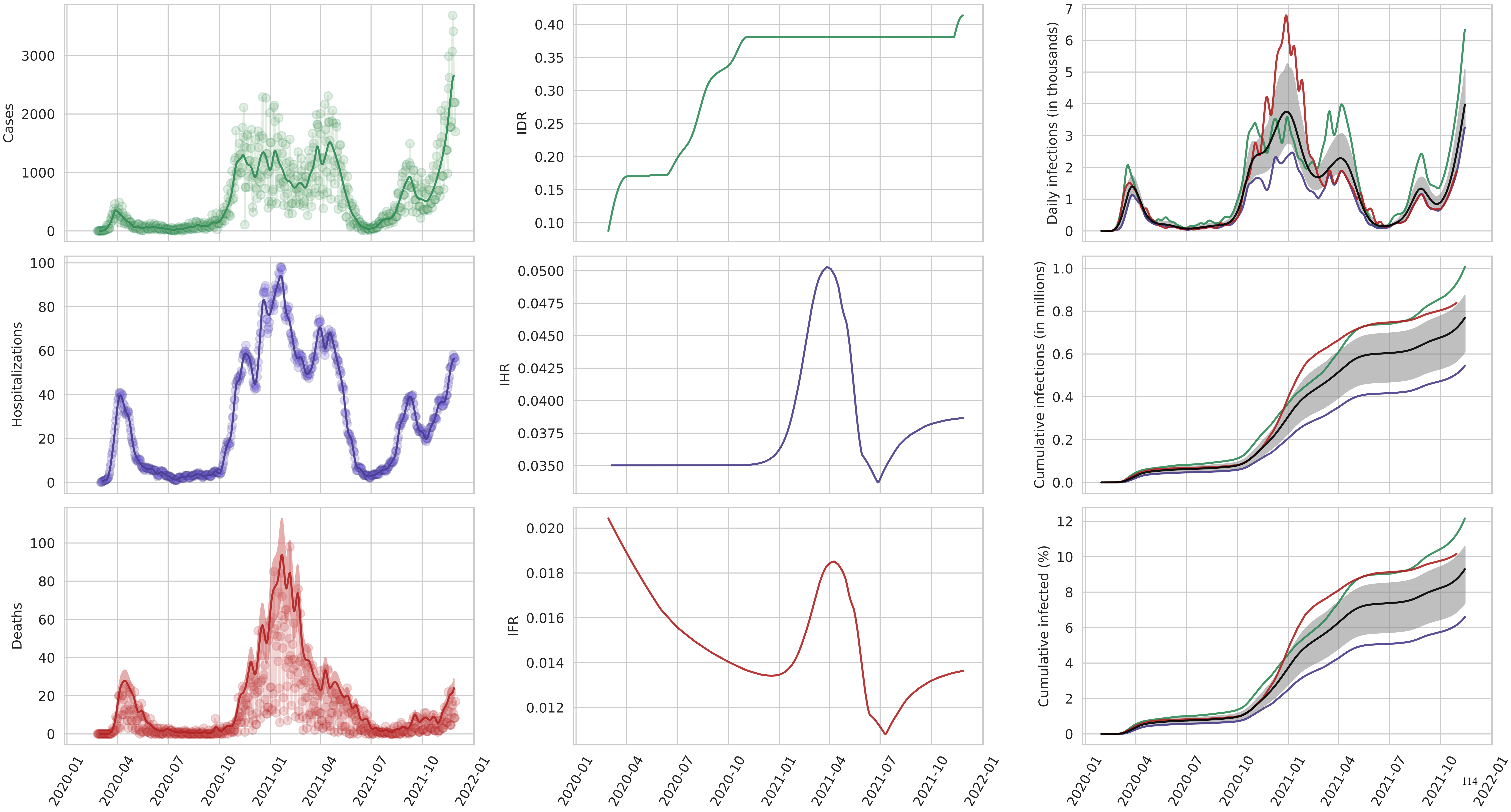

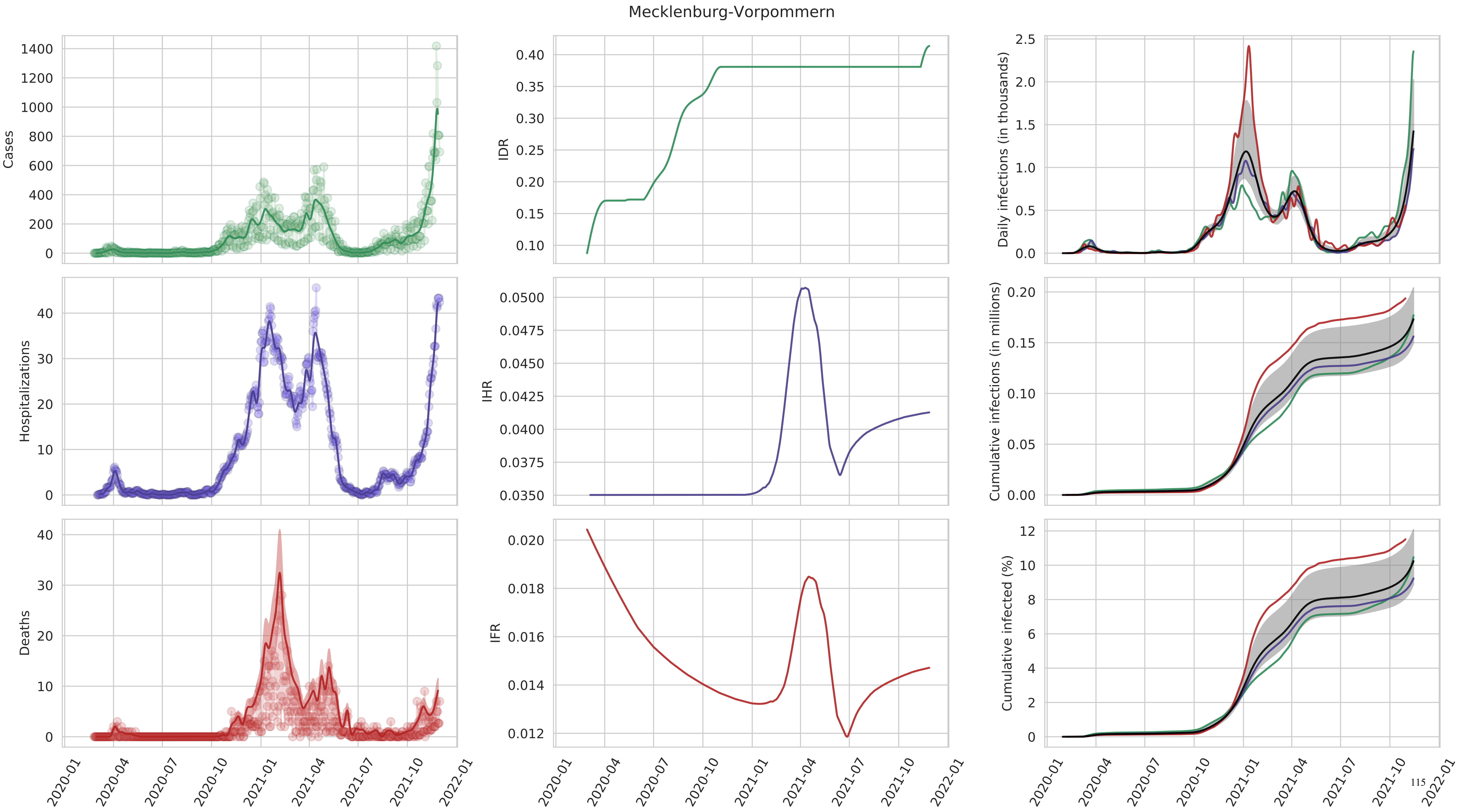

Mecklenburg-Vorpommern
Cases
IDR
Daily infections (in thousands)
Hospitalizations
IHR
Cumulative infections (in millions)
Deaths
IFR
Cumulative infected (%)
2020-01
2020-04
2020-07
2020-10
2021-01
2021-04
2021-07
2021-10
2022-01

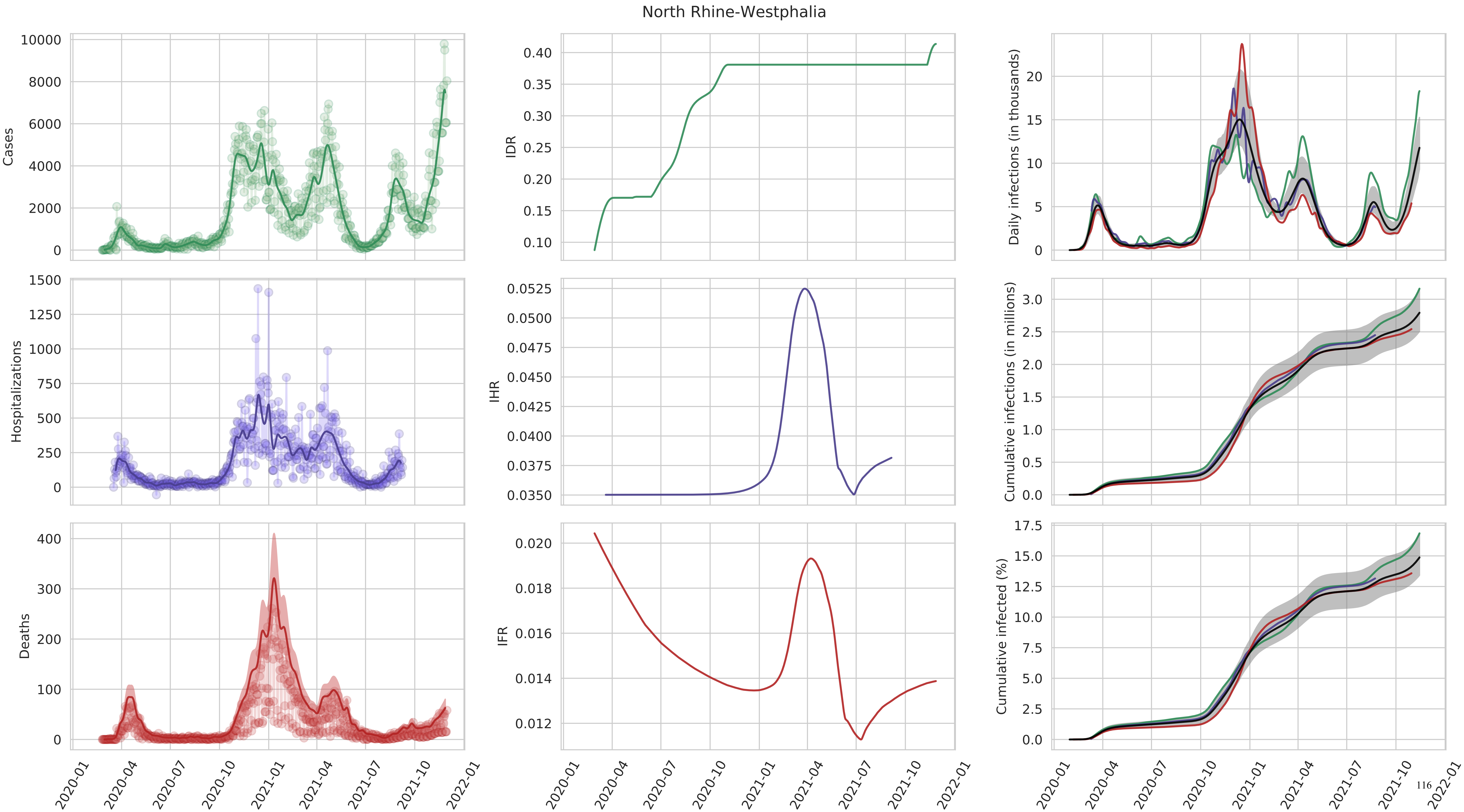

North Rhine-Westphalia
Cases
Hospitalizations
Deaths
IDR
IHR
IFR
Daily infections (in thousands)
Cumulative infections (in millions)
Cumulative infected (%)
2020-01
2020-04
2020-07
2020-10
2021-01
2021-04
2021-07
2021-10
2022-01

Rhineland-Palatinate

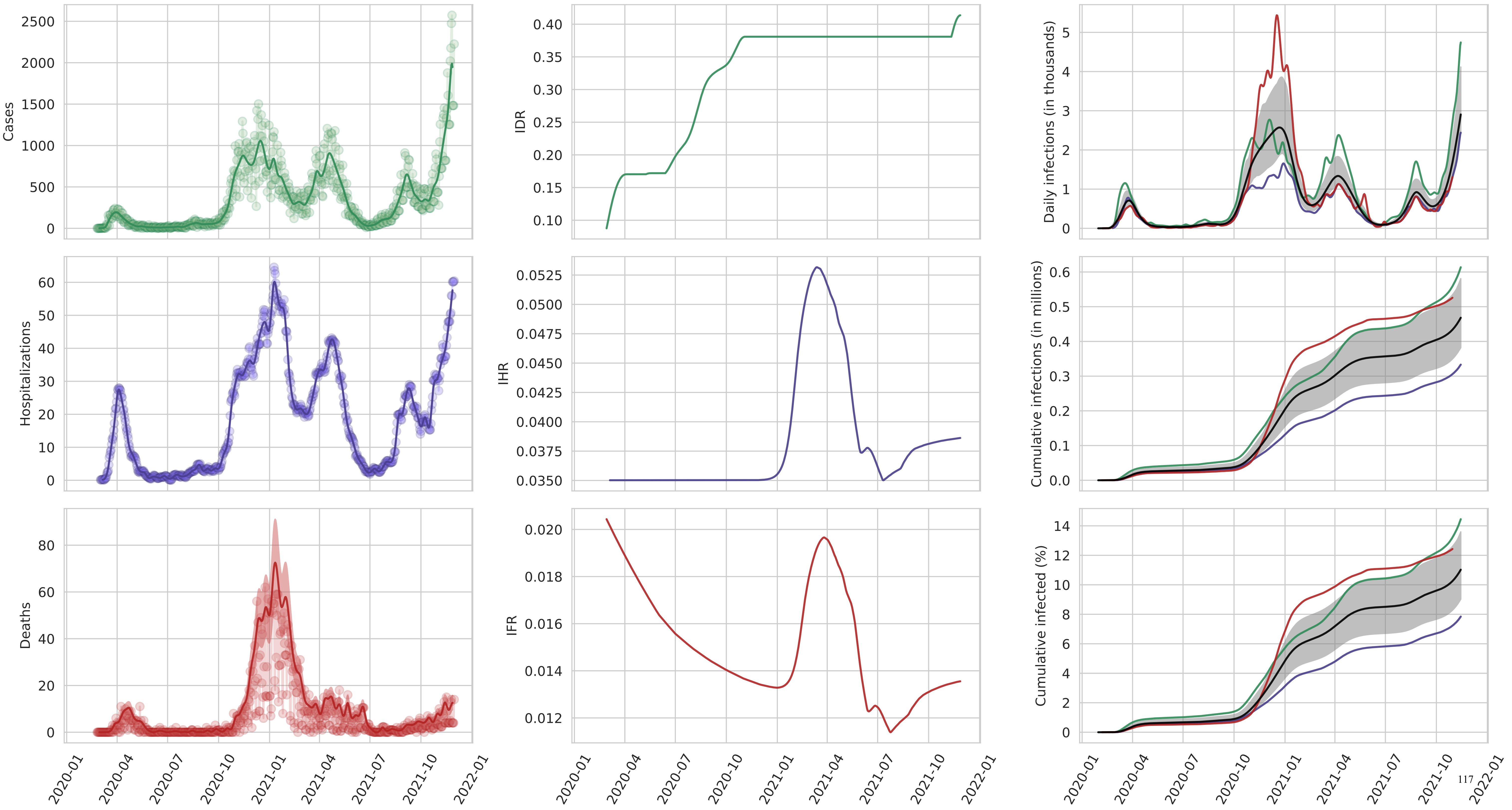

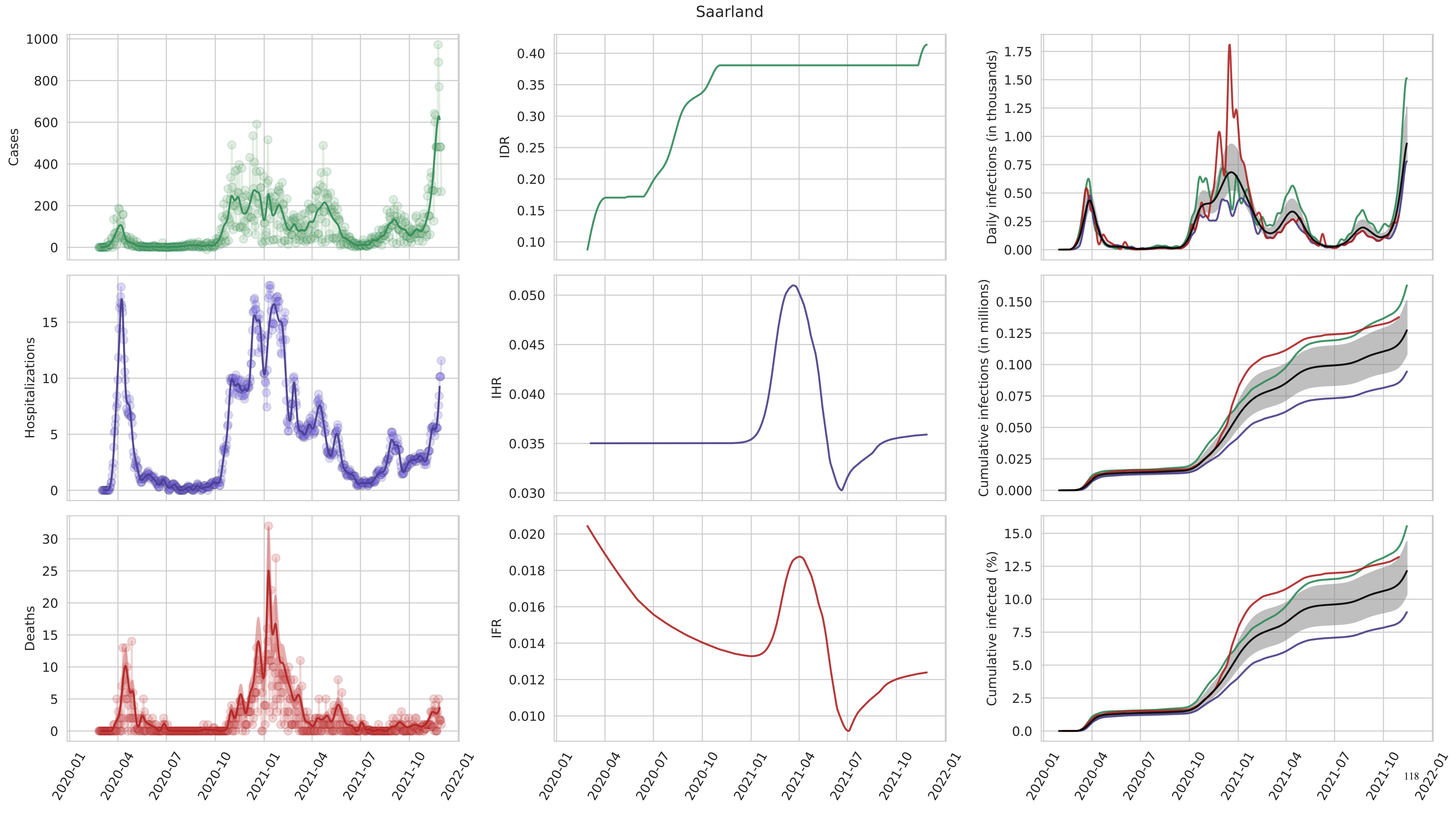
Saarland
Cases
IDR
Daily infections (in thousands)
Hospitalizations
IHR
Cumulative infections (in millions)
Deaths
IFR
Cumulative infected (%)
2020-01
2020-04
2020-07
2020-10
2021-01
2021-04
2021-07
2021-10
2022-01

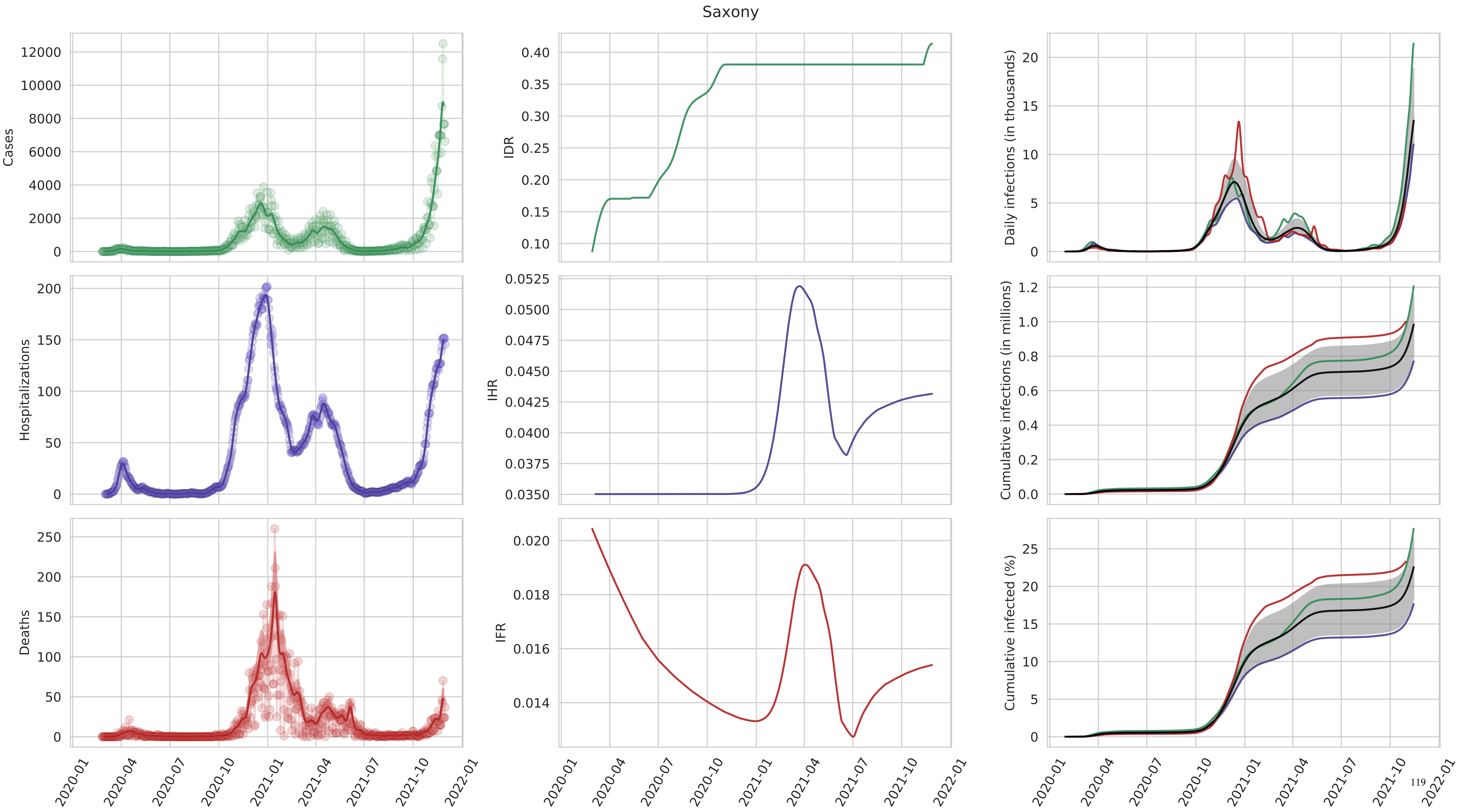
Saxony
Cases
Hospitalizations
Deaths
IDR
IHR
IFR
Daily infections (in thousands)
Cumulative infections (in millions)
Cumulative infected (%)
2020-01
2020-04
2020-07
2020-10
2021-01
2021-04
2021-07
2021-10
2022-01

Saxony-Anhalt

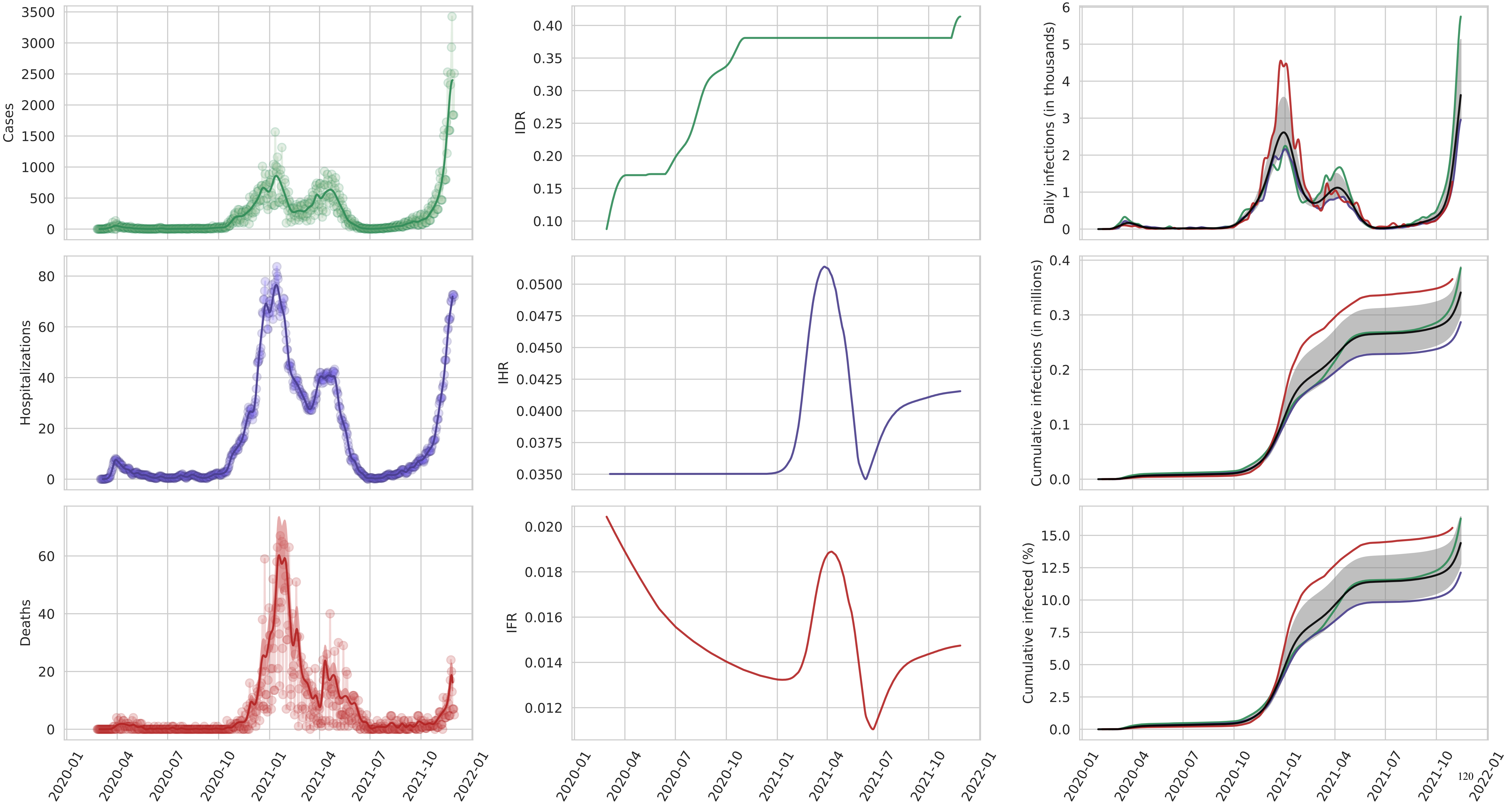

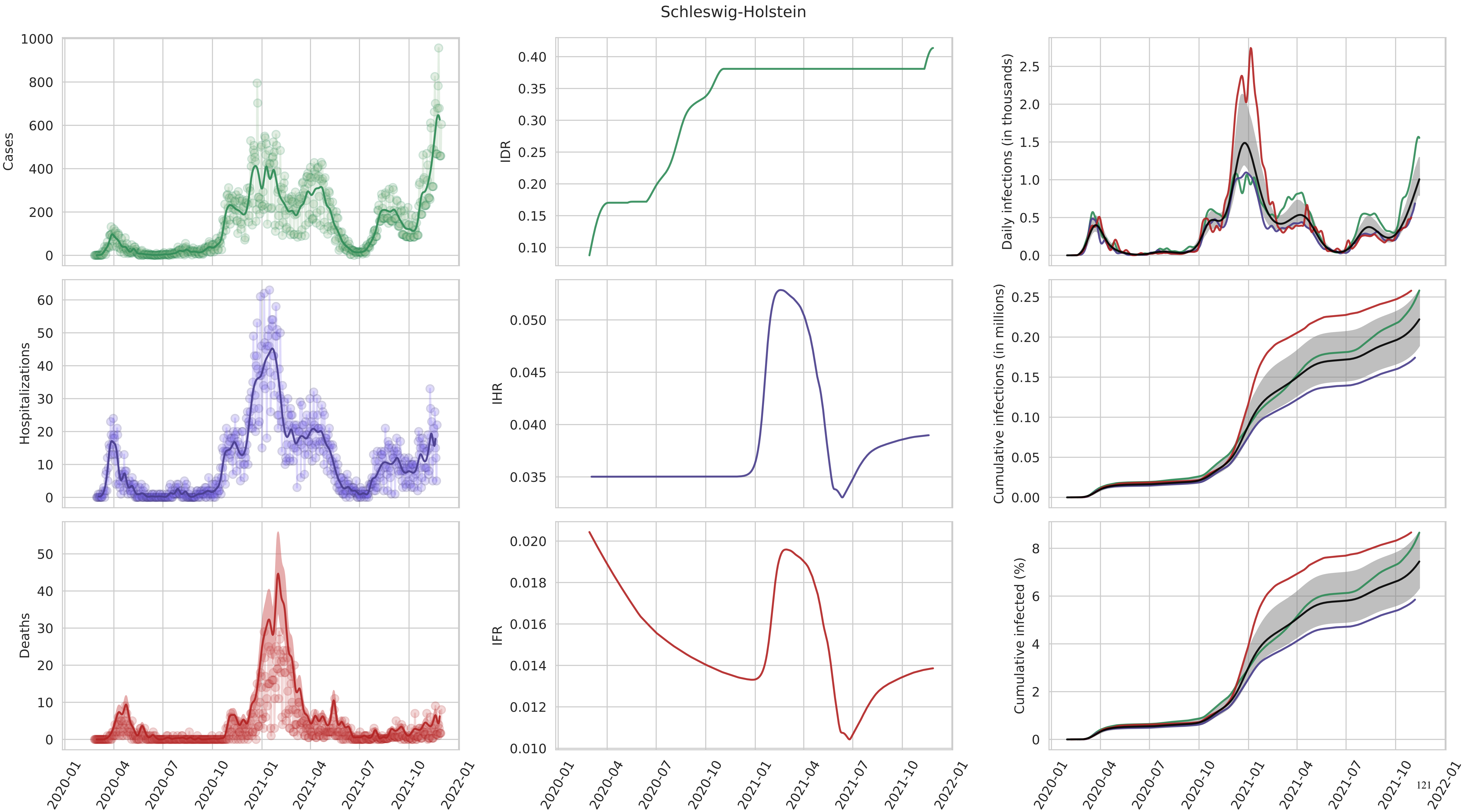

Schleswig-Holstein
Cases
Hospitalizations
Deaths
IDR
IHR
IFR
Daily infections (in thousands)
Cumulative infections (in millions)
Cumulative infected (%)
2020-01
2020-04
2020-07
2020-10
2021-01
2021-04
2021-07
2021-10
2022-01

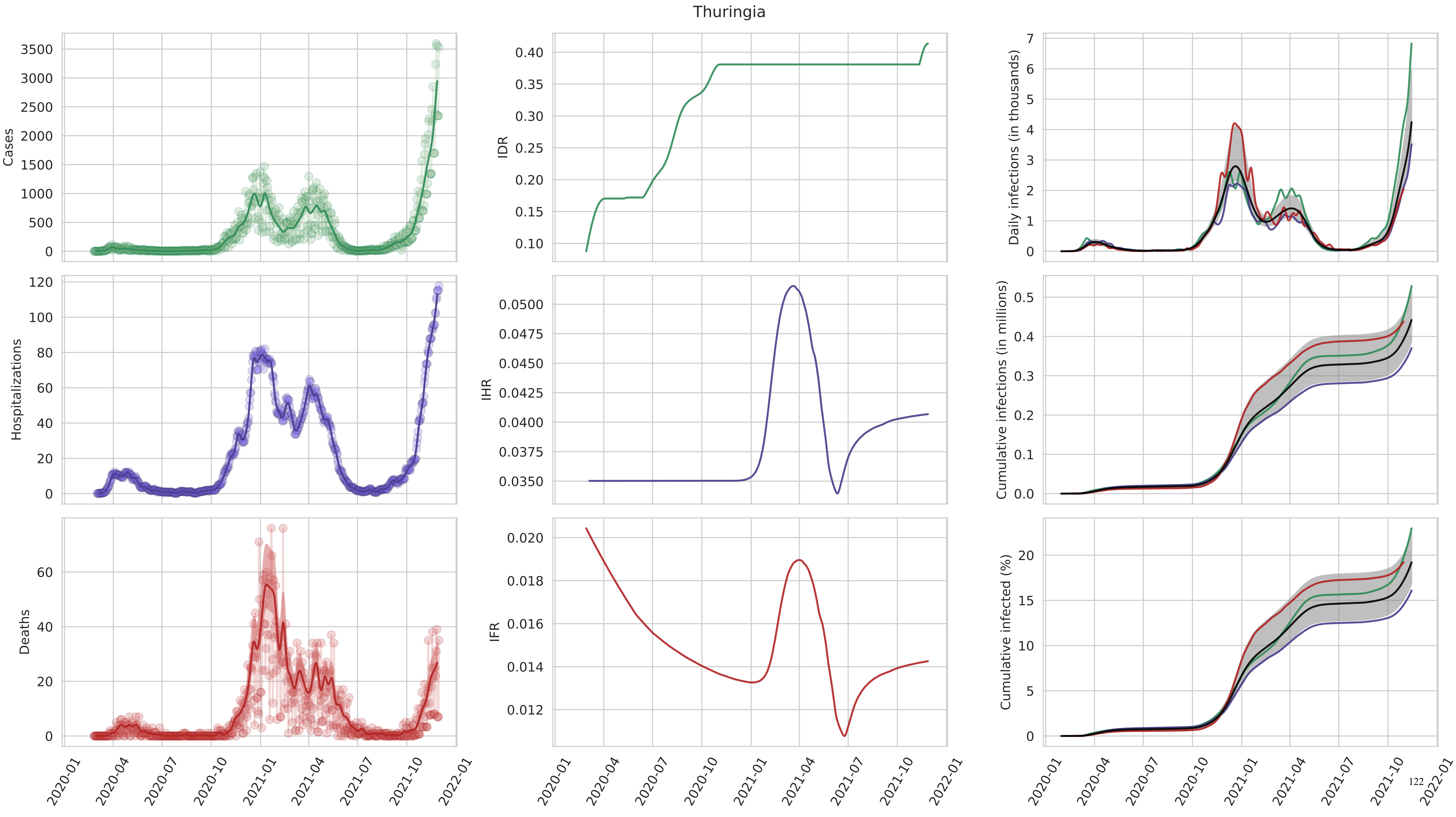

Thuringia
Cases
Hospitalizations
Deaths
IDR
IHR
IFR
Daily infections (in thousands)
Cumulative infections (in millions)
Cumulative infected (%)
2020-01
2020-04
2020-07
2020-10
2021-01
2021-04
2021-07
2021-10
2022-01

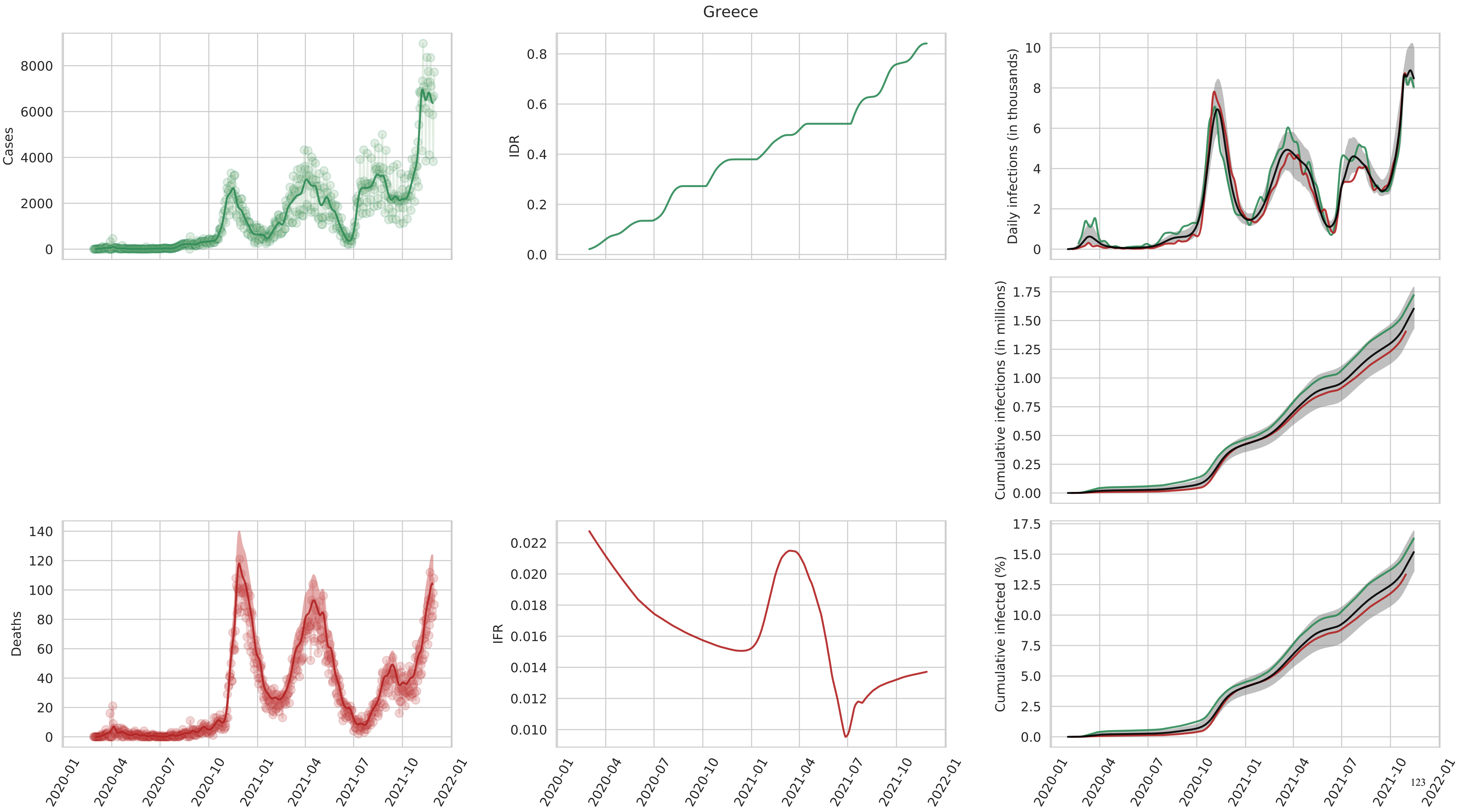

Greece
Cases
IDR
Daily infections (in thousands)
Cumulative infections (in millions)
Deaths
IFR
Cumulative infected (%)

Iceland

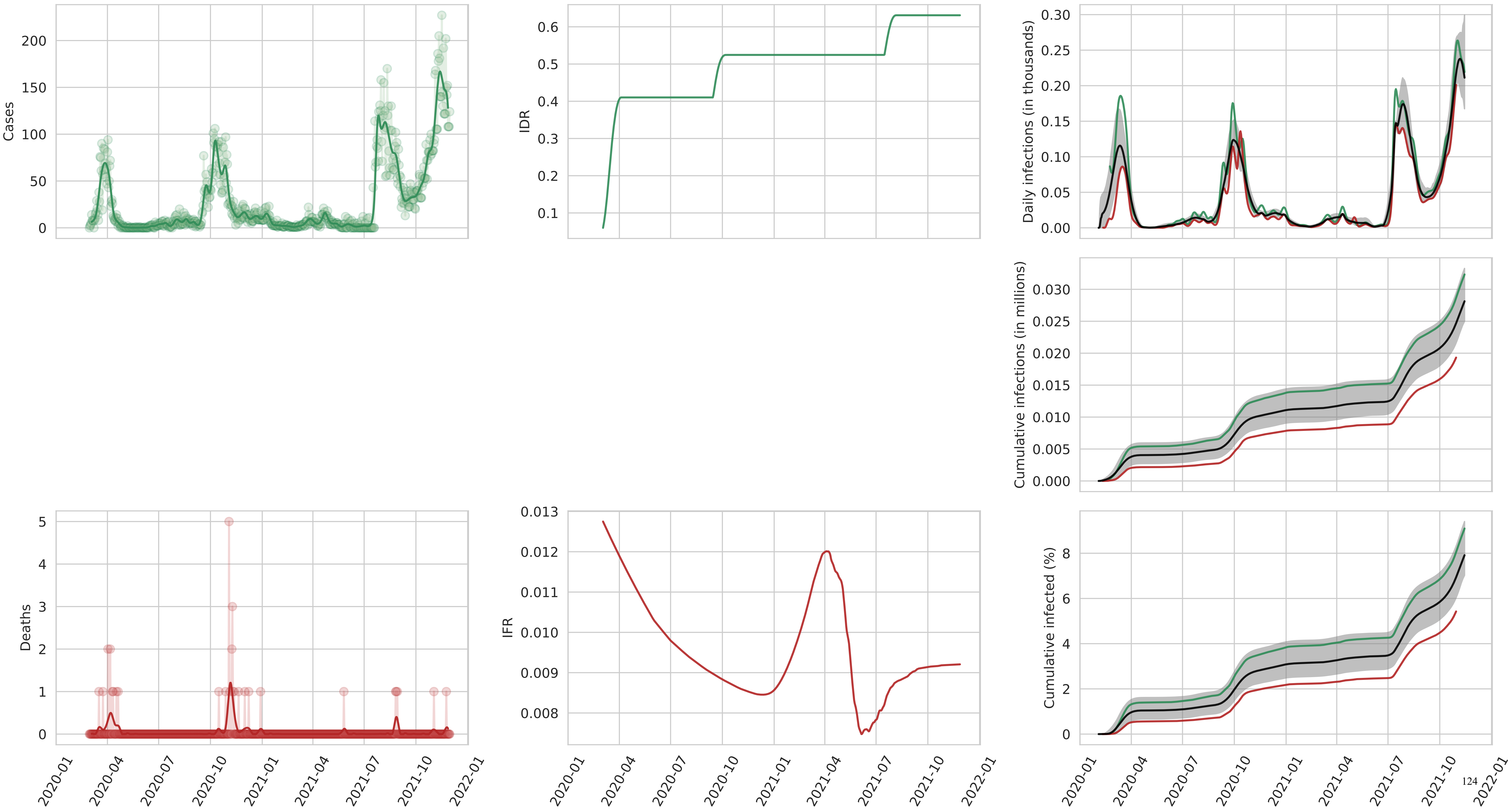
Cases
IDR
Daily infections (in thousands)
Cumulative infections (in millions)
Deaths
IFR
Cumulative infected (%)
2020-01
2020-04
2020-07
2020-10
2021-01
2021-04
2021-07
2021-10
2022-01

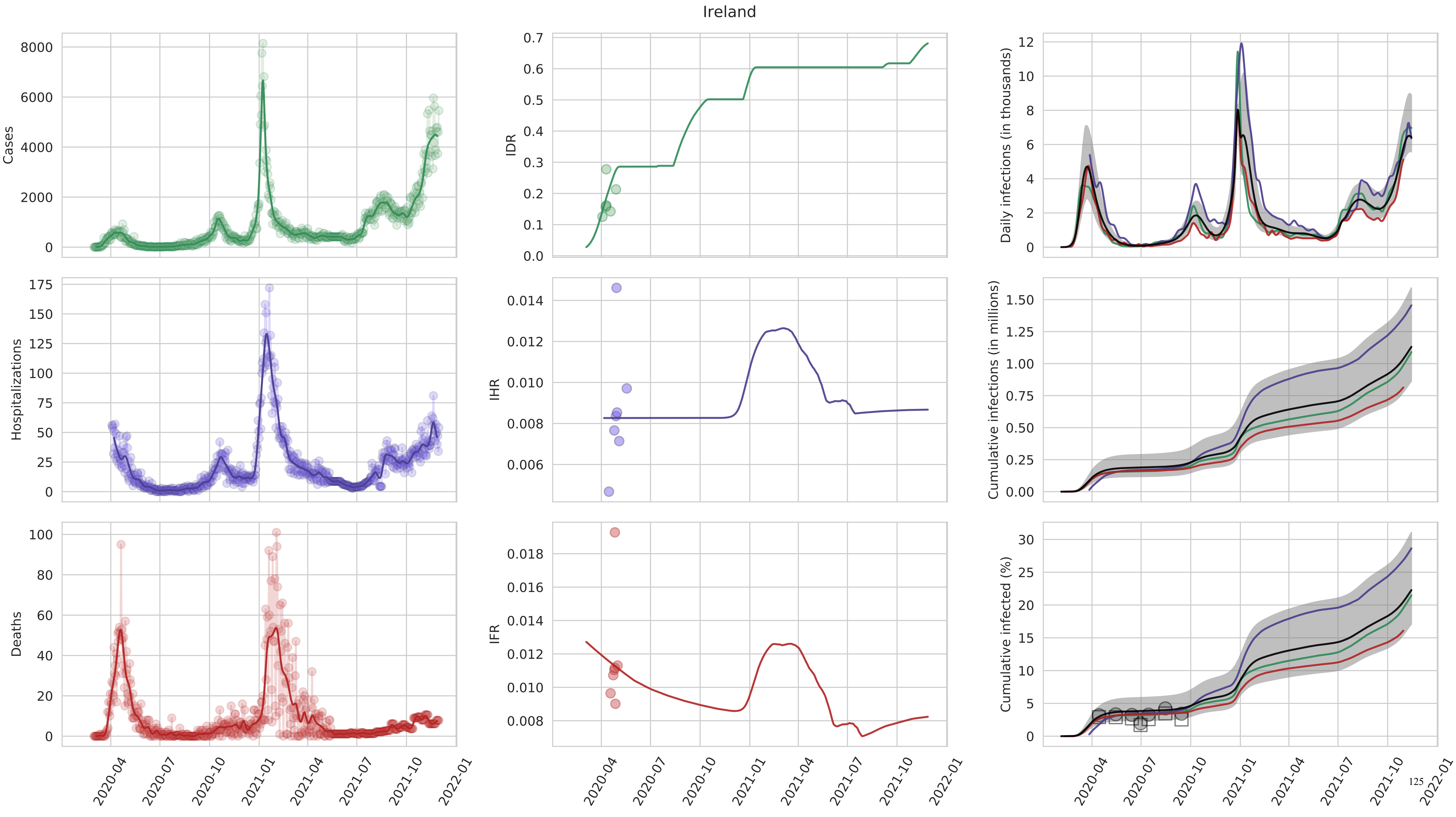
Ireland
Cases
Hospitalizations
Deaths
IDR
IHR
IFR
Daily infections (in thousands)
Cumulative infections (in millions)
Cumulative infected (%)
2020-04
2020-07
2020-10
2021-01
2021-04
2021-07
2021-10
2022-01

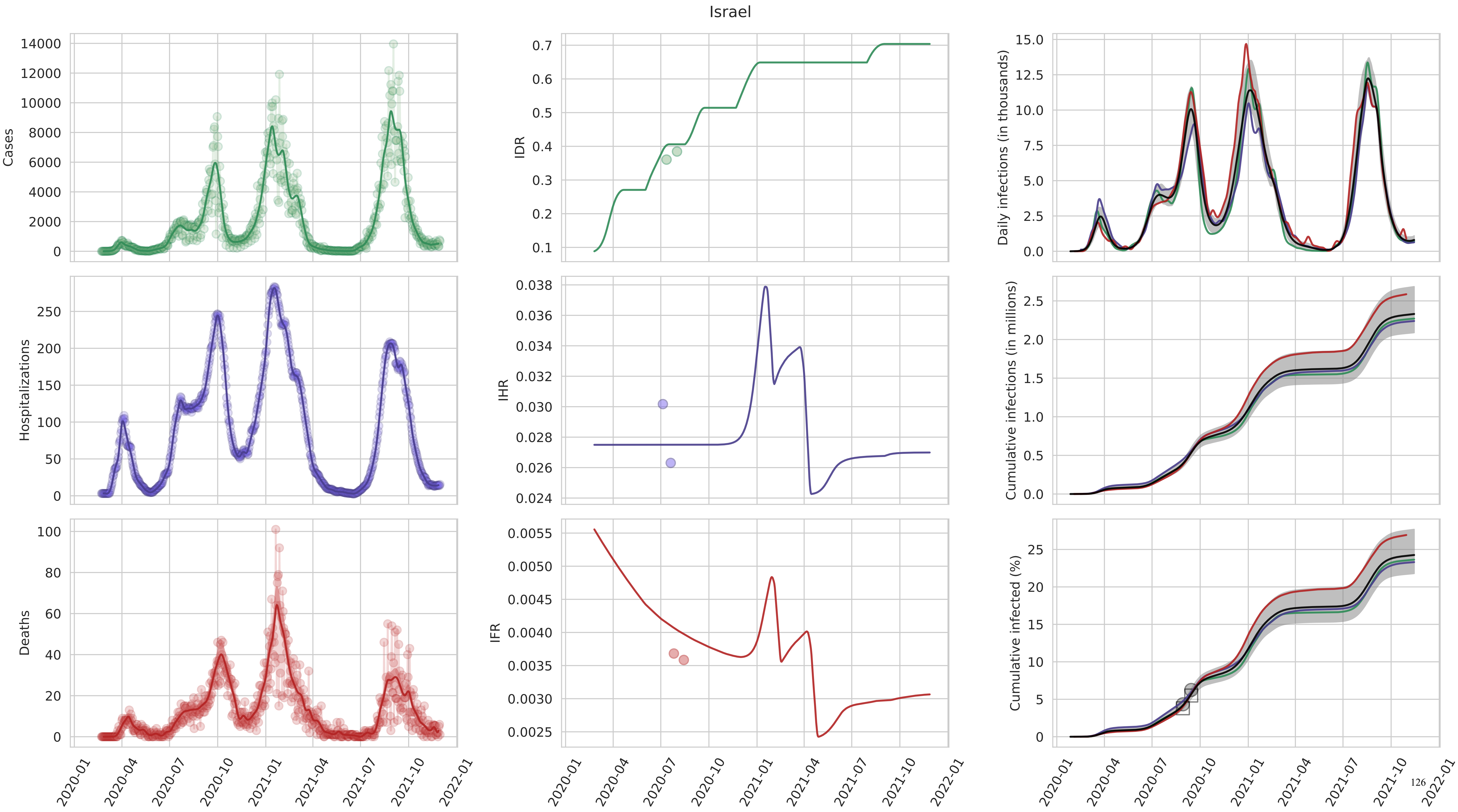
Israel
Cases
Hospitalizations
Deaths
IDR
IHR
IFR
Daily infections (in thousands)
Cumulative infections (in millions)
Cumulative infected (%)
2020-01
2020-04
2020-07
2020-10
2021-01
2021-04
2021-07
2021-10
2022-01

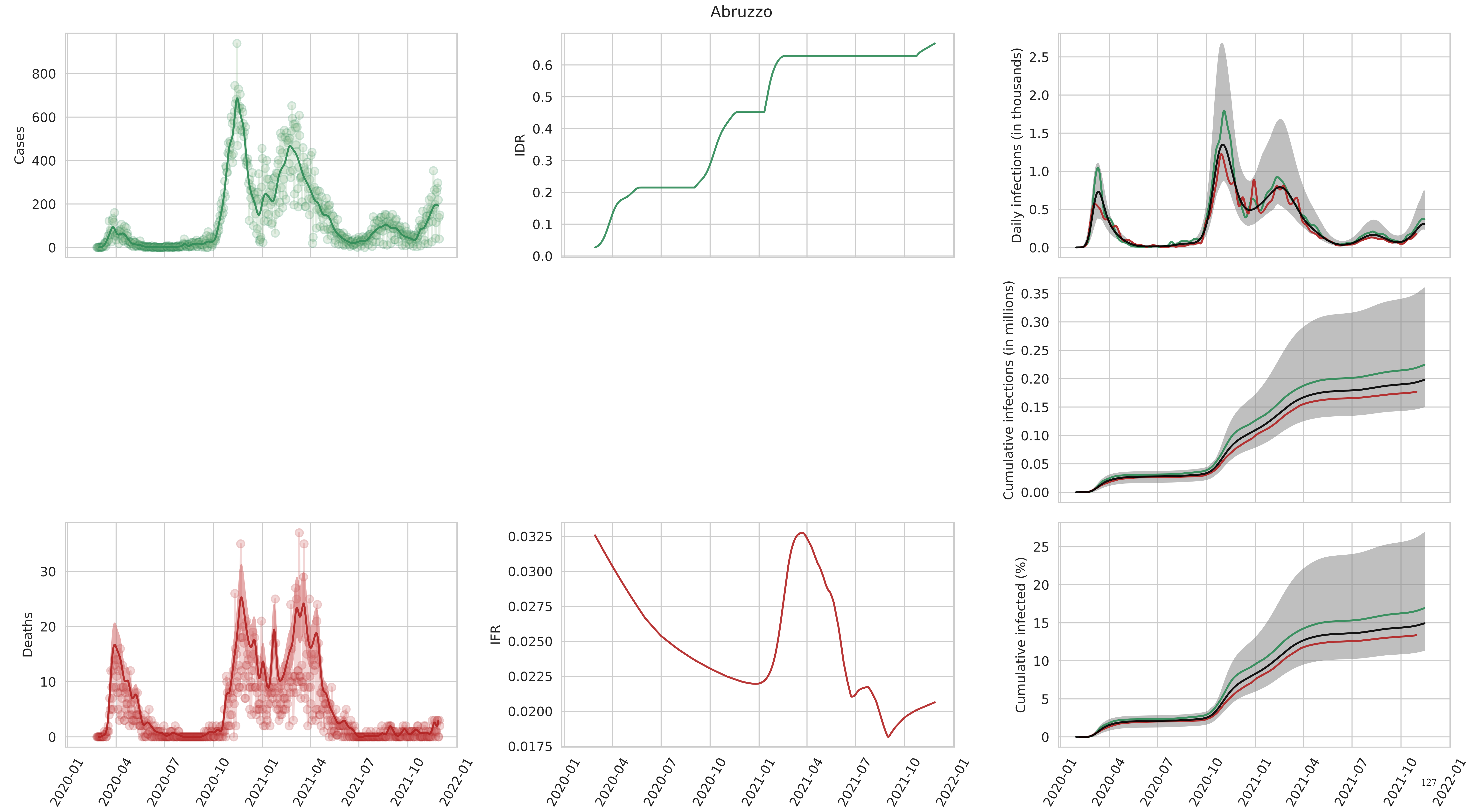
Abruzzo
Cases
IDR
Daily infections (in thousands)
Cumulative infections (in millions)
Deaths
IFR
Cumulative infected (%)
2020-01
2020-04
2020-07
2020-10
2021-01
2021-04
2021-07
2021-10
2022-01

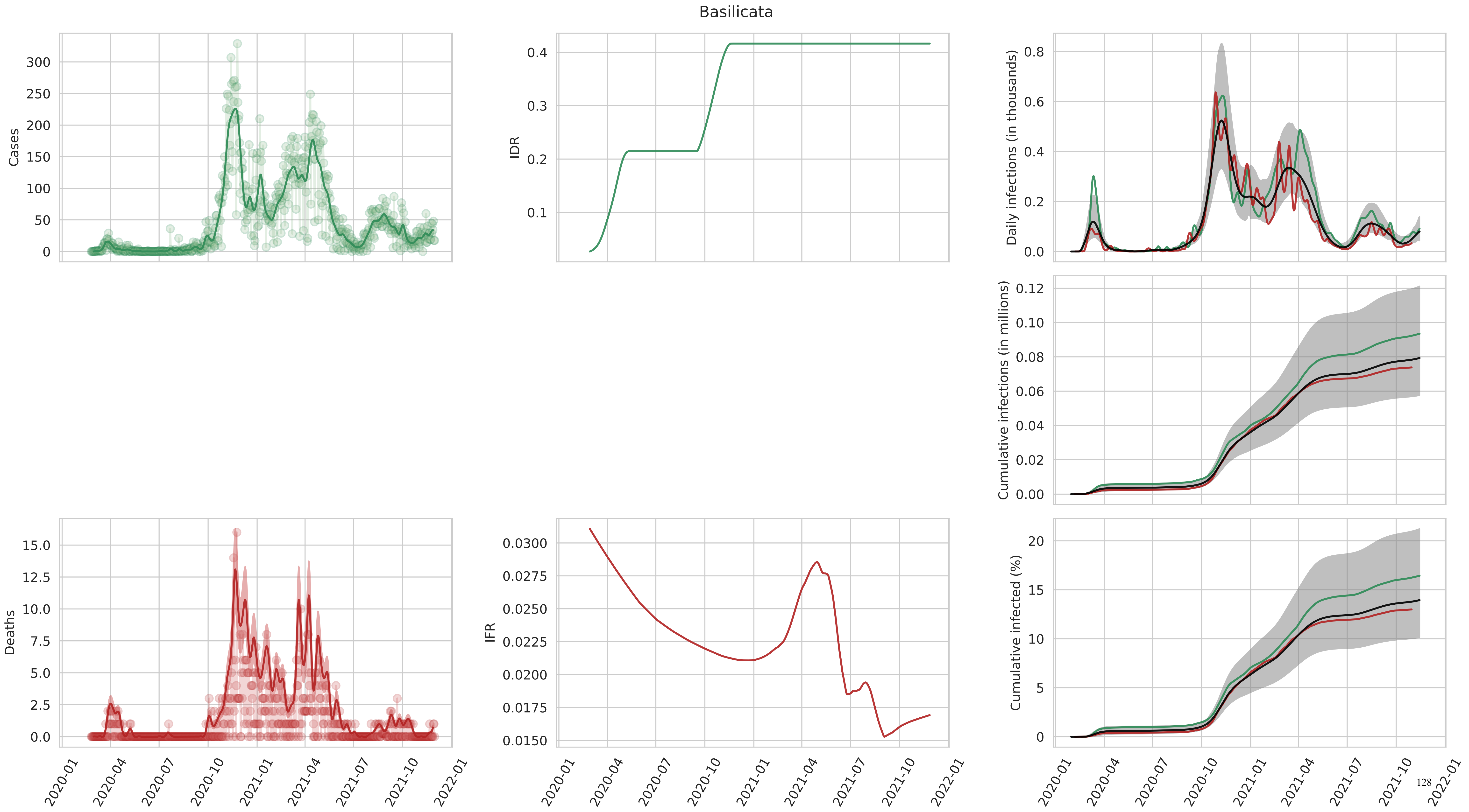

Basilicata
Cases
IDR
Daily infections (in thousands)
Cumulative infections (in millions)
Deaths
IFR
Cumulative infected (%)
2020-01
2020-04
2020-07
2020-10
2021-01
2021-04
2021-07
2021-10
2022-01

Calabria

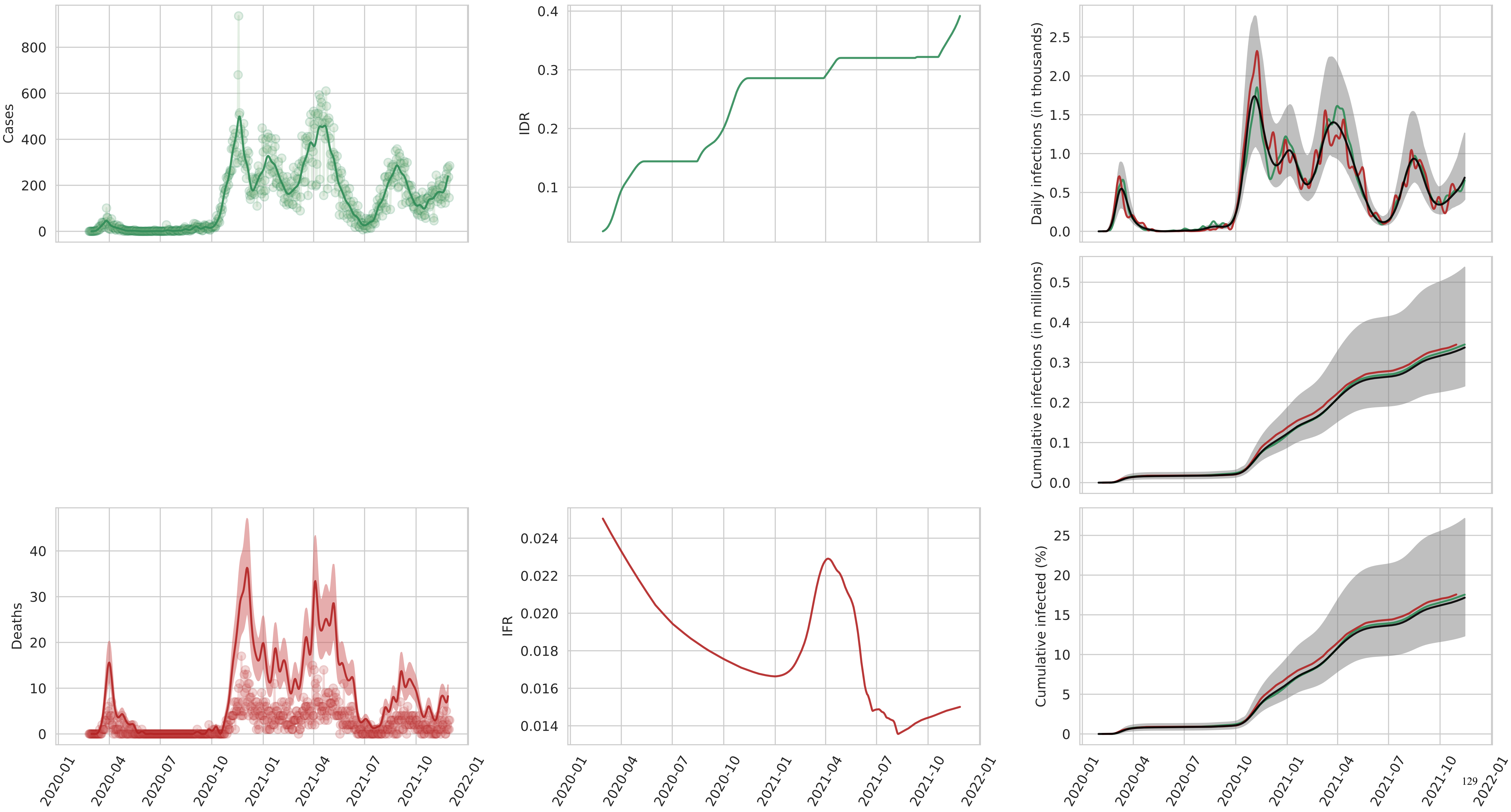

Cases
IDR
Daily infections (in thousands)
Cumulative infections (in millions)
Deaths
IFR
Cumulative infected (%)
2020-01
2020-04
2020-07
2020-10
2021-01
2021-04
2021-07
2021-10
2022-01

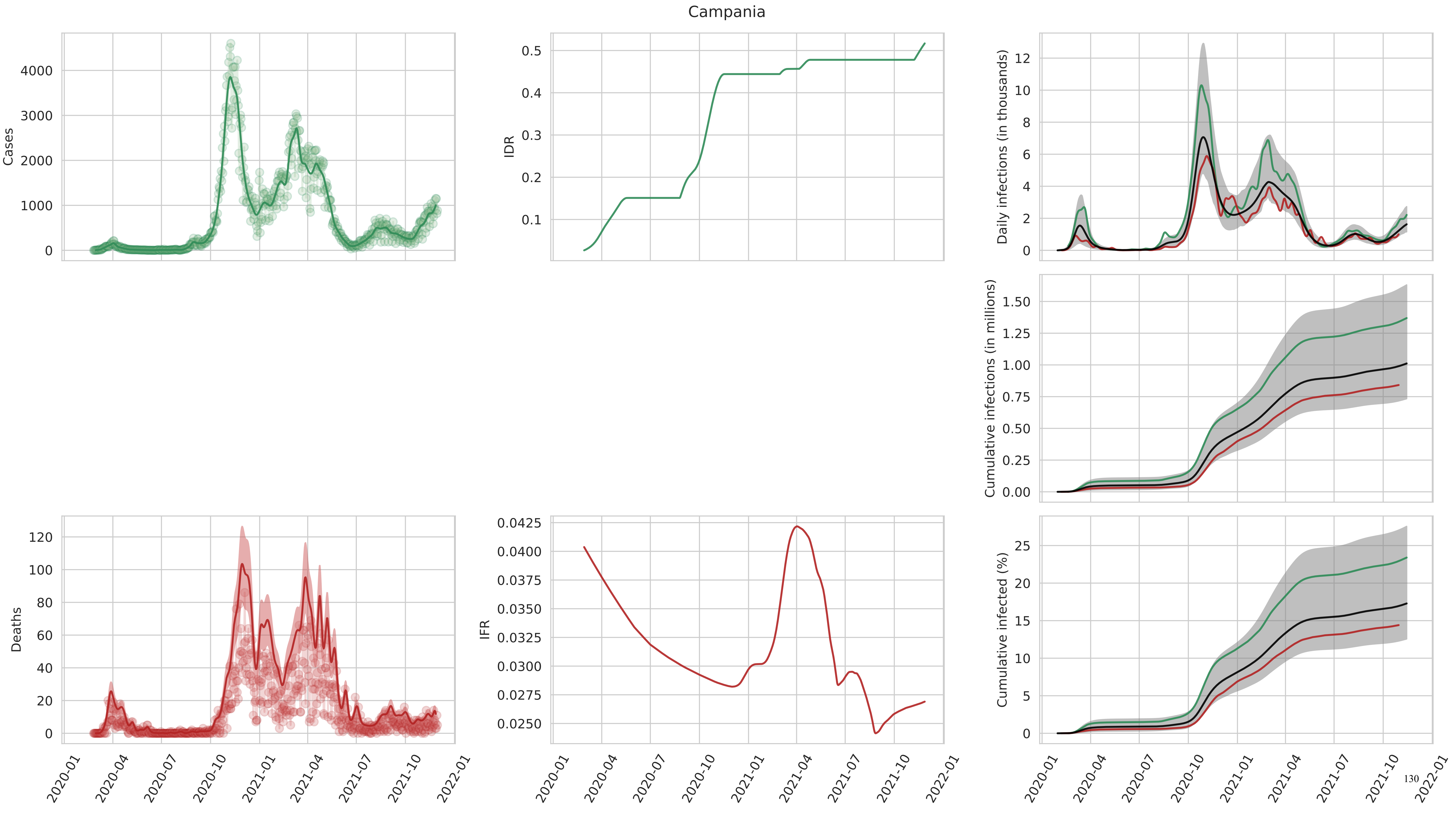
Campania
Cases
IDR
Daily infections (in thousands)
Cumulative infections (in millions)
Deaths
IFR
Cumulative infected (%)
2020-01
2020-04
2020-07
2020-10
2021-01
2021-04
2021-07
2021-10
2022-01

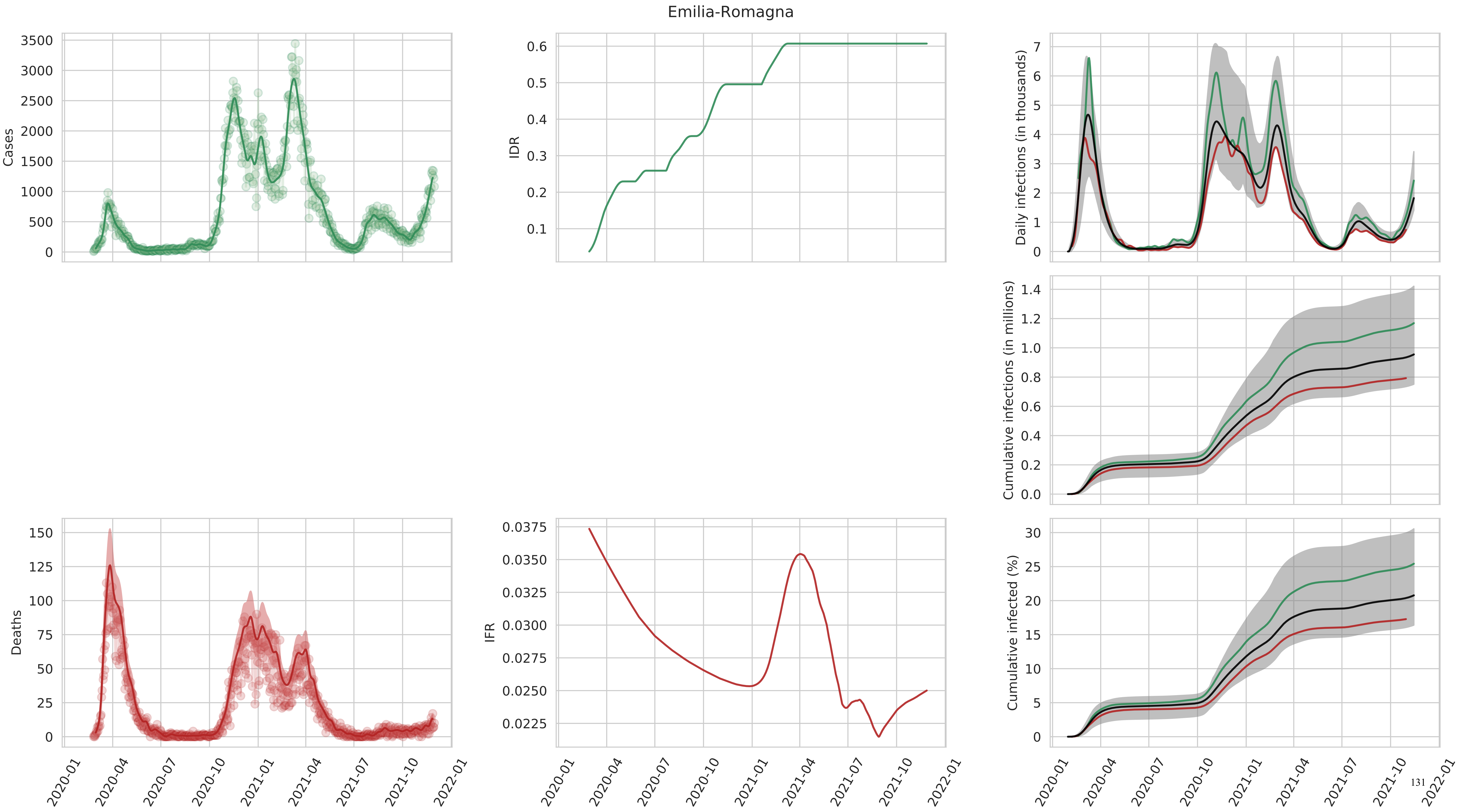

Emilia-Romagna
Cases
IDR
Daily infections (in thousands)
Cumulative infections (in millions)
Deaths
IFR
Cumulative infected (%)
2020-01
2020-04
2020-07
2020-10
2021-01
2021-04
2021-07
2021-10
2022-01

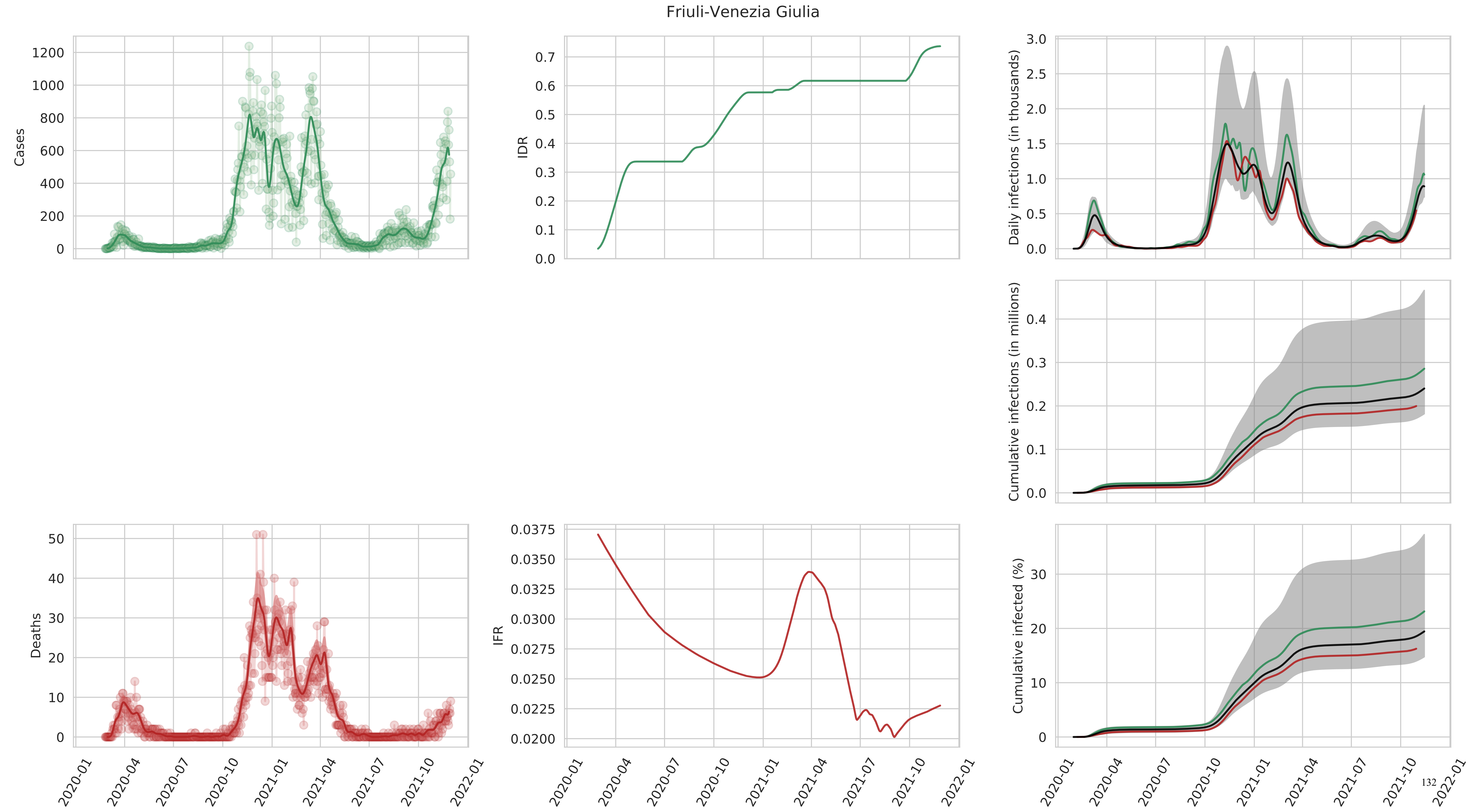

Friuli-Venezia Giulia
Cases
IDR
Daily infections (in thousands)
Cumulative infections (in millions)
Deaths
IFR
Cumulative infected (%)
2020-01
2020-04
2020-07
2020-10
2021-01
2021-04
2021-07
2021-10
2022-01

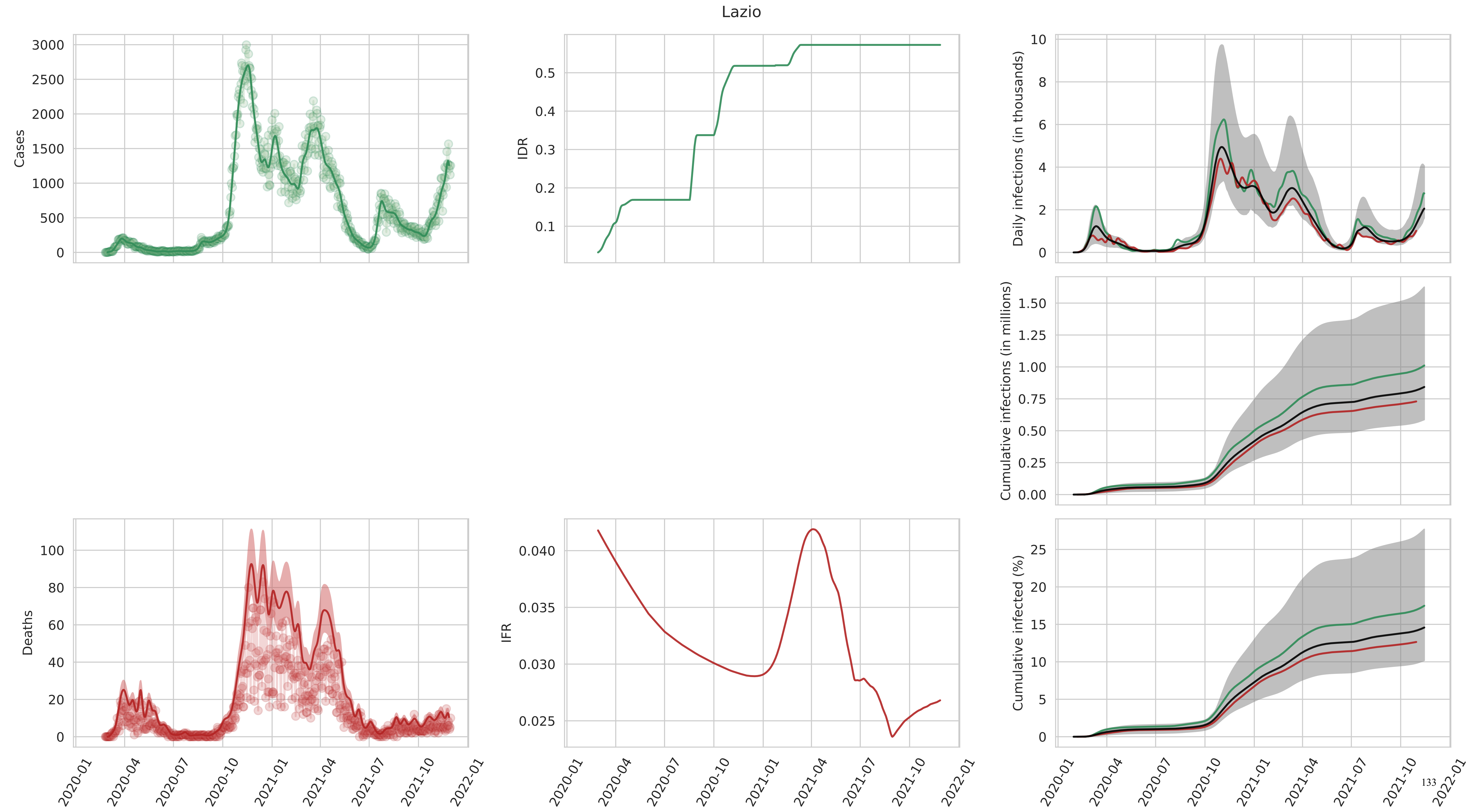
Lazio
Cases
IDR
Daily infections (in thousands)
Cumulative infections (in millions)
Deaths
IFR
Cumulative infected (%)
2020-01
2020-04
2020-07
2020-10
2021-01
2021-04
2021-07
2021-10
2022-01

Liguria

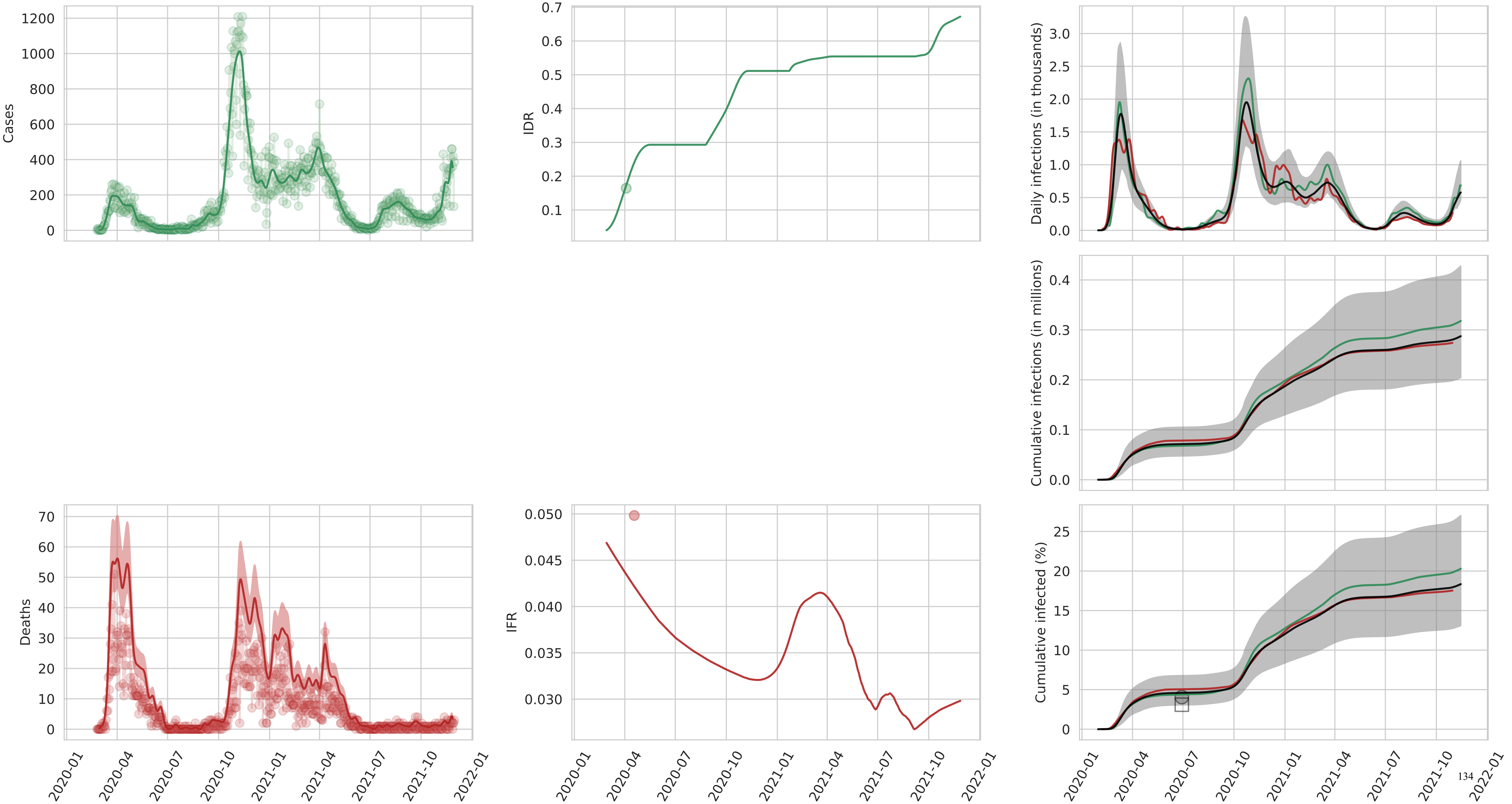

Cases
IDR
Daily infections (in thousands)
Cumulative infections (in millions)
Deaths
IFR
Cumulative infected (%)
2020-01
2020-04
2020-07
2020-10
2021-01
2021-04
2021-07
2021-10
2022-01

Lombardia

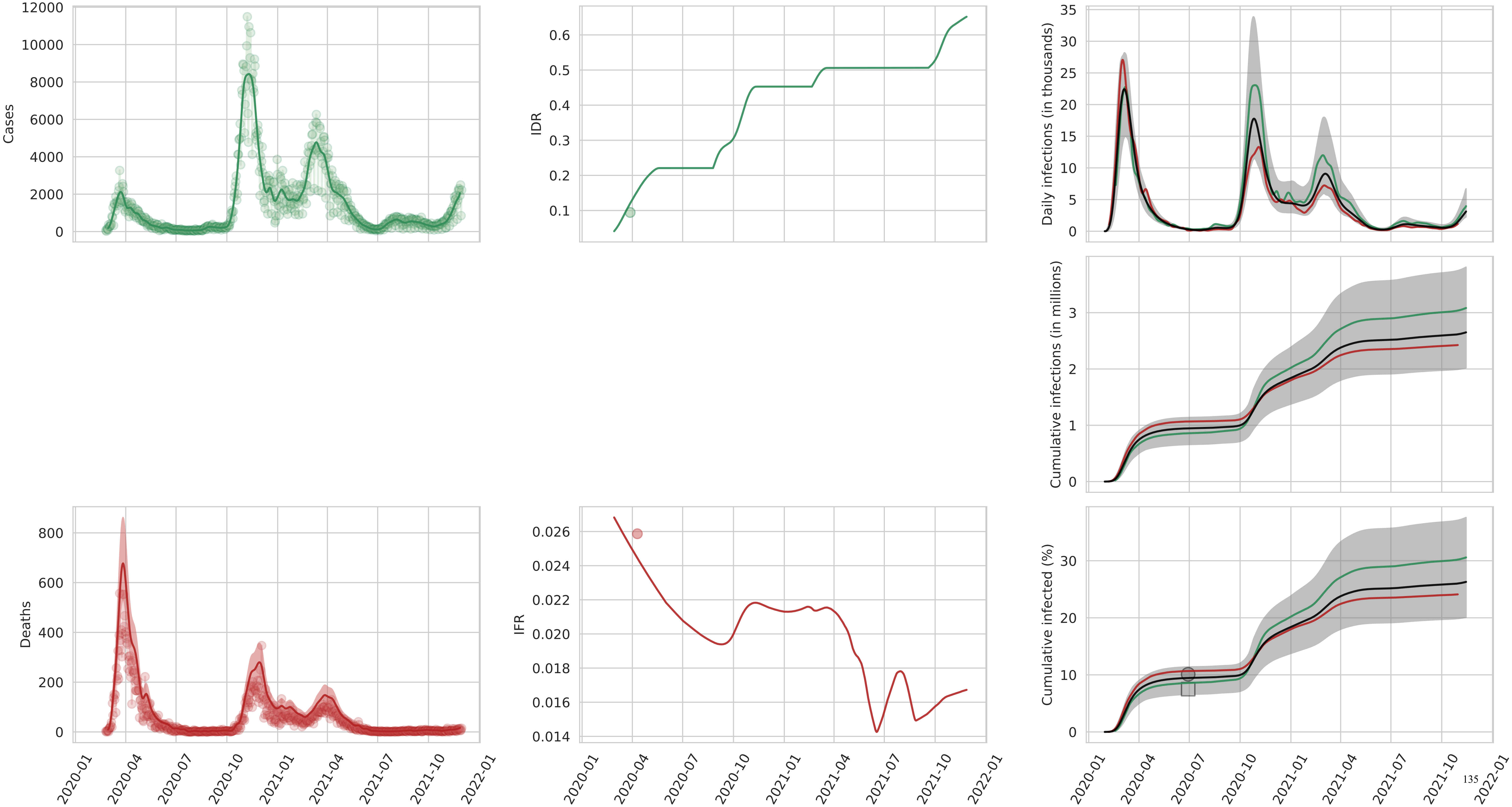

Cases
12000
10000
8000
6000
4000
2000
0
IDR
0.6
0.5
0.4
0.3
0.2
0.1
Daily infections (in thousands)
35
30
25
20
15
10
5
0
Cumulative infections (in millions)
3
2
1
0
Deaths
800
600
400
200
0
IFR
0.026
0.024
0.022
0.020
0.018
0.016
0.014
Cumulative infected (%)
30
20
10
0
2020-01
2020-04
2020-07
2020-10
2021-01
2021-04
2021-07
2021-10
2022-01

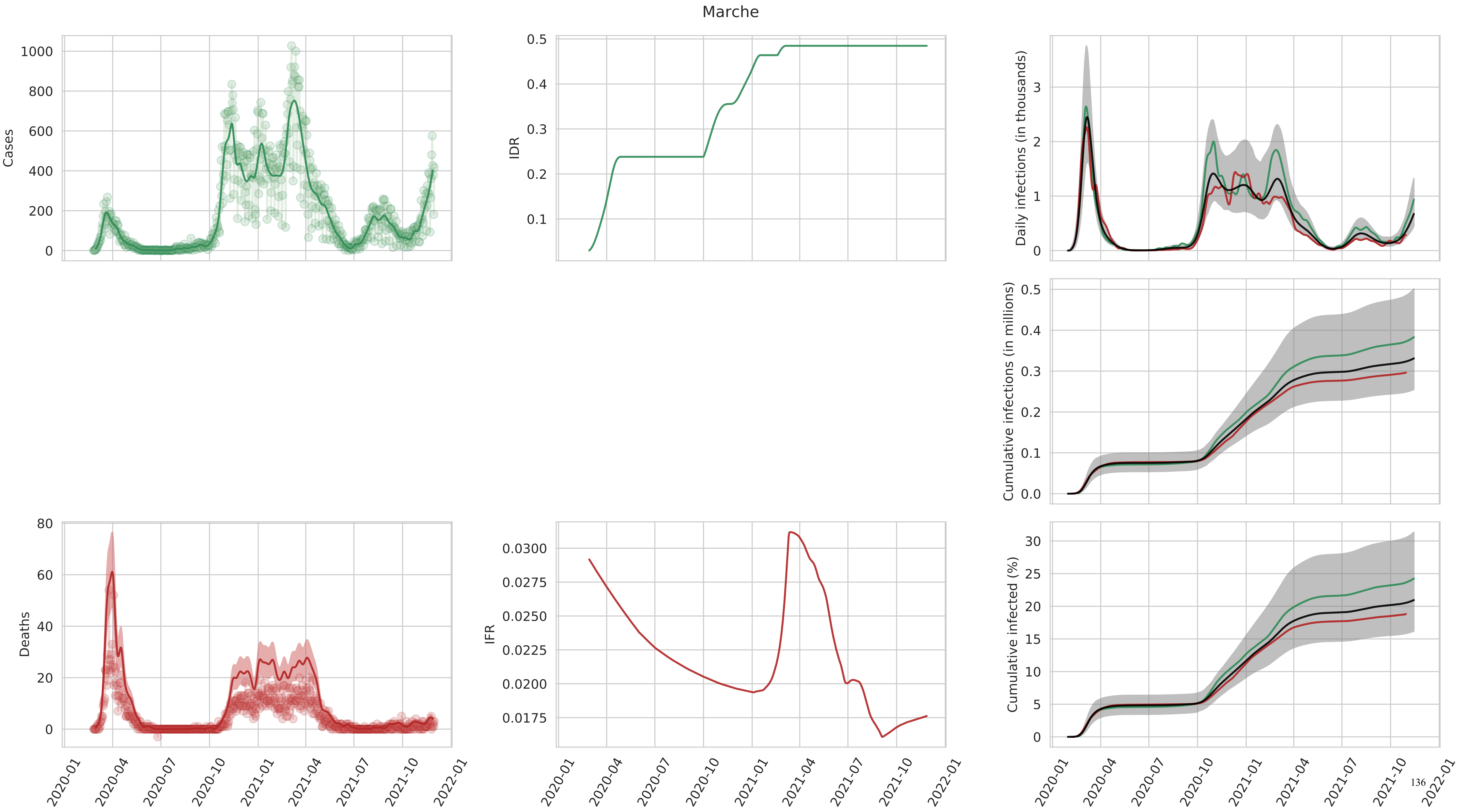
Marche
Cases
IDR
Daily infections (in thousands)
Cumulative infections (in millions)
Deaths
IFR
Cumulative infected (%)
2020-01
2020-04
2020-07
2020-10
2021-01
2021-04
2021-07
2021-10
2022-01

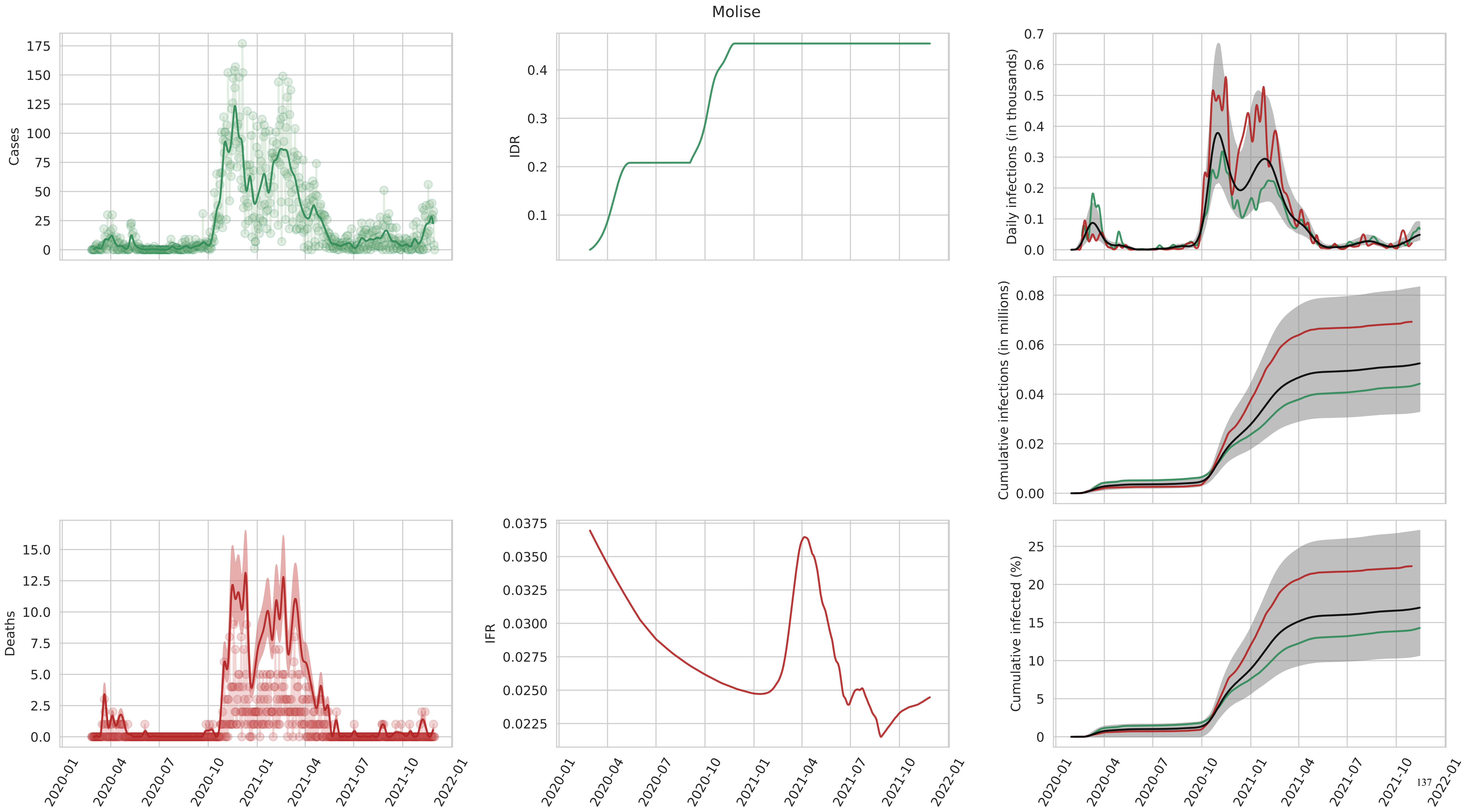
Molise
Cases
IDR
Daily infections (in thousands)
Cumulative infections (in millions)
Deaths
IFR
Cumulative infected (%)
2020-01
2020-04
2020-07
2020-10
2021-01
2021-04
2021-07
2021-10
2022-01

Piemonte

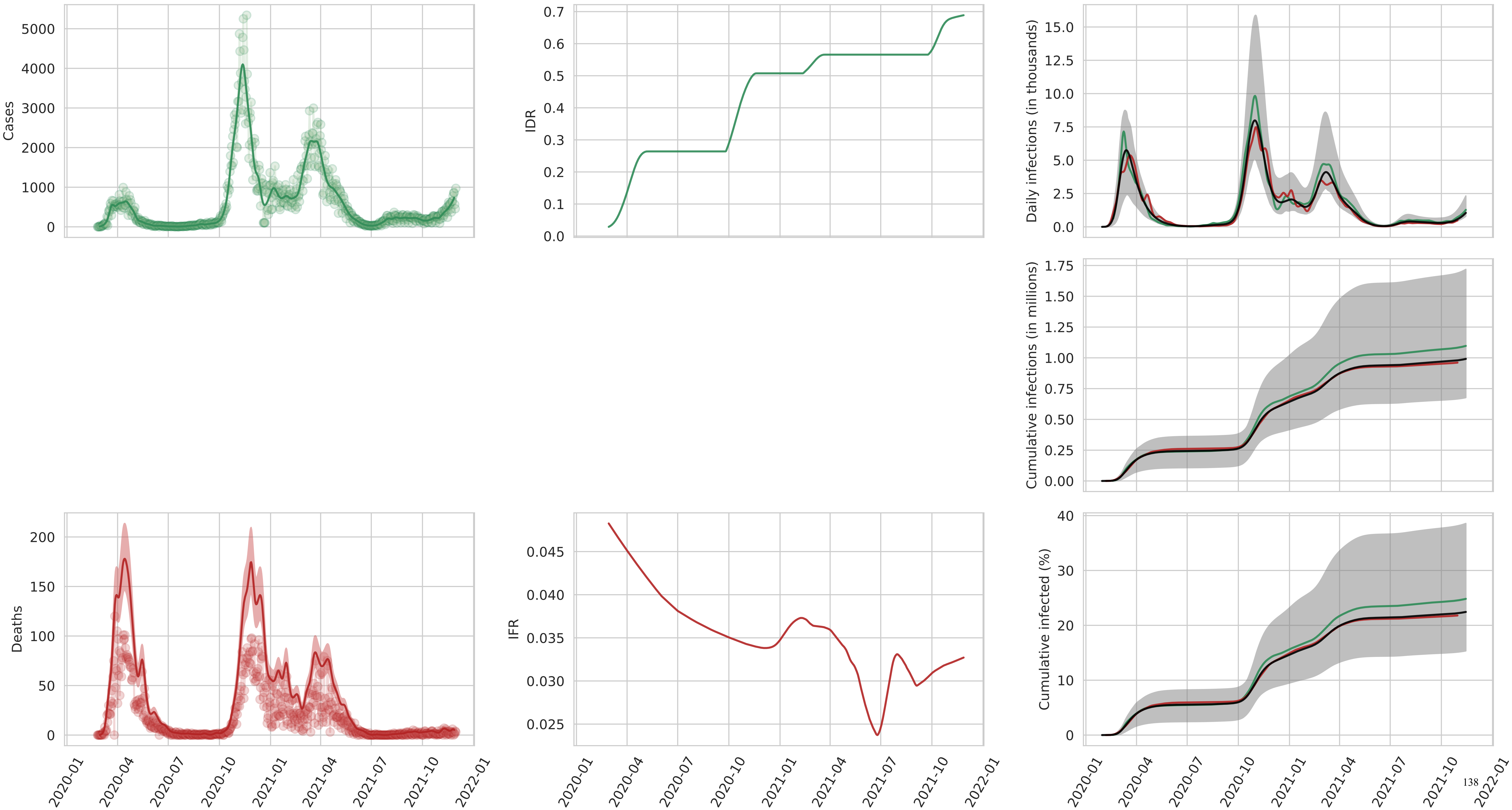
Cases
IDR
Daily infections (in thousands)
Cumulative infections (in millions)
Deaths
IFR
Cumulative infected (%)

Provincia autonoma di Bolzano

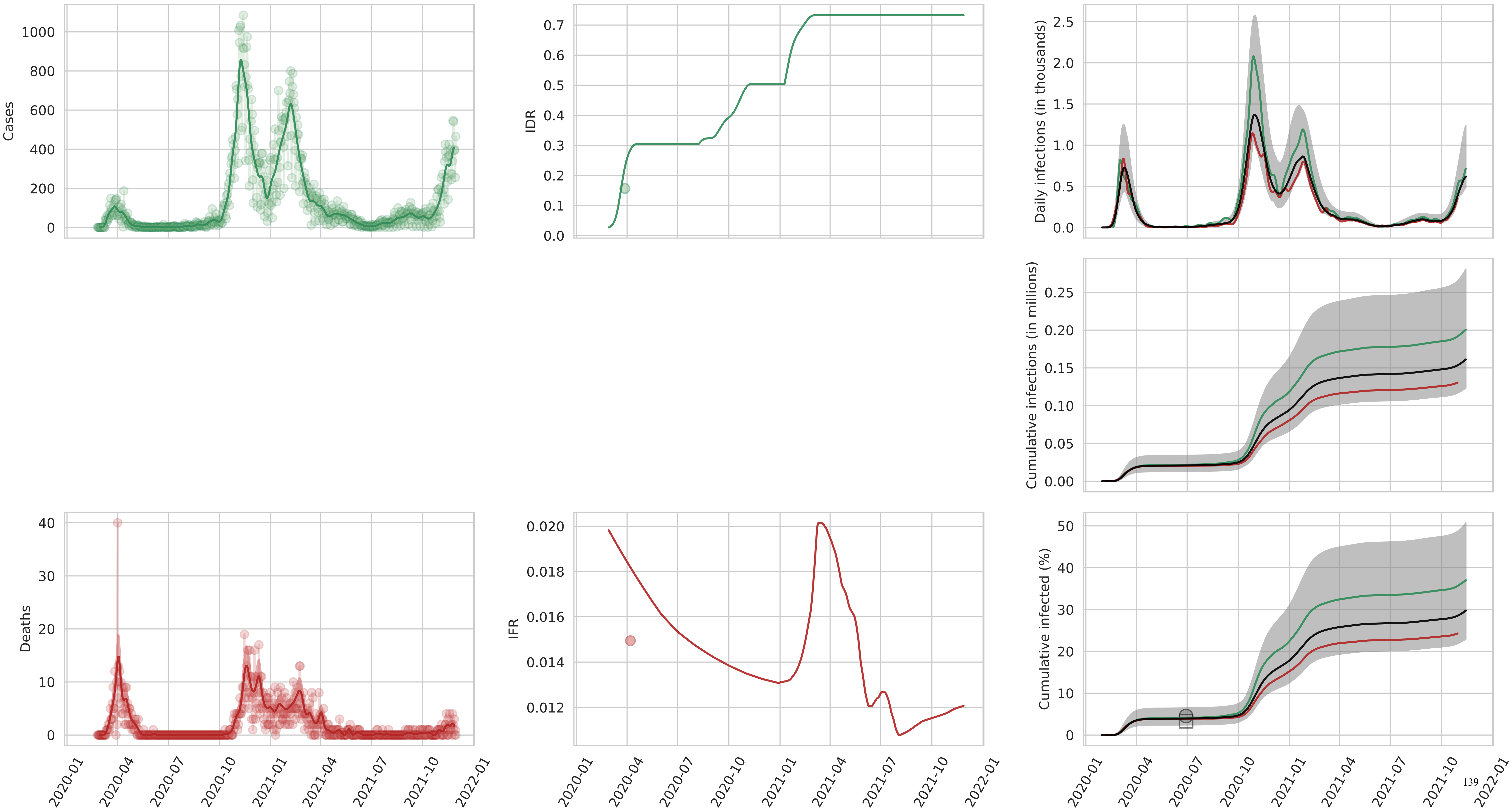

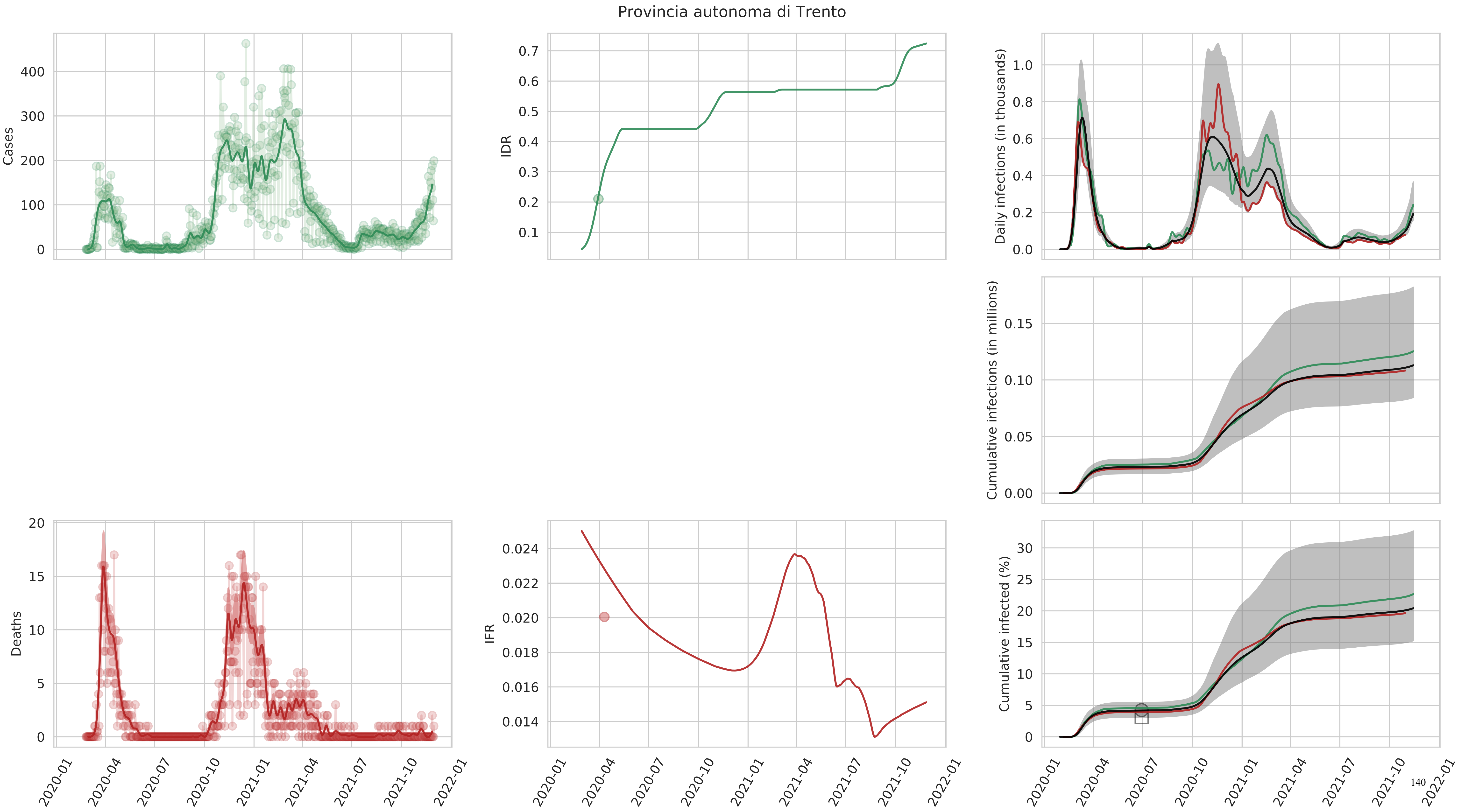

Provincia autonoma di Trento
Cases
IDR
Daily infections (in thousands)
Cumulative infections (in millions)
Deaths
IFR
Cumulative infected (%)
2020-01
2020-04
2020-07
2020-10
2021-01
2021-04
2021-07
2021-10
2022-01

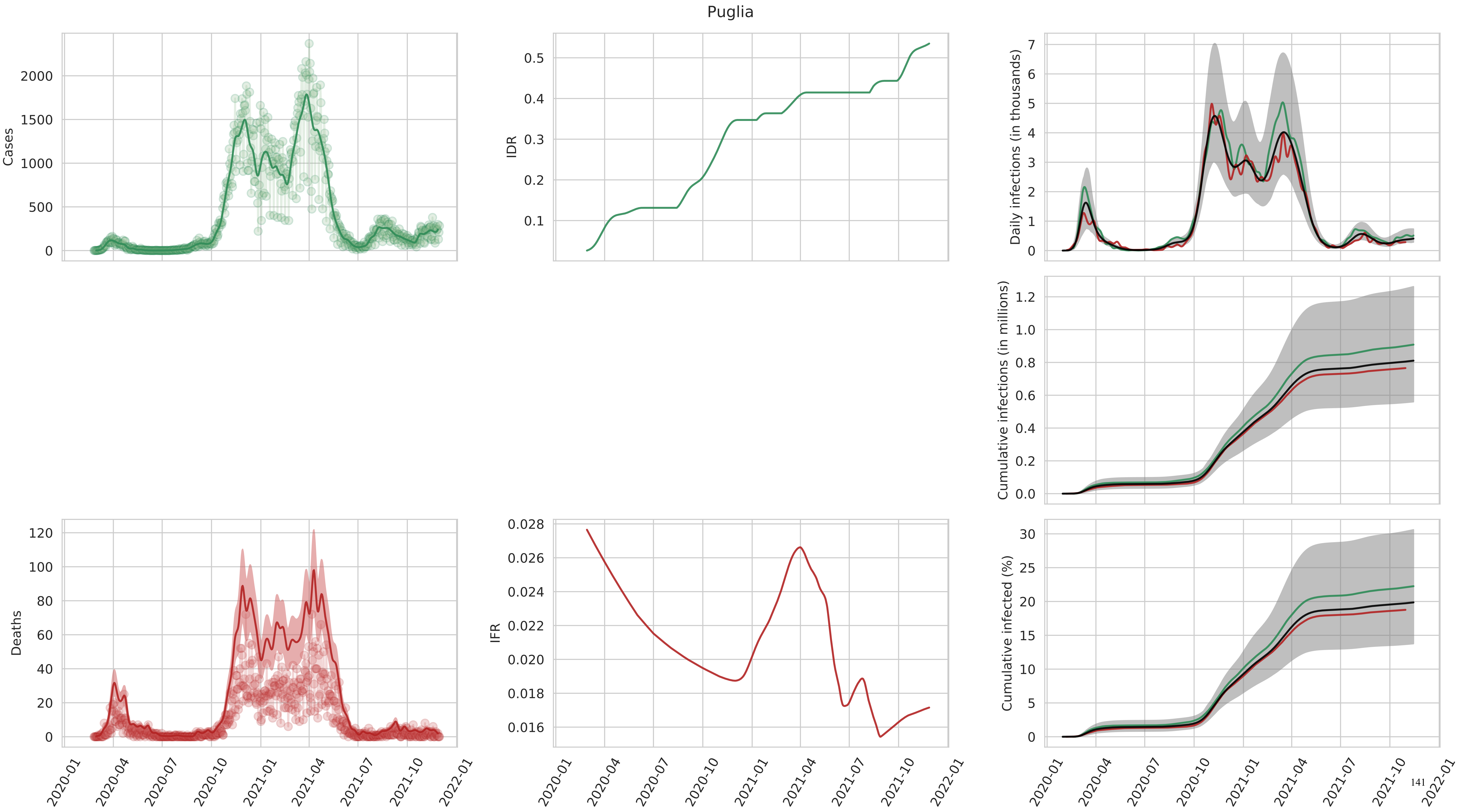

Puglia
Cases
IDR
Daily infections (in thousands)
Cumulative infections (in millions)
Deaths
IFR
Cumulative infected (%)
2020-01
2020-04
2020-07
2020-10
2021-01
2021-04
2021-07
2021-10
2022-01

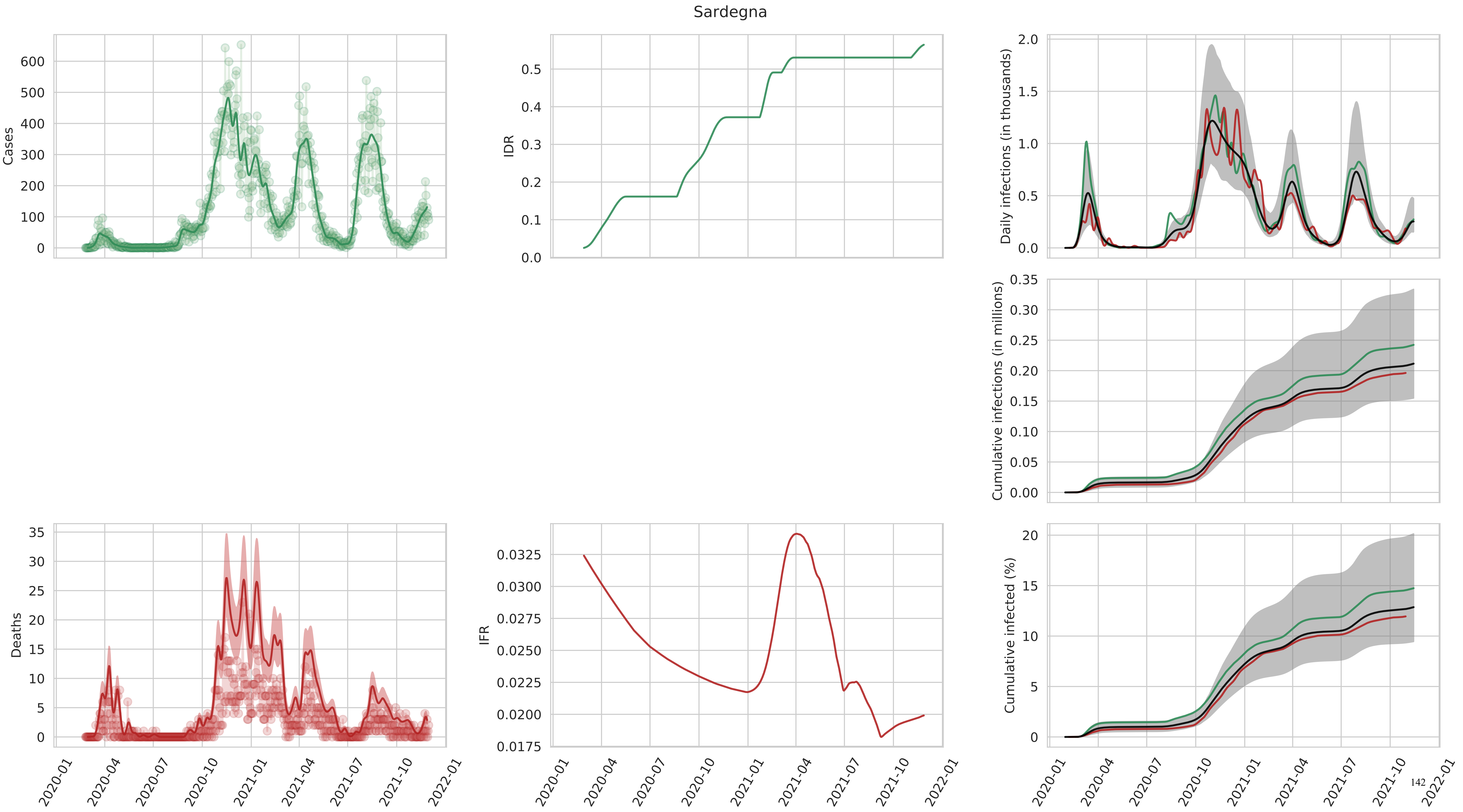

Sardegna
Cases
IDR
Daily infections (in thousands)
Cumulative infections (in millions)
Deaths
IFR
Cumulative infected (%)
2020-01
2020-04
2020-07
2020-10
2021-01
2021-04
2021-07
2021-10
2022-01

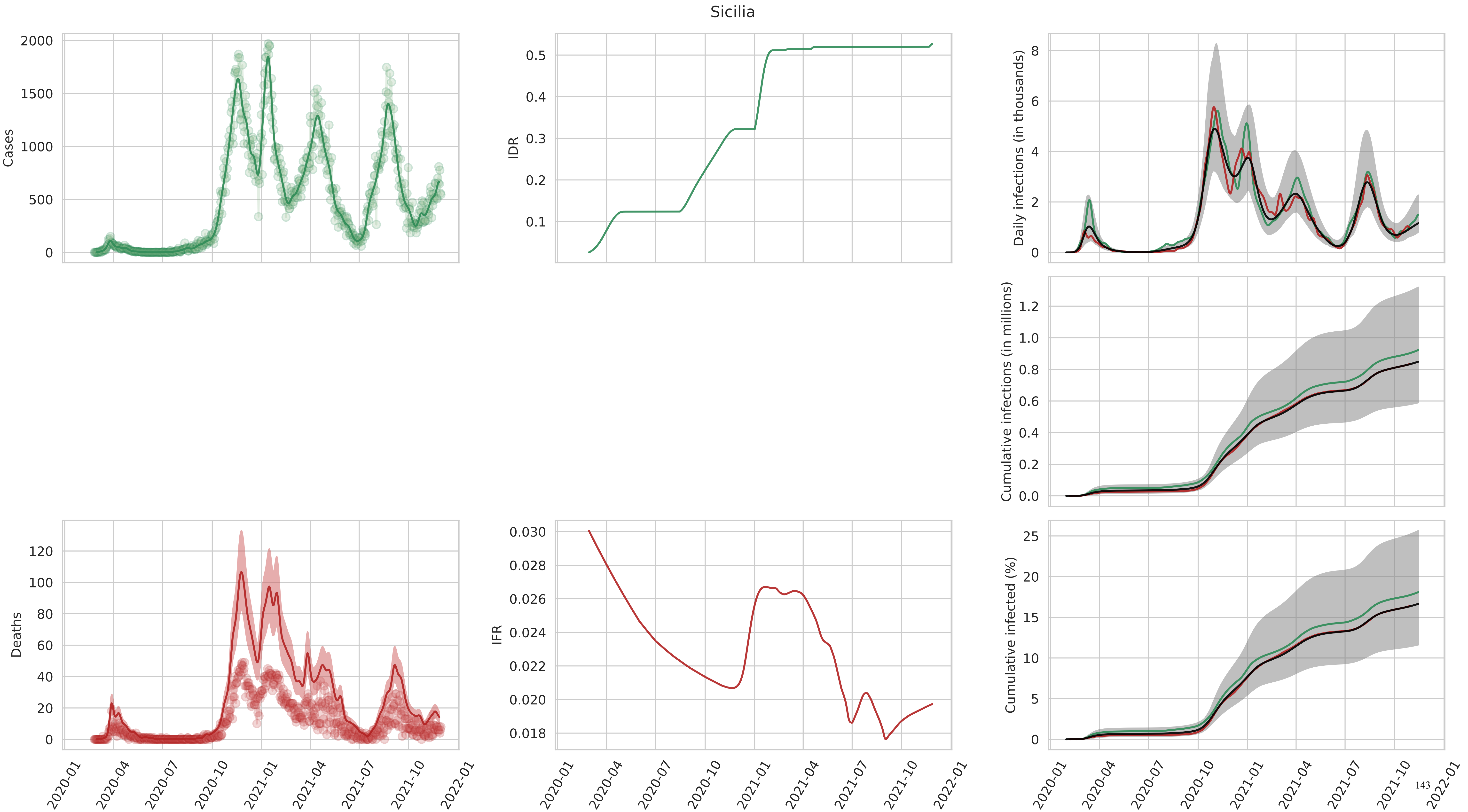

Sicilia
Cases
IDR
Daily infections (in thousands)
Cumulative infections (in millions)
Deaths
IFR
Cumulative infected (%)
2020-01
2020-04
2020-07
2020-10
2021-01
2021-04
2021-07
2021-10
2022-01

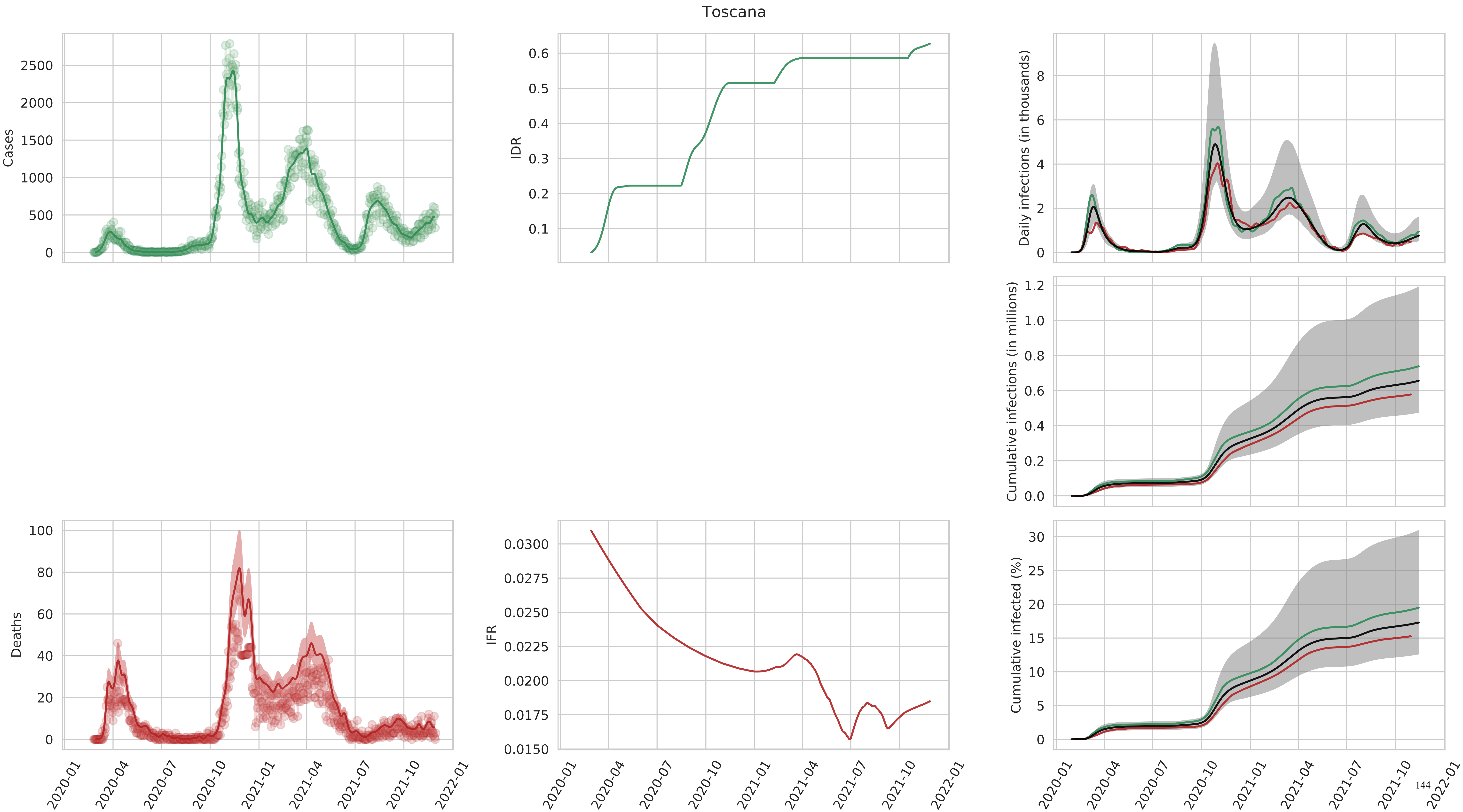

Toscana
Cases
IDR
Daily infections (in thousands)
Cumulative infections (in millions)
Deaths
IFR
Cumulative infected (%)

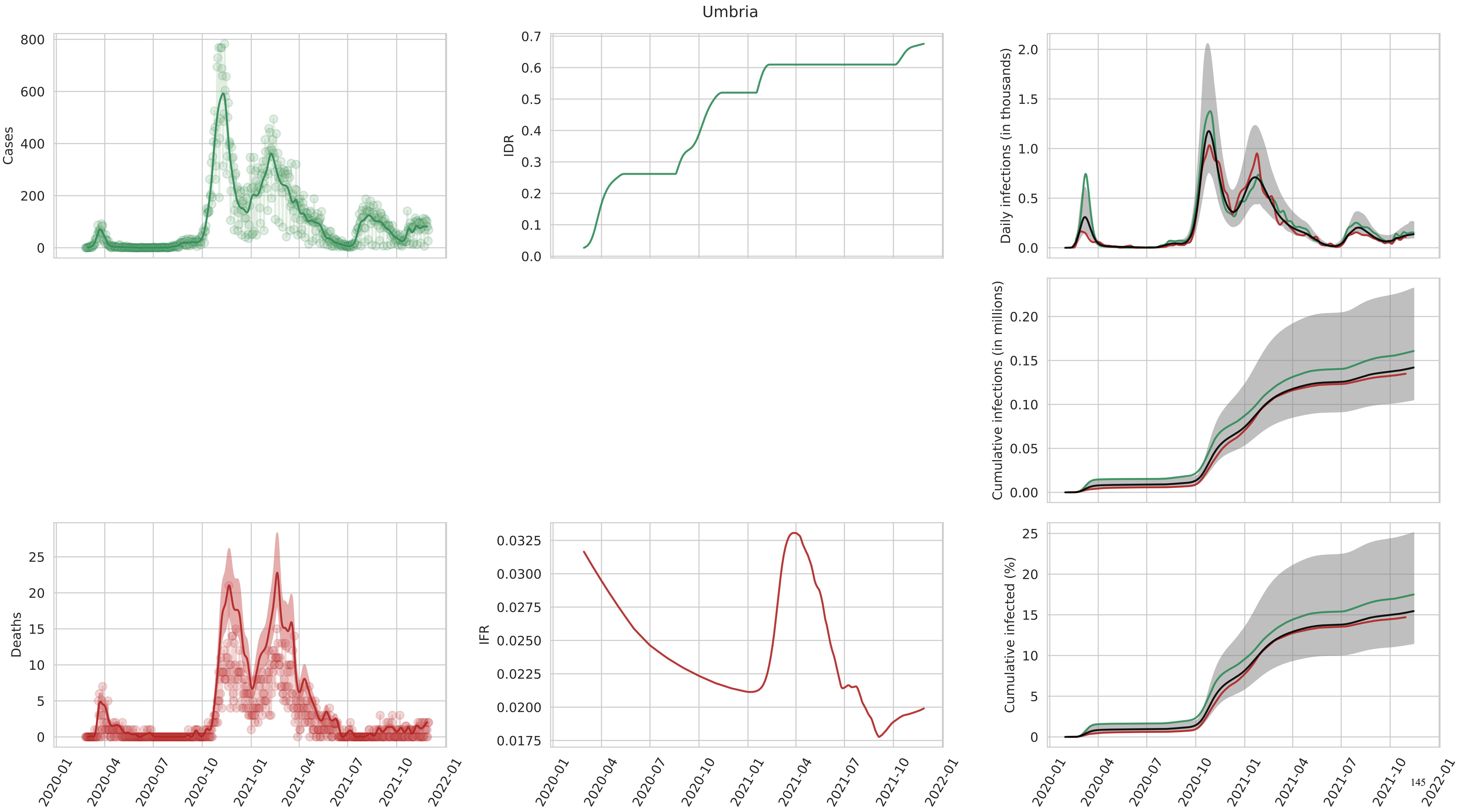

Umbria
Cases
IDR
Daily infections (in thousands)
Cumulative infections (in millions)
Deaths
IFR
Cumulative infected (%)
2020-01
2020-04
2020-07
2020-10
2021-01
2021-04
2021-07
2021-10
2022-01

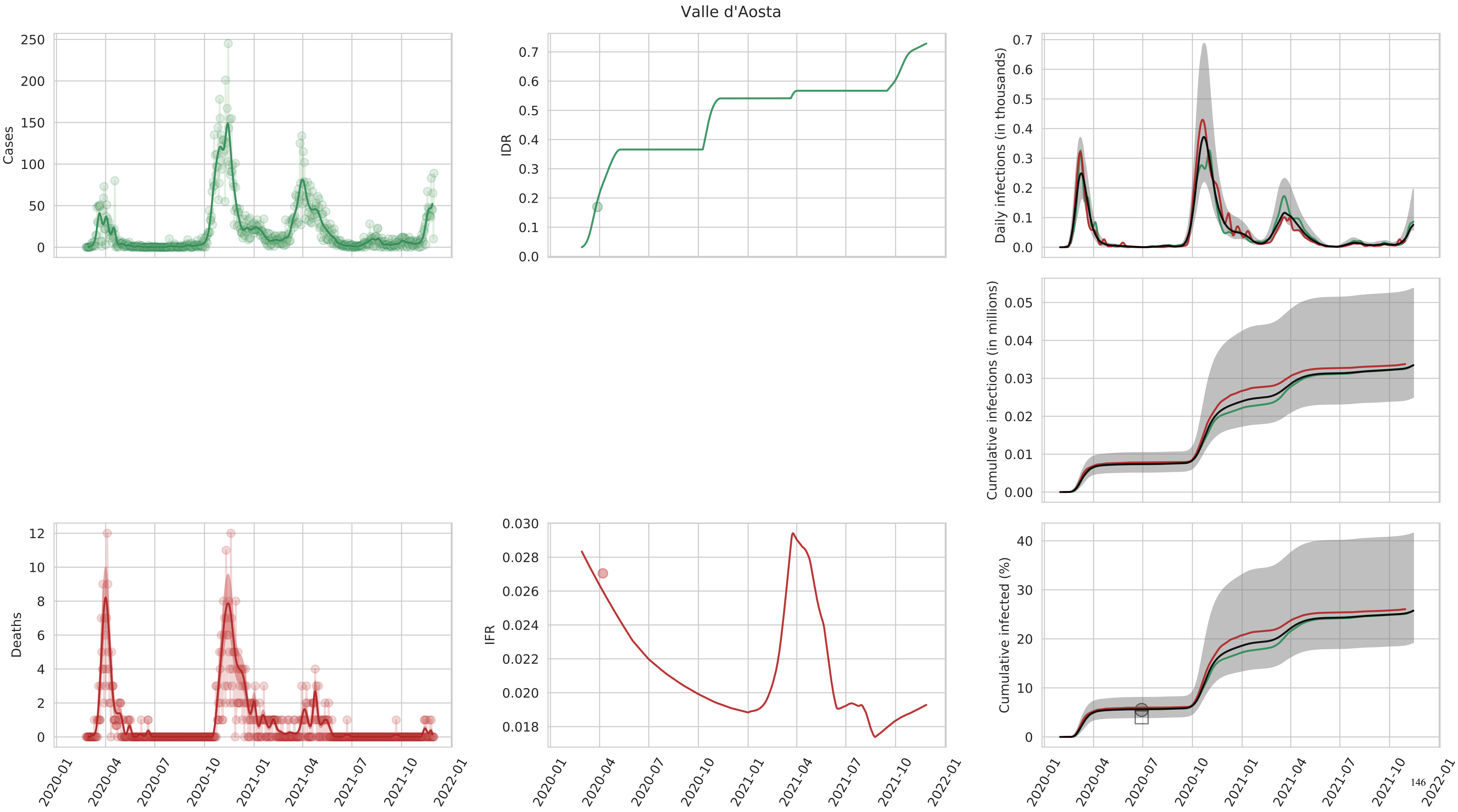
Valle d'Aosta
Cases
IDR
Daily infections (in thousands)
Cumulative infections (in millions)
Deaths
IFR
Cumulative infected (%)
2020-01
2020-04
2020-07
2020-10
2021-01
2021-04
2021-07
2021-10
2022-01

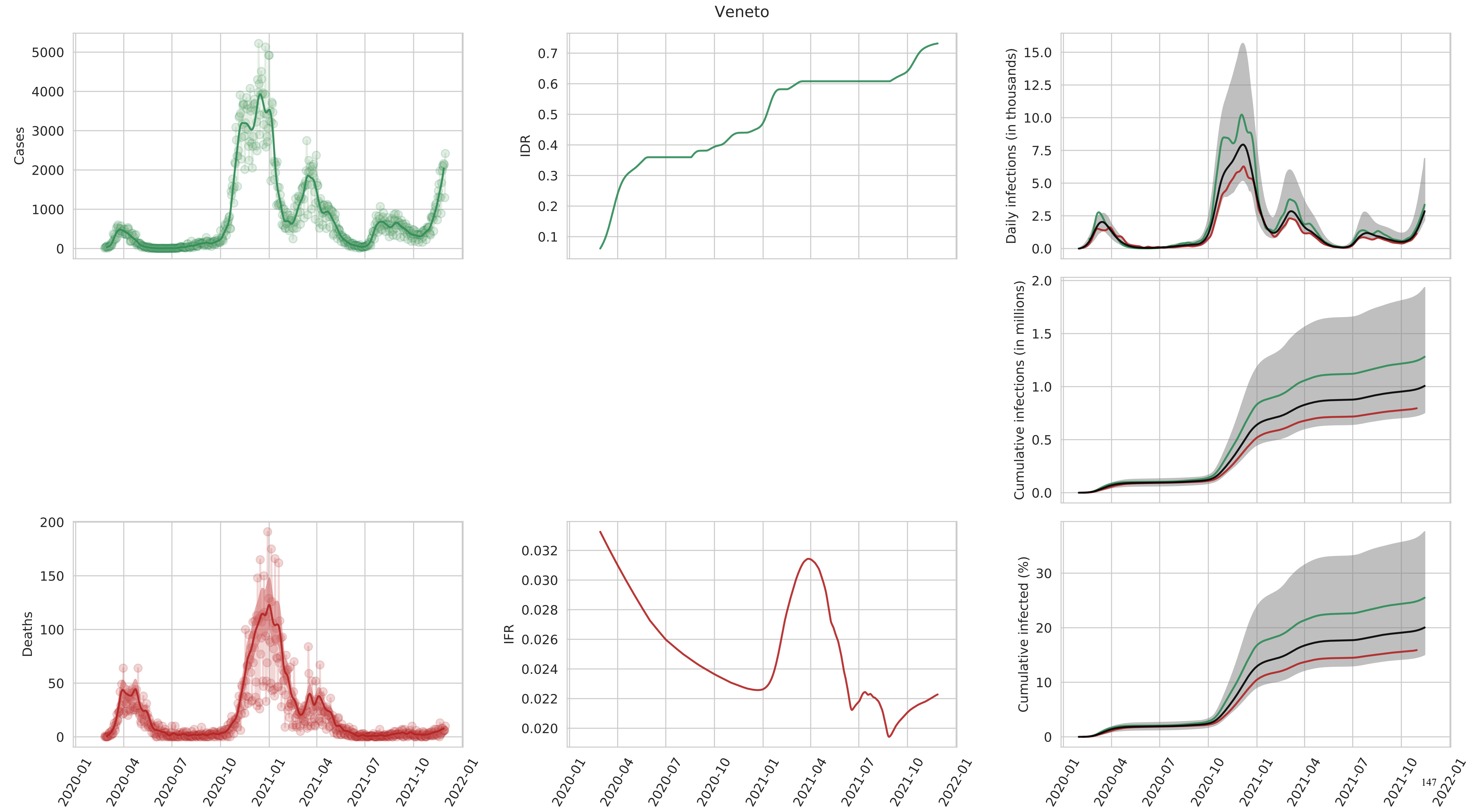

Veneto
Cases
IDR
Daily infections (in thousands)
Cumulative infections (in millions)
Deaths
IFR
Cumulative infected (%)
2020-01
2020-04
2020-07
2020-10
2021-01
2021-04
2021-07
2021-10
2022-01

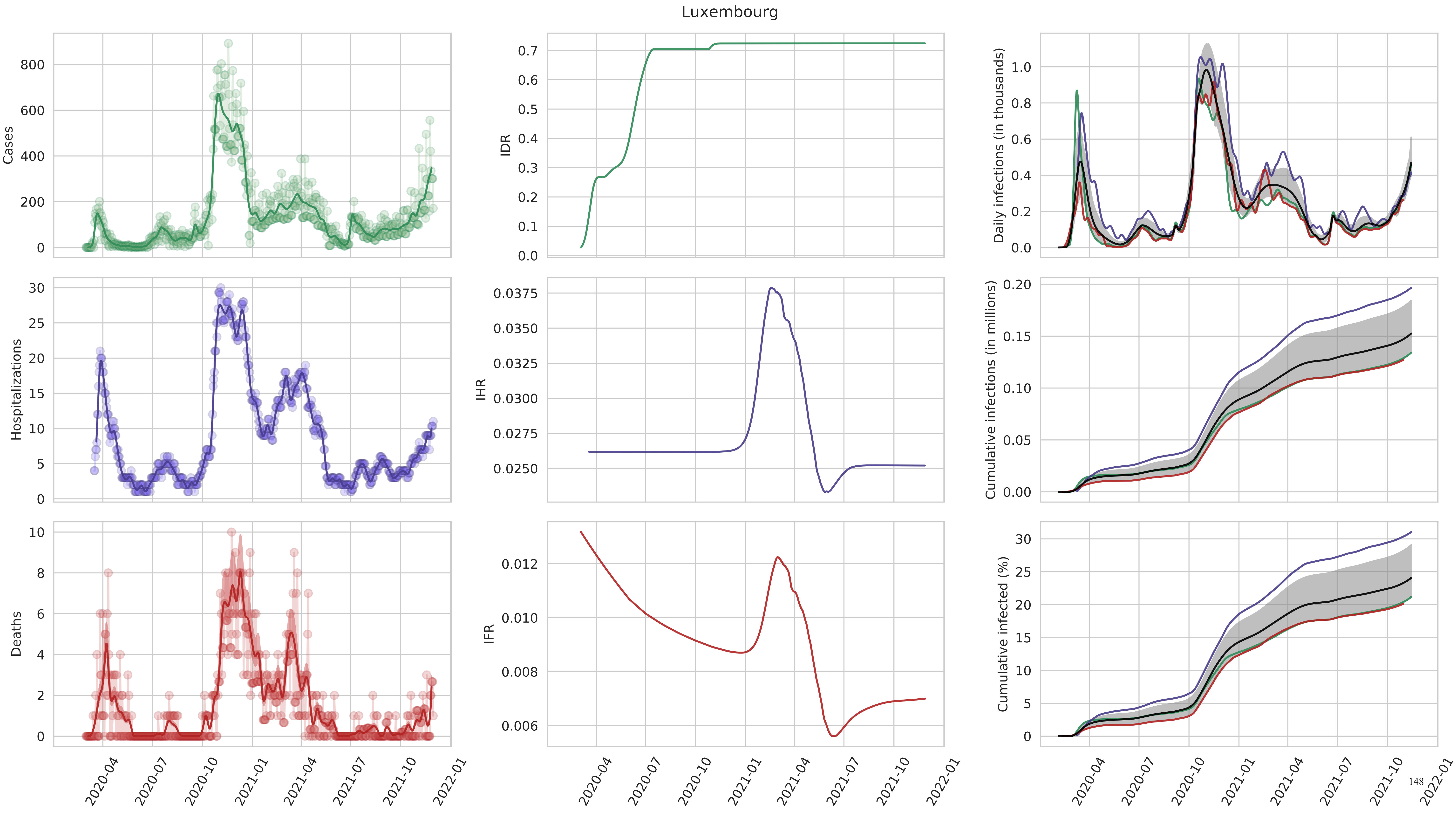
Luxembourg
Cases
IDR
Daily infections (in thousands)
Hospitalizations
IHR
Cumulative infections (in millions)
Deaths
IFR
Cumulative infected (%)
2020-04
2020-07
2020-10
2021-01
2021-04
2021-07
2021-10
2022-01

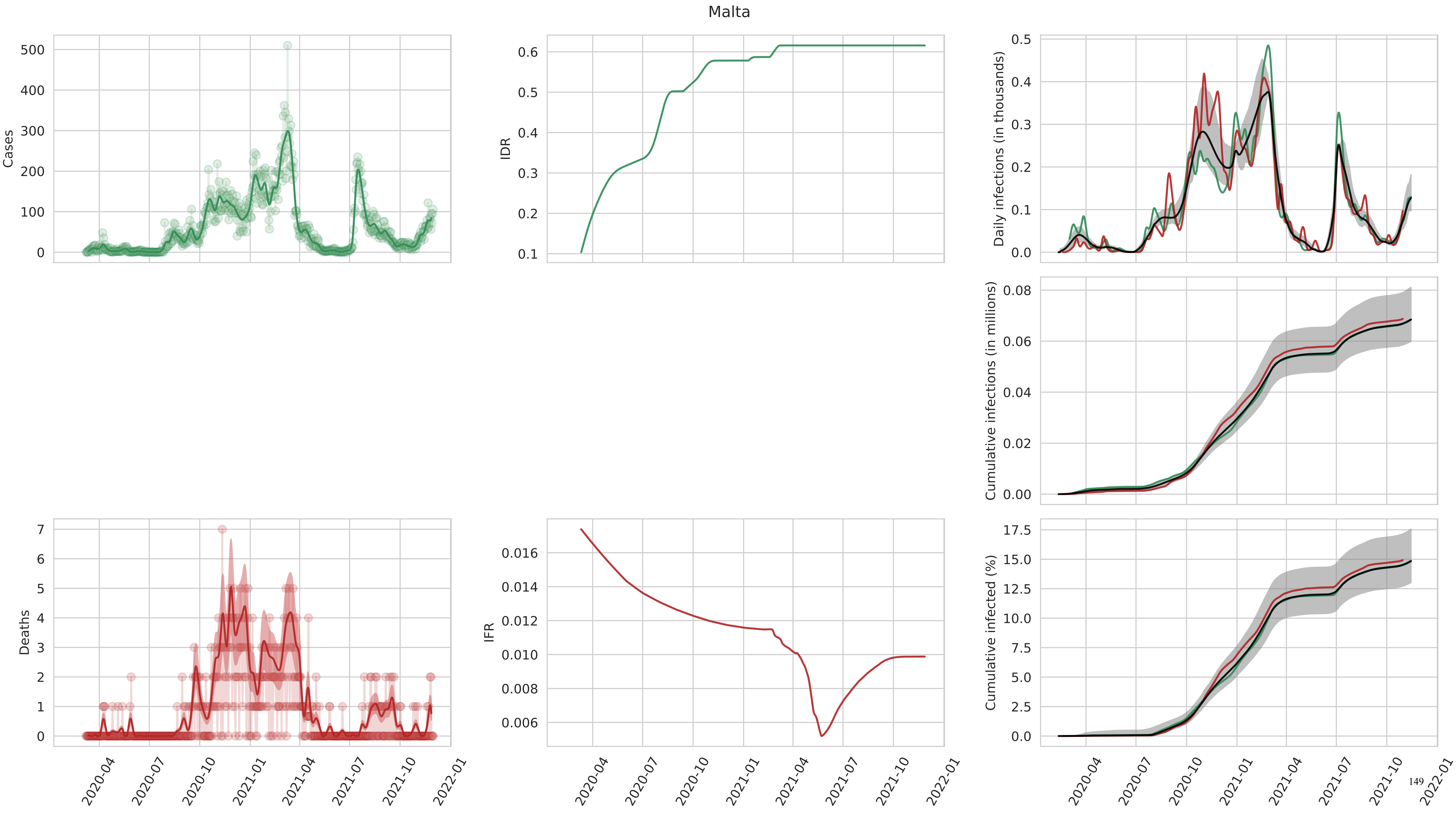

Malta
Cases
IDR
Daily infections (in thousands)
Cumulative infections (in millions)
Deaths
IFR
Cumulative infected (%)
2020-04
2020-07
2020-10
2021-01
2021-04
2021-07
2021-10
2022-01

Monaco

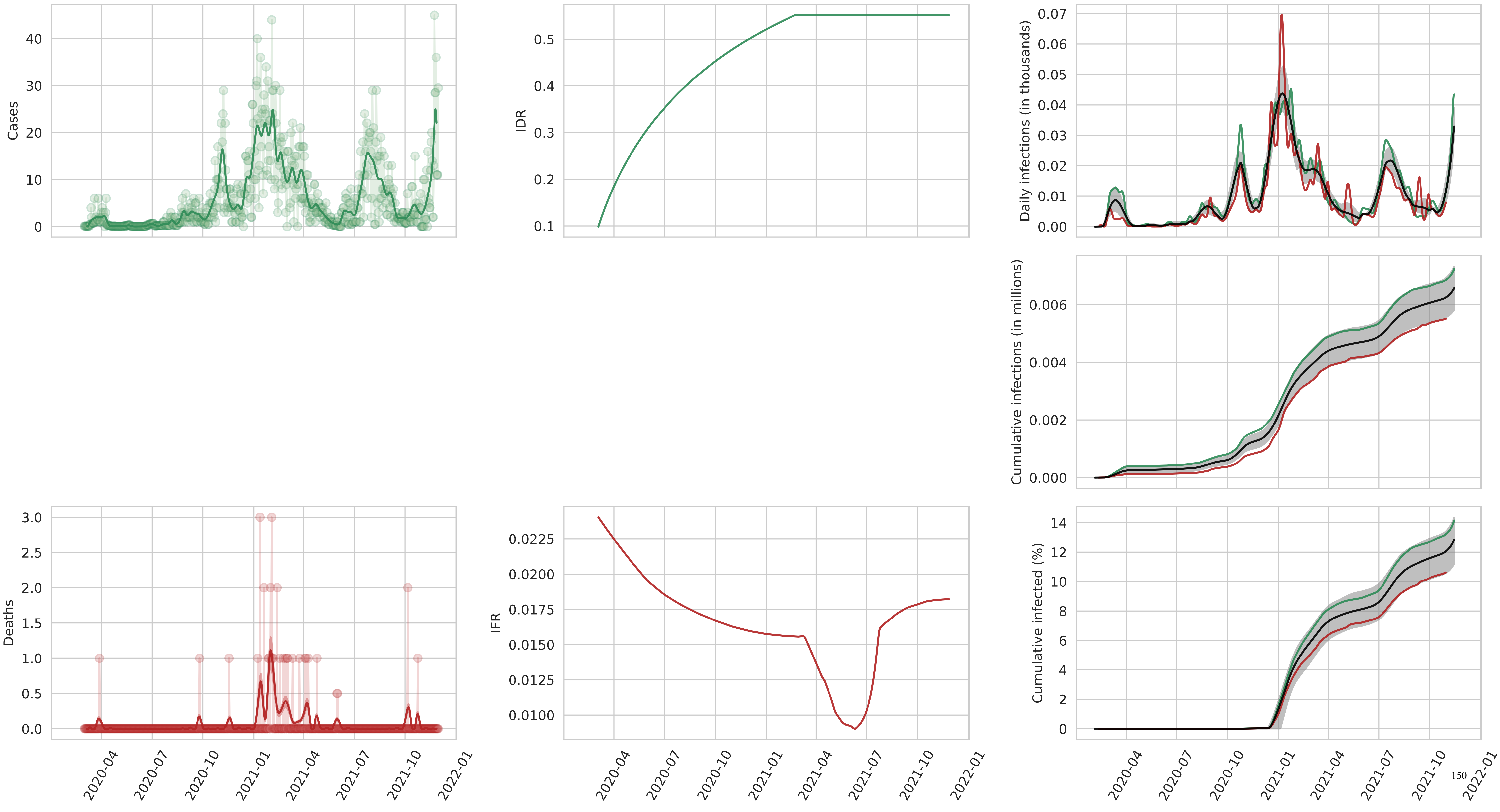

Cases
Deaths
IDR
IFR
Daily infections (in thousands)
Cumulative infections (in millions)
Cumulative infected (%)
2020-04
2020-07
2020-10
2021-01
2021-04
2021-07
2021-10
2022-01

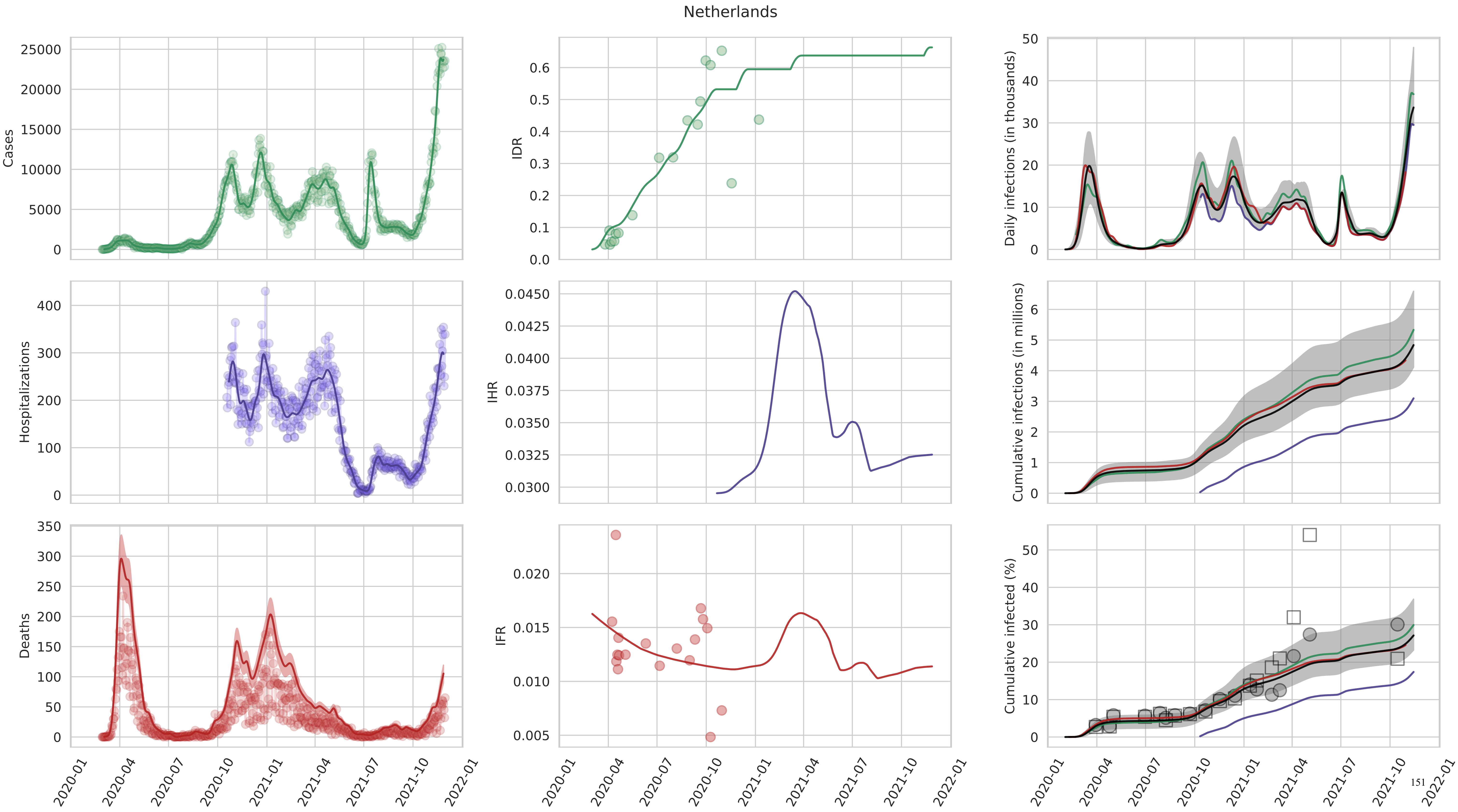
Netherlands
Cases
IDR
Daily infections (in thousands)
Hospitalizations
IHR
Cumulative infections (in millions)
Deaths
IFR
Cumulative infected (%)
2020-01
2020-04
2020-07
2020-10
2021-01
2021-04
2021-07
2021-10
2022-01

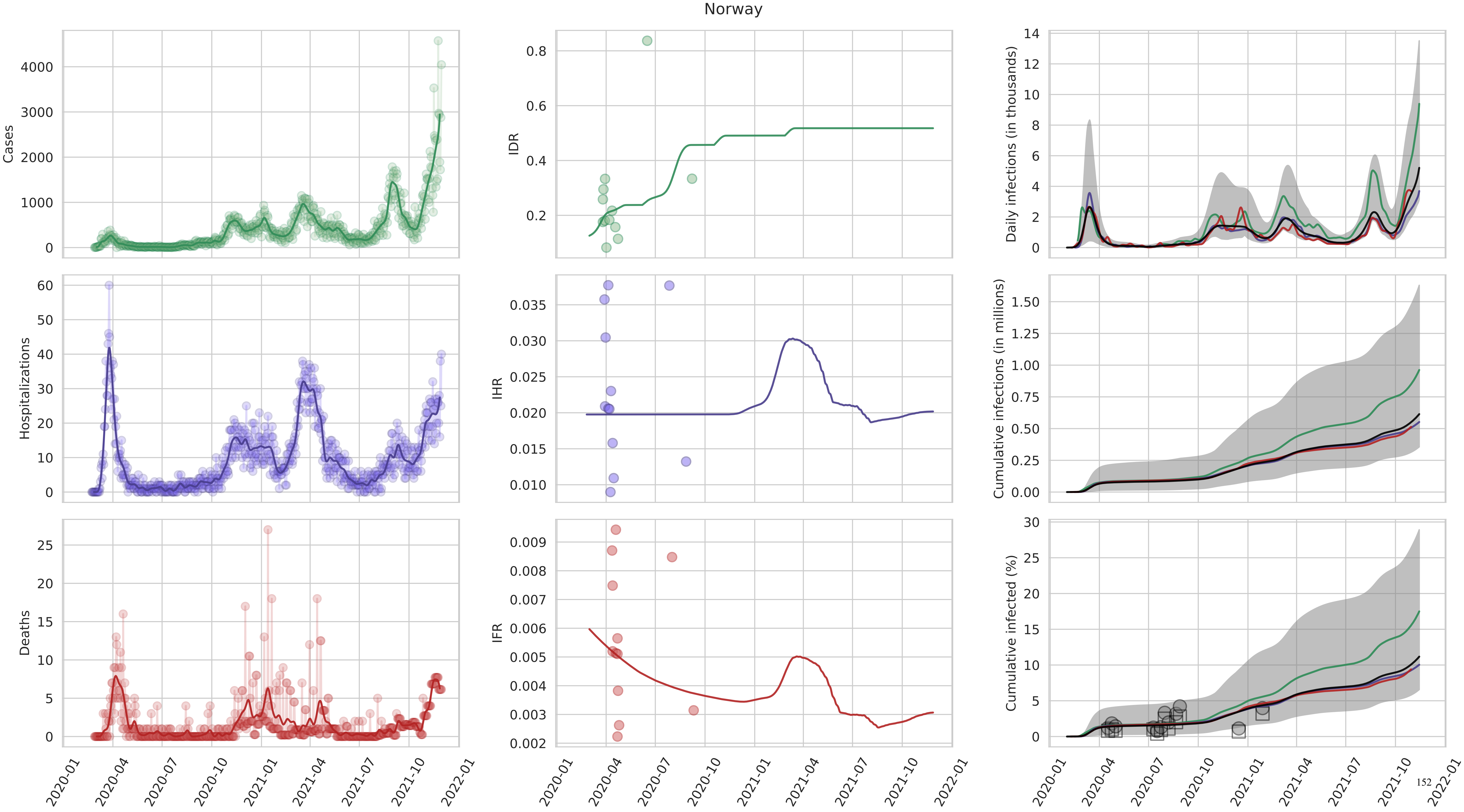
Norway
Cases
Hospitalizations
Deaths
IDR
IHR
IFR
Daily infections (in thousands)
Cumulative infections (in millions)
Cumulative infected (%)
2020-01
2020-04
2020-07
2020-10
2021-01
2021-04
2021-07
2021-10
2022-01

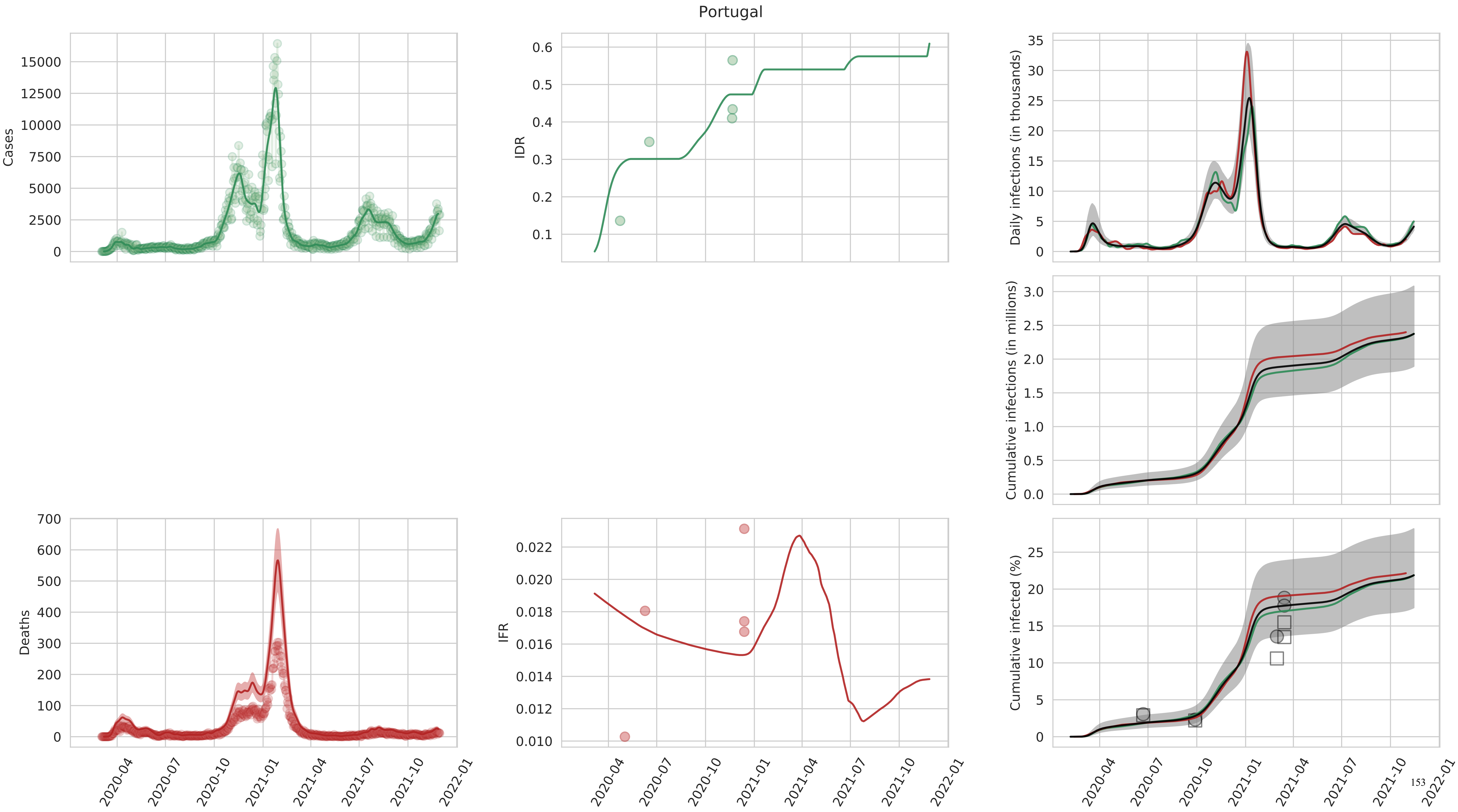
Portugal
Cases
IDR
Daily infections (in thousands)
Cumulative infections (in millions)
Deaths
IFR
Cumulative infected (%)
2020-04
2020-07
2020-10
2021-01
2021-04
2021-07
2021-10
2022-01

San Marino

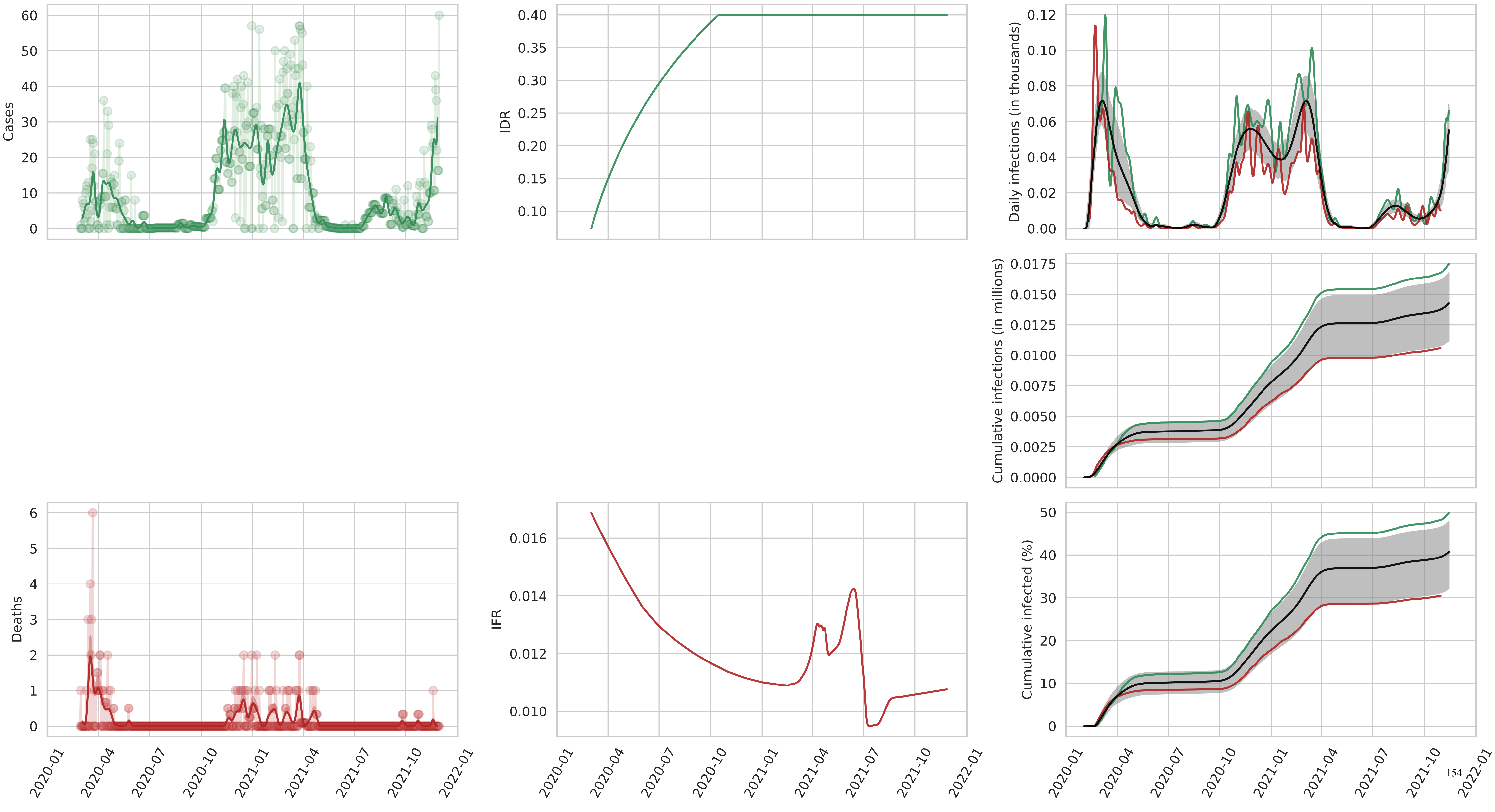

Cases
IDR
Daily infections (in thousands)
Cumulative infections (in millions)
Deaths
IFR
Cumulative infected (%)

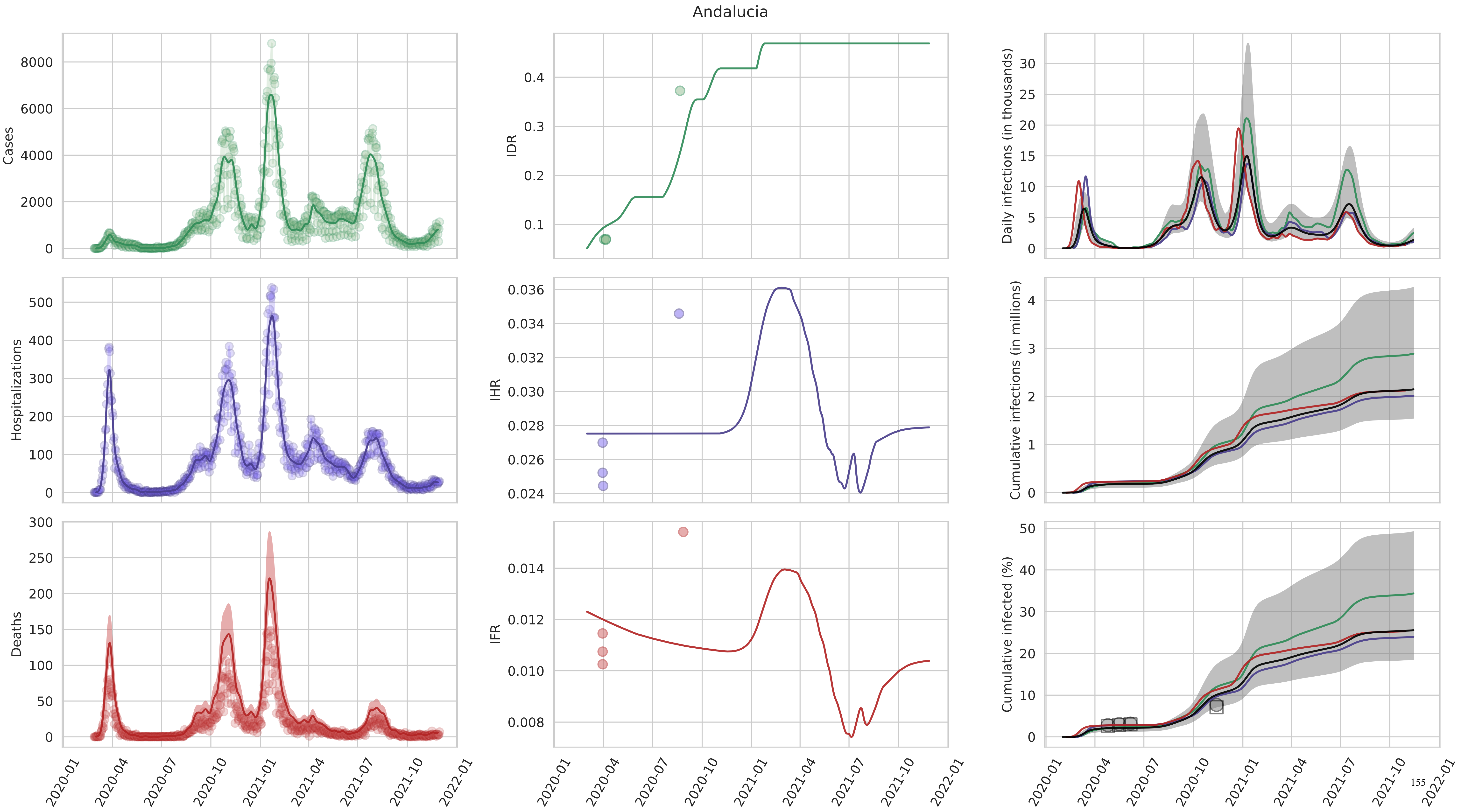

Andalucia
Cases
IDR
Daily infections (in thousands)
Hospitalizations
IHR
Cumulative infections (in millions)
Deaths
IFR
Cumulative infected (%)
2020-01
2020-04
2020-07
2020-10
2021-01
2021-04
2021-07
2021-10
2022-01

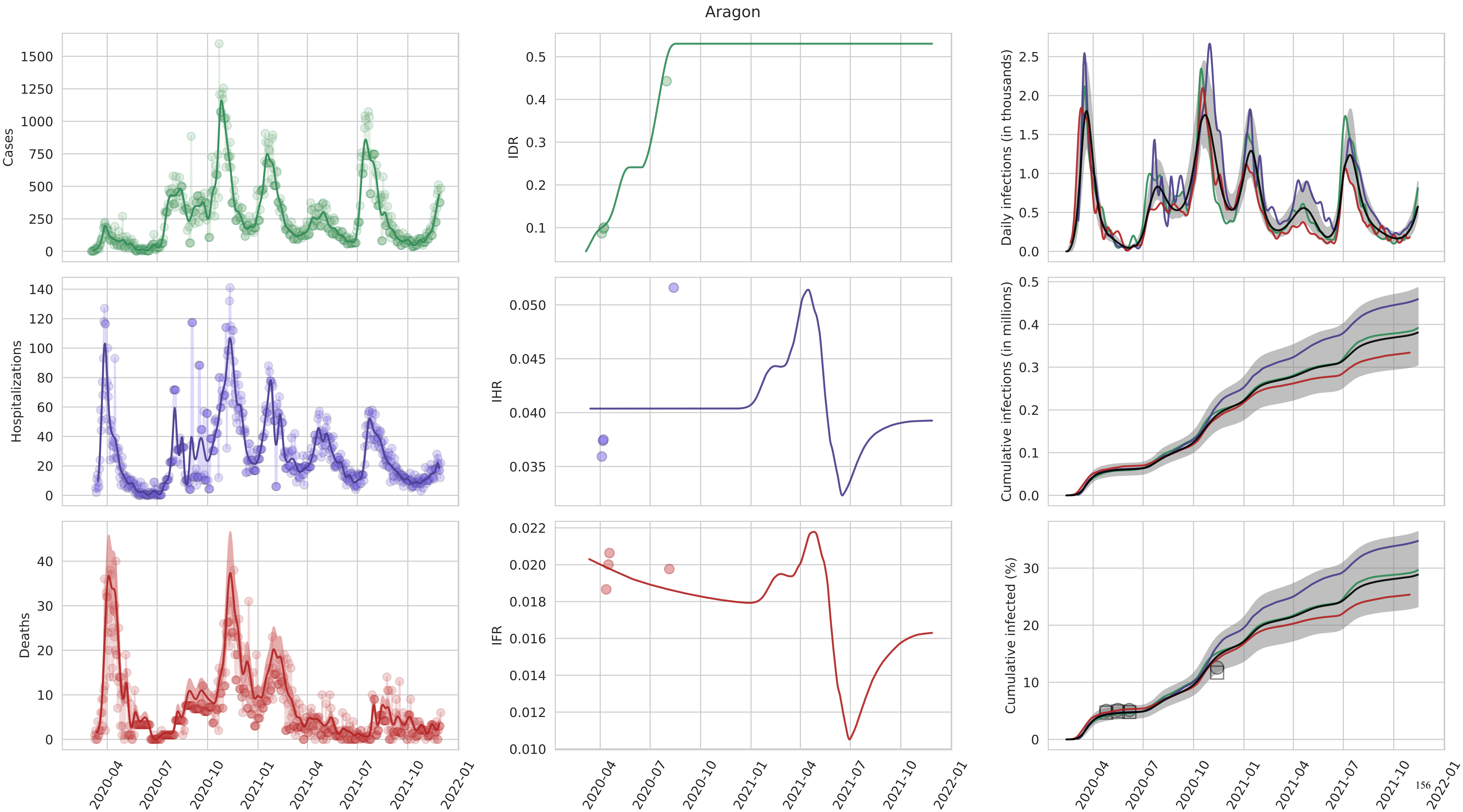

Aragon
Cases
Hospitalizations
Deaths
IDR
IHR
IFR
Daily infections (in thousands)
Cumulative infections (in millions)
Cumulative infected (%)
2020-04
2020-07
2020-10
2021-01
2021-04
2021-07
2021-10
2022-01

Asturias

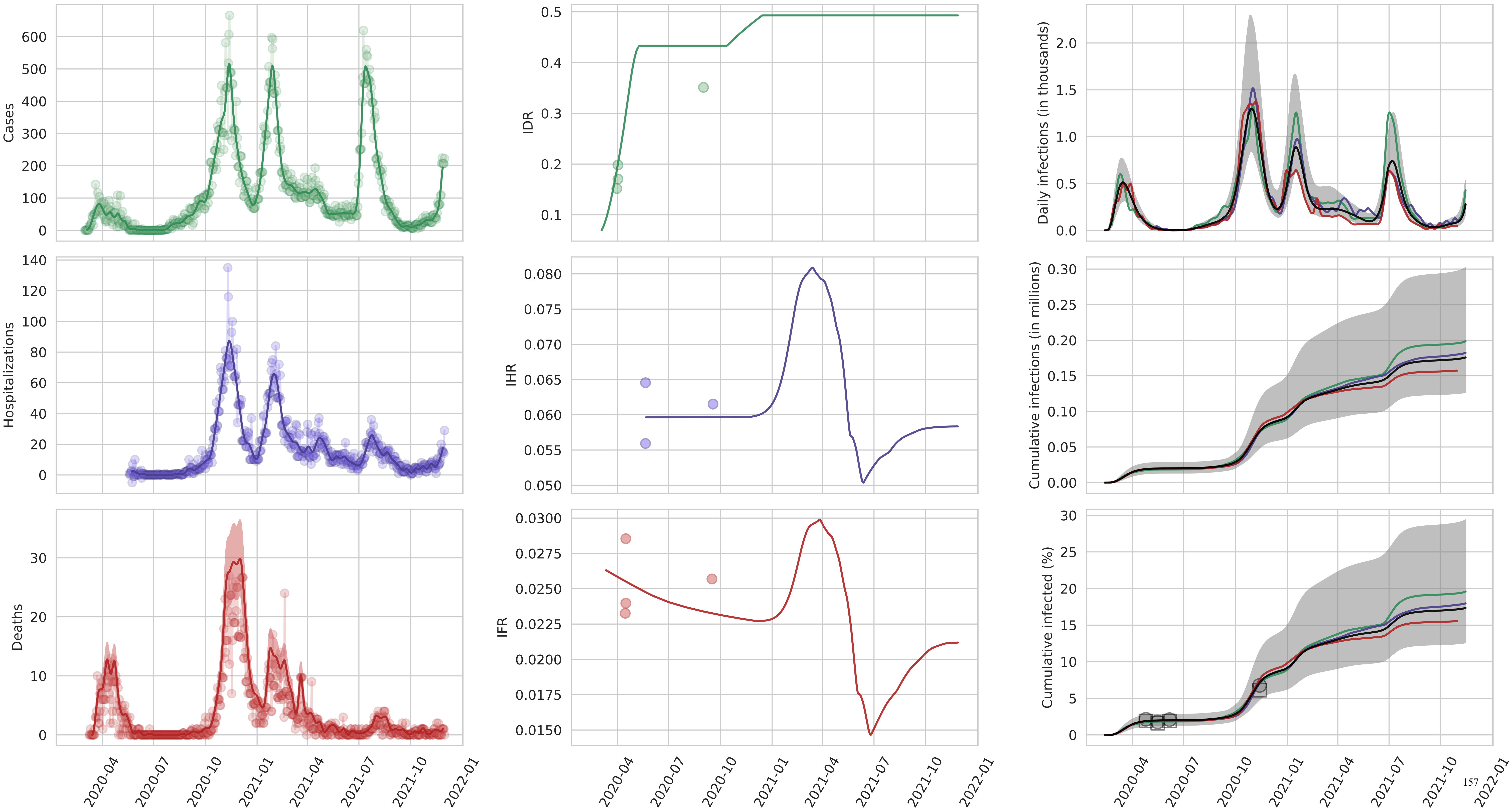

Cases
Hospitalizations
Deaths
IDR
IHR
IFR
Daily infections (in thousands)
Cumulative infections (in millions)
Cumulative infected (%)
2020-04
2020-07
2020-10
2021-01
2021-04
2021-07
2021-10
2022-01

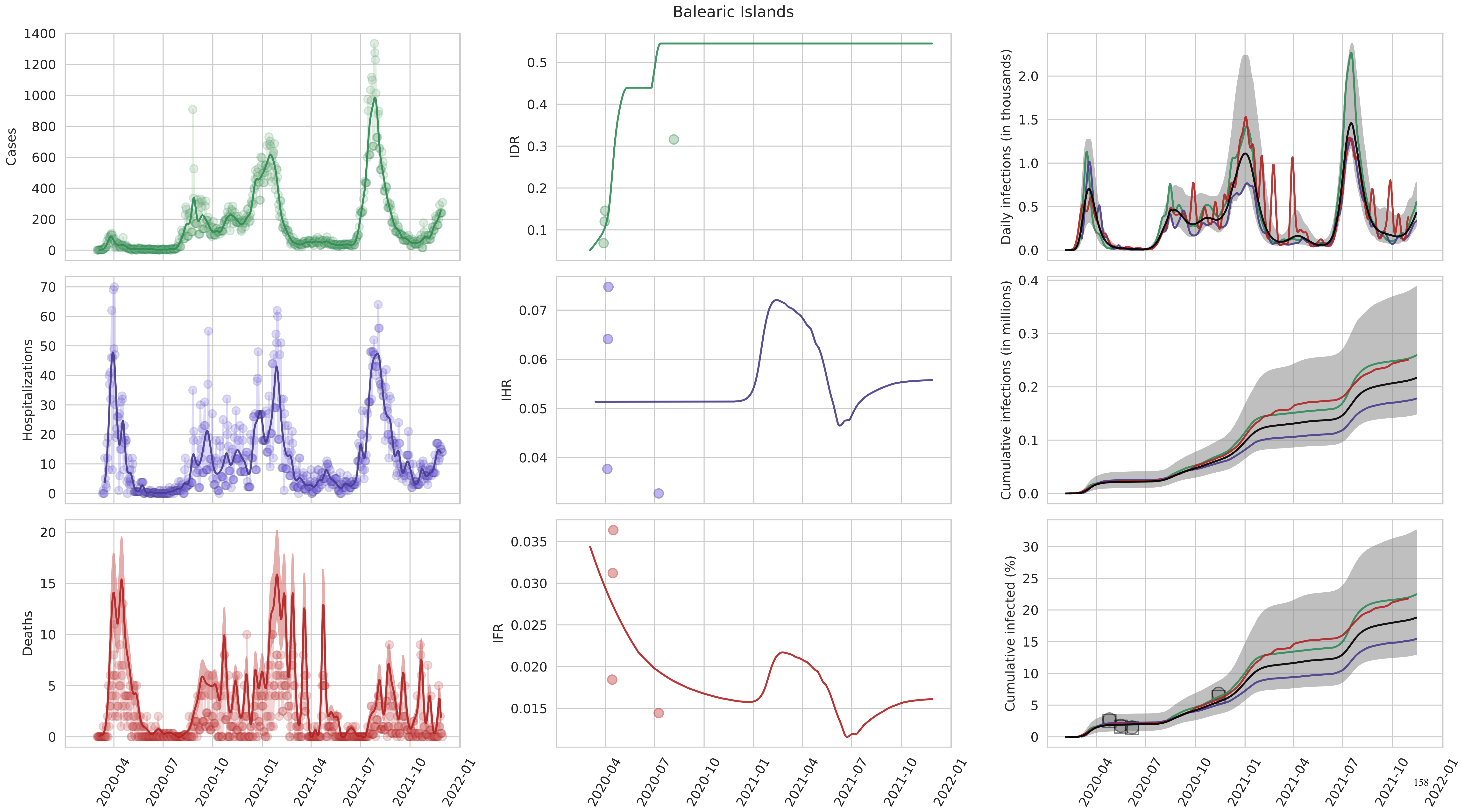
Balearic Islands
Cases
Hospitalizations
Deaths
IDR
IHR
IFR
Daily infections (in thousands)
Cumulative infections (in millions)
Cumulative infected (%)
2020-04
2020-07
2020-10
2021-01
2021-04
2021-07
2021-10
2022-01

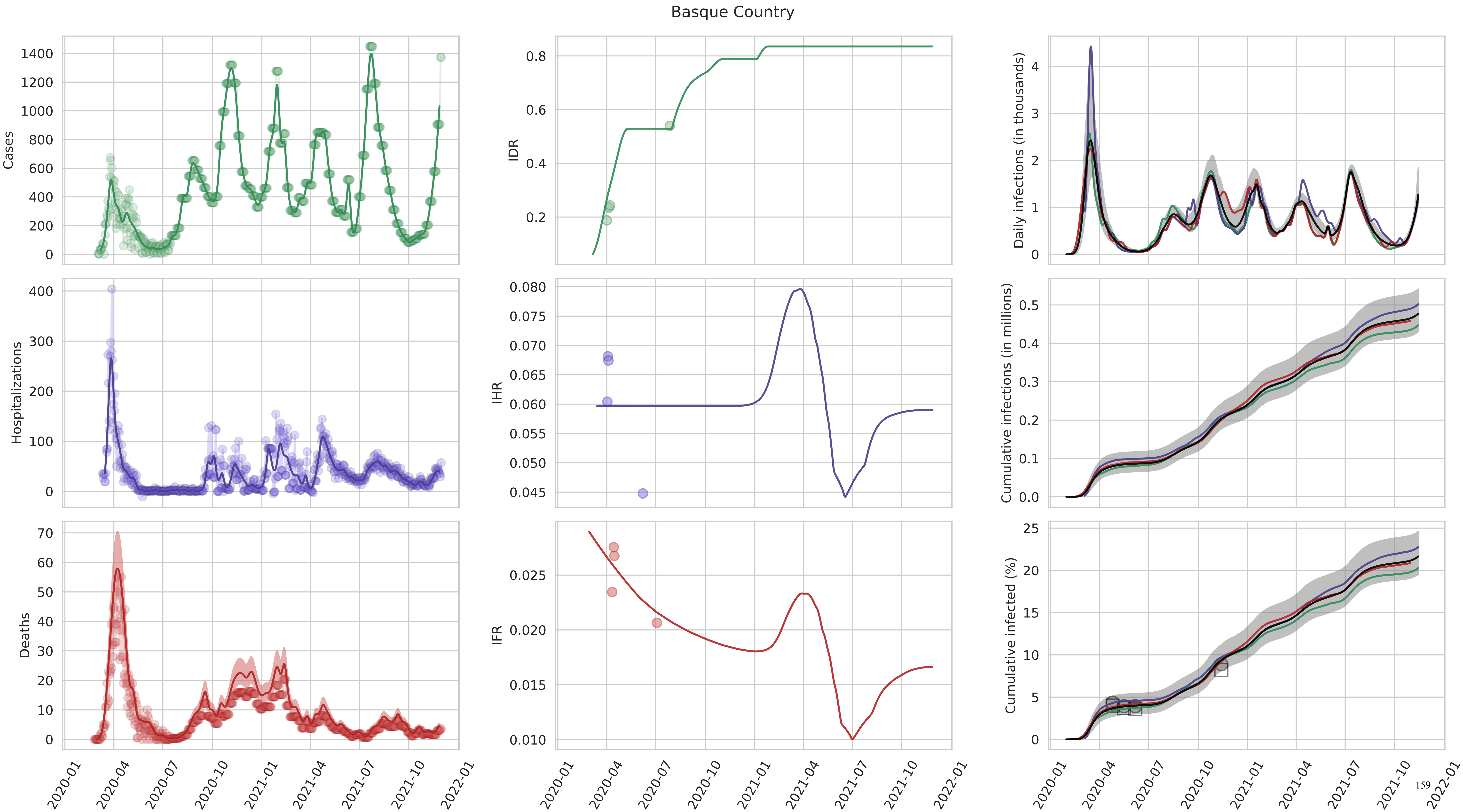

Basque Country
Cases
IDR
Daily infections (in thousands)
Hospitalizations
IHR
Cumulative infections (in millions)
Deaths
IFR
Cumulative infected (%)

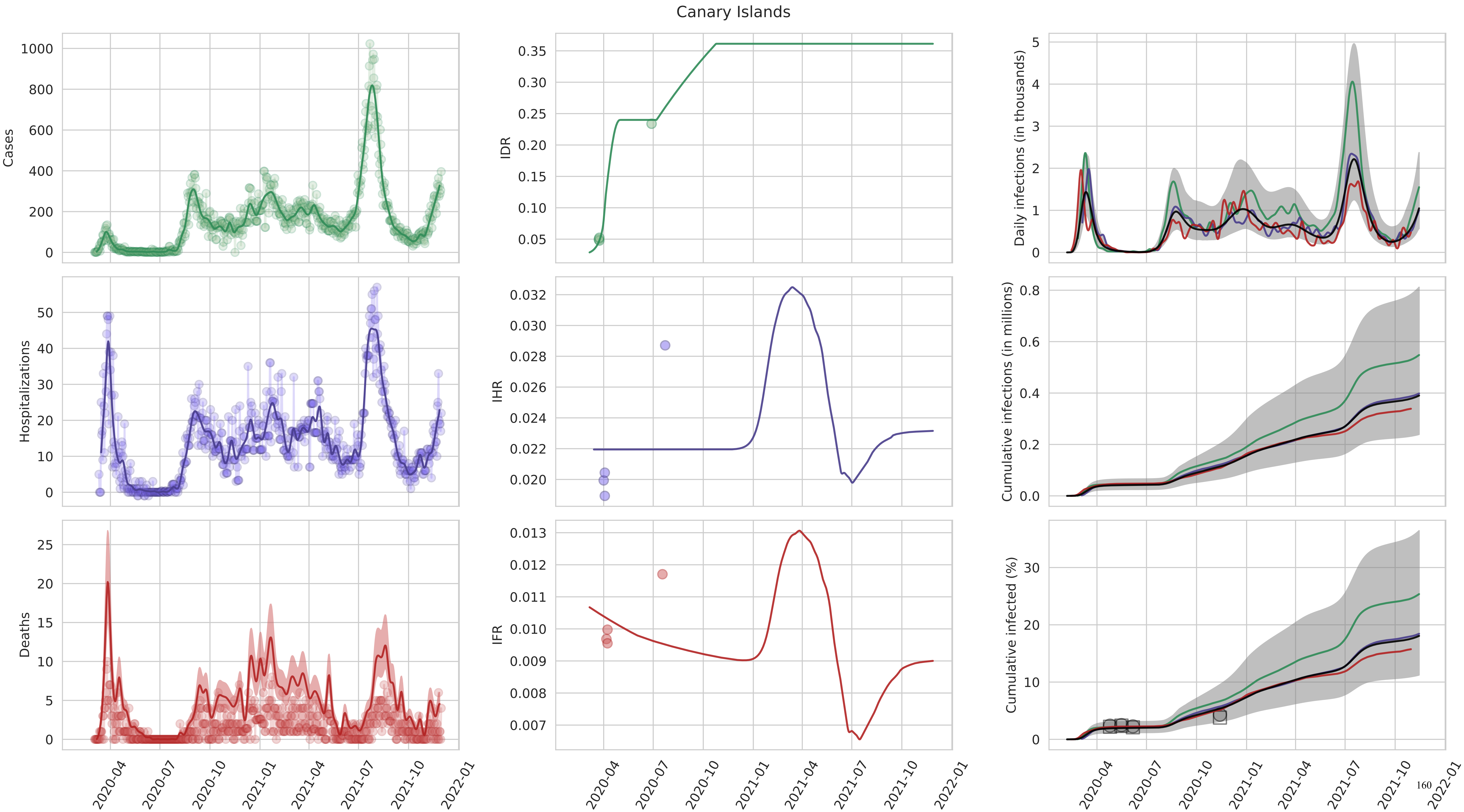

Canary Islands
Cases
IDR
Daily infections (in thousands)
Hospitalizations
IHR
Cumulative infections (in millions)
Deaths
IFR
Cumulative infected (%)
2020-04
2020-07
2020-10
2021-01
2021-04
2021-07
2021-10
2022-01

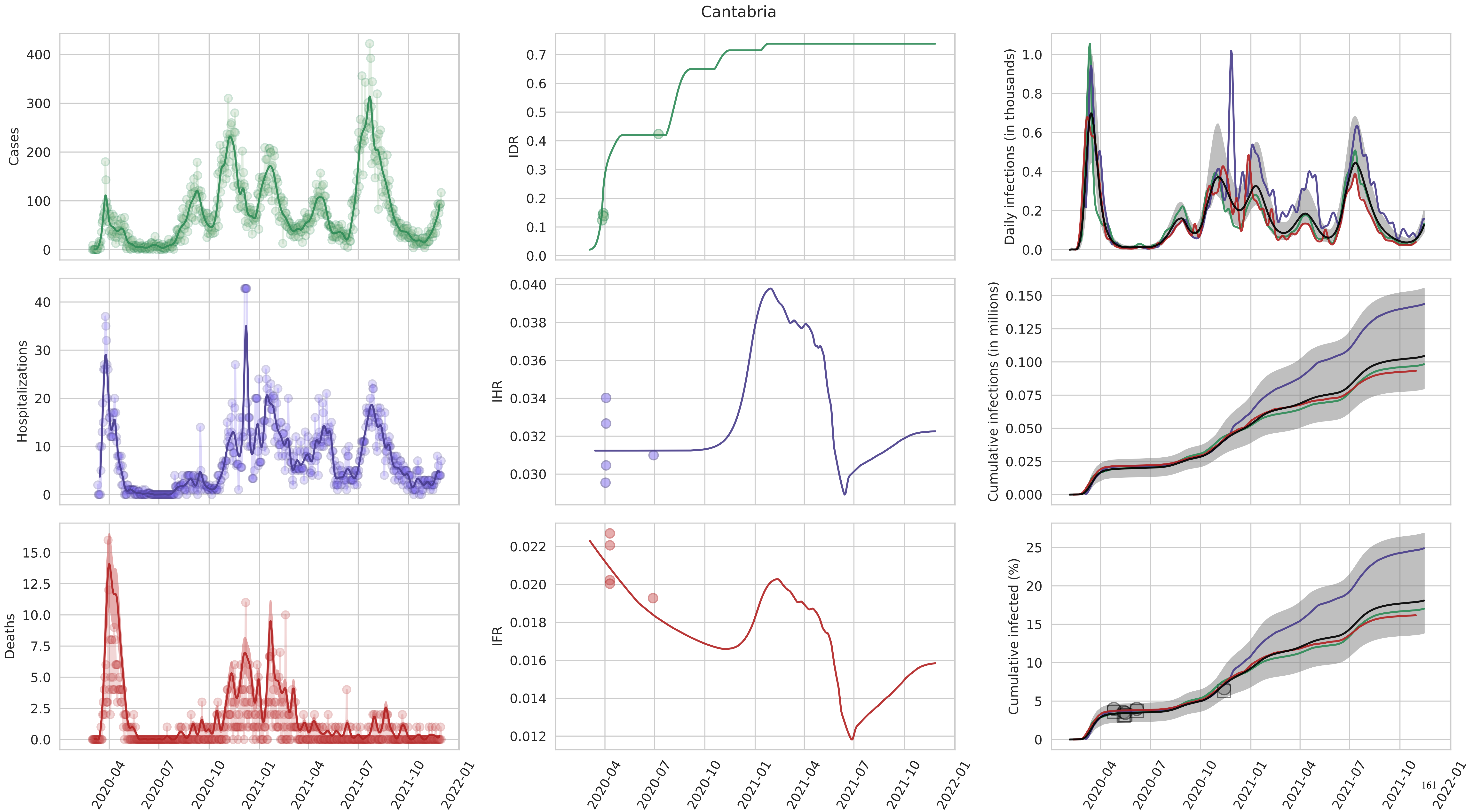
Cantabria
Cases
IDR
Daily infections (in thousands)
Hospitalizations
IHR
Cumulative infections (in millions)
Deaths
IFR
Cumulative infected (%)
2020-04
2020-07
2020-10
2021-01
2021-04
2021-07
2021-10
2022-01

Castile and León

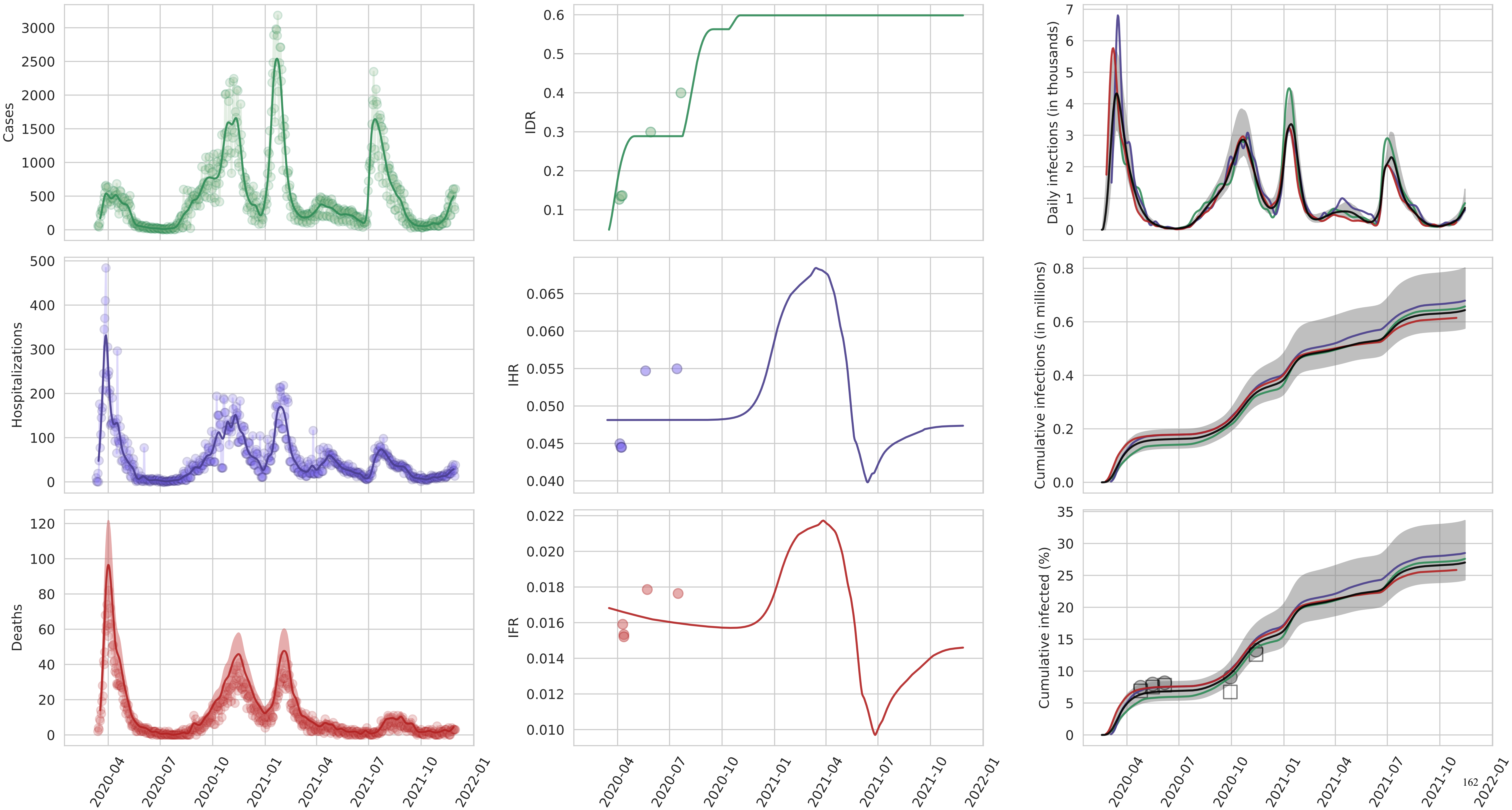

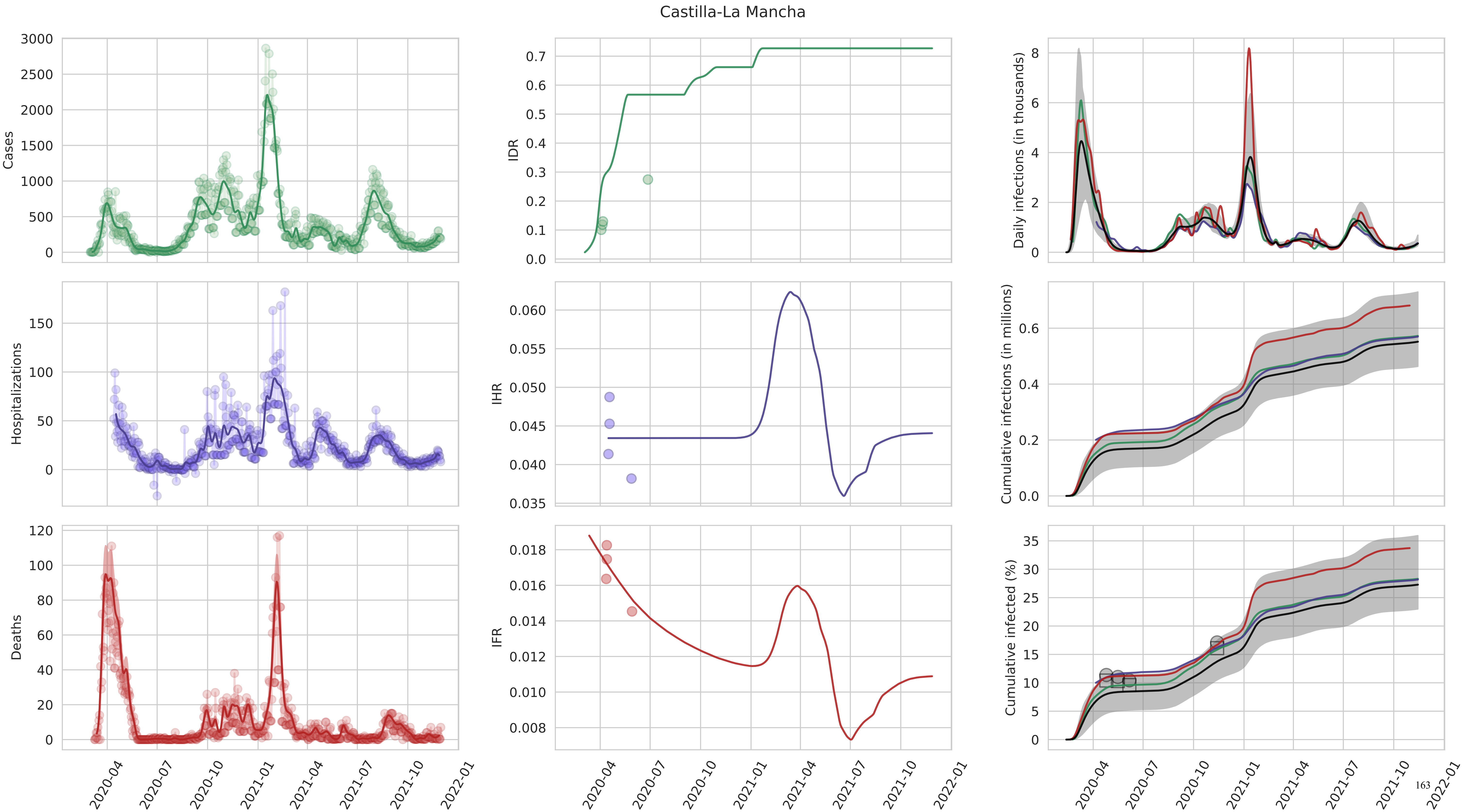
Castilla-La Mancha
Cases
Hospitalizations
Deaths
IDR
IHR
IFR
Daily infections (in thousands)
Cumulative infections (in millions)
Cumulative infected (%)
2020-04
2020-07
2020-10
2021-01
2021-04
2021-07
2021-10
2022-01

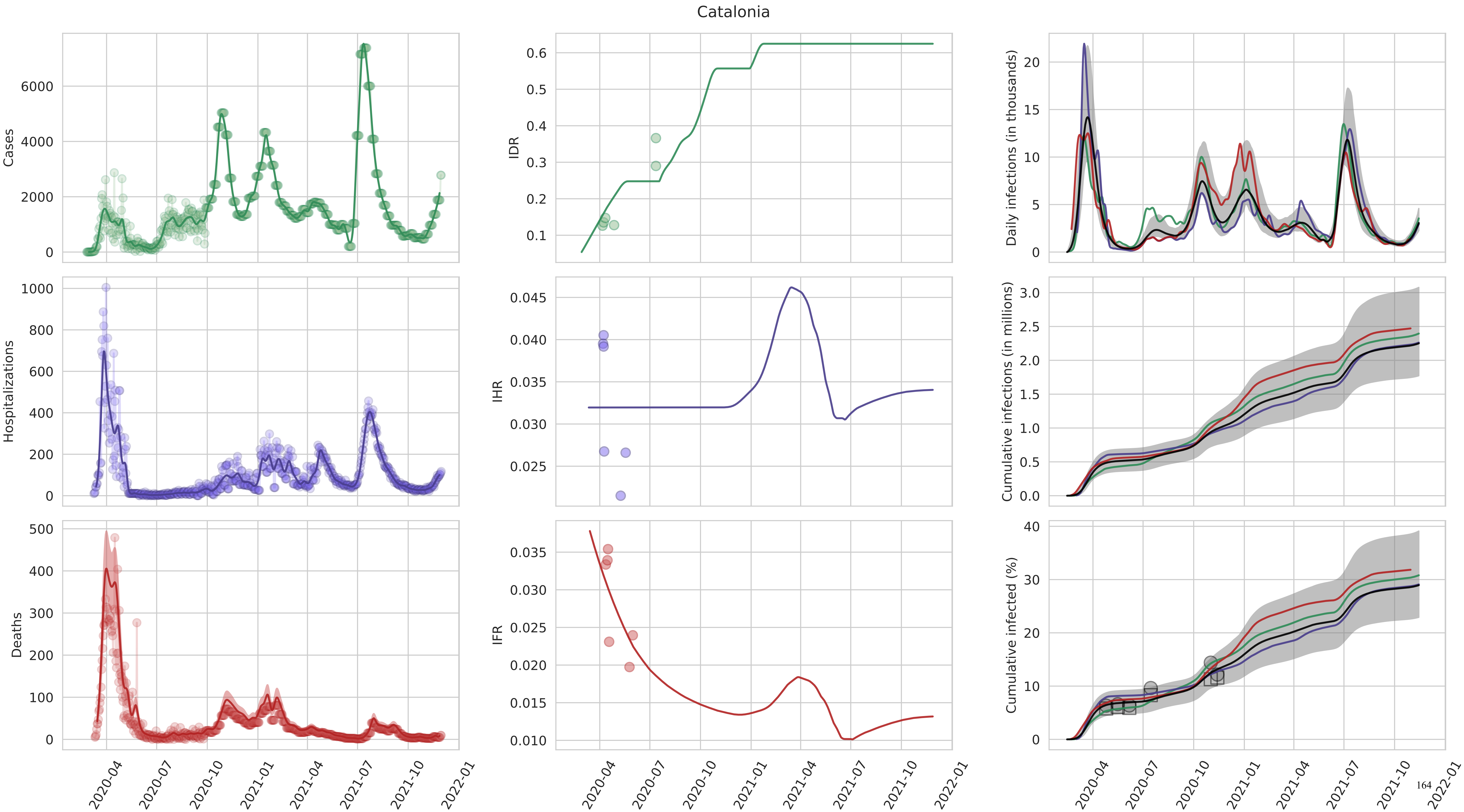
Catalonia
Cases
Hospitalizations
Deaths
IDR
IHR
IFR
Daily infections (in thousands)
Cumulative infections (in millions)
Cumulative infected (%)
2020-04
2020-07
2020-10
2021-01
2021-04
2021-07
2021-10
2022-01

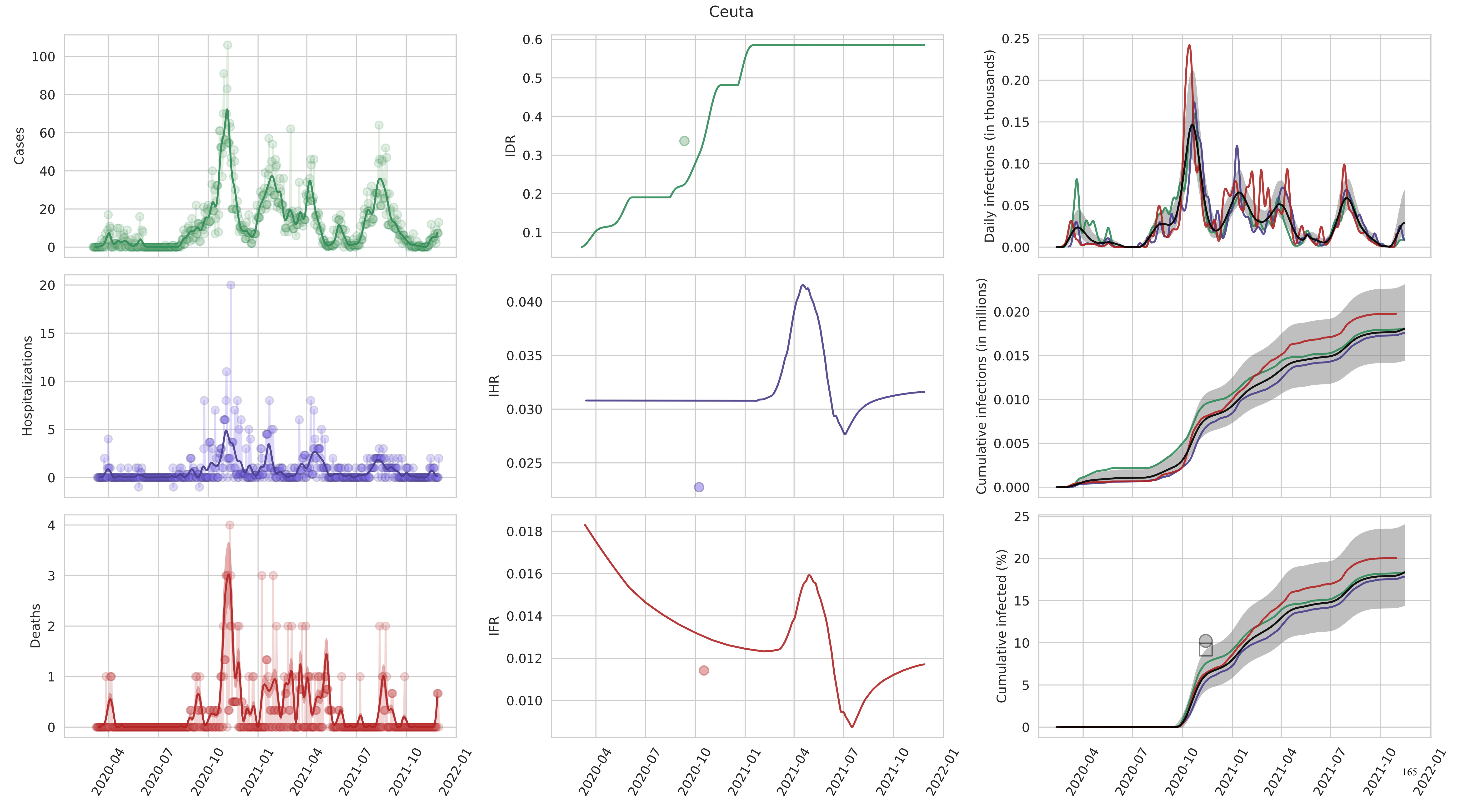
Ceuta
Cases
Hospitalizations
Deaths
IDR
IHR
IFR
Daily infections (in thousands)
Cumulative infections (in millions)
Cumulative infected (%)
2020-04
2020-07
2020-10
2021-01
2021-04
2021-07
2021-10
2022-01

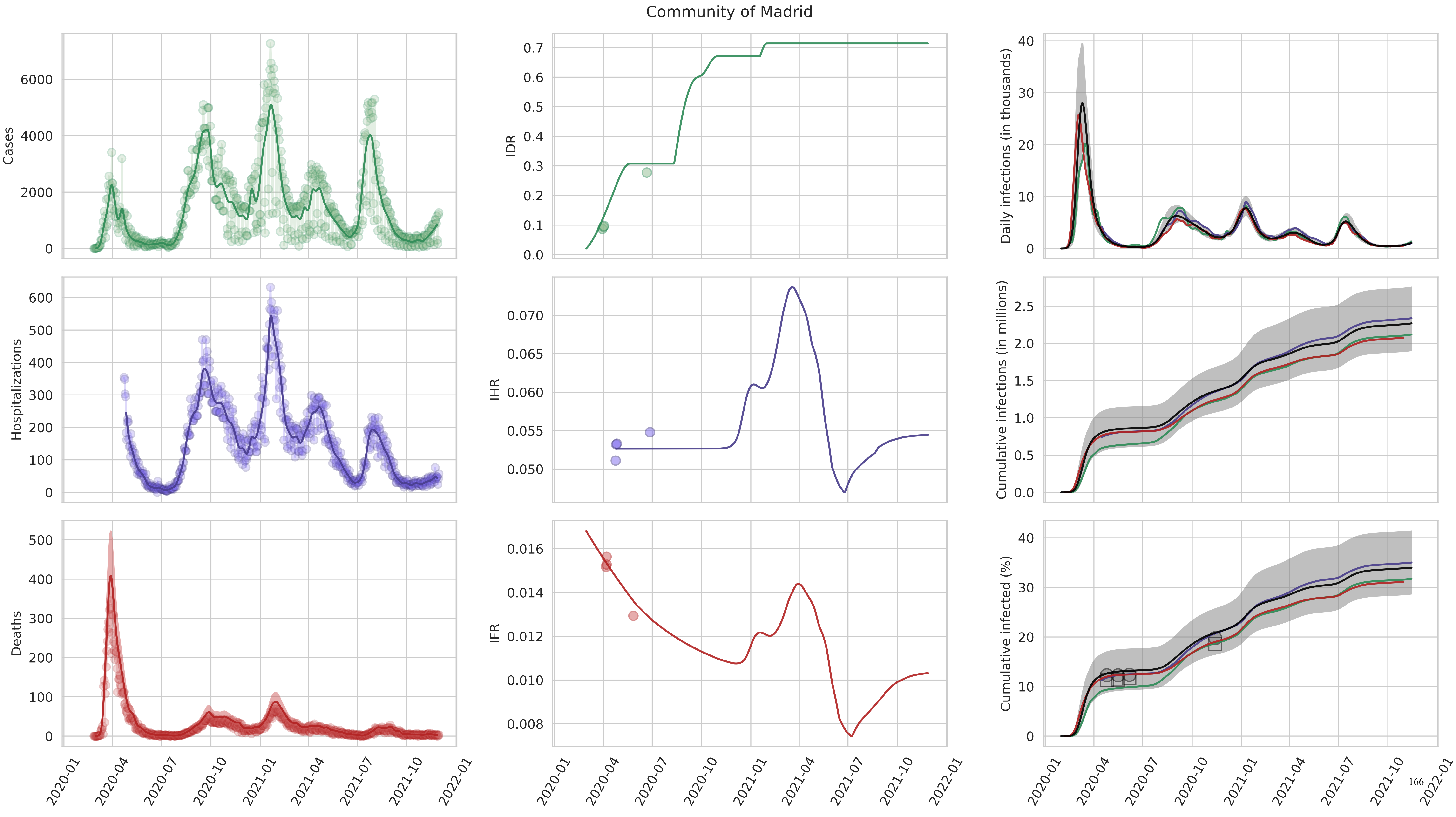
Community of Madrid
Cases
IDR
Daily infections (in thousands)
Hospitalizations
IHR
Cumulative infections (in millions)
Deaths
IFR
Cumulative infected (%)
2020-01
2020-04
2020-07
2020-10
2021-01
2021-04
2021-07
2021-10
2022-01

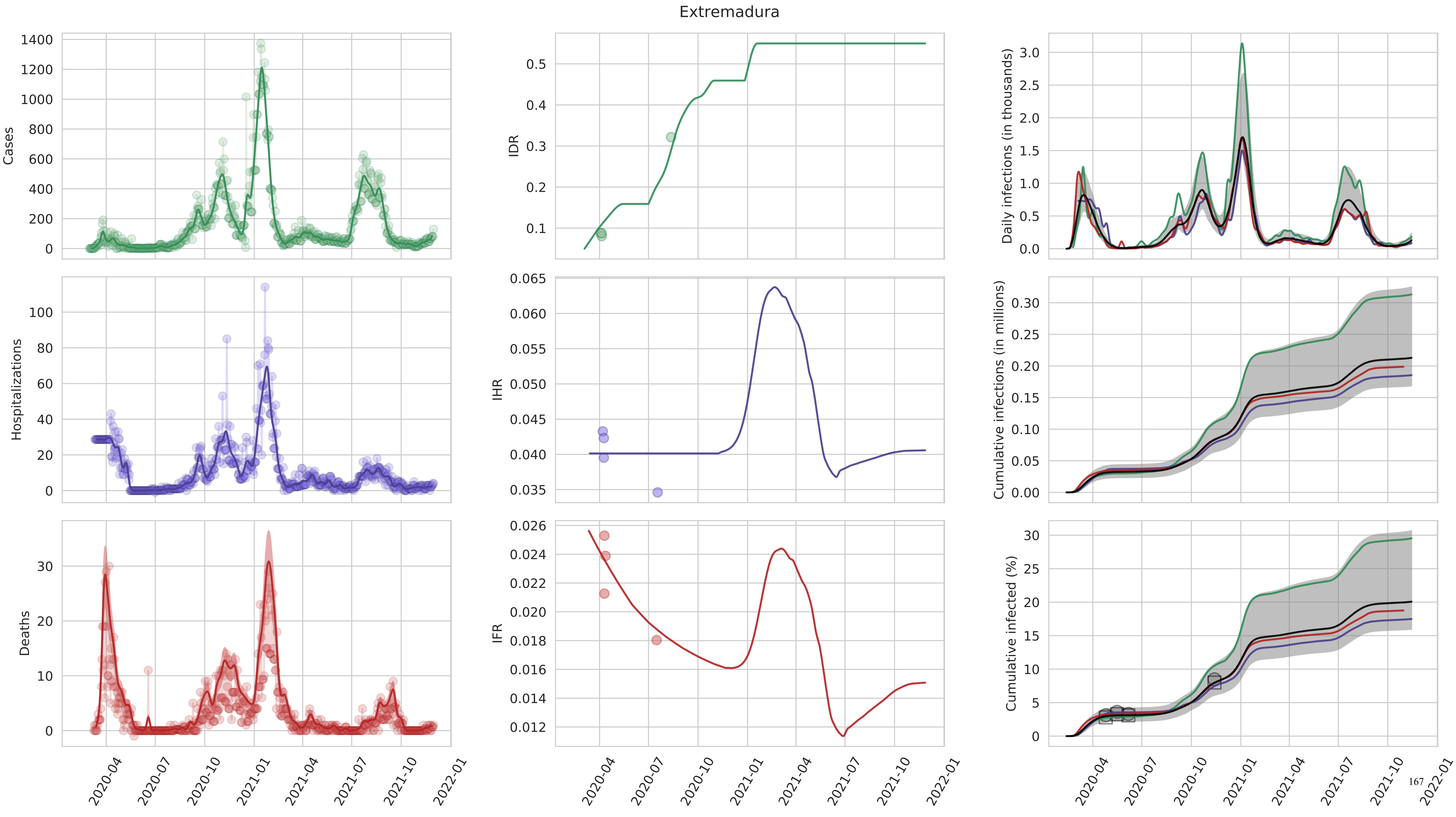
Extremadura
Cases
Hospitalizations
Deaths
IDR
IHR
IFR
Daily infections (in thousands)
Cumulative infections (in millions)
Cumulative infected (%)
2020-04
2020-07
2020-10
2021-01
2021-04
2021-07
2021-10
2022-01

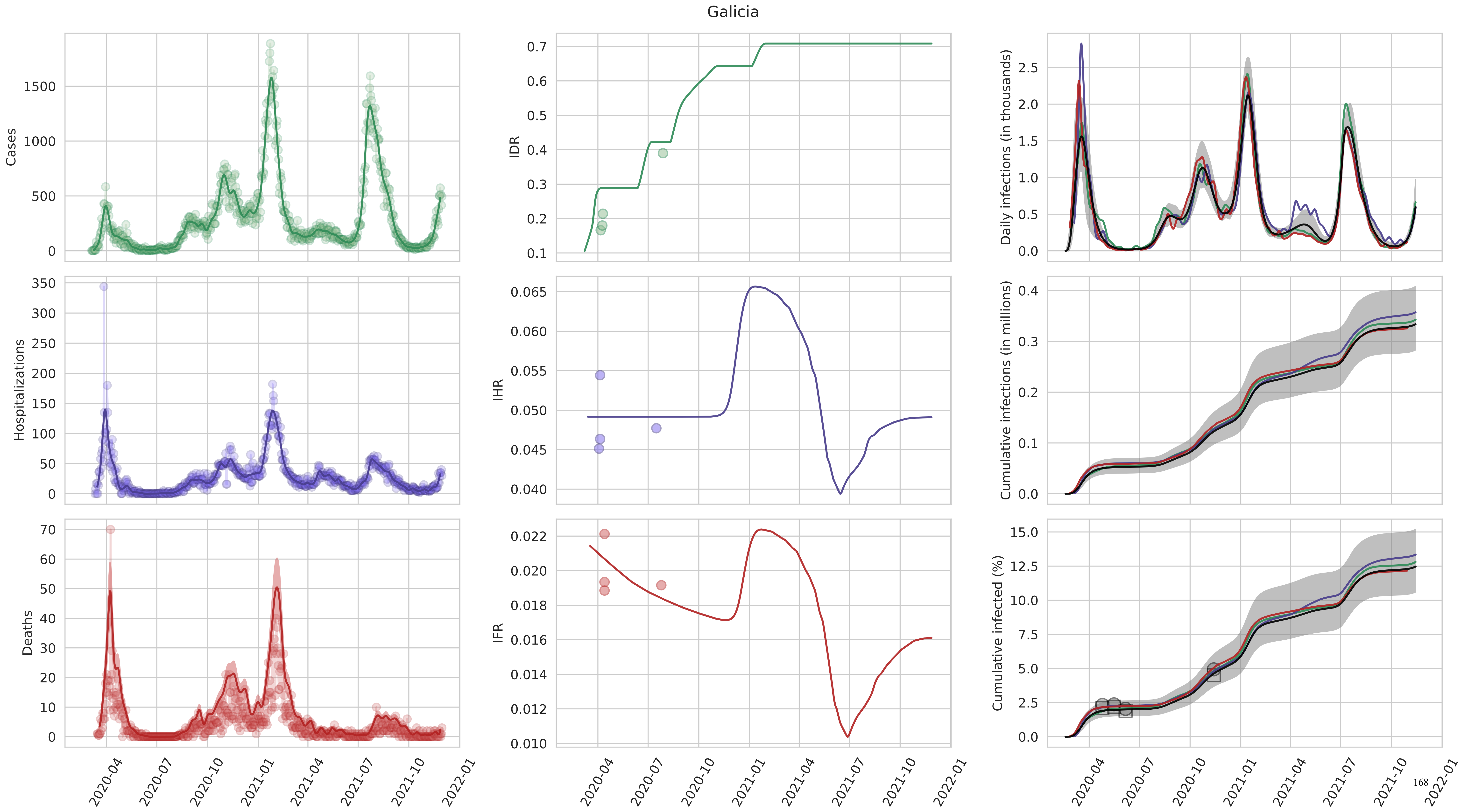
Galicia
Cases
IDR
Daily infections (in thousands)
Hospitalizations
IHR
Cumulative infections (in millions)
Deaths
IFR
Cumulative infected (%)
2020-04
2020-07
2020-10
2021-01
2021-04
2021-07
2021-10
2022-01

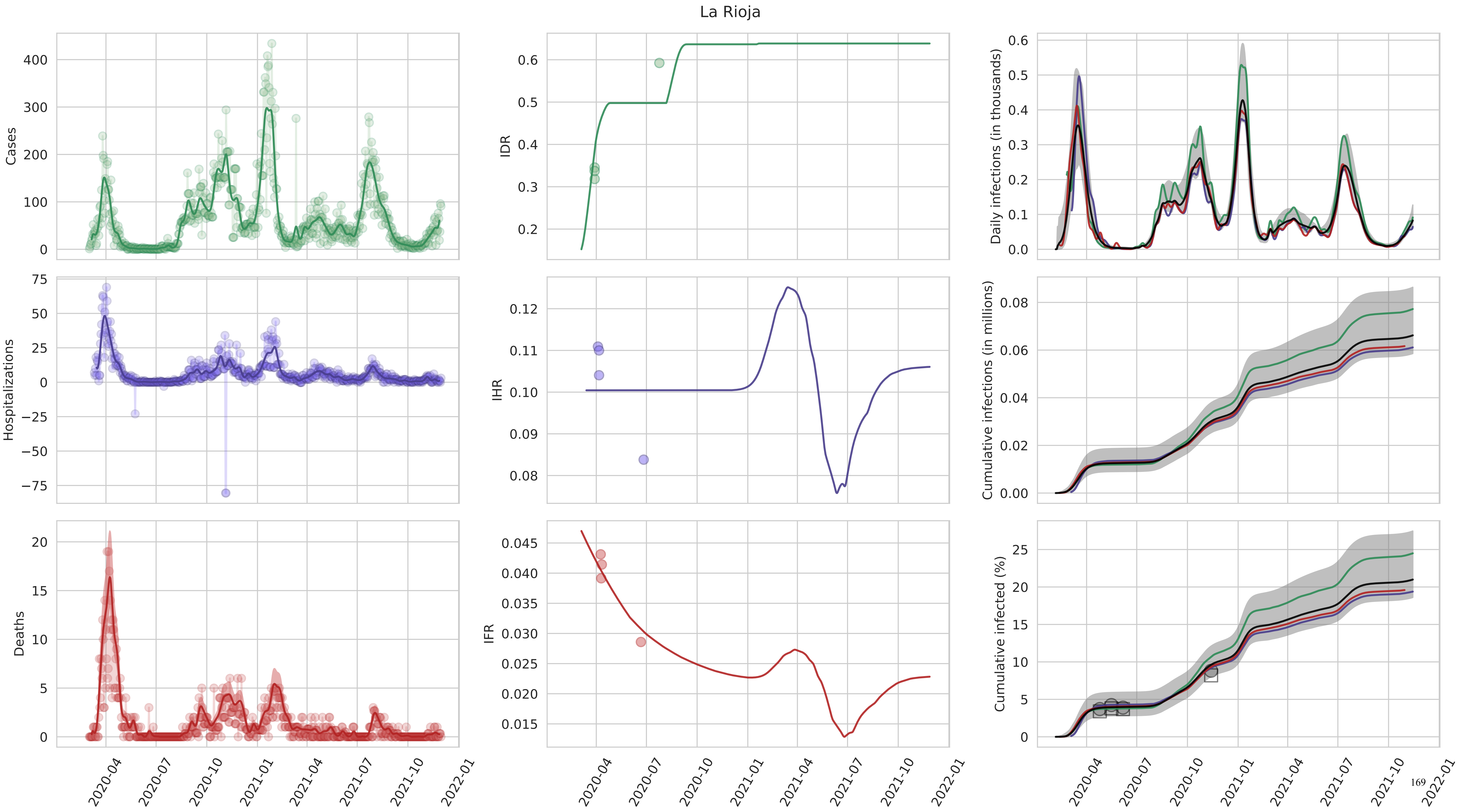

La Rioja
Cases
IDR
Daily infections (in thousands)
Hospitalizations
IHR
Cumulative infections (in millions)
Deaths
IFR
Cumulative infected (%)
2020-04
2020-07
2020-10
2021-01
2021-04
2021-07
2021-10
2022-01

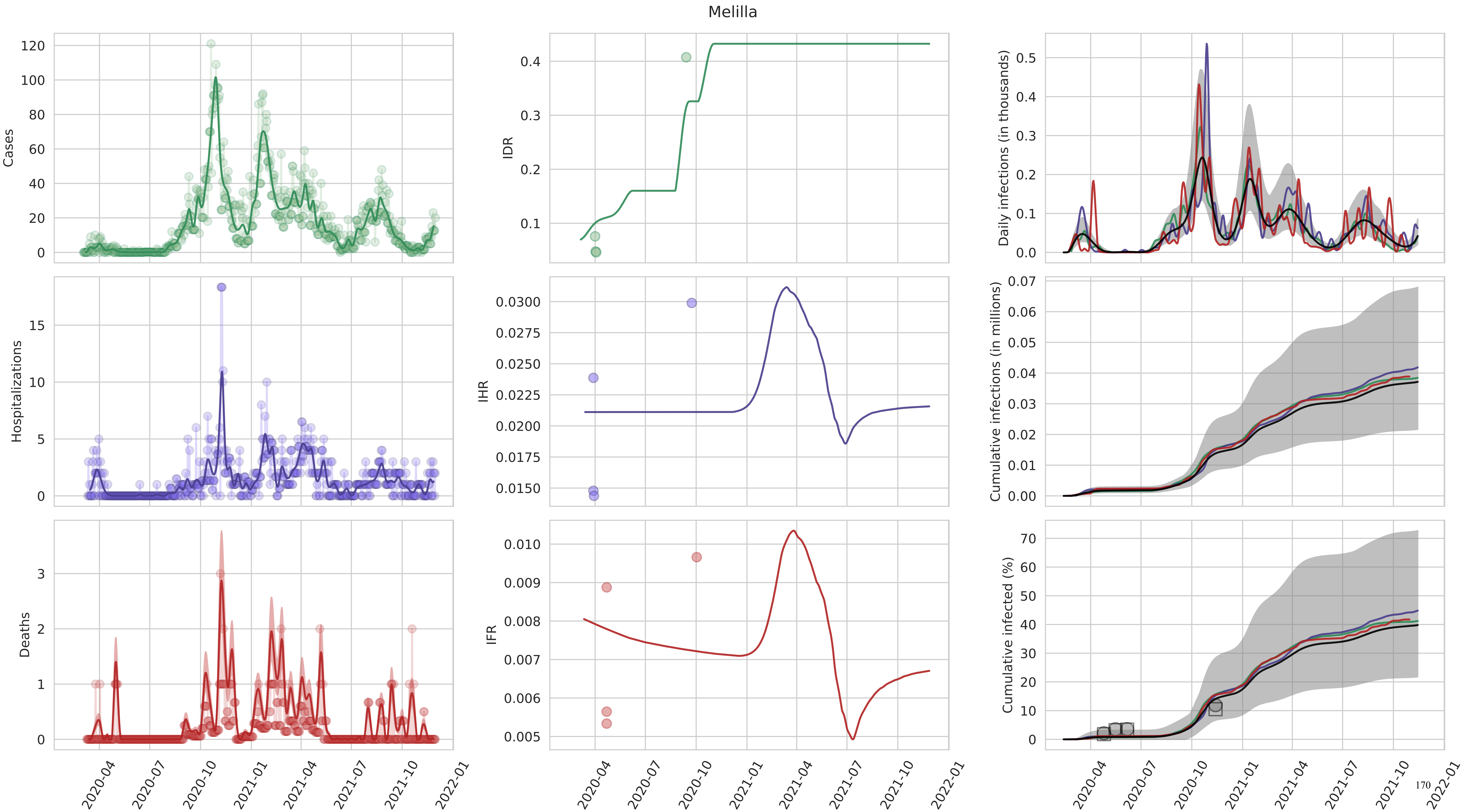
Melilla
Cases
IDR
Daily infections (in thousands)
Hospitalizations
IHR
Cumulative infections (in millions)
Deaths
IFR
Cumulative infected (%)
2020-04
2020-07
2020-10
2021-01
2021-04
2021-07
2021-10
2022-01

Murcia

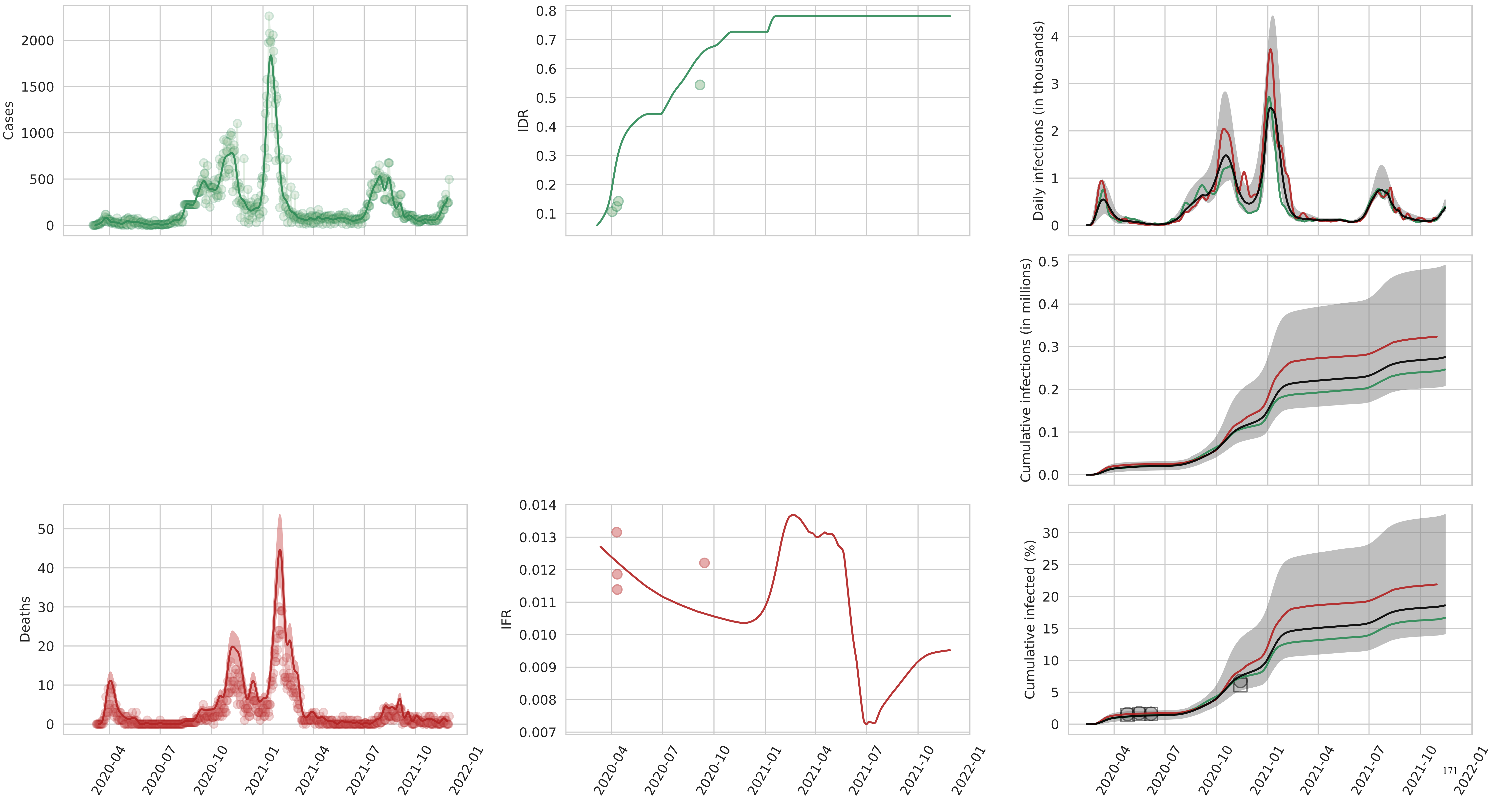

Cases
IDR
Daily infections (in thousands)
Cumulative infections (in millions)
Deaths
IFR
Cumulative infected (%)
2020-04
2020-07
2020-10
2021-01
2021-04
2021-07
2021-10
2022-01

Navarre

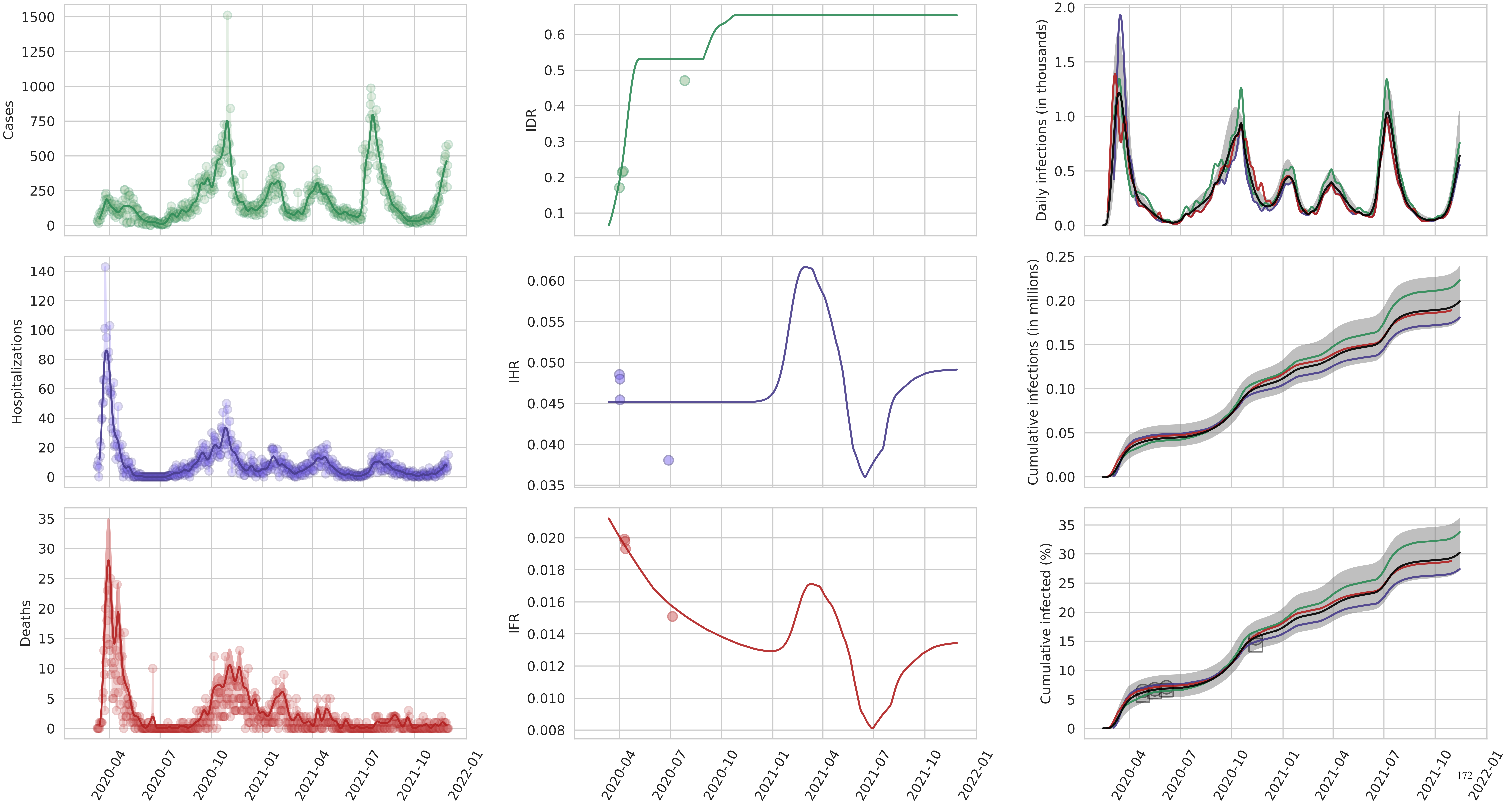
Cases
IDR
Daily infections (in thousands)
Hospitalizations
IHR
Cumulative infections (in millions)
Deaths
IFR
Cumulative infected (%)
2020-04
2020-07
2020-10
2021-01
2021-04
2021-07
2021-10
2022-01

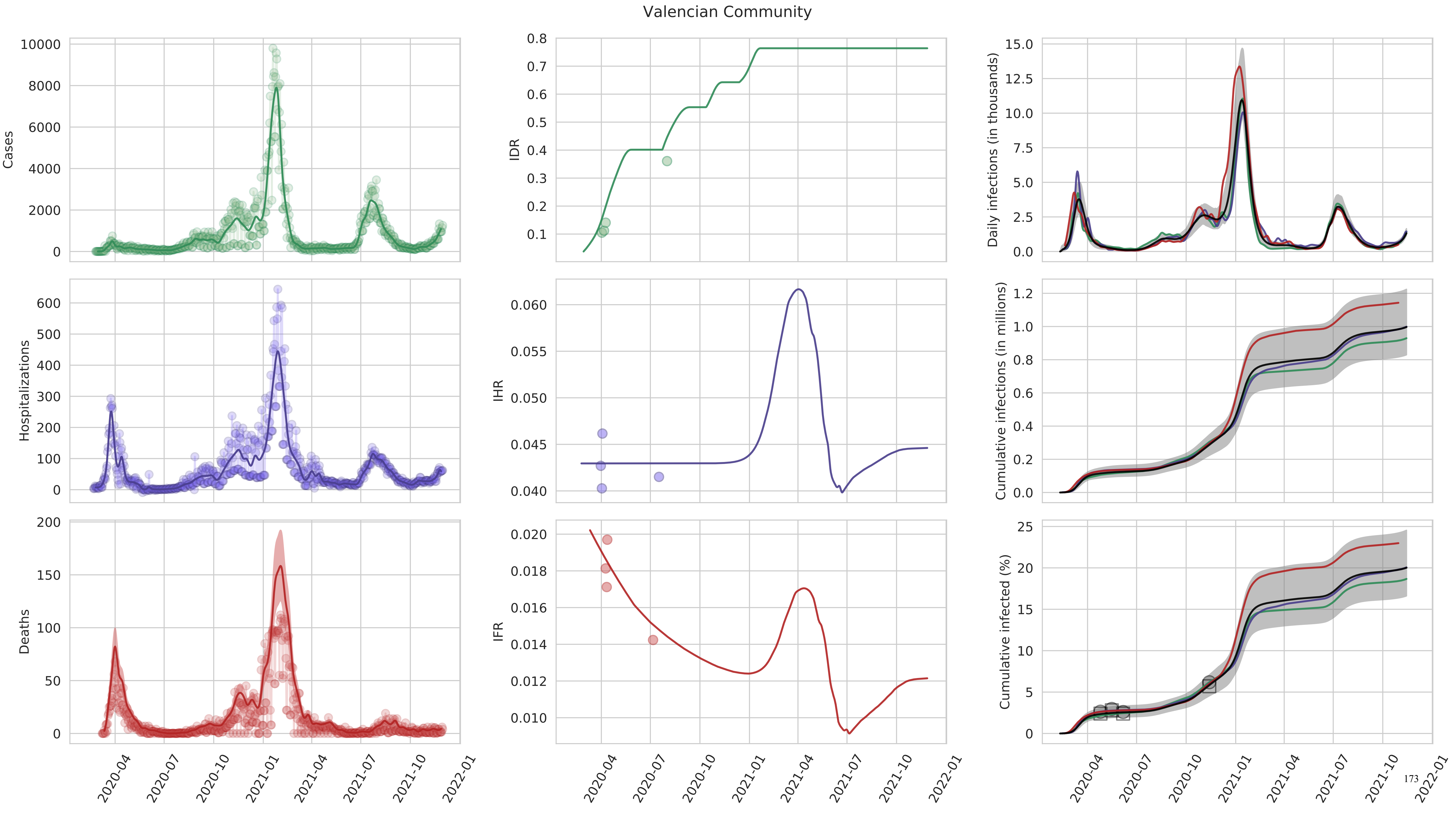
Valencian Community
Cases
Hospitalizations
Deaths
IDR
IHR
IFR
Daily infections (in thousands)
Cumulative infections (in millions)
Cumulative infected (%)
2020-04
2020-07
2020-10
2021-01
2021-04
2021-07
2021-10
2022-01

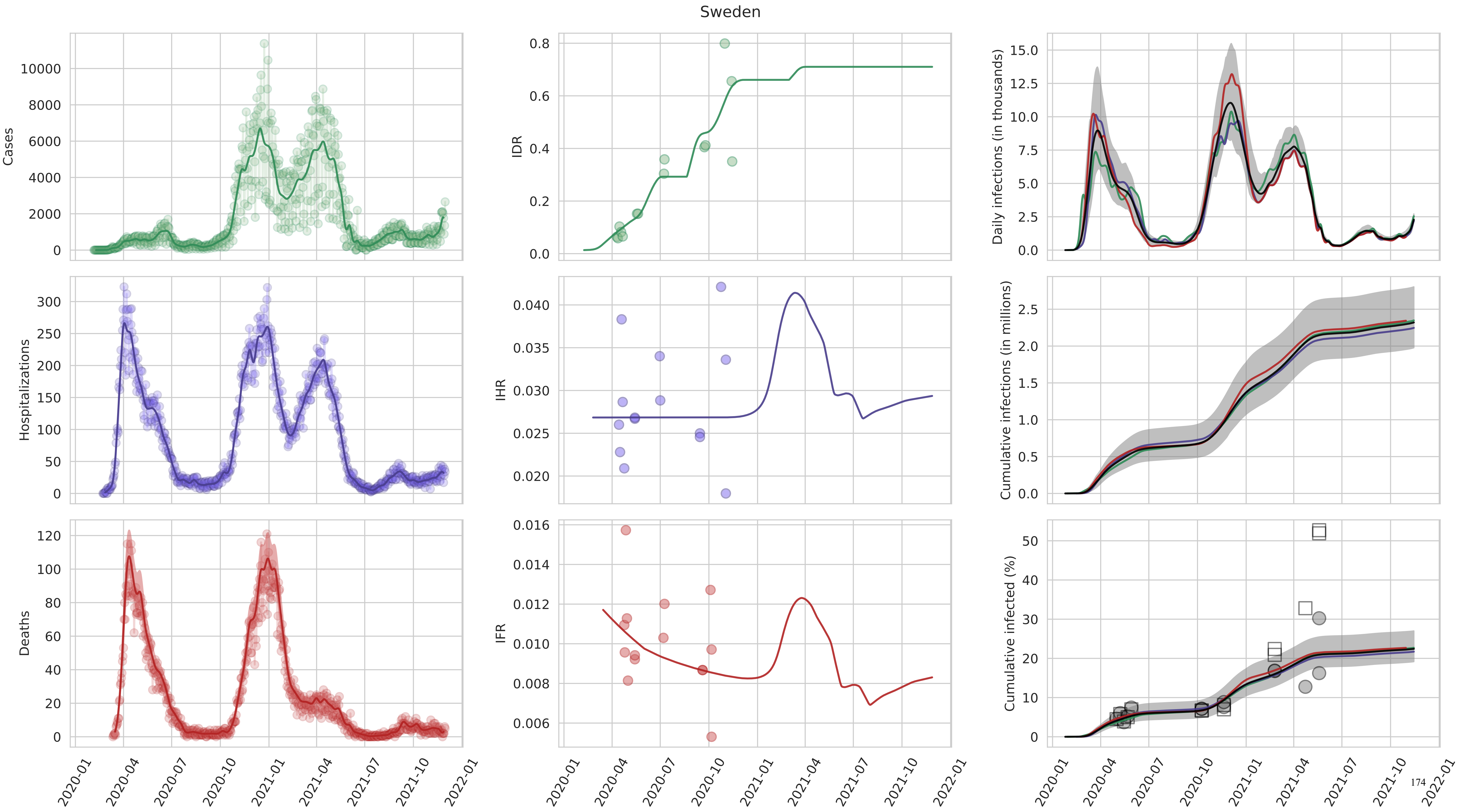
Sweden
Cases
IDR
Daily infections (in thousands)
Hospitalizations
IHR
Cumulative infections (in millions)
Deaths
IFR
Cumulative infected (%)
2020-01
2020-04
2020-07
2020-10
2021-01
2021-04
2021-07
2021-10
2022-01

Switzerland

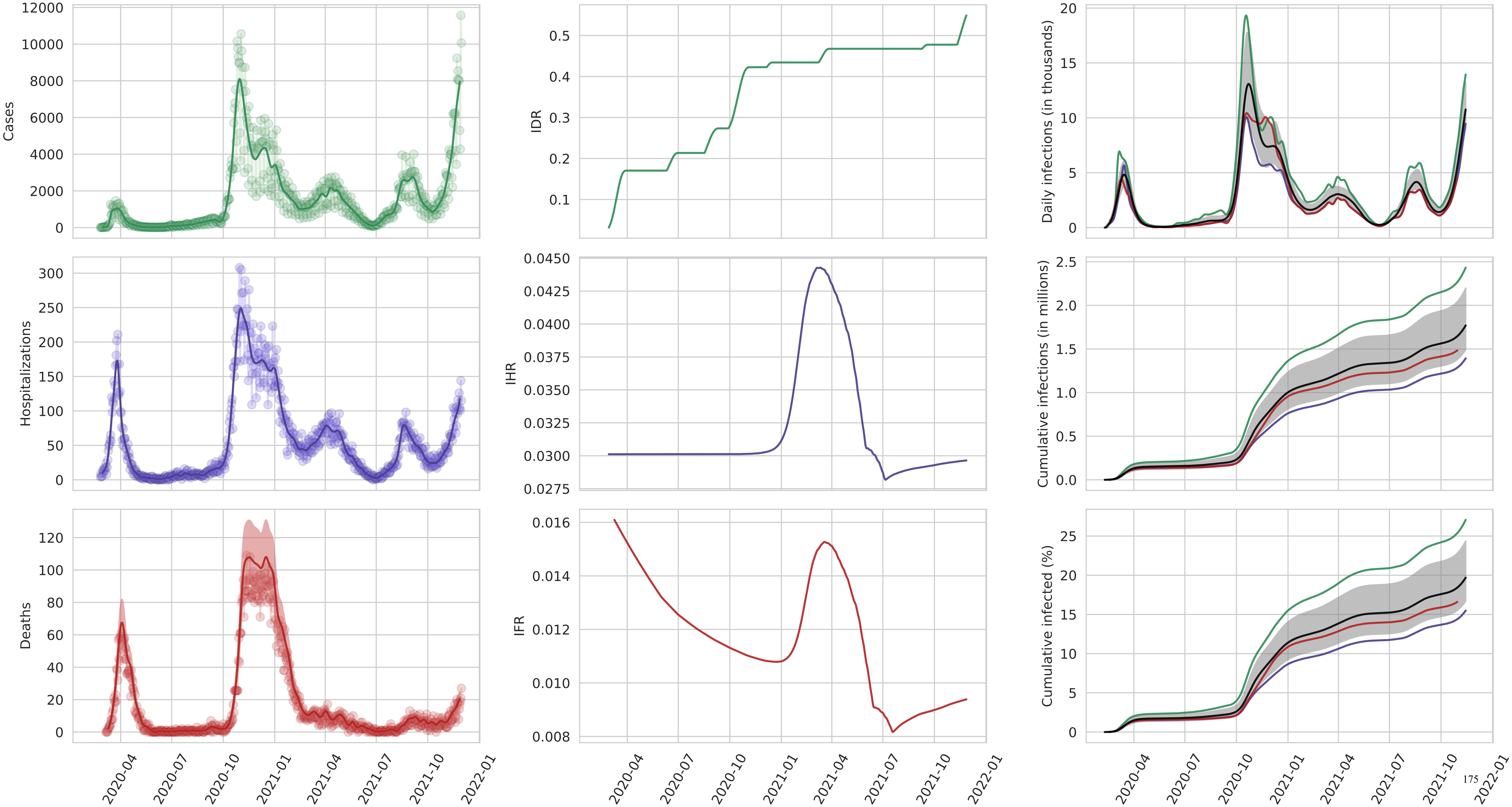

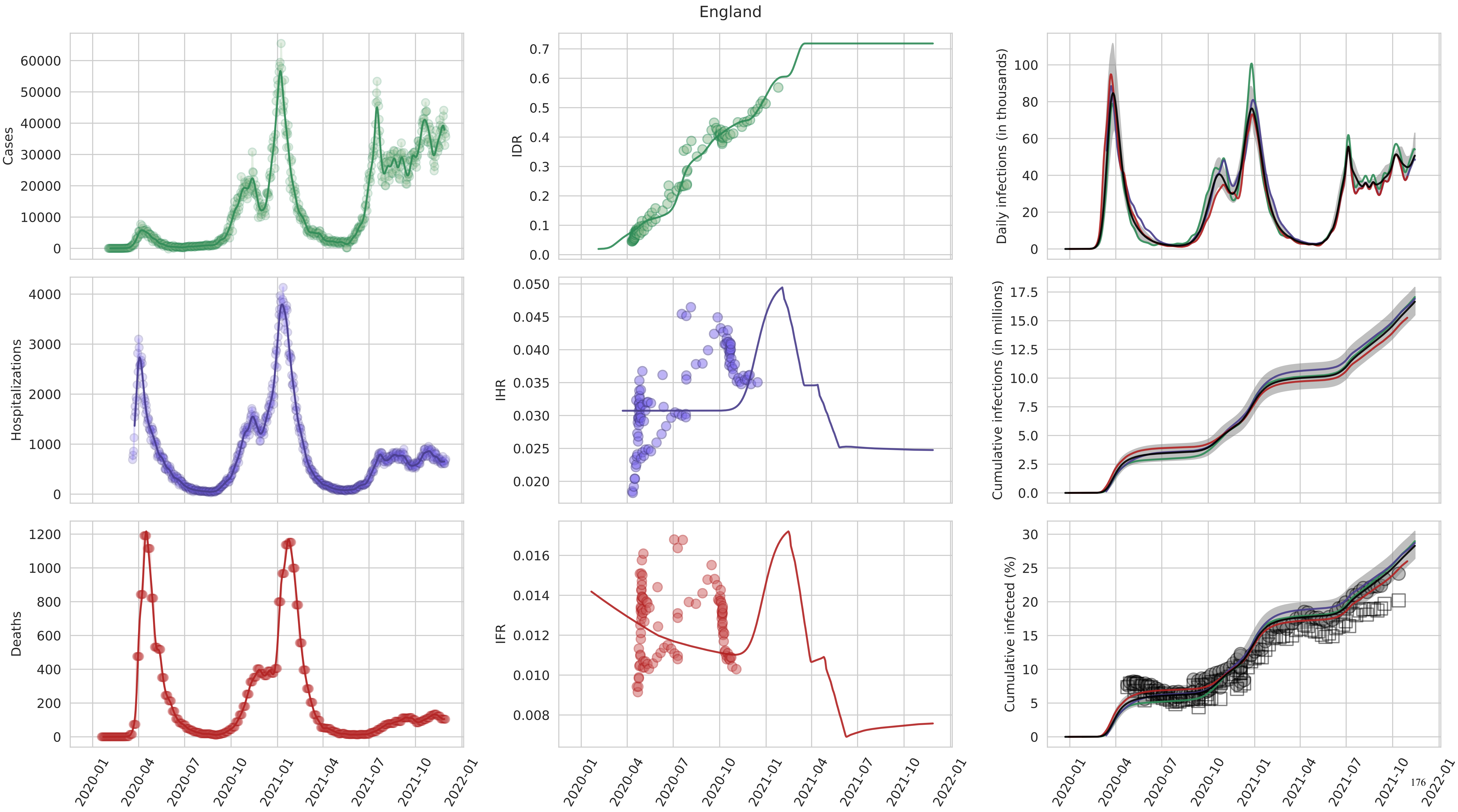

England
Cases
IDR
Daily infections (in thousands)
Hospitalizations
IHR
Cumulative infections (in millions)
Deaths
IFR
Cumulative infected (%)
2020-01
2020-04
2020-07
2020-10
2021-01
2021-04
2021-07
2021-10
2022-01

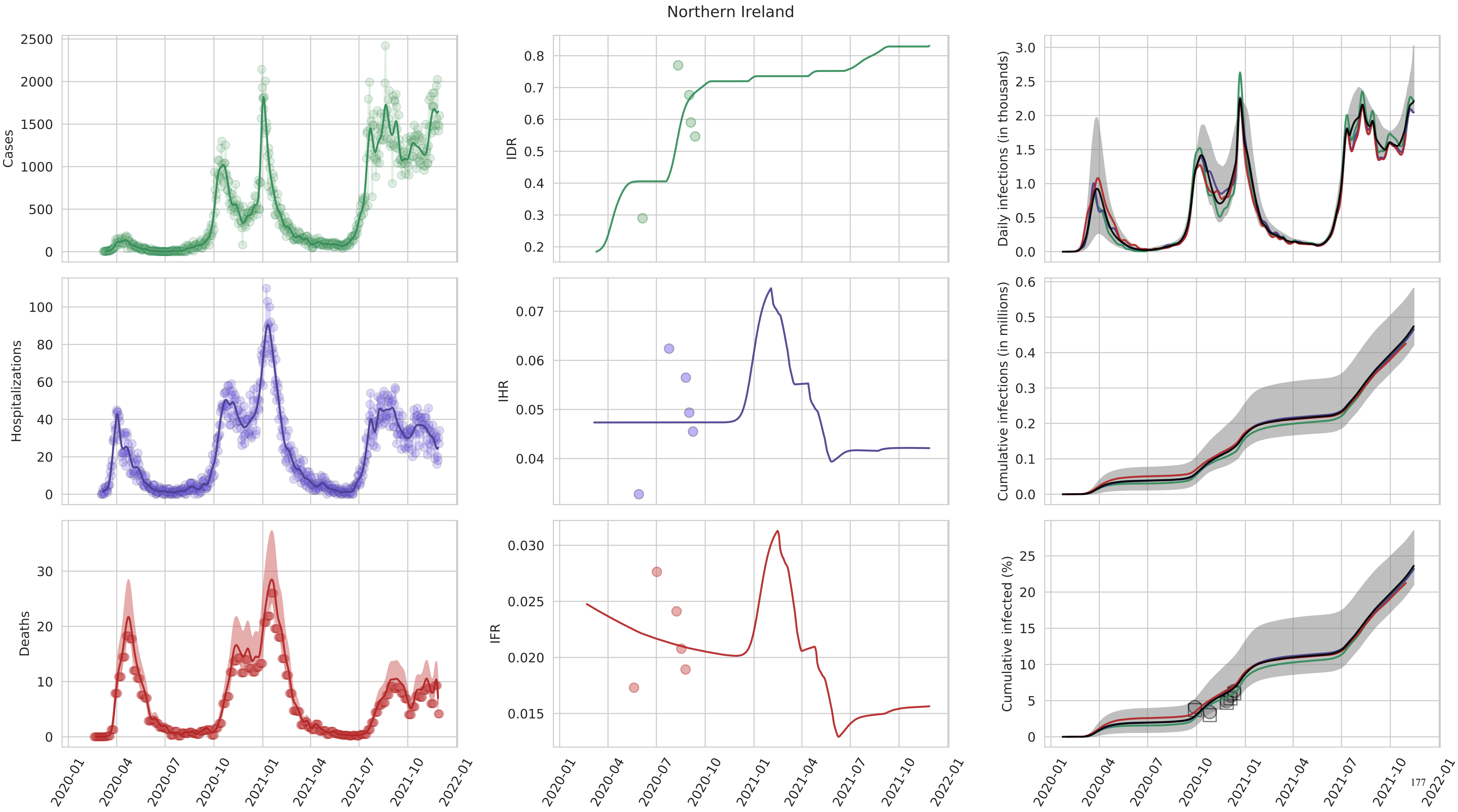
Northern Ireland
Cases
IDR
Daily infections (in thousands)
Hospitalizations
IHR
Cumulative infections (in millions)
Deaths
IFR
Cumulative infected (%)
2020-01
2020-04
2020-07
2020-10
2021-01
2021-04
2021-07
2021-10
2022-01

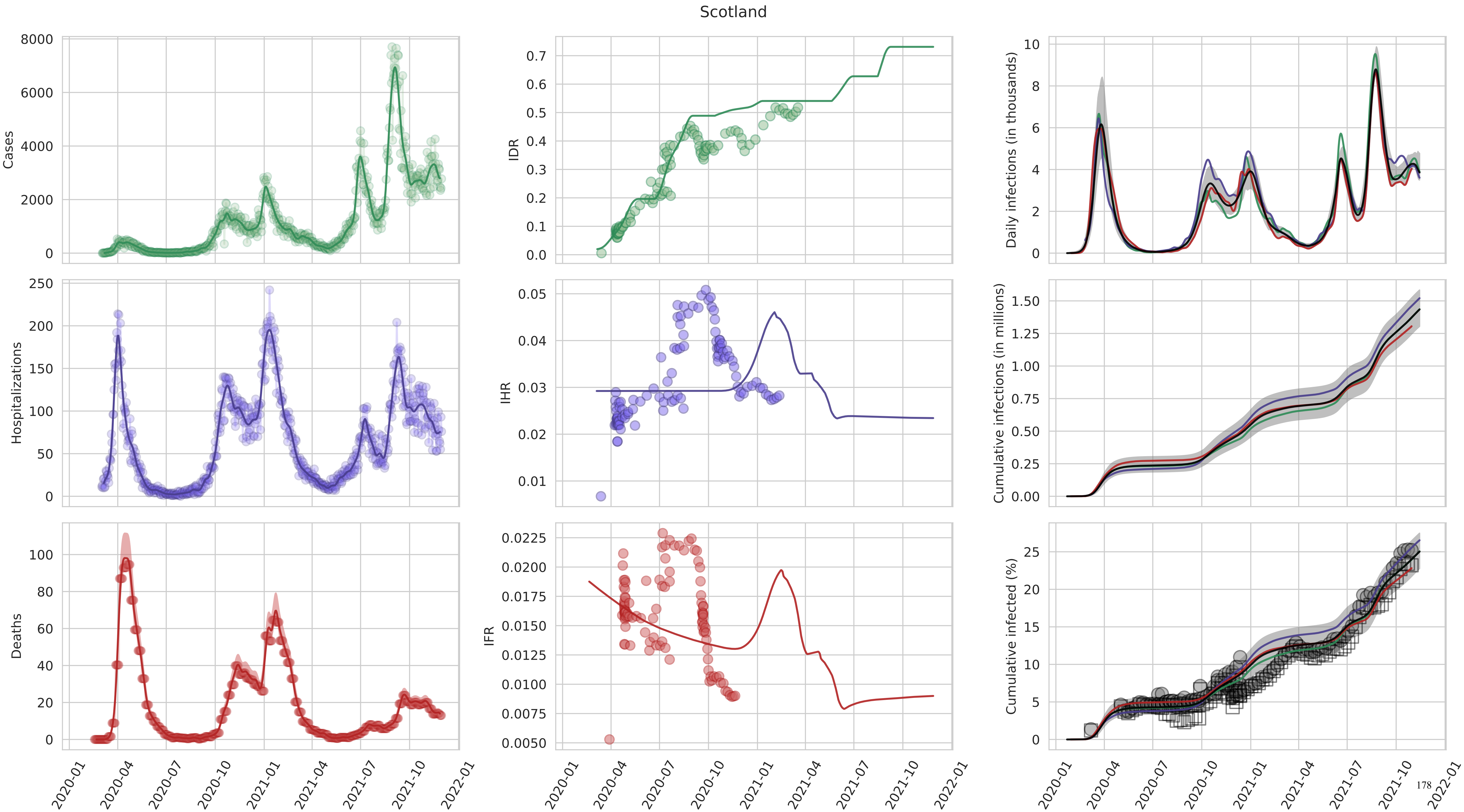

Scotland
Cases
IDR
Daily infections (in thousands)
Hospitalizations
IHR
Cumulative infections (in millions)
Deaths
IFR
Cumulative infected (%)
2020-01
2020-04
2020-07
2020-10
2021-01
2021-04
2021-07
2021-10
2022-01

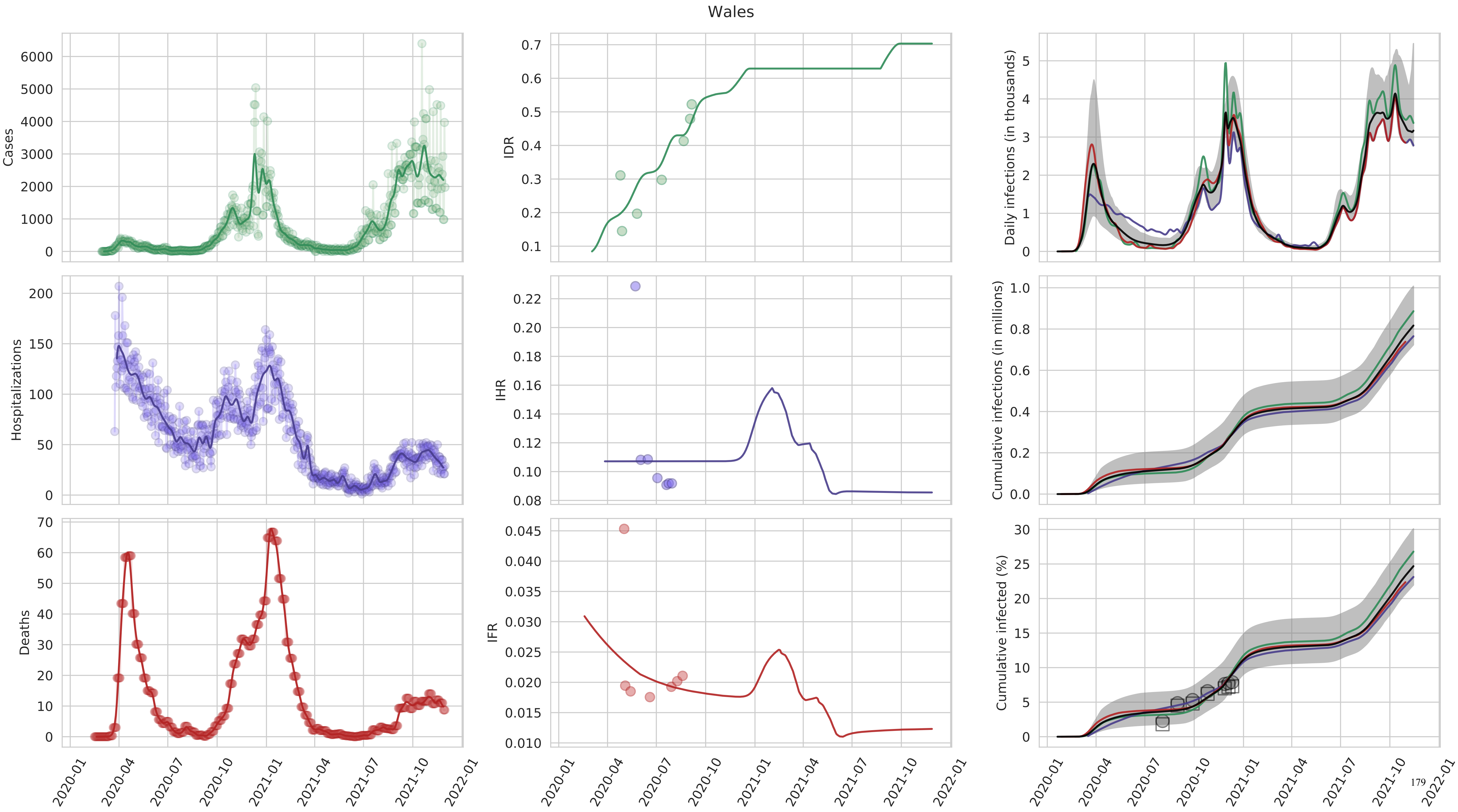

Wales
Cases
Hospitalizations
Deaths
IDR
IHR
IFR
Daily infections (in thousands)
Cumulative infections (in millions)
Cumulative infected (%)
2020-01
2020-04
2020-07
2020-10
2021-01
2021-04
2021-07
2021-10
2022-01

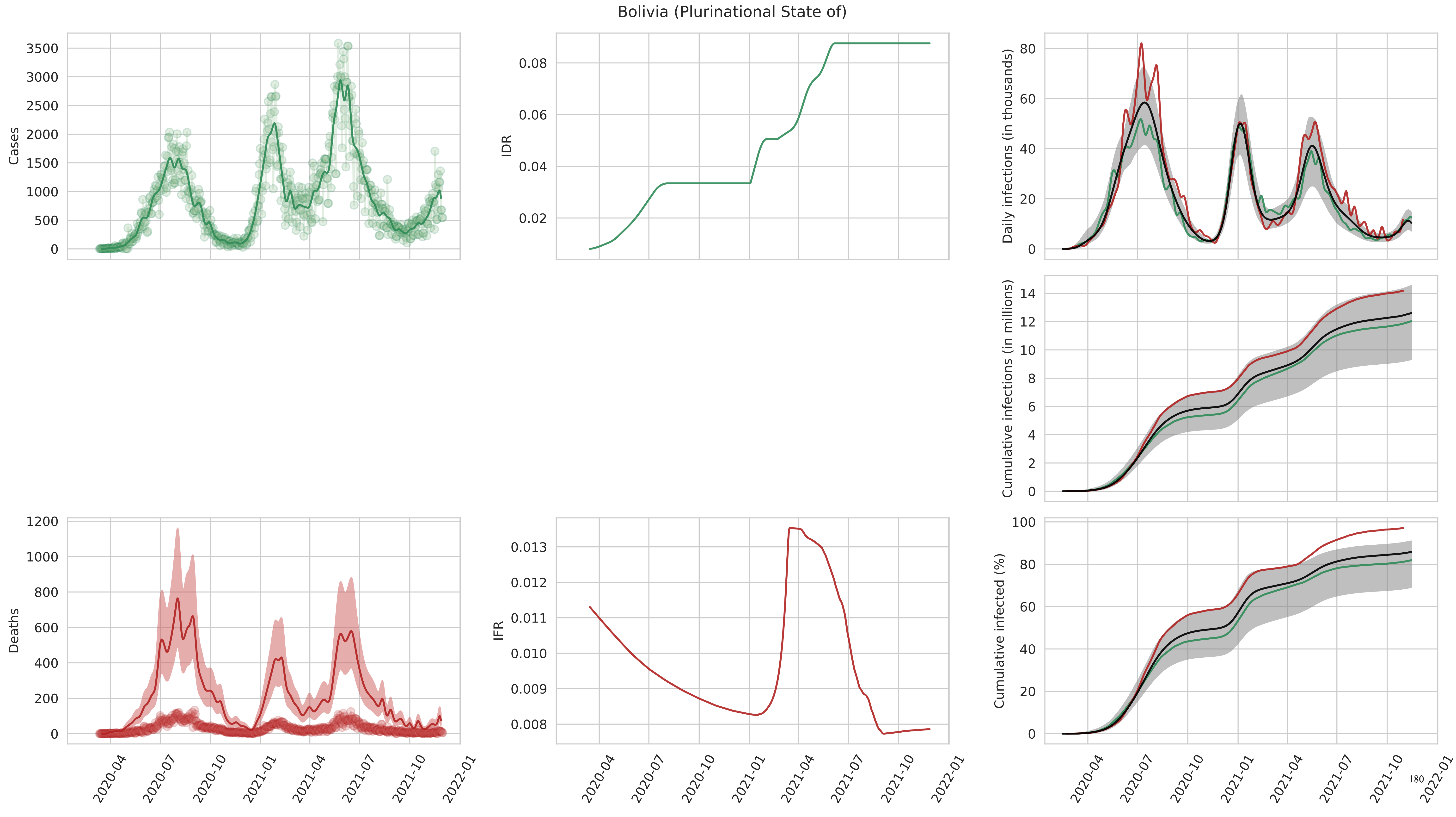
Bolivia (Plurinational State of)
Cases
IDR
Daily infections (in thousands)
Cumulative infections (in millions)
Deaths
IFR
Cumulative infected (%)
2020-04
2020-07
2020-10
2021-01
2021-04
2021-07
2021-10
2022-01

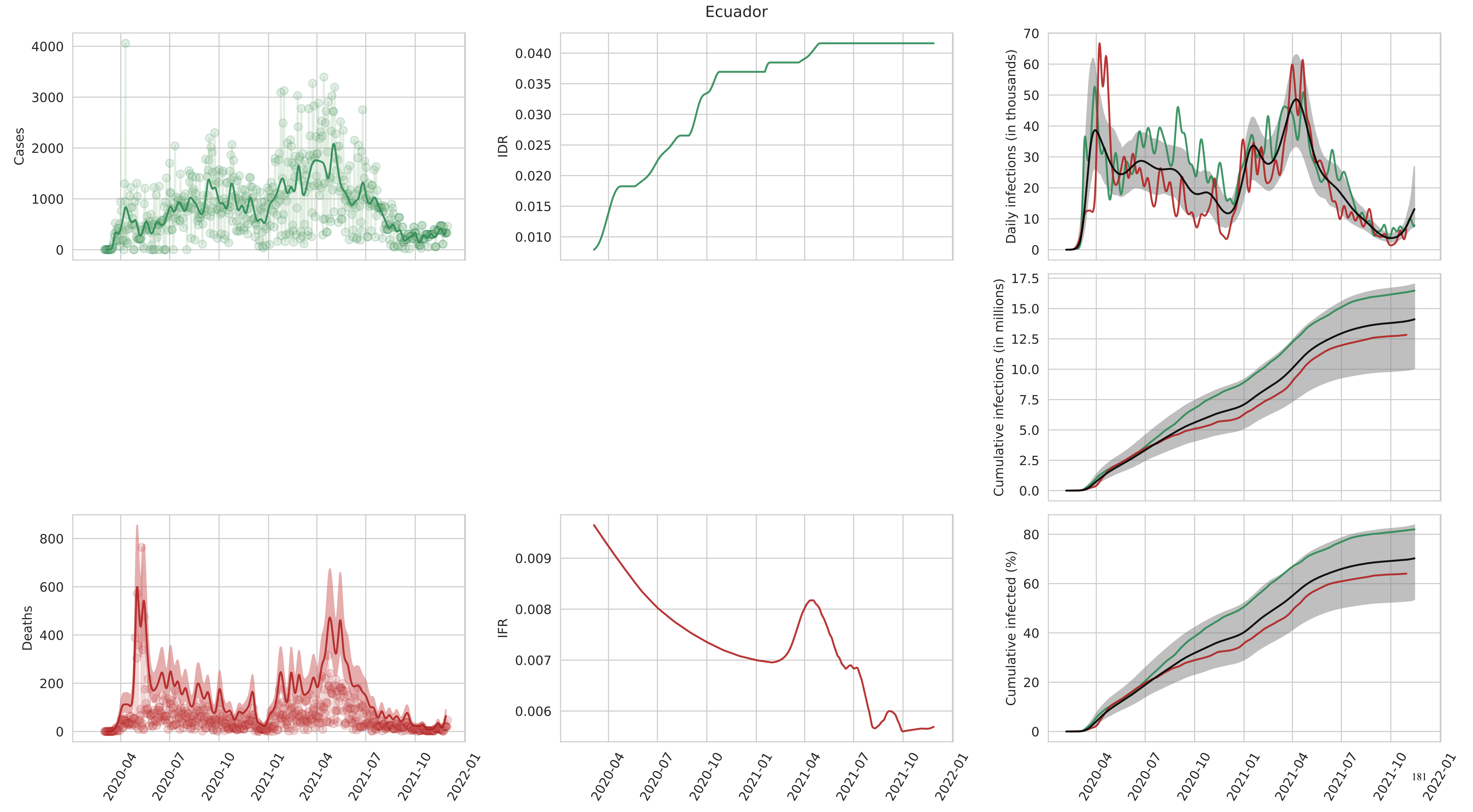
Ecuador
Cases
IDR
Daily infections (in thousands)
Cumulative infections (in millions)
Deaths
IFR
Cumulative infected (%)
2020-04
2020-07
2020-10
2021-01
2021-04
2021-07
2021-10
2022-01

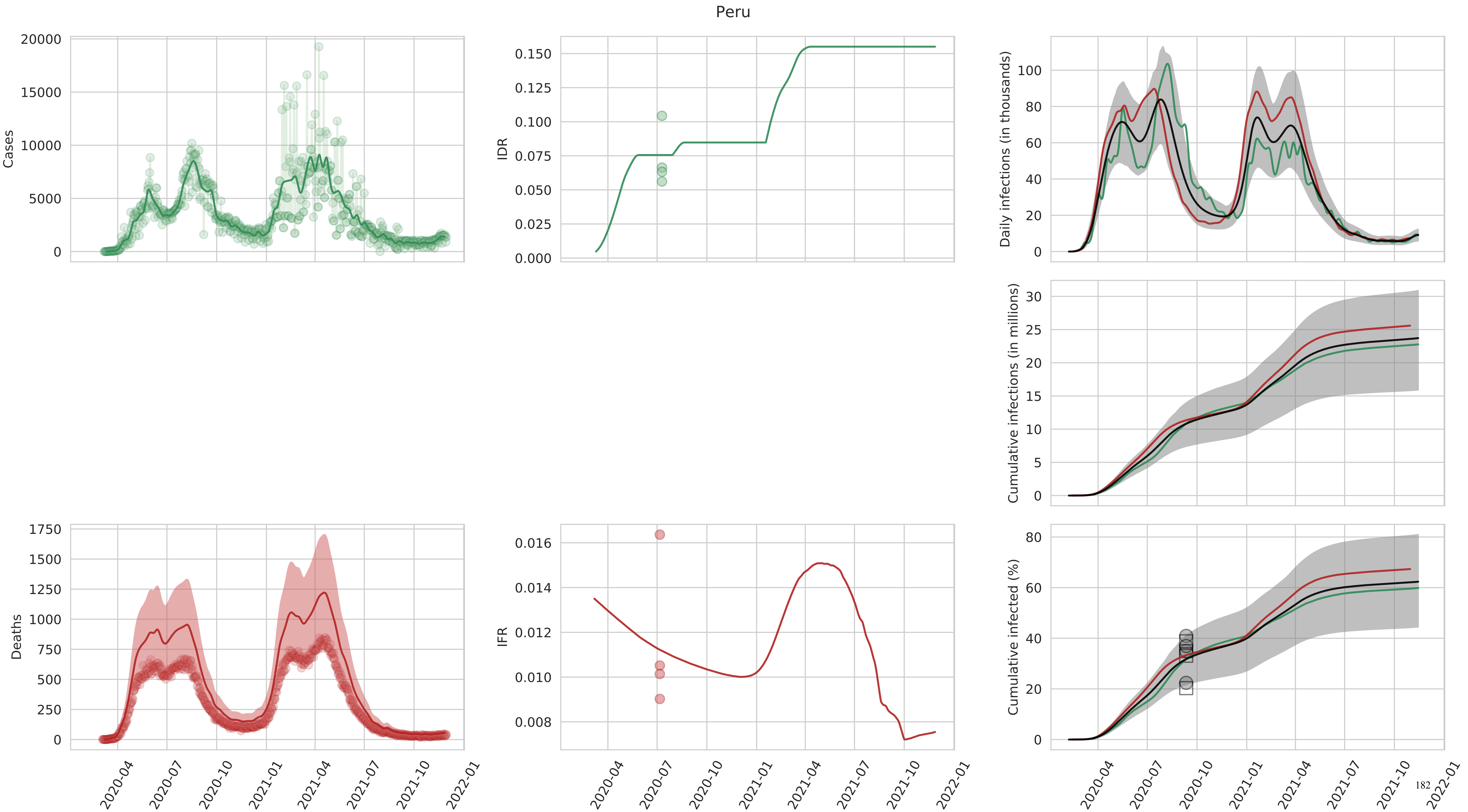

Peru
Cases
IDR
Daily infections (in thousands)
Cumulative infections (in millions)
Deaths
IFR
Cumulative infected (%)
2020-04
2020-07
2020-10
2021-01
2021-04
2021-07
2021-10
2022-01

Antigua and Barbuda

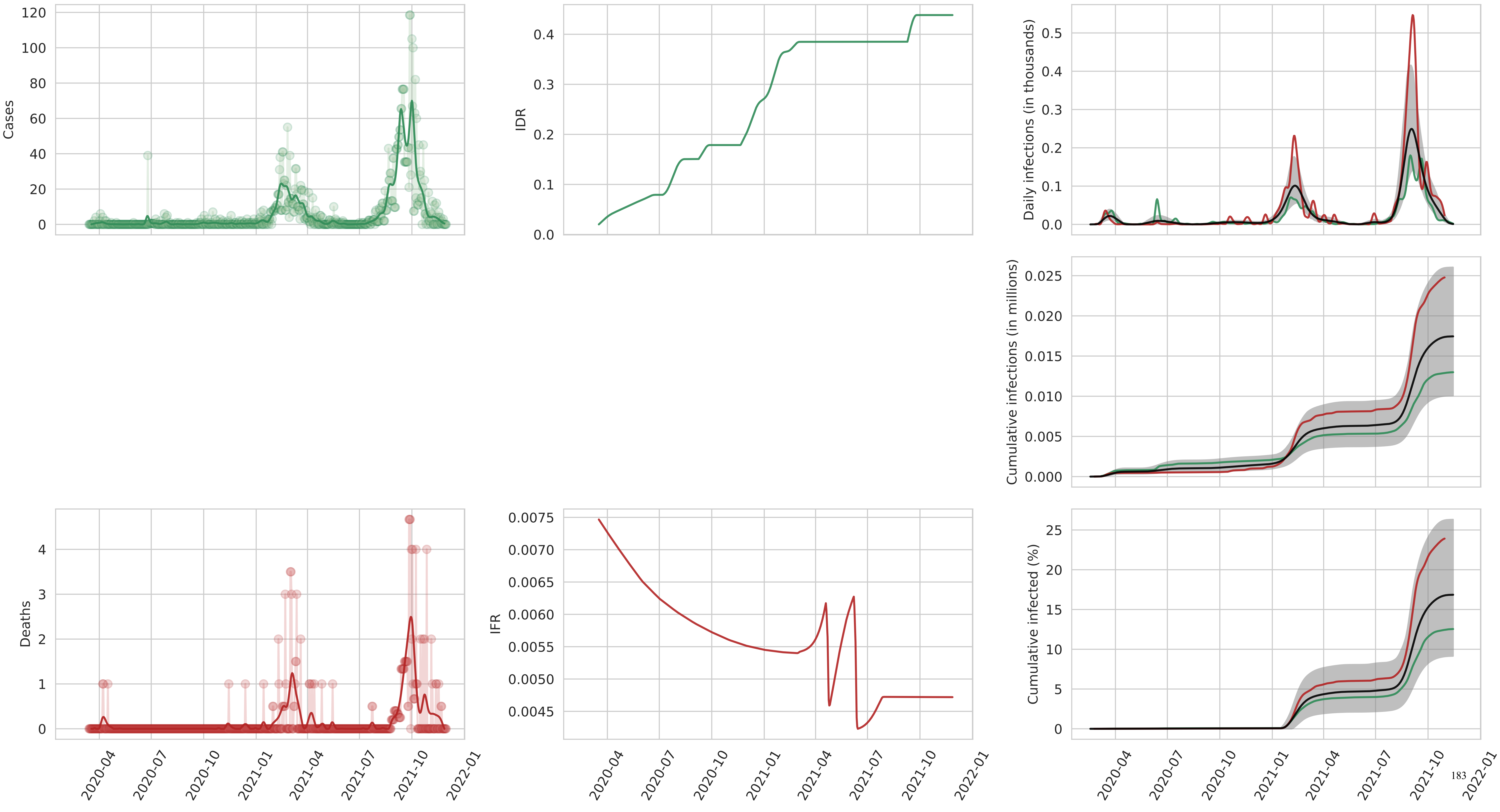

Bahamas

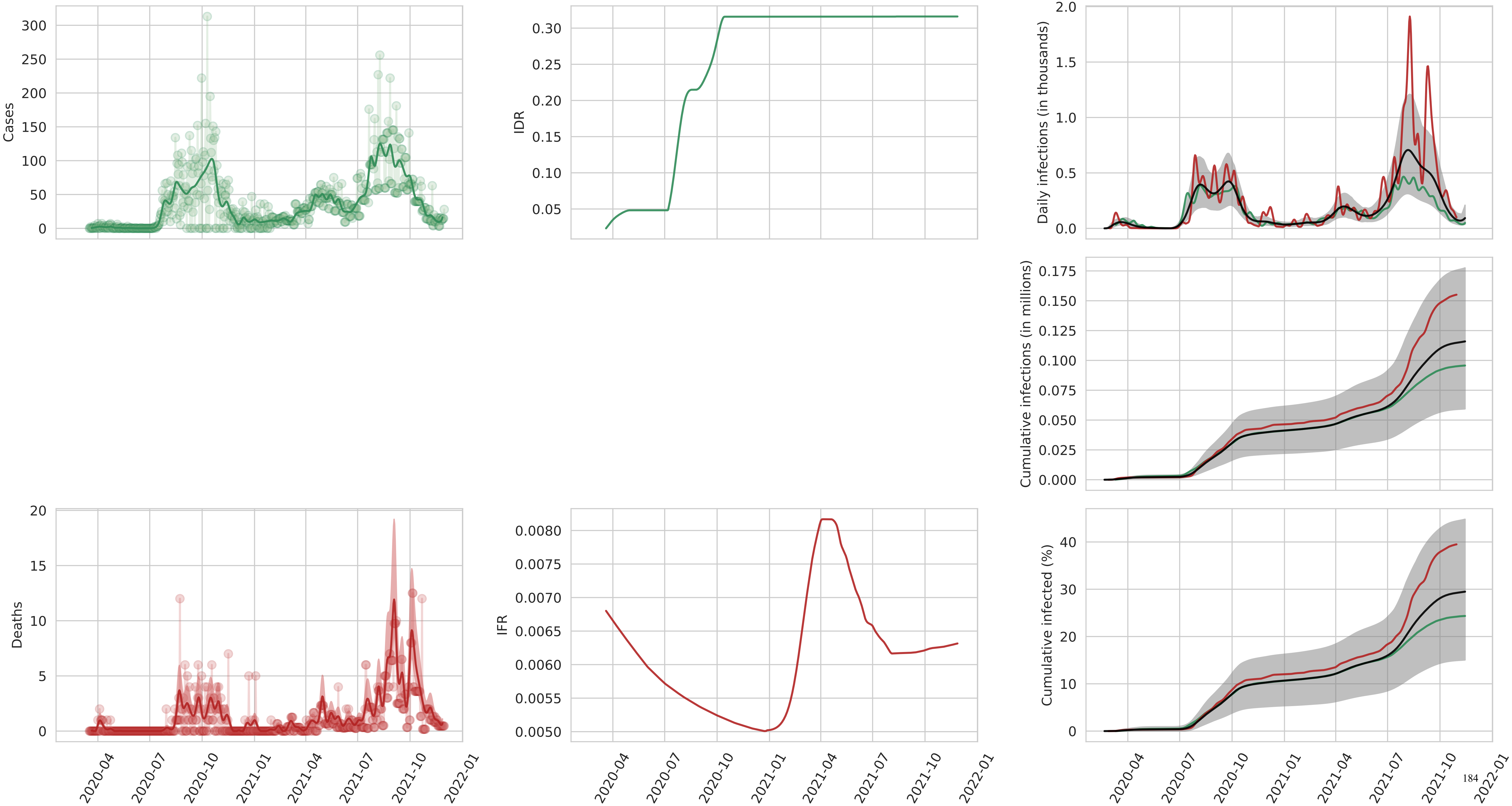

Cases
IDR
Daily infections (in thousands)
Cumulative infections (in millions)
Deaths
IFR
Cumulative infected (%)
2020-04
2020-07
2020-10
2021-01
2021-04
2021-07
2021-10
2022-01

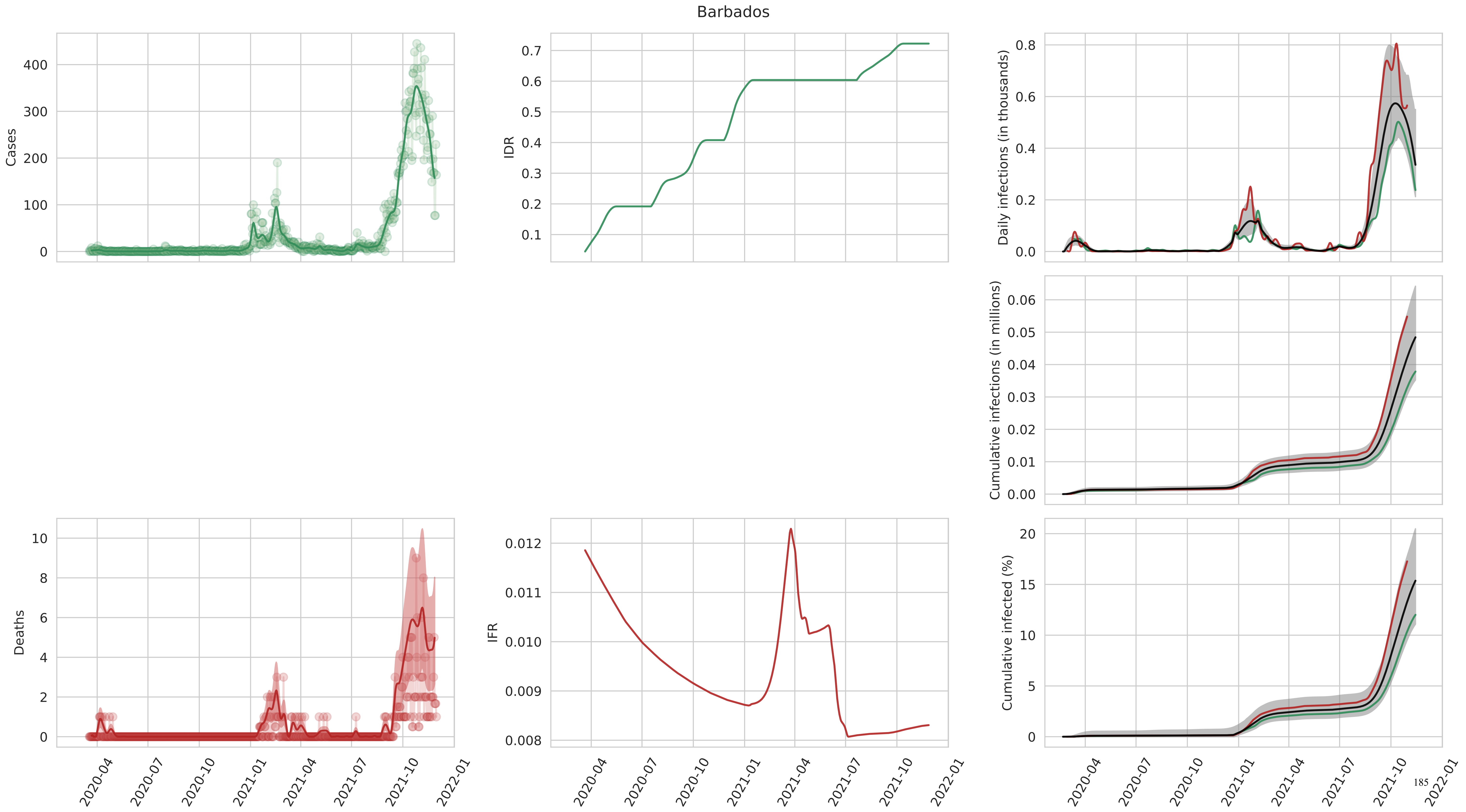
Barbados
Cases
IDR
Daily infections (in thousands)
Cumulative infections (in millions)
Deaths
IFR
Cumulative infected (%)
2020-04
2020-07
2020-10
2021-01
2021-04
2021-07
2021-10
2022-01

Belize

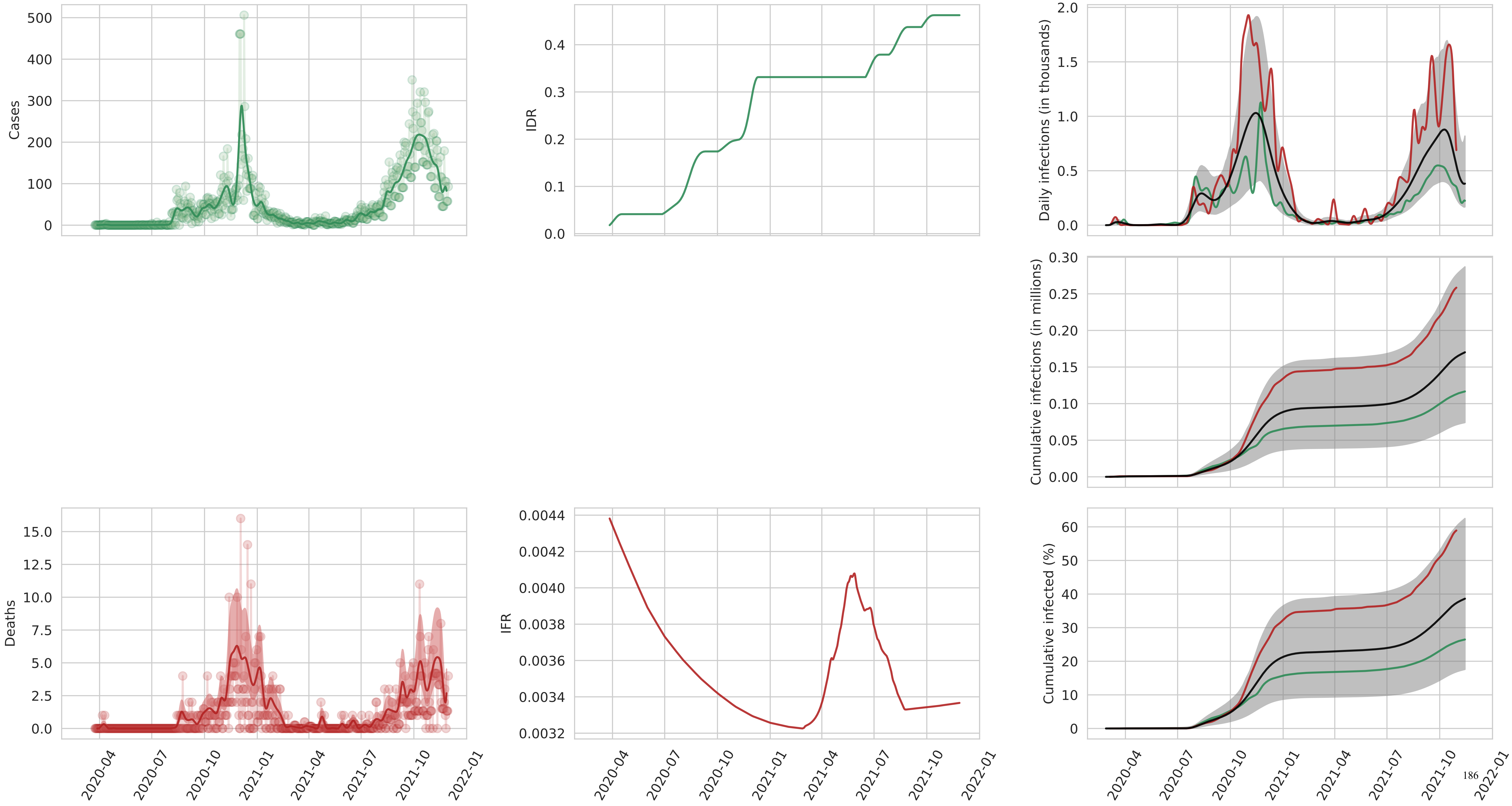

Cases
IDR
Daily infections (in thousands)
Cumulative infections (in millions)
Deaths
IFR
Cumulative infected (%)
2020-04
2020-07
2020-10
2021-01
2021-04
2021-07
2021-10
2022-01

Bermuda

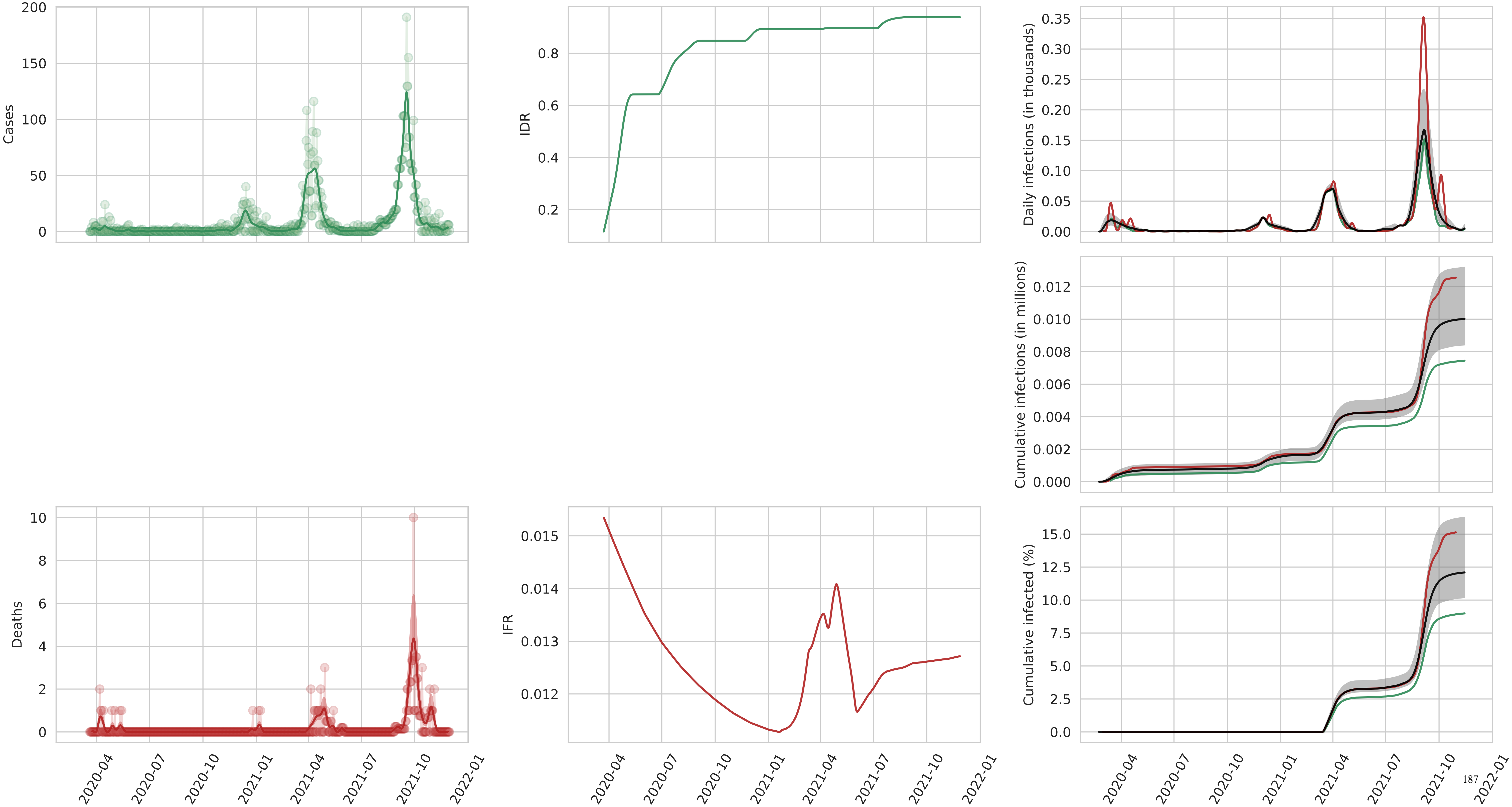

Cases
IDR
Daily infections (in thousands)
Cumulative infections (in millions)
Deaths
IFR
Cumulative infected (%)
2020-04
2020-07
2020-10
2021-01
2021-04
2021-07
2021-10
2022-01

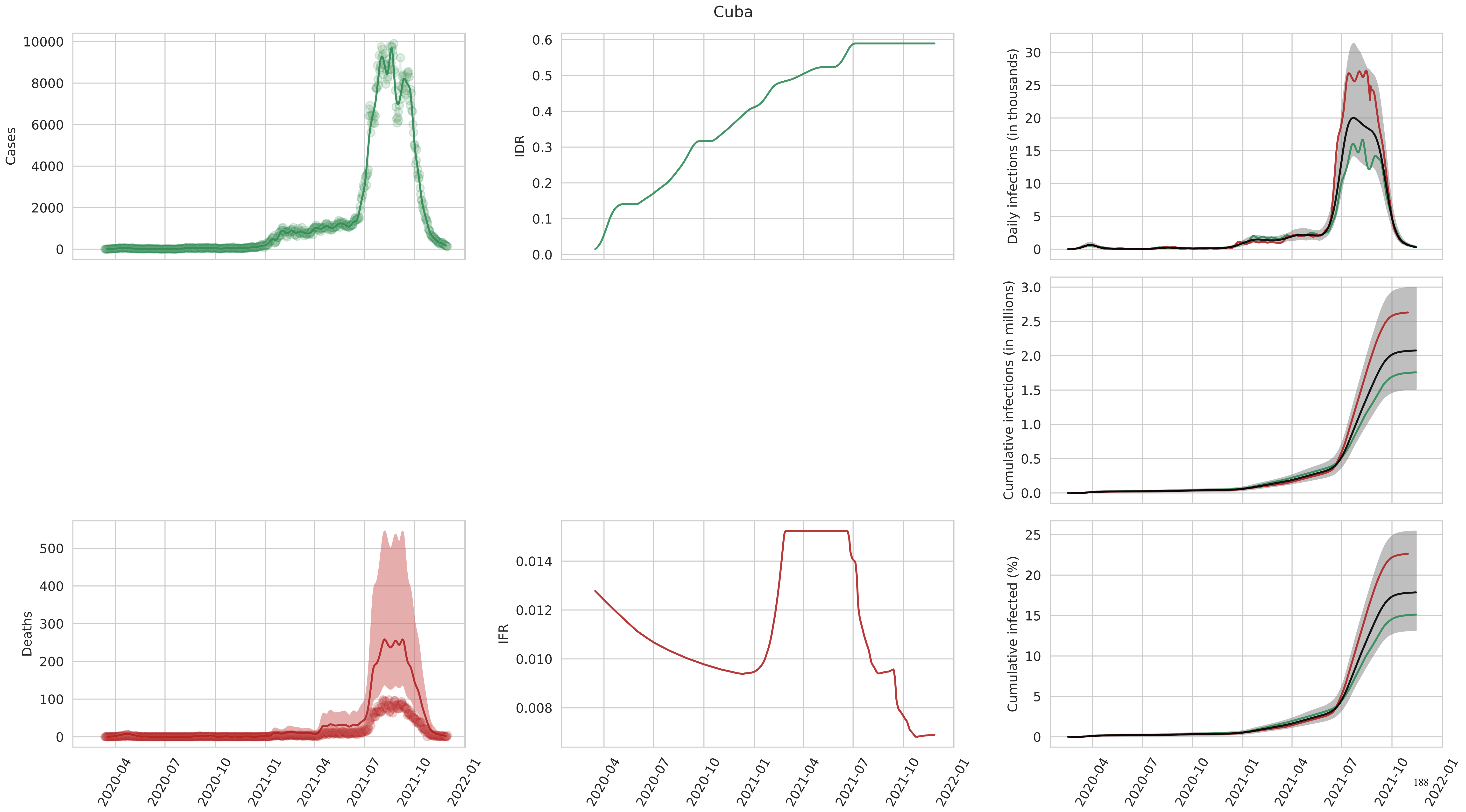
Cuba
Cases
IDR
Daily infections (in thousands)
Cumulative infections (in millions)
Deaths
IFR
Cumulative infected (%)
2020-04
2020-07
2020-10
2021-01
2021-04
2021-07
2021-10
2022-01

Dominica

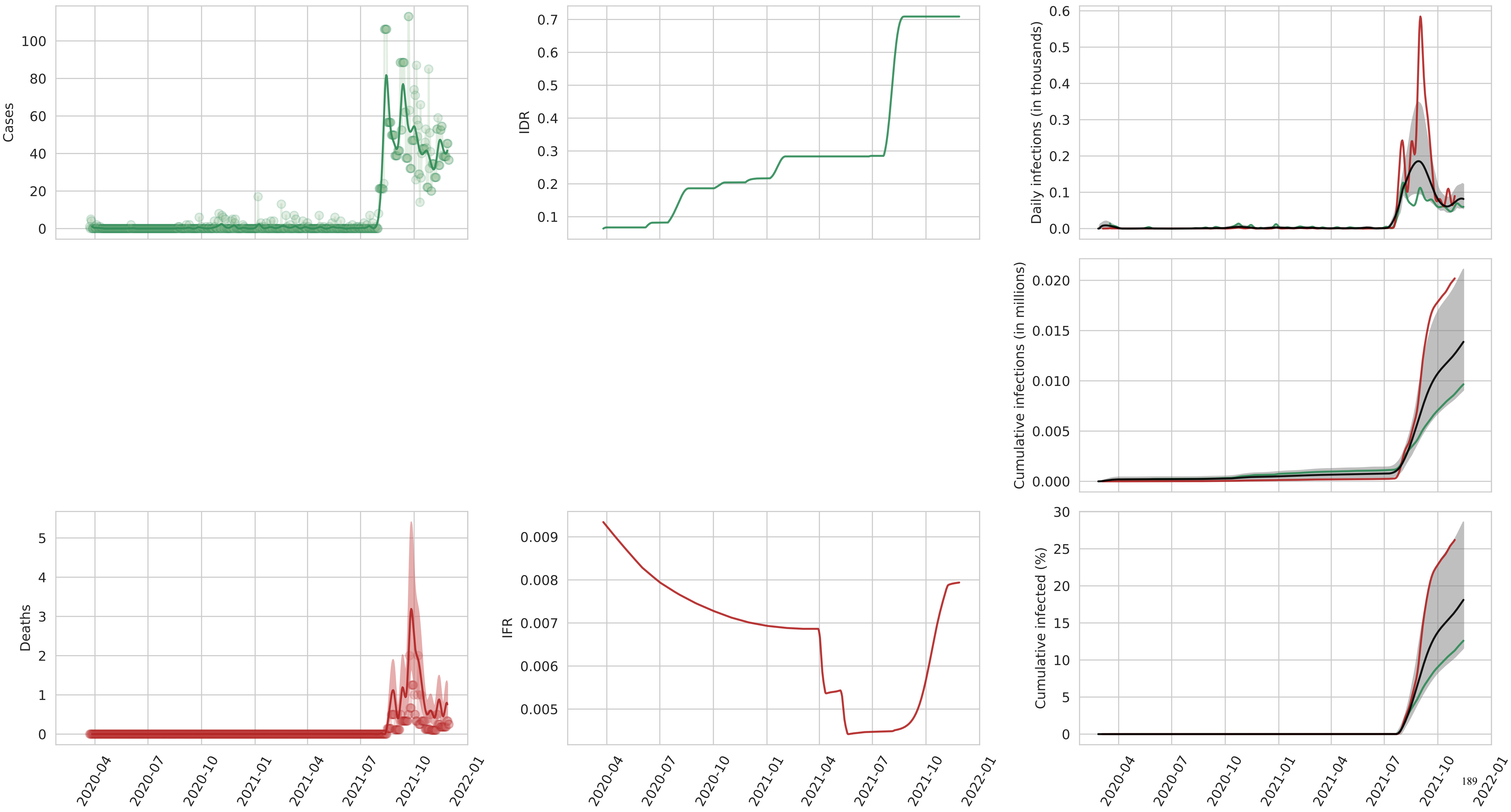

Cases
IDR
Daily infections (in thousands)
Cumulative infections (in millions)
Deaths
IFR
Cumulative infected (%)
2020-04
2020-07
2020-10
2021-01
2021-04
2021-07
2021-10
2022-01

Dominican Republic

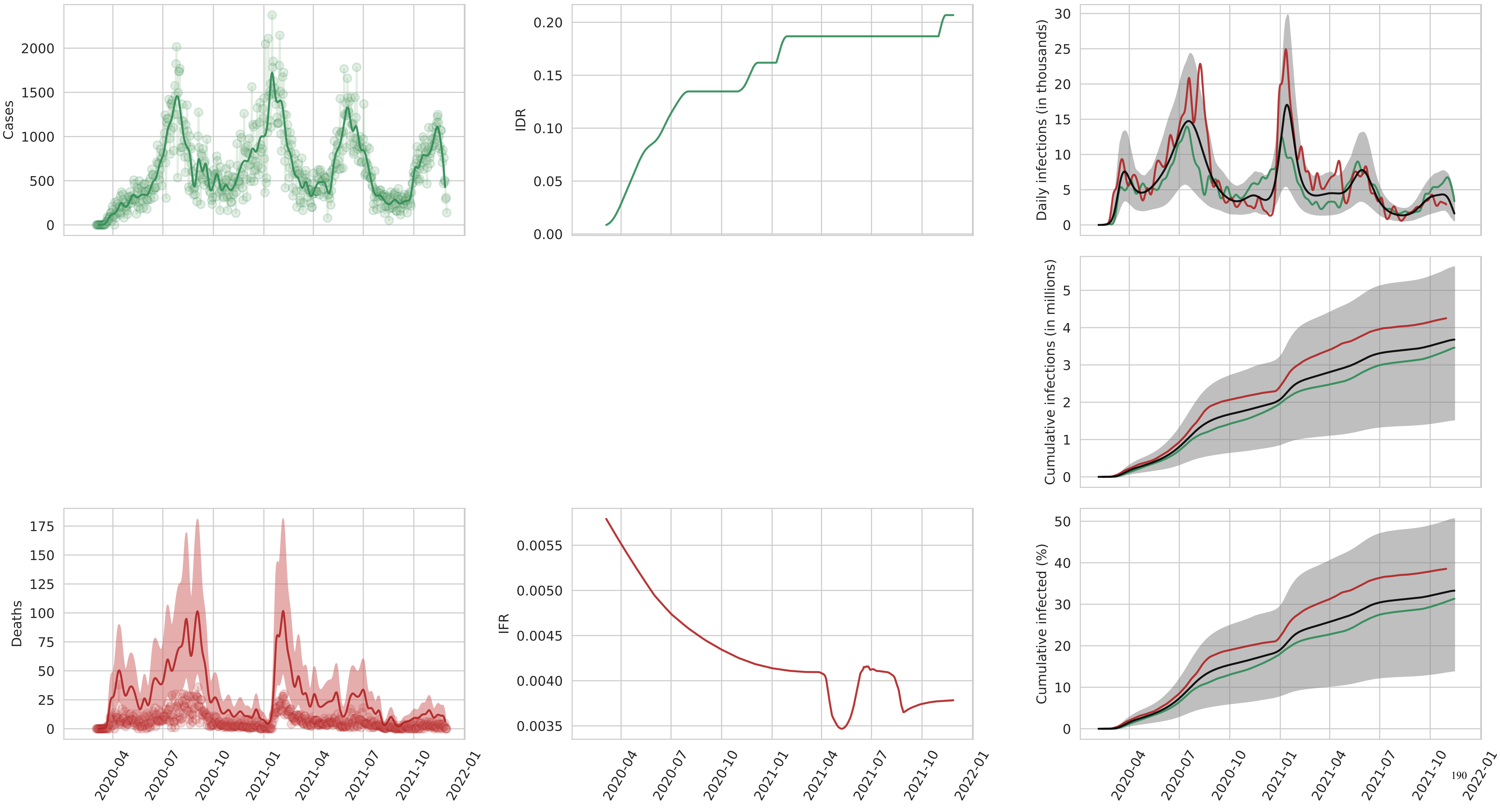

Grenada

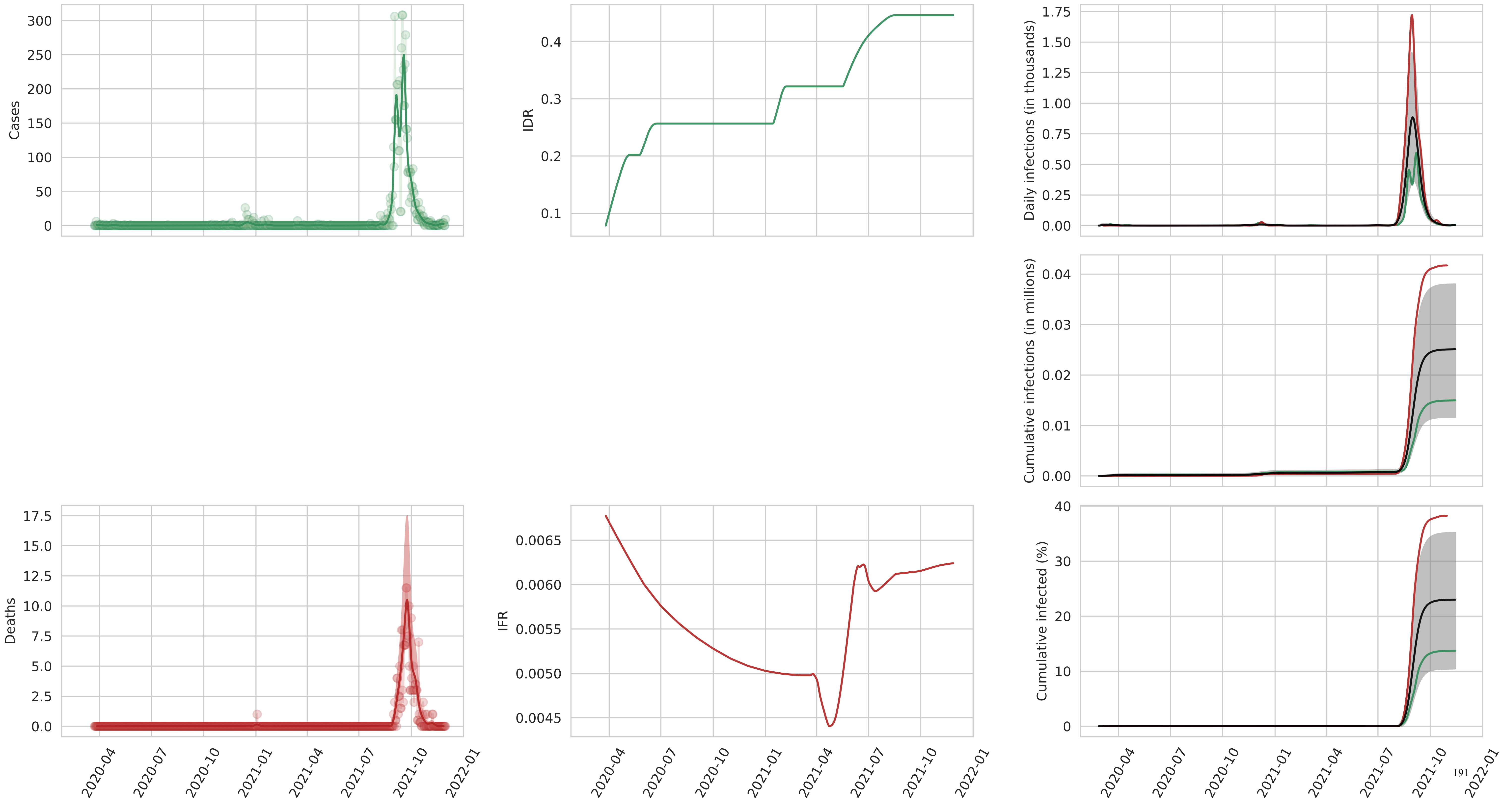
Cases
IDR
Daily infections (in thousands)
Cumulative infections (in millions)
Deaths
IFR
Cumulative infected (%)
2020-04
2020-07
2020-10
2021-01
2021-04
2021-07
2021-10
2022-01

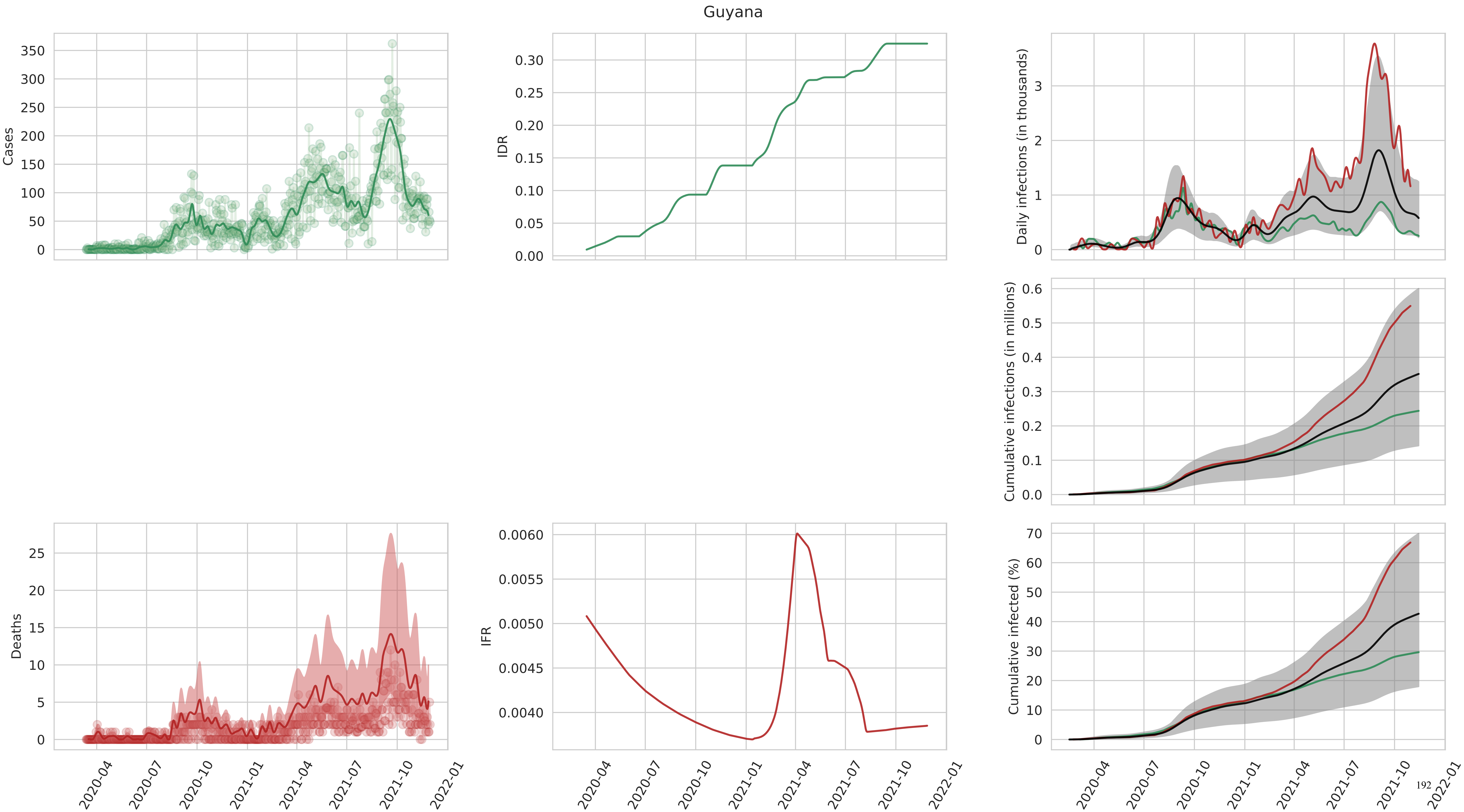
Guyana
Cases
IDR
Daily infections (in thousands)
Cumulative infections (in millions)
Deaths
IFR
Cumulative infected (%)
2020-04
2020-07
2020-10
2021-01
2021-04
2021-07
2021-10
2022-01

Haiti

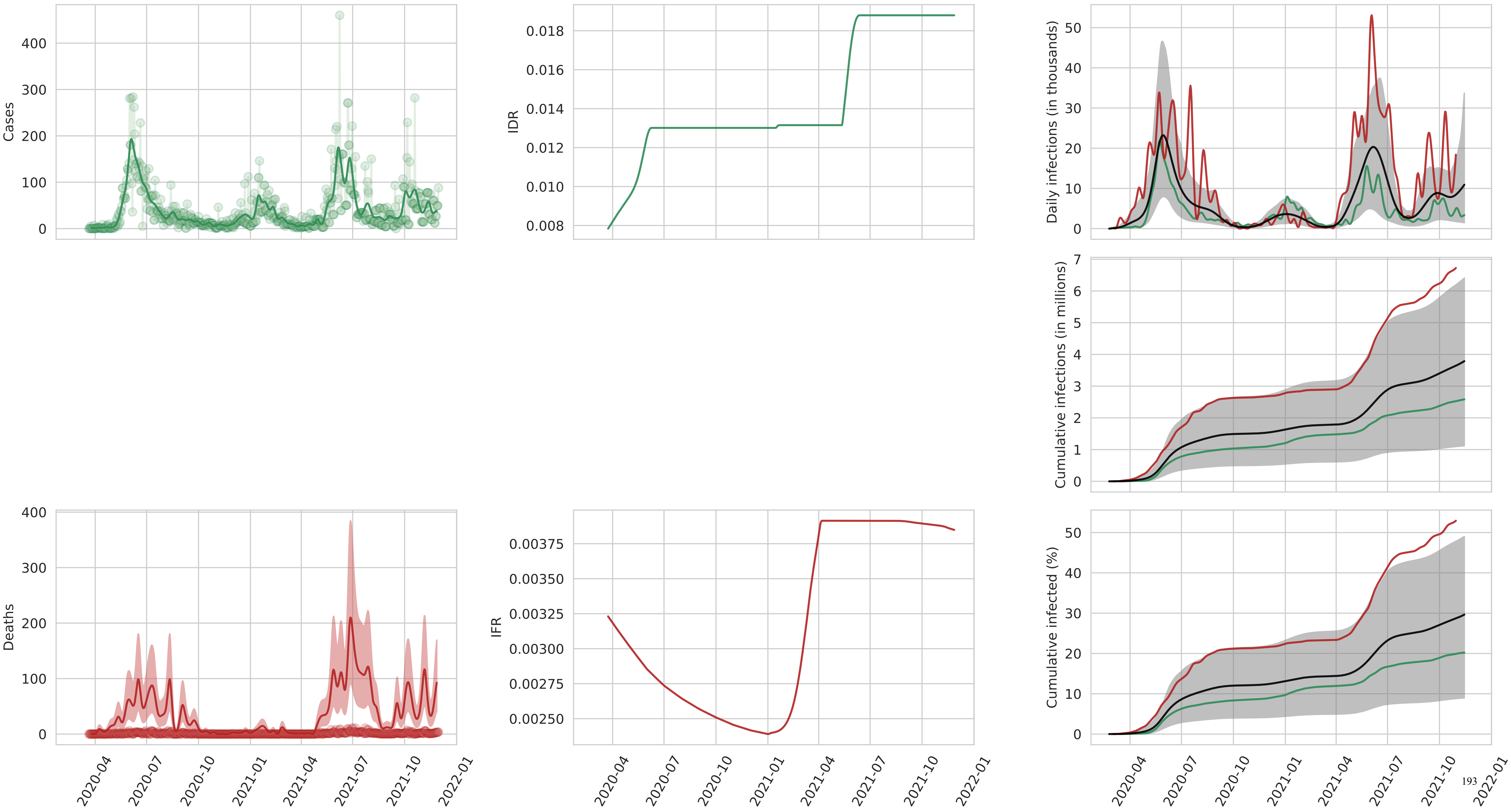
Cases
IDR
Daily infections (in thousands)
Cumulative infections (in millions)
Deaths
IFR
Cumulative infected (%)
2020-04
2020-07
2020-10
2021-01
2021-04
2021-07
2021-10
2022-01

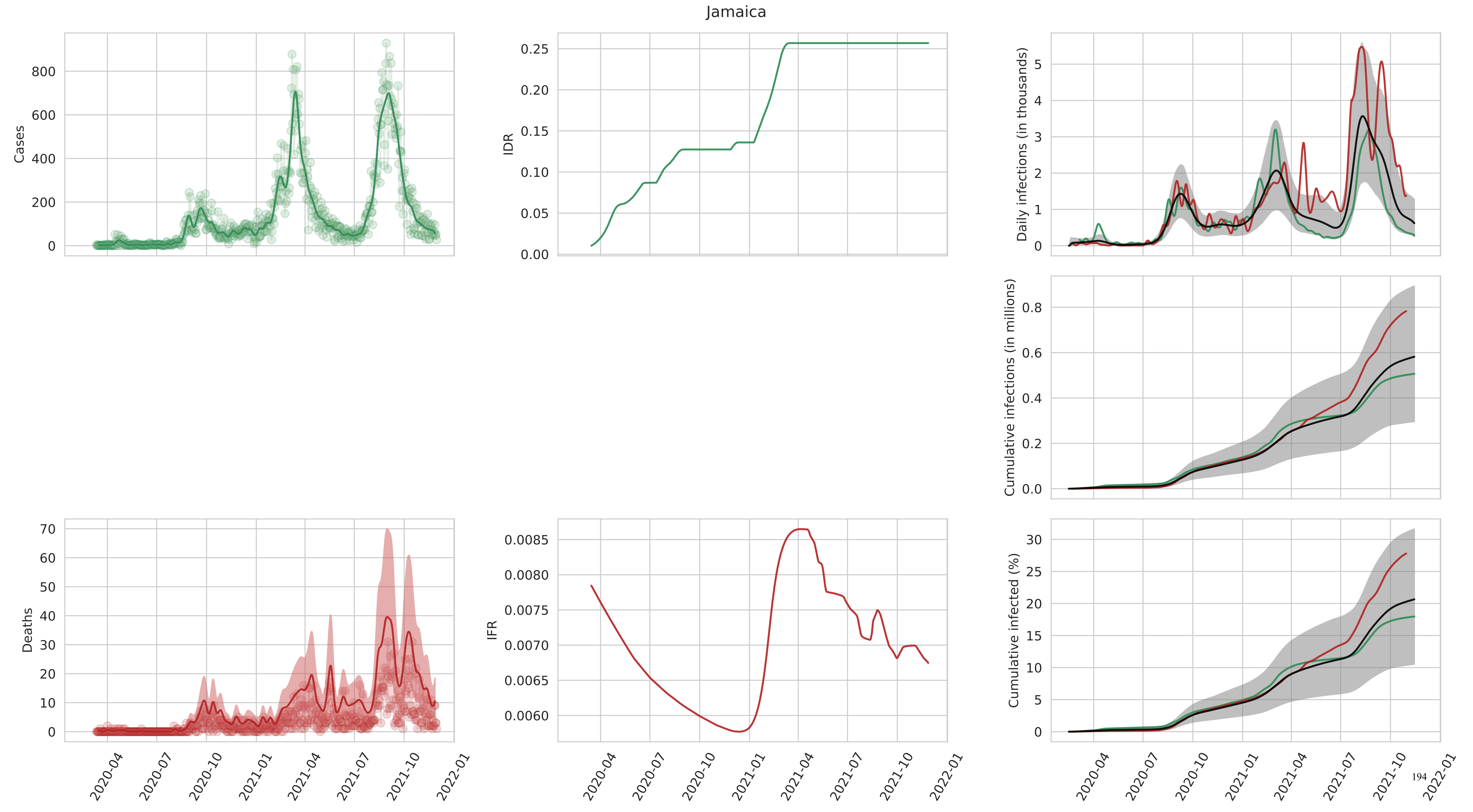

Jamaica
Cases
IDR
Daily infections (in thousands)
Cumulative infections (in millions)
Deaths
IFR
Cumulative infected (%)
2020-04
2020-07
2020-10
2021-01
2021-04
2021-07
2021-10
2022-01

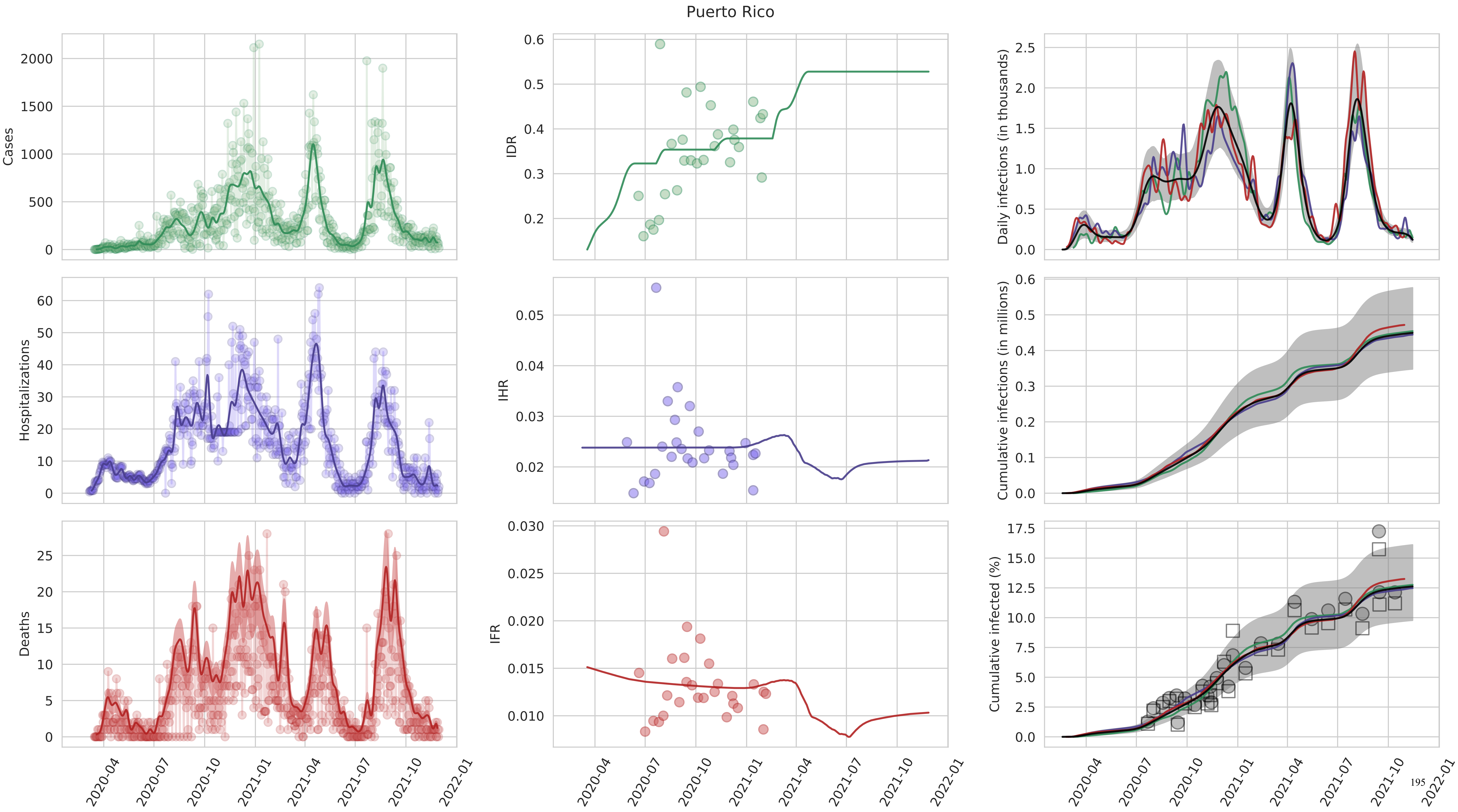
Puerto Rico
Cases
Hospitalizations
Deaths
IDR
IHR
IFR
Daily infections (in thousands)
Cumulative infections (in millions)
Cumulative infected (%)

Saint Kitts and Nevis

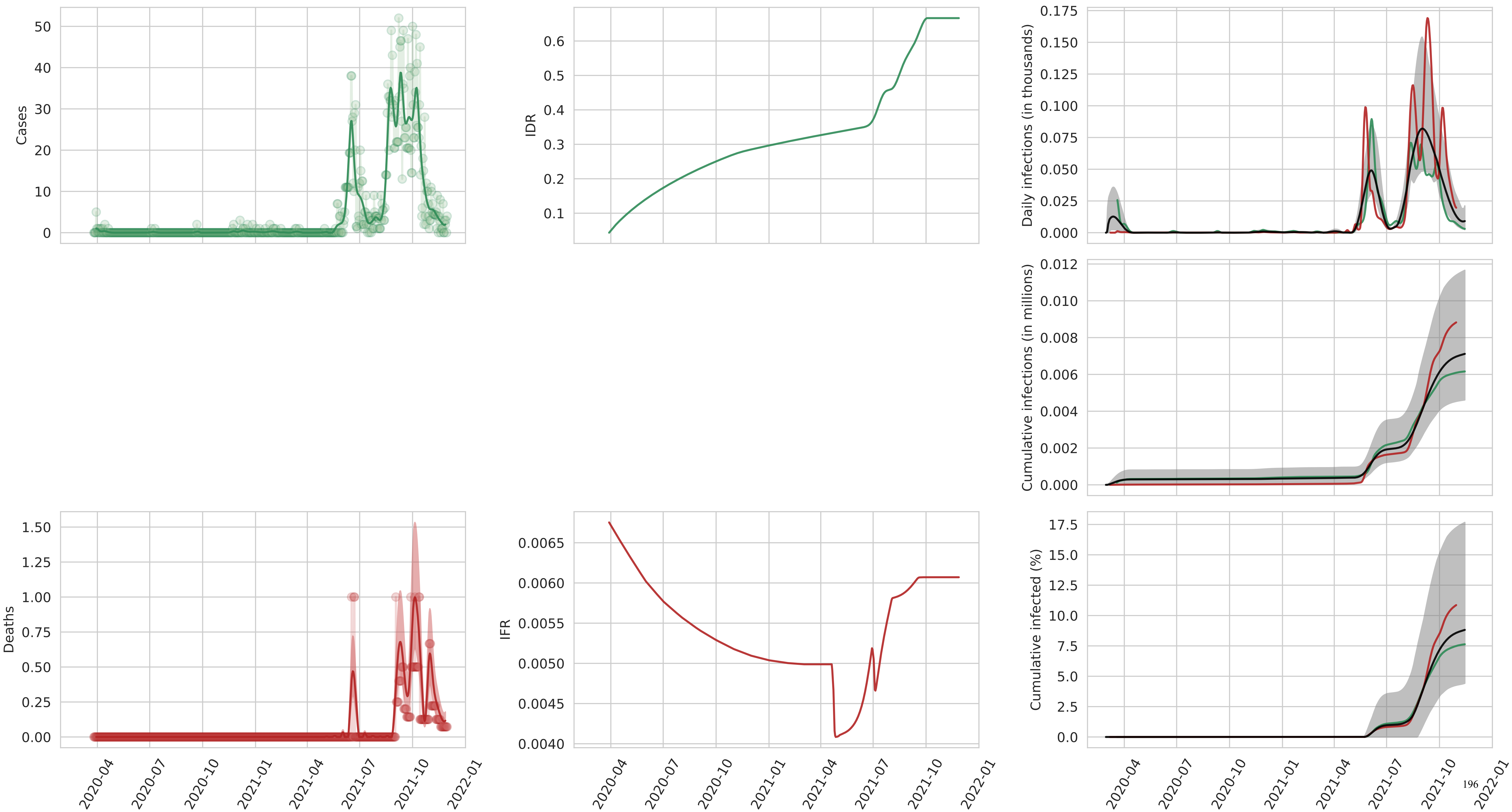

Saint Lucia

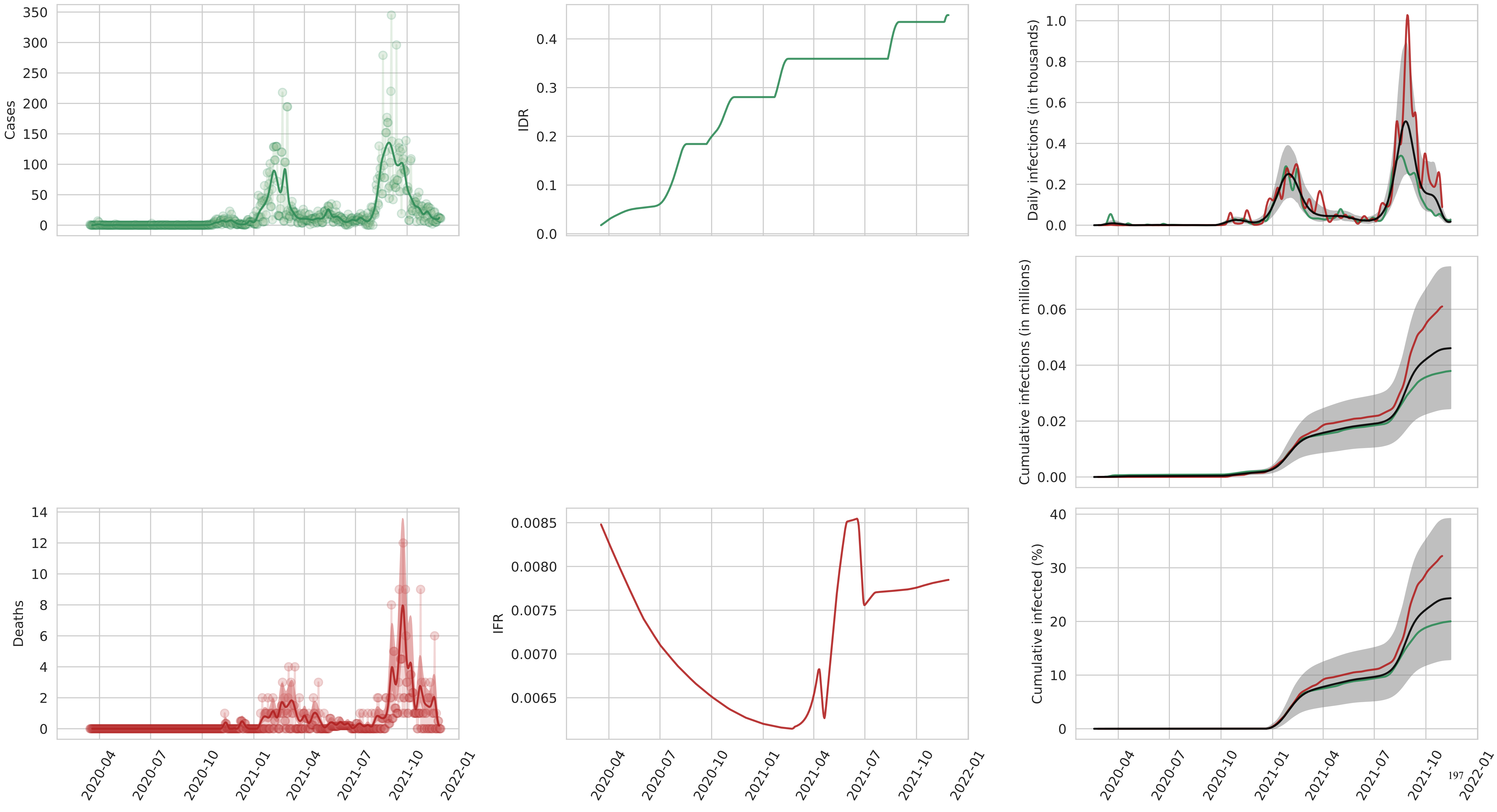

Cases
IDR
Daily infections (in thousands)
Cumulative infections (in millions)
Deaths
IFR
Cumulative infected (%)
2020-04
2020-07
2020-10
2021-01
2021-04
2021-07
2021-10
2022-01

Saint Vincent and the Grenadines

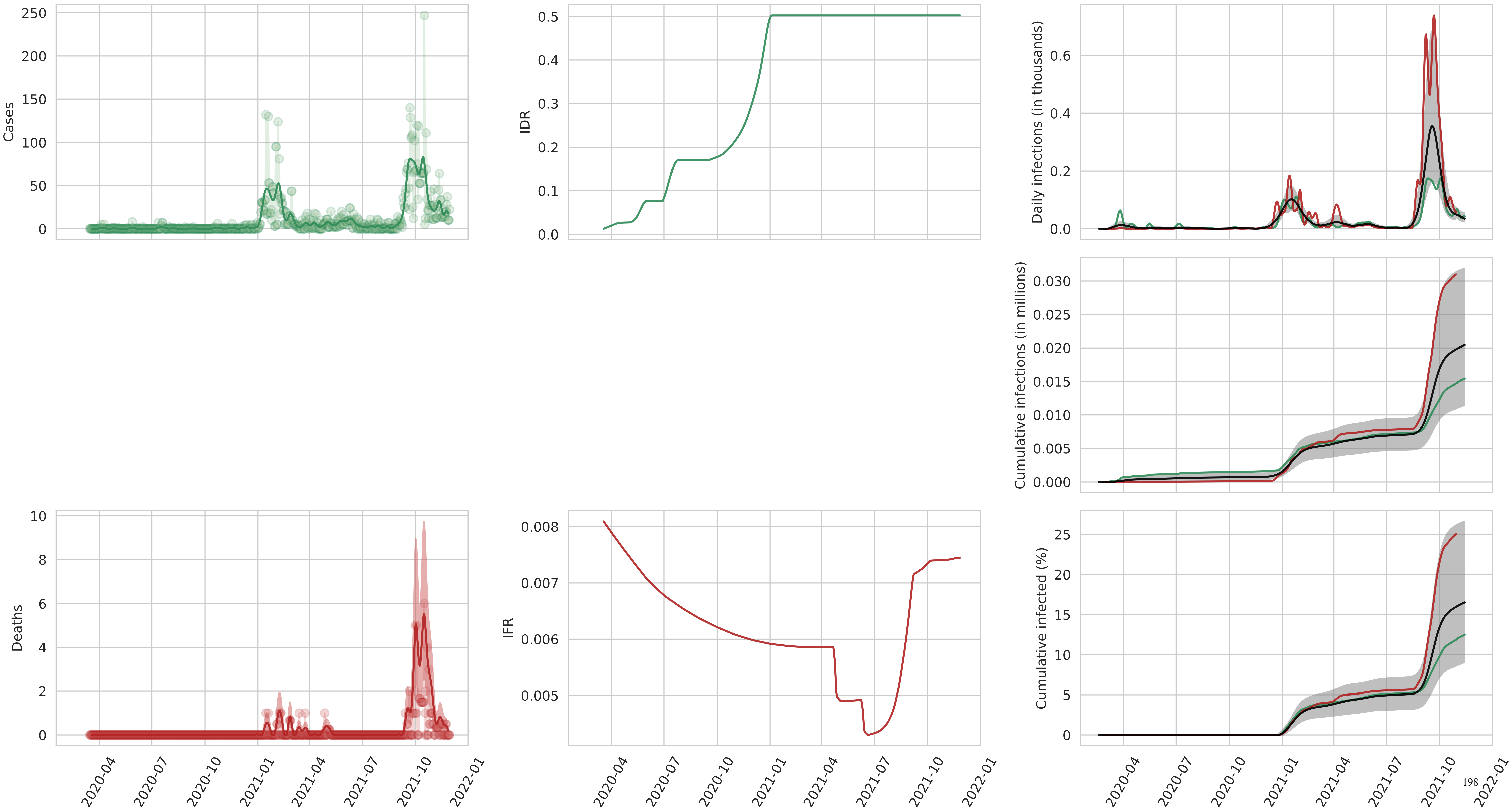

Suriname

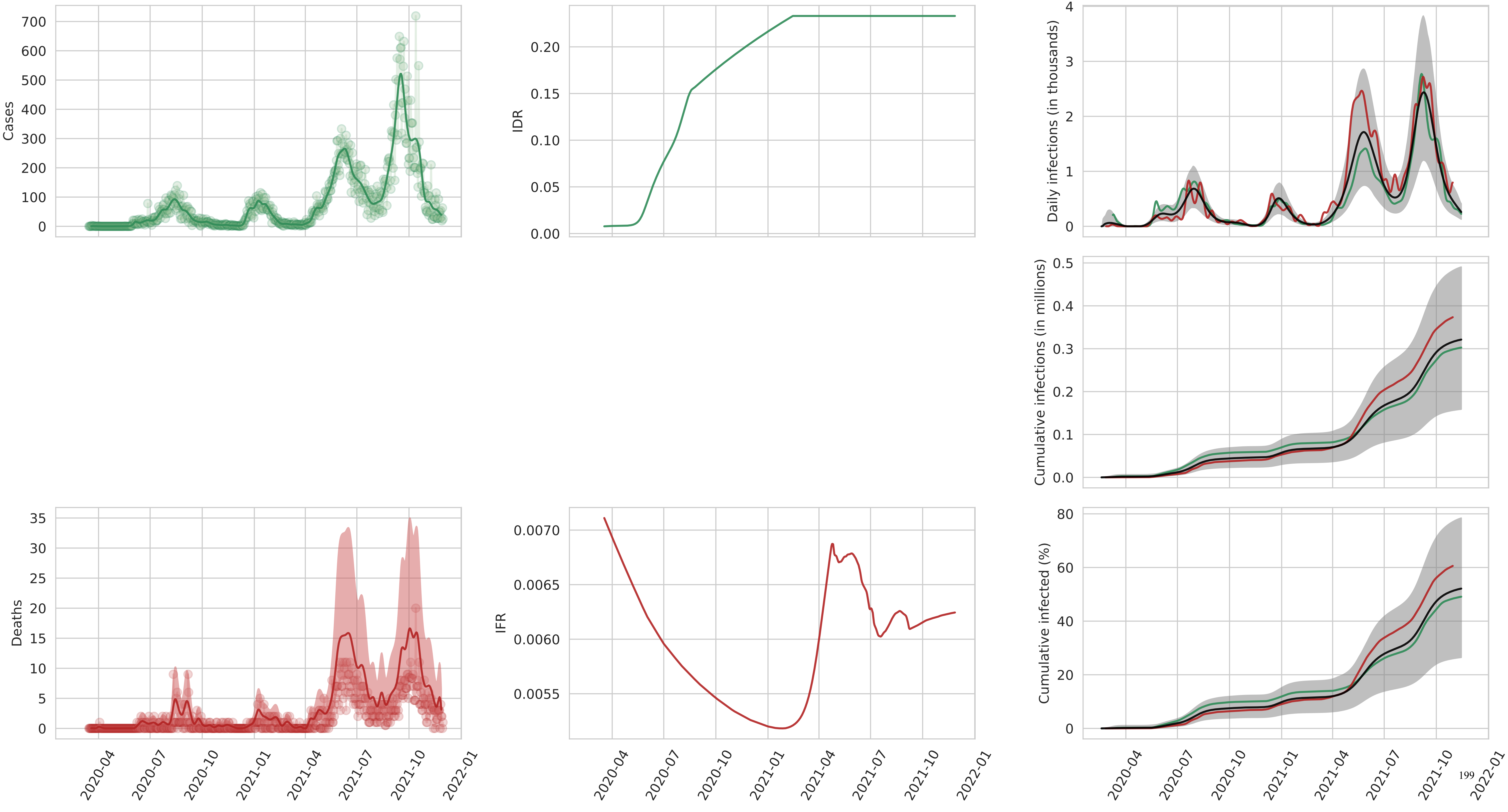
Cases
IDR
Daily infections (in thousands)
Cumulative infections (in millions)
Deaths
IFR
Cumulative infected (%)
2020-04
2020-07
2020-10
2021-01
2021-04
2021-07
2021-10
2022-01

Trinidad and Tobago

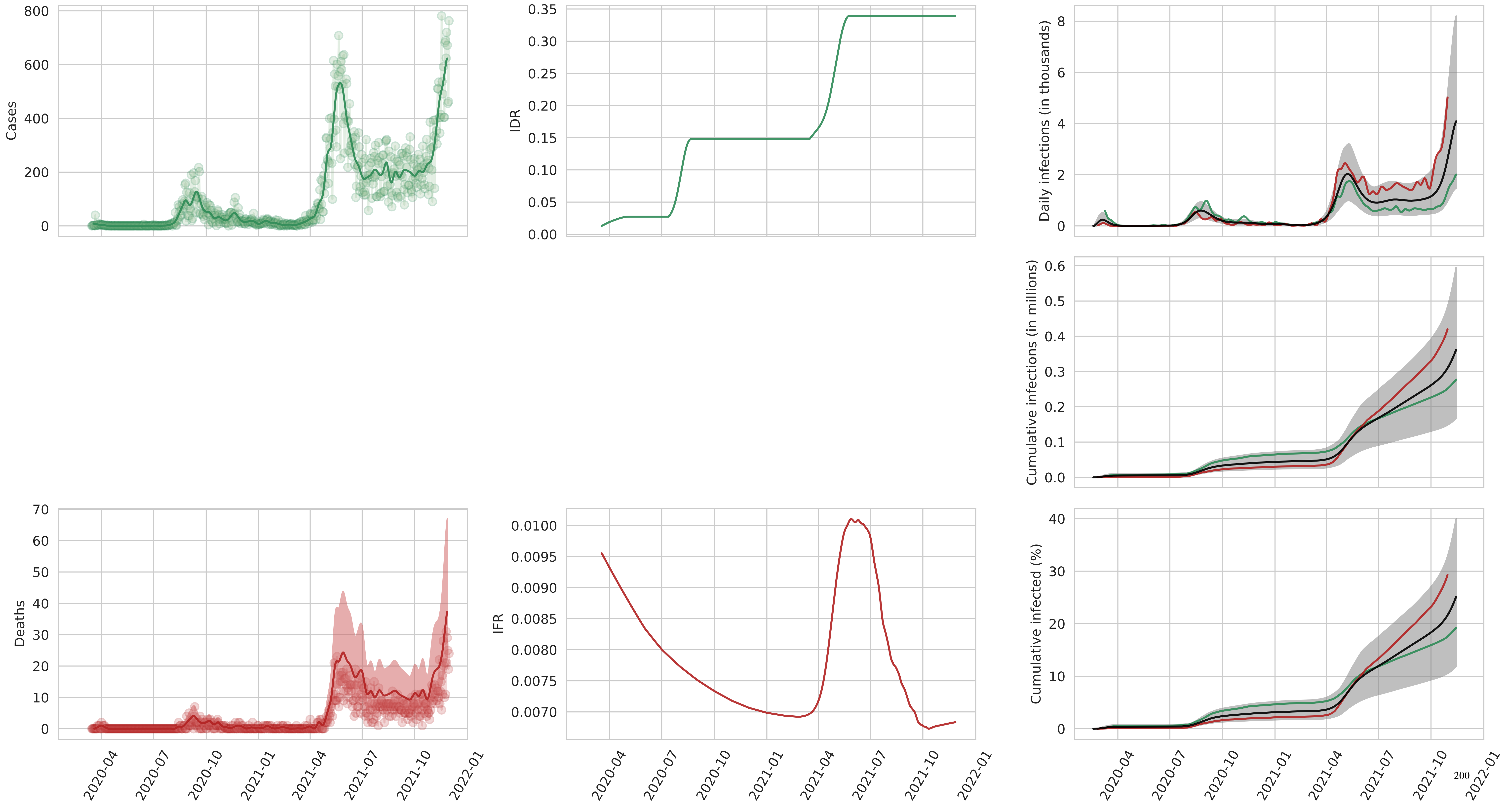

United States Virgin Islands

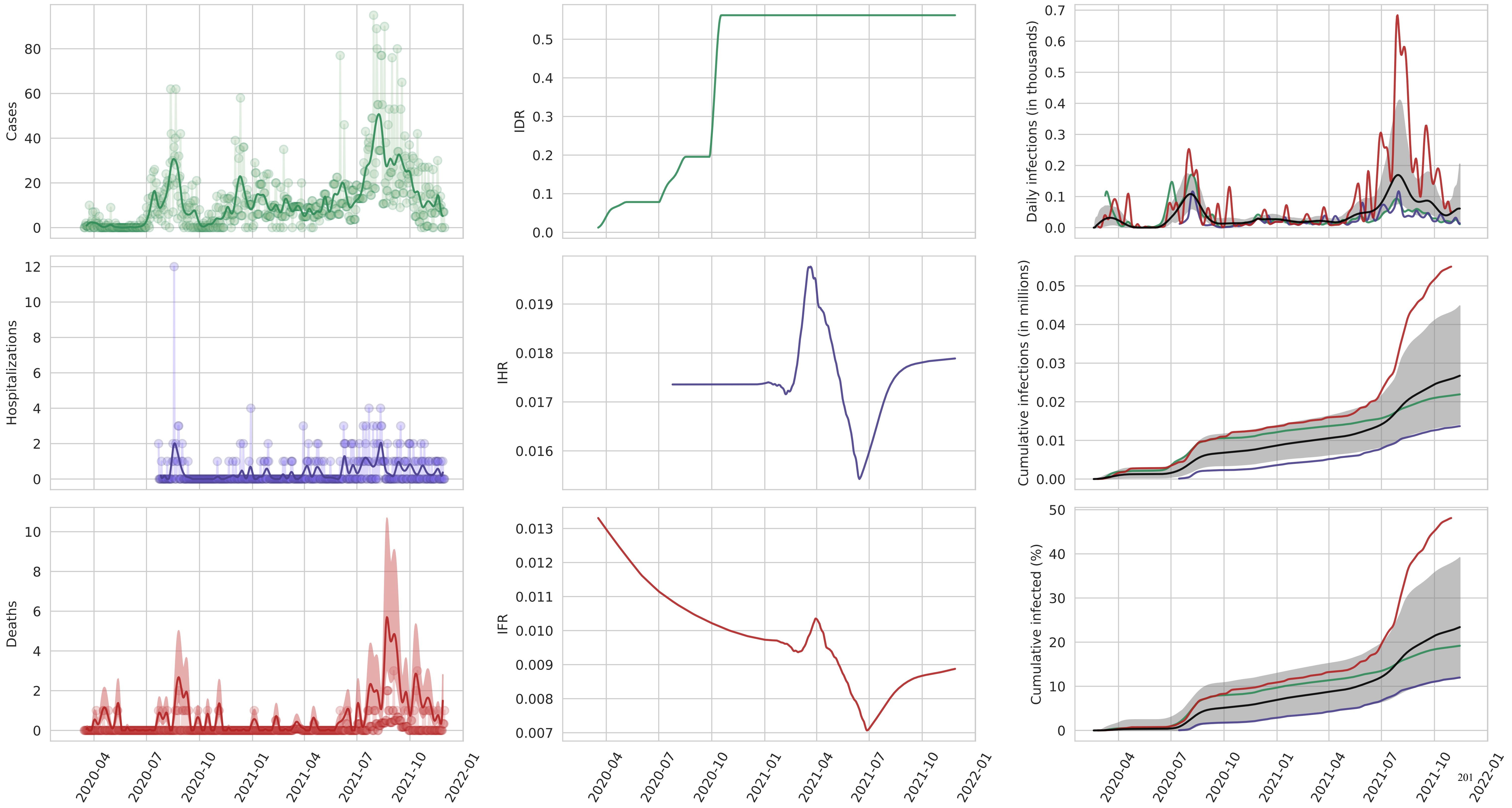

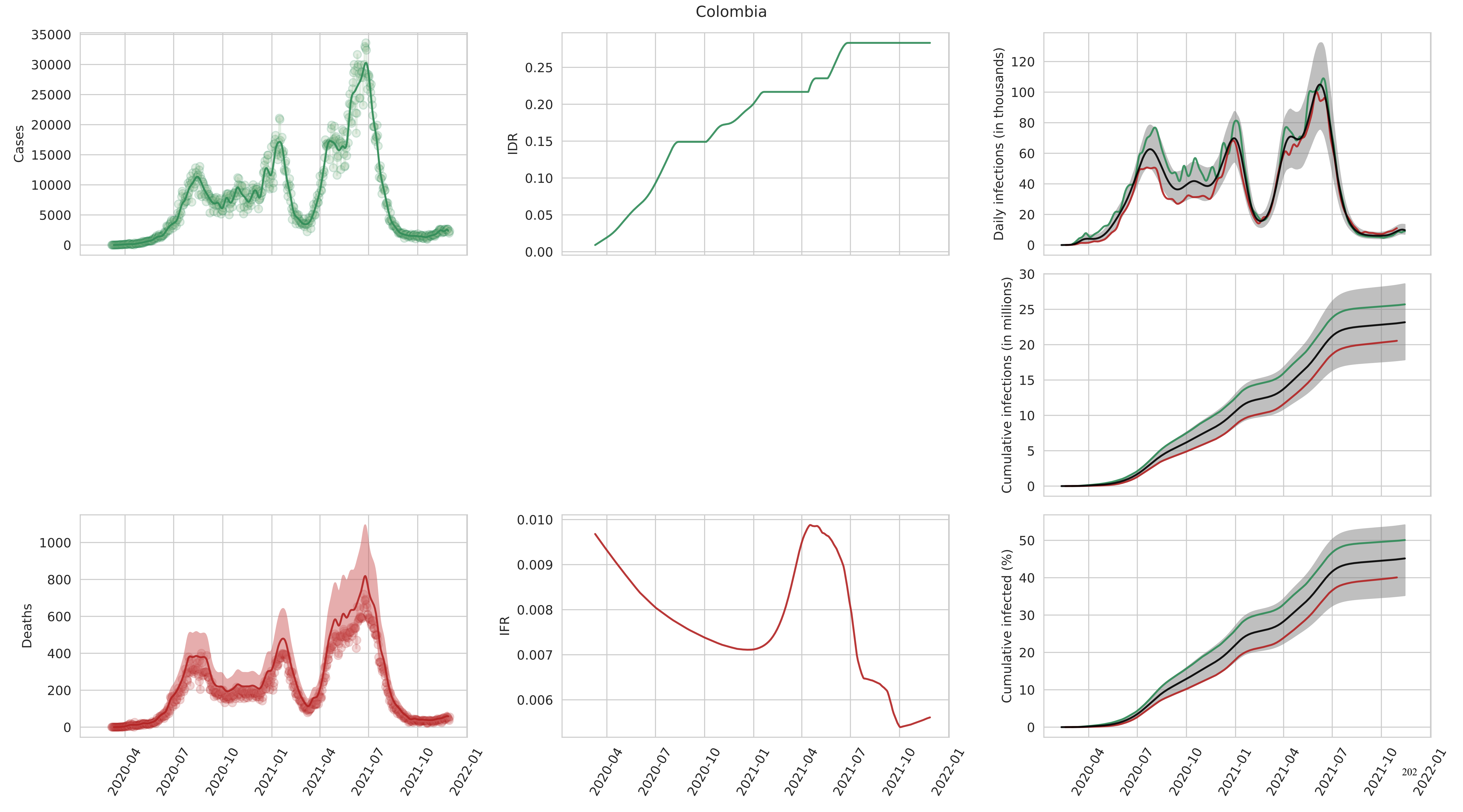

Colombia
Cases
IDR
Daily infections (in thousands)
Cumulative infections (in millions)
Deaths
IFR
Cumulative infected (%)
2020-04
2020-07
2020-10
2021-01
2021-04
2021-07
2021-10
2022-01
202

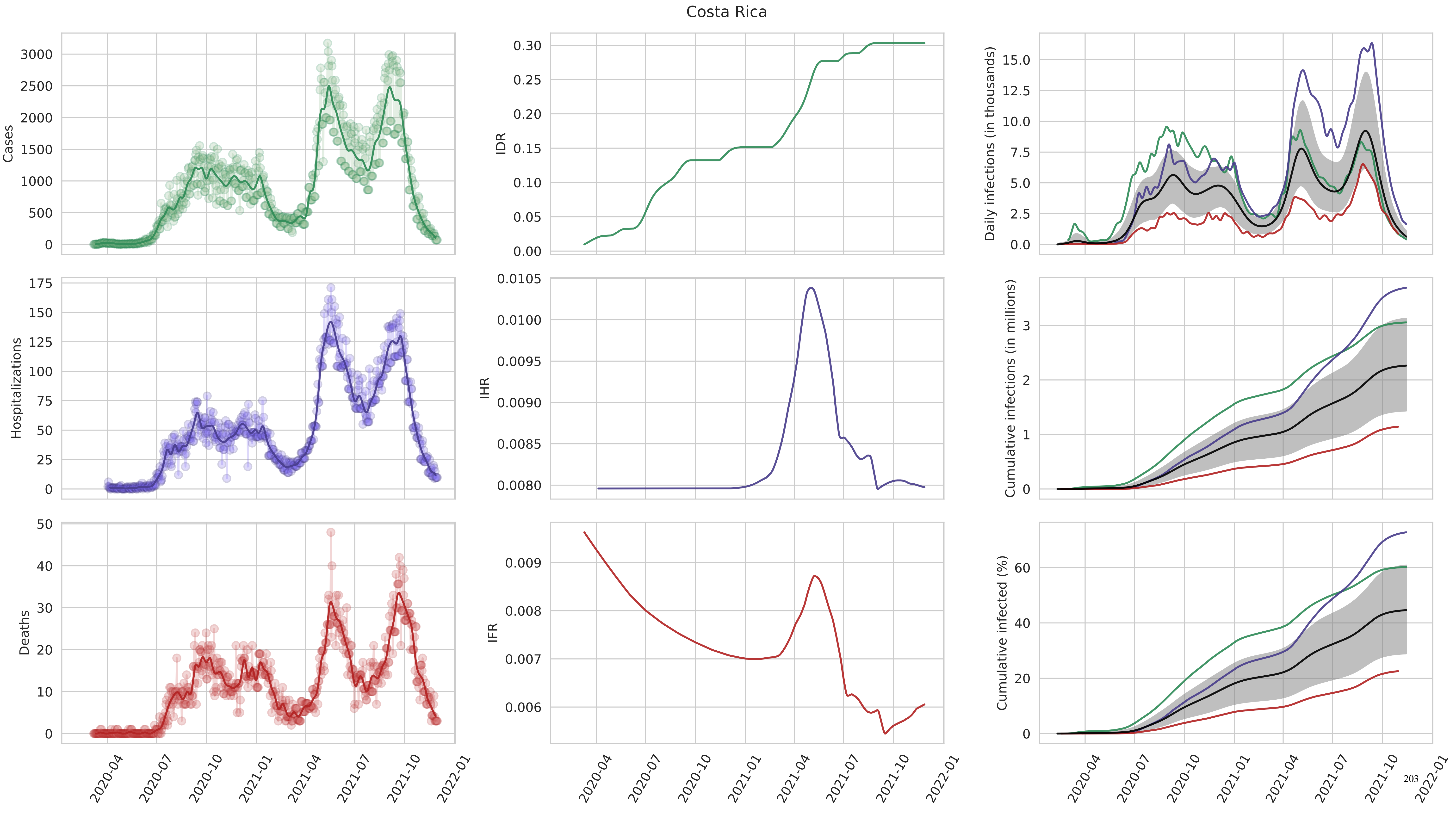
Costa Rica
Cases
IDR
Daily infections (in thousands)
Hospitalizations
IHR
Cumulative infections (in millions)
Deaths
IFR
Cumulative infected (%)
2020-04
2020-07
2020-10
2021-01
2021-04
2021-07
2021-10
2022-01

El Salvador

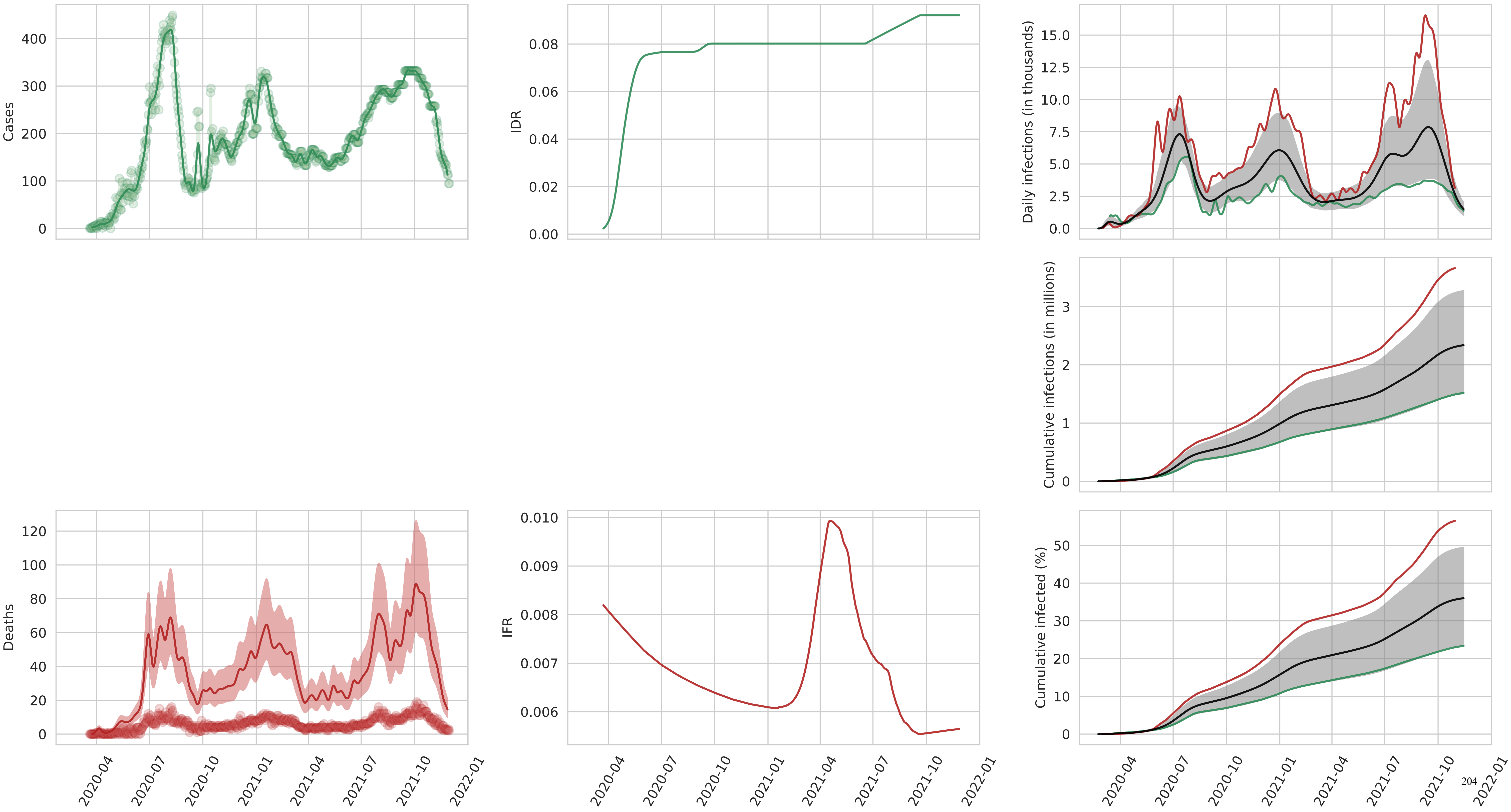

Cases
IDR
Daily infections (in thousands)
Cumulative infections (in millions)
Deaths
IFR
Cumulative infected (%)
2020-04
2020-07
2020-10
2021-01
2021-04
2021-07
2021-10
2022-01

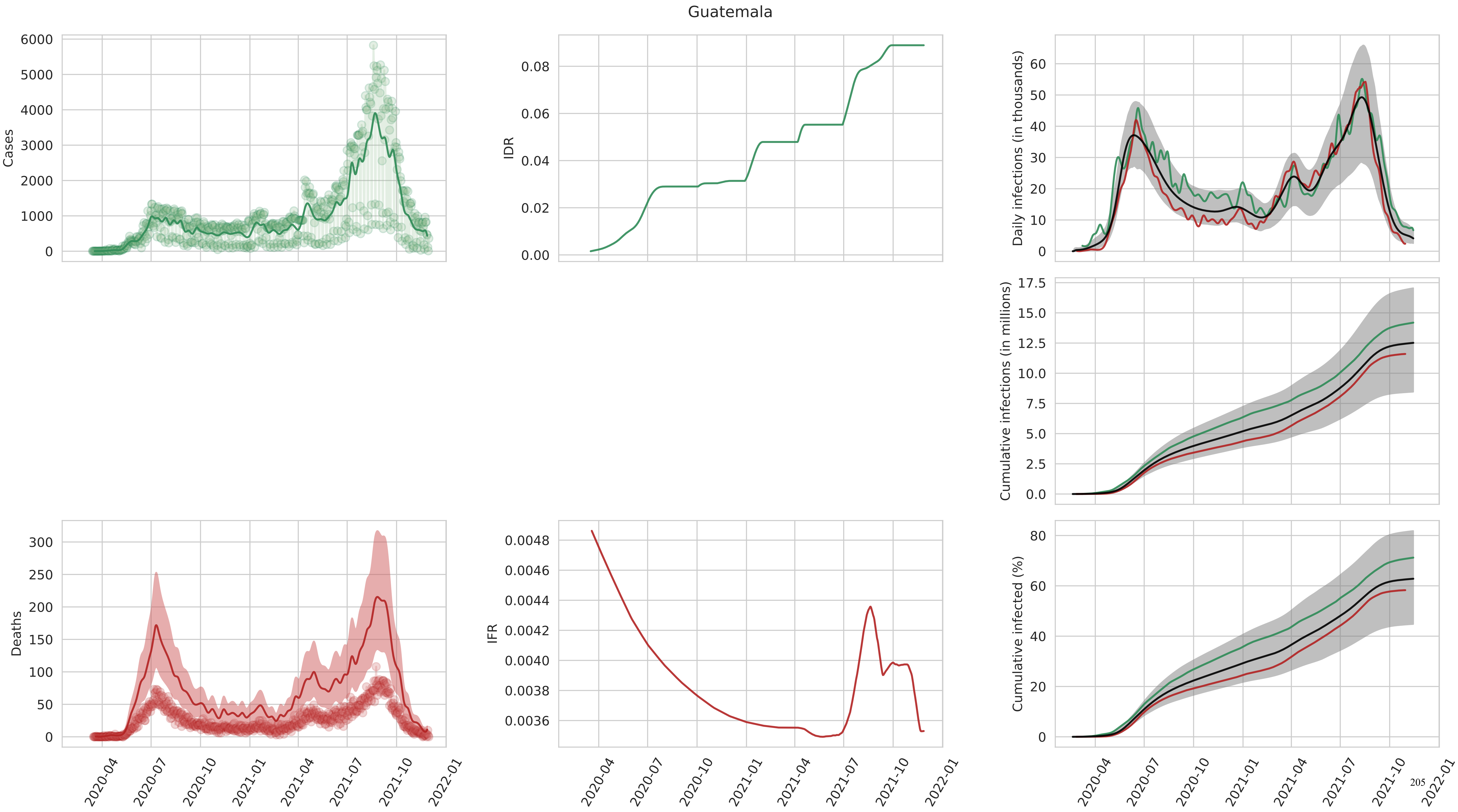
Guatemala
Cases
IDR
Daily infections (in thousands)
Cumulative infections (in millions)
Deaths
IFR
Cumulative infected (%)
2020-04
2020-07
2020-10
2021-01
2021-04
2021-07
2021-10
2022-01

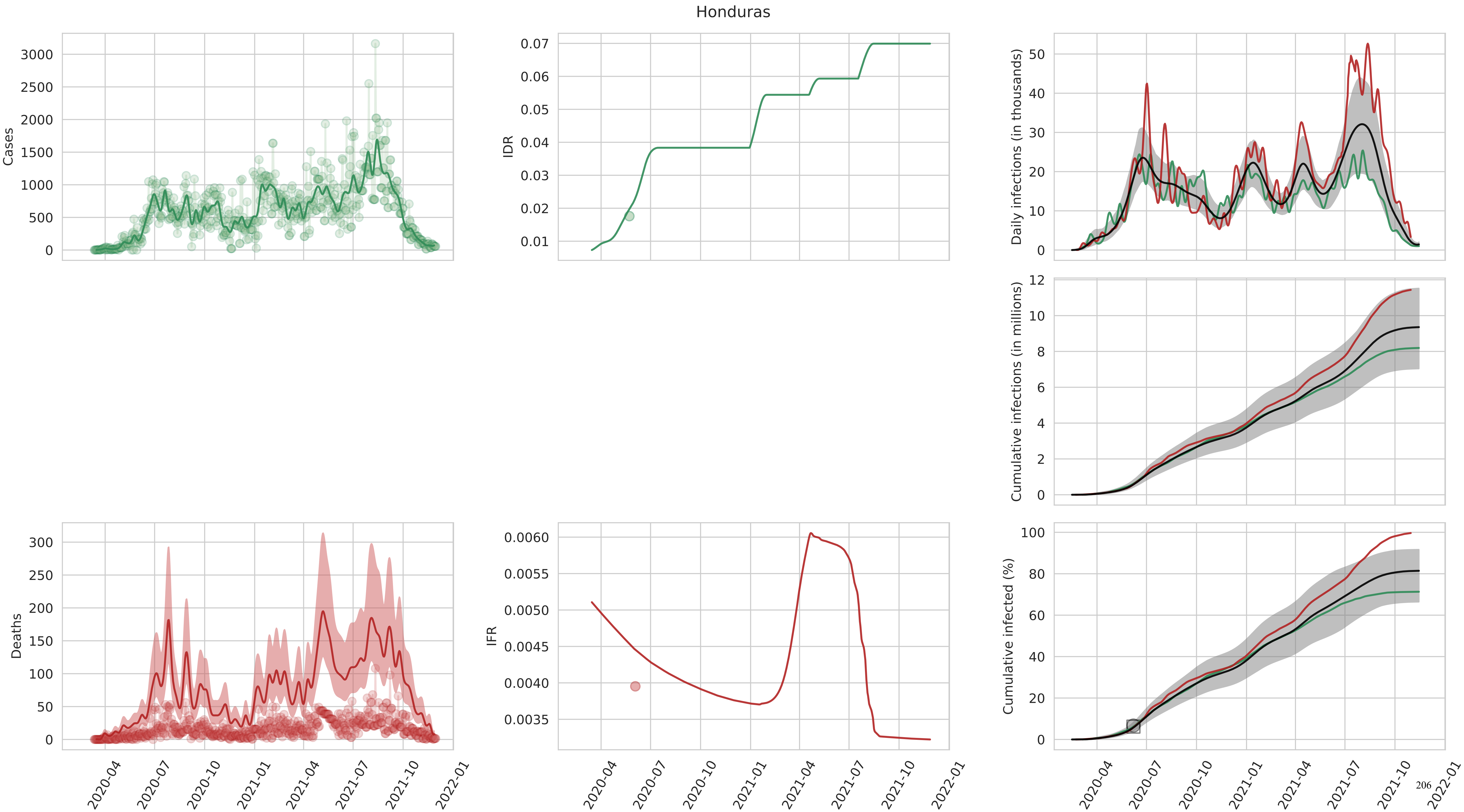
Honduras
Cases
IDR
Daily infections (in thousands)
Cumulative infections (in millions)
Deaths
IFR
Cumulative infected (%)

Aguascalientes

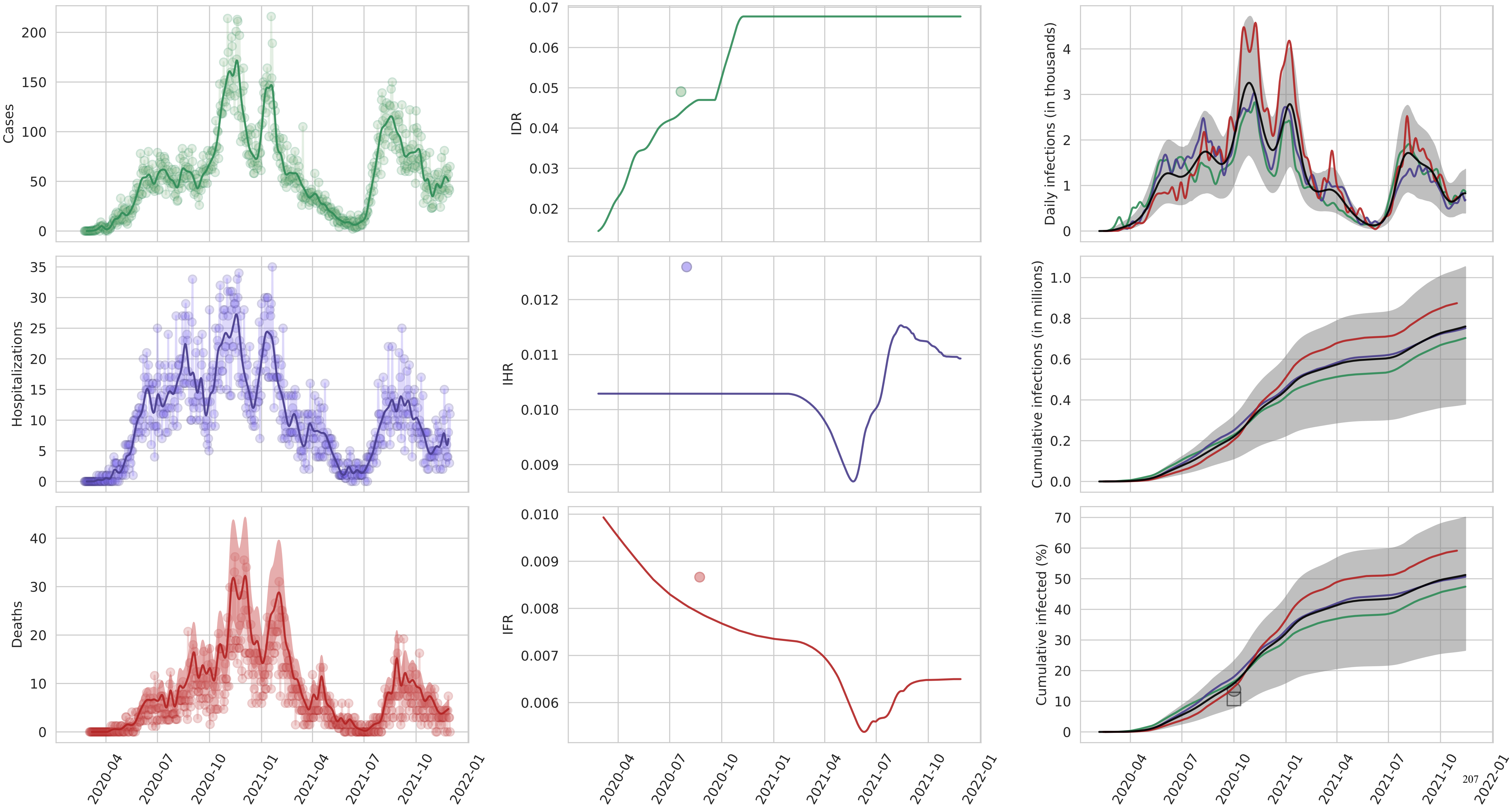

Baja California

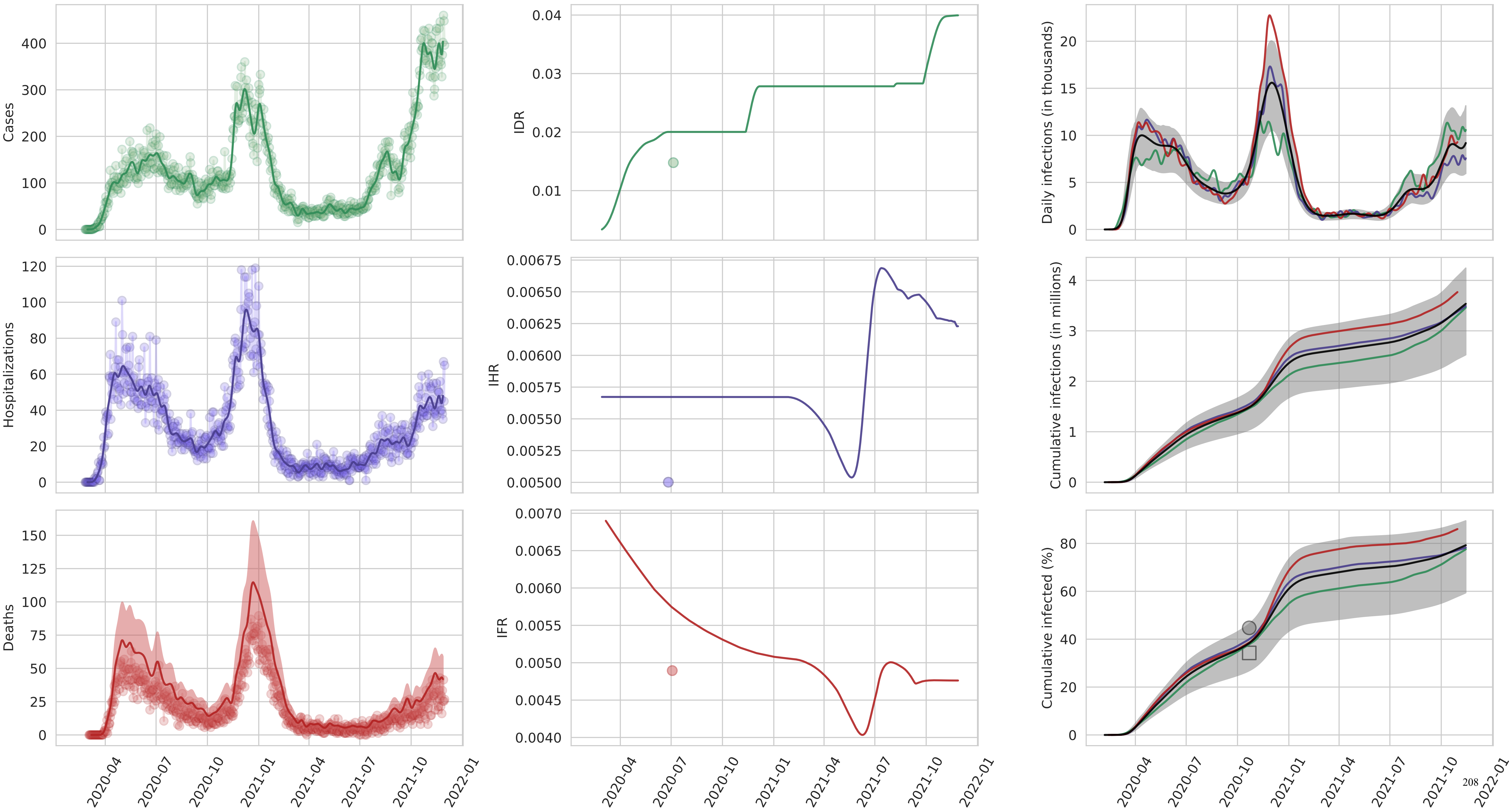

Baja California Sur

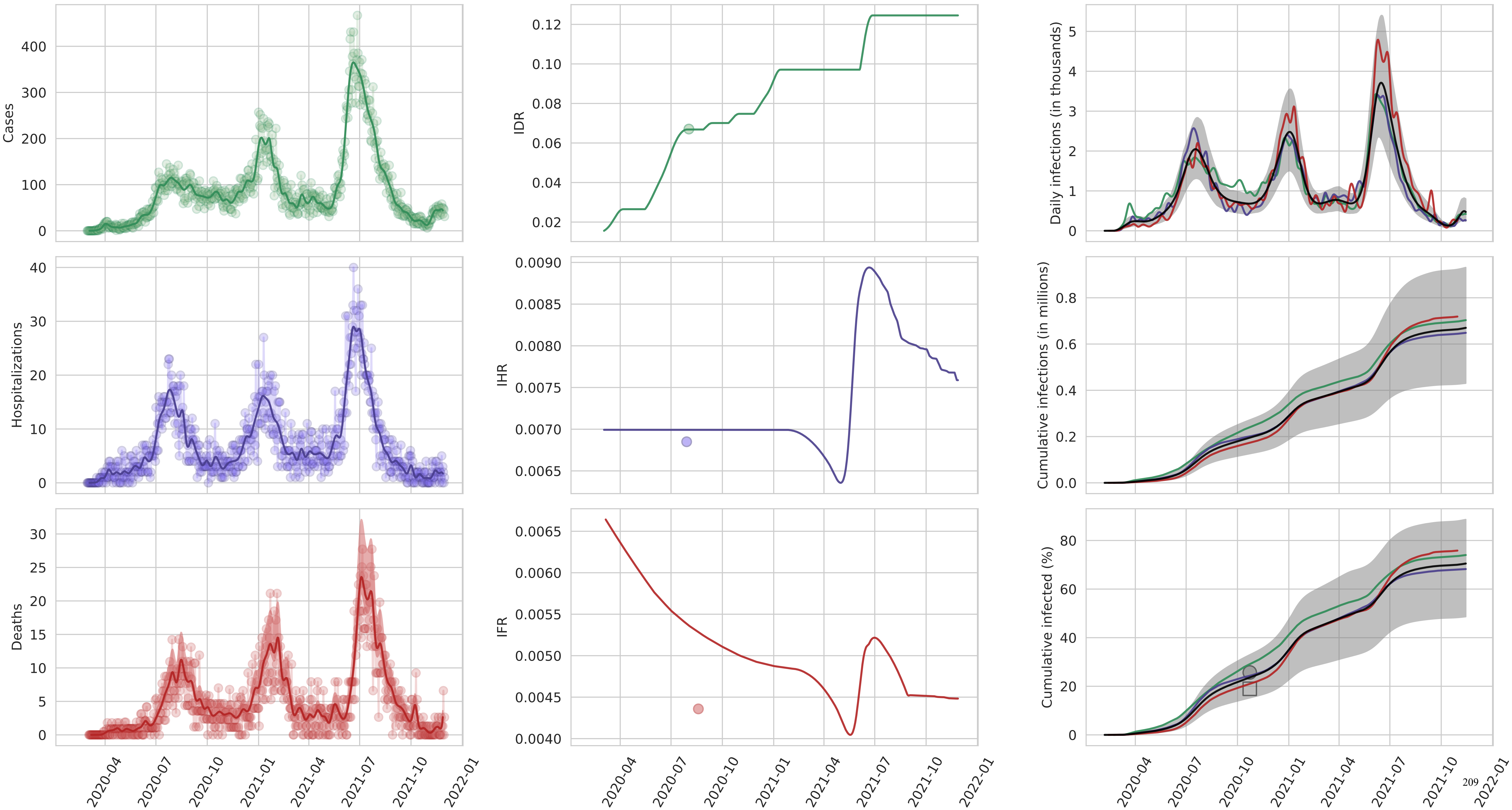

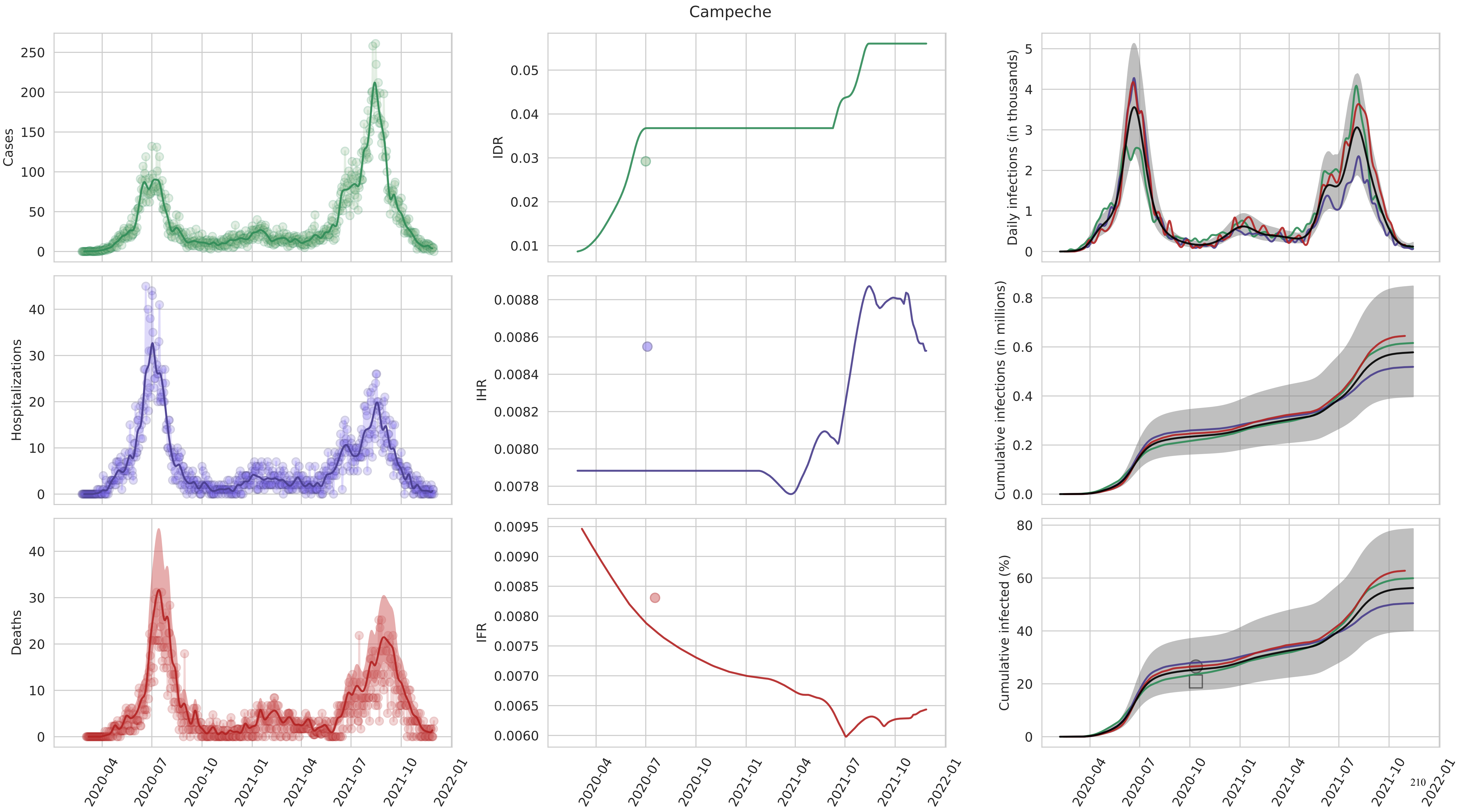
Campeche
Cases
IDR
Daily infections (in thousands)
Hospitalizations
IHR
Cumulative infections (in millions)
Deaths
IFR
Cumulative infected (%)
2020-04
2020-07
2020-10
2021-01
2021-04
2021-07
2021-10
2022-01

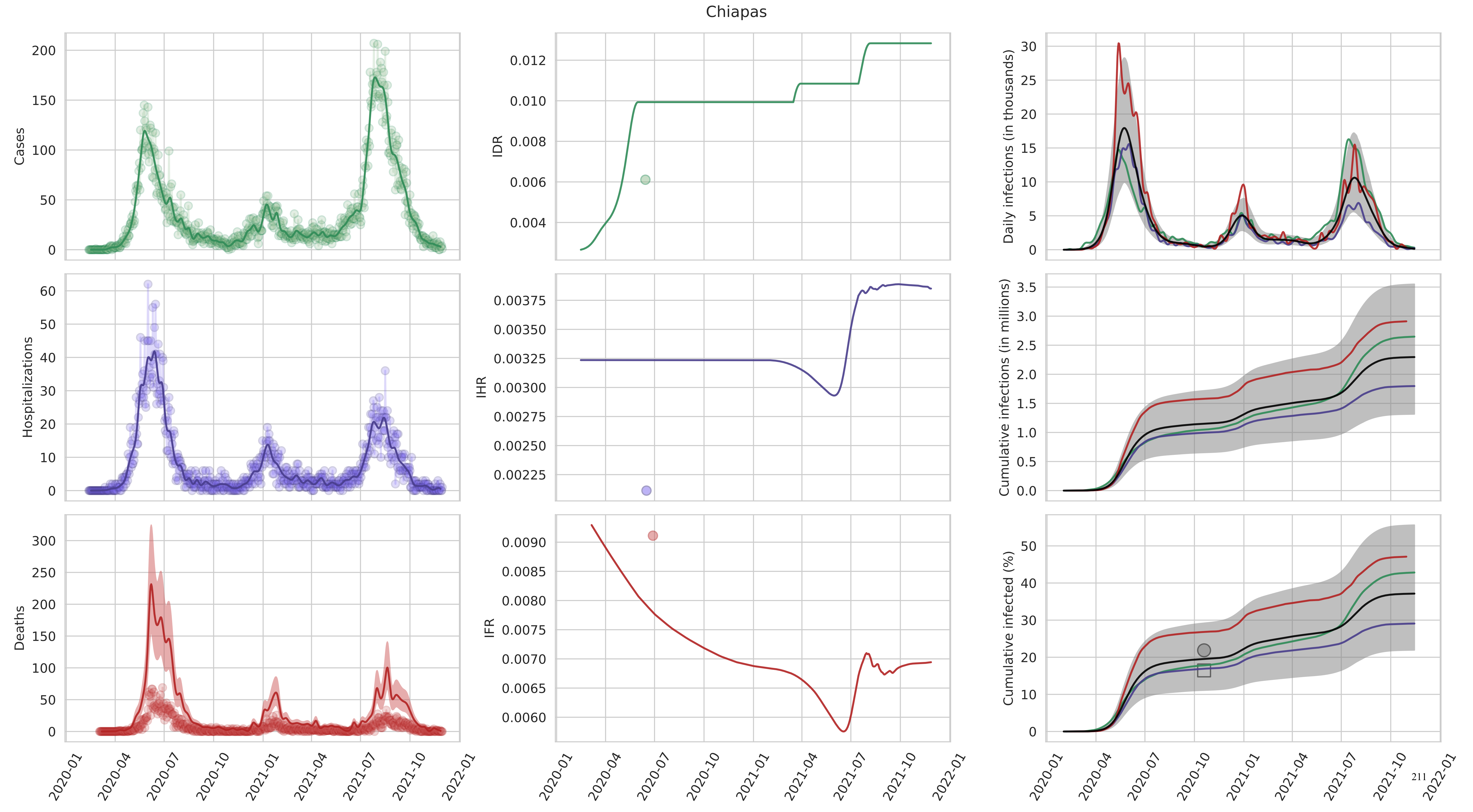
Chiapas
Cases
IDR
Daily infections (in thousands)
Hospitalizations
IHR
Cumulative infections (in millions)
Deaths
IFR
Cumulative infected (%)
2020-01
2020-04
2020-07
2020-10
2021-01
2021-04
2021-07
2021-10
2022-01

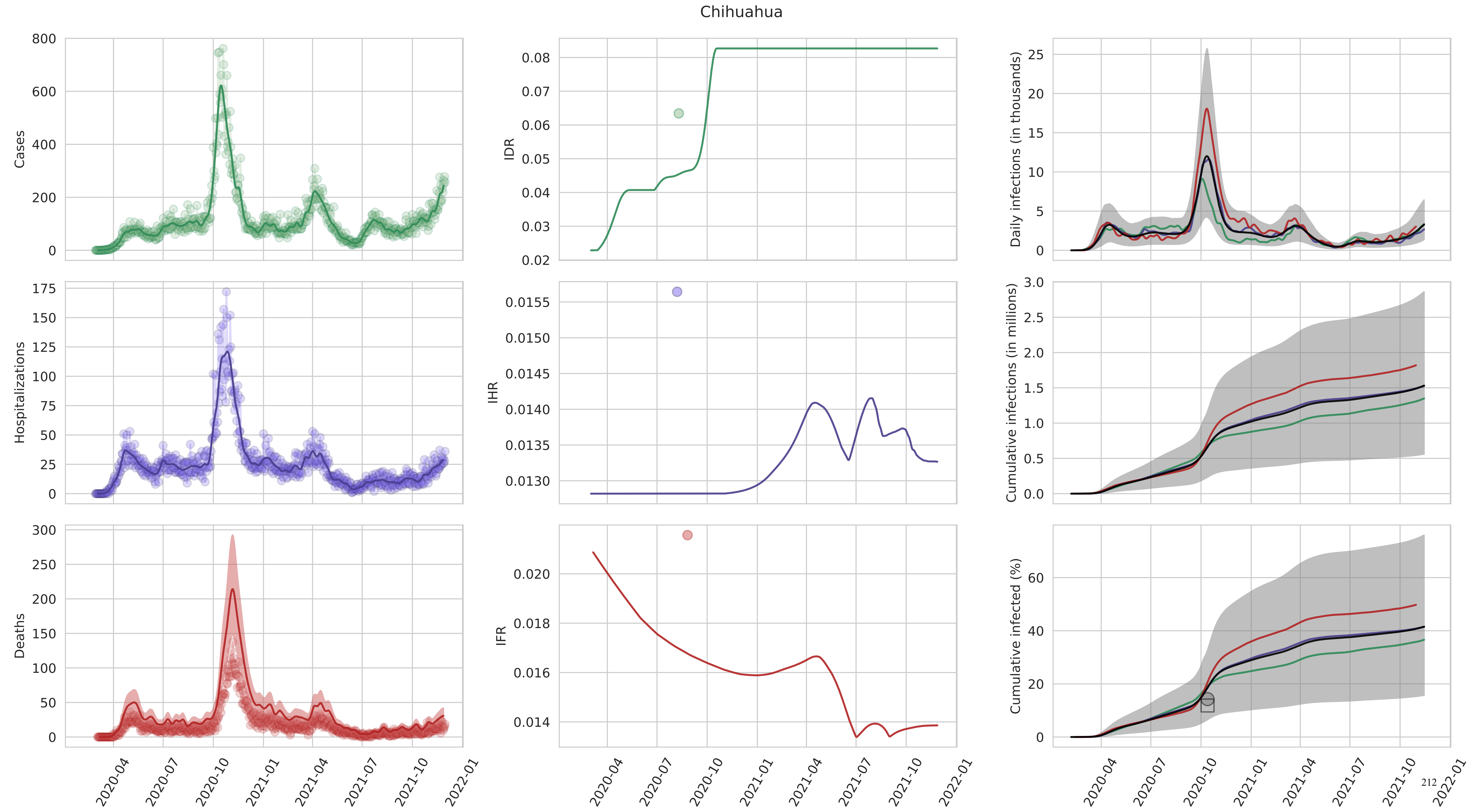
Chihuahua
Cases
Hospitalizations
Deaths
IDR
IHR
IFR
Daily infections (in thousands)
Cumulative infections (in millions)
Cumulative infected (%)
2020-04
2020-07
2020-10
2021-01
2021-04
2021-07
2021-10
2022-01

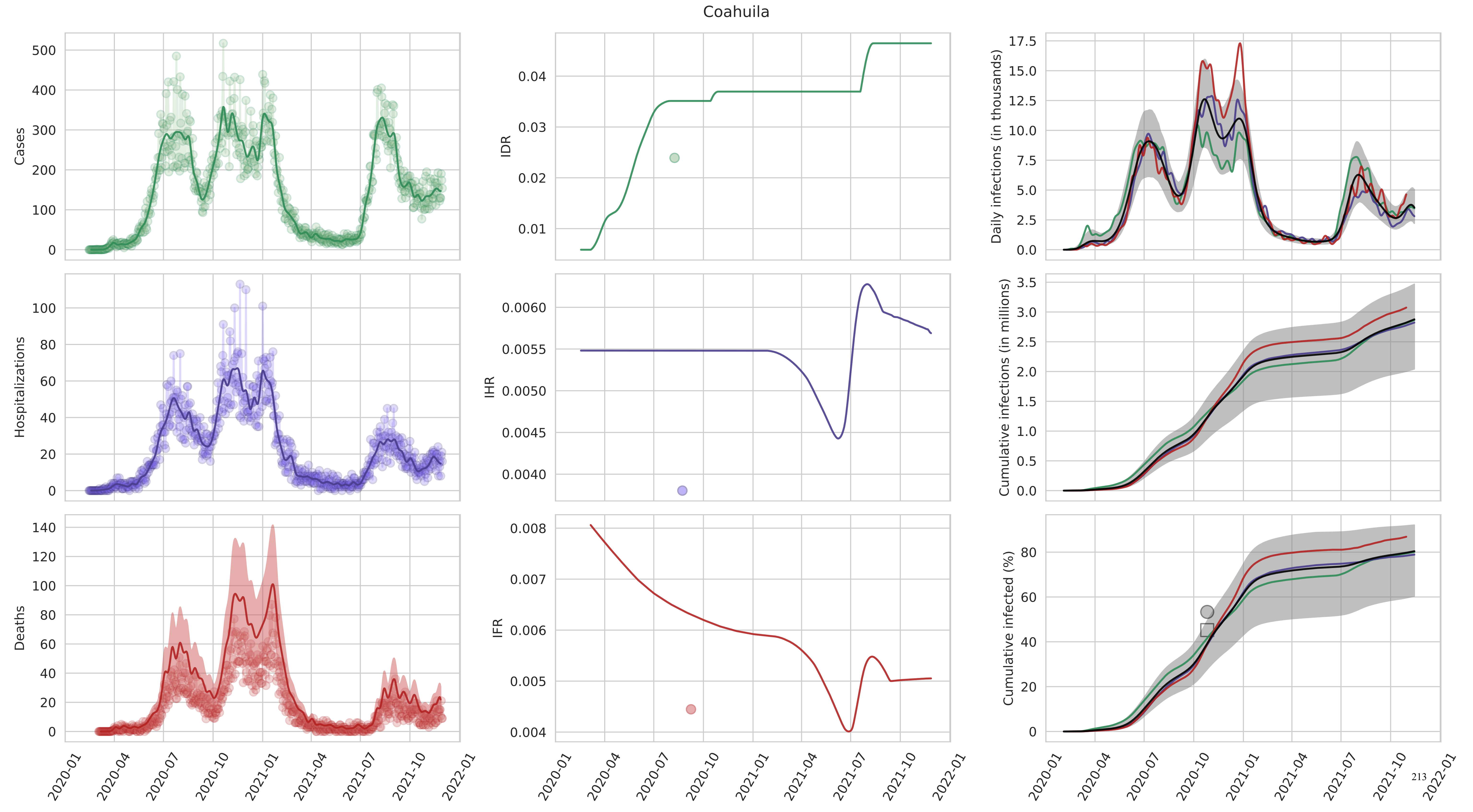
Coahuila
Cases
IDR
Daily infections (in thousands)
Hospitalizations
IHR
Cumulative infections (in millions)
Deaths
IFR
Cumulative infected (%)
2020-01
2020-04
2020-07
2020-10
2021-01
2021-04
2021-07
2021-10
2022-01

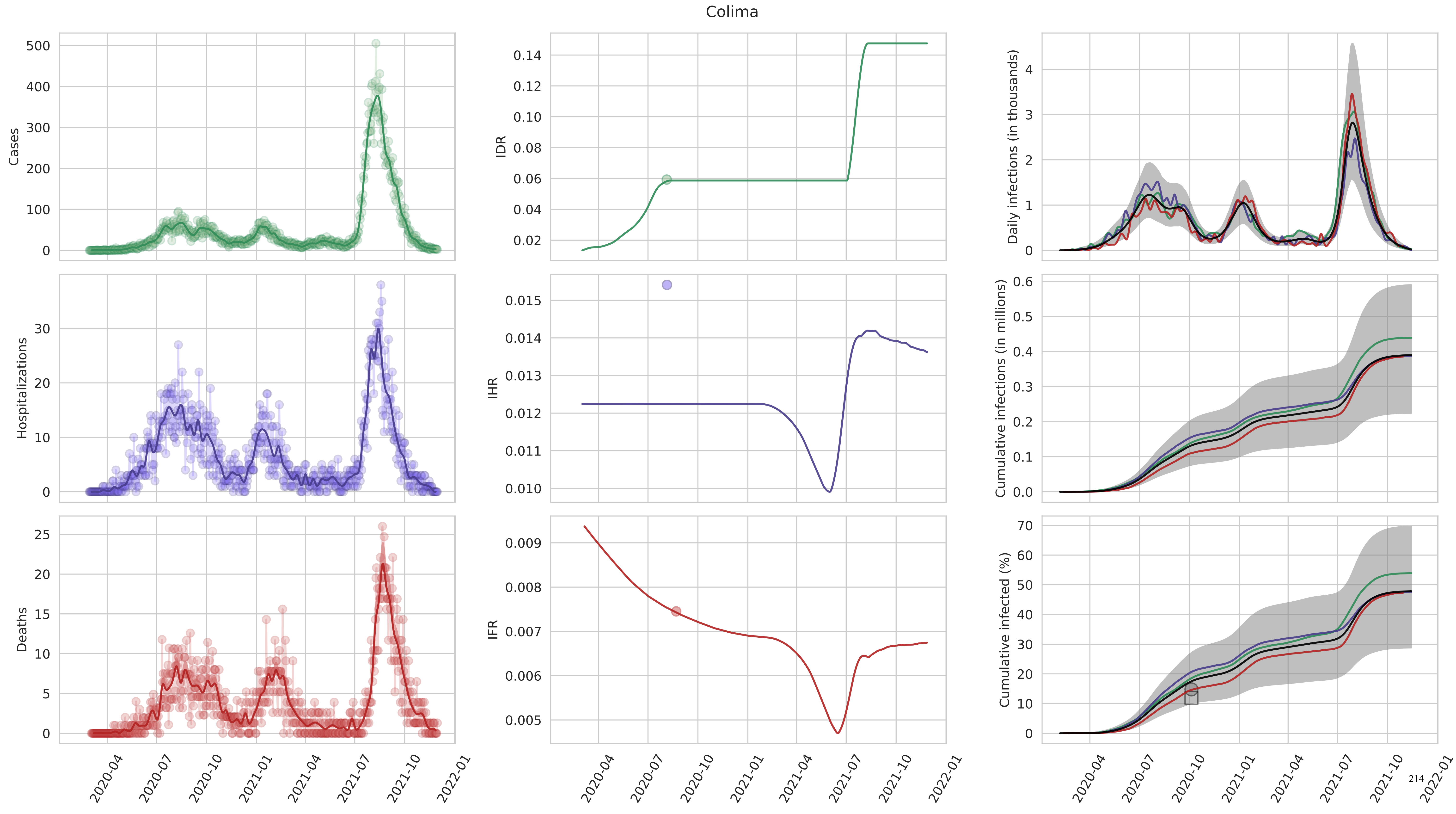
Colima
Cases
IDR
Daily infections (in thousands)
Hospitalizations
IHR
Cumulative infections (in millions)
Deaths
IFR
Cumulative infected (%)
2020-04
2020-07
2020-10
2021-01
2021-04
2021-07
2021-10
2022-01

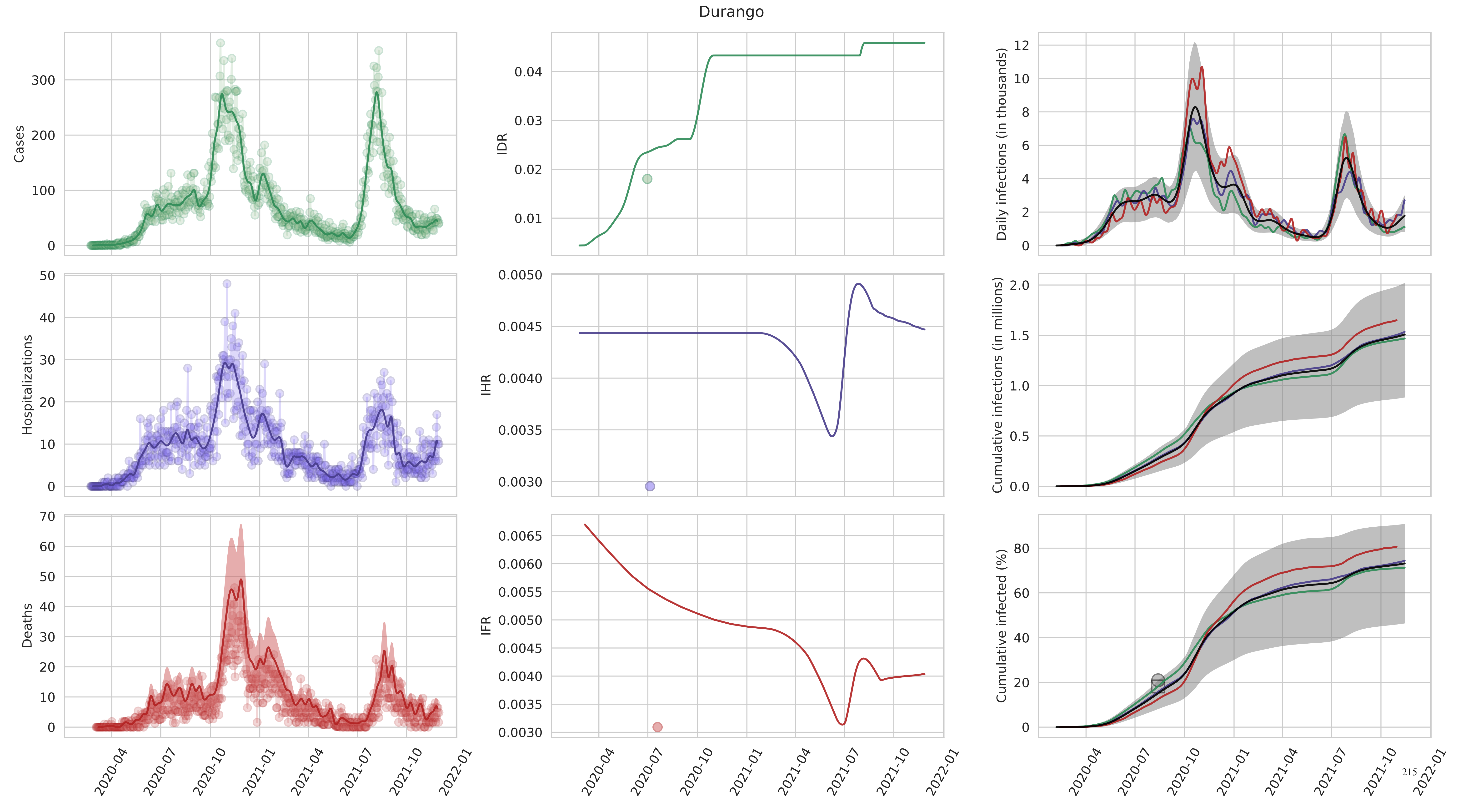

Durango
Cases
IDR
Daily infections (in thousands)
Hospitalizations
IHR
Cumulative infections (in millions)
Deaths
IFR
Cumulative infected (%)
2020-04
2020-07
2020-10
2021-01
2021-04
2021-07
2021-10
2022-01

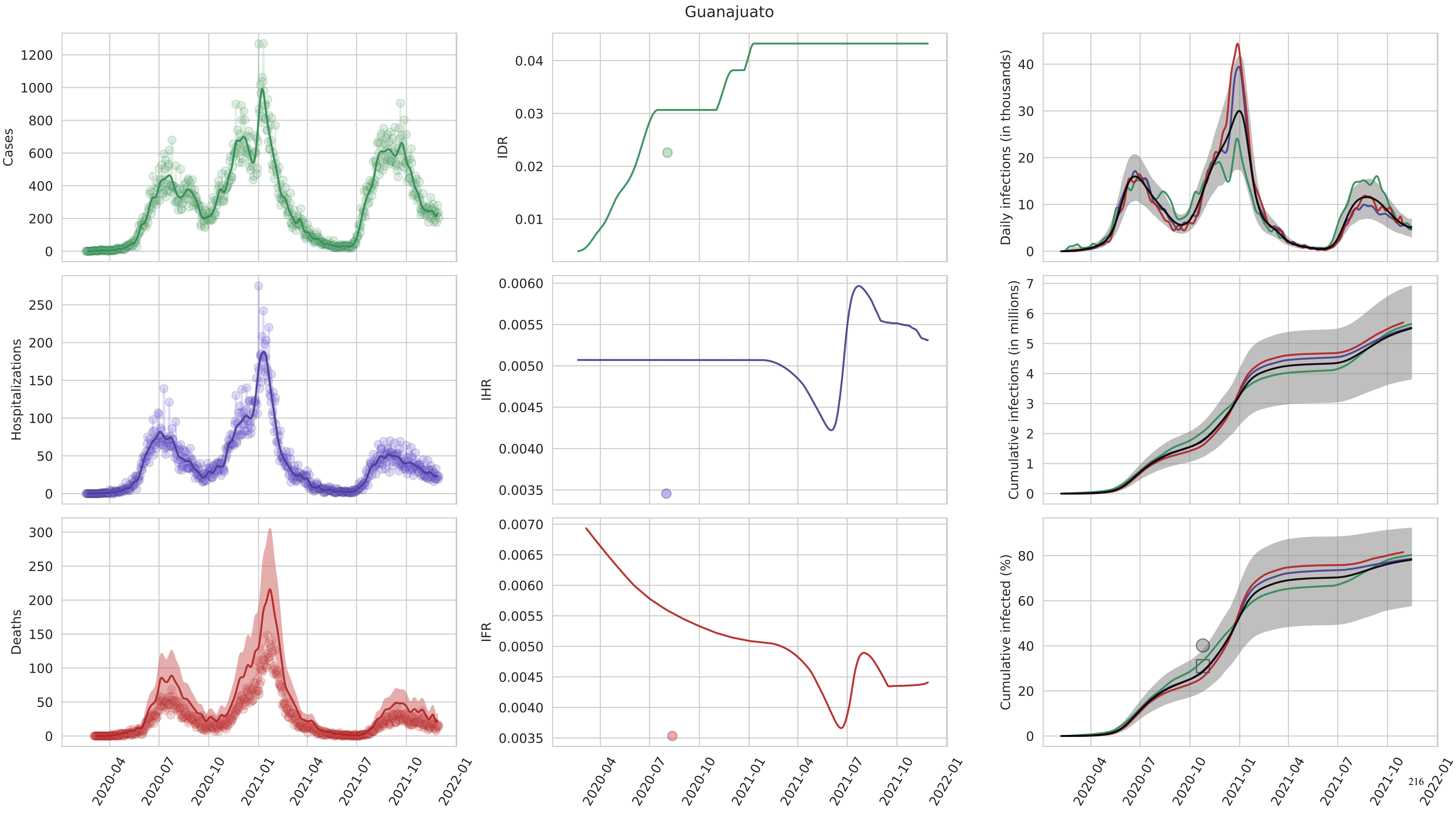
Guanajuato
Cases
IDR
Daily infections (in thousands)
Hospitalizations
IHR
Cumulative infections (in millions)
Deaths
IFR
Cumulative infected (%)
2020-04
2020-07
2020-10
2021-01
2021-04
2021-07
2021-10
2022-01

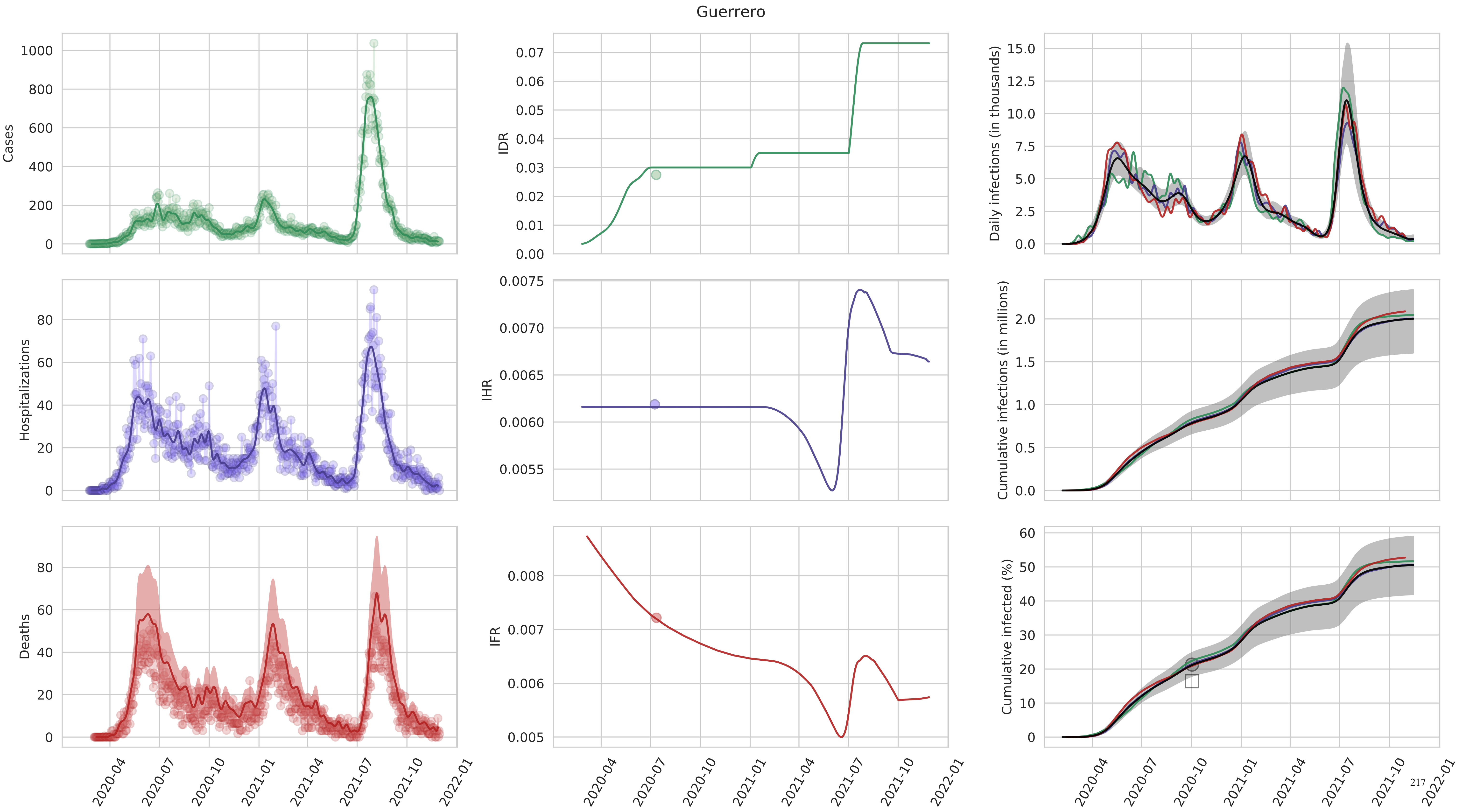

Guerrero
Cases
Hospitalizations
Deaths
IDR
IHR
IFR
Daily infections (in thousands)
Cumulative infections (in millions)
Cumulative infected (%)
2020-04
2020-07
2020-10
2021-01
2021-04
2021-07
2021-10
2022-01

Hidalgo

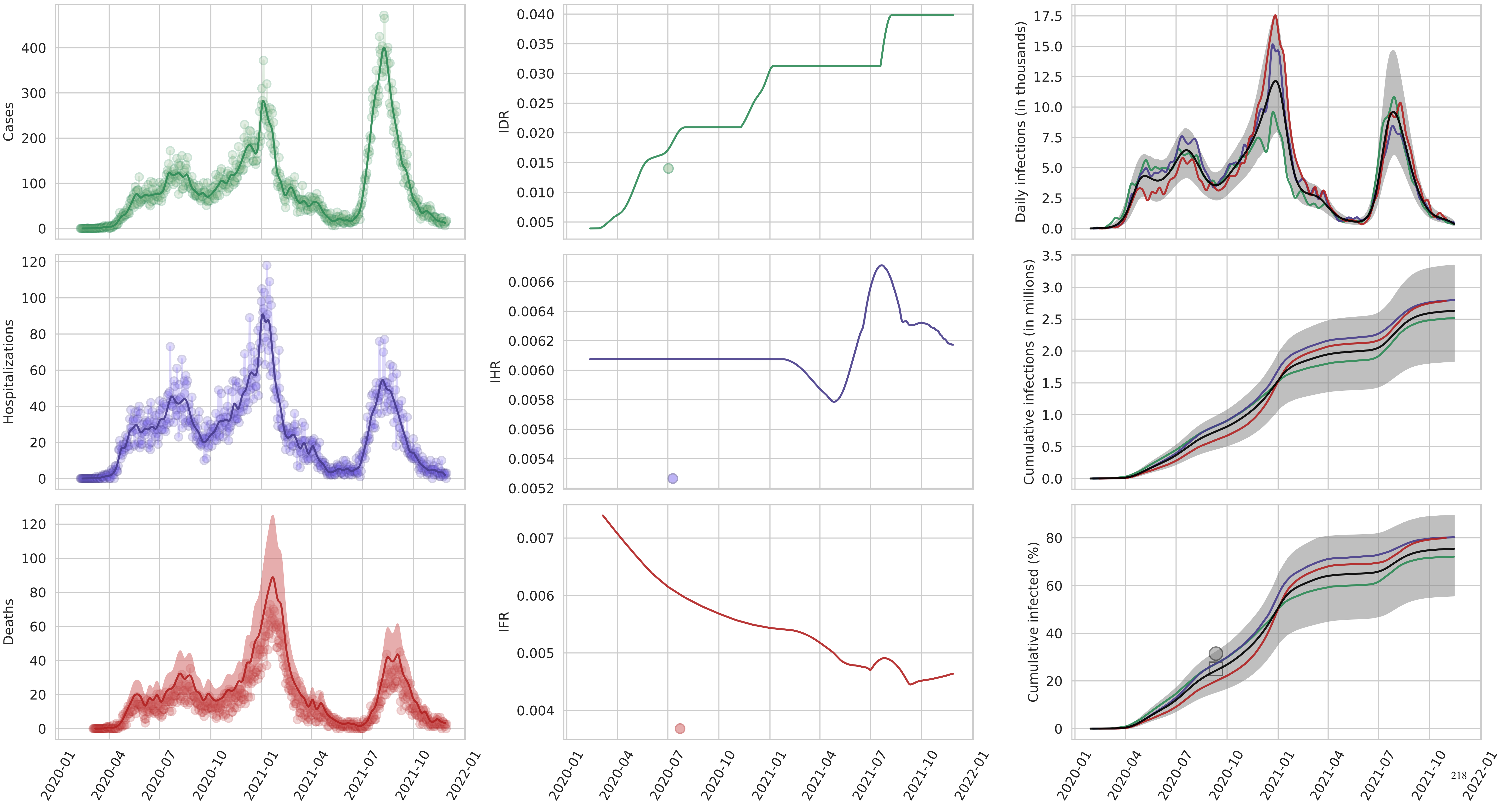
Cases
Hospitalizations
Deaths
IDR
IHR
IFR
Daily infections (in thousands)
Cumulative infections (in millions)
Cumulative infected (%)

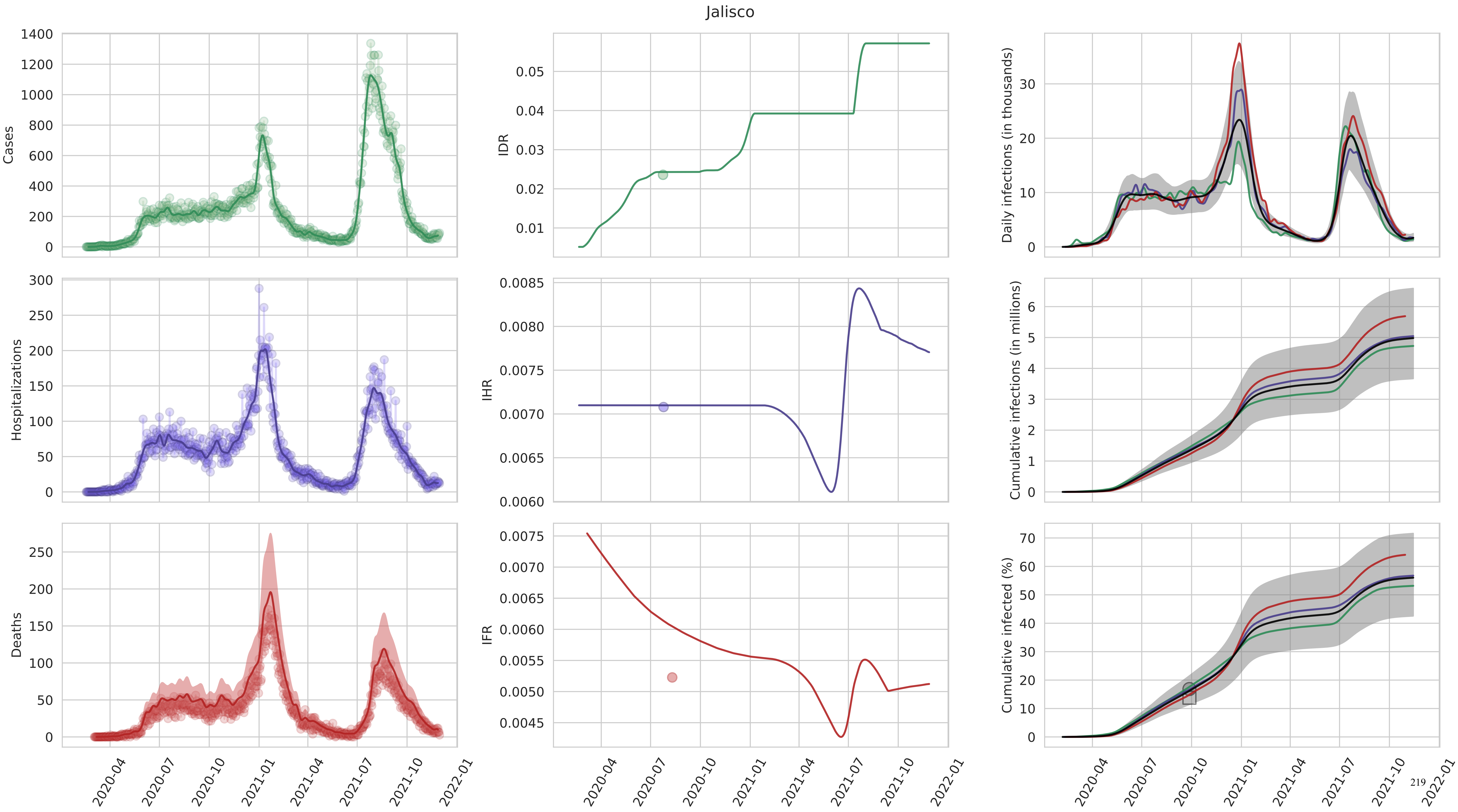

Jalisco
Cases
IDR
Daily infections (in thousands)
Hospitalizations
IHR
Cumulative infections (in millions)
Deaths
IFR
Cumulative infected (%)
2020-04
2020-07
2020-10
2021-01
2021-04
2021-07
2021-10
2022-01

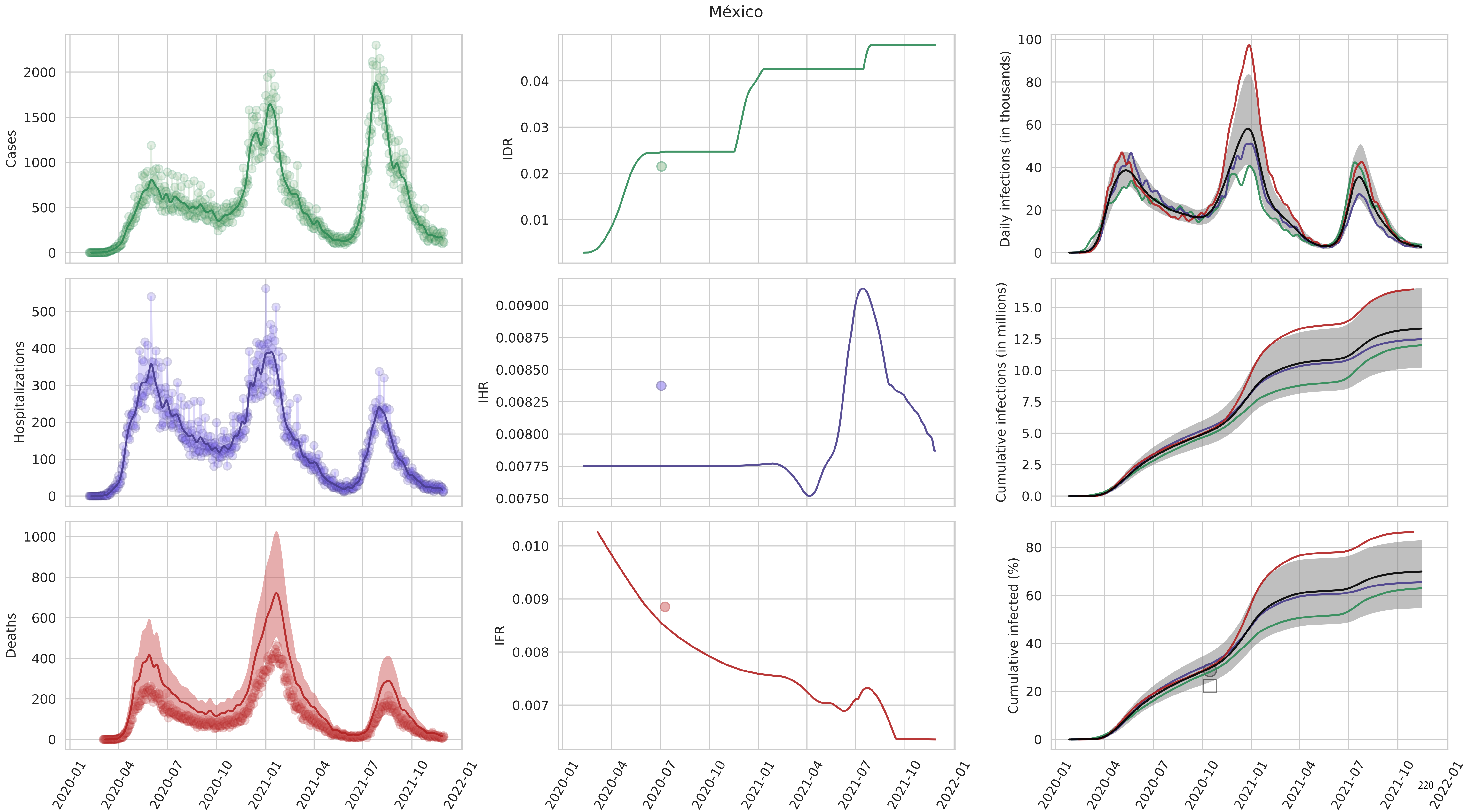
México
Cases
IDR
Daily infections (in thousands)
Hospitalizations
IHR
Cumulative infections (in millions)
Deaths
IFR
Cumulative infected (%)

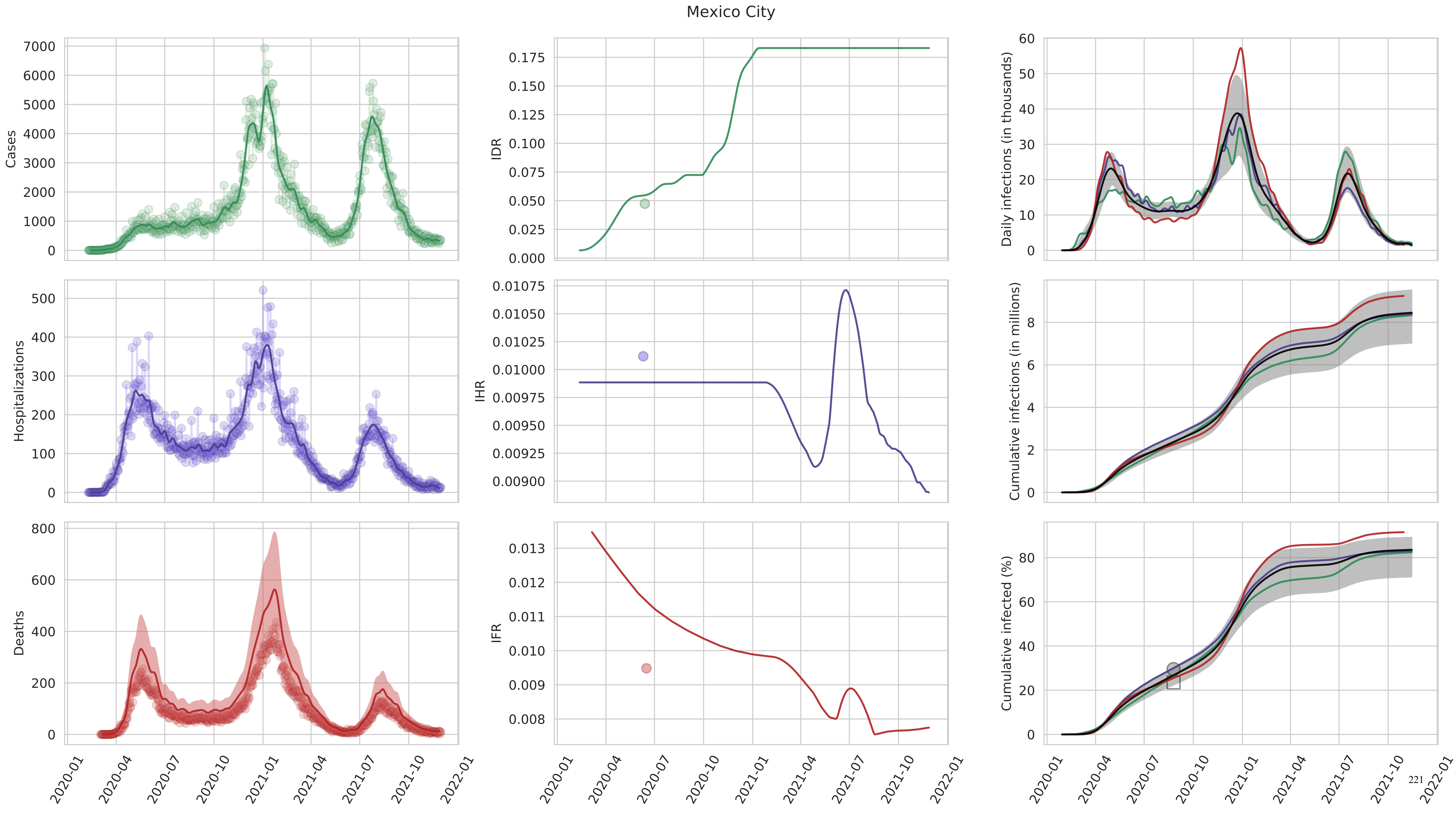
Mexico City
Cases
Hospitalizations
Deaths
IDR
IHR
IFR
Daily infections (in thousands)
Cumulative infections (in millions)
Cumulative infected (%)
2020-01
2020-04
2020-07
2020-10
2021-01
2021-04
2021-07
2021-10
2022-01

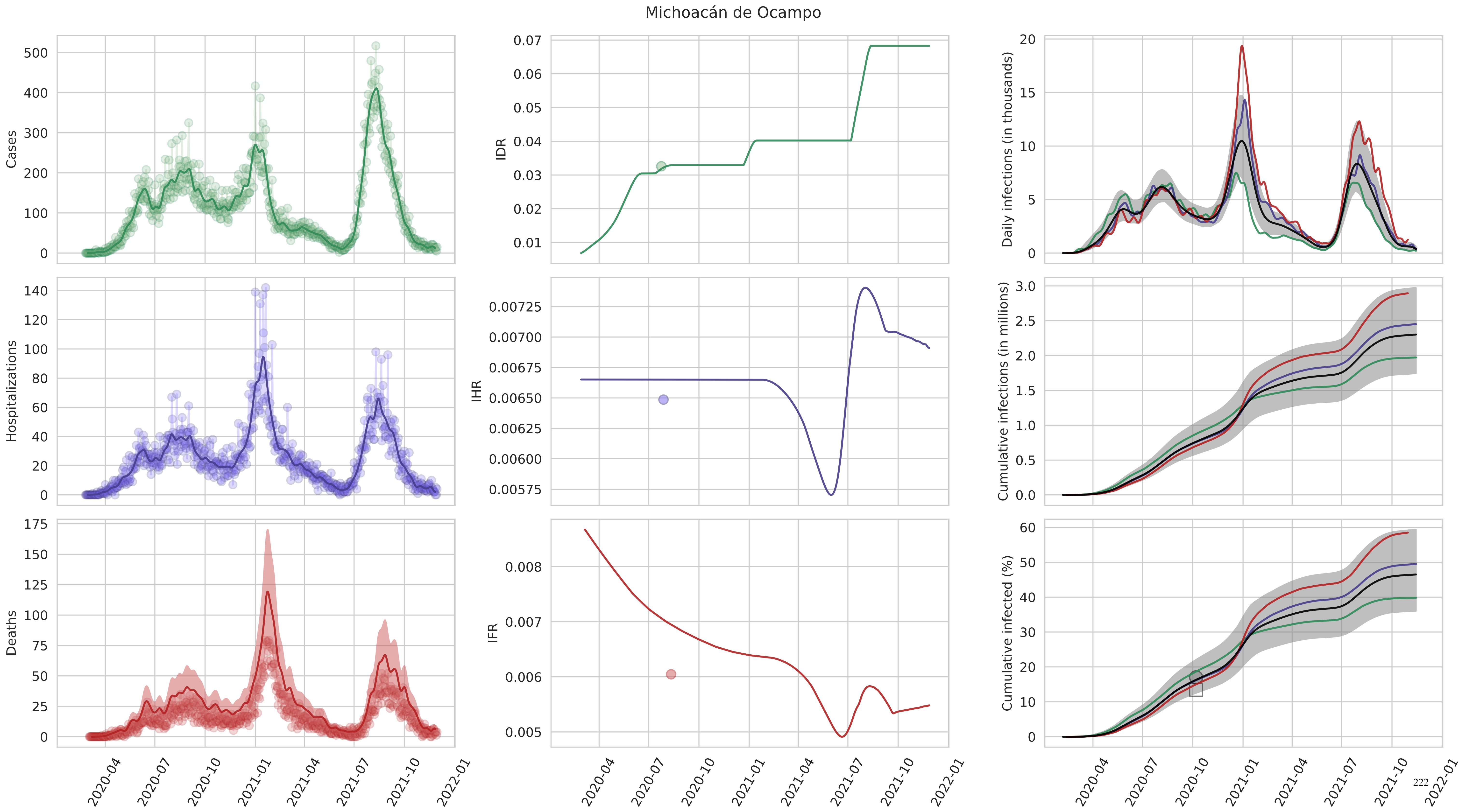
Michoacán de Ocampo
Cases
Hospitalizations
Deaths
IDR
IHR
IFR
Daily infections (in thousands)
Cumulative infections (in millions)
Cumulative infected (%)
2020-04
2020-07
2020-10
2021-01
2021-04
2021-07
2021-10
2022-01

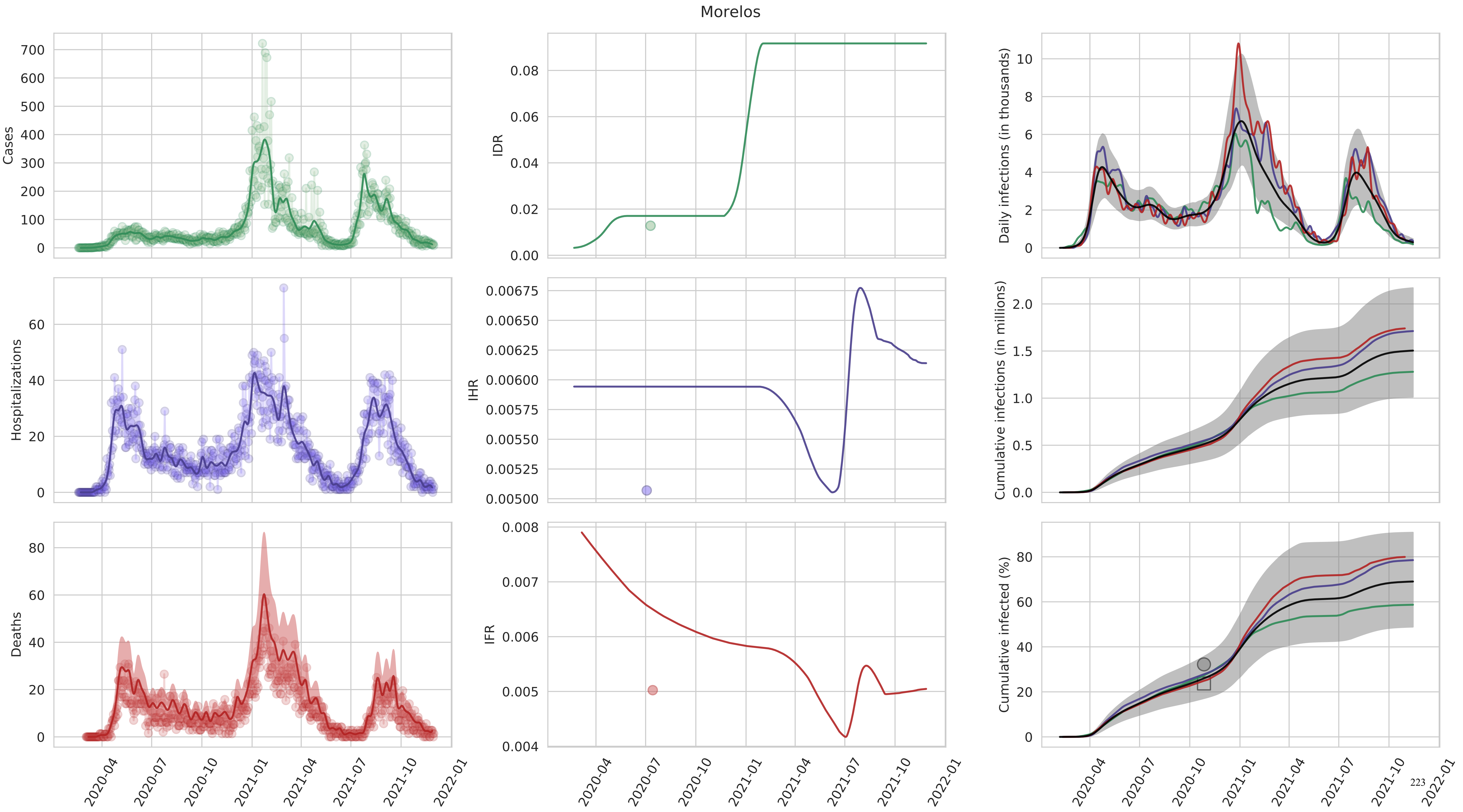
Morelos
Cases
IDR
Daily infections (in thousands)
Hospitalizations
IHR
Cumulative infections (in millions)
Deaths
IFR
Cumulative infected (%)
2020-04
2020-07
2020-10
2021-01
2021-04
2021-07
2021-10
2022-01

Nayarit

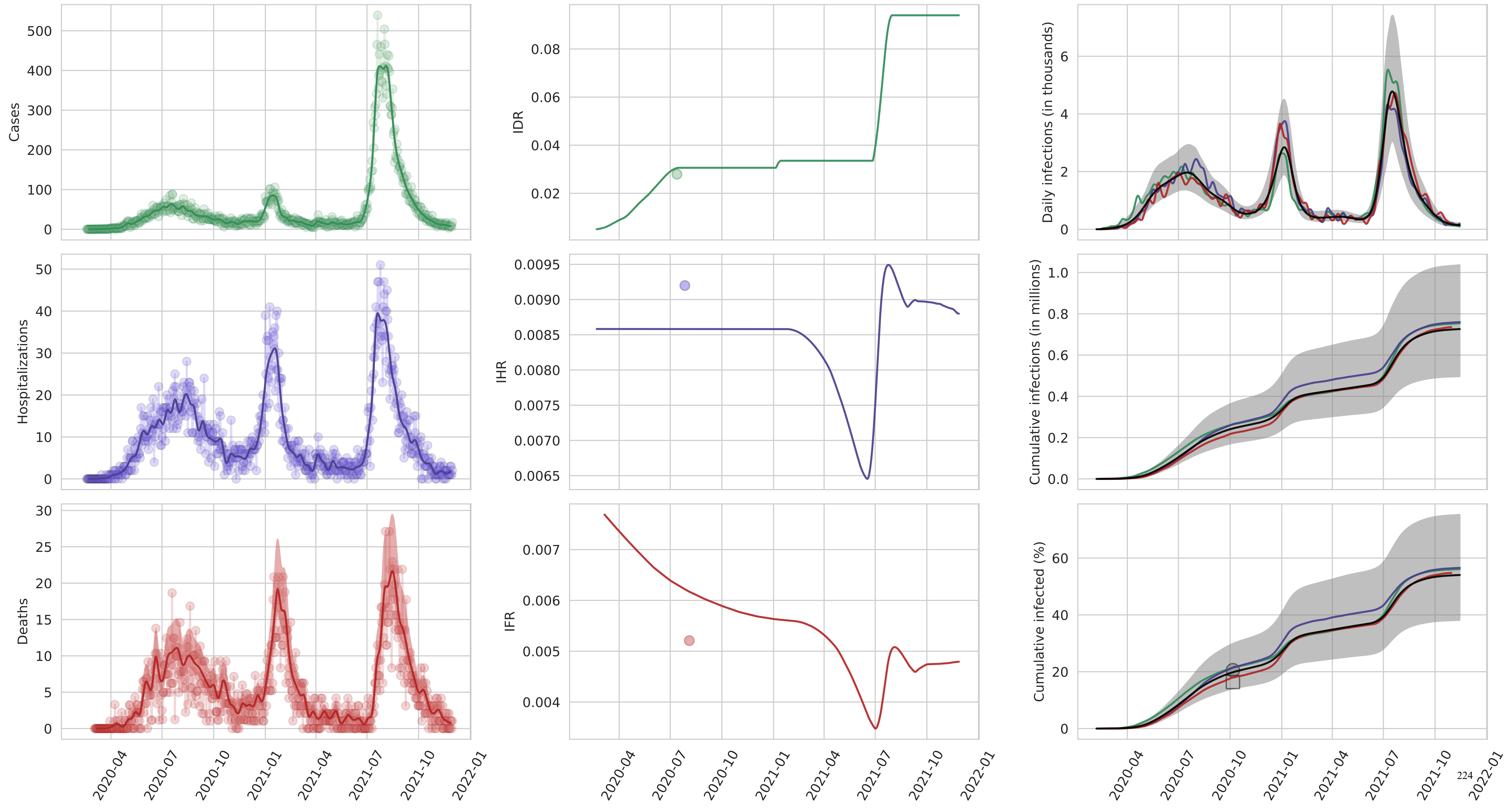

Cases
IDR
Daily infections (in thousands)
Hospitalizations
IHR
Cumulative infections (in millions)
Deaths
IFR
Cumulative infected (%)
2020-04
2020-07
2020-10
2021-01
2021-04
2021-07
2021-10
2022-01

Nuevo León

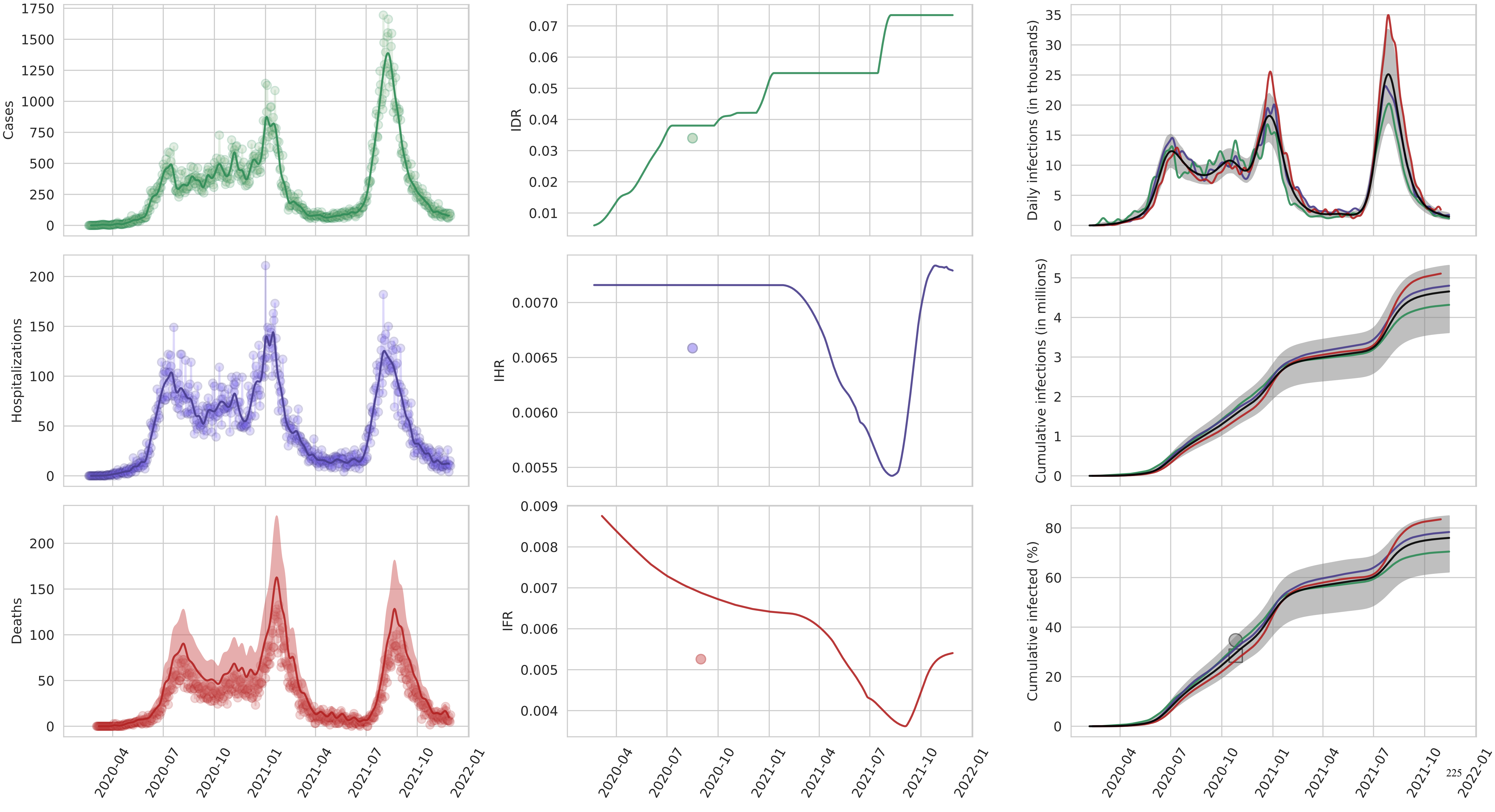
Cases
Hospitalizations
Deaths
IDR
IHR
IFR
Daily infections (in thousands)
Cumulative infections (in millions)
Cumulative infected (%)
2020-04
2020-07
2020-10
2021-01
2021-04
2021-07
2021-10
2022-01

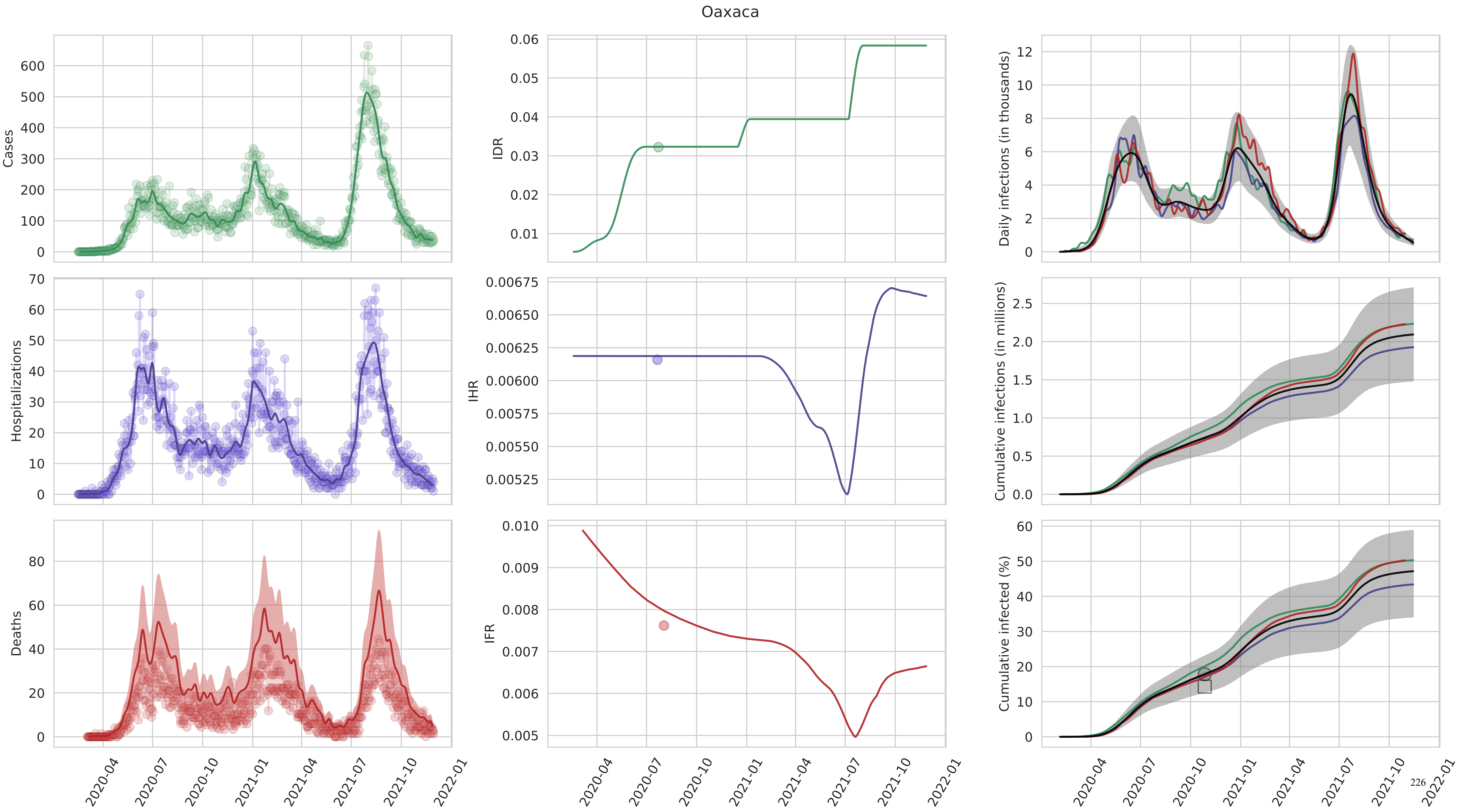
Oaxaca
Cases
Hospitalizations
Deaths
IDR
IHR
IFR
Daily infections (in thousands)
Cumulative infections (in millions)
Cumulative infected (%)
2020-04
2020-07
2020-10
2021-01
2021-04
2021-07
2021-10
2022-01

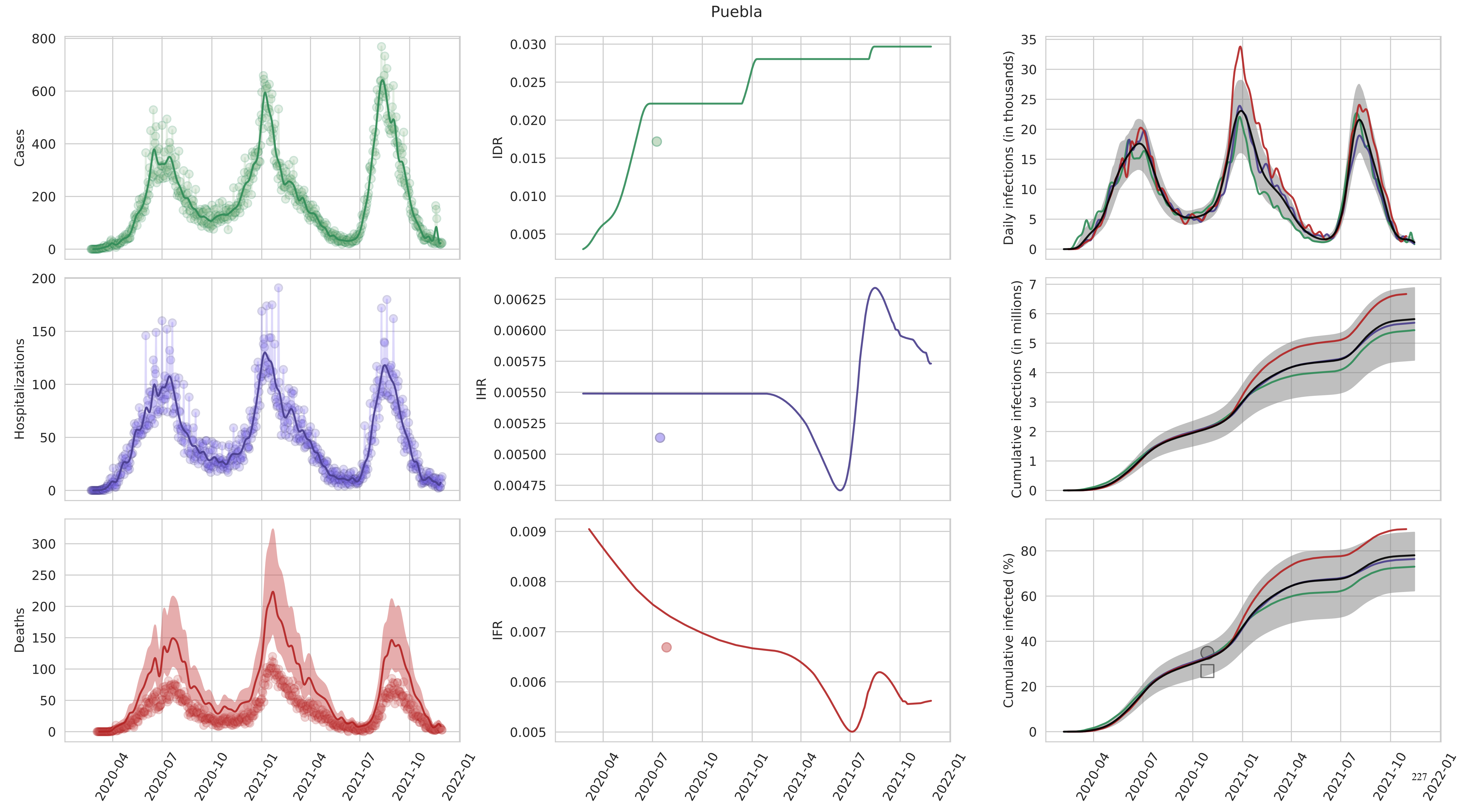

Puebla
Cases
Hospitalizations
Deaths
IDR
IHR
IFR
Daily infections (in thousands)
Cumulative infections (in millions)
Cumulative infected (%)
2020-04
2020-07
2020-10
2021-01
2021-04
2021-07
2021-10
2022-01

Querétaro

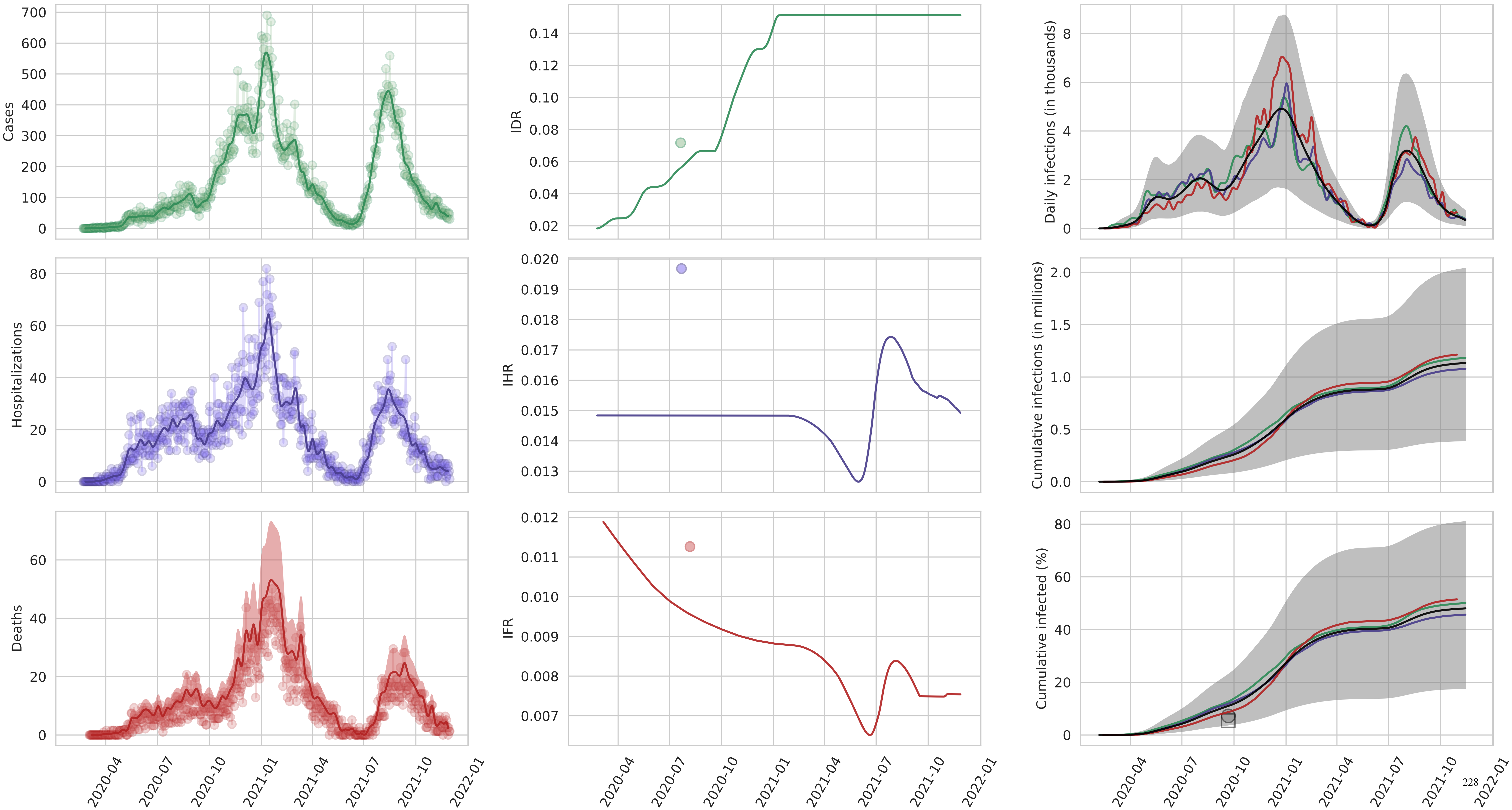

Cases
Hospitalizations
Deaths
IDR
IHR
IFR
Daily infections (in thousands)
Cumulative infections (in millions)
Cumulative infected (%)
2020-04
2020-07
2020-10
2021-01
2021-04
2021-07
2021-10
2022-01

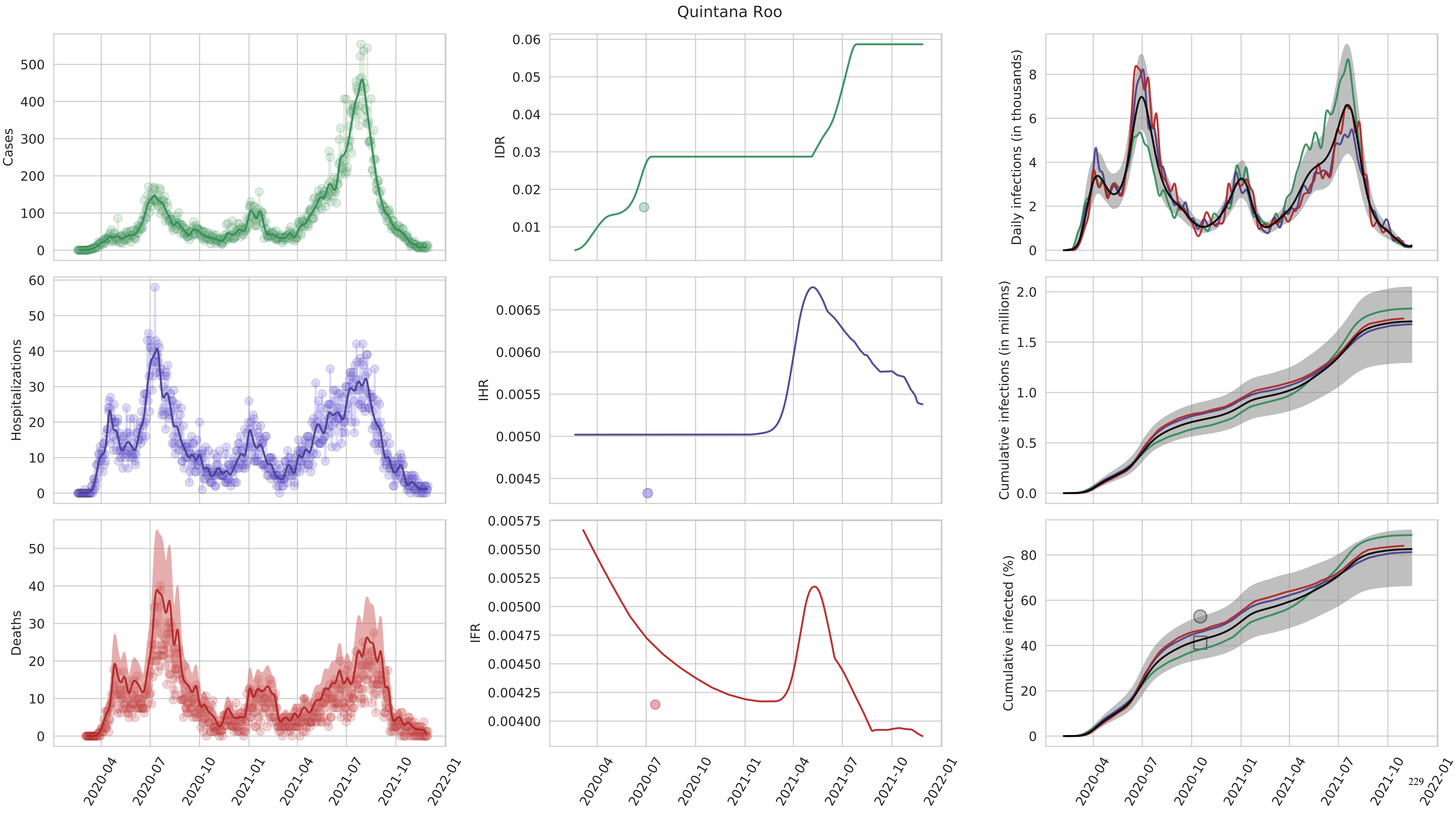
Quintana Roo
Cases
Hospitalizations
Deaths
IDR
IHR
IFR
Daily infections (in thousands)
Cumulative infections (in millions)
Cumulative infected (%)
2020-04
2020-07
2020-10
2021-01
2021-04
2021-07
2021-10
2022-01

San Luis Potosí

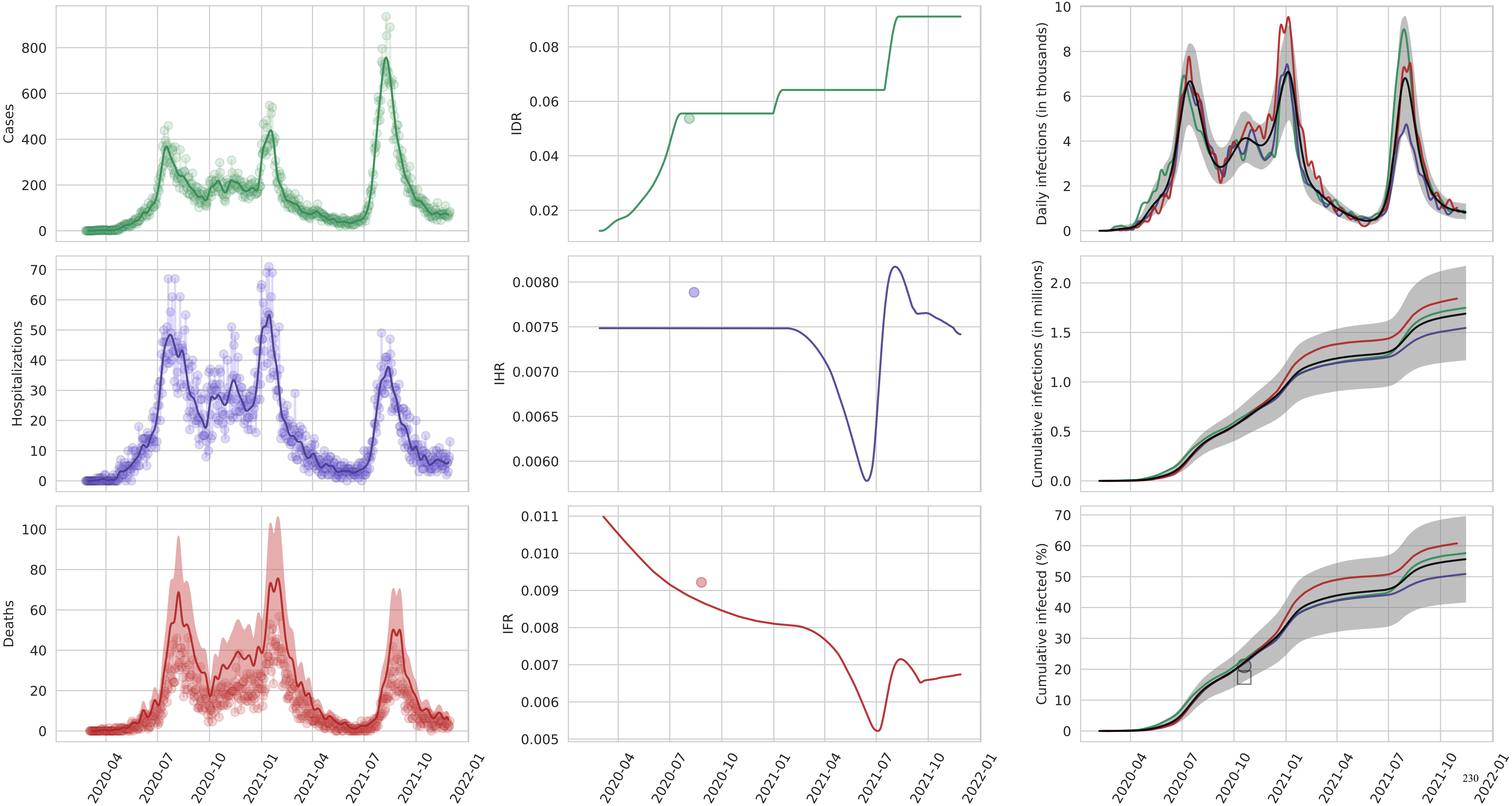

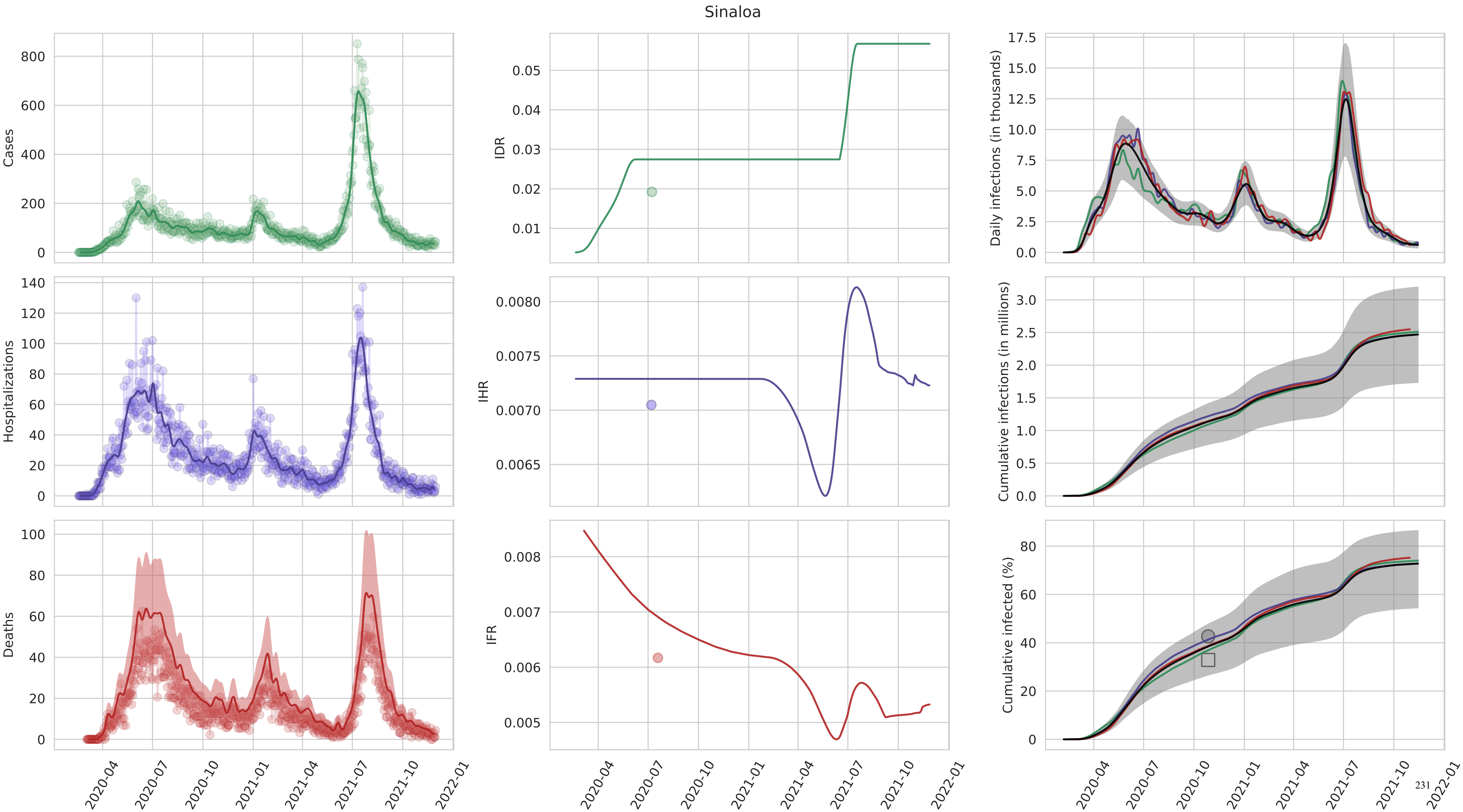

Sinaloa
Cases
Hospitalizations
Deaths
IDR
IHR
IFR
Daily infections (in thousands)
Cumulative infections (in millions)
Cumulative infected (%)
2020-04
2020-07
2020-10
2021-01
2021-04
2021-07
2021-10
2022-01

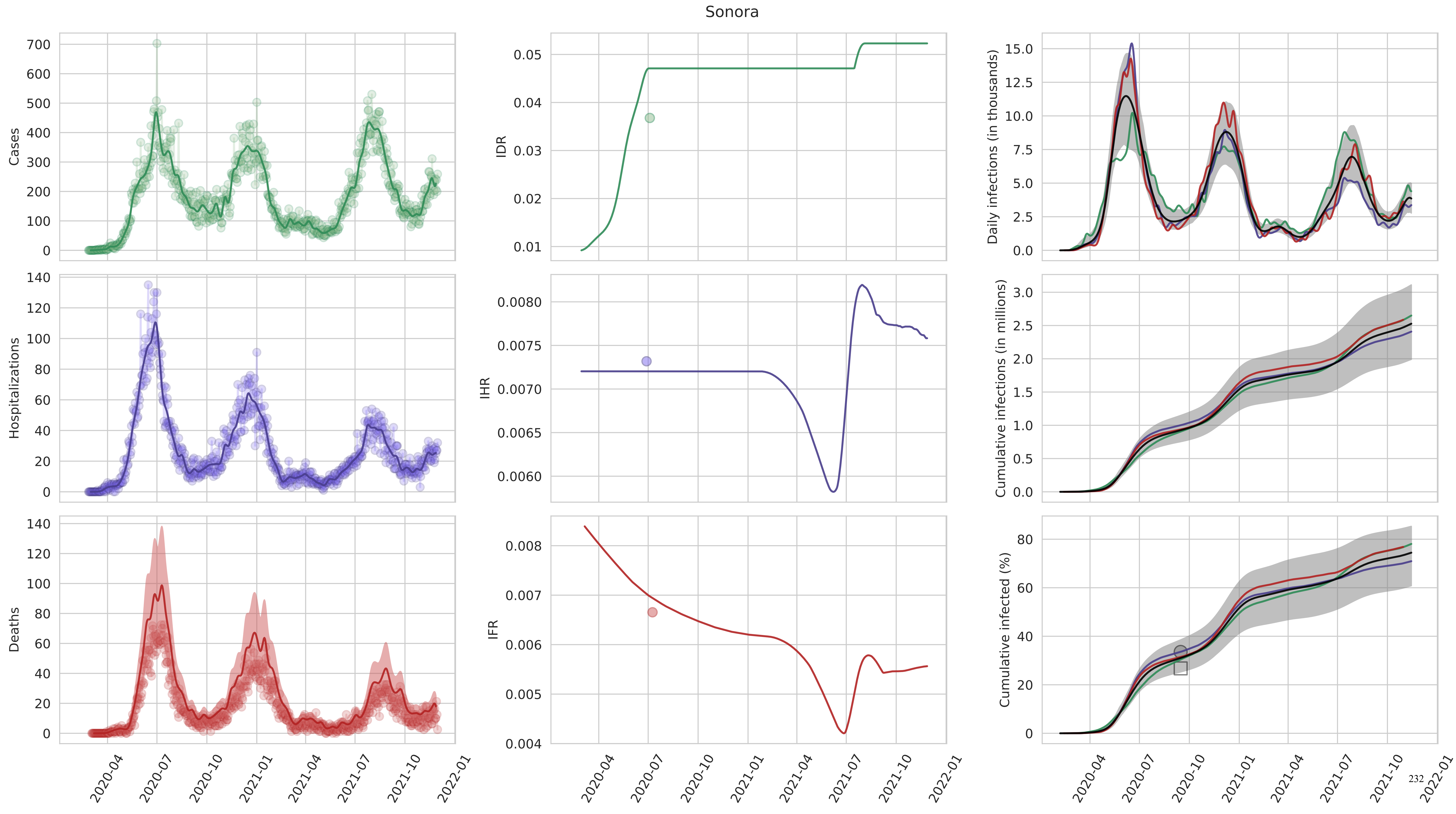

Sonora
Cases
Hospitalizations
Deaths
IDR
IHR
IFR
Daily infections (in thousands)
Cumulative infections (in millions)
Cumulative infected (%)
2020-04
2020-07
2020-10
2021-01
2021-04
2021-07
2021-10
2022-01

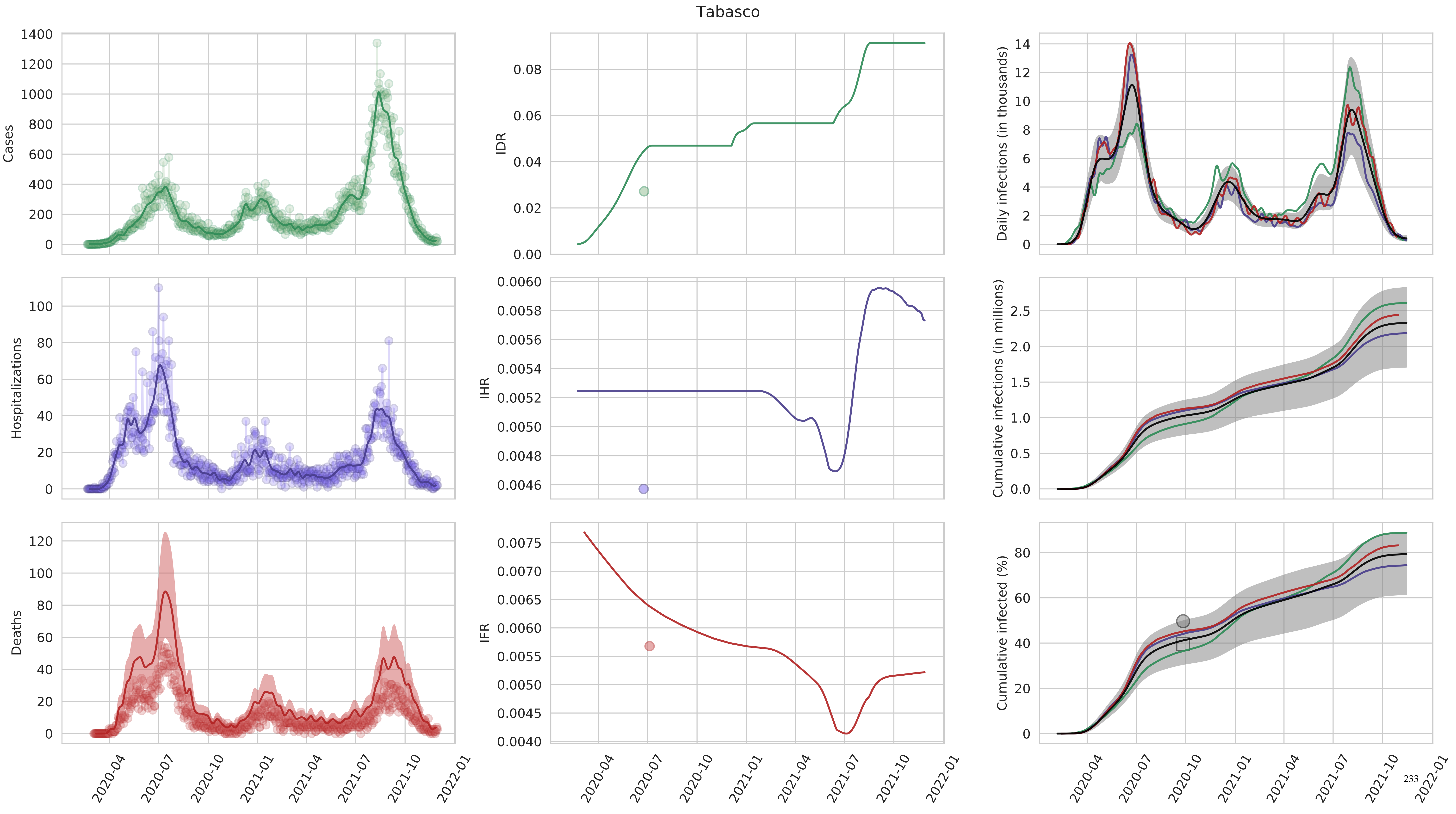
Tabasco
Cases
Hospitalizations
Deaths
IDR
IHR
IFR
Daily infections (in thousands)
Cumulative infections (in millions)
Cumulative infected (%)
2020-04
2020-07
2020-10
2021-01
2021-04
2021-07
2021-10
2022-01

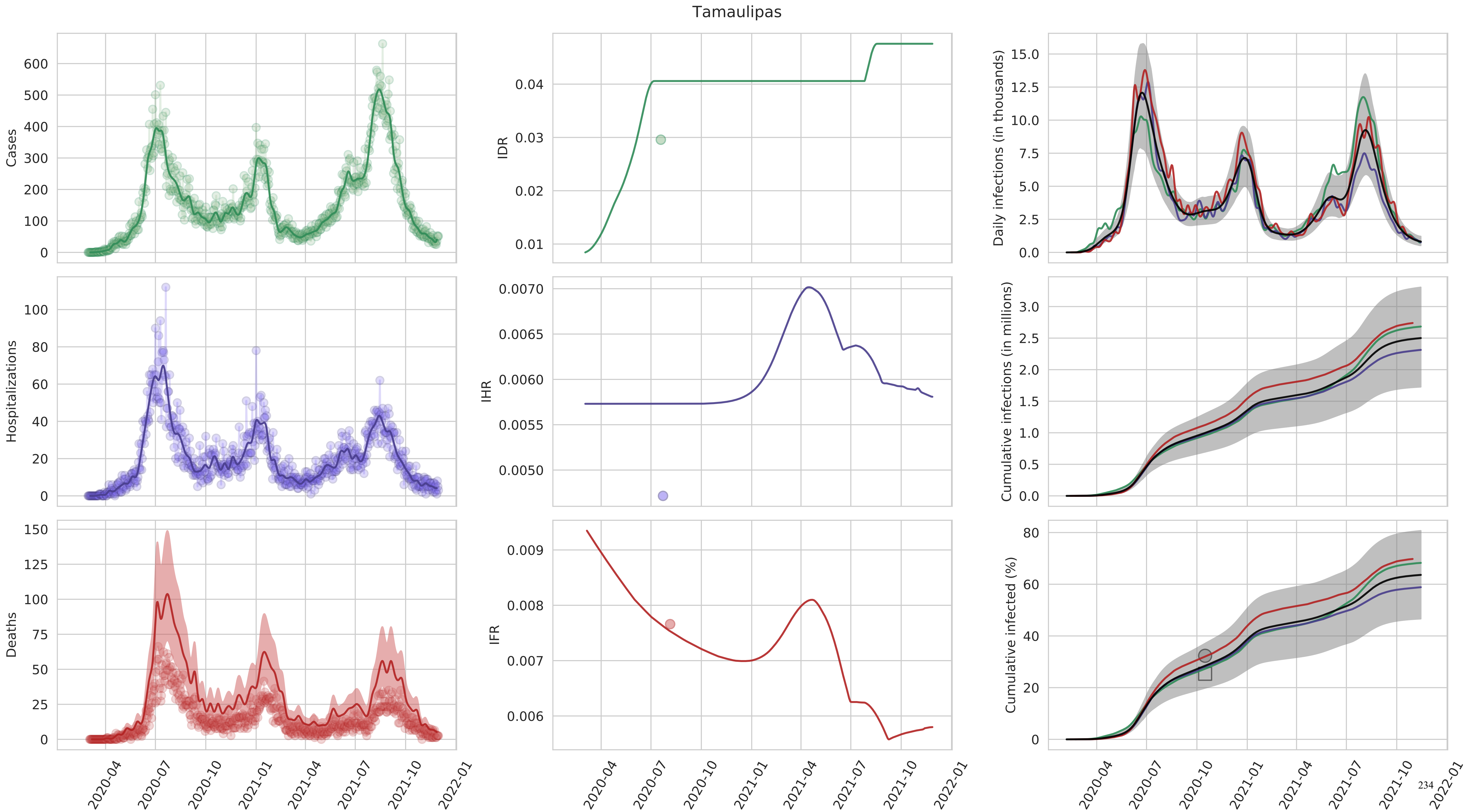

Tamaulipas
Cases
Hospitalizations
Deaths
IDR
IHR
IFR
Daily infections (in thousands)
Cumulative infections (in millions)
Cumulative infected (%)
2020-04
2020-07
2020-10
2021-01
2021-04
2021-07
2021-10
2022-01

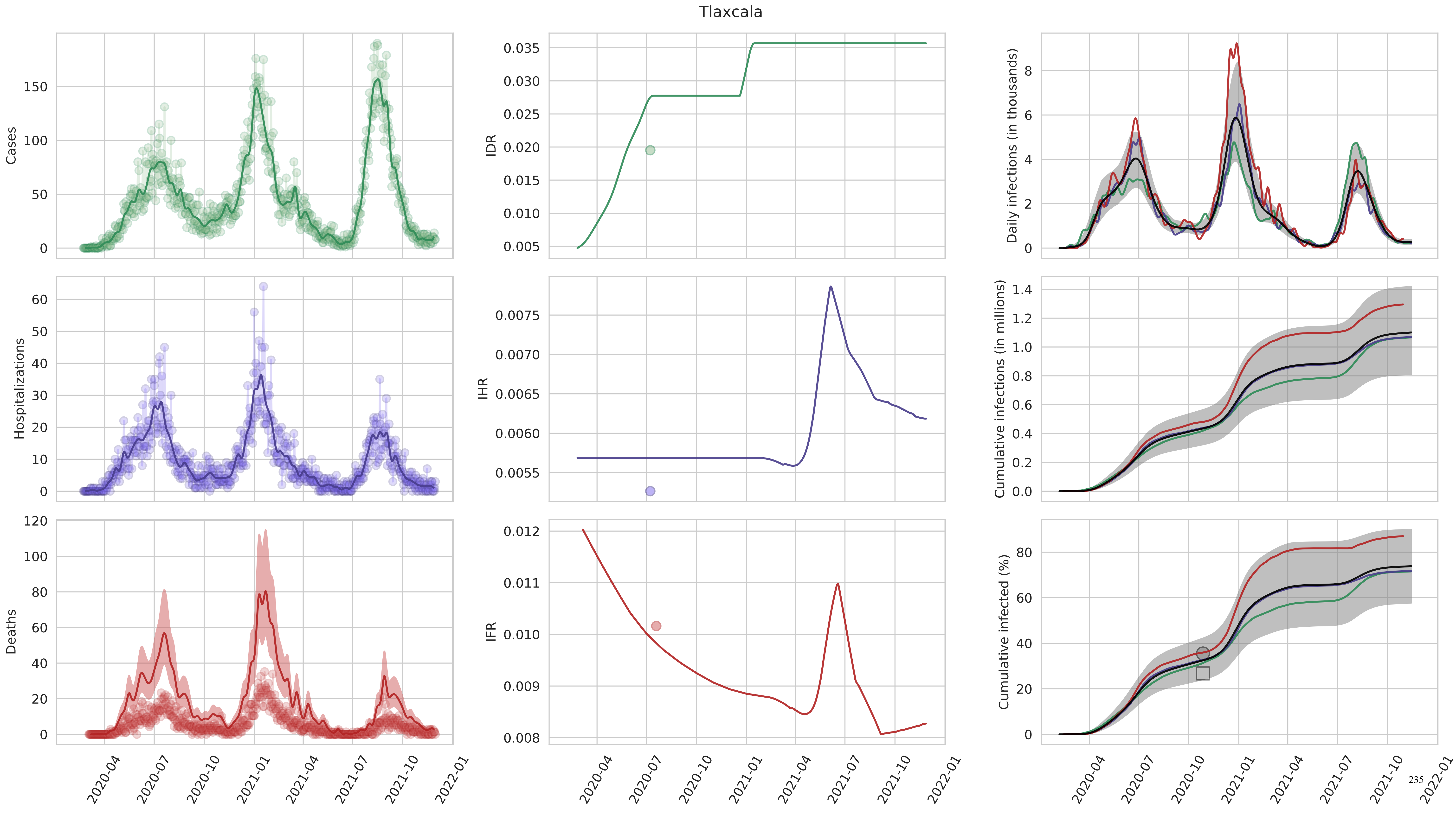
Tlaxcala
Cases
IDR
Daily infections (in thousands)
Hospitalizations
IHR
Cumulative infections (in millions)
Deaths
IFR
Cumulative infected (%)
2020-04
2020-07
2020-10
2021-01
2021-04
2021-07
2021-10
2022-01

Veracruz de Ignacio de la Llave

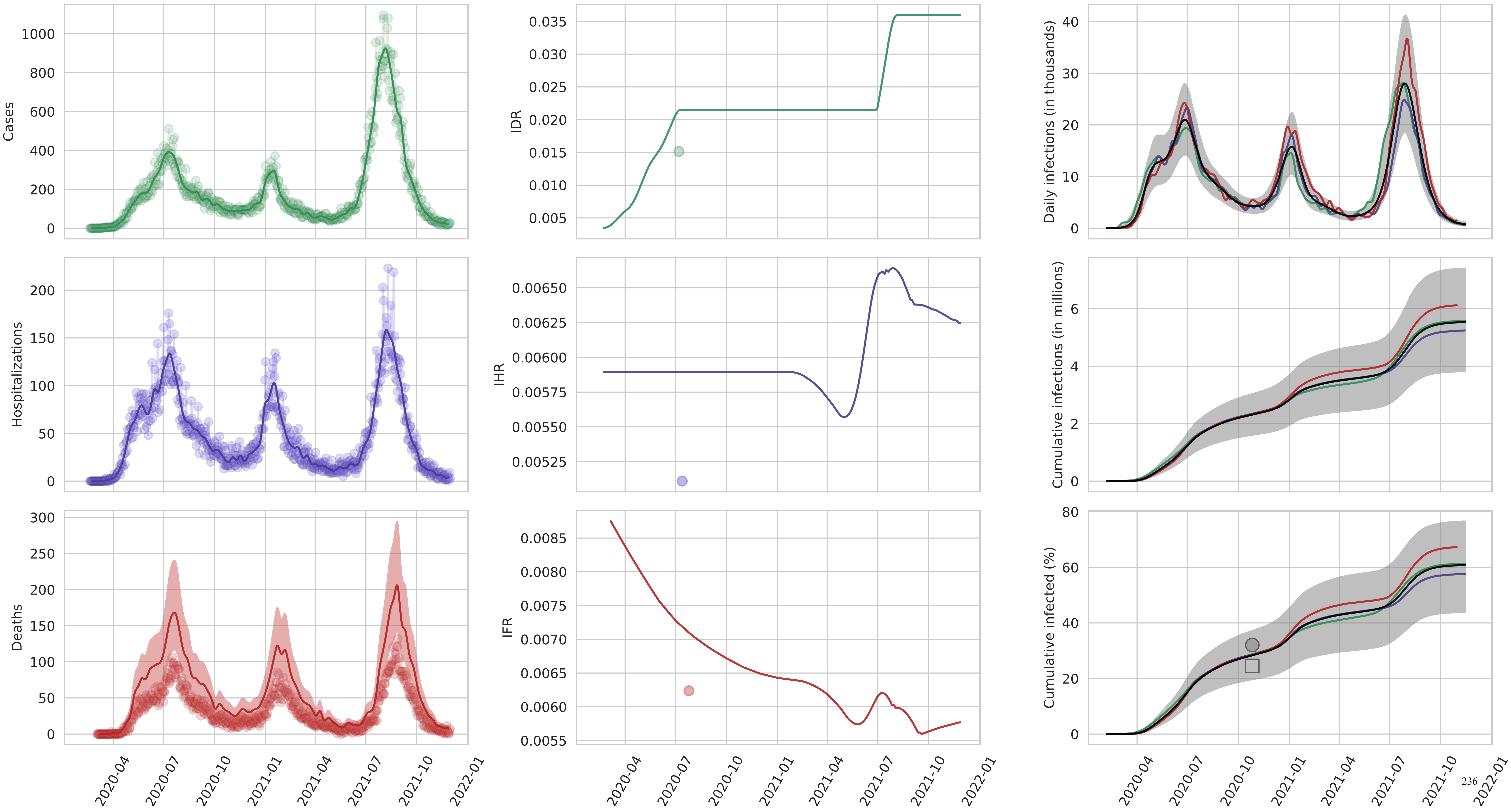

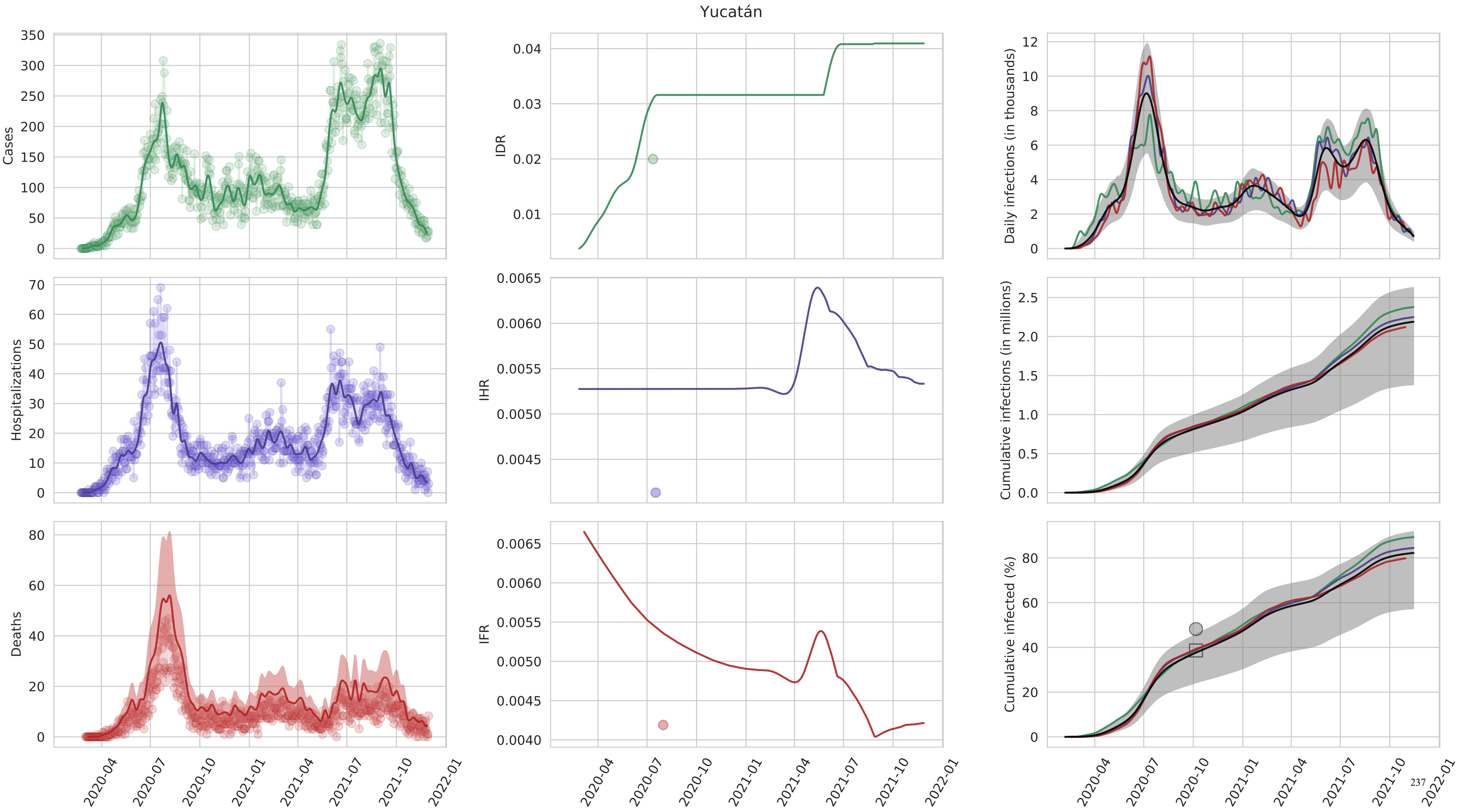

Yucatán
Cases
IDR
Daily infections (in thousands)
Hospitalizations
IHR
Cumulative infections (in millions)
Deaths
IFR
Cumulative infected (%)

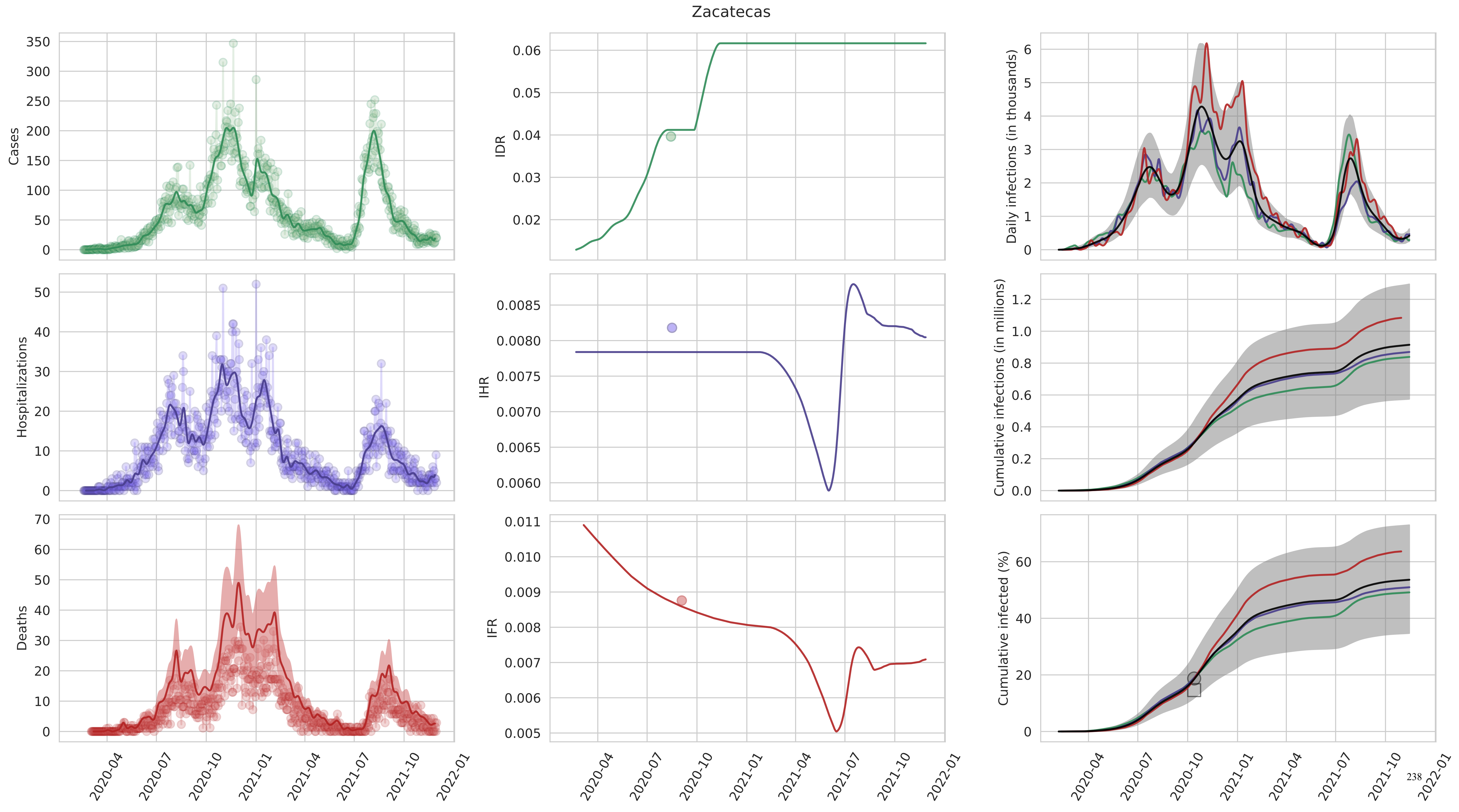
Zacatecas
Cases
IDR
Daily infections (in thousands)
Hospitalizations
IHR
Cumulative infections (in millions)
Deaths
IFR
Cumulative infected (%)
2020-04
2020-07
2020-10
2021-01
2021-04
2021-07
2021-10
2022-01

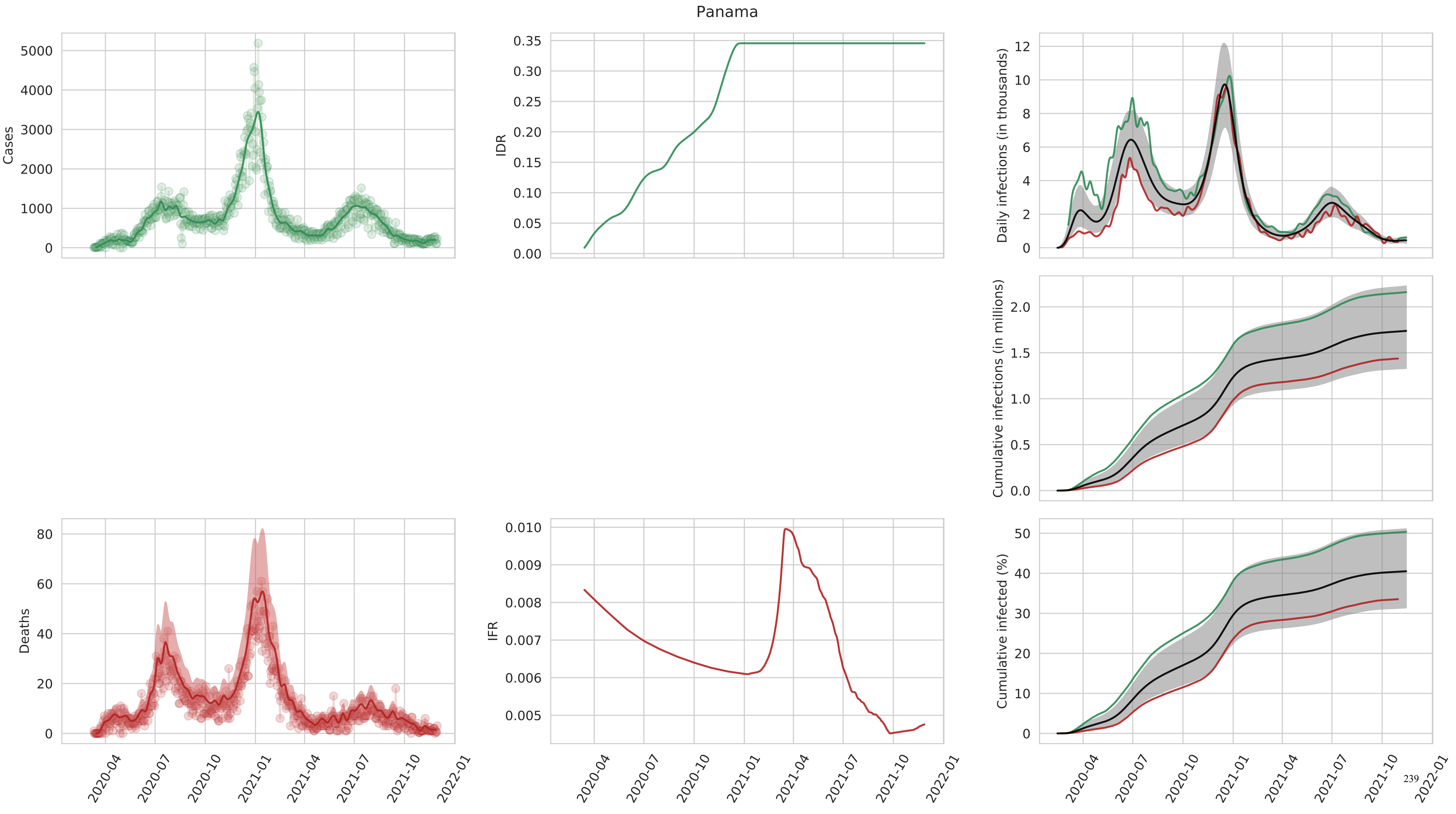
Panama
Cases
IDR
Daily infections (in thousands)
Cumulative infections (in millions)
Deaths
IFR
Cumulative infected (%)
2020-04
2020-07
2020-10
2021-01
2021-04
2021-07
2021-10
2022-01

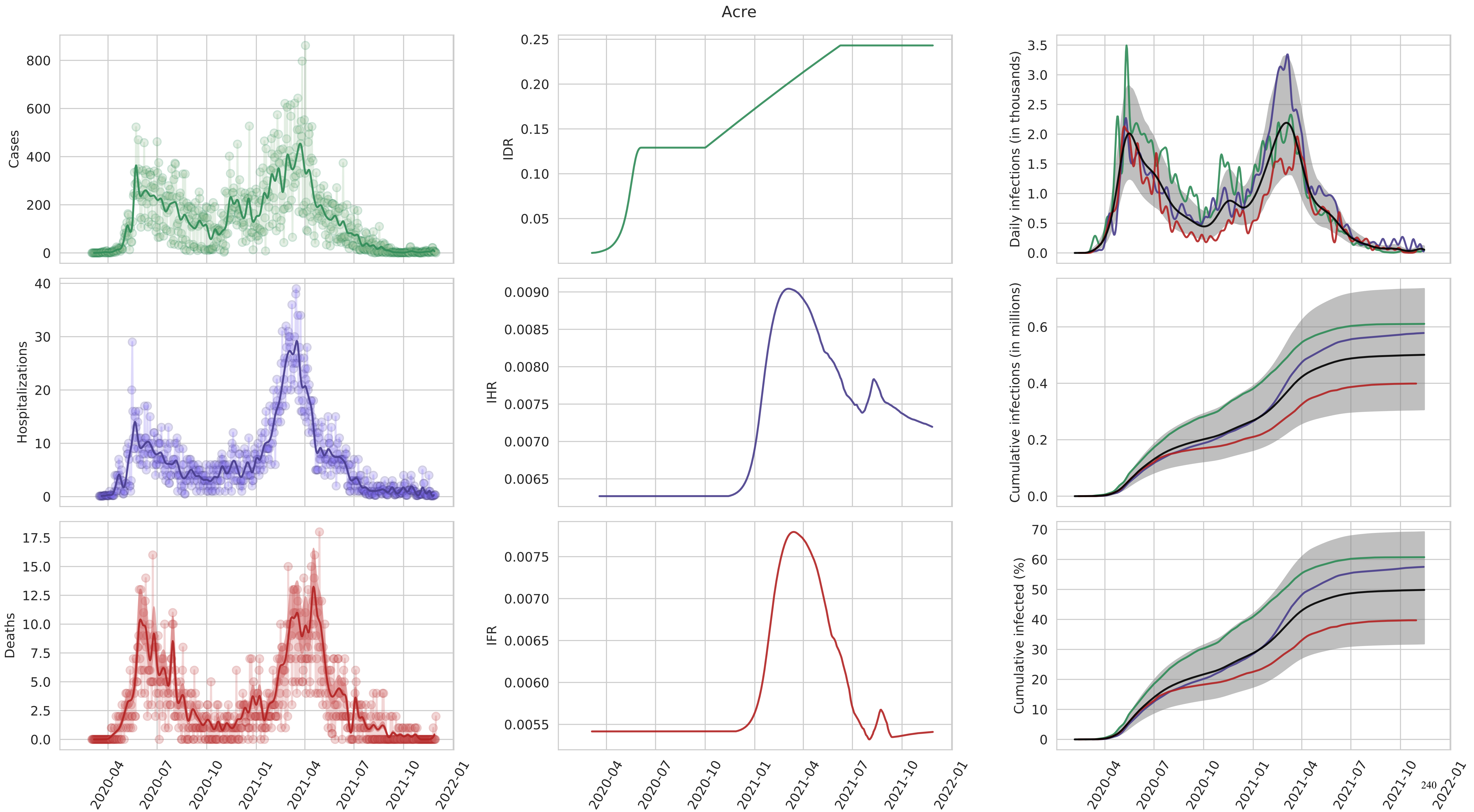
Acre
Cases
IDR
Daily infections (in thousands)
Hospitalizations
IHR
Cumulative infections (in millions)
Deaths
IFR
Cumulative infected (%)
2020-04
2020-07
2020-10
2021-01
2021-04
2021-07
2021-10
2022-01

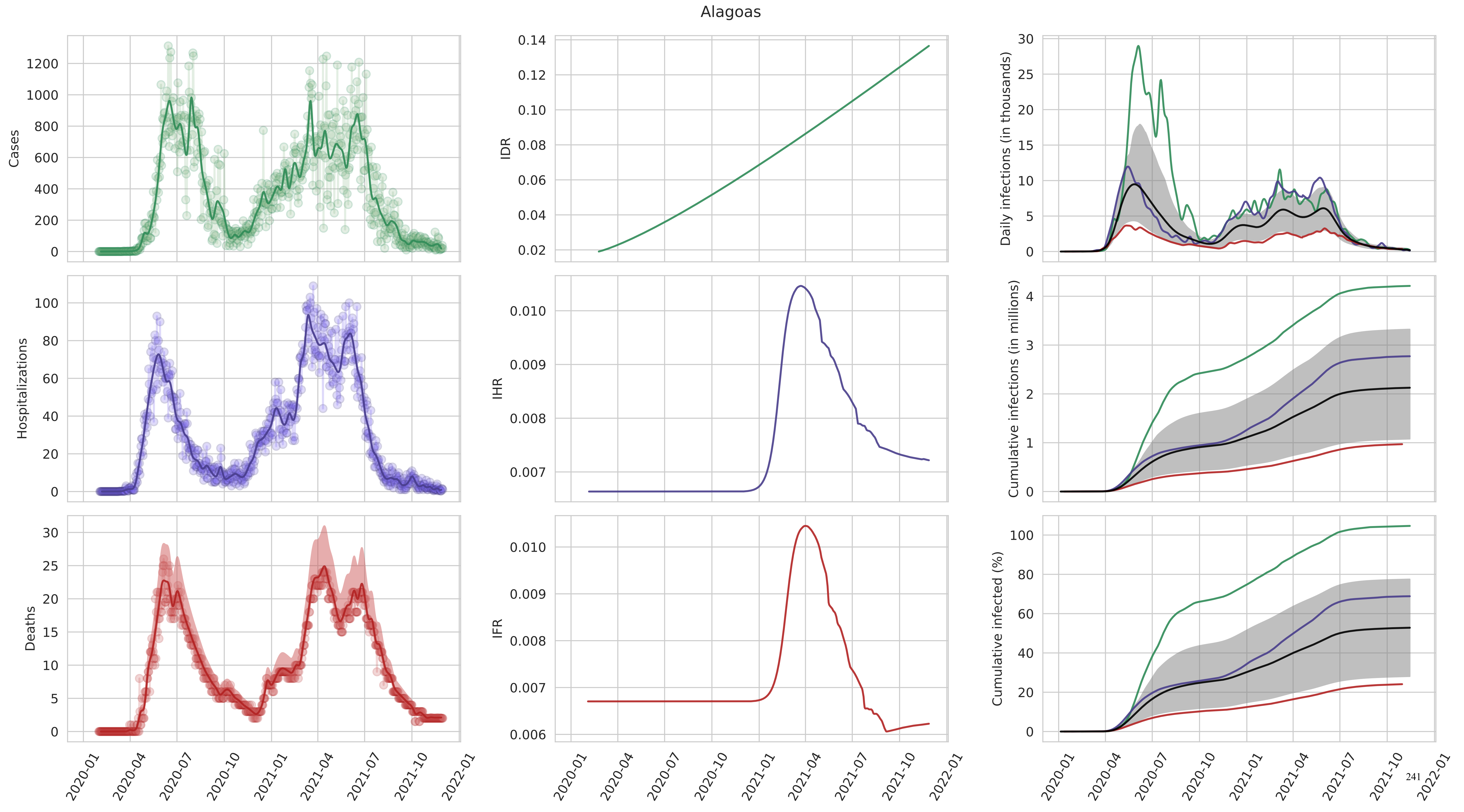
Alagoas
Cases
Hospitalizations
Deaths
IDR
IHR
IFR
Daily infections (in thousands)
Cumulative infections (in millions)
Cumulative infected (%)
2020-01
2020-04
2020-07
2020-10
2021-01
2021-04
2021-07
2021-10
2022-01

Amapá

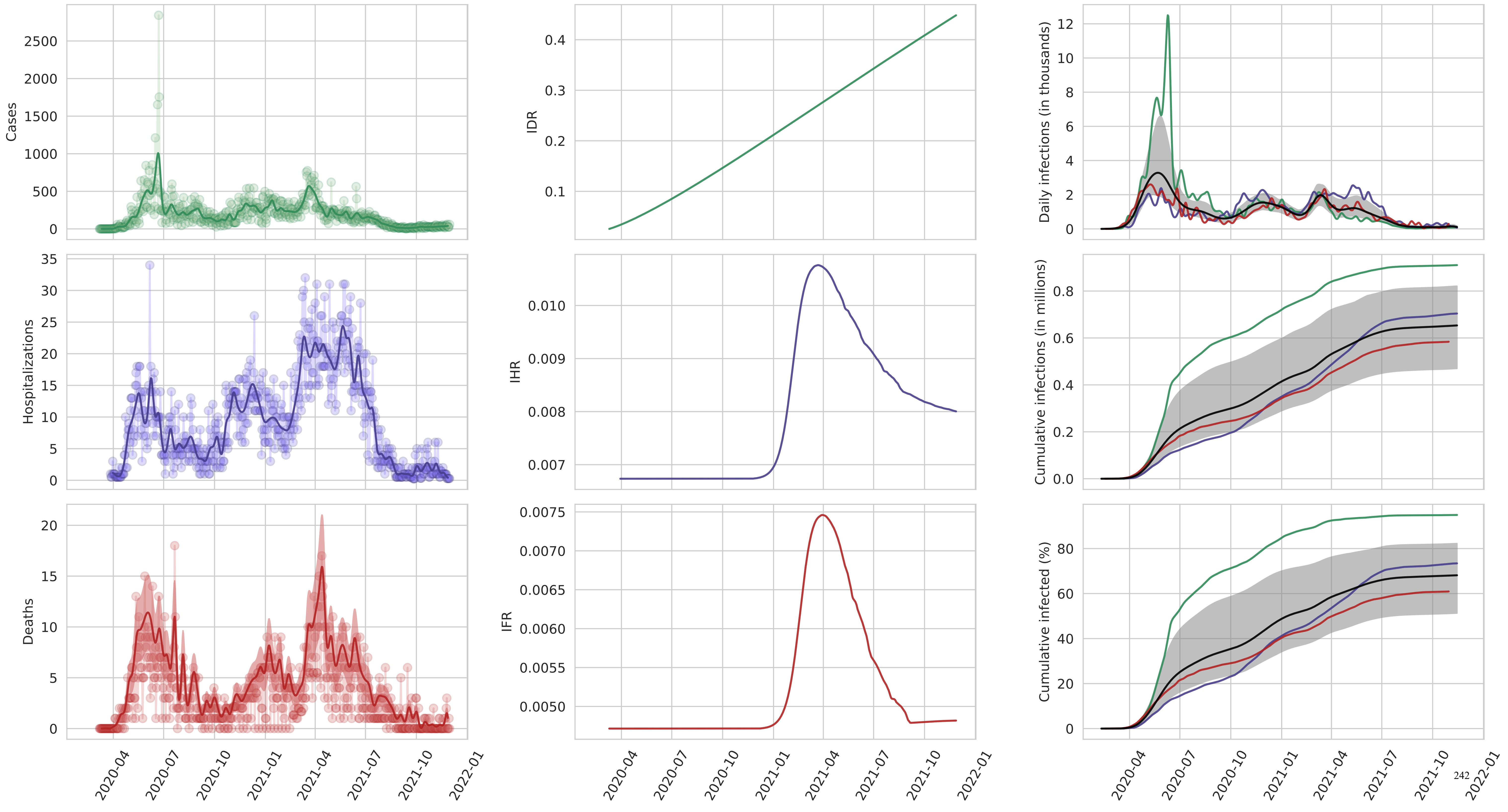

Cases
Hospitalizations
Deaths
IDR
IHR
IFR
Daily infections (in thousands)
Cumulative infections (in millions)
Cumulative infected (%)
2020-04
2020-07
2020-10
2021-01
2021-04
2021-07
2021-10
2022-01

Amazonas

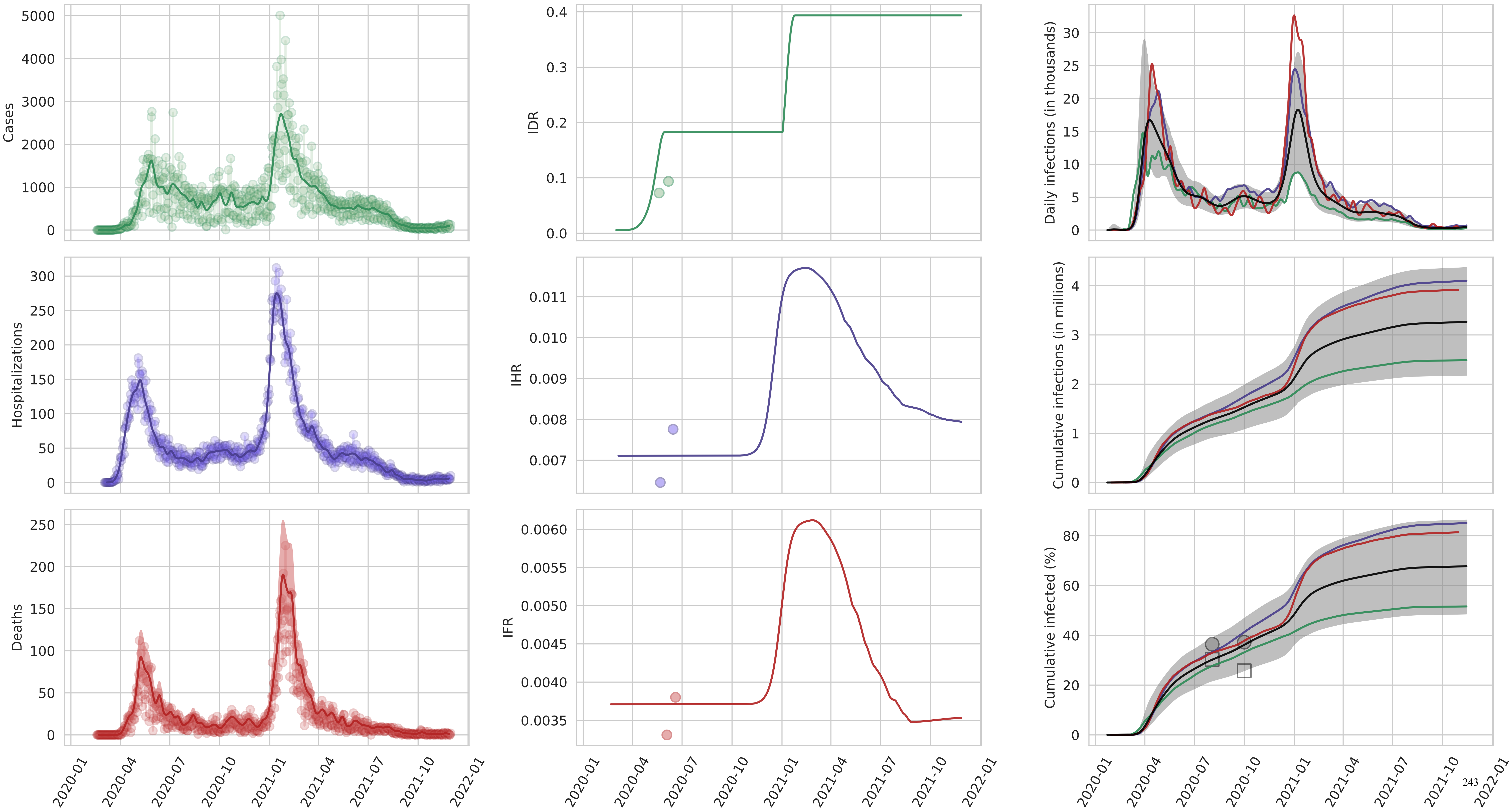

Cases
IDR
Daily infections (in thousands)
Hospitalizations
IHR
Cumulative infections (in millions)
Deaths
IFR
Cumulative infected (%)
2020-01
2020-04
2020-07
2020-10
2021-01
2021-04
2021-07
2021-10
2022-01

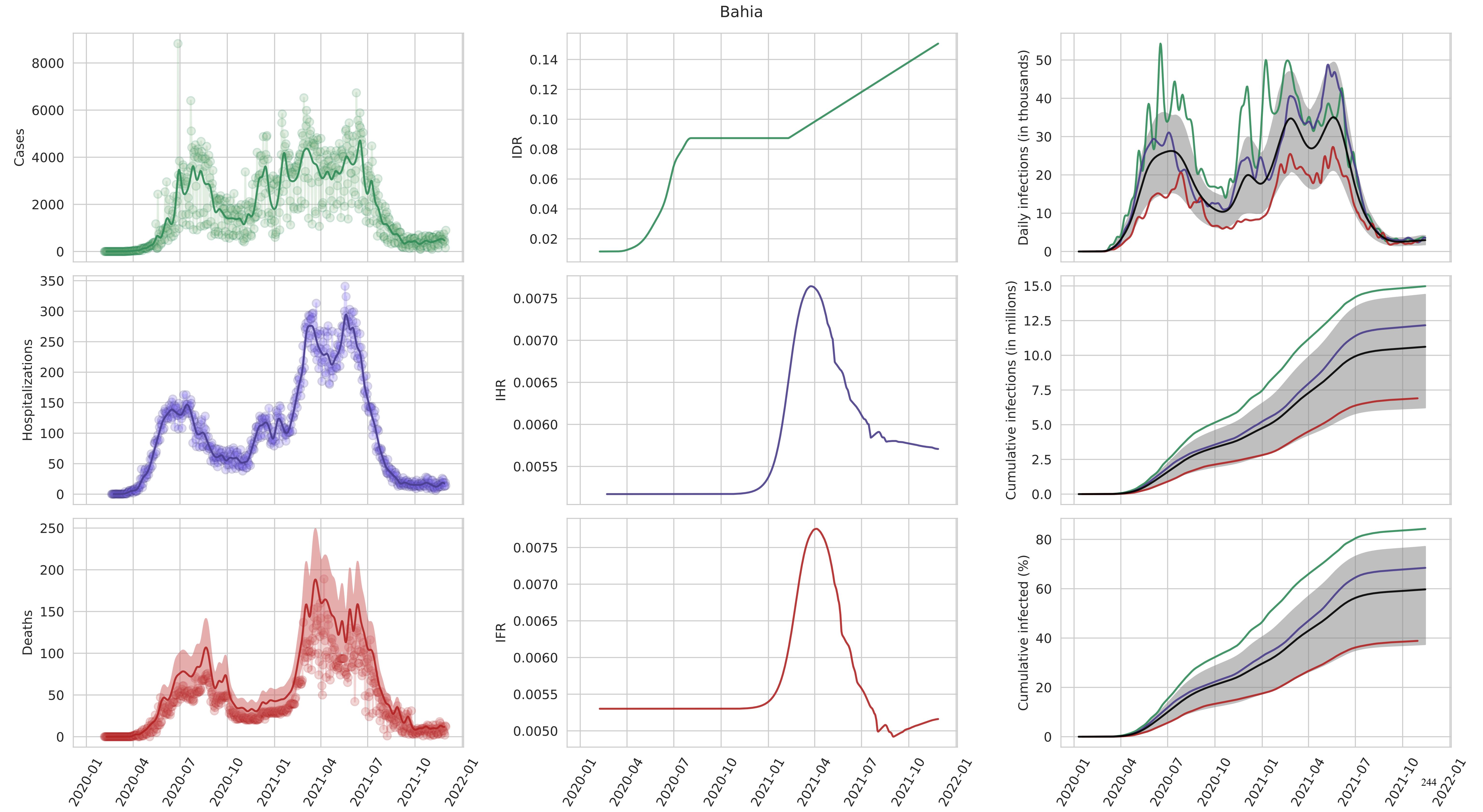
Bahia
Cases
Hospitalizations
Deaths
IDR
IHR
IFR
Daily infections (in thousands)
Cumulative infections (in millions)
Cumulative infected (%)
2020-01
2020-04
2020-07
2020-10
2021-01
2021-04
2021-07
2021-10
2022-01

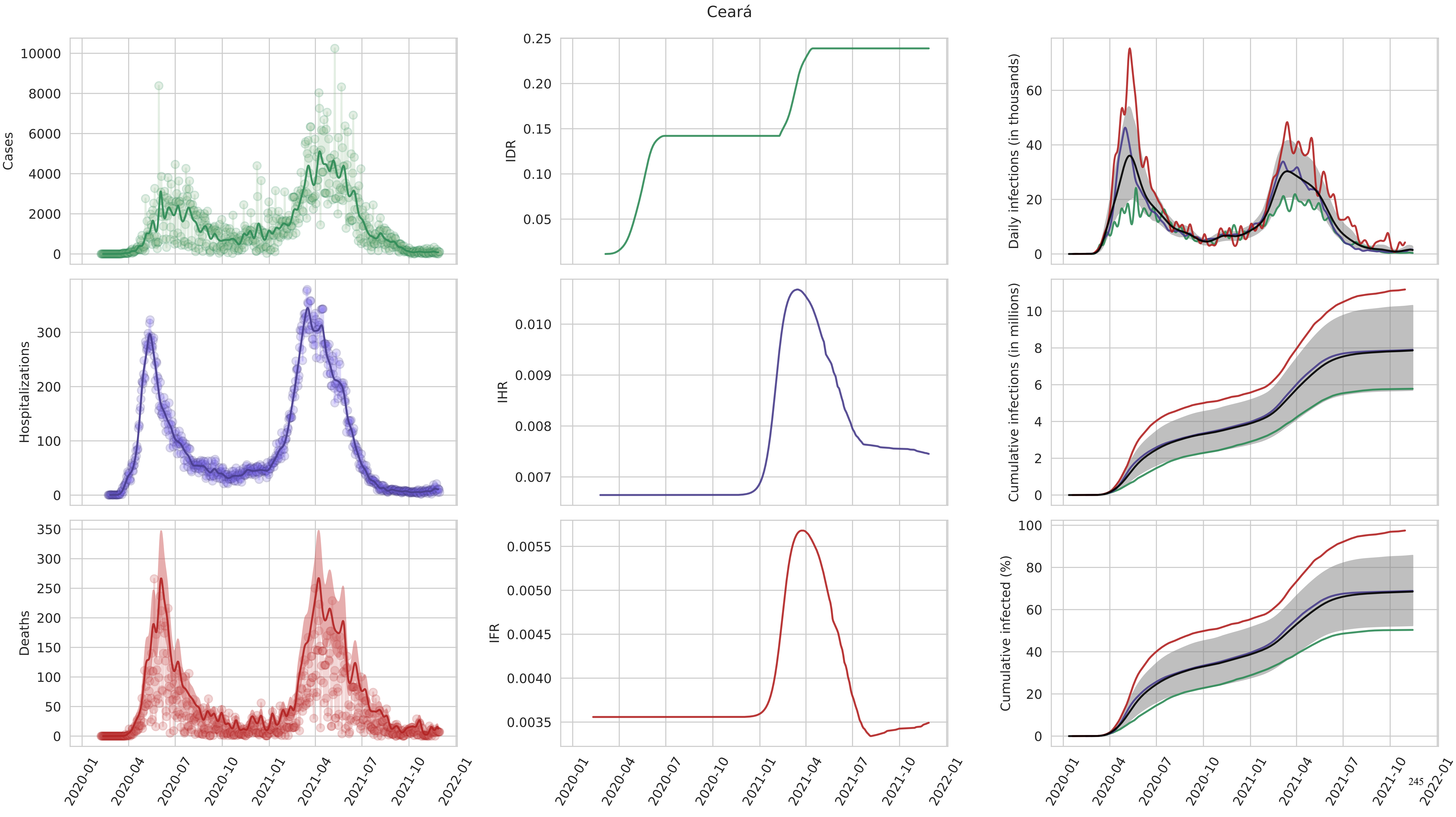

Ceará
Cases
Hospitalizations
Deaths
IDR
IHR
IFR
Daily infections (in thousands)
Cumulative infections (in millions)
Cumulative infected (%)
2020-01
2020-04
2020-07
2020-10
2021-01
2021-04
2021-07
2021-10
2022-01

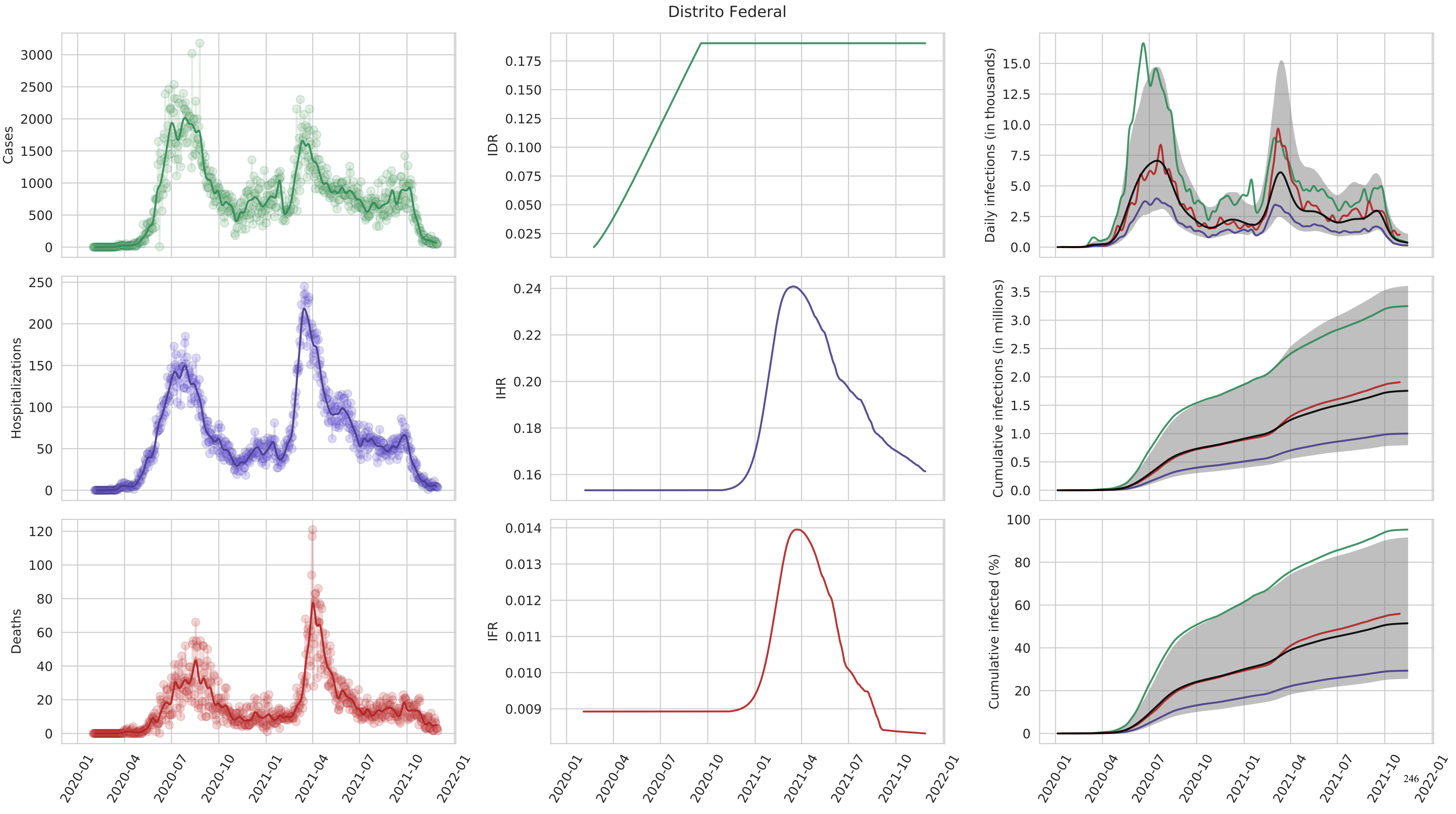

Distrito Federal
Cases
Hospitalizations
Deaths
IDR
IHR
IFR
Daily infections (in thousands)
Cumulative infections (in millions)
Cumulative infected (%)

Espírito Santo

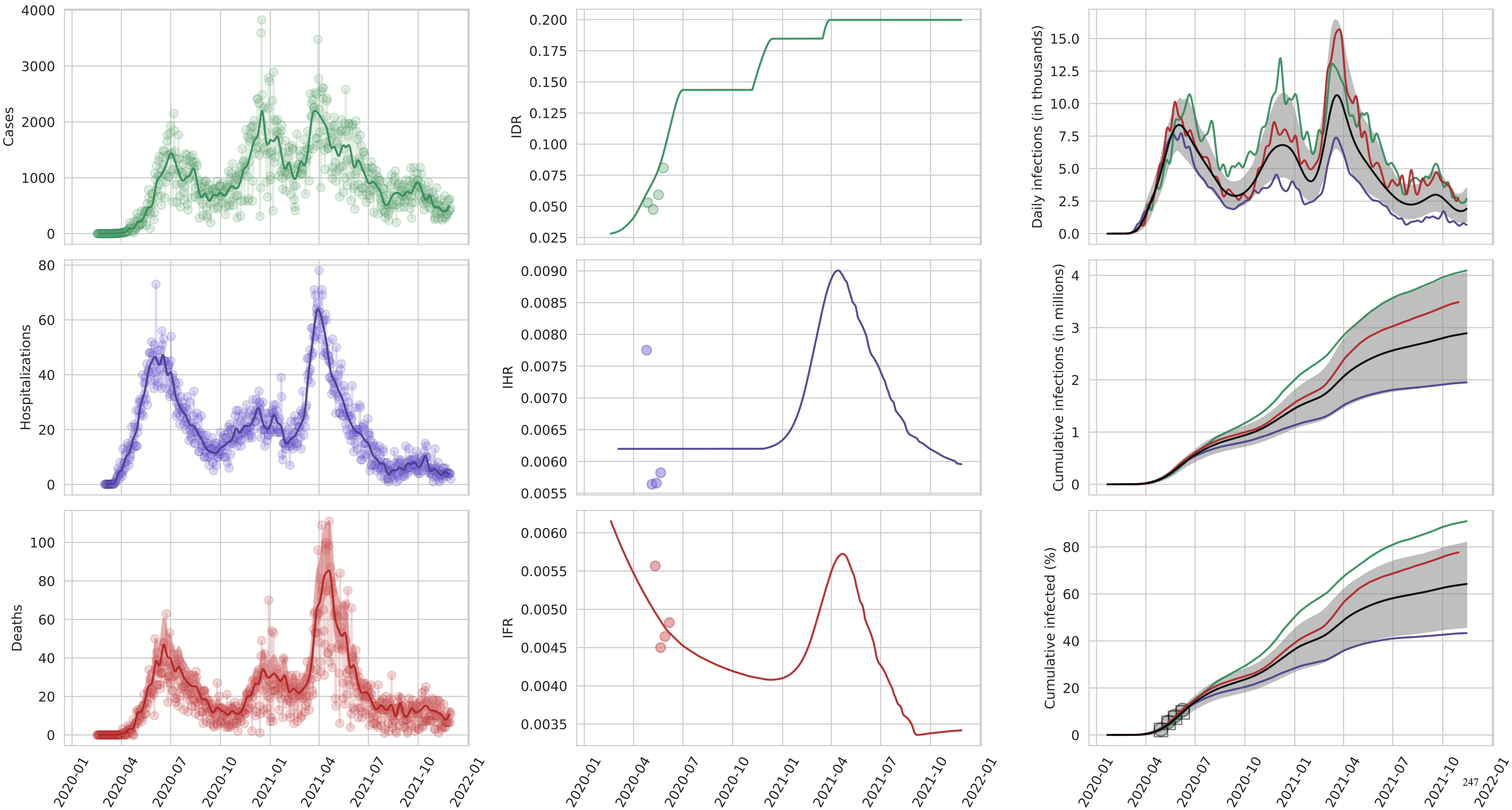

Goiás

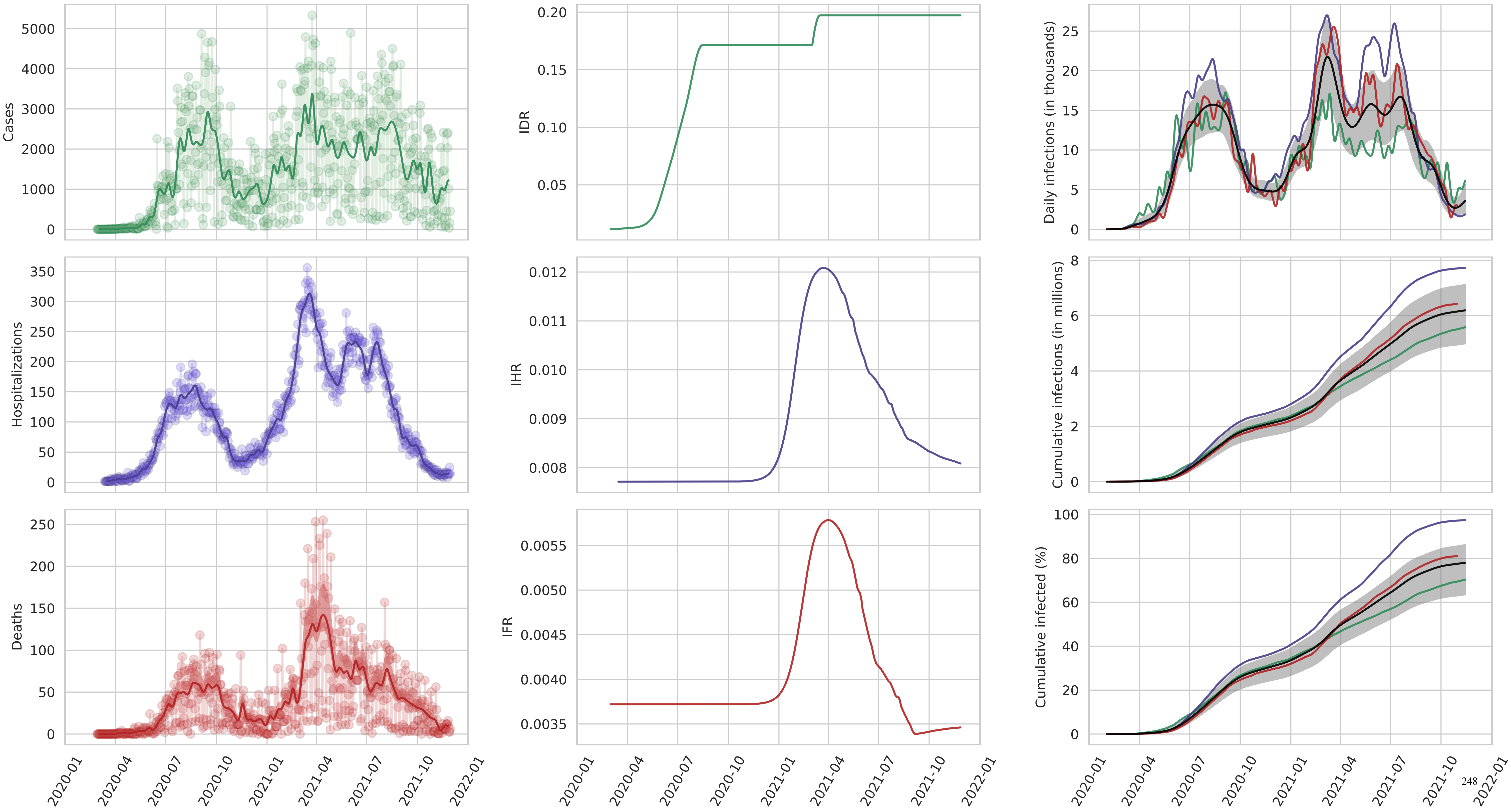

Cases
Hospitalizations
Deaths
IDR
IHR
IFR
Daily infections (in thousands)
Cumulative infections (in millions)
Cumulative infected (%)

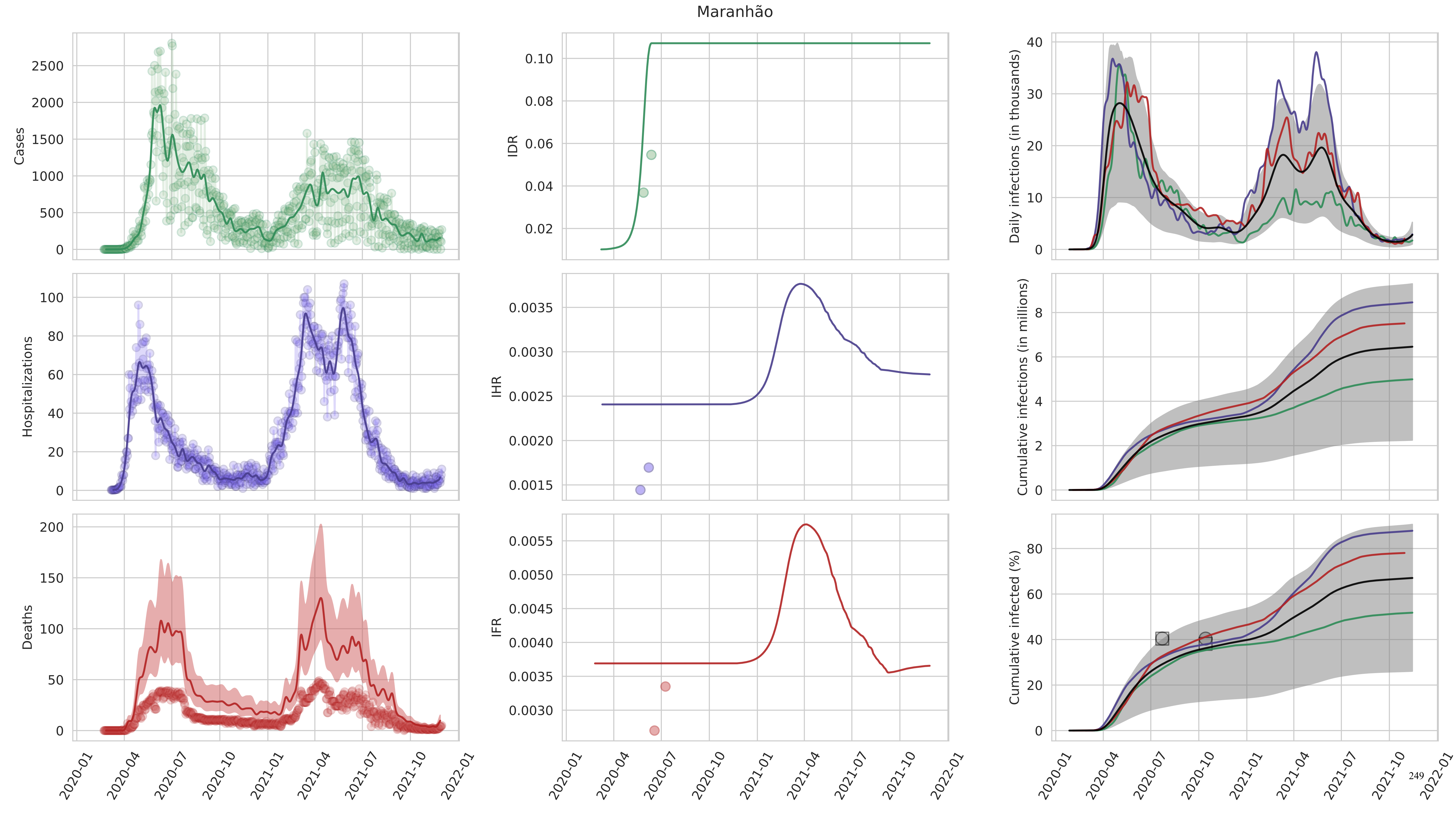

Maranhão
Cases
Hospitalizations
Deaths
IDR
IHR
IFR
Daily infections (in thousands)
Cumulative infections (in millions)
Cumulative infected (%)
2020-01
2020-04
2020-07
2020-10
2021-01
2021-04
2021-07
2021-10
2022-01

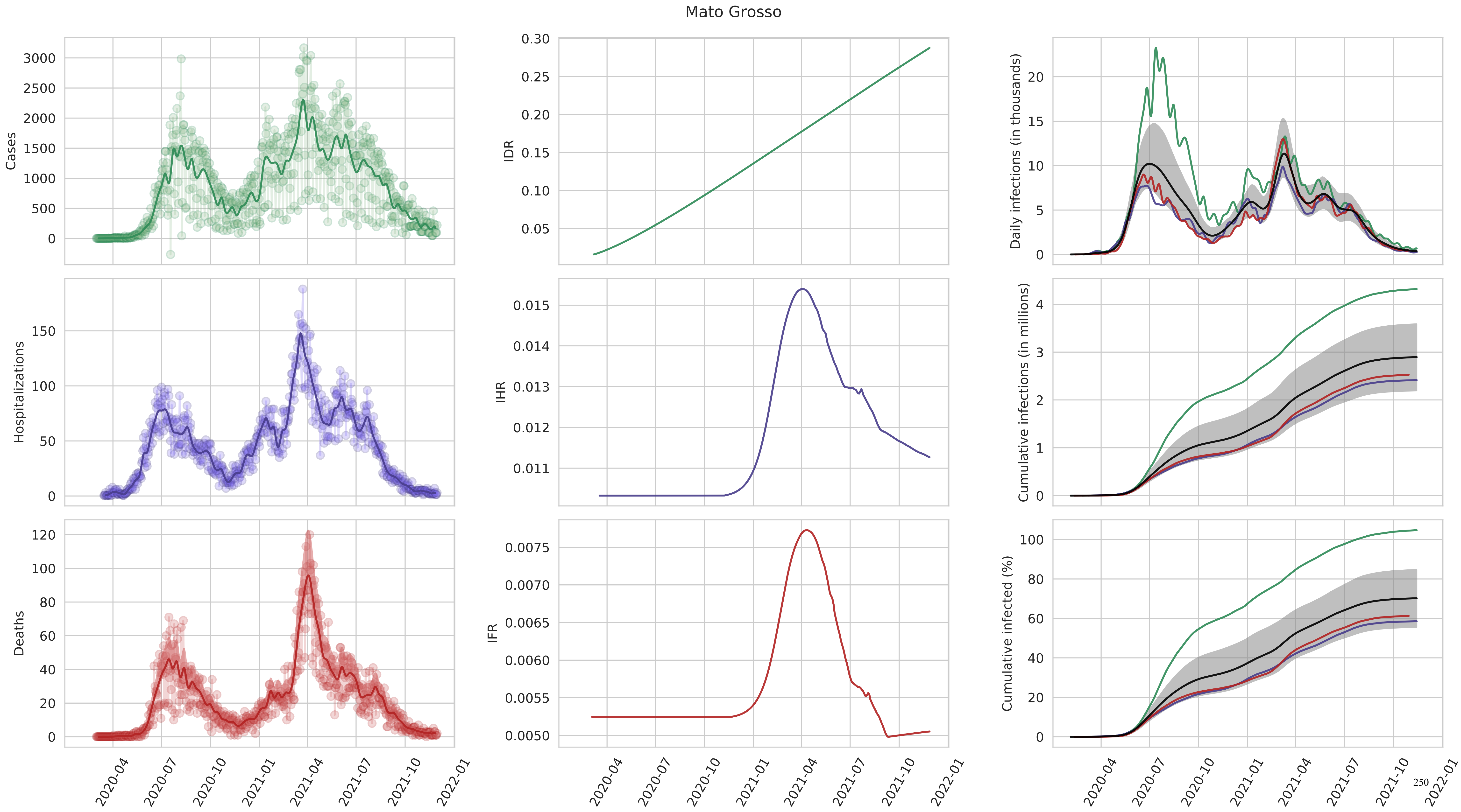
Mato Grosso
Cases
Hospitalizations
Deaths
IDR
IHR
IFR
Daily infections (in thousands)
Cumulative infections (in millions)
Cumulative infected (%)
2020-04
2020-07
2020-10
2021-01
2021-04
2021-07
2021-10
2022-01

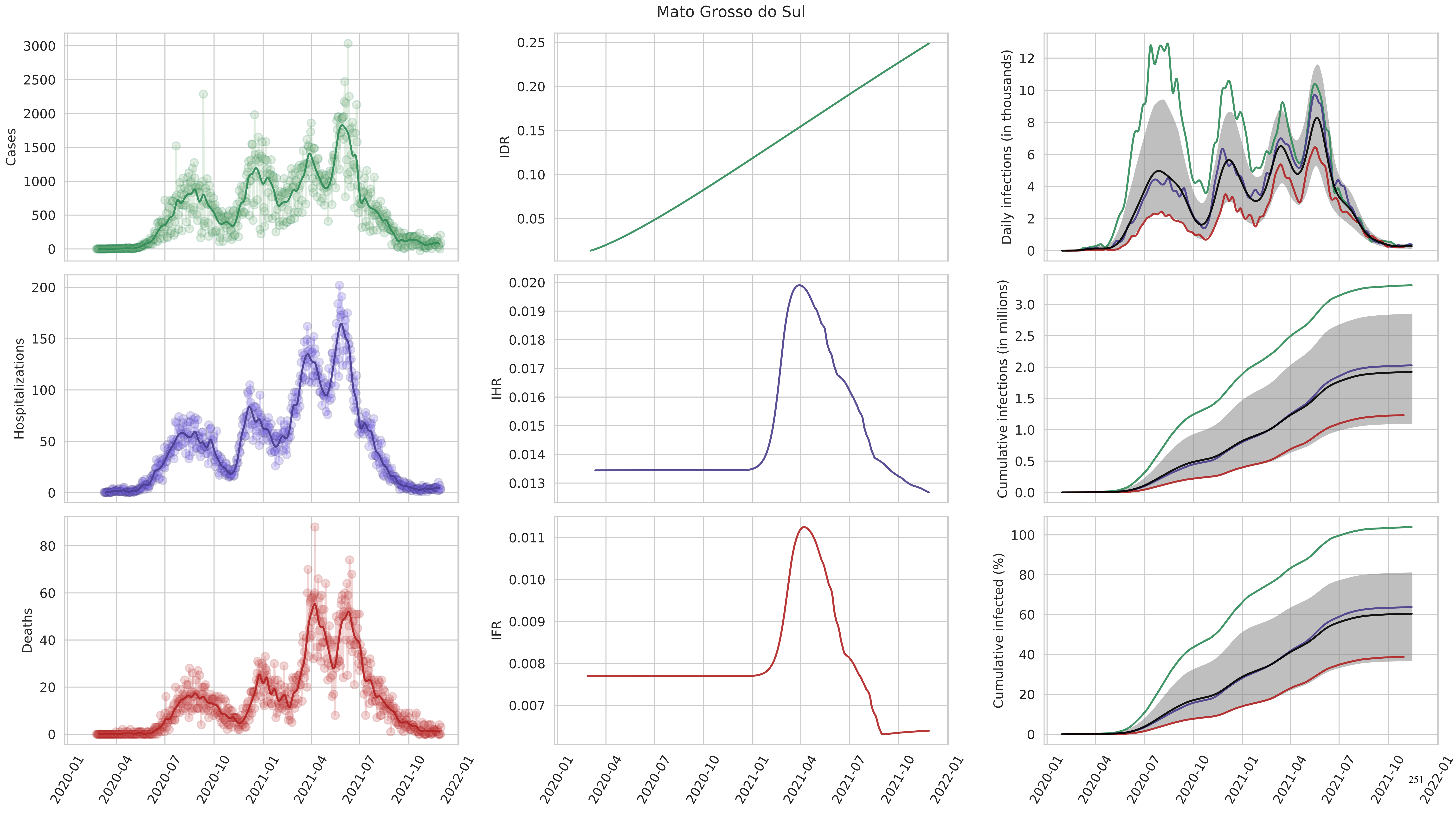

Mato Grosso do Sul
Cases
Hospitalizations
Deaths
IDR
IHR
IFR
Daily infections (in thousands)
Cumulative infections (in millions)
Cumulative infected (%)
2020-01
2020-04
2020-07
2020-10
2021-01
2021-04
2021-07
2021-10
2022-01

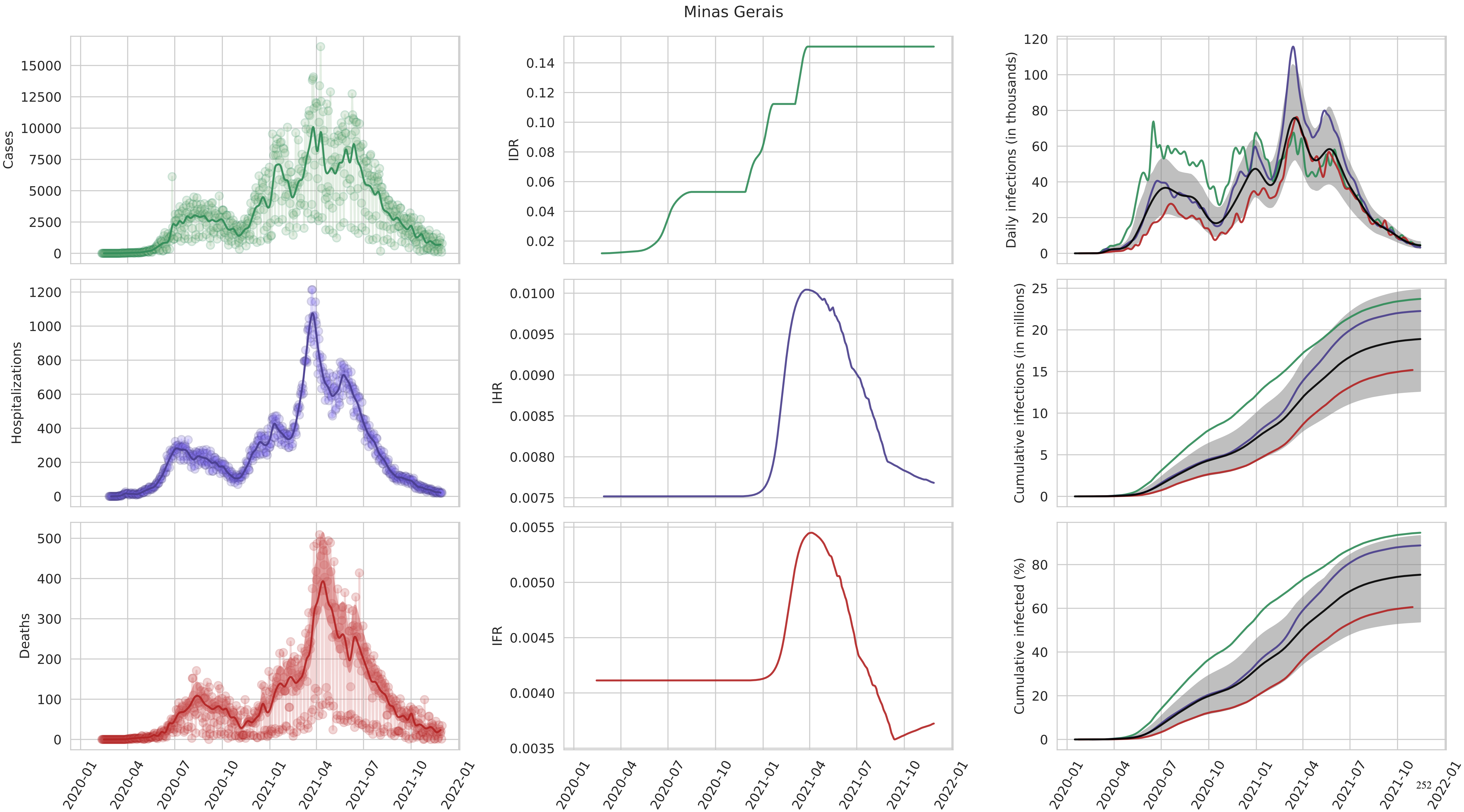
Minas Gerais
Cases
Hospitalizations
Deaths
IDR
IHR
IFR
Daily infections (in thousands)
Cumulative infections (in millions)
Cumulative infected (%)
2020-01
2020-04
2020-07
2020-10
2021-01
2021-04
2021-07
2021-10
2022-01

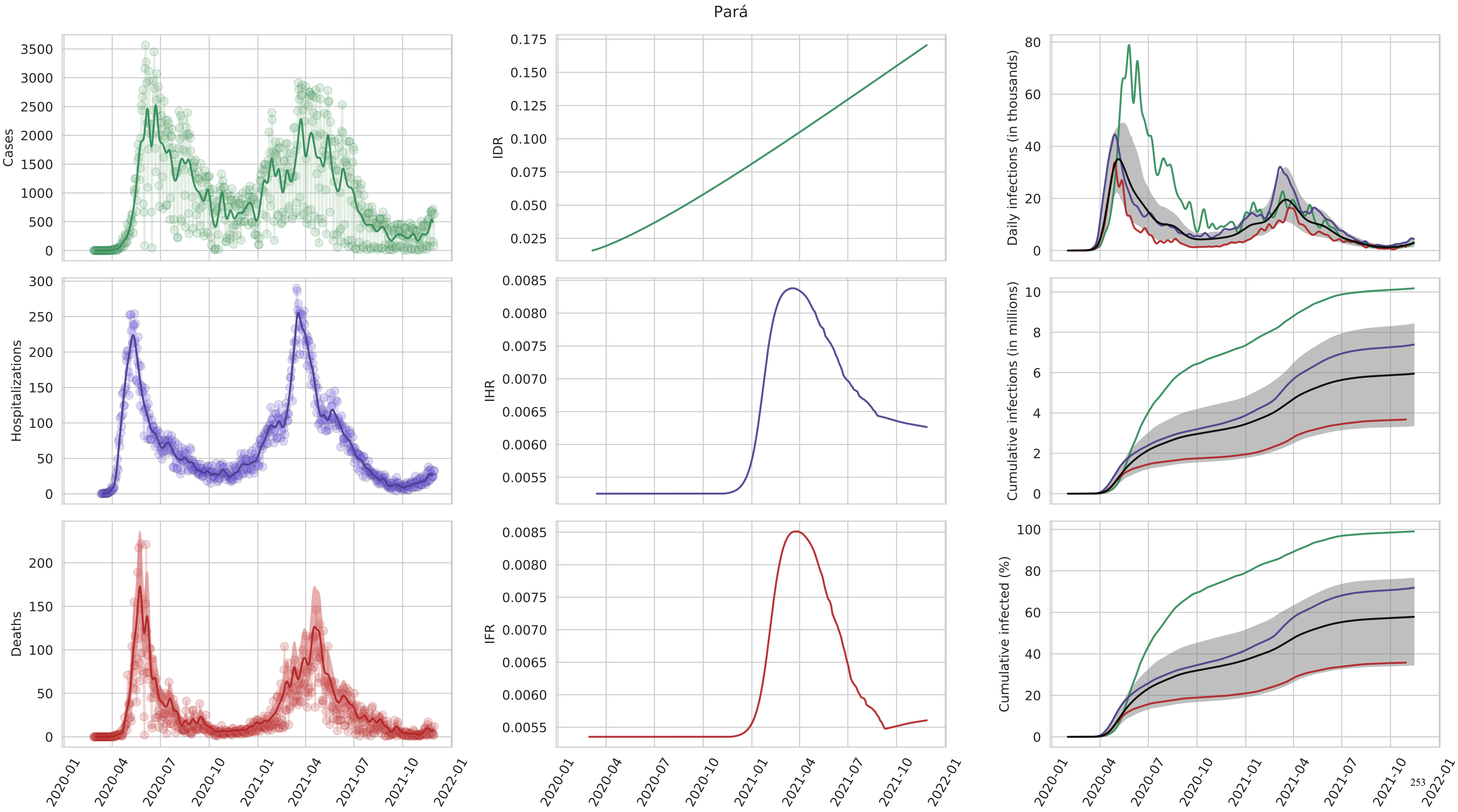
Pará
Cases
Hospitalizations
Deaths
IDR
IHR
IFR
Daily infections (in thousands)
Cumulative infections (in millions)
Cumulative infected (%)
2020-01
2020-04
2020-07
2020-10
2021-01
2021-04
2021-07
2021-10
2022-01

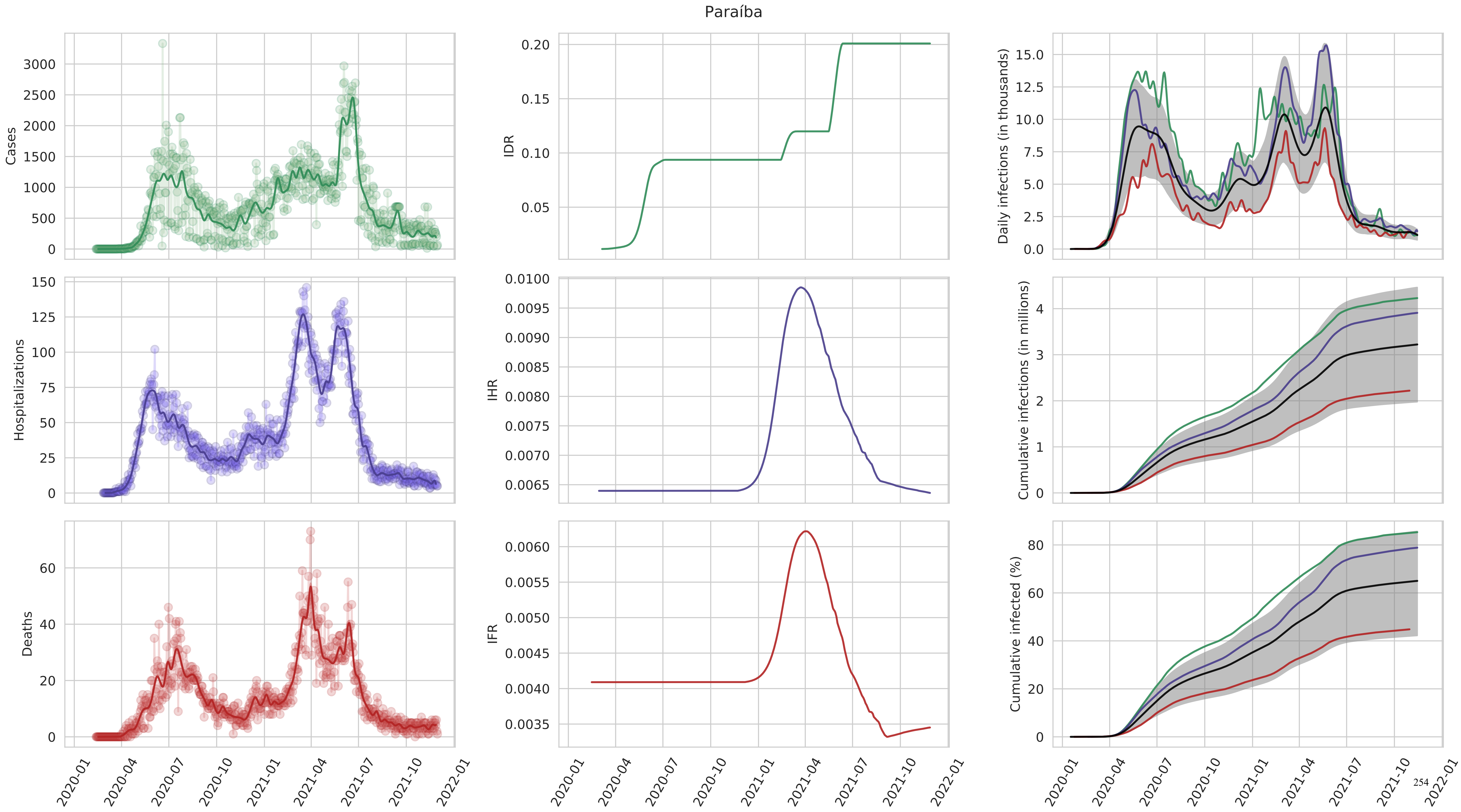
Paraíba
Cases
Hospitalizations
Deaths
IDR
IHR
IFR
Daily infections (in thousands)
Cumulative infections (in millions)
Cumulative infected (%)
2020-01
2020-04
2020-07
2020-10
2021-01
2021-04
2021-07
2021-10
2022-01

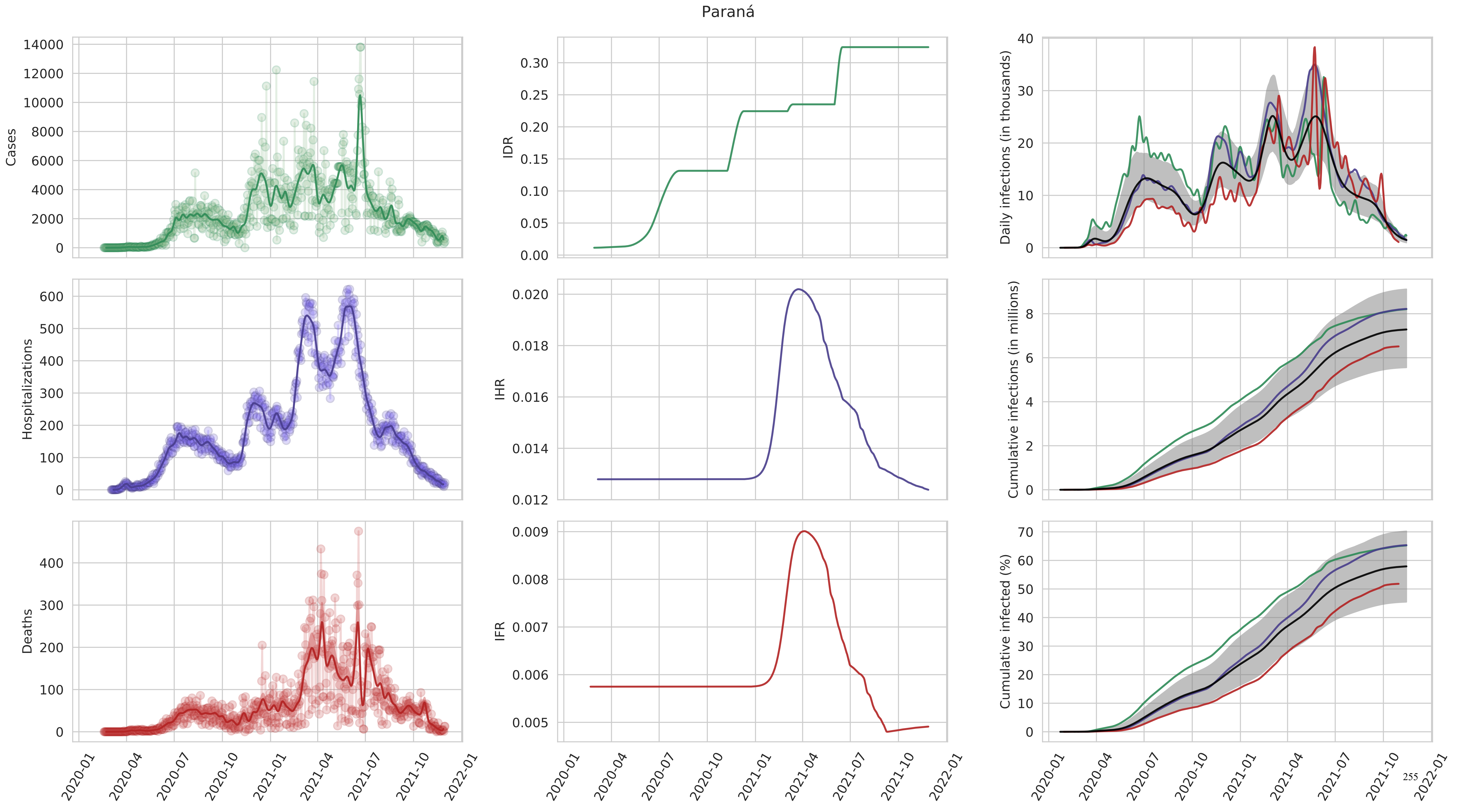
Paraná
Cases
Hospitalizations
Deaths
IDR
IHR
IFR
Daily infections (in thousands)
Cumulative infections (in millions)
Cumulative infected (%)
2020-01
2020-04
2020-07
2020-10
2021-01
2021-04
2021-07
2021-10
2022-01

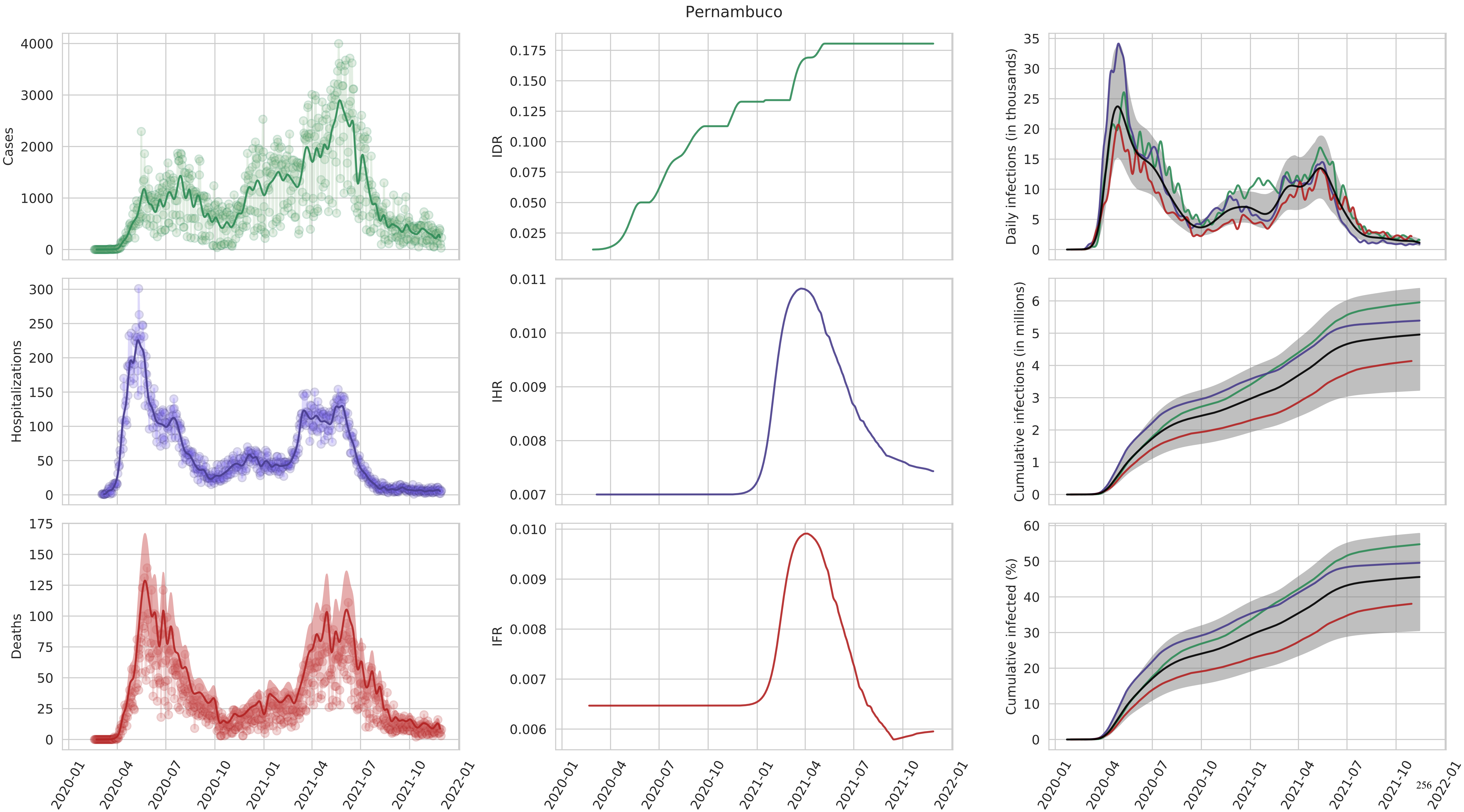

Pernambuco
Cases
IDR
Daily infections (in thousands)
Hospitalizations
IHR
Cumulative infections (in millions)
Deaths
IFR
Cumulative infected (%)

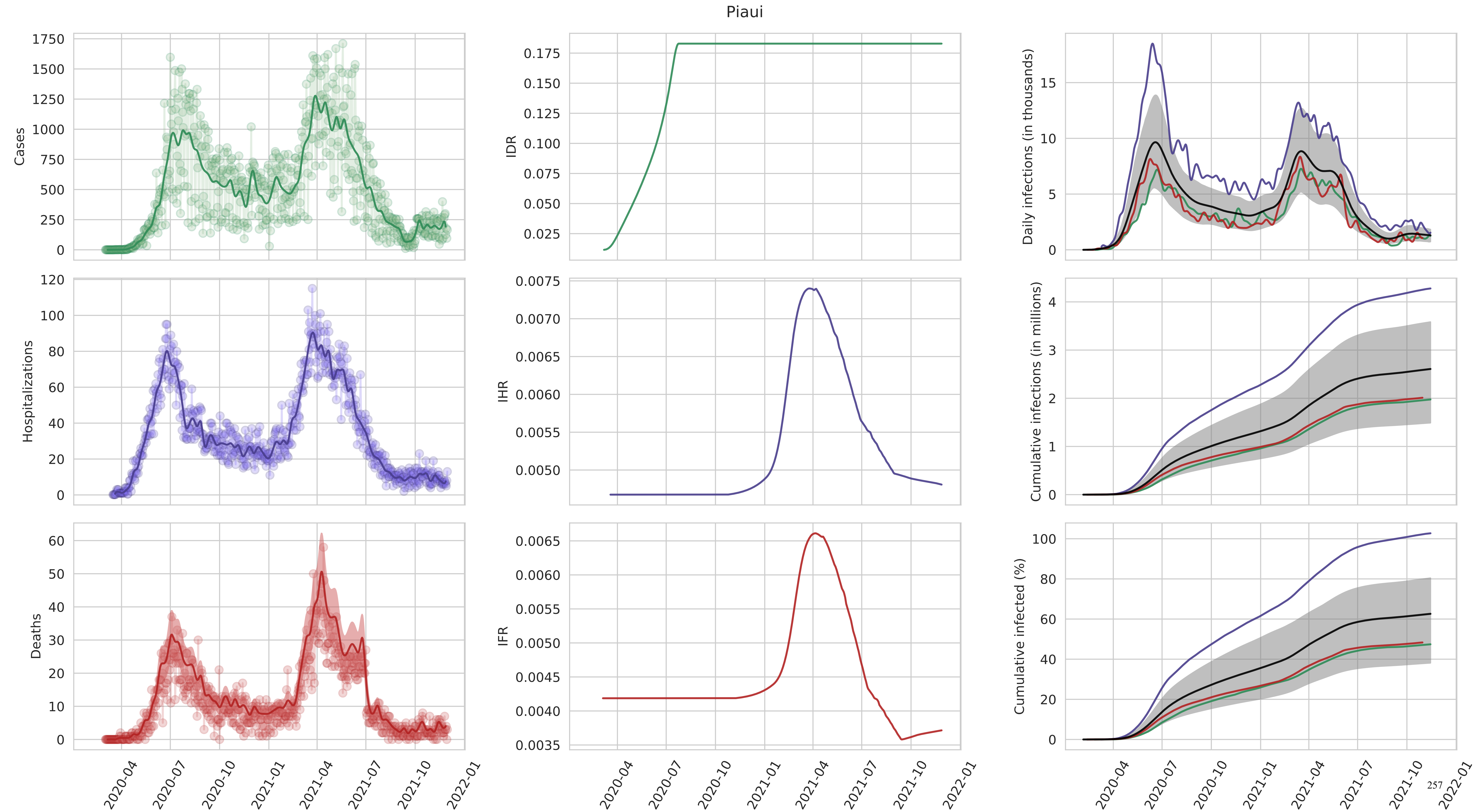

Piaui
Cases
Hospitalizations
Deaths
IDR
IHR
IFR
Daily infections (in thousands)
Cumulative infections (in millions)
Cumulative infected (%)
2020-04
2020-07
2020-10
2021-01
2021-04
2021-07
2021-10
2022-01

Rio de Janeiro

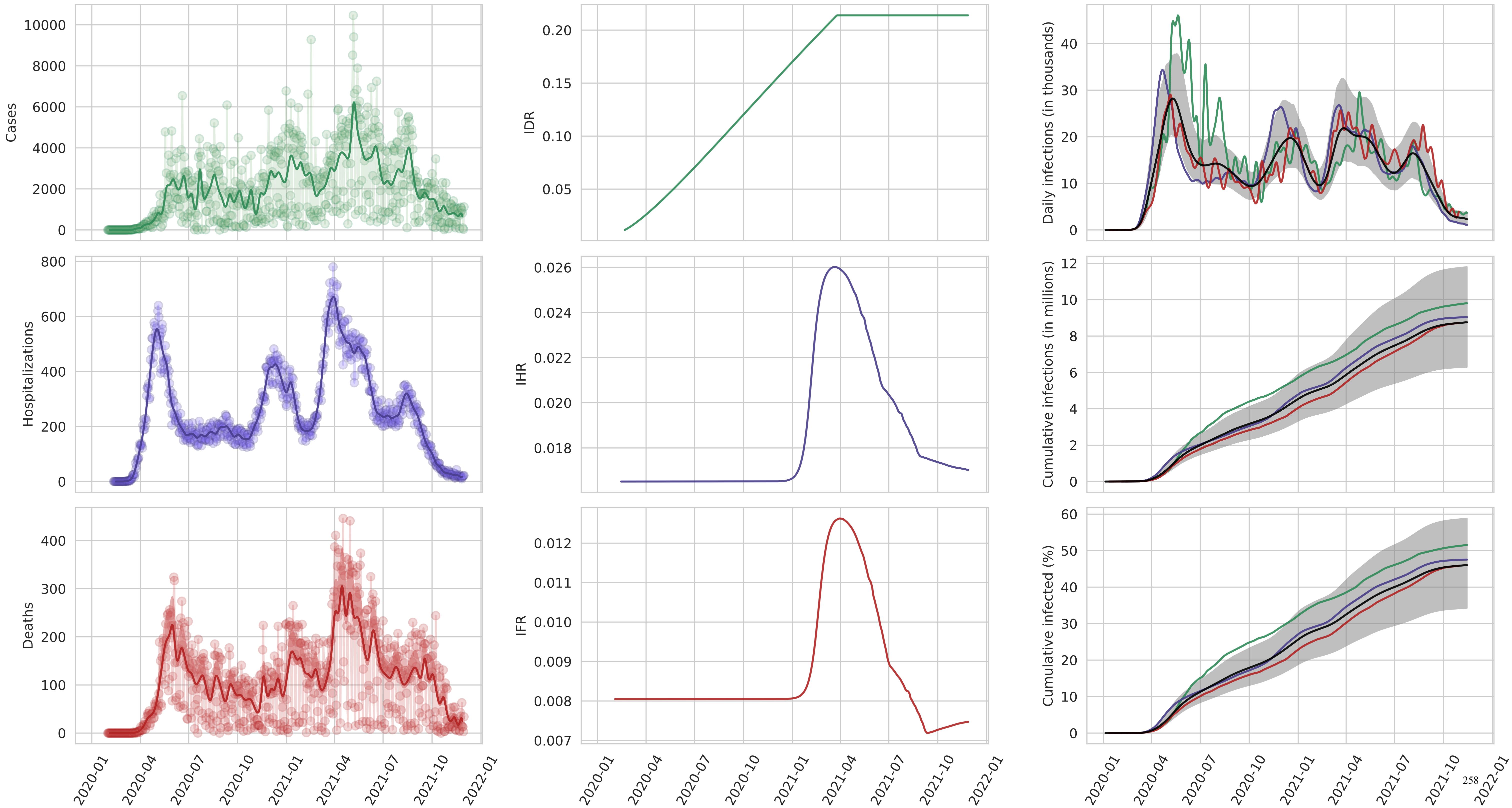

Rio Grande do Norte

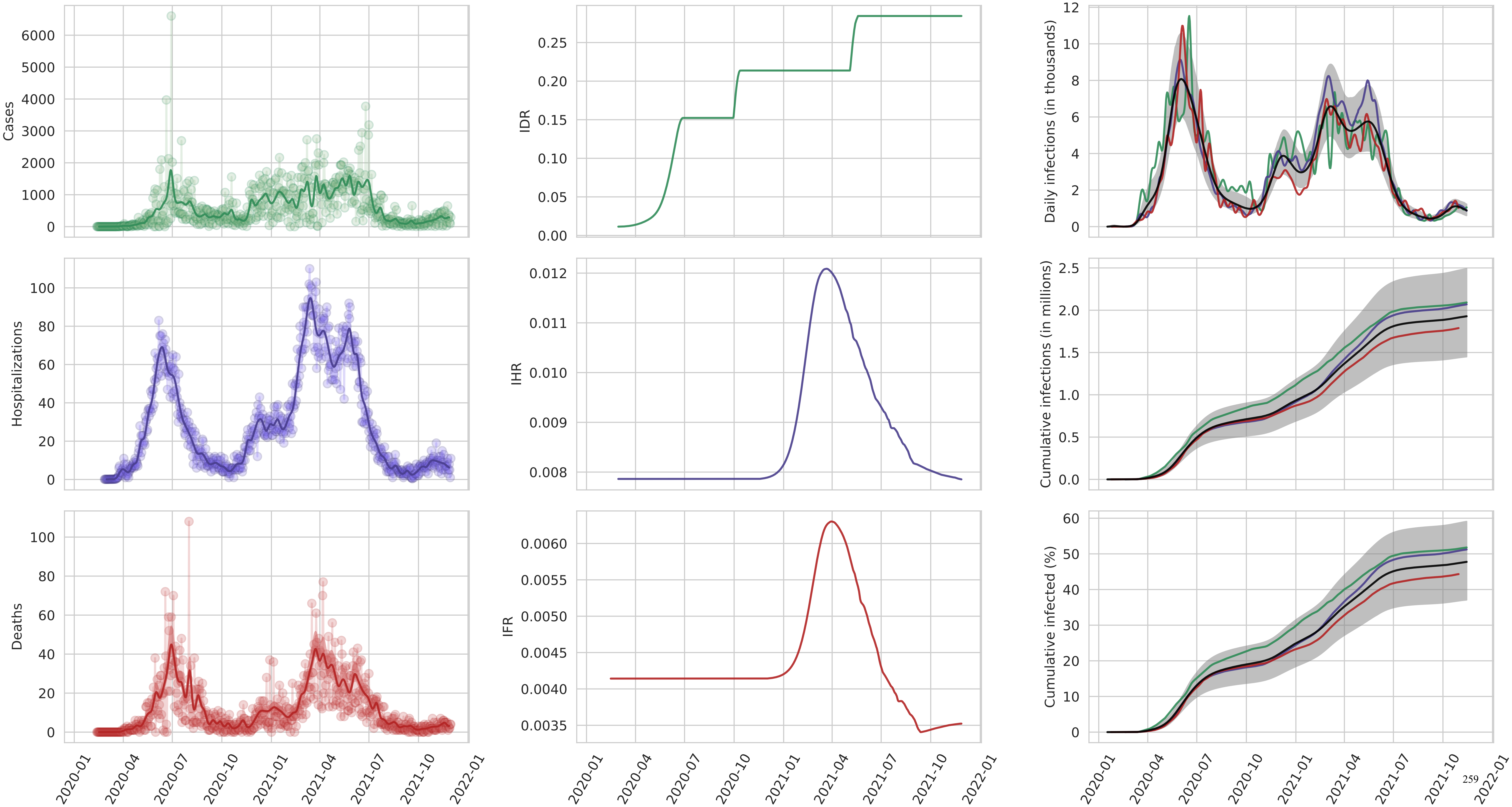

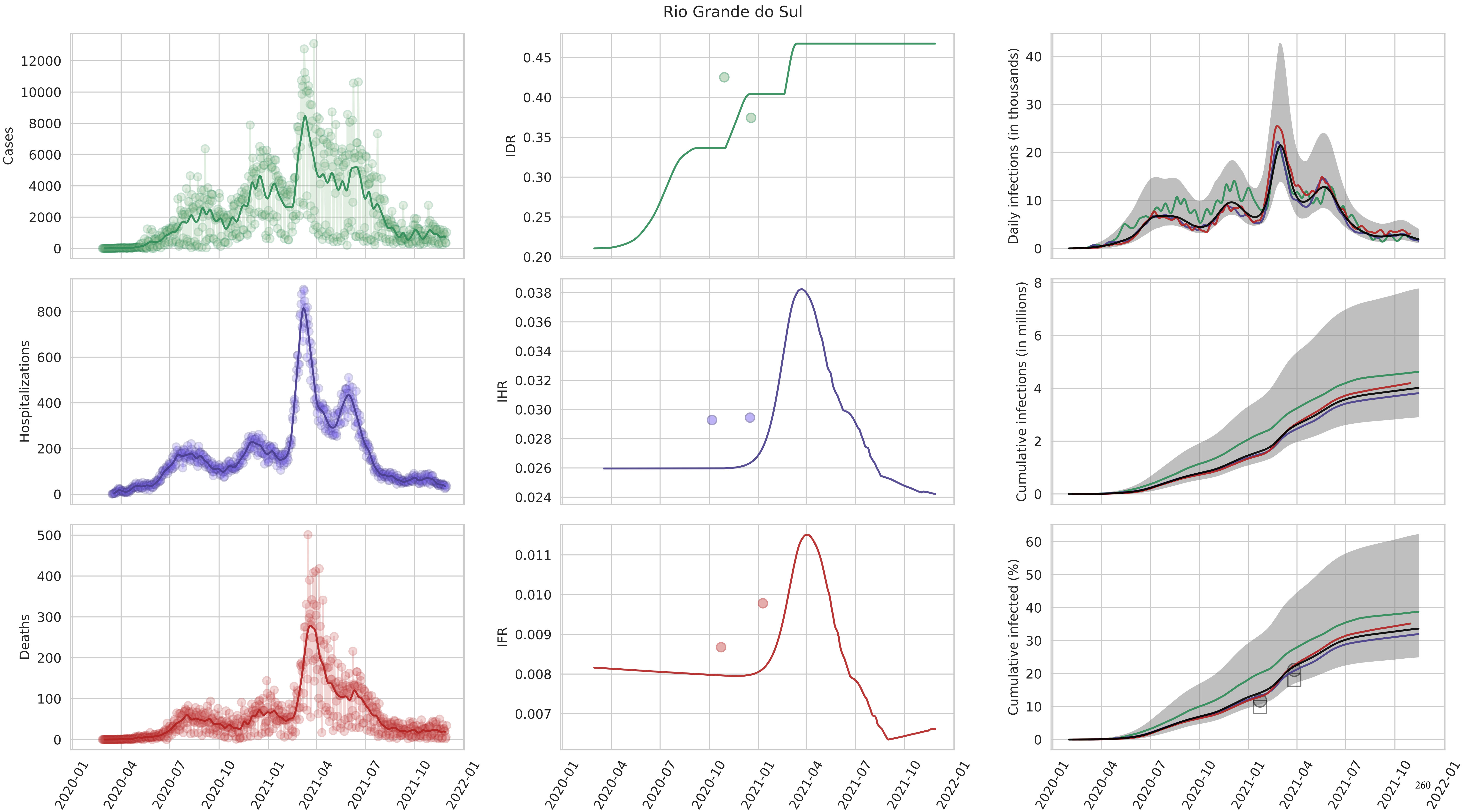

Rio Grande do Sul
Cases
Hospitalizations
Deaths
IDR
IHR
IFR
Daily infections (in thousands)
Cumulative infections (in millions)
Cumulative infected (%)

Rondônia

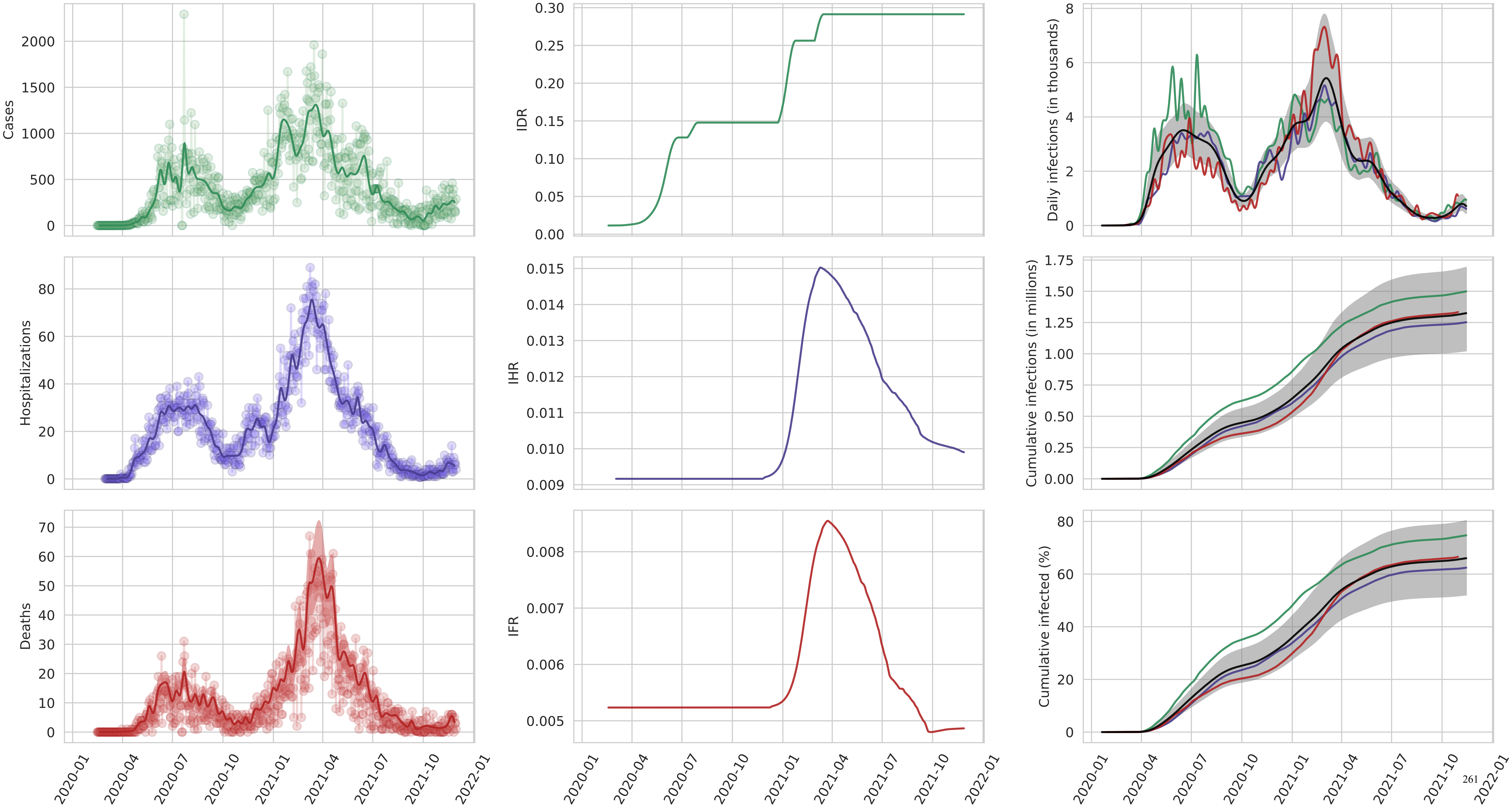

Cases
Hospitalizations
Deaths
IDR
IHR
IFR
Daily infections (in thousands)
Cumulative infections (in millions)
Cumulative infected (%)

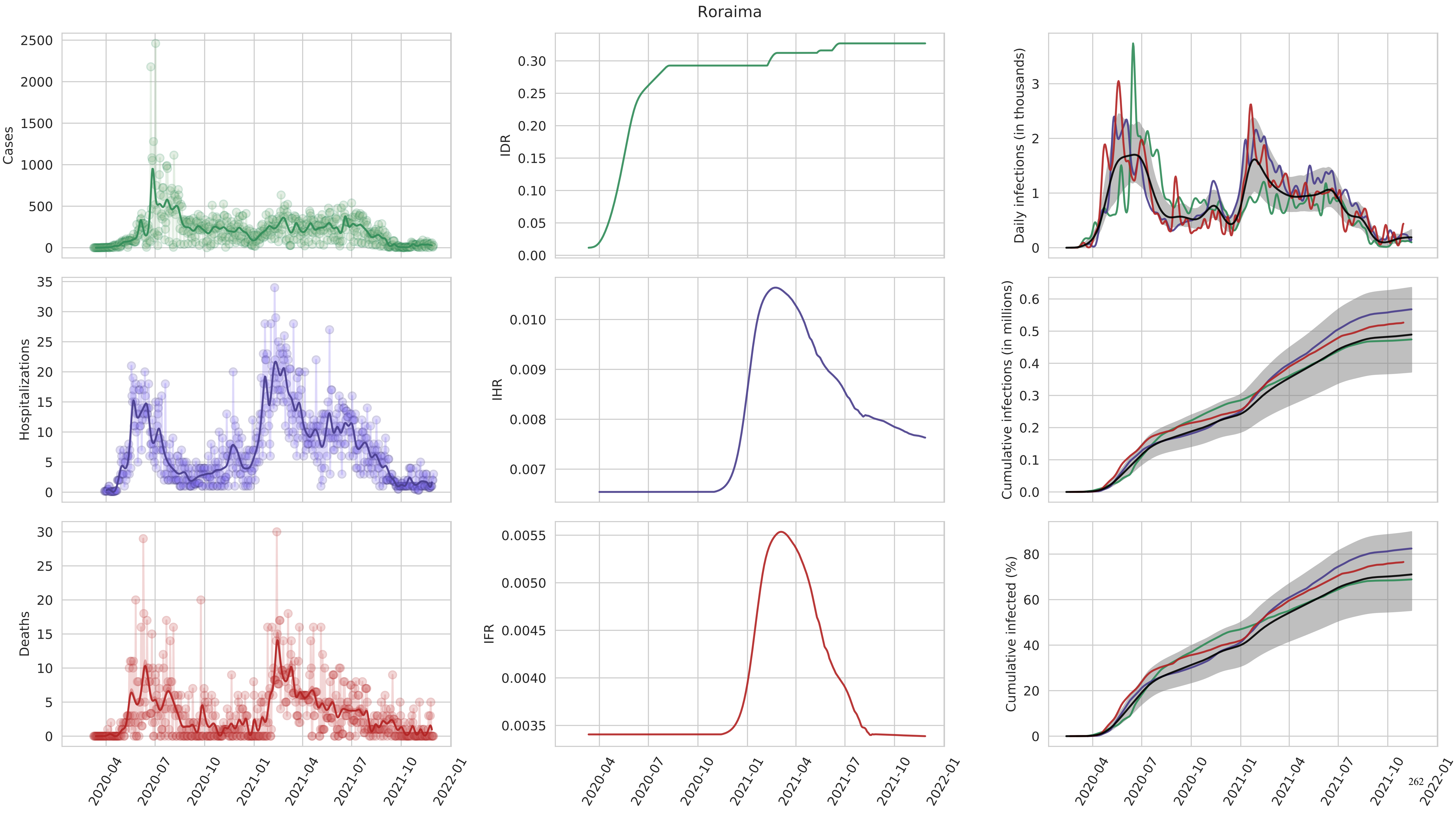

Roraima
Cases
Hospitalizations
Deaths
IDR
IHR
IFR
Daily infections (in thousands)
Cumulative infections (in millions)
Cumulative infected (%)

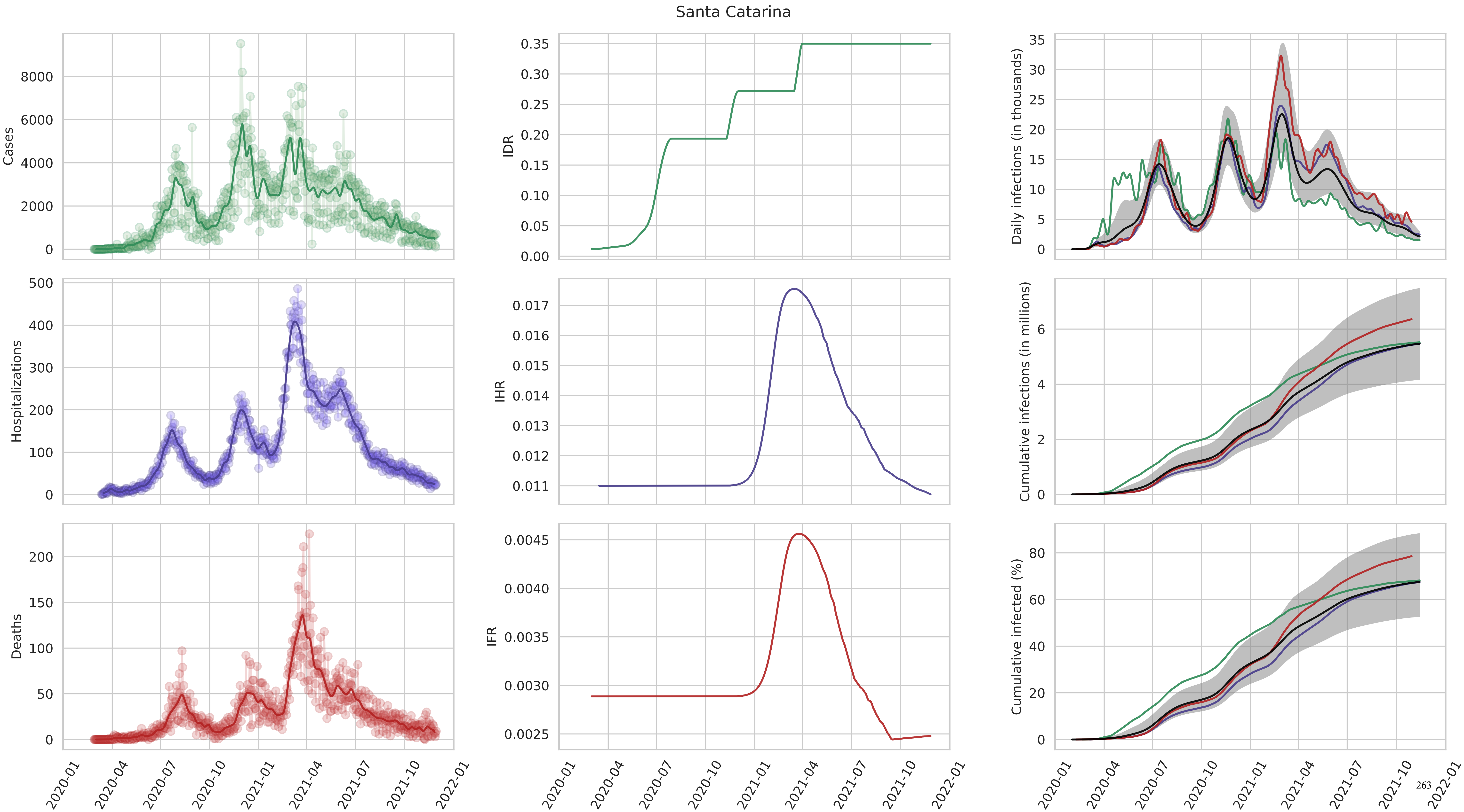
Santa Catarina
Cases
IDR
Daily infections (in thousands)
Hospitalizations
IHR
Cumulative infections (in millions)
Deaths
IFR
Cumulative infected (%)
2020-01
2020-04
2020-07
2020-10
2021-01
2021-04
2021-07
2021-10
2022-01

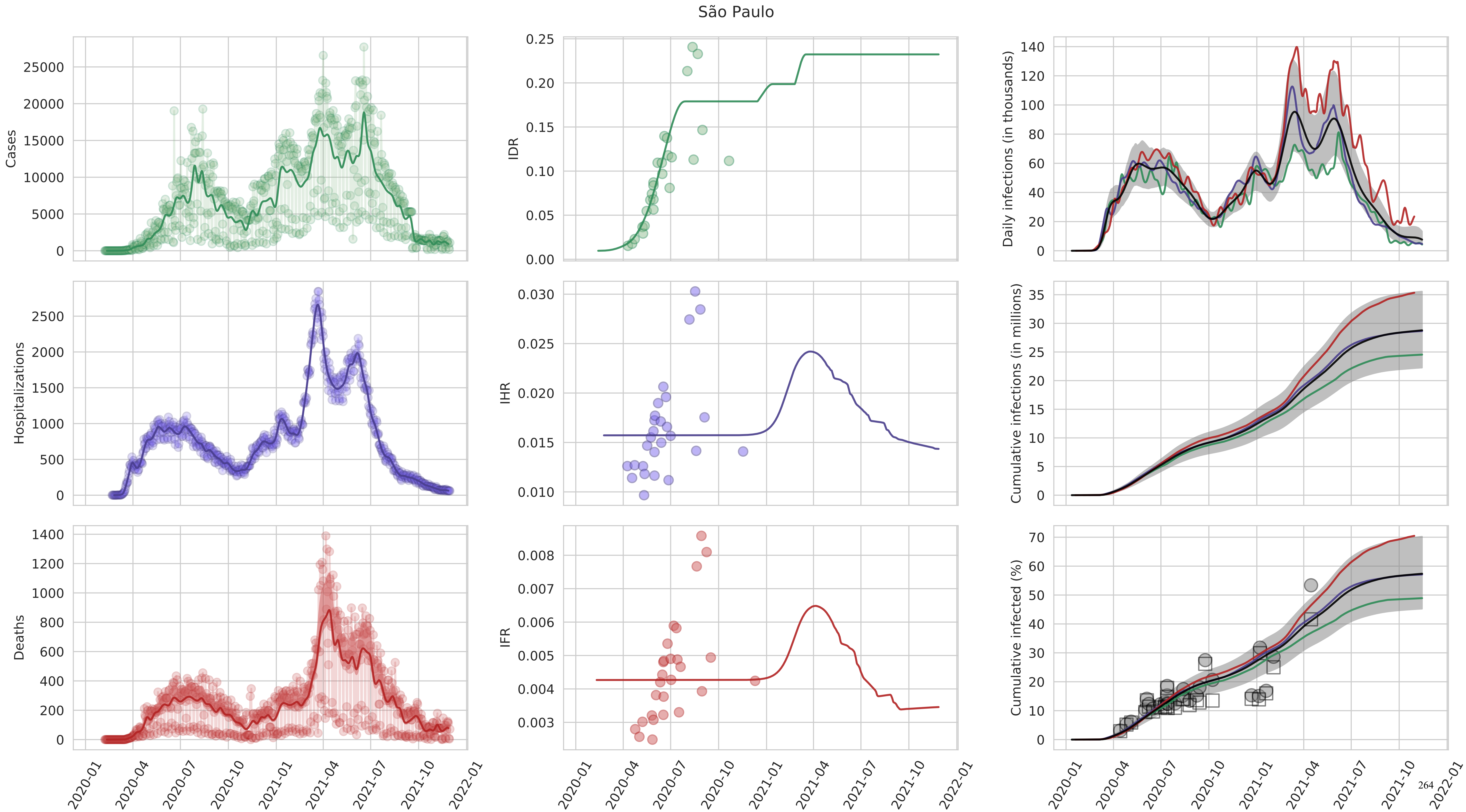
São Paulo
Cases
IDR
Daily infections (in thousands)
Hospitalizations
IHR
Cumulative infections (in millions)
Deaths
IFR
Cumulative infected (%)
2020-01
2020-04
2020-07
2020-10
2021-01
2021-04
2021-07
2021-10
2022-01

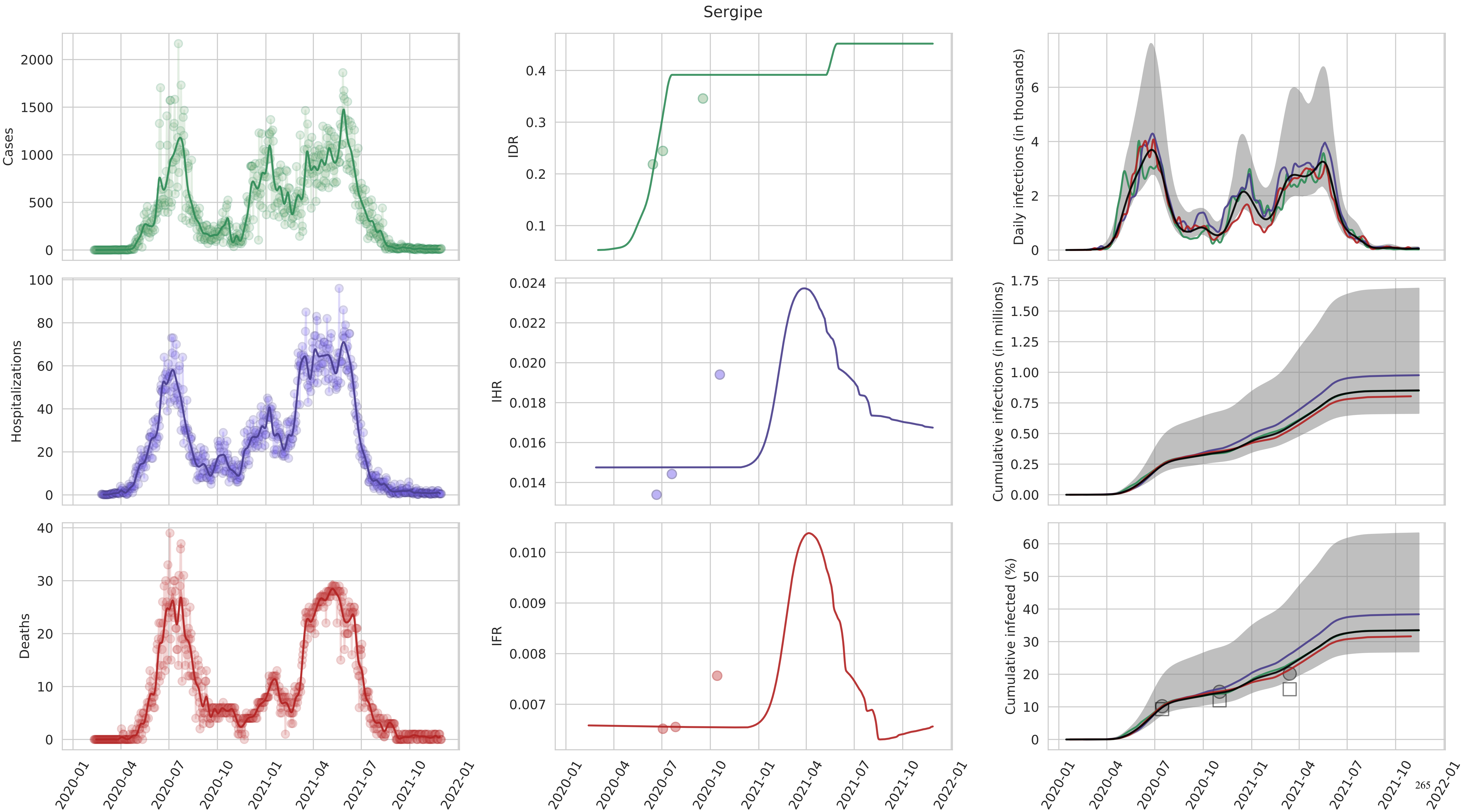

Sergipe
Cases
Hospitalizations
Deaths
IDR
IHR
IFR
Daily infections (in thousands)
Cumulative infections (in millions)
Cumulative infected (%)
2020-01
2020-04
2020-07
2020-10
2021-01
2021-04
2021-07
2021-10
2022-01

Tocantins

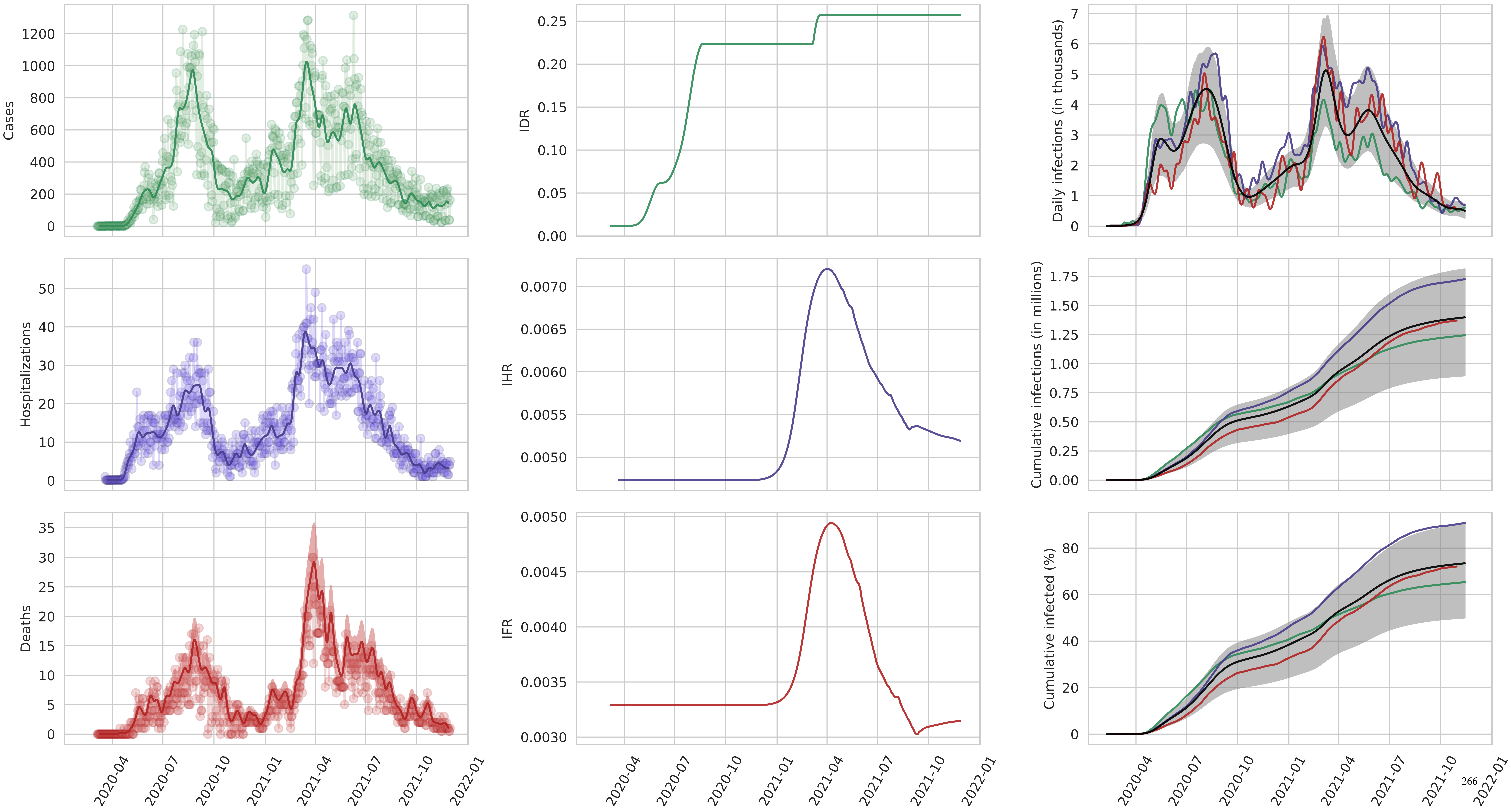

Cases
Hospitalizations
Deaths
IDR
IHR
IFR
Daily infections (in thousands)
Cumulative infections (in millions)
Cumulative infected (%)
2020-04
2020-07
2020-10
2021-01
2021-04
2021-07
2021-10
2022-01

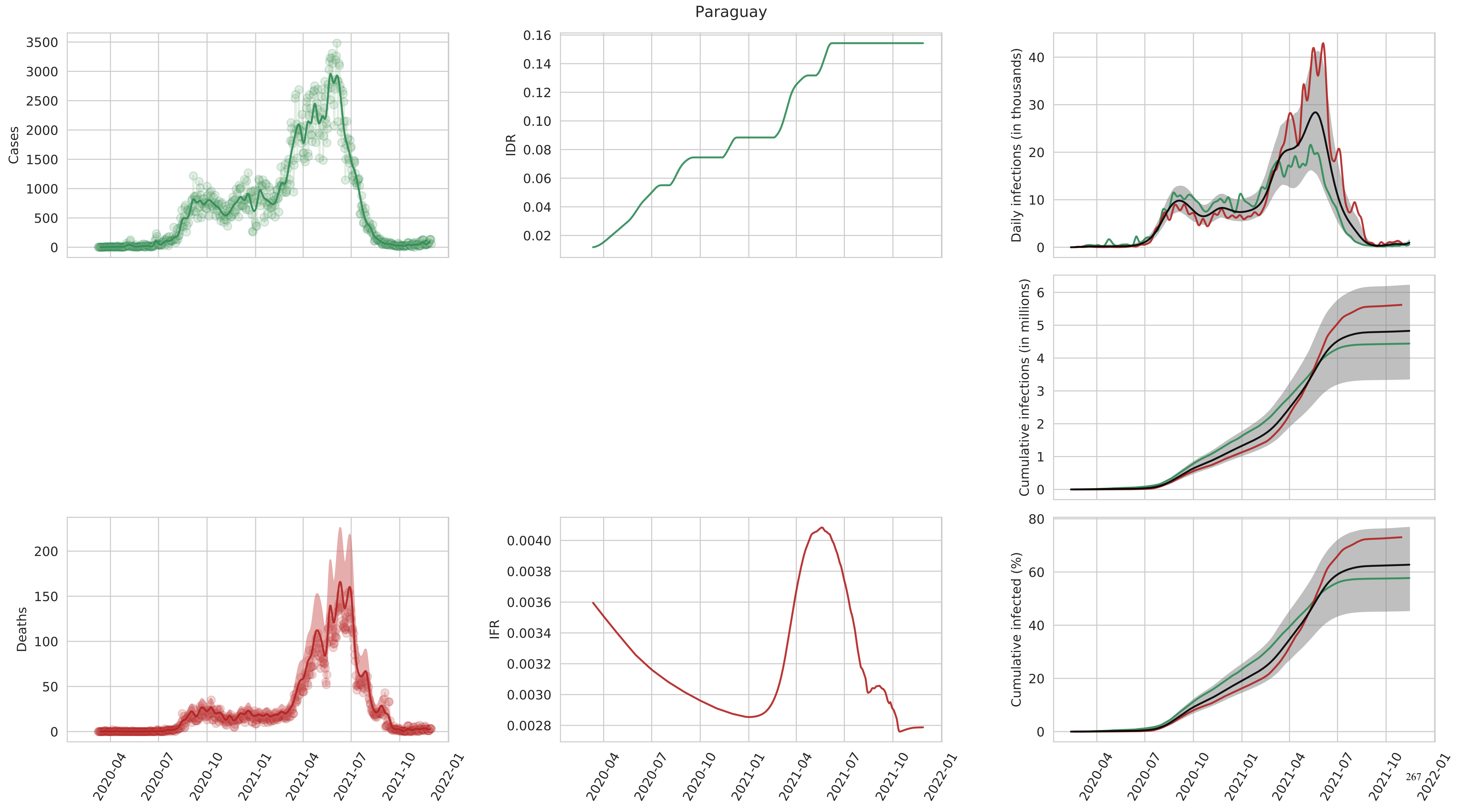
Paraguay
Cases
3500
3000
2500
2000
1500
1000
500
0
IDR
0.16
0.14
0.12
0.10
0.08
0.06
0.04
0.02
Daily infections (in thousands)
40
30
20
10
0
Cumulative infections (in millions)
6
5
4
3
2
1
0
Deaths
200
150
100
50
0
IFR
0.0040
0.0038
0.0036
0.0034
0.0032
0.0030
0.0028
Cumulative infected (%)
80
60
40
20
0
2020-04
2020-07
2020-10
2021-01
2021-04
2021-07
2021-10
2022-01

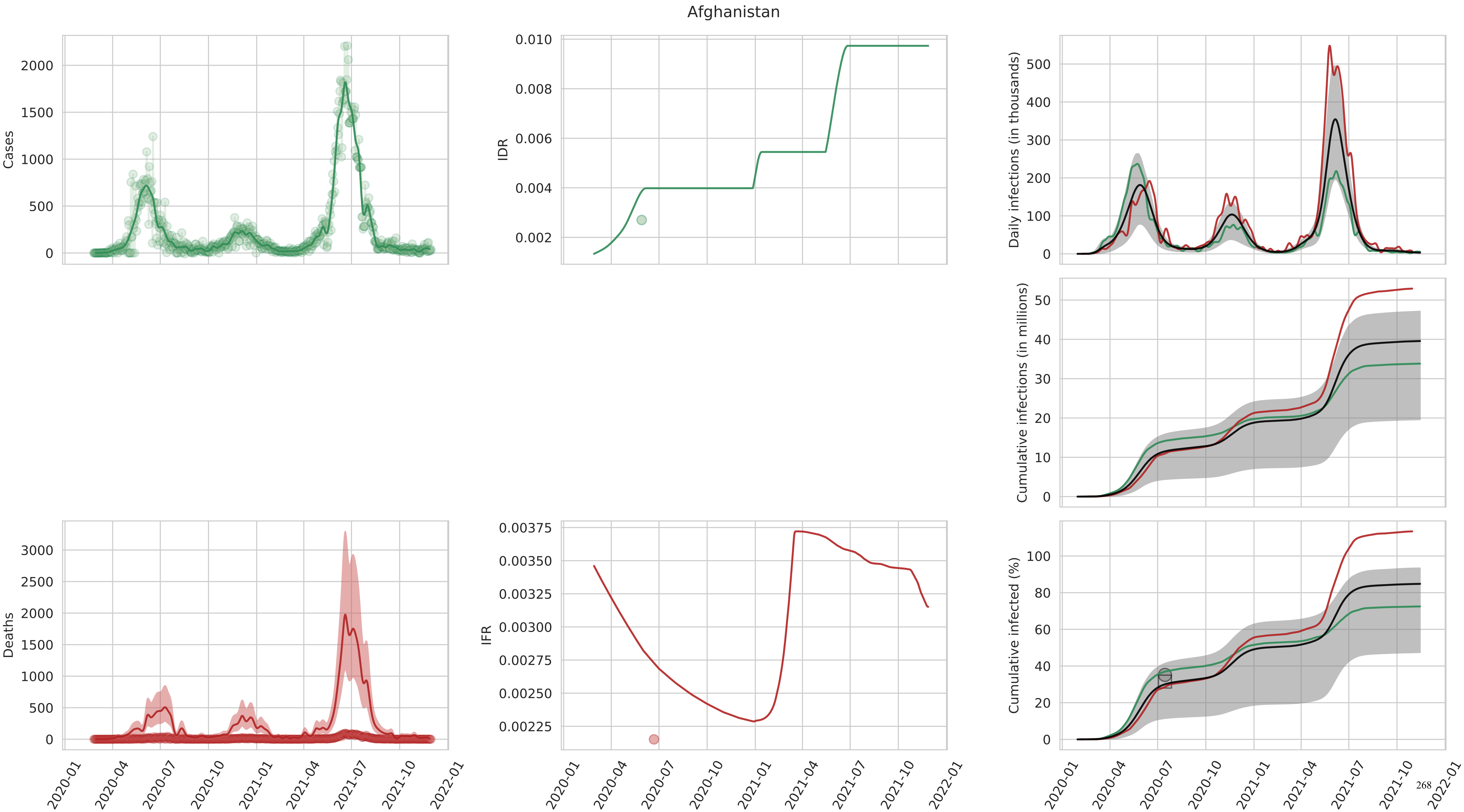
Afghanistan
Cases
IDR
Daily infections (in thousands)
Cumulative infections (in millions)
Deaths
IFR
Cumulative infected (%)
2020-01
2020-04
2020-07
2020-10
2021-01
2021-04
2021-07
2021-10
2022-01

Algeria

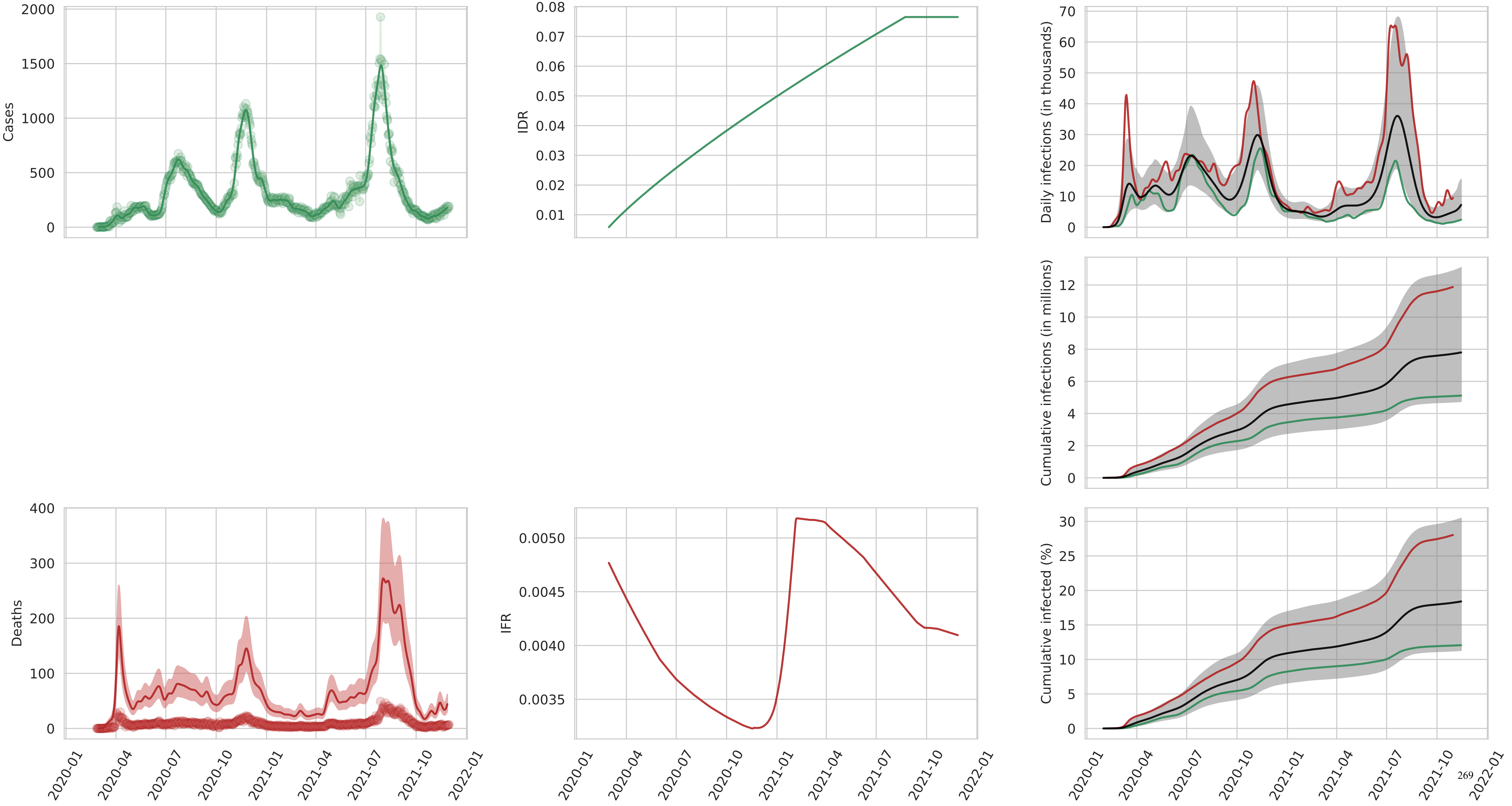

Cases
2000
1500
1000
500
0
IDR
0.08
0.07
0.06
0.05
0.04
0.03
0.02
0.01
Daily infections (in thousands)
70
60
50
40
30
20
10
0
Cumulative infections (in millions)
12
10
8
6
4
2
0
Deaths
400
300
200
100
0
IFR
0.0050
0.0045
0.0040
0.0035
Cumulative infected (%)
30
25
20
15
10
5
0
2020-01
2020-04
2020-07
2020-10
2021-01
2021-04
2021-07
2021-10
2022-01

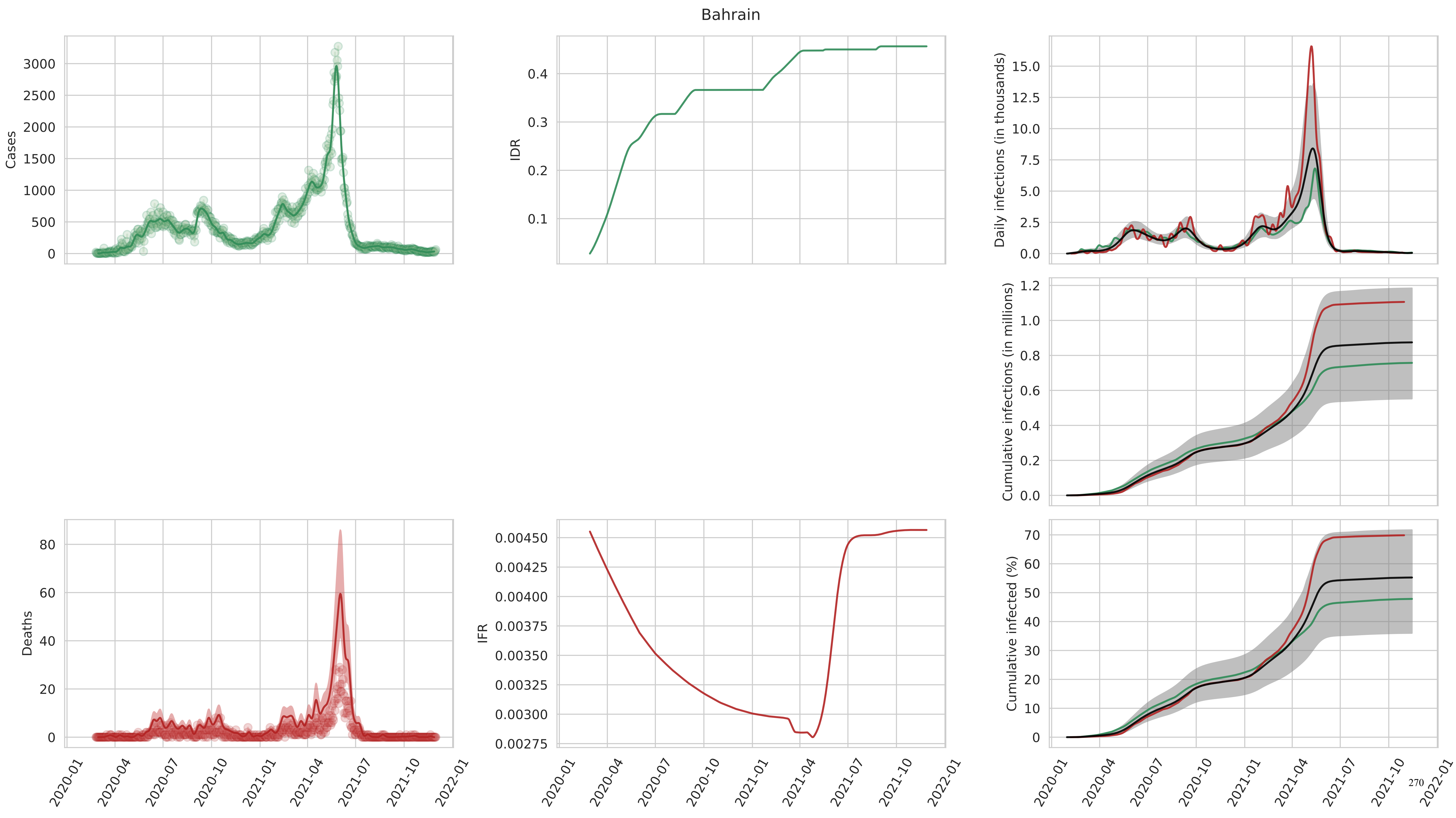
Bahrain
Cases
IDR
Daily infections (in thousands)
Cumulative infections (in millions)
Deaths
IFR
Cumulative infected (%)
2020-01
2020-04
2020-07
2020-10
2021-01
2021-04
2021-07
2021-10
2022-01

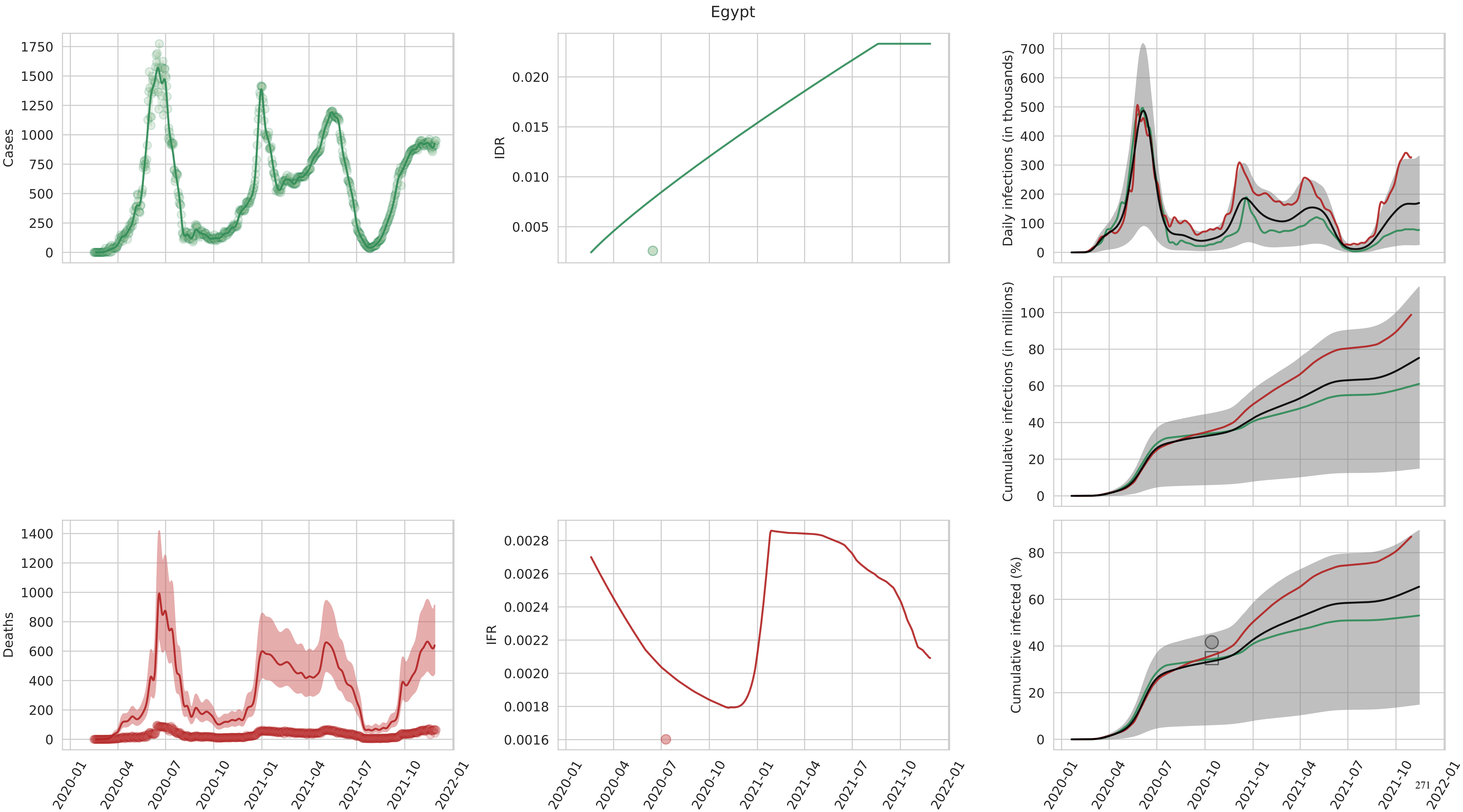

Egypt
Cases
IDR
Daily infections (in thousands)
Cumulative infections (in millions)
Deaths
IFR
Cumulative infected (%)

Iran (Islamic Republic of)

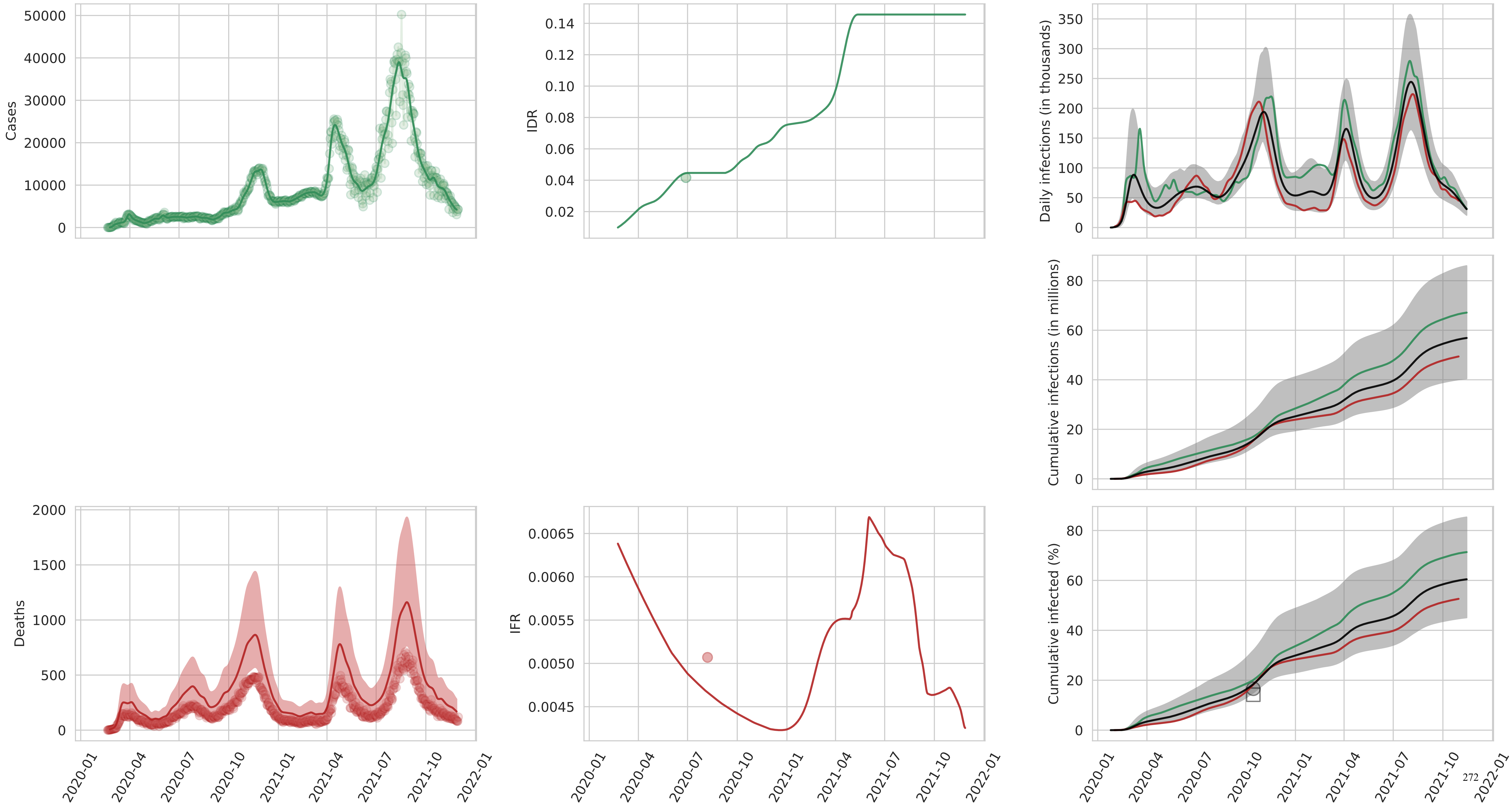

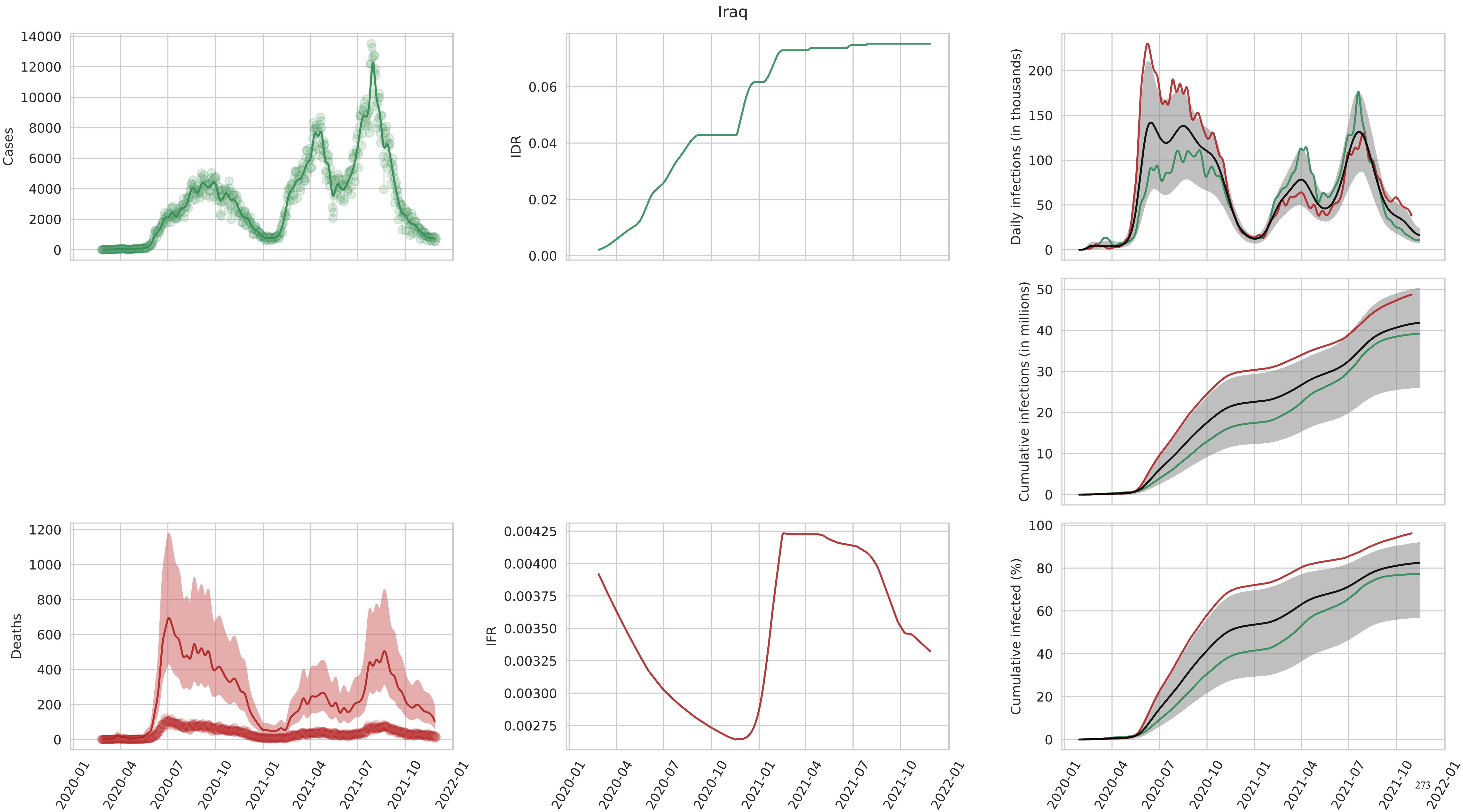

Iraq
Cases
IDR
Daily infections (in thousands)
Cumulative infections (in millions)
Deaths
IFR
Cumulative infected (%)
2020-01
2020-04
2020-07
2020-10
2021-01
2021-04
2021-07
2021-10
2022-01

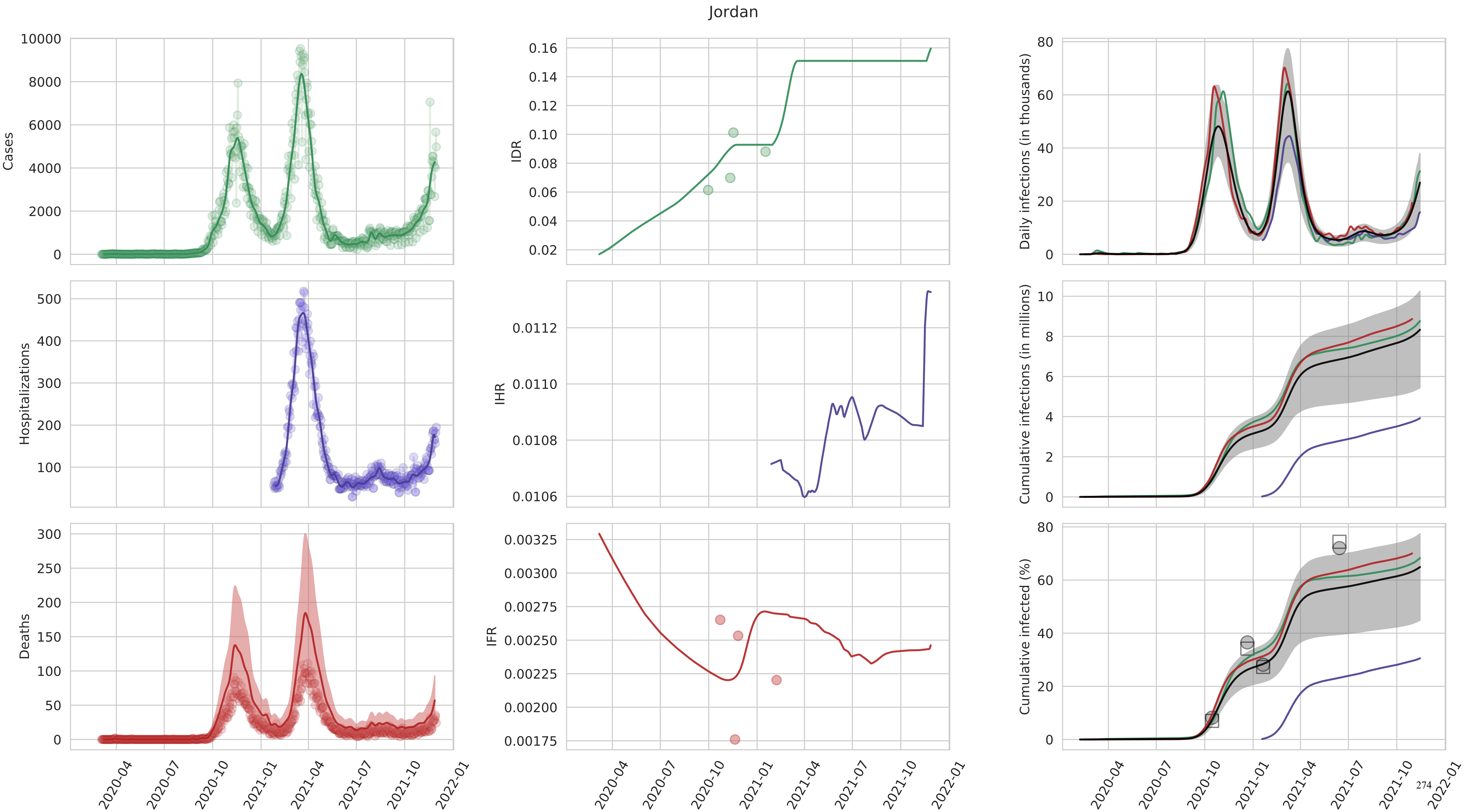

Jordan
Cases
Hospitalizations
Deaths
IDR
IHR
IFR
Daily infections (in thousands)
Cumulative infections (in millions)
Cumulative infected (%)
2020-04
2020-07
2020-10
2021-01
2021-04
2021-07
2021-10
2022-01

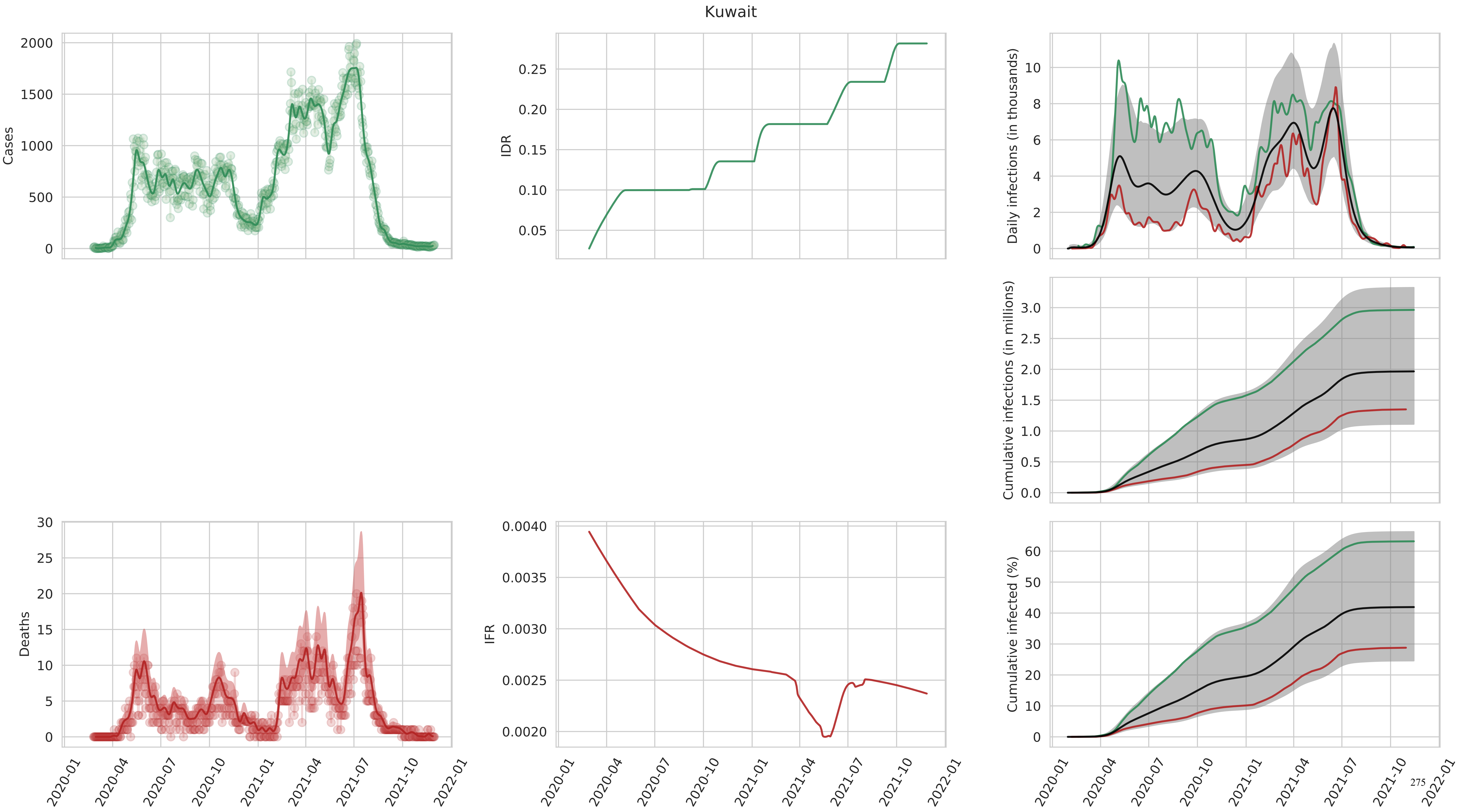
Kuwait
Cases
IDR
Daily infections (in thousands)
Cumulative infections (in millions)
Deaths
IFR
Cumulative infected (%)
2020-01
2020-04
2020-07
2020-10
2021-01
2021-04
2021-07
2021-10
2022-01

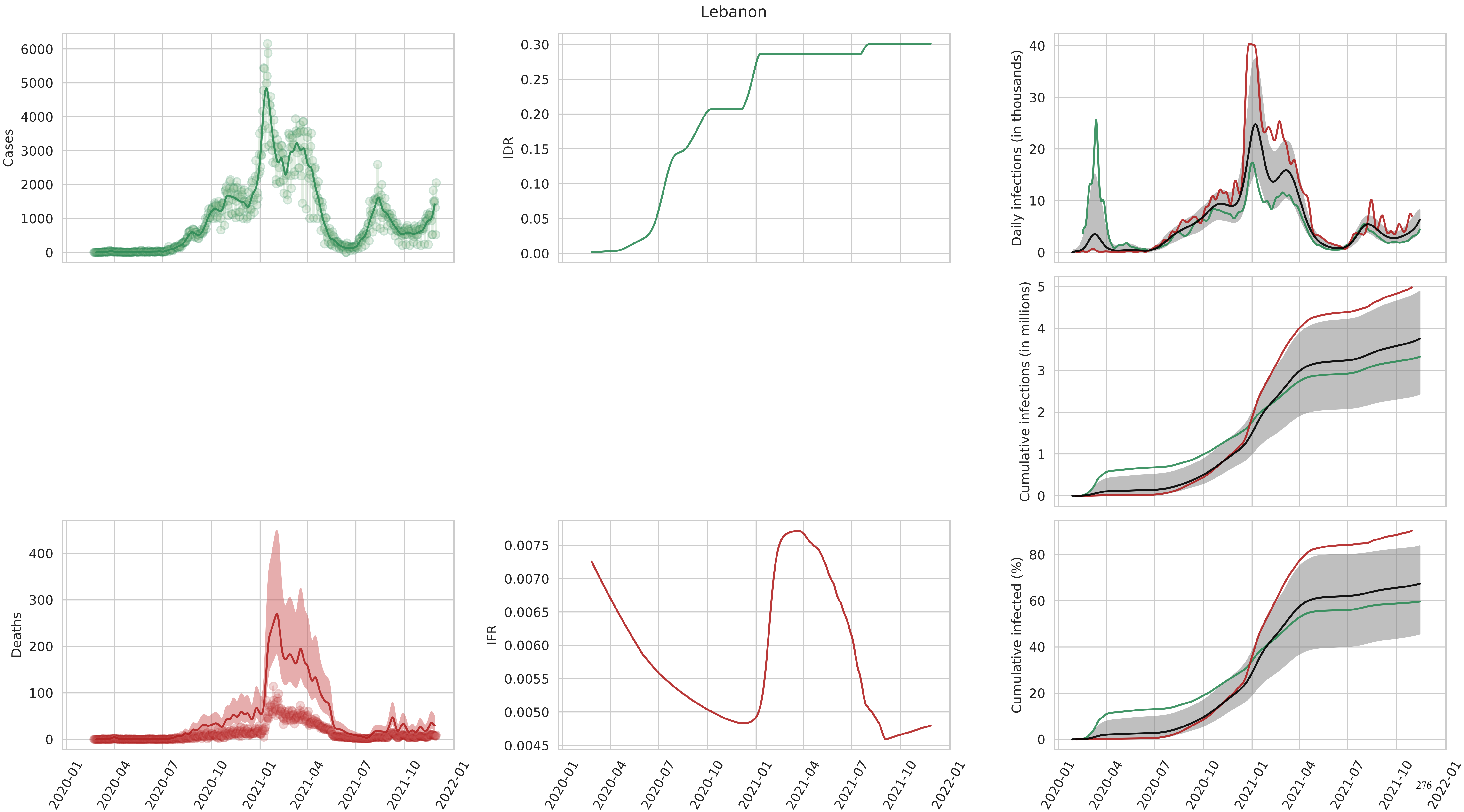
Lebanon
Cases
IDR
Daily infections (in thousands)
Cumulative infections (in millions)
Deaths
IFR
Cumulative infected (%)
2020-01
2020-04
2020-07
2020-10
2021-01
2021-04
2021-07
2021-10
2022-01

Libya

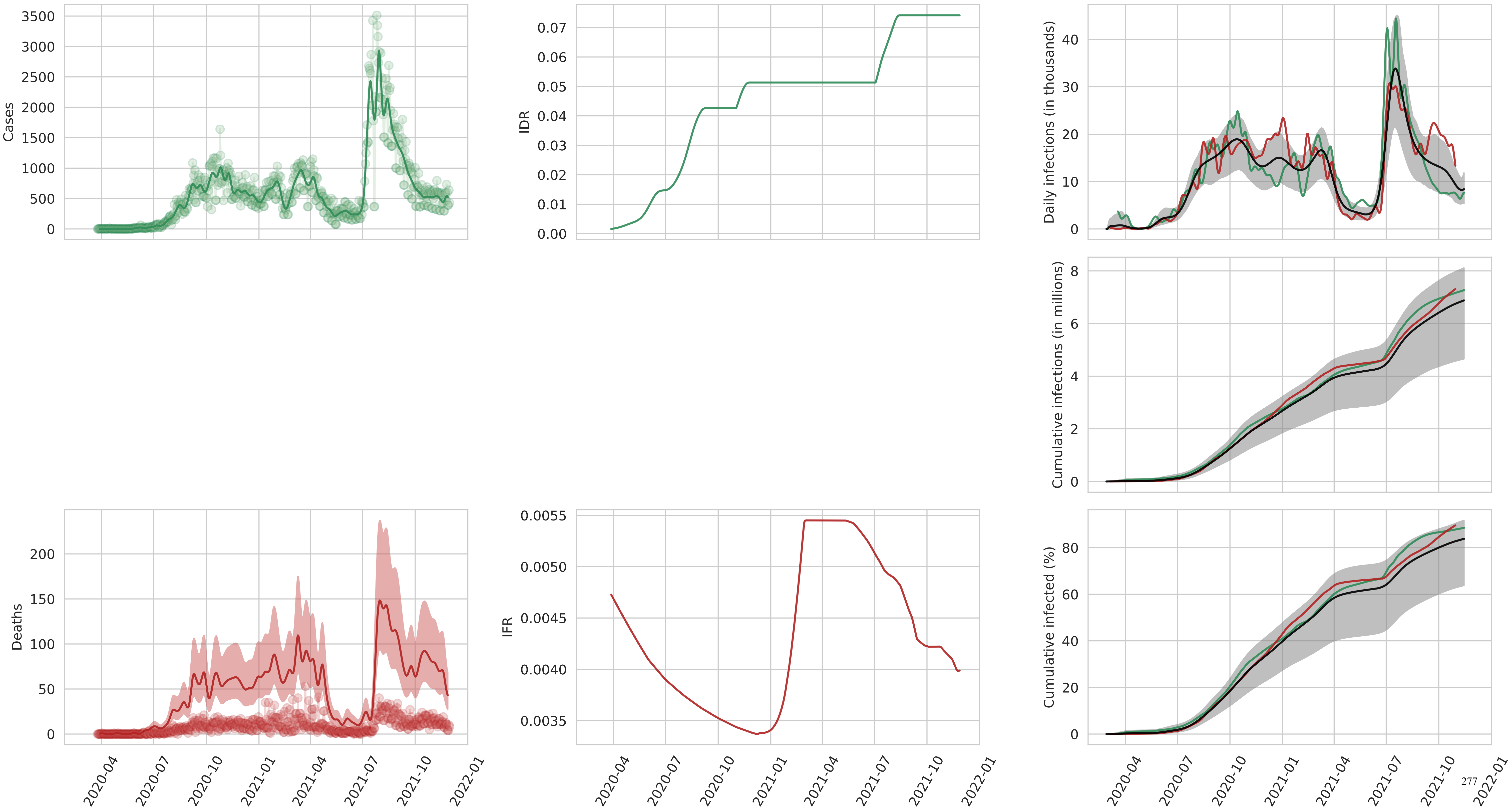

Cases
IDR
Daily infections (in thousands)
Cumulative infections (in millions)
Deaths
IFR
Cumulative infected (%)
2020-04
2020-07
2020-10
2021-01
2021-04
2021-07
2021-10
2022-01

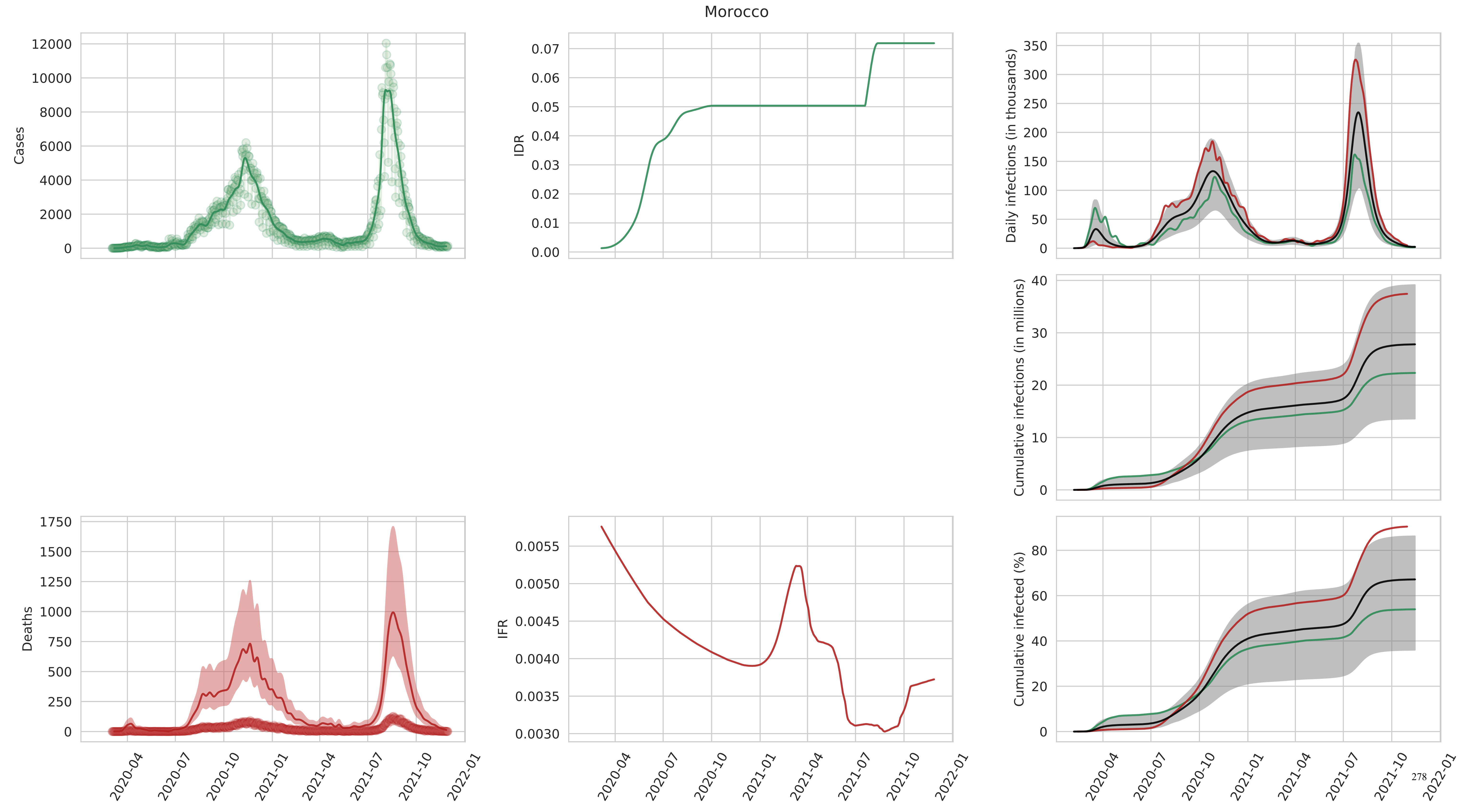

Morocco
Cases
IDR
Daily infections (in thousands)
Cumulative infections (in millions)
Deaths
IFR
Cumulative infected (%)
2020-04
2020-07
2020-10
2021-01
2021-04
2021-07
2021-10
2022-01

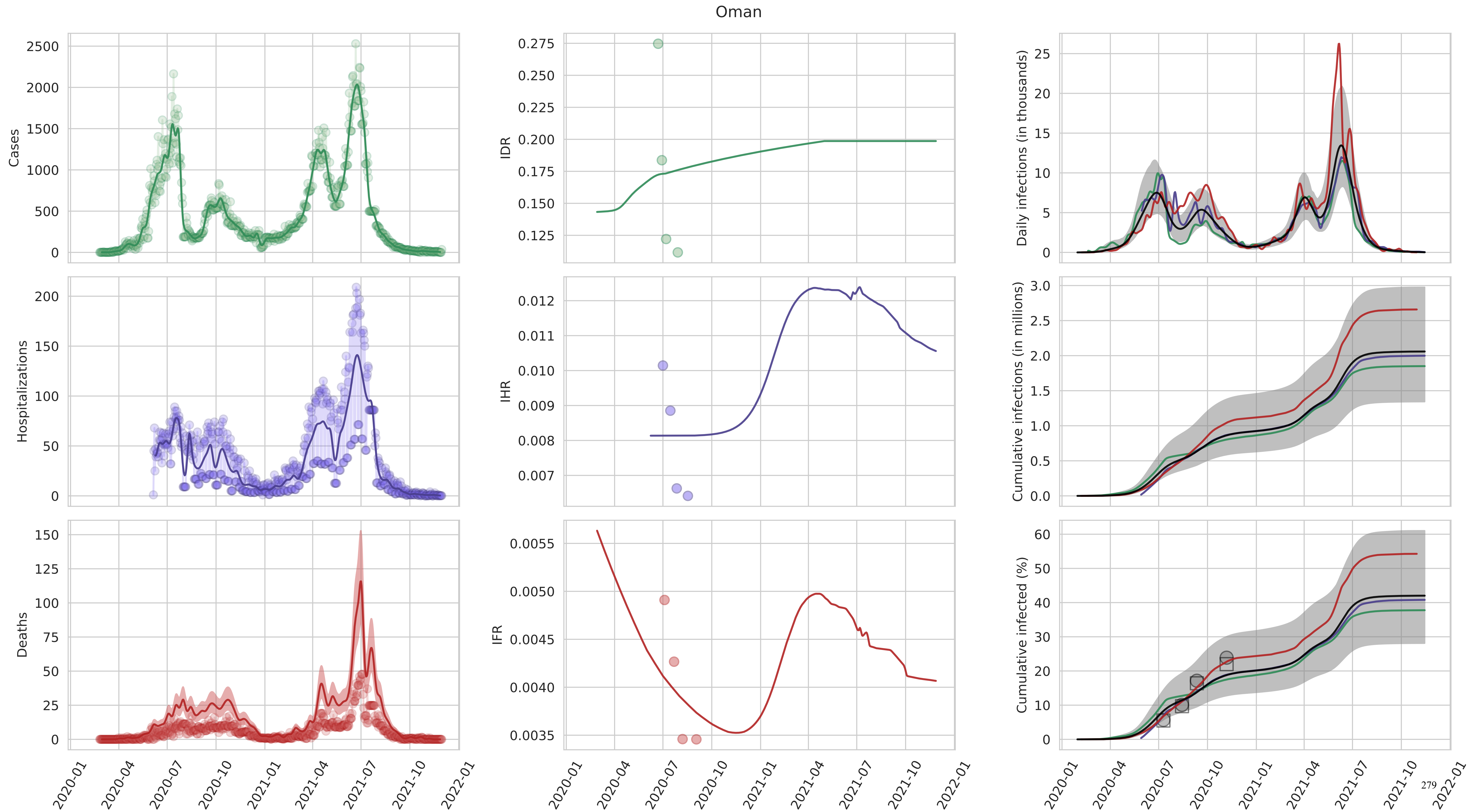
Oman
Cases
IDR
Daily infections (in thousands)
Hospitalizations
IHR
Cumulative infections (in millions)
Deaths
IFR
Cumulative infected (%)

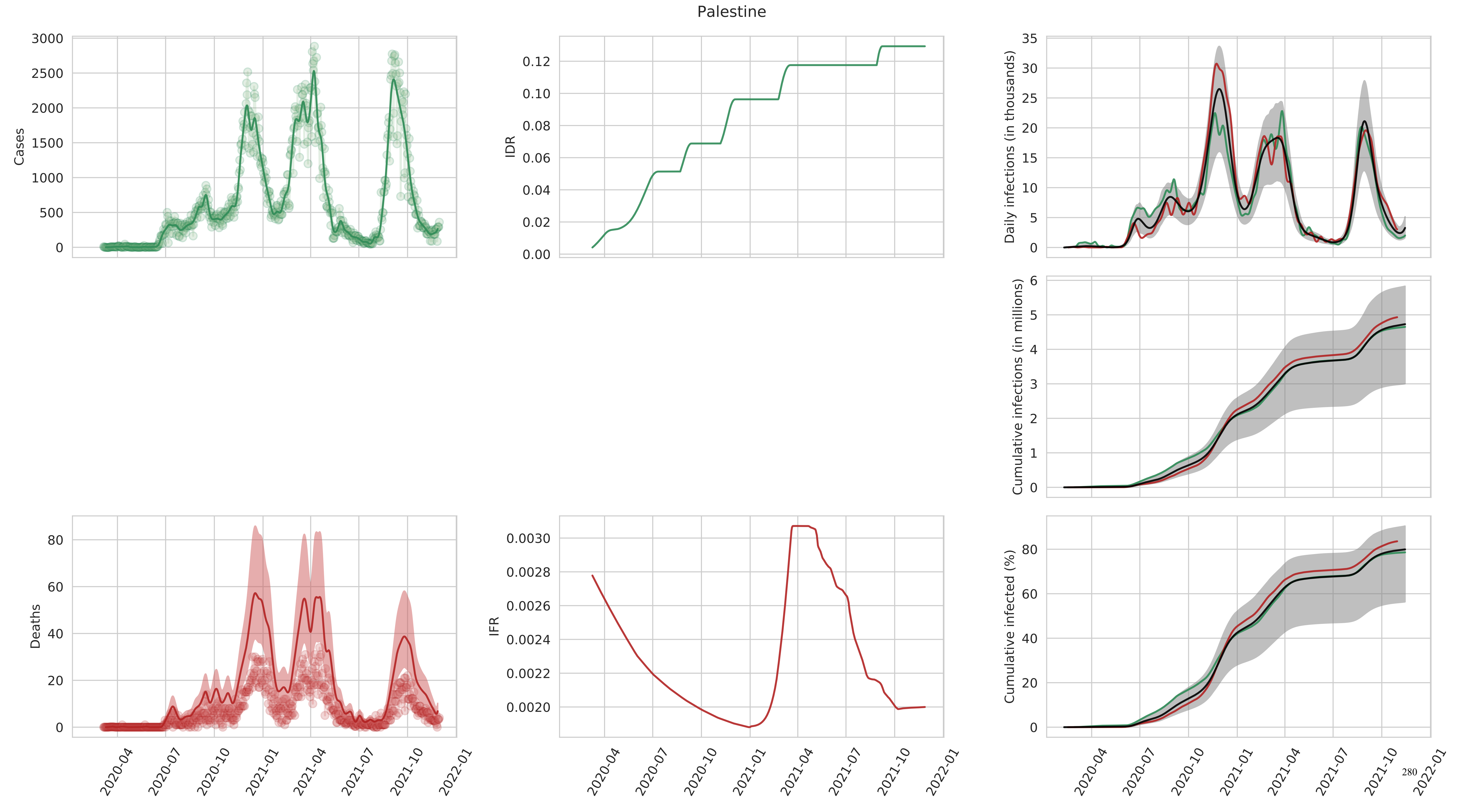

Palestine
Cases
IDR
Daily infections (in thousands)
Cumulative infections (in millions)
Deaths
IFR
Cumulative infected (%)
2020-04
2020-07
2020-10
2021-01
2021-04
2021-07
2021-10
2022-01

Qatar

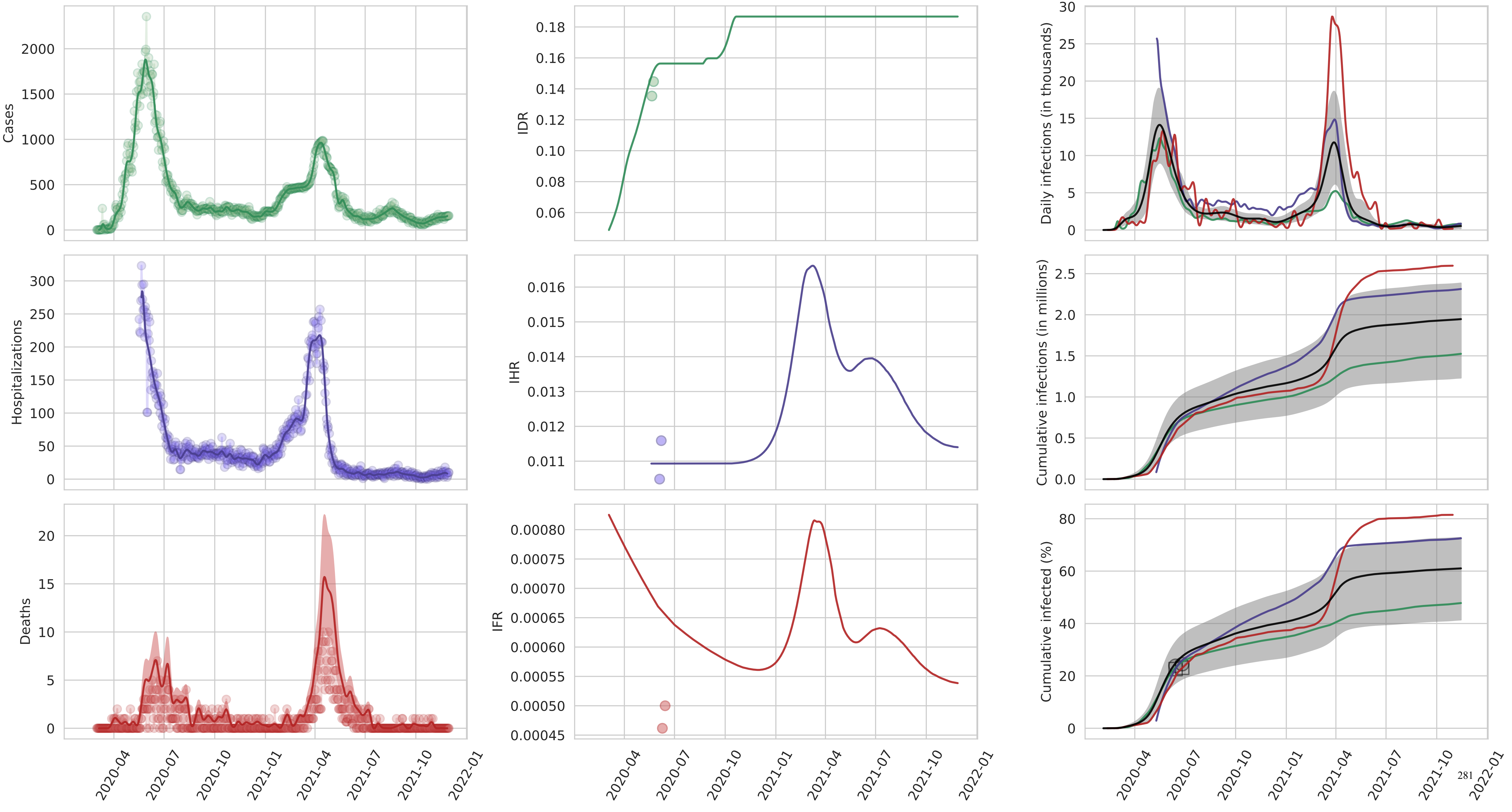

Cases
Hospitalizations
Deaths
IDR
IHR
IFR
Daily infections (in thousands)
Cumulative infections (in millions)
Cumulative infected (%)
2020-04
2020-07
2020-10
2021-01
2021-04
2021-07
2021-10
2022-01

Saudi Arabia

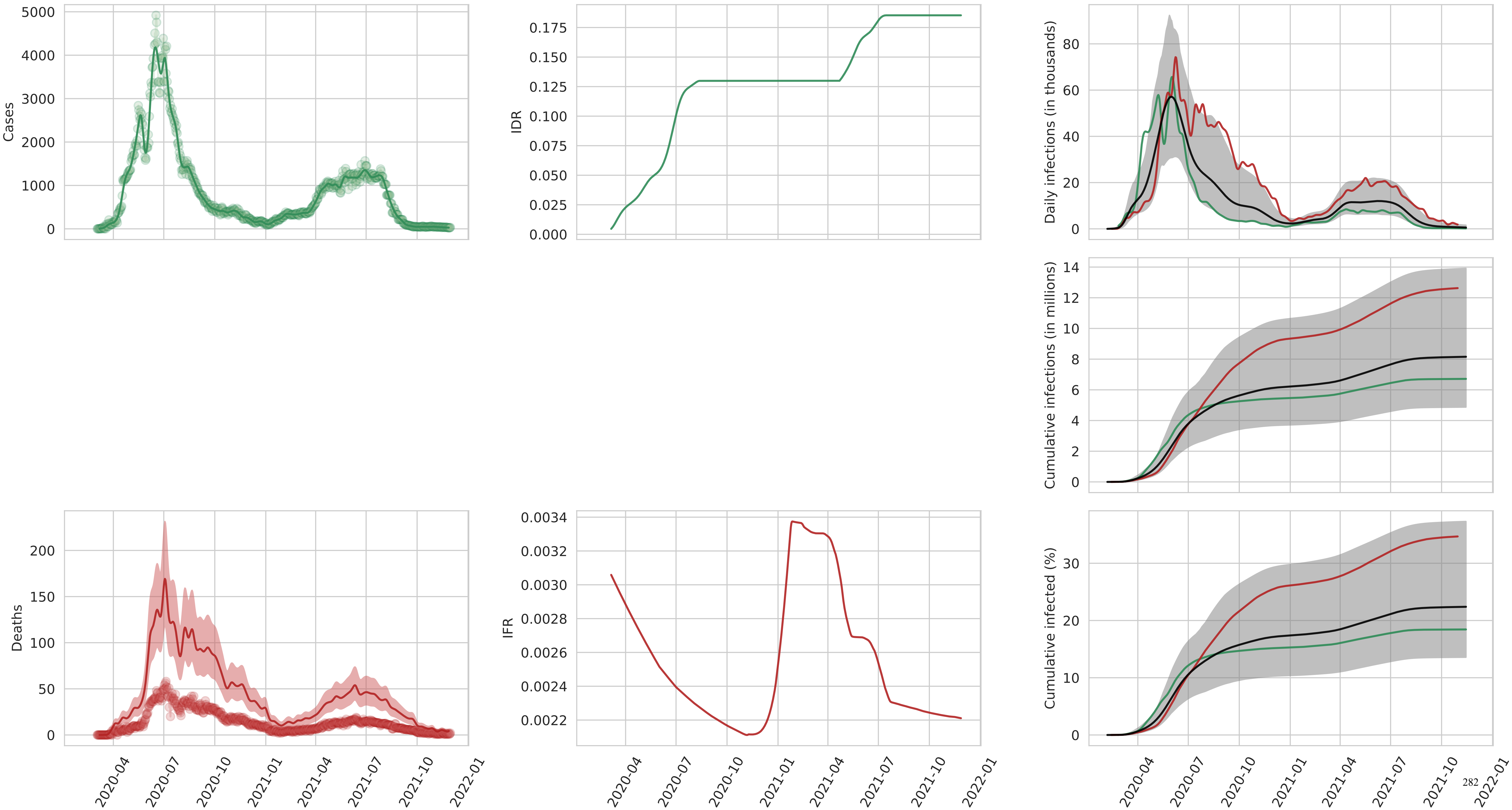

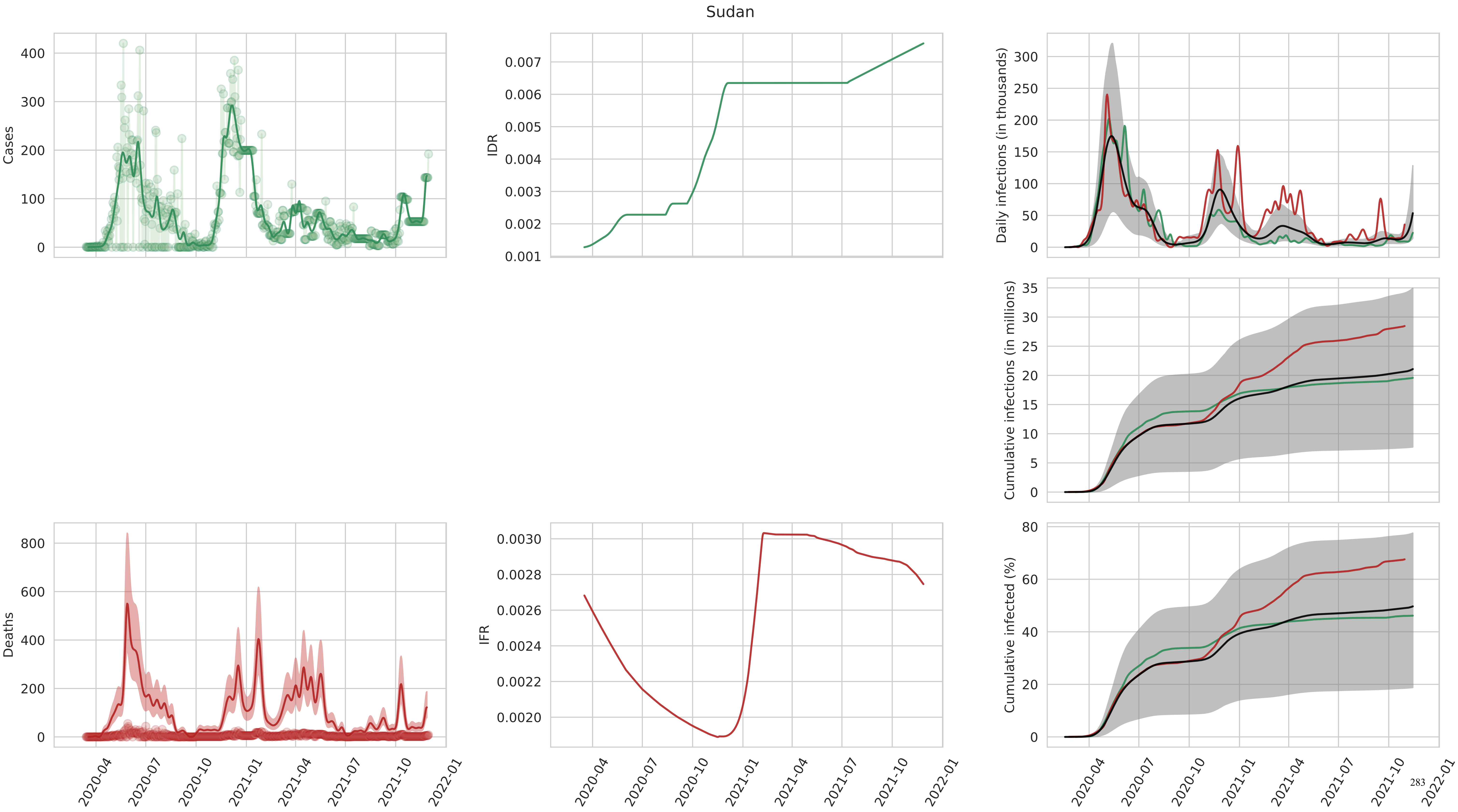

Sudan
Cases
IDR
Daily infections (in thousands)
Cumulative infections (in millions)
Deaths
IFR
Cumulative infected (%)
2020-04
2020-07
2020-10
2021-01
2021-04
2021-07
2021-10
2022-01

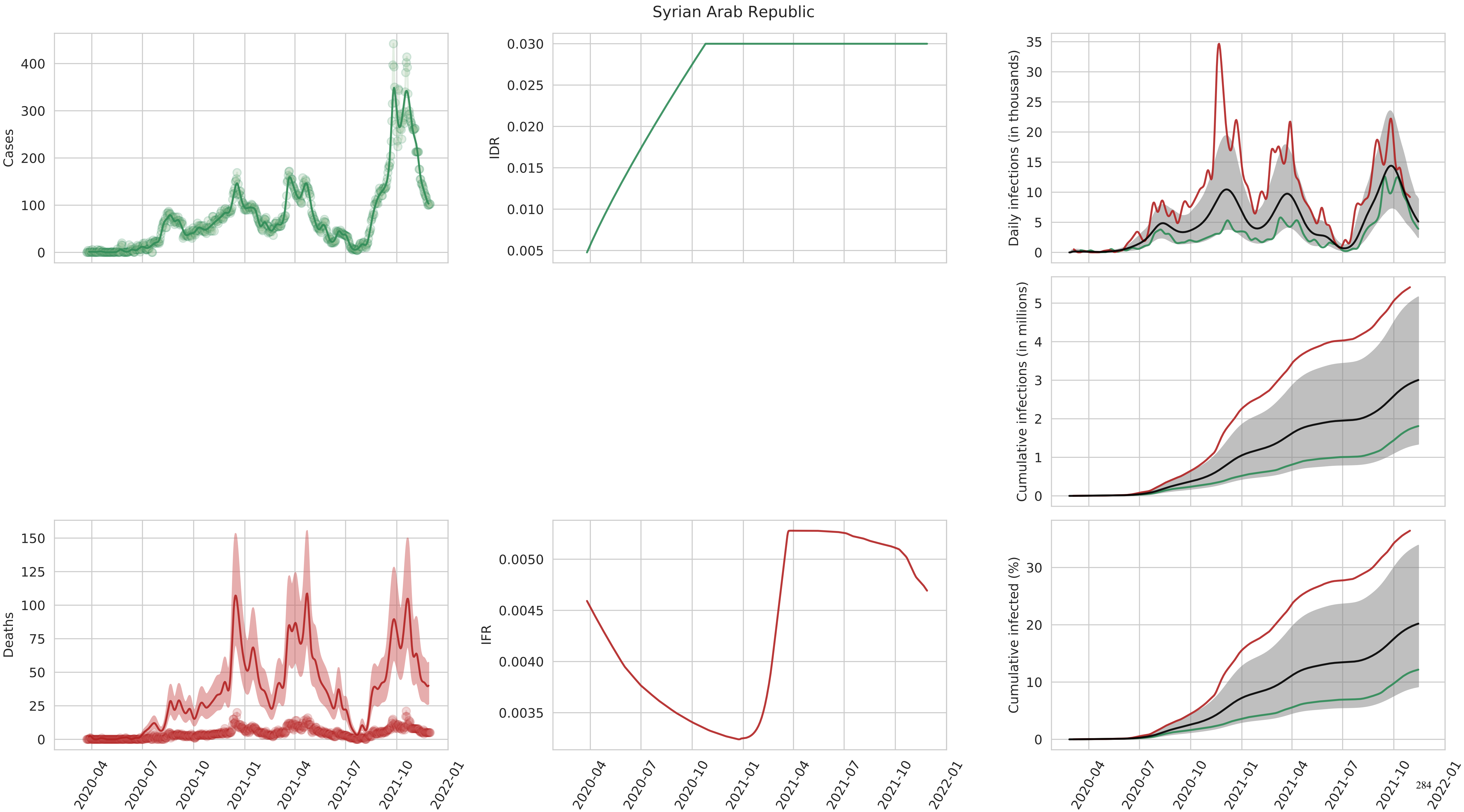
Syrian Arab Republic
Cases
IDR
Daily infections (in thousands)
Cumulative infections (in millions)
Deaths
IFR
Cumulative infected (%)
2020-04
2020-07
2020-10
2021-01
2021-04
2021-07
2021-10
2022-01

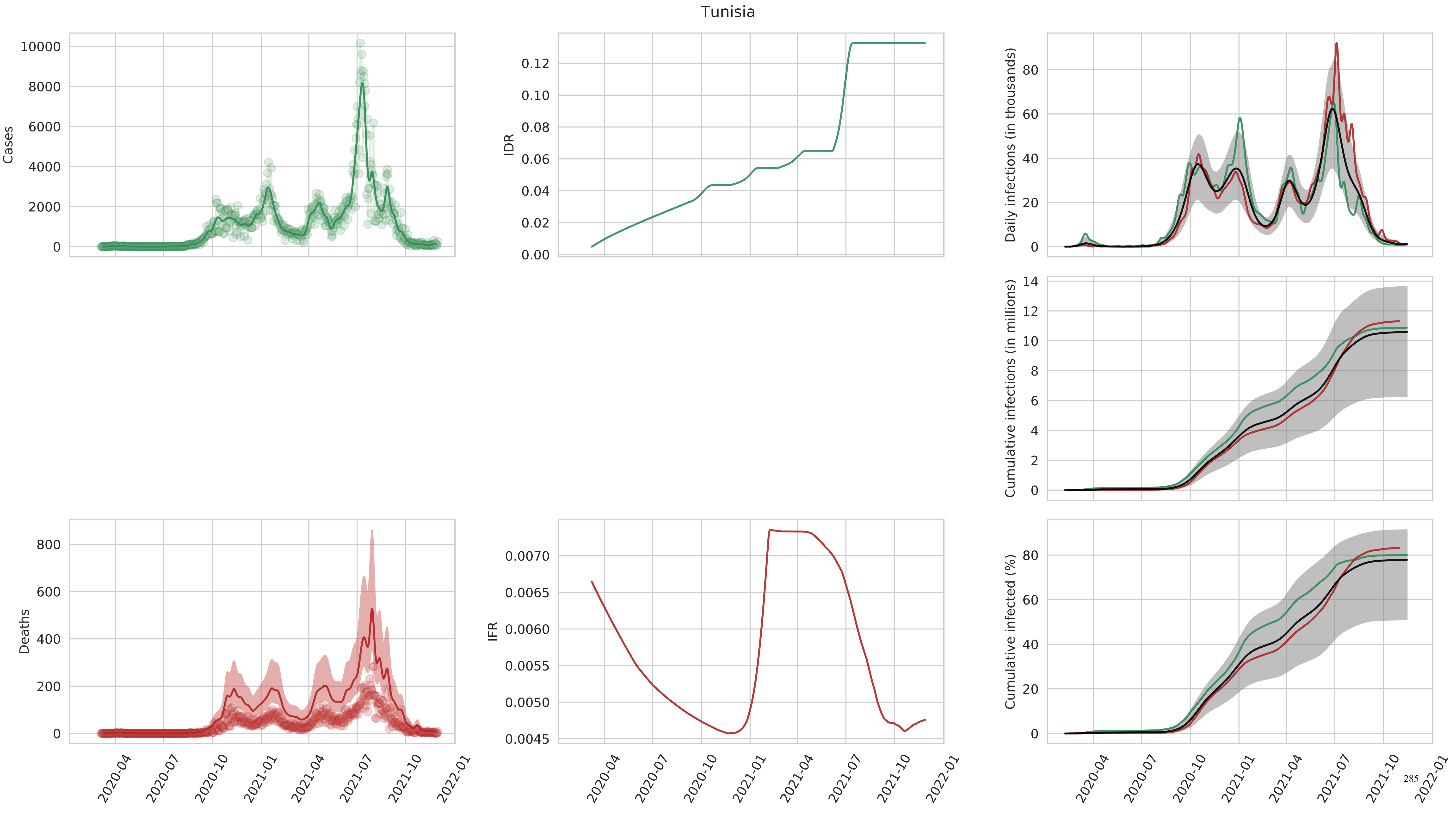

Tunisia
Cases
IDR
Daily infections (in thousands)
Cumulative infections (in millions)
Deaths
IFR
Cumulative infected (%)
2020-04
2020-07
2020-10
2021-01
2021-04
2021-07
2021-10
2022-01

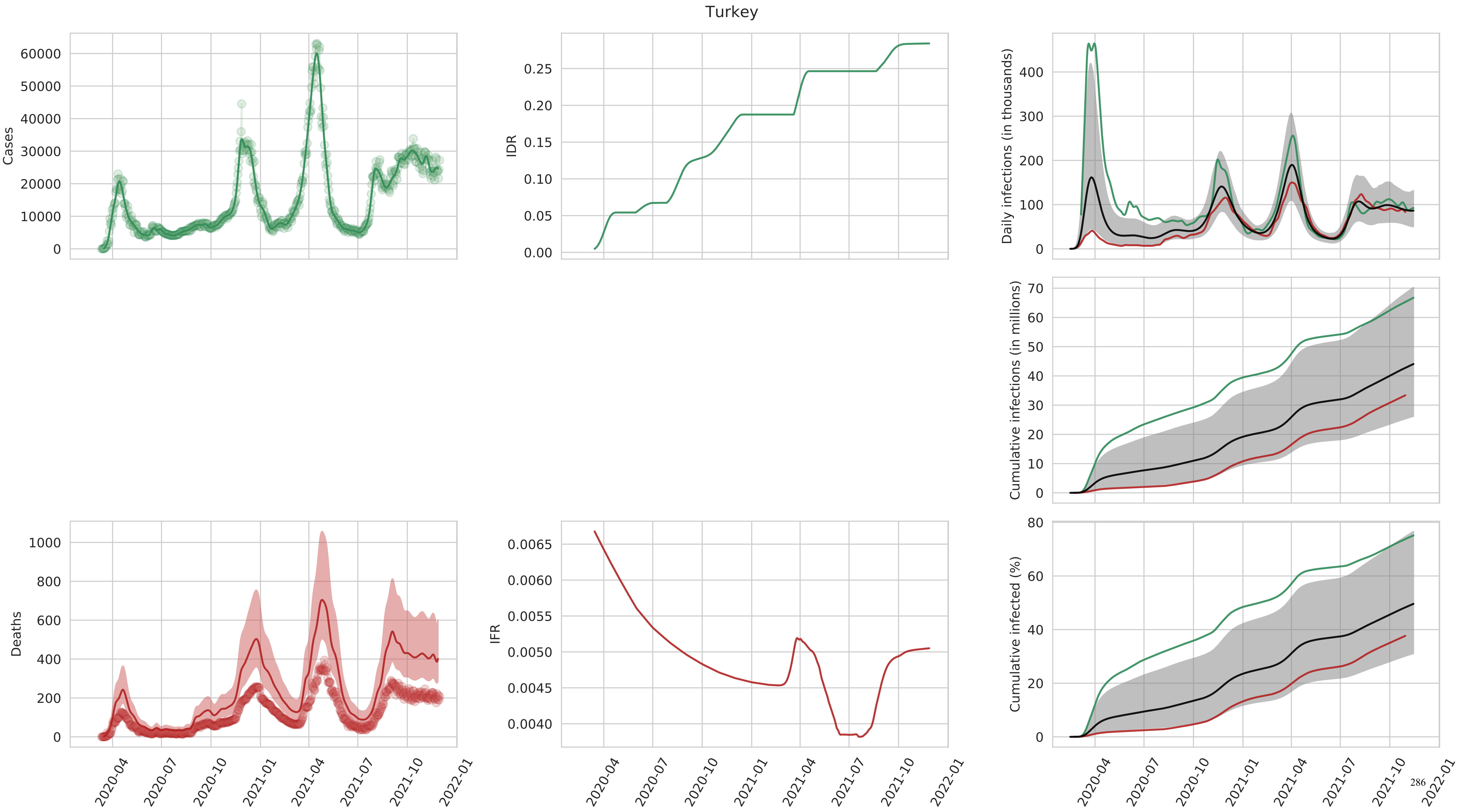
Turkey
Cases
IDR
Daily infections (in thousands)
Cumulative infections (in millions)
Deaths
IFR
Cumulative infected (%)
2020-04
2020-07
2020-10
2021-01
2021-04
2021-07
2021-10
2022-01

United Arab Emirates

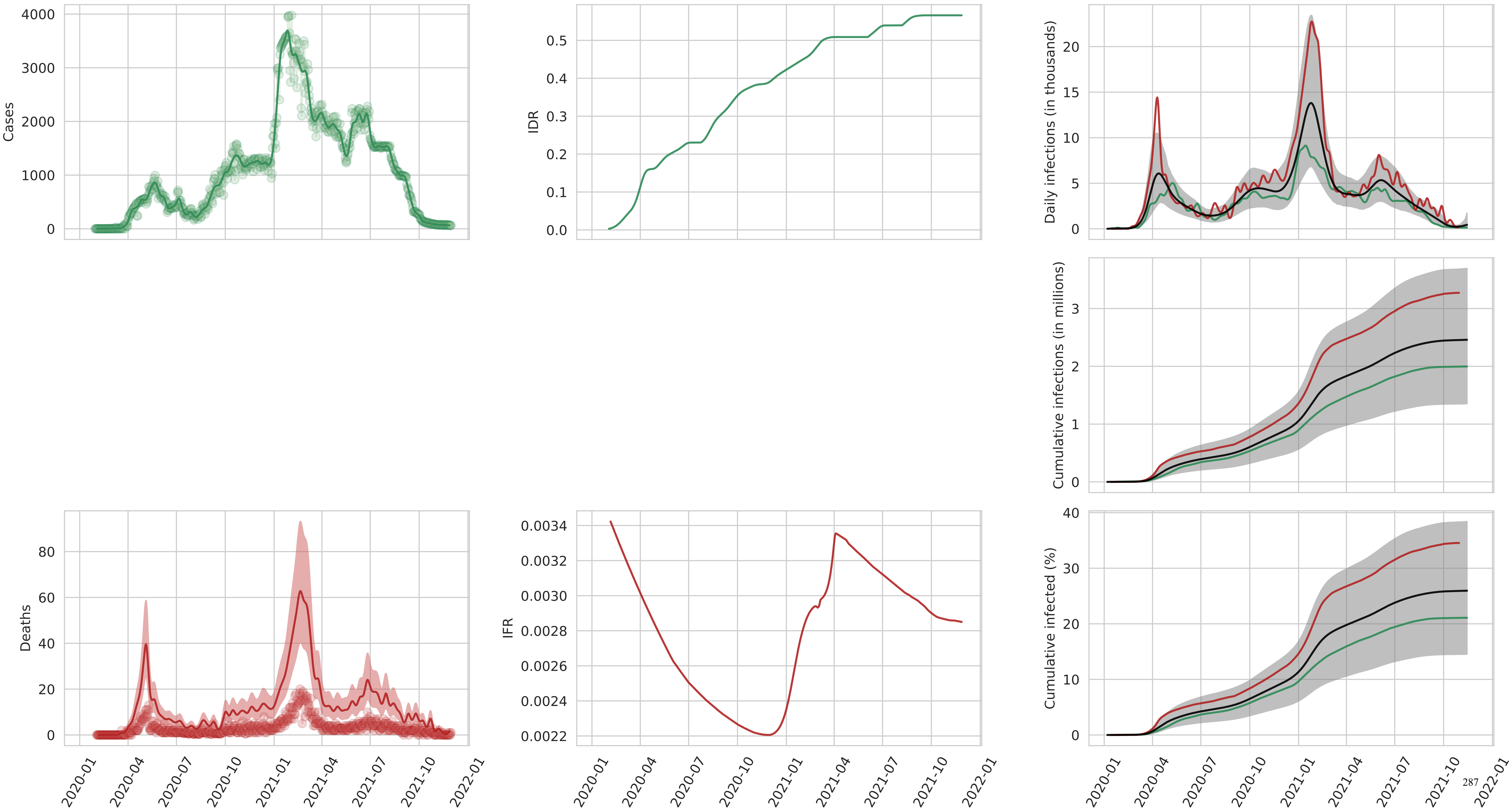

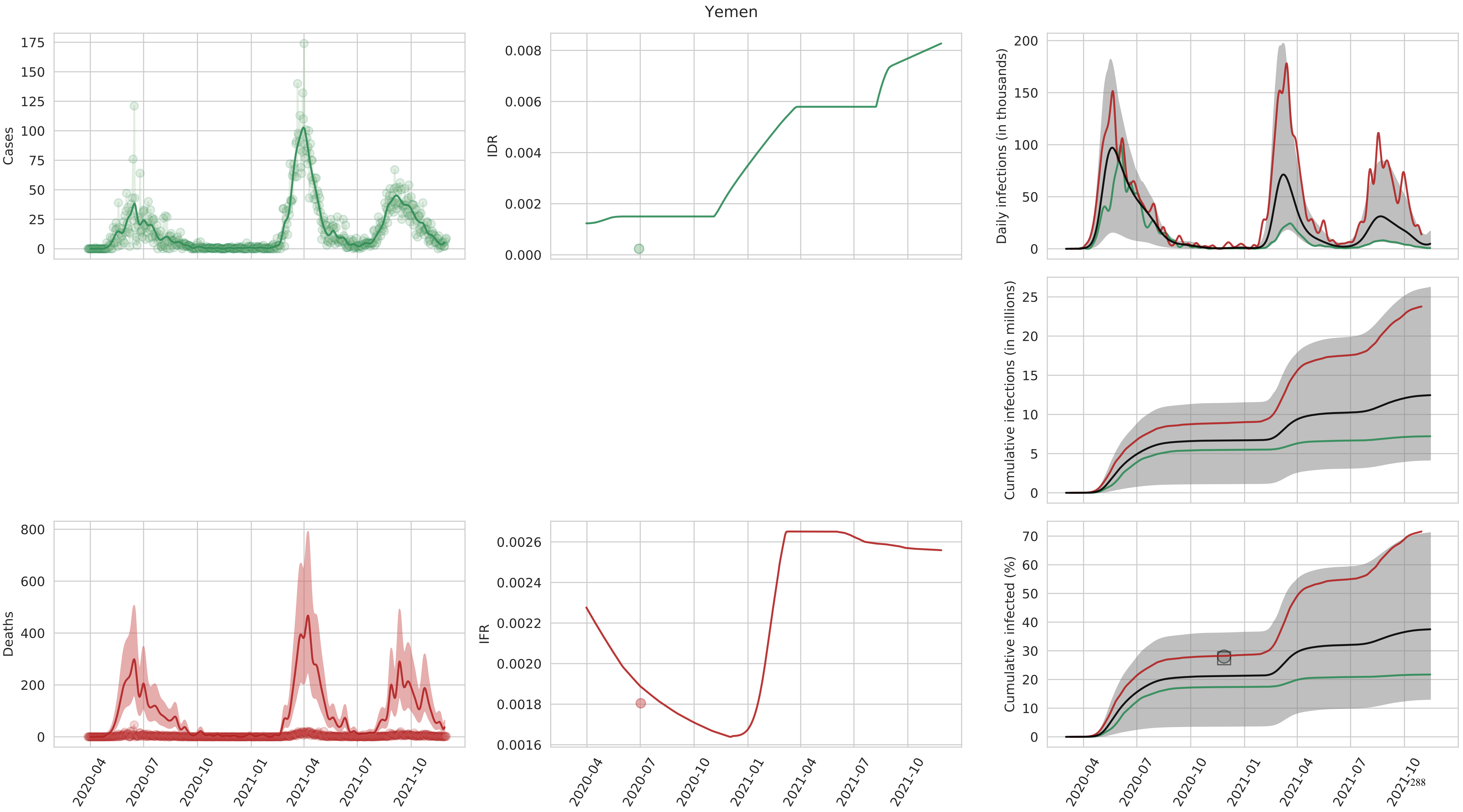
Yemen
Cases
IDR
Daily infections (in thousands)
Cumulative infections (in millions)
Deaths
IFR
Cumulative infected (%)
2020-04
2020-07
2020-10
2021-01
2021-04
2021-07
2021-10

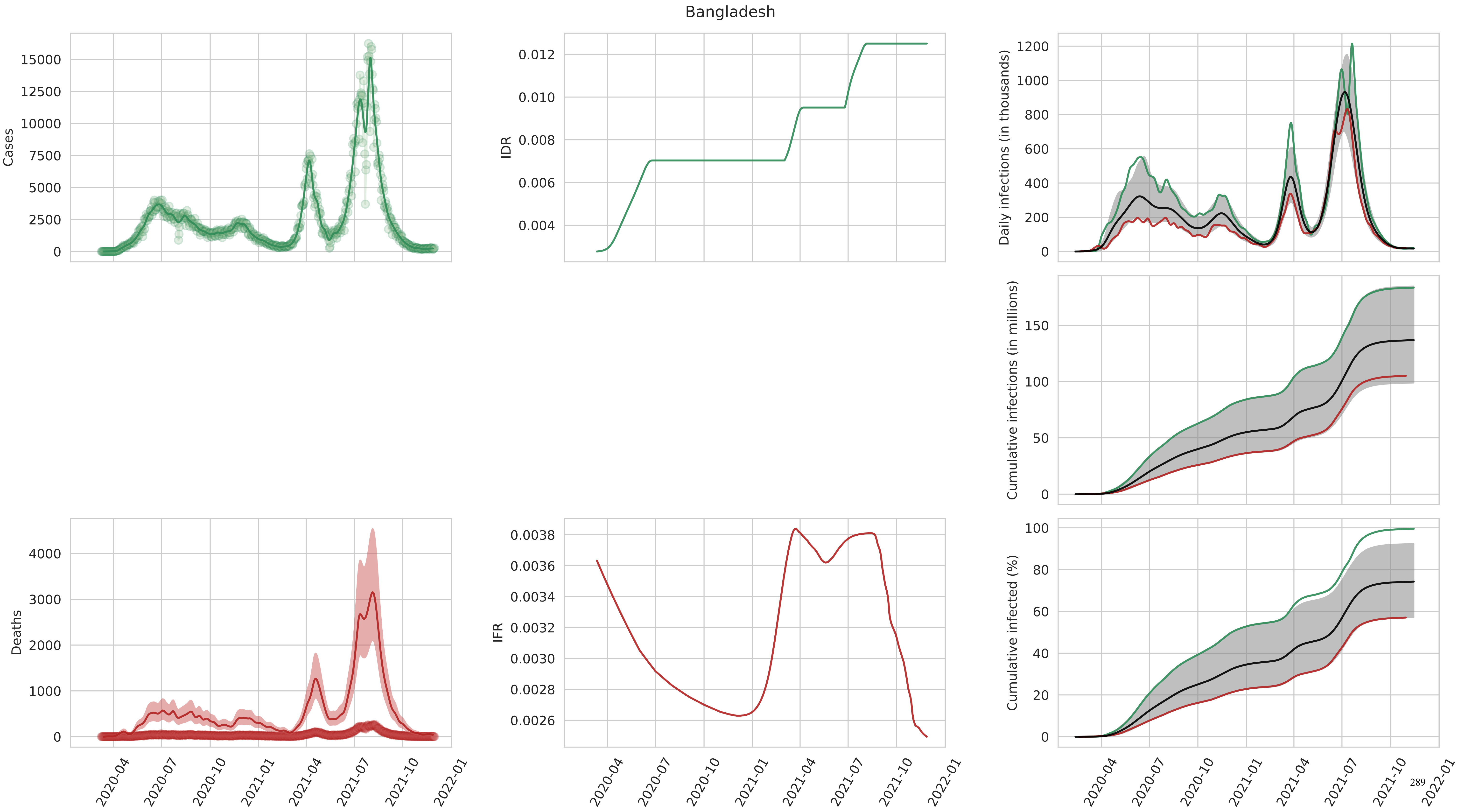
Bangladesh
Cases
IDR
Daily infections (in thousands)
Cumulative infections (in millions)
Deaths
IFR
Cumulative infected (%)
2020-04
2020-07
2020-10
2021-01
2021-04
2021-07
2021-10
2022-01

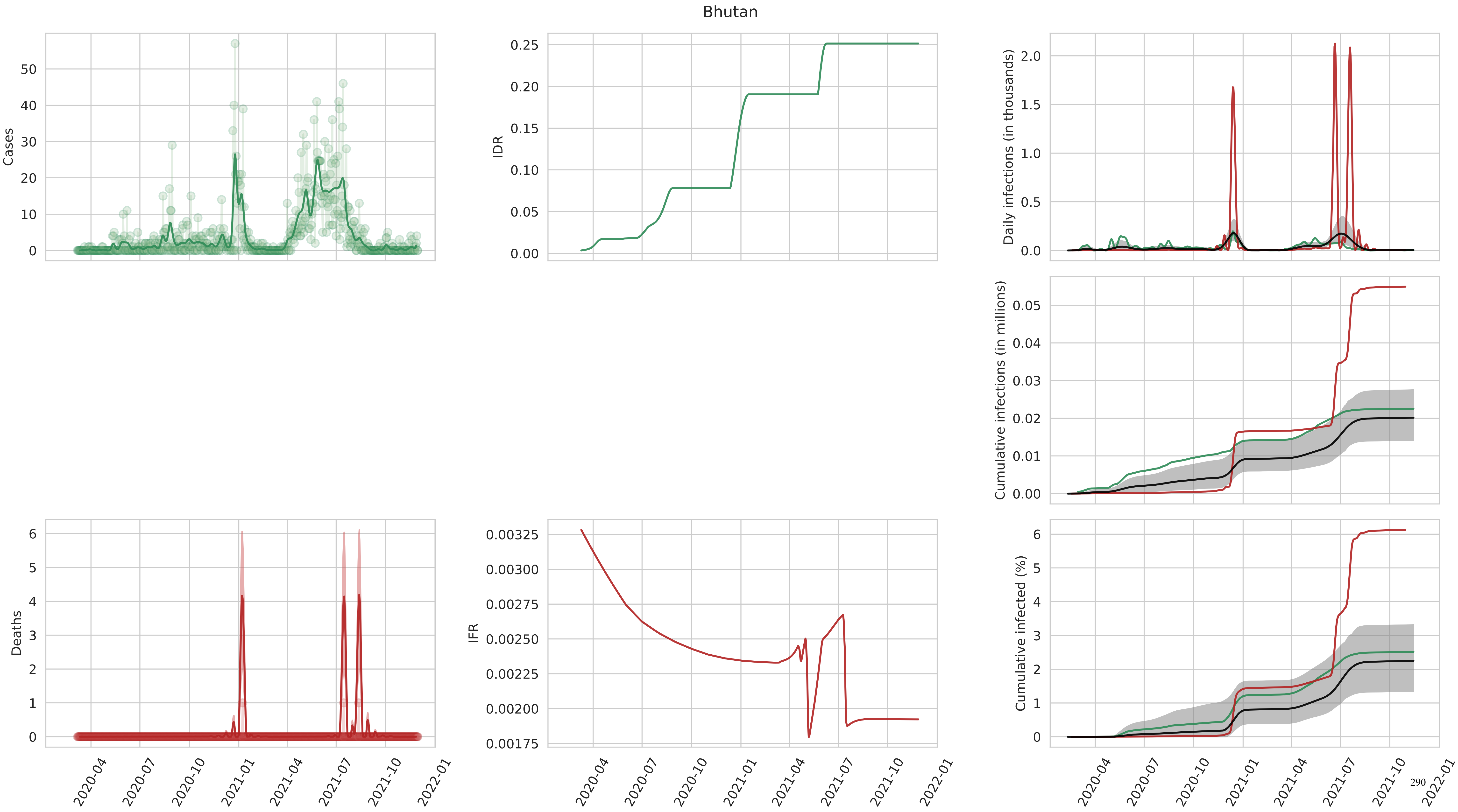
Bhutan
Cases
IDR
Daily infections (in thousands)
Cumulative infections (in millions)
Deaths
IFR
Cumulative infected (%)
2020-04
2020-07
2020-10
2021-01
2021-04
2021-07
2021-10
2022-01

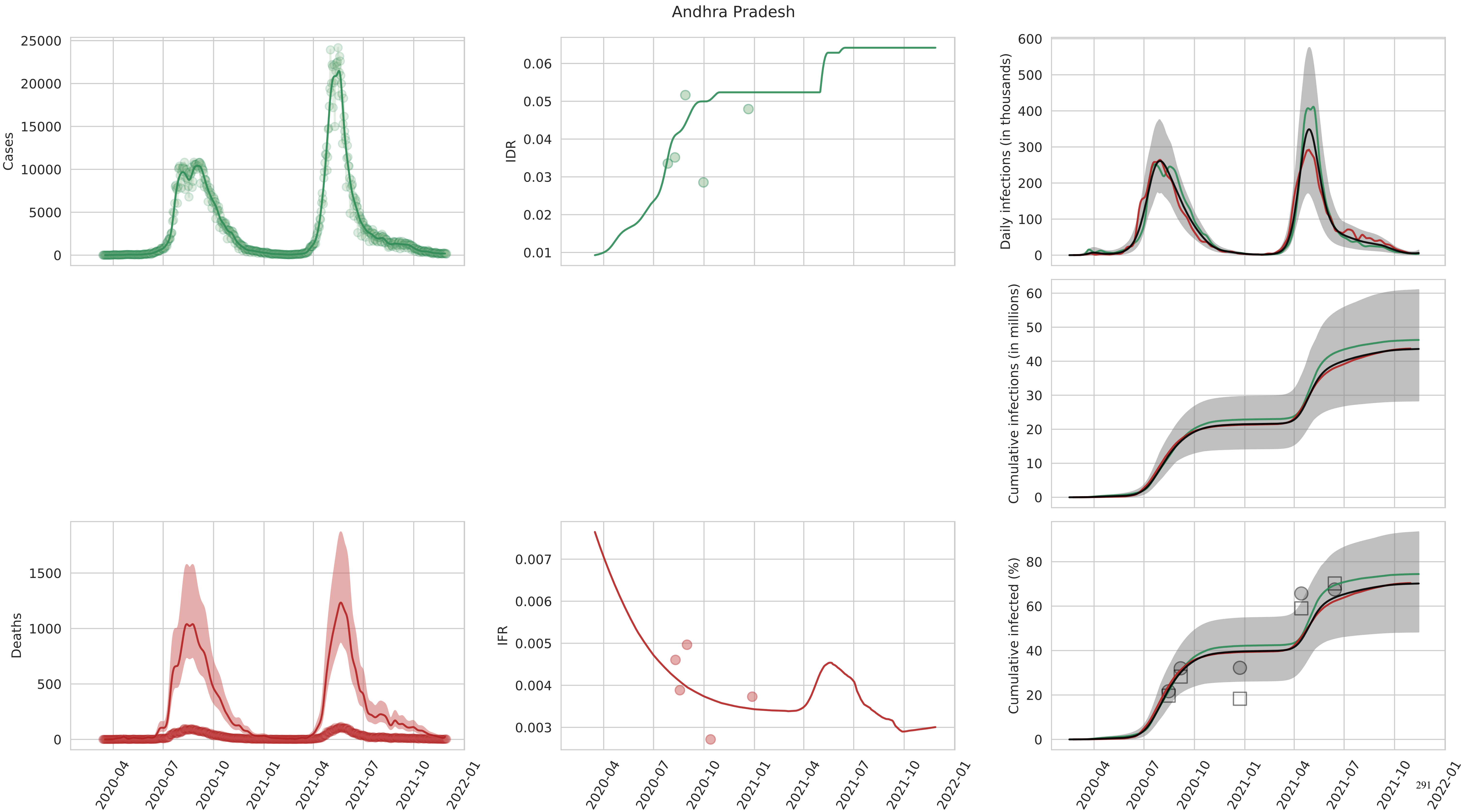

Andhra Pradesh
Cases
IDR
Daily infections (in thousands)
Cumulative infections (in millions)
Deaths
IFR
Cumulative infected (%)
2020-04
2020-07
2020-10
2021-01
2021-04
2021-07
2021-10
2022-01

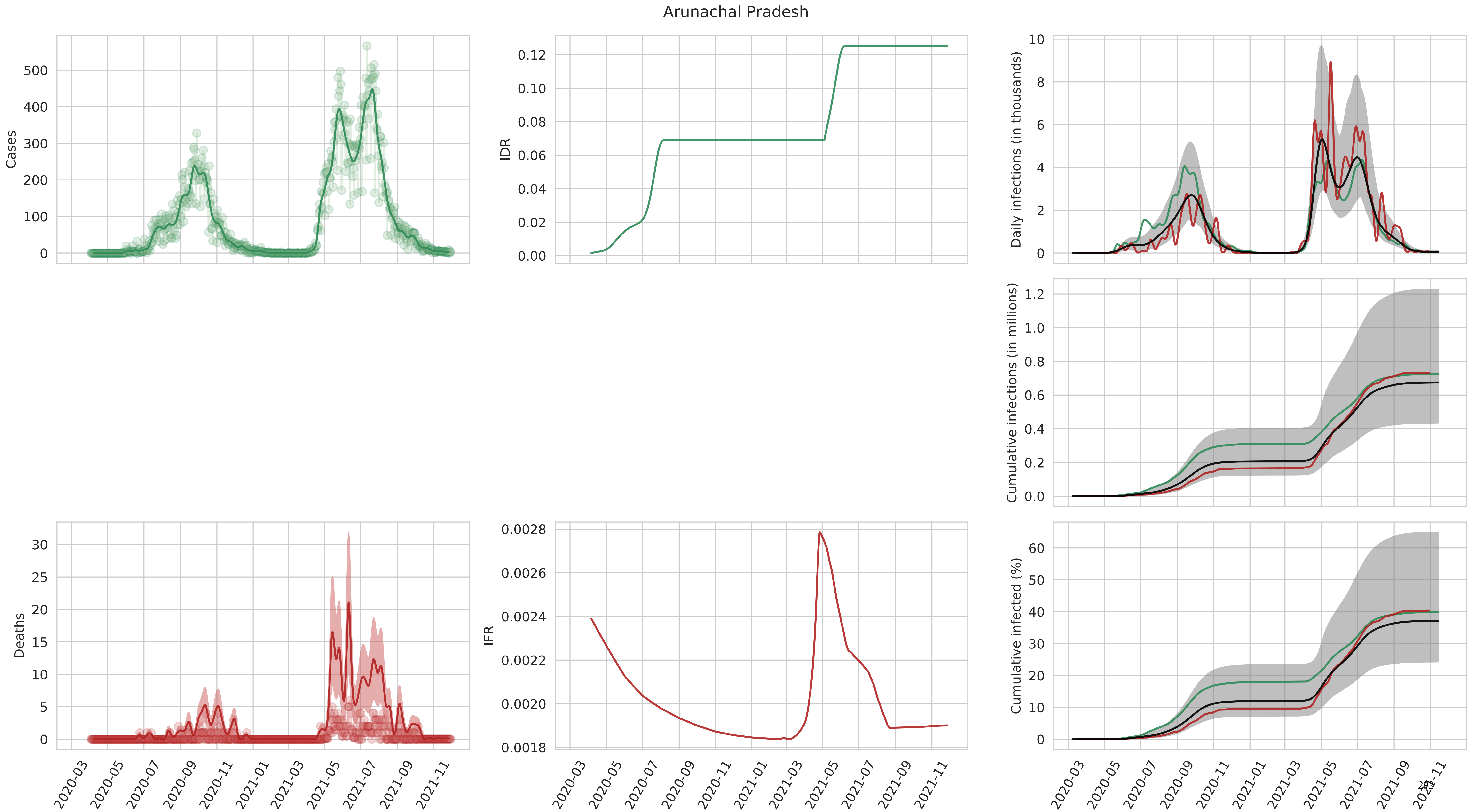
Arunachal Pradesh
Cases
IDR
Daily infections (in thousands)
Cumulative infections (in millions)
Deaths
IFR
Cumulative infected (%)
2020-03
2020-05
2020-07
2020-09
2020-11
2021-01
2021-03
2021-05
2021-07
2021-09
2021-11

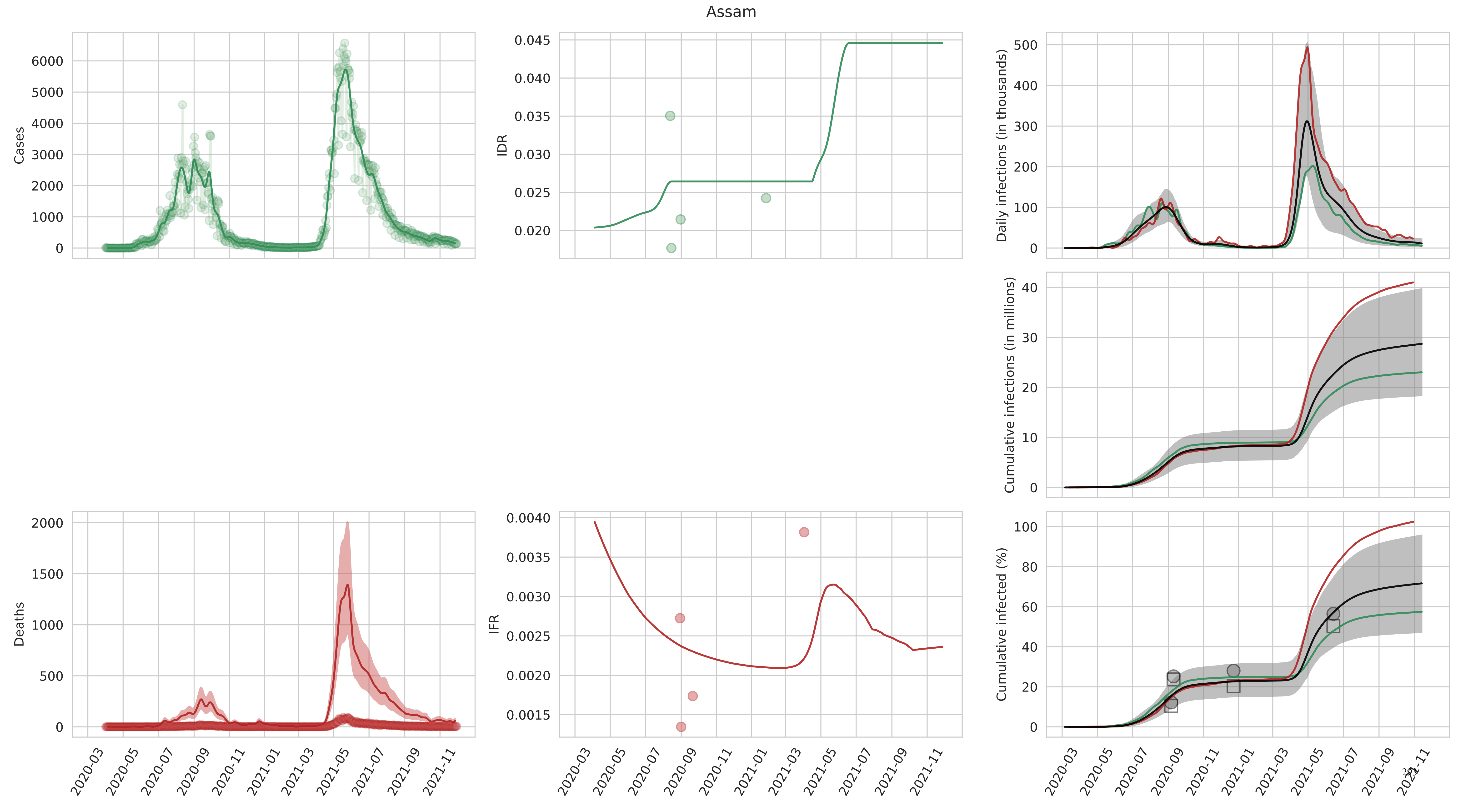
Assam
Cases
IDR
Daily infections (in thousands)
Cumulative infections (in millions)
Deaths
IFR
Cumulative infected (%)
2020-03
2020-05
2020-07
2020-09
2020-11
2021-01
2021-03
2021-05
2021-07
2021-09
2021-11

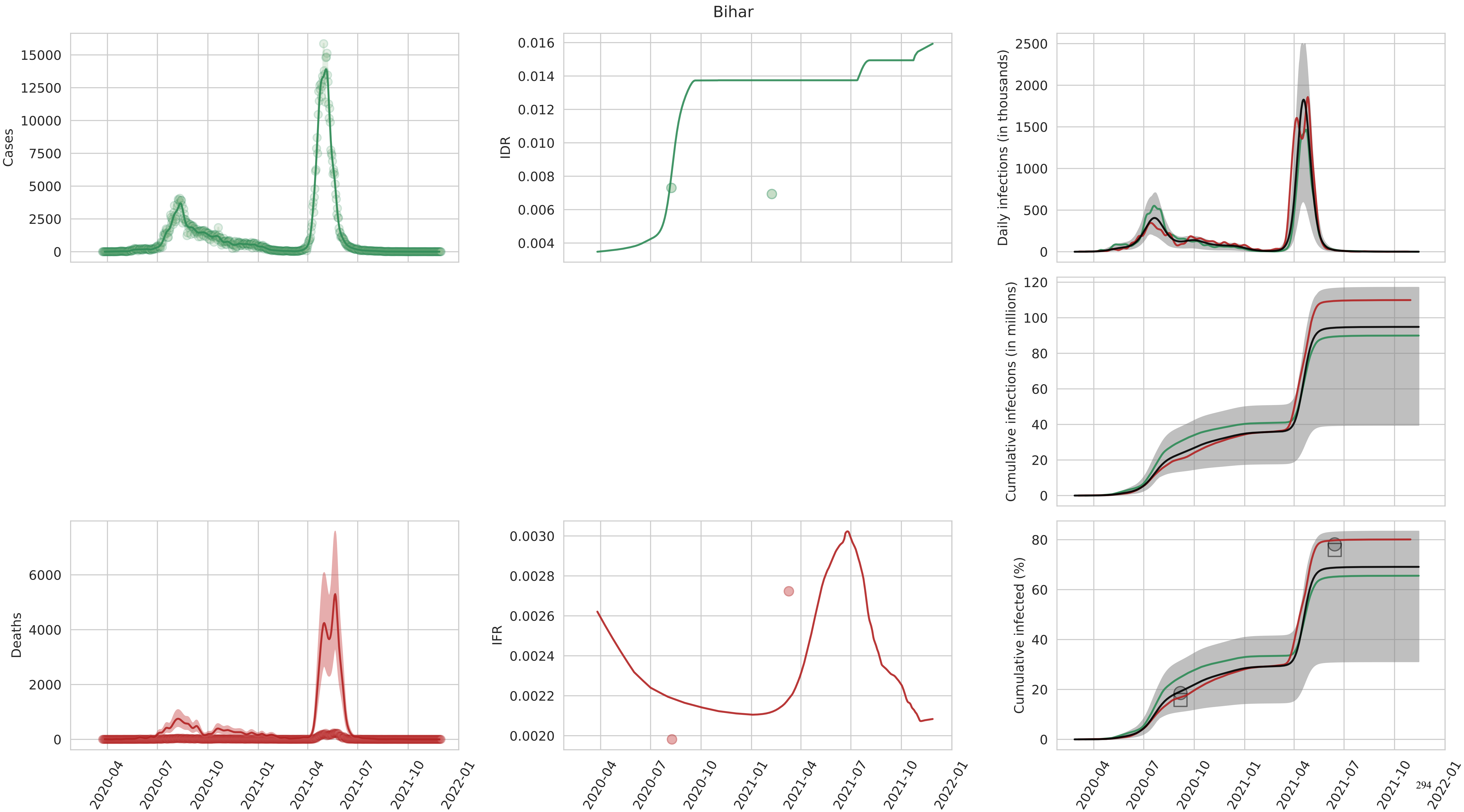

Bihar
Cases
IDR
Daily infections (in thousands)
Cumulative infections (in millions)
Deaths
IFR
Cumulative infected (%)
2020-04
2020-07
2020-10
2021-01
2021-04
2021-07
2021-10
2022-01

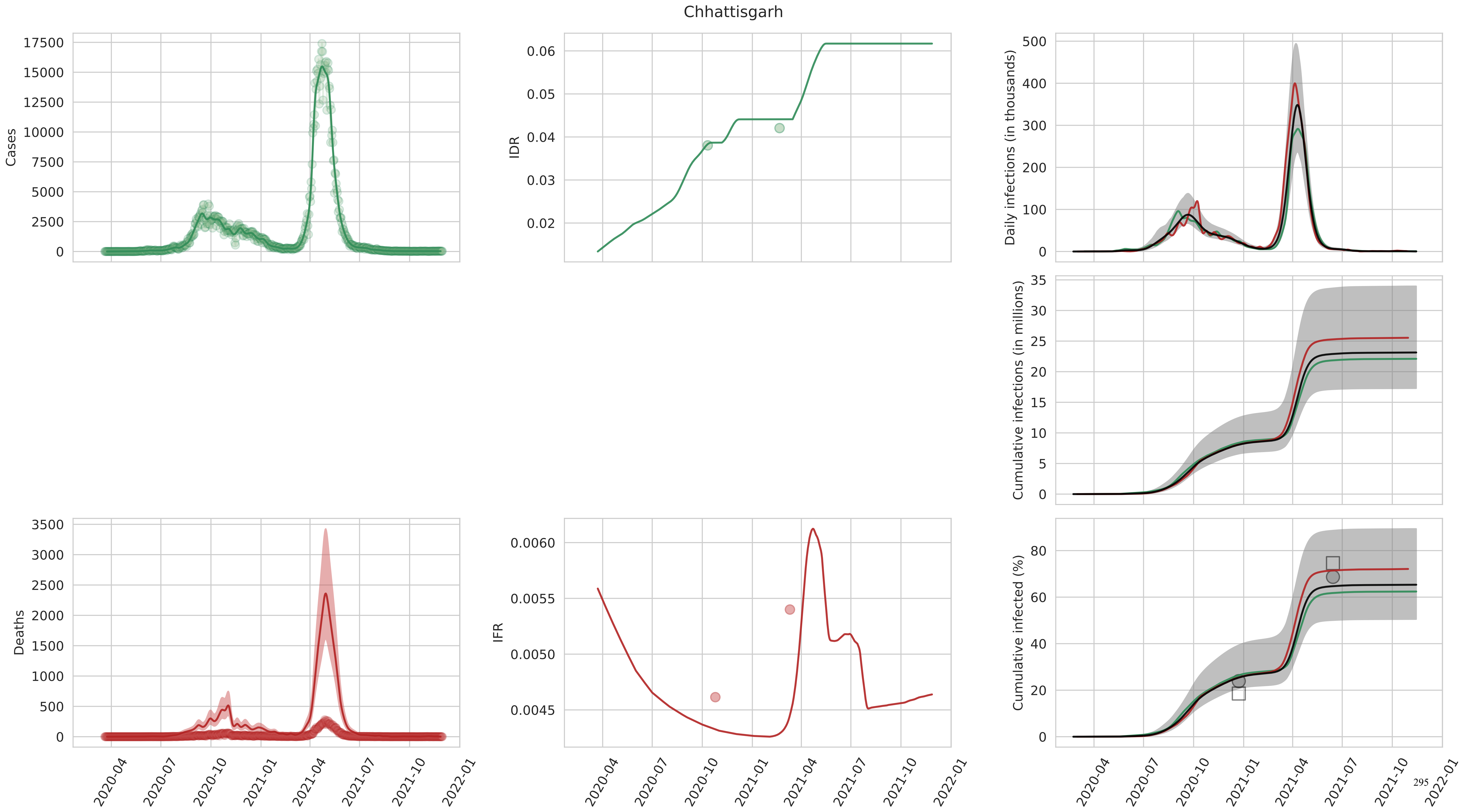
Chhattisgarh
Cases
IDR
Daily infections (in thousands)
Cumulative infections (in millions)
Deaths
IFR
Cumulative infected (%)
2020-04
2020-07
2020-10
2021-01
2021-04
2021-07
2021-10
2022-01

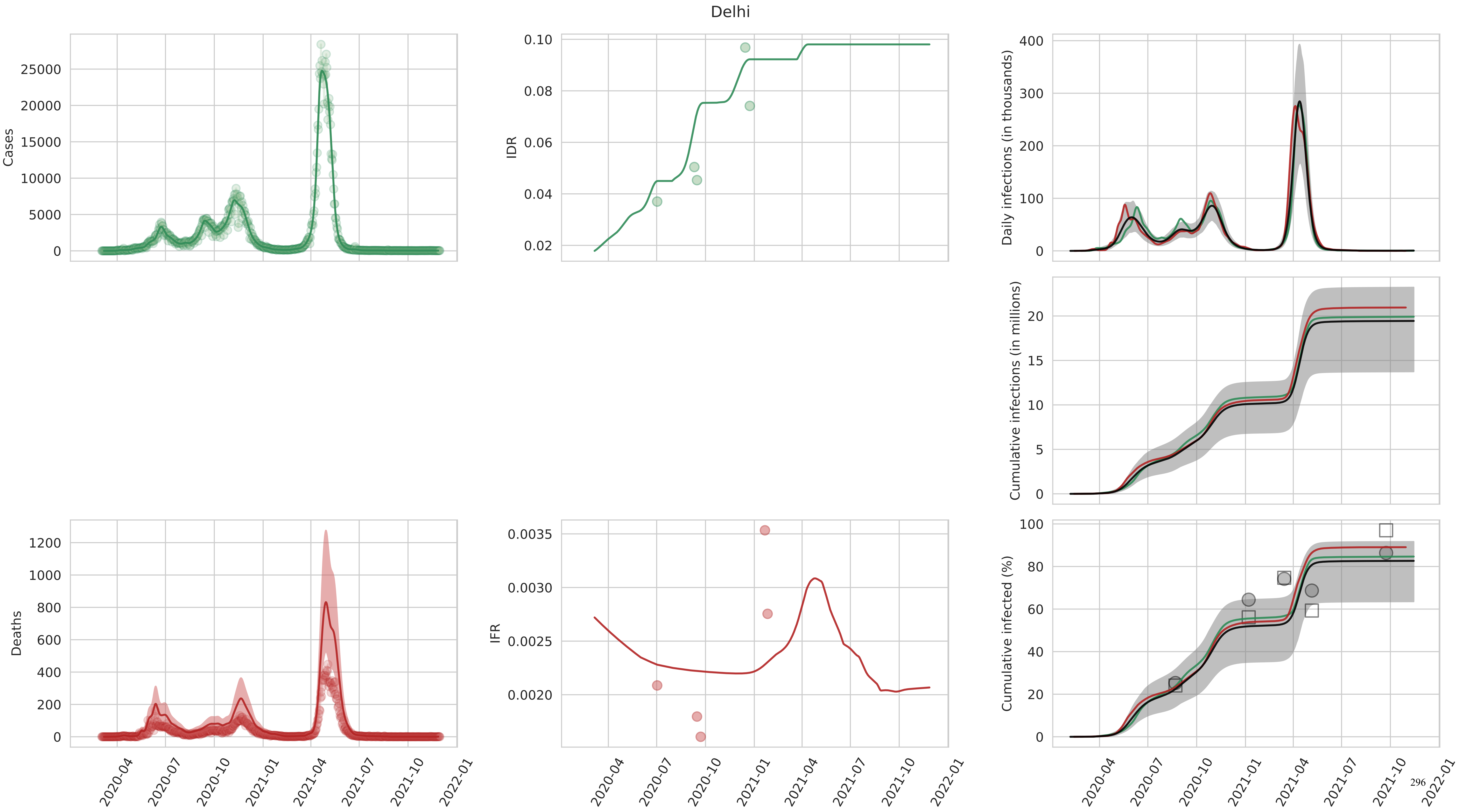
Delhi
Cases
IDR
Daily infections (in thousands)
Cumulative infections (in millions)
Deaths
IFR
Cumulative infected (%)
2020-04
2020-07
2020-10
2021-01
2021-04
2021-07
2021-10
2022-01

Goa

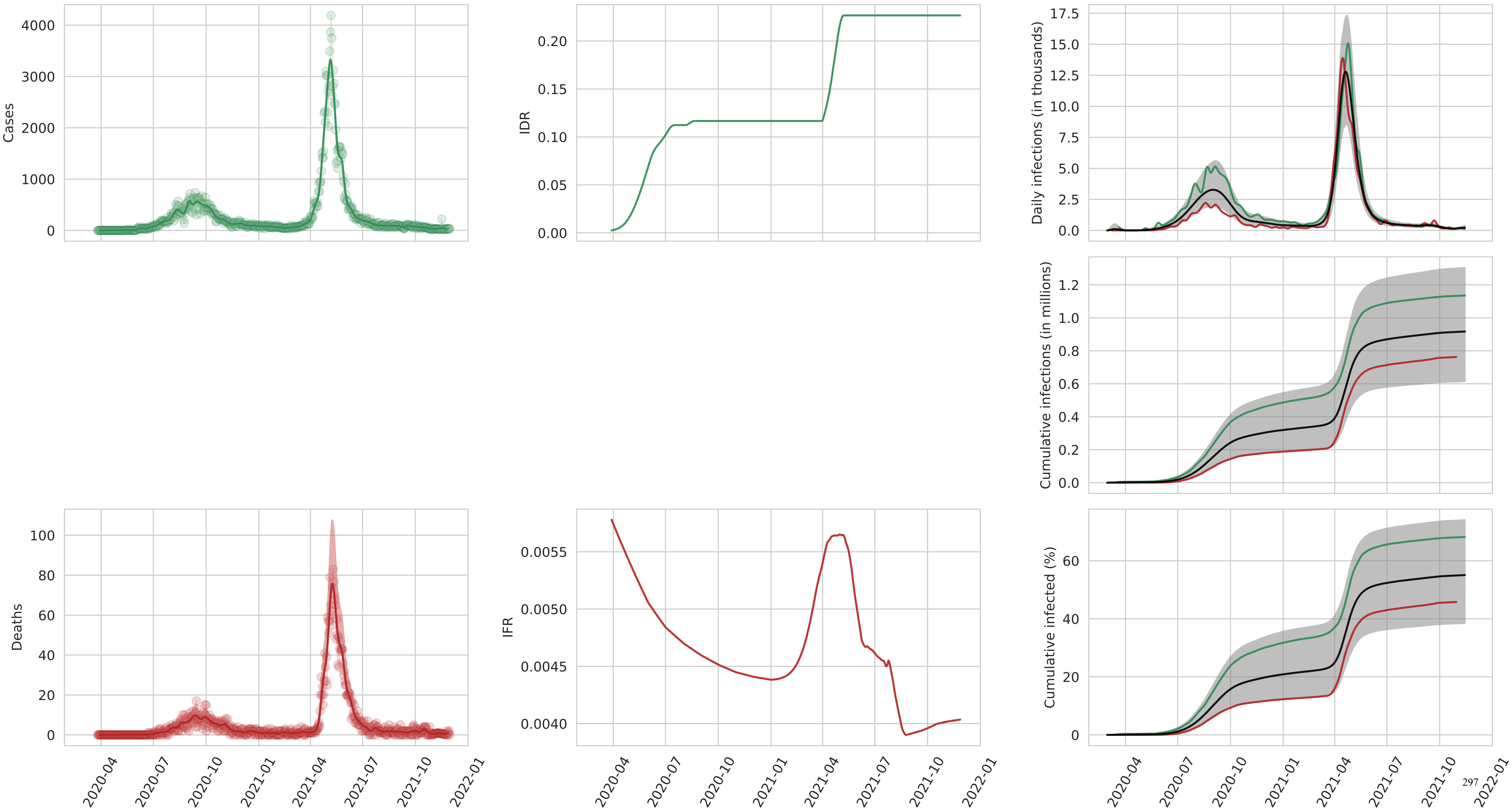

Cases
IDR
Daily infections (in thousands)
Cumulative infections (in millions)
Deaths
IFR
Cumulative infected (%)

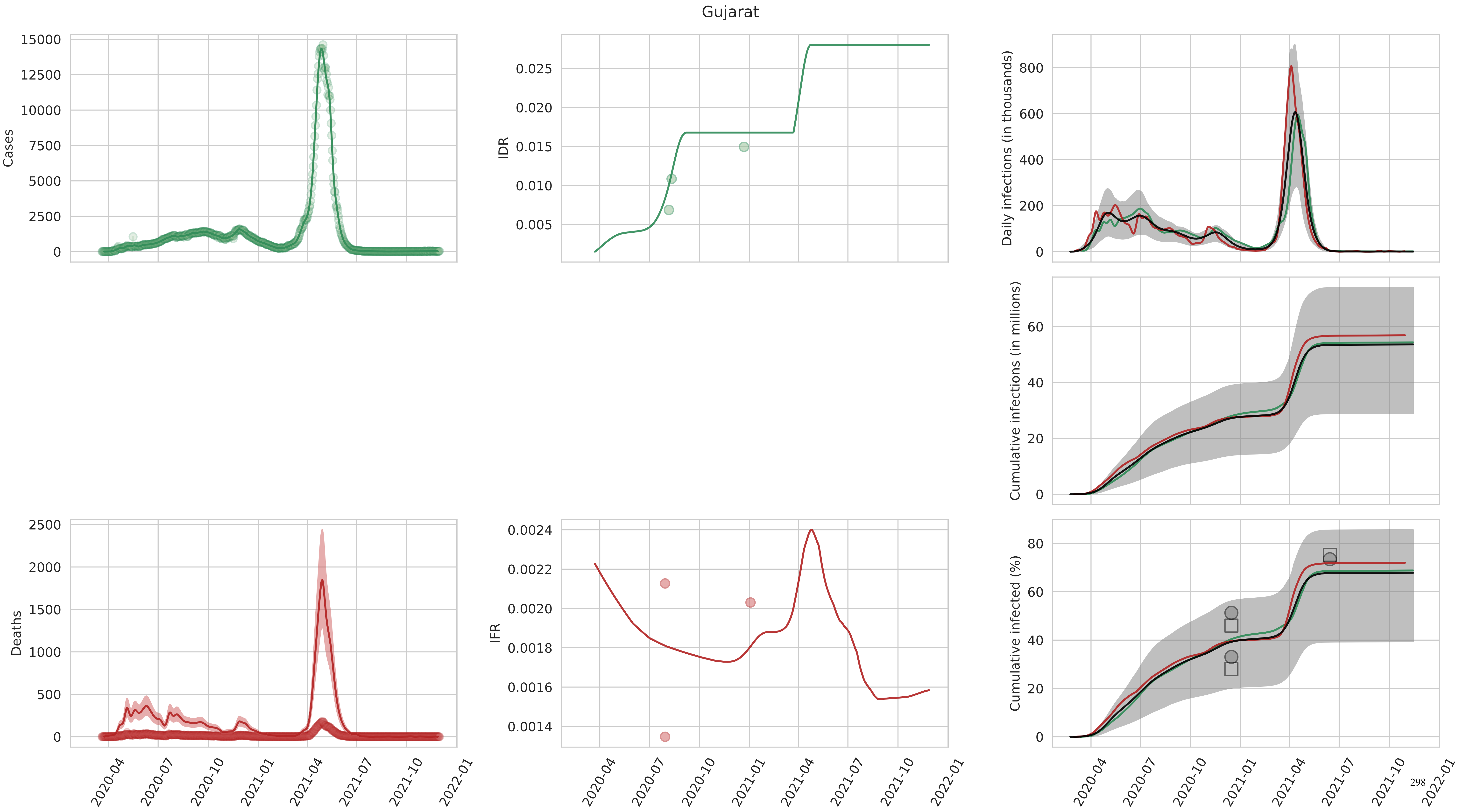

Gujarat
Cases
IDR
Daily infections (in thousands)
Cumulative infections (in millions)
Deaths
IFR
Cumulative infected (%)
2020-04
2020-07
2020-10
2021-01
2021-04
2021-07
2021-10
2022-01

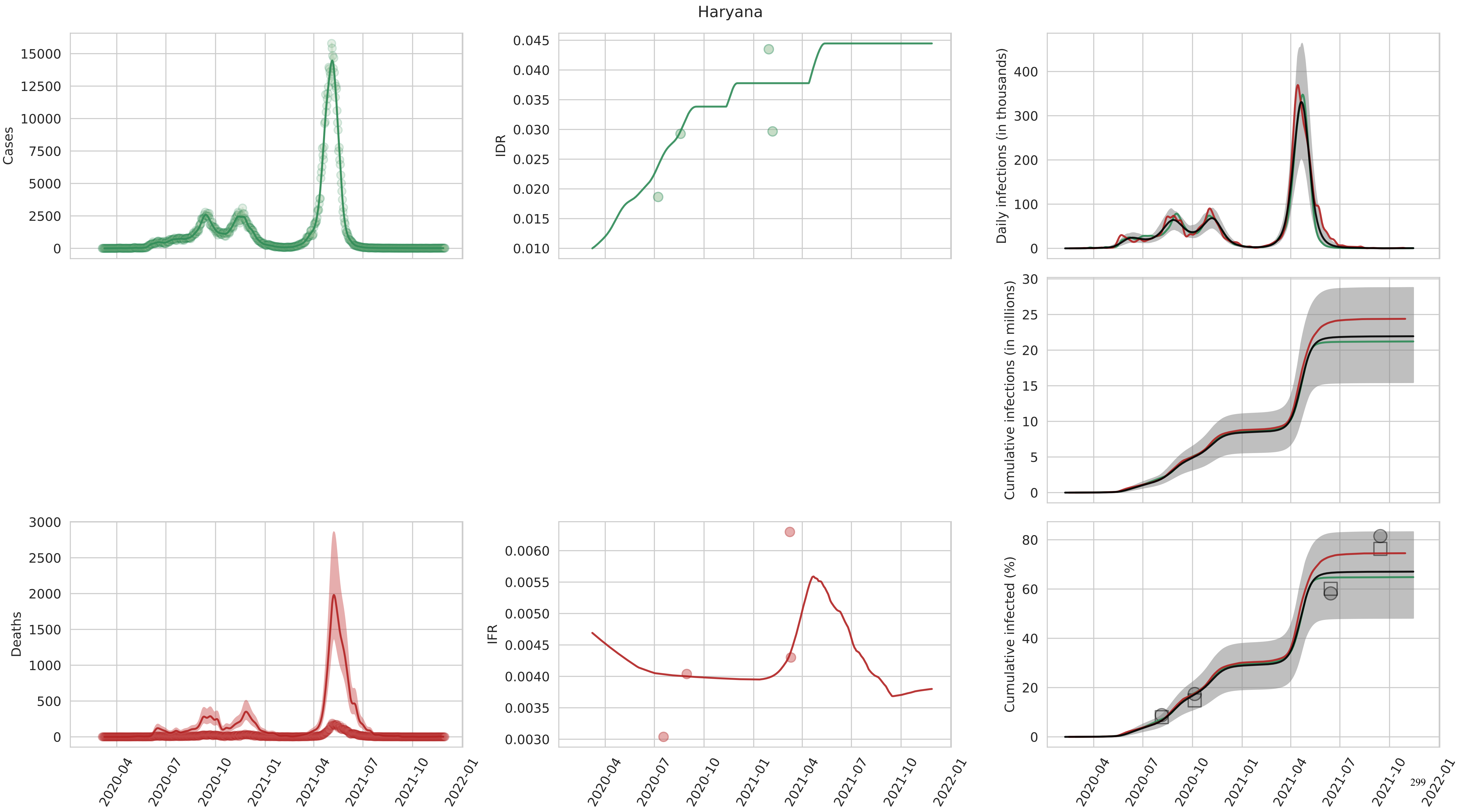
Haryana
Cases
IDR
Daily infections (in thousands)
Cumulative infections (in millions)
Deaths
IFR
Cumulative infected (%)
2020-04
2020-07
2020-10
2021-01
2021-04
2021-07
2021-10
2022-01

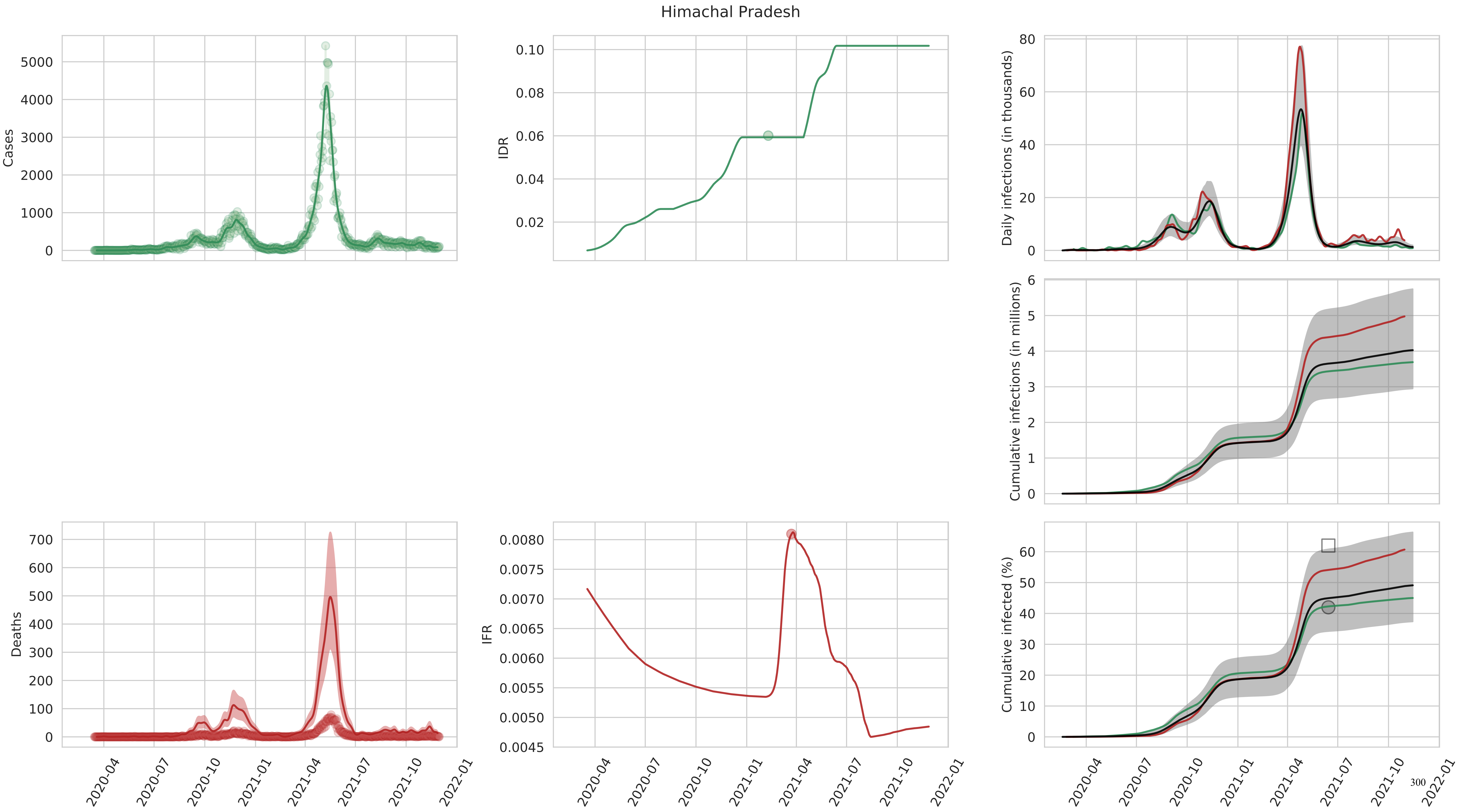

Himachal Pradesh
Cases
IDR
Daily infections (in thousands)
Cumulative infections (in millions)
Deaths
IFR
Cumulative infected (%)

Jammu & Kashmir and Ladakh

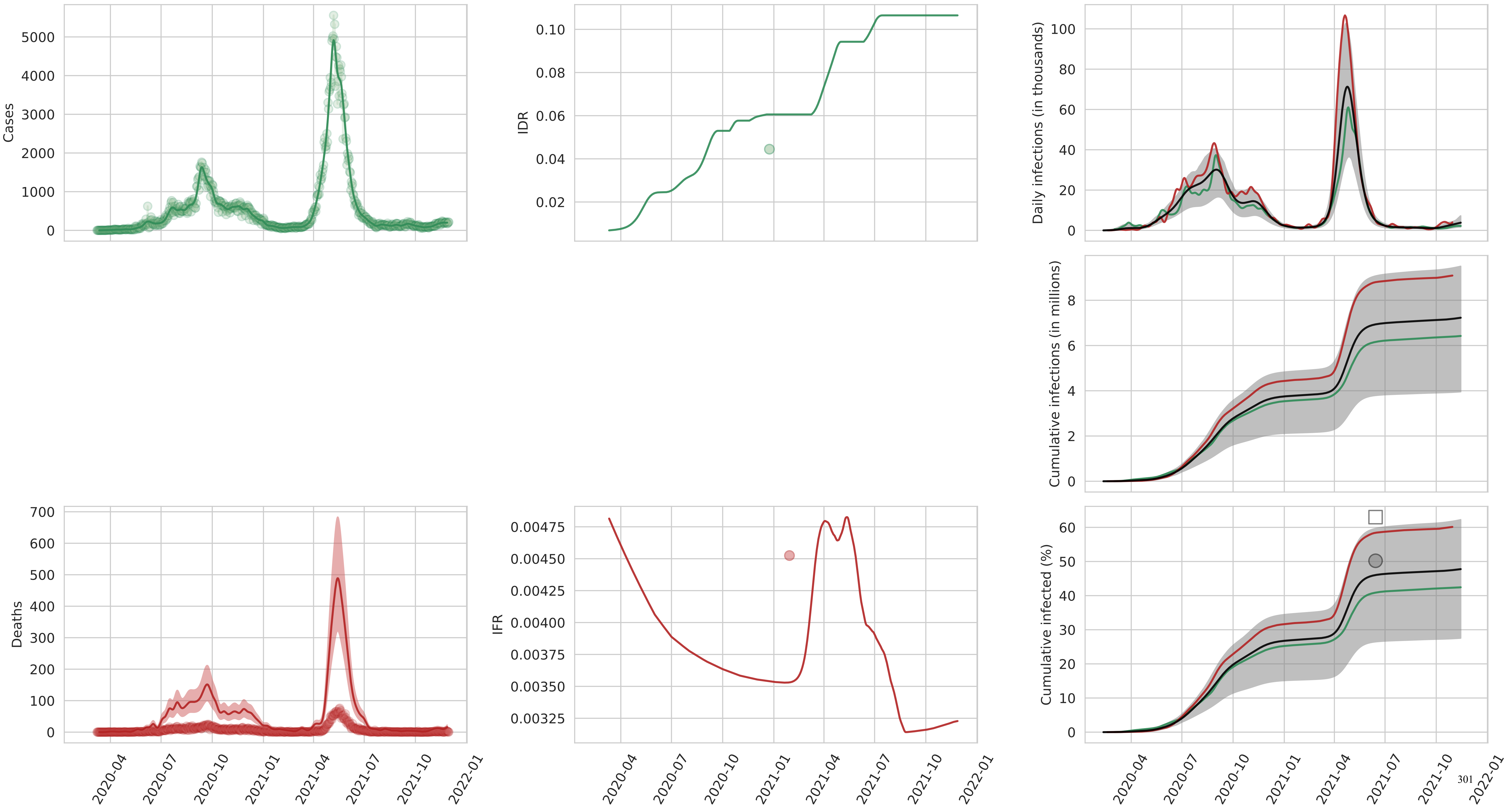

Jharkhand

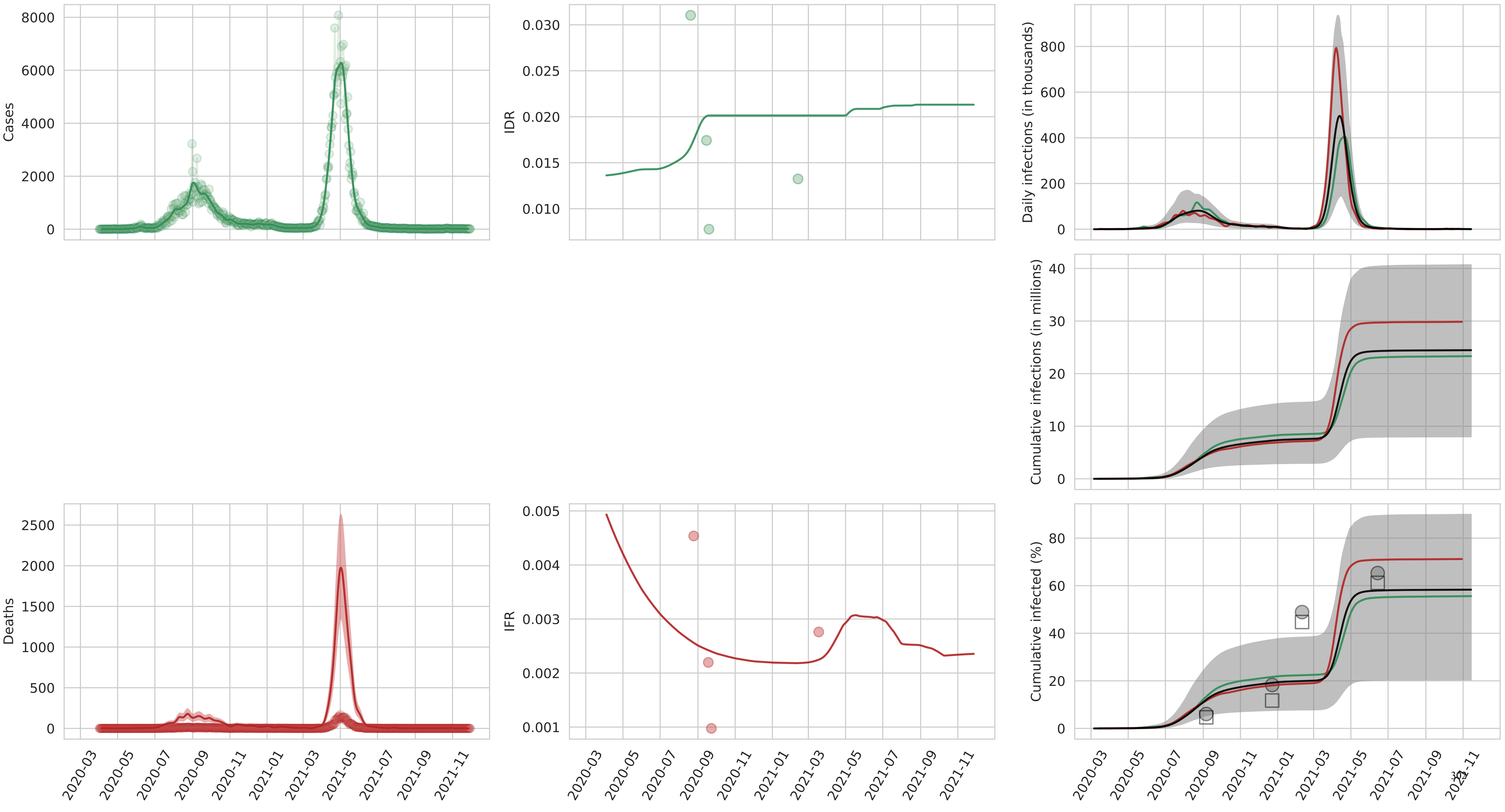

Cases
IDR
Daily infections (in thousands)
Cumulative infections (in millions)
Deaths
IFR
Cumulative infected (%)
2020-03
2020-05
2020-07
2020-09
2020-11
2021-01
2021-03
2021-05
2021-07
2021-09
2021-11

Karnataka

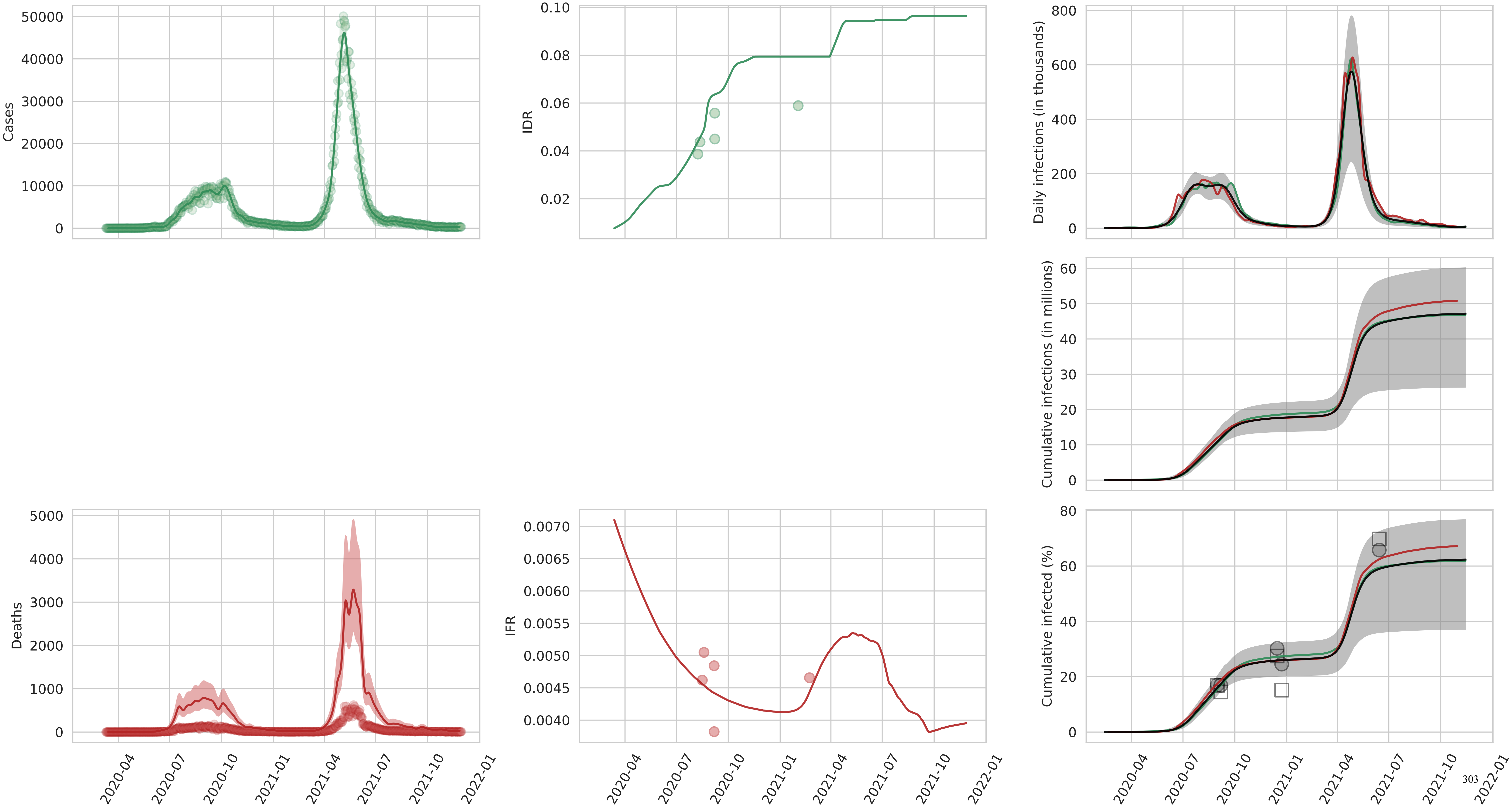

Cases
IDR
Daily infections (in thousands)
Cumulative infections (in millions)
Deaths
IFR
Cumulative infected (%)
2020-04
2020-07
2020-10
2021-01
2021-04
2021-07
2021-10
2022-01

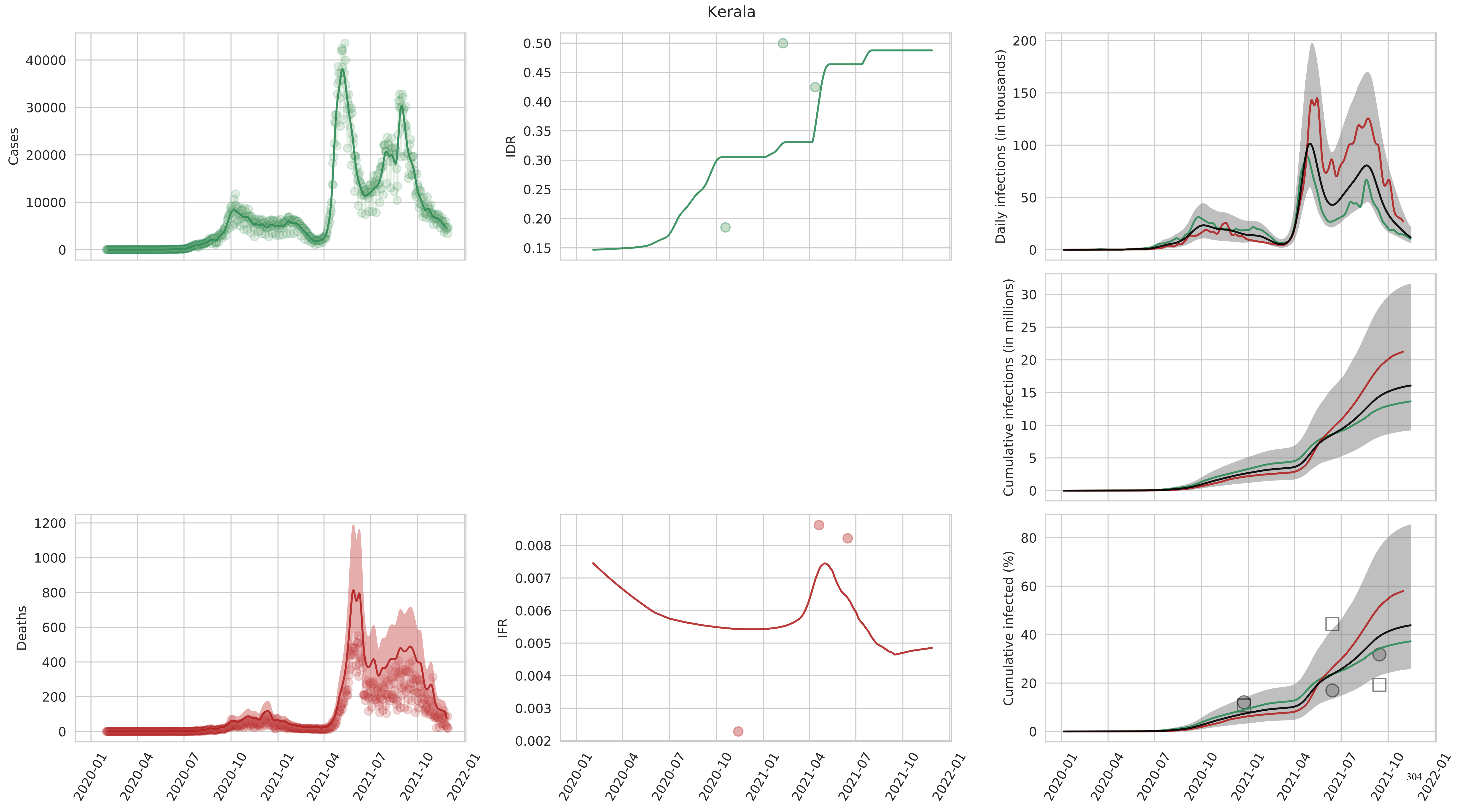
Kerala
Cases
IDR
Daily infections (in thousands)
Cumulative infections (in millions)
Deaths
IFR
Cumulative infected (%)
2020-01
2020-04
2020-07
2020-10
2021-01
2021-04
2021-07
2021-10
2022-01

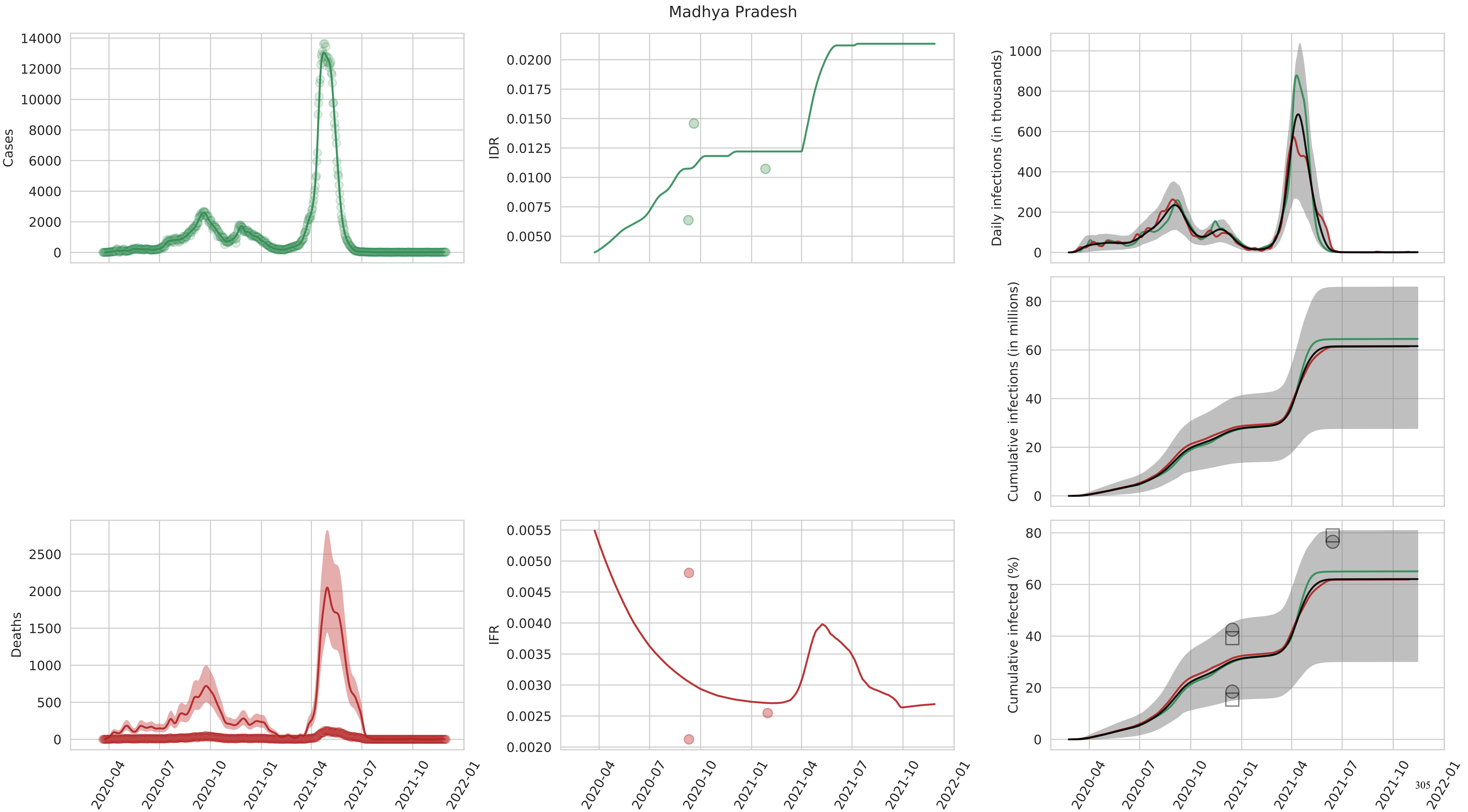
Madhya Pradesh
Cases
IDR
Daily infections (in thousands)
Cumulative infections (in millions)
Deaths
IFR
Cumulative infected (%)
2020-04
2020-07
2020-10
2021-01
2021-04
2021-07
2021-10
2022-01

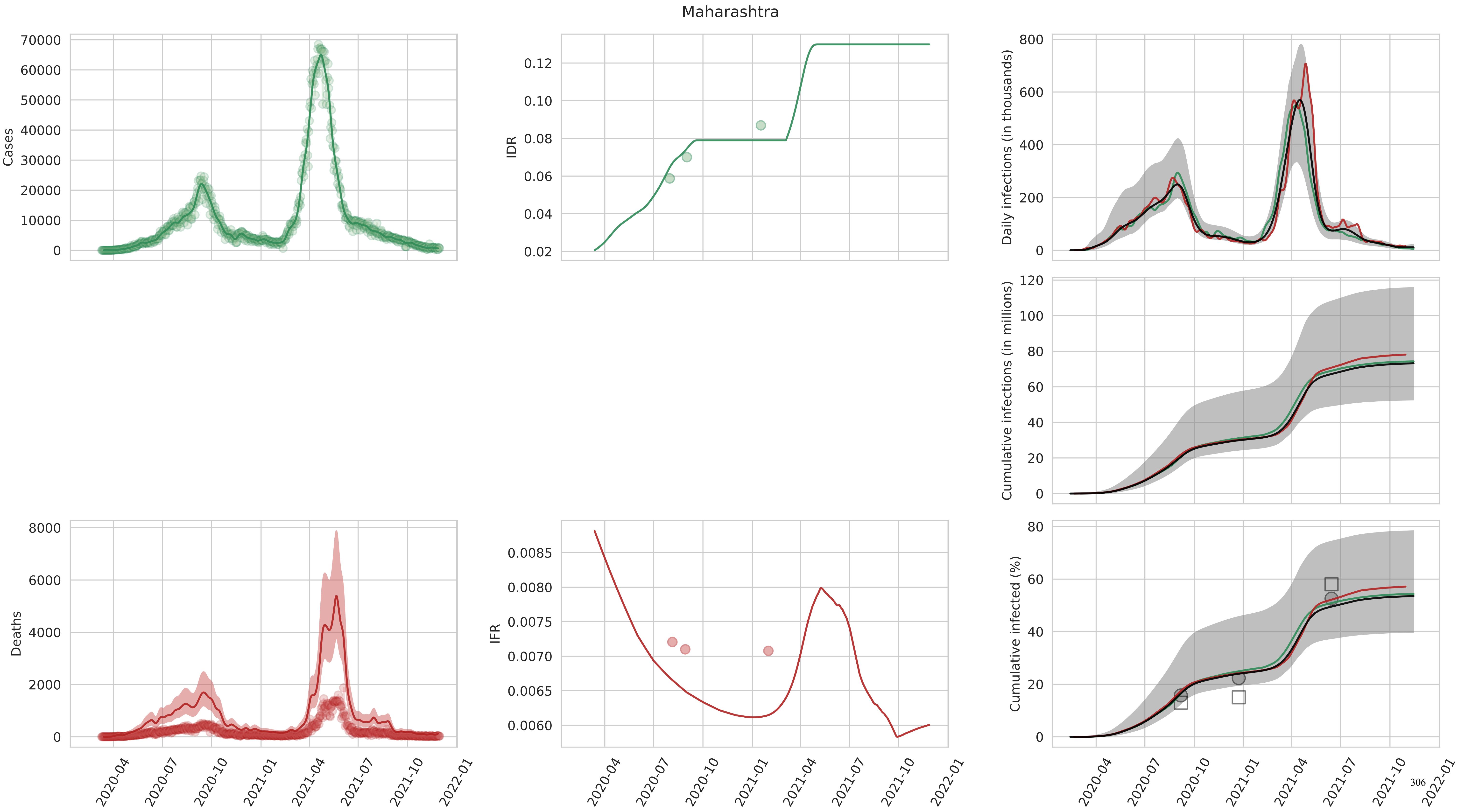
Maharashtra
Cases
IDR
Daily infections (in thousands)
Cumulative infections (in millions)
Deaths
IFR
Cumulative infected (%)
2020-04
2020-07
2020-10
2021-01
2021-04
2021-07
2021-10
2022-01

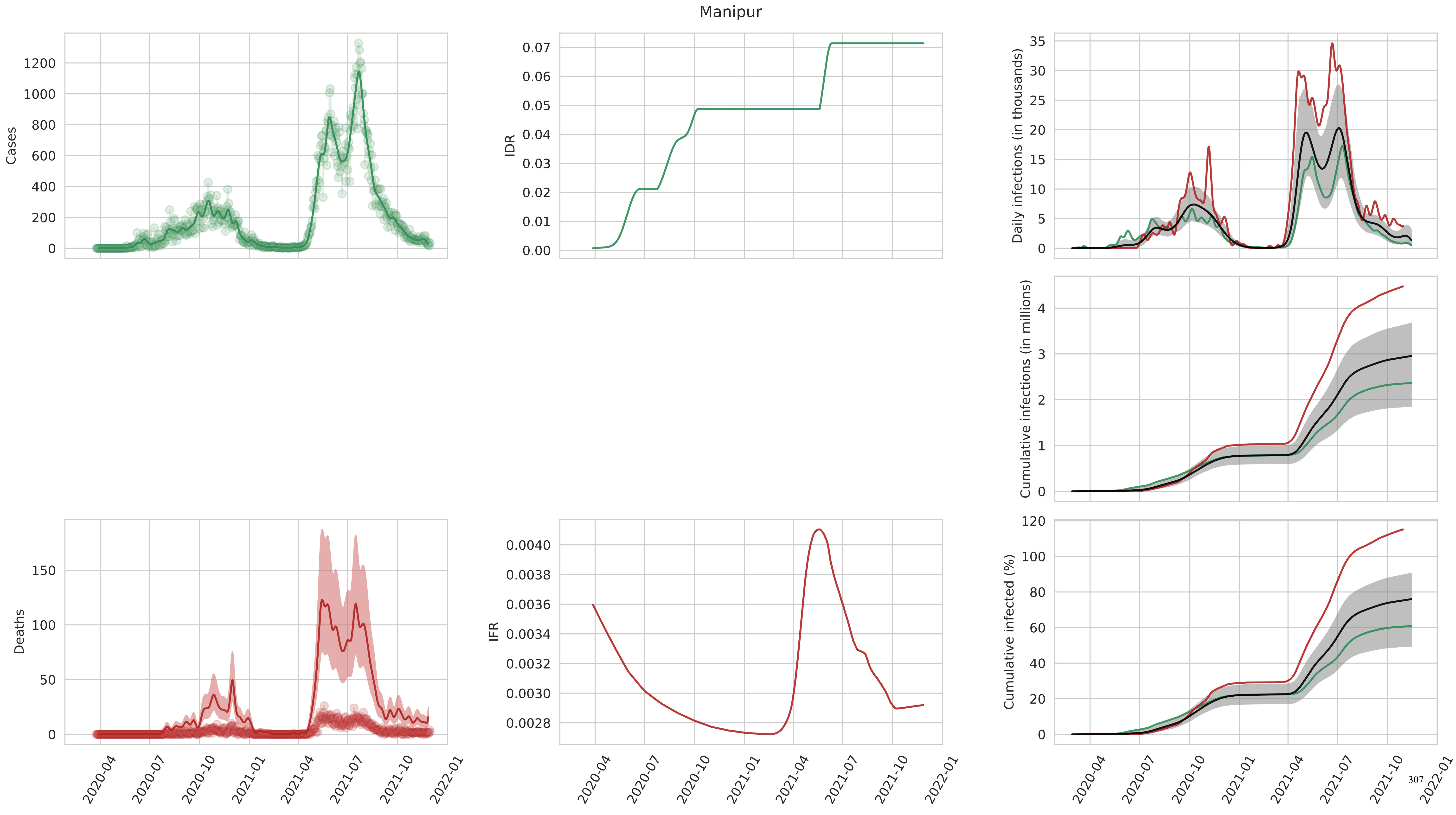
Manipur
Cases
IDR
Daily infections (in thousands)
Cumulative infections (in millions)
Deaths
IFR
Cumulative infected (%)
2020-04
2020-07
2020-10
2021-01
2021-04
2021-07
2021-10
2022-01

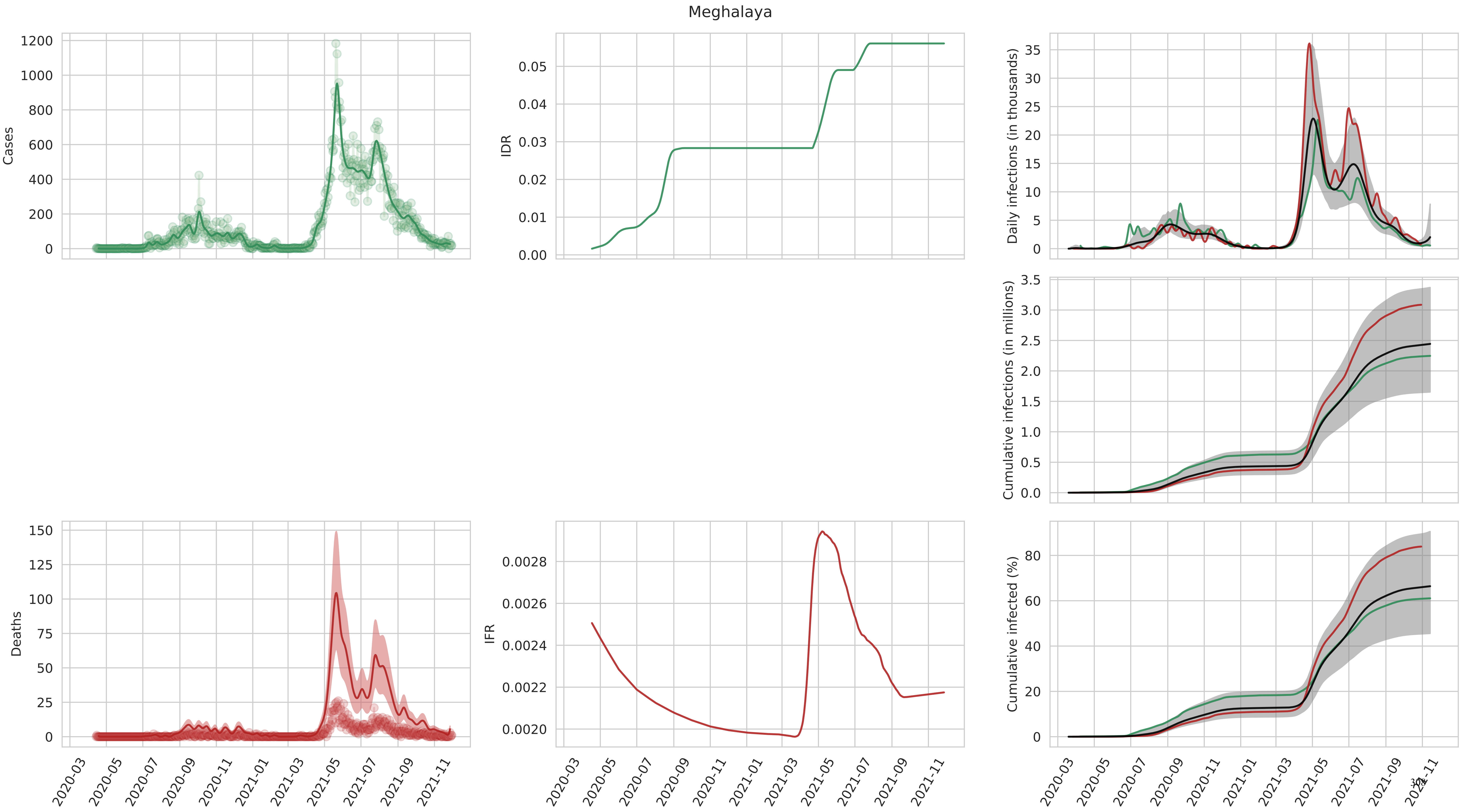
Meghalaya
Cases
1200
1000
800
600
400
200
0
IDR
0.05
0.04
0.03
0.02
0.01
0.00
Daily infections (in thousands)
35
30
25
20
15
10
5
0
Cumulative infections (in millions)
3.5
3.0
2.5
2.0
1.5
1.0
0.5
0.0
Deaths
150
125
100
75
50
25
0
IFR
0.0028
0.0026
0.0024
0.0022
0.0020
Cumulative infected (%)
80
60
40
20
0
2020-03
2020-05
2020-07
2020-09
2020-11
2021-01
2021-03
2021-05
2021-07
2021-09
2021-11

Mizoram

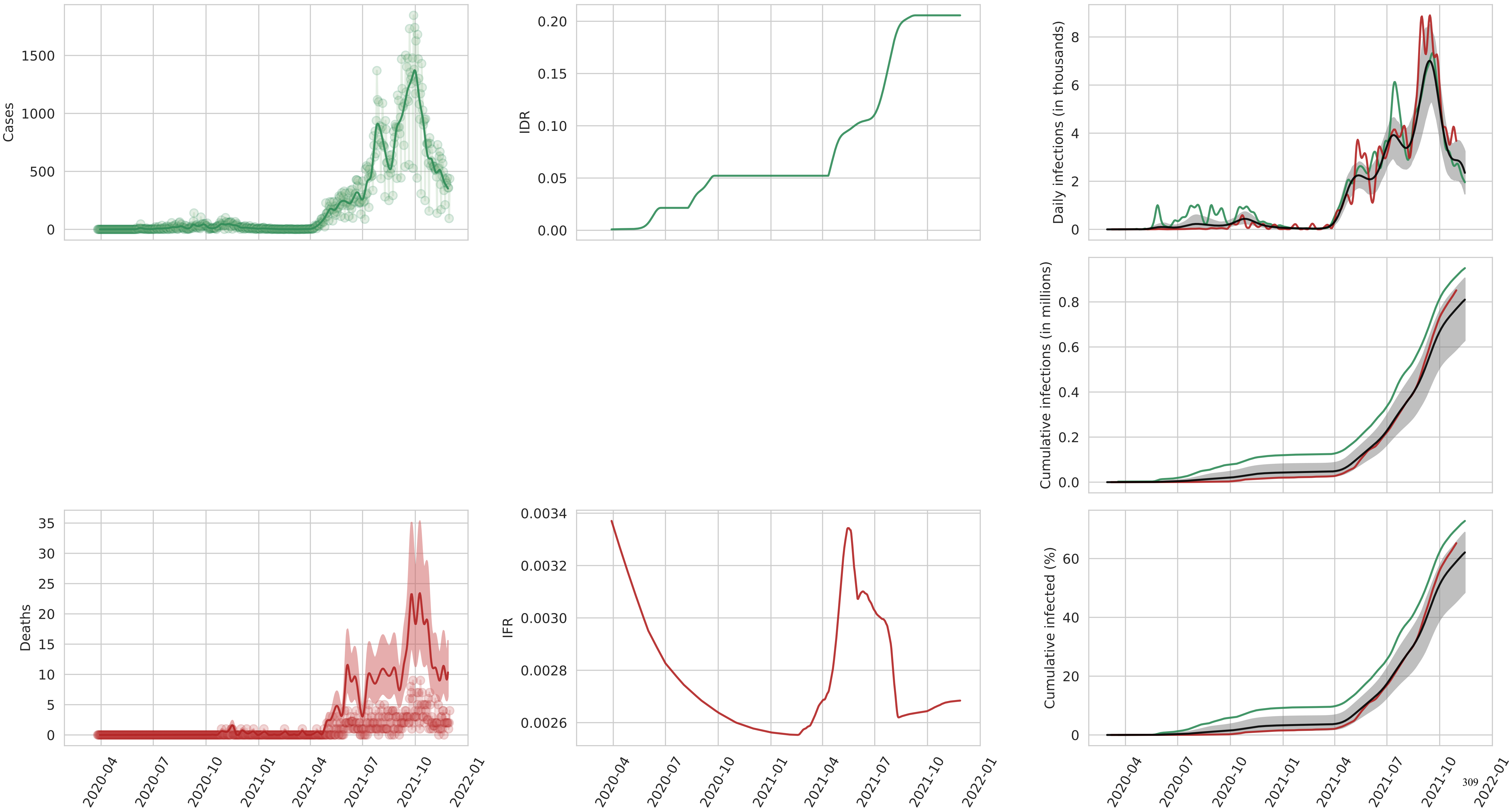

Cases
IDR
Daily infections (in thousands)
Cumulative infections (in millions)
Deaths
IFR
Cumulative infected (%)
2020-04
2020-07
2020-10
2021-01
2021-04
2021-07
2021-10
2022-01

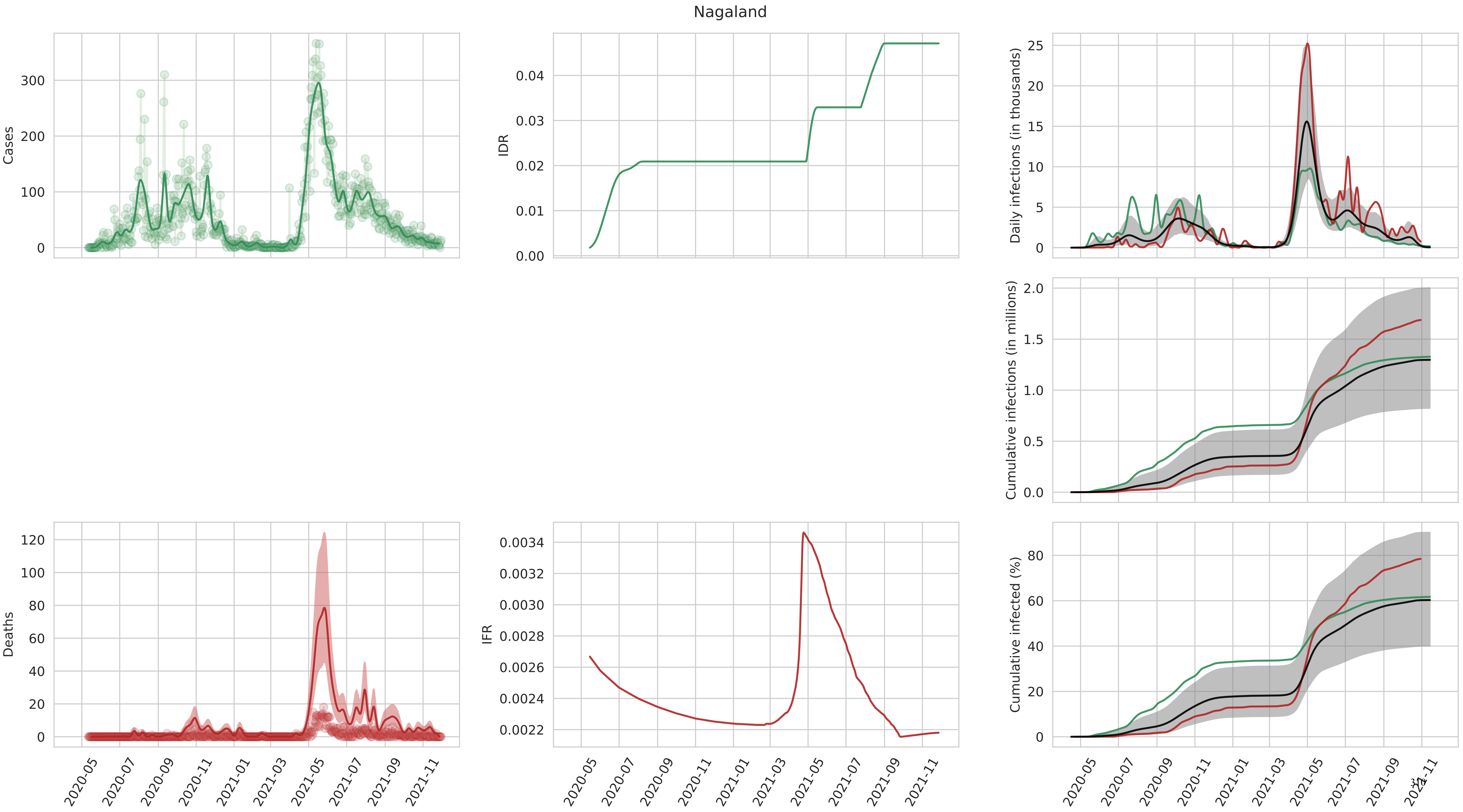

Nagaland
Cases
IDR
Daily infections (in thousands)
Cumulative infections (in millions)
Deaths
IFR
Cumulative infected (%)
2020-05
2020-07
2020-09
2020-11
2021-01
2021-03
2021-05
2021-07
2021-09
2021-11

Odisha

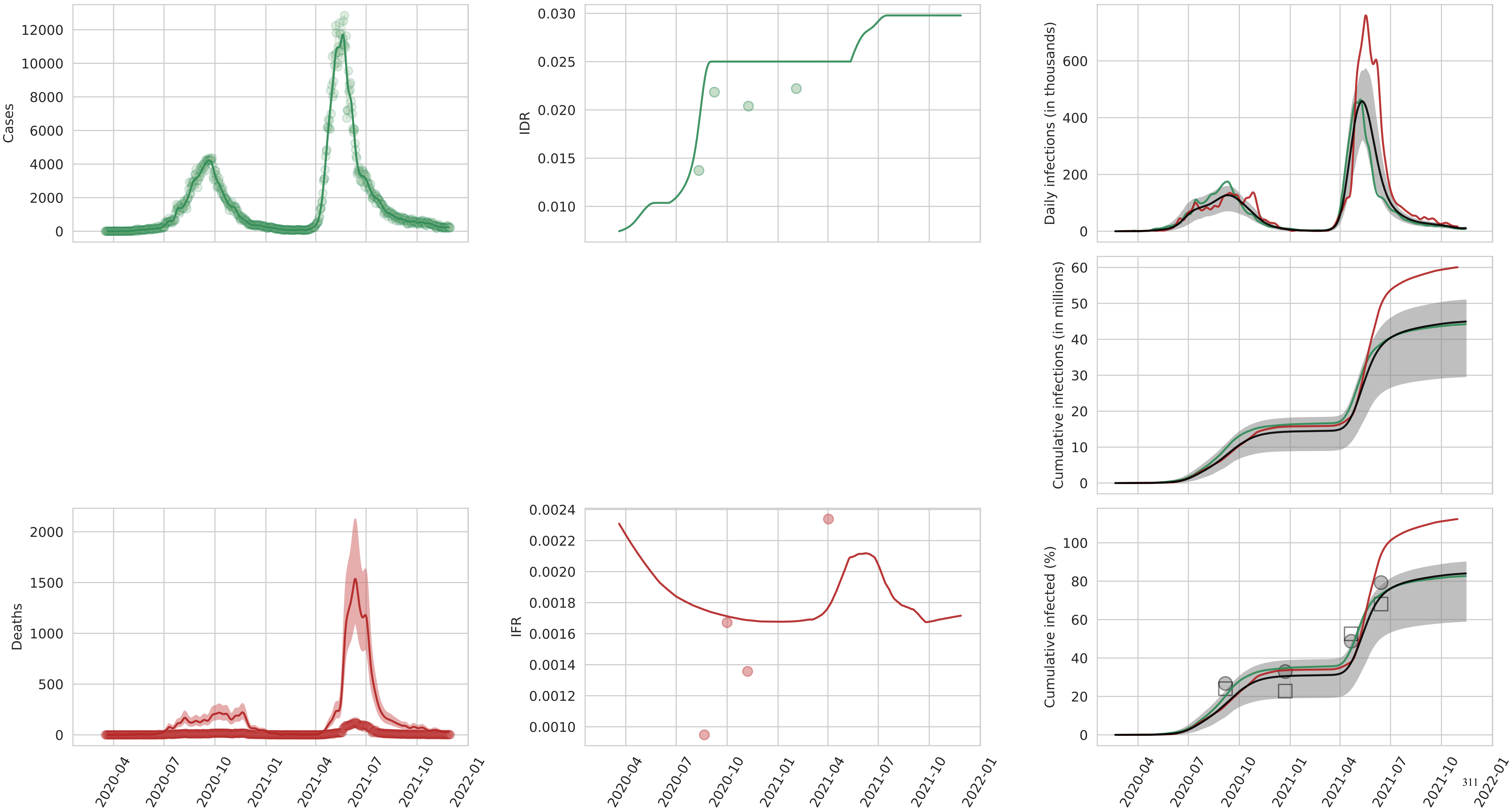

Cases
IDR
Daily infections (in thousands)
Cumulative infections (in millions)
Deaths
IFR
Cumulative infected (%)

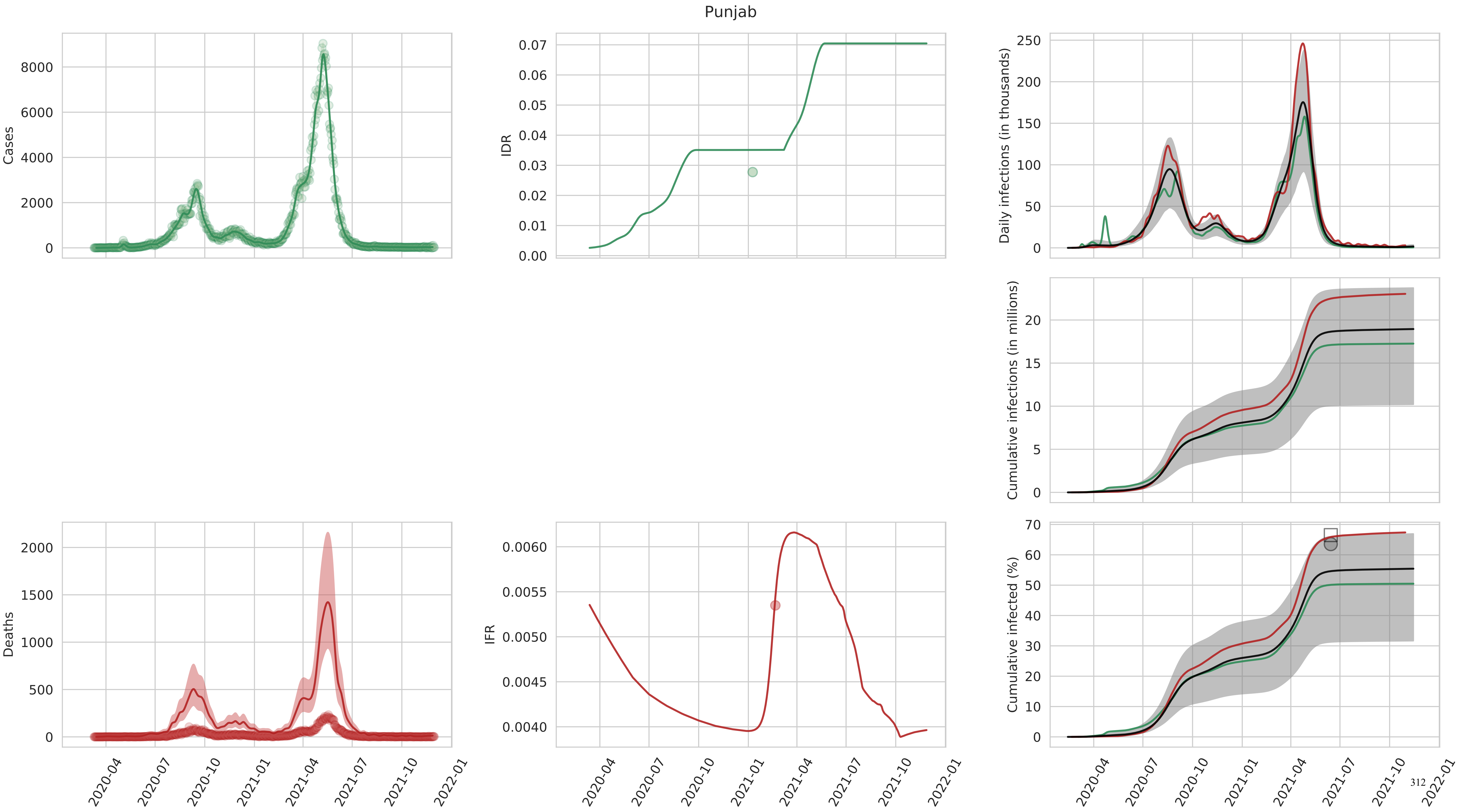
Punjab
Cases
IDR
Daily infections (in thousands)
Cumulative infections (in millions)
Deaths
IFR
Cumulative infected (%)
2020-04
2020-07
2020-10
2021-01
2021-04
2021-07
2021-10
2022-01

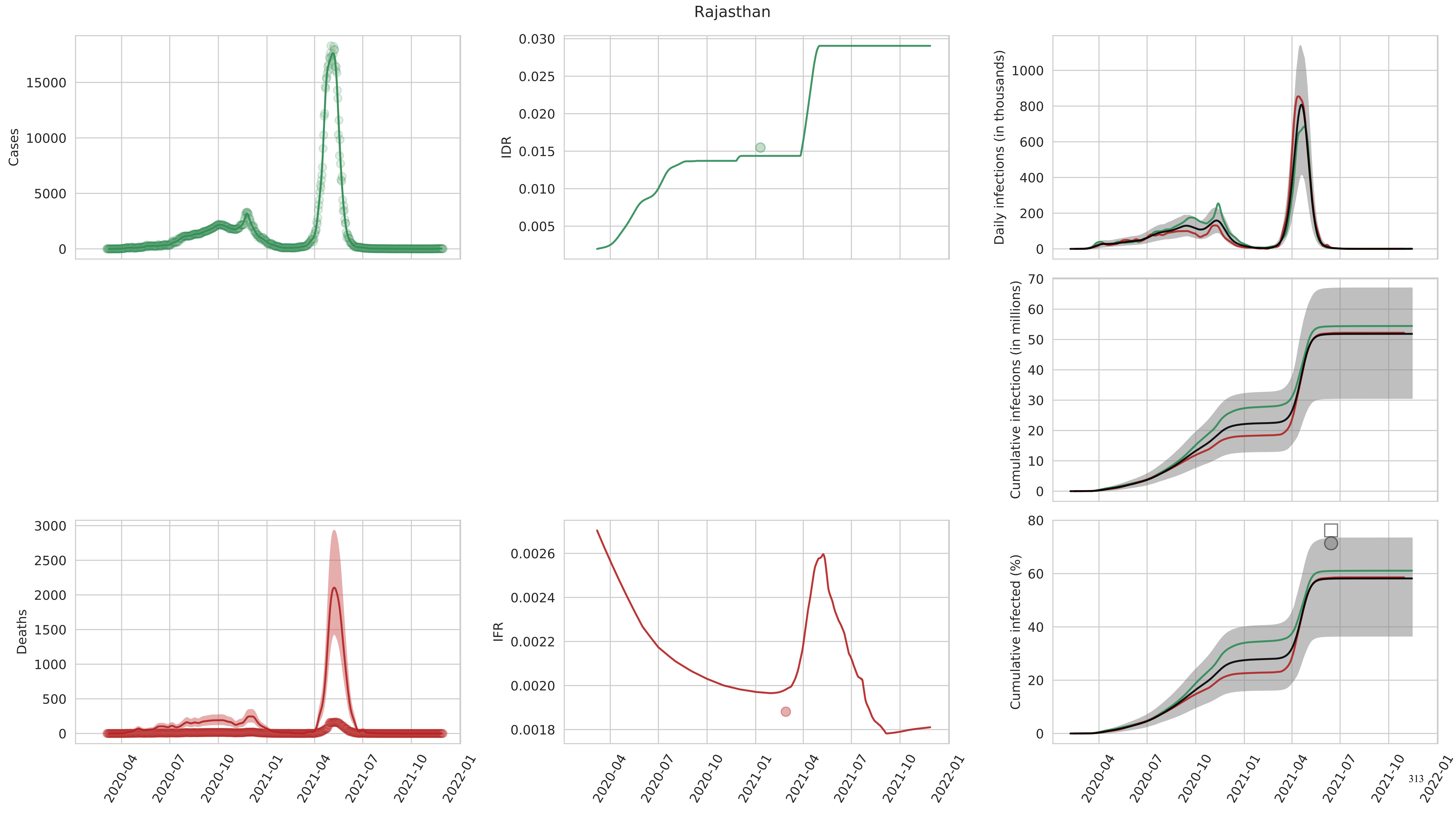
Rajasthan
Cases
Deaths
IDR
IFR
Daily infections (in thousands)
Cumulative infections (in millions)
Cumulative infected (%)
2020-04
2020-07
2020-10
2021-01
2021-04
2021-07
2021-10
2022-01

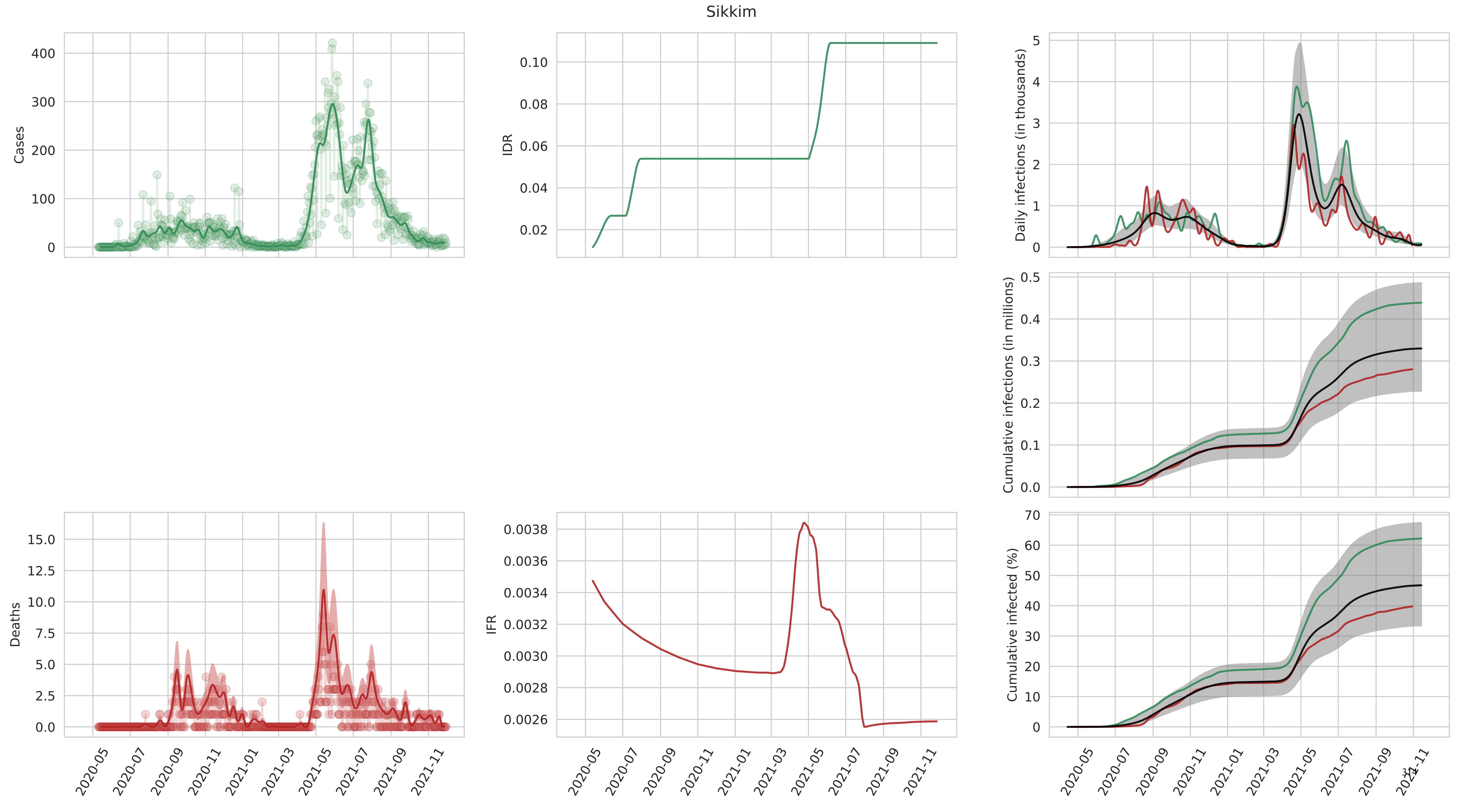
Sikkim
Cases
IDR
Daily infections (in thousands)
Cumulative infections (in millions)
Deaths
IFR
Cumulative infected (%)
2020-05
2020-07
2020-09
2020-11
2021-01
2021-03
2021-05
2021-07
2021-09
2021-11

Tamil Nadu

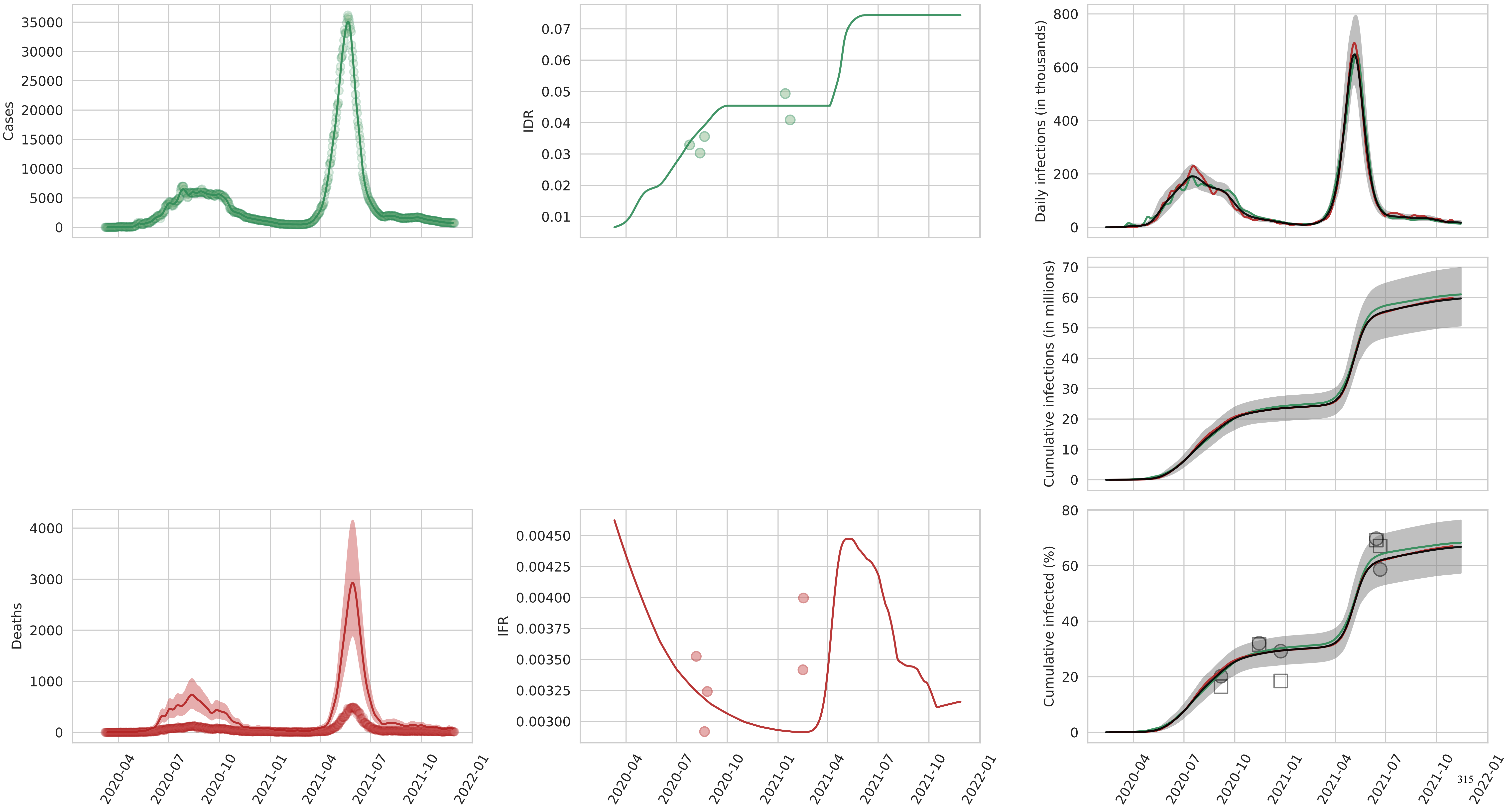
Cases
IDR
Daily infections (in thousands)
Cumulative infections (in millions)
Deaths
IFR
Cumulative infected (%)
2020-04
2020-07
2020-10
2021-01
2021-04
2021-07
2021-10
2022-01

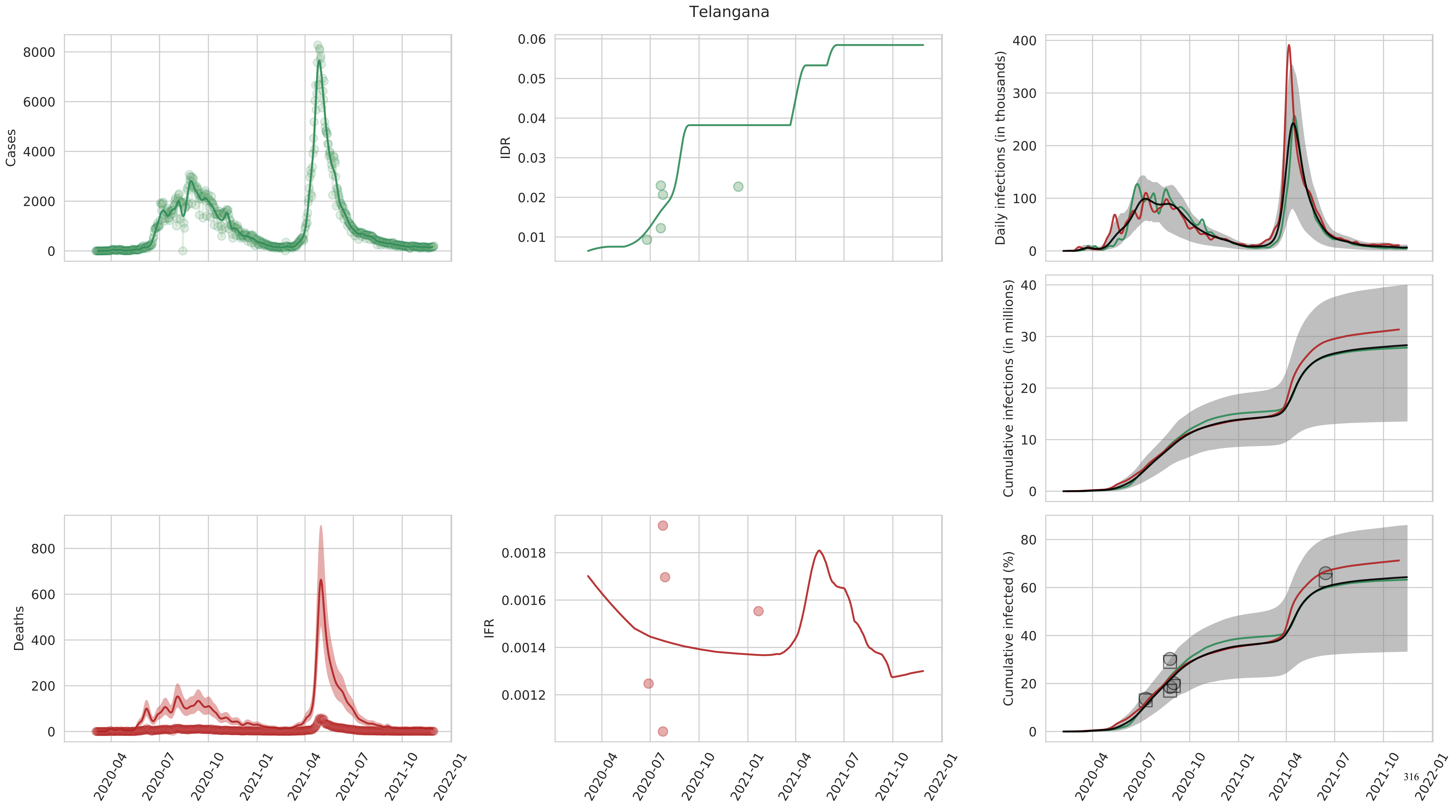
Telangana
Cases
IDR
Daily infections (in thousands)
Cumulative infections (in millions)
Deaths
IFR
Cumulative infected (%)
2020-04
2020-07
2020-10
2021-01
2021-04
2021-07
2021-10
2022-01

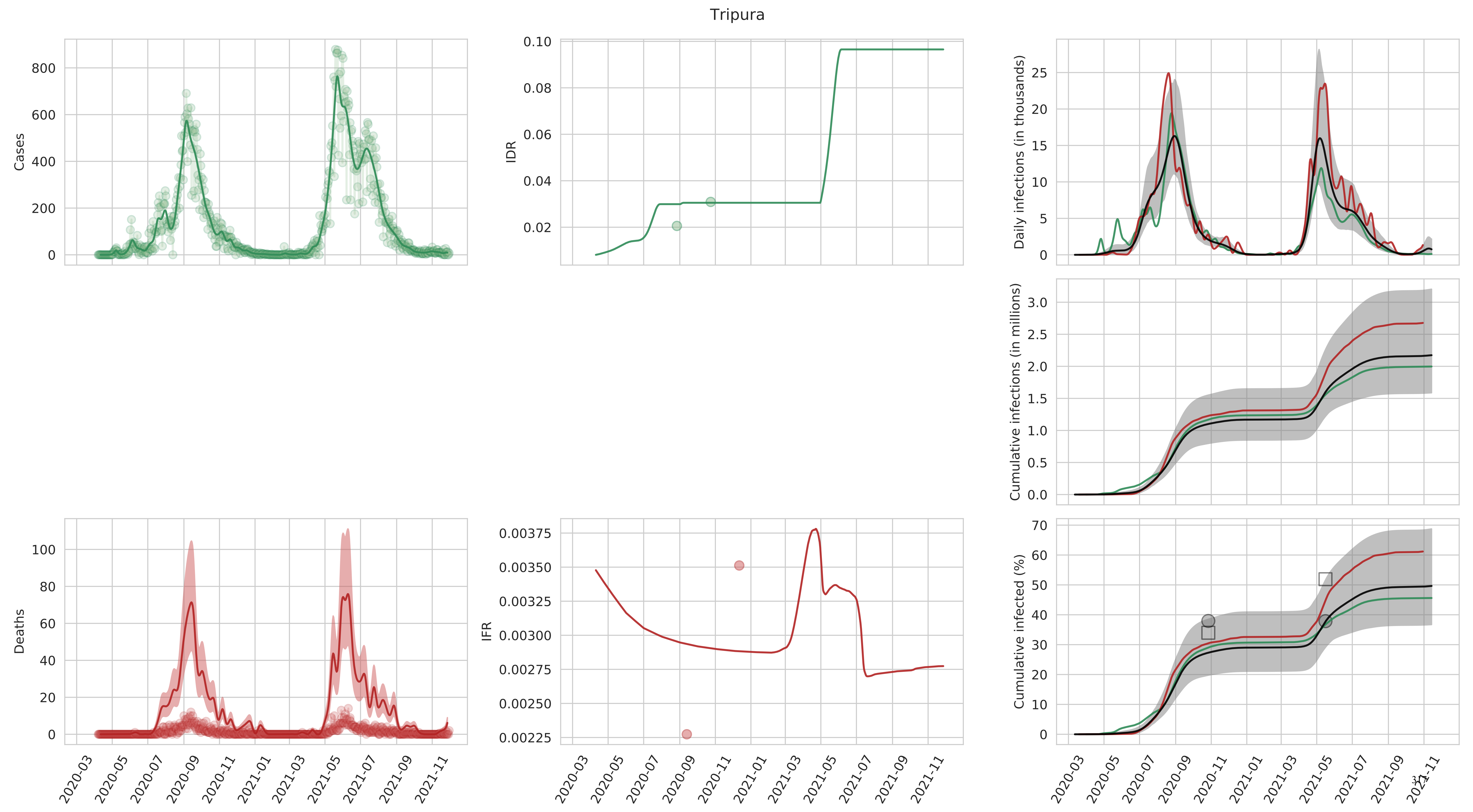
Tripura
Cases
IDR
Daily infections (in thousands)
Cumulative infections (in millions)
Deaths
IFR
Cumulative infected (%)
2020-03
2020-05
2020-07
2020-09
2020-11
2021-01
2021-03
2021-05
2021-07
2021-09
2021-11

Uttar Pradesh

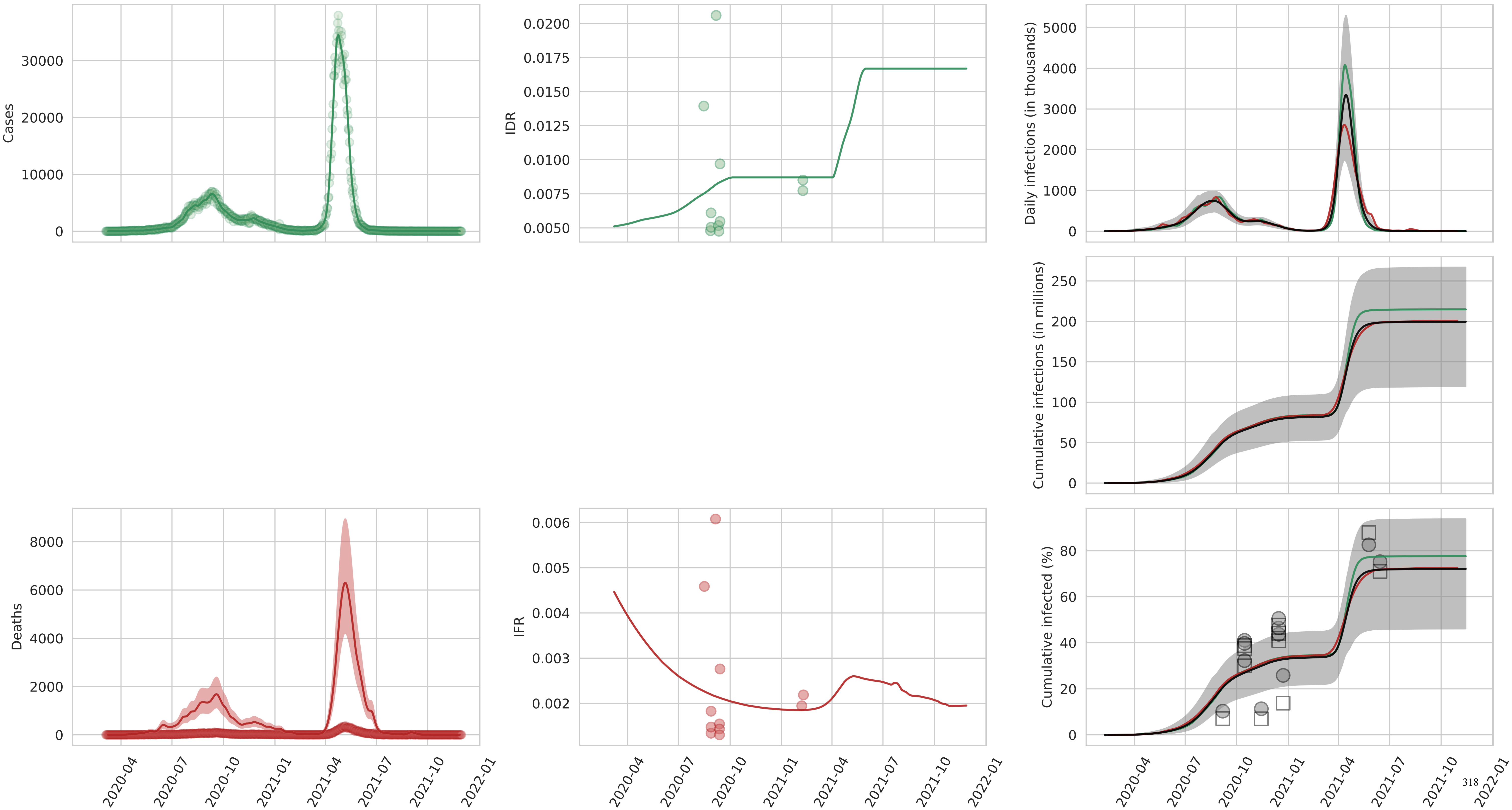

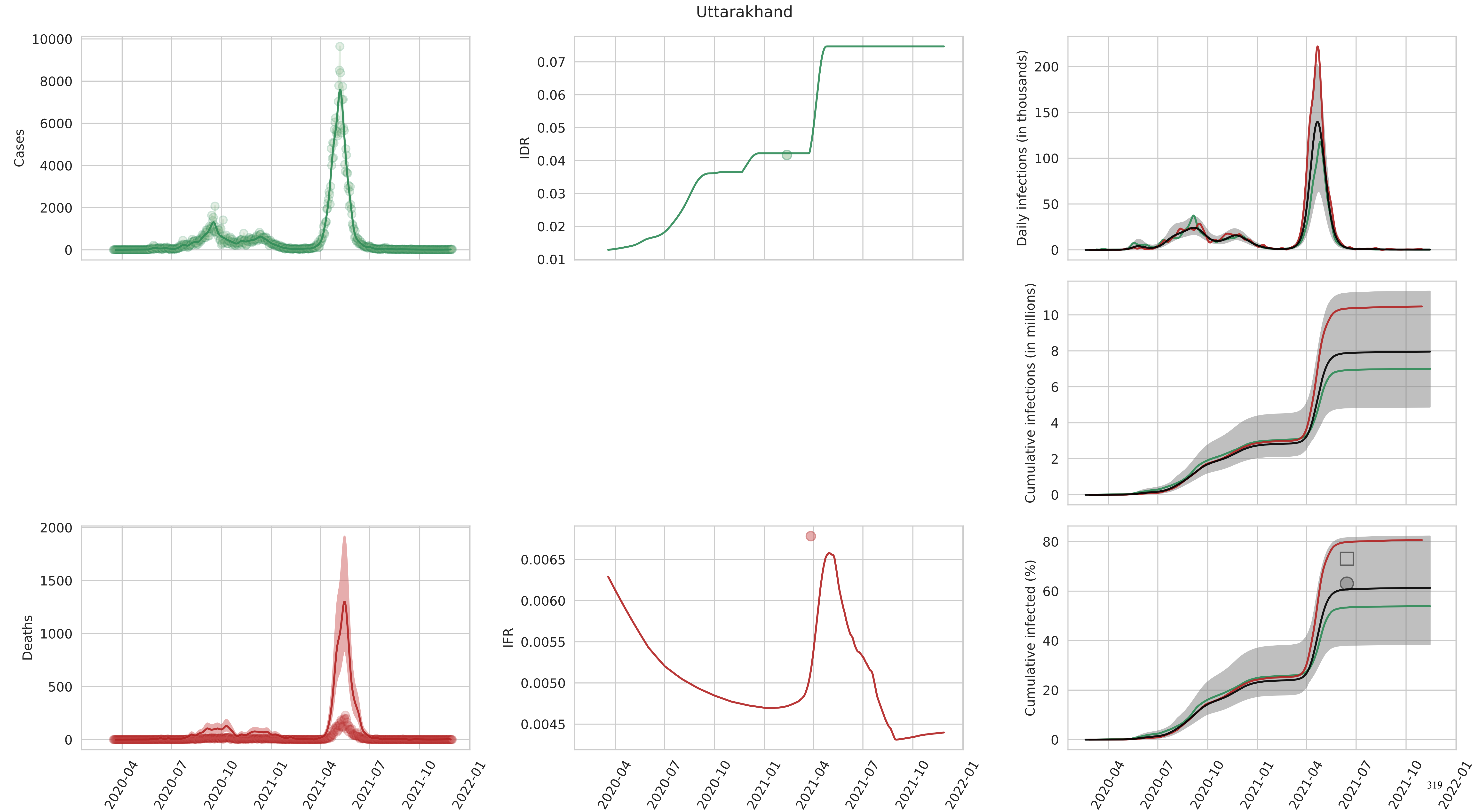

Uttarakhand
Cases
IDR
Daily infections (in thousands)
Cumulative infections (in millions)
Deaths
IFR
Cumulative infected (%)

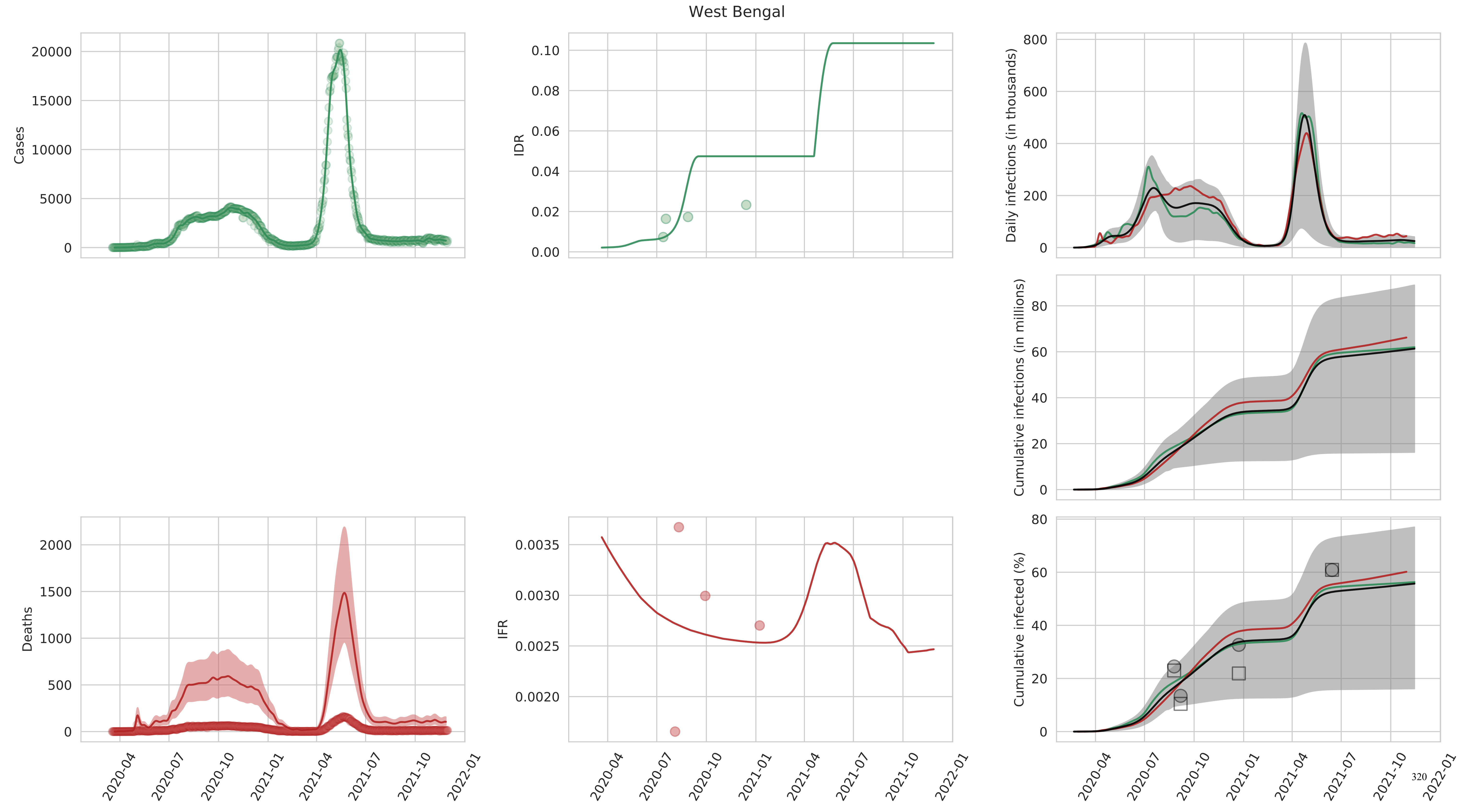

West Bengal
Cases
IDR
Daily infections (in thousands)
Cumulative infections (in millions)
Deaths
IFR
Cumulative infected (%)
2020-04
2020-07
2020-10
2021-01
2021-04
2021-07
2021-10
2022-01

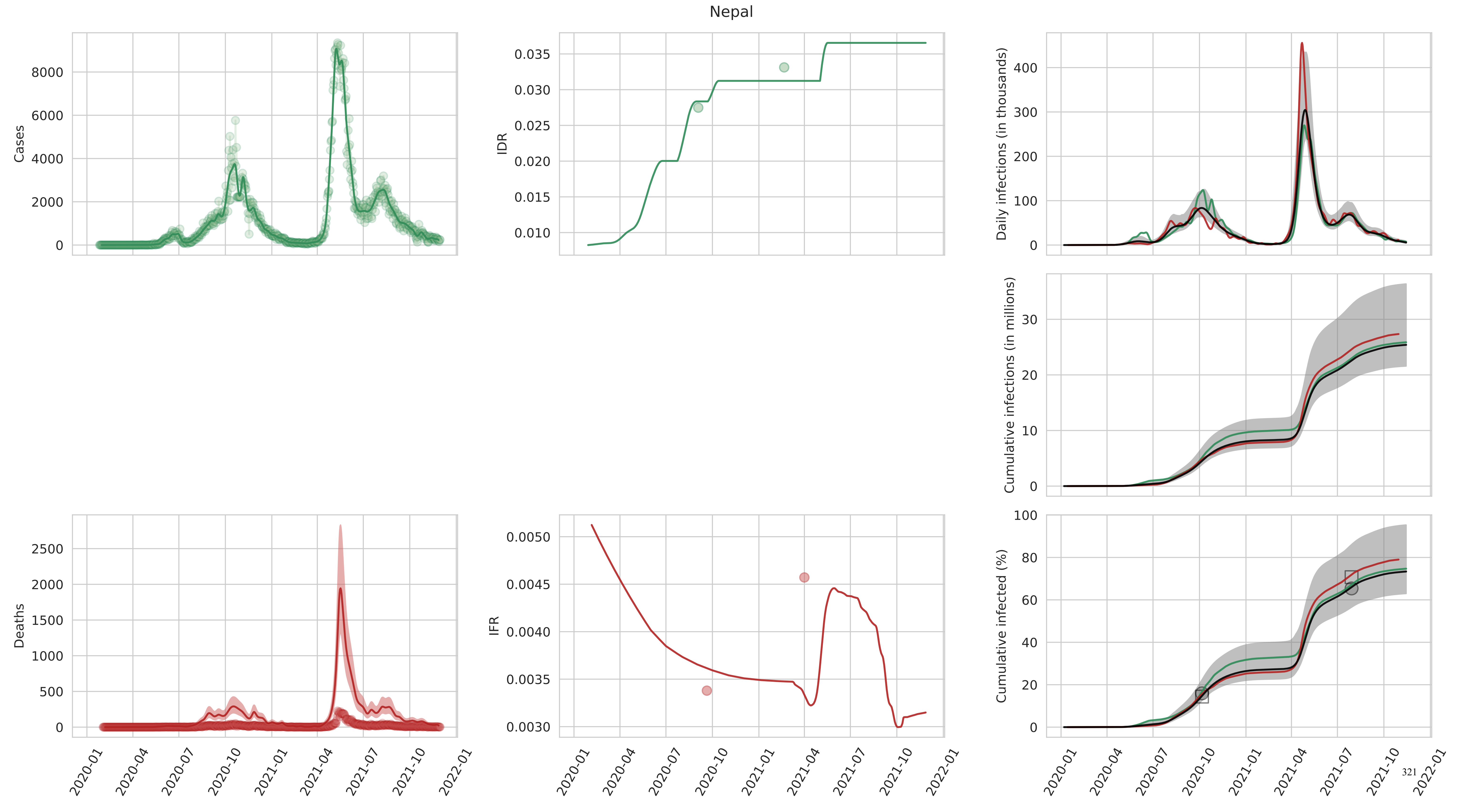

Nepal
Cases
IDR
Daily infections (in thousands)
Cumulative infections (in millions)
Deaths
IFR
Cumulative infected (%)

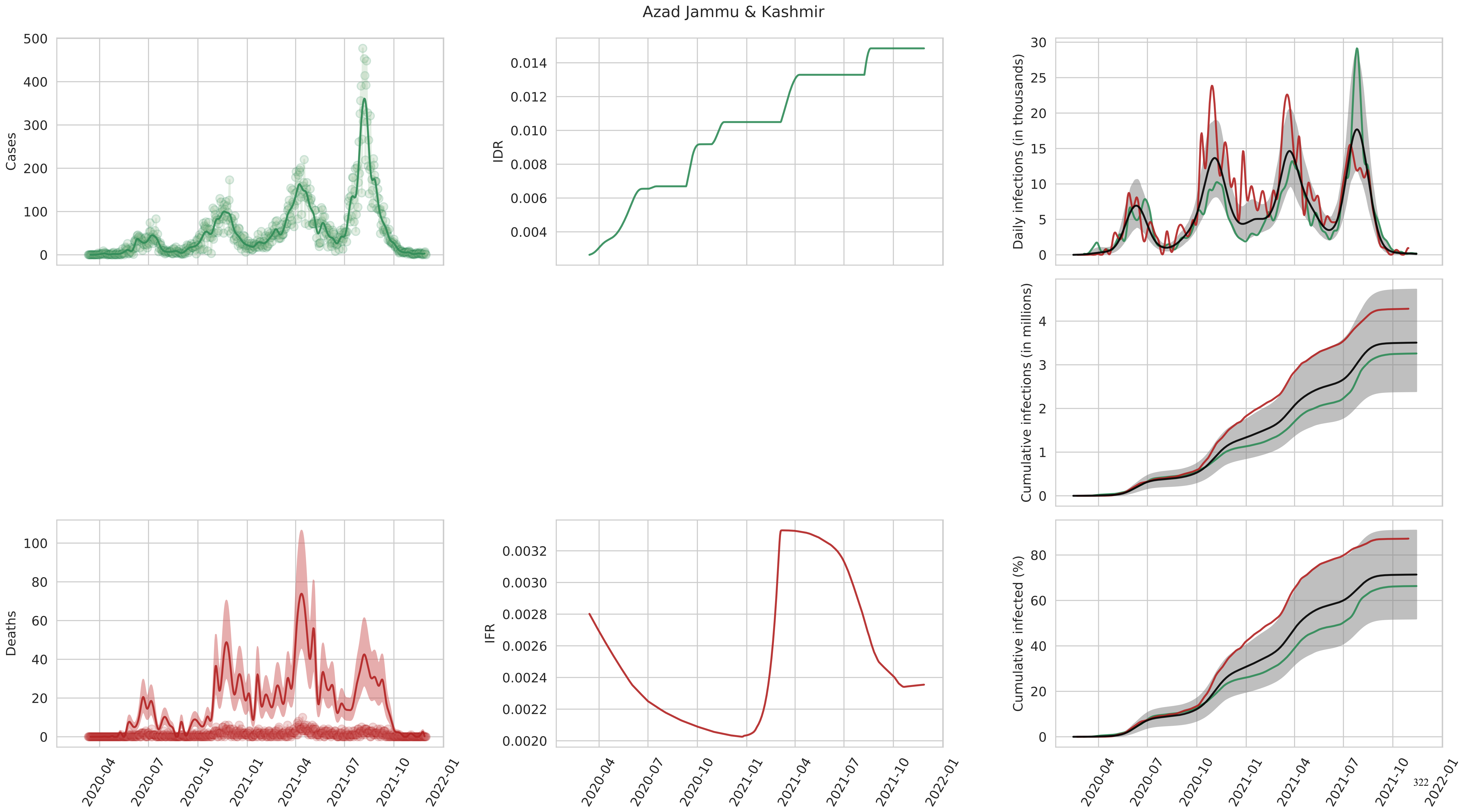

Azad Jammu & Kashmir
Cases
IDR
Daily infections (in thousands)
Cumulative infections (in millions)
Deaths
IFR
Cumulative infected (%)
2020-04
2020-07
2020-10
2021-01
2021-04
2021-07
2021-10
2022-01

Balochistan

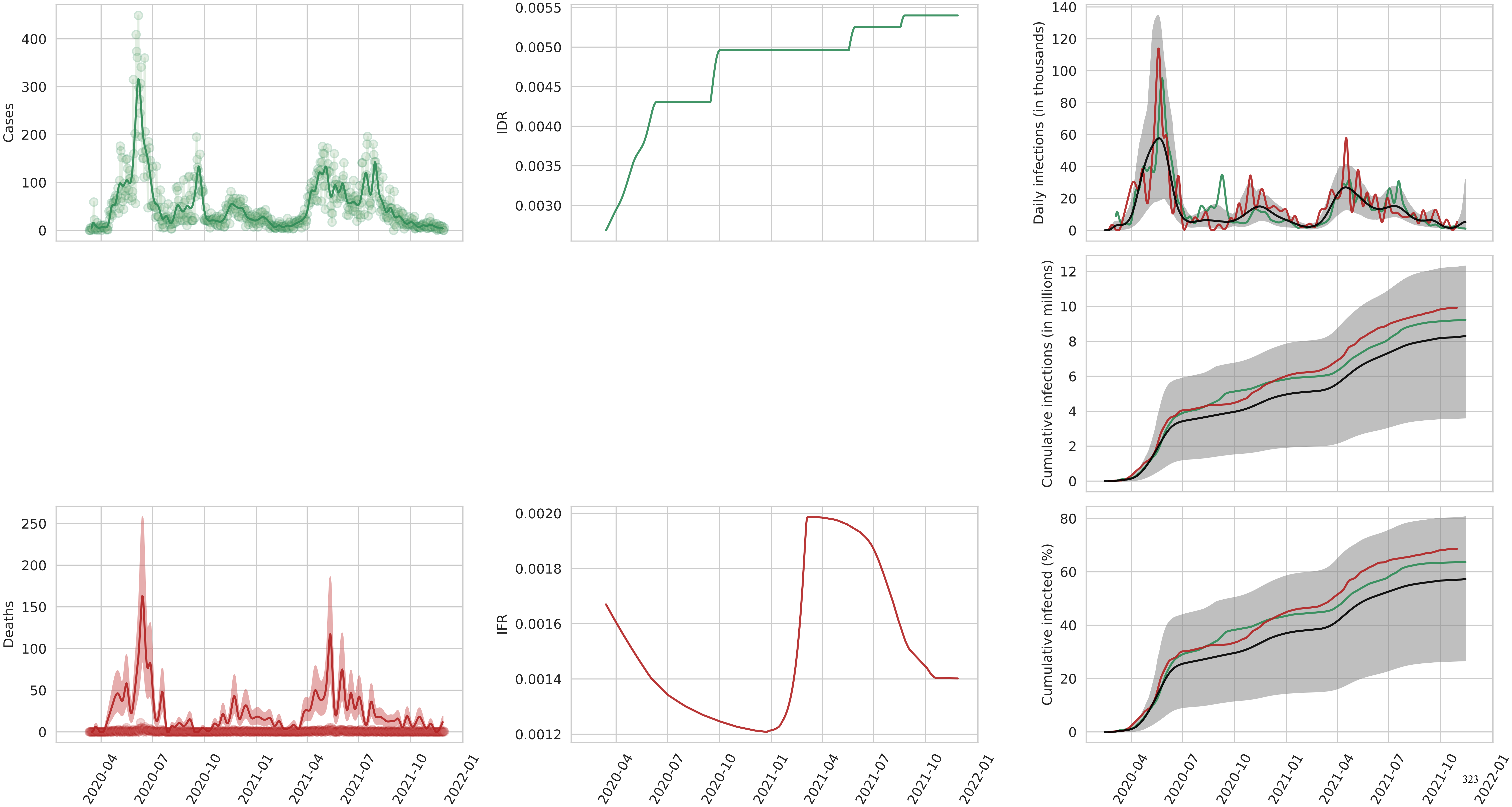

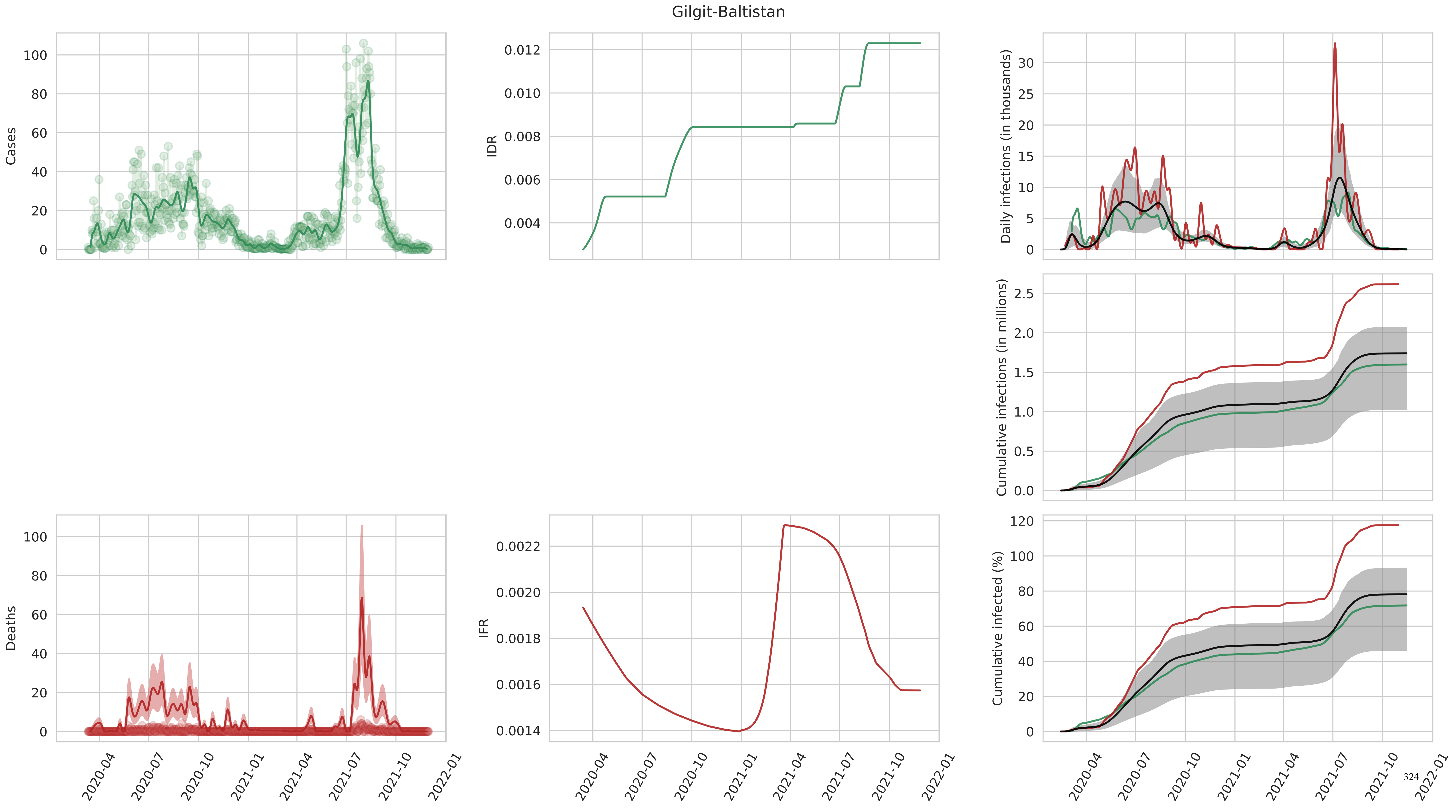
Gilgit-Baltistan
Cases
IDR
Daily infections (in thousands)
Cumulative infections (in millions)
Deaths
IFR
Cumulative infected (%)
2020-04
2020-07
2020-10
2021-01
2021-04
2021-07
2021-10
2022-01

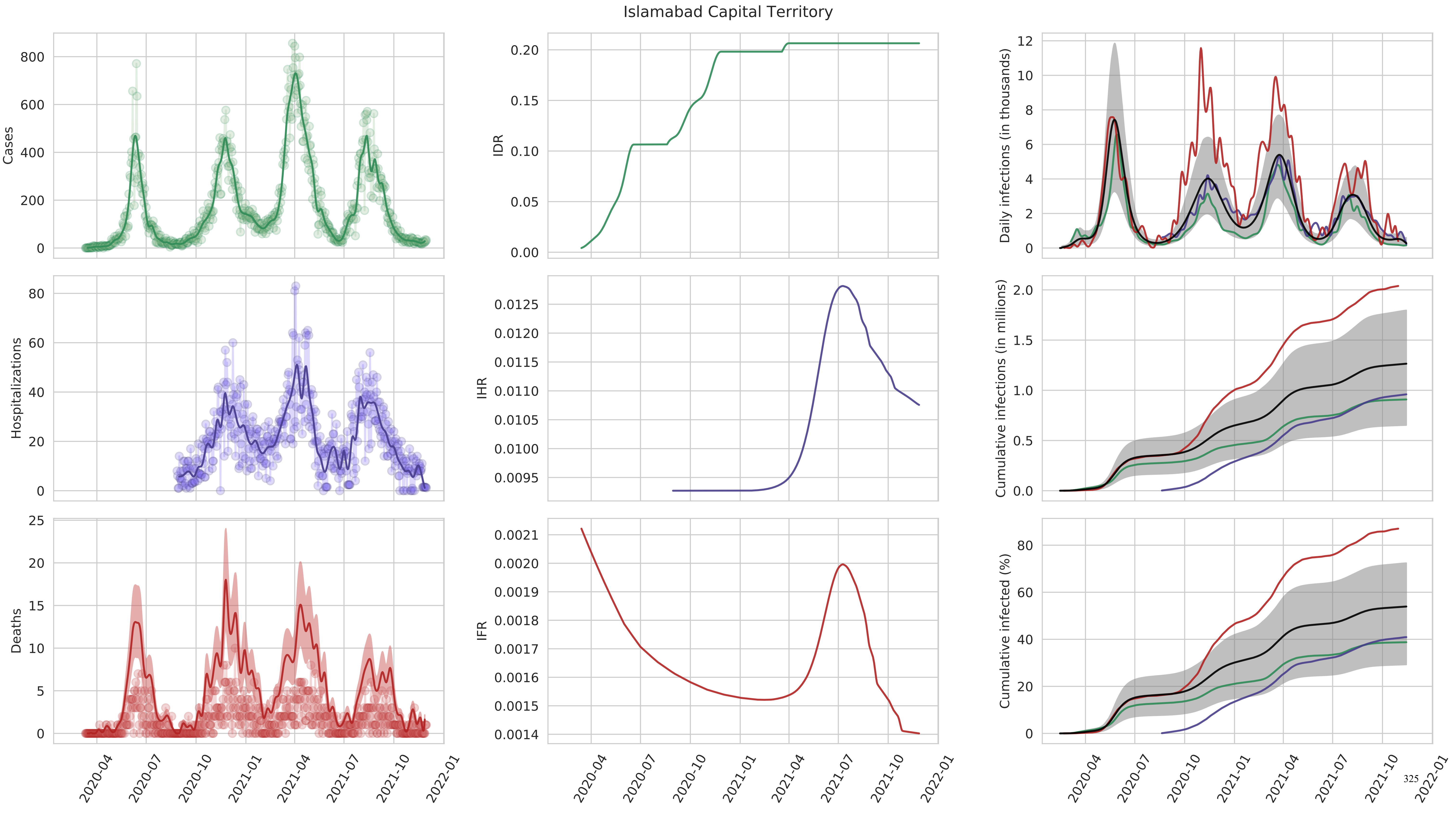
Islamabad Capital Territory
Cases
Hospitalizations
Deaths
IDR
IHR
IFR
Daily infections (in thousands)
Cumulative infections (in millions)
Cumulative infected (%)
2020-04
2020-07
2020-10
2021-01
2021-04
2021-07
2021-10
2022-01

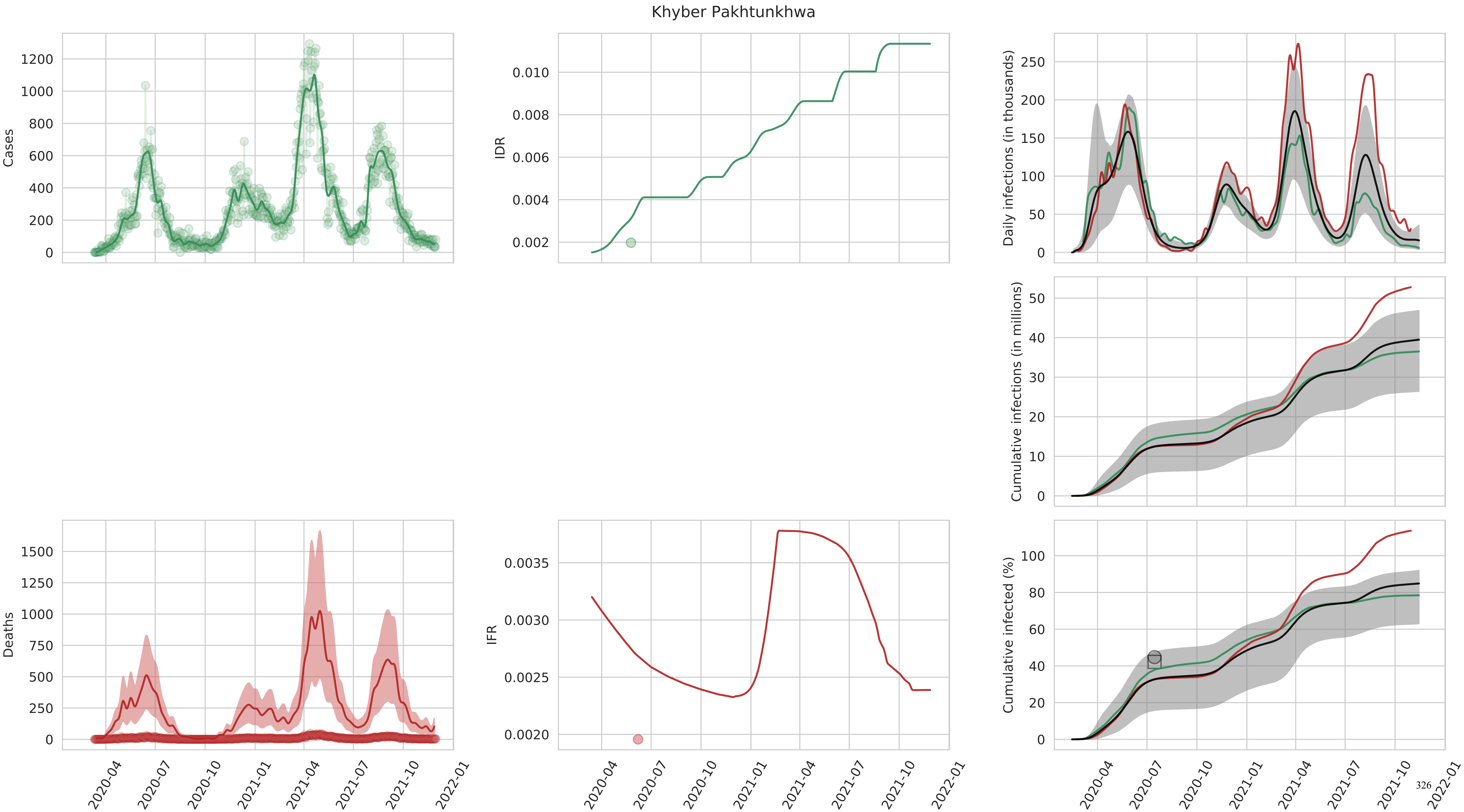

Khyber Pakhtunkhwa
Cases
IDR
Daily infections (in thousands)
Cumulative infections (in millions)
Deaths
IFR
Cumulative infected (%)
2020-04
2020-07
2020-10
2021-01
2021-04
2021-07
2021-10
2022-01

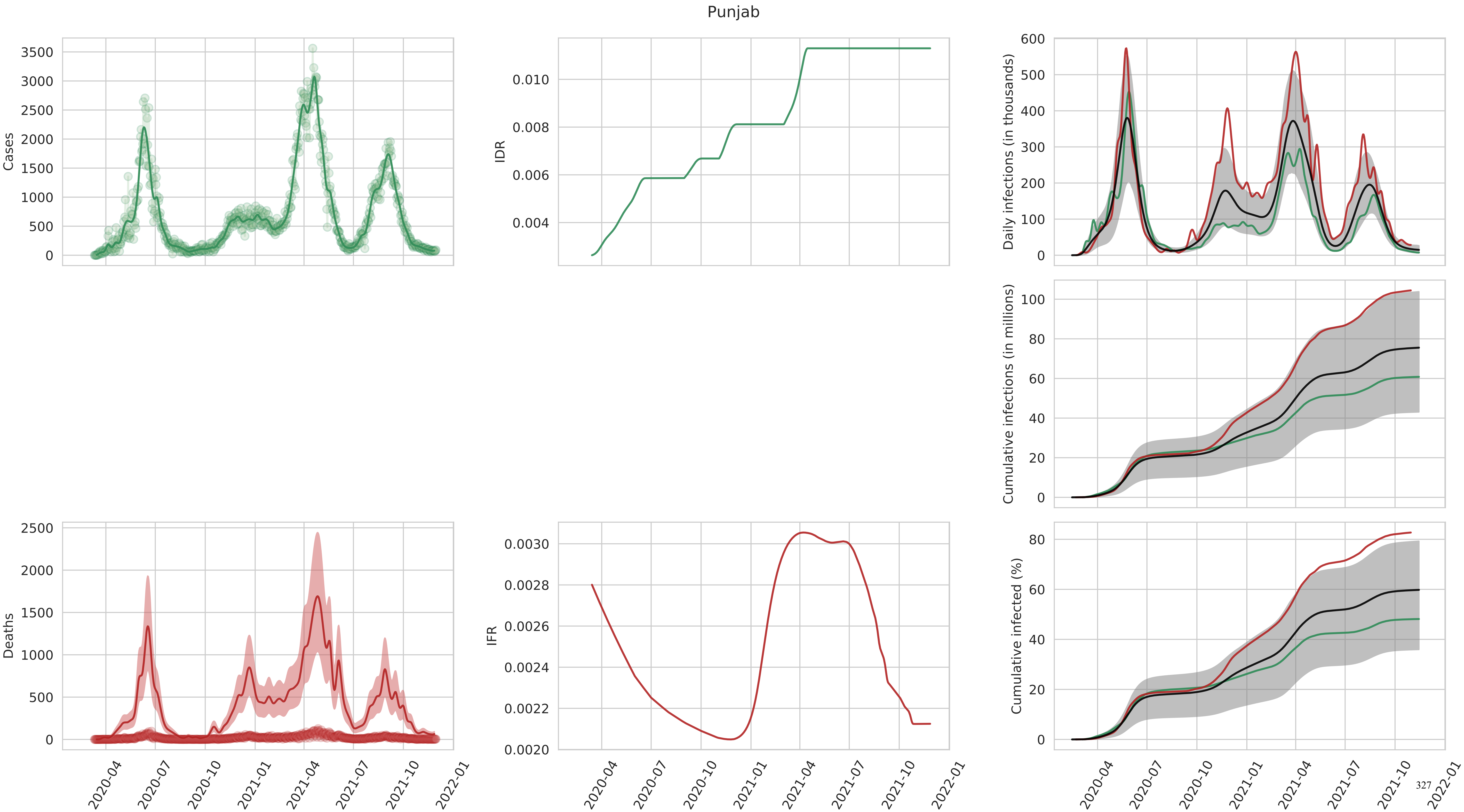

Punjab
Cases
IDR
Daily infections (in thousands)
Cumulative infections (in millions)
Deaths
IFR
Cumulative infected (%)
2020-04
2020-07
2020-10
2021-01
2021-04
2021-07
2021-10
2022-01

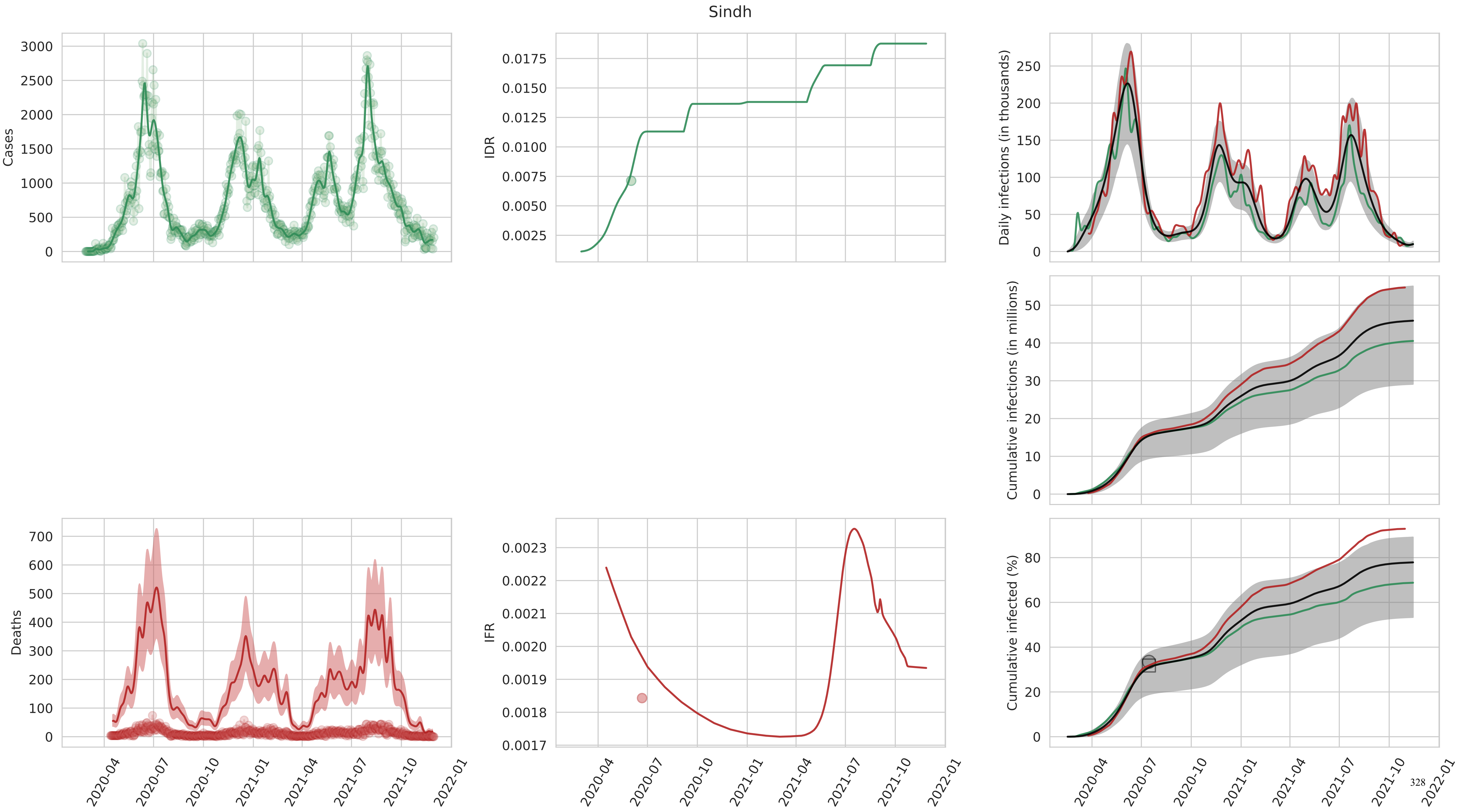
Sindh
Cases
IDR
Daily infections (in thousands)
Cumulative infections (in millions)
Deaths
IFR
Cumulative infected (%)
2020-04
2020-07
2020-10
2021-01
2021-04
2021-07
2021-10
2022-01

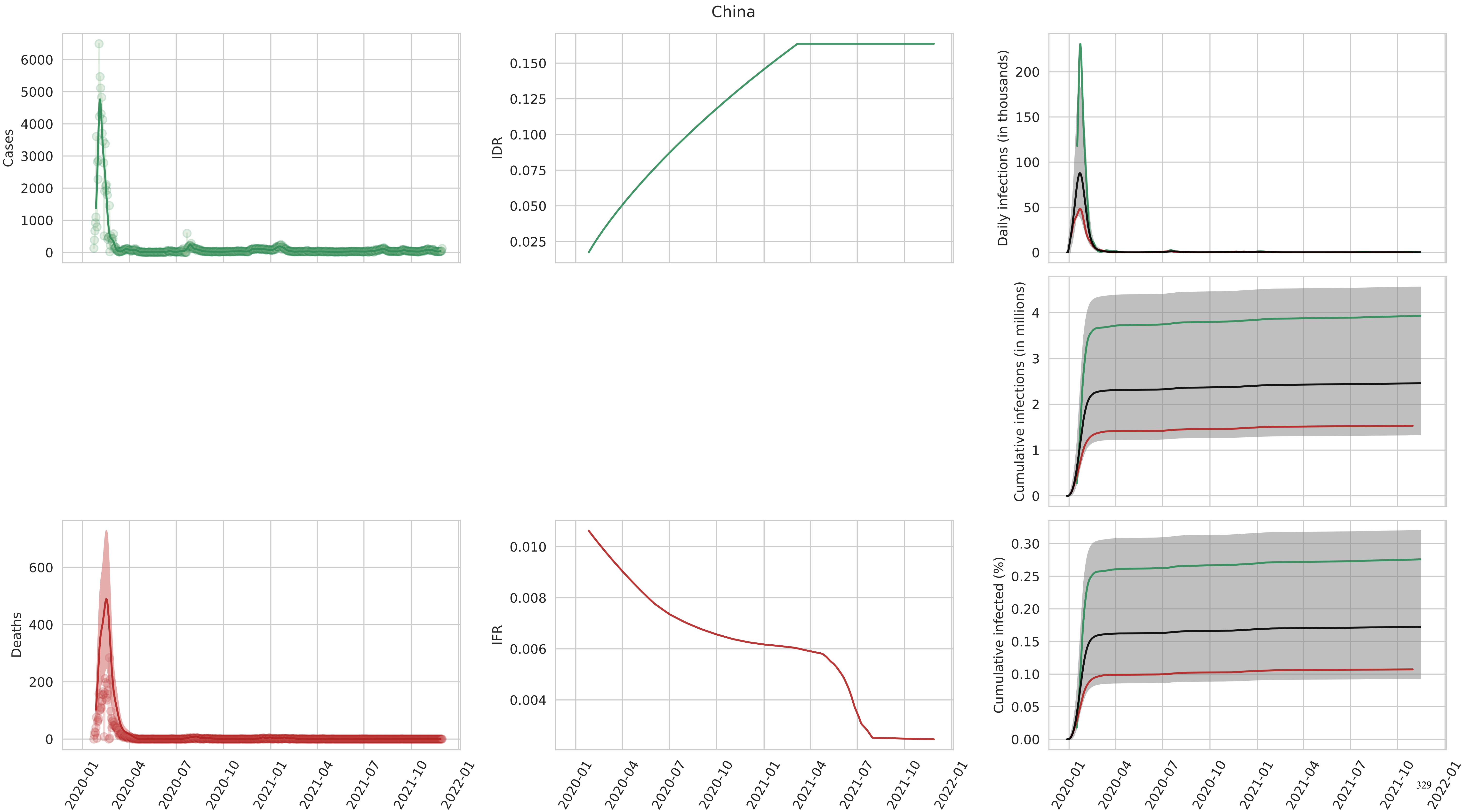
China
Cases
IDR
Daily infections (in thousands)
Cumulative infections (in millions)
Deaths
IFR
Cumulative infected (%)
2020-01
2020-04
2020-07
2020-10
2021-01
2021-04
2021-07
2021-10
2022-01

Taiwan (Province of China)

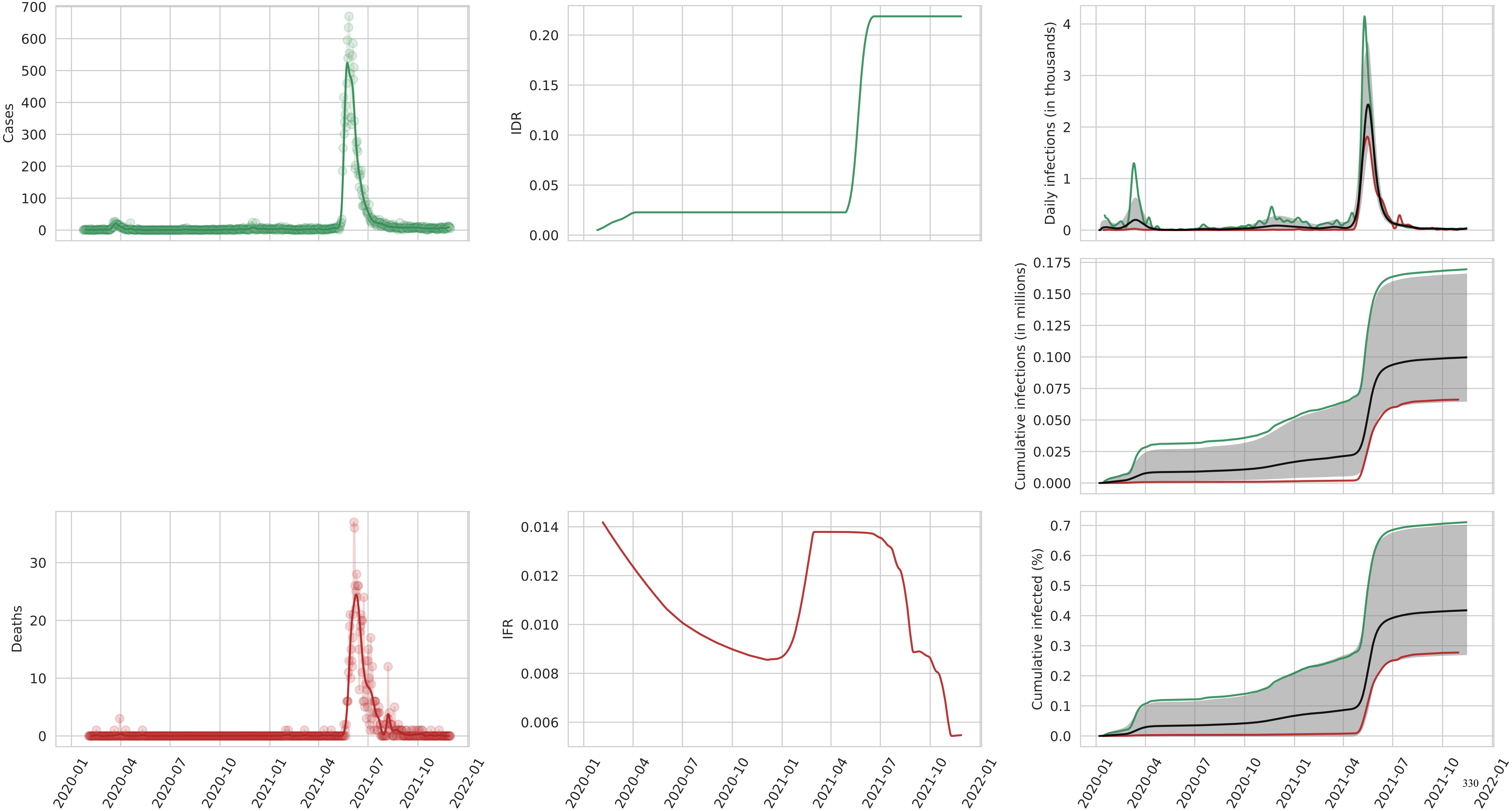

Fiji

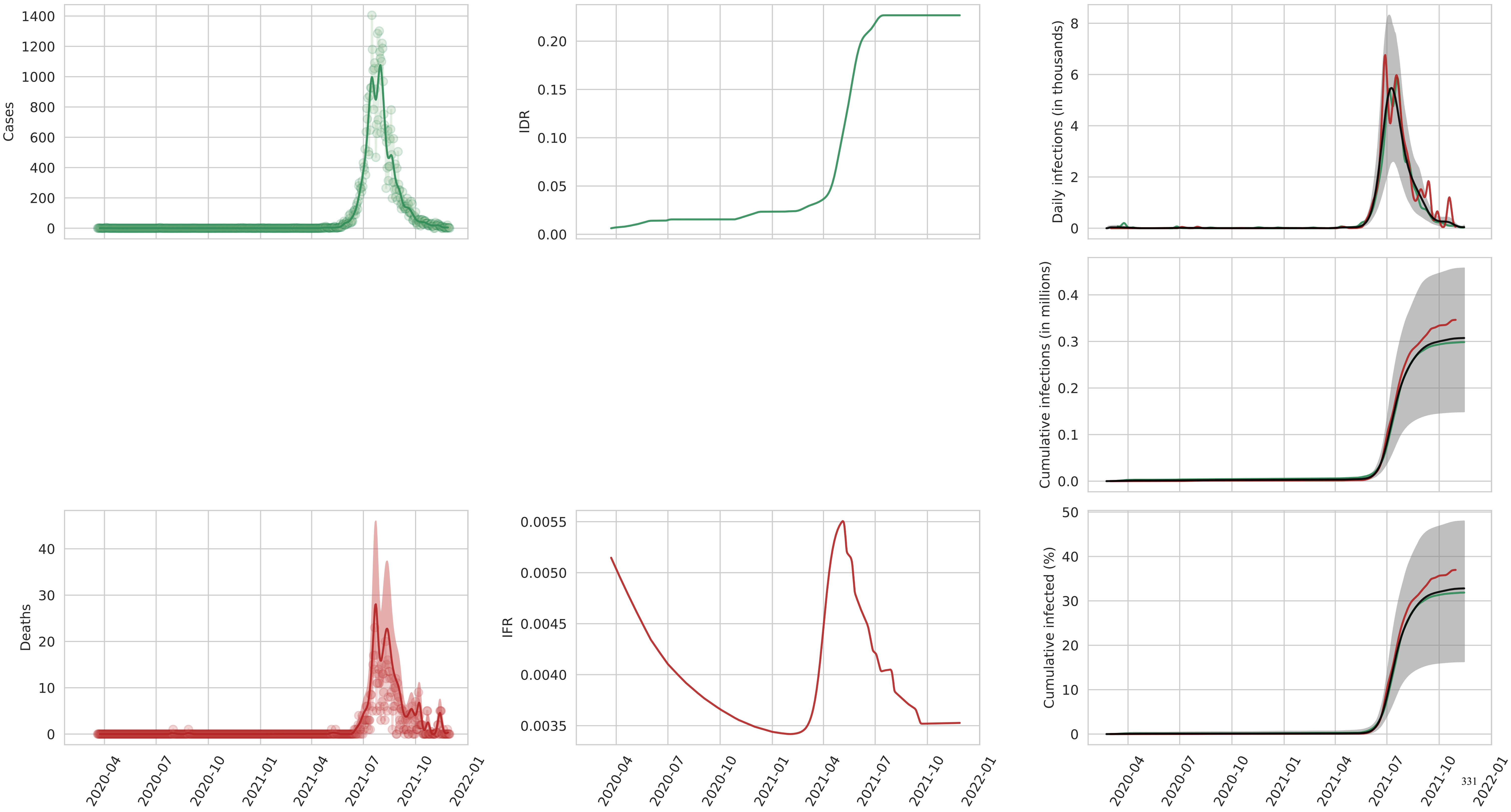

Cases
IDR
Daily infections (in thousands)
Cumulative infections (in millions)
Deaths
IFR
Cumulative infected (%)
2020-04
2020-07
2020-10
2021-01
2021-04
2021-07
2021-10
2022-01

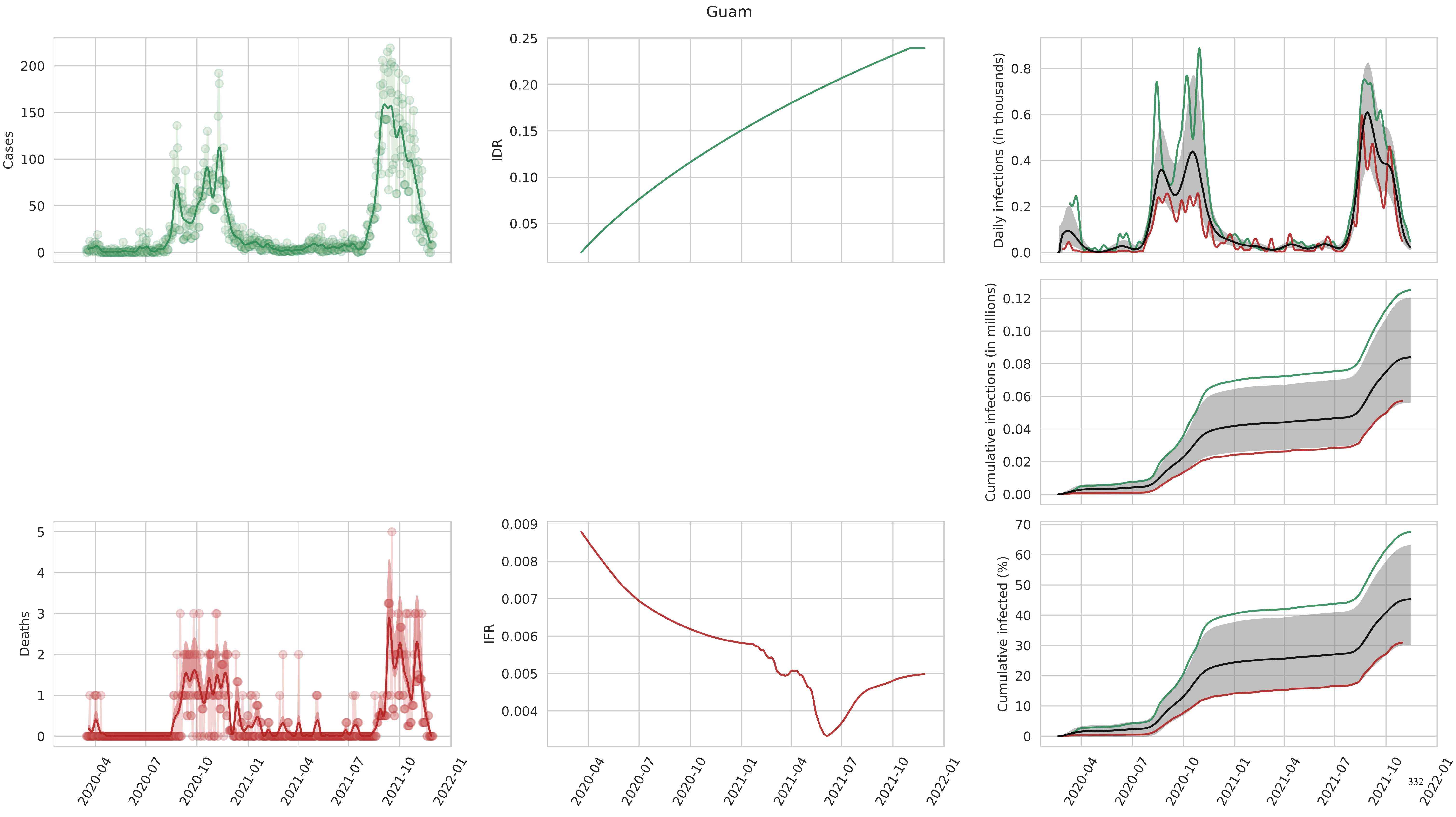
Guam
Cases
IDR
Daily infections (in thousands)
Cumulative infections (in millions)
Deaths
IFR
Cumulative infected (%)
2020-04
2020-07
2020-10
2021-01
2021-04
2021-07
2021-10
2022-01

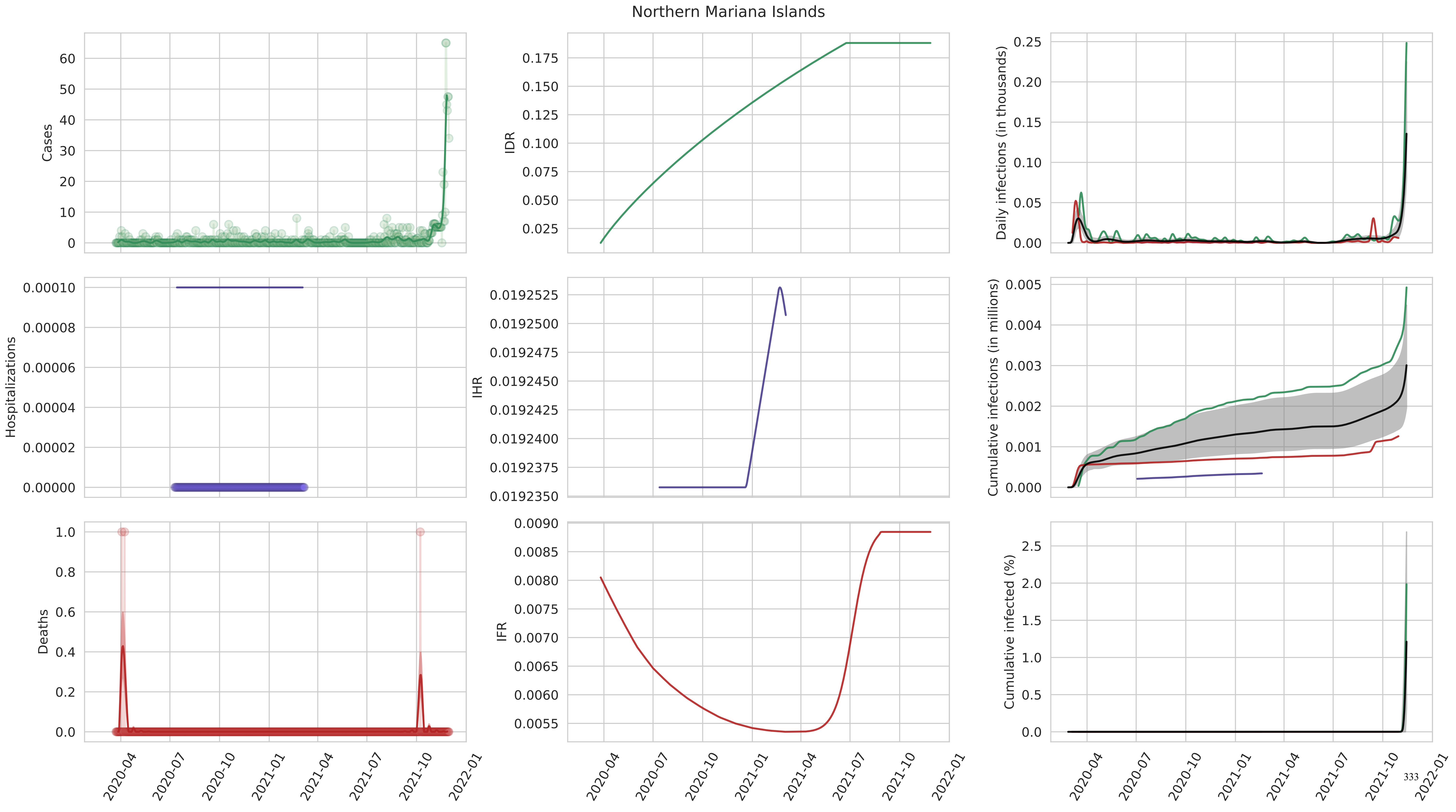
Northern Mariana Islands
Cases
Hospitalizations
Deaths
IDR
IHR
IFR
Daily infections (in thousands)
Cumulative infections (in millions)
Cumulative infected (%)
2020-04
2020-07
2020-10
2021-01
2021-04
2021-07
2021-10
2022-01

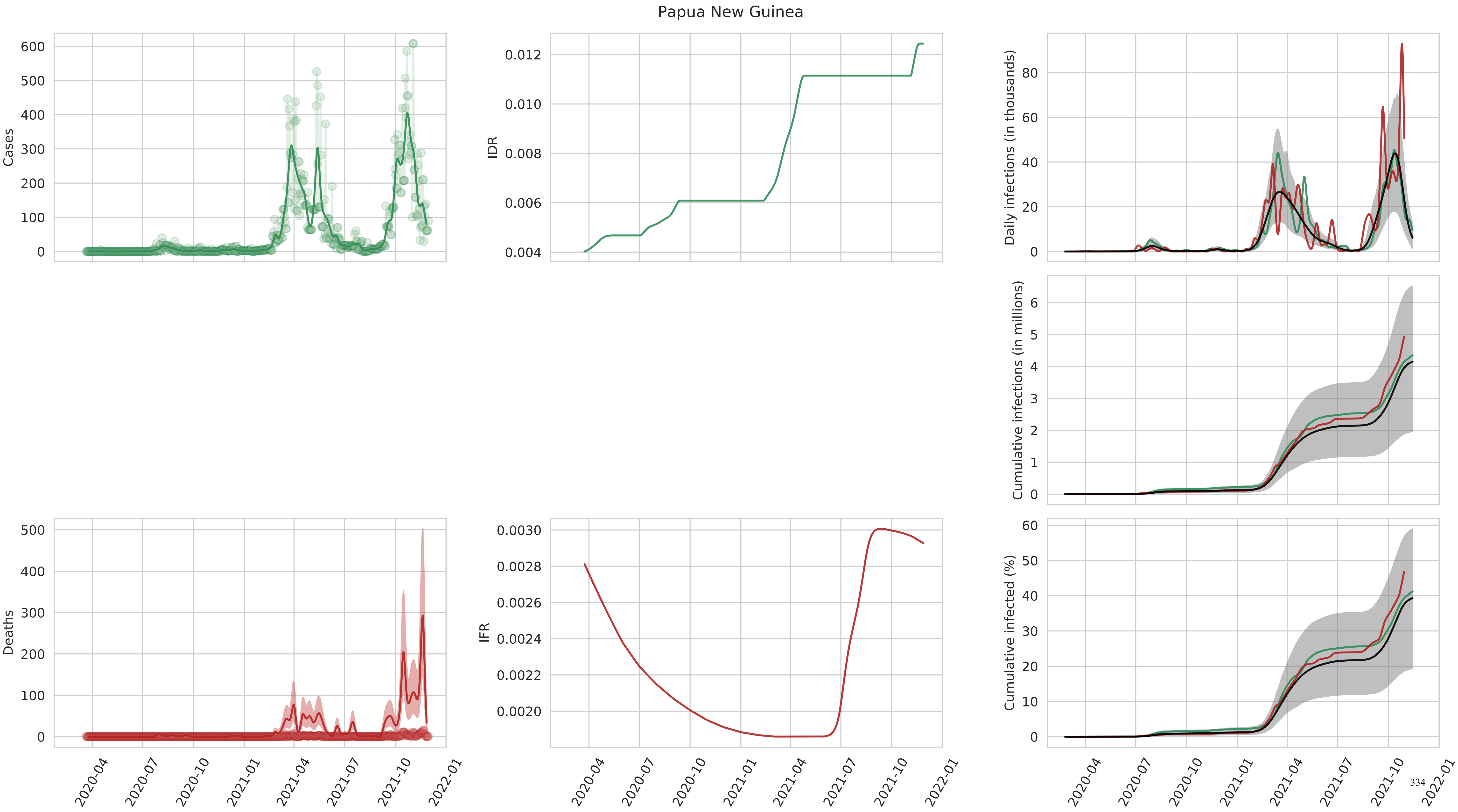

Papua New Guinea
Cases
IDR
Daily infections (in thousands)
Cumulative infections (in millions)
Deaths
IFR
Cumulative infected (%)

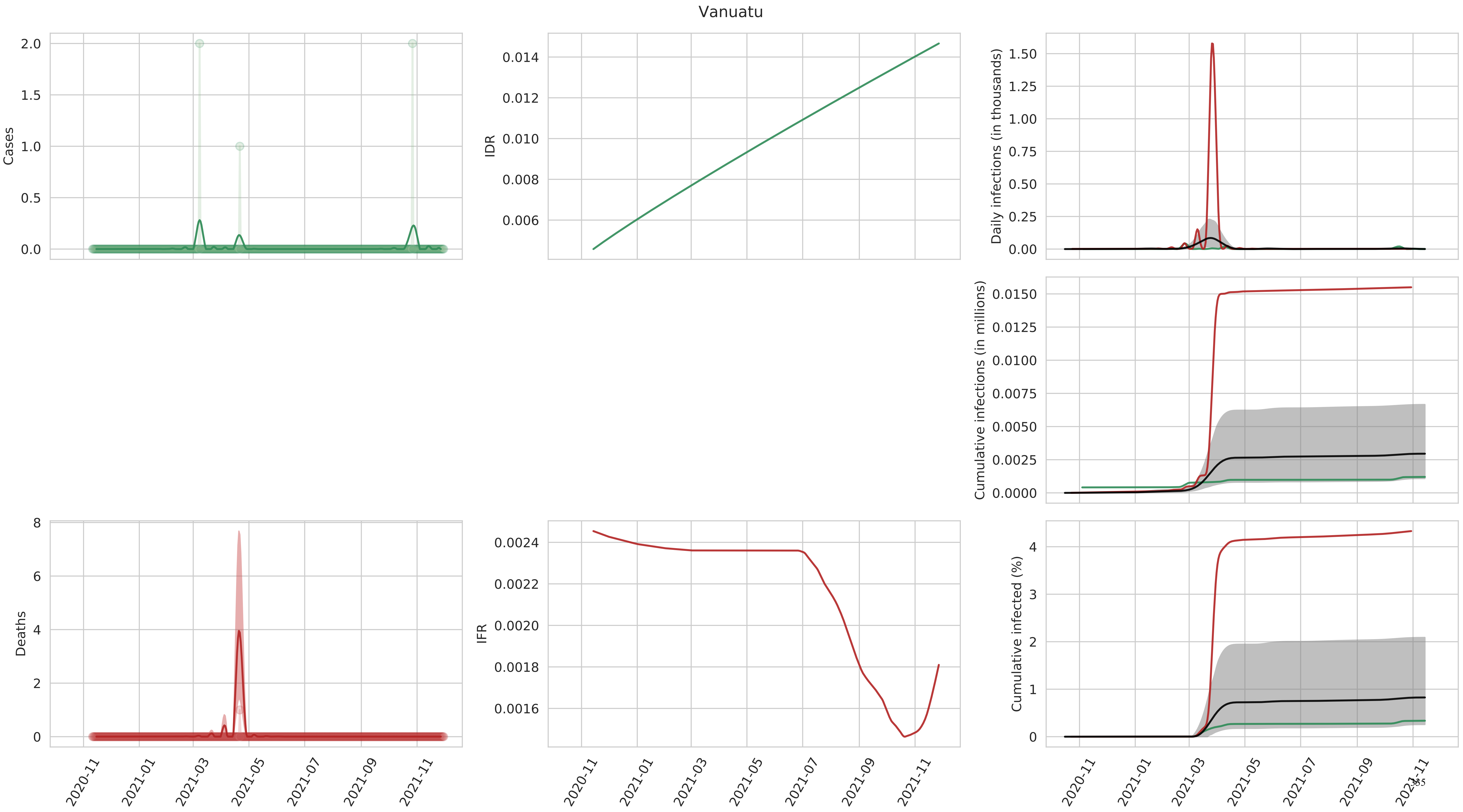

Vanuatu
Cases
IDR
Daily infections (in thousands)
Cumulative infections (in millions)
Deaths
IFR
Cumulative infected (%)
2020-11
2021-01
2021-03
2021-05
2021-07
2021-09
2021-11

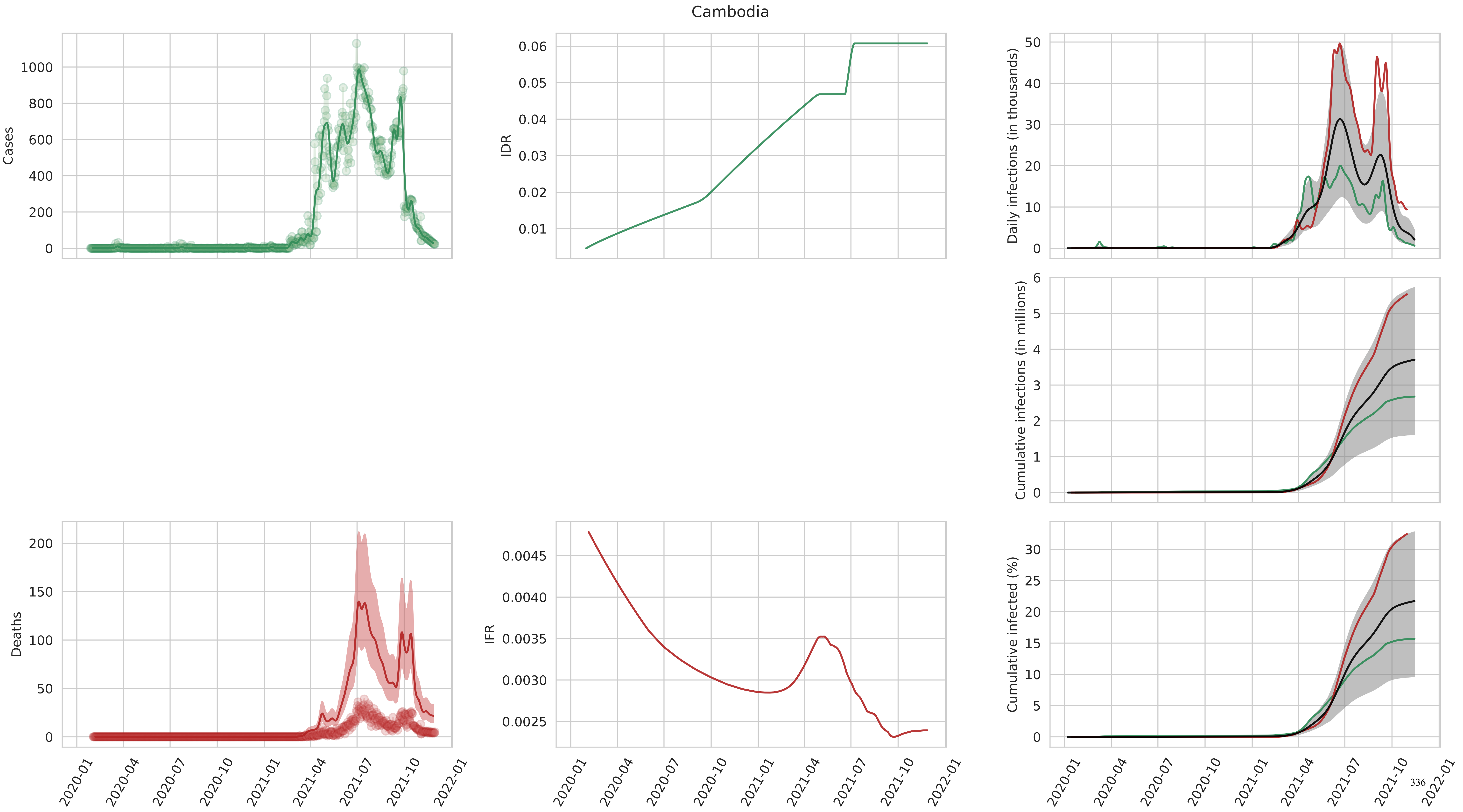
Cambodia
Cases
IDR
Daily infections (in thousands)
Cumulative infections (in millions)
Deaths
IFR
Cumulative infected (%)

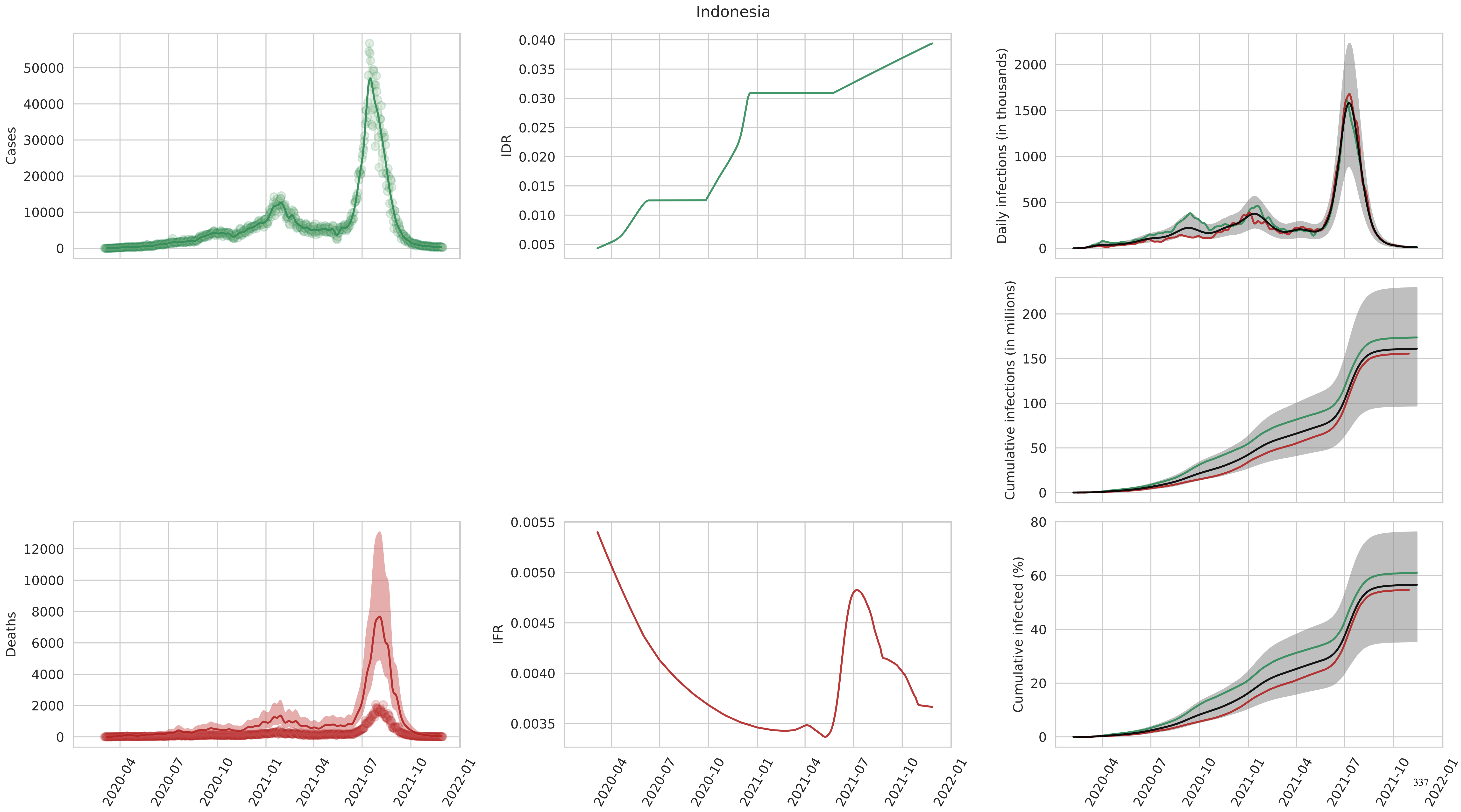

Indonesia
Cases
IDR
Daily infections (in thousands)
Cumulative infections (in millions)
Deaths
IFR
Cumulative infected (%)
2020-04
2020-07
2020-10
2021-01
2021-04
2021-07
2021-10
2022-01

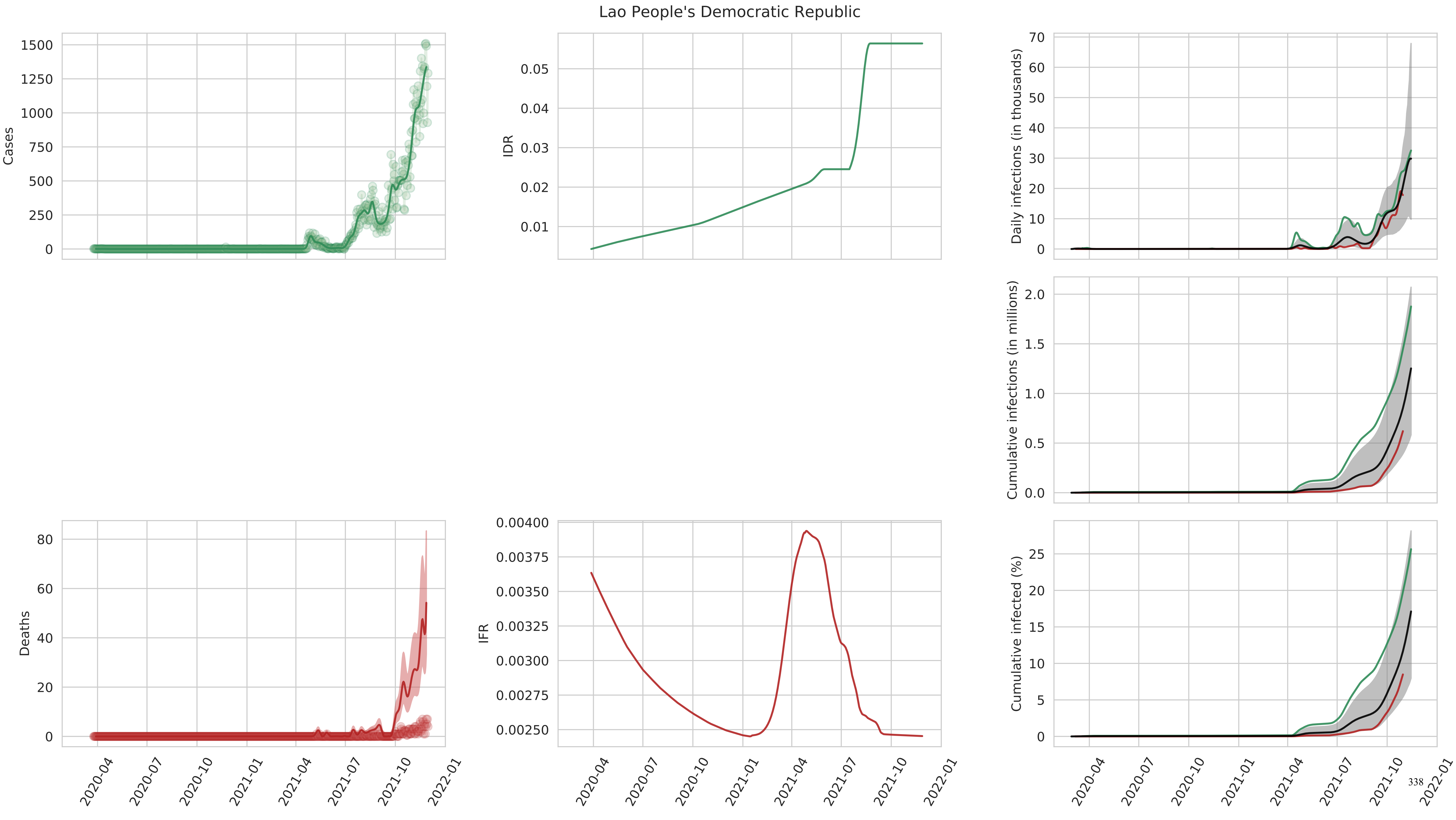
Lao People's Democratic Republic
Cases
IDR
Daily infections (in thousands)
Cumulative infections (in millions)
Deaths
IFR
Cumulative infected (%)
2020-04
2020-07
2020-10
2021-01
2021-04
2021-07
2021-10
2022-01

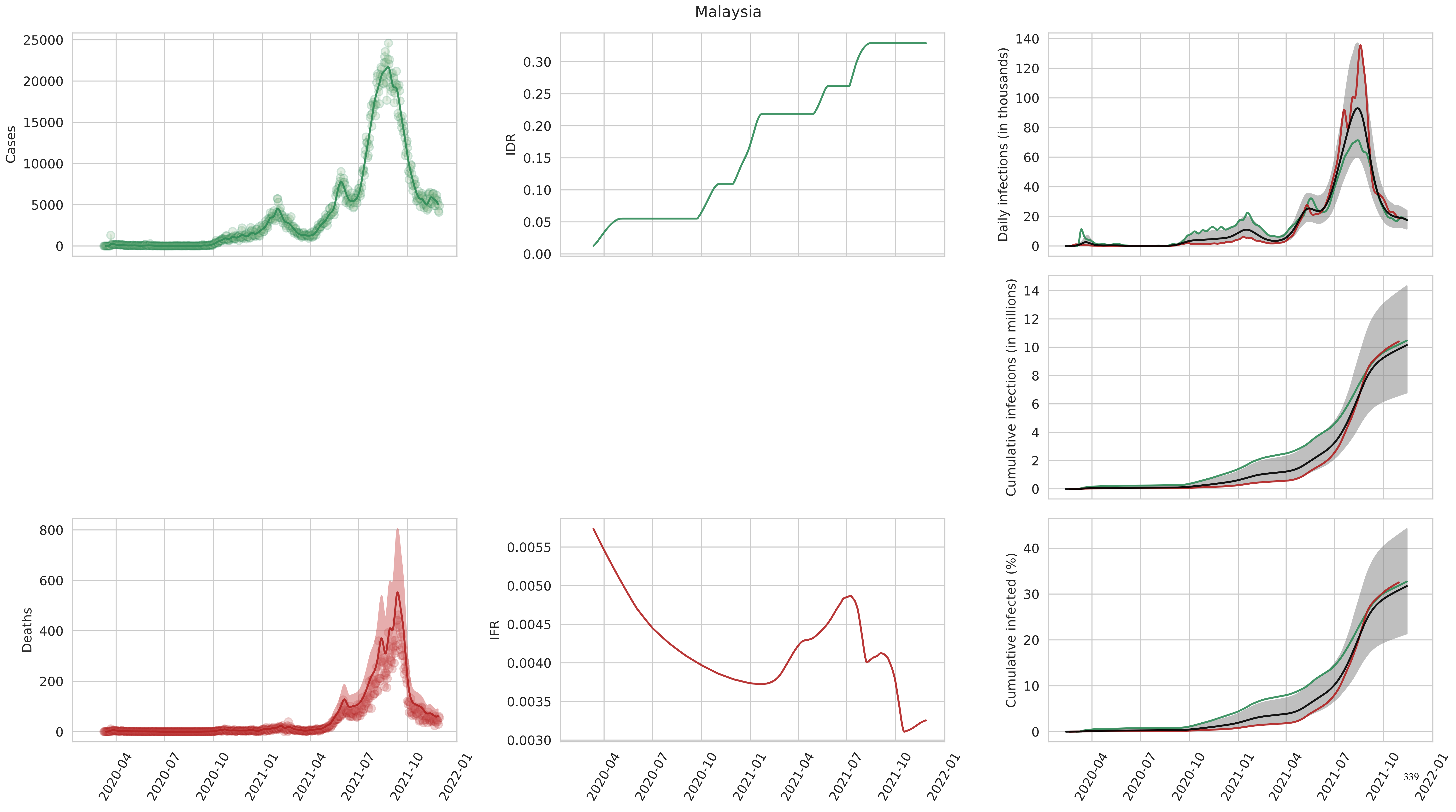

Malaysia
Cases
IDR
Daily infections (in thousands)
Cumulative infections (in millions)
Deaths
IFR
Cumulative infected (%)
2020-04
2020-07
2020-10
2021-01
2021-04
2021-07
2021-10
2022-01

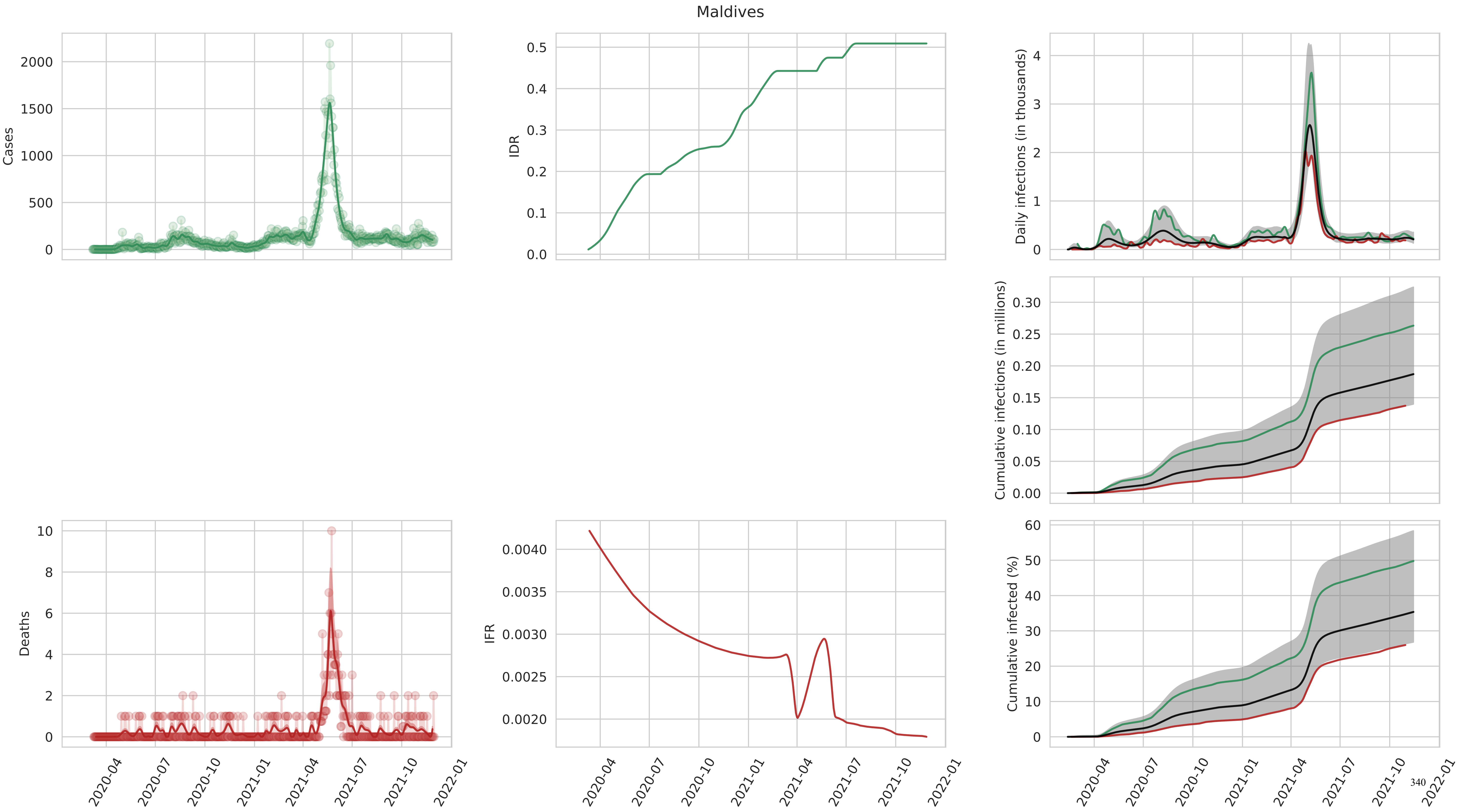

Maldives
Cases
Deaths
IDR
IFR
Daily infections (in thousands)
Cumulative infections (in millions)
Cumulative infected (%)
2020-04
2020-07
2020-10
2021-01
2021-04
2021-07
2021-10
2022-01

Mauritius

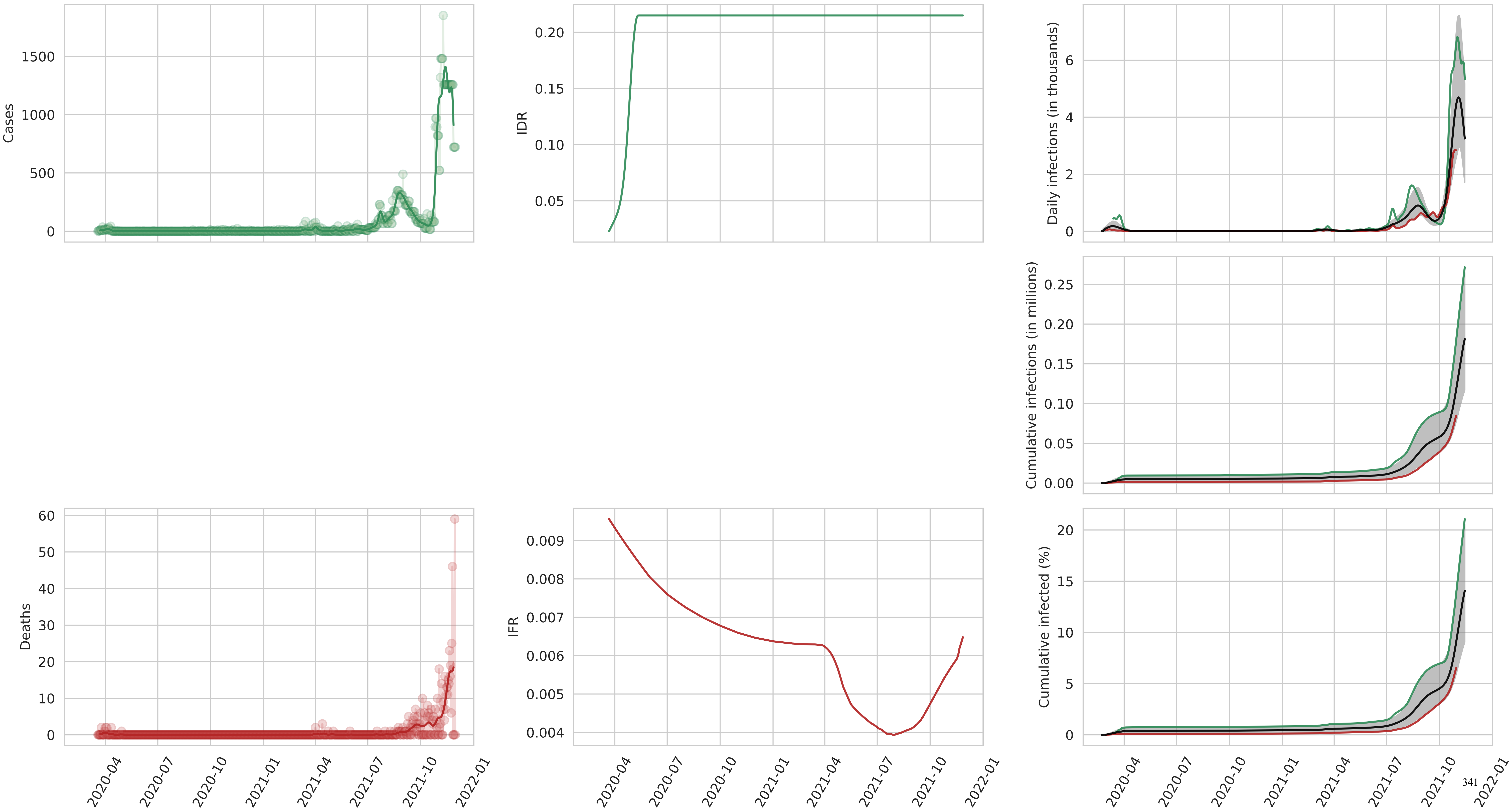
Cases
IDR
Daily infections (in thousands)
Cumulative infections (in millions)
Deaths
IFR
Cumulative infected (%)
2020-04
2020-07
2020-10
2021-01
2021-04
2021-07
2021-10
2022-01

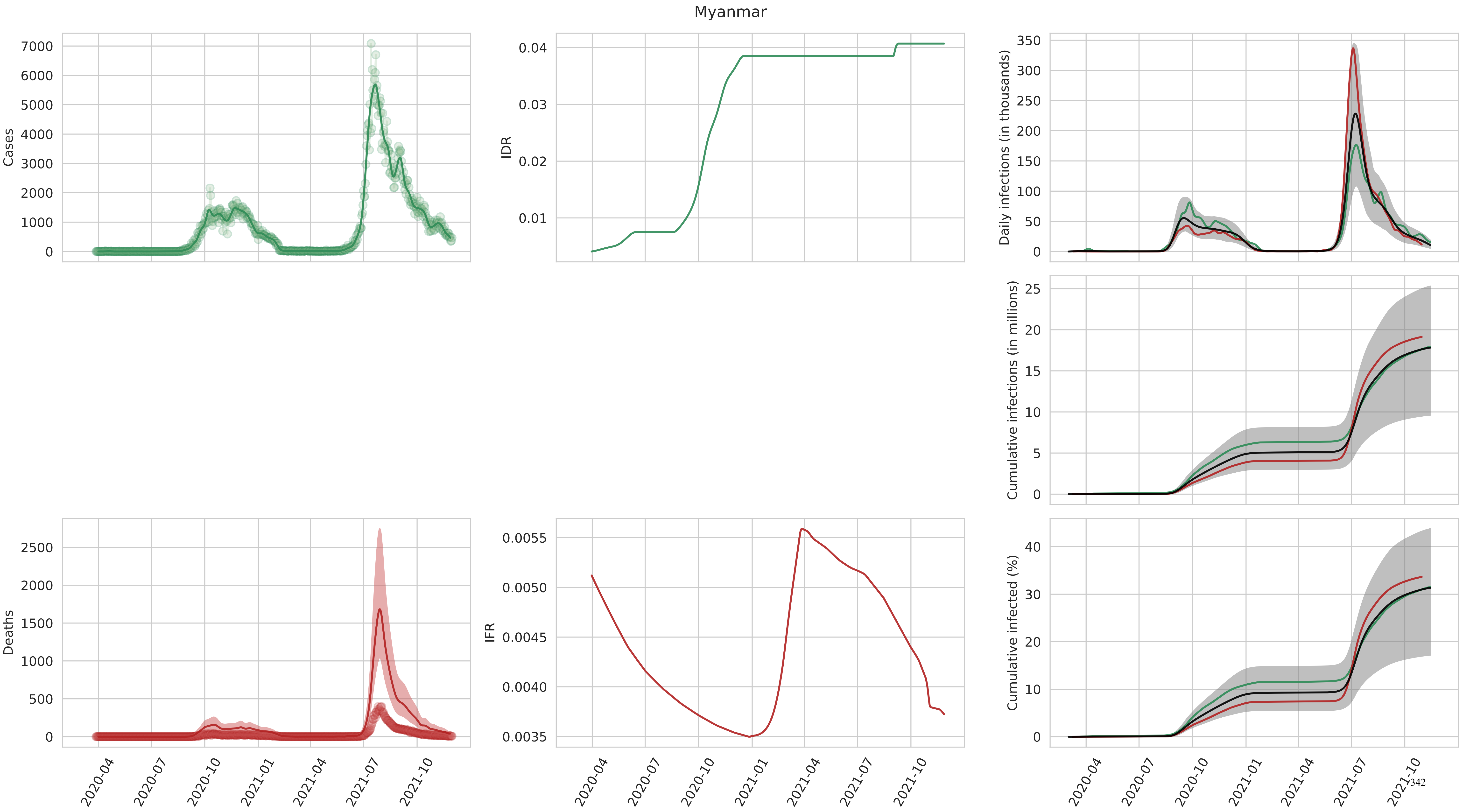

Myanmar
Cases
IDR
Daily infections (in thousands)
Cumulative infections (in millions)
Deaths
IFR
Cumulative infected (%)
2020-04
2020-07
2020-10
2021-01
2021-04
2021-07
2021-10

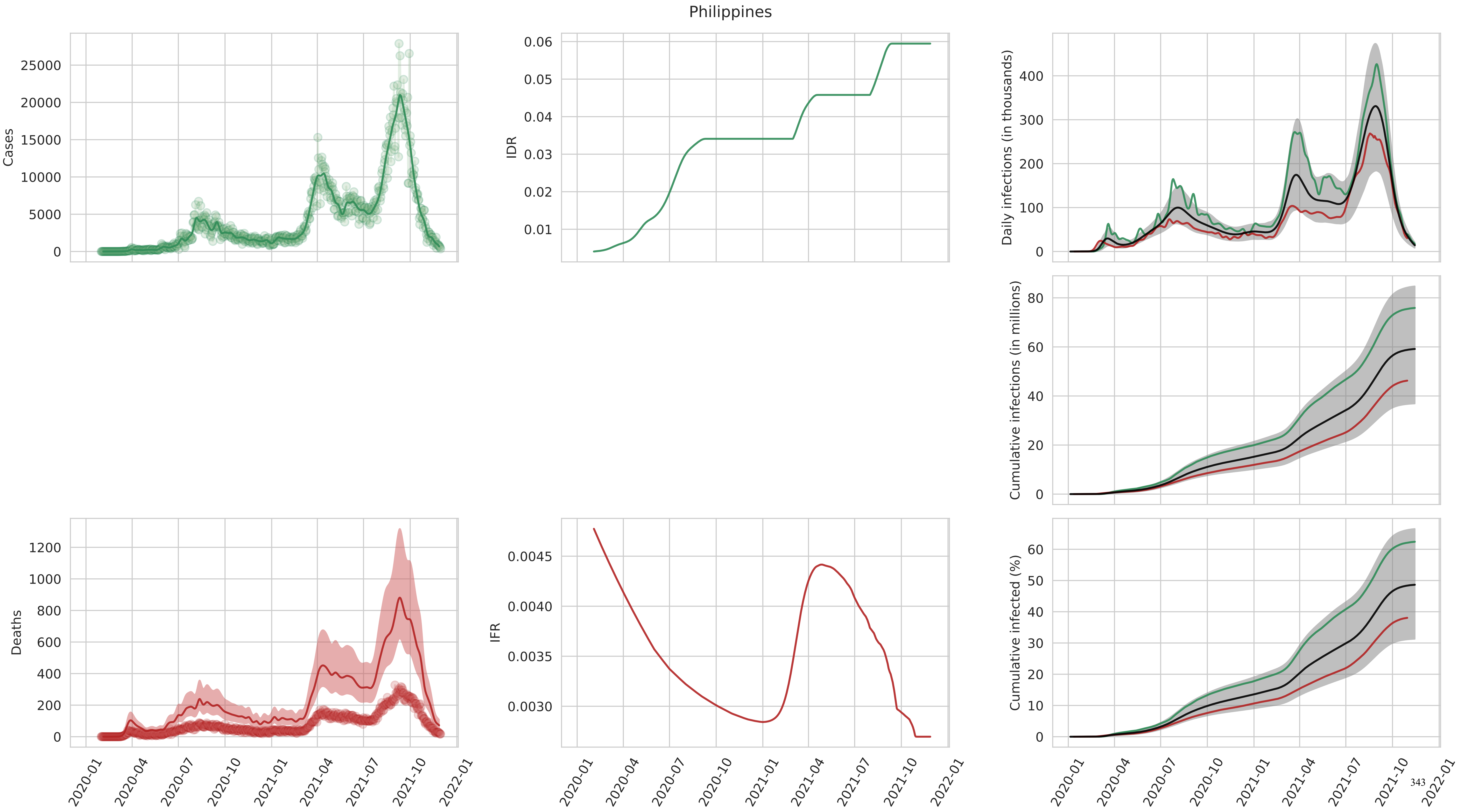

Philippines
Cases
IDR
Daily infections (in thousands)
Cumulative infections (in millions)
Deaths
IFR
Cumulative infected (%)
2020-01
2020-04
2020-07
2020-10
2021-01
2021-04
2021-07
2021-10
2022-01

Seychelles

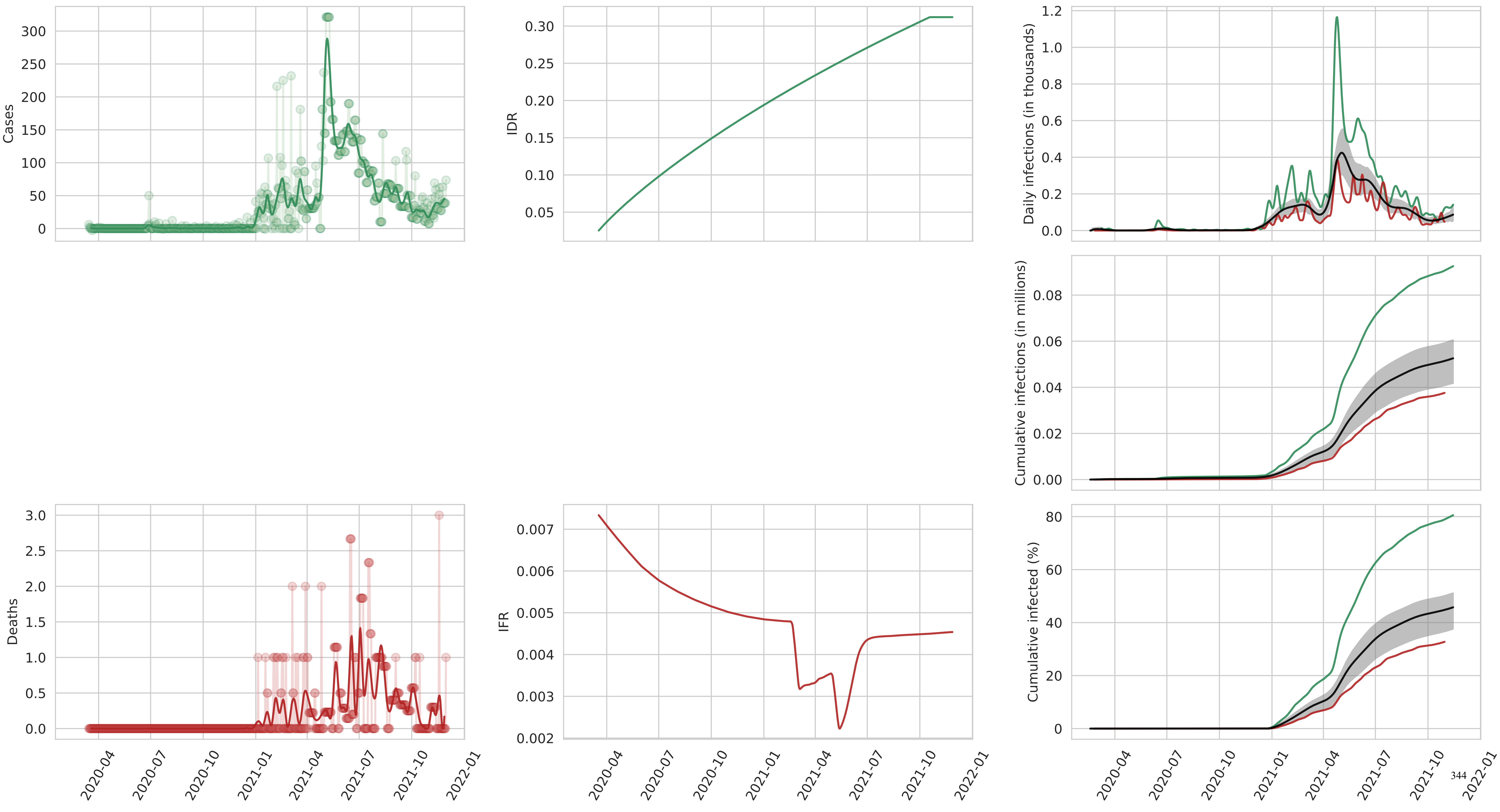

Cases
IDR
Daily infections (in thousands)
Cumulative infections (in millions)
Deaths
IFR
Cumulative infected (%)
2020-04
2020-07
2020-10
2021-01
2021-04
2021-07
2021-10
2022-01

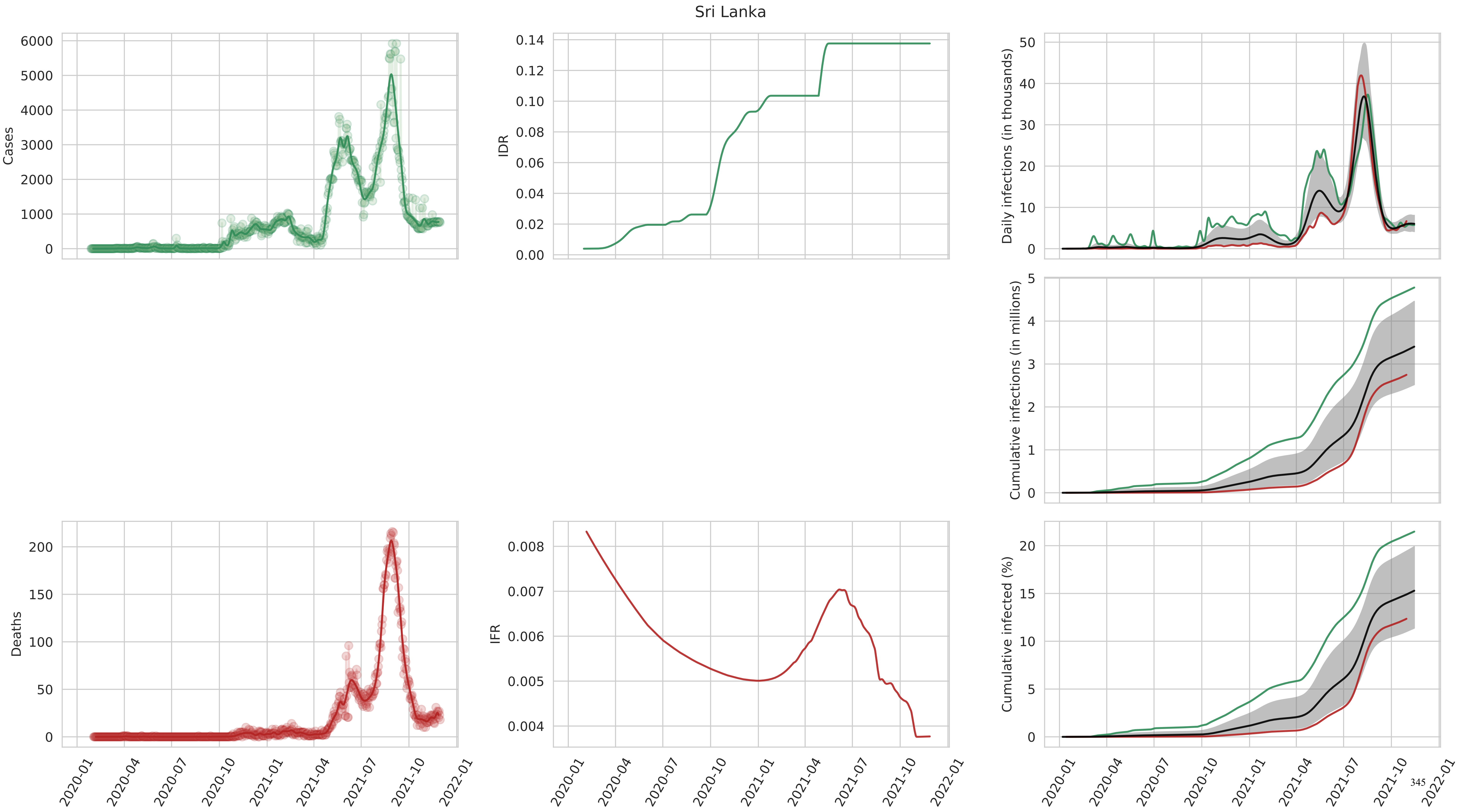

Sri Lanka
Cases
IDR
Daily infections (in thousands)
Cumulative infections (in millions)
Deaths
IFR
Cumulative infected (%)

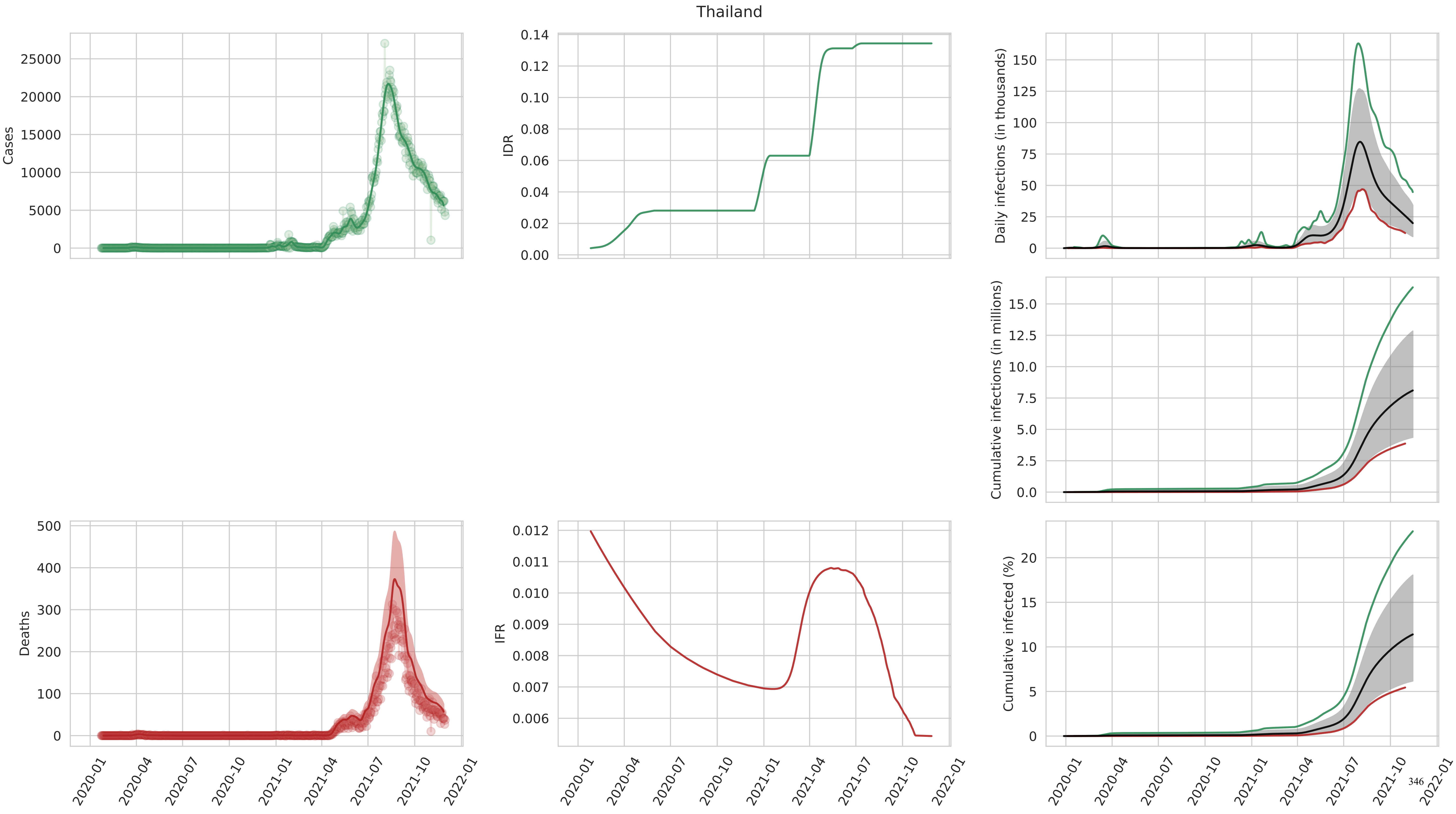
Thailand
Cases
IDR
Daily infections (in thousands)
Cumulative infections (in millions)
Deaths
IFR
Cumulative infected (%)
2020-01
2020-04
2020-07
2020-10
2021-01
2021-04
2021-07
2021-10
2022-01

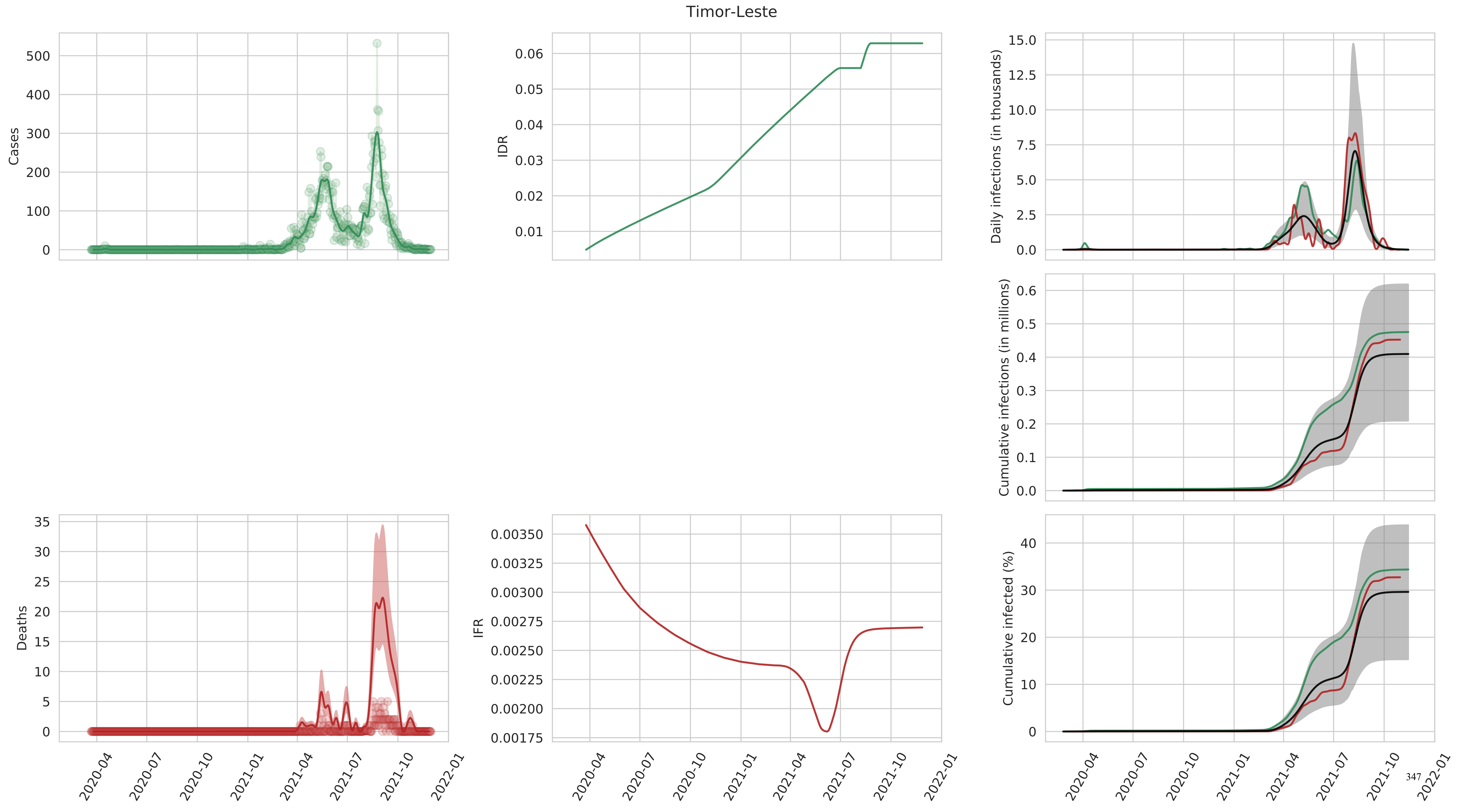
Timor-Leste
Cases
IDR
Daily infections (in thousands)
Cumulative infections (in millions)
Deaths
IFR
Cumulative infected (%)
2020-04
2020-07
2020-10
2021-01
2021-04
2021-07
2021-10
2022-01

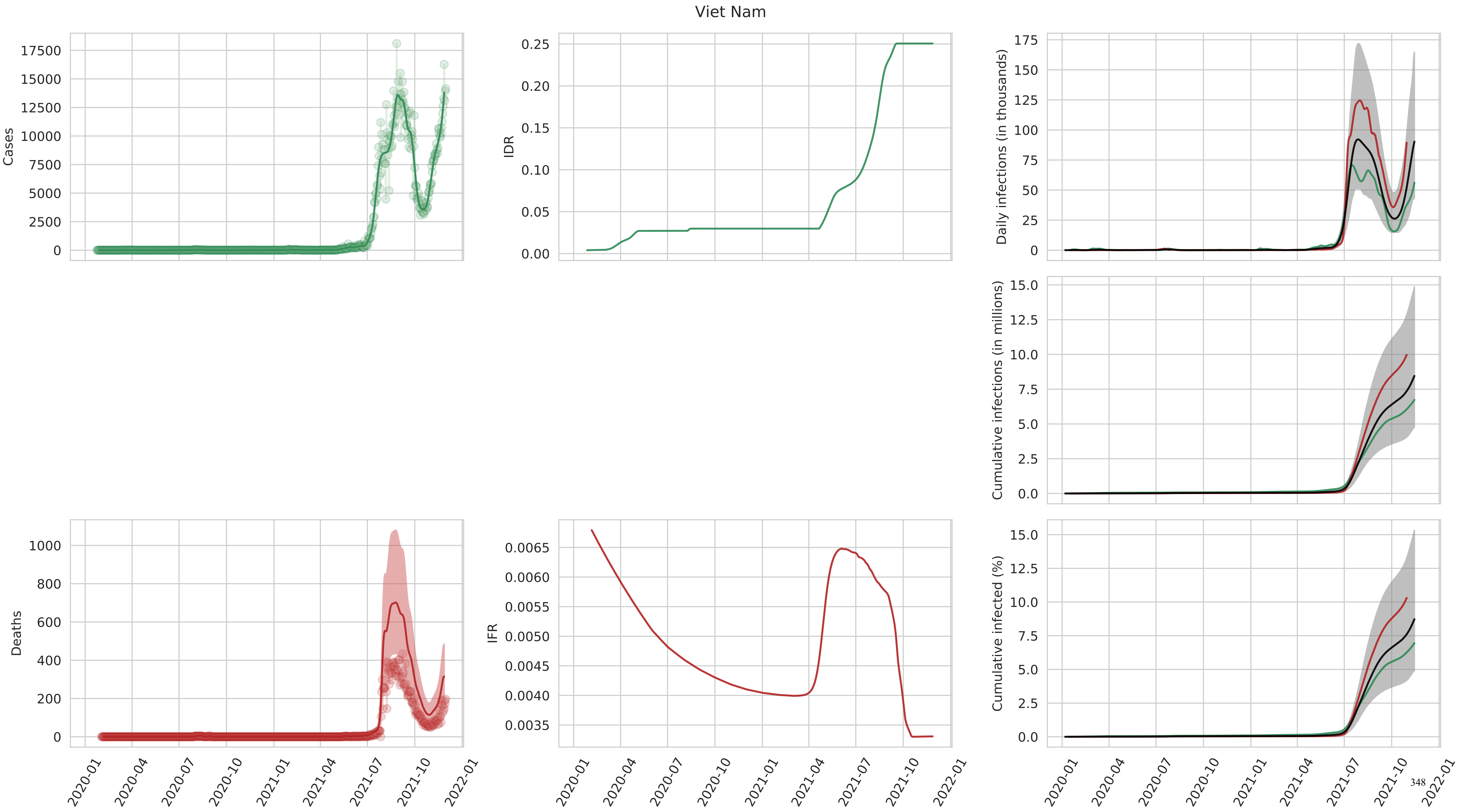
Viet Nam
Cases
IDR
Daily infections (in thousands)
Cumulative infections (in millions)
Deaths
IFR
Cumulative infected (%)
2020-01
2020-04
2020-07
2020-10
2021-01
2021-04
2021-07
2021-10
2022-01

Angola

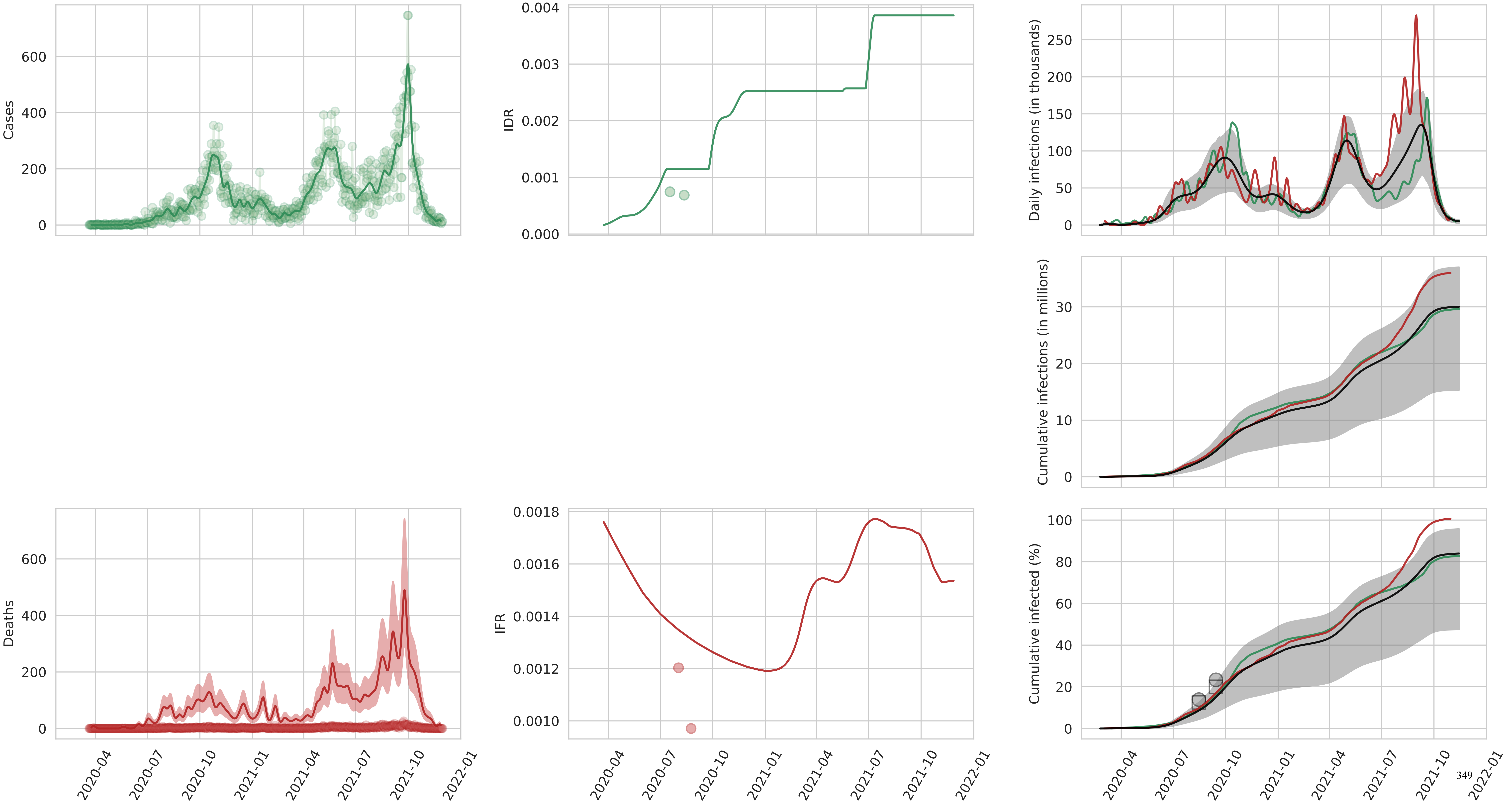

Cases
IDR
Daily infections (in thousands)
Cumulative infections (in millions)
Deaths
IFR
Cumulative infected (%)
2020-04
2020-07
2020-10
2021-01
2021-04
2021-07
2021-10
2022-01

Central African Republic

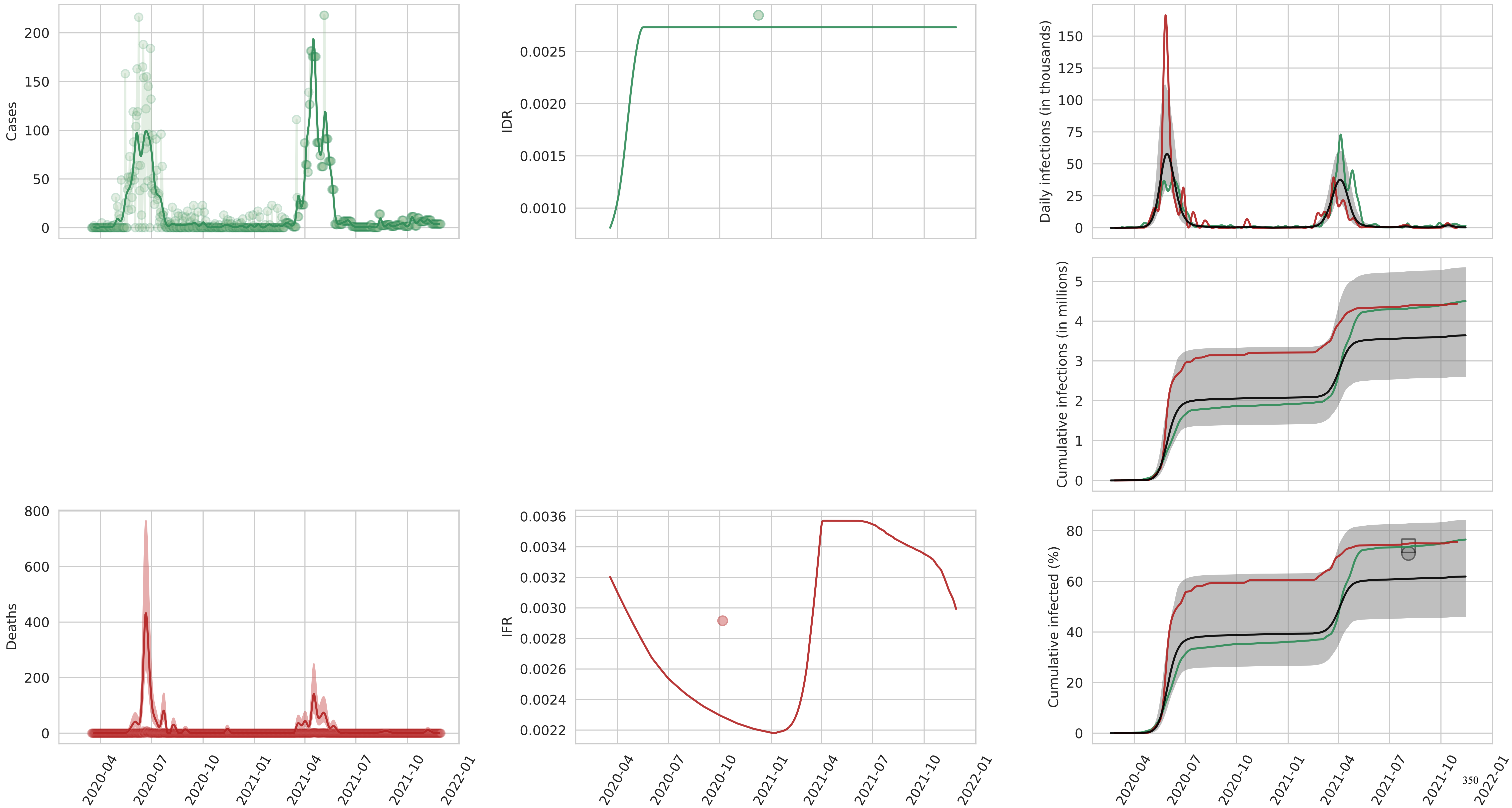

Congo

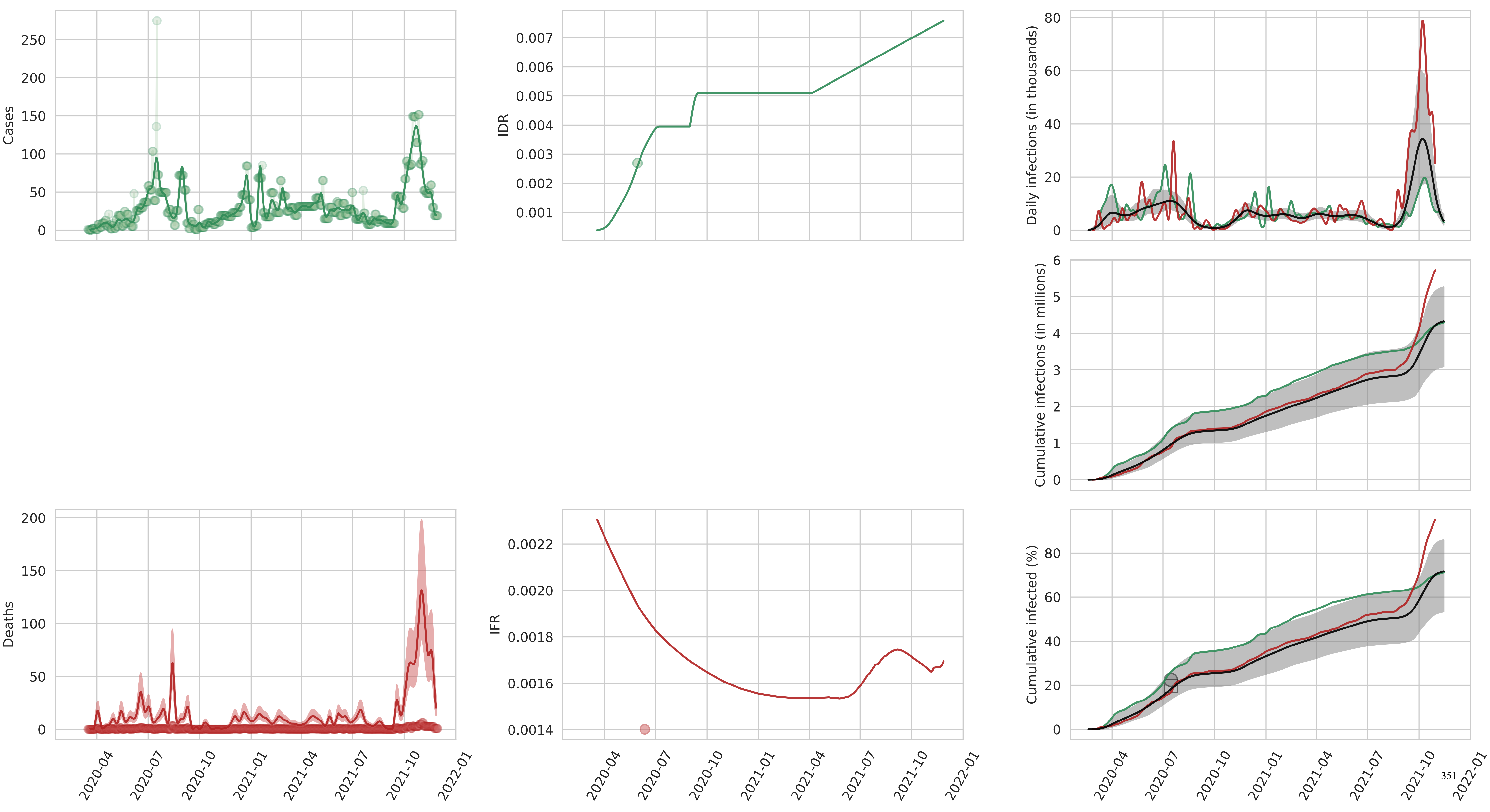

Cases
IDR
Daily infections (in thousands)
Cumulative infections (in millions)
Deaths
IFR
Cumulative infected (%)
2020-04
2020-07
2020-10
2021-01
2021-04
2021-07
2021-10
2022-01

Democratic Republic of the Congo

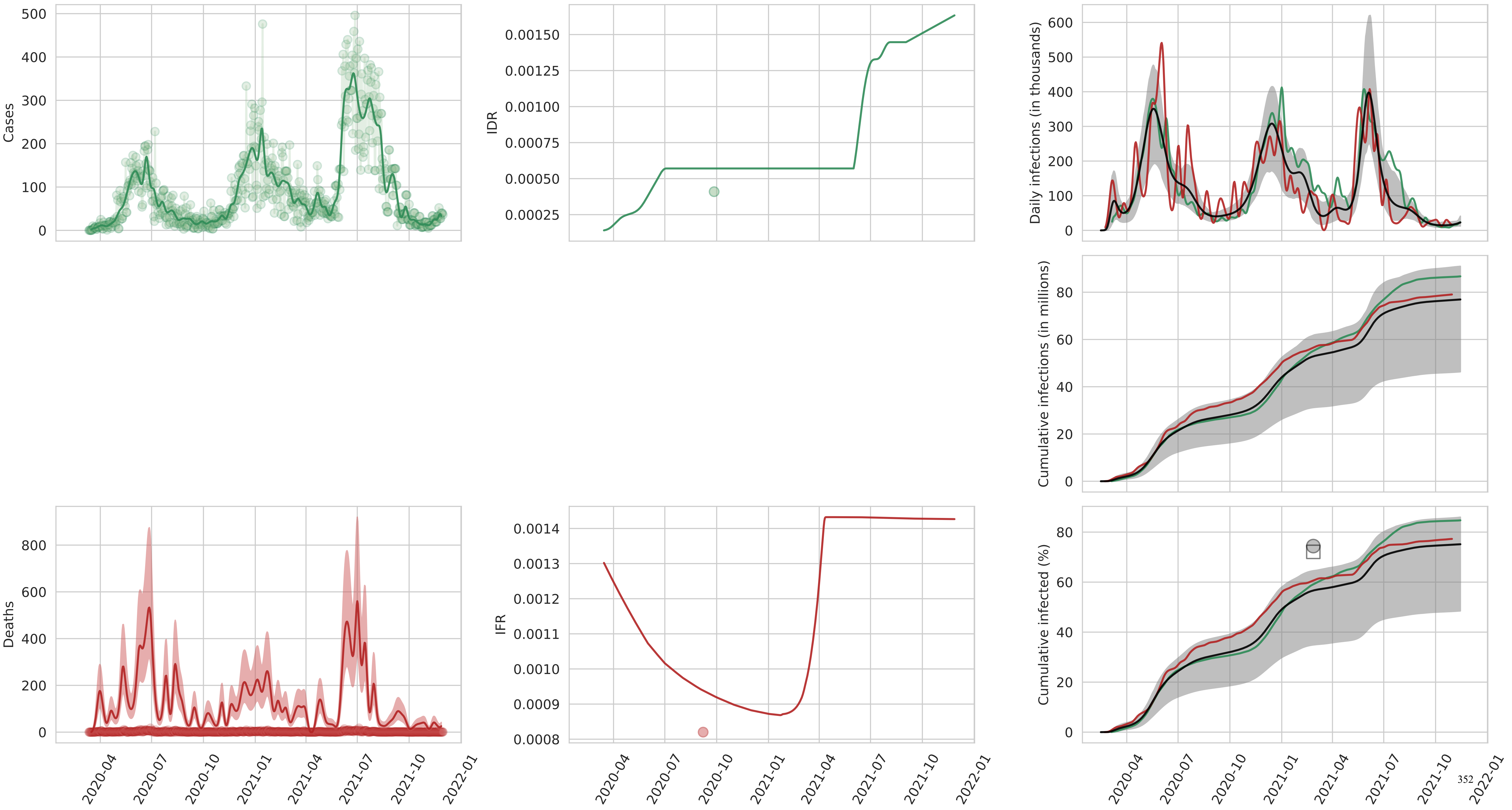

Equatorial Guinea

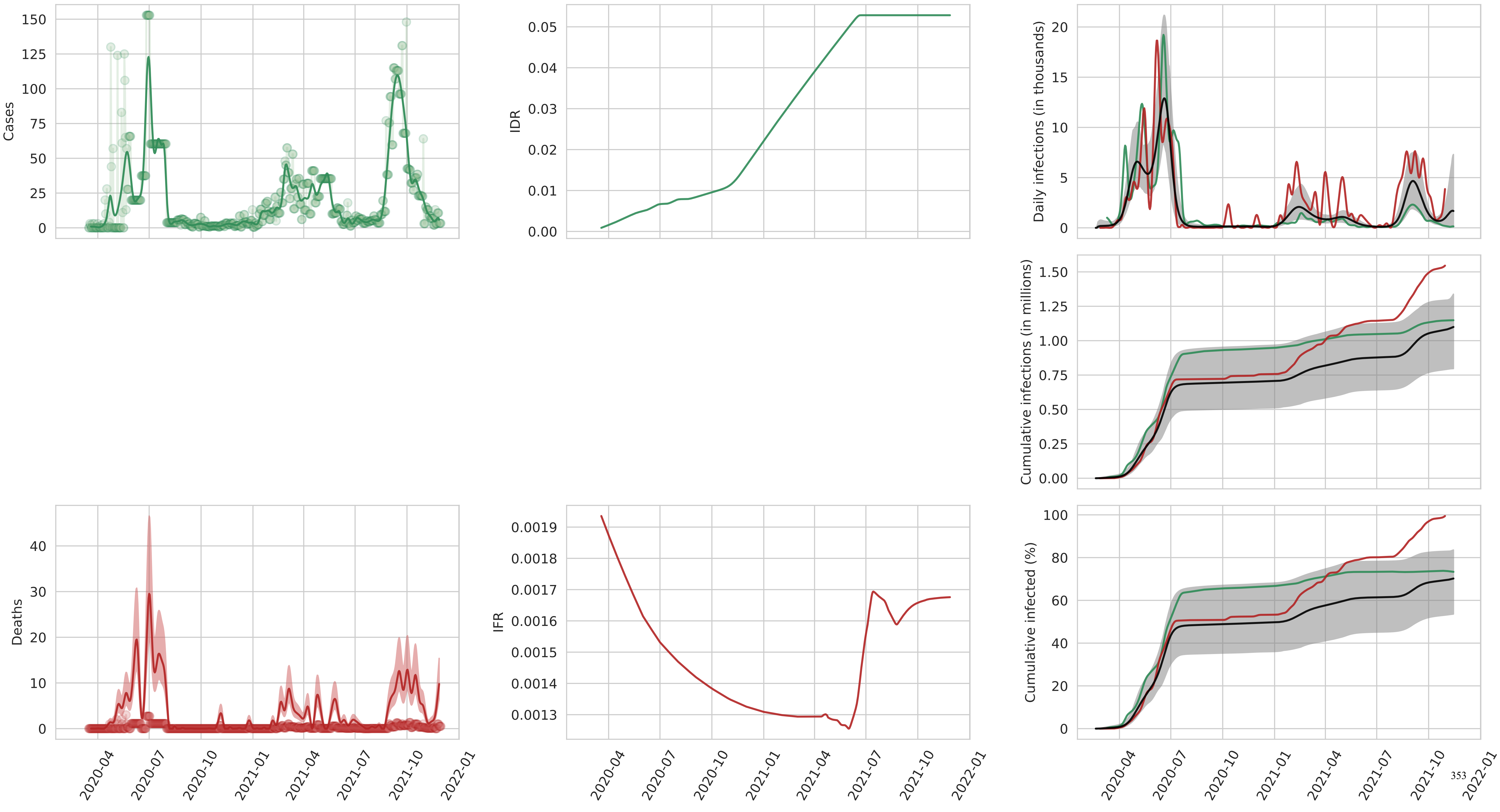

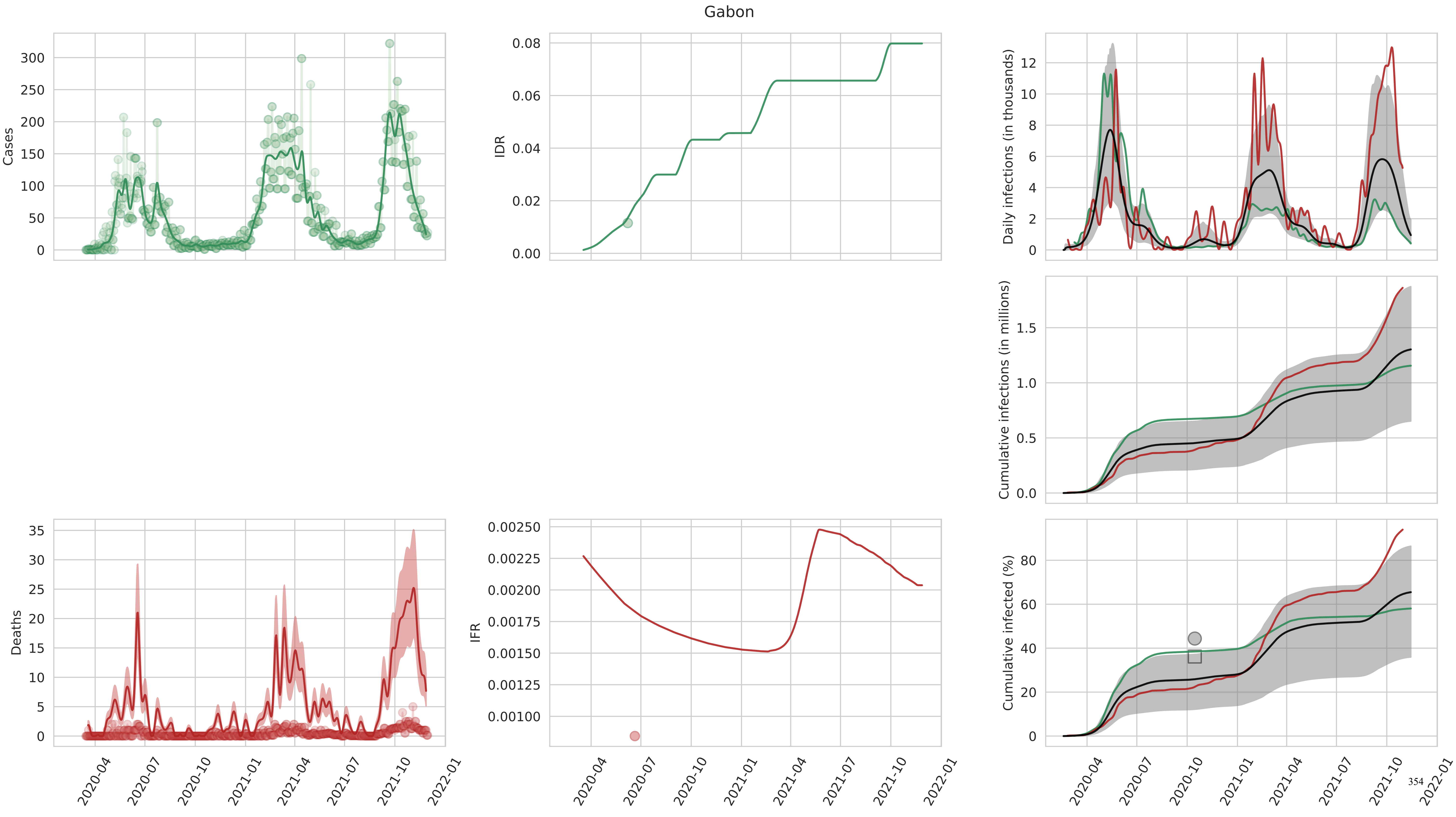
Gabon
Cases
IDR
Daily infections (in thousands)
Cumulative infections (in millions)
Deaths
IFR
Cumulative infected (%)
2020-04
2020-07
2020-10
2021-01
2021-04
2021-07
2021-10
2022-01

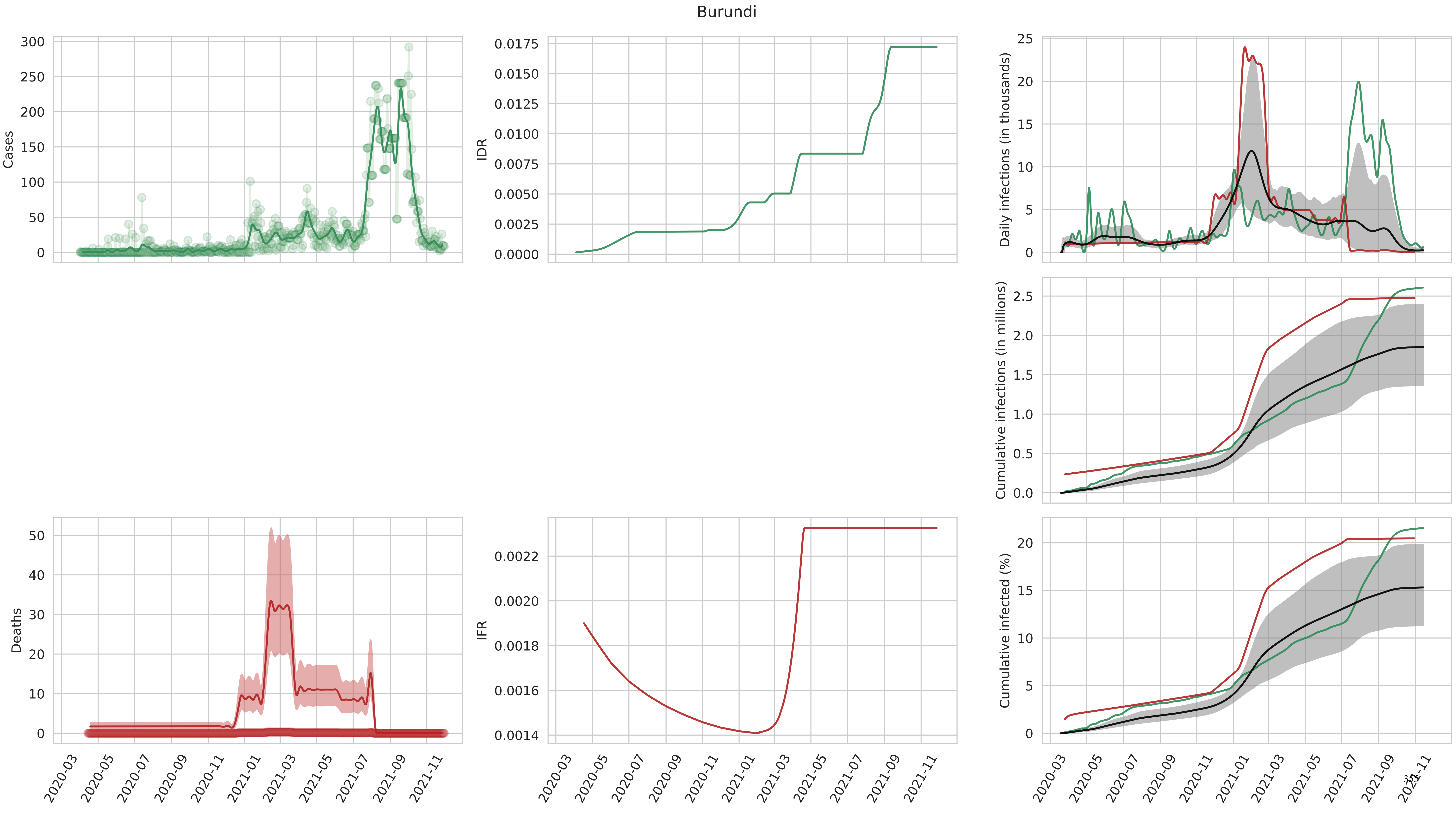

Burundi
Cases
IDR
Daily infections (in thousands)
Cumulative infections (in millions)
Deaths
IFR
Cumulative infected (%)
2020-03
2020-05
2020-07
2020-09
2020-11
2021-01
2021-03
2021-05
2021-07
2021-09
2021-11

Comoros

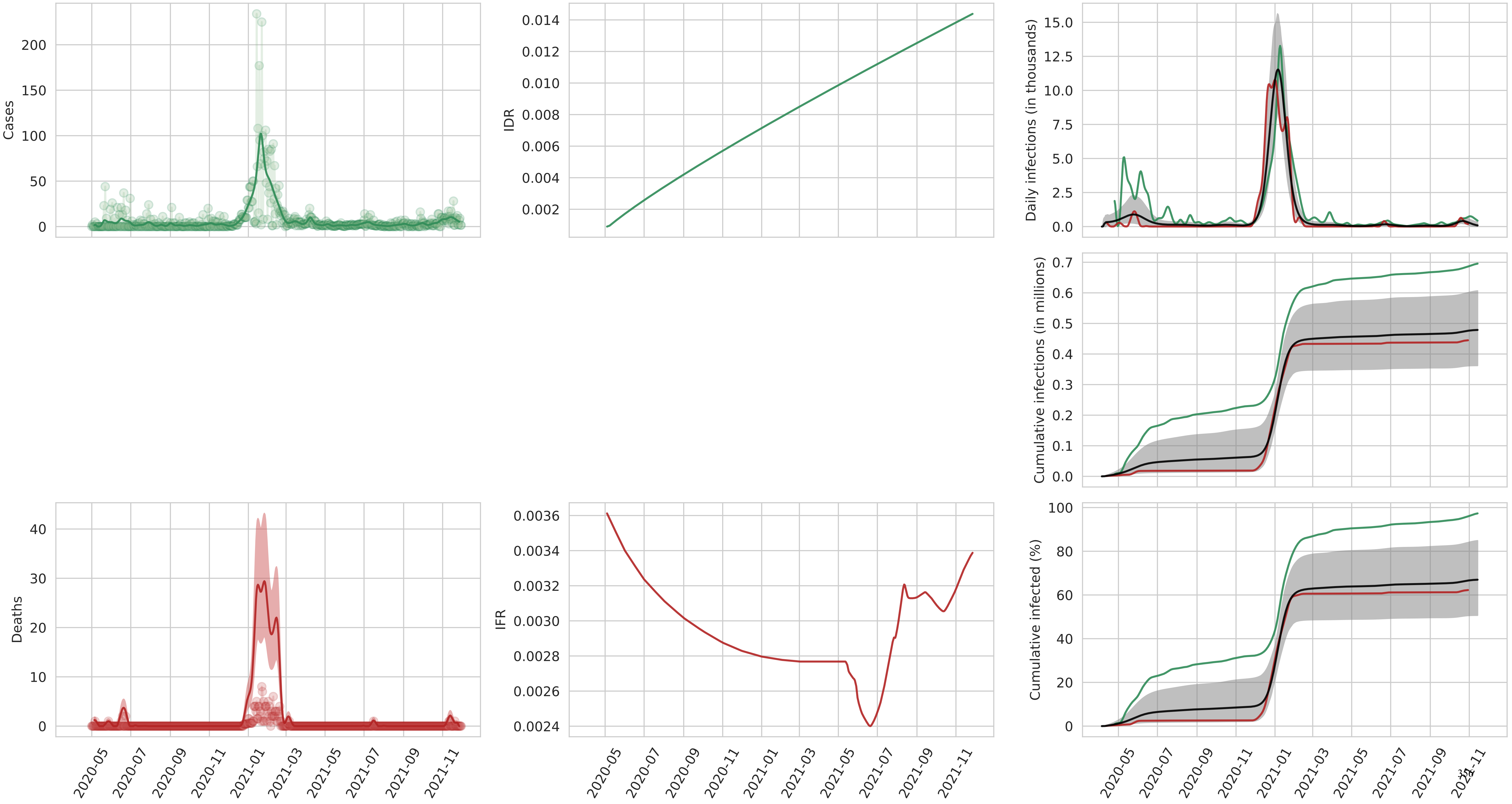

Cases
IDR
Daily infections (in thousands)
Cumulative infections (in millions)
Deaths
IFR
Cumulative infected (%)
2020-05
2020-07
2020-09
2020-11
2021-01
2021-03
2021-05
2021-07
2021-09
2021-11

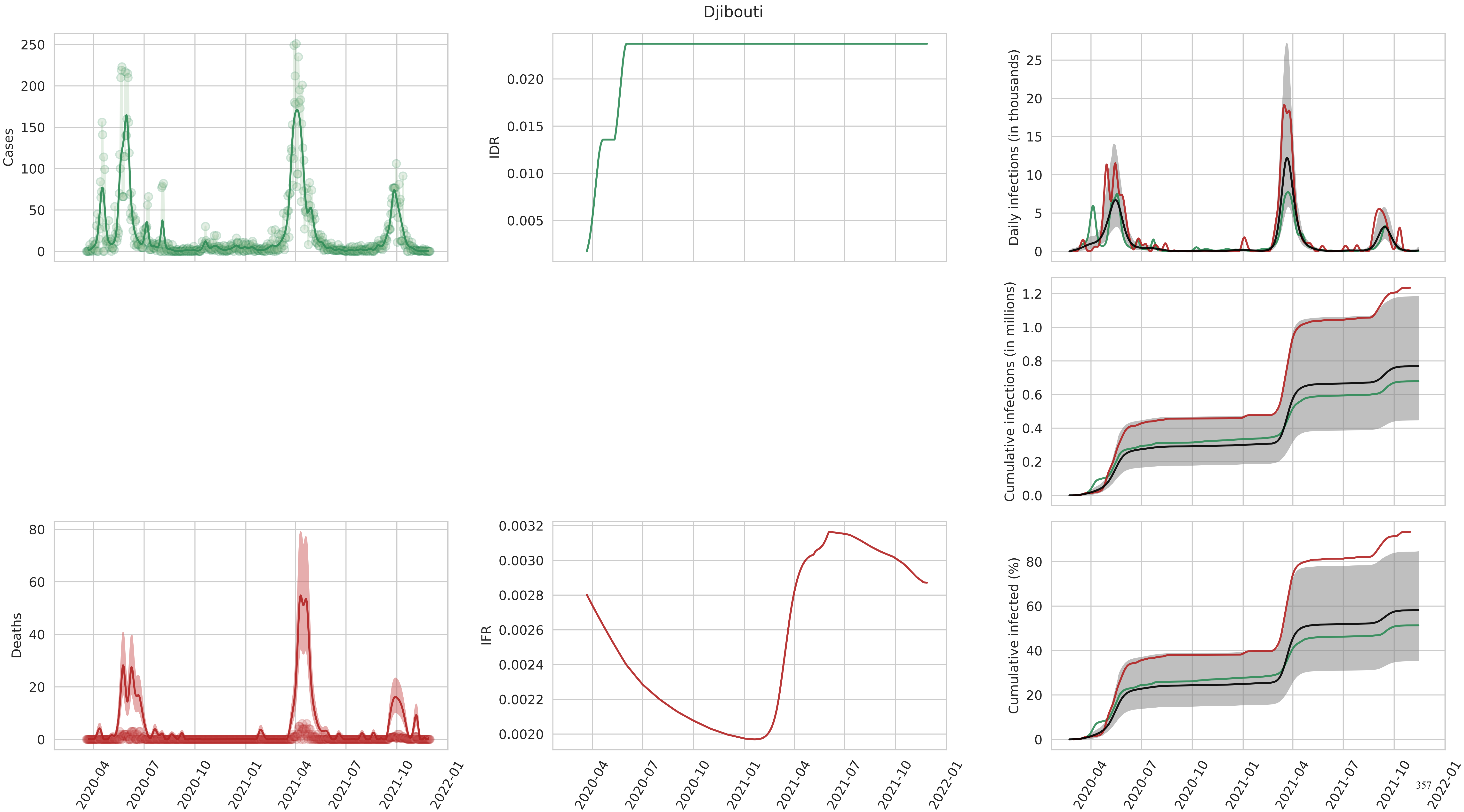
Djibouti
Cases
IDR
Daily infections (in thousands)
Cumulative infections (in millions)
Deaths
IFR
Cumulative infected (%)
2020-04
2020-07
2020-10
2021-01
2021-04
2021-07
2021-10
2022-01

Eritrea

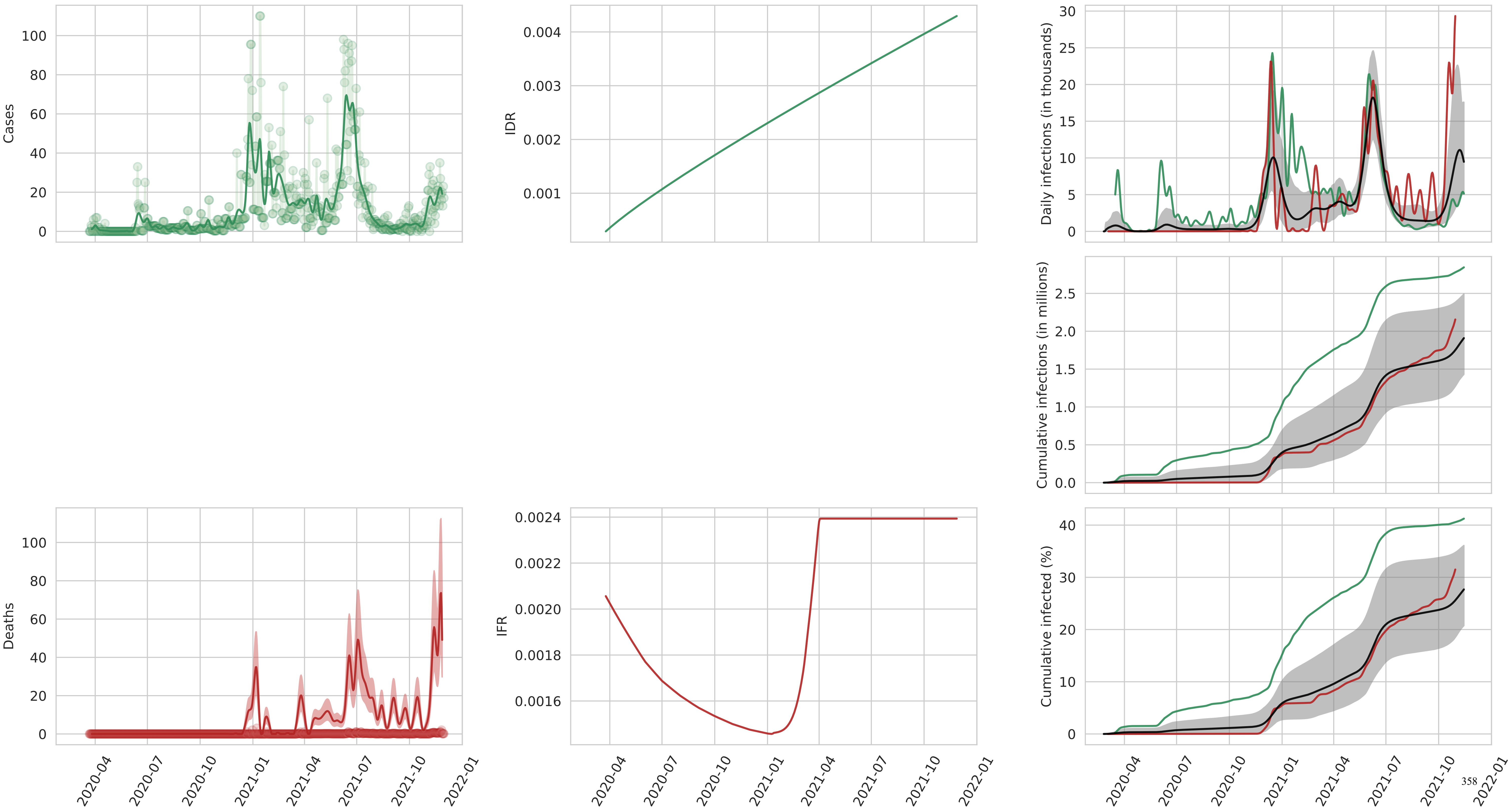

Cases
IDR
Daily infections (in thousands)
Cumulative infections (in millions)
Deaths
IFR
Cumulative infected (%)
2020-04
2020-07
2020-10
2021-01
2021-04
2021-07
2021-10
2022-01

Ethiopia

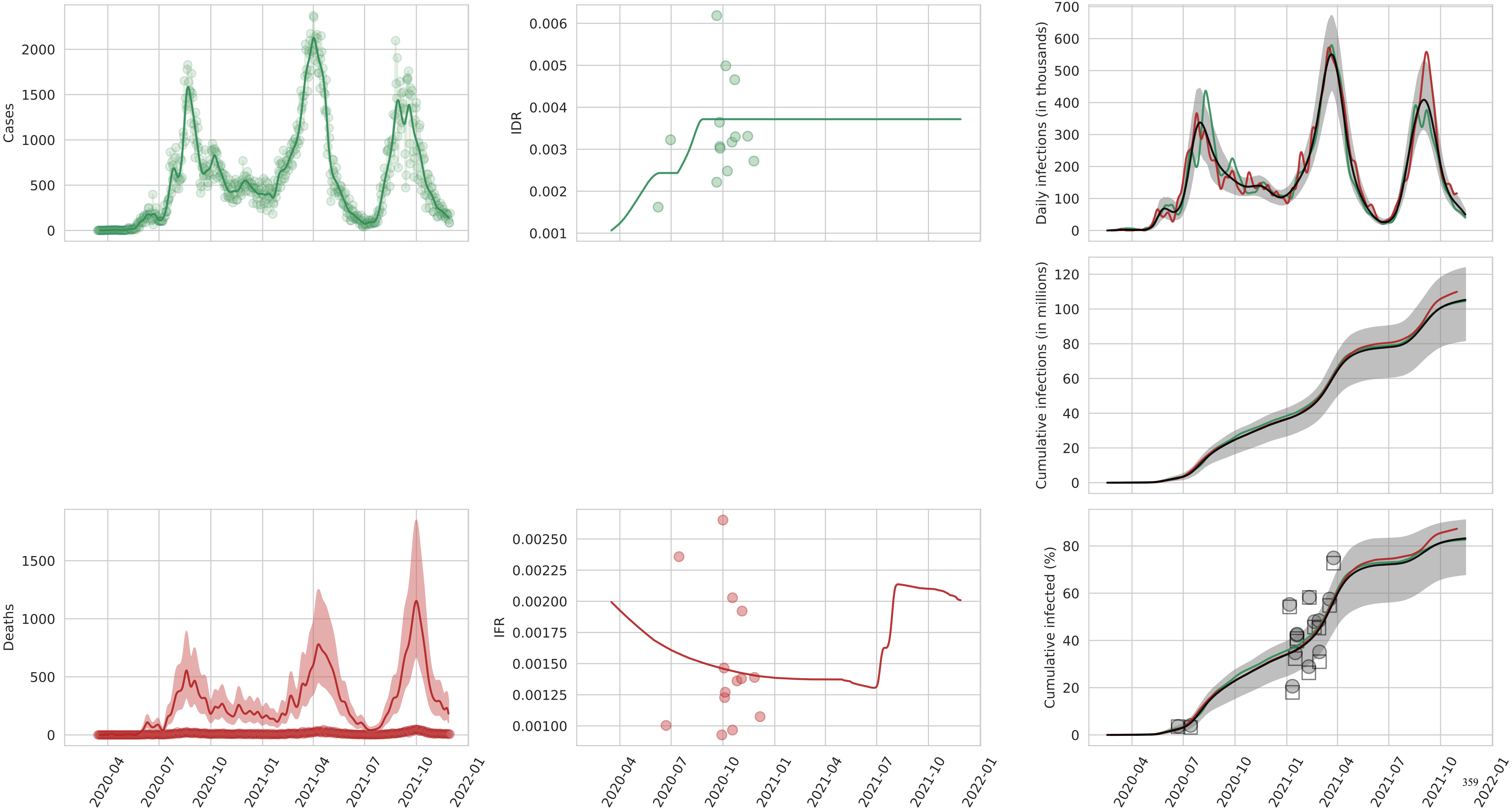

Cases
IDR
Daily infections (in thousands)
Cumulative infections (in millions)
Deaths
IFR
Cumulative infected (%)
2020-04
2020-07
2020-10
2021-01
2021-04
2021-07
2021-10
2022-01

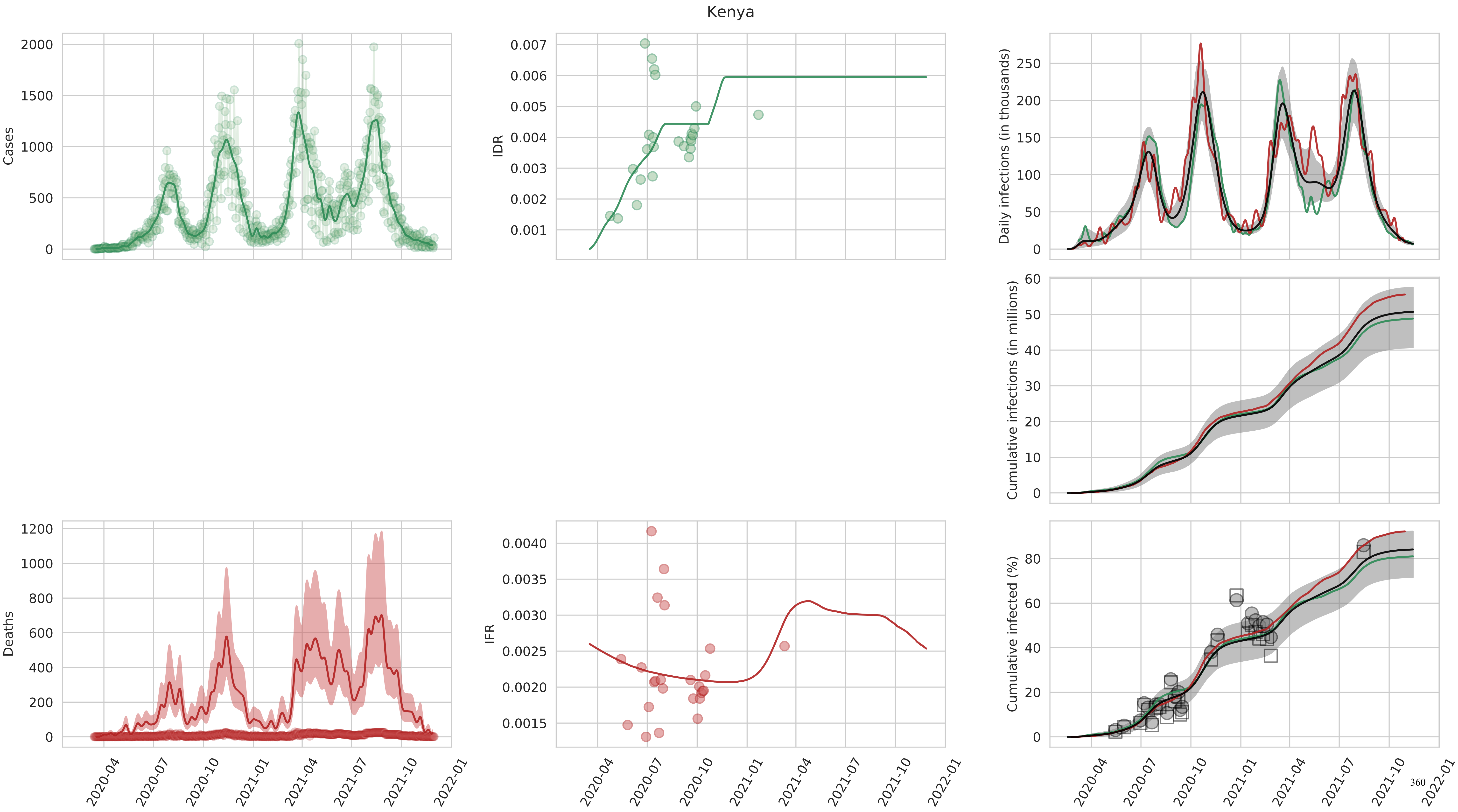
Kenya
Cases
2000
1500
1000
500
0
IDR
0.007
0.006
0.005
0.004
0.003
0.002
0.001
Daily infections (in thousands)
250
200
150
100
50
0
Cumulative infections (in millions)
60
50
40
30
20
10
0
Deaths
1200
1000
800
600
400
200
0
IFR
0.0040
0.0035
0.0030
0.0025
0.0020
0.0015
Cumulative infected (%)
80
60
40
20
0
2020-04
2020-07
2020-10
2021-01
2021-04
2021-07
2021-10
2022-01

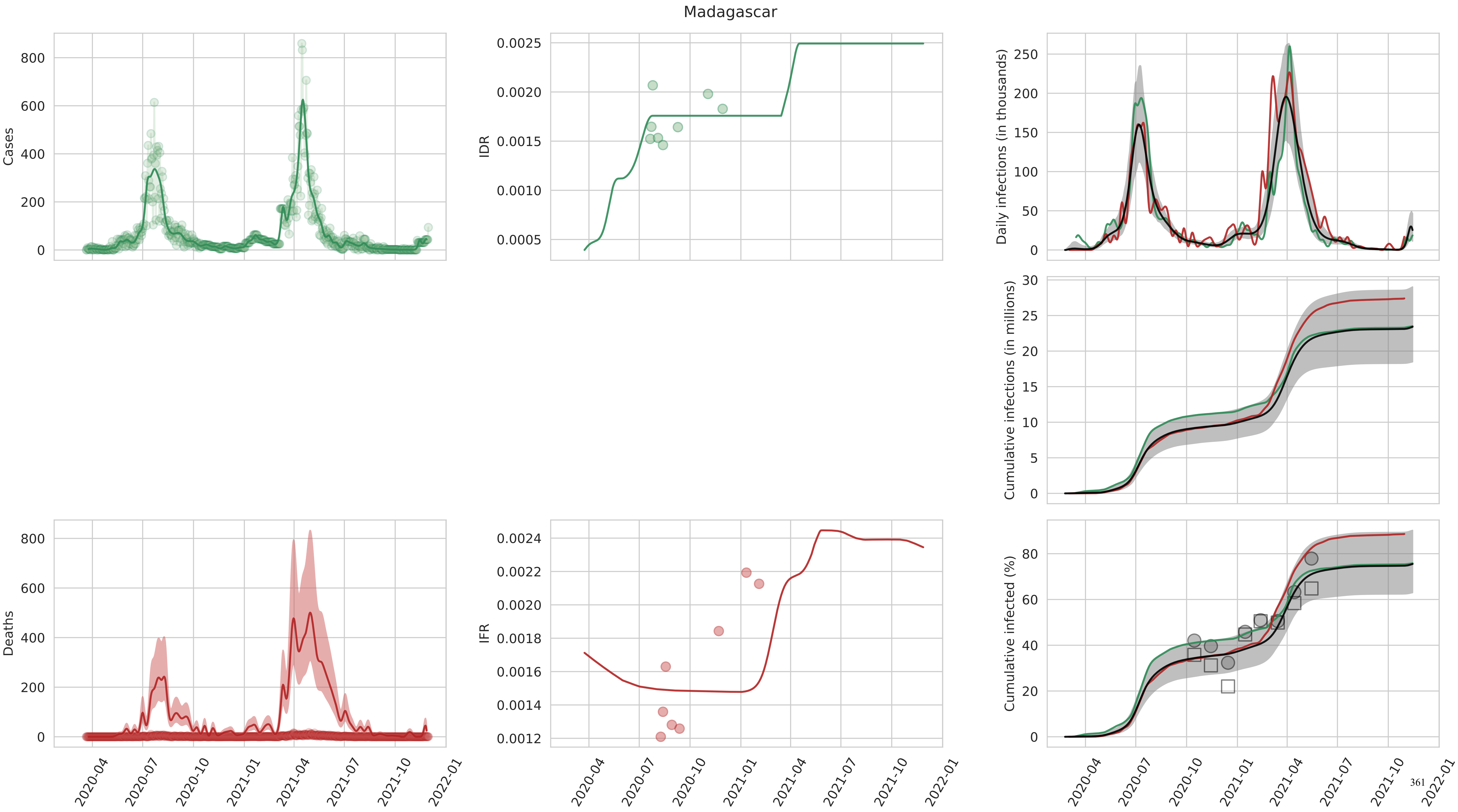
Madagascar
Cases
IDR
Daily infections (in thousands)
Cumulative infections (in millions)
Deaths
IFR
Cumulative infected (%)
2020-04
2020-07
2020-10
2021-01
2021-04
2021-07
2021-10
2022-01

Malawi

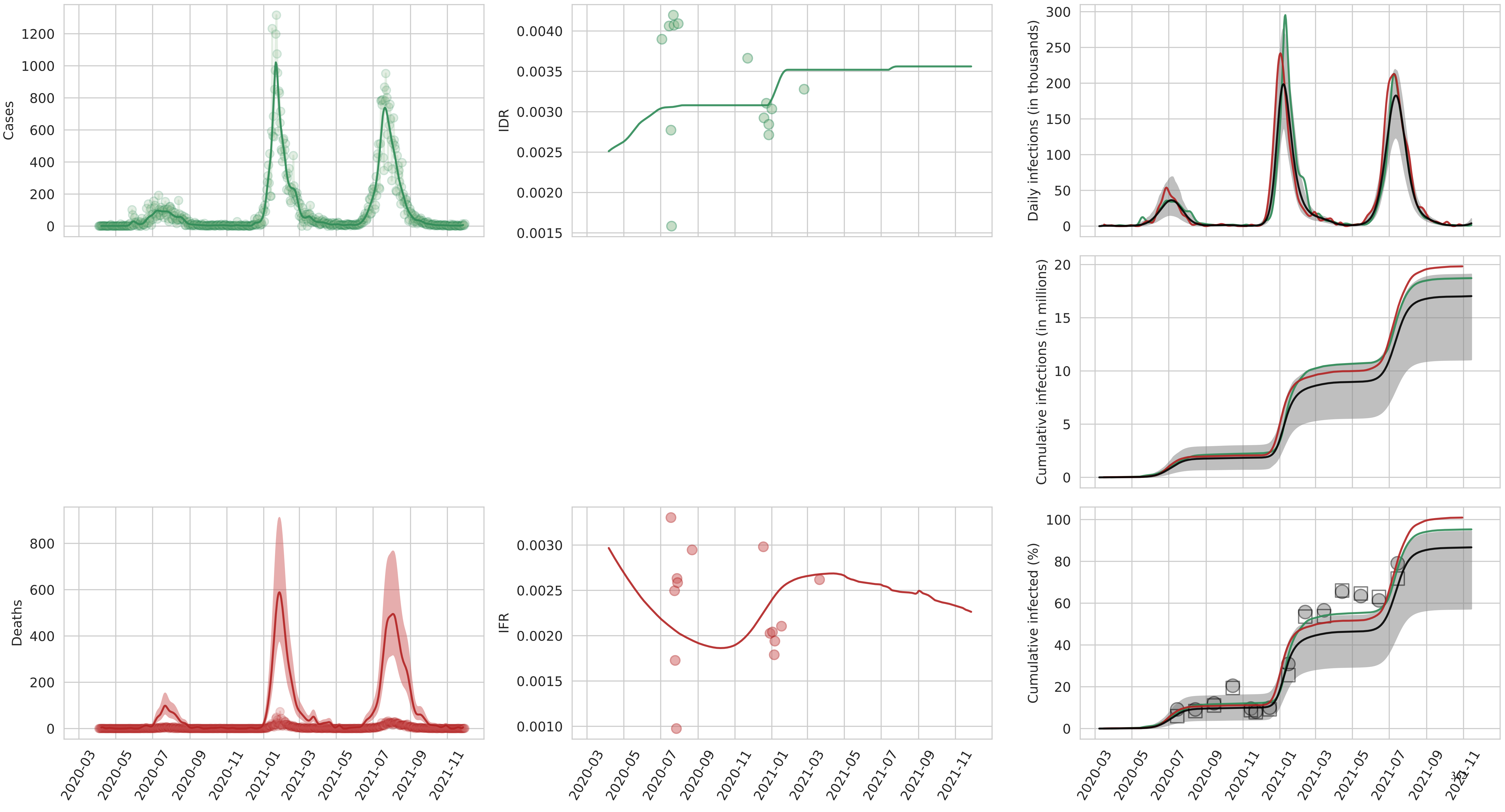

Cases
IDR
Daily infections (in thousands)
Cumulative infections (in millions)
Deaths
IFR
Cumulative infected (%)
2020-03
2020-05
2020-07
2020-09
2020-11
2021-01
2021-03
2021-05
2021-07
2021-09
2021-11

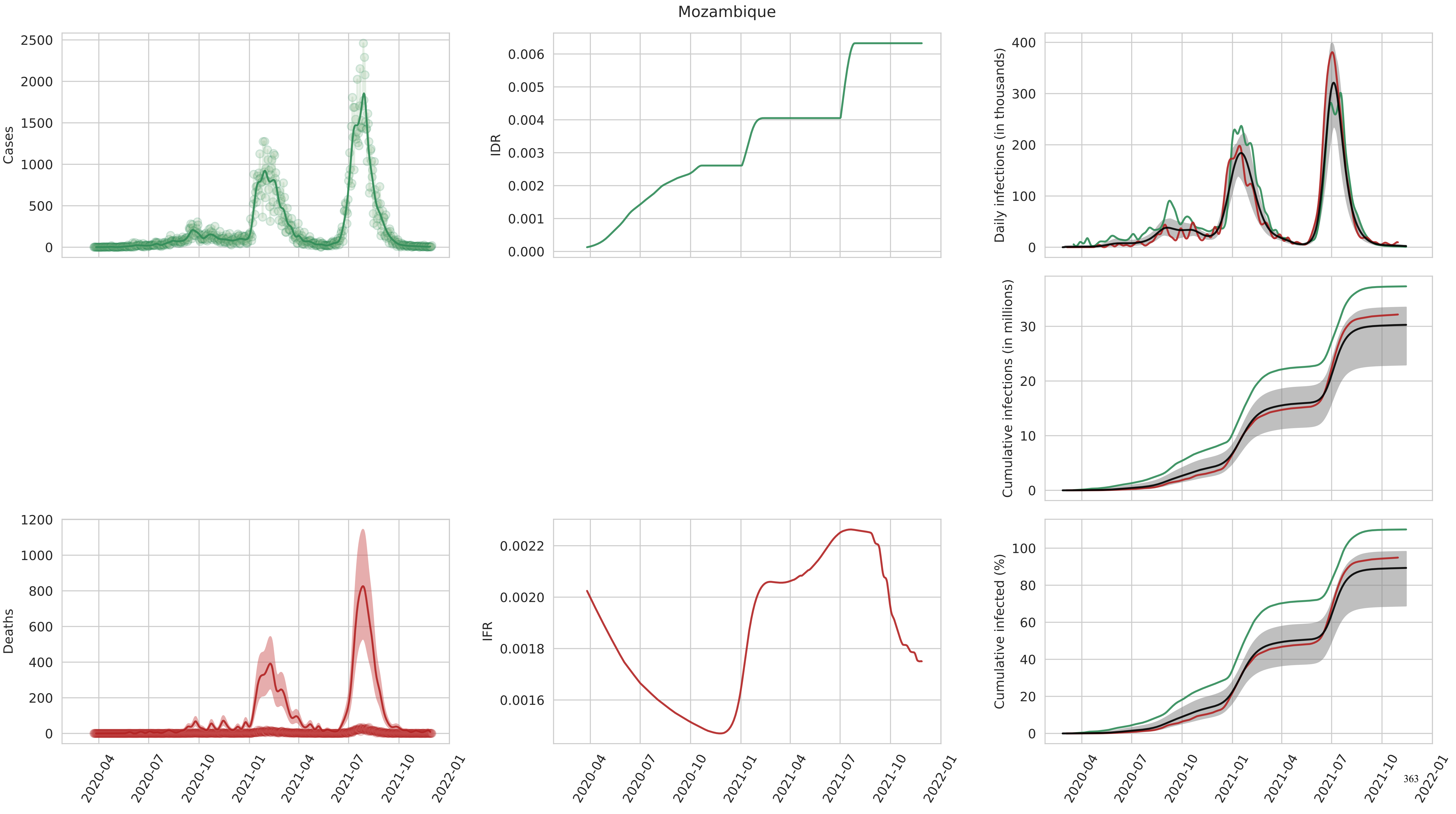
Mozambique
Cases
IDR
Daily infections (in thousands)
Cumulative infections (in millions)
Deaths
IFR
Cumulative infected (%)
2020-04
2020-07
2020-10
2021-01
2021-04
2021-07
2021-10
2022-01

Rwanda

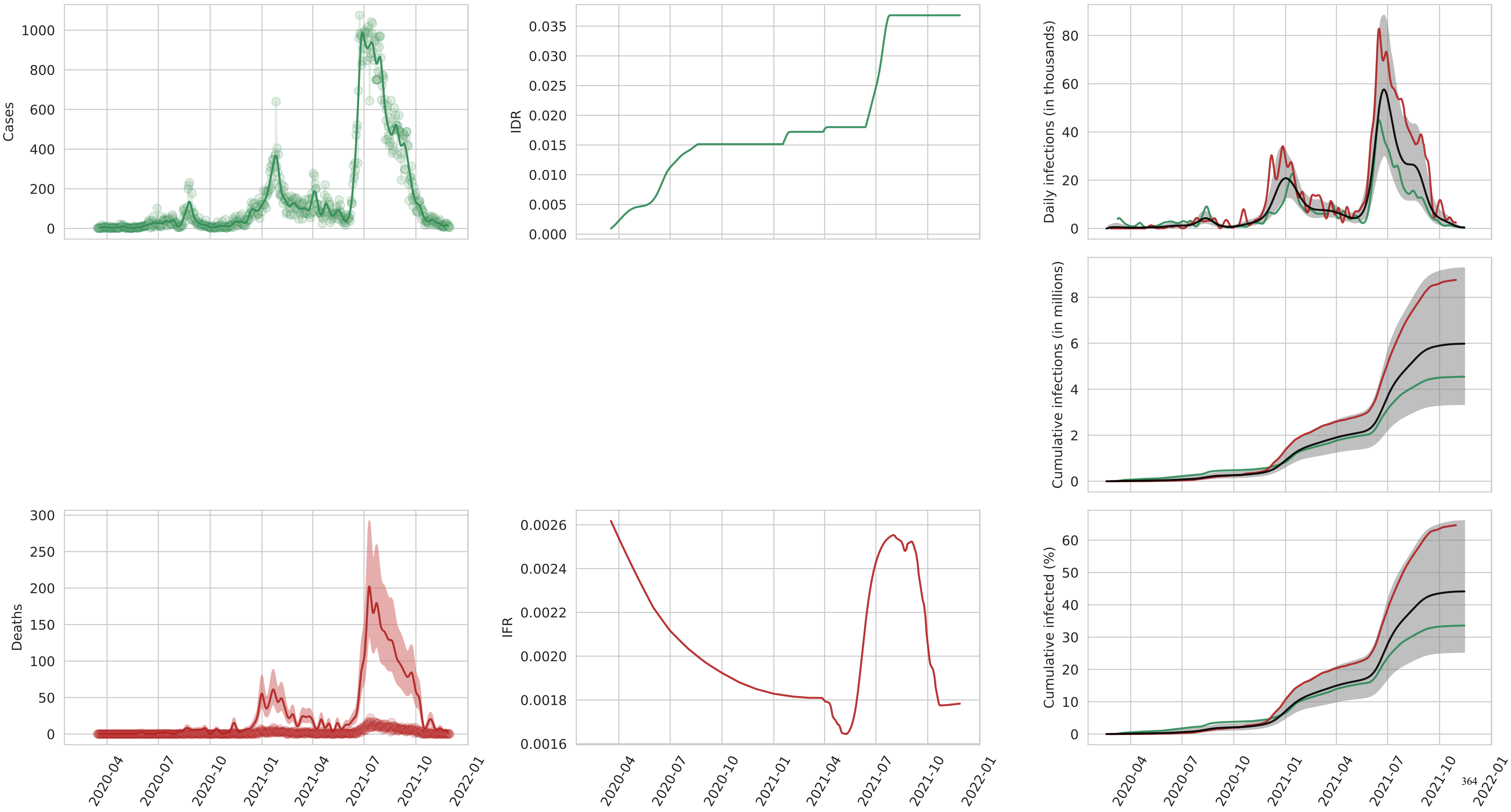

Cases
1000
800
600
400
200
0
IDR
0.035
0.030
0.025
0.020
0.015
0.010
0.005
0.000
Daily infections (in thousands)
80
60
40
20
0
Cumulative infections (in millions)
8
6
4
2
0
Deaths
300
250
200
150
100
50
0
IFR
0.0026
0.0024
0.0022
0.0020
0.0018
0.0016
Cumulative infected (%)
60
50
40
30
20
10
0
2020-04
2020-07
2020-10
2021-01
2021-04
2021-07
2021-10
2022-01

Somalia

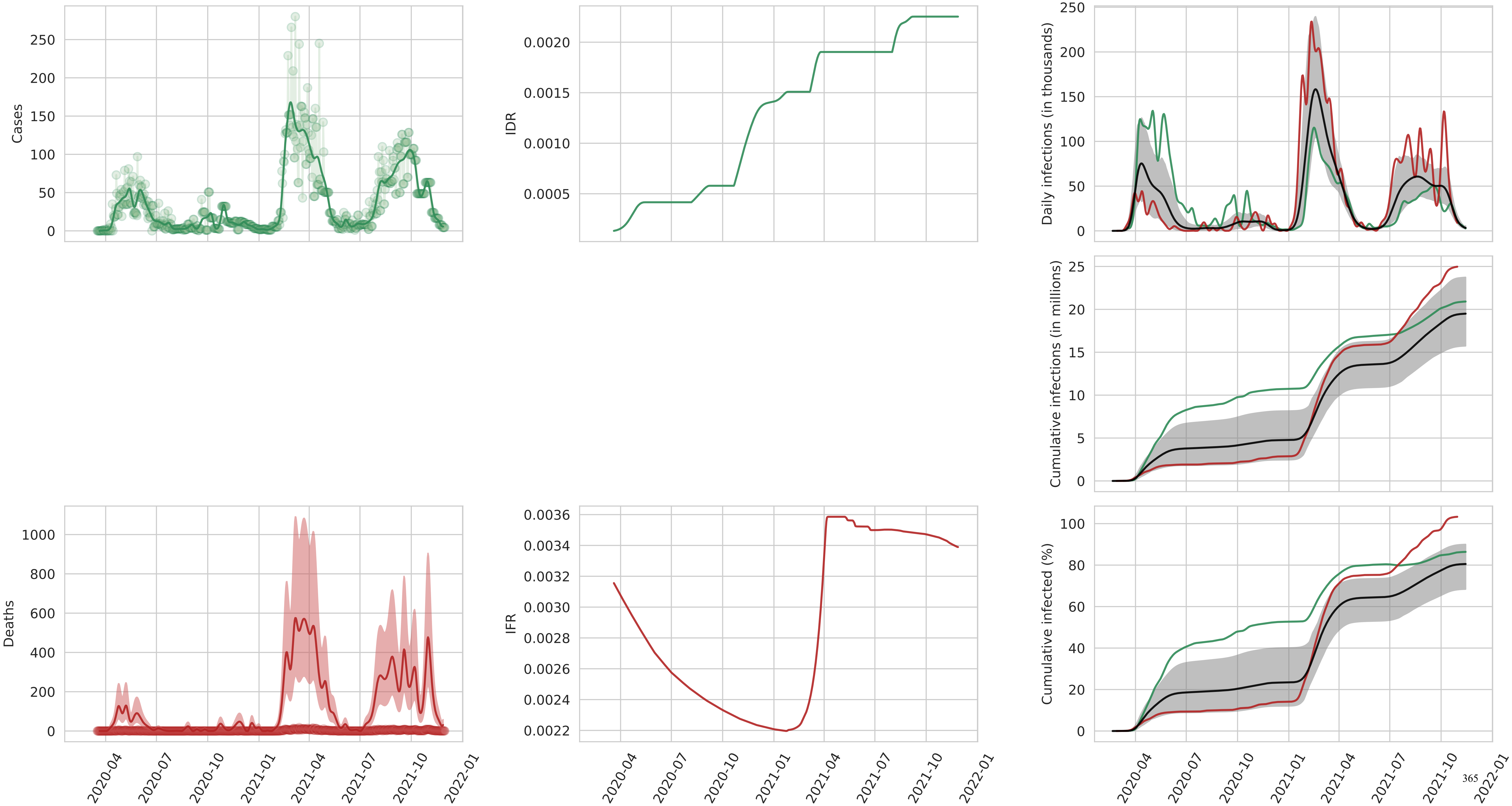

Cases
IDR
Daily infections (in thousands)
Cumulative infections (in millions)
Deaths
IFR
Cumulative infected (%)
2020-04
2020-07
2020-10
2021-01
2021-04
2021-07
2021-10
2022-01

South Sudan

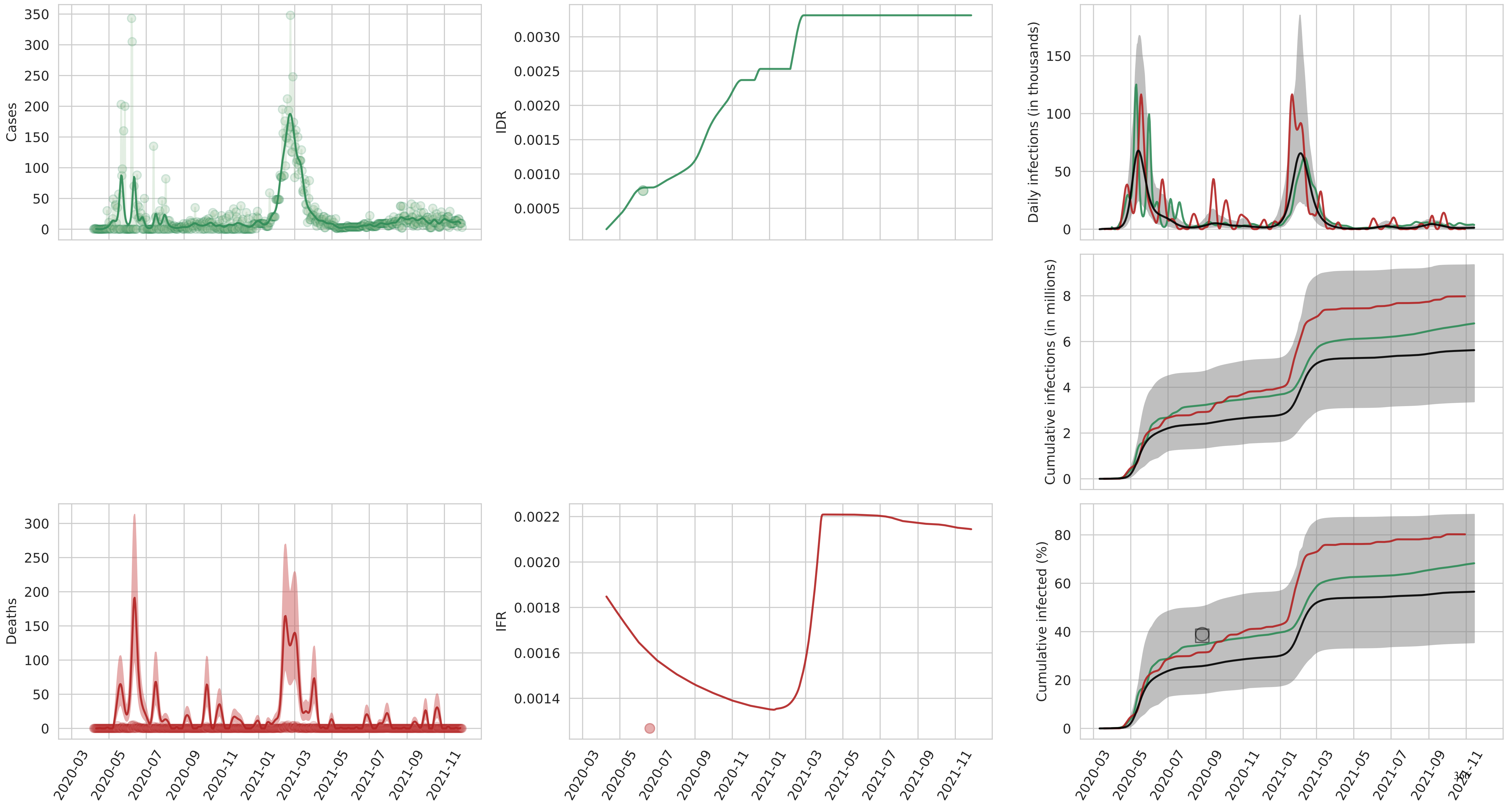

Cases
IDR
Daily infections (in thousands)
Cumulative infections (in millions)
Deaths
IFR
Cumulative infected (%)
2020-03
2020-05
2020-07
2020-09
2020-11
2021-01
2021-03
2021-05
2021-07
2021-09
2021-11

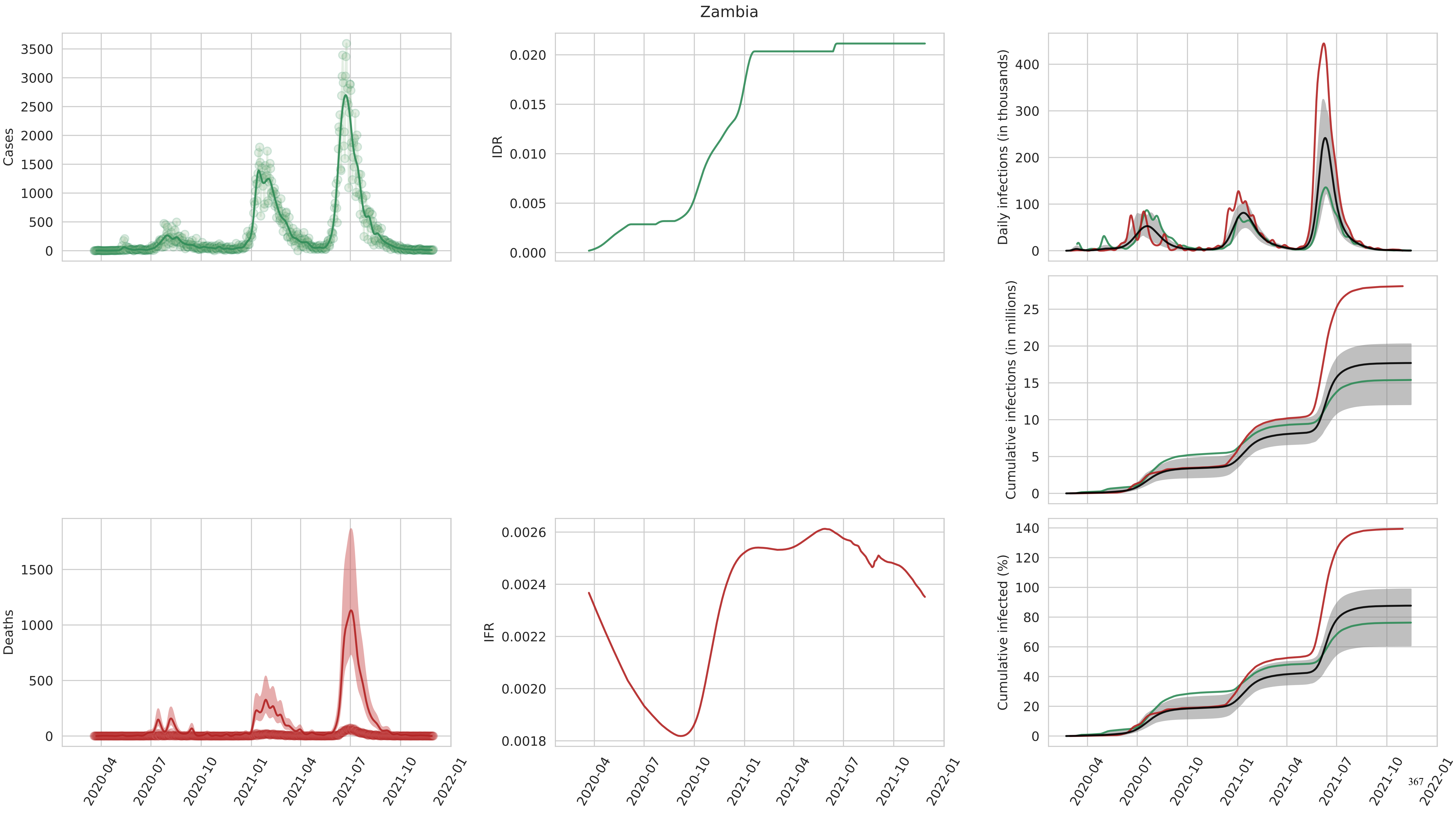
Zambia
Cases
IDR
Daily infections (in thousands)
Cumulative infections (in millions)
Deaths
IFR
Cumulative infected (%)
2020-04
2020-07
2020-10
2021-01
2021-04
2021-07
2021-10
2022-01

Botswana

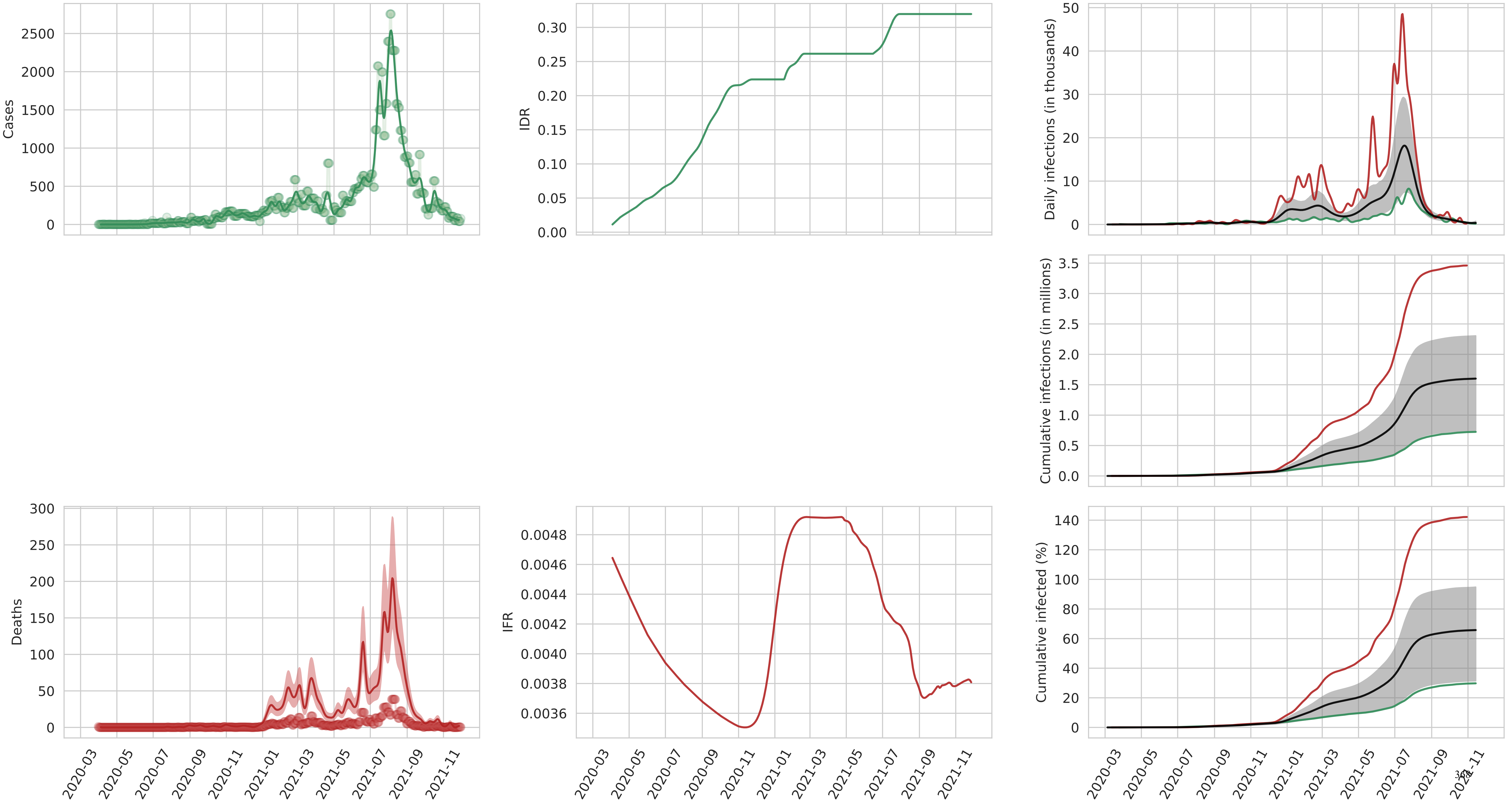
Cases
IDR
Daily infections (in thousands)
Cumulative infections (in millions)
Deaths
IFR
Cumulative infected (%)
2020-03
2020-05
2020-07
2020-09
2020-11
2021-01
2021-03
2021-05
2021-07
2021-09
2021-11

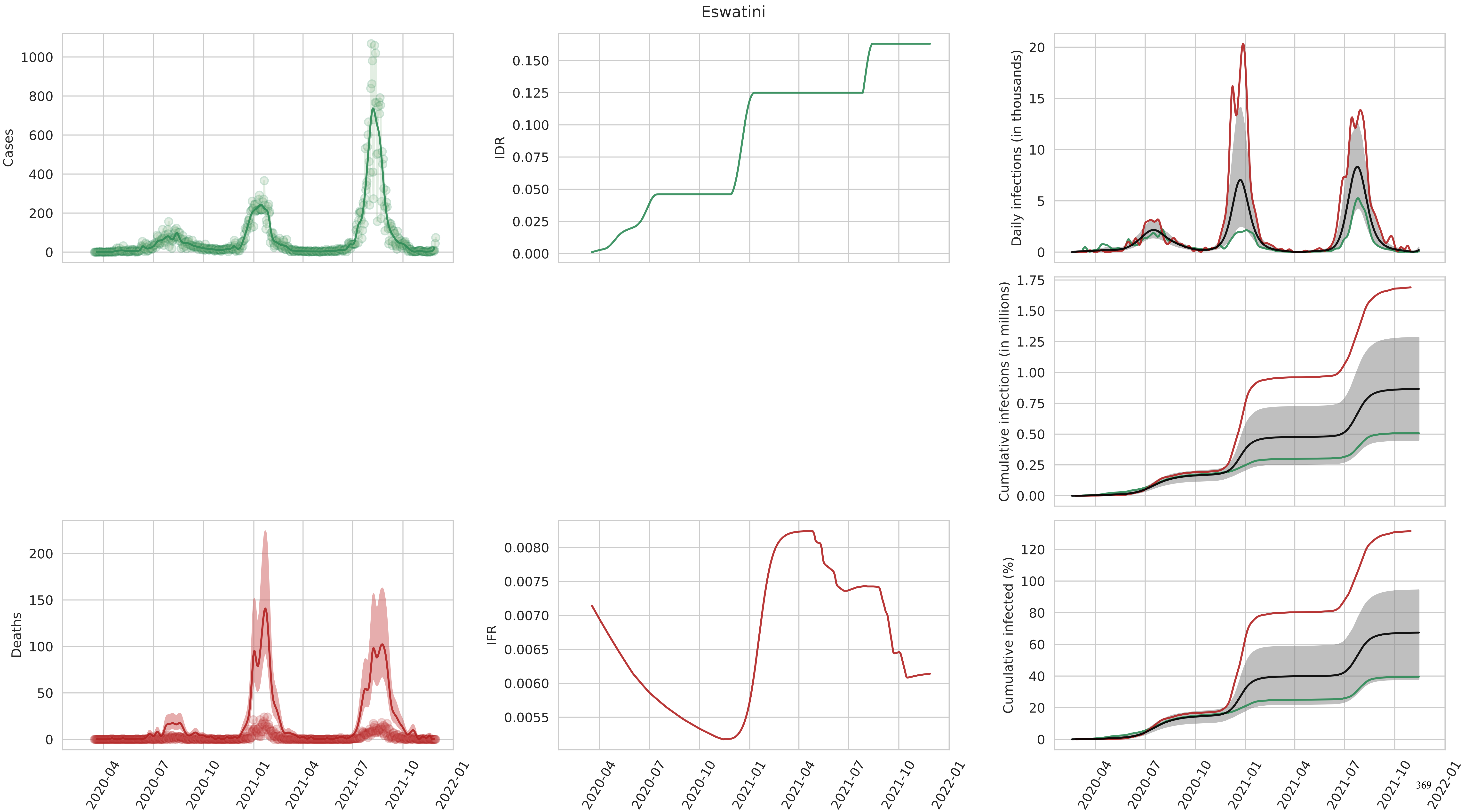

Eswatini
Cases
IDR
Daily infections (in thousands)
Cumulative infections (in millions)
Deaths
IFR
Cumulative infected (%)

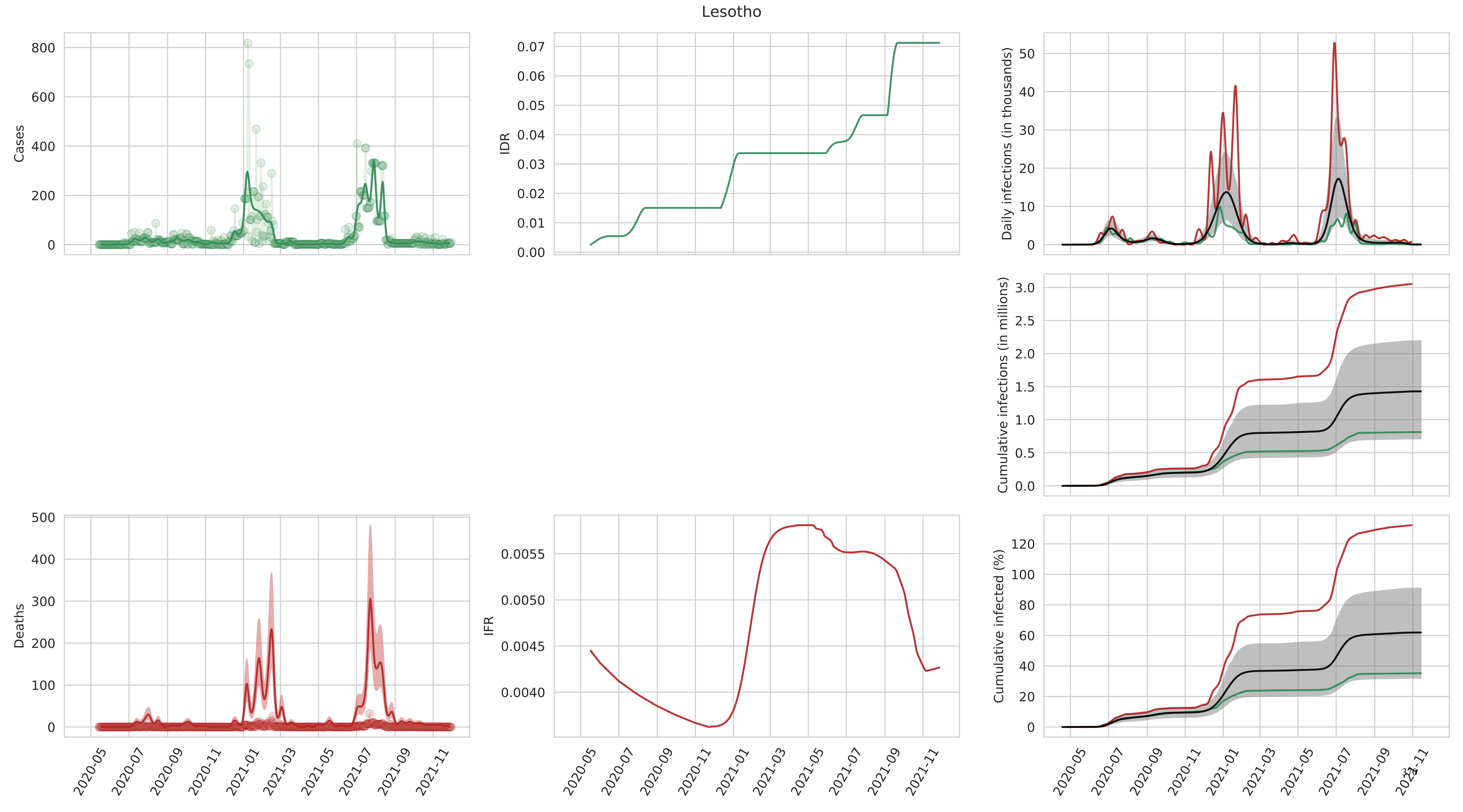
Lesotho
Cases
IDR
Daily infections (in thousands)
Cumulative infections (in millions)
Deaths
IFR
Cumulative infected (%)
2020-05
2020-07
2020-09
2020-11
2021-01
2021-03
2021-05
2021-07
2021-09
2021-11

Namibia

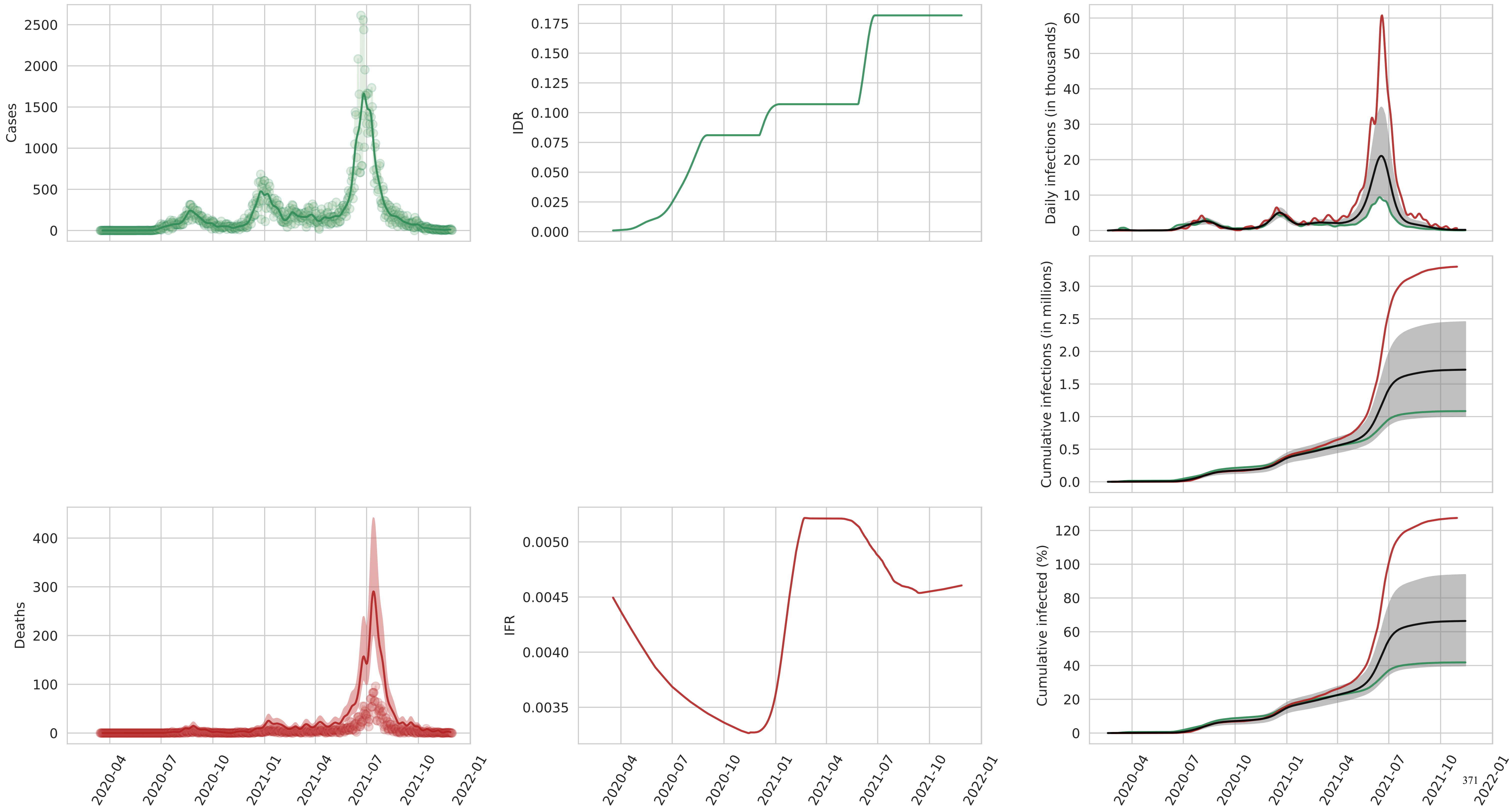
Cases
IDR
Daily infections (in thousands)
Cumulative infections (in millions)
Deaths
IFR
Cumulative infected (%)
2020-04
2020-07
2020-10
2021-01
2021-04
2021-07
2021-10
2022-01

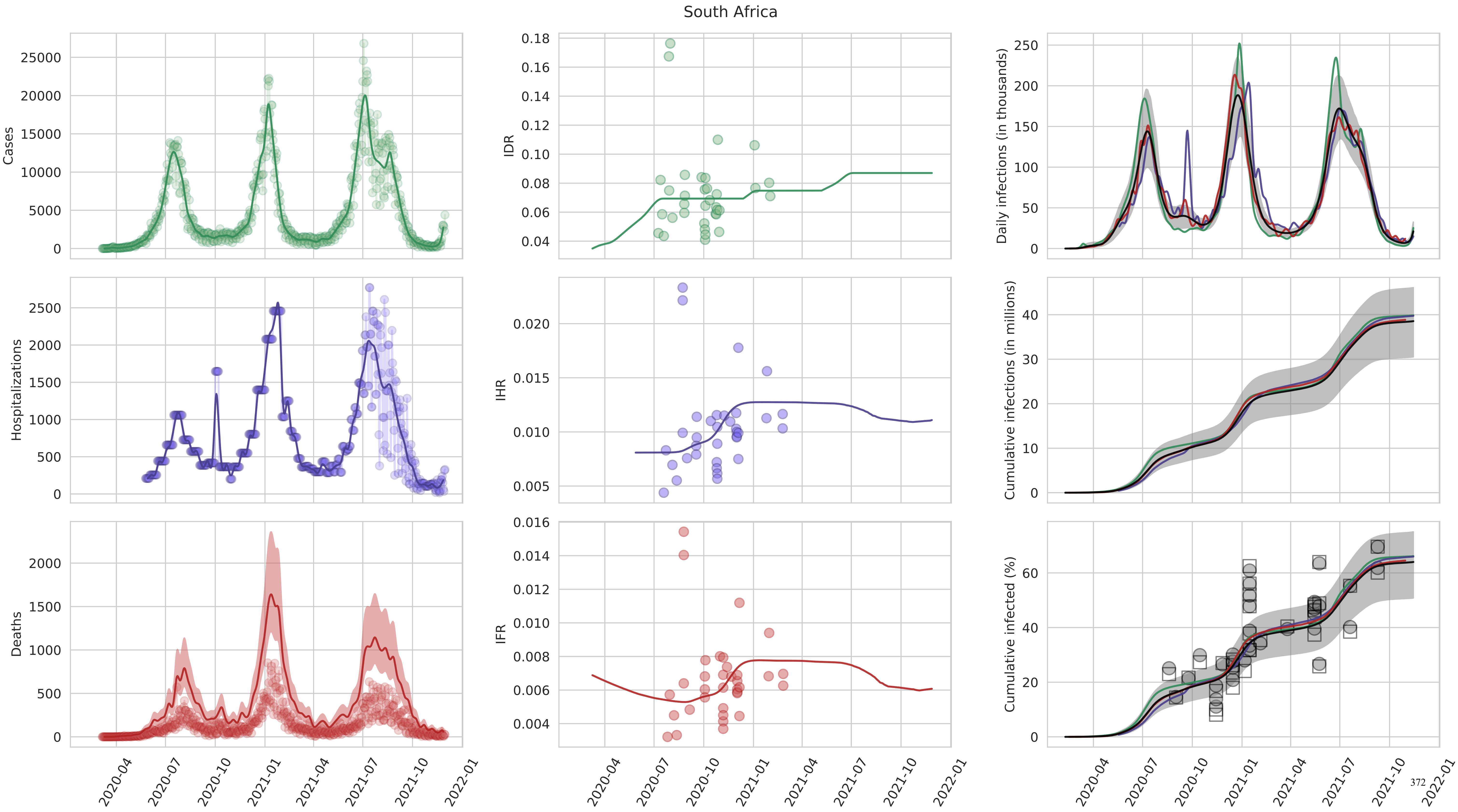
South Africa
Cases
Hospitalizations
Deaths
IDR
IHR
IFR
Daily infections (in thousands)
Cumulative infections (in millions)
Cumulative infected (%)
2020-04
2020-07
2020-10
2021-01
2021-04
2021-07
2021-10
2022-01

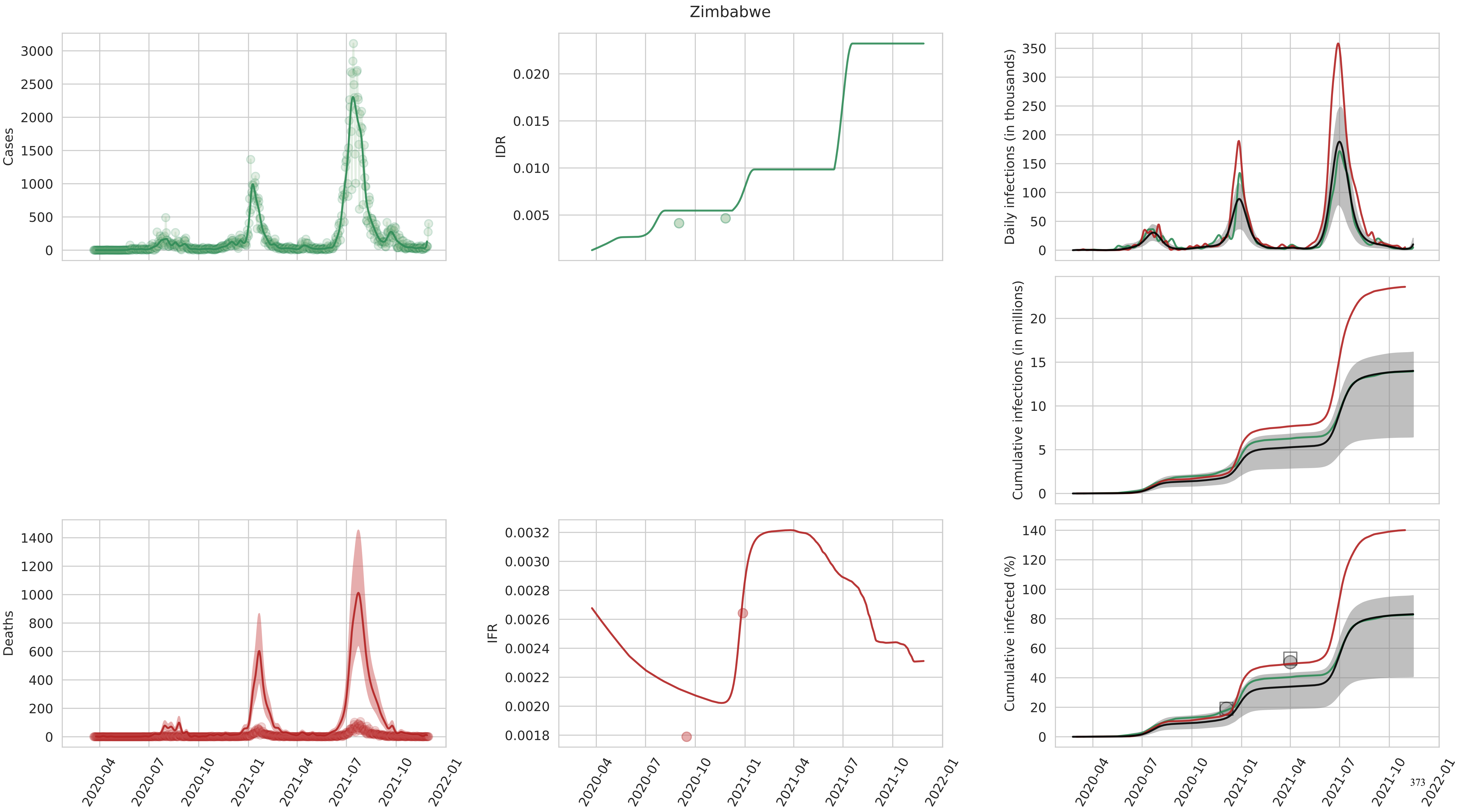
Zimbabwe
Cases
IDR
Daily infections (in thousands)
Cumulative infections (in millions)
Deaths
IFR
Cumulative infected (%)
2020-04
2020-07
2020-10
2021-01
2021-04
2021-07
2021-10
2022-01

Benin

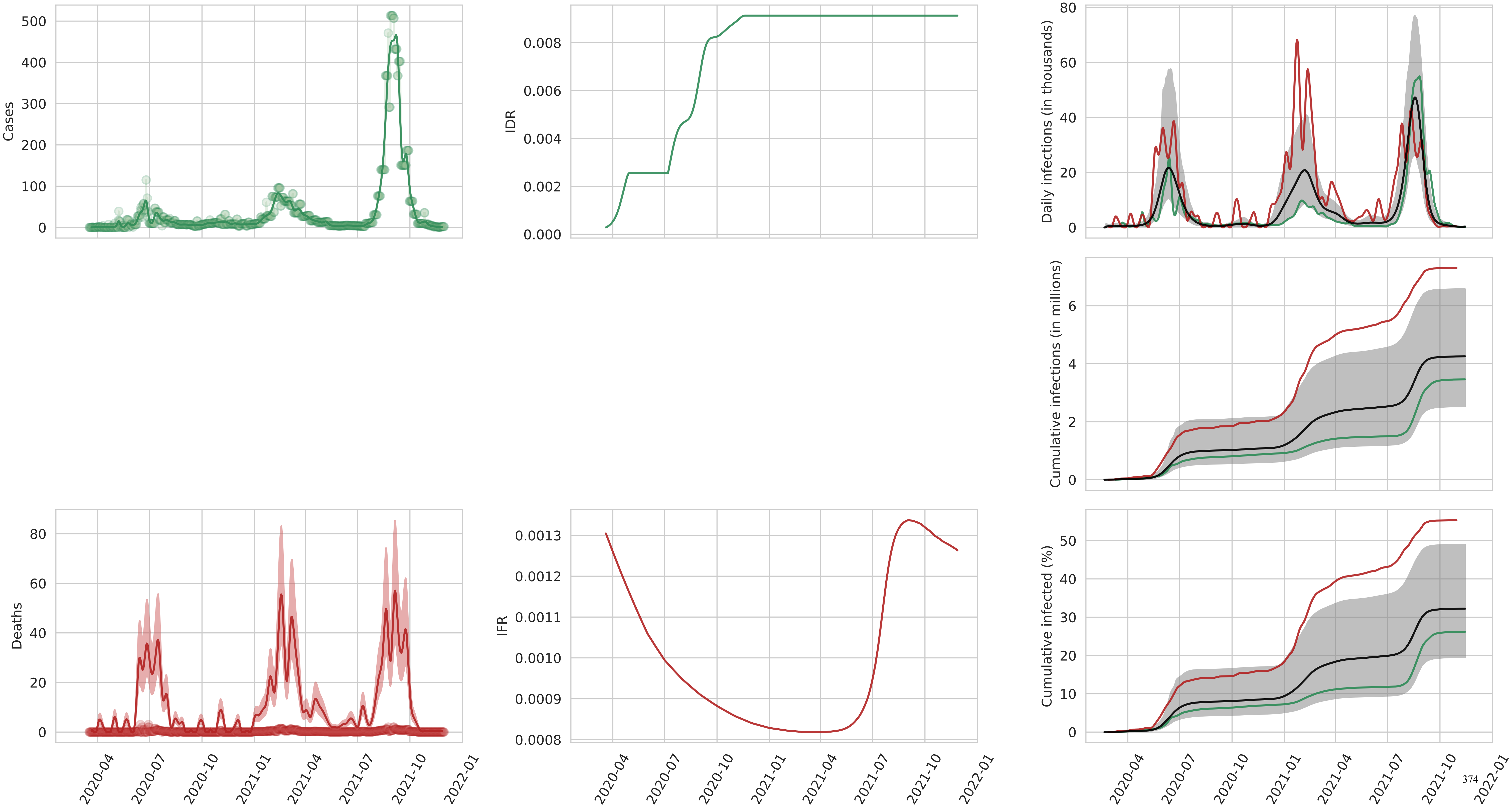

Cases
IDR
Daily infections (in thousands)
Cumulative infections (in millions)
Deaths
IFR
Cumulative infected (%)
2020-04
2020-07
2020-10
2021-01
2021-04
2021-07
2021-10
2022-01

Burkina Faso

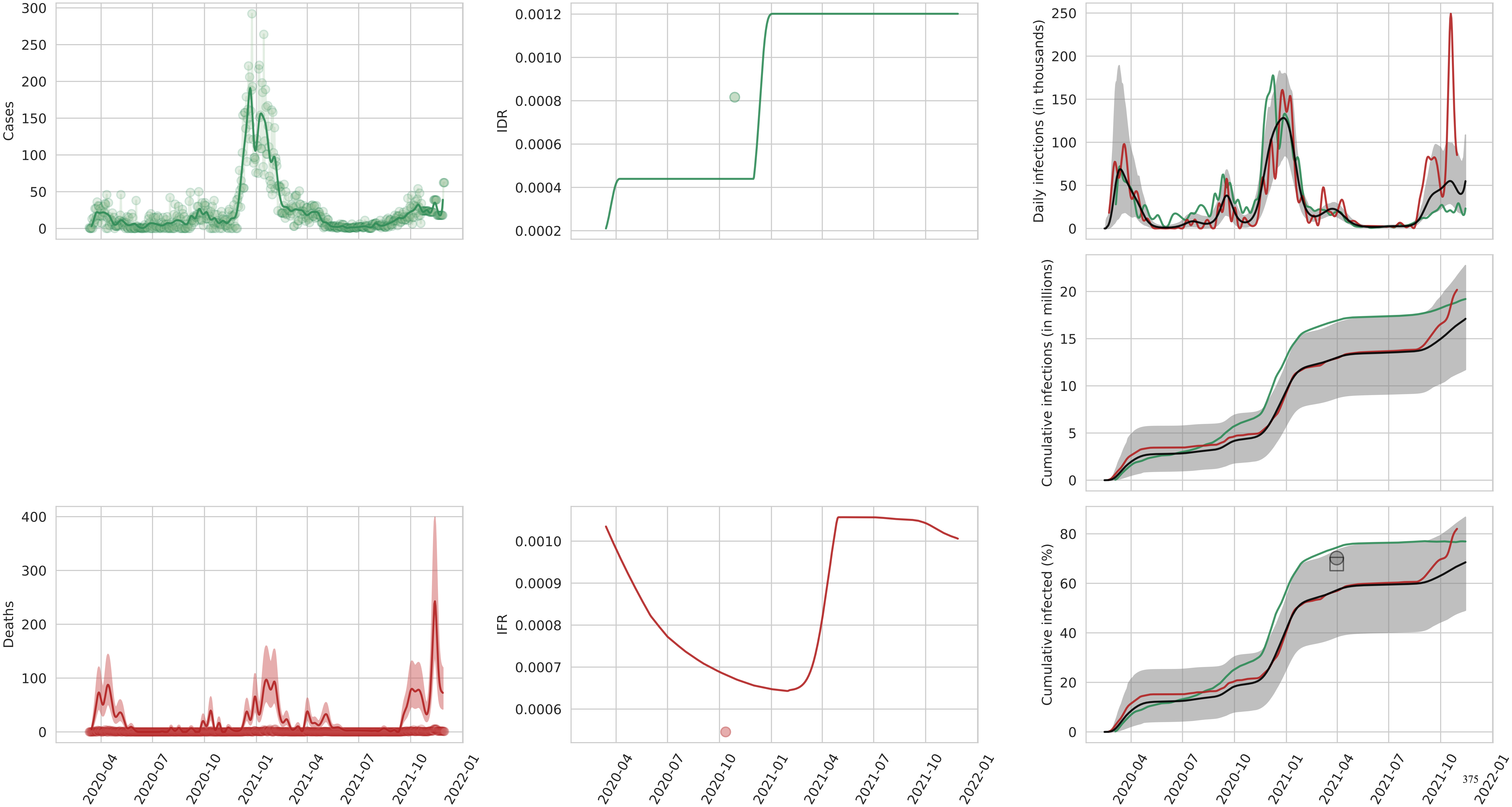

Cabo Verde

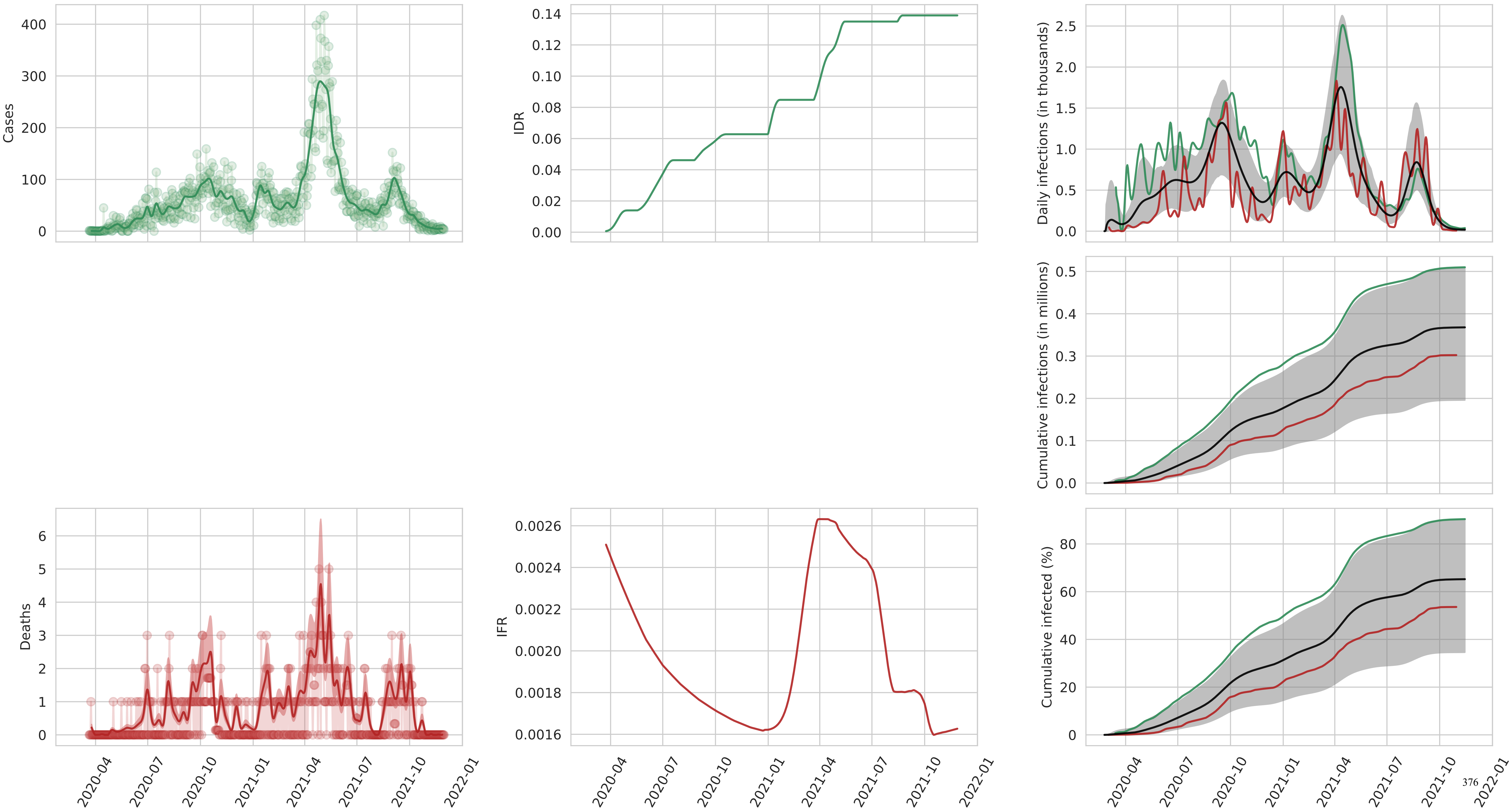
Cases
IDR
Daily infections (in thousands)
Cumulative infections (in millions)
Deaths
IFR
Cumulative infected (%)

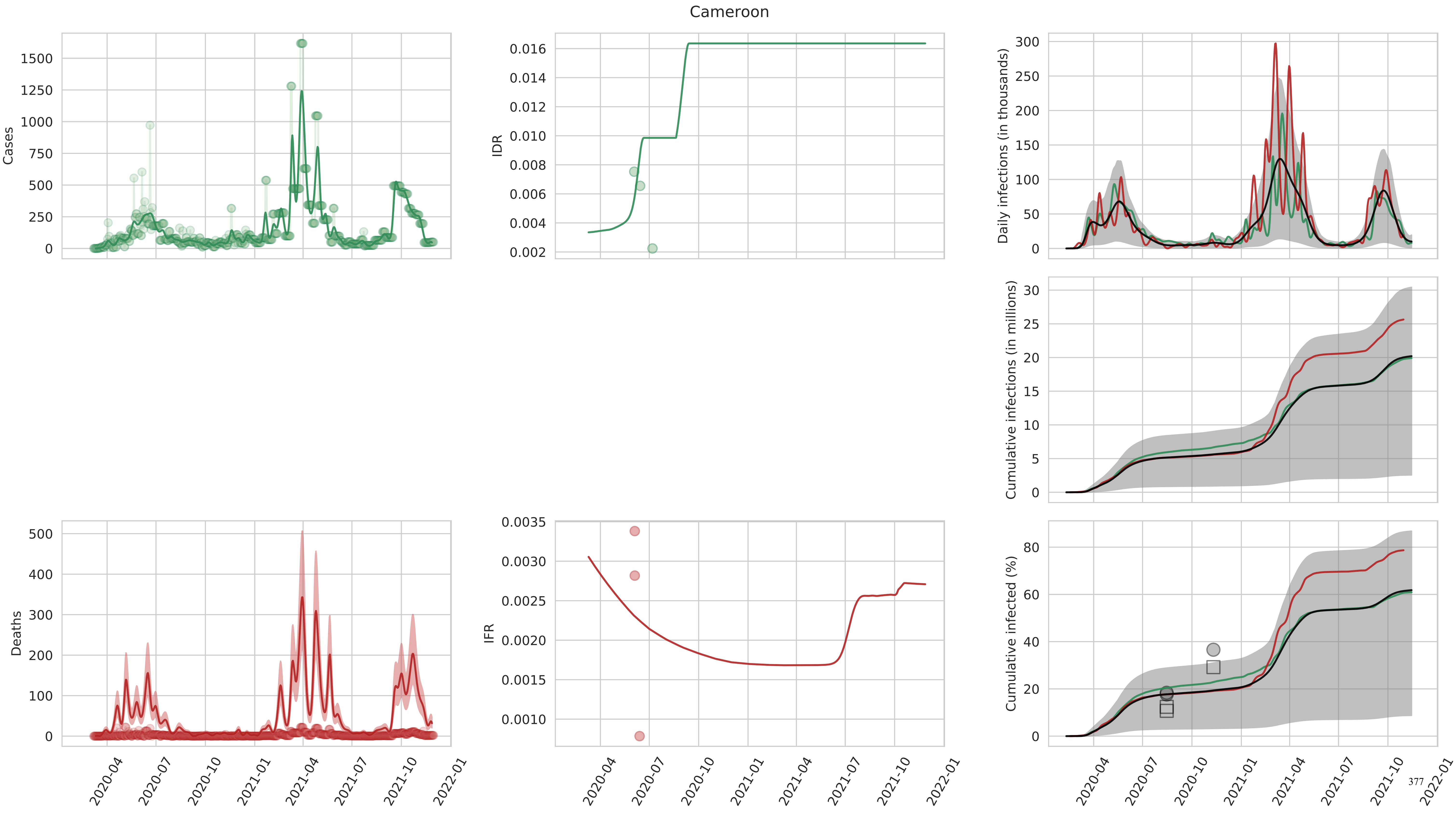
Cameroon
Cases
IDR
Daily infections (in thousands)
Cumulative infections (in millions)
Deaths
IFR
Cumulative infected (%)
2020-04
2020-07
2020-10
2021-01
2021-04
2021-07
2021-10
2022-01

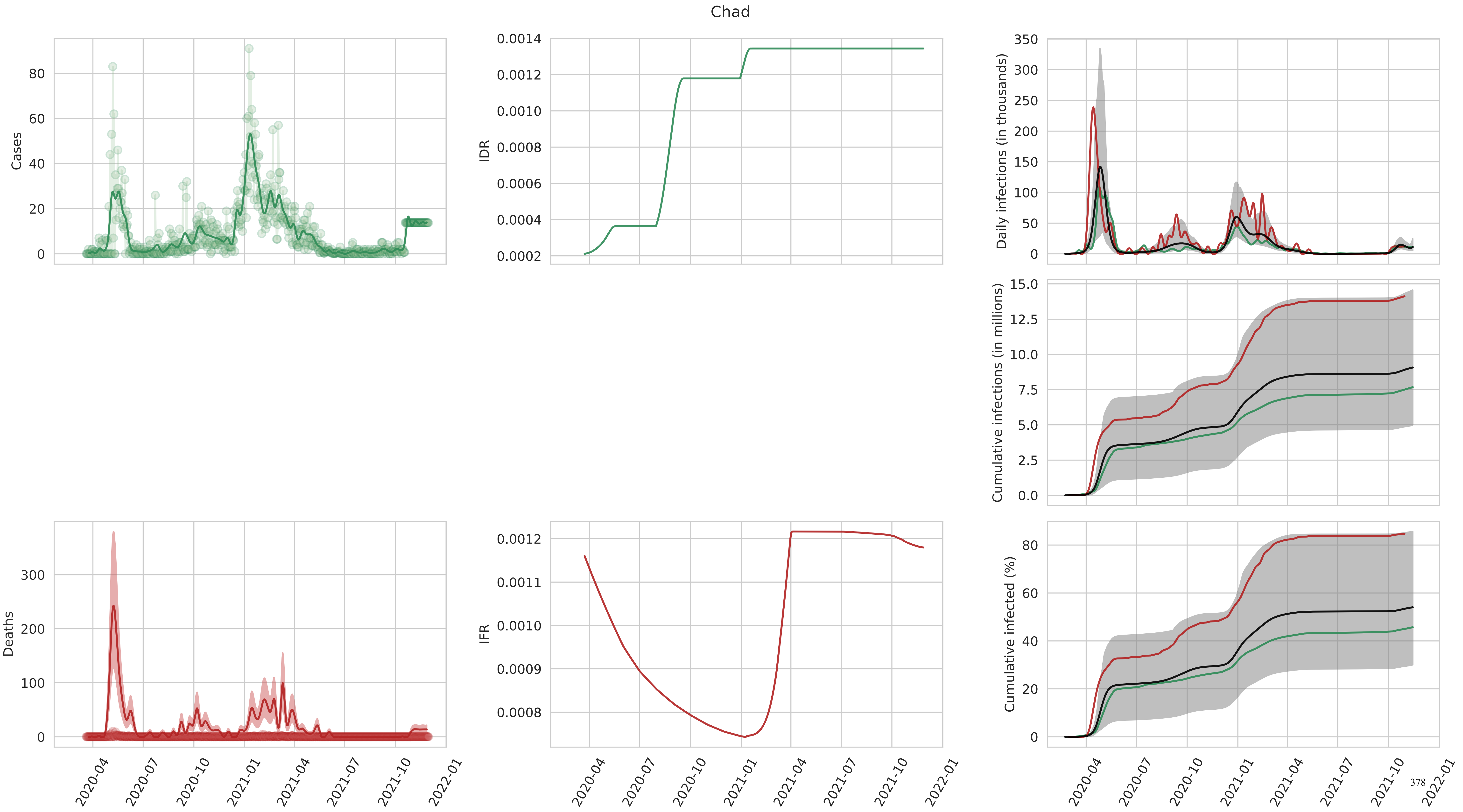
Chad
Cases
IDR
Daily infections (in thousands)
Cumulative infections (in millions)
Deaths
IFR
Cumulative infected (%)
2020-04
2020-07
2020-10
2021-01
2021-04
2021-07
2021-10
2022-01

Côte d'Ivoire

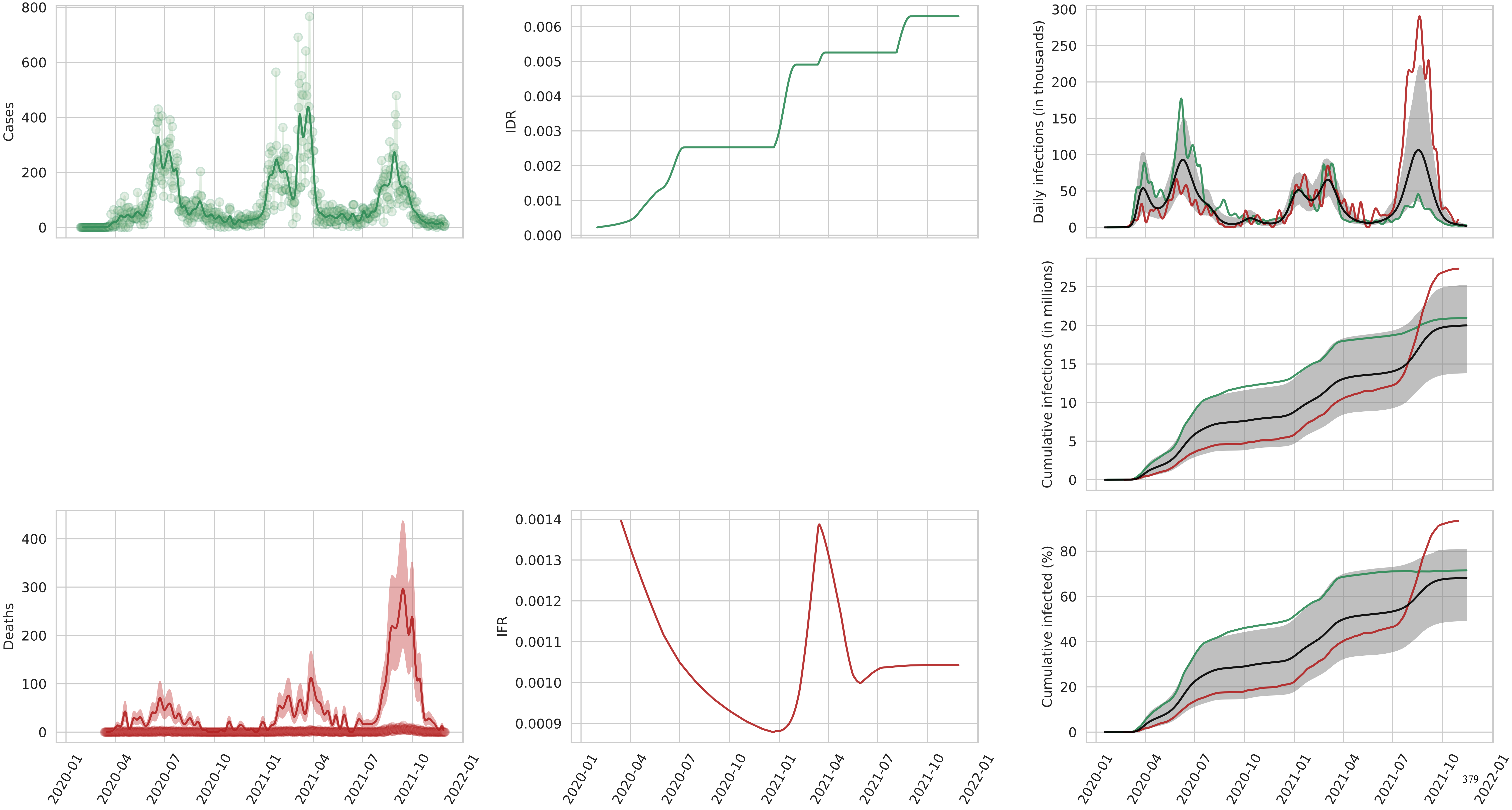

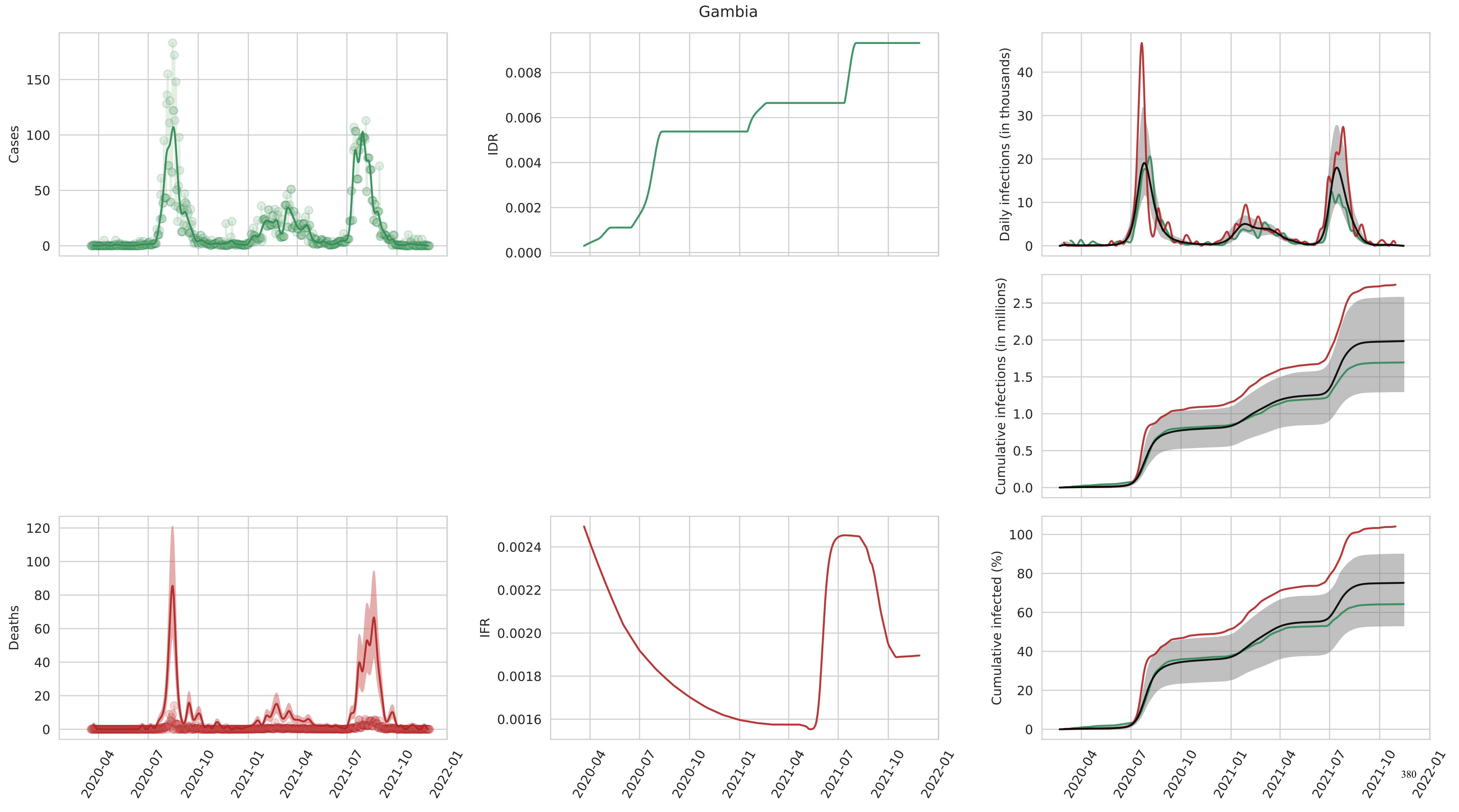
Gambia
Cases
IDR
Daily infections (in thousands)
Cumulative infections (in millions)
Deaths
IFR
Cumulative infected (%)
0
50
100
150
0.000
0.002
0.004
0.006
0.008
0
10
20
30
40
0.0
0.5
1.0
1.5
2.0
2.5
0
20
40
60
80
100
120
0.0016
0.0018
0.0020
0.0022
0.0024
0
20
40
60
80
100
2020-04
2020-07
2020-10
2021-01
2021-04
2021-07
2021-10
2022-01

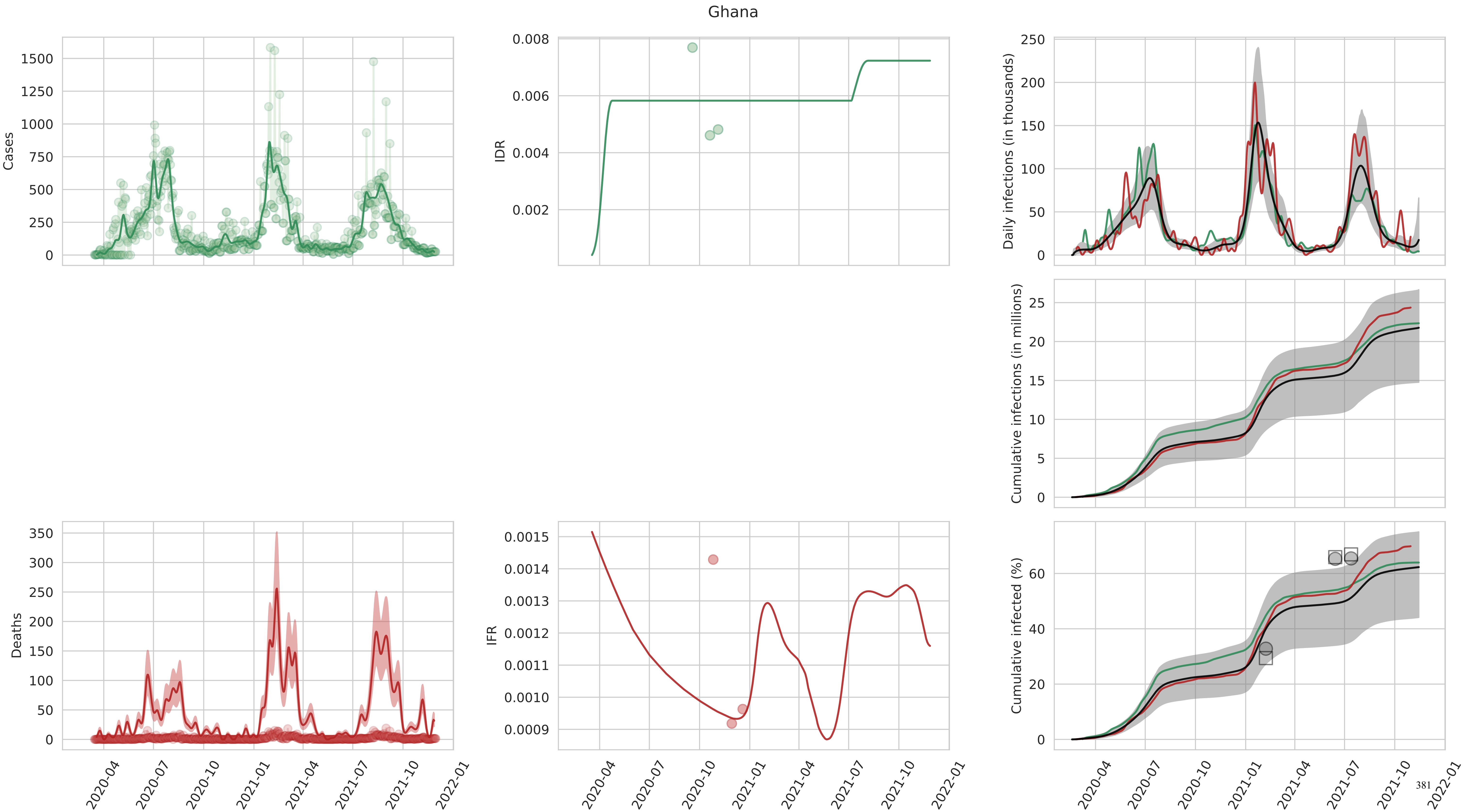

Ghana
Cases
IDR
Daily infections (in thousands)
Cumulative infections (in millions)
Deaths
IFR
Cumulative infected (%)
2020-04
2020-07
2020-10
2021-01
2021-04
2021-07
2021-10
2022-01

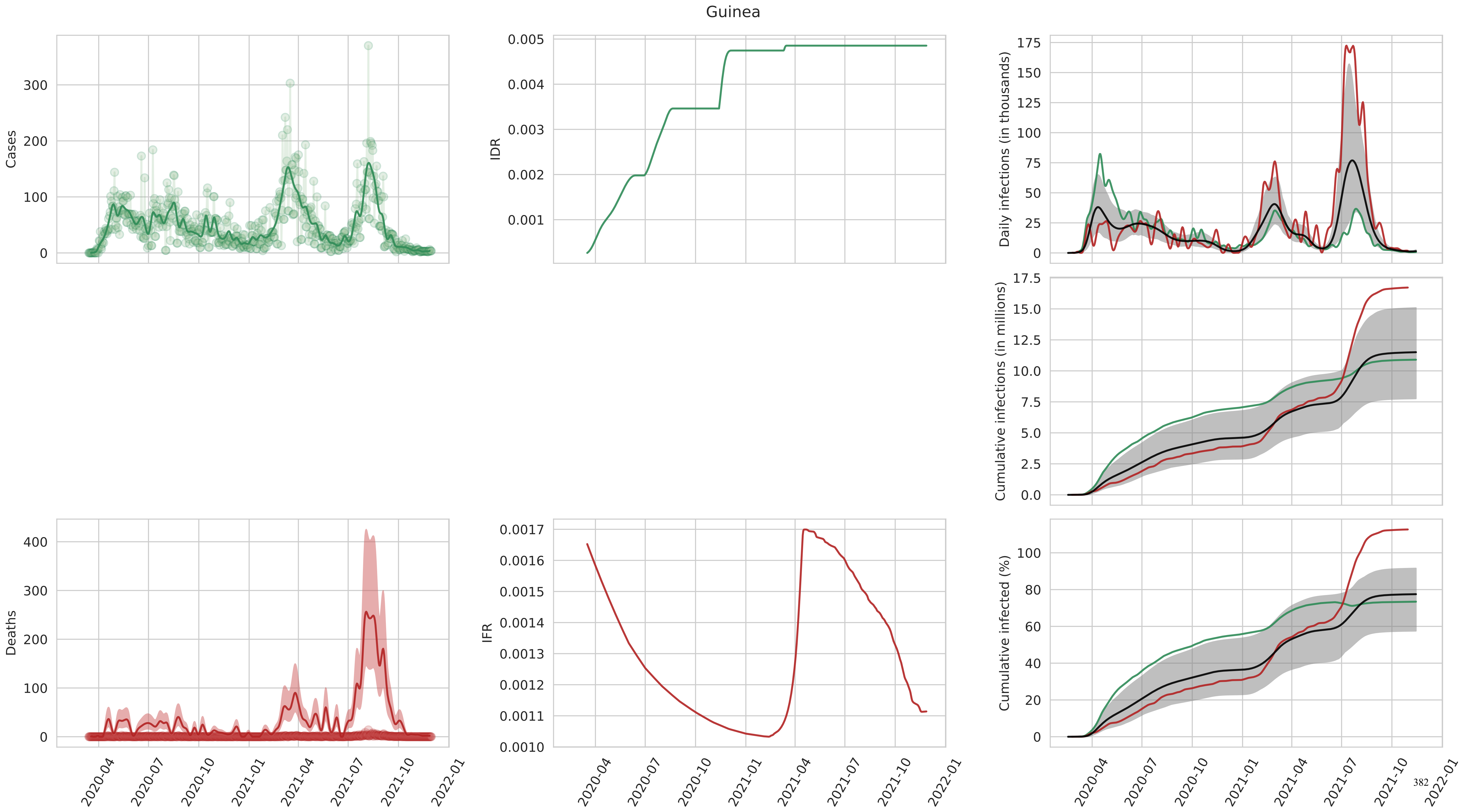
Guinea
Cases
IDR
Daily infections (in thousands)
Cumulative infections (in millions)
Deaths
IFR
Cumulative infected (%)
2020-04
2020-07
2020-10
2021-01
2021-04
2021-07
2021-10
2022-01

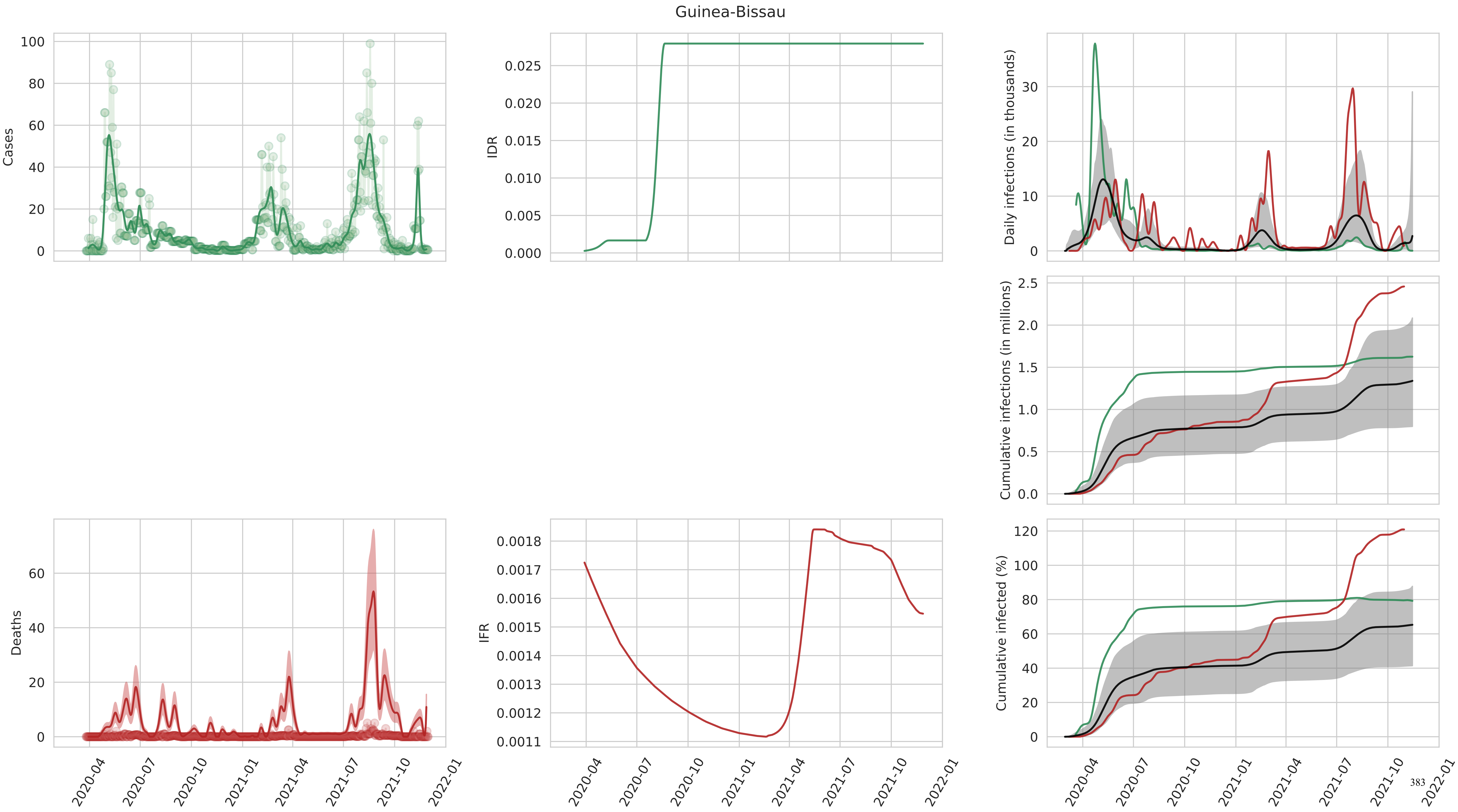

Guinea-Bissau
Cases
IDR
Daily infections (in thousands)
Cumulative infections (in millions)
Deaths
IFR
Cumulative infected (%)
2020-04
2020-07
2020-10
2021-01
2021-04
2021-07
2021-10
2022-01

Liberia

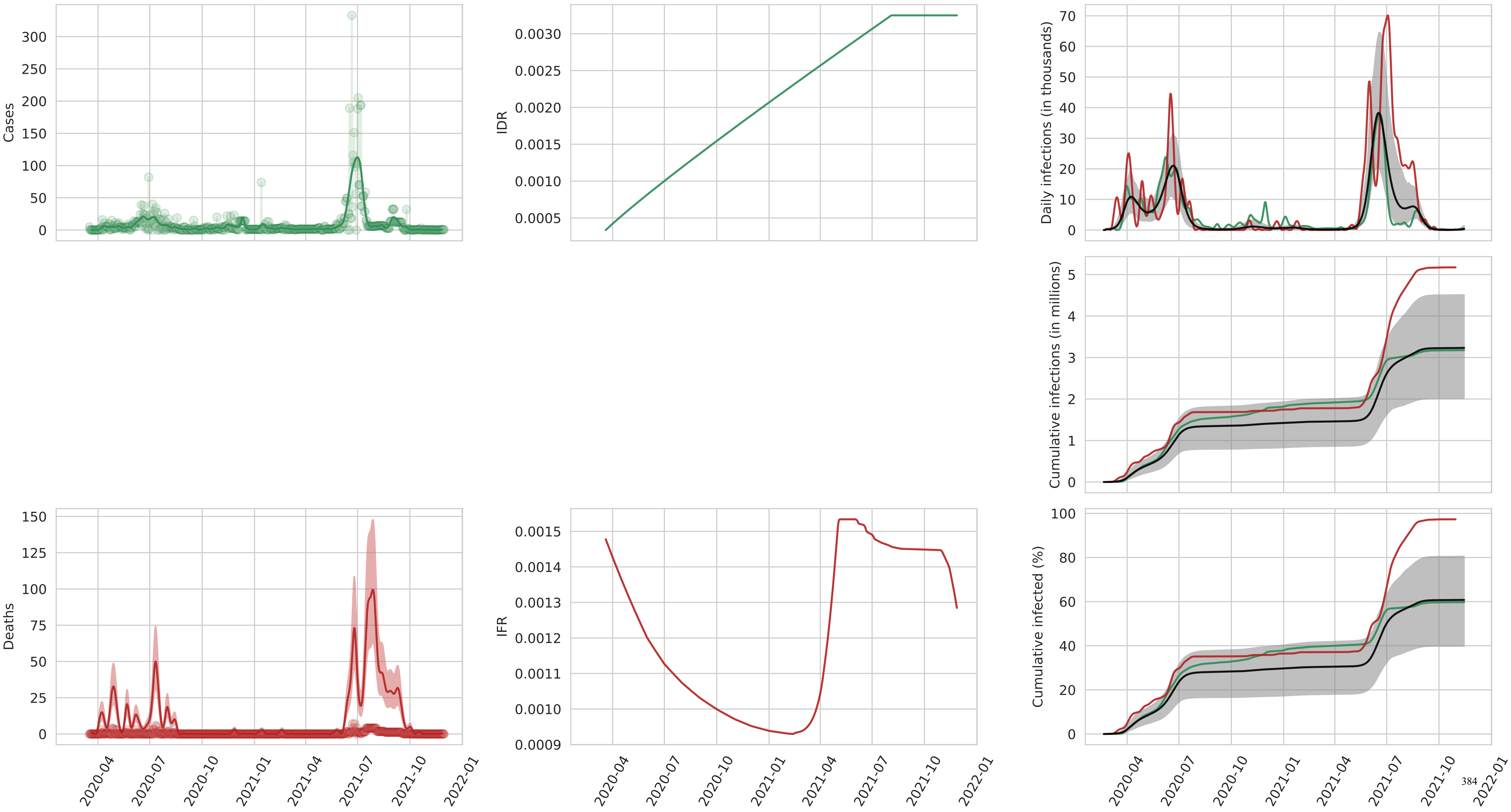
Cases
IDR
Daily infections (in thousands)
Cumulative infections (in millions)
Deaths
IFR
Cumulative infected (%)
2020-04
2020-07
2020-10
2021-01
2021-04
2021-07
2021-10
2022-01

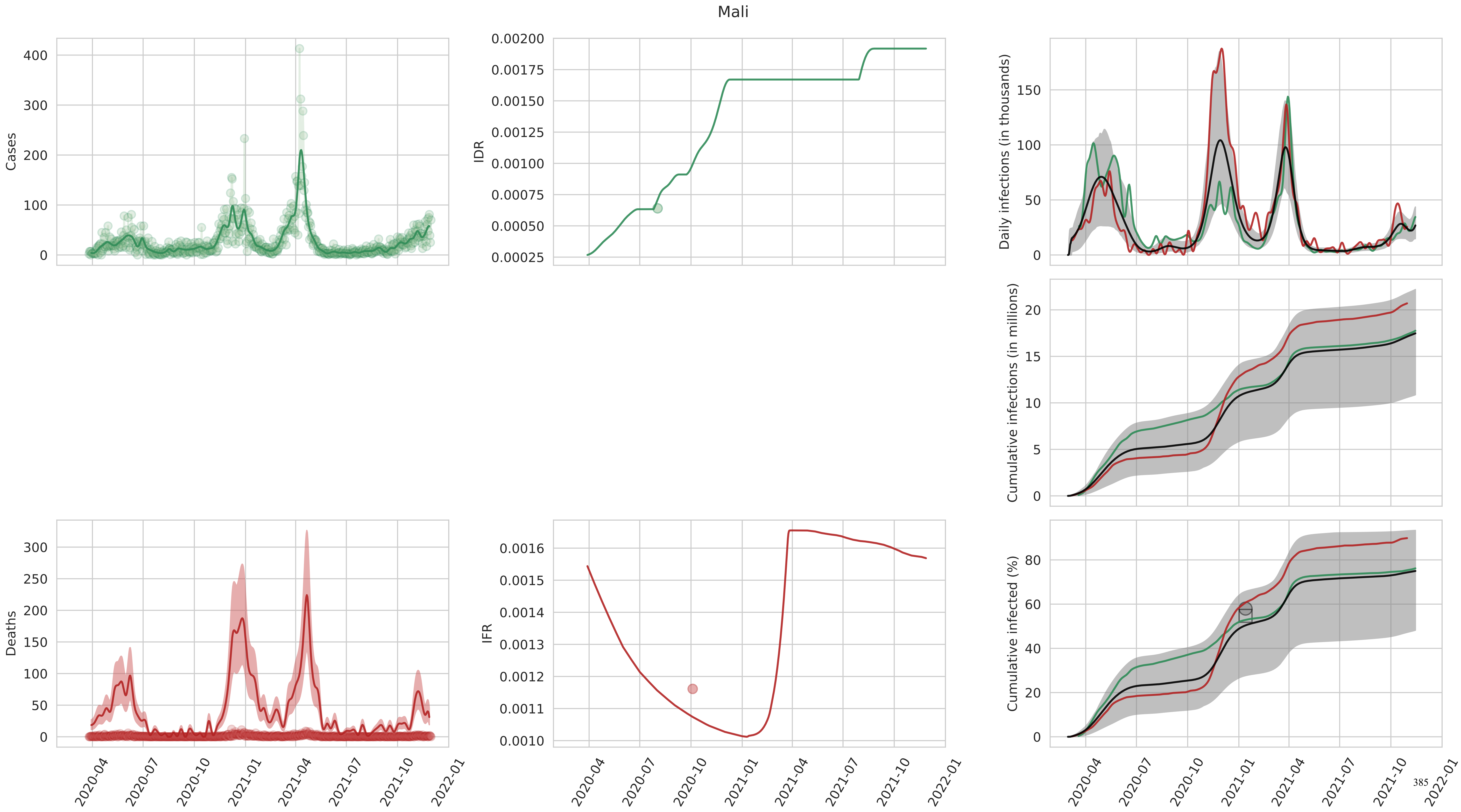

Mali
Cases
IDR
Daily infections (in thousands)
Cumulative infections (in millions)
Deaths
IFR
Cumulative infected (%)
2020-04
2020-07
2020-10
2021-01
2021-04
2021-07
2021-10
2022-01

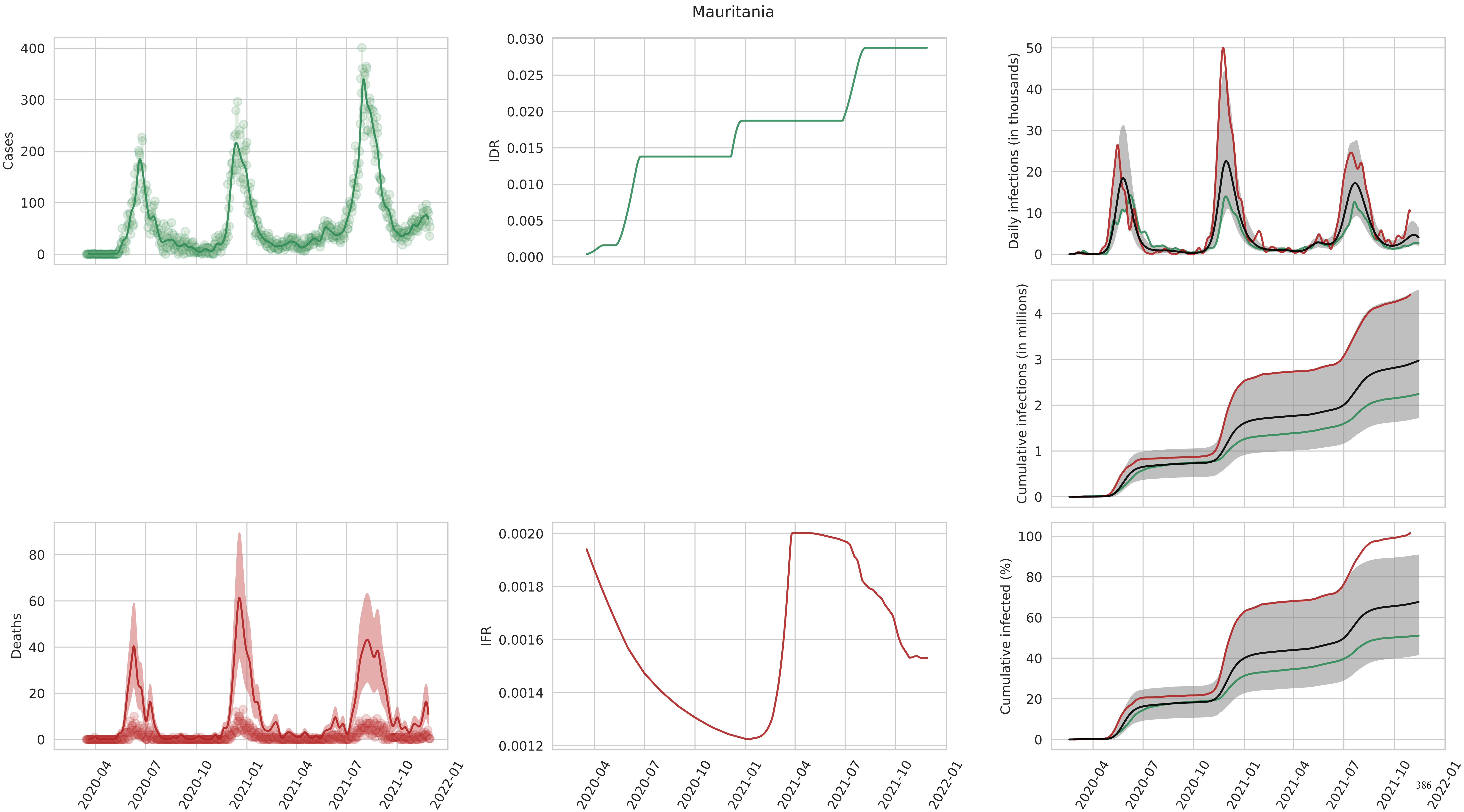

Mauritania
Cases
IDR
Daily infections (in thousands)
Cumulative infections (in millions)
Deaths
IFR
Cumulative infected (%)
2020-04
2020-07
2020-10
2021-01
2021-04
2021-07
2021-10
2022-01

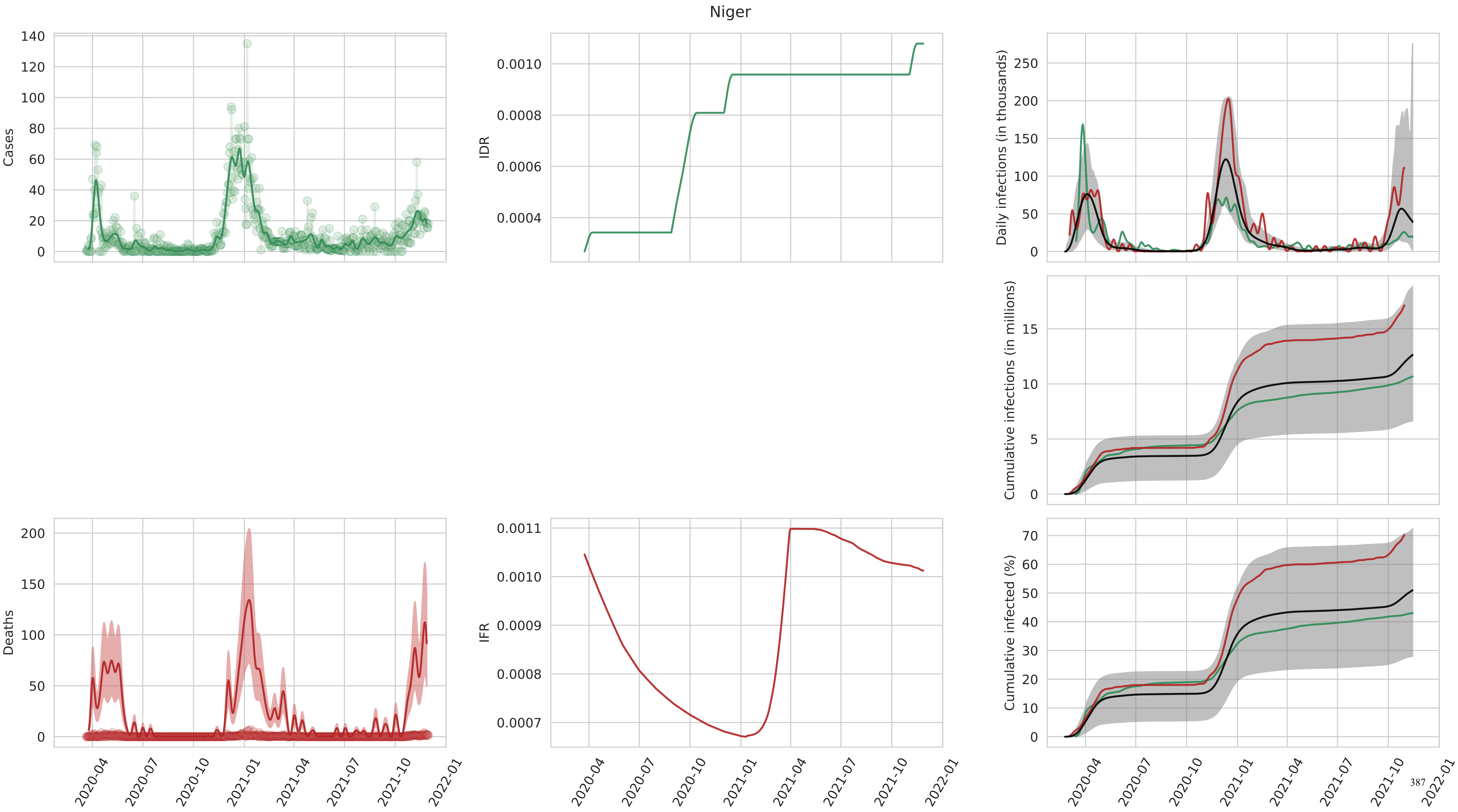
Niger
Cases
IDR
Daily infections (in thousands)
Cumulative infections (in millions)
Deaths
IFR
Cumulative infected (%)
2020-04
2020-07
2020-10
2021-01
2021-04
2021-07
2021-10
2022-01

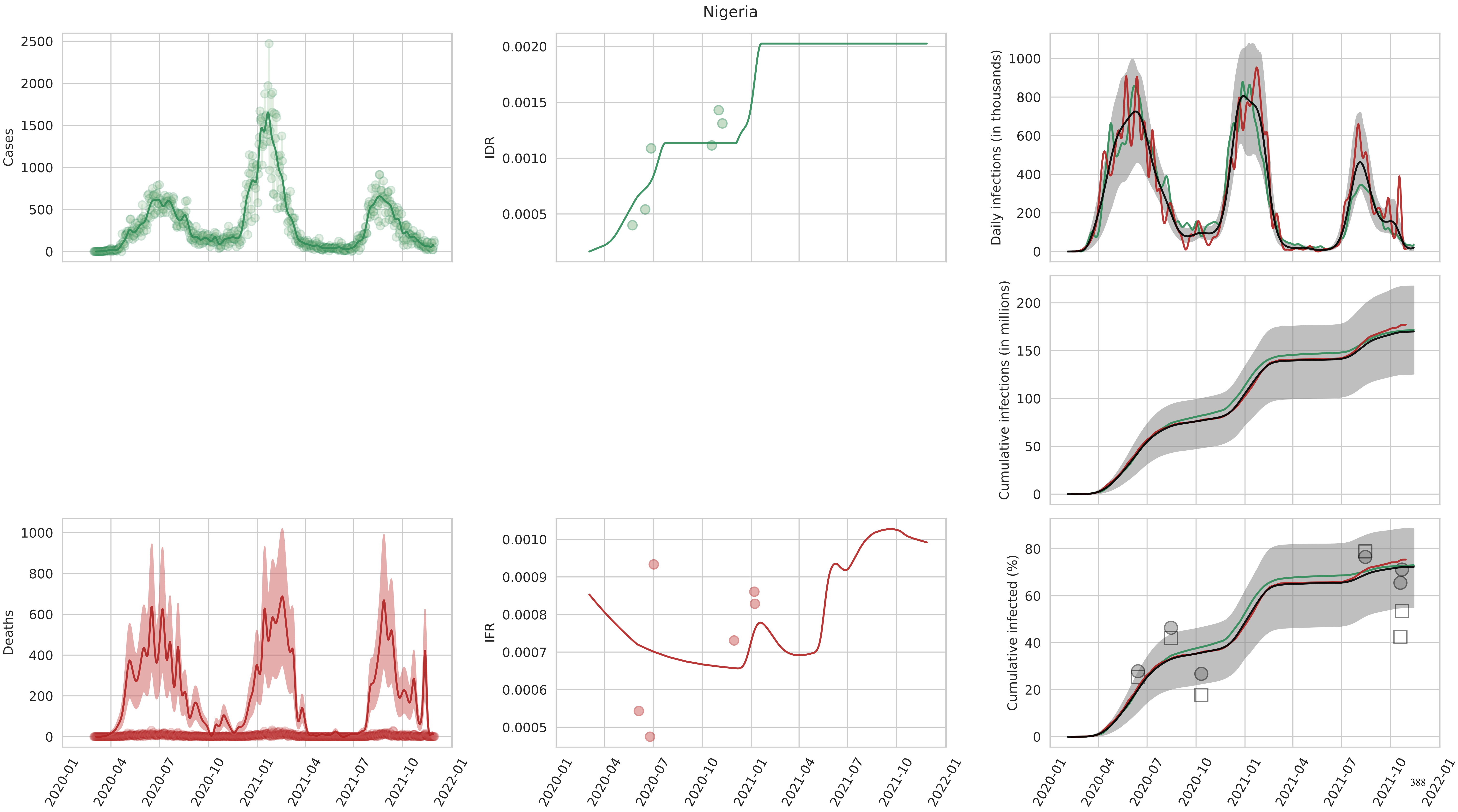

Nigeria
Cases
IDR
Daily infections (in thousands)
Cumulative infections (in millions)
Deaths
IFR
Cumulative infected (%)

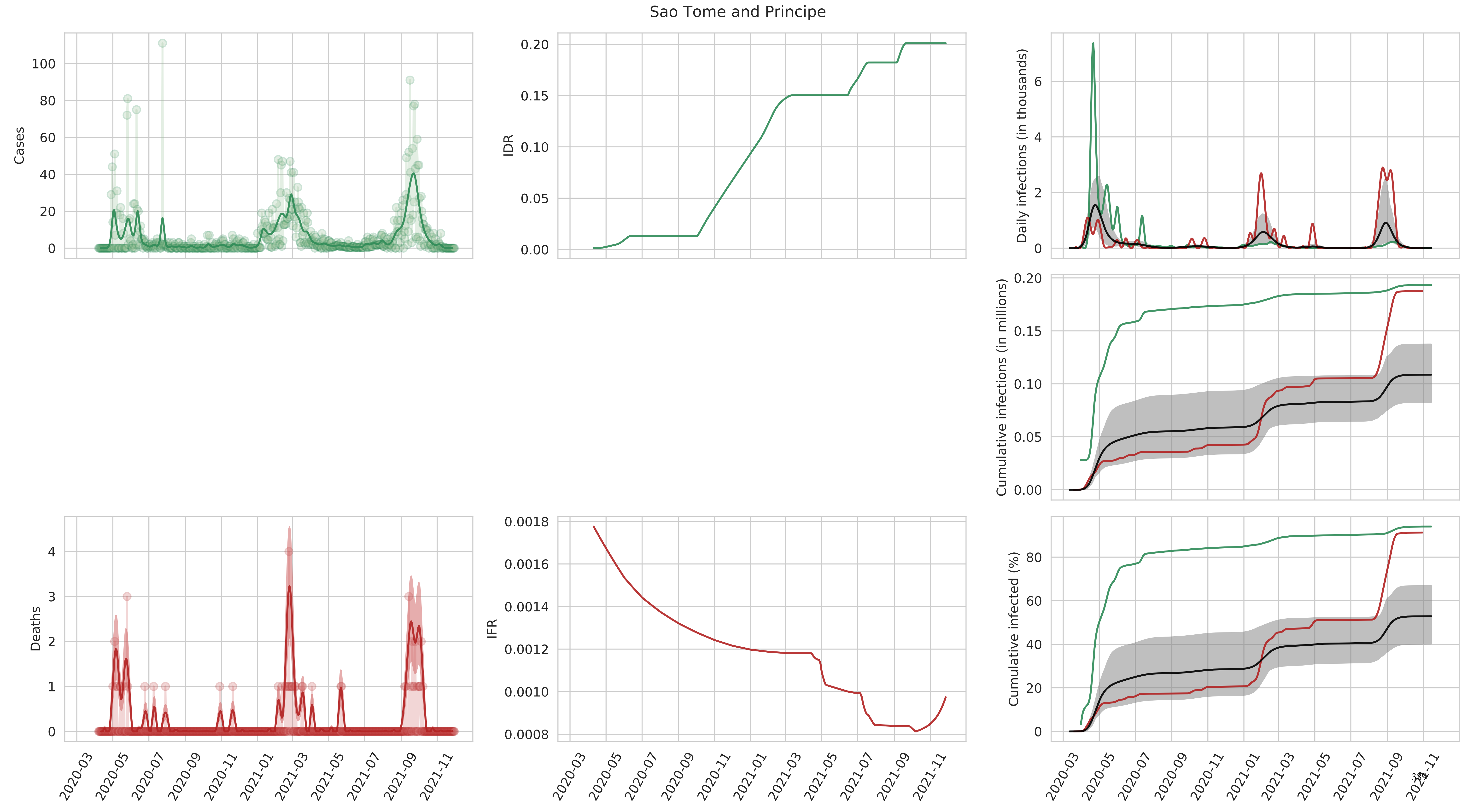
Sao Tome and Principe
Cases
IDR
Daily infections (in thousands)
Cumulative infections (in millions)
Deaths
IFR
Cumulative infected (%)
2020-03
2020-05
2020-07
2020-09
2020-11
2021-01
2021-03
2021-05
2021-07
2021-09
2021-11

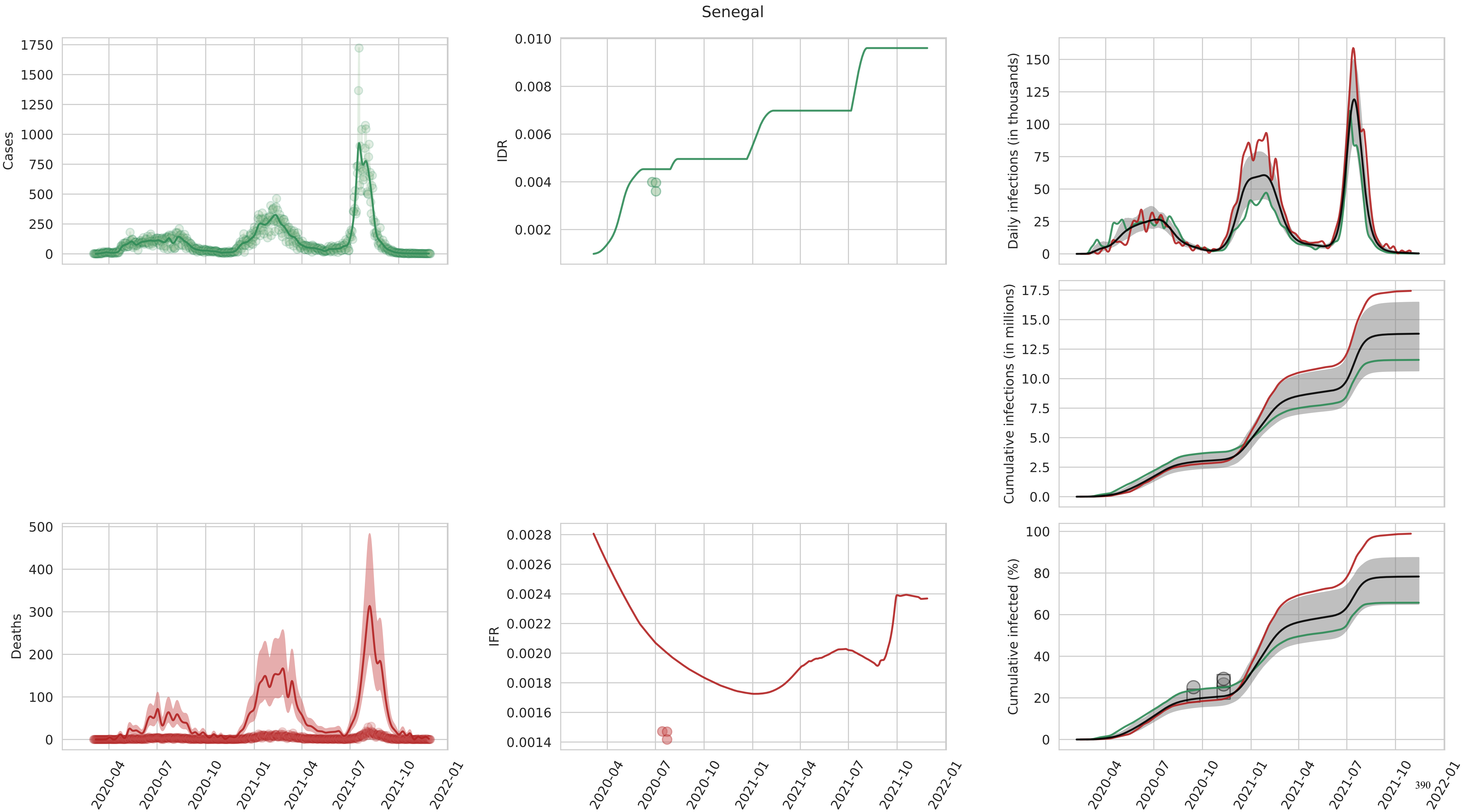
Senegal
Cases
IDR
Daily infections (in thousands)
Cumulative infections (in millions)
Deaths
IFR
Cumulative infected (%)
2020-04
2020-07
2020-10
2021-01
2021-04
2021-07
2021-10
2022-01

Sierra Leone

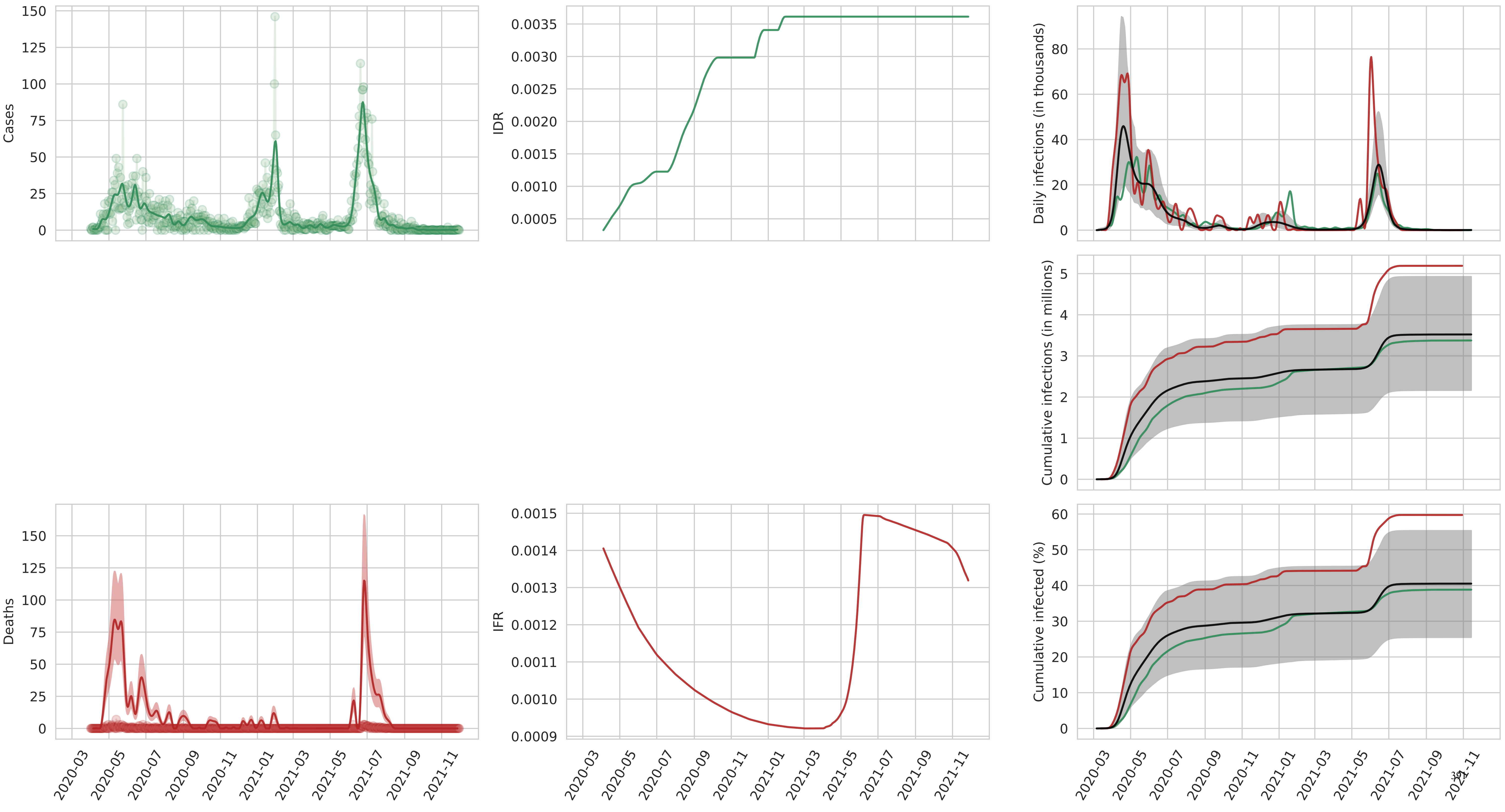

Togo

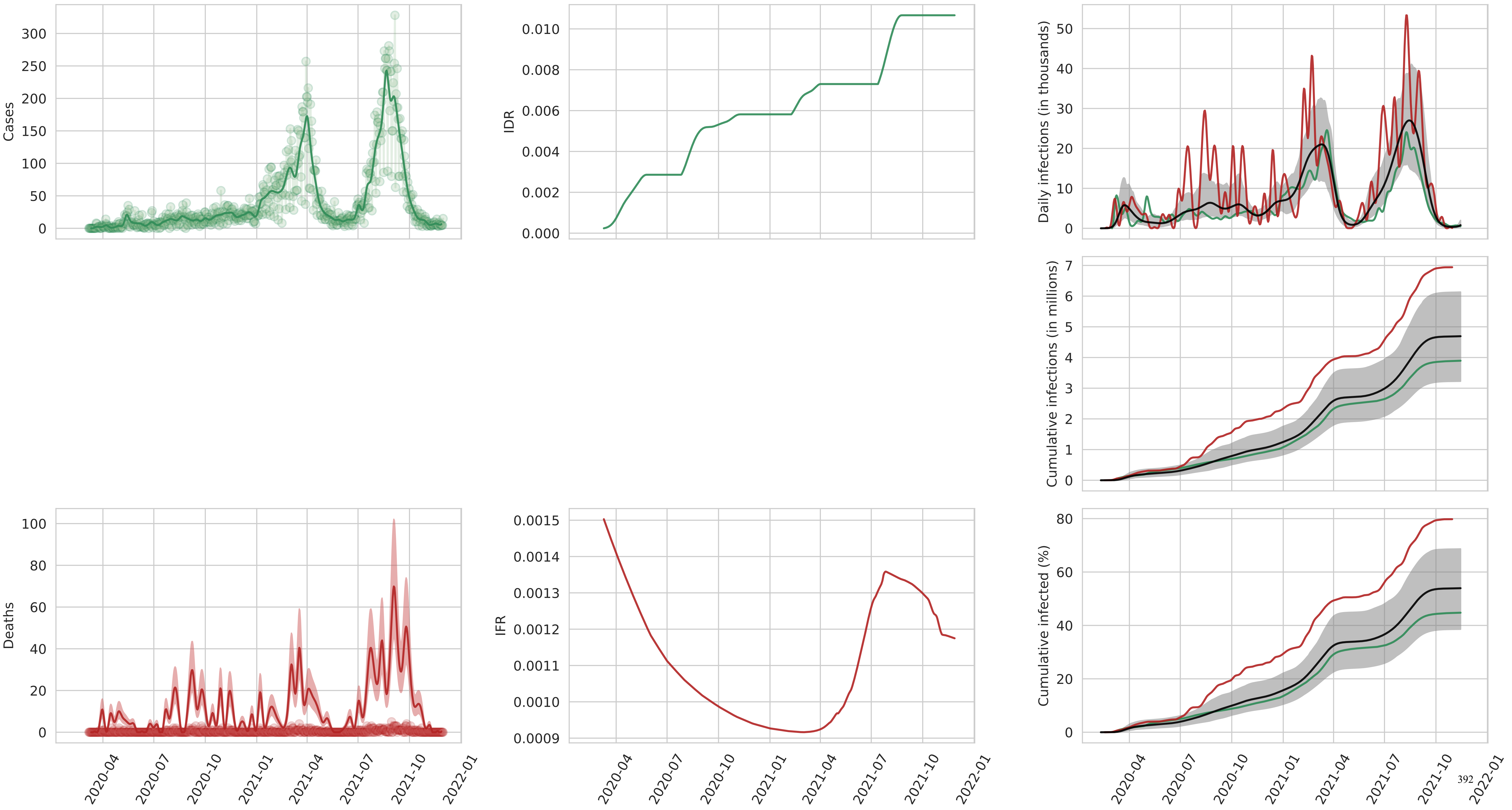
Cases
IDR
Daily infections (in thousands)
Cumulative infections (in millions)
Deaths
IFR
Cumulative infected (%)
2020-04
2020-07
2020-10
2021-01
2021-04
2021-07
2021-10
2022-01

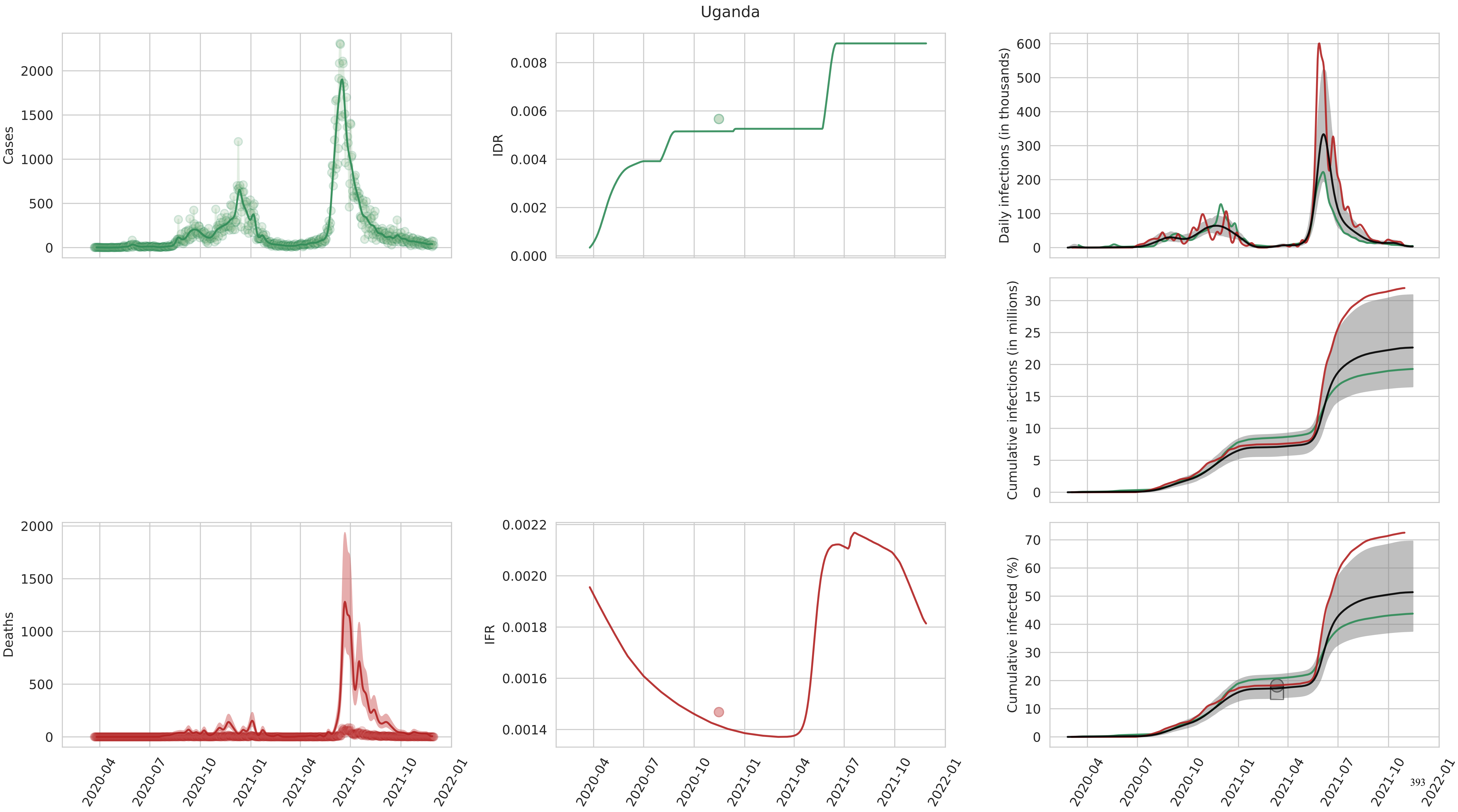

Uganda
Cases
IDR
Daily infections (in thousands)
Cumulative infections (in millions)
Deaths
IFR
Cumulative infected (%)
2020-04
2020-07
2020-10
2021-01
2021-04
2021-07
2021-10
2022-01
